# Rates of Mutation and Host Transmission for an *Escherichia coli* Clone over 3 Years

**DOI:** 10.1371/journal.pone.0026907

**Published:** 2011-10-27

**Authors:** Peter R. Reeves, Bin Liu, Zhemin Zhou, Dan Li, Dan Guo, Yan Ren, Connie Clabots, Ruiting Lan, James R. Johnson, Lei Wang

**Affiliations:** 1 School of Molecular and Microbial Biosciences, University of Sydney, Sydney, Australia; 2 Tianjin Economic-Technological Development Area School of Biological Sciences and Biotechnology, Nankai University, Tianjin, China; 3 The Key Laboratory of Molecular Microbiology and Technology, Ministry of Education, Tianjin, China; 4 Veterans Affairs Medical Center and Department of Medicine, University of Minnesota, Minneapolis, Minnesota, United States of America; 5 School of Biotechnology and Biomolecular Sciences, University of New South Wales, Sydney, Australia; 6 Tianjin Research Center for Functional Genomics and Biochip, Tianjin, China; Naval Research Laboratory, United States of America

## Abstract

Although over 50 complete *Escherichia coli*/*Shigella* genome sequences are available, it is only for closely related strains, for example the O55:H7 and O157:H7 clones of *E. coli*, that we can assign differences to individual evolutionary events along specific lineages. Here we sequence the genomes of 14 isolates of a uropathogenic *E. coli* clone that persisted for 3 years within a household, including a dog, causing a urinary tract infection (UTI) in the dog after 2 years. The 20 mutations observed fit a single tree that allows us to estimate the mutation rate to be about 1.1 per genome per year, with minimal evidence for adaptive change, including in relation to the UTI episode. The host data also imply at least 6 host transfer events over the 3 years, with 2 lineages present over much of that period. To our knowledge, these are the first direct measurements for a clone in a well-defined host community that includes rates of mutation and host transmission. There is a concentration of non-synonymous mutations associated with 2 transfers to the dog, suggesting some selection pressure from the change of host. However, there are no changes to which we can attribute the UTI event in the dog, which suggests that this occurrence after 2 years of the clone being in the household may have been due to chance, or some unknown change in the host or environment. The ability of a UTI strain to persist for 2 years and also to transfer readily within a household has implications for epidemiology, diagnosis, and clinical intervention.

## Introduction

DNA sequencing has revolutionised the study of bacterial evolution. Taxonomy is now based primarily on sequence data, and whole genome sequences have greatly advanced our understanding of bacterial diversity. The recent availability of multiple genome sequences for several species has given new insights into the evolutionary processes affecting bacterial clones. In comparing isolates of a species we find base substitutions in shared genes, other genes present in only some isolates, and variation in the presence of elements such as transposable elements or phages, all representing changes since the most recent common ancestor (MRCA).

However, for natural populations of bacteria the dynamics of such changes are still poorly understood. The population structures of bacteria are very different from those of more complex organisms as the very low frequency of genetic recombination relative to reproduction allows development of multiple clones adapted to specific niches, such as the well-known pathogenic forms of *E. coli* that occur as a series of clones with an O157:H7 clone causing haemolytic uremic syndrome perhaps the best known. Bacterial species also characteristically have a core genome of genes shared by all members of the species and an auxiliary genome of genes present in only some. A recent estimate [1] for *E. coli* based on 17 genome sequences, is that the core genome is approximately 2,200 genes, and as the genomes had from 4238 to 5589 genes, each has a substantial number from the auxilliary genome. The total number of genes, the pangenome, was estimated to be 13,000 genes,

In order to understand bacterial evolution we need details of mutation, recombination, gain and loss of genes in the auxiliary genome, and also gain and loss of mobile elements such as plasmids and bacteriophages, that are additional to the auxiliary genome, but often carry genes affecting host functions. *E. coli* is a clonal species in the sense that specific clones are often isolated over many years, although clonality in the strict sense of origin from a single ancestor, is often lost due to recombination. However the term clone is commonly (and usefully) applied to isolates that clearly have a single recent ancestor in terms of cell division, regardless of having a part of their genome derived by recombination since that MRCA. It is only when we have a group of genomes that are very similar that a high proportion of the differences can be interpreted in terms of individual evolutionary events along specific lineages [2].

In this paper we describe a clone that persisted in a family for three years [3], [4] and examine the genetic and host transmission events that occurred during that period. Clone D was one of two clones that caused a UTI event during the course of a three-year study of *E. coli* in a six-member household. This study was initiated in January 2005 at the time of an acute UTI episode in one household member due to a different strain (clone A), and for each household member five *E. coli* isolates were typed by PFGE. The sampling was repeated on five further occasions. A total of 14 PFGE types or clones were observed of which only 4 lasted for more than a year, and clone D was by far the most frequently isolated.

Clone D was found in one or more household members on each of the 6 sampling dates over the 3 years, and on more than one occasion in five of the six individuals. For four of these individuals clone D accounted for all 5 colonies selected from the sample at least once. The number of clone D-positive sampling dates per host was 5 (dog), 4 (daughter D1), 2 (daughter D2) and 1 (father and son), for a total of 13 of the 32 faecal samples taken [4].

Commensal *E. coli* have long been seen as either transient or persistent in a given individual, as first documented using molecular markers by Caugant et al. [5]. Clone D was clearly a persistent clone. To our knowledge it is the most extensively isolated *E. coli* clone reported to date, in terms of number of hosts, number of samples positive, and duration of detection within the household, and offers an excellent opportunity to study mutation and other genetic events over that timeframe. Accordingly, it was selected for full genome sequencing to (i) estimate the natural mutation rate in the wild (ii) obtain details of host-to-host transmission, (iii) seek evidence of host- or species-specific adaptation during long-term residence of a clone in a particular host, and (iv) determine if there were changes in the dog's UTI isolate that would account for the occurrence of acute UTI.

## Results and Discussion

In the original household studies [3], [4], only 1 arbitrarily selected isolate was retained if there was more than 1 for any clone from a faecal sample. We sequenced that isolate for each of the 13 samples in which clone D was found, plus the urine isolate from the dog at the time of the UTI event, to give 14 genome sequences for analysis. We also obtained a genome sequence of the mother's clone A urine isolate i1. Full genome sequences were obtained for isolates i2 and i14 using 454 and ABI Sanger sequencing, from the first and last samplings, respectively, and the details are presented in Table S1. The remaining 12 isolates were sequenced using Illumina GA2 sequencing.

### The genome sequences of *E. coli* clone D and clone A

**Figure 1:**
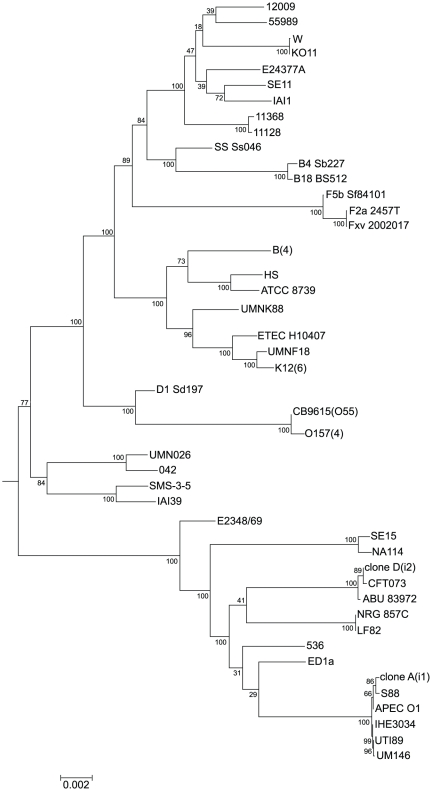
A tree of the *E. coli* strains (including *Shigella* strains) with full genome sequences. The phylogenetic tree was constructed based on the alignments of the core genomes of the 56 *E. coli*/*Shigella* genome sequences available (Table S9), including clone D (i2) and clone A (i1) described in this paper. The genome of *E. fergusonii* was used as outgroup. Bootstrap values are given at each node. The multiple genome sequences for *E. coli* K-12, *E. coli* B, and *E. coli* O157:H7 were each combined as a single entry.

**Figure 2:**
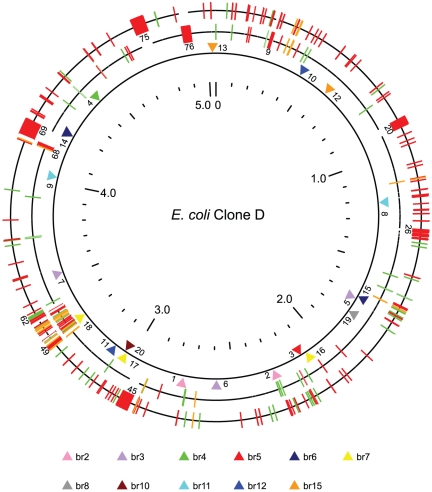
Comparison of the genomes of clone D and CFT073, and distribution of SNPs in clone D isolates. The outer ring represents the genome of CFT073 [7] and the middle ring that of clone D. Both rings show insertions (red) and deletions (green) with respect to the other strain, with the distinction being determined by outgroup analysis, as described in the supporting information. Indels for which the distinction between insertion and deletion cannot be made are marked on both genomes (orange). The large indels (>10 kb) are numbered as in Table S3. The inner ring with the triangular pointers shows the SNPs found in the 14 clone D isolates. The SNPs are also numbered as in Table 2.

Our description of clone D is based on isolate i2, from the first sampling in 2005, and the variation exhibited by the other clone D isolates is discussed below. Clone D has a single circle 5038386 bp chromosome and no plasmids. The genome has 4963 protein-coding genes and 19 pseudogenes. The details are shown in Table S1. Clone D is in sequence type ST73 [4], and the most similar of the available *E. coli* genome sequences is that of CFT073 [6], a blood isolate from a woman with acute pyelonephritis, with the asymptomatic bacteriuria (ABU) strain 83972 [7], also extremely close. Both are also in ST73 (Figure 1). We compared clone D primarily with CFT073 (Figure 2). Of the 4982 genes of clone D (excluding rRNA and tRNA genes), 98% (4894) are shared with CFT073, with only 88 additional genes in clone D, and 383 in CFT073, making these 2 strains and strain 83972 among the most closely related of *E. coli* with a published genome (Figure 1, Table S2). Clone D carries a typical set of virulence factors for a UTI strain (Table 1), which are clearly related to those of CFT073 and 83972, as distinct from those found in clone A and APEC O1. Clone D has however lost the *pap* genes present in CFT073 but it is known that *pap* genes are not essential for UTI as other genes can carry out the role of adhesion, and Clone D has a range of fimbrial genes shared with CFT073 as shown in Table 1. Of interest is that APEC O1 has 2 plasmids not present in clone A, one of which gives it copies of 5 virulence factors present in the chromosome of 83972 (Table 1),

**Table 1:** Distribution of selected virulence factors^a^^,^ b ^,^ c ^,^ d ^,^ e ^,^ f ^,^ g.

Virulence gene	ST distribution pattern	Function	CFT073	Clone D	ABU 83972	APEC O1	Clone A
*fyuA*	both	Yersiniabactin siderophore synthesis	+	+	+	+	+
*kps*	both	group II capsular polysaccharide gene cluster	+	+	+	+	+
*ompT*	both	outer membrane protease	+	+	+	+	+
*usp*	both	uropathogenic specific protein	+	+	+	+	+
*malX*	both	bifunctional maltose and glucose-specific PTS^c^	+	+	+	+	+
*yad* d	both	fimbrial gene cluster	+	+	+	+	+
*auf* d	both	fimbrial gene cluster	+	+	+	+	+
*pap* e	both	*pap* fimbrial gene cluster	+		+	+	+
*pap 2*		a second *pap* gene cluster on different pathogenicity island	+				
*fim* f	both	type 1 fimbrial gene cluster	+	+		+	+
*iutA*	73	aerobactin siderophore synthesis	+	+	+	p	
*iroN*	73	salmochelin siderophore receptor	+	+	+	p	
*sitA to sitD*	73	iron/manganese transport	+	+	+	p	
*iha*	73	siderophore receptor	+	+	+		
*sfa/foc* d	73	S and F1 C fimbriae combined operon	+	+	+		
*hly* g	73	hemolysin	+		+		
*iss*	73	serum survival			+	p	
*tsh*	73	autotransporter/adhesin			+	p	
*tia*	95	invasion				+	+
*cdtB*	95	cytolethal distending toxin				+	
*ibeA*	95	invasion				+	
*traT*	95	outer membrane protein; serum resistance				p	+
*ireA*	95	siderophore receptor	+			+	+

a genes and gene clusters grouped into those found in both ST73 and ST95, those characteristic of ST73 strains and those characteristic of ST95 strains.

b +, gene or gene cluster present and apparently functional. p, gene on a plasmid. If cell blank that gene or gene cluster is absent unless there is a specific footnote describing the nature of a deletion or other deficiency in that strain.

c PTS, phosphotransferase system.

d These fimbrial operons look to be functional in all genomes where present (*sfa*/*foc*, *auf*, *yad*) Note that *sfa* and *foc* are in a shared gene cluster.

e *papI* to *papG* deleted in clone D. Pap fimbriae appear not to be functional in ABU 83972 [23].

f ABU 83972 has a deletion in the *fim* operon [23].

g *hly*ABCD genes deleted in clone D.

A draft genome sequence of strain i1 (clone A) (see Materials and Methods) shows it to be very similar to the well-documented *E. coli* APEC O1, in a cluster of ExPEC genomes from phylogenetic group B2 (Figure 1). Clone A, APEC O1, S88, IHE3034, UTI89 and UM146 (all ST95) constitute a subcluster of very closely related strains, which is quite divergent from the ST73 subcluster with clone D and its relatives (Figure 1). No convergent SNPs were found in clone A and clone D, and no significant recombination events or evidence for any mobile element transfer between them, showing that the two UTI strains were not related in any way other than as shown in Figure 1.

### Evolution of *E. coli* clone D, as constructed by genome sequencing

The 14 clone D isolates sequenced (designated i2 to i15) included one each from the 13 faecal samplings that yielded this clone, as listed above, plus one dog UTI urine isolate (i7). The full genome sequences obtained for i2 and i14, the first and last isolates of clone D (38 months apart), differed by only 8 base substitutions. Only 12 more differences were observed in the 12 Illumina GA2 sequences of the remaining isolates of clone D, each involving a single base. The 20 sites are well separated and all are attributed to mutation in clone D, with none to recombination (details in Table 2). For the 12 Illumina sequences, 96.67% of the genome was covered and all synonymous and non-synonymous SNPS in non-repeat sequenced regions were identified. Of the 8 differences between i2 and i14, all but one would have been detected by the Illumina sequencing, indicating that very few SNPs have been overlooked. We would also have observed any movement of mobile elements, but not mutations within them.

**Table 2:** Details of mutations in clone D isolates.

No.	Lineage	Site	Base change	Mutation type^a^	Gene	Gene name	Product
1	br2	2757922	G**C**G-G**T**G	ns (A-V)	i02_2755	*cysW*	sulfate/thiosulfate transporter permease
2	br2	2290533	**G**-**A**	nc	**-**	**-**	
3	br5	2127286	**C**-**T**	nc	**-**	**-**	
4	br4	4420058	C**C**G-C**T**G	ns(P-L)	i02_4426	*yihX*	phosphatase
5	br3	1713549	**A**GT-**C**GT	ns(S-R)	i02_1752	*arnB*	oxidoreductase
6	br3	2589907	**A**TT-**G**TT	ns(I-V)	i02_2594	*yfbE*	UDP-4-amino-4-deoxy-L-arabinose-oxoglutarate aminotransferase
7	br3	3530555	**G**-**A**	nc	**-**	**-**	
8	br11	1216864	**C**TG-**A**TG	ns(L-M)	i02_1200	*mdoH*	glucosyltransferase
9	br11	4025065	AC**C**-AC**T**	s	i02_4037	*bisC*	biotin sulfoxide reductase
10	br12	449533	**A**AA-**G**AA	ns (K-E)	i02_0442	*lacY*	galactoside permease
11	br12	3101973	GC**G**-GC**A**	s	i02_3096	*fucK*	L-fuculokinase
12	br15	605675	AT**T**-AT**G**	ns(I-M)	i02_0599	*gip*	hydroxypyruvate isomerase
13	br15	5017924	CT**G**-CT**A**	s	i02_4981	*deoD*	purine nucleoside phosphorylase
14	br6	4198963	**T**-**C**	nc	**-**	**-**	
15	br6	1698848	GC**G**-GC**A**	s	i02_1743	*xasA*	amino acid antiporter
16	br7	2100118	C**A**G-C**T**G	ns (Q-L)	i02_2160	*yedE*	putative inner membrane protein
17	br7	3031138	**G**-**T**	nc	**-**	**-**	
18	br7	3304036	TC**C**-TC**T**	s	i02_3289	-	transposase
19	br8	1788891	G**C**A-G**T**A	ns (A-V)	i02_1823	*fumC*	fumarate hydratase
20	br10	3033977	TT**A**-TT**T**	ns(L-F)	i02_3029	*ygbM*	hypothetical protein

a ns, non-synonymous, s, synonymous, nc, non-coding.

**Figure 3:**
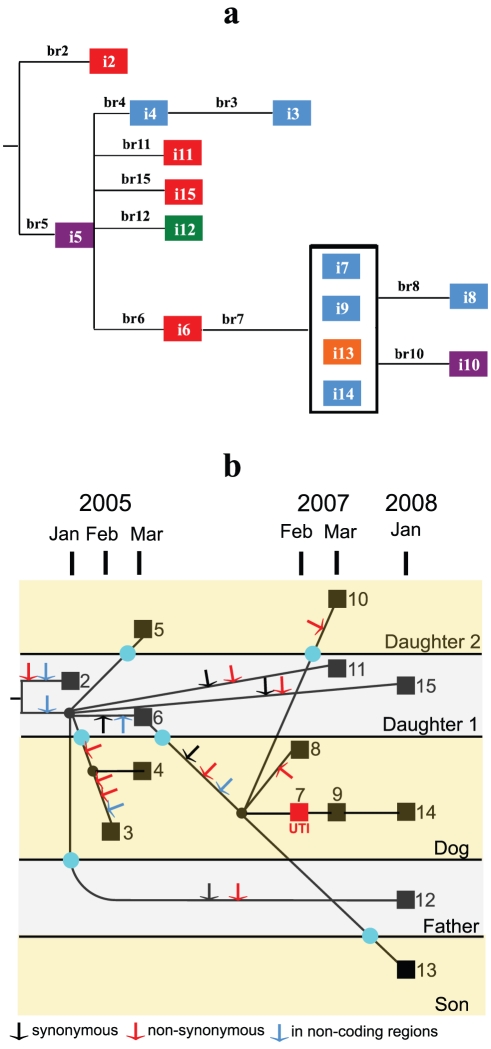
The relationships of the clone D isolates. **a,** Phylogenetic tree of the 11 genotypes observed in clone D. Note that the tree is fully consistent with the data and outgroup analysis and requires no parallel or reverse mutations. The isolates are colour-coded for the host (Daughter 1, red; Daughter 2, dark violet; Dog, blue; Father, green; Son, orange). Branches are numbered and lengths are proportional to the number of mutations. **b,** Tree showing the individual isolates (squares) in relation to date of isolation. The mutations along each branch are shown (arrows). Isolates i7, i9, i13 and i14 are genetically identical, and so there are no mutations to record on their branches, which relate only to passage of time. It can be seen that two mutations (No. 1 and 2) were present in isolate i2 (January 2005), and another mutation (No. 3) was present in all other isolates, and must have arisen before February 2005, when first seen in i3. The distribution of isolation dates and hosts shows that there must have been several instances of transfer of this lineage between hosts, with the minimum number consistent with the tree data being 6, as marked on the tree (aqua circles). Black circles indicate branch points (no isolate).

The 20 SNPs yielded a single tree (Figure 3A) with no reverse or parallel changes, and thus a homoplasy index of zero. The 14 isolates fall into 11 genotypes, one isolated 4 times and the others once each (Figure 3A). The January 2005 isolate (i2) differs by two mutations (No. 1 and 2, see Table 2) from the inferred MRCA of the 14 isolates. All other isolates share a different mutation (No. 3), found only in combination with other mutations, showing that it was present before these isolates diverged. The data show that there are likely to have been at least 6 host transfer events, and this most conservative pattern of transfers is shown in Figure 3B. It is, of course, possible that there were more transmission events not captured by the limited screening. It should also be noted that any of the events could have occurred earlier than indicated by the time scale, which tells us only when each mutation was first detected. It is also clear that this conservative pattern of transfers entails some hosts carrying more than one genotype at the same time and, again, limited screening is likely to have led to underestimation of this.

The dog isolates are in 2 groups, which are distinguished by 6 SNPs. The first group comprises isolates i3 and i4, recovered in February and March 2005, respectively, the former having a sequence derived from the latter. Clearly, both forms coexisted at that time. The second group was derived from daughter 1's 2005 isolate i6, and comprises 6 isolates from 2007 or 2008, including the dog's UTI isolate and 1 isolate each from daughter 2 and the son, showing further host transfers.

The tree had 2 branches running in parallel for nearly 3 years, i.e., the “ancestral” branch found in humans only, comprising i5, i6, i11, i12, and i15, with i5 having the ancestral sequence, and the second dog-related branch, with i6 having its ancestral sequence. Isolate i6 is the bridge between the ancestral human-only branch and the derived dog-related branch, as this human isolate has the genotype that gave rise to the dog branch, and has no descendents among the human isolates. Both i5 and i6 were isolated in March 2005, and 2 derivatives of each were isolated in the last sampling in January 2008.

There is no evidence from the clone D genomes for any recombination or gain or loss of genes or mobile elements over the 3 years.

### Evidence for adaptation

Three of the 10 non-synonymous mutations in clone D are in isolate i3, a 2005 dog isolate, and another nsSNP, plus a mutation in a regulatory sequence, occurred in the group of dog-associated isolates found in 2007 and 2008 (i7, i8, i9, and i14 from the dog, and i10 and i13 after transmission to humans) ( Figure S1). This suggests that change of host species from human to dog may exert some selection pressure.

To put this in context, 14 of the 20 SNPs observed in the study are present in only one isolate, and may be transient; only 6 conserved SNPs are found in two or more isolates (Figure S1). These conserved SNPs form 3 internal branches and it is interesting that the first internal branch gives rise to the first dog set of isolates (i3 and i4) in 2005, while the 2 other internal branches are consecutive and lead to the second set of dog isolates in 2007 and 2008. If we take the MRCA of all isolates as the ancestral type, then the MRCA of i3 and i4 (DOG-2005 type) has acquired one conserved non-synonymous SNP, and the MRCA of i7, i8, i9, i10, i13, and i14 (DOG-2007 type) accumulated 5 conserved SNPs, of which 2 are shared with i6, and 3 arose in the derivation of the DOG-2007 type from i6.

These dog-related lineages have a higher proportion of nsSNPs. That is, of the 20 SNPs there are 4, 4, and 3 nc, ns, and s SNPs, respectively, in the human branches (i1, i4, i6, i11, 112, i15), and 1, 6, and 2, respectively, in the dog-related branches (i3, i4, i7, i8, i9, i10, i13, i14), 2 of which are dog-derived human isolates (Figure S1). The differences are not statistically significant due to low numbers, but the ns frequency (66.7%) in dog branches is much higher than in human branches (40.4%).

It is also interesting that i3 from the DOG-2005 colonisation has 3 non-conserved SNPs, more than any other terminal branch. Furthermore, if we include the single SNP shared with i4, also from the dog, 3/4 SNPs arising since the MRCA of clone D as studied may affect function. Likewise, the DOG-2007 set isolates are derived from i6 by 3 mutational steps, including 1 nsSNPs and 1 SNP in a gene regulatory region, with no isolates of any of the intermediates. Thus, the 2 branches with 3 SNPs are dog related.

We can also look at the distribution over time. If we set February 2007 (clone D UTI episode in dog) as a cutoff date, there are 5 earlier isolates (all 2005), all of the ancestral type, and 9 isolates from February 2007 onwards, of which 6 are in the derived DOG-2007 set and 3 in the ancestral set. By using the Fisher exact test we find that the distributions of the ancestral type and DOG-2007 type of isolates before and after the cutoff date are significantly different (P = 0.03). This distinction indicates that the DOG-2007 type was rare or absent in 2005, and then became the dominant type after the cutoff date.

We can also calculate the variation within each of the groups. The average number of SNPs in the genomes of the “ancestor” type isolates that have remained in the humans (i2, i5, i6, i11, i12, i15) is 1.833 SNPs per genome, whereas among the DOG-2007 type isolates (i7, i8, i9, i10, i13 and i14) this average is 0.333 SNPs per genome. The difference in extent of variation between these groups is statistically significant (student t test, 2 independent samples, t = 3.308, df = 10, P = 0.008). The variation of DOG-2005 type is 0.5 SNP per genome.

Overall, this analysis indicates that the DOG-2007 set isolates are newly evolved. They became dominant after 2007 and also transferred to at least two human household members. They have undergone relatively little change since their appearance, but the data suggest that there may have been adaptation related to the change of host. However the numbers of mutations are low and there are no changes in clone D to which we can attribute the UTI event in 2007. Although it is possible that one of the mutations had an effect that we have not recognised, it may well be that the inferred adaptation was to the new host rather than increased urovirulence, and that chance, or some unknown change in the host or environment, resulted in clone D invading the dog's urinary tract and causing disease, after persisting in the household, including the dog, for at least 2 years as a commensal.

It should be noted that Clone D carries a typical set of virulence factors for a UTI strain (Table 1), which are clearly related to those of the other ST73 strains (i.e., CFT073 and 83972), as distinct from those found in clone A and APEC O1, representing ST95 (see Table 1).

Our findings are in marked contrast to the those of Weissman et al. [8], who documented a single nsSNP within the *fimH* pilin gene in an *E. coli* isolate from the urine of a woman with acute cystitis, compared with the same clone as recovered concurrently from faeces. This nsSNP caused a shift in receptor specificity that was regarded as patho-adaptive, and presumably explained the occurrence of the acute UTI episode due to what otherwise behaved as a harmless commensal strain. In our study the faecal and urine isolates from the dog at the time of the UTI event were identical (i7 and i8), and we find no obvious genetic basis for the UTI event in a clone that had been present in the household dog for at least 2 years before that event.

### Mutation rate

For analysis of mutation rates we used the 11 mutations that were first observed in isolates from 2007 and 2008, because mutations present in early 2005 may predate the study. For isolates i11, i12, and i15 we assumed that the mutations occurred between January 2005 and the isolation date. For isolate i6 and its descendents i7, i8, i9, i10, i14, and i15, we took the branch point shown in Figure S1 to be in November 2006, 20 months into the 23-month period from March 2005 to Feb 2007, which gave approximately equal average mutation rates before and after that date. The average rate for the 11 mutations involved was 1.1 mutations per year, being 0.17 and 0.93 for the 4 sSNPs and 7 combined ns and nc mutations, respectively. The regions with sequence suitable for mutation analysis cover 96.67% of the 5038386 bp clone D genome, to give a rate of 2.26×10^−7^ mutations per site per year, and 3.49×10^−8^ and 1.9×10^−7^ for sSNPs and nsSNP, respectively, as Ks and Ka.

This rate of 2.26×10^−7^ mutations per site per year can be compared with the values commonly used for estimation of divergence dates in bacteria, which are 3×10^−8^ mutations per site per year (estimated by Guttman et al. [9]), and 3.1×10^−10^ for Ka and 6.7×10^−9^ for Ks (estimated by Whittam [10]), the first based on laboratory mutation rates in *E. coli* and the second on divergence between *E. coli* and *Salmonella*. The overall rate of 2.26×10^−7^ mutations per site per year for clone D is 7.5-fold higher than the Guttman estimate, and the Ks of 3.49×10^−8^ is 6-fold higher than the Whittam estimate.

This study involves a clone that was present in the household at the time the study started and was not related to the human UTI strain that prompted the study. It is thus a study of *E. coli* under natural conditions. It is likely that some of the mutations relate to a change of host from human to dog, but this is a normal event for *E. coli*, and while these mutations may well not survive subsequent changes of host, it is reasonable to include them in estimating the short term clock rate, as only more studies will tell us how typical is clone D.

It is notable that, as for *V. cholerae* [11], where extrapolation from current mutation data gave a Ks value 100-fold higher than expected for the estimated time frame, our estimated rate is significantly higher than usually assumed. This is consistent with other studies including in *Yersinia pestis* [12], and several other studies summarised in that paper. This pattern for rates of accumulation of mutations estimated for closely related strains being faster than those generally accepted, shows that some revision of conventional assumptions is needed. There is also a range of values, adding to the need for further direct measurements of mutation rates in nature.

Most of the studies cited used isolates obtained for purposes unrelated to study of mutation rates: more accurate estimates of population dynamics and rates would be possible in targeted studies with higher sampling frequencies. This is important, since Rocha et al. [13] and Ho et al. [14] have shown that for closely related taxa, Ks/Ka and levels of mutational difference, respectively, vary depending on time since divergence, in both cases attributed to the time frame for elimination of many of the mutations. We need better data to evaluate the processes involved, and for estimating mutation rates that can then be used to estimate dates of origin for novel variants within species.

It is interesting to compare the observed natural evolution of clone D with clonal development in a laboratory evolution study of *E. coli* over 20,000 generations [15], in which 45 substitutions were accumulated progressively with no evidence of parallel branches, although these have been prominent in other *in vitro* studies [16], [17]. In the Barrick study, the 26 point mutations in coding regions were all non-synonymous. In contrast, clone D had 2 branches running in parallel for the 3 years, and only 10 of the 16 SNPs in coding regions were non-synonymous, which is very different from the *in vitro* observations.

Importantly, we find that the *E. coli* mutation rate in nature is sufficient to be detected by sampling a persistent clone over a few years. This approach has the advantage that the mutation rate can be estimated for individual clones and variation within as well as between species will be determined if enough clones are studied.

### Host transmission and genetic variation within a household

Our study is, we believe, the first household study of an organism growing in its primary natural environment without experimental manipulation, under circumstances where we can obtain solid evidence of the frequency of within-household transmission, and observe the effects of host transmissions on rates of genetic change. Previous studies of households or couples, including the earlier study of clone D [4], could not distinguish definitively between transmission during (or before) the study vs. parallel acquisition from an external source, since they lacked detailed genetic information. Here, the evidence for transmission reported in the earlier studies of clone D is confirmed and transmission is shown to be quite frequent relative to mutational change. Indeed, there is evidence that individuals who have repeated isolation of a particular clone at successive sampling points may not have simple persistence of the clone, but instead are repeatedly reacquiring it, sometimes from different household members. The situation is even more dynamic than expected, with a lot of “ping-ponging” evidently occurring.

This combination of long term persistence and high rate of transmission suggests that such strains have abundant opportunity to pass from individual to individual while residing within the normal microbiota, which can obscure their original entry to the individual when they do happen to cause disease. It is only by study of a household or similar community with several members that one can assess the frequency of such transfer events. Consequently, for determination of mutation rates, there is a need for many such studies on clonal persistence in families or other groups where it is possible to study transfer over periods of several years, in order to assess the variation in frequency of transmission, as in each such study very few persistent strains will emerge. We would expect that opportunities for transmission would also exist outside of the household, but we have no way of estimating if overall these would be of similar or greater magnitude.

The observation that disease occurred after a latent period of at least 2 years may also provide opportunities for intervention. That is, if the duration of commensal residence of such organisms in the host could be shortened by an intervention that selectively reduces their colonization fitness in comparison with non-virulent *E. coli*, this should decrease both the colonized hosts' time at risk for developing acute disease and the likelihood of the strain transferring to a new host, thereby putting another individual at risk and prolonging the strain's persistence within the household. The findings also have implications in relation to recurrent UTI, which may involve re-infection from household-associated clones rather than treatment failures or auto-reinfection from the host's own intestinal or vaginal reservoir.

### Conclusions

We have followed an *E. coli* clone for 3 years in a family of 6 individuals. The 14 isolates analyzed had a total of 20 mutational base changes, and fell into 11 genotypes. We estimate there is an average of about 1 mutation fixed per year, about 6-fold higher than a widely accepted rate for bacteria in general. No recombination events occurred and there was no gain of mobile elements. This absence of any reassortment of existing diversity over 3 years can be compared with the situation in most multicellular eukaryotes, which in each generation have a round of chromosome reassortment during meiosis and associated recombination between pairs of homologous chromosomes present in a diploid cell.

A phylogenetic tree based on the mutational changes allowed us to demonstrate 6 host transfer events over the 3 years, with the 2 sublineages that diverged in the first year still present at the end of the study. This is the first such study of a clone of bacteria living in their natural environment, allowing both individual genetic and host transmission events to be observed. It is clear that we need more such studies, ideally over a longer time frame, to get better estimates of mutation and other rates. These rates are likely to vary within species such as *E. coli*, with populations that comprise a series of niche-adapted clones. Our study took advantage of isolates taken for another purpose, but the rapidly falling costs of sequencing should allow studies focussed on estimating rates of change to have more frequent sampling than for our study, plus use of many more isolates to improve statistical power.

## Materials and Methods

### Genome sequencing

Chromosomal DNA from isolates i2 and i14 was sequenced using a 454/Roche FLX machine, according to the manufacturer's protocols. The i2 sequencing produced 282,115 reads with an average length of 242 bp, representing a theoretical 12.8-fold coverage of the genome, and 98.4% of the reads were assembled de novo into 222 contigs with an average of 13.0-fold coverage, using the 454/Roche Newbler assembly program. The i14 sequencing produced 337,723 reads with an average length of 231 bp, representing a theoretical 15.5-fold coverage of the genome, and 98.5% of the reads were assembled de novo into 203 contigs with an average of 15.8-fold coverage.

The gaps between these contigs were closed by targeted PCR and sequencing the products with BigDye terminator chemistry on ABI 3730 capillary sequencers. To detect 454 FLX sequence miscalls in homopolymers, all questionable sites, including sites called inconsistently among clone D isolates were checked by sequencing PCR products using an ABI 3730.

Whole genome sequencing of the other 12 clone D isolates was performed with Solexa mate-pair and pair-end sequencing technology [18]. The Solexa Genome Analyzer IIx (Illumina, Little Chesterford, Essex) gave 126.9-fold coverage on average. The Solexa reads generated were culled of duplicates with identical reads that are presumed to be from replicates arising in the PCR step, and were then mapped to the i2 genome to generate the assembly using BWA [19] with default parameters, which allows 4% mismatches. All reads with extremely large or small insert size (<50% or >200% of normal) were mapped again using BLASTn with an e-value of 0.0001 and the -F F flag. Only read pairs that mapped at an appropriate separation (300–700 bp for pair-end sequencing and 1.5–4.5 kb for mate-pair sequencing) and with at least one end mapping in a non-repeat region, were taken into account. The SAMtools program [20] was used to calculate the per-position coverage and base calls for each position.

SNPs between i2 and i14 were detected by genome alignment. Potential SNPs in the other 12 clone D isolates were called where a position was covered by at least 10 reads, with at least 80% of the covering reads showing the same SNP. All potential SNPs were recorded by position in reference to i2 genome. All potential SNPs among the 14 clone D isolates and other suspected positions were confirmed by resequencing of PCR products covering the SNP using an ABI 3730 sequencer.

A draft genome sequence of strain i1 (clone A) was obtained by paired-end sequencing in a Illumina GA IIx. The draft genome consists 56 non-redundant scaffolds (including 79 contigs), representing a typical *E. coli* chromosome with a GC content of 50.51%. All the scaffolds were re-ordered based on its closely related reference genome, APEC O1, and 4792 protein-coding genes were annotated.

We checked for insertion, duplication or deletion differences among the clone D genomes. Indels in homopolymers are not subject to elevated error or bias in Solexa sequencing [18], and small indels could be reliably resolved by the set of individual Solexa reads spanning the homopolymer region. Large segments present only in the i2 sequence would be apparent as gaps in the mapping onto i2 and none were seen. The position of a large insertion or duplication present only in the Illumina sequence, such as an IS movement or copy number variation, would be detected as read pairs aligned around the insert positions that have one end unaligned or aligned to a different region than the first end. Again, no evidence of large indel or duplication was found among the clone D isolates.

### Data deposition

The genome sequences of *E. coli* clone D (strains i2 and i14) and Clone A (strain i1) have been deposited in the GenBank database [accession numbers CP002211, CP002212 and AEYT00000000, respectively].

### Annotation and Analysis

Open reading frames from 30 amino acids in length were predicted using Glimmer 3.0 and verified manually using the annotation of *E. coli* CFT073. Transfer RNA and ribosomal RNA genes were predicted using tRNAscan-SE. Artemis [21] was used to collate data and facilitate annotation. Function predictions (Table S1) were based on BLASTp similarity searches in the UniProtKB, GenBank, and Swiss-Prot protein databases, and the clusters of orthologous groups (COG) database (www.ncbi.nlm.nih.gov/COG). Pseudogenes were detected by BLASTn comparisons of the genome sequences of i2 and CFT073 and manually revised.

The phylogenetic tree of the *E. coli/Shigella* core genome (including Clone D (i2) and Clone A (i1)) (Figure 1) was constructed from the concatenated alignments of the 1698 genes in the *E. coli/Shigella* core genome using the method described previously [2].

### Alignments between clone D and CFT073

Blocks of sequence substantially shared by clone D and CFT073 were determined using BLAST, and the alignment within each block was based on the *Mauve* method [22], with a seed length set equal to 11. The final plot of the 2-genome alignment (Figure 2), including indels and SNPs, was generated by methods developed for *V. cholerae* and *E. coli* (Tables S2, S3, S5 and S7) [2], [11], and collectively known as GA-Plot (Genome Alignment Plot).

### Virtual outgroup analysis for assignment of mutations and indels to a lineage

Virtual Outgroup analysis was applied to the CFT073 and i2 comparison as described previously [2], [11] to allocate base differences to mutations in specific lineages (Tables S4, S6 and S8). Strain *E. coli* ED1a was the most closely related to CFT073 and i2 (Figure 1), and in cases of ambiguity it was given priority in allocation. The details and criteria for estimating the level of support are given in the footnotes to Tables S4, S6 and S8.

## Supporting Information

Figure S1 **Phylogenetic tree of the 14 clone D isolates presented in relation to date of isolation.** The mutations along each branch are shown, and this information is highlighted for the branches used for estimating the mutation rate. The distribution of isolation dates and hosts shows that there must have been several instances of transfer of this lineage between hosts, with the minimum number consistent with the tree data being 6, as marked on the tree.
(PDF)

Table S1 **Clone D genes and products.** All genes are shown with locus tag, start and end positions, name and gene product.
(PDF)

Table S2 **The orthologs of clone D and CFT073 genomes.** Genes present in 1 or both of the genomes are listed with the gene tag number, gene name and product.
(PDF)

Table S3 **Large insertions and deletions.** Large indels (more than 20 bp) affecting the genomes are shown with the length (bp), if thought to be an insertion or deletion, the strain affected and the gene or genes affected.
(PDF)

Table S4 **Large insertions and deletions in clone D relative to CFT073: allocation to lineage by virtual outgroup analysis.** The *E. coli*/*Shigella* genomes used for the analysis are shown, with details of the blocks present in outgroup genomes and genomes under analysis, and also the final allocation and a measure of support level for that allocation.
(PDF)

Table S5 **Small insertions and deletions.** Small indels (not more than 20 bp) affecting the genomes are shown with the length (bp), if thought to be an insertion or deletion, the strain affected and the genes affected.
(PDF)

Table S6 **Small insertions and deletions in clone D relative to CFT073: allocation to lineage by virtual outgroup analysis.** The *E. coli*/*Shigella* genomes used for the analysis are shown, with details of the blocks present in outgroup genomes and genomes under analysis, and also the final allocation and a measure of support level for that allocation.
(PDF)

Table S7 **SNPs between clone D and CFT073 genomes.** A full list of single base differences between clone D and CFT073 genomes, including location, nature of difference, and name of gene affected.
(PDF)

Table S8 **Allocation of SNPs to lineages by virtual outgroup analysis.** The *E. coli*/*Shigella* genomes used for the analysis are shown, with details of the base in outgroup genomes and genomes under analysis, and also the final allocation and a measure of support level for that allocation.
(PDF)

Table S9 **Genbank accession numbers for genome sequences included in** **Figure 1** **.**
(PDF)
