# Supplementary material for: Rates of Mutation and Host Transmission for an Escherichia coli Clone over 3 Years
Source: PLoS One. 2011 Oct 27;6(10):e26907. doi: 10.1371/journal.pone.0026907 (PMC3203180; doi:10.1371/journal.pone.0026907)
Supplement: Figure S1 — Phylogenetic tree of the 14 clone D isolates presented in relation to date of isolation. The mutations along each branch are shown, and this information is highlighted for the branches used for estimating the mutation rate. The distribution of isolation dates and hosts shows that there must have been several instances of transfer of this lineage between hosts, with the minimum number consistent with the tree data being 6, as marked on the tree. (PDF) [file pone.0026907.s001.pdf]

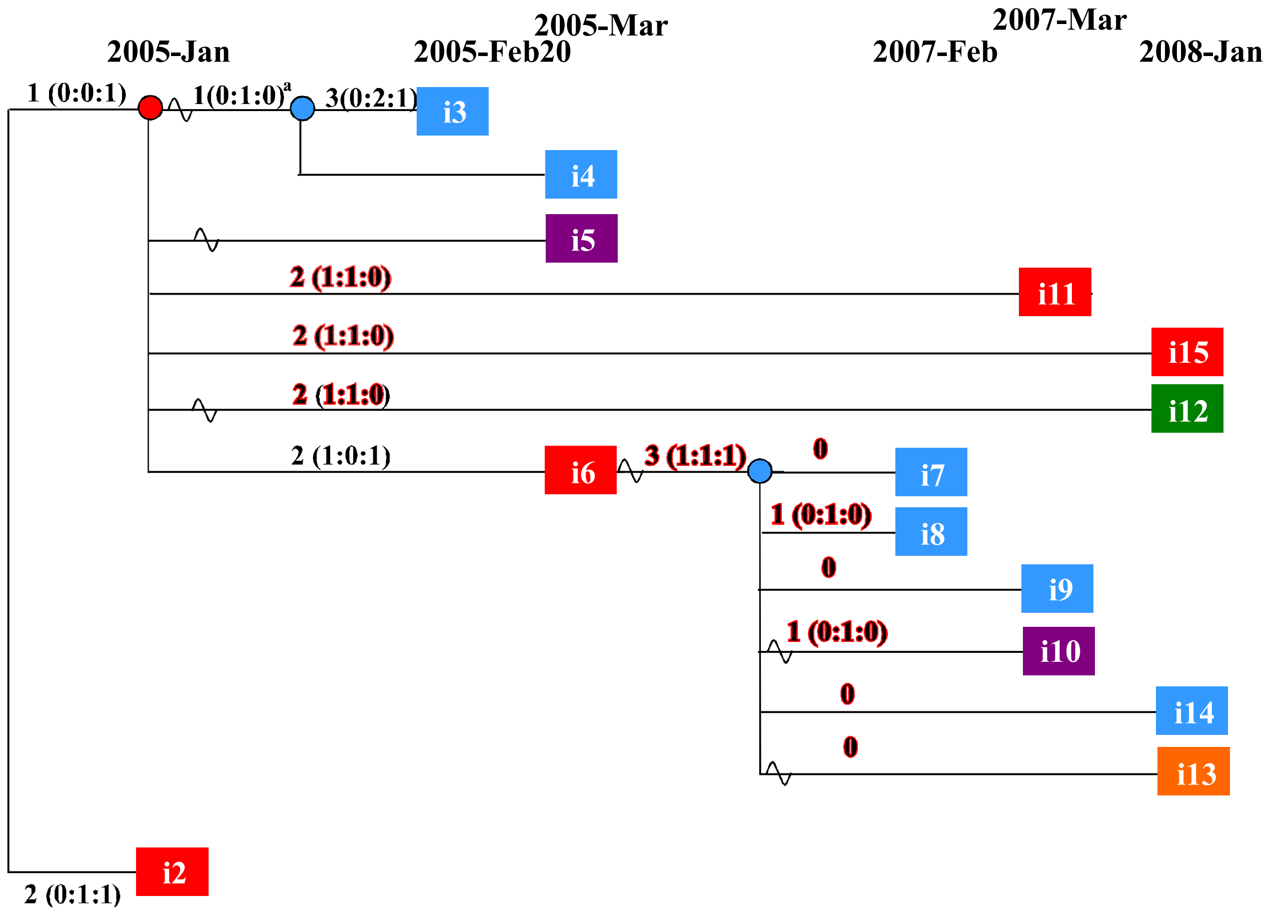

[a] SNP(S: NS: NC)

[b] ∿, transmission between different host

[c] Daughter 1 Daughter 2 Dog Father Son

Figure S1. Phylogenetic tree of the 14 clone D isolates presented in relation to date of isolation. The mutations along each branch are shown, and this information is highlighted for the branches used for estimating the mutation rate. The distribution of isolation dates and hosts shows that there must have been several instances of transfer of this lineage between hosts, with the minimum number consistent with the tree data being 6, as marked on the tree.
