# Supplementary material for: Rates of Mutation and Host Transmission for an Escherichia coli Clone over 3 Years
Source: PLoS One. 2011 Oct 27;6(10):e26907. doi: 10.1371/journal.pone.0026907 (PMC3203180; doi:10.1371/journal.pone.0026907)
Supplement: Table S1 — Clone D genes and products. All genes are shown with locus tag, start and end positions, name and gene product. (PDF) [file pone.0026907.s002.pdf]

Table S1 Clone D genes and products. All genes are shown with locus tag, start and end positions, name and gene product.

Locus_tag	Type	start	End	+/-[a]	Gene[b]	Product
i02_5004	CDS	190	255	+	thrL	thr operon leader peptide homoserine kinase
i02_0001	CDS	336	2798	+	thrA	bifunctional aspartokinase I/homeserine
i02_0002	CDS	2800	3732	+	thrB	homoserine kinase
i02_0003	CDS	3733	5019	+	thrC	threonine synthase
i02_0004	CDS	5087	5236	+	/	hypothetical protein
i02_0005	CDS	5233	5529	+	yaaX	hypothetical protein
i02_0006	CDS	5589	6365	-	yaaA	hypothetical protein
i02_0007	CDS	6435	7865	-	yaaJ	putative transporter yaaJ
i02_0008	CDS	8144	9097	+	talB	transaldolase B
i02_0009	CDS	9209	9799	+	mogA	molybdenum cofactor biosynthesis protein
i02_0010	CDS	10004	10570	-	yaaH	hypothetical protein
i02_0011	CDS	10719	11432	-	yaaW	hypothetical protein
i02_0012	CDS	11458	11862	-	yaaI	hypothetical protein
i02_0013	CDS	12234	14150	+	dnaK	molecular chaperone DnaK
i02_0014	CDS	14239	15369	+	dnaJ	chaperone protein DnaJ
i02_0015	CDS	16241	17002	+	/	hypothetical protein
i02_0016	CDS	16986	18515	+	/	hypothetical protein
i02_0017	CDS	18638	19903	+	/	hypothetical protein
i02_0018	CDS	20138	21304	+	nhaA	pH-dependent sodium/proton antiporter
i02_0019	CDS	21364	22269	+	nhaR	transcriptional activator NhaR
i02_0020	CDS	22236	22364	-	/	hypothetical protein
i02_0021	CDS	22365	22628	-	rpsT	30S ribosomal protein S20
i02_0022	CDS	22731	22949	+	yaaY	hypothetical protein
i02_0023	CDS	22957	23898	+	ribF	bifunctional riboflavin kinase/FMN
i02_0024	CDS	23941	26757	+	ileS	isoleucyl-tRNA synthetase
i02_0025	CDS	26757	27251	+	lspA	lipoprotein signal peptidase
i02_0026	CDS	27359	27808	+	slpA	FKBP-type 16 kDa peptidyl-prolyl cis-trans
i02_0027	CDS	27810	28760	+	ispH	1-hydroxy-2-methyl-2-(E)-butenyl 4-diphosphate reductase
i02_0028	pseudo	28826	29740	+	rihC	ribonucleoside hydrolase RihC
i02_0029	CDS	29763	30128	+	/	hypothetical protein
i02_0030	CDS	30289	31110	+	dapB	dihydrodipicolinate reductase
i02_0031	CDS	31152	31319	-	/	hypothetical protein
i02_0032	CDS	31389	31526	+	/	hypothetical protein
i02_0033	CDS	31539	32714	+	carA	carbamoyl phosphate synthase small subunit
i02_0034	CDS	32732	35953	+	carB	carbamoyl phosphate synthase large subunit
i02_0035	CDS	35961	36179	-	/	hypothetical protein
i02_0036	CDS	36109	36609	+	caiF	DNA-binding transcriptional activator CaiF
i02_0037	CDS	36728	37339	-	caiE	carnitine operon protein CaiE
i02_0038	CDS	37324	38109	-	caiD	carnitinyl-CoA dehydratase
i02_0039	CDS	38218	39786	-	caiC	putative crotonobetaine/carnitine-CoA ligase
i02_0040	CDS	39844	41061	-	caiB	crotonobetainyl-CoA:carnitine CoA-transferase
i02_0041	CDS	41189	42331	-	caiA	crotonobetainyl-CoA dehydrogenase
i02_0042	CDS	42362	43876	-	caiT	L-carnitine/gamma-butyrobetaine antiporter
i02_0043	CDS	44286	45119	+	fixA	putative electron transfer flavoprotein FixA
i02_0044	CDS	45134	46075	+	fixB	putative electron transfer flavoprotein FixB
i02_0045	CDS	46098	47384	+	fixC	putative oxidoreductase FixC
i02_0046	CDS	47381	47668	+	fixX	ferredoxin-like protein
i02_0047	CDS	47727	49058	+	yaaU	metabolite transporter
i02_0048	CDS	49166	49696	+	yabF	glutathione-regulated potassium-efflux system
i02_0049	CDS	49617	51551	+	kefC	glutathione-regulated potassium-efflux system
i02_0050	CDS	51608	52222	+	folA	dihydrofolate reductase
i02_0051	CDS	52308	52541	+	/	putative antitoxin of gyrase inhibiting
i02_0052	CDS	52544	52858	+	/	putative toxin of gyrase inhibiting
i02_0053	CDS	52855	53703	-	apaH	diadenosine tetraphosphatase

Locus_tag	Type	start	End	+/-[a]	Gene[b]	Product
i02_0054	CDS	53710	54087	-	apaG	ApaG
i02_0055	CDS	54090	54911	-	ksgA	dimethyladenosine transferase
i02_0056	CDS	54908	55897	-	pdxA	4-hydroxythreonine-4-phosphate dehydrogenase
i02_0057	CDS	55897	57183	-	surA	peptidyl-prolyl cis-trans isomerase SurA
i02_0058	CDS	57236	59590	-	imp	organic solvent tolerance protein
i02_0059	CDS	59752	60660	+	djlA	Dna-J like membrane chaperone protein
i02_0060	CDS	60778	61437	-	yabO	23S rRNA/tRNA pseudouridine synthase A
i02_0061	CDS	61449	64355	-	hepA	ATP-dependent helicase HepA
i02_0062	CDS	64520	66871	-	polB	DNA polymerase II
i02_0063	CDS	66946	67641	-	araD	L-ribulose-5-phosphate 4-epimerase
i02_0064	CDS	67926	69428	-	araA	L-arabinose isomerase
i02_0065	CDS	69439	71139	-	araB	ribulokinase
i02_0066	CDS	71427	72323	+	araC	DNA-binding transcriptional regulator AraC
i02_0067	CDS	72588	72710	+	/	hypothetical protein
i02_0068	CDS	72834	73070	+	/	hypothetical protein
i02_0069	CDS	73112	73684	+	/	hypothetical protein
i02_0070	CDS	73691	74113	+	/	hypothetical protein
i02_0071	CDS	74268	75032	+	yabI	hypothetical protein
i02_0072	CDS	75146	75844	-	thiQ	thiamine transporter ATP-binding subunit
i02_0073	CDS	75828	77438	-	thiP	thiamine transporter membrane protein
i02_0074	CDS	77414	78409	-	tbpA	thiamine transporter substrate binding subunit
i02_0075	CDS	78441	78560	-	/	hypothetical protein
i02_0076	CDS	78561	80237	-	yabN	transcriptional regulator SgrR
i02_0077	CDS	80305	80436	+	/	hypothetical protein
i02_0078	CDS	80544	81149	-	leuD	isopropylmalate isomerase small subunit
i02_0079	CDS	81160	82560	-	leuC	isopropylmalate isomerase large subunit
i02_0080	CDS	82563	83657	-	leuB	3-isopropylmalate dehydrogenase
i02_0081	CDS	83654	85267	-	leuA	2-isopropylmalate synthase
i02_0082	CDS	85316	85402	-	leuL	leu operon leader peptide
i02_0083	CDS	85976	87004	+	leuO	leucine transcriptional activator
i02_0084	CDS	87040	87168	-	/	hypothetical protein
i02_0085	CDS	87232	89046	+	ilvI	acetolactate synthase 3 catalytic subunit
i02_0086	CDS	89019	89540	+	ilvH	acetolactate synthase 3 regulatory subunit
i02_0087	CDS	89592	89723	+	/	hypothetical protein
i02_0088	CDS	89720	90724	+	fruR	DNA-binding transcriptional regulator FruR
i02_0089	CDS	90886	91002	-	/	hypothetical protein
i02_0090	CDS	91290	91784	+	yabB	cell division protein MraZ
i02_0091	CDS	91687	92727	+	mraW	S-adenosyl-methyltransferase MraW
i02_0092	CDS	92724	93089	+	ftsL	cell division protein FtsL
i02_0093	CDS	93105	94871	+	ftsI	peptidoglycan synthetase ftsI precursor
i02_0094	CDS	94858	96345	+	murE	UDP-N-acetylmuramoylalanyl-D-glutamate 2,6-diaminopimelate ligase
i02_0095	CDS	96342	97700	+	murF	D-alanyl-D-alanine-adding enzyme
i02_0096	CDS	97694	98776	+	mraY	phospho-N-acetylmuramoyl-pentapeptide transferase
i02_0097	CDS	98779	100095	+	murD	UDP-N-acetylmuramoyl-L-alanine:D-glutamate ligase
i02_0098	CDS	100092	101339	+	ftsW	cell division protein FtsW
i02_0099	CDS	101336	102403	+	murG	N-acetylglucosaminyl transferase
i02_0100	CDS	102457	103932	+	murC	UDP-N-acetylmuramate--L-alanine ligase
i02_0101	CDS	103925	104845	+	ddl	D-alanine--D-alanine ligase
i02_0102	CDS	104847	105677	+	ftsQ	cell division protein FtsQ
i02_0103	CDS	105674	106936	+	ftsA	cell division protein FtsA
i02_0104	CDS	106997	108148	+	ftsZ	cell division protein FtsZ
i02_0105	CDS	108249	109166	+	lpxC	UDP-3-O-[3-hydroxymyristoyl] N-acetylglucosamine
i02_0106	CDS	109322	109909	+	yacA	SecA regulator SecM
i02_0107	CDS	109971	112676	+	secA	preprotein translocase subunit SecA

Locus_tag	Type	start	End	+/-[a]	Gene[b]	Product
i02_0108	CDS	112736	113134	+	mutT	nucleoside triphosphate pyrophosphohydrolase
i02_0109	CDS	113228	113758	+	/	hypothetical protein
i02_0110	CDS	113858	114580	+	/	transposase insK
i02_0111	CDS	114653	114850	-	yacG	zinc-binding protein
i02_0112	CDS	114860	115603	-	yacF	hypothetical protein
i02_0113	CDS	115603	116223	-	coaE	dephospho-CoA kinase
i02_0114	CDS	116448	117491	+	guaC	guanosine 5'-monophosphate oxidoreductase
i02_0115	CDS	117526	118728	-	hofC	type IV pilin biogenesis protein
i02_0116	CDS	118718	120103	-	hofB	hypothetical protein
i02_0117	CDS	120113	120553	-	ppdD	putative major pilin subunit
i02_0118	CDS	120757	121692	-	nadC	quinolinate phosphoribosyltransferase
i02_0119	CDS	121738	122289	+	ampD	N-acetyl-anhydromuranmyl-L-alanine amidase
i02_0120	CDS	122286	123140	+	ampE	regulatory protein AmpE
i02_0121	CDS	123183	124553	-	aroP	aromatic amino acid transporter
i02_0122	CDS	125081	126862	+	usp	uropathogenic specific protein
i02_0123	CDS	126862	127158	+	/	hypothetical protein
i02_0124	CDS	127132	127275	-	/	hypothetical protein
i02_0125	CDS	127556	127849	+	/	hypothetical protein
i02_0126	CDS	127833	127955	-	/	hypothetical protein
i02_0127	CDS	128245	128535	+	/	hypothetical protein
i02_0128	CDS	128991	129755	+	pdhR	transcriptional regulator PdhR
i02_0129	CDS	129752	129934	-	/	hypothetical protein
i02_0130	CDS	129916	132579	+	aceE	pyruvate dehydrogenase subunit E1
i02_0131	CDS	132594	134486	+	aceF	dihydrolipoamide acetyltransferase
i02_0132	CDS	134691	136118	+	lpdA	dihydrolipoamide dehydrogenase
i02_0133	CDS	136360	138141	-	yacH	hypothetical protein
i02_0134	CDS	138343	141093	+	acnB	bifunctional aconitate hydratase
i02_0135	CDS	141220	141630	+	yacL	hypothetical protein
i02_0136	CDS	141668	142444	-	speD	S-adenosylmethionine decarboxylase
i02_0137	CDS	142478	143344	-	speE	spermidine synthase
i02_0138	CDS	143450	143968	-	yacC	hypothetical protein
i02_0139	CDS	143963	145519	+	yacK	multicopper oxidase
i02_0140	CDS	145597	148005	-	gcd	glucose dehydrogenase
i02_0141	CDS	148154	148729	+	hpt	hypoxanthine-guanine phosphoribosyltransferase
i02_0142	CDS	148770	149432	-	yadF	carbonic anhydrase
i02_0143	CDS	149541	150467	+	yadG	ABC transporter ATP-binding protein
i02_0144	CDS	150464	151234	+	yadH	hypothetical protein
i02_0145	CDS	151339	151779	+	yadI	putative PTS system IIA component yadI
i02_0146	CDS	151843	153072	+	yadE	hypothetical protein
i02_0147	CDS	153076	153456	-	panD	aspartate alpha-decarboxylase
i02_0148	CDS	153424	153609	+	/	hypothetical protein
i02_0149	CDS	153730	154686	+	yadD	hypothetical protein
i02_0150	CDS	154755	154952	+	/	hypothetical protein
i02_0151	CDS	155034	155885	-	panC	pantoate--beta-alanine ligase
i02_0152	CDS	155897	156691	-	panB	3-methyl-2-oxobutanoate
i02_0153	CDS	156803	158092	-	yadC	putative fimbrial-like adhesin protein
i02_0154	CDS	158118	158714	-	yadK	hypothetical protein
i02_0155	CDS	158741	159355	-	yadL	hypothetical protein
i02_0156	CDS	159361	159927	-	yadM	hypothetical protein
i02_0157	CDS	159944	162532	-	htrE	putative outer membrane usher protein
i02_0158	CDS	162567	163307	-	ecpD	putative chaperone protein EcpD
i02_0159	CDS	163416	164030	-	yadN	fimbrial-like protein yadN precursor
i02_0160	CDS	164370	164849	-	folK	2-amino-4-hydroxy-6-hydroxymethyldihyropteridine
i02_0161	CDS	164846	166264	-	pcnB	poly(A) polymerase I
i02_0162	CDS	166303	167199	-	yadB	glutamyl-Q tRNA(Asp) synthetase
i02_0163	CDS	167266	167739	-	dksA	DnaK transcriptional regulator DksA
i02_0164	CDS	167899	168603	-	sfsA	sugar fermentation stimulation protein A
i02_0165	CDS	168618	169361	-	yadP	2'-5' RNA ligase

Locus_tag	Type	start	End	+/-[a]	Gene[b]	Product
i02_0166	CDS	169177	171651	+	hrpB	ATP-dependent RNA helicase HrpB
i02_0167	CDS	171579	171746	-	/	hypothetical protein
i02_0168	CDS	171745	174279	+	mrcB	penicillin-binding protein 1b
i02_0169	CDS	174344	174526	-	/	hypothetical protein
i02_0170	CDS	174499	176757	+	fhuA	ferrichrome outer membrane transporter
i02_0171	CDS	176808	177605	+	fhuC	iron-hydroxamate transporter ATP-binding subunit
i02_0172	CDS	177602	178495	+	fhuD	iron-hydroxamate transporter substrate-binding subunit
i02_0173	CDS	178492	180474	+	fhuB	iron-hydroxamate transporter permease subunit
i02_0174	CDS	180509	181789	-	hemL	glutamate-1-semialdehyde aminotransferase
i02_0175	CDS	182014	183435	+	yadQ	chloride channel protein
i02_0176	CDS	183484	183861	+	yadR	iron-sulfur cluster insertion protein ErpA
i02_0177	CDS	183613	183873	-	/	hypothetical protein
i02_0178	CDS	183908	184531	-	yadS	hypothetical protein
i02_0179	CDS	184569	185369	-	yadT	vitamin B12-transporter protein BtuF
i02_0180	CDS	185362	186060	-	pfs	5'-methylthioadenosine/S-adenosylhomocysteine
i02_0181	CDS	186144	187661	+	dgt	deoxyguanosinetriphosphate triphosphohydrolase
i02_0182	CDS	187791	189215	+	htrA	serine endoprotease
i02_0183	CDS	189370	190527	+	yaeG	carbohydrate diacid transcriptional activator
i02_0184	CDS	190580	190966	-	yaeH	hypothetical protein
i02_0185	CDS	191278	192102	-	dapD	2,3,4,5-tetrahydropyridine-2,6-carboxylate
i02_0186	CDS	192133	194805	-	glnD	PII uridylyl-transferase
i02_0187	CDS	194867	195661	-	map	methionine aminopeptidase
i02_0188	CDS	196029	196754	+	rpsB	30S ribosomal protein S2
i02_0189	CDS	196706	197635	-	/	hypothetical protein
i02_0190	CDS	196889	197740	+	tsf	elongation factor Ts
i02_0191	CDS	197887	198612	+	pyrH	uridylate kinase
i02_0192	CDS	198762	199319	+	frr	ribosome recycling factor
i02_0193	CDS	199411	200607	+	dxr	1-deoxy-D-xylulose 5-phosphate reductoisomerase
i02_0194	CDS	200793	201554	+	yaeS	undecaprenyl pyrophosphate synthase
i02_0195	CDS	201567	202424	+	cdsA	CDP-diglyceride synthase
i02_0196	CDS	202391	203788	+	yaeL	zinc metallopeptidase RseP
i02_0197	CDS	203818	206250	+	yaeT	outer membrane protein assembly factor YaeT
i02_0198	CDS	206372	206857	+	hlpA	periplasmic chaperone
i02_0199	CDS	206861	207886	+	lpxD	UDP-3-O-[3-hydroxymyristoyl] glucosamine
i02_0200	CDS	207898	208446	+	fabZ	(3R)-hydroxymyristoyl-ACP dehydratase
i02_0201	CDS	208450	209238	+	lpxA	UDP-N-acetylglucosamine acyltransferase
i02_0202	CDS	209238	210386	+	lpxB	lipid-A-disaccharide synthase
i02_0203	CDS	210383	210979	+	rnhB	ribonuclease HII
i02_0204	CDS	211016	214498	+	dnaE	DNA polymerase III subunit alpha
i02_0205	CDS	214511	215470	+	accA	acetyl-CoA carboxylase carboxyltransferase
i02_0206	CDS	215568	217709	+	ldcC	lysine decarboxylase
i02_0207	CDS	217739	218155	+	yaeR	hypothetical protein
i02_0208	CDS	218220	219533	+	tilS	tRNA(Ile)-lysidine synthetase
i02_0209	CDS	219567	219827	-	yaeO	Rho-binding antiterminator
i02_0210	CDS	219814	220014	-	/	hypothetical protein
i02_0211	CDS	220180	220725	+	yaeQ	hypothetical protein
i02_0212	CDS	220722	221144	+	yaeJ	peptidyl-tRNA hydrolase domain protein
i02_0213	CDS	221158	221868	+	cutF	outer membrane lipoprotein NlpE, involved in surface sensing
i02_0214	CDS	222016	222738	-	/	transposase insK
i02_0215	CDS	222838	223368	-	/	hypothetical protein
i02_0216	CDS	223454	224278	-	yaeF	hypothetical protein
i02_0217	CDS	224331	226049	-	proS	prolyl-tRNA synthetase
i02_0218	CDS	226160	226867	-	yaeB	hypothetical protein
i02_0219	CDS	226864	227268	-	rcsF	outer membrane lipoprotein
i02_0220	CDS	227386	228201	-	metQ	DL-methionine transporter substrate-binding

Locus_tag	Type	start	End	+/-[a]	Gene[b]	Product
i02_0221	CDS	228241	228894	-	yaeE	DL-methionine transporter permease subunit
i02_0222	CDS	228887	229918	-	metN	DL-methionine transporter ATP-binding subunit
i02_0223	CDS	230106	230678	+	yaeD	D,D-heptose 1,7-bisphosphate phosphatase
i02_0224	CDS	230826	230987	-	/	hypothetical protein
i02_0226	CDS	236327	237130	+	dkgB	2,5-diketo-D-gluconate reductase B
i02_0227	CDS	237127	238041	-	yafC	putative transcriptional regulator YafC
i02_0228	CDS	238258	239082	+	yafD	hypothetical protein
i02_0229	CDS	239160	239930	+	yafE	hypothetical protein
i02_0230	CDS	239979	241337	-	mltD	membrane-bound lytic murein transglycosylase D
i02_0231	CDS	241409	242164	-	gloB	hydroxyacylglutathione hydrolase
i02_0232	CDS	242180	242920	+	yafS	hypothetical protein
i02_0233	CDS	242917	243495	-	rnhA	ribonuclease H
i02_0234	CDS	243440	244180	+	dnaQ	DNA polymerase III subunit epsilon
i02_0235	CDS	244429	244773	+	/	hypothetical protein
i02_0236	CDS	244921	245367	-	/	hypothetical protein
i02_0237	CDS	245682	245903	+	/	hypothetical protein
i02_0238	CDS	245973	246323	+	/	hypothetical protein
i02_0239	CDS	246354	247001	+	/	hypothetical protein
i02_0240	CDS	246952	247902	+	/	hypothetical protein
i02_0241	CDS	248012	248950	-	/	hypothetical protein
i02_0242	CDS	248922	249236	-	/	hypothetical protein
i02_0243	CDS	249379	250323	+	/	putative integrase of prophage
i02_0244	CDS	250486	250611	-	/	hypothetical protein
i02_0245	CDS	250932	252122	+	/	hypothetical protein
i02_0246	CDS	252142	252348	+	/	hypothetical protein
i02_0247	CDS	252338	252574	+	/	hypothetical protein
i02_0248	CDS	252567	252779	+	/	hypothetical protein
i02_0249	CDS	252994	253293	+	/	hypothetical protein
i02_0250	CDS	253290	254705	+	/	hypothetical protein
i02_0251	CDS	254946	255158	+	/	hypothetical protein
i02_0252	CDS	255155	255577	+	/	putative single stranded DNA-binding protein
i02_0253	CDS	255993	256199	+	/	hypothetical protein
i02_0254	CDS	256199	257254	+	/	putative capsid protein of prophage
i02_0255	CDS	257266	257601	+	/	hypothetical protein
i02_0256	CDS	257614	258027	+	/	hypothetical protein
i02_0257	CDS	258100	258234	+	/	conserved hypothetical protein
i02_0258	CDS	258234	258776	+	/	gp1
i02_0259	CDS	258850	259131	+	/	hypothetical protein
i02_0260	CDS	259740	259994	-	/	hypothetical protein
i02_0261	CDS	260058	260243	+	/	hypothetical protein
i02_0262	CDS	260391	261077	+	/	putative transposase
i02_0263	CDS	261125	261379	+	/	hypothetical protein
i02_0264	CDS	261458	261805	-	/	hypothetical protein
i02_0265	CDS	261947	262324	-	/	hypothetical protein
i02_0266	CDS	262371	262826	-	/	hypothetical protein
i02_0267	CDS	262795	263439	-	/	hypothetical protein
i02_0268	CDS	263742	264218	-	yeeS	putative radC-like protein YeeS
i02_0269	CDS	264234	264779	-	/	hypothetical protein
i02_0270	CDS	264801	265025	-	/	hypothetical protein
i02_0271	CDS	265046	265867	-	/	hypothetical protein
i02_0272	CDS	265967	266200	-	/	hypothetical protein
i02_0273	CDS	266318	266803	-	/	hypothetical protein
i02_0274	CDS	266807	266926	-	/	hypothetical protein
i02_0275	CDS	266926	267174	-	/	hypothetical protein
i02_0276	CDS	267205	267843	-	/	hypothetical protein
i02_0277	CDS	267890	268396	-	/	hypothetical protein
i02_0278	CDS	268486	269556	-	/	hypothetical protein
i02_0279	CDS	269553	270458	-	/	hypothetical protein

Locus_tag	Type	start	End	+/-[a]	Gene[b]	Product
i02_0280	CDS	270455	272851	-	/	hypothetical protein
i02_0281	CDS	273069	273566	-	/	hypothetical protein
i02_0282	CDS	273725	274828	-	/	hypothetical protein
i02_0283	CDS	275218	275826	+	/	hypothetical protein
i02_0284	CDS	276054	276791	+	/	conserved hypothetical protein
i02_0285	CDS	276909	277718	-	/	hypothetical protein
i02_0286	CDS	278201	280366	+	/	TonB dependent receptor
i02_0287	CDS	280374	281366	+	/	periplasmic binding protein
i02_0288	CDS	281385	282443	+	/	ABC transporter
i02_0289	CDS	282440	283207	+	/	putative ABC transporter
i02_0290	CDS	284056	284424	+	/	hypothetical protein
i02_0291	CDS	284069	284494	+	/	hypothetical protein
i02_0292	CDS	285026	285211	+	/	hypothetical protein
i02_0293	CDS	285312	285449	+	/	hypothetical protein
i02_0294	CDS	285358	285501	-	/	hypothetical protein
i02_0295	CDS	285948	286148	+	/	hypothetical protein
i02_0296	CDS	286051	286659	-	/	hypothetical protein
i02_0297	CDS	287337	288128	-	/	hypothetical protein
i02_0298	CDS	289203	289343	+	/	hemolysin expression modulating protein
i02_0299	CDS	289575	290174	+	/	hypothetical protein
i02_0300	CDS	290540	290929	+	/	hypothetical protein
i02_0301	CDS	290926	291351	+	/	hypothetical protein
i02_0302	CDS	291553	291795	+	/	hypothetical protein
i02_0303	CDS	291823	292389	+	/	hypothetical protein
i02_0304	CDS	292442	292597	+	/	hypothetical protein
i02_0305	CDS	292494	292709	+	/	hypothetical protein
i02_0306	CDS	292615	292905	-	/	hypothetical protein
i02_0307	CDS	293294	293932	-	/	hypothetical protein
i02_0308	CDS	294201	295373	+	/	putative oligogalacturonide lyase
i02_0309	CDS	295403	296203	+	/	gluconate 5-dehydrogenase
i02_0310	CDS	296947	298461	+	/	putative oligogalacturonide transporter
i02_0311	CDS	298391	300697	+	/	putative exopolygalacturonate lyase
i02_0313	CDS	300790	301770	+	/	hypothetical protein
i02_0314	CDS	302090	302518	-	/	hypothetical protein
i02_0315	CDS	302861	303331	-	/	hypothetical protein
i02_0316	CDS	303837	304076	-	/	hypothetical protein
i02_0317	CDS	304206	304331	+	/	hypothetical protein
i02_0318	CDS	304858	305643	-	/	putative deoxyribose operon repressor
i02_0319	CDS	305946	306866	+	/	putative ribokinase
i02_0320	CDS	306894	308210	+	/	putative L-fucose permease
i02_0321	CDS	308222	309235	+	/	putative cytoplasmic protein
i02_0322	CDS	310158	311414	-	ulaA	ascorbate-specific PTS system enzyme IIC
i02_0323	CDS	311427	311714	-	/	hypothetical protein
i02_0324	CDS	311730	312173	-	/	PTS system, mannitol (Cryptic)-specific IIA
i02_0325	CDS	312444	313475	+	/	hypothetical protein
i02_0326	CDS	313762	313893	-	/	hypothetical protein
i02_0327	CDS	313978	314130	-	/	hypothetical protein
i02_0328	CDS	314194	314853	+	/	hypothetical protein
i02_0329	CDS	314844	315032	-	/	hypothetical protein
i02_0330	CDS	316536	316847	+	/	hypothetical protein
i02_0331	CDS	316860	326510	+	/	ShlA/HecA/FhaA exofamily protein
i02_0332	CDS	328295	329419	+	/	transposase
i02_0333	CDS	329903	330175	-	/	PefB protein
i02_0334	CDS	330422	334537	-	/	Pic serine protease precursor
i02_0335	CDS	334604	334765	+	/	hypothetical protein
i02_0336	CDS	337392	337775	+	/	hypothetical protein
i02_0337	CDS	337772	338251	+	/	hypothetical protein
i02_0339	CDS	338958	340193	+	/	hypothetical protein

Locus_tag	Type	start	End	+/-[a]	Gene[b]	Product
i02_0340	CDS	340218	342365	+	/	putative cytoplasmic membrane export protein
i02_0341	CDS	342411	343673	+	/	putative membrane spanning export protein
i02_0342	CDS	343872	347699	+	/	RTX family exoprotein A gene
i02_0343	CDS	347820	347939	+	/	hypothetical protein
i02_0344	CDS	348302	348832	+	/	hypothetical protein
i02_0345	CDS	348783	349052	-	/	hypothetical protein
i02_0346	CDS	349193	349669	-	/	hypothetical protein
i02_0347	CDS	349736	349945	+	/	hypothetical protein
i02_0348	CDS	350047	350247	+	/	hypothetical protein
i02_0349	CDS	350519	351298	-	yafV	hypothetical protein
i02_0350	CDS	351443	351916	+	ykfE	C-lysozyme inhibitor
i02_0351	CDS	351959	354403	-	fadE	acyl-CoA dehydrogenase
i02_0352	CDS	354481	355221	+	gmhA	phosphoheptose isomerase
i02_0353	CDS	355411	356193	+	yafJ	hypothetical protein
i02_0354	CDS	356164	356904	-	yafK	hypothetical protein
i02_0355	CDS	357207	357965	+	yafL	lipoprotein yafL precursor
i02_0356	CDS	358240	359979	-	fhiA	FhiA protein
i02_0357	CDS	359921	360709	+	mbhA	hypothetical protein
i02_0358	CDS	360780	361835	+	dinP	DNA polymerase IV
i02_0359	CDS	361832	362284	+	yafP	hypothetical protein
i02_0360	CDS	362462	363613	+	/	hypothetical protein
i02_0361	CDS	363526	364224	+	prfH	peptide chain release factor-like protein
i02_0362	CDS	364281	365738	-	pepD	aminoacyl-histidine dipeptidase
i02_0363	CDS	365999	366457	+	gpt	xanthine-guanine phosphoribosyltransferase
i02_0364	CDS	366131	366583	-	/	hypothetical protein
i02_0365	CDS	366549	367793	+	frsA	fermentation/respiration switch protein
i02_0366	CDS	367851	368252	+	crl	DNA-binding transcriptional regulator Crl
i02_0367	CDS	368291	369352	-	phoE	outer membrane phosphoporin protein
i02_0368	CDS	369635	370738	+	proB	gamma-glutamyl kinase
i02_0369	CDS	370750	372003	+	proA	gamma-glutamyl phosphate reductase
i02_0370	CDS	372348	372686	+	/	CP4-like integrase
i02_0371	CDS	373121	373645	-	/	hypothetical protein
i02_0372	pseudo	373771	377903	-	/	vacuolating autotransporter toxin
i02_0373	CDS	378216	378413	+	/	hypothetical protein
i02_0374	CDS	378331	378456	+	/	hypothetical protein
i02_0375	CDS	378496	378711	+	/	insertion element IS1 1/2/3/5/6 protein insA
i02_0376	CDS	378902	379177	-	/	hypothetical protein
i02_0377	CDS	379013	379189	+	/	InsB protein
i02_0378	CDS	379568	379750	+	/	hypothetical protein
i02_0379	CDS	380013	380627	+	yagU	hypothetical protein
i02_0380	CDS	380876	381205	-	ykgJ	putative ferredoxin
i02_0381	CDS	381512	382267	-	yagV	hypothetical protein
i02_0382	CDS	382191	383834	-	yagW	hypothetical protein
i02_0383	CDS	383824	386349	-	yagX	hypothetical protein
i02_0384	CDS	386375	387091	-	yagY	hypothetical protein
i02_0385	CDS	387100	387687	-	matB	hypothetical protein
i02_0386	CDS	387762	388352	-	matA	hypothetical protein
i02_0387	CDS	388776	389066	+	/	hypothetical protein
i02_0388	CDS	389388	389531	-	rpmJ	50S ribosomal protein L36
i02_0389	CDS	389528	389794	-	rpmE2	50S ribosomal protein L31 type B
i02_0390	CDS	389832	389963	+	/	hypothetical protein
i02_0391	CDS	390730	391872	-	/	putative oxidoreductase
i02_0392	CDS	392044	392964	-	/	hypothetical protein
i02_0393	CDS	393058	394047	+	/	LysR family transcriptional regulator
i02_0394	CDS	394172	395140	+	/	putative transcriptional regulator YcjZ
i02_0395	CDS	395171	396160	-	/	putative aldo/keto reductase
i02_0396	CDS	396187	397038	-	/	2,5-diketo-D-gluconic acid reductase A
i02_0397	CDS	397604	401854	+	eaeH	putative adhesin

Locus_tag	Type	start	End	+/-[a]	Gene[b]	Product
i02_0398	CDS	401979	402869	-	ykgA	putative transcriptional regulator YkgA
i02_0399	CDS	403069	403953	+	/	2,5-diketo-D-gluconic acid reductase A
i02_0400	CDS	404113	404715	-	ykgB	hypothetical protein
i02_0401	CDS	404718	404969	-	ykgI	hypothetical protein
i02_0402	CDS	405063	406415	-	ykgC	pyridine nucleotide-disulfide oxidoreductase
i02_0403	CDS	406564	407469	+	ykgD	putative transcriptional regulator YkgD
i02_0404	CDS	408016	408714	+	ykgE	hypothetical protein
i02_0405	CDS	408725	410152	+	ykgF	putative electron transport protein ykgF
i02_0407	CDS	409992	410840	+	ykgG	hypothetical protein
i02_0408	CDS	411083	411751	-	ykgH	hypothetical protein
i02_0409	CDS	411935	414265	-	/	hypothetical protein
i02_0410	CDS	414274	415035	-	/	hypothetical protein
i02_0411	CDS	415052	415192	-	/	hypothetical protein
i02_0412	CDS	415189	415983	-	/	hypothetical protein
i02_0413	CDS	415865	416020	-	/	hypothetical protein
i02_0414	CDS	416042	416644	-	/	Type 1 fimbriae regulatory protein fimB
i02_0415	CDS	417367	417648	+	/	hypothetical protein
i02_0416	CDS	417690	419360	-	betA	choline dehydrogenase
i02_0417	CDS	419374	420849	-	betB	betaine aldehyde dehydrogenase
i02_0418	CDS	420860	421465	-	betI	transcriptional regulator BetI
i02_0419	CDS	421576	423609	+	betT	choline transport protein BetT
i02_0420	CDS	424479	425573	+	/	hypothetical protein
i02_0421	CDS	425615	426547	-	yahB	putative transcriptional regulator YahB
i02_0422	CDS	426639	427136	-	yahC	hypothetical protein
i02_0423	CDS	427213	427455	+	/	hypothetical protein
i02_0424	CDS	427394	427999	+	yahD	ankyrin repeat-containing protein
i02_0425	CDS	428039	428902	+	yahE	hypothetical protein
i02_0426	CDS	428892	430439	+	/	hypothetical protein
i02_0427	CDS	430439	431857	+	/	hypothetical protein
i02_0428	CDS	432187	433137	+	/	putative carbamate kinase
i02_0429	CDS	433147	434529	+	yahJ	deaminase
i02_0430	CDS	434751	434885	-	/	hypothetical protein
i02_0431	CDS	434906	435955	+	yahK	hypothetical protein
i02_0432	CDS	436837	437511	-	yahN	hypothetical protein
i02_0433	CDS	437658	437933	+	yahO	hypothetical protein
i02_0434	CDS	438034	439620	-	prpR	propionate catabolism operon regulatory protein
i02_0435	CDS	439859	440749	+	prpB	2-methylisocitrate lyase
i02_0436	CDS	440909	442078	+	prpC	methylcitrate synthase
i02_0437	CDS	442112	443563	+	prpD	2-methylcitrate dehydratase
i02_0438	CDS	443603	445489	+	prpE	propionyl-CoA synthetase
i02_0439	CDS	445814	447073	+	codB	cytosine permease
i02_0440	CDS	447048	448346	+	codA	cytosine deaminase
i02_0441	CDS	448467	449129	-	lacA	galactoside O-acetyltransferase
i02_0442	CDS	449144	450397	-	lacY	galactoside permease
i02_0443	CDS	450449	453523	-	lacZ	beta-D-galactosidase
i02_0444	CDS	453646	454737	-	lacI	lac repressor
i02_0445	CDS	454930	455469	+	yaiL	hypothetical protein
i02_0446	CDS	455697	456530	-	yaiM	hypothetical protein
i02_0447	CDS	456623	457732	-	adhC	alcohol dehydrogenase class III
i02_0448	CDS	457767	458063	-	yaiN	regulator protein FrmR
i02_0449	CDS	458229	459002	-	/	hypothetical protein
i02_0450	CDS	459004	459714	-	/	putative transferase
i02_0451	CDS	459563	460759	-	/	hypothetical protein
i02_0452	CDS	460769	461455	-	/	hypothetical protein
i02_0453	CDS	461900	462094	-	/	hypothetical protein
i02_0454	CDS	461991	463010	+	tauA	taurine transporter substrate binding subunit
i02_0455	CDS	463023	463790	+	tauB	taurine transporter ATP-binding subunit
i02_0457	CDS	463787	464614	+	tauC	taurine transporter subunit

Locus_tag	Type	start	End	+/-[a]	Gene[b]	Product
i02_0458	CDS	464611	465462	+	tauD	taurine dioxygenase
i02_0459	CDS	465502	466509	-	hemB	delta-aminolevulinic acid dehydratase
i02_0460	CDS	467002	469989	+	/	putative structural protein
i02_0461	CDS	470030	470698	+	yaiV	putative DNA-binding transcriptional regulator
i02_0462	CDS	470699	471856	-	yaiH	beta-lactam binding protein AmpH
i02_0463	CDS	471994	472188	+	/	hypothetical protein
i02_0464	CDS	472202	473428	+	sbmA	transport protein
i02_0465	CDS	473441	474535	+	yaiW	hypothetical protein
i02_0466	CDS	474594	474902	-	yaiY	hypothetical protein
i02_0467	CDS	474832	474966	+	/	hypothetical protein
i02_0468	CDS	475030	475374	+	yaiZ	hypothetical protein
i02_0469	CDS	475398	476492	-	ddl	D-alanyl-alanine synthetase A
i02_0470	CDS	476570	476692	+	/	hypothetical protein
i02_0471	CDS	476955	477215	+	yaiB	hypothetical protein
i02_0472	CDS	477247	478731	+	phoA	alkaline phosphatase
i02_0473	CDS	478832	479170	+	psiF	hypothetical protein
i02_0474	CDS	479272	480387	+	adrA	diguanylate cyclase AdrA
i02_0475	CDS	480404	481213	-	proC	pyrroline-5-carboxylate reductase
i02_0476	CDS	481333	481791	+	yaiI	hypothetical protein
i02_0477	CDS	481974	482498	+	aroL	shikimate kinase II
i02_0479	CDS	482548	482739	+	yaiA	hypothetical protein
i02_0480	CDS	482997	483674	+	aroM	hypothetical protein
i02_0481	CDS	483746	484030	+	yaiE	hypothetical protein
i02_0482	CDS	484134	484289	-	/	hypothetical protein
i02_0483	CDS	484238	484519	+	ykiA	hypothetical protein
i02_0484	CDS	484677	485588	-	rdgC	recombination associated protein
i02_0485	CDS	485587	486621	+	/	fructokinase
i02_0486	CDS	486890	488158	-	araJ	MFS transport protein AraJ
i02_0487	CDS	488200	491343	-	sbcC	exonuclease subunit SbcC
i02_0488	CDS	491340	492566	-	sbcD	exonuclease subunit SbcD
i02_0489	CDS	492528	492659	+	/	hypothetical protein
i02_0490	CDS	492732	493421	+	phoB	transcriptional regulator PhoB
i02_0491	CDS	493479	494774	+	phoR	phosphate regulon sensor protein
i02_0492	CDS	494987	495136	-	/	hypothetical protein
i02_0493	CDS	495181	496500	+	brnQ	branched chain amino acid ABC transporter
i02_0494	CDS	496573	497949	+	proY	putative proline-specific permease
i02_0495	CDS	498105	499922	+	malZ	maltodextrin glucosidase
i02_0496	CDS	499927	500565	-	yajB	acyl carrier protein phosphodiesterase
i02_0497	CDS	500727	501797	+	queA	S-adenosylmethionine:tRNA
i02_0498	CDS	501852	502979	+	tgt	queuine tRNA-ribosyltransferase
i02_0499	CDS	503002	503334	+	yajC	preprotein translocase subunit YajC
i02_0500	CDS	503362	505209	+	secD	preprotein translocase subunit SecD
i02_0501	CDS	505175	506191	+	secF	preprotein translocase subunit SecF
i02_0502	CDS	506252	506668	+	yajD	hypothetical protein
i02_0503	CDS	506706	507638	-	tsx	nucleoside-specific channel-forming protein tsx
i02_0504	CDS	507889	508488	-	yajI	hypothetical protein
i02_0505	CDS	508579	509028	+	nrdR	transcriptional regulator NrdR
i02_0506	CDS	509032	510135	+	ribD	bifunctional
i02_0507	CDS	510128	510694	+	ribH	6,7-dimethyl-8-ribityllumazine synthase
i02_0508	CDS	510714	511133	+	nusB	transcription antitermination protein NusB
i02_0509	CDS	511211	512188	+	thiL	thiamine monophosphate kinase
i02_0510	CDS	512166	512684	+	pgpA	phosphatidylglycerophosphatase A
i02_0511	CDS	512738	513712	-	yajO	oxidoreductase yajO
i02_0512	CDS	513767	515629	-	dxs	1-deoxy-D-xylulose-5-phosphate synthase
i02_0513	CDS	515654	516553	-	ispA	geranyltranstransferase
i02_0514	CDS	516553	516795	-	xseB	exodeoxyribonuclease VII small subunit
i02_0515	CDS	517001	518449	+	yajK	thiamine biosynthesis protein ThiI
i02_0516	CDS	518503	519099	-	thiJ	hypothetical protein

Locus_tag	Type	start	End	+/-[a]	Gene[b]	Product
i02_0517	CDS	519056	519967	-	apbA	2-dehydropantoate 2-reductase
i02_0518	CDS	520117	520626	+	yajQ	putative nucleotide-binding protein
i02_0519	CDS	520754	522316	-	yajR	putative transport protein YajR
i02_0520	CDS	522267	523202	-	cyoE	protoheme IX farnesyltransferase
i02_0521	CDS	523169	523579	-	cyoD	cytochrome o ubiquinol oxidase subunit IV
i02_0522	CDS	523498	524112	-	cyoC	cytochrome o ubiquinol oxidase subunit III
i02_0523	CDS	524102	526093	-	cyoB	cytochrome o ubiquinol oxidase subunit I
i02_0524	CDS	526115	527062	-	cyoA	cytochrome o ubiquinol oxidase subunit II
i02_0525	CDS	527521	528996	-	ampG	muropeptide transporter
i02_0526	CDS	529040	529720	-	yajG	hypothetical protein
i02_0527	CDS	529668	529799	-	/	hypothetical protein
i02_0528	CDS	529923	530240	+	bolA	transcriptional regulator BolA
i02_0529	CDS	530249	530437	+	/	hypothetical protein
i02_0530	CDS	530584	531882	+	tig	trigger factor
i02_0531	CDS	532128	532751	+	clpP	ATP-dependent Clp protease proteolytic subunit
i02_0532	CDS	532877	534151	+	clpX	ATP-dependent protease ATP-binding subunit ClpX
i02_0533	CDS	534294	536693	+	lon	DNA-binding ATP-dependent protease La
i02_0534	CDS	536902	537174	+	hupB	transcriptional regulator HU subunit beta
i02_0535	CDS	537366	539237	+	ybaU	peptidyl-prolyl cis-trans isomerase (rotamase
i02_0536	CDS	539388	539759	+	ybaV	hypothetical protein
i02_0537	CDS	539865	540263	+	ybaW	hypothetical protein
i02_0538	CDS	540315	541010	-	ybaX	queuosine biosynthesis protein QueC
i02_0539	CDS	541075	542790	-	ybaE	hypothetical protein
i02_0540	CDS	542863	543693	+	cof	Cof protein
i02_0541	CDS	543696	544304	+	ybaO	putative transcriptional regulator YbaO
i02_0542	CDS	544334	546106	+	mdlA	putative multidrug transporter
i02_0543	CDS	546099	547880	+	mdlB	putative multidrug transporter
i02_0544	CDS	547923	548399	+	glnK	nitrogen regulatory protein P-II 2
i02_0546	CDS	548429	549715	+	amtB	ammonium transporter
i02_0547	CDS	549764	550708	-	tesB	acyl-CoA thioesterase II
i02_0548	CDS	550703	550825	+	/	hypothetical protein
i02_0549	CDS	550842	551414	+	ybaY	hypothetical protein
i02_0550	CDS	551445	551855	-	ybaZ	hypothetical protein
i02_0551	CDS	552135	552488	+	ybaA	hypothetical protein
i02_0552	CDS	552530	554086	-	ylaB	hypothetical protein
i02_0553	CDS	554244	554753	-	ylaC	hypothetical protein
i02_0554	CDS	554830	555381	-	ylaD	maltose O-acetyltransferase
i02_0555	CDS	555554	555973	-	hha	hemolysin expression-modulating protein
i02_0556	CDS	555798	556172	-	ybaJ	hypothetical protein
i02_0557	CDS	556717	559866	-	acrB	acriflavin resistance protein B
i02_0558	CDS	559889	561118	-	acrA	acriflavin resistance protein A
i02_0559	CDS	561224	561871	+	acrR	DNA-binding transcriptional repressor AcrR
i02_0560	CDS	561999	565361	+	aefA	potassium efflux protein KefA
i02_0561	CDS	565573	565734	-	ybaM	hypothetical protein
i02_0562	CDS	565748	566275	-	priC	primosomal replication protein N''
i02_0563	CDS	566345	566722	+	ybaN	hypothetical protein
i02_0564	CDS	566821	567426	+	apt	adenine phosphoribosyltransferase
i02_0565	CDS	567555	569486	+	dnaX	DNA polymerase III subunits gamma and tau
i02_0566	CDS	569530	569868	+	ybaB	hypothetical protein
i02_0567	CDS	569868	570473	+	recR	recombination protein RecR
i02_0568	CDS	570583	572457	+	htpG	heat shock protein 90
i02_0569	CDS	572638	573282	+	adk	adenylate kinase
i02_0570	CDS	573414	574376	+	hemH	ferrochelatase
i02_0571	CDS	574373	575332	-	ybaC	acetyl esterase
i02_0572	CDS	575484	576788	+	gsk	inosine-guanosine kinase
i02_0573	CDS	576918	578594	-	ybaL	putative cation:proton antiport protein
i02_0574	CDS	578832	580052	-	fsr	fosmidomycin resistance protein

Locus_tag	Type	start	End	+/-[a]	Gene[b]	Product
i02_0575	CDS	580270	581922	+	ushA	bifunctional UDP-sugar hydrolase/5'-nucleotidase
i02_0576	CDS	581959	582438	-	ybaK	hypothetical protein
i02_0577	CDS	582642	583436	-	ybaP	hypothetical protein
i02_0578	CDS	583685	583870	+	/	hypothetical protein
i02_0579	CDS	583906	584247	+	ybaQ	hypothetical protein
i02_0580	CDS	584305	586809	-	copA	copper exporting ATPase
i02_0581	CDS	587072	588004	+	ybaS	glutaminase
i02_0582	CDS	588007	589299	+	ybaT	putative transport protein YbaT
i02_0583	CDS	589424	589831	+	ybbI	DNA-binding transcriptional regulator CueR
i02_0584	CDS	589832	591055	-	/	hypothetical protein
i02_0585	CDS	591174	591632	-	ybbJ	hypothetical protein
i02_0586	CDS	591629	592546	-	ybbK	hypothetical protein
i02_0587	CDS	592689	593369	+	ybbL	putative ABC transporter ATP-binding protein
i02_0588	CDS	593329	594135	+	ybbM	putative metal resistance protein
i02_0589	CDS	594198	595088	-	ybbN	hypothetical protein
i02_0590	CDS	595113	595922	-	ybbO	short chain dehydrogenase
i02_0591	CDS	595912	596568	-	tesA	multifunctional acyl-CoA thioesterase
i02_0592	CDS	596506	597192	+	ybbA	putative ABC transporter ATP-binding protein
i02_0593	CDS	597189	599603	+	ybbP	hypothetical protein
i02_0594	CDS	599744	600838	-	ybbB	tRNA 2-selenouridine synthase
i02_0595	CDS	600907	601833	-	ybbS	DNA-binding transcriptional activator AllS
i02_0596	CDS	602063	602545	+	ybbT	ureidoglycolate hydrolase
i02_0597	CDS	602623	603438	+	ybbU	DNA-binding transcriptional repressor AllR
i02_0598	CDS	603438	605309	+	gcl	glyoxylate carboligase
i02_0599	CDS	605322	606098	+	gip	hydroxypyruvate isomerase
i02_0600	CDS	606198	607076	+	ybbQ	2-hydroxy-3-oxopropionate reductase
i02_0601	CDS	607246	608700	+	ybbW	allantoin permease
i02_0602	CDS	608760	610121	+	ybbX	allantoinase
i02_0603	CDS	610171	611478	+	ybbY	putative purine permease YbbY
i02_0604	CDS	611494	612645	+	ybbZ	glycerate kinase II
i02_0605	CDS	612774	613559	-	ylbA	hypothetical protein
i02_0606	CDS	613570	614823	-	ylbB	allantoate amidohydrolase
i02_0607	CDS	614827	615876	-	/	ureidoglycolate dehydrogenase
i02_0608	CDS	616193	617860	+	fdrA	membrane protein FdrA
i02_0609	CDS	617870	619129	+	ylbE	hypothetical protein
i02_0610	CDS	619047	619955	+	ylbF	hypothetical protein
i02_0611	CDS	619952	620845	+	arcC	carbamate kinase
i02_0612	CDS	620978	622045	-	purK	phosphoribosylaminoimidazole carboxylase ATPase subunit
i02_0613	CDS	622042	622578	-	purE	phosphoribosylaminoimidazole carboxylase
i02_0614	CDS	622647	622976	-	/	hypothetical protein
i02_0615	CDS	623087	623809	-	ybbF	UDP-2,3-diacylglucosamine hydrolase
i02_0616	CDS	623812	624306	-	ppiB	peptidyl-prolyl cis-trans isomerase B
i02_0617	CDS	624480	625865	+	cysS	cysteinyl-tRNA synthetase
i02_0618	pseudo	625901	626422	-	ybcI	conserved hypothetical protein; putative inner membrane protein
i02_0619	CDS	626530	626742	-	ybcJ	hypothetical protein
i02_0620	CDS	626744	627610	-	folD	bifunctional 5,10-methylene-tetrahydrofolate
i02_0621	CDS	627651	627848	+	/	hypothetical protein
i02_0622	CDS	627965	628345	-	intD	prophage DLP12 integrase
i02_0623	CDS	628293	628700	-	intD	prophage DLP12 integrase
i02_0624	CDS	628599	628955	+	ydfM	tail fiber assembly protein
i02_0625	CDS	629010	629675	-	/	hypothetical protein
i02_0626	CDS	629910	630863	-	ompT	outer membrane protease
i02_0627	CDS	631522	632412	-	ybcH	hypothetical protein
i02_0628	CDS	632413	635406	-	nfrA	bacteriophage N4 receptor, outer membrane
i02_0629	CDS	635372	637609	-	nfrB	bacteriophage N4 adsorption protein B
i02_0630	CDS	637759	639201	-	cusS	sensor kinase CusS

Locus_tag	Type	start	End	+/-[a]	Gene[b]	Product
i02_0631	CDS	639191	639874	-	cusR	DNA-binding transcriptional activator CusR
i02_0632	CDS	640031	641413	+	cusC	copper/silver efflux system outer membrane
i02_0633	CDS	641437	641769	+	cusX	periplasmic copper-binding protein
i02_0634	CDS	641785	643008	+	cusB	copper/silver efflux system membrane fusion
i02_0635	CDS	643020	646163	+	cusA	putative cation efflux system protein cusA
i02_0636	CDS	646229	647647	+	pheP	phenylalanine transporter
i02_0637	CDS	647804	649051	-	ybdG	hypothetical protein
i02_0638	CDS	649159	649812	-	nfnB	dihydropteridine reductase
i02_0639	CDS	649891	650274	-	ybdF	hypothetical protein
i02_0640	CDS	650339	650587	-	ybdJ	hypothetical protein
i02_0641	CDS	650653	651771	-	ybdK	carboxylate-amine ligase
i02_0642	CDS	651985	652254	-	/	hypothetical protein
i02_0643	CDS	652497	653267	-	entD	phosphopantetheinyltransferase
i02_0644	CDS	653292	655532	-	fepA	outer membrane receptor FepA
i02_0645	CDS	655525	655656	+	/	hypothetical protein
i02_0646	CDS	655775	656977	+	fes	enterobactin/ferric enterobactin esterase
i02_0647	CDS	657057	661076	+	entF	enterobactin synthase subunit F
i02_0648	CDS	661323	662456	+	fepE	ferric enterobactin transport protein FepE
i02_0649	CDS	662453	663586	-	fepC	iron-enterobactin transporter ATP-binding component
i02_0650	CDS	663265	664257	-	fepG	iron-enterobactin transporter permease
i02_0651	CDS	664254	665270	-	fepD	iron-enterobactin transporter membrane protein
i02_0652	CDS	665369	666619	+	ybdA	enterobactin exporter EntS
i02_0653	CDS	666623	667579	-	fepB	iron-enterobactin transporter periplasmic
i02_0654	CDS	667756	668943	+	entC	isochorismate synthase
i02_0655	CDS	668953	670563	+	entE	enterobactin synthase subunit E
i02_0656	CDS	670577	671434	+	entB	isochorismatase
i02_0657	CDS	671404	672180	+	entA	2,3-dihydroxybenzoate-2,3-dehydrogenase
i02_0658	CDS	672183	672596	+	ybdB	hypothetical protein
i02_0659	CDS	672777	674882	+	cstA	carbon starvation protein A
i02_0660	CDS	674995	675192	+	/	hypothetical protein
i02_0661	CDS	675202	676290	-	ybdH	hypothetical protein
i02_0662	CDS	676369	677559	+	ybdL	putative aminotransferase
i02_0663	CDS	677560	678189	-	/	hypothetical protein
i02_0664	CDS	678162	679382	-	/	hypothetical protein
i02_0665	CDS	679529	680467	-	ybdO	putative transcriptional regulator YbdO
i02_0666	CDS	680636	681442	-	dsbG	disulfide isomerase/thiol-disulfide oxidase
i02_0667	CDS	681754	682317	+	ahpC	alkyl hydroperoxide reductase subunit C
i02_0668	CDS	682458	684053	+	ahpF	Alkyl hydroperoxide reductase subunit F
i02_0669	CDS	684174	684635	-	ybdQ	hypothetical protein
i02_0670	CDS	684959	685369	-	rnk	nucleoside diphosphate kinase regulator
i02_0671	CDS	685599	686423	-	rna	ribonuclease I
i02_0672	CDS	686519	687982	-	ybdS	citrate transporter
i02_0673	CDS	688033	688911	-	citG	triphosphoribosyl-dephospho-CoA synthase
i02_0674	CDS	688886	689437	-	citX	apo-citrate lyase phosphoribosyl-dephospho-CoA transferase
i02_0675	CDS	689441	691012	-	citF	citrate lyase alpha chain
i02_0676	CDS	690984	691907	-	citE	citrate lyase beta chain
i02_0677	CDS	692200	693345	-	citC	[citrate [pro-3S]-lyase] ligase
i02_0678	CDS	693578	695296	+	citA	sensor kinase dpiB
i02_0679	CDS	695265	695945	+	dpiA	two-component response regulator DpiA
i02_0680	CDS	695986	697371	-	dcuC	C4-dicarboxylate transporter DcuC
i02_0681	CDS	697958	698518	+	pagP	palmitoyl transferase
i02_0682	CDS	698693	698902	+	cspE	cold shock protein CspE
i02_0683	CDS	698956	699339	-	ccrB	camphor resistance protein CrcB
i02_0684	CDS	699426	700220	+	ybeM	putative amidase
i02_0685	CDS	700349	700552	+	tatE	twin arginine translocase protein E
i02_0686	CDS	700652	701617	-	lipA	lipoyl synthase

Locus_tag	Type	start	End	+/-[a]	Gene[b]	Product
i02_0687	CDS	701849	702190	-	/	hypothetical protein
i02_0688	CDS	702437	703078	-	lipB	lipoyltransferase
i02_0689	CDS	703179	703442	-	ybeD	hypothetical protein
i02_0690	CDS	703552	704763	-	dacA	D-alanyl-D-alanine carboxypeptidase fraction A
i02_0691	CDS	704801	704926	+	/	hypothetical protein
i02_0692	CDS	704903	705991	-	rlpA	rare lipoprotein A
i02_0693	CDS	706002	707114	-	mrdB	cell wall shape-determining protein
i02_0694	CDS	707117	709018	-	mrdA	penicillin-binding protein 2
i02_0695	CDS	709049	709516	-	ybeA	rRNA large subunit methyltransferase
i02_0696	CDS	709520	709837	-	ybeB	hypothetical protein
i02_0697	CDS	709851	710141	+	/	hypothetical protein
i02_0698	CDS	710097	710711	-	phpB	Alpha-ribazole-5'-phosphate phosphatase
i02_0699	CDS	710732	711436	-	nadD	nicotinic acid mononucleotide
i02_0700	CDS	711375	712406	-	holA	DNA polymerase III subunit delta
i02_0701	CDS	712406	712987	-	rlpB	LPS-assembly lipoprotein RlpB
i02_0702	CDS	713002	715725	-	leuS	leucyl-tRNA synthetase
i02_0703	CDS	715819	716301	+	ybeL	hypothetical protein
i02_0704	CDS	716346	717281	-	rihA	ribonucleoside hydrolase 1
i02_0705	CDS	717399	718124	-	gltL	glutamate/aspartate transport ATP-binding component
i02_0706	CDS	718124	718798	-	gltK	glutamate/aspartate transport system permease component
i02_0707	CDS	718798	719538	-	gltJ	glutamate/aspartate transport system permease component
i02_0708	CDS	719708	720685	-	ybeJ	glutamate and aspartate transporter subunit
i02_0709	CDS	720751	720873	+	/	hypothetical protein
i02_0710	CDS	721070	722947	+	/	hypothetical protein
i02_0711	CDS	723025	724563	-	lnt	apolipoprotein N-acyltransferase
i02_0712	CDS	724588	725466	-	ybeX	magnesium and cobalt efflux protein corC
i02_0713	CDS	725556	726023	-	ybeY	putative metalloprotease
i02_0714	CDS	726020	727099	-	ybeZ	PhoH-like protein
i02_0715	CDS	727079	727222	+	/	hypothetical protein
i02_0716	CDS	727213	728637	-	yleA	hypothetical protein
i02_0717	CDS	728783	729958	+	ubiF	2-octaprenyl-3-methyl-6-methoxy-1,4-benzoquinone hydroxylase
i02_0718	CDS	731198	733096	-	asnB	asparagine synthetase B
i02_0719	CDS	733118	733870	-	nagD	UMP phosphatase
i02_0720	CDS	733918	735138	-	nagC	N-acetylglucosamine repressor
i02_0721	CDS	735147	736295	-	nagA	N-acetylglucosamine-6-phosphate deacetylase
i02_0722	CDS	736355	737155	-	nagB	glucosamine-6-phosphate deaminase
i02_0723	CDS	737204	737338	-	/	hypothetical protein
i02_0724	CDS	737488	739434	+	nagE	N-acetyl glucosamine specific PTS system
i02_0725	CDS	739404	739556	-	/	hypothetical protein
i02_0726	CDS	739523	741049	-	/	hypothetical protein
i02_0727	CDS	740817	741275	-	/	hypthetical protein
i02_0728	CDS	741284	742453	-	/	hypthetical protein
i02_0729	CDS	742489	743247	-	/	hypothetical protein
i02_0731	CDS	743307	744194	-	/	putative dihydrodipicolinate synthase
i02_0732	CDS	744198	745355	-	/	putative alcohol dehydrogenase
i02_0733	CDS	745519	746760	+	/	putative inner membrane protein
i02_0734	CDS	746753	747739	+	pdxA	4-hydroxythreonine-4-phosphate dehydrogenase 2
i02_0735	CDS	747741	748502	+	/	putative transcriptional regulator
i02_0736	CDS	748722	750386	+	glnS	glutaminyl-tRNA synthetase
i02_0737	CDS	750828	752234	+	ybfM	hypothetical protein
i02_0738	CDS	752284	752610	+	ybfN	lipoprotein ybfN precursor
i02_0739	CDS	752694	753140	-	fur	ferric uptake regulator
i02_0740	CDS	753429	754076	-	fldA	flavodoxin FldA
i02_0741	CDS	754099	754461	-	ybfE	LexA regulated protein

Locus_tag	Type	start	End	+/-[a]	Gene[b]	Product
i02_0742	CDS	754532	755296	-	ybfF	hypothetical protein
i02_0743	CDS	755481	756026	+	seqA	replication initiation regulator SeqA
i02_0744	CDS	756001	757692	+	pgm	phosphoglucomutase
i02_0745	CDS	757749	759068	-	potE	putrescine transporter
i02_0746	CDS	759065	761272	-	speF	ornithine decarboxylase
i02_0747	CDS	761277	761429	-	/	hypothetical protein
i02_0748	CDS	761656	761790	-	/	hypothetical protein
i02_0749	CDS	761793	761930	+	/	conserved hypothetical protein
i02_0750	CDS	761952	762629	-	kdpE	DNA-binding transcriptional activator KdpE
i02_0751	CDS	762626	765313	-	kdpD	sensor protein KdpD
i02_0752	CDS	765303	765899	-	kdpC	potassium-transporting ATPase subunit C
i02_0753	CDS	765884	767932	-	kdpB	potassium-transporting ATPase subunit B
i02_0754	CDS	767955	769724	-	kdpA	potassium-transporting ATPase subunit A
i02_0755	CDS	769628	769831	-	/	hypothetical protein
i02_0756	CDS	770030	770236	+	ybfA	hypothetical protein
i02_0757	CDS	770337	770846	+	ybgA	hypothetical protein
i02_0758	CDS	770843	772261	+	phrB	deoxyribodipyrimidine photolyase
i02_0759	CDS	772303	773784	-	ybgH	putative transporter YbgH
i02_0760	CDS	774055	774798	+	ybgI	putative hydrolase-oxidase
i02_0761	CDS	774821	775477	+	ybgJ	hypothetical protein
i02_0762	CDS	775471	776403	+	ybgK	hypothetical protein
i02_0763	CDS	776393	777127	+	ybgL	LamB/YcsF family protein
i02_0764	CDS	777163	777954	+	nei	endonuclease VIII
i02_0765	CDS	777951	779042	-	abrB	AbrB protein
i02_0766	CDS	779281	779523	-	/	hypothetical protein
i02_0767	CDS	779671	780954	-	gltA	type II citrate synthase
i02_0768	CDS	780936	781127	+	/	hypothetical protein
i02_0769	CDS	781648	782052	+	sdhC	succinate dehydrogenase cytochrome b556 large subunit
i02_0770	CDS	782046	782393	+	sdhD	succinate dehydrogenase cytochrome b556 small subunit
i02_0771	CDS	782381	784159	+	sdhA	succinate dehydrogenase flavoprotein subunit
i02_0772	CDS	784175	784891	+	sdhB	succinate dehydrogenase iron-sulfur subunit
i02_0773	CDS	785442	788243	+	sucA	2-oxoglutarate dehydrogenase E1 component
i02_0774	CDS	788258	789475	+	sucB	dihydrolipoamide succinyltransferase
i02_0775	CDS	789569	790735	+	sucC	succinyl-CoA synthetase subunit beta
i02_0776	CDS	790735	791604	+	sucD	succinyl-CoA synthetase subunit alpha
i02_0777	CDS	791844	792803	+	/	hypothetical protein
i02_0778	CDS	792832	793185	+	/	hypothetical protein
i02_0779	CDS	793272	793376	-	/	hypothetical protein
i02_0780	CDS	793471	793539	-	/	hypothetical protein
i02_0781	CDS	794286	795857	+	cydA	cytochrome D ubiquinol oxidase subunit I
i02_0782	CDS	795873	797012	+	cydB	cytochrome D ubiquinol oxidase subunit II
i02_0783	CDS	797027	797140	+	/	hypothetical protein
i02_0784	CDS	797140	797433	+	ybgE	hypothetical protein
i02_0785	CDS	797583	797987	+	ybgC	acyl-CoA thioester hydrolase YbgC
i02_0786	CDS	797984	798676	+	tolQ	colicin uptake protein TolQ
i02_0787	CDS	798710	799108	+	tolR	colicin uptake protein TolR
i02_0788	CDS	799173	800393	+	tolA	cell envelope integrity inner membrane protein
i02_0789	CDS	800523	801818	+	tolB	translocation protein TolB
i02_0790	CDS	801829	802374	+	pal	peptidoglycan-associated outer membrane
i02_0791	CDS	802384	803175	+	ybgF	hypothetical protein
i02_0792	CDS	803020	803319	-	/	hypothetical protein
i02_0793	CDS	804321	805364	+	nadA	quinolinate synthetase
i02_0794	CDS	805402	806121	+	pnuC	protein pnuC
i02_0795	CDS	806118	806516	-	/	hypothetical protein
i02_0796	CDS	806620	807000	-	ybgS	hypothetical protein
i02_0797	CDS	807316	808368	+	aroG	phospho-2-dehydro-3-deoxyheptonate aldolase

Locus_tag	Type	start	End	+/-[a]	Gene[b]	Product
i02_0798	CDS	808534	809301	-	gpmA	phosphoglyceromutase
i02_0799	CDS	809489	810529	-	galM	aldose 1-epimerase
i02_0800	CDS	810523	811671	-	galK	galactokinase
i02_0801	CDS	811675	812721	-	galT	galactose-1-phosphate uridylyltransferase
i02_0802	CDS	812731	813789	-	galE	UDP-galactose-4-epimerase
i02_0803	CDS	814009	815481	-	modF	putative molybdenum transport ATP-binding protein
i02_0804	CDS	815549	816337	-	modE	DNA-binding transcriptional regulator ModE
i02_0805	CDS	816460	816615	+	ybhT	hypothetical protein
i02_0806	CDS	816551	816760	+	/	hypothetical protein
i02_0807	CDS	816782	817555	+	modA	molybdate transporter periplasmic protein
i02_0808	CDS	817555	818244	+	modB	molybdate ABC transporter permease protein
i02_0809	CDS	818247	819305	+	modC	molybdate transporter ATP-binding protein
i02_0810	CDS	819306	820220	-	ybhA	phosphotransferase
i02_0811	CDS	820279	821274	+	ybhE	6-phosphogluconolactonase
i02_0812	CDS	821315	822331	-	ybhD	putative transcriptional regulator YbhD
i02_0813	CDS	822452	823504	+	ybhH	hypothetical protein
i02_0814	CDS	823580	825013	+	ybhI	hypothetical protein
i02_0815	CDS	825172	827457	+	ybhJ	hypothetical protein
i02_0816	CDS	827597	828880	-	ybhC	putative pectinesterase
i02_0817	CDS	829032	829508	-	ybhB	putative kinase inhibitor protein
i02_0818	CDS	829617	830747	+	gipA	Peyer's patch-specific virulence factor GipA
i02_0820	CDS	830858	832147	-	bioA	adenosylmethionine-8-amino-7-oxononanoate aminotransferase monomer
i02_0821	CDS	832234	833274	+	bioB	biotin synthase
i02_0822	CDS	833271	834425	+	bioF	8-amino-7-oxononanoate synthase
i02_0823	CDS	834412	835167	+	bioC	biotin biosynthesis protein BioC
i02_0824	CDS	835160	835837	+	bioD	dithiobiotin synthetase
i02_0825	CDS	836004	836141	-	/	hypothetical protein
i02_0826	CDS	836416	838437	+	uvrB	excinuclease ABC subunit B
i02_0827	CDS	838475	839443	-	ybhK	hypothetical protein
i02_0828	CDS	839699	840769	+	moaA	molybdenum cofactor biosynthesis protein A
i02_0829	CDS	840791	841303	+	moaB	molybdenum cofactor biosynthesis protein B
i02_0830	CDS	841045	841536	-	/	hypothetical protein
i02_0831	CDS	841306	841791	+	moaC	molybdenum cofactor biosynthesis protein C
i02_0832	CDS	841784	842029	+	moaD	molybdopterin synthase small subunit
i02_0833	CDS	842031	842483	+	moaE	molybdopterin synthase large subunit
i02_0834	CDS	842539	843324	+	ybhL	hypothetical protein
i02_0835	CDS	843256	843387	-	/	hypothetical protein
i02_0836	CDS	843503	844243	+	ybhM	hypothetical protein
i02_0837	CDS	844279	845235	-	ybhN	hypothetical protein
i02_0838	CDS	845235	846476	-	ybhO	cardiolipin synthase 2
i02_0839	CDS	846473	847234	-	ybhP	hypothetical protein
i02_0840	CDS	847367	847777	+	ybhQ	hypothetical protein
i02_0841	CDS	847739	848845	-	ybhR	hypothetical protein
i02_0842	CDS	848856	849989	-	ybhS	hypothetical protein
i02_0843	CDS	849982	851733	-	ybhF	ABC transporter ATP-binding protein
i02_0844	CDS	851711	852709	-	ybhG	hypothetical protein
i02_0845	CDS	852709	853392	-	ybiH	putative DNA-binding transcriptional regulator
i02_0846	CDS	853609	854970	+	rhlE	ATP-dependent RNA helicase RhlE
i02_0847	CDS	855203	855508	-	/	hypothetical protein
i02_0848	CDS	855507	857657	+	dinG	ATP-dependent DNA helicase DinG
i02_0849	CDS	857685	858647	+	ybiB	glycosyl transferase family protein
i02_0850	CDS	858788	859873	+	ybiC	hypothetical protein
i02_0851	CDS	860101	860361	-	ybiJ	hypothetical protein
i02_0852	CDS	860626	860892	-	/	hypothetical protein
i02_0853	CDS	860966	861679	-	ybiX	putative hydroxylase
i02_0854	CDS	861859	864141	-	ybiL	catecholate siderophore receptor Fiu

Locus_tag	Type	start	End	+/-[a]	Gene[b]	Product
i02_0855	CDS	864406	864813	-	ybiM	hypothetical protein
i02_0856	CDS	864852	865868	+	ybiN	putative SAM-dependent methyltransferase
i02_0857	CDS	865865	868090	-	ybiO	hypothetical protein
i02_0858	CDS	868207	868929	-	glnQ	glutamine ABC transporter ATP-binding protein
i02_0859	CDS	868926	869585	-	glnP	glutamine ABC transporter permease protein
i02_0860	CDS	869724	870470	-	glnH	glutamine ABC transporter periplasmic protein
i02_0861	CDS	870397	870588	-	/	hypothetical protein
i02_0862	CDS	870874	871470	-	dps	DNA starvation/stationary phase protection
i02_0863	CDS	871678	872577	-	ybiF	threonine and homoserine efflux system
i02_0864	CDS	872912	873433	+	ompX	outer membrane protein X
i02_0865	CDS	873483	875066	-	ybiP	hypothetical protein
i02_0866	CDS	875338	875466	-	/	hypothetical protein
i02_0867	CDS	875652	876119	+	/	manganese transport regulator MntR
i02_0868	CDS	876116	877234	+	ybiR	hypothetical protein
i02_0869	CDS	877292	878212	-	ybiS	hypothetical protein
i02_0870	CDS	878431	880023	+	ybiT	ABC transporter ATP-binding protein
i02_0871	CDS	880223	881038	-	ybiV	hypothetical protein
i02_0872	CDS	881184	883616	-	ybiW	putative formate acetyltransferase 3
i02_0873	CDS	883622	884548	-	/	putative pyruvate formate-lyase 3 activating
i02_0874	CDS	884652	885314	+	mipB	fructose-6-phosphate aldolase
i02_0875	CDS	885390	886139	-	moeB	molybdopterin biosynthesis protein MoeB
i02_0876	CDS	886139	887374	-	moeA	molybdopterin biosynthesis protein MoeA
i02_0877	CDS	887578	888543	+	ybiK	L-asparaginase
i02_0878	CDS	888422	890401	+	yliA	glutathione transporter ATP-binding protein
i02_0879	CDS	890154	891959	+	/	putative binding protein yliB precursor
i02_0880	CDS	891977	892897	+	yliC	ABC transporter permease
i02_0881	CDS	892900	893811	+	yliD	ABC transporter permease
i02_0882	CDS	893988	896336	+	yliE	hypothetical protein
i02_0883	CDS	896344	897672	+	yliF	membrane protein yliF
i02_0884	CDS	897719	899044	-	yliG	hypothetical protein
i02_0885	CDS	899257	899640	+	bssR	biofilm formation regulatory protein BssR
i02_0886	CDS	899751	900866	+	yliI	hypothetical protein
i02_0887	CDS	900863	901495	-	yliJ	GST-like protein yliJ
i02_0888	CDS	901715	902938	+	dacC	D-alanyl-D-alanine carboxypeptidase fraction C
i02_0889	CDS	902985	903743	-	deoR	DNA-binding transcriptional repressor DeoR
i02_0890	CDS	903801	904397	-	ybjG	undecaprenyl pyrophosphate phosphatase
i02_0891	CDS	904652	905914	+	cmr	multidrug translocase mdfA
i02_0892	CDS	905955	906239	-	ybjH	hypothetical protein
i02_0893	CDS	906325	907143	-	/	hypothetical protein
i02_0894	CDS	907140	908411	-	ybjJ	hypothetical protein
i02_0895	CDS	908402	908968	+	ybjK	hypothetical protein
i02_0896	CDS	909143	910828	-	ybjL	hypothetical protein
i02_0897	CDS	911098	911475	+	ybjM	hypothetical protein
i02_0898	CDS	911505	911777	-	grxA	glutaredoxin 1
i02_0899	CDS	911922	912209	+	ybjC	hypothetical protein
i02_0900	CDS	912193	912915	+	mdaA	nitroreductase A
i02_0901	CDS	912976	913878	+	rimK	ribosomal protein S6 modification protein
i02_0902	CDS	913918	914442	+	ybjN	hypothetical protein
i02_0903	CDS	914647	915903	+	potF	putrescine transporter subunit
i02_0904	CDS	915917	917131	+	potG	putrescine transporter ATP-binding subunit
i02_0905	CDS	917141	918085	+	potH	putrescine transporter subunit
i02_0906	pseudo	918040	918927	+	potI	putrescine transporter subunit
i02_0907	CDS	918987	919475	+	ybjO	hypothetical protein
i02_0908	CDS	919516	920643	+	rumB	23S rRNA methyluridine methyltransferase
i02_0909	CDS	920672	921454	-	artJ	arginine-binding periplasmic protein 2
i02_0910	CDS	921629	922297	-	artM	arginine transporter permease subunit ArtM
i02_0911	CDS	922297	923013	-	artQ	arginine transporter permease subunit ArtQ
i02_0912	CDS	923020	923751	-	artI	arginine-binding periplasmic protein 1

Locus_tag	Type	start	End	+/-[a]	Gene[b]	Product
i02_0913	CDS	923769	924503	-	artP	arginine transporter ATP-binding subunit
i02_0914	CDS	924715	925254	-	ybjP	putative lipoprotein
i02_0915	CDS	925356	925679	+	ybjQ	hypothetical protein
i02_0916	CDS	925676	926506	+	ybjR	N-acetylmuramoyl-L-alanine amidase ybjR
i02_0917	CDS	926503	927579	-	/	hypothetical protein
i02_0918	CDS	927615	929120	-	ybjT	hypothetical protein
i02_0919	CDS	929056	930057	-	ybjU	L-threonine aldolase
i02_0920	CDS	930094	931812	-	poxB	pyruvate dehydrogenase
i02_0921	CDS	931945	932919	-	/	HCP oxidoreductase, NADH-dependent
i02_0922	CDS	932925	934583	-	ybjW	hydroxylamine reductase
i02_0923	CDS	934721	935665	-	ybjE	hypothetical protein
i02_0924	CDS	935598	935732	+	/	hypothetical protein
i02_0925	CDS	936114	936815	-	aqpZ	aquaporin Z
i02_0926	CDS	937022	937177	-	/	hypothetical protein
i02_0927	CDS	937235	938893	+	ybjD	hypothetical protein
i02_0928	CDS	938890	939882	-	ybjX	hypothetical protein
i02_0929	CDS	939951	940085	+	/	hypothetical protein
i02_0930	CDS	939997	941112	+	macA	macrolide transporter subunit MacA
i02_0931	CDS	941109	943055	+	/	hypothetical protein
i02_0932	CDS	943128	943382	-	cspD	stationary phase/starvation inducible regulatory
i02_0933	CDS	943675	943995	+	clpS	ATP-dependent Clp protease adaptor protein ClpS
i02_0934	CDS	944020	946302	+	clpA	ATP-dependent Clp protease ATP-binding subunit
i02_0935	CDS	946987	947205	-	infA	translation initiation factor IF-1
i02_0936	CDS	947490	948194	-	aat	leucyl/phenylalanyl-tRNA--protein transferase
i02_0937	CDS	948236	949957	-	cydC	cysteine/glutathione ABC transporter
i02_0938	CDS	949958	951724	-	cydD	cysteine/glutathione ABC transporter
i02_0939	CDS	951847	952932	-	trxB	thioredoxin reductase
i02_0940	CDS	953302	953850	+	lrp	leucine-responsive transcriptional regulator
i02_0941	CDS	953985	958028	+	ftsK	DNA translocase FtsK
i02_0942	CDS	958184	958798	+	lolA	outer-membrane lipoprotein carrier protein
i02_0943	CDS	958809	960152	+	ycaJ	recombination factor protein RarA
i02_0944	CDS	960243	961535	+	serS	seryl-tRNA synthetase
i02_0945	CDS	961774	964218	+	dmsA	anaerobic dimethyl sulfoxide reductase chain A
i02_0946	CDS	964229	964846	+	dmsB	anaerobic dimethyl sulfoxide reductase chain B
i02_0947	CDS	964788	965711	+	dmsC	anaerobic dimethyl sulfoxide reductase chain C
i02_0948	CDS	965747	966472	-	ycaC	hypothetical protein
i02_0949	CDS	966528	966656	-	/	hypothetical protein
i02_0950	CDS	966687	967835	+	ycaD	putative MFS family transporter protein
i02_0951	CDS	967932	968672	-	pflA	pyruvate formate lyase-activating enzyme 1
i02_0952	CDS	968864	971146	-	pflB	PflB
i02_0954	CDS	971201	972397	-	focA_2	formate transporter
i02_0955	CDS	972464	974257	-	ycaO	hypothetical protein
i02_0956	CDS	974354	975046	+	ycaP	hypothetical protein
i02_0957	CDS	975245	976333	+	serC	phosphoserine aminotransferase
i02_0958	CDS	976404	977687	+	aroA	3-phosphoshikimate 1-carboxyvinyltransferase
i02_0959	CDS	977848	978621	+	ycaL	putative metalloprotease ycaL
i02_0960	CDS	978794	979477	+	cmk	cytidylate kinase
i02_0961	CDS	979588	981261	+	rpsA	30S ribosomal protein S1
i02_0962	CDS	981421	981705	+	ihfB	integration host factor subunit beta
i02_0963	CDS	981911	984175	+	ycaI	hypothetical protein
i02_0964	CDS	984212	985960	+	msbA	lipid transporter ATP-binding/permease protein
i02_0965	CDS	985957	986943	+	lpxK	tetraacyldisaccharide 4'-kinase
i02_0966	CDS	986980	988212	+	ycaQ	hypothetical protein
i02_0967	CDS	988264	988446	+	ycaR	hypothetical protein
i02_0968	CDS	988443	989189	+	kdsB	3-deoxy-manno-octulosonate cytidylyltransferase
i02_0969	CDS	989343	990236	+	ycbJ	hypothetical protein
i02_0970	CDS	990213	990992	-	ycbC	hypothetical protein
i02_0971	CDS	990895	991059	-	/	hypothetical protein

Locus_tag	Type	start	End	+/-[a]	Gene[b]	Product
i02_0972	CDS	991116	991913	+	smtA	putative metallothionein SmtA
i02_0973	CDS	991910	993232	+	mukF	condesin subunit F
i02_0974	CDS	993186	993917	+	mukE	condesin subunit E
i02_0975	CDS	993917	998377	+	mukB	cell division protein MukB
i02_0976	CDS	998638	1000485	+	ycbB	hypothetical protein
i02_0977	CDS	1000657	1001214	+	ycbK	hypothetical protein
i02_0978	CDS	1001241	1001888	+	ycbL	hypothetical protein
i02_0979	CDS	1001938	1003128	-	aspC	aromatic amino acid aminotransferase
i02_0980	CDS	1003313	1004401	-	ompF	outer membrane protein F
i02_0981	CDS	1005004	1006404	-	asnC	asparaginyl-tRNA synthetase
i02_0982	CDS	1006573	1007823	-	pncB	nicotinate phosphoribosyltransferase
i02_0983	CDS	1008041	1010653	+	pepN	aminopeptidase N
i02_0984	CDS	1010848	1011615	-	ssuB	aliphatic sulfonates transport ATP-binding component
i02_0985	CDS	1011612	1012406	-	ssuC	alkanesulfonate transporter permease subunit
i02_0986	CDS	1012415	1013560	-	ycbN	alkanesulfonate monooxygenase
i02_0987	CDS	1013557	1014558	-	ycbO	alkanesulfonate transporter substrate-binding component
i02_0988	CDS	1014509	1015084	-	ycbP	NAD(P)H-dependent FMN reductase
i02_0989	CDS	1015294	1016331	+	pyrD	dihydroorotate dehydrogenase 2
i02_0990	CDS	1016469	1017047	+	ycbW	hypothetical protein
i02_0991	CDS	1017044	1018153	-	ycbX	hypothetical protein
i02_0992	CDS	1018251	1020359	+	rlmL	23S rRNA m(2)G2445 methyltransferase
i02_0993	CDS	1020371	1022278	+	uup	ABC transporter ATPase component
i02_0994	CDS	1022408	1023661	+	pqiA	paraquat-inducible protein A
i02_0995	CDS	1023606	1025306	+	pqiB	paraquat-inducible protein B
i02_0996	CDS	1025303	1025866	+	ymbA	hypothetical protein
i02_0997	CDS	1026031	1026162	-	/	hypothetical protein
i02_0998	CDS	1026358	1027074	-	fabA	3-hydroxydecanoyl-(acyl carrier protein)
i02_0999	CDS	1026945	1028705	-	/	putative protease La-like protein
i02_1000	CDS	1028891	1029343	+	ycbG	hypothetical protein
i02_1001	CDS	1029421	1030560	-	ompA	outer membrane protein A
i02_1002	CDS	1030627	1030845	+	/	hypothetical protein
i02_1003	CDS	1030829	1031344	-	sulA	SOS cell division inhibitor
i02_1004	CDS	1031557	1032186	+	yccR	hypothetical protein
i02_1005	CDS	1032149	1034311	-	yccS	hypothetical protein
i02_1006	CDS	1034321	1034767	-	yccF	hypothetical protein
i02_1007	CDS	1034797	1036944	+	helD	DNA helicase IV
i02_1008	CDS	1036976	1037443	-	mgsA	methylglyoxal synthase
i02_1009	CDS	1037530	1038192	-	yccT	hypothetical protein
i02_1010	CDS	1038284	1038778	+	/	hypothetical protein
i02_1011	CDS	1038823	1039191	-	yccV	hypothetical protein
i02_1012	CDS	1039198	1040388	-	yccW	hypothetical protein
i02_1013	CDS	1040270	1040761	+	/	putative acylphosphatase
i02_1014	CDS	1040758	1041087	-	yccK	sulfur transfer protein TusE
i02_1015	CDS	1041179	1041838	-	yccA	hypothetical protein
i02_1016	CDS	1042400	1042540	-	/	hypothetical protein
i02_1017	CDS	1042532	1043677	+	hyaA	hydrogenase-1 small chain precursor
i02_1018	CDS	1043674	1045467	+	hyaB	hydrogenase 1 large subunit
i02_1019	CDS	1045486	1046193	+	hyaC	hydrogenase 1 b-type cytochrome subunit
i02_1020	CDS	1046190	1046777	+	hyaD	hydrogenase 1 maturation protease
i02_1021	CDS	1046774	1047172	+	hyaE	hydrogenase-1 operon protein HyaE
i02_1022	CDS	1047109	1048026	+	hyaF	hydrogenase-1 operon protein HyaF
i02_1023	CDS	1048154	1049704	+	appC	cytochrome bd-II oxidase subunit I
i02_1024	CDS	1049716	1050852	+	appB	cytochrome bd-II oxidase subunit II
i02_1025	CDS	1051007	1052347	+	appA	phosphoanhydride phosphorylase
i02_1026	CDS	1052335	1052547	-	cspH	cold shock-like protein cspH
i02_1027	CDS	1052833	1053045	+	cspG	cold shock protein CspG

Locus_tag	Type	start	End	+/-[a]	Gene[b]	Product
i02_1028	CDS	1053186	1053449	+	sfa	cold shock gene
i02_1029	CDS	1053439	1053612	+	/	GnsB protein
i02_1030	CDS	1053661	1054734	-	yccM	putative electron transport protein yccM
i02_1031	CDS	1054817	1057549	-	torS	hybrid sensory histidine kinase TorS
i02_1032	CDS	1057632	1058660	+	torT	TMAO reductase system periplasmic protein TorT
i02_1033	CDS	1058633	1059325	-	torR	DNA-binding transcriptional regulator TorR
i02_1034	CDS	1059407	1060627	+	torC	cytochrome c-type protein torC
i02_1035	CDS	1060627	1063173	+	torA	trimethylamine-N-oxide reductase 1 precursor
i02_1036	CDS	1063137	1063769	+	torD	chaperone protein TorD
i02_1037	CDS	1064125	1064430	-	yccD	chaperone-modulator protein CbpM
i02_1038	CDS	1064430	1065350	-	cbpA	curved DNA-binding protein CbpA
i02_1039	CDS	1065885	1067126	+	agp	glucose-1-phosphatase/inositol phosphatase
i02_1040	CDS	1067164	1067391	-	yccJ	hypothetical protein
i02_1041	CDS	1067412	1068008	-	wrbA	TrpR binding protein WrbA
i02_1042	CDS	1068412	1068570	+	/	hypothetical protein
i02_1043	CDS	1068637	1070031	-	ycdG	putative purine permease ycdG
i02_1044	CDS	1069986	1070561	-	/	putative flavin:NADH reductase ycdH
i02_1045	CDS	1070491	1071081	-	/	hypothetical protein
i02_1046	CDS	1071091	1071891	-	ycdJ	hypothetical protein
i02_1047	CDS	1071899	1072285	-	ycdK	hypothetical protein
i02_1048	CDS	1072297	1073031	-	ycdL	isochorismatase family protein ycdL
i02_1049	CDS	1072989	1074170	-	/	putative monooxygenase ycdM
i02_1050	CDS	1074341	1075006	+	ycdC	putative transcriptional regulator YcdC
i02_1051	CDS	1075046	1079008	-	putA	trifunctional transcriptional regulator/proline
i02_1052	CDS	1079063	1079272	+	/	hypothetical protein
i02_1053	CDS	1079160	1079351	-	/	hypothetical protein
i02_1054	CDS	1079431	1080939	+	putP	Sodium/proline symporter
i02_1055	CDS	1081150	1081989	+	/	putative cytochrome
i02_1056	CDS	1082047	1083174	+	ycdO	hypothetical protein
i02_1057	CDS	1083180	1084451	+	ycdB	hypothetical protein
i02_1058	CDS	1084588	1084722	-	/	hypothetical protein
i02_1059	CDS	1084942	1085865	+	phoH	hypothetical protein
i02_1060	CDS	1085915	1086328	-	ycdP	hypothetical protein
i02_1061	CDS	1086330	1087655	-	ycdQ	N-glycosyltransferase PgaC
i02_1062	CDS	1087648	1089666	-	ycdR	lipoprotein ycdR precursor
i02_1063	CDS	1089675	1092098	-	pgaA	outer membrane protein PgaA
i02_1064	CDS	1092563	1094053	+	ycdT	hypothetical protein
i02_1065	CDS	1094332	1095534	+	/	P4 family integrase
i02_1066	CDS	1095722	1097539	-	/	hypothetical protein
i02_1067	CDS	1097825	1097989	+	/	hypothetical protein
i02_1068	CDS	1098037	1098429	+	/	hypothetical protein
i02_1069	CDS	1098701	1098949	+	alpA	prophage CP4-57 regulatory protein alpA
i02_1070	CDS	1099574	1101034	+	/	hypothetical protein
i02_1071	CDS	1101998	1102591	+	/	hypothetical protein
i02_1072	CDS	1102693	1102899	+	/	hypothetical protein
i02_1073	CDS	1103068	1105068	-	/	hypothetical protein
i02_1074	CDS	1104983	1106149	-	/	putative aminotransferase
i02_1075	CDS	1106571	1107194	-	/	hypothetical protein
i02_1076	CDS	1109059	1109583	+	/	hypothetical protein
i02_1077	CDS	1109622	1110047	+	/	hypothetical protein
i02_1079	CDS	1110357	1111586	-	/	3-oxoacyl-(acyl carrier protein) synthase II
i02_1080	CDS	1111583	1112314	-	fabG	3-ketoacyl-(acyl-carrier-protein) reductase
i02_1081	CDS	1112314	1112778	-	/	hypothetical protein
i02_1082	CDS	1112775	1113953	-	/	3-oxoacyl-(acyl carrier protein) synthase I
i02_1083	CDS	1113946	1114530	-	/	hypothetical protein
i02_1084	CDS	1114527	1116845	-	/	hypothetical protein
i02_1085	CDS	1116814	1117419	-	/	hypothetical protein
i02_1086	CDS	1117416	1117838	-	/	hypothetical protein

Locus_tag	Type	start	End	+/-[a]	Gene[b]	Product
i02_1087	CDS	1117842	1119464	-	/	hypothetical protein
i02_1088	CDS	1119436	1119558	-	/	hypothetical protein
i02_1089	CDS	1119509	1119886	-	/	hypothetical protein
i02_1090	CDS	1119849	1121207	-	/	hypothetical protein
i02_1091	CDS	1121204	1121788	-	/	hypothetical protein
i02_1092	CDS	1121790	1122041	-	/	acyl carrier protein
i02_1093	CDS	1122053	1122310	-	/	putative acyl carrier protein
i02_1094	CDS	1122285	1123124	-	/	putative phospholipid biosynthesis
i02_1095	CDS	1123103	1123849	-	/	hypothetical protein
i02_1096	CDS	1123866	1124924	-	/	putative O-methyltransferase
i02_1097	CDS	1124989	1125378	-	/	hypothetical protein
i02_1098	CDS	1125943	1128669	+	/	hypothetical protein
i02_1099	CDS	1129856	1131433	+	/	hypothetical protein
i02_1100	CDS	1131954	1132331	+	/	hypothetical protein
i02_1101	CDS	1132830	1133225	+	/	hypothetical protein
i02_1103	CDS	1133196	1133720	-	/	hypothetical protein
i02_1104	CDS	1133740	1134255	-	/	conserved hypothetical protein
i02_1105	CDS	1134347	1134547	-	/	hypothetical protein
i02_1106	CDS	1134451	1135014	+	/	hypothetical protein
i02_1107	CDS	1135091	1135330	-	/	Cea protein
i02_1108	CDS	1136088	1136297	+	/	entry exclusion protein 2
i02_1109	CDS	1136420	1136575	-	/	hypothetical protein
i02_1110	CDS	1136596	1136904	-	/	hypothetical protein
i02_1111	CDS	1136986	1137489	-	/	hypothetical protein
i02_1112	CDS	1137461	1138210	-	/	putative transposase
i02_1113	CDS	1138632	1138745	-	/	conserved hypothetical protein
i02_1114	CDS	1139113	1140189	+	/	phospho-2-dehydro-3-deoxyheptonate aldolase
i02_1115	CDS	1140077	1140310	+	/	hypothetical protein
i02_1116	CDS	1140660	1140791	+	/	hypothetical protein
i02_1117	CDS	1141066	1142007	-	/	transposase insF
i02_1118	CDS	1141991	1142215	-	/	transposase insE
i02_1119	CDS	1143383	1143676	+	mchB	MchB protein
i02_1120	CDS	1143948	1145498	+	mchC	MchC protein
i02_1121	CDS	1145524	1145976	+	mchD	MchD protein
i02_1122	CDS	1146129	1147403	+	mchE	microcin H47 secretion protein
i02_1123	CDS	1147378	1149492	+	mchF	microcin H47 secretion ATP-binding protein
i02_1124	CDS	1149528	1149749	+	/	conserved domain protein
i02_1125	CDS	1150199	1150885	-	/	hypothetical protein
i02_1126	CDS	1150985	1151455	-	/	hypothetical protein
i02_1127	CDS	1151901	1152413	+	/	hypothetical protein
i02_1128	CDS	1152646	1153224	+	/	hypothetical protein
i02_1129	CDS	1153247	1153393	+	/	hypothetical protein
i02_1130	CDS	1153499	1153783	-	sfaC	putative F1C and S fimbrial switch regulatory protein
i02_1131	CDS	1154139	1154468	+	sfaB	putative F1C and S fimbrial switch regulatory protein
i02_1132	CDS	1154840	1155382	+	focA	F1C major fimbrial subunit precursor
i02_1133	CDS	1155468	1155992	+	sfaD	putative minor F1C fimbrial subunit precursor
i02_1134	CDS	1156033	1156728	+	focC	F1C periplasmic chaperone
i02_1135	CDS	1156750	1159428	+	focD	F1C fimbrial usher
i02_1136	CDS	1159441	1159968	+	focF	F1C minor fimbrial subunit F precursor
i02_1137	CDS	1159990	1160493	+	focG	F1C minor fimbrial subunit protein G presursor
i02_1138	CDS	1160444	1161454	+	focH	F1C putative fimbrial adhesin precursor
i02_1139	CDS	1161510	1161638	+	/	conserved hypothetical protein
i02_1140	CDS	1161758	1162498	+	/	hypothetical protein
i02_1141	CDS	1162645	1163196	+	focX	putative regulatory protein
i02_1142	CDS	1163522	1164559	-	/	hypothetical protein
i02_1143	CDS	1164569	1164763	+	/	hypothetical protein

Locus_tag	Type	start	End	+/-[a]	Gene[b]	Product
i02_1144	CDS	1164789	1166966	+	iroN	outer membrane receptor FepA
i02_1145	CDS	1167011	1167967	-	iroE	IroE protein
i02_1146	CDS	1168052	1169281	-	iroD	ferric enterochelin esterase
i02_1147	CDS	1169385	1173122	-	iroC	ABC transporter ATP-binding protein
i02_1148	CDS	1173184	1174347	-	iroB	putative glucosyltransferase
i02_1149	CDS	1174837	1175136	+	/	hypothetical protein
i02_1150	CDS	1175263	1175772	+	/	transposase
i02_1151	CDS	1175900	1176262	-	/	transposase insG
i02_1152	CDS	1176278	1176433	-	/	conserved hypothetical protein
i02_1153	CDS	1176953	1177315	+	/	hypothetical protein
i02_1154	CDS	1177137	1177571	+	/	hypothetical protein
i02_1155	CDS	1178134	1178592	-	/	putative transposase
i02_1157	CDS	1178604	1179497	-	/	hypothetical protein
i02_1158	CDS	1179843	1181813	+	/	Outer membrane heme/hemoglobin receptor
i02_1159	CDS	1181832	1182623	+	/	hypothetical protein
i02_1160	CDS	1182824	1184086	+	/	hypothetical protein
i02_1161	CDS	1184193	1184375	-	/	hypothetical protein
i02_1162	CDS	1185037	1186173	+	/	hypothetical protein
i02_1163	CDS	1186209	1186751	+	/	hypothetical protein
i02_1164	CDS	1189125	1189421	-	/	hypothetical protein
i02_1165	CDS	1189721	1190593	+	/	hypothetical protein
i02_1166	CDS	1190768	1194043	+	/	antigen 43 precursor
i02_1167	CDS	1194158	1196680	+	/	hypothetical protein
i02_1168	CDS	1196756	1197211	+	/	hypothetical protein
i02_1169	CDS	1197229	1197357	+	/	hypothetical protein
i02_1170	CDS	1197330	1197518	-	/	hypothetical protein
i02_1171	CDS	1197622	1198443	+	/	hypothetical protein
i02_1172	CDS	1197628	1198536	-	/	hypothetical protein
i02_1173	CDS	1198634	1199179	+	/	hypothetical protein
i02_1174	CDS	1199195	1199671	+	/	putative radC-like protein yeeS
i02_1175	CDS	1199974	1200618	+	/	hypothetical protein
i02_1176	CDS	1201089	1201469	+	/	hypothetical protein
i02_1177	CDS	1201466	1201954	+	/	hypothetical protein
i02_1178	CDS	1201974	1202171	+	/	hypothetical protein
i02_1179	CDS	1202256	1203083	+	/	hypothetical protein
i02_1181	CDS	1203531	1204508	+	ycdW	putative 2-hydroxyacid dehydrogenase ycdW
i02_1182	CDS	1204563	1205300	+	ycdX	putative hydrolase
i02_1183	CDS	1205324	1205878	+	ycdY	hypothetical protein
i02_1184	CDS	1205932	1206471	+	ycdZ	hypothetical protein
i02_1185	CDS	1206536	1207369	-	csgG	curli production assembly/transport component
i02_1186	CDS	1207396	1207812	-	csgF	curli assembly protein CsgF
i02_1187	CDS	1207837	1208226	-	csgE	curli assembly protein CsgE
i02_1188	CDS	1208231	1208881	-	csgD	DNA-binding transcriptional regulator CsgD
i02_1189	CDS	1208855	1208995	+	/	hypothetical protein
i02_1190	CDS	1209406	1209540	-	/	hypothetical protein
i02_1191	CDS	1209607	1210089	+	csgB	curlin minor subunit
i02_1192	CDS	1210130	1210588	+	csgA	cryptic curlin major subunit
i02_1193	CDS	1210647	1210979	+	csgC	putative autoagglutination protein
i02_1194	CDS	1211100	1211411	+	ymdA	hypothetical protein
i02_1195	CDS	1211506	1212039	+	ymdB	hypothetical protein
i02_1196	CDS	1211981	1213462	+	ymdC	hypothetical protein
i02_1197	CDS	1213470	1214627	-	mdoC	glucans biosynthesis protein
i02_1198	CDS	1214672	1214815	-	/	hypothetical protein
i02_1199	CDS	1215003	1216556	+	mdoG	glucan biosynthesis protein G
i02_1200	CDS	1216519	1219092	+	mdoH	glucosyltransferase MdoH
i02_1201	CDS	1219101	1219325	+	/	hypothetical protein
i02_1202	CDS	1219264	1219491	+	yceK	hypothetical protein
i02_1203	CDS	1219492	1219869	-	msyB	hypothetical protein

Locus_tag	Type	start	End	+/-[a]	Gene[b]	Product
i02_1204	CDS	1219949	1221175	-	yceE	drug efflux system protein MdtG
i02_1205	CDS	1221347	1222267	-	htrB	lipid A biosynthesis lauroyl acyltransferase
i02_1206	CDS	1222438	1223544	+	yceA	hypothetical protein
i02_1207	CDS	1223586	1224161	-	yceI	hypothetical protein
i02_1208	CDS	1224165	1224743	-	/	cytochrome b561 2
i02_1209	CDS	1224721	1224867	+	/	hypothetical protein
i02_1210	CDS	1224993	1225130	-	/	hypothetical protein
i02_1211	CDS	1225151	1226269	-	solA	N-methyltryptophan oxidase
i02_1212	CDS	1226384	1226641	-	bssS	biofilm formation regulatory protein BssS
i02_1213	CDS	1226928	1227173	-	dinI	DNA damage-inducible protein I
i02_1214	CDS	1227247	1228293	-	pyrC	dihydroorotase
i02_1215	CDS	1228399	1229016	-	yceB	hypothetical protein
i02_1216	CDS	1229093	1229740	-	grxB	glutaredoxin 2
i02_1217	CDS	1229804	1231042	-	yceL	multidrug resistance protein MdtH
i02_1218	CDS	1231248	1231832	+	rimJ	ribosomal-protein-S5-alanine
i02_1219	CDS	1231843	1232490	+	yceH	hypothetical protein
i02_1220	CDS	1232492	1233415	+	/	hypothetical protein
i02_1221	CDS	1233050	1233448	-	/	hypothetical protein
i02_1222	CDS	1233525	1235060	+	mviN	virulence factor mviN-like protein
i02_1223	CDS	1235100	1235516	-	flgN	flagella synthesis protein FlgN
i02_1224	CDS	1235521	1235814	-	flgM	anti-sigma28 factor FlgM
i02_1225	CDS	1235890	1236549	-	flgA	flagellar basal body P-ring biosynthesis protein
i02_1226	CDS	1236705	1237121	+	flgB	flagellar basal body rod protein FlgB
i02_1227	CDS	1237125	1237529	+	flgC	flagellar basal body rod protein FlgC
i02_1228	CDS	1237541	1238236	+	flgD	flagellar basal body rod modification protein
i02_1229	CDS	1238261	1239466	+	flgE	flagellar hook protein FlgE
i02_1230	CDS	1239486	1240241	+	flgF	flagellar basal body rod protein FlgF
i02_1231	CDS	1240413	1241195	+	flgG	flagellar basal body rod protein FlgG
i02_1232	CDS	1241233	1241946	+	flgH	flagellar basal body L-ring protein
i02_1233	CDS	1241955	1243055	+	flgI	flagellar basal body P-ring protein
i02_1234	CDS	1243055	1243996	+	flgJ	flagellar rod assembly protein/muramidase FlgJ
i02_1235	CDS	1244062	1245705	+	flgK	flagellar hook-associated protein FlgK
i02_1236	CDS	1245717	1246670	+	flgL	flagellar hook-associated protein FlgL
i02_1237	CDS	1246865	1250050	-	rne	ribonuclease E
i02_1238	CDS	1250131	1250505	+	/	hypothetical protein
i02_1239	CDS	1250623	1251582	+	rluC	23S rRNA pseudouridylate synthase C
i02_1240	CDS	1251694	1252317	-	maf	Maf-like protein
i02_1241	CDS	1252290	1252418	-	/	hypothetical protein
i02_1242	CDS	1252476	1252997	+	yceD	hypothetical protein
i02_1243	CDS	1253049	1253222	+	/	50S ribosomal protein L32
i02_1244	CDS	1253303	1254373	+	plsX	putative glycerol-3-phosphate acyltransferase
i02_1245	CDS	1254441	1255394	+	fabH	3-oxoacyl-(acyl carrier protein) synthase III
i02_1246	CDS	1255410	1256339	+	fabD	acyl carrier protein S-malonyltransferase
i02_1247	CDS	1256352	1257086	+	/	gluconate 5-dehydrogenase
i02_1248	CDS	1257297	1257533	+	acpP	acyl carrier protein
i02_1249	CDS	1257620	1258861	+	fabF	3-oxoacyl-(acyl carrier protein) synthase II
i02_1250	CDS	1258981	1259790	+	pabC	4-amino-4-deoxychorismate lyase
i02_1251	CDS	1259793	1260815	+	yceG	hypothetical protein
i02_1252	CDS	1260805	1261446	+	tmk	thymidylate kinase
i02_1253	CDS	1261443	1262447	+	holB	DNA polymerase III subunit delta'
i02_1254	CDS	1262458	1263255	+	ycfH	putative metallodependent hydrolase
i02_1255	CDS	1263550	1264983	+	ptsG	glucose-specific PTS system IIBC components
i02_1256	CDS	1265043	1267232	-	fhuE	ferric-rhodotorulic acid outer membrane
i02_1257	CDS	1267265	1267435	-	/	hypothetical protein
i02_1258	CDS	1267548	1267925	+	ycfF	purine nucleoside phosphoramidase
i02_1259	CDS	1267928	1268305	+	ycfL	hypothetical protein
i02_1260	CDS	1268316	1268960	+	ycfM	hypothetical protein
i02_1261	CDS	1268941	1269765	+	thiK	thiamine kinase

Locus_tag	Type	start	End	+/-[a]	Gene[b]	Product
i02_1262	CDS	1269776	1270801	+	nagZ	beta-hexosaminidase
i02_1263	CDS	1270767	1271366	+	ycfP	hypothetical protein
i02_1264	CDS	1271738	1273069	+	ndh	NADH dehydrogenase
i02_1265	CDS	1273257	1273835	+	ycfJ	hypothetical protein
i02_1266	CDS	1273897	1274607	-	ycfQ	putative transcriptional regulator YcfQ
i02_1267	CDS	1274770	1275027	+	ycfR	hypothetical protein
i02_1268	CDS	1275109	1276242	-	ycfS	hypothetical protein
i02_1269	CDS	1276214	1279723	-	mfd	transcription-repair coupling factor
i02_1270	CDS	1279788	1280861	-	ycfT	hypothetical protein
i02_1271	CDS	1281018	1281236	+	ycfU	outer membrane-specific lipoprotein transporter subunit LolC
i02_1272	CDS	1281122	1282321	+	lolC	outer membrane-specific lipoprotein transporter
i02_1273	CDS	1282176	1283015	+	lolD	lipoprotein transporter ATP-binding subunit
i02_1274	CDS	1283015	1284259	+	lolE	outer membrane-specific lipoprotein transporter
i02_1275	CDS	1284288	1285199	+	ycfX	N-acetyl-D-glucosamine kinase
i02_1276	CDS	1285215	1286036	+	cobB	NAD-dependent deacetylase
i02_1277	CDS	1286174	1286962	-	ycfZ	hypothetical protein
i02_1278	CDS	1286959	1287462	-	ymfA	hypothetical protein
i02_1279	CDS	1287478	1288524	-	potD	spermidine/putrescine ABC transporter
i02_1280	CDS	1288521	1289315	-	potC	spermidine/putrescine ABC transporter membrane
i02_1281	CDS	1289482	1290600	-	/	prophage lambda integrase
i02_1282	CDS	1290569	1290847	-	xisN	putative excisionase for prophage
i02_1283	CDS	1290900	1293341	-	recE	exodeoxyribonuclease VIII
i02_1284	CDS	1293435	1293635	-	ydfD	hypothetical protein
i02_1285	CDS	1293623	1293916	-	dicB	division inhibition protein dicB
i02_1286	CDS	1293922	1294077	+	/	hypothetical protein
i02_1287	CDS	1294116	1294415	+	/	hypothetical protein
i02_1288	CDS	1294380	1294598	-	ydfC	hypothetical protein
i02_1289	CDS	1294628	1294798	-	ydfB	hypothetical protein
i02_1290	CDS	1294758	1294994	-	ydfA	hypothetical protein
i02_1291	CDS	1295179	1295598	-	/	hypothetical protein
i02_1292	CDS	1295961	1296389	+	/	hypothetical protein
i02_1293	CDS	1296455	1297525	+	/	hypothetical protein
i02_1294	CDS	1297566	1297988	+	/	hypothetical protein
i02_1295	CDS	1297989	1298402	+	/	hypothetical protein
i02_1296	CDS	1298496	1298678	+	/	hypothetical protein
i02_1297	CDS	1298671	1298847	+	/	hypothetical protein
i02_1298	CDS	1299292	1299510	+	/	hypothetical protein
i02_1299	CDS	1299963	1300094	+	/	hypothetical protein
i02_1300	CDS	1300383	1300661	+	/	hypothetical protein
i02_1302	CDS	1300663	1301712	+	/	hypothetical protein
i02_1303	CDS	1301725	1302081	+	/	hypothetical protein
i02_1304	CDS	1302096	1302917	+	/	cryptic prophage antitermination protein Q
i02_1305	CDS	1303810	1303941	+	/	hypothetical protein
i02_1306	CDS	1304222	1304557	-	ycgW	hypothetical protein
i02_1307	CDS	1304683	1304832	-	/	hypothetical protein
i02_1308	CDS	1304806	1305006	+	/	hypothetical protein
i02_1309	CDS	1305003	1305164	+	/	hypothetical protein
i02_1310	CDS	1305209	1305529	+	/	lambdoid prophage DLP12 lysis protein S
i02_1311	CDS	1305534	1305878	+	ydfR	hypothetical protein
i02_1312	CDS	1305844	1306122	-	/	hypothetical protein
i02_1313	CDS	1306222	1306755	+	/	lysozyme from lambdoid prophage Qin
i02_1314	CDS	1306737	1307219	+	/	putative Rz endopeptidase from lambdoid prophage
i02_1315	CDS	1307207	1307359	+	/	hypothetical protein
i02_1316	CDS	1307526	1308086	+	/	hypothetical protein
i02_1317	CDS	1308389	1308814	-	ydfO	hypothetical protein
i02_1318	CDS	1308857	1309090	-	/	hypothetical protein

Locus_tag	Type	start	End	+/-[a]	Gene[b]	Product
i02_1319	CDS	1309256	1309363	+	/	hypothetical protein
i02_1320	CDS	1309485	1309994	+	/	prophage Qin DNA packaging protein NU1-like
i02_1321	CDS	1309966	1311894	+	/	putative DNA packaging protein of prophage;
i02_1322	CDS	1311878	1312084	+	/	putative DNA packaging protein of prophage
i02_1323	CDS	1312081	1313673	+	/	putative capsid protein of prophage
i02_1324	CDS	1313663	1315168	+	/	putative capsid assembly protein of prophage
i02_1325	CDS	1315205	1315552	+	/	putative capsid protein of prophage
i02_1326	CDS	1315610	1316638	+	/	putative capsid protein of prophage
i02_1327	CDS	1316648	1317073	+	/	hypothetical protein
i02_1328	CDS	1317066	1317419	+	/	putative head-tail joining protein of prophage
i02_1329	CDS	1317435	1317968	+	/	putative tail component of prophage
i02_1330	CDS	1317965	1318360	+	/	putative tail component of prophage
i02_1331	CDS	1318368	1319114	+	/	putative tail fiber component V of prophage
i02_1332	CDS	1319133	1319564	+	/	putative tail component of prophage
i02_1333	CDS	1319591	1320004	+	/	putative tail component of prophage
i02_1334	CDS	1319985	1322546	+	/	putative tail component of prophage
i02_1335	CDS	1322543	1322872	+	/	putative tail fiber component M of prophage
i02_1336	CDS	1322872	1323570	+	/	putative tail component of prophage
i02_1337	CDS	1323621	1324319	+	/	putative tail fiber component K of prophage
i02_1338	CDS	1324283	1324897	+	/	putative tail assembly protein of cryptic
i02_1339	CDS	1325259	1328933	+	/	putative tail component of prophage
i02_1340	CDS	1329001	1329600	+	lomP	putative Lom-like outer membrane protein of
i02_1341	CDS	1329752	1331815	+	/	hypothetical protein
i02_1342	CDS	1331812	1332090	+	/	hypothetical protein
i02_1343	CDS	1332100	1332393	+	/	hypothetical protein
i02_1344	CDS	1332586	1333251	-	/	hypothetical protein
i02_1345	CDS	1333311	1333577	+	ylcE	hypothetical protein
i02_1346	CDS	1333809	1334672	-	potB	spermidine/putrescine ABC transporter membrane
i02_1347	CDS	1334656	1335792	-	potA	putrescine/spermidine ABC transporter ATPase
i02_1348	CDS	1335771	1336022	-	/	hypothetical protein
i02_1349	CDS	1336021	1337268	+	pepT	peptidase T
i02_1350	CDS	1337317	1338447	-	ycfD	hypothetical protein
i02_1351	CDS	1338514	1339974	-	phoQ	sensor protein PhoQ
i02_1352	CDS	1339974	1340645	-	phoP	DNA-binding transcriptional regulator PhoP
i02_1353	CDS	1340815	1342185	-	purB	adenylosuccinate lyase
i02_1354	CDS	1342189	1342836	-	ycfC	hypothetical protein
i02_1355	CDS	1342866	1344017	-	mnmA	tRNA-specific 2-thiouridylase MnmA
i02_1356	CDS	1344026	1344487	-	/	putative Nudix hydrolase ymfB
i02_1357	CDS	1344497	1345150	-	/	hypothetical protein
i02_1358	CDS	1345070	1345192	+	/	hypothetical protein
i02_1359	CDS	1345322	1346572	+	icdA	isocitrate dehydrogenase
i02_1360	CDS	1346686	1347828	-	/	prophage lambda integrase
i02_1361	CDS	1348194	1348433	-	/	hypothetical protein
i02_1362	CDS	1348459	1348827	-	/	hypothetical protein
i02_1363	CDS	1348736	1349476	-	/	hypothetical protein
i02_1364	CDS	1349473	1349724	-	/	hypothetical protein
i02_1365	CDS	1349684	1350244	-	/	hypothetical protein
i02_1366	CDS	1350235	1350510	-	/	hypothetical protein
i02_1367	CDS	1350491	1351255	-	/	hypothetical protein
i02_1369	CDS	1351572	1351853	-	/	hypothetical protein
i02_1370	CDS	1351864	1351977	-	/	hypothetical protein
i02_1371	CDS	1352028	1352216	-	/	hypothetical protein
i02_1372	CDS	1352207	1352887	-	/	putative exonuclease encoded by prophage
i02_1373	CDS	1352884	1353669	-	/	putative recombination protein Bet of prophage
i02_1374	CDS	1353675	1353971	-	gamW	putative host-nuclease inhibitor protein Gam of
i02_1375	CDS	1353940	1354092	-	/	hypothetical protein
i02_1376	CDS	1354159	1354323	-	/	lambda regulatory protein CIII
i02_1377	CDS	1354396	1354764	-	/	putative single-stranded DNA binding protein of

Locus_tag	Type	start	End	+/-[a]	Gene[b]	Product
i02_1378	CDS	1354947	1355132	-	/	lambda ant-restriction protein
i02_1379	CDS	1355361	1355942	+	/	putative superinfection exclusion protein B of
i02_1380	CDS	1355959	1356303	-	/	N protein
i02_1381	CDS	1356668	1357420	-	/	hypothetical protein
i02_1382	CDS	1357417	1357974	-	/	hypothetical protein
i02_1383	CDS	1358014	1358709	-	/	repressor protein
i02_1384	CDS	1358716	1359000	+	/	hypothetical protein
i02_1385	CDS	1359142	1359438	+	/	putative regulatory protein CII of
i02_1386	CDS	1359471	1360370	+	/	putative replication protein O of bacteriophage
i02_1387	CDS	1360367	1361068	+	/	putative replication protein P of bacteriophage
i02_1388	CDS	1361065	1361355	+	/	putative exclusion protein ren of prophage
i02_1389	CDS	1361429	1361869	+	/	hypothetical protein
i02_1390	CDS	1361806	1362393	+	/	putative DNA N-6-adenine-methyltransferase of
i02_1391	CDS	1362527	1362910	+	/	hypothetical protein
i02_1392	CDS	1363117	1363479	+	rus	endodeoxyribonuclease RUS
i02_1393	CDS	1363476	1363616	+	/	hypothetical protein
i02_1394	CDS	1363687	1364085	+	ybcQ	lambdoid prophage DLP12 antitermination protein
i02_1395	CDS	1364274	1365416	-	nmpC	Outer membrane porin protein nmpC precursor
i02_1396	CDS	1365945	1366160	+	/	lambdoid prophage DLP12 lysis protein S
i02_1397	CDS	1366160	1366657	+	ybcS	lysozyme from lambdoid prophage DLP12
i02_1398	CDS	1366642	1367115	+	/	putative Rz endopeptidase from lambdoid prophage
i02_1399	CDS	1367147	1367488	-	ybcU	Bor protein homolog from lambdoid prophage DLP12
i02_1400	CDS	1367867	1368247	+	/	partial tonB-like membrane protein encoded
i02_1401	CDS	1368370	1368723	-	/	hypothetical protein
i02_1403	CDS	1368798	1368914	-	/	hypothetical protein
i02_1404	CDS	1368863	1369117	+	nohA2	prophage Qin DNA packaging protein NU1-like protein
i02_1405	CDS	1369199	1369747	+	nohA	prophage Qin DNA packaging protein NU1-like protein
i02_1406	CDS	1369677	1371647	+	/	putative DNA packaging protein of prophage
i02_1407	CDS	1371631	1371837	+	/	putative DNA packaging protein of prophage
i02_1408	CDS	1371804	1373426	+	/	putative capsid protein of prophage
i02_1409	CDS	1374958	1375305	+	/	putative capsid protein of prophage
i02_1410	CDS	1375363	1376391	+	/	putative capsid protein of prophage
i02_1411	CDS	1376401	1376817	+	/	hypothetical protein
i02_1412	CDS	1376774	1377163	+	/	putative head-tail joining protein of prophage
i02_1413	CDS	1377175	1377753	+	/	putative tail fiber component Z of prophage
i02_1414	CDS	1377750	1378145	+	/	putative tail component of prophage
i02_1415	CDS	1378123	1378893	+	/	putative tail component of prophage
i02_1416	CDS	1378909	1379331	+	/	putative tail component of prophage
i02_1417	CDS	1379313	1379747	+	/	putative tail component of prophage
i02_1418	CDS	1379740	1382301	+	/	putative tail component of prophage
i02_1419	CDS	1382298	1382627	+	/	putative tail component of prophage
i02_1420	CDS	1382627	1383325	+	/	putative tail component of prophage
i02_1421	CDS	1383331	1384074	+	/	putative tail component of prophage
i02_1422	CDS	1383972	1384643	+	/	putative tail component of prophage
i02_1423	CDS	1384704	1388186	+	/	putative tail component of prophage
i02_1424	CDS	1388239	1390305	+	/	hypothetical protein
i02_1425	CDS	1390302	1390580	+	/	hypothetical protein
i02_1426	CDS	1390593	1390886	+	/	hypothetical protein
i02_1427	CDS	1390978	1391835	-	sitD	SitD protein
i02_1428	CDS	1391832	1392689	-	sitC	SitC protein
i02_1429	CDS	1392686	1393513	-	sitB	SitB protein
i02_1430	CDS	1393513	1394427	-	sitA	SitA protein
i02_1431	CDS	1395114	1395884	+	/	hypothetical protein
i02_1432	CDS	1396770	1397174	-	ycgX	hypothetical protein
i02_1433	CDS	1397395	1398126	-	ycgE	putative transcriptional regulator YcgE

Locus_tag	Type	start	End	+/-[a]	Gene[b]	Product
i02_1434	CDS	1398036	1398188	-	/	hypothetical protein
i02_1435	CDS	1398331	1399620	-	ycgF	hypothetical protein
i02_1436	CDS	1399856	1400092	+	ycgZ	hypothetical protein
i02_1437	CDS	1400135	1400407	+	/	hypothetical protein
i02_1438	CDS	1400436	1400702	+	ymgB	hypothetical protein
i02_1439	CDS	1400887	1401063	+	/	hypothetical protein
i02_1440	CDS	1401403	1402254	+	/	hypothetical protein
i02_1441	CDS	1402350	1402604	+	/	hypothetical protein
i02_1442	CDS	1403002	1403181	+	/	hypothetical protein
i02_1443	CDS	1403197	1403334	+	/	conserved hypothetical protein
i02_1444	CDS	1403471	1403554	+	/	hypothetical protein
i02_1445	CDS	1403665	1403847	+	/	hypothetical protein
i02_1446	CDS	1403993	1404520	+	/	hypothetical protein
i02_1447	CDS	1404578	1404898	-	ymgD	hypothetical protein
i02_1448	CDS	1404917	1405261	-	/	hypothetical protein
i02_1449	CDS	1405263	1405436	-	/	hypothetical protein
i02_1450	CDS	1405854	1406180	+	/	hypothetical protein
i02_1451	CDS	1406226	1406411	+	/	hypothetical protein
i02_1452	CDS	1406553	1406819	-	minE	cell division topological specificity factor
i02_1453	CDS	1406823	1407635	-	minD	cell division inhibitor MinD
i02_1454	CDS	1407659	1408354	-	minC	septum formation inhibitor
i02_1455	CDS	1408494	1408721	+	/	hypothetical protein
i02_1456	CDS	1408874	1409242	+	ycgJ	hypothetical protein
i02_1457	CDS	1409362	1409763	-	ycgK	hypothetical protein
i02_1458	CDS	1409972	1410298	+	ycgL	hypothetical protein
i02_1459	CDS	1410370	1411029	+	/	hypothetical protein
i02_1460	CDS	1411121	1411567	+	ycgN	hypothetical protein
i02_1461	CDS	1411774	1412055	-	/	hypothetical protein
i02_1462	CDS	1412360	1412779	+	umuD	DNA polymerase V subunit UmuD
i02_1463	CDS	1412779	1414047	+	umuC	DNA polymerase V subunit UmuC
i02_1464	CDS	1414092	1414628	-	dsbB	disulfide bond formation protein B
i02_1465	CDS	1414768	1416309	-	nhaB	sodium/proton antiporter
i02_1466	CDS	1416531	1417250	+	fadR	fatty acid metabolism regulator
i02_1467	CDS	1417238	1417363	-	/	hypothetical protein
i02_1468	CDS	1417413	1418945	-	ycgB	SpoVR family protein
i02_1469	CDS	1419269	1420573	+	dadA	D-amino acid dehydrogenase small subunit
i02_1470	CDS	1420583	1421653	+	dadX	alanine racemase
i02_1471	CDS	1421710	1423446	-	ycgO	potassium/proton antiporter
i02_1472	CDS	1423541	1424455	-	ldcA	L,D-carboxypeptidase A
i02_1473	CDS	1424441	1425166	+	mltE	membrane-bound lytic murein transglycosylase E
i02_1474	CDS	1425168	1425902	-	ycgR	hypothetical protein
i02_1475	CDS	1426070	1426357	+	ymgE	transglycosylase associated protein
i02_1476	CDS	1426407	1428398	-	prrA	outer membrane receptor
i02_1477	CDS	1428403	1429257	-	modD	molybdenum transport protein ModD
i02_1478	CDS	1429254	1430066	-	/	hypothetical protein
i02_1479	CDS	1430076	1430972	-	/	putative iron compound ABC transporter,
i02_1480	CDS	1430831	1431811	-	fecD	Iron(III) dicitrate transport system permease
i02_1481	CDS	1431811	1432833	-	/	hypothetical protein
i02_1482	CDS	1432901	1433026	-	/	hypothetical protein
i02_1483	CDS	1433164	1434861	-	treA	trehalase
i02_1484	CDS	1434806	1435021	+	/	hypothetical protein
i02_1485	CDS	1435040	1435153	-	/	hypothetical protein
i02_1486	CDS	1435180	1436604	-	ycgC	dihydroxyacetone kinase subunit M
i02_1487	CDS	1436609	1437319	-	/	dihydroxyacetone kinase ADP-binding subunit
i02_1488	CDS	1437252	1438352	-	ycgT	dihydroxyacetone kinase subunit DhaK
i02_1489	CDS	1438541	1440469	+	/	DNA-binding transcriptional regulator DhaR
i02_1490	CDS	1440506	1440736	-	/	hypothetical protein
i02_1491	CDS	1440874	1441965	-	ychF	GTP-dependent nucleic acid-binding protein EngD

Locus_tag	Type	start	End	+/-[a]	Gene[b]	Product
i02_1492	CDS	1442082	1442666	-	pth	peptidyl-tRNA hydrolase
i02_1493	CDS	1442944	1443222	+	ychH	hypothetical protein
i02_1494	CDS	1443277	1444956	-	ychM	putative sulfate transporter YchM
i02_1495	CDS	1445081	1446094	-	prsA	ribose-phosphate pyrophosphokinase
i02_1496	CDS	1446179	1447030	-	ipk	4-diphosphocytidyl-2-C-methylerythritol kinase
i02_1497	CDS	1447030	1447719	-	lolB	outer membrane lipoprotein LolB
i02_1498	CDS	1447867	1449123	+	hemA	glutamyl-tRNA reductase
i02_1499	CDS	1449165	1450247	+	prfA	peptide chain release factor 1
i02_1500	CDS	1450247	1451080	+	hemK	N5-glutamine S-adenosyl-L-methionine-dependent
i02_1501	CDS	1451077	1451469	+	/	putative transcriptional regulator
i02_1502	CDS	1451473	1452282	+	ychA	putative transcriptional regulator
i02_1503	CDS	1452318	1453172	+	kdsA	2-dehydro-3-deoxyphosphooctonate aldolase
i02_1504	CDS	1453321	1453599	-	/	hypothetical protein
i02_1505	CDS	1453633	1453752	-	/	hypothetical protein
i02_1506	CDS	1453838	1455025	-	chaA	calcium/sodium:proton antiporter
i02_1507	CDS	1455208	1455438	+	chaB	cation transport regulator
i02_1508	CDS	1455548	1456279	+	chaC	cation transport protein chaC
i02_1509	CDS	1456323	1456676	-	ychN	hypothetical protein
i02_1510	CDS	1456861	1458255	+	ychP	hypothetical protein
i02_1511	CDS	1458256	1458972	-	narL	transcriptional regulator NarL
i02_1512	CDS	1458899	1460695	-	narX	nitrate/nitrite sensor protein NarX
i02_1513	CDS	1460721	1460939	+	/	hypothetical protein
i02_1514	CDS	1461028	1462425	+	narK	nitrite extrusion protein 1
i02_1515	CDS	1462818	1466561	+	narG	respiratory nitrate reductase 1 alpha chain
i02_1516	CDS	1466558	1468096	+	narH	respiratory nitrate reductase 1 beta chain
i02_1517	CDS	1468093	1468803	+	narJ	respiratory nitrate reductase 1 delta chain
i02_1518	CDS	1468803	1469480	+	narI	respiratory nitrate reductase 1 gamma chain
i02_1519	CDS	1469556	1470599	+	/	hypothetical protein
i02_1520	CDS	1470596	1471612	+	/	hypothetical protein
i02_1521	CDS	1471590	1472396	+	/	putative gumP-like protein
i02_1522	CDS	1472393	1473679	+	/	hypothetical protein
i02_1523	CDS	1473664	1474278	+	/	hypothetical protein
i02_1524	CDS	1474271	1475395	+	/	hypothetical protein
i02_1525	CDS	1475382	1476473	+	/	hypothetical protein
i02_1527	CDS	1477030	1477872	-	purU	formyltetrahydrofolate deformylase
i02_1528	CDS	1477922	1478380	-	ychJ	hypothetical protein
i02_1529	CDS	1478454	1479398	+	ychK	hypothetical protein
i02_1530	CDS	1479490	1480503	+	hnr	response regulator of RpoS
i02_1531	CDS	1480705	1481613	+	galU	UTP--glucose-1-phosphate uridylyltransferase
i02_1532	CDS	1481757	1482170	-	hns	global DNA-binding transcriptional factor
i02_1533	CDS	1482262	1482390	-	/	hypothetical protein
i02_1534	CDS	1482774	1483391	+	tdk	thymidine kinase
i02_1535	CDS	1483518	1484723	-	/	transposase insG
i02_1537	CDS	1484849	1487524	-	adhE	bifunctional acetaldehyde-CoA/alcohol dehydrogenase
i02_1538	CDS	1488001	1488648	+	ychE	hypothetical protein
i02_1539	CDS	1488806	1489102	-	/	hypothetical protein
i02_1540	CDS	1489314	1491017	+	oppA	periplasmic oligopeptide-binding protein
i02_1541	CDS	1491103	1492023	+	oppB	oligopeptide transporter permease
i02_1542	CDS	1492038	1492946	+	oppC	oligopeptide transport system permease protein
i02_1543	CDS	1492958	1493971	+	oppD	oligopeptide transporter ATP-binding component
i02_1544	CDS	1493968	1494972	+	oppF	oligopeptide transport ATP-binding protein oppF
i02_1545	CDS	1495025	1495432	-	yciU	dsDNA-mimic protein
i02_1546	CDS	1495389	1496849	-	cls	cardiolipin synthetase
i02_1547	CDS	1496881	1497165	+	/	hypothetical protein
i02_1548	CDS	1497220	1498491	-	kch	voltage-gated potassium channel
i02_1549	CDS	1498773	1499069	-	yciI	YciI-like protein
i02_1550	CDS	1499242	1500009	+	tonB	transport protein TonB

Locus_tag	Type	start	End	+/-[a]	Gene[b]	Product
i02_1551	CDS	1500049	1500447	-	yciA	acyl-CoA thioester hydrolase
i02_1552	CDS	1500552	1501091	-	ispZ	intracellular septation protein A
i02_1553	CDS	1501121	1501864	-	yciC	hypothetical protein
i02_1554	CDS	1502143	1502859	+	ompW	outer membrane protein W
i02_1555	CDS	1502918	1503424	-	yciE	hypothetical protein
i02_1556	CDS	1503470	1503973	-	yciF	hypothetical protein
i02_1557	CDS	1504297	1505103	-	trpA	tryptophan synthase subunit alpha
i02_1558	CDS	1505103	1506296	-	trpB	tryptophan synthase subunit beta
i02_1559	CDS	1506308	1507666	-	trpC	bifunctional indole-3-glycerol phosphate synthase/phosphoribosylanthranilate isomerase
i02_1560	CDS	1507670	1509265	-	trpD	bifunctional glutamine
i02_1561	CDS	1509265	1510827	-	trpE	anthranilate synthase component I
i02_1562	CDS	1511101	1511982	+	trpH	protein trpH
i02_1563	CDS	1511943	1512599	+	yciO	hypothetical protein
i02_1564	CDS	1512624	1514516	+	yciQ	hypothetical protein
i02_1565	CDS	1514729	1515604	+	yciL	23S rRNA pseudouridylate synthase B
i02_1566	CDS	1515644	1516234	-	btuR	cobinamide adenosyltransferase / cobalamin adenosyltransferase
i02_1567	CDS	1516231	1516989	-	yciK	short chain dehydrogenase
i02_1568	CDS	1517209	1518258	+	sohB	putative periplasmic protease
i02_1569	CDS	1518294	1518545	-	yciN	hypothetical protein
i02_1570	CDS	1518538	1518693	+	/	hypothetical protein
i02_1571	CDS	1518883	1521522	+	topA	DNA topoisomerase I
i02_1572	CDS	1521732	1522706	+	cysB	transcriptional regulator CysB
i02_1573	CDS	1523001	1523165	+	/	hypothetical protein
i02_1574	CDS	1523147	1523335	+	/	hypothetical protein
i02_1575	CDS	1523694	1526369	+	acnA	aconitate hydratase
i02_1576	CDS	1526433	1527086	-	ribA	GTP cyclohydrolase II
i02_1577	CDS	1527193	1527957	+	pgpB	phosphatidylglycerophosphatase B
i02_1578	CDS	1528106	1528414	+	yciS	hypothetical protein
i02_1579	CDS	1528448	1529590	+	yciM	tetratricopeptide repeat protein
i02_1580	pseudo	1529630	1530427	+	pyrF	orotidine 5'-phosphate decarboxylase
i02_1581	CDS	1530515	1530844	+	yciH	translation initiation factor Sui1
i02_1582	CDS	1530868	1531020	+	/	hypothetical protein
i02_1583	CDS	1530970	1531188	-	osmB	lipoprotein
i02_1584	CDS	1531457	1532206	-	yciT	putative transcriptional regulator YciT
i02_1585	CDS	1532295	1532468	-	/	hypothetical protein
i02_1586	CDS	1532616	1534601	-	yciR	RNase II stability modulator
i02_1587	CDS	1534835	1536769	-	rnb	exoribonuclease II
i02_1588	CDS	1536837	1537964	-	yciW	hypothetical protein
i02_1589	CDS	1538109	1538897	-	fabI	enoyl-(acyl carrier protein) reductase
i02_1590	CDS	1539276	1539941	+	/	putative transcriptional repressor
i02_1591	CDS	1540057	1541211	+	/	acriflavin resistance protein A
i02_1593	pseudo	1541211	1544317	+	/	acriflavin resistance protein B
i02_1594	CDS	1544321	1545694	+	/	partial putative outer membrane channel protein
i02_1595	CDS	1545703	1546866	+	/	membrane transporter
i02_1596	CDS	1546914	1547720	-	sapF	peptide transport system ATP-binding protein
i02_1597	CDS	1547722	1548714	-	sapD	peptide transport system ATP-binding protein
i02_1598	CDS	1548714	1549604	-	sapC	peptide transport system permease protein sapC
i02_1599	CDS	1549591	1550556	-	sapB	peptide transport system permease protein sapB
i02_1600	CDS	1550553	1552196	-	sapA	peptide transport periplasmic protein sapA
i02_1601	CDS	1552509	1552754	-	ymjA	hypothetical protein
i02_1602	CDS	1553181	1554173	-	pspF	phage shock protein
i02_1603	CDS	1554324	1554992	+	pspA	phage shock protein PspA
i02_1604	CDS	1555046	1555270	+	pspB	phage shock protein B
i02_1605	CDS	1555270	1555629	+	pspC	DNA-binding transcriptional activator PspC
i02_1606	CDS	1555638	1555859	+	pspD	peripheral inner membrane phage-shock protein
i02_1607	CDS	1555934	1556248	+	pspE	thiosulfate:cyanide sulfurtransferase

Locus_tag	Type	start	End	+/-[a]	Gene[b]	Product
i02_1608	CDS	1556435	1558141	+	ycjM	putative sucrose phosphorylase
i02_1609	CDS	1558155	1559447	+	ycjN	ABC transporter periplasmic-binding protein
i02_1610	CDS	1559468	1560349	+	ycjO	ABC transporter permease
i02_1611	CDS	1560336	1561178	+	ycjP	ABC transporter permease
i02_1612	CDS	1561209	1562261	+	ycjQ	hypothetical protein
i02_1613	CDS	1562271	1563068	+	ycjR	hypothetical protein
i02_1614	CDS	1563078	1564133	+	ycjS	oxidoreductase ycjS
i02_1615	CDS	1564130	1566397	+	ycjT	putative transport protein YcjT
i02_1616	CDS	1566394	1567053	+	ycjU	putative beta-phosphoglucomutase
i02_1617	CDS	1567067	1568149	+	ycjV	ABC transporter ATP-binding protein
i02_1618	CDS	1568143	1569099	+	ompG	Outer membrane protein G precursor
i02_1619	CDS	1569150	1570148	-	ycjW	putative transcriptional regulator YcjW
i02_1620	CDS	1570232	1571701	+	ycjX	hypothetical protein
i02_1621	CDS	1571698	1572759	+	ycjF	hypothetical protein
i02_1622	CDS	1572880	1574448	+	tyrR	DNA-binding transcriptional regulator TyrR
i02_1623	CDS	1574492	1574998	-	tpx	thiol peroxidase
i02_1624	CDS	1575075	1576082	+	ycjG	hypothetical protein
i02_1625	pseudo	1576057	1576785	-	ycjI	murein peptide amidase A
i02_1626	CDS	1577076	1577714	-	/	hypothetical protein
i02_1627	CDS	1577785	1578717	-	/	hypothetical protein
i02_1628	CDS	1578843	1579742	+	/	putative transcriptional regulator YcjZ
i02_1629	CDS	1580058	1581692	+	mppA	periplasmic murein peptide-binding protein
i02_1630	CDS	1581743	1582774	-	ynaI	hypothetical protein
i02_1631	CDS	1583018	1583275	+	ynaJ	hypothetical protein
i02_1632	CDS	1583325	1584275	-	ydaA	universal stress protein UspE
i02_1633	CDS	1584427	1585221	-	fnr	fumarate/nitrate reduction transcriptional regulator
i02_1634	CDS	1585374	1585889	-	ogt	O-6-alkylguanine-DNA:cysteine-protein
i02_1635	CDS	1585900	1586091	-	/	hypothetical protein
i02_1636	CDS	1586402	1587217	-	/	hypothetical protein
i02_1637	CDS	1587311	1587619	+	/	hypothetical protein
i02_1638	CDS	1587604	1588002	+	/	hypothetical protein
i02_1639	CDS	1588197	1588760	+	ydaL	hypothetical protein
i02_1640	CDS	1588781	1590073	-	ydaM	hypothetical protein
i02_1641	CDS	1590268	1591251	+	zntB	zinc transporter
i02_1642	CDS	1591729	1593102	+	dbpA	ATP-dependent RNA helicase DbpA
i02_1643	CDS	1593231	1594166	-	ydaO	C32 tRNA thiolase
i02_1644	CDS	1594218	1594526	-	/	hypothetical protein
i02_1645	CDS	1596035	1596403	-	/	hypothetical protein
i02_1646	CDS	1596492	1596998	-	ynaF	hypothetical protein
i02_1647	CDS	1597067	1598200	-	ompN	Outer membrane protein N precursor
i02_1648	CDS	1598566	1602090	-	ydbK	pyruvate-flavodoxin oxidoreductase
i02_1649	CDS	1602161	1602601	-	/	hypothetical protein
i02_1650	CDS	1602475	1602630	+	/	hypothetical protein
i02_1651	CDS	1602627	1603061	-	hslJ	heat-inducible protein
i02_1652	CDS	1603160	1604149	-	ldhA	D-lactate dehydrogenase
i02_1653	CDS	1604357	1606996	+	ydbH	hypothetical protein
i02_1654	CDS	1606993	1607178	+	ynbE	hypothetical protein
i02_1655	CDS	1607111	1607512	+	ydbL	hypothetical protein
i02_1656	CDS	1607905	1613778	+	/	possible autotransporter/adhesin
i02_1657	CDS	1613997	1614857	+	ydbC	putative oxidoreductase
i02_1658	CDS	1614902	1615126	-	/	hypothetical protein
i02_1659	CDS	1615156	1615761	+	ynbA	hypothetical protein
i02_1660	CDS	1615761	1616657	+	ynbB	hypothetical protein
i02_1661	CDS	1616673	1618430	+	ynbC	hypothetical protein
i02_1662	CDS	1618345	1619736	+	ynbD	hypothetical protein
i02_1663	CDS	1619790	1620395	-	acpD	azoreductase
i02_1664	CDS	1620596	1624498	+	hrpA	ATP-dependent RNA helicase HrpA
i02_1665	CDS	1624771	1625571	+	ydcF	hypothetical protein

Locus_tag	Type	start	End	+/-[a]	Gene[b]	Product
i02_1666	CDS	1625768	1627207	+	aldA	aldehyde dehydrogenase A
i02_1667	CDS	1627249	1628250	-	/	glyceraldehyde 3-phosphate dehydrogenase A
i02_1668	CDS	1628439	1628969	+	cybB	cytochrome b561
i02_1669	CDS	1629214	1629387	+	ydcA	hypothetical protein
i02_1670	CDS	1629547	1630014	+	/	hypothetical protein
i02_1671	CDS	1630052	1631116	-	ycdI	putative transcriptional regulator YdcI
i02_1672	CDS	1631192	1632535	+	ydcJ	hypothetical protein
i02_1673	CDS	1632760	1634415	+	mdoD	glucan biosynthesis protein D
i02_1674	CDS	1634549	1634779	+	ydcH	hypothetical protein
i02_1675	CDS	1634827	1635381	+	rimL	ribosomal-protein-L7/L12-serine
i02_1676	CDS	1635373	1636353	-	ydcK	hypothetical protein
i02_1677	CDS	1636369	1637469	+	tehA	potassium-tellurite ethidium and proflavin
i02_1678	CDS	1637466	1638059	+	tehB	tellurite resistance protein TehB
i02_1679	CDS	1638211	1638330	+	/	hypothetical protein
i02_1680	CDS	1638362	1639030	+	ydcL	lipoprotein ydcL precursor
i02_1681	CDS	1639065	1640360	-	ydcO	hypothetical protein
i02_1682	CDS	1640329	1640865	+	ydcN	hypothetical protein
i02_1683	CDS	1640896	1642899	+	ydcP	putative protease ydcP precursor
i02_1684	CDS	1642991	1643221	-	yncJ	hypothetical protein
i02_1685	CDS	1643278	1643406	-	/	hypothetical protein
i02_1686	CDS	1643433	1643636	+	/	hypothetical protein
i02_1687	CDS	1643715	1645121	+	ydcR	hypothetical protein
i02_1688	CDS	1645366	1646511	+	ydcS	ABC transporter periplasmic-binding protein
i02_1689	CDS	1646529	1647542	+	ydcT	ABC transporter ATP-binding protein
i02_1690	CDS	1647543	1648484	+	ydcU	ABC transporter permease
i02_1691	CDS	1648474	1649268	+	ydcV	ABC transporter permease
i02_1692	CDS	1649290	1650714	+	ydcW	gamma-aminobutyraldehyde dehydrogenase
i02_1693	CDS	1650711	1650827	-	/	hypothetical protein
i02_1694	CDS	1650903	1651181	-	/	hypothetical protein
i02_1695	CDS	1651026	1651274	+	/	hypothetical protein
i02_1696	CDS	1651360	1651593	+	ydc	hypothetical protein
i02_1697	CDS	1651594	1652043	-	ydcZ	hypothetical protein
i02_1698	CDS	1652040	1652621	-	yncA	acetyltransferase yncA
i02_1699	CDS	1652551	1652892	+	/	hypothetical protein
i02_1700	CDS	1652765	1653001	+	/	hypothetical protein
i02_1701	CDS	1653039	1655141	-	yncD	iron outer membrane transporter
i02_1702	CDS	1655383	1656444	+	yncE	hypothetical protein
i02_1703	CDS	1656557	1658107	-	ansP	L-asparagine permease
i02_1704	CDS	1658323	1658499	+	/	hypothetical protein
i02_1705	CDS	1658612	1658941	+	/	hypothetical protein
i02_1706	CDS	1659361	1659510	-	/	hypothetical protein
i02_1707	CDS	1659482	1659766	+	/	hypothetical protein
i02_1708	CDS	1659785	1661743	+	/	hypothetical protein
i02_1709	CDS	1661875	1662582	+	/	hypothetical protein
i02_1710	CDS	1662919	1664154	+	/	hypothetical protein
i02_1711	CDS	1664165	1665208	+	/	hypothetical protein
i02_1712	CDS	1665320	1665604	+	/	hypothetical protein
i02_1713	CDS	1665623	1668028	+	/	hypothetical protein
i02_1714	CDS	1668015	1668617	+	/	hypothetical protein
i02_1715	CDS	1668955	1669407	+	/	hypothetical protein
i02_1717	CDS	1669626	1670597	+	/	hypothetical protein
i02_1718	CDS	1670710	1670853	+	/	hypothetical protein
i02_1719	CDS	1670931	1671146	+	/	hypothetical protein
i02_1720	CDS	1671463	1672032	-	yddH	hypothetical protein
i02_1721	CDS	1672207	1673052	+	nhoA	N-hydroxyarylamine O-acetyltransferase
i02_1722	CDS	1673148	1674041	-	yddE	hypothetical protein
i02_1723	CDS	1674121	1674801	-	narV	respiratory nitrate reductase 2 gamma chain
i02_1724	CDS	1674798	1675493	-	narW	respiratory nitrate reductase 2 delta chain

Locus_tag	Type	start	End	+/-[a]	Gene[b]	Product
i02_1725	CDS	1675493	1677037	-	narY	respiratory nitrate reductase 2 beta chain
i02_1726	CDS	1677034	1680897	-	narZ	respiratory nitrate reductase 2 alpha chain
i02_1727	CDS	1680869	1682275	-	narU	nitrite extrusion protein 2
i02_1728	CDS	1682530	1683411	-	yddG	hypothetical protein
i02_1729	pseudo	1683644	1686691	+	fdnG	formate dehydrogenase
i02_1730	CDS	1686704	1687588	+	fdnH	formate dehydrogenase-N beta subunit
i02_1731	CDS	1687563	1688234	+	fdnI	formate dehydrogenase-N subunit gamma
i02_1732	CDS	1688463	1688747	-	yddM	hypothetical protein
i02_1733	CDS	1688747	1689025	-	/	hypothetical protein
i02_1734	CDS	1689211	1690251	-	adhP	alcohol dehydrogenase
i02_1735	CDS	1690355	1692052	-	sfcA	malate dehydrogenase
i02_1736	CDS	1692209	1692346	-	rpsV	30S ribosomal subunit S22
i02_1737	CDS	1692448	1692726	-	/	biofilm-dependent modulation protein
i02_1738	CDS	1692830	1692970	+	/	hypothetical protein
i02_1739	CDS	1693008	1693439	+	osmC	osmotically inducible protein C
i02_1740	CDS	1693628	1696051	-	/	putative sensor kinase
i02_1741	CDS	1696052	1696270	-	/	hypothetical protein
i02_1742	CDS	1696594	1697913	-	yddW	lipoprotein yddW precursor
i02_1743	CDS	1698044	1699579	-	xasA	amino acid antiporter
i02_1744	CDS	1699735	1701204	-	gadB	glutamate decarboxylase beta
i02_1745	CDS	1701497	1704292	-	pqqL	zinc protease pqqL
i02_1746	CDS	1704337	1706709	-	yddB	hypothetical protein
i02_1747	CDS	1706747	1708432	-	yddA	ABC transporter ATP-binding protein
i02_1748	CDS	1708723	1709895	-	ydeM	hypothetical protein
i02_1749	CDS	1709932	1711647	-	ydeN	putative sulfatase ydeN precursor
i02_1750	CDS	1712016	1712777	-	ydeO	transcriptional regulator YdeO
i02_1751	CDS	1712853	1713050	-	/	hypothetical protein
i02_1752	CDS	1713298	1715577	-	ydeP	putative oxidoreductase
i02_1753	CDS	1715916	1716830	-	ydeQ	fimbrial-like protein ydeQ precursor
i02_1754	CDS	1716890	1717393	-	ydeR	fimbrial-like protein ydeR precursor
i02_1755	CDS	1717406	1717936	-	ydeS	fimbrial-like protein ydeS precursor
i02_1756	CDS	1717950	1720601	-	/	Outer membrane usher protein fimD precursor
i02_1757	CDS	1720643	1721362	-	/	chaperone protein fimC precursor
i02_1758	CDS	1721715	1722326	-	/	Type-1 fimbrial protein, A chain precursor
i02_1759	CDS	1723054	1723353	-	/	hypothetical protein
i02_1760	CDS	1723579	1724031	+	/	hypothetical protein
i02_1761	CDS	1724290	1725669	-	hipA	protein hipA
i02_1762	CDS	1725612	1725896	-	hipB	DNA-binding transcriptional regulator HipB
i02_1763	CDS	1726186	1726944	+	tam	trans-aconitate 2-methyltransferase
i02_1764	CDS	1726948	1727913	-	yneE	hypothetical protein
i02_1765	CDS	1728059	1729510	-	uxaB	altronate oxidoreductase
i02_1766	CDS	1729737	1731155	-	yneF	hypothetical protein
i02_1767	CDS	1731294	1731653	-	yneG	hypothetical protein
i02_1768	CDS	1731653	1732579	-	yneH	glutaminase
i02_1769	CDS	1732643	1734055	-	yneI	putative succinate semialdehyde dehydrogenase
i02_1770	CDS	1734132	1735013	+	yneJ	putative transcriptional regulator YneJ
i02_1771	CDS	1735091	1735210	+	/	hypothetical protein
i02_1772	CDS	1735352	1736542	+	sotB	sugar efflux transporter
i02_1773	CDS	1736567	1737232	-	marC	multiple drug resistance protein MarC
i02_1774	CDS	1737444	1737878	+	marR	DNA-binding transcriptional repressor MarR
i02_1775	CDS	1737893	1738282	+	marA	DNA-binding transcriptional activator MarA
i02_1776	CDS	1738314	1738532	+	marB	hypothetical protein
i02_1777	CDS	1738589	1740046	-	celA	6-phospho-beta-glucosidase
i02_1778	CDS	1740053	1741726	-	/	hypothetical protein
i02_1779	CDS	1741781	1742095	-	/	PTS system, cellobiose-specific IIA component
i02_1780	CDS	1742120	1743481	-	/	hypothetical protein
i02_1781	CDS	1743557	1743868	-	/	PTS system, cellobiose-specific IIB component
i02_1782	CDS	1744067	1744765	+	/	hypothetical protein

Locus_tag	Type	start	End	+/-[a]	Gene[b]	Product
i02_1783	CDS	1744810	1745709	-	ydeD	O-acetylserine/cysteine export protein
i02_1784	CDS	1745904	1747091	+	ydeF	putative MFS-type transporter YdeE
i02_1785	CDS	1747533	1748429	-	ydeH	hypothetical protein
i02_1786	CDS	1748677	1748838	-	/	conserved hypothetical protein
i02_1787	CDS	1748930	1751041	-	dcp	dipeptidyl carboxypeptidase II
i02_1788	CDS	1751097	1751858	+	ydfG	3-hydroxy acid dehydrogenase
i02_1789	CDS	1751947	1752633	+	ydfH	putative transcriptional regulator YdfH
i02_1790	CDS	1752810	1753013	+	ydfZ	hypothetical protein
i02_1791	CDS	1753049	1754509	-	/	oxidoreductase ydfI
i02_1792	CDS	1754598	1755965	-	ydfJ	metabolite transport protein
i02_1793	CDS	1756023	1757042	-	rspB	putative dehydrogenase
i02_1794	CDS	1757054	1758301	-	rspA	starvation sensing protein rspA
i02_1795	CDS	1758500	1758835	-	ynfA	hypothetical protein
i02_1796	CDS	1758961	1759302	+	ynfB	hypothetical protein
i02_1797	CDS	1759337	1759897	+	speG	spermidine N(1)-acetyltransferase
i02_1798	CDS	1759900	1760646	-	ynfC	hypothetical protein
i02_1799	CDS	1760676	1761023	+	ynfD	hypothetical protein
i02_1800	CDS	1761222	1763648	+	ynfE	putative dimethyl sulfoxide reductase chain ynfE
i02_1801	CDS	1763706	1766132	+	ynfF	dimethyl sulfoxide reductase chain ynfF
i02_1802	CDS	1766143	1766760	+	ynfG	anaerobic dimethyl sulfoxide reductase chain
i02_1803	CDS	1766522	1767616	+	ynfH	anaerobic dimethyl sulfoxide reductase chain
i02_1804	CDS	1767650	1768273	+	ynfI	twin-argninine leader-binding protein DmsD
i02_1805	CDS	1768468	1769724	+	ynfJ	putative voltage-gated ClC-type chloride channel
i02_1806	CDS	1769677	1770384	-	bioD	putative dithiobiotin synthetase
i02_1807	CDS	1770497	1771717	-	mlc	Mlc protein
i02_1808	CDS	1771852	1772745	-	ynfL	putative transcriptional regulator YnfL
i02_1809	CDS	1772852	1774105	+	ynfM	putative transport protein YnfM
i02_1810	CDS	1775101	1775922	+	/	putative protease ydgD precursor
i02_1811	CDS	1775961	1776290	-	ydgE	multidrug efflux system protein MdtI
i02_1812	CDS	1776277	1776642	-	ydgF	multidrug efflux system protein MdtJ
i02_1813	CDS	1777054	1778088	+	tqsA	putative transport protein
i02_1814	CDS	1778113	1779501	-	pntB	pyridine nucleotide transhydrogenase
i02_1815	CDS	1779512	1781098	-	pntA	NAD(P) transhydrogenase subunit alpha
i02_1816	CDS	1781568	1782512	+	ydgH	hypothetical protein
i02_1817	CDS	1782671	1784080	+	ydgI	putative arginine/ornithine antiporter
i02_1818	CDS	1784117	1784839	+	ydgB	short chain dehydrogenase
i02_1819	CDS	1784836	1785171	-	ydgC	hypothetical protein
i02_1820	CDS	1785240	1786019	+	rstA	DNA-binding transcriptional regulator RstA
i02_1821	CDS	1786023	1787324	+	rstB	sensor protein RstB
i02_1822	CDS	1787388	1788329	+	tus	DNA replication terminus site-binding protein
i02_1823	CDS	1788326	1789729	-	fumC	fumarate hydratase
i02_1824	CDS	1789872	1791518	-	fumA	fumarate hydratase class I, aerobic
i02_1825	CDS	1791717	1792892	+	manA	mannose-6-phosphate isomerase
i02_1826	CDS	1792993	1794501	+	ydgA	hypothetical protein
i02_1827	CDS	1794546	1795811	-	uidC	putative outer membrane porin protein
i02_1828	CDS	1795850	1797223	-	uidB	glucuronide transporter
i02_1829	CDS	1797220	1799031	-	uidA	beta-D-glucuronidase
i02_1830	CDS	1799421	1800011	-	uidR	uid operon repressor
i02_1831	CDS	1800240	1801007	-	hdhA	7-alpha-hydroxysteroid dehydrogenase
i02_1832	CDS	1801119	1802147	-	malI	DNA-binding transcriptional repressor MalI
i02_1833	CDS	1802322	1803914	+	malX	bifunctional maltose and glucose-specific PTS
i02_1834	CDS	1803924	1805096	+	malY	cystathionine beta-lyase
i02_1835	CDS	1805200	1806201	+	add	adenosine deaminase
i02_1836	CDS	1806237	1807316	-	ydgJ	putative oxidoreductase
i02_1837	CDS	1807734	1807997	+	/	hypothetical protein
i02_1838	CDS	1807918	1808133	+	ydgT	oriC-binding nucleoid-associated protein
i02_1839	CDS	1808195	1808659	+	ydgK	hypothetical protein
i02_1840	CDS	1808736	1809317	+	/	Na(+)-translocating NADH-quinone reductase

Locus_tag	Type	start	End	+/-[a]	Gene[b]	Product
i02_1841	CDS	1809317	1809895	+	/	electron transport complex protein RnfB
i02_1842	CDS	1809888	1812116	+	/	electron transport complex protein RnfC
i02_1843	CDS	1812117	1813175	+	rnfD	electron transport complex protein RnfD
i02_1844	CDS	1813179	1813799	+	/	electron transport complex protein RnfG
i02_1845	CDS	1813803	1814498	+	ydgQ	electron transport complex RsxE subunit
i02_1846	CDS	1814498	1815133	+	nth	endonuclease III
i02_1847	CDS	1815321	1815461	+	/	hypothetical protein
i02_1848	CDS	1815744	1817246	+	tppB	putative tripeptide transporter permease
i02_1849	CDS	1817352	1817957	+	gst	glutathionine S-transferase
i02_1850	CDS	1818001	1818864	-	pdxY	pyridoxamine kinase
i02_1851	CDS	1818923	1820209	-	tyrS	tyrosyl-tRNA synthetase
i02_1852	CDS	1820326	1820982	-	pdxH	pyridoxamine 5'-phosphate oxidase
i02_1853	CDS	1821041	1821370	-	ydhA	lysozyme inhibitor
i02_1854	CDS	1821468	1822577	-	anmK	anhydro-N-acetylmuramic acid kinase
i02_1855	CDS	1822851	1823318	+	slyB	Outer membrane lipoprotein slyB precursor
i02_1856	CDS	1823365	1823805	-	slyA	transcriptional regulator SlyA
i02_1857	CDS	1823824	1823943	-	/	hypothetical protein
i02_1858	CDS	1824000	1824236	+	ydhI	hypothetical protein
i02_1859	CDS	1824197	1825096	+	ydhJ	hypothetical protein
i02_1860	CDS	1825096	1827108	+	ydhK	hypothetical protein
i02_1861	CDS	1827109	1827681	-	sodC	superoxide dismutase
i02_1862	CDS	1827711	1828607	-	ydhF	oxidoreductase ydhF
i02_1863	CDS	1828656	1829033	-	ydhL	hypothetical protein
i02_1864	CDS	1828998	1829597	+	ydhM	putative transcriptional regulator YdhM
i02_1865	CDS	1829634	1830731	+	nemA	N-ethylmaleimide reductase
i02_1866	CDS	1830812	1831219	+	gloA	glyoxalase I
i02_1867	CDS	1831322	1831969	+	rnt	ribonuclease T
i02_1868	CDS	1832031	1832174	-	/	hypothetical protein
i02_1869	CDS	1832062	1832205	+	/	hypothetical protein
i02_1870	CDS	1832341	1832688	-	ydhD	hypothetical protein
i02_1871	CDS	1833023	1833850	+	ydhO	hypothetical protein
i02_1872	CDS	1833978	1834559	+	sodB	superoxide dismutase
i02_1873	CDS	1834705	1835874	-	ydhP	putative transport protein YdhP
i02_1874	CDS	1836040	1836159	-	/	hypothetical protein
i02_1875	CDS	1836428	1837453	+	purR	DNA-binding transcriptional repressor PurR
i02_1876	CDS	1837450	1838382	-	ydhB	putative DNA-binding transcriptional regulator
i02_1877	CDS	1838495	1839706	+	ydhC	inner membrane transport protein YdhC
i02_1878	CDS	1839997	1841145	+	cfa	cyclopropane fatty acyl phospholipid synthase
i02_1879	CDS	1841185	1841826	-	ribE	riboflavin synthase subunit alpha
i02_1880	CDS	1842041	1843414	+	ydhE	multidrug efflux protein
i02_1881	CDS	1843455	1844711	-	ydhQ	hypothetical protein
i02_1882	CDS	1845285	1845590	+	/	hypothetical protein
i02_1883	CDS	1845716	1847326	+	ydhS	hypothetical protein
i02_1884	CDS	1847332	1848144	-	ydhT	hypothetical protein
i02_1885	CDS	1847533	1848279	+	/	hypothetical protein
i02_1886	CDS	1848148	1848933	-	ydhU	PhsC protein
i02_1887	CDS	1848930	1849649	-	/	putative ferredoxin-like protein ydhX
i02_1888	CDS	1849662	1850309	-	ydhW	hypothetical protein
i02_1889	CDS	1850313	1852415	-	ydhV	putative oxidoreductase
i02_1890	CDS	1852436	1853062	-	/	hypothetical protein
i02_1891	CDS	1853518	1853727	-	ydhZ	hypothetical protein
i02_1892	CDS	1853839	1853967	+	/	hypothetical protein
i02_1893	CDS	1854069	1855697	+	pykF	pyruvate kinase
i02_1894	CDS	1856008	1856244	+	lpp	major outer membrane lipoprotein precursor
i02_1895	CDS	1856307	1857311	-	ynhG	hypothetical protein
i02_1896	CDS	1857460	1857876	-	ynhA	cysteine desufuration protein SufE
i02_1897	CDS	1857889	1859109	-	/	bifunctional cysteine desulfurase/selenocysteine
i02_1898	CDS	1859106	1860377	-	ynhC	cysteine desulfurase activator complex subunit

Locus_tag	Type	start	End	+/-[a]	Gene[b]	Product
i02_1899	CDS	1860352	1861098	-	sufC	cysteine desulfurase ATPase component
i02_1900	CDS	1861108	1862634	-	ynhE	cysteine desulfurase activator complex subunit
i02_1901	CDS	1862604	1862972	-	sufA	iron-sulfur cluster assembly scaffold protein
i02_1902	CDS	1863521	1863790	-	ydiH	hypothetical protein
i02_1903	CDS	1863809	1864219	-	ydiI	hypothetical protein
i02_1904	CDS	1864216	1867272	-	ydiJ	hypothetical protein
i02_1905	CDS	1867486	1868598	+	ydiK	putative inner membrane protein
i02_1906	CDS	1869000	1869383	+	ydiL	hypothetical protein
i02_1907	CDS	1869483	1870697	+	ydiM	putative transport protein YdiM
i02_1908	CDS	1870918	1872189	+	ydiN	putative transport protein YdiN
i02_1909	CDS	1872201	1873067	+	aroE	quinate/shikimate dehydrogenase
i02_1910	CDS	1873098	1873856	+	aroD	3-dehydroquinate dehydratase
i02_1911	CDS	1874002	1875597	+	ydiF	hypothetical protein
i02_1912	CDS	1875611	1876762	+	ydiO	putative acyl-CoA dehydrogenase
i02_1913	CDS	1876805	1877716	-	ydiP	putative transcriptional regulator YdiP
i02_1914	CDS	1877819	1877995	-	/	hypothetical protein
i02_1915	CDS	1878011	1878796	+	ydiQ	putative electron transfer flavoprotein YdiQ
i02_1916	CDS	1878816	1879754	+	ydiR	electron transfer flavoprotein subunit YdiR
i02_1917	CDS	1879809	1881098	+	ydiS	hypothetical protein
i02_1918	CDS	1881095	1881388	+	ydiT	ferredoxin-like protein ydiT
i02_1919	CDS	1881391	1883091	+	ydiD	short chain acyl-CoA synthetase
i02_1920	CDS	1883148	1885526	-	ppsA	phosphoenolpyruvate synthase
i02_1921	CDS	1885859	1886692	+	ydiA	hypothetical protein
i02_1922	CDS	1886849	1887895	+	aroH	phospho-2-dehydro-3-deoxyheptonate aldolase
i02_1923	CDS	1888000	1888218	+	ydiE	hypothetical protein
i02_1924	CDS	1888222	1889658	-	ydiU	hypothetical protein
i02_1925	CDS	1889721	1890434	-	ydiV	hypothetical protein
i02_1926	CDS	1890681	1891145	-	nlpC	lipoprotein NlpC
i02_1927	CDS	1891223	1891972	-	btuD	vitamin B12-transporter ATPase
i02_1928	CDS	1891972	1892523	-	btuE	putative glutathione peroxidase
i02_1929	CDS	1892585	1893565	-	btuC	vtamin B12-transporter permease
i02_1930	CDS	1893462	1893590	-	/	hypothetical protein
i02_1931	CDS	1893666	1893965	-	ihfA	integration host factor subunit alpha
i02_1932	CDS	1893970	1896357	-	pheT	phenylalanyl-tRNA synthetase subunit beta
i02_1933	CDS	1896372	1897355	-	pheS	phenylalanyl-tRNA synthetase subunit alpha
i02_1934	CDS	1897806	1898162	-	rplT	50S ribosomal protein L20
i02_1935	CDS	1898284	1898895	+	/	hypothetical protein
i02_1936	CDS	1898509	1898943	-	infC	translation initiation factor IF-3
i02_1937	CDS	1899055	1900983	-	thrS	threonyl-tRNA synthetase
i02_1938	CDS	1901669	1902034	+	/	hypothetical protein
i02_1939	CDS	1902052	1902363	+	/	hypothetical protein
i02_1940	CDS	1902344	1902535	+	/	hypothetical protein
i02_1941	CDS	1902691	1902813	+	/	hypothetical protein
i02_1942	CDS	1902866	1903624	-	ydiY	hypothetical protein
i02_1943	CDS	1903908	1904840	+	pfkB	6-phosphofructokinase 2
i02_1944	CDS	1904941	1905231	+	ydiZ	hypothetical protein
i02_1945	CDS	1905337	1906197	+	yniA	hypothetical protein
i02_1946	CDS	1906238	1906774	-	yniB	hypothetical protein
i02_1947	CDS	1906921	1907589	+	yniC	2-deoxyglucose-6-phosphatase
i02_1948	CDS	1907741	1908343	+	ydjM	hypothetical protein
i02_1949	CDS	1908476	1909867	+	ydjN	putative symporter ydjN
i02_1950	CDS	1909914	1910177	-	/	cell division modulator
i02_1951	CDS	1910360	1912621	+	katE	hydroperoxidase II
i02_1952	CDS	1912668	1913426	-	ydjC	hypothetical protein
i02_1953	CDS	1913439	1914845	-	celF	6-phospho-beta-glucosidase
i02_1954	CDS	1914894	1915736	-	celD	DNA-binding transcriptional regulator ChbR
i02_1955	CDS	1915744	1916094	-	celC	N,N'-diacetylchitobiose-specific PTS system
i02_1956	CDS	1916145	1917503	-	celB	N,N'-diacetylchitobiose-specific PTS system

Locus_tag	Type	start	End	+/-[a]	Gene[b]	Product
i02_1957	CDS	1917588	1917908	-	celA	N,N'-diacetylchitobiose-specific PTS system
i02_1958	CDS	1918208	1918546	-	osmE	DNA-binding transcriptional activator OsmE
i02_1959	CDS	1918748	1919575	+	nadE	NAD synthetase
i02_1960	CDS	1919805	1920692	+	ydjQ	nucleotide excision repair endonuclease
i02_1961	CDS	1920652	1921290	-	ydjR	hypothetical protein
i02_1962	CDS	1921430	1921915	-	spy	periplasmic protein
i02_1963	CDS	1922245	1923213	-	ydjS	succinylglutamate desuccinylase
i02_1964	CDS	1923206	1924549	-	/	succinylarginine dihydrolase
i02_1965	CDS	1924546	1926024	-	astD	succinylglutamic semialdehyde dehydrogenase
i02_1966	CDS	1926021	1927055	-	/	arginine succinyltransferase
i02_1967	CDS	1927052	1928272	-	argD	bifunctional succinylornithine
i02_1968	CDS	1928535	1928705	+	/	hypothetical protein
i02_1969	CDS	1928706	1929524	+	xthA	exonuclease III
i02_1970	CDS	1929643	1930401	+	ydjX	hypothetical protein
i02_1971	CDS	1930244	1931083	+	ydjY	hypothetical protein
i02_1972	CDS	1931097	1931804	+	ydjZ	hypothetical protein
i02_1973	CDS	1931804	1932352	+	ynjA	hypothetical protein
i02_1974	CDS	1932362	1933528	+	/	putative ABC transporter solute-binding protein
i02_1975	CDS	1933477	1935036	+	ynjC	ABC transporter permease
i02_1976	CDS	1934949	1935689	+	ynjD	ABC transporter ATP-binding protein
i02_1977	CDS	1935741	1937063	+	/	putative thiosulfate sulfurtransferase ynjE
i02_1978	CDS	1937072	1937698	-	ynjF	hypothetical protein
i02_1979	CDS	1937779	1938186	+	/	pyrimidine (deoxy)nucleoside triphosphate
i02_1980	CDS	1938152	1938424	-	ynjH	hypothetical protein
i02_1981	CDS	1938660	1940003	+	gdhA	glutamate dehydrogenase
i02_1982	CDS	1940059	1942002	-	/	hypothetical protein
i02_1983	CDS	1941999	1943699	-	ybeW	chaperone protein hscC
i02_1984	CDS	1943769	1944569	-	/	hypothetical protein
i02_1985	CDS	1944688	1946691	-	topB	DNA topoisomerase III
i02_1986	CDS	1946639	1947688	-	selD	selenophosphate synthetase
i02_1987	CDS	1947799	1948350	-	ydjA	hypothetical protein
i02_1988	CDS	1948404	1948568	+	/	hypothetical protein
i02_1989	CDS	1948511	1950367	+	sppA	protease 4
i02_1990	CDS	1950534	1951550	+	ansA	cytoplasmic asparaginase I
i02_1991	CDS	1951543	1952202	+	ydjB	nicotinamidase/pyrazinamidase
i02_1992	CDS	1952295	1953722	-	ydjE	metabolite transporter
i02_1993	CDS	1953771	1954529	-	ydjF	putative transcriptional regulator YdjF
i02_1994	CDS	1954666	1955646	-	ydjG	oxidoreductase ydjG
i02_1995	CDS	1955656	1956624	-	ydjH	putative sugar kinase ydjH
i02_1996	CDS	1956608	1957444	-	ydjI	hypothetical protein
i02_1997	CDS	1957465	1958508	-	ydjJ	hypothetical protein
i02_1998	CDS	1958525	1959904	-	ydjK	metabolite transporter
i02_1999	CDS	1959789	1959941	-	/	hypothetical protein
i02_2000	CDS	1959931	1961007	-	ydjL	hypothetical protein
i02_2001	CDS	1961377	1961664	-	yeaC	hypothetical protein
i02_2002	CDS	1961693	1962136	-	yeaA	methionine sulfoxide reductase B
i02_2003	CDS	1962439	1963443	+	gapA	glyceraldehyde-3-phosphate dehydrogenase
i02_2004	CDS	1963506	1964411	+	yeaD	hypothetical protein
i02_2005	CDS	1964462	1965361	-	yeaE	hypothetical protein
i02_2006	CDS	1965406	1966152	-	yeaF	MltA-interacting protein precursor
i02_2007	CDS	1966588	1968522	+	yeaG	hypothetical protein
i02_2008	CDS	1968635	1969918	+	yeaH	hypothetical protein
i02_2009	CDS	1970065	1971540	+	yeaI	hypothetical protein
i02_2010	CDS	1971541	1973211	+	yeaJ	hypothetical protein
i02_2011	CDS	1973254	1973757	+	yeaK	hypothetical protein
i02_2012	CDS	1973758	1973910	-	/	hypothetical protein
i02_2013	CDS	1974032	1974478	+	yeaL	hypothetical protein
i02_2014	CDS	1974435	1975256	-	yeaM	putative transcriptional regulator YeaM

Locus_tag	Type	start	End	+/-[a]	Gene[b]	Product
i02_2015	CDS	1975353	1976534	+	yeaN	putative transport protein YeaN
i02_2016	CDS	1976589	1976936	+	yeaO	hypothetical protein
i02_2017	CDS	1976958	1977212	-	yoaF	hypothetical protein
i02_2018	CDS	1977266	1978420	+	yeaP	hypothetical protein
i02_2019	CDS	1978438	1978551	+	/	hypothetical protein
i02_2020	CDS	1978688	1978936	-	yeaQ	hypothetical protein
i02_2021	CDS	1978948	1979070	-	/	hypothetical protein
i02_2022	CDS	1979084	1979266	-	yoaG	hypothetical protein
i02_2023	CDS	1979270	1979629	-	yeaR	hypothetical protein
i02_2024	CDS	1979600	1979734	+	/	hypothetical protein
i02_2025	CDS	1979802	1980440	-	yeaS	leucine export protein LeuE
i02_2026	CDS	1980567	1980899	-	/	hypothetical protein
i02_2028	CDS	1980983	1982110	-	rnd	ribonuclease D
i02_2029	CDS	1982180	1983931	-	fadD	long-chain-fatty-acid--CoA ligase
i02_2030	CDS	1984070	1984651	-	yeaY	lipoprotein yeaY precursor
i02_2031	CDS	1984691	1985386	-	yeaZ	hypothetical protein
i02_2032	CDS	1985444	1987354	-	/	ATP-dependent helicase yoaA
i02_2033	CDS	1987438	1987830	+	yoaB	hypothetical protein
i02_2034	CDS	1987998	1988111	-	/	hypothetical protein
i02_2035	CDS	1988192	1988551	+	yoaC	hypothetical protein
i02_2036	CDS	1988668	1988832	+	/	hypothetical protein
i02_2037	CDS	1988924	1990285	+	pabB	para-aminobenzoate synthase component I
i02_2038	CDS	1990289	1990867	+	yeaB	hypothetical protein
i02_2039	CDS	1991051	1992415	+	sdaA	L-serine dehydratase 1
i02_2040	CDS	1992516	1994144	+	yoaD	hypothetical protein
i02_2041	CDS	1994148	1995704	-	yoaE	hypothetical protein
i02_2042	CDS	1996167	1997138	+	manX	PTS system, mannose-specific IIAB component
i02_2043	CDS	1997201	1998001	+	manY	PTS system, mannose-specific IIC component
i02_2044	CDS	1998005	1998865	+	manZ	mannose-specific PTS system protein IID
i02_2045	CDS	1998920	1999378	+	yobD	hypothetical protein
i02_2046	CDS	1999753	2000373	+	yebN	hypothetical protein
i02_2047	CDS	2000370	2001179	-	rrmA	23S rRNA methyltransferase A
i02_2048	CDS	2001345	2001554	-	cspC	cold shock-like protein CspC
i02_2049	CDS	2001567	2001710	-	yobF	hypothetical protein
i02_2050	CDS	2001672	2001878	+	/	hypothetical protein
i02_2051	CDS	2002380	2002667	-	yebO	hypothetical protein
i02_2052	CDS	2002742	2002885	-	/	hypothetical protein
i02_2053	CDS	2003044	2003283	+	/	hypothetical protein
i02_2054	CDS	2003427	2004218	-	/	transcriptional regulator kdgR
i02_2055	CDS	2004284	2005768	+	yebQ	putative transport protein YebQ
i02_2056	CDS	2005814	2006695	-	htpX	heat shock protein HtpX
i02_2057	CDS	2006887	2008935	-	prc	carboxy-terminal protease
i02_2058	CDS	2008955	2009653	-	proQ	putative solute/DNA competence effector
i02_2059	CDS	2009750	2010196	-	/	hypothetical protein
i02_2060	CDS	2010377	2011660	+	yebS	hypothetical protein
i02_2061	CDS	2011587	2014262	+	yebT	hypothetical protein
i02_2062	CDS	2014336	2015781	+	yebU	rRNA (cytosine-C(5)-)-methyltransferase RsmF
i02_2063	CDS	2015884	2016135	+	yebV	hypothetical protein
i02_2064	CDS	2016156	2016431	+	yebW	hypothetical protein
i02_2065	CDS	2016432	2017091	-	pphA	serine/threonine protein phosphatase 1
i02_2066	CDS	2017313	2017465	-	/	hypothetical protein
i02_2067	CDS	2017483	2017824	-	yebY	hypothetical protein
i02_2068	CDS	2017837	2018709	-	yebZ	hypothetical protein
i02_2069	CDS	2018713	2019087	-	/	hypothetical protein
i02_2070	CDS	2019226	2019456	+	holE	DNA polymerase III subunit theta
i02_2071	CDS	2019559	2020215	+	yobB	hypothetical protein
i02_2072	CDS	2020239	2020901	+	/	exodeoxyribonuclease X
i02_2073	pseudo	2020898	2022958	-	ptrB	protease II

Locus_tag	Type	start	End	+/-[a]	Gene[b]	Product
i02_2074	CDS	2023044	2023646	+	/	hypothetical protein
i02_2075	CDS	2023167	2023826	-	yebE	hypothetical protein
i02_2076	CDS	2024153	2024521	-	yebF	hypothetical protein
i02_2077	CDS	2024576	2024866	-	yebG	DNA damage-inducible protein YebG
i02_2078	CDS	2025000	2026178	+	purK	phosphoribosylaminoimidazole carboxylase ATPase
i02_2079	CDS	2026234	2026875	-	eda	keto-hydroxyglutarate-aldolase/ 2-keto-4-hydroxyglutarate aldolase decarboxylase
i02_2080	CDS	2026912	2028789	-	edd	phosphogluconate dehydratase
i02_2081	CDS	2028958	2030433	-	zwf	glucose-6-phosphate 1-dehydrogenase
i02_2082	CDS	2030599	2030748	-	/	hypothetical protein
i02_2083	CDS	2030771	2031640	+	yebK	DNA-binding transcriptional regulator HexR
i02_2084	CDS	2031627	2033210	+	pykA	pyruvate kinase
i02_2085	CDS	2033342	2034313	-	msbB	lipid A biosynthesis (KDO)2-(lauroyl)-lipid IVA
i02_2086	CDS	2034432	2035754	-	yebA	hypothetical protein
i02_2087	CDS	2035770	2036756	-	znuA	high-affinity zinc transporter periplasmic
i02_2088	CDS	2036781	2037536	+	znuC	high-affinity zinc transporter ATPase
i02_2089	CDS	2037533	2038318	+	znuB	high-affinity zinc transporter membrane
i02_2090	CDS	2038465	2039475	-	ruvB	Holliday junction DNA helicase RuvB
i02_2091	CDS	2039484	2040095	-	ruvA	Holliday junction DNA helicase RuvA
i02_2092	CDS	2040371	2040973	+	yebB	hypothetical protein
i02_2093	CDS	2040975	2041499	-	ruvC	Holliday junction resolvase
i02_2094	CDS	2041531	2042271	-	yebC	hypothetical protein
i02_2095	CDS	2042300	2042743	-	ntpA	dATP pyrophosphohydrolase
i02_2096	CDS	2042745	2044517	-	aspS	aspartyl-tRNA synthetase
i02_2097	CDS	2044827	2045393	+	yecD	hypothetical protein
i02_2098	CDS	2045390	2046208	+	yecE	hypothetical protein
i02_2099	CDS	2046231	2046656	+	yecN	hypothetical protein
i02_2100	CDS	2046697	2047440	+	yecO	hypothetical protein
i02_2101	CDS	2047437	2048408	+	yecP	hypothetical protein
i02_2102	CDS	2048573	2051020	-	bisZ	trimethylamine-N-oxide reductase 2 precursor
i02_2104	CDS	2051027	2052127	-	yecK	cytochrome c-type protein torY
i02_2105	CDS	2052033	2052164	-	/	hypothetical protein
i02_2106	CDS	2052515	2053261	-	/	copper homeostasis protein CutC
i02_2107	CDS	2053275	2053847	-	yecM	hypothetical protein
i02_2108	CDS	2054057	2055790	+	argS	arginyl-tRNA synthetase
i02_2109	CDS	2055843	2056235	-	flhE	flagellar protein flhE precursor
i02_2110	CDS	2056235	2058313	-	flhA	flagellar biosynthesis protein FlhA
i02_2111	CDS	2058306	2059454	-	flhB	flagellar biosynthesis protein FlhB
i02_2112	CDS	2059487	2059633	+	/	hypothetical protein
i02_2113	CDS	2059663	2060307	-	cheZ	chemotaxis regulator CheZ
i02_2114	CDS	2060318	2060707	-	cheY	chemotaxis regulatory protein CheY
i02_2115	CDS	2060722	2061771	-	cheB	chemotaxis-specific methylesterase
i02_2116	CDS	2061774	2062634	-	cheR	chemotaxis methyltransferase CheR
i02_2117	CDS	2062653	2062772	-	/	methyl-accepting protein IV
i02_2118	CDS	2062760	2062882	-	/	hypothetical protein
i02_2119	CDS	2062925	2064607	-	tar	methyl-accepting chemotaxis protein II
i02_2120	CDS	2064731	2065234	-	cheW	purine-binding chemotaxis protein
i02_2121	CDS	2065255	2067273	-	cheA	chemotaxis protein CheA
i02_2122	CDS	2067224	2068150	-	motB	flagellar motor protein MotB
i02_2123	CDS	2068147	2069034	-	motA	flagellar motor protein MotA
i02_2124	CDS	2069160	2069738	-	flhC	transcriptional activator FlhC
i02_2125	CDS	2069741	2070100	-	flhD	transcriptional activator FlhD
i02_2126	CDS	2070871	2071305	+	yecG	universal stress protein UspC
i02_2127	CDS	2071312	2072745	-	otsA	trehalose-6-phosphate synthase
i02_2128	CDS	2072711	2073568	-	otsB	trehalose-6-phosphate phosphatase
i02_2129	CDS	2073678	2074667	-	araH	L-arabinose transporter permease protein
i02_2130	CDS	2074679	2076238	-	araG	L-arabinose transporter ATP-binding protein

Locus_tag	Type	start	End	+/-[a]	Gene[b]	Product
i02_2131	CDS	2076263	2077303	-	araF	L-arabinose-binding periplasmic protein
i02_2132	CDS	2078049	2078552	+	yecI	Ferritin-like protein 2
i02_2133	CDS	2078631	2078882	-	/	hypothetical protein
i02_2134	CDS	2078994	2079140	+	/	hypothetical protein
i02_2135	CDS	2079388	2079669	+	yecR	hypothetical protein
i02_2136	CDS	2079679	2079816	-	/	hypothetical protein
i02_2137	CDS	2079826	2080338	+	ftn	ferritin
i02_2138	CDS	2080376	2080615	-	yecH	hypothetical protein
i02_2139	CDS	2080806	2082017	+	tyrP	tyrosine transporter
i02_2140	CDS	2082079	2082744	-	yecA	hypothetical protein
i02_2141	CDS	2083393	2083941	-	pgsA	phosphatidylglycerophosphate synthetase
i02_2142	CDS	2083998	2085830	-	uvrC	excinuclease ABC subunit C
i02_2143	CDS	2085827	2086483	-	uvrY	response regulator
i02_2144	CDS	2086722	2086850	-	/	hypothetical protein
i02_2145	CDS	2086942	2087166	+	yecF	hypothetical protein
i02_2146	CDS	2087234	2087956	-	sdiA	DNA-binding transcriptional activator SdiA
i02_2147	CDS	2088186	2088938	-	yecC	putative amino-acid ABC transporter ATP-binding component
i02_2148	CDS	2088935	2089603	-	yecS	amino-acid ABC transporter permease protein
i02_2149	CDS	2089618	2090700	-	yedO	D-cysteine desulfhydrase
i02_2150	CDS	2090709	2091566	-	fliY	cystine transporter subunit
i02_2151	CDS	2091597	2092184	-	fliZ	flagella biosynthesis protein FliZ
i02_2152	CDS	2092194	2092913	-	fliA	flagellar biosynthesis sigma factor
i02_2153	CDS	2092987	2093106	+	/	hypothetical protein
i02_2154	CDS	2093233	2095020	-	fliC	flagellin
i02_2155	CDS	2095286	2096692	+	fliD	flagellar capping protein
i02_2156	CDS	2096717	2097127	+	fliS	flagellar protein FliS
i02_2157	CDS	2097127	2097492	+	fliT	flagellar biosynthesis protein FliT
i02_2158	CDS	2097570	2099057	+	amyA	cytoplasmic alpha-amylase
i02_2159	CDS	2099091	2099507	-	yedD	hypothetical protein
i02_2160	CDS	2099658	2100896	+	yedE	putative inner membrane protein
i02_2161	CDS	2100893	2101126	+	yedF	hypothetical protein
i02_2162	CDS	2101142	2101903	+	yedK	hypothetical protein
i02_2163	CDS	2102416	2103498	+	/	Outer membrane porin protein nmpC precursor
i02_2164	CDS	2104000	2104797	-	ybcM	putative transcriptional regulator YbcM
i02_2165	CDS	2104807	2105358	-	ybcL	putative kinase inhibitor
i02_2167	CDS	2105527	2106024	-	emrE	multidrug efflux protein
i02_2168	CDS	2106204	2106518	-	fliE	flagellar hook-basal body protein FliE
i02_2169	CDS	2106733	2108391	+	fliF	flagellar MS-ring protein
i02_2170	CDS	2108384	2109379	+	fliG	flagellar motor switch protein G
i02_2171	CDS	2108537	2109466	-	/	hypothetical protein
i02_2172	CDS	2109372	2110058	+	fliH	flagellar assembly protein H
i02_2173	CDS	2110094	2111431	+	fliI	flagellum-specific ATP synthase
i02_2174	CDS	2111450	2111893	+	fliJ	flagellar biosynthesis chaperone
i02_2175	CDS	2111890	2113017	+	fliK	flagellar hook-length control protein
i02_2176	CDS	2113122	2113586	+	fliL	flagellar basal body-associated protein FliL
i02_2177	CDS	2113546	2114595	+	fliM	flagellar motor switch protein FliM
i02_2178	CDS	2114592	2115005	+	fliN	flagellar motor switch protein FliN
i02_2179	CDS	2115005	2115373	+	fliO	flagellar biosynthesis protein FliO
i02_2180	CDS	2115373	2116110	+	fliP	flagellar biosynthesis protein FliP
i02_2181	CDS	2116120	2116389	+	fliQ	flagellar biosynthesis protein FliQ
i02_2182	CDS	2116398	2117183	+	fliR	flagellar biosynthesis protein FliR
i02_2183	CDS	2117473	2118096	+	rcsA	colanic acid capsular biosynthesis activation protein
i02_2184	CDS	2118173	2118448	+	/	hypothetical protein
i02_2185	CDS	2118476	2118718	+	yodD	hypothetical protein
i02_2186	CDS	2119016	2119831	+	yedP	mannosyl-3-phosphoglycerate phosphatase
i02_2187	CDS	2119828	2121537	-	yedQ	hypothetical protein

Locus_tag	Type	start	End	+/-[a]	Gene[b]	Product
i02_2188	CDS	2121443	2121631	-	/	hypothetical protein
i02_2189	CDS	2121693	2121875	-	yodC	hypothetical protein
i02_2190	CDS	2121954	2122871	-	yedI	hypothetical protein
i02_2191	CDS	2123044	2123964	+	yedA	hypothetical protein
i02_2192	CDS	2123953	2124423	-	vsr	very short patch repair protein
i02_2193	CDS	2124404	2125822	-	dcm	DNA cytosine methylase
i02_2194	CDS	2125934	2126611	-	yedJ	hypothetical protein
i02_2195	CDS	2126624	2127007	-	yedR	hypothetical protein
i02_2196	CDS	2127486	2128613	+	/	Outer membrane protein N precursor
i02_2197	CDS	2129045	2129188	+	/	conserved hypothetical protein
i02_2198	CDS	2129209	2130060	+	yedU	chaperone protein HchA
i02_2199	CDS	2130168	2131526	-	yedV	putative sensor-like histidine kinase yedV
i02_2200	CDS	2131526	2132308	-	yedW	transcriptional regulatory protein YedW
i02_2201	CDS	2132330	2132743	+	/	transthyretin-like protein precursor
i02_2202	CDS	2132852	2133856	+	yedY	putative sulfite oxidase subunit YedY
i02_2203	CDS	2133857	2134492	+	yedZ	putative sulfite oxidase subunit YedZ
i02_2204	CDS	2134750	2135400	+	yodA	hypothetical protein
i02_2205	CDS	2135436	2135765	+	/	hypothetical protein
i02_2206	CDS	2136248	2137468	+	/	P4 family integrase
i02_2207	CDS	2137582	2139402	+	/	hypothetical protein
i02_2208	CDS	2139517	2140980	-	/	PilV-like protein
i02_2209	CDS	2141033	2141590	-	/	putative type IV pilin protein precursor
i02_2210	CDS	2141989	2142303	-	/	hypothetical protein
i02_2211	CDS	2142940	2144442	+	/	hypothetical protein
i02_2212	CDS	2144499	2145422	+	/	hypothetical protein
i02_2213	CDS	2145554	2146063	-	/	hypothetical protein
i02_2214	CDS	2146200	2147036	+	/	hypothetical protein
i02_2215	CDS	2147382	2147957	-	/	hypothetical protein
i02_2216	CDS	2147998	2148135	-	/	hypothetical protein
i02_2217	CDS	2148329	2148817	+	/	hypothetical protein
i02_2218	CDS	2148807	2149019	+	/	hypothetical protein
i02_2219	CDS	2149048	2149215	+	/	hypothetical protein
i02_2220	CDS	2149307	2150488	-	/	hypothetical protein
i02_2221	CDS	2150540	2151502	-	/	hypothetical protein
i02_2222	CDS	2151850	2151972	+	/	conserved hypothetical protein
i02_2223	CDS	2152855	2153436	+	/	hypothetical protein
i02_2224	CDS	2153521	2154252	-	/	hypothetical protein
i02_2225	CDS	2154251	2154367	+	/	hypothetical protein
i02_2226	CDS	2154426	2154905	+	/	hypothetical protein
i02_2227	CDS	2155064	2155468	+	/	DNA-binding protein H-NS
i02_2228	CDS	2155532	2155912	-	/	hypothetical protein
i02_2229	CDS	2155991	2156233	-	/	hypothetical protein
i02_2230	CDS	2156306	2156602	-	/	hypothetical protein
i02_2231	CDS	2156655	2156957	-	/	hypothetical protein
i02_2232	CDS	2157027	2157245	-	/	putative regulatory protein
i02_2233	CDS	2157462	2158355	-	/	hypothetical protein
i02_2234	CDS	2158721	2159557	+	yeeI	hypothetical protein
i02_2235	CDS	2160114	2161157	+	/	prophage P4 integrase
i02_2236	CDS	2161351	2162655	-	/	salicylate synthase Irp9
i02_2237	CDS	2162683	2164086	-	/	putative cytoplasmic transmembrane protein
i02_2238	CDS	2163956	2165785	-	/	ABC transporter
i02_2239	CDS	2165745	2167547	-	/	putative inner membrane ABC-transporter
i02_2240	CDS	2167714	2168673	+	/	putative AraC type regulator
i02_2241	CDS	2168846	2174980	+	/	phenyloxazoline synthase MbtB
i02_2242	CDS	2175068	2184559	+	/	yersiniabactin biosynthetic protein
i02_2244	CDS	2184556	2185656	+	/	hypothetical protein
i02_2245	CDS	2185653	2186456	+	/	putative thioesterase
i02_2246	CDS	2186460	2188037	+	/	2,3-dihydroxybenzoate-AMP ligase

Locus_tag	Type	start	End	+/-[a]	Gene[b]	Product
i02_2247	CDS	2188168	2190189	+	/	putative pesticin receptor precursor
i02_2248	CDS	2190779	2191585	+	/	hypothetical protein
i02_2249	CDS	2191512	2191682	+	/	hypothetical protein
i02_2250	CDS	2191718	2193187	+	/	hypothetical protein
i02_2250a	CDS	2193200	2193676	+	/	hypothetical protein
i02_2251	CDS	2193804	2194856	-	/	hypothetical protein
i02_2252	CDS	2195171	2196487	+	shiA	shikimate transporter
i02_2253	CDS	2196541	2198043	+	amn	AMP nucleosidase
i02_2254	CDS	2198386	2199102	+	yeeN	hypothetical protein
i02_2255	CDS	2199158	2199445	-	/	hypothetical protein
i02_2256	CDS	2199958	2200875	+	nac	nitrogen assimilation transcriptional regulator
i02_2257	CDS	2200977	2201927	+	cbl	transcriptional regulator Cbl
i02_2258	CDS	2202048	2203688	+	yeeO	hypothetical protein
i02_2259	CDS	2204026	2205297	+	/	prophage P4 integrase
i02_2260	CDS	2205577	2206089	-	/	hypothetical protein
i02_2261	CDS	2206124	2206846	-	/	putative thioesterase
i02_2262	CDS	2206839	2208353	-	/	hypothetical protein
i02_2263	CDS	2208366	2210825	-	/	putative polyketide synthase
i02_2264	CDS	2210856	2215310	-	/	putative peptide synthetase
i02_2266	CDS	2215220	2216659	-	/	hypothetical protein
i02_2267	CDS	2216721	2218208	-	/	putative amidase
i02_2268	CDS	2218177	2224641	-	/	putative peptide synthetase
i02_2269	CDS	2224652	2231152	-	/	putative peptide synthetase
i02_2270	CDS	2231196	2234228	-	/	putative polyketide synthase
i02_2271	CDS	2234278	2239089	-	/	hypothetical protein
i02_2273	CDS	2239122	2240390	-	/	putative transacylase
i02_2274	CDS	2240387	2241415	-	/	putative acyl-coa dehydrogenase
i02_2275	CDS	2241521	2241769	-	/	hypothetical protein
i02_2276	CDS	2241799	2242668	-	/	putative 3-hydroxyacyl-CoA dehydrogenase
i02_2277	CDS	2242678	2245287	-	/	putative polyketide synthase
i02_2279	CDS	2245319	2254948	-	/	putative peptide/polyketide synthase
i02_2280	CDS	2255578	2256312	+	/	hypothetical protein
i02_2281	CDS	2256458	2256724	+	/	transposase
i02_2282	CDS	2256721	2256834	-	/	hypothetical protein
i02_2283	CDS	2256829	2257308	+	/	transposase
i02_2284	CDS	2257299	2257526	+	/	transposase
i02_2285	CDS	2257616	2258041	-	/	hypothetical protein
i02_2286	CDS	2258333	2259268	-	erfK	hypothetical protein
i02_2287	CDS	2259330	2260409	-	cobT	nicotinate-nucleotide--dimethylbenzimidazole
i02_2288	CDS	2260421	2261200	-	cobS	cobalamin synthase
i02_2289	CDS	2261161	2261706	-	cobU	adenosylcobinamide
i02_2290	CDS	2261858	2261980	-	/	hypothetical protein
i02_2291	CDS	2262171	2262335	-	/	hypothetical protein
i02_2292	CDS	2263378	2265525	+	/	putative outer membrane receptor for iron
i02_2293	CDS	2265513	2265641	+	/	hypothetical protein
i02_2294	CDS	2265896	2266540	-	/	hypothetical protein
i02_2295	CDS	2266525	2267814	-	/	hypothetical protein
i02_2296	CDS	2268739	2269449	+	/	hypothetical protein
i02_2297	CDS	2270694	2271218	+	/	putative transferase
i02_2298	CDS	2271172	2271993	+	/	hypothetical protein
i02_2299	CDS	2272562	2272876	-	/	hypothetical protein
i02_2300	CDS	2272995	2274209	+	/	putative carbohydrate kinase
i02_2301	CDS	2274223	2274981	+	/	hypothetical protein
i02_2302	CDS	2275035	2276000	+	/	hypothetical protein
i02_2303	CDS	2276003	2277037	+	/	putative phosphotriesterase-related protein
i02_2304	pseudo	2277689	2279068	-	/	transposase
i02_2305	CDS	2279352	2279849	-	/	hypothetical protein
i02_2306	CDS	2279750	2280154	-	/	hypothetical protein

Locus_tag	Type	start	End	+/-[a]	Gene[b]	Product
i02_2307	CDS	2280317	2280922	-	/	hypothetical protein
i02_2308	CDS	2281452	2281628	-	/	hypothetical protein
i02_2309	CDS	2281579	2281959	+	/	hypothetical protein
i02_2310	CDS	2282392	2283588	+	/	transposase
i02_2312	CDS	2283616	2283990	+	/	hypothetical protein
i02_2313	CDS	2284069	2284686	+	/	hypothetical protein
i02_2314	CDS	2285712	2286494	-	/	transposase/IS protein
i02_2315	CDS	2286491	2287513	-	/	transposase
i02_2316	CDS	2287608	2287838	+	/	putative transposase subunit
i02_2317	CDS	2288064	2288441	-	/	hypothetical protein
i02_2318	CDS	2289010	2289285	+	/	hypothetical protein
i02_2319	CDS	2291023	2291790	-	/	putative ABC transporter
i02_2320	CDS	2291787	2292845	-	/	ABC transporter
i02_2321	CDS	2292864	2293856	-	/	periplasmic binding protein
i02_2322	CDS	2293864	2296029	-	/	TonB dependent receptor
i02_2323	CDS	2297109	2299493	+	/	hypothetical protein
i02_2325	CDS	2299490	2300395	+	/	hypothetical protein
i02_2326	CDS	2300392	2301462	+	/	hypothetical protein
i02_2327	CDS	2301490	2302275	+	/	hypothetical protein
i02_2328	CDS	2302291	2302701	+	/	hypothetical protein
i02_2329	CDS	2302922	2303743	+	/	hypothetical protein
i02_2330	CDS	2303888	2304427	-	/	hypothetical protein
i02_2331	CDS	2304880	2305101	+	/	hypothetical protein
i02_2332	CDS	2305175	2305543	+	/	hypothetical protein
i02_2333	CDS	2305632	2306006	+	/	hypothetical protein
i02_2334	CDS	2306003	2306197	+	yeeW	hypothetical protein
i02_2335	CDS	2306638	2306994	+	yoeF	hypothetical protein
i02_2336	CDS	2307095	2307490	-	yeeX	hypothetical protein
i02_2337	CDS	2307596	2308654	-	yeeA	hypothetical protein
i02_2338	CDS	2308852	2309325	-	sbmC	DNA gyrase inhibitor
i02_2339	CDS	2309444	2310616	-	dacD	D-alanyl-D-alanine carboxypeptidase
i02_2340	CDS	2310771	2312246	+	sbcB	exonuclease I
i02_2341	CDS	2312320	2313684	-	yeeF	putative transport protein YeeF
i02_2342	CDS	2313945	2314895	-	yeeY	putative transcriptional regulator YeeY
i02_2343	CDS	2314920	2315744	-	/	hypothetical protein
i02_2344	CDS	2315827	2316081	-	/	hypothetical protein
i02_2345	CDS	2316078	2316356	-	yefM	antitoxin YefM
i02_2346	CDS	2316808	2317707	+	hisG	ATP phosphoribosyltransferase
i02_2347	CDS	2317704	2319017	+	hisD	histidinol dehydrogenase
i02_2348	CDS	2319014	2320084	+	hisC	histidinol-phosphate aminotransferase
i02_2349	CDS	2320084	2321151	+	hisB	imidazole glycerol-phosphate
i02_2350	CDS	2321151	2321741	+	hisH	imidazole glycerol phosphate synthase subunit
i02_2351	CDS	2321738	2322478	+	hisA	N-(5'-phospho-L-ribosyl-formimino)-5-amino-1-(5'-phosphoribosyl)-4-imidazolecarboxamide isomerase (HisA)
i02_2352	CDS	2322460	2323236	+	hisF	imidazole glycerol phosphate synthase subunit
i02_2353	CDS	2323230	2323841	+	hisI	phosphoribosyl-AMP cyclohydrolase / phosphoribosyl-ATP pyrophosphatase
i02_2354	CDS	2324027	2325040	-	wzzB	chain length determinant protein
i02_2355	CDS	2325147	2326313	-	ugd	UDP-glucose 6-dehydrogenase
i02_2356	CDS	2326563	2327969	-	gnd	6-phosphogluconate dehydrogenase
i02_2357	CDS	2328132	2329502	-	manB	phosphomannomutase
i02_2358	CDS	2329585	2331036	-	/	mannose-1-phosphate guanylyltransferase
i02_2359	CDS	2331014	2332129	-	/	hypothetical protein
i02_2360	CDS	2332240	2333247	-	/	UDP-glucose 4-epimerase
i02_2361	CDS	2333264	2334409	-	/	hypothetical protein
i02_2362	CDS	2334396	2335478	-	/	glycosyl transferase
i02_2363	CDS	2335424	2336293	-	/	glycosyl transferase

Locus_tag	Type	start	End	+/-[a]	Gene[b]	Product
i02_2364	CDS	2336290	2337651	-	/	hypothetical protein
i02_2365	CDS	2337633	2338889	-	/	hypothetical protein
i02_2366	CDS	2339246	2340151	-	galF	UTP--glucose-1-phosphate uridylyltransferase
i02_2367	CDS	2340314	2341708	-	wcaM	putative colanic acid biosynthesis protein
i02_2368	CDS	2341719	2342939	-	wcaL	putative colanic acid biosynthesis glycosyltransferase
i02_2369	CDS	2342936	2344216	-	wcaK	putative pyruvyl transferase
i02_2370	CDS	2344878	2346356	-	wzxC	colanic acid exporter
i02_2371	CDS	2346358	2347752	-	wcaJ	putative UDP-glucose lipid carrier transferase
i02_2372	CDS	2347808	2349226	-	cpsG	phosphomannomutase
i02_2373	CDS	2349204	2349362	+	/	hypothetical protein
i02_2374	CDS	2349371	2350807	-	cpsB	mannose-1-phosphate guanylyltransferase
i02_2375	CDS	2350810	2352033	-	wcaI	putative glycosyl transferase
i02_2376	CDS	2352030	2352512	-	wcaH	GDP-mannose mannosyl hydrolase
i02_2377	CDS	2352512	2353477	-	wcaG	GDP-4-keto-6-L-galactose reductase
i02_2378	CDS	2353480	2354601	-	gmd	GDP-mannose 4,6-dehydratase
i02_2379	CDS	2354628	2355176	-	wcaF	putative colanic acid biosynthesis
i02_2380	CDS	2355192	2355938	-	wcaE	putative glycosyl transferase
i02_2381	CDS	2355949	2357166	-	wcaD	putative colanic acid biosynthesis protein
i02_2382	CDS	2357141	2358358	-	wcaC	putative glycosyl transferase
i02_2383	CDS	2358355	2358843	-	wcaB	putative colanic acid biosynthesis
i02_2384	CDS	2358846	2359685	-	wcaA	putative glycosyl transferase
i02_2385	CDS	2359778	2361976	-	/	tyrosine kinase
i02_2386	CDS	2361943	2362386	-	/	hypothetical protein
i02_2387	CDS	2362392	2363510	-	/	hypothetical protein
i02_2388	CDS	2364124	2365773	+	yegH	hypothetical protein
i02_2389	CDS	2365841	2367694	-	asmA	putative assembly protein
i02_2390	CDS	2367716	2368297	-	dcd	deoxycytidine triphosphate deaminase
i02_2391	CDS	2368389	2369030	-	udk	uridine kinase
i02_2392	CDS	2369348	2372665	+	yegE	putative sensor protein
i02_2393	CDS	2372775	2373623	-	alkA	3-methyl-adenine DNA glycosylase II
i02_2394	CDS	2373757	2375109	+	yegD	putative chaperone
i02_2396	CDS	2375122	2377071	-	yegI	hypothetical protein
i02_2397	CDS	2377059	2377820	-	yegK	hypothetical protein
i02_2398	CDS	2377817	2378476	-	yegL	hypothetical protein
i02_2399	CDS	2379210	2380604	+	yegM	multidrug efflux system subunit MdtA
i02_2400	CDS	2380604	2383726	+	yegN	multidrug efflux system subunit MdtB
i02_2401	CDS	2383727	2386804	+	yegO	multidrug efflux system subunit MdtC
i02_2402	CDS	2386805	2388220	+	yegB	multidrug efflux system protein MdtE
i02_2403	CDS	2388217	2389620	+	baeS	signal transduction histidine-protein kinase
i02_2404	CDS	2389617	2390339	+	baeR	DNA-binding transcriptional regulator BaeR
i02_2405	CDS	2390480	2390851	+	yegP	hypothetical protein
i02_2406	CDS	2390976	2391590	-	/	hypothetical protein
i02_2407	CDS	2391642	2393789	-	/	hypothetical protein
i02_2409	CDS	2393761	2394477	-	/	hypothetical protein
i02_2410	CDS	2394477	2395691	-	/	hypothetical protein
i02_2411	CDS	2396179	2397540	+	yegQ	putative protease yegQ
i02_2412	CDS	2397480	2397617	-	/	hypothetical protein
i02_2413	CDS	2397895	2398020	-	/	hypothetical protein
i02_2414	CDS	2398040	2398372	-	yegR	hypothetical protein
i02_2415	CDS	2398772	2399671	+	yegS	lipid kinase
i02_2416	CDS	2399753	2400532	-	/	hypothetical protein
i02_2417	CDS	2400632	2401672	-	gatD	galactitol-1-phosphate dehydrogenase
i02_2418	CDS	2401720	2403075	-	gatC	PTS system, galactitol-specific IIC component
i02_2419	CDS	2403079	2403363	-	gatB	galactitol-specific PTS system component IIB
i02_2420	CDS	2403394	2403846	-	gatA	galactitol-specific PTS system component IIA
i02_2421	CDS	2403856	2405118	-	gatZ	putative tagatose 6-phosphate kinase gatZ
i02_2422	CDS	2405147	2406001	-	gatY	tagatose-bisphosphate aldolase

Locus_tag	Type	start	End	+/-[a]	Gene[b]	Product
i02_2423	CDS	2406228	2407352	-	/	fructose-bisphosphate aldolase
i02_2424	CDS	2407339	2407485	+	/	hypothetical protein
i02_2425	CDS	2407537	2408814	+	yegT	putative nucleoside transporter yegT
i02_2426	CDS	2408811	2409815	+	yegU	hypothetical protein
i02_2427	CDS	2409812	2410777	+	yegV	putative sugar kinase yegV
i02_2428	CDS	2410751	2411497	-	yegW	putative transcriptional regulator YegW
i02_2429	CDS	2411549	2412376	-	yegX	hypothetical protein
i02_2430	CDS	2412432	2413232	-	thiD	phosphomethylpyrimidine kinase
i02_2431	CDS	2413229	2414017	-	thiM	hydroxyethylthiazole kinase
i02_2432	CDS	2414240	2414512	-	yohL	hypothetical protein
i02_2433	CDS	2414633	2415457	+	yohM	nickel/cobalt efflux protein RcnA
i02_2434	CDS	2415496	2416014	+	yohN	hypothetical protein
i02_2435	CDS	2416096	2417130	-	yehA	hypothetical protein
i02_2436	CDS	2417146	2419641	-	yehB	outer membrane usher protein yehB precursor
i02_2437	CDS	2419642	2420316	-	yehC	fimbrial chaperone yehC precursor
i02_2438	CDS	2420397	2420939	-	yehD	hypothetical protein
i02_2439	CDS	2421233	2421514	-	yehE	hypothetical protein
i02_2440	CDS	2421777	2422916	-	mrp	putative ATPase
i02_2441	CDS	2423009	2425051	+	metG	methionyl-tRNA synthetase
i02_2442	CDS	2425192	2428980	+	/	hypothetical protein
i02_2443	CDS	2428990	2432622	+	yehI	hypothetical protein
i02_2444	CDS	2430062	2430313	-	/	hypothetical protein
i02_2445	CDS	2431474	2431632	-	/	hypothetical protein
i02_2446	CDS	2432683	2432868	+	/	yehK protein
i02_2447	CDS	2433565	2434719	+	yehL	hypothetical protein
i02_2448	CDS	2434655	2437009	+	yehM	hypothetical protein
i02_2449	CDS	2436795	2438138	+	yehP	hypothetical protein
i02_2450	CDS	2438135	2440138	+	yehQ	hypothetical protein
i02_2451	CDS	2440251	2440724	+	yehR	lipoprotein yehR precursor
i02_2452	CDS	2440765	2441292	-	yehS	hypothetical protein
i02_2453	CDS	2441282	2442016	-	yehT	putative two-component response-regulator
i02_2454	CDS	2441998	2443698	-	yehU	hypothetical protein
i02_2455	CDS	2443905	2444636	+	yehV	MerR-like regulator A
i02_2456	CDS	2444784	2445515	-	yehW	ABC transporter permease
i02_2457	CDS	2445520	2446446	-	yehX	ABC transporter ATP-binding protein
i02_2458	CDS	2446439	2447596	-	yehY	ABC transporter permease
i02_2459	CDS	2447603	2448520	-	yehZ	hypothetical protein
i02_2462	CDS	2448551	2448703	-	/	hypothetical protein
i02_2463	CDS	2448710	2451079	-	bglX	periplasmic beta-glucosidase precursor
i02_2464	CDS	2451203	2452918	+	dld	D-lactate dehydrogenase
i02_2465	CDS	2452957	2453898	-	pbpG	D-alanyl-D-alanine endopeptidase
i02_2466	CDS	2453968	2454090	-	/	hypothetical protein
i02_2467	CDS	2454063	2454674	-	yohC	hypothetical protein
i02_2468	CDS	2454784	2455398	+	yohD	hypothetical protein
i02_2469	CDS	2455518	2455904	-	/	hypothetical protein
i02_2469a	CDS	2455971	2456291	-	/	hypothetical protein
i02_2470	CDS	2456332	2457849	-	yohG	multidrug resistance outer membrane protein
i02_2471	CDS	2458462	2459478	-	yohI	tRNA-dihydrouridine synthase C
i02_2472	CDS	2459615	2460049	+	yohJ	hypothetical protein
i02_2473	CDS	2460046	2460741	+	yohK	hypothetical protein
i02_2474	CDS	2460871	2461755	+	cdd	cytidine deaminase
i02_2476	CDS	2461905	2462624	+	sanA	hypothetical protein
i02_2477	CDS	2462627	2462866	+	yeiS	hypothetical protein
i02_2478	CDS	2463060	2464298	+	yeiT	putative oxidoreductase
i02_2479	CDS	2464292	2465527	+	yeiA	dihydropyrimidine dehydrogenase
i02_2480	CDS	2465549	2465692	+	/	hypothetical protein
i02_2481	CDS	2465770	2466780	-	mglC	beta-methylgalactoside transporter
i02_2482	CDS	2466796	2468316	-	mglA	galactose/methyl galaxtoside transporter

Locus_tag	Type	start	End	+/-[a]	Gene[b]	Product
i02_2483	CDS	2468377	2469375	-	mglB	D-galactose-binding periplasmic protein
i02_2484	CDS	2469655	2470701	-	galS	DNA-binding transcriptional regulator GalS
i02_2485	CDS	2470685	2470888	+	/	hypothetical protein
i02_2486	CDS	2470837	2471994	-	yeiB	hypothetical protein
i02_2487	CDS	2472011	2472679	-	folE	GTP cyclohydrolase I
i02_2488	CDS	2472937	2473773	+	yeiG	hypothetical protein
i02_2489	CDS	2473805	2475796	-	cirA	colicin I receptor
i02_2490	CDS	2476088	2477557	-	lysP	lysine transporter
i02_2491	CDS	2477762	2478643	-	yeiE	putative DNA-binding transcriptional regulator
i02_2492	CDS	2478742	2479791	+	yeiH	hypothetical protein
i02_2493	CDS	2479865	2480722	+	nfo	endonuclease IV
i02_2494	CDS	2480726	2481814	+	yeiI	hypothetical protein
i02_2495	CDS	2481996	2482112	-	/	hypothetical protein
i02_2497	CDS	2482082	2483023	-	rihB	ribonucleoside hydrolase 2
i02_2498	CDS	2483153	2483851	+	yeiL	DNA-binding transcriptional activator YeiL
i02_2499	CDS	2483918	2485168	-	yeiM	putative transport protein YeiM
i02_2500	CDS	2485262	2486200	-	yeiN	hypothetical protein
i02_2501	CDS	2486188	2487243	-	yeiC	hypothetical protein
i02_2502	CDS	2487585	2489276	-	fruA	fructose-specific PTS system IIBC component
i02_2503	CDS	2489293	2490231	-	fruK	1-phosphofructokinase
i02_2504	CDS	2490231	2491361	-	fruB	fructose PTS transporter
i02_2505	CDS	2491526	2491657	+	/	hypothetical protein
i02_2506	CDS	2491728	2492909	+	yeiO	sugar efflux transporter B
i02_2507	CDS	2492906	2493094	-	/	hypothetical protein
i02_2508	CDS	2493060	2493887	+	yeiP	elongation factor P
i02_2509	CDS	2494101	2495576	+	yeiQ	oxidoreductase yeiQ
i02_2510	CDS	2495694	2496680	+	yeiR	hypothetical protein
i02_2511	CDS	2496683	2497432	+	yeiU	hypothetical protein
i02_2512	CDS	2497844	2498410	+	spr	putative outer membrane lipoprotein
i02_2513	CDS	2498591	2500147	+	rtn	hypothetical protein
i02_2514	CDS	2500229	2502043	+	yejA	hypothetical protein
i02_2515	CDS	2502044	2503138	+	yejB	ABC transporter permease
i02_2516	CDS	2503138	2504163	+	yejE	ABC transporter permease
i02_2517	CDS	2504165	2505754	+	yejF	ABC transporter ATP-binding protein
i02_2518	CDS	2505758	2506102	-	yejG	hypothetical protein
i02_2519	CDS	2506436	2507626	-	bcr	bicyclomycin/multidrug efflux system
i02_2520	CDS	2507654	2508349	-	rsuA	16S rRNA pseudouridylate synthase A
i02_2521	CDS	2508499	2510259	+	yejH	hypothetical protein
i02_2522	CDS	2510384	2510668	+	rplY	50S ribosomal protein L25
i02_2523	CDS	2510807	2511814	-	yejK	nucleoid-associated protein NdpA
i02_2524	CDS	2511859	2511975	-	/	hypothetical protein
i02_2526	CDS	2511996	2512223	+	yejL	hypothetical protein
i02_2527	CDS	2512243	2514003	+	yejM	hypothetical protein
i02_2528	CDS	2514256	2514411	-	/	hypothetical protein
i02_2529	CDS	2514493	2514882	-	/	hypothetical protein
i02_2530	CDS	2515225	2515482	-	/	hypothetical protein
i02_2531	CDS	2515484	2516161	+	narP	transcriptional regulator NarP
i02_2532	CDS	2516196	2517248	-	ccmH	cytochrome c-type biogenesis protein ccmH
i02_2533	CDS	2517245	2517802	-	dsbE	Thiol:disulfide interchange protein dsbE
i02_2534	CDS	2517799	2519742	-	ccmF	cytochrome c-type biogenesis protein ccmF
i02_2535	CDS	2519739	2520218	-	ccmE	cytochrome c-type biogenesis protein CcmE
i02_2536	CDS	2520215	2520424	-	ccmD	Heme exporter protein D
i02_2537	CDS	2520421	2521182	-	ccmC	Heme exporter protein C
i02_2538	CDS	2521200	2521862	-	ccmB	Heme exporter protein B
i02_2539	CDS	2521859	2522482	-	ccmA	cytochrome c biogenesis protein CcmA
i02_2540	CDS	2522495	2523097	-	napC	cytochrome c-type protein NapC
i02_2541	CDS	2523107	2523577	-	napB	citrate reductase cytochrome c-type subunit
i02_2542	CDS	2523553	2524416	-	napH	quinol dehydrogenase membrane component

Locus_tag	Type	start	End	+/-[a]	Gene[b]	Product
i02_2543	CDS	2524403	2525098	-	napG	quinol dehydrogenase periplasmic component
i02_2544	CDS	2525105	2527591	-	napA	nitrate reductase catalytic subunit
i02_2545	CDS	2527588	2527851	-	napD	assembly protein for periplasmic nitrate
i02_2546	CDS	2527841	2528335	-	napF	ferredoxin-type protein
i02_2547	CDS	2528435	2528608	+	/	hypothetical protein
i02_2548	CDS	2528528	2528656	-	/	hypothetical protein
i02_2549	CDS	2528749	2529231	+	eco	ecotin
i02_2550	CDS	2528923	2529381	-	eco	ecotin precursor
i02_2551	CDS	2529381	2531027	-	yojH	malate:quinone oxidoreductase
i02_2552	CDS	2531245	2532888	-	yojI	multidrug transporter membrane
i02_2553	CDS	2532964	2533614	-	alkB	alkylated DNA repair protein AlkB
i02_2554	CDS	2533614	2534678	-	ada	ADA regulatory protein
i02_2555	CDS	2534752	2535807	-	yojL	thiamine biosynthesis lipoprotein ApbE
i02_2556	CDS	2535919	2537046	-	ompC	outer membrane porin protein C
i02_2557	CDS	2537785	2540457	+	yojN	phosphotransfer intermediate protein
i02_2558	CDS	2540429	2541124	+	rcsB	transcriptional regulator RcsB
i02_2559	CDS	2541210	2544059	-	rcsC	hybrid sensory kinase in two-component
i02_2560	CDS	2544214	2546052	+	atoS	sensory histidine kinase AtoS
i02_2561	CDS	2546049	2547434	+	atoC	acetoacetate metabolism regulatory protein AtoC
i02_2562	CDS	2547630	2548292	+	atoD	acetyl-CoA:acetoacetyl-CoA transferase subunit
i02_2563	CDS	2548292	2548942	+	atoA	acetate CoA-transferase beta subunit
i02_2564	CDS	2548939	2550261	+	atoE	Short-chain fatty acids transporter
i02_2565	CDS	2550292	2551476	+	atoB	Acetyl-CoA acetyltransferase
i02_2566	CDS	2551550	2552326	-	yfaP	hypothetical protein
i02_2567	CDS	2552331	2553980	-	yfaQ	hypothetical protein
i02_2568	CDS	2553981	2558585	-	/	hypothetical protein
i02_2569	CDS	2558519	2559142	-	yfaT	hypothetical protein
i02_2570	CDS	2559139	2560827	-	yfaA	hypothetical protein
i02_2571	CDS	2560976	2563603	-	gyrA	DNA gyrase subunit A
i02_2572	CDS	2563702	2564472	+	ubiG	3-demethylubiquinone-9 3-methyltransferase
i02_2573	CDS	2564612	2568376	-	yfaL	adhesin
i02_2574	CDS	2568794	2569006	-	/	conserved domain protein
i02_2575	CDS	2569052	2571337	+	nrdA	ribonucleotide-diphosphate reductase subunit
i02_2576	CDS	2571527	2572657	+	nrdB	ribonucleotide-diphosphate reductase subunit
i02_2577	CDS	2572657	2572911	+	yfaE	2Fe-2S ferredoxin YfaE
i02_2578	CDS	2572965	2573615	-	inaA	hypothetical protein
i02_2579	CDS	2573818	2574894	-	glpQ	glycerophosphodiester phosphodiesterase
i02_2580	CDS	2574899	2576257	-	glpT	sn-glycerol-3-phosphate transporter
i02_2581	CDS	2576530	2578158	+	glpA	sn-glycerol-3-phosphate dehydrogenase subunit A
i02_2582	CDS	2578076	2579407	+	glpB	anaerobic glycerol-3-phosphate dehydrogenase
i02_2583	CDS	2579404	2580594	+	glpC	sn-glycerol-3-phosphate dehydrogenase subunit C
i02_2584	CDS	2580619	2580744	+	/	hypothetical protein
i02_2585	CDS	2580811	2581713	+	yfaD	hypothetical protein
i02_2586	CDS	2581754	2582557	-	yfaU	putative aldolase
i02_2587	CDS	2582575	2583903	-	yfaV	putative transport protein YfaV
i02_2588	CDS	2583921	2585138	-	yfaW	hypothetical protein
i02_2589	CDS	2585141	2585923	-	yfaX	putative transcriptional regulator YfaX
i02_2590	CDS	2586143	2587345	-	/	competence damage-inducible protein A
i02_2591	CDS	2587445	2588008	-	yfaZ	hypothetical protein
i02_2592	CDS	2588266	2588691	+	yfaO	putative Nudix hydrolase yfaO
i02_2593	CDS	2588730	2589359	-	ais	Ais protein
i02_2594	CDS	2589607	2590779	+	yfbE	UDP-L-Ara4O C-4 transaminase
i02_2595	CDS	2590783	2591751	+	/	undecaprenyl phosphate
i02_2596	CDS	2591751	2593733	+	yfbG	bifunctional UDP-glucuronic acid
i02_2597	CDS	2593730	2594620	+	yfbH	hypothetical protein
i02_2598	CDS	2594620	2596272	+	arnT	4-amino-4-deoxy-L-arabinose transferase
i02_2599	CDS	2596269	2596604	+	/	hypothetical protein
i02_2600	CDS	2596604	2596990	+	yfbJ	hypothetical protein

Locus_tag	Type	start	End	+/-[a]	Gene[b]	Product
i02_2601	CDS	2596984	2597280	-	pmrD	polymyxin B resistance protein pmrD
i02_2602	CDS	2597360	2598715	-	menE	O-succinylbenzoic acid--CoA ligase
i02_2603	CDS	2598712	2599674	-	menC	O-succinylbenzoate synthase
i02_2604	CDS	2599674	2600531	-	menB	naphthoate synthase
i02_2605	CDS	2599863	2600696	+	/	hypothetical protein
i02_2606	CDS	2600546	2601304	-	yfbB	acyl-CoA thioester hydrolase YfbB
i02_2607	CDS	2601301	2603160	-	menD	2-hydroxyglutarate synthase
i02_2608	CDS	2603060	2604355	-	menF	menaquinone-specific isochorismate synthase
i02_2609	CDS	2604434	2604739	-	elaB	hypothetical protein
i02_2610	CDS	2604794	2605255	-	elaA	hypothetical protein
i02_2611	CDS	2605320	2606237	+	elaC	ribonuclease Z
i02_2612	CDS	2606358	2608100	-	yfbK	hypothetical protein
i02_2613	CDS	2608242	2609210	+	yfbL	hypothetical protein
i02_2614	CDS	2609313	2609816	+	yfbM	hypothetical protein
i02_2615	CDS	2609883	2611340	-	nuoN	NADH dehydrogenase subunit N
i02_2616	CDS	2611347	2612876	-	nuoM	NADH dehydrogenase subunit M
i02_2617	CDS	2613040	2614881	-	nuoL	NADH dehydrogenase subunit L
i02_2618	CDS	2614878	2615180	-	nuoK	NADH dehydrogenase subunit K
i02_2619	CDS	2615177	2615731	-	nuoJ	NADH dehydrogenase subunit J
i02_2620	CDS	2615743	2616285	-	nuoI	NADH dehydrogenase subunit I
i02_2621	CDS	2616300	2617277	-	nuoH	NADH dehydrogenase subunit H
i02_2622	CDS	2617274	2620006	-	nuoG	NADH dehydrogenase subunit G
i02_2623	CDS	2620053	2621390	-	nuoF	NADH dehydrogenase I subunit F
i02_2624	CDS	2621387	2621887	-	nuoE	NADH dehydrogenase subunit E
i02_2625	CDS	2621890	2623692	-	nuoC	bifunctional NADH:ubiquinone oxidoreductase
i02_2626	CDS	2623786	2624448	-	nuoB	NADH dehydrogenase subunit B
i02_2627	CDS	2624464	2624907	-	nuoA	NADH dehydrogenase subunit A
i02_2628	CDS	2625054	2625323	-	/	hypothetical protein
i02_2629	CDS	2625537	2626475	-	lrhA	transcriptional regulator lrhA
i02_2630	CDS	2627395	2628612	+	/	aminotransferase AlaT
i02_2631	CDS	2628696	2629295	+	yfbR	hypothetical protein
i02_2632	CDS	2629354	2631186	-	yfbS	hypothetical protein
i02_2633	CDS	2631273	2631923	-	yfbT	putative phosphatase
i02_2634	CDS	2631934	2632446	-	/	hypothetical protein
i02_2635	CDS	2632510	2632965	-	yfbV	hypothetical protein
i02_2636	CDS	2633034	2633165	-	/	hypothetical protein
i02_2637	CDS	2633303	2634505	+	ackA	acetate kinase
i02_2638	CDS	2634580	2636724	+	pta	phosphate acetyltransferase
i02_2639	CDS	2636893	2638434	+	yfcC	hypothetical protein
i02_2640	CDS	2638467	2639009	-	yfcD	hypothetical protein
i02_2641	CDS	2639067	2639621	-	yfcE	phosphodiesterase
i02_2642	CDS	2639674	2640318	-	yfcF	GST-like protein yfcF
i02_2643	CDS	2640454	2641101	+	yfcG	glutathione S-transferase
i02_2644	CDS	2641158	2641520	+	folX	D-erythro-7,8-dihydroneopterin triphosphate
i02_2645	CDS	2641541	2642434	+	yfcH	hypothetical protein
i02_2646	CDS	2642593	2643366	-	hisP	histidine/lysine/arginine/ornithine transporter
i02_2647	CDS	2643374	2644090	-	hisM	histidine transport system permease protein
i02_2648	CDS	2644087	2644773	-	hisQ	histidine transport system permease protein
i02_2649	CDS	2644863	2645645	-	hisJ	histidine-binding periplasmic protein precursor
i02_2650	pseudo	2645866	2646648	-	hisJ	histidine-binding periplasmic protein precursor
i02_2651	CDS	2646793	2646936	-	/	hypothetical protein
i02_2652	CDS	2646914	2647483	-	ubiX	3-octaprenyl-4-hydroxybenzoate carboxy-lyase
i02_2653	CDS	2647578	2649095	-	purF	amidophosphoribosyltransferase
i02_2654	CDS	2649132	2649620	-	cvpA	colicin V production protein
i02_2655	CDS	2649722	2649892	+	/	hypothetical protein
i02_2656	CDS	2650152	2650814	-	dedD	hypothetical protein
i02_2657	CDS	2650804	2652072	-	folC	bifunctional folylpolyglutamate synthase
i02_2658	CDS	2652142	2653056	-	accD	acetyl-CoA carboxylase subunit beta

Locus_tag	Type	start	End	+/-[a]	Gene[b]	Product
i02_2659	CDS	2653212	2653871	-	dedA	hypothetical protein
i02_2660	CDS	2653954	2654766	-	truA	tRNA pseudouridine synthase A
i02_2661	CDS	2654766	2655779	-	usg	putative semialdehyde dehydrogenase
i02_2662	CDS	2655760	2655873	+	/	hypothetical protein
i02_2663	CDS	2655845	2656981	-	pdxB	erythronate-4-phosphate dehydrogenase
i02_2664	CDS	2657080	2658075	+	flk	flagella biosynthesis regulator
i02_2665	CDS	2658072	2659250	-	yfcJ	hypothetical protein
i02_2666	CDS	2659515	2660735	-	fabB	3-oxoacyl-(acyl carrier protein) synthase I
i02_2667	CDS	2660894	2662900	+	mnmC	5-methylaminomethyl-2-thiouridine
i02_2668	CDS	2663021	2663299	-	yfcL	hypothetical protein
i02_2669	CDS	2663333	2663881	-	yfcM	hypothetical protein
i02_2670	CDS	2663881	2664690	-	yfcA	hypothetical protein
i02_2671	CDS	2664690	2665514	-	mepA	penicillin-insensitive murein endopeptidase
i02_2672	CDS	2665518	2666606	-	aroC	chorismate synthase
i02_2673	CDS	2666638	2667903	-	yfcB	N5-glutamine S-adenosyl-L-methionine-dependent
i02_2674	CDS	2667736	2668287	+	yfcN	hypothetical protein
i02_2675	CDS	2668607	2669479	-	yfcO	hypothetical protein
i02_2676	CDS	2669466	2669993	-	yfcP	fimbrial-like protein yfcP precursor
i02_2677	CDS	2669990	2670469	-	yfcQ	fimbrial-like protein yfcQ precursor
i02_2678	CDS	2670466	2671011	-	yfcR	hypothetical protein
i02_2679	CDS	2670986	2671738	-	yfcS	fimbrial chaperone yfcS precursor
i02_2680	CDS	2671758	2674412	-	yfcU	hypothetical protein
i02_2681	CDS	2674487	2675053	-	yfcV	fimbrial-like protein yfcV precursor
i02_2682	CDS	2675620	2676105	-	/	phosphohistidine phosphatase
i02_2683	CDS	2676308	2678452	-	fadJ	multifunctional fatty acid oxidation complex
i02_2684	CDS	2678452	2679762	-	fadI	3-ketoacyl-CoA thiolase
i02_2685	CDS	2679942	2680250	-	yfcZ	hypothetical protein
i02_2686	CDS	2680311	2680562	-	/	hypothetical protein
i02_2687	CDS	2680592	2681938	+	fadL	long-chain fatty acid outer membrane
i02_2688	CDS	2682000	2682755	-	vacJ	VacJ lipoprotein precursor
i02_2689	CDS	2682781	2682951	-	/	hypothetical protein
i02_2690	CDS	2683049	2683981	+	yfdC	hypothetical protein
i02_2691	CDS	2684569	2685099	-	/	hypothetical protein
i02_2693	CDS	2685147	2693078	-	/	outer membrane autotransporter
i02_2694	CDS	2693103	2693723	-	/	hypothetical protein
i02_2695	CDS	2694041	2694670	-	/	Type 1 fimbriae regulatory protein fimB
i02_2696	CDS	2695418	2695987	+	/	Type 1 fimbriae regulatory protein fimB
i02_2697	CDS	2696382	2697335	-	dsdC	DNA-binding transcriptional regulator DsdC
i02_2698	CDS	2697547	2698884	+	dsdX	permease DsdX
i02_2699	CDS	2698902	2700230	+	dsdA	D-serine dehydratase
i02_2700	CDS	2700338	2701876	-	emrY	multidrug resistance protein Y
i02_2701	CDS	2701563	2701901	+	/	hypothetical protein
i02_2702	CDS	2701876	2703039	-	emrK	multidrug resistance protein K
i02_2703	CDS	2703455	2704069	+	evgA	DNA-binding transcriptional activator EvgA
i02_2704	CDS	2704074	2707667	+	evgS	hybrid sensory histidine kinase in two-component
i02_2705	CDS	2707723	2708907	-	yfdE	hypothetical protein
i02_2706	CDS	2708942	2709886	-	yfdV	putative transporter YfdV
i02_2707	CDS	2709956	2711650	-	/	putative oxalyl-CoA decarboxylase
i02_2708	CDS	2711704	2712954	-	yfdW	formyl-coenzyme A transferase
i02_2709	CDS	2713466	2714098	-	/	hypothetical protein
i02_2710	CDS	2714393	2714668	+	ypdI	lipoprotein ypdI precursor
i02_2711	CDS	2714745	2714987	-	yfdY	hypothetical protein
i02_2712	CDS	2715340	2716260	+	ddg	lipid A biosynthesis protein
i02_2713	CDS	2716752	2717990	-	yfdZ	aminotransferase
i02_2715	CDS	2718178	2718315	-	/	hypothetical protein
i02_2716	CDS	2718367	2720064	+	ypdA	hypothetical protein
i02_2717	CDS	2720061	2720816	+	ypdB	hypothetical protein
i02_2718	CDS	2720829	2721686	+	ypdC	putative transcriptional regulator YpdC

Locus_tag	Type	start	End	+/-[a]	Gene[b]	Product
i02_2719	CDS	2721689	2724184	-	/	putative phosphoenolpyruvate-protein
i02_2720	CDS	2724209	2725246	-	ypdE	exoaminopeptidase
i02_2721	CDS	2725246	2726331	-	/	aminopeptidase
i02_2722	CDS	2726347	2727594	-	/	putative PTS system IIC component ypdG
i02_2723	CDS	2727616	2727993	-	/	putative PTS system IIB component ypdH
i02_2724	CDS	2728161	2729126	-	glk	glucokinase
i02_2725	CDS	2729330	2730586	+	yfeO	hypothetical protein
i02_2726	CDS	2730701	2731027	+	ypeC	hypothetical protein
i02_2727	CDS	2731168	2732406	-	/	manganese transport protein MntH
i02_2728	CDS	2732703	2733944	+	nupC	nucleoside permease nupC
i02_2729	CDS	2733993	2736242	-	yfeA	hypothetical protein
i02_2730	CDS	2736685	2737044	+	yfeC	hypothetical protein
i02_2731	CDS	2737046	2737438	+	yfeD	hypothetical protein
i02_2732	CDS	2737489	2738904	-	gltX	glutamyl-tRNA synthetase
i02_2733	CDS	2738908	2739039	+	/	hypothetical protein
i02_2734	CDS	2739548	2740447	-	xapR	DNA-binding transcriptional activator XapR
i02_2735	CDS	2740473	2740631	-	/	hypothetical protein
i02_2736	CDS	2740684	2741940	-	xapB	xanthosine permease
i02_2737	CDS	2742001	2742834	-	xapA	purine nucleoside phosphorylase
i02_2738	CDS	2742928	2743047	-	/	hypothetical protein
i02_2739	CDS	2743082	2743846	+	yfeN	hypothetical protein
i02_2740	CDS	2743885	2744811	-	yfeR	putative transcriptional regulator YfeR
i02_2741	CDS	2744901	2745899	+	yfeH	hypothetical protein
i02_2742	CDS	2745896	2746114	-	/	hypothetical protein
i02_2743	CDS	2746116	2748131	-	ligA	NAD-dependent DNA ligase LigA
i02_2744	CDS	2748202	2749251	-	zipA	cell division protein ZipA
i02_2745	CDS	2749430	2750191	+	cysZ	putative sulfate transport protein CysZ
i02_2746	CDS	2750376	2751347	+	cysK	cysteine synthase A
i02_2747	CDS	2751466	2751594	-	/	hypothetical protein
i02_2748	CDS	2751731	2751988	+	ptsH	phosphohistidinoprotein-hexose
i02_2749	CDS	2752033	2753760	+	ptsI	phosphoenolpyruvate-protein phosphotransferase
i02_2750	CDS	2753801	2754310	+	crr	glucose-specific PTS system component
i02_2751	CDS	2754353	2755204	-	pdxK	pyridoxal kinase
i02_2752	CDS	2755309	2755677	+	yfeK	hypothetical protein
i02_2753	CDS	2755680	2756591	-	cysM	cysteine synthase B
i02_2754	CDS	2756726	2757823	-	cysA	sulfate/thiosulfate transporter subunit
i02_2755	CDS	2757813	2758688	-	cysW	sulfate/thiosulfate transporter permease
i02_2756	CDS	2758688	2759521	-	cysU	sulfate/thiosulfate transporter subunit
i02_2757	CDS	2759521	2760537	-	cysP	thiosulfate transporter subunit
i02_2758	CDS	2760695	2761486	-	ucpA	short chain dehydrogenase
i02_2759	CDS	2761766	2762662	+	murQ	N-acetylmuramic acid-6-phosphate etherase
i02_2760	CDS	2762666	2764090	+	murP	N-acetylmuramic acid phosphotransfer permease
i02_2761	CDS	2764110	2764253	-	/	hypothetical protein
i02_2762	CDS	2764253	2765179	+	/	hypothetical protein
i02_2763	CDS	2765263	2765838	-	yfeY	hypothetical protein
i02_2764	CDS	2765899	2766354	-	yfeZ	hypothetical protein
i02_2765	CDS	2766335	2766760	-	ypeA	putative acetyltransferase
i02_2766	CDS	2766974	2767843	+	amiA	N-acetylmuramoyl-l-alanine amidase I
i02_2767	CDS	2767844	2768746	+	hemF	coproporphyrinogen III oxidase
i02_2768	CDS	2768752	2769804	-	yfeG	transcriptional regulator EutR
i02_2769	CDS	2769850	2770356	-	/	ethanolamine utilization protein eutK precursor
i02_2770	CDS	2770363	2771022	-	/	ethanolamine utilization protein eutL
i02_2771	CDS	2771032	2771919	-	eutC	ethanolamine ammonia-lyase small subunit
i02_2772	CDS	2771940	2773301	-	eutB	ethanolamine ammonia-lyase heavy chain
i02_2773	CDS	2773313	2774716	-	eutA	reactivating factor for ethanolamine ammonia
i02_2774	CDS	2774713	2775939	-	eutH	ethanolamine utilization protein eutH
i02_2775	CDS	2776156	2777370	-	eutG	ethanolamine utilization protein eutG
i02_2776	CDS	2777333	2778169	-	eutJ	ethanolamine utilization protein eutJ

Locus_tag	Type	start	End	+/-[a]	Gene[b]	Product
i02_2777	CDS	2778180	2779583	-	eutE	ethanolamine utilization protein eutE
i02_2778	CDS	2779595	2779882	-	cchB	ethanolamine utilization protein eutN
i02_2779	CDS	2779989	2780324	-	cchA	ethanolamine utilization protein eutM precursor
i02_2780	CDS	2780321	2781337	-	eutD	phosphotransacetylase
i02_2781	CDS	2781334	2782137	-	/	ethanolamine utilization cobalamin
i02_2782	CDS	2782134	2782835	-	/	ethanolamine utilization protein EutQ
i02_2783	CDS	2782810	2783289	-	/	ethanolamine utilization protein eutP
i02_2784	CDS	2783302	2783709	-	/	ethanolamine utilization protein eutS
i02_2785	CDS	2783930	2786209	-	/	malic enzyme
i02_2786	CDS	2786498	2787448	+	talA	transaldolase A
i02_2787	CDS	2787468	2789471	+	tktB	transketolase
i02_2788	CDS	2789574	2790617	-	ypfG	hypothetical protein
i02_2789	CDS	2790743	2791345	-	yffH	hypothetical protein
i02_2790	CDS	2791386	2793365	-	yffG	putative oxidoreductase Fe-S binding subunit
i02_2791	CDS	2793556	2795271	+	narQ	nitrate/nitrite sensor protein NarQ
i02_2792	CDS	2795435	2798548	+	acrD	aminoglycoside/multidrug efflux system
i02_2793	CDS	2798726	2798848	+	/	conserved hypothetical protein
i02_2794	CDS	2799087	2799443	+	yffB	hypothetical protein
i02_2795	CDS	2799447	2800574	+	dapE	succinyl-diaminopimelate desuccinylase
i02_2796	CDS	2800602	2800802	+	/	hypothetical protein
i02_2797	CDS	2800883	2801605	-	ypfH	esterase YpfH
i02_2798	CDS	2801655	2803670	-	ypfI	hypothetical protein
i02_2799	CDS	2803685	2804548	-	ypfJ	hypothetical protein
i02_2800	CDS	2804716	2805438	-	purC	phosphoribosylaminoimidazole-succinocarboxamide
i02_2801	CDS	2805642	2806679	-	nlpB	lipoprotein
i02_2802	CDS	2806693	2807589	-	dapA	dihydrodipicolinate synthase
i02_2803	CDS	2807717	2808289	+	gcvR	predicted transcriptional regulator
i02_2804	CDS	2808289	2808759	+	bcp	thioredoxin-dependent thiol peroxidase
i02_2805	CDS	2808858	2809919	-	perM	putative permease PerM
i02_2806	CDS	2809950	2810126	+	/	hypothetical protein
i02_2807	CDS	2810132	2811595	+	yfgC	hypothetical protein
i02_2808	CDS	2811616	2811975	+	/	hypothetical protein
i02_2809	CDS	2812113	2812859	-	yfgE	DNA replication initiation factor
i02_2810	CDS	2812909	2814240	-	uraA	uracil transporter
i02_2811	CDS	2814284	2814910	-	upp	uracil phosphoribosyltransferase
i02_2812	CDS	2814872	2815216	+	/	hypothetical protein
i02_2813	CDS	2815220	2816272	+	purM	phosphoribosylaminoimidazole synthetase
i02_2814	CDS	2816272	2816910	+	purN	phosphoribosylglycinamide formyltransferase
i02_2815	CDS	2817076	2819148	+	ppk	polyphosphate kinase
i02_2816	CDS	2819153	2820694	+	ppx	exopolyphosphatase
i02_2817	CDS	2820733	2822976	-	yfgF	hypothetical protein
i02_2819	CDS	2823193	2823483	-	/	hypothetical protein
i02_2820	CDS	2823830	2824348	+	yfgH	lipoprotein yfgH precursor
i02_2821	CDS	2824364	2824903	+	yfgI	hypothetical protein
i02_2822	CDS	2824997	2826574	-	guaA	GMP synthase
i02_2823	CDS	2826643	2828178	-	guaB	inosine 5'-monophosphate dehydrogenase
i02_2824	CDS	2828271	2829647	+	xseA	exodeoxyribonuclease VII large subunit
i02_2825	CDS	2829716	2837767	-	/	RatA-like protein
i02_2826	CDS	2837888	2838865	-	/	SinI-like protein
i02_2827	CDS	2838920	2841127	-	/	SinH-like protein
i02_2828	CDS	2841306	2841521	-	yfgJ	hypothetical protein
i02_2829	CDS	2841584	2843095	-	engA	GTP-binding protein EngA
i02_2830	CDS	2843174	2844316	-	yfgL	outer membrane protein assembly complex subunit
i02_2831	CDS	2844363	2844983	-	yfgM	hypothetical protein
i02_2832	CDS	2845001	2846275	-	hisS	histidyl-tRNA synthetase
i02_2833	CDS	2846386	2847504	-	ispG	4-hydroxy-3-methylbut-2-en-1-yl diphosphate

Locus_tag	Type	start	End	+/-[a]	Gene[b]	Product
i02_2834	CDS	2847531	2848538	-	yfgA	hypothetical protein
i02_2835	CDS	2848823	2849977	-	yfgB	hypothetical protein
i02_2836	CDS	2850127	2850558	-	ndk	nucleoside diphosphate kinase
i02_2837	CDS	2850707	2853028	-	pbpC	penicillin-binding protein 1C
i02_2838	CDS	2853029	2857990	-	yfhM	lipoprotein yfhM precursor
i02_2839	CDS	2858044	2859042	+	sseA	3-mercaptopyruvate sulfurtransferase
i02_2840	CDS	2859026	2859178	+	/	hypothetical protein
i02_2841	CDS	2859370	2859507	+	/	hypothetical protein
i02_2842	CDS	2859539	2860324	-	sseB	enhanced serine sensitivity protein SseB
i02_2843	CDS	2860458	2861741	-	pepB	aminopeptidase B
i02_2844	CDS	2861800	2862000	-	yfhJ	hypothetical protein
i02_2845	CDS	2862012	2862347	-	fdx	ferredoxin, 2Fe-2S
i02_2846	CDS	2862349	2864253	-	hscA	chaperone protein HscA
i02_2847	CDS	2864216	2864731	-	hscB	co-chaperone HscB
i02_2848	CDS	2864739	2864855	-	/	hypothetical protein
i02_2849	CDS	2864827	2865150	-	iscA	iron-sulfur cluster assembly protein
i02_2850	CDS	2865167	2865553	-	/	scaffold protein
i02_2851	CDS	2865581	2866819	-	yfhO	cysteine desulfurase
i02_2852	CDS	2866907	2867395	-	yfhP	DNA-binding transcriptional regulator IscR
i02_2853	CDS	2867666	2868406	-	yfhQ	putative tRNA/rRNA methyltransferase YfhQ
i02_2854	CDS	2868525	2869328	+	suhB	inositol monophosphatase
i02_2855	CDS	2869446	2870327	+	yfhR	hypothetical protein
i02_2856	CDS	2870497	2871798	+	csiE	stationary phase inducible protein CsiE
i02_2857	CDS	2871790	2872929	-	hcaT	putative 3-phenylpropionic acid transporter
i02_2858	CDS	2872963	2873106	-	/	hypothetical protein
i02_2859	CDS	2873149	2873301	+	/	hypothetical protein
i02_2860	CDS	2873253	2873720	+	yphA	hypothetical protein
i02_2861	CDS	2873880	2874752	-	yphB	hypothetical protein
i02_2862	CDS	2874764	2875858	-	yphC	hypothetical protein
i02_2863	CDS	2875891	2876889	-	yphD	ABC transporter permease
i02_2864	CDS	2876914	2878425	-	yphE	ABC transporter ATP-binding protein
i02_2865	CDS	2878448	2879431	-	yphF	ABC transporter periplasmic-binding protein
i02_2866	CDS	2879528	2882902	-	yphG	hypothetical protein
i02_2867	CDS	2882921	2884120	+	yphH	hypothetical protein
i02_2868	CDS	2884184	2885443	-	glyA	serine hydroxymethyltransferase
i02_2869	CDS	2885589	2885717	+	/	hypothetical protein
i02_2870	CDS	2885766	2886956	+	hmpA	nitric oxide dioxygenase
i02_2871	CDS	2887001	2887339	-	glnB	nitrogen regulatory protein P-II 1
i02_2872	CDS	2887400	2888773	-	yfhA	hypothetical protein
i02_2873	CDS	2888724	2889443	-	yfhG	hypothetical protein
i02_2874	CDS	2889602	2891092	-	yfhK	putative sensor-like histidine kinase yfhK
i02_2875	CDS	2891605	2895492	-	purL	phosphoribosylformylglycinamidine synthase
i02_2876	pseudo	2895750	2897306	+	yfhD	putative transglycosylase
i02_2877	CDS	2897303	2897839	-	yfhC	tRNA-specific adenosine deaminase
i02_2878	CDS	2897864	2898499	-	yfhB	hypothetical protein
i02_2879	CDS	2898460	2898597	-	/	hypothetical protein
i02_2880	CDS	2898636	2899556	+	yfhH	putative DNA-binding transcriptional regulator
i02_2881	CDS	2899612	2899872	+	yfhL	putative ferredoxin-like protein yfhL
i02_2882	CDS	2900567	2900947	-	acpS	4'-phosphopantetheinyl transferase
i02_2883	CDS	2900947	2901693	-	pdxJ	pyridoxine 5'-phosphate synthase
i02_2884	CDS	2901690	2902418	-	recO	DNA repair protein RecO
i02_2885	CDS	2902430	2903335	-	era	GTP-binding protein Era
i02_2886	CDS	2903332	2904012	-	rnc	ribonuclease III
i02_2887	CDS	2904284	2905258	-	lepB	signal peptidase I
i02_2888	CDS	2905274	2907073	-	lepA	GTP-binding protein LepA
i02_2889	CDS	2907271	2907750	-	rseC	SoxR reducing system protein RseC
i02_2890	CDS	2907747	2908703	-	rseB	periplasmic negative regulator of sigmaE
i02_2891	CDS	2908703	2909353	-	rseA	anti-RNA polymerase sigma factor SigE

Locus_tag	Type	start	End	+/-[a]	Gene[b]	Product
i02_2892	CDS	2909386	2909994	-	rpoE	RNA polymerase sigma factor RpoE
i02_2893	CDS	2910369	2911991	+	nadB	L-aspartate oxidase
i02_2894	CDS	2911976	2912833	-	yfiC	hypothetical protein
i02_2895	CDS	2912845	2914179	+	srmB	ATP-dependent RNA helicase SrmB
i02_2896	CDS	2914212	2915138	-	yfiE	putative transcriptional regulator YfiE
i02_2897	CDS	2915196	2915783	+	yfiK	neutral amino-acid efflux protein
i02_2898	CDS	2915839	2916222	-	yfiD	autonomous glycyl radical cofactor GrcA
i02_2899	CDS	2915956	2916333	+	/	hypothetical protein
i02_2900	CDS	2916527	2917216	+	ung	uracil-DNA glycosylase
i02_2901	CDS	2917264	2918301	-	yfiF	putative methyltransferase
i02_2902	CDS	2917837	2918439	+	yfiF	rRNA methyltransferase YfiF
i02_2903	CDS	2918508	2918927	+	trxC	thioredoxin 2
i02_2904	CDS	2918945	2919694	+	yfiP	hypothetical protein
i02_2905	CDS	2919726	2922386	+	yfiQ	hypothetical protein
i02_2906	CDS	2922497	2923855	+	pssA	phosphatidylserine synthase
i02_2907	CDS	2923880	2924224	+	yfiM	hypothetical protein
i02_2908	CDS	2924221	2925612	-	kgtP	alpha-ketoglutarate transporter
i02_2910	CDS	2931121	2931255	+	/	hypothetical protein
i02_2911	CDS	2931295	2933880	-	clpB	protein disaggregation chaperone
i02_2912	CDS	2933998	2934729	-	yfiH	hypothetical protein
i02_2913	CDS	2934726	2935706	-	rluD	23S rRNA pseudouridine synthase D
i02_2914	CDS	2935841	2936578	+	yfiO	outer membrane protein assembly complex subunit
i02_2915	CDS	2936848	2937189	+	yfiA	translation inhibitor protein RaiA
i02_2916	CDS	2937439	2938599	+	pheA	bifunctional chorismate mutase/prephenate
i02_2917	CDS	2938642	2939763	-	tyrA	bifunctional chorismate mutase/prephenate
i02_2918	CDS	2939774	2940844	-	aroF	phospho-2-dehydro-3-deoxyheptonate aldolase
i02_2919	CDS	2941066	2941419	+	yfiL	hypothetical protein
i02_2920	CDS	2941566	2942084	+	yfiR	hypothetical protein
i02_2921	CDS	2942074	2943300	+	yfiN	hypothetical protein
i02_2922	CDS	2943316	2943798	+	yfiB	putative outer membrane lipoprotein
i02_2923	CDS	2943875	2944222	-	rplS	50S ribosomal protein L19
i02_2924	CDS	2944264	2945031	-	trmD	tRNA (guanine-N(1)-)-methyltransferase
i02_2925	CDS	2945062	2945613	-	rimM	16S rRNA-processing protein RimM
i02_2926	CDS	2945629	2945937	-	rpsP	30S ribosomal protein S16
i02_2927	CDS	2946014	2947375	-	ffh	signal recognition particle protein
i02_2928	CDS	2947467	2948333	+	ypjD	hypothetical protein
i02_2929	CDS	2948414	2949640	+	/	hypothetical protein
i02_2930	CDS	2949695	2950288	-	grpE	heat shock protein GrpE
i02_2931	CDS	2950285	2950536	-	/	hypothetical protein
i02_2932	CDS	2950411	2951289	+	ppnK	inorganic polyphosphate/ATP-NAD kinase
i02_2933	CDS	2951375	2953036	+	recN	recombination and repair protein
i02_2934	CDS	2953185	2953526	+	/	hypothetical protein
i02_2935	CDS	2953588	2953878	-	/	hypothetical protein
i02_2936	CDS	2953868	2954344	-	yfjG	hypothetical protein
i02_2937	CDS	2954476	2954958	+	smpB	SsrA-binding protein
i02_2938	CDS	2956218	2957195	+	ygaT	hypothetical protein
i02_2939	CDS	2957149	2958483	+	ygaF	hydroxyglutarate oxidase
i02_2940	CDS	2958506	2959954	+	gabD	succinate-semialdehyde dehydrogenase I
i02_2941	CDS	2959962	2961248	+	gabT	4-aminobutyrate aminotransferase
i02_2942	CDS	2961422	2962885	+	gabP	gamma-aminobutyrate transporter
i02_2943	CDS	2962888	2963568	+	ygaE	DNA-binding transcriptional regulator CsiR
i02_2944	CDS	2963569	2964018	-	ygaU	LysM domain/BON superfamily protein
i02_2945	CDS	2964090	2964275	+	/	hypothetical protein
i02_2946	CDS	2964443	2964742	+	ygaV	putative transcriptional regulator YgaV
i02_2947	CDS	2964752	2965276	+	ygaP	hypothetical protein
i02_2948	CDS	2965323	2965727	-	stpA	DNA binding protein, nucleoid-associated
i02_2949	CDS	2965889	2966020	-	/	hypothetical protein

Locus_tag	Type	start	End	+/-[a]	Gene[b]	Product
i02_2950	CDS	2966140	2966268	+	/	hypothetical protein
i02_2951	CDS	2966394	2966843	+	ygaW	hypothetical protein
i02_2952	CDS	2966880	2967224	-	ygaC	hypothetical protein
i02_2953	CDS	2967364	2967705	+	ygaM	hypothetical protein
i02_2954	CDS	2967762	2969096	-	/	hypothetical protein
i02_2955	CDS	2969184	2969615	+	/	hypothetical protein
i02_2956	CDS	2969823	2970068	+	nrdH	glutaredoxin-like protein
i02_2957	CDS	2970065	2970475	+	nrdI	ribonucleotide reductase stimulatory protein
i02_2958	CDS	2970448	2972592	+	nrdE	ribonucleotide-diphosphate reductase subunit
i02_2959	CDS	2972602	2973561	+	nrdF	ribonucleotide-diphosphate reductase subunit
i02_2960	CDS	2973918	2975120	+	proV	glycine betaine transporter ATP-binding subunit
i02_2961	CDS	2975113	2976177	+	proW	glycine betaine transporter membrane protein
i02_2962	CDS	2976234	2977226	+	proX	glycine betaine transporter periplasmic subunit
i02_2963	CDS	2977279	2977425	+	/	hypothetical protein
i02_2964	CDS	2977418	2978602	+	/	putative transport protein
i02_2965	CDS	2978726	2979463	+	ygaZ	hypothetical protein
i02_2966	CDS	2979453	2979788	+	ygaH	hypothetical protein
i02_2967	CDS	2979879	2980409	+	emrR	transcriptional repressor MprA
i02_2968	CDS	2980524	2981708	+	emrA	multidrug resistance protein A
i02_2969	CDS	2981716	2983263	+	emrB	multidrug resistance protein B
i02_2970	CDS	2983521	2983877	+	/	hypothetical protein
i02_2972	CDS	2983889	2984218	+	/	hypothetical protein
i02_2973	CDS	2984215	2985327	+	/	hypothetical protein
i02_2974	CDS	2985376	2985891	-	ygaG	S-ribosylhomocysteinase
i02_2975	CDS	2986041	2987597	-	gshA	glutamate--cysteine ligase
i02_2976	CDS	2987670	2988098	-	yqaA	hypothetical protein
i02_2977	CDS	2988095	2988661	-	yqaB	fructose-1-phosphatase
i02_2978	CDS	2988691	2988816	-	/	hypothetical protein
i02_2979	CDS	2989989	2990174	-	csrA	carbon storage regulator
i02_2980	CDS	2990284	2990400	+	/	hypothetical protein
i02_2981	CDS	2990409	2993039	-	alaS	alanyl-tRNA synthetase
i02_2982	CDS	2992944	2993114	+	/	hypothetical protein
i02_2983	CDS	2993168	2993668	-	recX	recombination regulator RecX
i02_2984	CDS	2993737	2994813	-	recA	recombinase A
i02_2985	CDS	2994878	2995378	-	ygaD	competence damage-inducible protein A
i02_2986	CDS	2995520	2996710	-	mltB	murein hydrolase B
i02_2987	CDS	2996862	2997425	+	srlA	PTS system, glucitol/sorbitol-specific IIC2
i02_2988	CDS	2997422	2998381	+	srlE	PTS system, glucitol/sorbitol-specific IIBC
i02_2989	CDS	2998392	2998763	+	srlB	glucitol/sorbitol-specific PTS system component
i02_2990	CDS	2998767	2999546	+	srlD	sorbitol-6-phosphate dehydrogenase
i02_2991	CDS	2999652	3000011	+	gutM	DNA-binding transcriptional activator GutM
i02_2992	CDS	3000078	3000851	+	srlR	DNA-bindng transcriptional repressor SrlR
i02_2993	CDS	3000844	3001809	+	gutQ	D-arabinose 5-phosphate isomerase
i02_2994	CDS	3001806	3003320	-	ygaA	anaerobic nitric oxide reductase transcription
i02_2995	CDS	3003507	3004946	+	norV	anaerobic nitric oxide reductase flavorubredoxin
i02_2997	CDS	3004943	3006076	+	ygbD	nitric oxide reductase
i02_2998	CDS	3006300	3008624	-	hypF	hydrogenase maturation protein hypF
i02_2999	CDS	3008705	3009247	-	hydN	electron transport protein HydN
i02_3000	CDS	3009326	3009511	-	/	hypothetical protein
i02_3001	CDS	3009617	3010045	+	/	hypothetical protein
i02_3002	CDS	3010008	3010133	-	/	hypothetical protein
i02_3003	CDS	3010137	3010556	-	/	hypothetical protein
i02_3004	CDS	3010553	3010864	-	/	hypothetical protein
i02_3005	CDS	3011027	3011800	-	/	hypothetical protein
i02_3006	CDS	3011825	3012295	-	hycI	hydrogenase 3 maturation protease
i02_3007	CDS	3012288	3012698	-	hycH	formate hydrogenlyase maturation protein hycH
i02_3008	CDS	3012695	3013462	-	hycG	formate hydrogenlyase subunit 7
i02_3009	CDS	3013462	3014004	-	hycF	formate hydrogenlyase complex iron-sulfur

Locus_tag	Type	start	End	+/-[a]	Gene[b]	Product
i02_3010	CDS	3014014	3015723	-	hycE	formate hydrogenlyase subunit 5 precursor
i02_3011	CDS	3015741	3016664	-	hycD	formate hydrogenlyase subunit 4
i02_3012	CDS	3016667	3018493	-	hycC	formate hydrogenlyase subunit 3
i02_3013	CDS	3018490	3019101	-	hycB	formate hydrogenlyase subunit 2
i02_3014	CDS	3019226	3019687	-	hycA	formate hydrogenlyase regulatory protein HycA
i02_3015	CDS	3019887	3020249	+	hypA	hydrogenase nickel incorporation protein
i02_3016	CDS	3020253	3021125	+	hypB	hydrogenase nickel incorporation protein HypB
i02_3017	CDS	3021116	3021388	+	hypC	hydrogenase assembly chaperone
i02_3018	CDS	3021388	3022509	+	hypD	hydrogenase isoenzyme formation protein hypD
i02_3019	CDS	3022506	3023516	+	hypE	hydrogenase isoenzyme formation protein hypE
i02_3020	CDS	3023557	3025668	+	fhlA	formate hydrogenlyase transcriptional activator
i02_3021	CDS	3025674	3026141	+	/	molybdenum-pterin-binding-protein
i02_3022	CDS	3026180	3026524	-	ygbA	hypothetical protein
i02_3023	CDS	3026810	3029371	+	mutS	DNA mismatch repair protein MutS
i02_3024	CDS	3029477	3030133	+	pphB	serine/threonine-specific protein phosphatase 2
i02_3025	CDS	3030184	3030981	-	ygbI	putative transcriptional regulator YgbI
i02_3026	CDS	3031147	3032055	+	ygbJ	oxidoreductase ygbJ
i02_3027	CDS	3032052	3033314	+	ygbK	hypothetical protein
i02_3028	CDS	3033311	3033949	+	ygbL	putative aldolase
i02_3029	CDS	3033954	3034730	+	ygbM	hypothetical protein
i02_3030	CDS	3034819	3036183	+	ygbN	inner membrane permease YgbN
i02_3031	CDS	3036326	3037369	-	/	hypothetical protein
i02_3032	CDS	3037388	3037534	+	/	hypothetical protein
i02_3033	CDS	3037594	3038145	+	/	hypothetical protein
i02_3034	CDS	3038323	3039351	-	/	hypothetical protein
i02_3035	CDS	3039378	3040517	-	nlpD	lipoprotein NlpD
i02_3036	CDS	3040375	3040542	+	/	hypothetical protein
i02_3037	CDS	3040657	3041283	-	pcm	protein-L-isoaspartate O-methyltransferase
i02_3038	CDS	3041277	3042083	-	surE	stationary phase survival protein SurE
i02_3039	CDS	3042019	3043068	-	truD	tRNA pseudouridine synthase D
i02_3040	CDS	3043065	3043544	-	ispF	2-C-methyl-D-erythritol 2,4-cyclodiphosphate synthase monomer
i02_3041	CDS	3043544	3044254	-	ispD	4-diphosphocytidyl-2C-methyl-D-erythritol synthetase monomer
i02_3042	CDS	3044273	3044539	-	ftsB	cell division protein FtsB
i02_3043	CDS	3044777	3045100	-	ygbE	hypothetical protein
i02_3044	CDS	3045150	3045755	-	cysC	adenylylsulfate kinase
i02_3045	CDS	3045755	3047182	-	cysN	sulfate adenylyltransferase subunit 1
i02_3046	CDS	3047184	3048092	-	cysD	sulfate adenylyltransferase subunit 2
i02_3047	CDS	3048344	3049381	+	iap	alkaline phosphatase isozyme conversion
i02_3048	CDS	3049923	3050657	-	cysH	phosphoadenosine phosphosulfate reductase
i02_3049	CDS	3050731	3052443	-	cysI	sulfite reductase subunit beta
i02_3050	CDS	3052443	3054242	-	cysJ	sulfite reductase subunit alpha
i02_3051	CDS	3054474	3054923	+	ygcM	putative 6-pyruvoyl tetrahydrobiopterin
i02_3052	CDS	3054971	3056272	+	ygcN	electron transfer flavoprotein-quinone
i02_3053	CDS	3056227	3056523	+	ygcO	ferredoxin-like protein ygcO
i02_3054	CDS	3056540	3057115	+	/	putative anti-terminator regulatory protein
i02_3055	CDS	3057263	3058156	-	ygcQ	putative electron transfer flavoprotein subunit
i02_3056	CDS	3058120	3058902	-	ygcR	putative electron transfer flavoprotein subunit
i02_3057	CDS	3058874	3060283	-	ygcS	metabolite transport protein
i02_3058	CDS	3060305	3061759	-	/	hypothetical protein
i02_3059	CDS	3061829	3062689	-	ygcW	oxidoreductase ygcW
i02_3060	CDS	3062933	3064210	+	yqcE	hypothetical protein
i02_3061	CDS	3064237	3065715	+	ygcE	putative sugar kinase ygcE
i02_3062	CDS	3066189	3066860	-	ygcF	hypothetical protein
i02_3063	CDS	3066894	3067019	+	/	hypothetical protein
i02_3064	CDS	3067069	3067635	+	/	hypothetical protein
i02_3065	CDS	3067619	3068527	+	/	hypothetical protein

Locus_tag	Type	start	End	+/-[a]	Gene[b]	Product
i02_3067	CDS	3068542	3069696	+	/	hypothetical protein
i02_3068	CDS	3069690	3070574	+	ygcG	hypothetical protein
i02_3069	CDS	3070634	3071932	-	eno	phosphopyruvate hydratase
i02_3070	CDS	3072020	3073657	-	pyrG	CTP synthetase
i02_3071	CDS	3073885	3074676	-	mazG	nucleoside triphosphate pyrophosphohydrolase
i02_3072	CDS	3074772	3077006	-	relA	GDP/GTP pyrophosphokinase
i02_3073	CDS	3077054	3078355	-	rumA	23S rRNA 5-methyluridine methyltransferase
i02_3074	CDS	3078412	3081168	+	barA	hybrid sensory histidine kinase BarA
i02_3075	CDS	3081298	3081414	-	/	hypothetical protein
i02_3076	CDS	3081399	3082739	-	ygcX	glucarate dehydratase
i02_3077	CDS	3082760	3084100	-	ygcY	glucarate dehydratase related protein
i02_3078	CDS	3084102	3085454	-	/	glucarate transporter
i02_3079	CDS	3085547	3085672	-	/	hypothetical protein
i02_3080	CDS	3085666	3085794	+	/	hypothetical protein
i02_3081	CDS	3085887	3086336	-	yqcA	flavodoxin
i02_3082	CDS	3086354	3087136	-	yqcB	tRNA pseudouridine synthase C
i02_3083	CDS	3087136	3087465	-	yqcC	hypothetical protein
i02_3084	CDS	3088087	3088632	-	syd	SecY interacting protein Syd
i02_3085	CDS	3088700	3089548	+	queF	7-cyano-7-deazaguanine reductase
i02_3086	CDS	3089660	3091024	+	ygdH	hypothetical protein
i02_3087	CDS	3091108	3091239	+	/	hypothetical protein
i02_3088	CDS	3091581	3092870	+	sdaC	Serine transporter
i02_3089	CDS	3092928	3094295	+	sdaB	L-serine dehydratase 2
i02_3090	CDS	3094317	3095162	+	xni	exonuclease IX
i02_3091	CDS	3095267	3096418	-	fucO	L-1,2-propanediol oxidoreductase
i02_3092	CDS	3096443	3097090	-	fucA	L-fuculose phosphate aldolase
i02_3093	CDS	3097280	3097417	+	/	hypothetical protein
i02_3094	CDS	3097637	3098953	+	fucP	L-fucose transporter
i02_3095	CDS	3098986	3100761	+	fucI	L-fucose isomerase
i02_3096	CDS	3100840	3102288	+	fucK	culokinase
i02_3097	CDS	3102290	3102712	+	fucU	fucose operon fucU protein
i02_3098	CDS	3102761	3103501	+	fucR	DNA-binding transcriptional activator FucR
i02_3099	CDS	3103545	3104645	-	ygdE	putative RNA 2'-O-ribose methyltransferase
i02_3100	CDS	3104638	3105033	-	ygdD	hypothetical protein
i02_3101	CDS	3105052	3105969	-	gcvA	DNA-binding transcriptional activator GcvA
i02_3102	CDS	3106320	3106550	-	ygdI	lipoprotein ygdI precursor
i02_3103	CDS	3106703	3107944	+	/	cysteine sulfinate desulfinase
i02_3104	CDS	3107944	3108387	+	ygdK	hypothetical protein
i02_3105	CDS	3108438	3109244	-	ygdL	hypothetical protein
i02_3106	CDS	3109321	3110418	-	mltA	murein transglycosylase A
i02_3107	CDS	3111538	3112059	+	/	hypothetical protein
i02_3108	CDS	3112112	3113656	+	/	hypothetical protein
i02_3109	CDS	3113676	3115013	+	/	hypothetical protein
i02_3110	CDS	3115010	3115675	+	/	hypothetical protein
i02_3111	CDS	3115688	3117340	+	/	hypothetical protein
i02_3112	CDS	3117398	3117889	+	/	secreted protein Hcp
i02_3113	CDS	3118081	3120717	+	/	ClpB protein
i02_3114	CDS	3120717	3123191	+	/	hypothetical protein
i02_3115	CDS	3123206	3124030	+	/	hypothetical protein
i02_3116	CDS	3124027	3126003	+	/	hypothetical protein
i02_3117	CDS	3126013	3126285	+	/	hypothetical protein
i02_3118	CDS	3126285	3127718	+	/	hypothetical protein
i02_3119	CDS	3127800	3131063	+	/	hypothetical protein
i02_3120	CDS	3131029	3132666	+	/	hypothetical protein
i02_3121	CDS	3132700	3134460	+	/	hypothetical protein
i02_3122	CDS	3134424	3135503	+	/	hypothetical protein
i02_3123	CDS	3135484	3136020	+	/	hypothetical protein
i02_3124	CDS	3136024	3136452	+	/	hypothetical protein

Locus_tag	Type	start	End	+/-[a]	Gene[b]	Product
i02_3125	CDS	3136452	3137828	+	/	hypothetical protein
i02_3127	CDS	3138128	3139084	-	/	2-hydroxyacid dehydrogenase
i02_3128	CDS	3139147	3139743	-	/	phosphosugar isomerase
i02_3129	CDS	3139746	3140921	-	/	Beta-cystathionase
i02_3130	CDS	3140921	3142501	-	/	PTS system, maltose and glucose-specific IIABC
i02_3131	CDS	3142533	3143414	-	/	antiterminator
i02_3132	CDS	3143453	3143605	+	/	hypothetical protein
i02_3133	CDS	3143615	3144958	-	/	N-acetylmuramoyl-L-alanine amidase amiC
i02_3134	CDS	3145100	3146431	+	argA	N-acetylglutamate synthase
i02_3135	CDS	3146493	3148319	-	recD	exonuclease V subunit alpha
i02_3136	CDS	3148319	3151870	-	recB	exonuclease V subunit beta
i02_3137	CDS	3151854	3154742	-	ptr	protease III
i02_3138	CDS	3154918	3158286	-	recC	exonuclease V subunit gamma
i02_3139	CDS	3158299	3158622	-	ppdC	hypothetical protein
i02_3140	CDS	3158607	3159014	-	ygdB	hypothetical protein
i02_3141	CDS	3159011	3159814	-	ppdB	hypothetical protein
i02_3142	CDS	3159565	3160035	-	ppdA	hypothetical protein
i02_3143	CDS	3160220	3161014	-	thyA	thymidylate synthase
i02_3144	CDS	3161021	3161896	-	lgt	prolipoprotein diacylglyceryl transferase
i02_3145	CDS	3162047	3164293	-	ptsP	fused phosphoenolpyruvate-protein
i02_3146	CDS	3164306	3164836	-	ygdP	dinucleoside polyphosphate hydrolase
i02_3147	CDS	3165521	3166210	+	mutH	DNA mismatch repair protein
i02_3148	CDS	3166279	3166992	+	/	hypothetical protein
i02_3149	CDS	3167130	3167348	+	ygdR	lipoprotein ygdR precursor
i02_3150	CDS	3167456	3168496	+	tas	putative aldo-keto reductase
i02_3151	CDS	3168528	3169721	-	ygeD	lysophospholipid transporter LplT
i02_3152	CDS	3169714	3171873	-	aas	bifunctional acyl-[acyl carrier protein]
i02_3153	CDS	3172459	3173490	+	galR	DNA-binding transcriptional regulator GalR
i02_3154	CDS	3173497	3174759	-	lysA	diaminopimelate decarboxylase
i02_3155	CDS	3174881	3175816	+	lysR	DNA-binding transcriptional regulator LysR
i02_3156	CDS	3175803	3176495	-	ygeA	putative racemase
i02_3157	CDS	3176624	3178171	-	araE	arabinose-proton symporter
i02_3158	CDS	3178357	3179118	-	kduD	2-deoxy-D-gluconate 3-dehydrogenase
i02_3159	CDS	3179148	3179984	-	kduI	5-keto-4-deoxyuronate isomerase
i02_3160	CDS	3180271	3181452	-	yqeF	acetyl-CoA acetyltransferase
i02_3161	CDS	3181707	3182936	+	yqeG	putative transport protein YqeG
i02_3162	CDS	3183405	3184184	-	ygeR	lipoprotein ygeR precursor
i02_3163	CDS	3184574	3186871	+	/	xanthine dehydrogenase subunit XdhA
i02_3164	CDS	3186882	3187760	+	ygeT	xanthine dehydrogenase subunit XdhB
i02_3165	CDS	3187757	3188236	+	/	xanthine dehydrogenase subunit XdhC
i02_3166	CDS	3188276	3190054	-	ygeV	putative sigma-54-dependent transcriptional
i02_3167	CDS	3190530	3191720	+	ygeW	aspartate/ornithine carbamoyltransferase family
i02_3168	CDS	3191778	3192974	+	ygeX	diaminopropionate ammonia-lyase
i02_3169	CDS	3193032	3194243	+	ygeY	peptidase
i02_3170	CDS	3194297	3195682	+	ygeZ	phenylhydantoinase
i02_3171	CDS	3195730	3196662	+	yqeA	carbamate kinase
i02_3172	CDS	3196703	3198328	-	yqeB	hypothetical protein
i02_3173	CDS	3198376	3199146	-	yqeC	hypothetical protein
i02_3174	CDS	3199250	3199828	+	ygfJ	hypothetical protein
i02_3175	CDS	3200150	3203248	+	ygfK	putative selenate reductase subunit YgfK
i02_3176	CDS	3203251	3204579	+	/	putative chlorohydrolase/aminohydrolase
i02_3177	CDS	3204630	3205409	+	ygfM	putative selenate reductase subunit YgfM
i02_3178	CDS	3205406	3208276	+	/	putative selenate reductase subunit YgfN
i02_3179	CDS	3208384	3209841	+	ygfO	putative purine permease ygfO
i02_3180	CDS	3209736	3211175	+	ygfP	guanine deaminase
i02_3181	CDS	3211211	3212578	+	/	hypothetical protein
i02_3182	CDS	3212614	3213105	-	ygfS	putative electron transport protein ygfS
i02_3183	CDS	3213102	3215036	-	ygfT	putative oxidoreductase Fe-S binding subunit

Locus_tag	Type	start	End	+/-[a]	Gene[b]	Product
i02_3184	CDS	3215328	3216905	+	ygfU	putative purine permease ygfU
i02_3185	CDS	3216907	3217032	+	/	hypothetical protein
i02_3186	CDS	3217155	3217703	+	/	isopentenyl-diphosphate delta-isomerase
i02_3187	CDS	3217746	3219263	-	lysS	lysyl-tRNA synthetase
i02_3188	CDS	3217863	3219278	+	/	hypothetical protein
i02_3189	CDS	3219273	3220154	-	prfB	peptide chain release factor 2
i02_3190	CDS	3220155	3220310	+	/	conserved hypothetical protein
i02_3191	CDS	3220294	3220371	-	prfB	peptide chain release factor 2
i02_3192	CDS	3220462	3222195	-	recJ	ssDNA exonuclease RecJ
i02_3193	CDS	3222201	3222911	-	dsbC	thiol:disulfide interchange protein DsbC
i02_3194	CDS	3222936	3223832	-	xerD	site-specific tyrosine recombinase XerD
i02_3195	CDS	3223944	3224465	+	fldB	flavodoxin FldB
i02_3196	CDS	3224505	3224912	-	ygfX	hypothetical protein
i02_3197	CDS	3224893	3225159	-	ygfY	hypothetical protein
i02_3198	CDS	3225134	3225325	+	/	hypothetical protein
i02_3199	CDS	3225402	3226382	+	ygfZ	putative global regulator
i02_3200	CDS	3226459	3227118	-	yqfA	hypothetical protein
i02_3201	CDS	3227282	3227593	-	yqfB	hypothetical protein
i02_3202	CDS	3227614	3229071	+	bglA	6-phospho-beta-glucosidase BglA
i02_3203	CDS	3229237	3232110	-	gcvP	glycine dehydrogenase
i02_3204	CDS	3232228	3232620	-	gcvH	glycine cleavage system protein H
i02_3205	CDS	3232641	3233735	-	gcvT	glycine cleavage system aminomethyltransferase
i02_3206	CDS	3233743	3233898	-	/	hypothetical protein
i02_3207	CDS	3233911	3234060	-	/	hypothetical protein
i02_3208	CDS	3234183	3235385	-	visC	hypothetical protein
i02_3209	CDS	3235408	3236586	-	ubiH	2-octaprenyl-6-methoxyphenyl hydroxylase
i02_3210	CDS	3236583	3237908	-	pepP	proline aminopeptidase P II
i02_3211	CDS	3237934	3238512	-	ygfB	hypothetical protein
i02_3212	CDS	3238680	3239009	+	ygfE	Z-ring-associated protein
i02_3213	CDS	3239309	3239857	+	ygfA	putative ligase
i02_3214	CDS	3240247	3241479	-	serA	D-3-phosphoglycerate dehydrogenase
i02_3215	CDS	3241735	3242466	-	rpiA	ribose-5-phosphate isomerase A
i02_3216	CDS	3242450	3242728	-	yqfE	hypothetical protein
i02_3217	CDS	3242732	3242845	-	/	conserved hypothetical protein
i02_3218	CDS	3242821	3243714	+	iciA	chromosome replication initiation inhibitor
i02_3219	CDS	3243917	3244972	+	/	hypothetical protein
i02_3220	CDS	3244969	3245865	-	ygfI	putative transcriptional regulator YgfI
i02_3221	CDS	3246023	3246763	-	yggE	hypothetical protein
i02_3222	CDS	3246856	3247491	-	yggA	arginine exporter protein
i02_3223	CDS	3247630	3248490	-	yggB	mechanosensitive channel MscS
i02_3224	CDS	3248681	3249760	-	fba	fructose-bisphosphate aldolase
i02_3225	CDS	3249974	3251194	-	pgk	phosphoglycerate kinase
i02_3226	CDS	3251187	3252206	-	epd	erythrose 4-phosphate dehydrogenase
i02_3227	CDS	3252578	3253006	+	/	hypothetical protein
i02_3228	CDS	3253033	3253611	+	/	hypothetical protein
i02_3229	CDS	3253612	3254319	+	/	hypothetical protein
i02_3230	CDS	3254307	3254984	+	/	ABC transporter ATP-binding protein
i02_3231	CDS	3254945	3255655	+	/	ABC transporter ATP-binding protein
i02_3232	CDS	3255627	3256340	-	frcK	putative fructose transport system kinase
i02_3233	CDS	3256337	3256846	-	yggD	putative DNA-binding transcriptional regulator
i02_3234	CDS	3256995	3257183	-	/	hypothetical protein
i02_3235	CDS	3257180	3258457	-	/	putative oxidoreductase
i02_3236	CDS	3258472	3259860	-	cmtA	PTS system, mannitol (Cryptic)-specific IIBC
i02_3237	CDS	3259888	3260331	-	cmtB	putative mannitol phosphotransferase subunit
i02_3238	CDS	3260648	3261616	+	/	hypothetical protein
i02_3239	CDS	3261835	3263826	-	tktA	transketolase
i02_3240	CDS	3263885	3264862	+	yggG	putative metalloprotease yggG
i02_3241	CDS	3265066	3265986	-	speB	agmatinase

Locus_tag	Type	start	End	+/-[a]	Gene[b]	Product
i02_3242	CDS	3266122	3266853	-	/	hypothetical protein
i02_3243	CDS	3266999	3268987	-	speA	arginine decarboxylase
i02_3244	CDS	3268984	3269130	-	yqgB	hypothetical protein
i02_3245	CDS	3269463	3269729	-	yqgD	hypothetical protein
i02_3246	CDS	3269770	3270924	+	metK	S-adenosylmethionine synthetase
i02_3247	CDS	3271348	3272754	+	galP	galactose-proton symporter
i02_3248	CDS	3272777	3273328	+	sprT	hypothetical protein
i02_3249	CDS	3273420	3274130	+	endA	endonuclease I precursor
i02_3250	CDS	3274210	3274941	+	yggJ	16S ribosomal RNA methyltransferase RsmE
i02_3251	CDS	3274954	3275904	+	gshB	glutathione synthetase
i02_3252	CDS	3276013	3276576	+	yqgE	hypothetical protein
i02_3253	CDS	3276576	3276992	+	yqgF	Holliday junction resolvase-like protein
i02_3254	CDS	3277166	3278221	-	yggR	hypothetical protein
i02_3255	CDS	3278164	3278868	+	yggS	hypothetical protein
i02_3256	CDS	3278886	3279452	+	yggT	hypothetical protein
i02_3257	CDS	3279449	3279739	+	yggU	hypothetical protein
i02_3258	CDS	3279747	3280340	+	yggV	putative deoxyribonucleotide triphosphate
i02_3259	CDS	3280333	3281469	+	yggW	coproporphyrinogen III oxidase
i02_3260	CDS	3281538	3282545	-	yggM	hypothetical protein
i02_3261	CDS	3282662	3283708	-	ansB	L-asparaginase II
i02_3262	CDS	3283884	3284603	-	yggN	hypothetical protein
i02_3263	CDS	3284624	3284764	-	/	hypothetical protein
i02_3264	CDS	3284787	3285113	-	yggL	hypothetical protein
i02_3265	CDS	3285113	3285832	-	trmB	tRNA (guanine-N(7)-)-methyltransferase
i02_3266	CDS	3285963	3287045	+	mutY	adenine DNA glycosylase
i02_3267	CDS	3287073	3287348	+	yggX	hypothetical protein
i02_3268	CDS	3287410	3288492	+	mltC	murein transglycosylase C
i02_3269	CDS	3288646	3289950	+	nupG	nucleoside permease nupG
i02_3270	CDS	3289999	3292134	-	speC	ornithine decarboxylase
i02_3271	CDS	3292215	3292418	-	/	hypothetical protein
i02_3272	CDS	3292533	3293240	+	yqgA	hypothetical protein
i02_3273	CDS	3293619	3294884	+	/	prophage P4 integrase
i02_3274	CDS	3295110	3296183	+	/	ShiA-like protein
i02_3275	CDS	3296326	3296556	+	/	hypothetical protein
i02_3276	CDS	3296713	3296988	+	insA	InsA protein
i02_3277	CDS	3296907	3297410	+	insb	InsB protein
i02_3278	CDS	3297877	3298428	-	papX	PapX protein
i02_3279	CDS	3298542	3298667	+	/	hypothetical protein
i02_3280	CDS	3298700	3299203	-	/	IS1 protein InsB
i02_3281	CDS	3299122	3299397	-	/	insertion element IS1 1/2/3/5/6 protein insA
i02_3282	pseudo	3299426	3300015	-	/	major pilin subunit PapA
i02_3284	CDS	3300408	3300515	-	/	PapB-like protein
i02_3286	CDS	3300920	3301153	+	papI	PapI protein
i02_3287	pseudo	3301717	3302607	-	/	transposase IS629
i02_3288	CDS	3303203	3303331	-	/	hypothetical protein
i02_3289	CDS	3303670	3304374	-	/	transposase
i02_3290	CDS	3304377	3304538	-	/	hypothetical protein
i02_3291	CDS	3304968	3305228	+	/	hypothetical protein
i02_3292	CDS	3305270	3305830	+	/	hypothetical protein
i02_3293	CDS	3305840	3306298	+	/	hypothetical protein
i02_3294	CDS	3306962	3307372	+	/	transposase insC
i02_3294a	CDS	3307330	3308235	+	/	insertion element IS2 transposase InsD
i02_3295	CDS	3308735	3310825	+	/	bifunctional enterobactin receptor/adhesin
i02_3296	CDS	3311059	3311283	+	/	hypothetical protein
i02_3297	CDS	3311609	3311929	+	/	hypothetical protein
i02_3298	CDS	3312017	3312208	+	/	hypothetical protein
i02_3299	CDS	3312205	3312588	+	/	hypothetical protein
i02_3300	CDS	3312592	3312807	+	/	hypothetical protein

Locus_tag	Type	start	End	+/-[a]	Gene[b]	Product
i02_3301	CDS	3312949	3313179	-	/	conserved hypothetical protein
i02_3302	CDS	3313310	3313582	-	/	putative transposase
i02_3303	CDS	3314057	3314260	+	/	conserved hypothetical protein
i02_3304	CDS	3314270	3314692	-	/	IS1353 transposase family protein
i02_3305	CDS	3314765	3315304	-	/	transposase
i02_3307	CDS	3315341	3315802	-	/	transposase IS3 family
i02_3308	CDS	3316218	3316673	-	/	hypothetical protein
i02_3309	CDS	3316685	3317503	-	/	transposase insF
i02_3310	CDS	3317588	3318739	-	insI	transposase insI
i02_3311	CDS	3318696	3319064	-	/	hypothetical protein
i02_3312	CDS	3319116	3319406	-	/	hypothetical protein
i02_3313	CDS	3319336	3323235	-	sat	Aecreted auto transpoter toxin
i02_3315	CDS	3323473	3323616	+	/	hypothetical protein
i02_3316	CDS	3324167	3326392	-	iutA	IutA protein
i02_3317	CDS	3326450	3327727	-	iucD	IucD protein
i02_3318	CDS	3327724	3329466	-	iucC	IucC protein
i02_3319	CDS	3329466	3330413	-	iucB	IucB protein
i02_3320	CDS	3330414	3332156	-	iucA	IucA protein
i02_3321	CDS	3332274	3333467	+	shiF	shiF protein
i02_3323	CDS	3333580	3334485	-	/	insertion element IS2 transposase InsD
i02_3324	CDS	3334443	3334853	-	/	transposase insC
i02_3325	CDS	3334960	3335949	-	/	hypothetical protein
i02_3326	CDS	3336039	3336422	-	/	hypothetical protein
i02_3327	CDS	3336698	3336829	-	/	hypothetical protein
i02_3328	CDS	3336816	3337751	-	/	hypothetical protein
i02_3329	CDS	3337801	3338919	-	/	hypothetical protein
i02_3330	CDS	3338923	3339663	-	/	hypothetical protein
i02_3331	CDS	3339679	3340488	-	/	hypothetical protein
i02_3332	CDS	3340513	3341985	-	/	putative sialic acid transporter
i02_3333	CDS	3342034	3342909	-	/	N-acetylmannosamine kinase
i02_3334	CDS	3342943	3343860	-	/	N-acetylneuraminate lyase
i02_3335	CDS	3344584	3344946	+	/	hypothetical protein
i02_3336	CDS	3344943	3345293	+	/	hypothetical protein
i02_3337	CDS	3345324	3346424	+	/	hypothetical protein
i02_3338	CDS	3346421	3347959	+	/	hypothetical protein
i02_3339	CDS	3347875	3348069	-	/	hypothetical protein
i02_3340	CDS	3348180	3348569	-	/	hypothetical protein
i02_3341	CDS	3348730	3349299	-	/	hypothetical protein
i02_3342	CDS	3349501	3349695	+	/	predicted protein
i02_3343	CDS	3350028	3350252	-	/	hemolysin expression modulating protein
i02_3344	CDS	3350776	3350898	-	/	hypothetical protein
i02_3345	CDS	3351071	3351331	+	/	hypothetical protein
i02_3346	CDS	3351390	3351989	+	/	hypothetical protein
i02_3347	CDS	3352110	3353618	-	/	hypothetical protein
i02_3348	CDS	3353795	3354016	-	/	hypothetical protein
i02_3349	CDS	3354031	3354153	-	/	conserved hypothetical protein
i02_3350	CDS	3354932	3355804	+	/	hypothetical protein
i02_3351	CDS	3356132	3359260	+	/	antigen 43 precursor
i02_3352	CDS	3360558	3361013	+	/	hypothetical protein
i02_3353	CDS	3361092	3361325	+	/	hypothetical protein
i02_3354	CDS	3361426	3362244	+	/	hypothetical protein
i02_3355	CDS	3362353	3363264	-	/	hypothetical protein
i02_3356	CDS	3362785	3363276	+	/	putative radC-like protein yeeS
i02_3357	CDS	3363273	3364001	-	/	hypothetical protein
i02_3358	CDS	3363579	3364223	+	/	hypothetical protein
i02_3359	pseudo	3364246	3364809	-	/	hypothetical protein
i02_3360	CDS	3364730	3365107	+	/	hypothetical protein
i02_3361	CDS	3365104	3365592	+	/	hypothetical protein

Locus_tag	Type	start	End	+/-[a]	Gene[b]	Product
i02_3362	CDS	3365891	3366736	+	/	hypothetical protein
i02_3363	CDS	3366805	3367200	+	/	hypothetical protein
i02_3364	CDS	3367193	3367933	+	/	hypothetical protein
i02_3365	CDS	3367926	3368126	+	/	hypothetical protein
i02_3366	CDS	3368558	3368713	+	/	hypothetical protein
i02_3367	CDS	3368881	3369024	-	/	hypothetical protein
i02_3368	CDS	3369488	3370507	+	/	hypothetical protein
i02_3369	CDS	3370579	3371727	+	kpsE	KpsE protein
i02_3370	CDS	3371748	3373427	+	kpsD	KpsD protein
i02_3371	CDS	3373437	3374177	+	/	3-deoxy-manno-octulosonate cytidylyltransferase
i02_3372	CDS	3374174	3376201	+	kpsC	KpsC protein
i02_3373	CDS	3376236	3377405	+	kpsS	KpsS protein
i02_3373a	CDS	3377747	3378937	-	kfiD	UDP-glucose 6-dehydrogenase
i02_3374	CDS	3378964	3380526	-	/	glycosyltransferase KfiC
i02_3375	CDS	3381222	3381524	+	/	conserved hypothetical protein
i02_3376	CDS	3381818	3383506	-	kfiB	kfiB protein
i02_3377	CDS	3385642	3386316	-	kpsT	KpsT protein
i02_3378	CDS	3386313	3387089	-	kpsM	KpsM protein
i02_3379	CDS	3388147	3388683	-	yghD	putative general secretion pathway protein YghD
i02_3380	CDS	3388685	3389545	-	gspL	GspL-like protein
i02_3381	CDS	3389860	3390837	-	/	hypothetical protein
i02_3382	CDS	3390834	3391439	-	gspJ	putative type II secretion protein GspJ
i02_3383	CDS	3391436	3391807	-	gspI	putative type II secretion protein GspI
i02_3384	CDS	3391804	3392367	-	gspH	putative type II secretion protein GspH
i02_3385	CDS	3392371	3392826	-	gspG	putative type II secretion protein GspG
i02_3386	CDS	3392843	3394066	-	gspF	putative type II secretion protein GspF
i02_3387	CDS	3394066	3395559	-	gspE	putative type II secretion protein GspE
i02_3388	CDS	3395559	3397619	-	gspD	putative type II secretion protein GspD
i02_3389	CDS	3397649	3398491	-	gspC	putative type II secretion protein GspC
i02_3390	CDS	3398626	3399036	-	/	hypothetical protein
i02_3391	CDS	3399102	3399992	-	pppA	putative prepilin peptidase A
i02_3392	CDS	3400041	3404597	-	yghJ	putative lipoprotein AcfD-like precursor
i02_3393	CDS	3404748	3404948	+	/	hypothetical protein
i02_3394	CDS	3404945	3405067	-	/	conserved hypothetical protein
i02_3395	CDS	3405082	3406764	-	yghK	glycolate transporter
i02_3396	CDS	3407119	3409290	-	glcB	malate synthase G
i02_3397	CDS	3409312	3409734	-	glcG	hypothetical protein
i02_3398	CDS	3409721	3410944	-	pir	glycolate oxidase iron-sulfur subunit
i02_3399	CDS	3410955	3412007	-	glcE	glycolate oxidase FAD binding subunit
i02_3400	CDS	3412007	3413506	-	glcD	glycolate oxidase subunit GlcD
i02_3401	CDS	3413697	3414521	+	glcC	DNA-binding transcriptional regulator GlcC
i02_3402	CDS	3414528	3415679	-	yghO	hypothetical protein
i02_3403	CDS	3416032	3417762	+	/	acyl-CoA synthetase
i02_3404	CDS	3417549	3418673	+	/	hypothetical protein
i02_3405	CDS	3418705	3418953	+	/	hypothetical protein
i02_3406	CDS	3418953	3420125	+	/	hypothetical protein
i02_3407	CDS	3420160	3421320	-	/	hypothetical protein
i02_3408	CDS	3421236	3422306	-	/	hypothetical protein
i02_3409	CDS	3422337	3422897	-	/	hypothetical protein
i02_3410	CDS	3422897	3423733	-	/	hypothetical protein
i02_3411	CDS	3423733	3425082	-	yghQ	hypothetical protein
i02_3412	CDS	3425128	3425898	-	yghR	ATP-binding protein yghR
i02_3413	CDS	3425918	3426631	-	yghS	ATP-binding protein yghS
i02_3414	CDS	3426806	3427498	+	yghT	ATP-binding protein yghT
i02_3415	CDS	3427547	3429046	-	pitB	inorganic phosphate transporter
i02_3416	CDS	3429338	3431200	-	gsp	bifunctional glutathionylspermidine
i02_3417	CDS	3431354	3432268	+	yghU	putative glutathione S-transferase YghU
i02_3418	CDS	3432410	3432658	-	hybG	hydrogenase 2 accessory protein HypG

Locus_tag	Type	start	End	+/-[a]	Gene[b]	Product
i02_3419	CDS	3432671	3433066	-	hypA	hydrogenase nickel incorporation protein HybF
i02_3420	CDS	3433005	3433493	-	hybE	hydrogenase 2-specific chaperone
i02_3421	CDS	3433486	3433980	-	hybD	hydrogenase 2 maturation endopeptidase
i02_3422	CDS	3433980	3435683	-	hybC	hydrogenase 2 large subunit
i02_3423	CDS	3435680	3436858	-	hybB	putative hydrogenase 2 b cytochrome subunit
i02_3424	CDS	3436848	3437834	-	hybA	hydrogenase 2 protein HybA
i02_3425	CDS	3437837	3438955	-	/	hydrogenase 2 small subunit
i02_3426	CDS	3438939	3439067	+	/	hypothetical protein
i02_3427	CDS	3439144	3439431	-	yghW	hypothetical protein
i02_3428	CDS	3439550	3440476	-	/	hypothetical protein
i02_3429	CDS	3440594	3441634	+	yghZ	aldo-keto reductase
i02_3430	CDS	3441674	3442198	-	yqhA	hypothetical protein
i02_3431	CDS	3442359	3443243	+	yghA	oxidoreductase
i02_3432	CDS	3443282	3443707	-	exbD	biopolymer transport protein ExbD
i02_3433	CDS	3443714	3444448	-	exbB	biopolymer transport protein ExbB
i02_3434	CDS	3444565	3445887	+	metC	cystathionine beta-lyase
i02_3435	CDS	3446027	3446686	+	yghB	hypothetical protein
i02_3436	CDS	3446726	3447853	-	yqhC	putative transcriptional regulator YqhC
i02_3437	CDS	3447819	3448982	+	yqhD	oxidoreductase yqhD
i02_3438	CDS	3449087	3449914	+	dkgA	2,5-diketo-D-gluconate reductase A
i02_3439	CDS	3450114	3451040	+	yqhG	hypothetical protein
i02_3440	CDS	3451091	3451348	+	yqhH	putative outer membrane lipoprotein
i02_3441	CDS	3451390	3453609	-	/	hypothetical protein
i02_3442	CDS	3453861	3454610	+	/	putative regulator
i02_3443	CDS	3454897	3456405	+	/	oxidoreductase ydfI
i02_3444	CDS	3456402	3457418	+	/	hypothetical protein
i02_3445	CDS	3457429	3458439	+	/	ureidoglycolate dehydrogenase
i02_3446	CDS	3458512	3459495	+	/	putative c4-dicarboxylate transport system
i02_3447	CDS	3459537	3460019	+	/	hypothetical protein
i02_3448	CDS	3459988	3461334	+	ygiK	c4-dicarboxylate permease
i02_3449	CDS	3461405	3462817	-	sufI	repressor protein for FtsI
i02_3450	CDS	3462892	3463629	-	plsC	1-acyl-sn-glycerol-3-phosphate acyltransferase
i02_3451	CDS	3463678	3463833	-	/	hypothetical protein
i02_3452	CDS	3463864	3466122	-	parC	DNA topoisomerase IV subunit A
i02_3453	CDS	3466260	3467930	-	/	putative binding protein
i02_3454	CDS	3467976	3468458	-	ygiV	hypothetical protein
i02_3455	CDS	3468511	3468960	-	ygiW	hypothetical protein
i02_3456	CDS	3469055	3469714	+	ygiX	DNA-binding transcriptional regulator QseB
i02_3457	CDS	3469711	3471060	+	ygiY	sensor protein QseC
i02_3458	CDS	3471106	3471438	-	/	hypothetical protein
i02_3459	CDS	3471445	3472182	-	/	hypothetical protein
i02_3460	CDS	3472158	3472637	-	/	hypothetical protein
i02_3461	CDS	3472887	3473468	+	mdaB	modulator of drug activity B
i02_3462	CDS	3473499	3473813	+	ygiN	hypothetical protein
i02_3463	CDS	3473858	3474745	-	/	hypothetical protein
i02_3464	CDS	3474742	3475752	-	/	iron ABC transporter substrate-binding protein
i02_3465	CDS	3475700	3476749	-	/	iron ABC transporter permease
i02_3466	CDS	3476746	3477729	-	/	iron ABC transporter permease
i02_3467	CDS	3477726	3478535	-	/	ferric enterobactin transport ATP-binding
i02_3468	CDS	3478615	3478734	-	/	hypothetical protein
i02_3469	CDS	3478909	3481050	+	/	putative iron compound receptor
i02_3470	CDS	3481114	3483006	-	parE	DNA topoisomerase IV subunit B
i02_3471	CDS	3483035	3483616	-	yqiA	esterase YqiA
i02_3472	CDS	3483616	3484443	-	icc	cyclic 3',5'-adenosine monophosphate
i02_3473	CDS	3484468	3484890	-	yqiB	hypothetical protein
i02_3474	CDS	3484891	3485520	-	nudF	ADP-ribose pyrophosphatase NudF
i02_3475	CDS	3485719	3487206	+	tolC	outer membrane channel protein
i02_3476	CDS	3487206	3487466	+	ygiA	hypothetical protein

Locus_tag	Type	start	End	+/-[a]	Gene[b]	Product
i02_3477	CDS	3487321	3488025	+	ygiB	hypothetical protein
i02_3478	CDS	3488031	3489191	+	ygiC	hypothetical protein
i02_3479	CDS	3489676	3491472	+	asst	arylsulfate sulfotransferase
i02_3480	CDS	3491490	3492158	+	/	putative disulfide isomerase
i02_3481	CDS	3492173	3492844	+	/	putative disulfide oxidoreductase
i02_3482	CDS	3492890	3493705	-	ygiD	hypothetical protein
i02_3483	CDS	3493779	3494594	+	ygiE	zinc transporter ZupT
i02_3484	CDS	3494656	3494841	-	/	hypothetical protein
i02_3485	CDS	3495397	3495960	+	ygiL	putative fimbrial protein
i02_3486	CDS	3496047	3498569	+	yqiG	outer membrane usher protein yqiG precursor
i02_3487	CDS	3498576	3499325	+	yqiH	fimbrial chaperone yqiH precursor
i02_3488	CDS	3499322	3500371	+	/	hypothetical protein
i02_3489	CDS	3500425	3501078	-	ribB	3,4-dihydroxy-2-butanone 4-phosphate synthase
i02_3490	CDS	3501392	3501751	+	yqiC	hypothetical protein
i02_3491	CDS	3501786	3501992	-	glgS	glycogen synthesis protein GlgS
i02_3492	CDS	3502256	3502885	+	yqiJ	hypothetical protein
i02_3493	CDS	3502912	3504573	+	yqiK	hypothetical protein
i02_3494	CDS	3504992	3506425	-	/	bifunctional heptose 7-phosphate kinase/heptose
i02_3495	CDS	3506473	3509385	-	glnE	bifunctional glutamine-synthetase
i02_3496	CDS	3509336	3510664	-	ygiF	hypothetical protein
i02_3497	CDS	3510707	3511159	-	/	putative transposase
i02_3498	CDS	3511172	3512377	+	ydcM	hypothetical protein
i02_3499	CDS	3512495	3512680	-	/	hypothetical protein
i02_3500	CDS	3512720	3513340	+	ygiM	putative signal transduction protein
i02_3501	CDS	3513404	3514642	+	cca	multifunctional tRNA nucleotidyl
i02_3502	CDS	3514722	3515546	-	uppP	undecaprenyl pyrophosphate phosphatase
i02_3503	CDS	3515634	3516005	-	folB	bifunctional dihydroneopterin
i02_3504	CDS	3516107	3516724	+	ygiH	putative glycerol-3-phosphate acyltransferase
i02_3505	CDS	3516737	3517723	-	ygiP	transcriptional activator TtdR
i02_3506	CDS	3517700	3517822	+	/	hypothetical protein
i02_3507	CDS	3517876	3518787	+	ttdA	tartrate dehydratase subunit alpha
i02_3508	CDS	3518784	3519389	+	ttdB	L(+)-tartrate dehydratase subunit beta
i02_3509	CDS	3519437	3520900	+	ygjE	putative tartrate carrier/transporter
i02_3510	CDS	3520943	3521956	-	ygjD	putative DNA-binding/iron metalloprotein
i02_3511	CDS	3522194	3522409	+	rpsU	30S ribosomal protein S21
i02_3512	CDS	3522520	3524265	+	dnaG	DNA primase
i02_3513	CDS	3524279	3524401	+	/	hypothetical protein
i02_3514	CDS	3524460	3526301	+	/	hypothetical protein
i02_3515	CDS	3524571	3526355	-	/	hypothetical protein
i02_3516	CDS	3526379	3526885	-	ygjF	G/U mismatch-specific DNA glycosylase
i02_3517	CDS	3527139	3527918	-	yqjH	hypothetical protein
i02_3518	CDS	3528191	3528814	+	yqjI	hypothetical protein
i02_3519	CDS	3528968	3530488	-	aer	aerotaxis receptor
i02_3520	CDS	3530795	3532285	+	ygjG	putrescine--2-oxoglutarate aminotransferase
i02_3521	CDS	3532269	3532679	+	/	hypothetical protein
i02_3522	CDS	3532766	3532948	+	/	hypothetical protein
i02_3523	CDS	3532878	3533861	+	ebgR	DNA-binding transcriptional repressor EbgR
i02_3524	CDS	3534009	3537137	+	ebgA	cryptic beta-D-galactosidase subunit alpha
i02_3525	CDS	3537134	3537583	+	ebgC	cryptic beta-D-galactosidase subunit beta
i02_3527	CDS	3537646	3539079	+	ygjI	putative transporter YgjI
i02_3528	CDS	3539213	3540283	+	ygjJ	hypothetical protein
i02_3529	CDS	3540300	3542651	+	ygjK	putative glycosyl hydrolase
i02_3530	CDS	3542877	3544895	+	ygjL	2,4-dienoyl-CoA reductase
i02_3531	CDS	3544940	3545356	-	/	hypothetical protein
i02_3532	CDS	3545353	3545523	-	/	hypothetical protein
i02_3533	CDS	3545627	3546763	-	ygjO	putative ribosomal RNA small subunit
i02_3534	CDS	3546812	3547351	+	ygjP	hypothetical protein
i02_3535	CDS	3547419	3548120	+	ygjQ	hypothetical protein

Locus_tag	Type	start	End	+/-[a]	Gene[b]	Product
i02_3536	CDS	3548181	3549185	+	ygjR	oxidoreductase ygjR
i02_3537	CDS	3549469	3550434	+	ygjT	hypothetical protein
i02_3538	CDS	3550758	3552002	+	ygjU	serine/threonine transporter SstT
i02_3539	CDS	3552006	3552557	-	ygjV	hypothetical protein
i02_3540	CDS	3552640	3554127	-	uxaA	Altronate hydrolase
i02_3541	CDS	3554142	3555554	-	uxaC	glucuronate isomerase
i02_3542	CDS	3555917	3557335	+	exuT	hexuronate transporter
i02_3543	CDS	3557450	3558241	+	exuR	DNA-binding transcriptional repressor ExuR
i02_3544	CDS	3558586	3559248	+	yqjA	hypothetical protein
i02_3545	CDS	3559252	3559635	+	yqjB	hypothetical protein
i02_3546	CDS	3559782	3560150	+	yqjC	hypothetical protein
i02_3547	CDS	3560188	3560493	+	yqjD	hypothetical protein
i02_3548	CDS	3560427	3560900	+	yqjE	hypothetical protein
i02_3549	CDS	3560848	3561189	+	yqjK	hypothetical protein
i02_3550	CDS	3561286	3561768	+	yqjF	hypothetical protein
i02_3551	CDS	3561838	3562824	+	yqjG	putative glutathione S-transferase
i02_3552	CDS	3563086	3563475	+	yhaH	hypothetical protein
i02_3553	CDS	3563509	3564405	-	yhaJ	putative transcriptional regulator YhaJ
i02_3554	CDS	3564510	3565211	+	yhaK	hypothetical protein
i02_3555	CDS	3565228	3565398	+	yhaL	hypothetical protein
i02_3556	CDS	3565532	3566842	-	/	hypothetical protein
i02_3557	CDS	3566870	3568201	-	/	putative amino acid permease
i02_3558	CDS	3568479	3569849	-	/	L-serine dehydratase 1
i02_3559	CDS	3569915	3570367	-	yhaR	hypothetical protein
i02_3560	CDS	3570318	3572612	-	tdcE	keto-acid formate acetyltransferase
i02_3561	CDS	3572646	3573866	-	tdcD	propionate/acetate kinase
i02_3562	CDS	3573880	3575211	-	tdcC	threonine/serine transporter TdcC
i02_3563	CDS	3575233	3576222	-	tdcB	threonine dehydratase
i02_3564	CDS	3576321	3577259	-	tdcA	DNA-binding transcriptional activator TdcA
i02_3565	CDS	3577448	3577792	+	tdcR	DNA-binding transcriptional activator TdcR
i02_3566	CDS	3578018	3578146	+	/	hypothetical protein
i02_3567	CDS	3578306	3578830	+	/	hypothetical protein
i02_3568	CDS	3578758	3579984	-	yhaD	glycerate kinase I
i02_3569	CDS	3580000	3580899	-	garR	tartronate semialdehyde reductase
i02_3570	CDS	3580920	3581690	-	yhaF	alpha-dehydro-beta-deoxy-D-glucarate aldolase
i02_3571	CDS	3581706	3583040	-	yhaU	galactarate transporter
i02_3572	CDS	3583249	3583368	+	/	hypothetical protein
i02_3573	CDS	3583415	3584986	+	yhaG	D-galactarate dehydratase
i02_3574	CDS	3585135	3585470	+	sohA	putative regulator PrlF
i02_3575	CDS	3585470	3585934	+	yhaV	hypothetical protein
i02_3576	CDS	3585989	3586798	-	agaR	DNA-binding transcriptional regulator AgaR
i02_3577	CDS	3587047	3588327	+	agaZ	putative tagatose 6-phosphate kinase agaZ
i02_3578	CDS	3588314	3588823	+	agaV	N-acetylgalactosamine-specific PTS system
i02_3579	CDS	3588834	3589613	+	/	putative phosphotransferase system enzyme
i02_3580	CDS	3589597	3590481	+	/	PTS system, N-acetylgalactosamine-specific IID
i02_3581	CDS	3590499	3590933	+	/	putative phosphotransferase system enzyme
i02_3582	CDS	3590909	3592063	+	/	putative N-acetylgalctosamine-6-phosphate
i02_3583	CDS	3592412	3593566	+	agaS	putative tagatose-6-phosphate aldose/ketose isomerase (AgaS)
i02_3584	CDS	3593579	3594439	+	kbaY	tagatose-bisphosphate aldolase
i02_3585	CDS	3594606	3595082	+	agaB	N-acetylgalactosamine-specific PTS system
i02_3586	CDS	3595121	3595924	+	agaC	N-acetylgalactosamine-specific PTS system
i02_3587	CDS	3595833	3596705	+	agaD	N-acetylgalactosamine-specific PTS system
i02_3588	CDS	3596706	3597473	+	agaI	galactosamine-6-phosphate isomerase
i02_3589	CDS	3597523	3598386	-	yraL	hypothetical protein
i02_3590	CDS	3598326	3600488	+	yraM	hypothetical protein
i02_3591	CDS	3600446	3600841	+	yraN	hypothetical protein
i02_3593	CDS	3600861	3601451	+	yraO	DnaA initiator-associating protein DiaA

Locus_tag	Type	start	End	+/-[a]	Gene[b]	Product
i02_3594	CDS	3601461	3602036	+	yraP	hypothetical protein
i02_3595	CDS	3602149	3603189	-	yraQ	hypothetical protein
i02_3596	CDS	3603262	3603906	-	yraR	hypothetical protein
i02_3597	CDS	3603939	3604361	-	yhbP	hypothetical protein
i02_3598	CDS	3604412	3604714	+	yhbQ	GIY-YIG nuclease superfamily protein
i02_3599	CDS	3604701	3605204	-	yhbS	acetyltransferase YhbS
i02_3600	CDS	3605198	3605746	-	yhbT	hypothetical protein
i02_3601	CDS	3605904	3606926	+	yhbU	putative protease yhbU precursor
i02_3602	CDS	3606917	3607813	+	yhbV	hypothetical protein
i02_3603	CDS	3607894	3608901	+	yhbW	hypothetical protein
i02_3604	CDS	3609020	3610264	-	mtr	tryptophan permease
i02_3605	CDS	3610280	3610417	+	/	hypothetical protein
i02_3606	CDS	3610418	3612358	-	deaD	ATP-dependent RNA helicase DeaD
i02_3607	CDS	3612487	3613371	-	yhbM	lipoprotein NlpI
i02_3608	CDS	3613480	3615702	-	pnp	polynucleotide phosphorylase/polyadenylase
i02_3609	CDS	3615862	3616131	-	rpsO	30S ribosomal protein S15
i02_3610	CDS	3616280	3617224	-	truB	tRNA pseudouridine synthase B
i02_3611	CDS	3617224	3617625	-	rbfA	ribosome-binding factor A
i02_3612	CDS	3617789	3620461	-	infB	translation initiation factor IF-2
i02_3613	CDS	3620318	3621787	+	/	hypothetical protein
i02_3614	CDS	3620486	3621973	-	nusA	transcription elongation factor NusA
i02_3615	CDS	3622001	3622453	-	yhbC	hypothetical protein
i02_3616	CDS	3623084	3624427	+	argG	argininosuccinate synthase
i02_3617	CDS	3624435	3626093	-	yhbX	Outer-membrane protein yhbX precursor
i02_3618	CDS	3626618	3626950	-	secG	preprotein translocase subunit SecG
i02_3619	CDS	3627178	3628515	-	glmM	phosphoglucosamine mutase
i02_3620	CDS	3628508	3629356	-	folP	dihydropteroate synthase
i02_3621	CDS	3629446	3631380	-	hflB	ATP-dependent metalloprotease
i02_3622	CDS	3631349	3631483	+	/	hypothetical protein
i02_3623	CDS	3631480	3632109	-	rrmJ	23S rRNA methyltransferase J
i02_3624	CDS	3632235	3632528	+	yhbY	RNA-binding protein YhbY
i02_3625	CDS	3632684	3633160	-	greA	transcription elongation factor GreA
i02_3626	CDS	3633409	3634842	+	dacB	D-alanyl-D-alanine
i02_3627	CDS	3634883	3636055	-	obgE	GTPase ObgE
i02_3628	CDS	3636071	3637036	-	yhbE	putative transport protein YhbE
i02_3629	CDS	3637163	3637420	-	rpmA	50S ribosomal protein L27
i02_3630	CDS	3637414	3637959	+	/	hypothetical protein
i02_3631	CDS	3638011	3638982	+	ispB	octaprenyl diphosphate synthase
i02_3632	CDS	3639215	3639493	+	nlp	DNA-binding transcriptional regulator Nlp
i02_3633	CDS	3639541	3640800	-	murA	UDP-N-acetylglucosamine
i02_3634	CDS	3640855	3641124	-	yrbA	hypothetical protein
i02_3635	CDS	3641269	3641658	-	yrbB	hypothetical protein
i02_3636	CDS	3641562	3642197	-	yrbC	hypothetical protein
i02_3637	CDS	3642216	3642782	-	yrbD	hypothetical protein
i02_3638	CDS	3642772	3643554	-	yrbE	hypothetical protein
i02_3639	CDS	3643562	3644371	-	yrbF	putative ABC transporter ATP-binding protein
i02_3640	CDS	3644581	3645558	+	yrbG	putative calcium/sodium:proton antiporter
i02_3641	CDS	3645551	3646558	+	/	D-arabinose 5-phosphate isomerase
i02_3642	CDS	3646579	3647145	+	yrbI	3-deoxy-D-manno-octulosonate 8-phosphate phosphatase monomer (YrbI)
i02_3643	CDS	3647142	3647717	+	yrbK	hypothetical protein
i02_3644	CDS	3647665	3648243	+	yhbN	lipopolysaccharide transport periplasmic protein
i02_3645	CDS	3648250	3648975	+	yhbG	putative ABC transporter ATP-binding protein
i02_3646	CDS	3649023	3650456	+	rpoN	RNA polymerase factor sigma-54
i02_3647	CDS	3650479	3650766	+	yhbH	putative sigma(54) modulation protein
i02_3648	CDS	3650860	3651375	+	ptsN	PTS IIA-like nitrogen-regulatory protein PtsN
i02_3649	CDS	3651421	3652275	+	yhbJ	hypothetical protein
i02_3650	CDS	3652272	3652544	+	ptsO	phosphohistidinoprotein-hexose

Locus_tag	Type	start	End	+/-[a]	Gene[b]	Product
i02_3651	CDS	3652757	3653389	+	yrbL	hypothetical protein
i02_3652	CDS	3653386	3654114	-	mtgA	monofunctional biosynthetic peptidoglycan
i02_3653	CDS	3654111	3654764	-	yhbL	isoprenoid biosynthesis protein
i02_3654	CDS	3654994	3657330	-	arcB	aerobic respiration control sensor protein ArcB
i02_3655	CDS	3657426	3658355	-	yhcC	hypothetical protein
i02_3656	CDS	3658620	3658757	-	/	hypothetical protein
i02_3657	CDS	3658922	3663490	+	gltB	glutamate synthase subunit alpha
i02_3658	CDS	3663503	3664921	+	gltD	glutamate synthase subunit beta
i02_3659	CDS	3664989	3665453	-	yhcH	hypothetical protein
i02_3660	CDS	3665450	3666325	-	/	N-acetylmannosamine kinase
i02_3661	CDS	3666322	3667053	-	yhcJ	N-acetylmannosamine-6-phosphate 2-epimerase
i02_3662	CDS	3667059	3668579	-	nanT	putative sialic acid transporter
i02_3663	CDS	3668658	3669551	-	nanA	N-acetylneuraminate lyase
i02_3664	CDS	3669673	3670464	-	yhcK	transcriptional regulator NanR
i02_3665	CDS	3670572	3671069	-	sspB	ClpXP protease specificity-enhancing factor
i02_3666	CDS	3671075	3671713	-	sspA	stringent starvation protein A
i02_3667	CDS	3671655	3671864	+	/	hypothetical protein
i02_3668	CDS	3672108	3672500	-	rpsI	30S ribosomal protein S9
i02_3669	CDS	3672516	3672944	-	rplM	50S ribosomal protein L13
i02_3670	CDS	3673164	3674291	-	yhcM	hypothetical protein
i02_3671	CDS	3674479	3674883	+	yhcB	cytochrome d ubiquinol oxidase subunit III
i02_3672	CDS	3674995	3676404	+	degQ	serine endoprotease
i02_3673	CDS	3676494	3677561	+	degS	serine endoprotease
i02_3674	CDS	3677624	3678562	-	mdh	malate dehydrogenase
i02_3675	CDS	3678997	3679467	+	argR	arginine repressor
i02_3676	CDS	3679780	3680094	+	yhcN	hypothetical protein
i02_3677	CDS	3680149	3680421	-	yhcO	hypothetical protein
i02_3678	CDS	3680513	3682480	-	yhcP	p-hydroxybenzoic acid efflux subunit AaeB
i02_3679	CDS	3682486	3683418	-	yhcQ	p-hydroxybenzoic acid efflux subunit AaeA
i02_3680	CDS	3683426	3683629	-	yhcR	hypothetical protein
i02_3681	CDS	3683812	3684741	+	yhcS	putative DNA-binding transcriptional regulator
i02_3682	CDS	3684869	3686314	-	tldD	protease TldD
i02_3683	CDS	3686470	3690270	-	yhdP	hypothetical protein
i02_3684	CDS	3690338	3691807	-	cafA	ribonuclease G
i02_3685	CDS	3691797	3692390	-	maf	Maf-like protein
i02_3686	CDS	3692399	3692887	-	mreD	rod shape-determining protein MreD
i02_3687	CDS	3692887	3693990	-	mreC	rod shape-determining protein MreC
i02_3688	CDS	3694056	3695099	-	mreB	rod shape-determining protein MreB
i02_3689	CDS	3695207	3695395	+	/	hypothetical protein
i02_3690	CDS	3695404	3697344	-	yhdA	regulatory protein CsrD
i02_3691	CDS	3697496	3698470	+	yhdH	hypothetical protein
i02_3692	CDS	3698507	3698671	+	/	hypothetical protein
i02_3693	CDS	3699448	3699918	+	accB	acetyl-CoA carboxylase biotin carboxyl carrier
i02_3694	CDS	3699929	3701278	+	accC	acetyl-CoA carboxylase biotin carboxylase
i02_3695	CDS	3701370	3702416	-	/	hypothetical protein
i02_3696	CDS	3702413	3703375	-	/	hypothetical protein
i02_3697	CDS	3703397	3704386	-	/	ribose ABC transporter permease
i02_3698	CDS	3704387	3705916	-	/	ribose ABC transporter ATP-binding protein
i02_3699	CDS	3705947	3706843	-	/	putative ribose ABC transporter
i02_3700	CDS	3706873	3707727	-	gatY	tagatose-bisphosphate aldolase
i02_3701	CDS	3707702	3707830	+	/	hypothetical protein
i02_3702	CDS	3708069	3708899	+	/	hypothetical protein
i02_3703	CDS	3708856	3709866	+	/	hypothetical protein
i02_3704	CDS	3709941	3710201	+	yhdT	hypothetical protein
i02_3705	CDS	3710185	3711642	+	panF	sodium/panthothenate symporter
i02_3706	CDS	3711654	3712535	+	prmA	ribosomal protein L11 methyltransferase
i02_3707	CDS	3712516	3712644	-	/	hypothetical protein
i02_3708	CDS	3712864	3713829	+	yhdG	tRNA-dihydrouridine synthase B

Locus_tag	Type	start	End	+/-[a]	Gene[b]	Product
i02_3709	CDS	3713855	3714151	+	fis	DNA-binding protein Fis
i02_3710	CDS	3714237	3715121	+	yhdJ	putative methyltransferase
i02_3711	CDS	3715178	3715384	+	yhdU	hypothetical protein
i02_3712	CDS	3715387	3716049	-	envR	DNA-binding transcriptional regulator EnvR
i02_3713	CDS	3716448	3717605	+	acrE	acriflavin resistance protein E
i02_3714	CDS	3717617	3720721	+	acrF	acriflavin resistance protein F
i02_3715	CDS	3720974	3721195	+	yhdV	hypothetical protein
i02_3716	CDS	3721431	3721550	+	/	hypothetical protein
i02_3717	CDS	3721626	3722651	+	yhdW	putative amino-acid ABC transporter
i02_3718	CDS	3722701	3723900	+	yhdX	amino-acid ABC transporter permease protein
i02_3719	CDS	3723907	3725013	+	yhdY	amino-acid ABC transporter permease protein
i02_3720	CDS	3725021	3725779	+	yhdZ	amino-acid ABC transporter ATP-binding protein
i02_3722	CDS	3731367	3732215	+	yrdA	hypothetical protein
i02_3723	CDS	3732191	3732448	-	yrdB	hypothetical protein
i02_3724	CDS	3732445	3733263	-	aroE	shikimate 5-dehydrogenase
i02_3725	CDS	3733268	3733885	-	yrdC	putative ribosome maturation factor
i02_3726	CDS	3733845	3734387	-	yrdD	hypothetical protein
i02_3727	CDS	3734416	3734889	-	smg	hypothetical protein
i02_3728	CDS	3734861	3735985	-	smf	DNA protecting protein DprA
i02_3729	CDS	3736115	3736624	+	def	peptide deformylase
i02_3730	CDS	3736639	3737586	+	fmt	methionyl-tRNA formyltransferase
i02_3731	CDS	3737638	3738927	+	sun	16S rRNA methyltransferase B
i02_3732	CDS	3738949	3740325	+	trkA	potassium transporter
i02_3733	CDS	3740455	3740868	+	mscL	large-conductance mechanosensitive channel
i02_3734	CDS	3740865	3741083	-	yhdL	putative regulator
i02_3735	CDS	3741139	3741564	-	zntR	zinc-responsive transcriptional regulator
i02_3736	CDS	3741575	3741943	-	yhdN	hypothetical protein
i02_3737	CDS	3742050	3742433	-	rplQ	50S ribosomal protein L17
i02_3738	CDS	3742474	3743463	-	rpoA	DNA-directed RNA polymerase subunit alpha
i02_3739	CDS	3743489	3744109	-	rpsD	30S ribosomal protein S4
i02_3740	CDS	3744143	3744532	-	rpsK	30S ribosomal protein S11
i02_3741	CDS	3744549	3744905	-	rpsM	30S ribosomal protein S13
i02_3742	CDS	3745052	3745168	-	/	predicted protein
i02_3743	CDS	3745200	3746531	-	secY	preprotein translocase subunit SecY
i02_3744	CDS	3746539	3746973	-	rplO	50S ribosomal protein L15
i02_3745	CDS	3746977	3747156	-	rpmD	50S ribosomal protein L30
i02_3746	CDS	3747160	3747663	-	rpsE	30S ribosomal protein S5
i02_3747	CDS	3747678	3748031	-	rplR	50S ribosomal protein L18
i02_3748	CDS	3748041	3748574	-	rplF	50S ribosomal protein L6
i02_3750	CDS	3748587	3748979	-	rpsH	30S ribosomal protein S8
i02_3751	CDS	3749013	3749318	-	rpsN	30S ribosomal protein S14
i02_3752	CDS	3749333	3749872	-	rplE	50S ribosomal protein L5
i02_3753	CDS	3749887	3750201	-	rplX	50S ribosomal protein L24
i02_3755	CDS	3750212	3750583	-	rplN	50S ribosomal protein L14
i02_3756	CDS	3750748	3751002	-	rpsQ	30S ribosomal protein S17
i02_3757	CDS	3751002	3751193	-	rpmC	50S ribosomal protein L29
i02_3758	CDS	3751193	3751603	-	rplP	50S ribosomal protein L16
i02_3759	CDS	3751616	3752317	-	rpsC	30S ribosomal protein S3
i02_3760	CDS	3752335	3752661	-	rplV	50S ribosomal protein L22
i02_3761	CDS	3752682	3752960	-	rpsS	30S ribosomal protein S19
i02_3762	CDS	3752977	3753798	-	rplB	50S ribosomal protein L2
i02_3763	CDS	3753816	3754118	-	rplW	50S ribosomal protein L23
i02_3764	CDS	3754115	3754720	-	rplD	50S ribosomal protein L4
i02_3765	CDS	3754731	3755360	-	rplC	50S ribosomal protein L3
i02_3766	CDS	3755393	3755704	-	rpsJ	30S ribosomal protein S10
i02_3767	CDS	3755942	3756364	-	pinO	chromosome replication protein PioO
i02_3768	CDS	3756366	3757835	-	yheD	general secretion pathway protein A
i02_3769	CDS	3758015	3758830	+	yheE	general secretion pathway protein C

Locus_tag	Type	start	End	+/-[a]	Gene[b]	Product
i02_3770	CDS	3758802	3760766	+	yheF	general secretion pathway protein D precursor
i02_3771	CDS	3760776	3762257	+	yheG	general secretion pathway protein E
i02_3772	CDS	3762254	3763450	+	hofF	putative general secretion pathway protein F
i02_3773	CDS	3763460	3763897	+	hofG	putative general secretion pathway protein G
i02_3774	CDS	3763905	3764414	+	hofH	putative general secretion pathway protein H
i02_3775	CDS	3764372	3764788	+	yheH	general secretion pathway protein I precursor
i02_3776	CDS	3764781	3765368	+	yheI	general secretion pathway protein J precursor
i02_3777	CDS	3765361	3766266	+	yheJ	general secretion pathway protein K
i02_3778	CDS	3766356	3767522	+	yheK	general secretion pathway protein L
i02_3779	CDS	3767495	3767980	+	pshM	putative general secretion pathway protein M
i02_3780	CDS	3767980	3768657	+	hofD	leader peptidase
i02_3781	CDS	3768686	3769162	-	bfr	bacterioferritin
i02_3782	CDS	3769234	3769428	-	yheA	bacterioferritin-associated ferredoxin
i02_3783	CDS	3769478	3769594	-	/	hypothetical protein
i02_3784	CDS	3769597	3772299	-	chiA	secreted endochitinase
i02_3785	CDS	3772384	3772542	-	/	hypothetical protein
i02_3785a	CDS	3772591	3773775	-	tuf	elongation factor Tu
i02_3786	CDS	3773846	3775960	-	fusA	elongation factor G
i02_3787	CDS	3776057	3776527	-	rpsG	30S ribosomal protein S7
i02_3788	CDS	3776624	3776998	-	rpsL	30S ribosomal protein S12
i02_3789	CDS	3777124	3777411	-	yheL	sulfur transfer complex subunit TusB
i02_3790	CDS	3777419	3777778	-	yheM	sulfur relay protein TusC
i02_3791	CDS	3777778	3778164	-	yheN	sulfur transfer complex subunit TusD
i02_3792	CDS	3778164	3778898	-	yheO	hypothetical protein
i02_3793	CDS	3779053	3779865	-	fkpA	FKBP-type peptidyl-prolyl cis-trans isomerase
i02_3794	CDS	3780086	3780304	+	slyX	hypothetical protein
i02_3795	CDS	3780353	3780943	-	slyD	FKBP-type peptidyl-prolyl cis-trans isomerase
i02_3796	CDS	3781038	3781238	-	/	hypothetical protein
i02_3797	CDS	3781248	3783053	-	kefB	glutathione-regulated potassium-efflux system
i02_3798	CDS	3783053	3783607	-	yheR	glutathione-regulated potassium-efflux system
i02_3799	CDS	3783735	3785648	+	yheS	putative ABC transporter ATP-binding protein
i02_3800	CDS	3785648	3786670	+	yheT	putative hydrolase
i02_3801	CDS	3786664	3786882	+	yheU	hypothetical protein
i02_3802	CDS	3786936	3787805	+	prkB	phosphoribulokinase
i02_3803	CDS	3787860	3788264	-	yhfA	hypothetical protein
i02_3804	CDS	3788566	3789198	+	crp	cAMP-regulatory protein
i02_3805	CDS	3789237	3791339	+	yhfK	hypothetical protein
i02_3806	CDS	3791406	3792626	-	argD	bifunctional
i02_3807	CDS	3792712	3793275	-	pabA	para-aminobenzoate synthase component II
i02_3808	CDS	3793307	3793909	-	fic	cell filamentation protein Fic
i02_3809	CDS	3793899	3794066	-	yhfG	hypothetical protein
i02_3810	CDS	3794171	3794743	-	ppiA	peptidyl-prolyl cis-trans isomerase A
i02_3811	CDS	3795014	3796195	+	yhfC	hypothetical protein
i02_3812	CDS	3796328	3796504	-	/	hypothetical protein
i02_3813	CDS	3796457	3799000	+	nirB	nitrite reductase [NAD(P)H] large subunit
i02_3814	CDS	3798997	3799323	+	nirD	nitrite reductase small subunit
i02_3815	CDS	3799449	3800255	+	nirC	nitrite transporter NirC
i02_3816	CDS	3800274	3801647	+	cysG	siroheme synthase
i02_3817	CDS	3801899	3802066	+	yhfL	hypothetical protein
i02_3818	CDS	3802174	3803259	-	yhfS	hypothetical protein
i02_3819	CDS	3803271	3804575	-	yhfT	hypothetical protein
i02_3820	CDS	3804587	3804979	-	yhfU	hypothetical protein
i02_3821	CDS	3804951	3805829	-	yhfV	putative hydrolase
i02_3822	CDS	3805826	3807052	-	yhfW	putative mutase
i02_3823	CDS	3807053	3808216	-	yhfX	hypothetical protein
i02_3824	CDS	3808300	3808704	-	yhfY	hypothetical protein
i02_3825	CDS	3808679	3809584	-	yhfZ	hypothetical protein
i02_3826	CDS	3809744	3810748	-	trpS	tryptophanyl-tRNA synthetase

Locus_tag	Type	start	End	+/-[a]	Gene[b]	Product
i02_3827	CDS	3810741	3811499	-	gph	phosphoglycolate phosphatase
i02_3828	CDS	3811492	3812169	-	rpe	ribulose-phosphate 3-epimerase
i02_3829	CDS	3812187	3813023	-	dam	DNA adenine methylase
i02_3830	CDS	3813130	3814416	-	damX	hypothetical protein
i02_3831	CDS	3814508	3815596	-	aroB	3-dehydroquinate synthase
i02_3832	CDS	3815653	3816174	-	aroK	shikimate kinase I
i02_3833	CDS	3816575	3817813	-	hofQ	outer membrane porin HofQ
i02_3834	CDS	3817725	3818168	-	yrfA	hypothetical protein
i02_3835	CDS	3818119	3818559	-	yrfB	hypothetical protein
i02_3836	CDS	3818543	3819082	-	yrfC	hypothetical protein
i02_3837	CDS	3819082	3819888	-	yrfD	hypothetical protein
i02_3838	CDS	3819981	3822533	+	mrcA	peptidoglycan synthetase
i02_3839	CDS	3822699	3823259	-	nudE	ADP-ribose diphosphatase NudE
i02_3840	CDS	3823460	3823582	+	/	hypothetical protein
i02_3841	CDS	3823579	3825714	+	yrfF	putative membrane protein igaA-like protein
i02_3842	CDS	3825734	3826447	+	yrfG	hypothetical protein
i02_3843	CDS	3826458	3826859	+	yrfH	ribosome-associated heat shock protein Hsp15
i02_3844	CDS	3826878	3827762	+	hslO	Hsp33-like chaperonin
i02_3845	CDS	3827900	3829624	-	yhgE	hypothetical protein
i02_3846	CDS	3829700	3829831	-	/	hypothetical protein
i02_3847	CDS	3829934	3831625	+	pckA	phosphoenolpyruvate carboxykinase
i02_3848	CDS	3831748	3831927	+	/	hypothetical protein
i02_3849	CDS	3831900	3832052	+	/	hypothetical protein
i02_3850	CDS	3832111	3832407	+	/	hypothetical protein
i02_3851	CDS	3832438	3833790	-	envZ	osmolarity sensor protein
i02_3852	CDS	3833787	3834506	-	ompR	osmolarity response regulator
i02_3853	CDS	3834596	3834781	-	/	hypothetical protein
i02_3854	CDS	3834734	3835210	+	greB	transcription elongation factor GreB
i02_3855	CDS	3835307	3837628	+	yhgF	hypothetical protein
i02_3856	CDS	3838084	3838311	+	feoA	ferrous iron transport protein A
i02_3857	CDS	3838316	3840649	+	feoB	ferrous iron transport protein B
i02_3858	CDS	3840640	3840885	+	yhgG	hypothetical protein
i02_3859	CDS	3841118	3841711	+	yhgA	hypothetical protein
i02_3860	CDS	3841740	3842510	-	bioH	carboxylesterase BioH
i02_3861	CDS	3842500	3843231	+	yhgH	gluconate periplasmic binding protein
i02_3862	CDS	3843290	3843865	+	yhgI	putative DNA uptake protein
i02_3863	CDS	3844229	3845545	+	gntT	high-affinity gluconate transporter
i02_3864	CDS	3845656	3847740	-	malQ	4-alpha-glucanotransferase
i02_3865	CDS	3847750	3850143	-	malP	maltodextrin phosphorylase
i02_3866	CDS	3850488	3850634	-	/	hypothetical protein
i02_3867	CDS	3850766	3853471	+	malT	transcriptional regulator MalT
i02_3868	CDS	3853514	3854533	-	/	RNA 3'-terminal-phosphate cyclase
i02_3869	CDS	3854534	3855823	-	rtcB	protein rtcB
i02_3870	CDS	3855949	3857547	+	rtcR	transcriptional regulatory protein rtcR
i02_3872	CDS	3857529	3858338	-	glpR	DNA-binding transcriptional repressor GlpR
i02_3873	CDS	3858304	3859134	-	glpG	intramembrane serine protease GlpG
i02_3874	CDS	3859179	3859505	-	glpE	thiosulfate sulfurtransferase
i02_3875	CDS	3859695	3861200	+	glpD	glycerol-3-phosphate dehydrogenase
i02_3876	CDS	3861254	3861859	-	/	hypothetical protein
i02_3877	CDS	3861862	3862629	-	/	hypothetical protein
i02_3878	CDS	3862632	3863342	-	/	hypothetical protein
i02_3879	CDS	3863516	3864646	-	/	putative fimbrial adhesin precursor
i02_3880	CDS	3864665	3865462	-	/	putative fimbrial chaperone precursor
i02_3881	CDS	3865407	3865985	-	/	putative minor fimbrial subunit precursor
i02_3882	CDS	3865930	3866502	-	/	putative minor fimbrial subunit precursor
i02_3883	CDS	3866493	3869087	-	ycbS	outer membrane usher protein ycbS precursor
i02_3884	CDS	3869124	3869903	-	/	chaperone protein fimC precursor
i02_3885	CDS	3869977	3870681	-	/	putative major fimbrial subunit precursor

Locus_tag	Type	start	End	+/-[a]	Gene[b]	Product
i02_3886	CDS	3871176	3873623	-	glgP	glycogen phosphorylase
i02_3887	CDS	3873642	3875075	-	glgA	glycogen synthase
i02_3888	CDS	3875075	3876406	-	glgC	glucose-1-phosphate adenylyltransferase
i02_3889	CDS	3876388	3878361	-	glgX	glycogen debranching enzyme
i02_3890	CDS	3878358	3880544	-	glgB	glycogen branching enzyme
i02_3891	CDS	3880817	3881980	-	asd	aspartate-semialdehyde dehydrogenase
i02_3892	CDS	3882112	3882705	+	yhgN	putative dITP- and XTP- hydrolase
i02_3893	CDS	3882751	3884046	-	/	putative DNA processing protein
i02_3894	CDS	3883953	3886052	-	/	hypothetical protein
i02_3895	CDS	3886190	3887503	-	gntU	low affinity gluconate transporter
i02_3896	CDS	3887534	3888061	-	gntK	gluconate kinase 1
i02_3897	CDS	3888200	3889195	-	gntR	gluconate utilization system GNT-I
i02_3898	CDS	3889288	3889983	-	yhhW	hypothetical protein
i02_3899	CDS	3890106	3891143	-	yhhX	putative dehydrogenase
i02_3900	CDS	3891090	3891248	+	/	hypothetical protein
i02_3901	CDS	3891200	3891436	-	/	hypothetical protein
i02_3902	pseudo	3891477	3891965	+	yhhY	putative acetyltransferase YhhY
i02_3903	CDS	3892201	3893379	+	yhhZ	hypothetical protein
i02_3904	CDS	3893376	3893870	+	yrhA	hypothetical protein
i02_3905	CDS	3894326	3894604	+	yrhB	hypothetical protein
i02_3906	CDS	3894642	3896375	-	ggt	gamma-glutamyltranspeptidase
i02_3907	CDS	3896495	3896935	+	yhhA	hypothetical protein
i02_3908	CDS	3896922	3897665	-	ugpQ	cytoplasmic glycerophosphodiester
i02_3909	CDS	3897662	3898732	-	ugpC	glycerol-3-phosphate transporter ATP-binding component
i02_3910	CDS	3898734	3899579	-	ugpE	glycerol-3-phosphate transporter membrane component
i02_3911	CDS	3899576	3900463	-	ugpA	glycerol-3-phosphate transporter permease component
i02_3912	CDS	3900561	3901877	-	ugpB	glycerol-3-phosphate transporter periplasmic component
i02_3913	CDS	3902154	3902285	+	/	hypothetical protein
i02_3914	CDS	3902274	3902999	-	livF	leucine/isoleucine/valine transporter
i02_3915	CDS	3902989	3903756	-	livG	leucine/isoleucine/valine transporter
i02_3916	CDS	3903753	3905030	-	livM	leucine/isoleucine/valine transporter permease
i02_3917	CDS	3905027	3905953	-	livH	branched-chain amino acid transporter permease
i02_3918	CDS	3906001	3907164	-	livK	leucine-specific binding protein precursor
i02_3919	CDS	3907137	3907283	+	/	hypothetical protein
i02_3920	CDS	3907262	3907399	-	/	hypothetical protein
i02_3921	CDS	3907534	3907917	+	yhhK	hypothetical protein
i02_3922	CDS	3907927	3908049	+	/	hypothetical protein
i02_3923	CDS	3908105	3909265	-	livJ	Leu/Ile/Val-binding protein precursor
i02_3924	CDS	3909479	3910333	-	rpoH	RNA polymerase factor sigma-32
i02_3925	CDS	3910578	3911663	-	ftsX	cell division protein FtsX
i02_3926	CDS	3911629	3912297	-	ftsE	cell division protein FtsE
i02_3927	CDS	3912300	3913790	-	ftsY	cell division protein FtsY
i02_3928	CDS	3913940	3914536	+	rsmD	16S rRNA m(2)G966-methyltransferase
i02_3929	CDS	3914523	3914795	+	yhhL	hypothetical protein
i02_3930	CDS	3914798	3915157	-	yhhM	hypothetical protein
i02_3931	CDS	3915298	3915924	+	yhhN	hypothetical protein
i02_3932	CDS	3915998	3918196	+	zntA	zinc/cadmium/mercury/lead-transporting ATPase
i02_3933	CDS	3918457	3918702	-	yhhP	sulfur transfer protein SirA
i02_3934	CDS	3918923	3919588	+	yhhQ	hypothetical protein
i02_3935	CDS	3919619	3920218	+	/	hypothetical protein
i02_3936	CDS	3920222	3921439	-	yhhS	major facilitator superfamily transporter
i02_3937	CDS	3921490	3922620	+	yhhT	hypothetical protein
i02_3938	CDS	3922675	3923262	+	yhhU	holo-(acyl carrier protein) synthase 2
i02_3939	CDS	3923373	3924947	+	nikA	nickel-binding periplasmic protein precursor

Locus_tag	Type	start	End	+/-[a]	Gene[b]	Product
i02_3940	CDS	3924947	3925891	+	nikB	nickel transporter permease NikB
i02_3941	CDS	3925888	3926721	+	nikC	nickel transporter permease NikC
i02_3942	CDS	3926721	3927485	+	nikD	nickel transporter ATP-binding protein NikD
i02_3943	CDS	3927482	3928288	+	nikE	nickel transporter ATP-binding protein NikE
i02_3944	CDS	3928294	3928695	+	yhhG	nickel responsive regulator
i02_3946	CDS	3928885	3929640	+	/	putative regulator
i02_3947	CDS	3929653	3930138	+	/	putative phosphotransferase system enzyme
i02_3948	CDS	3930135	3930416	+	/	putative phosphotransferase system enzyme
i02_3949	CDS	3930463	3931851	+	/	PTS system, galactitol-specific IIC component
i02_3950	CDS	3931844	3933352	+	/	putative xylulose kinase
i02_3951	CDS	3933204	3933566	-	/	hypothetical protein
i02_3952	CDS	3933342	3933611	+	/	putative phosphocarrier protein
i02_3953	CDS	3933643	3934494	+	gatY	putative fructose-1,6-bisphosphate aldolase
i02_3954	CDS	3934604	3935812	-	yhhJ	hypothetical protein
i02_3955	CDS	3935728	3938463	-	yhiH	ABC transporter ATP-binding protein
i02_3956	CDS	3938460	3939527	-	yhiI	hypothetical protein
i02_3957	CDS	3939520	3939651	-	/	hypothetical protein
i02_3958	CDS	3940249	3941313	+	yhiM	hypothetical protein
i02_3959	CDS	3941400	3942593	-	yhiN	hypothetical protein
i02_3960	CDS	3942825	3944324	+	pitA	inorganic phosphate transporter
i02_3961	CDS	3944395	3944730	-	yhiO	universal stress protein UspB
i02_3962	CDS	3945121	3945555	+	uspA	universal stress protein A
i02_3963	CDS	3945738	3945917	-	/	hypothetical protein
i02_3964	CDS	3945872	3947341	+	yhiP	inner membrane transporter YhiP
i02_3965	CDS	3947390	3948247	-	yhiQ	putative methyltransferase
i02_3966	CDS	3948150	3950231	-	prlC	oligopeptidase A
i02_3967	CDS	3950344	3951237	+	yhiR	hypothetical protein
i02_3968	CDS	3951309	3952661	+	gor	glutathione reductase
i02_3969	CDS	3952715	3952864	-	/	hypothetical protein
i02_3970	CDS	3953133	3953558	+	arsC	arsenate reductase
i02_3971	CDS	3953612	3953776	+	/	hypothetical protein
i02_3972	CDS	3954175	3954348	+	/	hypothetical protein
i02_3973	CDS	3954709	3955308	+	slp	Outer membrane protein Slp precursor
i02_3974	CDS	3955349	3955522	+	/	hypothetical protein
i02_3975	CDS	3955461	3955991	+	yhiF	putative transcriptional regulator YhiF
i02_3976	CDS	3956048	3957076	-	chuS	putative heme/hemoglobin transport protein
i02_3977	CDS	3957125	3959107	-	chuA	Outer membrane heme/hemoglobin receptor
i02_3978	CDS	3959046	3959204	+	/	hypothetical protein
i02_3979	CDS	3959208	3959393	-	/	hypothetical protein
i02_3980	CDS	3959476	3959637	+	/	hypothetical protein
i02_3981	CDS	3959713	3960705	+	chuT	putative periplasmic binding protein
i02_3982	CDS	3960725	3962062	+	chuW	coproporphyrinogen III oxidase
i02_3983	CDS	3962075	3962569	+	chuX	hypothetical protein
i02_3984	CDS	3962569	3963192	+	chuY	hypothetical protein
i02_3985	CDS	3963241	3964233	+	chuU	iron ABC transporter permease
i02_3986	CDS	3964200	3965000	+	hmuV	hemin importer ATP-binding subunit
i02_3987	CDS	3965052	3965699	-	yhiD	putative Mg(2+) transport ATPase
i02_3988	CDS	3965763	3966101	-	hdeB	acid-resistance protein
i02_3989	CDS	3966205	3966483	-	hdeA	acid-resistance protein
i02_3990	CDS	3966792	3967364	+	hdeD	acid-resistance membrane protein
i02_3991	CDS	3968085	3968690	+	yhiE	hypothetical protein
i02_3992	CDS	3968734	3969027	+	/	hypothetical protein
i02_3993	CDS	3969029	3970186	+	yhiU	multidrug efflux system protein MdtE
i02_3994	CDS	3970211	3973324	+	yhiV	hypothetical protein
i02_3995	CDS	3973687	3974415	-	yhiW	putative transcriptional regulator YhiW
i02_3996	pseudo	3974784	3975608	-	yhiX	DNA-binding transcriptional regulator GadX
i02_3997	CDS	3975976	3977439	-	gadA	glutamate decarboxylase alpha
i02_3998	CDS	3977587	3978984	-	yhjA	cytochrome C peroxidase

Locus_tag	Type	start	End	+/-[a]	Gene[b]	Product
i02_3999	CDS	3979389	3981038	+	treF	trehalase
i02_4000	CDS	3981089	3981691	-	yhjB	putative transcriptional regulator YhjB
i02_4001	CDS	3981871	3981999	-	/	conserved hypothetical protein
i02_4002	CDS	3982139	3983110	+	yhjC	putative transcriptional regulator YhjC
i02_4003	CDS	3983159	3984172	+	yhjD	hypothetical protein
i02_4004	CDS	3984565	3985887	+	yhjE	metabolite transport protein
i02_4005	CDS	3986066	3988141	-	yhjG	hypothetical protein
i02_4006	CDS	3988196	3988966	-	yhjH	EAL domain-containing protein
i02_4007	CDS	3988847	3990124	+	kdgK	2-dehydro-3-deoxygluconokinase
i02_4008	CDS	3990220	3991716	-	yhjJ	hypothetical protein
i02_4009	CDS	3991936	3993222	-	dctA	C4-dicarboxylate transporter DctA
i02_4010	CDS	3993405	3995321	-	yhjK	putative phosphodiesterase
i02_4011	CDS	3995475	3998948	-	yhjL	cellulose synthase subunit BcsC
i02_4012	CDS	3998930	4000042	-	yhjM	endo-1,4-D-glucanase
i02_4013	CDS	4000043	4002469	-	yhjN	cellulose synthase regulator protein
i02_4014	CDS	4002393	4005059	-	bcsA	cellulose synthase catalytic subunit
i02_4015	CDS	4005008	4005760	-	yhjQ	cell division protein
i02_4016	CDS	4005772	4005960	-	yhjR	hypothetical protein
i02_4017	CDS	4006233	4007804	+	yhjS	hypothetical protein
i02_4018	CDS	4007801	4007992	+	yhjT	hypothetical protein
i02_4019	CDS	4007989	4009668	+	yhjU	hypothetical protein
i02_4020	CDS	4009754	4009996	-	/	hypothetical protein
i02_4021	CDS	4009924	4010292	+	/	hypothetical protein
i02_4023	CDS	4010237	4010512	-	/	hypothetical protein
i02_4024	CDS	4010509	4010670	-	/	hypothetical protein
i02_4025	CDS	4010769	4012091	+	yhjV	putative transport protein YhjV
i02_4026	CDS	4012121	4013125	-	dppF	dipeptide transporter ATP-binding subunit
i02_4027	CDS	4013122	4014105	-	dppD	dipeptide transporter ATP-binding subunit
i02_4028	CDS	4014116	4015018	-	dppC	dipeptide transporter
i02_4029	CDS	4015028	4016047	-	dppB	dipeptide transporter permease DppB
i02_4030	CDS	4016354	4017961	-	dppA	periplasmic dipeptide transport protein
i02_4031	CDS	4018313	4018468	-	/	Hypothetical protein c4363
i02_4032	CDS	4018873	4020564	-	yhjW	phosphoethanolamine transferase
i02_4033	CDS	4020888	4022096	-	yhjX	hypothetical protein
i02_4034	CDS	4022325	4023029	-	yhjY	hypothetical protein
i02_4035	CDS	4023181	4023744	+	tag	3-methyl-adenine DNA glycosylase I
i02_4036	CDS	4023741	4024181	+	yiaC	hypothetical protein
i02_4037	CDS	4024150	4026483	-	bisC	biotin sulfoxide reductase
i02_4038	CDS	4026636	4027295	+	yiaD	putative outer membrane lipoprotein
i02_4039	CDS	4027387	4028373	+	yiaE	2-hydroxyacid dehydrogenase
i02_4040	CDS	4028423	4029253	-	yiaF	hypothetical protein
i02_4041	CDS	4029288	4029437	+	/	hypothetical protein
i02_4042	CDS	4029567	4029857	+	/	putative transcriptional regulator
i02_4043	CDS	4030138	4030350	+	cspA	major cold shock protein
i02_4044	CDS	4030538	4030690	-	/	small toxic polypeptide
i02_4045	CDS	4031013	4033082	-	glyS	glycyl-tRNA synthetase subunit beta
i02_4046	CDS	4033092	4034003	-	glyQ	glycyl-tRNA synthetase subunit alpha
i02_4047	CDS	4034098	4034397	-	/	hypothetical protein
i02_4048	CDS	4034572	4035567	+	yiaH	hypothetical protein
i02_4049	CDS	4035609	4036049	-	yiaA	hypothetical protein
i02_4050	CDS	4036092	4036475	-	yiaB	hypothetical protein
i02_4051	CDS	4036602	4038056	-	xylB	xylulokinase
i02_4052	CDS	4038128	4039450	-	xylA	xylose isomerase
i02_4053	CDS	4039738	4040808	+	xylF	D-xylose transporter subunit XylF
i02_4054	CDS	4040886	4042427	+	xylG	xylose transporter ATP-binding subunit
i02_4055	CDS	4042405	4043586	+	xylH	xylose transport system permease protein xylH
i02_4056	CDS	4043664	4044842	+	xylR	xylose operon regulatory protein
i02_4057	CDS	4044950	4045774	-	bax	hypothetical protein

Locus_tag	Type	start	End	+/-[a]	Gene[b]	Product
i02_4058	CDS	4045924	4046097	+	/	hypothetical protein
i02_4059	CDS	4046094	4048124	+	malS	periplasmic alpha-amylase precursor
i02_4060	CDS	4048302	4049555	+	avtA	valine--pyruvate transaminase
i02_4061	CDS	4049707	4050186	-	yiaI	putative electron transport protein ysaA
i02_4062	CDS	4050282	4051130	-	yiaJ	putative transcriptional regulator YiaJ
i02_4063	CDS	4051331	4052329	+	yiaK	2,3-diketo-L-gulonate reductase
i02_4064	CDS	4052341	4052805	+	yiaL	hypothetical protein
i02_4065	CDS	4052828	4053748	+	/	hypothetical protein
i02_4066	CDS	4053862	4054329	+	yiaM	2,3-diketo-L-gulonate TRAP transporter
i02_4067	CDS	4054134	4055609	+	yiaN	hypothetical protein
i02_4068	CDS	4055622	4056608	+	yiaO	ABC transporter periplasmic-binding protein
i02_4069	CDS	4056612	4058108	+	lyxK	Cryptic L-xylulose kinase
i02_4070	CDS	4058105	4058767	+	sgbH	3-keto-L-gulonate-6-phosphate decarboxylase
i02_4071	CDS	4058727	4059620	+	sgbU	putative L-xylulose 5-phosphate 3-epimerase
i02_4072	CDS	4059614	4060309	+	sgbE	L-ribulose-5-phosphate 4-epimerase
i02_4073	CDS	4060356	4061984	-	aldB	aldehyde dehydrogenase B
i02_4074	CDS	4062002	4063297	-	/	hypothetical protein
i02_4075	CDS	4063427	4064578	-	yiaY	putative alcohol dehydrogenase
i02_4076	CDS	4064769	4066613	-	selB	selenocysteinyl-tRNA-specific translation factor
i02_4077	CDS	4066610	4068001	-	selA	selenocysteine synthase
i02_4078	CDS	4068099	4068707	-	yibF	putative glutathione S-transferase
i02_4079	CDS	4068778	4069914	-	yibH	hypothetical protein
i02_4080	CDS	4069917	4070279	-	yibI	hypothetical protein
i02_4081	CDS	4070816	4072729	+	mtlA	PTS system, mannitol-specific IIABC component
i02_4082	CDS	4072969	4074297	+	mtlD	mannitol-1-phosphate 5-dehydrogenase
i02_4083	CDS	4074297	4074884	+	mtlR	mannitol repressor protein
i02_4084	CDS	4074895	4075104	-	/	hypothetical protein
i02_4085	CDS	4075388	4075750	+	yibL	hypothetical protein
i02_4086	CDS	4076006	4076173	-	/	hypothetical protein
i02_4087	CDS	4076309	4076992	+	/	hypothetical protein
i02_4088	CDS	4077036	4082372	+	/	putative adhesin
i02_4089	CDS	4082763	4084439	+	lldP	L-lactate permease
i02_4090	CDS	4084424	4085215	+	lldR	DNA-binding transcriptional repressor LldR
i02_4091	CDS	4085212	4086402	+	lldD	L-lactate dehydrogenase
i02_4092	CDS	4086450	4086923	+	yibK	putative tRNA/rRNA methyltransferase YibK
i02_4093	CDS	4086978	4087799	-	cysE	serine acetyltransferase
i02_4094	CDS	4087879	4088898	-	gpsA	NAD(P)H-dependent glycerol-3-phosphate
i02_4095	CDS	4088877	4089362	+	/	hypothetical protein
i02_4096	CDS	4088898	4089425	-	secB	preprotein translocase subunit SecB
i02_4097	CDS	4089429	4089680	-	grxC	glutaredoxin 3
i02_4098	CDS	4089495	4089758	+	/	hypothetical protein
i02_4099	CDS	4089821	4090264	-	yibN	hypothetical protein
i02_4100	CDS	4090276	4090473	+	/	hypothetical protein
i02_4101	CDS	4090497	4092041	+	yibO	phosphoglyceromutase
i02_4102	CDS	4092051	4093334	+	yibP	hypothetical protein
i02_4103	CDS	4093338	4094297	+	yibQ	hypothetical protein
i02_4104	CDS	4094284	4095318	-	yibD	putative glycosyl transferase
i02_4105	CDS	4095557	4096582	-	tdh	L-threonine 3-dehydrogenase
i02_4106	CDS	4096592	4097788	-	kbl	2-amino-3-ketobutyrate coenzyme A ligase
i02_4107	CDS	4098002	4098934	+	rfaD	ADP-L-glycero-D-mannoheptose-6-epimerase
i02_4108	CDS	4098944	4099990	+	rfaF	ADP-heptose:LPS heptosyltransferase II
i02_4109	CDS	4099994	4100974	+	rfaC	ADP-heptose:LPS heptosyl transferase I
i02_4110	CDS	4101044	4102297	-	waaL	Lipid A-core, surface polymer ligase
i02_4111	CDS	4102343	4103326	-	waaV	putative beta1,3-glucosyltransferase
i02_4112	CDS	4103408	4104436	-	waaW	UDP-galactose:(galactosyl) LPS
i02_4113	CDS	4104462	4105154	-	rfaY	lipopolysaccharide core biosynthesis protein
i02_4114	CDS	4105164	4106159	-	rfaJ	lipopolysaccharide 1,2-glucosyltransferase
i02_4115	CDS	4106176	4107192	-	rfaI	lipopolysaccharide 1,3-galactosyltransferase

Locus_tag	Type	start	End	+/-[a]	Gene[b]	Product
i02_4116	CDS	4107208	4108014	-	rfaP	lipopolysaccharide core biosynthesis protein
i02_4117	CDS	4107998	4109122	-	rfaG	lipopolysaccharide core biosynthesis protein
i02_4118	CDS	4109119	4110177	-	rfaQ	lipopolysaccharide core biosynthesis protein
i02_4119	CDS	4110590	4111867	+	kdtA	3-deoxy-D-manno-octulosonic-acid transferase
i02_4120	CDS	4111875	4112354	+	coaD	phosphopantetheine adenylyltransferase
i02_4121	CDS	4112393	4113202	-	mutM	formamidopyrimidine-DNA glycosylase
i02_4122	CDS	4113300	4113467	-	rpmG	50S ribosomal protein L33
i02_4123	CDS	4113488	4113724	-	rpmB	50S ribosomal protein L28
i02_4124	CDS	4113941	4114615	-	radC	DNA repair protein RadC
i02_4125	CDS	4114781	4116001	+	dfp	bifunctional phosphopantothenoylcysteine
i02_4126	CDS	4115979	4116437	+	dut	deoxyuridine triphosphatase
i02_4127	CDS	4116502	4117140	+	slmA	nucleoid occlusion protein
i02_4128	CDS	4117177	4117833	-	pyrE	orotate phosphoribosyltransferase
i02_4129	CDS	4117884	4118600	-	rph	ribonuclease PH
i02_4130	CDS	4118727	4119590	+	yicC	hypothetical protein
i02_4131	CDS	4119800	4120636	+	dinD	DNA-damage-inducible protein D
i02_4132	CDS	4120874	4121545	+	yicG	hypothetical protein
i02_4133	CDS	4121542	4123230	-	ligB	NAD-dependent DNA ligase LigB
i02_4134	CDS	4123085	4123348	-	/	hypothetical protein
i02_4135	CDS	4123425	4124105	+	gmk	guanylate kinase
i02_4136	CDS	4124160	4124435	+	rpoZ	DNA-directed RNA polymerase subunit omega
i02_4137	CDS	4124454	4126562	+	spoT	bifunctional (p)ppGpp synthetase II
i02_4138	CDS	4126569	4127258	+	spoU	tRNA guanosine-2'-O-methyltransferase
i02_4139	CDS	4127231	4129345	+	recG	ATP-dependent DNA helicase RecG
i02_4140	CDS	4129379	4130584	-	gltS	Sodium/glutamate symport carrier protein
i02_4141	CDS	4130855	4132255	+	yicE	putative purine permease yicE
i02_4142	CDS	4132352	4134085	+	yicH	hypothetical protein
i02_4143	CDS	4134129	4134848	-	/	hypothetical protein
i02_4144	CDS	4134790	4135755	-	/	hypothetical protein
i02_4145	CDS	4135770	4136630	-	/	hypothetical protein
i02_4146	CDS	4136711	4137562	-	/	putative aldolase
i02_4147	CDS	4137574	4138665	-	/	putative PTS enzyme-II fructose
i02_4148	CDS	4138690	4139004	-	/	PTS system, fructose-like-2 IIB component 1
i02_4149	CDS	4139022	4139492	-	/	putative phosphotransferase system (PTS),
i02_4150	CDS	4139519	4141072	-	/	putative transcriptional antiterminator
i02_4151	CDS	4141218	4141343	-	/	hypothetical protein
i02_4152	CDS	4141355	4143673	-	yicI	alpha-xylosidase YicI
i02_4153	CDS	4143683	4145122	-	yicJ	putative transporter
i02_4154	CDS	4145753	4146964	+	intC	putative prophage integrase
i02_4155	CDS	4147163	4148242	-	/	conserved hypothetical protein
i02_4156	CDS	4149042	4150868	-	/	conserved hypothetical protein
i02_4157	CDS	4151346	4152446	-	/	conserved hypothetical protein
i02_4158	CDS	4152900	4153583	+	/	putative transcriptional regulator YfjR
i02_4159	CDS	4153623	4154636	+	/	small GTP-binding domain protein
i02_4160	CDS	4154648	4155433	+	/	conserved hypothetical protein
i02_4161	CDS	4155635	4155853	-	/	hypothetical protein
i02_4162	CDS	4155971	4156585	-	/	hypothetical protein
i02_4163	CDS	4157178	4158131	+	/	conserved hypothetical protein
i02_4164	CDS	4158367	4158807	+	/	hypothetical protein
i02_4165	CDS	4159211	4159684	+	/	DNA repair protein RadC
i02_4166	CDS	4160050	4160475	+	/	hypothetical protein
i02_4167	CDS	4160496	4161893	+	/	ATP binding protein
i02_4168	CDS	4161890	4162621	+	/	conserved hypothetical protein
i02_4169	CDS	4162659	4162793	+	/	hypothetical protein
i02_4170	CDS	4163013	4163132	+	/	hypothetical protein
i02_4171	CDS	4163469	4163630	+	/	hypothetical protein
i02_4172	CDS	4163950	4164171	+	/	hypothetical protein
i02_4173	CDS	4164312	4164809	+	/	hypothetical protein

Locus_tag	Type	start	End	+/-[a]	Gene[b]	Product
i02_4174	CDS	4164922	4165899	+	yicL	putative transport protein YicL
i02_4175	CDS	4165903	4166721	-	nlpA	cytoplasmic membrane lipoprotein-28
i02_4176	CDS	4166718	4166861	-	/	hypothetical protein
i02_4177	CDS	4166874	4167236	+	/	hypothetical protein
i02_4178	CDS	4167277	4168467	-	nepI	ribonucleoside transporter
i02_4179	CDS	4168678	4169157	-	yicN	hypothetical protein
i02_4180	CDS	4169183	4170595	-	yicO	hypothetical protein
i02_4181	CDS	4170692	4172458	+	yicP	cryptic adenine deaminase
i02_4182	CDS	4172505	4173896	-	uhpT	sugar phosphate antiporter
i02_4183	CDS	4174034	4175356	-	uhpC	regulatory protein UhpC
i02_4184	CDS	4175363	4176871	-	uhpB	sensory histidine kinase UhpB
i02_4185	CDS	4176865	4177455	-	uhpA	DNA-binding transcriptional activator UhpA
i02_4186	CDS	4177870	4178730	+	/	hypothetical protein
i02_4187	CDS	4178921	4179211	-	ilvN	acetolactate synthase 1 regulatory subunit
i02_4188	CDS	4179215	4180903	-	ilvB	acetolactate synthase catalytic subunit
i02_4189	CDS	4180887	4181219	+	/	hypothetical protein
i02_4190	CDS	4181502	4182692	+	emrD	multidrug resistance protein D
i02_4191	CDS	4182700	4183197	-	yidF	hypothetical protein
i02_4192	CDS	4183194	4183556	-	yidG	hypothetical protein
i02_4193	CDS	4183546	4183953	-	yidH	hypothetical protein
i02_4194	CDS	4183953	4185446	-	yidJ	putative sulfatase yidJ
i02_4195	CDS	4185443	4187158	-	yidK	putative symporter YidK
i02_4196	CDS	4187295	4188191	+	yidL	putative transcriptional regulator YidL
i02_4197	CDS	4188281	4189966	-	yidE	hypothetical protein
i02_4198	CDS	4189972	4190142	+	/	hypothetical protein
i02_4199	CDS	4190139	4190567	-	ibpB	heat shock chaperone IbpB
i02_4200	CDS	4190679	4191092	-	ibpA	heat shock protein IbpA
i02_4201	CDS	4191323	4191730	+	yidQ	hypothetical protein
i02_4202	CDS	4191732	4192982	-	yidR	hypothetical protein
i02_4203	CDS	4192914	4193042	+	/	hypothetical protein
i02_4204	CDS	4193026	4194147	+	yidS	putative oxidoreductase
i02_4205	CDS	4194108	4195445	-	dgoT	D-galactonate transporter
i02_4206	CDS	4195520	4196668	-	rspA	starvation sensing protein rspA
i02_4207	CDS	4196665	4197282	-	dgoA	2-dehydro-3-deoxy-6-phosphogalactonate aldolase
i02_4208	CDS	4197266	4198144	-	dgoK	2-dehydro-3-deoxygalactonokinase
i02_4209	CDS	4198141	4198830	-	/	hypothetical protein
i02_4210	CDS	4199109	4199765	+	/	hypothetical protein
i02_4212	CDS	4199809	4200621	-	yidA	sugar phosphatase
i02_4213	CDS	4200736	4201143	-	yidB	hypothetical protein
i02_4214	CDS	4201374	4203791	-	gyrB	DNA gyrase subunit B
i02_4215	CDS	4203817	4204890	-	recF	recombination protein F
i02_4216	CDS	4204890	4205990	-	dnaN	DNA polymerase III subunit beta
i02_4217	CDS	4205995	4207410	-	yfgE	DNA replication initiation factor
i02_4218	CDS	4207692	4207892	-	/	hypothetical protein
i02_4219	CDS	4208005	4208145	+	/	50S ribosomal protein L34
i02_4220	CDS	4208162	4208521	+	rnpA	ribonuclease P
i02_4221	CDS	4208745	4210391	+	yidC	putative inner membrane protein translocase
i02_4222	CDS	4210497	4211861	+	trmE	tRNA modification GTPase TrmE
i02_4223	CDS	4212103	4212177	+	tnaL	tryptophanase leader peptide
i02_4224	CDS	4212383	4213813	+	tnaA	tryptophanase
i02_4225	CDS	4213905	4215152	+	tnaB	tryptophan permease TnaB
i02_4226	CDS	4215284	4216459	+	yidY	multidrug efflux system protein MdtL
i02_4227	CDS	4216425	4217393	+	yidZ	DNA-binding transcriptional regulator YidZ
i02_4228	CDS	4217538	4218299	+	yieE	hypothetical protein
i02_4229	CDS	4218321	4218887	+	yieF	hypothetical protein
i02_4230	CDS	4218941	4220278	-	yieG	hypothetical protein
i02_4231	CDS	4220445	4221110	+	yieH	6-phosphogluconate phosphatase
i02_4232	CDS	4221177	4221650	+	yieI	putative inner membrane protein

Locus_tag	Type	start	End	+/-[a]	Gene[b]	Product
i02_4233	CDS	4221732	4222448	-	yieK	putative 6-phosphogluconolactonase
i02_4234	CDS	4222469	4223671	-	yieL	hypothetical protein
i02_4235	CDS	4223665	4225281	-	/	hypothetical protein
i02_4236	CDS	4225367	4226761	-	bglB	6-phospho-beta-glucosidase bglB
i02_4237	CDS	4226780	4228729	-	bglF	beta-glucoside-specific PTS system components
i02_4239	CDS	4228788	4229648	-	bglG	transcriptional antiterminator BglG
i02_4240	CDS	4229690	4229812	+	/	hypothetical protein
i02_4241	CDS	4229910	4230635	-	phoU	transcriptional regulator PhoU
i02_4242	CDS	4230650	4231423	-	pstB	phosphate transporter subunit
i02_4243	CDS	4231514	4232404	-	pstA	phosphate transporter permease subunit PtsA
i02_4244	CDS	4232404	4233363	-	pstC	phosphate transporter permease subunit PstC
i02_4245	CDS	4233450	4234490	-	pstS	phosphate ABC transporter periplasmic
i02_4246	CDS	4234804	4236633	-	glmS	L-glutamine:D-fructose-6-phosphate aminotransferase
i02_4247	CDS	4236794	4238164	-	glmU	glucosamine uridyltransferase
i02_4249	CDS	4238518	4238937	-	atpC	F0F1 ATP synthase subunit epsilon
i02_4250	CDS	4238958	4240340	-	atpD	F0F1 ATP synthase subunit beta
i02_4251	CDS	4240367	4241230	-	atpG	F0F1 ATP synthase subunit gamma
i02_4252	CDS	4241281	4242822	-	atpA	F0F1 ATP synthase subunit alpha
i02_4253	CDS	4242835	4243368	-	atpH	F0F1 ATP synthase subunit delta
i02_4254	CDS	4243009	4243746	+	/	hypothetical protein
i02_4255	CDS	4243383	4243853	-	atpF	F0F1 ATP synthase subunit B
i02_4256	CDS	4243915	4244154	-	atpE	F0F1 ATP synthase subunit C
i02_4257	CDS	4244201	4245016	-	atpB	F0F1 ATP synthase subunit A
i02_4258	CDS	4245025	4245417	-	atpI	F0F1 ATP synthase subunit I
i02_4259	CDS	4246022	4246645	-	gidB	16S rRNA methyltransferase GidB
i02_4260	CDS	4246709	4248598	-	gidA	protein involved in a tRNA modification pathway
i02_4261	CDS	4248977	4249420	-	mioC	flavodoxin
i02_4262	CDS	4249510	4249968	-	asnC	DNA-binding transcriptional regulator AsnC
i02_4263	CDS	4250120	4251112	+	asnA	asparagine synthetase AsnA
i02_4264	CDS	4251117	4252568	-	yieM	hypothetical protein
i02_4265	CDS	4252562	4254058	-	yieN	regulatory ATPase RavA
i02_4266	CDS	4254281	4256149	+	trkD	potassium transport protein Kup
i02_4267	CDS	4256316	4256735	+	rbsD	D-ribose pyranase
i02_4268	CDS	4256743	4258248	+	rbsA	D-ribose transporter ATP binding protein
i02_4269	CDS	4258253	4259218	+	rbsC	ribose ABC transporter permease protein
i02_4270	CDS	4259237	4260133	+	rbsB	D-ribose transporter subunit RbsB
i02_4271	CDS	4260232	4261188	+	rbsK	ribokinase
i02_4272	CDS	4261192	4262184	+	rbsR	transcriptional repressor RbsR
i02_4273	CDS	4262150	4263577	-	yieO	putative transport protein YieO
i02_4274	CDS	4263600	4264292	-	yieP	putative transcriptional regulator YieP
i02_4276	CDS	4270092	4270931	-	yifA	transcriptional regulator HdfR
i02_4277	CDS	4271050	4271388	+	yifE	hypothetical protein
i02_4279	CDS	4271413	4272963	-	yifB	hypothetical protein
i02_4280	CDS	4272954	4273139	-	/	hypothetical protein
i02_4281	CDS	4273524	4275170	+	ilvG	acetolactate synthase 2 catalytic subunit
i02_4282	CDS	4275167	4275430	+	ilvM	acetolactate synthase 2 regulatory subunit
i02_4283	CDS	4275450	4276379	+	ilvE	branched-chain amino acid aminotransferase
i02_4284	CDS	4276444	4278294	+	ilvD	dihydroxy-acid dehydratase
i02_4285	CDS	4278294	4279841	+	ilvA	threonine dehydratase
i02_4286	CDS	4279838	4280812	-	ilvY	DNA-binding transcriptional regulator IlvY
i02_4287	CDS	4280878	4282353	+	ilvC	ketol-acid reductoisomerase
i02_4288	CDS	4282399	4282680	-	ppiC	peptidyl-prolyl cis-trans isomerase C
i02_4289	CDS	4282767	4284788	+	rep	ATP-dependent DNA helicase Rep
i02_4290	CDS	4284835	4286328	-	gppA	guanosine pentaphosphate phosphohydrolase
i02_4291	CDS	4286325	4286447	-	/	hypothetical protein
i02_4292	CDS	4286455	4287720	-	rhlB	ATP-dependent RNA helicase RhlB
i02_4293	CDS	4287746	4288180	+	trxA	thioredoxin

Locus_tag	Type	start	End	+/-[a]	Gene[b]	Product
i02_4294	CDS	4287845	4288237	-	/	hypothetical protein
i02_4295	CDS	4288435	4289766	+	rho	transcription termination factor Rho
i02_4296	CDS	4289776	4289901	+	/	hypothetical protein
i02_4297	CDS	4290006	4291109	+	rfe	undecaprenyl-phosphate
i02_4298	CDS	4291118	4292167	+	wzzE	lipopolysaccharide biosynthesis protein WzzE
i02_4299	CDS	4292205	4293353	+	wecB	UDP-N-acetylglucosamine 2-epimerase
i02_4300	CDS	4293350	4294612	+	wecC	UDP-N-acetyl-D-mannosamine dehydrogenase
i02_4301	CDS	4294612	4295679	+	rffG	dTDP-glucose 4,6-dehydratase
i02_4302	CDS	4295698	4296579	+	rffH	glucose-1-phosphate thymidylyltransferase
i02_4303	CDS	4296557	4297231	+	wecD	TDP-fucosamine acetyltransferase
i02_4304	CDS	4297236	4298366	+	wecE	TDP-4-oxo-6-deoxy-D-glucose transaminase
i02_4305	CDS	4297383	4298417	-	/	hypothetical protein
i02_4306	CDS	4298368	4299618	+	wzxE	WzxE protein
i02_4307	CDS	4299615	4300694	+	/	4-alpha-L-fucosyltransferase
i02_4308	CDS	4300691	4302043	+	wecF	putative common antigen polymerase
i02_4309	CDS	4302046	4302786	+	wecG	putative UDP-N-acetyl-D-mannosaminuronic acid transferase
i02_4310	CDS	4302977	4304362	+	yifK	putative transport protein YifK
i02_4311	CDS	4305048	4306283	+	aslB	putative arylsulfatase regulatory protein
i02_4312	CDS	4306441	4308096	-	aslA	arylsulfatase
i02_4313	CDS	4307993	4308148	+	/	hypothetical protein
i02_4314	CDS	4308775	4309938	-	hemY	putative protoheme IX biogenesis protein
i02_4315	CDS	4309974	4311185	-	hemX	putative uroporphyrinogen III
i02_4316	CDS	4311207	4311947	-	hemD	uroporphyrinogen-III synthase
i02_4317	CDS	4311944	4312906	-	hemC	porphobilinogen deaminase
i02_4318	CDS	4313272	4315818	+	cyaA	adenylate cyclase
i02_4319	CDS	4315858	4316178	-	cyaY	frataxin-like protein
i02_4320	CDS	4316226	4317545	-	/	hypothetical protein
i02_4321	CDS	4317542	4319041	-	/	hypothetical protein
i02_4322	CDS	4319200	4319403	+	/	hypothetical protein
i02_4323	CDS	4319437	4320264	+	dapF	diaminopimelate epimerase
i02_4324	CDS	4320261	4320968	+	yigA	hypothetical protein
i02_4325	CDS	4320965	4321861	+	xerC	site-specific tyrosine recombinase XerC
i02_4326	CDS	4321861	4322577	+	yigB	flavin mononucleotide phosphatase
i02_4327	CDS	4322661	4324823	+	uvrD	DNA-dependent helicase II
i02_4328	CDS	4324878	4325780	-	/	hypothetical protein
i02_4329	CDS	4325863	4326627	-	/	hypothetical protein
i02_4330	CDS	4326640	4326753	+	/	hypothetical protein
i02_4331	CDS	4326997	4327947	+	corA	magnesium/nickel/cobalt transporter CorA
i02_4332	CDS	4327983	4328480	-	/	hypothetical protein
i02_4333	CDS	4328477	4329325	-	/	hypothetical protein
i02_4334	CDS	4329636	4330526	-	rarD	protein rarD
i02_4335	CDS	4330578	4331063	-	yigI	hypothetical protein
i02_4336	CDS	4331209	4332078	+	pldA	phospholipase A
i02_4337	CDS	4332082	4332243	+	/	hypothetical protein
i02_4338	CDS	4332197	4334032	+	recQ	ATP-dependent DNA helicase RecQ
i02_4339	CDS	4334096	4334716	+	/	threonine efflux system
i02_4340	CDS	4334777	4335397	-	rhtB	homoserine/homoserine lactone efflux protein
i02_4341	CDS	4335508	4336530	+	pldB	lysophospholipase L2
i02_4342	CDS	4336421	4337338	+	/	putative sugar phosphatase
i02_4343	CDS	4337339	4338313	+	yigM	membrane protein yigM
i02_4344	CDS	4338201	4339154	-	metR	transcriptional activator protein metR
i02_4345	CDS	4339164	4339289	-	/	hypothetical protein
i02_4346	CDS	4339272	4341533	+	metE	cobalamin-independent homocysteine transmethylase
i02_4347	CDS	4341540	4341665	-	/	hypothetical protein
i02_4348	CDS	4341702	4341962	-	/	hypothetical protein
i02_4349	CDS	4342022	4342279	-	/	hypothetical protein

Locus_tag	Type	start	End	+/-[a]	Gene[b]	Product
i02_4350	CDS	4342276	4342965	-	/	hypothetical protein
i02_4351	CDS	4343644	4343775	+	/	hypothetical protein
i02_4352	CDS	4343882	4344451	+	/	hypothetical protein
i02_4353	CDS	4344493	4346001	+	/	PTS system, glucose-specific IIBC component
i02_4354	CDS	4346052	4348049	+	/	transketolase
i02_4355	CDS	4348079	4348924	-	/	hypothetical protein
i02_4356	CDS	4349125	4350030	-	/	hypothetical protein
i02_4357	CDS	4350108	4351331	-	/	putative permease
i02_4358	CDS	4351357	4351746	-	/	hypothetical protein
i02_4359	CDS	4351763	4352719	-	/	carbamate kinase
i02_4360	CDS	4352712	4354187	-	/	hypothetical protein
i02_4361	CDS	4354133	4355692	-	/	hypothetical protein
i02_4362	CDS	4355787	4356647	-	/	hypothetical protein
i02_4363	CDS	4356653	4357321	-	/	hypothetical protein
i02_4364	CDS	4357287	4357436	-	/	hypothetical protein
i02_4365	CDS	4357431	4357547	+	/	hypothetical protein
i02_4366	CDS	4357578	4358453	-	/	putative carboxymethylenebutenolidase
i02_4367	CDS	4358649	4359413	+	udp	uridine phosphorylase
i02_4368	CDS	4359448	4360938	-	/	hypothetical protein
i02_4369	CDS	4360951	4361460	-	/	hypothetical protein
i02_4370	CDS	4361474	4362448	-	/	hypothetical protein
i02_4371	CDS	4362473	4363102	-	/	2-dehydro-3-deoxy-6-phosphogalactonate aldolase
i02_4372	CDS	4362609	4363133	+	/	putative aldolase
i02_4373	CDS	4363092	4364096	-	/	hypothetical protein
i02_4374	CDS	4364120	4364974	-	/	putative transcriptional regulator
i02_4375	CDS	4365107	4366531	+	yigN	DNA recombination protein RmuC
i02_4376	CDS	4366626	4367381	+	ubiE	ubiquinone/menaquinone biosynthesis protein
i02_4377	CDS	4367350	4368000	+	yigP	hypothetical protein
i02_4378	CDS	4367997	4369637	+	ubiB	putative ubiquinone biosynthesis protein UbiB
i02_4379	CDS	4369674	4369985	+	tatA	twin arginine translocase protein A
i02_4380	CDS	4369989	4370504	+	/	sec-independent translocase
i02_4381	CDS	4370507	4371283	+	yigU	twin-arginine protein translocation system
i02_4382	CDS	4371313	4372107	+	yigW	DNase TatD
i02_4383	CDS	4372104	4372592	-	rfaH	transcriptional activator RfaH
i02_4384	CDS	4372759	4374252	+	yigC	3-octaprenyl-4-hydroxybenzoate decarboxylase
i02_4385	CDS	4374298	4374999	+	fre	FMN reductase
i02_4386	CDS	4375191	4376354	-	fadA	3-ketoacyl-CoA thiolase
i02_4387	CDS	4376364	4378679	-	fadB	multifunctional fatty acid oxidation complex
i02_4388	CDS	4378743	4380074	+	pepQ	proline dipeptidase
i02_4389	CDS	4380071	4380688	+	yigZ	hypothetical protein
i02_4390	CDS	4380727	4382178	+	trkH	potassium transporter
i02_4391	CDS	4382190	4382735	+	hemG	protoporphyrinogen oxidase
i02_4393	CDS	4388228	4388755	-	mobB	molybdopterin-guanine dinucleotide biosynthesis protien
i02_4394	CDS	4388737	4389321	-	mobA	molybdopterin-guanine dinucleotide biosynthesis protien
i02_4395	CDS	4389361	4389660	+	yihD	hypothetical protein
i02_4396	CDS	4389737	4390723	+	yihE	serine/threonine protein kinase
i02_4397	CDS	4390740	4391366	+	dsbA	periplasmic protein disulfide isomerase I
i02_4398	CDS	4391391	4391531	+	/	hypothetical protein
i02_4399	CDS	4391479	4392951	+	yihF	hypothetical protein
i02_4400	CDS	4392992	4393924	-	yihG	putative acyltransferase
i02_4401	CDS	4394044	4394286	-	/	hypothetical protein
i02_4402	CDS	4394288	4397074	+	xni	exonuclease IX
i02_4403	CDS	4397293	4397442	-	/	hypothetical protein
i02_4404	CDS	4397456	4398103	-	engB	ribosome biogenesis GTP-binding protein YsxC
i02_4405	CDS	4398107	4398247	-	/	hypothetical protein

Locus_tag	Type	start	End	+/-[a]	Gene[b]	Product
i02_4406	CDS	4398555	4398701	-	/	hypothetical protein
i02_4407	CDS	4398670	4399179	+	yihI	hypothetical protein
i02_4408	CDS	4399253	4399369	-	/	predicted protein
i02_4409	CDS	4399362	4400741	+	hemN	coproporphyrinogen III oxidase
i02_4410	CDS	4401196	4402605	-	glnG	nitrogen regulation protein NR(I)
i02_4411	CDS	4402617	4403666	-	glnL	nitrogen regulation protein NR(II)
i02_4412	CDS	4403840	4405249	-	glnA	glutamine synthetase
i02_4413	CDS	4405622	4407445	+	yihK	GTP-binding protein
i02_4414	CDS	4407663	4408373	+	yihL	putative transcriptional regulator YihL
i02_4415	CDS	4408381	4409361	+	yihM	hypothetical protein
i02_4416	CDS	4409463	4410728	+	yihN	hypothetical protein
i02_4417	CDS	4410788	4411576	-	yihW	putative transcriptional regulator YihW
i02_4418	CDS	4411616	4412563	-	yihV	putative sugar kinase yihV
i02_4419	CDS	4412484	4413563	+	yihU	oxidoreductase yihU
i02_4421	CDS	4413588	4414475	+	yihT	hypothetical protein
i02_4422	CDS	4414508	4415518	+	/	putative dehydrogenase
i02_4423	CDS	4415588	4417021	+	/	hypothetical protein
i02_4424	CDS	4417120	4418469	+	/	shikimate transporter
i02_4425	CDS	4418402	4419346	+	/	hypothetical protein
i02_4426	CDS	4419466	4420086	+	yihX	phosphatase
i02_4427	CDS	4420080	4420952	+	rbn	ribonuclease BN
i02_4428	CDS	4420949	4421386	+	yihZ	D-tyrosyl-tRNA(Tyr) deacylase
i02_4429	CDS	4421383	4422372	+	yiiD	hypothetical protein
i02_4430	CDS	4422436	4423356	-	/	putative lipase
i02_4431	CDS	4423217	4423420	-	/	putative lipase
i02_4432	CDS	4423574	4423885	+	/	hypothetical protein
i02_4433	CDS	4423886	4424176	+	/	hypothetical protein
i02_4434	CDS	4424535	4424813	+	/	hypothetical protein
i02_4435	CDS	4425182	4425427	+	yiiE	hypothetical protein
i02_4436	CDS	4425643	4426572	-	fdhE	formate dehydrogenase accessory protein FdhE
i02_4437	CDS	4426569	4427204	-	fdoI	formate dehydrogenase-O subunit gamma
i02_4438	CDS	4427201	4428103	-	fdoH	formate dehydrogenase-O beta subunit
i02_4439	pseudo	4428116	4431166	-	fdoG	formate dehydrogenase-O, major subunit
i02_4440	CDS	4430798	4431424	+	/	hypothetical protein
i02_4441	CDS	4431381	4432193	+	fdhD	formate dehydrogenase accessory protein
i02_4442	CDS	4432281	4432835	+	/	hypothetical protein
i02_4443	CDS	4433189	4434562	+	/	putative glycoporin
i02_4444	CDS	4434603	4434917	-	yiiL	hypothetical protein
i02_4445	CDS	4434927	4435844	-	rhaD	rhamnulose-1-phosphate aldolase
i02_4446	CDS	4436212	4437477	-	rhaA	L-rhamnose isomerase
i02_4447	CDS	4437468	4438937	-	rhaB	rhamnulokinase
i02_4448	CDS	4439016	4439165	+	/	hypothetical protein
i02_4449	CDS	4439225	4440061	+	rhaS	transcriptional activator RhaS
i02_4450	CDS	4440024	4440983	+	rhaR	transcriptional activator RhaR
i02_4451	CDS	4440980	4442014	-	rhaT	rhamnose-proton symporter
i02_4452	CDS	4441979	4442146	+	/	hypothetical protein
i02_4453	CDS	4442294	4442920	+	sodA	superoxide dismutase
i02_4454	CDS	4442949	4443077	-	/	hypothetical protein
i02_4455	CDS	4443180	4444163	+	kdgT	2-keto-3-deoxygluconate permease
i02_4456	CDS	4444297	4444986	+	yiiM	hypothetical protein
i02_4457	CDS	4445092	4446465	-	cpxA	two-component sensor protein
i02_4458	CDS	4446462	4447160	-	cpxR	DNA-binding transcriptional regulator CpxR
i02_4459	CDS	4447307	4447810	+	cpxP	periplasmic repressor CpxP
i02_4460	CDS	4447959	4448861	+	fieF	ferrous iron efflux protein F
i02_4461	CDS	4448946	4450004	+	pfkA	6-phosphofructokinase
i02_4462	CDS	4450324	4451313	+	sbp	sulfate transporter subunit
i02_4463	CDS	4451228	4452175	+	cdh	CDP-diacylglycerol pyrophosphatase
i02_4464	CDS	4452230	4452997	-	tpiA	triosephosphate isomerase

Locus_tag	Type	start	End	+/-[a]	Gene[b]	Product
i02_4465	CDS	4453105	4453704	-	yiiQ	hypothetical protein
i02_4466	CDS	4453805	4454245	+	yiiR	hypothetical protein
i02_4467	CDS	4454457	4454756	+	yiiS	hypothetical protein
i02_4468	CDS	4454783	4455211	+	yiiT	universal stress protein UspD
i02_4469	CDS	4455216	4455962	-	fpr	ferredoxin-NADP reductase
i02_4470	CDS	4456059	4457069	-	glpX	fructose 1,6-bisphosphatase II
i02_4471	CDS	4457240	4458853	-	glpK	glycerol kinase
i02_4472	CDS	4458771	4459616	-	glpF	glycerol uptake facilitator protein
i02_4473	CDS	4460023	4460286	+	yiiU	hypothetical protein
i02_4474	CDS	4460371	4460856	-	menG	ribonuclease activity regulator protein RraA
i02_4475	CDS	4460949	4461887	-	menA	1,4-dihydroxy-2-naphthoate
i02_4476	CDS	4461942	4463273	-	hslU	ATP-dependent protease ATP-binding subunit HslU
i02_4477	CDS	4463283	4463813	-	hslV	ATP-dependent protease peptidase subunit
i02_4478	CDS	4463906	4464865	-	ftsN	essential cell division protein FtsN
i02_4479	CDS	4464957	4465988	-	cytR	DNA-binding transcriptional regulator CytR
i02_4480	CDS	4466138	4468408	-	priA	primosome assembly protein PriA
i02_4481	CDS	4468521	4468661	-	/	hypothetical protein
i02_4482	CDS	4468812	4469378	-	yiiX	putative peptidoglycan peptidase
i02_4483	CDS	4469480	4469935	-	metJ	transcriptional repressor protein MetJ
i02_4484	CDS	4469960	4471234	+	metB	cystathionine gamma-synthase
i02_4485	CDS	4471237	4473669	+	metL	bifunctional aspartate kinase II/homoserine
i02_4486	CDS	4473887	4474747	-	/	nucleoside-specific channel-forming protein tsx
i02_4487	CDS	4474824	4476449	-	/	hypothetical protein
i02_4488	CDS	4476484	4478037	-	/	hypothetical protein
i02_4489	CDS	4478267	4479391	-	/	hypothetical protein
i02_4490	CDS	4479518	4481077	+	/	hypothetical protein
i02_4491	CDS	4481459	4482349	+	metF	5,10-methylenetetrahydrofolate reductase
i02_4492	CDS	4482639	4484858	+	katG	peroxidase/catalase HPI
i02_4493	CDS	4484886	4485857	+	yijE	putative transport protein YijE
i02_4494	CDS	4485884	4486501	-	yijF	hypothetical protein
i02_4495	CDS	4486401	4486595	-	/	hypothetical protein
i02_4496	CDS	4486777	4487919	-	gldA	glycerol dehydrogenase
i02_4497	CDS	4487891	4488553	-	talC	fructose-6-phosphate aldolase
i02_4498	CDS	4488565	4491066	-	ptsA	phosphoenolpyruvate-protein phosphotransferase
i02_4499	CDS	4491375	4492454	+	frwC	putative fructose-like permease EIIC subunit 2
i02_4500	CDS	4492469	4492789	+	frwB	putative fructose-like phosphotransferase EIIB
i02_4501	CDS	4492840	4495137	+	pflD	putative formate acetyltransferase 2
i02_4502	CDS	4495034	4495981	+	pflC	pyruvate formate lyase II activase
i02_4503	CDS	4495983	4496324	+	frwD	putative fructose-like phosphotransferase EIIB
i02_4504	CDS	4496311	4497162	-	yijO	putative transcriptional regulator YijO
i02_4505	CDS	4497388	4499121	-	yijP	hypothetical protein
i02_4506	CDS	4499304	4501955	-	ppc	phosphoenolpyruvate carboxylase
i02_4507	CDS	4502257	4503447	-	argE	acetylornithine deacetylase
i02_4508	CDS	4503562	4504566	+	argC	N-acetyl-gamma-glutamyl-phosphate reductase
i02_4509	CDS	4504541	4505350	+	argB	acetylglutamate kinase
i02_4510	CDS	4505411	4506784	+	argH	argininosuccinate lyase
i02_4511	CDS	4507222	4508496	+	/	starvation sensing protein rspA
i02_4512	CDS	4508563	4509846	+	yjiZ	putative transport protein YjjL
i02_4513	CDS	4510053	4511015	+	oxyR	DNA-binding transcriptional regulator OxyR
i02_4514	CDS	4510998	4512398	-	udhA	soluble pyridine nucleotide transhydrogenase
i02_4515	CDS	4512728	4513894	+	/	putative hippuricase
i02_4516	CDS	4513937	4515253	+	/	putative citrate permease
i02_4517	CDS	4515303	4516007	+	yijC	DNA-binding transcriptional repressor FabR
i02_4518	CDS	4516007	4516366	+	yijD	hypothetical protein
i02_4519	CDS	4516406	4517506	-	trmA	tRNA (uracil-5-)-methyltransferase
i02_4520	CDS	4517875	4519719	+	btuB	vitamin B12/cobalamin outer membrane
i02_4521	CDS	4519664	4520521	+	murI	glutamate racemase

Locus_tag	Type	start	End	+/-[a]	Gene[b]	Product
i02_4523	CDS	4526133	4527161	+	murB	UDP-N-acetylenolpyruvoylglucosamine reductase
i02_4524	CDS	4527158	4528123	+	birA	biotin--protein ligase
i02_4525	CDS	4528152	4529123	-	coaA	pantothenate kinase
i02_4526	CDS	4529159	4529293	-	/	hypothetical protein
i02_4527	CDS	4530020	4531204	+	tuf	elongation factor Tu
i02_4528	CDS	4531434	4531817	+	secE	preprotein translocase subunit SecE
i02_4529	CDS	4531819	4532364	+	nusG	transcription antitermination protein NusG
i02_4530	CDS	4532523	4532951	+	rplK	50S ribosomal protein L11
i02_4531	CDS	4532955	4533659	+	rplA	50S ribosomal protein L1
i02_4532	CDS	4533951	4534448	+	rplJ	50S ribosomal protein L10
i02_4533	CDS	4534515	4534880	+	rplL	50S ribosomal protein L7/L12
i02_4534	CDS	4535167	4539228	+	rpoB	DNA-directed RNA polymerase subunit beta
i02_4535	CDS	4539305	4543528	+	rpoC	DNA-directed RNA polymerase subunit beta'
i02_4536	CDS	4543770	4544903	-	thiH	thiamine biosynthesis protein ThiH
i02_4537	CDS	4544900	4545670	-	thiG	thiazole synthase
i02_4538	CDS	4545672	4545872	-	thiS	sulfur carrier protein ThiS
i02_4539	CDS	4545856	4546704	-	thiF	thiamine biosynthesis protein ThiF
i02_4540	CDS	4546604	4547242	-	thiE	thiamine-phosphate pyrophosphorylase
i02_4541	CDS	4547239	4549146	-	thiC	thiamine biosynthesis protein ThiC
i02_4542	CDS	4549367	4549843	-	yjaE	anti-RNA polymerase sigma 70 factor
i02_4543	CDS	4549938	4550711	+	nudC	NADH pyrophosphatase
i02_4544	CDS	4550751	4551815	+	hemE	uroporphyrinogen decarboxylase
i02_4545	CDS	4551825	4552496	+	nfi	endonuclease V
i02_4546	CDS	4552539	4553129	+	yjaG	hypothetical protein
i02_4547	CDS	4553316	4553588	+	hupA	transcriptional regulator HU subunit alpha
i02_4548	CDS	4553595	4554296	+	yjaH	hypothetical protein
i02_4549	CDS	4554298	4554858	-	zraP	zinc resistance protein
i02_4551	CDS	4554955	4556331	+	hydH	sensor protein ZraS
i02_4552	CDS	4556328	4557653	+	hydG	transcriptional regulatory protein ZraR
i02_4553	CDS	4557650	4558939	-	purD	phosphoribosylamine--glycine ligase
i02_4554	CDS	4558951	4560540	-	purH	bifunctional
i02_4555	CDS	4560658	4560780	+	/	hypothetical protein
i02_4556	CDS	4560939	4561100	-	/	hypothetical protein
i02_4558	CDS	4566208	4566627	+	yjaA	hypothetical protein
i02_4559	CDS	4566691	4567134	-	yjaB	hypothetical protein
i02_4560	CDS	4567291	4568220	+	metA	homoserine O-succinyltransferase
i02_4561	CDS	4568471	4570090	+	aceB	malate synthase
i02_4562	CDS	4570105	4571424	+	aceA	isocitrate lyase
i02_4563	CDS	4571640	4573364	+	aceK	bifunctional isocitrate dehydrogenase
i02_4564	CDS	4573381	4574244	-	iclR	transcriptional repressor IclR
i02_4565	CDS	4574390	4578088	+	metH	B12-dependent methionine synthase
i02_4566	CDS	4578142	4578468	+	/	hypothetical protein
i02_4567	CDS	4578511	4578960	+	/	hypothetical protein
i02_4568	CDS	4579131	4580762	+	yjbB	hypothetical protein
i02_4569	CDS	4580852	4581541	-	pepE	peptidase E
i02_4570	CDS	4581923	4583164	-	/	putative oxidoreductase
i02_4571	CDS	4583204	4584058	-	/	PTS system, mannose-specific IID component
i02_4572	CDS	4584039	4584836	-	/	PTS system, mannose-specific IIC component
i02_4573	CDS	4584902	4585396	-	/	putative sorbose PTS component
i02_4574	CDS	4585396	4585803	-	/	putative sorbose PTS component
i02_4575	CDS	4585813	4586619	-	/	sorbitol-6-phosphate 2-dehydrogenase
i02_4576	CDS	4586689	4587720	-	/	putative transcriptional regulator of sorbose
i02_4577	CDS	4587984	4588856	+	yjbC	23S rRNA pseudouridine synthase F
i02_4578	CDS	4588857	4589129	-	yjbD	hypothetical protein
i02_4579	CDS	4589382	4590731	-	lysC	aspartate kinase III
i02_4580	CDS	4591256	4592905	+	pgi	glucose-6-phosphate isomerase
i02_4581	CDS	4593802	4594470	+	yjbF	lipoprotein yjbF precursor
i02_4582	CDS	4594467	4595204	+	yjbG	hypothetical protein

Locus_tag	Type	start	End	+/-[a]	Gene[b]	Product
i02_4583	CDS	4595204	4597300	+	yjbH	lipoprotein yjbH precursor
i02_4584	CDS	4597304	4597465	+	/	hypothetical protein
i02_4585	CDS	4597622	4597744	-	/	hypothetical protein
i02_4586	CDS	4597840	4598250	+	yjbA	phosphate-starvation-inducible protein PsiE
i02_4587	CDS	4598344	4599234	-	malG	maltose transporter permease
i02_4588	CDS	4599249	4600808	-	malF	maltose transporter membrane protein
i02_4589	CDS	4600947	4602137	-	malE	maltose ABC transporter periplasmic protein
i02_4590	CDS	4602412	4603617	+	malK	maltose/maltodextrin transporter
i02_4591	CDS	4603689	4605029	+	lamB	maltoporin
i02_4592	CDS	4605252	4606181	+	malM	maltose regulon periplasmic protein
i02_4593	CDS	4606194	4606346	+	/	hypothetical protein
i02_4594	CDS	4606360	4606857	+	ubiC	chorismate pyruvate lyase
i02_4595	CDS	4606870	4607742	+	ubiA	4-hydroxybenzoate octaprenyltransferase
i02_4596	CDS	4607897	4610380	-	plsB	glycerol-3-phosphate acyltransferase
i02_4597	CDS	4610166	4610528	-	/	hypothetical protein
i02_4598	CDS	4610491	4610859	+	dgkA	diacylglycerol kinase
i02_4599	CDS	4610969	4611577	+	lexA	LexA repressor
i02_4600	CDS	4611596	4612975	+	dinF	DNA-damage-inducible SOS response protein
i02_4601	CDS	4613085	4613300	+	yjbJ	putative stress-response protein
i02_4602	CDS	4613342	4614028	-	yjbK	zinc uptake transcriptional repressor
i02_4603	CDS	4614013	4615008	+	yjbN	tRNA-dihydrouridine synthase A
i02_4604	CDS	4614932	4615384	+	pspG	phage shock protein G
i02_4605	CDS	4615549	4616532	-	qor	quinone oxidoreductase, NADPH-dependent
i02_4606	CDS	4616638	4618773	-	/	putative oxidoreductase
i02_4607	CDS	4618676	4620076	-	/	putative transmembrane transport protein
i02_4608	CDS	4620167	4620946	-	/	putative crotonase
i02_4609	CDS	4620939	4622510	-	/	putative propionate CoA-transferase
i02_4610	CDS	4622739	4623800	+	/	putative regulator
i02_4611	CDS	4623837	4625288	+	dnaB	replicative DNA helicase
i02_4612	CDS	4625323	4626063	-	/	hypothetical protein
i02_4613	CDS	4626010	4627089	+	alr	alanine racemase
i02_4614	CDS	4627112	4627669	+	/	putative transporter
i02_4615	CDS	4627642	4628670	+	/	NadR-like protein
i02_4616	CDS	4628752	4629945	+	tyrB	aromatic amino acid aminotransferase
i02_4617	CDS	4630052	4632871	+	sucA	2-oxoglutarate dehydrogenase E1 component
i02_4618	CDS	4632744	4632944	-	/	hypothetical protein
i02_4619	CDS	4632904	4634058	+	/	dihydrolipoamide succinyltransferase component
i02_4620	CDS	4634120	4635487	+	/	putative 2-oxoglutarate dehydrogenase
i02_4621	CDS	4635509	4636678	+	/	succinyl-CoA synthetase beta chain
i02_4622	CDS	4636691	4637563	+	/	succinyl-CoA synthetase alpha chain
i02_4623	CDS	4637769	4639274	+	/	putative membrane-bound protein
i02_4624	CDS	4639148	4640359	+	/	putative lactate dehydrogenase
i02_4625	CDS	4640348	4641706	-	/	putative c4-dicarboxylate transport
i02_4626	CDS	4641699	4643519	-	/	putative transport sensor protein
i02_4628	CDS	4643491	4643631	-	/	hypothetical protein
i02_4629	CDS	4643727	4644509	+	aphA	acid phosphatase/phosphotransferase
i02_4630	CDS	4644620	4645036	+	yjbQ	hypothetical protein
i02_4631	CDS	4645040	4645396	+	yjbR	hypothetical protein
i02_4632	CDS	4645431	4648253	-	uvrA	excinuclease ABC subunit A
i02_4633	CDS	4648508	4649044	+	ssb	single-stranded DNA-binding protein
i02_4634	CDS	4649143	4649523	-	yjcB	hypothetical protein
i02_4635	CDS	4649511	4649645	+	/	hypothetical protein
i02_4636	CDS	4649844	4651406	+	yjcC	hypothetical protein
i02_4637	CDS	4651442	4651765	-	soxS	DNA-binding transcriptional regulator SoxS
i02_4638	CDS	4651851	4652315	+	soxR	redox-sensitive transcriptional activator SoxR
i02_4639	CDS	4652695	4652919	+	/	hypothetical protein
i02_4640	CDS	4652820	4654169	+	/	hypothetical protein
i02_4641	CDS	4654320	4655969	+	/	putative Na(+)/H(+) exchanger yjcE

Locus_tag	Type	start	End	+/-[a]	Gene[b]	Product
i02_4642	CDS	4656005	4656889	-	/	putative transcriptional regulator
i02_4643	CDS	4656993	4657403	+	/	hypothetical protein
i02_4644	CDS	4657396	4658085	+	/	hypothetical protein
i02_4645	CDS	4658124	4659773	-	actP	acetate permease
i02_4646	CDS	4659770	4660087	-	yjcH	hypothetical protein
i02_4647	CDS	4660164	4660310	+	/	hypothetical protein
i02_4648	CDS	4660478	4662436	-	acs	acetyl-CoA synthetase
i02_4649	CDS	4662710	4662907	+	/	hypothetical protein
i02_4650	CDS	4662828	4664264	+	nrfA	cytochrome c552
i02_4651	CDS	4664303	4664875	+	nrfB	cytochrome c nitrite reductase pentaheme
i02_4652	CDS	4664872	4665543	+	nrfC	NrfC protein
i02_4653	CDS	4665540	4666496	+	nrfD	NrfD protein
i02_4654	CDS	4666552	4668234	+	nrfE	heme lyase subunit NrfE
i02_4655	CDS	4668227	4668610	+	nrfF	formate-dependent nitrite reductase complex
i02_4656	CDS	4668541	4669203	+	nrfG	formate-dependent nitrite reductase complex
i02_4657	CDS	4669338	4669469	-	/	hypothetical protein
i02_4658	CDS	4669546	4670859	+	gltP	glutamate/aspartate:proton symporter
i02_4659	CDS	4671770	4671928	+	/	hypothetical protein
i02_4660	CDS	4671914	4672657	-	yjcO	hypothetical protein
i02_4661	CDS	4672714	4673418	-	yddO	ABC transporter ATP-binding protein
i02_4662	CDS	4673405	4674301	-	/	putative oligopeptide ABC transporter
i02_4663	CDS	4674234	4675067	-	yddQ	ABC transporter permease
i02_4664	CDS	4675067	4676209	-	yddR	ABC transporter permease
i02_4665	CDS	4676216	4677784	-	/	hypothetical protein
i02_4666	pseudo	4678159	4680306	-	fdhF	formate dehydrogenase H
i02_4667	CDS	4679884	4680270	+	/	hypothetical protein
i02_4668	CDS	4680514	4681980	-	yjcP	putative outer membrane efflux protein MdtP
i02_4669	CDS	4681977	4684028	-	yjcQ	multidrug efflux system protein MdtO
i02_4670	CDS	4684028	4685059	-	yjcR	multidrug resistance protein MdtN
i02_4671	CDS	4685078	4685401	-	/	hypothetical protein
i02_4672	CDS	4685562	4687559	-	yjcS	hypothetical protein
i02_4673	CDS	4687820	4688749	-	yjcT	D-allose kinase
i02_4674	CDS	4688733	4689434	-	yjcU	allulose-6-phosphate 3-epimerase
i02_4675	CDS	4689439	4690419	-	yjcV	D-allose transporter subunit
i02_4676	CDS	4690398	4691930	-	yjcW	D-allose transporter ATP-binding protein
i02_4677	CDS	4692057	4692998	-	yjcX	D-allose transporter subunit
i02_4678	CDS	4693051	4693974	-	rpiR	DNA-binding transcriptional repressor RpiR
i02_4679	CDS	4694300	4694749	+	rpiB	ribose-5-phosphate isomerase B
i02_4680	CDS	4694818	4695147	+	/	hypothetical protein
i02_4681	CDS	4695175	4695897	-	phnP	carbon-phosphorus lyase complex accessory
i02_4682	CDS	4695935	4696369	-	phnO	aminoalkylphosphonic acid N-acetyltransferase
i02_4683	CDS	4696356	4696913	-	phnN	ribose 1,5-bisphosphokinase
i02_4684	CDS	4696913	4698049	-	phnM	PhnM protein
i02_4685	CDS	4698046	4698825	-	phnL	phosphonate ABC transporter ATP-binding protein
i02_4686	CDS	4698837	4699595	-	phnK	phosphonate C-P lyase system protein PhnK
i02_4687	CDS	4699592	4700521	-	phnJ	PhnJ protein
i02_4689	CDS	4700430	4701500	-	phnI	PhnI protein
i02_4690	CDS	4701494	4702078	-	phnH	carbon-phosphorus lyase complex subunit
i02_4691	CDS	4702075	4702527	-	phnG	PhnG protein
i02_4692	CDS	4702528	4703262	-	phnF	phosphonate metabolism transcriptional regulator
i02_4693	CDS	4703274	4704116	-	phnE	membrane channel protein component of Pn
i02_4694	CDS	4704159	4705175	-	phnD	phosphonates-binding periplasmic protein
i02_4695	CDS	4705200	4705988	-	phnC	phosphonate/organophosphate ester transporter
i02_4696	CDS	4706121	4706564	-	phnB	hypothetical protein
i02_4698	CDS	4706724	4707206	-	phnA	PhnA protein
i02_4699	CDS	4707462	4709690	+	yjdA	hypothetical protein
i02_4700	CDS	4709687	4710565	+	yjcZ	hypothetical protein
i02_4701	CDS	4710796	4712331	+	proP	proline/glycine betaine transporter

Locus_tag	Type	start	End	+/-[a]	Gene[b]	Product
i02_4702	CDS	4712508	4713608	-	basS	sensor protein BasS/PmrB
i02_4703	CDS	4713609	4714277	-	basR	DNA-binding transcriptional regulator BasR
i02_4704	CDS	4714274	4715947	-	yjdB	putative cell division protein
i02_4705	CDS	4716021	4717358	-	yjdE	arginine:agmatin antiporter
i02_4706	CDS	4717495	4718256	-	adiY	putative regulatory protein adiY
i02_4707	CDS	4718592	4720862	-	adiA	biodegradative arginine decarboxylase
i02_4708	CDS	4721058	4721966	-	melR	DNA-binding transcriptional regulator MelR
i02_4709	CDS	4722249	4723604	+	melA	alpha-galactosidase
i02_4710	CDS	4723601	4723882	-	/	membrane protein
i02_4711	CDS	4723863	4724243	-	yjdF	hypothetical protein
i02_4712	CDS	4724366	4726012	-	fumB	fumarate hydratase class I, anaerobic
i02_4713	CDS	4726090	4727430	-	dcuB	anaerobic C4-dicarboxylate transporter
i02_4714	CDS	4727498	4727656	+	/	hypothetical protein
i02_4715	CDS	4728001	4728720	-	yjdG	DNA-binding transcriptional activator DcuR
i02_4716	CDS	4728717	4730348	-	yjdH	sensory histidine kinase DcuS
i02_4717	CDS	4730340	4730474	+	/	hypothetical protein
i02_4718	CDS	4730529	4730759	+	yjdI	hypothetical protein
i02_4719	CDS	4730800	4731252	-	/	hypothetical protein
i02_4720	CDS	4731272	4731568	+	yjdK	hypothetical protein
i02_4721	CDS	4731596	4731769	+	/	hypothetical protein
i02_4722	CDS	4731785	4731922	+	/	hypothetical protein
i02_4723	CDS	4731888	4733405	-	lysS	lysyl-tRNA synthetase
i02_4724	CDS	4733642	4735099	-	yjdL	putative transporter YjdL
i02_4725	CDS	4735158	4737344	-	cadA	lysine decarboxylase
i02_4726	CDS	4737385	4738719	-	cadB	lysine/cadaverine antiporter
i02_4727	CDS	4739085	4740623	-	cadC	DNA-binding transcriptional activator CadC
i02_4728	CDS	4741422	4742021	-	yjdC	putative transcriptional regulator
i02_4729	CDS	4742034	4743812	-	dipZ	thiol:disulfide interchange protein precursor
i02_4730	CDS	4743707	4744045	-	cutA	divalent-cation tolerance protein CutA
i02_4731	CDS	4744161	4745462	-	dcuA	anaerobic C4-dicarboxylate transporter
i02_4732	CDS	4745580	4747061	-	aspA	aspartate ammonia-lyase
i02_4733	CDS	4747353	4747829	+	fxsA	FxsA
i02_4734	CDS	4747845	4749161	-	yjeH	inner membrane protein YjeH
i02_4735	CDS	4749244	4749567	-	/	hypothetical protein
i02_4736	CDS	4749377	4749670	+	groES	co-chaperonin GroES
i02_4737	CDS	4749714	4751360	+	groEL	chaperonin GroEL
i02_4738	CDS	4751465	4751851	+	yjeI	hypothetical protein
i02_4739	CDS	4751901	4752770	-	yjeJ	hypothetical protein
i02_4740	CDS	4753005	4754033	-	yjeK	hypothetical protein
i02_4741	CDS	4754075	4754641	+	efp	elongation factor P
i02_4742	CDS	4754693	4754818	+	ecnA	entericidin A
i02_4743	CDS	4754929	4755075	+	ecnB	entericidin B membrane lipoprotein
i02_4744	CDS	4755101	4755568	+	sugE	SugE protein
i02_4745	CDS	4755565	4756098	-	blc	outer membrane lipoprotein Blc
i02_4746	CDS	4756187	4757449	-	ampC	beta-lactamase
i02_4747	CDS	4757383	4757772	-	frdD	fumarate reductase subunit D
i02_4748	CDS	4757753	4758148	-	frdC	fumarate reductase subunit C
i02_4749	CDS	4758159	4758893	-	frdB	fumarate reductase iron-sulfur subunit
i02_4750	CDS	4758886	4760694	-	frdA	fumarate reductase flavoprotein subunit
i02_4751	CDS	4760989	4761996	+	yjeA	lysyl-tRNA synthetase
i02_4752	CDS	4762173	4763717	+	yjeM	putative transporter YjeM
i02_4753	CDS	4763868	4767191	-	yjeP	hypothetical protein
i02_4754	CDS	4767111	4768136	+	/	hypothetical protein
i02_4755	CDS	4767213	4768181	-	psd	phosphatidylserine decarboxylase
i02_4756	CDS	4768278	4769330	-	yjeQ	ribosome-associated GTPase
i02_4757	CDS	4769425	4769970	+	yjeR	oligoribonuclease
i02_4758	CDS	4770749	4771954	-	yjeS	putative electron transport protein yjeS
i02_4759	CDS	4771866	4773434	+	yjeF	hypothetical protein

Locus_tag	Type	start	End	+/-[a]	Gene[b]	Product
i02_4760	CDS	4773406	4773867	+	yjeE	putative ATPase
i02_4761	CDS	4773871	4775217	+	amiB	N-acetylmuramoyl-l-alanine amidase II
i02_4762	CDS	4775224	4777074	+	mutL	DNA mismatch repair protein
i02_4763	CDS	4777067	4778017	+	miaA	tRNA dimethylallyltransferase
i02_4764	CDS	4778103	4778411	+	hfq	RNA-binding protein Hfq
i02_4765	CDS	4778487	4779767	+	hflX	putative GTPase HflX
i02_4766	CDS	4779853	4781112	+	hflK	FtsH protease regulator HflK
i02_4767	CDS	4781115	4782119	+	hflC	FtsH protease regulator HflC
i02_4768	CDS	4782201	4782398	+	yjeT	hypothetical protein
i02_4769	CDS	4782502	4783800	+	purA	adenylosuccinate synthetase
i02_4770	CDS	4784005	4784430	+	yjeB	transcriptional repressor NsrR
i02_4771	CDS	4784427	4786910	+	vacB	exoribonuclease R
i02_4772	CDS	4787001	4787732	+	yjfH	23S rRNA (guanosine-2'-O-)-methyltransferase
i02_4773	CDS	4787859	4788260	+	yjfI	hypothetical protein
i02_4774	CDS	4788279	4788977	+	yjfJ	hypothetical protein
i02_4775	CDS	4789027	4789686	+	yjfK	hypothetical protein
i02_4776	CDS	4789623	4790102	+	yjfL	hypothetical protein
i02_4777	CDS	4790112	4790750	+	yjfM	hypothetical protein
i02_4778	CDS	4790753	4791916	+	yjfC	hypothetical protein
i02_4779	CDS	4791982	4793625	+	aidB	isovaleryl CoA dehydrogenase
i02_4780	CDS	4793742	4794044	-	yjfN	hypothetical protein
i02_4781	CDS	4794166	4794594	-	yjfO	hypothetical protein
i02_4782	CDS	4794677	4795426	+	yjfP	esterase
i02_4783	CDS	4795423	4796178	-	yjfQ	transcriptional repressor UlaR
i02_4784	CDS	4796286	4797356	-	yjfR	putative L-ascorbate 6-phosphate lactonase
i02_4785	CDS	4797705	4799102	+	ulaA	ascorbate-specific PTS system enzyme IIC
i02_4786	CDS	4799112	4799423	+	sgaB	L-ascorbate-specific enzyme IIB component
i02_4787	CDS	4799433	4799897	+	ptxA	L-ascorbate-specific enzyme IIA component
i02_4788	CDS	4799911	4800561	+	ulaD	3-keto-L-gulonate-6-phosphate decarboxylase
i02_4789	CDS	4800571	4801425	+	sgaU	L-xylulose 5-phosphate 3-epimerase
i02_4790	CDS	4801425	4802111	+	sgaE	L-ribulose-5-phosphate 4-epimerase
i02_4791	CDS	4802240	4802515	-	yjfY	hypothetical protein
i02_4792	CDS	4802843	4803238	+	rpsF	30S ribosomal protein S6
i02_4793	CDS	4803245	4803559	+	priB	primosomal replication protein N
i02_4794	CDS	4803564	4803791	+	rpsR	30S ribosomal protein S18
i02_4795	CDS	4803833	4804282	+	rplI	50S ribosomal protein L9
i02_4796	CDS	4804684	4805175	+	/	hypothetical protein
i02_4797	CDS	4805178	4805765	+	/	hypothetical protein
i02_4799	CDS	4805836	4806447	+	/	hypothetical protein
i02_4800	CDS	4806590	4807867	-	/	hexuronate transporter
i02_4801	CDS	4807960	4810032	-	/	putative oxidoreductase
i02_4802	CDS	4810029	4811570	-	/	putative acetyl-CoA:acetoacetyl-CoA transferase
i02_4803	CDS	4811580	4812356	-	/	hypothetical protein
i02_4805	CDS	4812366	4813208	-	/	hypothetical protein
i02_4806	CDS	4813218	4814009	-	fabG	3-ketoacyl-(acyl-carrier-protein) reductase
i02_4807	CDS	4814150	4814869	+	/	hypothetical protein
i02_4808	CDS	4814853	4815527	-	ytfB	hypothetical protein
i02_4809	CDS	4815550	4816329	+	fklB	peptidyl-prolyl cis-trans isomerase
i02_4810	CDS	4816639	4818051	+	cycA	D-alanine/D-serine/glycine permease
i02_4811	CDS	4818096	4818758	-	ytfE	iron-sulfur cluster repair di-iron protein
i02_4812	CDS	4818866	4819840	-	ytfF	hypothetical protein
i02_4813	CDS	4819939	4820799	-	ytfG	hypothetical protein
i02_4814	CDS	4820777	4821268	+	ytfH	hypothetical protein
i02_4815	CDS	4821397	4823349	-	/	hypothetical protein
i02_4816	CDS	4823530	4824270	+	cysQ	adenosine-3'(2'),5'-bisphosphate nucleotidase
i02_4817	CDS	4824260	4824817	-	/	hypothetical protein
i02_4818	CDS	4825076	4825348	+	ytfK	hypothetical protein
i02_4819	CDS	4825426	4826769	-	ytfL	hypothetical protein

Locus_tag	Type	start	End	+/-[a]	Gene[b]	Product
i02_4820	CDS	4827092	4827796	-	msrA	methionine sulfoxide reductase A
i02_4821	CDS	4827936	4829669	+	ytfM	hypothetical protein
i02_4822	CDS	4829666	4833445	+	ytfN	hypothetical protein
i02_4823	CDS	4833448	4833789	+	ytfP	hypothetical protein
i02_4824	CDS	4833939	4835411	+	/	hypothetical protein
i02_4825	CDS	4835474	4835614	-	/	hypothetical protein
i02_4826	CDS	4835825	4836355	-	ppa	inorganic pyrophosphatase
i02_4827	CDS	4836367	4836492	-	/	hypothetical protein
i02_4828	CDS	4836665	4837621	+	ytfQ	ABC transporter periplasmic-binding protein
i02_4829	CDS	4837761	4839263	+	ytfR	ABC transporter ATP-binding protein
i02_4830	CDS	4839253	4840299	+	ytfT	ABC transporter permease
i02_4831	CDS	4840286	4841281	+	yjfF	inner membrane ABC transporter permease protein
i02_4832	CDS	4841314	4842375	-	fbp	fructose-1,6-bisphosphatase
i02_4833	CDS	4842375	4842506	+	/	hypothetical protein
i02_4834	CDS	4842488	4843861	+	yjfG	UDP-N-acetylmuramate:L-alanyl-gamma-D-glutamyl-meso-diaminopimelate ligase
i02_4835	CDS	4844019	4844570	-	yjgA	hypothetical protein
i02_4836	CDS	4844664	4846016	+	pmbA	peptidase PmbA
i02_4837	CDS	4846055	4846465	-	/	hypothetical protein
i02_4838	CDS	4846209	4846586	+	cybC	soluble cytochrome b562 precursor
i02_4839	CDS	4846631	4847095	-	nrdG	anaerobic ribonucleotide reductase-activating
i02_4840	CDS	4847253	4849391	-	nrdD	anaerobic ribonucleoside triphosphate reductase
i02_4841	CDS	4849431	4849553	+	/	hypothetical protein
i02_4842	CDS	4849785	4851440	-	treC	trehalose-6-phosphate hydrolase
i02_4843	CDS	4851490	4852911	-	treB	trehalose(maltose)-specific PTS system
i02_4844	CDS	4853030	4853977	-	treR	trehalose repressor
i02_4845	CDS	4854356	4857052	+	mgtA	magnesium-transporting ATPase MgtA
i02_4846	CDS	4857043	4857156	+	/	hypothetical protein
i02_4847	CDS	4857258	4857716	-	yjgF	hypothetical protein
i02_4848	CDS	4857717	4858178	-	pyrI	aspartate carbamoyltransferase regulatory subunit
i02_4849	CDS	4858191	4859126	-	pyrB	aspartate carbamoyltransferase catalytic subunit
i02_4850	CDS	4859130	4859264	-	pyrL	pyrBI operon leader peptide
i02_4851	CDS	4859354	4859908	-	/	hypothetical protein
i02_4852	CDS	4859958	4861406	-	/	hypothetical protein
i02_4853	CDS	4861418	4862422	-	/	ornithine carbamoyltransferase
i02_4854	CDS	4862446	4863378	-	/	carbamate kinase
i02_4855	CDS	4863389	4864612	-	/	arginine deiminase
i02_4856	CDS	4864697	4864831	-	/	hypothetical protein
i02_4857	CDS	4865287	4865739	+	yjgK	hypothetical protein
i02_4858	CDS	4865784	4866788	-	argI	ornithine carbamoyltransferase subunit F
i02_4859	CDS	4866950	4867366	+	yjgD	hypothetical protein
i02_4860	CDS	4867544	4868065	-	/	hypothetical protein
i02_4861	CDS	4868240	4869427	+	/	hypothetical protein
i02_4862	CDS	4869474	4872329	-	valS	valyl-tRNA synthetase
i02_4863	CDS	4872329	4872772	-	holC	DNA polymerase III subunit chi
i02_4864	CDS	4873030	4874541	-	pepA	leucyl aminopeptidase
i02_4865	CDS	4874808	4875908	+	yjgP	hypothetical protein
i02_4866	CDS	4875905	4876990	+	yjgQ	hypothetical protein
i02_4867	CDS	4877151	4878653	-	yjgR	hypothetical protein
i02_4868	CDS	4878731	4879759	-	idnR	L-idonate regulatory protein
i02_4869	CDS	4879796	4881115	-	idnT	Gnt-II system L-idonate transporter
i02_4870	CDS	4881180	4881944	-	idnO	gluconate 5-dehydrogenase
i02_4871	CDS	4881968	4882999	-	idnD	L-idonate 5-dehydrogenase
i02_4872	CDS	4883216	4883779	+	idnK	D-gluconate kinase
i02_4873	CDS	4883783	4884844	-	yjgB	hypothetical protein
i02_4873a	CDS	4885269	4886534	+	/	prophage P4 integrase
i02_4874	CDS	4886796	4887044	+	/	HNH nuclease

Locus_tag	Type	start	End	+/-[a]	Gene[b]	Product
i02_4874a	CDS	4887198	4888196	-	/	conserved hypothetical protein
i02_4874b	CDS	4888341	4889096	+	/	conserved hypothetical protein
i02_4875	CDS	4889093	4889695	+	/	hypothetical protein
i02_4876	CDS	4889707	4893348	+	/	ATPase-like protein
i02_4877	CDS	4893394	4897008	+	/	hypothetical protein
i02_4878	CDS	4897185	4899782	+	/	hypothetical protein
i02_4879	CDS	4899793	4901877	+	/	hypothetical protein
i02_4880	CDS	4903949	4904146	+	/	predicted protein
i02_4881	CDS	4905509	4905715	+	/	hypothetical protein
i02_4882	CDS	4906701	4907915	+	/	transposase IS629
i02_4883	CDS	4908501	4908629	+	/	hypothetical protein
i02_4884	CDS	4908955	4909827	+	/	hypothetical protein
i02_4885	CDS	4909887	4910798	+	/	hypothetical protein
i02_4885a	CDS	4911095	4914016	+	/	antigen 43 precursor (AG43) (Fluffing protein)
i02_4885b	CDS	4914124	4916511	+	/	conserved hypothetical protein
i02_4885c	CDS	4916508	4917413	+	/	conserved hypothetical protein
i02_4885d	CDS	4917410	4918480	+	/	phospholipase, patatin family
i02_4886	CDS	4918820	4919638	+	/	hypothetical protein
i02_4887	CDS	4919730	4920215	+	/	hypothetical protein
i02_4888	CDS	4920261	4920707	+	/	putative radC-like protein yeeS
i02_4889	CDS	4921016	4921438	+	/	hypothetical protein
i02_4890	CDS	4921528	4921902	+	/	hypothetical protein
i02_4891	CDS	4923222	4924373	+	/	prophage P4 integrase
i02_4892	CDS	4924616	4927687	-	/	hypothetical protein
i02_4893	CDS	4927701	4929713	-	/	hypothetical protein
i02_4894	CDS	4929744	4930088	+	/	hypothetical protein
i02_4895	CDS	4929866	4930303	-	/	hypothetical protein
i02_4896	CDS	4930300	4931001	-	/	hypothetical protein
i02_4897	CDS	4930998	4934276	-	/	hypothetical protein
i02_4898	CDS	4934276	4935181	-	/	hypothetical protein
i02_4900	CDS	4935350	4935637	-	/	hypothetical protein
i02_4901	CDS	4935895	4936608	+	/	hypothetical protein
i02_4902	CDS	4937260	4937652	-	/	hypothetical protein
i02_4903	CDS	4937922	4938251	+	/	hypothetical protein
i02_4904	CDS	4938752	4938883	+	/	hypothetical protein
i02_4905	CDS	4939010	4939990	-	/	hypothetical protein
i02_4906	CDS	4940055	4941269	-	/	hypothetical protein
i02_4907	CDS	4941181	4941906	-	/	hypothetical protein
i02_4908	CDS	4942695	4943102	-	/	hypothetical protein
i02_4909	CDS	4943362	4943964	+	fimB	tyrosine recombinase
i02_4910	CDS	4944442	4945038	+	fimE	tyrosine recombinase
i02_4911	CDS	4945462	4946067	+	fimA	Type-1 fimbrial protein, A chain precursor
i02_4912	CDS	4946009	4946671	+	fimI	fimbrin-like protein fimI precursor
i02_4913	CDS	4946708	4947433	+	fimC	chaperone protein fimC precursor
i02_4914	CDS	4947499	4950135	+	fimD	Outer membrane usher protein fimD precursor
i02_4915	CDS	4950142	4950675	+	fimF	FimF protein precursor
i02_4916	CDS	4950682	4951191	+	fimG	FimG protein precursor
i02_4917	CDS	4951202	4952113	+	fimH	FimH protein precursor
i02_4918	CDS	4952355	4953698	-	gntP	fructuronate transporter
i02_4919	CDS	4954038	4955222	+	uxuA	mannonate dehydratase
i02_4920	CDS	4955303	4956763	+	uxuB	D-mannonate oxidoreductase
i02_4921	CDS	4956978	4957751	+	uxuR	DNA-binding transcriptional repressor UxuR
i02_4922	CDS	4958156	4958548	+	yjiD	hypothetical protein
i02_4923	CDS	4958596	4958937	-	/	hypothetical protein
i02_4924	CDS	4958974	4959963	-	yjiE	putative DNA-binding transcriptional regulator
i02_4925	CDS	4959950	4961122	-	iadA	isoaspartyl dipeptidase
i02_4926	CDS	4961135	4961596	-	yjiG	hypothetical protein
i02_4927	CDS	4961593	4962288	-	yjiH	hypothetical protein

Locus_tag	Type	start	End	+/-[a]	Gene[b]	Product
i02_4928	CDS	4962288	4962419	+	/	hypothetical protein
i02_4929	CDS	4962424	4962870	+	yjiI	2'-phosphotransferase-like protein
i02_4930	CDS	4962906	4963079	+	/	hypothetical protein
i02_4931	CDS	4963092	4964270	-	yjiJ	hypothetical protein
i02_4932	CDS	4964338	4965309	-	yjiK	hypothetical protein
i02_4933	CDS	4965263	4965520	-	/	hypothetical protein
i02_4934	CDS	4965517	4966290	-	yjiL	hypothetical protein
i02_4935	CDS	4966294	4967466	-	yjiM	hypothetical protein
i02_4936	CDS	4967561	4968877	-	yjiN	hypothetical protein
i02_4937	CDS	4969026	4969946	+	yfcI	hypothetical protein
i02_4938	CDS	4970226	4972445	+	/	hypothetical protein
i02_4939	CDS	4972492	4972833	-	yjiW	endoribonuclease SymE
i02_4940	CDS	4973054	4974823	-	/	putative restriction modification enzyme S
i02_4941	CDS	4974823	4976346	-	/	putative restriction modification enzyme M
i02_4942	CDS	4976359	4978791	-	/	putative restriction modification enzyme R
i02_4943	CDS	4979048	4980721	+	/	hypothetical protein
i02_4944	CDS	4980790	4981830	-	yjiA	putative GTP-binding protein YjiA
i02_4945	CDS	4981757	4981960	-	/	hypothetical protein
i02_4946	CDS	4982078	4984243	-	yjiY	hypothetical protein
i02_4947	CDS	4984605	4986269	+	tsr	methyl-accepting chemotaxis protein I
i02_4948	CDS	4986312	4987583	-	/	hypothetical protein
i02_4949	CDS	4987580	4988053	-	/	hypothetical protein
i02_4950	CDS	4988117	4989088	-	/	putative C4-dicarboxylate-binding periplasmic
i02_4951	CDS	4989758	4991431	+	/	putative Na(+)/H(+) exchanger yjcE
i02_4952	CDS	4991602	4992507	-	yjjM	hypothetical protein
i02_4953	CDS	4992646	4993668	+	/	hypothetical protein
i02_4954	CDS	4993808	4996099	-	mdoB	phosphoglycerol transferase I
i02_4955	CDS	4996353	4996850	-	yjjA	hypothetical protein
i02_4956	CDS	4996896	4997633	-	dnaC	DNA replication protein DnaC
i02_4957	CDS	4997636	4998175	-	dnaT	primosomal protein DnaI
i02_4958	CDS	4998283	4998756	-	yjjB	hypothetical protein
i02_4959	CDS	4998747	4999580	-	yjjP	hypothetical protein
i02_4960	CDS	5000137	5000862	+	yjjQ	hypothetical protein
i02_4961	CDS	5000820	5001497	+	bglJ	DNA-binding transcriptional activator BglJ
i02_4962	CDS	5001535	5002323	-	fhuF	ferric iron reductase involved in ferric
i02_4963	CDS	5002377	5002700	+	/	hypothetical protein
i02_4964	CDS	5002556	5002711	+	/	hypothetical protein
i02_4965	CDS	5003127	5003249	-	/	hypothetical protein
i02_4966	CDS	5003261	5004292	-	rsmC	16S ribosomal RNA m2G1207 methyltransferase
i02_4967	CDS	5004395	5004808	+	holD	DNA polymerase III subunit psi
i02_4968	CDS	5004777	5005223	+	rimI	ribosomal-protein-alanine N-acetyltransferase
i02_4969	CDS	5005238	5005915	+	yjjG	nucleotidase
i02_4970	CDS	5006006	5007595	+	prfC	peptide chain release factor 3
i02_4971	CDS	5007910	5008593	+	osmY	periplasmic protein
i02_4972	CDS	5008690	5008881	+	/	hypothetical protein
i02_4973	CDS	5008967	5010076	+	yjjU	hypothetical protein
i02_4974	CDS	5010073	5010852	+	yjjV	putative deoxyribonuclease YjjV
i02_4975	CDS	5011069	5011932	-	yjjW	hypothetical protein
i02_4976	CDS	5011904	5013454	-	yjjI	hypothetical protein
i02_4977	CDS	5013430	5013555	+	/	hypothetical protein
i02_4978	CDS	5013688	5014491	+	deoC	deoxyribose-phosphate aldolase
i02_4979	CDS	5014518	5015891	+	deoA	thymidine phosphorylase
i02_4980	CDS	5015943	5017166	+	deoB	phosphopentomutase
i02_4981	CDS	5017166	5017942	+	deoD	purine nucleoside phosphorylase
i02_4982	CDS	5018109	5019440	+	yjjJ	hypothetical protein
i02_4983	CDS	5019441	5020457	-	lplA	lipoate-protein ligase A
i02_4984	CDS	5020485	5021129	-	smp	hypothetical protein
i02_4985	CDS	5021235	5022203	+	serB	phosphoserine phosphatase

Locus_tag	Type	start	End	+/-[a]	Gene[b]	Product
i02_4986	CDS	5022204	5023634	+	sms	DNA repair protein RadA
i02_4987	CDS	5023634	5024887	+	nadR	nicotinamide-nucleotide adenylyltransferase
i02_4988	CDS	5024901	5025311	+	/	hypothetical protein
i02_4989	CDS	5025313	5025597	+	/	hypothetical protein
i02_4990	CDS	5025653	5027320	-	yjjK	putative ABC transporter ATP-binding protein
i02_4991	CDS	5027294	5027458	+	/	hypothetical protein
i02_4992	CDS	5027527	5029464	+	slt	lytic murein transglycosylase
i02_4993	CDS	5029554	5029880	+	trpR	Trp operon repressor
i02_4994	CDS	5029953	5030489	-	yjjX	NTPase
i02_4995	CDS	5030532	5031179	+	gpmB	phosphoglycerate mutase
i02_4996	CDS	5031176	5032045	-	rob	right origin-binding protein
i02_4997	CDS	5032256	5032729	+	creA	hypothetical protein
i02_4998	CDS	5032751	5033431	+	creB	DNA-binding response regulator CreB
i02_4999	CDS	5033431	5034855	+	creC	sensory histidine kinase CreC
i02_5000	CDS	5034913	5036265	+	creD	hypothetical protein
i02_5001	CDS	5036325	5037041	-	arcA	two-component response regulator
i02_5002	CDS	5037137	5037277	+	/	hypothetical protein
i02_5003	pseudo	5037677	5038362	+	lasT	putative RNA methyltransferase

[a] +/-, orientation as annotated.

[b] Gene name from CFT073 or APEC O1 genome annotation.
