# Supplementary material for: Rates of Mutation and Host Transmission for an Escherichia coli Clone over 3 Years
Source: PLoS One. 2011 Oct 27;6(10):e26907. doi: 10.1371/journal.pone.0026907 (PMC3203180; doi:10.1371/journal.pone.0026907)
Supplement: Table S2 — The orthologs of clone D and CFT073 genomes. Genes present in 1 or both of the genomes are listed with the gene tag number, gene name and product. (PDF) [file pone.0026907.s003.pdf]

Table S2 The orthologs of clone D and CFT073 genomes. Genes present in 1 or both of the genomes are listed with the gene tag numbers, gene name and product[a].

Clone D	CFT 073	Gene	Product
i02_5004	c5491	thrL	thr operon leader peptide
-	c0002	/	hypothetical protein
i02_0001	c0003	thrA	bifunctional aspartokinase I/homeserine
i02_0002	c0004	thrB	homoserine kinase
i02_0003	c0005	thrC	threonine synthase
i02_0004	c0006	/	hypothetical protein
i02_0005	c0007	yaaX	hypothetical protein
	c0008	/	hypothetical protein
	c0009	/	hypothetical protein
i02_0006	c0010	yaaA	hypothetical protein
i02_0007	c0011	yaaJ	putative transporter yaaJ
i02_0008	c0012	talB	transaldolase B
i02_0009	c0013	mogA	molybdenum cofactor biosynthesis protein
i02_0010	c0015	yaaH	hypothetical protein
i02_0011	c0016	yaaW	hypothetical protein
i02_0012	c0017	yaaI	hypothetical protein
	c0018	/	putative glutamate dehydrogenase
i02_0013	c0019	dnaK	molecular chaperone DnaK
i02_0014	c0020	dnaJ	chaperone protein DnaJ
i02_0015	c0021	/	hypothetical protein
i02_0016	c0022	/	hypothetical protein
i02_0017	c0023	/	hypothetical protein
i02_0018	c0024	nhaA	pH-dependent sodium/proton antiporter
i02_0019	c0025	nhaR	transcriptional activator NhaR
i02_0020	c0026	/	hypothetical protein
i02_0021	c0027	rpsT	30S ribosomal protein S20
i02_0022	c0028	yaaY	hypothetical protein
i02_0023	c0029	ribF	bifunctional riboflavin kinase/FMN
i02_0024	c0030	ileS	isoleucyl-tRNA synthetase
i02_0025	c0031	lspA	lipoprotein signal peptidase
i02_0026	c0032	slpA	FKBP-type 16 kDa peptidyl-prolyl cis-trans
i02_0027	c0033	ispH	1-hydroxy-2-methyl-2-(E)-butenyl 4-diphosphate reductase
i02_0028		rihC	ribonucleoside hydrolase RihC
i02_0029	c0036	/	hypothetical protein
i02_0030	c0037	dapB	dihydrodipicolinate reductase
i02_0031	c0038	/	hypothetical protein
i02_0032	c0039	/	hypothetical protein
i02_0033	c0040	carA	carbamoyl phosphate synthase small subunit
i02_0034	c0041	carB	carbamoyl phosphate synthase large subunit
i02_0036	c0043	caiF	DNA-binding transcriptional activator CaiF
i02_0035	c0042	/	hypothetical protein
i02_0037	c0044	caiE	carnitine operon protein CaiE
i02_0038	c0045	caiD	carnitinyl-CoA dehydratase
i02_0039	c0046	caiC	putative crotonobetaine/carnitine-CoA ligase
i02_0040	c0047	caiB	crotonobetainyl-CoA:carnitine CoA-transferase
i02_0041	c0048	caiA	crotonobetainyl-CoA dehydrogenase

Clone D	CFT 073	Gene	Product
i02_0042	c0049	caiT	L-carnitine/gamma-butyrobetaine antiporter
i02_0043	c0050	fixA	putative electron transfer flavoprotein FixA
i02_0044		fixB	putative electron transfer flavoprotein FixB
i02_0045	c0053	fixC	putative oxidoreductase FixC
i02_0046	c0054	fixX	ferredoxin-like protein
i02_0047	c0055	yaaU	metabolite transporter
i02_0048	c0056	yabF	glutathione-regulated potassium-efflux system
i02_0049	c0057	kefC	glutathione-regulated potassium-efflux system
i02_0050	c0058	folA	dihydrofolate reductase
i02_0051	c0059	/	putative antitoxin of gyrase inhibiting
i02_0052	c0060	/	putative toxin of gyrase inhibiting
	c0062	/	hypothetical protein
i02_0053	c0061	apaH	diadenosine tetraphosphatase
i02_0054	c0063	apaG	ApaG
i02_0055	c0064	ksgA	dimethyladenosine transferase
i02_0056	c0065	pdxA	4-hydroxythreonine-4-phosphate dehydrogenase
i02_0057	c0066	surA	peptidyl-prolyl cis-trans isomerase SurA
i02_0058	c0067	imp	organic solvent tolerance protein
i02_0059	c0068	djlA	Dna-J like membrane chaperone protein
i02_0060	c0069	yabO	23S rRNA/tRNA pseudouridine synthase A
i02_0061	c0070	hepA	ATP-dependent helicase HepA
i02_0062	c0071	polB	DNA polymerase II
-	c0072	/	transposase
i02_0063	c0073	araD	L-ribulose-5-phosphate 4-epimerase
i02_0064	c0074	araA	L-arabinose isomerase
i02_0065	c0075	araB	ribulokinase
i02_0066	c0076	araC	DNA-binding transcriptional regulator AraC
i02_0067	c0077	/	hypothetical protein
i02_0068	c0078	/	hypothetical protein
i02_0069	c0079	/	hypothetical protein
i02_0070	c0080	/	hypothetical protein
i02_0071	c0081	yabI	hypothetical protein
i02_0072	c0082	thiQ	thiamine transporter ATP-binding subunit
i02_0073	c0083	thiP	thiamine transporter membrane protein
i02_0074	c0084	tbpA	thiamine transporter substrate binding subunit
i02_0075		/	hypothetical protein
i02_0076	c0085	yabN	transcriptional regulator SgrR
i02_0077		/	hypothetical protein
-	c0086	/	transposase
	c0088	/	hypothetical protein
i02_0078	c0087	leuD	isopropylmalate isomerase small subunit
i02_0079	c0089	leuC	isopropylmalate isomerase large subunit
i02_0080	c0090	leuB	3-isopropylmalate dehydrogenase
i02_0081	c0091	leuA	2-isopropylmalate synthase
i02_0082	c5492	leuL	leu operon leader peptide
	c0092	/	hypothetical protein
i02_0083	c0093	leuO	leucine transcriptional activator
i02_0084	c0094	/	hypothetical protein
i02_0085	c0095	ilvI	acetolactate synthase 3 catalytic subunit
i02_0086	c0096	ilvH	acetolactate synthase 3 regulatory subunit
	c5493	fruL	hypothetical protein
i02_0087	c0097	/	hypothetical protein

Clone D	CFT 073	Gene	Product
i02_0088	c0098	fruR	DNA-binding transcriptional regulator FruR
i02_0089		/	hypothetical protein
i02_0090	c0099	yabB	cell division protein MraZ
i02_0091	c0100	mraW	S-adenosyl-methyltransferase MraW
i02_0092	c0101	ftsL	cell division protein FtsL
i02_0093	c0102	ftsI	peptidoglycan synthetase ftsI precursor
i02_0094	c0103	murE	UDP-N-acetylmuramoylalanyl-D-glutamate 2,6-diaminopimelate ligase
i02_0095	c0104	murF	D-alanyl-D-alanine-adding enzyme
i02_0096	c0105	mraY	phospho-N-acetylmuramoyl-pentapeptide transferase
i02_0097	c0106	murD	UDP-N-acetylmuramoyl-L-alanine:D-glutamate ligase
i02_0098	c0107	ftsW	cell division protein FtsW
i02_0099	c0108	murG	N-acetylglucosaminyl transferase
i02_0100	c0109	murC	UDP-N-acetylmuramate--L-alanine ligase
i02_0101	c0110	ddl	D-alanine--D-alanine ligase
i02_0102	c0111	ftsQ	cell division protein FtsQ
i02_0103	c0112	ftsA	cell division protein FtsA
i02_0104	c0113	ftsZ	cell division protein FtsZ
i02_0105	c0114	lpxC	UDP-3-O-[3-hydroxymyristoyl] N-acetylglucosamine
i02_0106	c0115	yacA	SecA regulator SecM
i02_0107	c0116	secA	preprotein translocase subunit SecA
i02_0108	c0117	mutT	nucleoside triphosphate pyrophosphohydrolase
i02_0109	c0118	/	hypothetical protein
i02_0110	c0119	/	transposase insK
	c0120	/	hypothetical protein
i02_0111	c0121	yacG	zinc-binding protein
i02_0112	c0122	yacF	hypothetical protein
i02_0113	c0123	coaE	dephospho-CoA kinase
i02_0114	c0124	guaC	guanosine 5'-monophosphate oxidoreductase
i02_0115	c0125	hofC	type IV pilin biogenesis protein
i02_0116	c0126	hofB	hypothetical protein
i02_0117	c0127	ppdD	putative major pilin subunit
i02_0118	c0128	nadC	quinolinate phosphoribosyltransferase
i02_0119	c0129	ampD	N-acetyl-anhydromuranmyl-L-alanine amidase
i02_0120	c0130	ampE	regulatory protein AmpE
i02_0121	c0131	aroP	aromatic amino acid transporter
i02_0122		usp	uropathogenic specific protein
i02_0123	c0133	/	hypothetical protein
i02_0124	c0134	/	hypothetical protein
i02_0125		/	hypothetical protein
i02_0126	c0136	/	hypothetical protein
i02_0127	c0137	/	hypothetical protein
-	c0138	/	hypothetical protein
-	c0139	/	transposase IS629
i02_0128	c0140	pdhR	transcriptional regulator PdhR
i02_0130	c0142	aceE	pyruvate dehydrogenase subunit E1
i02_0129	c0141	/	hypothetical protein
i02_0131		aceF	dihydrolipoamide acetyltransferase
i02_0132	c0145	lpdA	dihydrolipoamide dehydrogenase

Clone D	CFT 073	Gene	Product
i02_0133	c0146	yacH	hypothetical protein
i02_0134	c0147	acnB	bifunctional aconitate hydratase
i02_0135	c0148	yacL	hypothetical protein
i02_0136	c0149	speD	S-adenosylmethionine decarboxylase
i02_0137	c0150	speE	spermidine synthase
i02_0139	c0152	yacK	multicopper oxidase
i02_0138	c0151	yacC	hypothetical protein
i02_0140	c0153	gcd	glucose dehydrogenase
i02_0141	c0154	hpt	hypoxanthine-guanine phosphoribosyltransferase
i02_0142	c0155	yadF	carbonic anhydrase
i02_0143	c0156	yadG	ABC transporter ATP-binding protein
i02_0144	c0157	yadH	hypothetical protein
i02_0145	c0158	yadI	putative PTS system IIA component yadI
i02_0146	c0159	yadE	hypothetical protein
i02_0148	c0161	/	hypothetical protein
i02_0147	c0160	panD	aspartate alpha-decarboxylase
i02_0149	c0162	yadD	hypothetical protein
i02_0150	c0163	/	hypothetical protein
i02_0151	c0164	panC	pantoate--beta-alanine ligase
i02_0152	c0165	panB	3-methyl-2-oxobutanoate
i02_0153	c0166	yadC	putative fimbrial-like adhesin protein
i02_0154	c0167	yadK	hypothetical protein
i02_0155	c0168	yadL	hypothetical protein
i02_0156	c0169	yadM	hypothetical protein
i02_0157	c0170	htrE	putative outer membrane usher protein
i02_0158	c0171	ecpD	putative chaperone protein EcpD
	c0173	/	hypothetical protein
i02_0159	c0172	yadN	fimbrial-like protein yadN precursor
	c0174	/	hypothetical protein
i02_0160	c0175	folK	2-amino-4-hydroxy-6-hydroxymethyldihyropteridine
i02_0161	c0176	pcnB	poly(A) polymerase I
i02_0162	c0177	yadB	glutamyl-Q tRNA(Asp) synthetase
i02_0163	c0178	dksA	DnaK transcriptional regulator DksA
i02_0164	c0179	sfsA	sugar fermentation stimulation protein A
i02_0166	c0181	hrpB	ATP-dependent RNA helicase HrpB
i02_0165	c0180	yadP	2'-5' RNA ligase
i02_0168	c0183	mrcB	penicillin-binding protein 1b
i02_0167	c0182	/	hypothetical protein
i02_0170	c0185	fhuA	ferrichrome outer membrane transporter
i02_0169	c0184	/	hypothetical protein
i02_0171	c0186	fhuC	iron-hydroxamate transporter ATP-binding subunit
i02_0172	c0187	fhuD	iron-hydroxamate transporter substrate-binding subunit
i02_0173	c0188	fhuB	iron-hydroxamate transporter permease subunit
i02_0174	c0189	hemL	glutamate-1-semialdehyde aminotransferase
i02_0175	c0190	yadQ	chloride channel protein
i02_0176	c0191	yadR	iron-sulfur cluster insertion protein ErpA
i02_0177	c0192	/	hypothetical protein
i02_0178	c0193	yadS	hypothetical protein
i02_0179	c0194	yadT	vitamin B12-transporter protein BtuF
i02_0180	c0195	pfs	5'-methylthioadenosine/S-adenosylhomocysteine

Clone D	CFT 073	Gene	Product
i02_0181	c0196	dgt	deoxyguanosinetriphosphate triphosphohydrolase
i02_0182	c0197	htrA	serine endoprotease
-	c0198	/	transposase
i02_0183	c0199	yaeG	carbohydrate diacid transcriptional activator
i02_0184	c0200	yaeH	hypothetical protein
i02_0185	c0201	dapD	2,3,4,5-tetrahydropyridine-2,6-carboxylate
i02_0186	c0202	glnD	PII uridylyl-transferase
i02_0187	c0203	map	methionine aminopeptidase
i02_0188	c0204	rpsB	30S ribosomal protein S2
i02_0190	c0206	tsf	elongation factor Ts
i02_0189	c0205	/	hypothetical protein
i02_0191	c0207	pyrH	uridylate kinase
i02_0192	c0208	frr	ribosome recycling factor
i02_0193		dxr	1-deoxy-D-xylulose 5-phosphate reductoisomerase
i02_0194	c0211	yaeS	undecaprenyl pyrophosphate synthase
i02_0195	c0212	cdsA	CDP-diglyceride synthase
i02_0196	c0213	yaeL	zinc metallopeptidase RseP
i02_0197	c0214	yaeT	outer membrane protein assembly factor YaeT
i02_0198	c0215	hlpA	periplasmic chaperone
i02_0199	c0216	lpxD	UDP-3-O-[3-hydroxymyristoyl] glucosamine
i02_0200	c0217	fabZ	(3R)-hydroxymyristoyl-ACP dehydratase
i02_0201	c0218	lpxA	UDP-N-acetylglucosamine acyltransferase
i02_0202	c0219	lpxB	lipid-A-disaccharide synthase
i02_0203	c0220	rnhB	ribonuclease HII
i02_0204	c0221	dnaE	DNA polymerase III subunit alpha
	c0222	/	hypothetical protein
i02_0205	c0223	accA	acetyl-CoA carboxylase carboxyltransferase
i02_0206	c0224	ldcC	lysine decarboxylase
i02_0207	c0225	yaeR	hypothetical protein
i02_0208	c0226	tilS	tRNA(Ile)-lysidine synthetase
i02_0209	c0227	yaeO	Rho-binding antiterminator
i02_0210	c0228	/	hypothetical protein
i02_0211	c0229	yaeQ	hypothetical protein
i02_0212	c0230	yaeJ	peptidyl-tRNA hydrolase domain protein
i02_0213	c0231	cutF	outer membrane lipoprotein NlpE, involved in surface sensing
i02_0214	c0232	/	transposase insK
i02_0215	c0233	/	hypothetical protein
i02_0216	c0234	yaeF	hypothetical protein
i02_0217	c0235	proS	prolyl-tRNA synthetase
i02_0218	c0236	yaeB	hypothetical protein
i02_0219	c0237	rcsF	outer membrane lipoprotein
i02_0220	c0238	metQ	DL-methionine transporter substrate-binding
i02_0221	c0239	yaeE	DL-methionine transporter permease subunit
i02_0222	c0240	metN	DL-methionine transporter ATP-binding subunit
i02_0223	c0241	yaeD	D,D-heptose 1,7-bisphosphate phosphatase
i02_0224		/	hypothetical protein
i02_0226	c0244	dkgB	2,5-diketo-D-gluconate reductase B
i02_0227	c0245	yafC	putative transcriptional regulator YafC
i02_0228	c0246	yafD	hypothetical protein
i02_0229	c0247	yafE	hypothetical protein

Clone D	CFT 073	Gene	Product
i02_0230	c0248	mltD	membrane-bound lytic murein transglycosylase D
i02_0231	c0249	gloB	hydroxyacylglutathione hydrolase
i02_0232	c0250	yafS	hypothetical protein
i02_0234	c0252	dnaQ	DNA polymerase III subunit epsilon
i02_0233	c0251	rnhA	ribonuclease H
i02_0235	c0253	/	hypothetical protein
	c0254	/	hypothetical protein
i02_0236	c0255	/	hypothetical protein
i02_0237	c0256	/	hypothetical protein
i02_0238	c0257	/	hypothetical protein
i02_0239	c0258	/	hypothetical protein
i02_0240	c0259	/	hypothetical protein
i02_0241	c0261	/	transposase
	c0260	/	hypothetical protein
i02_0242	-	/	hypothetical protein
i02_0243	-	/	putative integrase of prophage
i02_0244	-	/	hypothetical protein
i02_0245	-	/	hypothetical protein
i02_0246	-	/	hypothetical protein
i02_0247	-	/	hypothetical protein
i02_0248	-	/	hypothetical protein
i02_0249	-	/	hypothetical protein
i02_0250	-	/	hypothetical protein
i02_0251	-	/	hypothetical protein
i02_0252	-	/	putative single stranded DNA-binding protein
i02_0253	-	/	hypothetical protein
i02_0254	-	/	putative capsid protein of prophage
i02_0255	-	/	hypothetical protein
i02_0256	-	/	hypothetical protein
i02_0257	-	/	conserved hypothetical protein
i02_0258	-	/	gp1
i02_0259	-	/	hypothetical protein
i02_0260	c0261	/	transposase
i02_0261	c0262	/	hypothetical protein
i02_0262	c0263	/	putative transposase
i02_0263	c0264	/	hypothetical protein
i02_0264	c0265	/	hypothetical protein
-	c0268	/	hypothetical protein
i02_0265	c0269	/	hypothetical protein
i02_0266	c0270	/	hypothetical protein
i02_0267	c0271	/	hypothetical protein
	c0272	/	hypothetical protein
	c0274	/	hypothetical protein
i02_0268	c0273	yeeS	putative radC-like protein yeeS
i02_0269	c0275	/	hypothetical protein
	c0277	/	hypothetical protein
i02_0270	c0276	/	hypothetical protein
i02_0271	c0278	/	hypothetical protein
i02_0272	c0279	/	hypothetical protein
i02_0273	c0280	/	hypothetical protein
i02_0274		/	hypothetical protein
i02_0275	c0281	/	hypothetical protein

Clone D	CFT 073	Gene	Product
i02_0276	c0282	/	hypothetical protein
i02_0277	c0283	/	hypothetical protein
i02_0278	c0284	/	hypothetical protein
i02_0279	c0285	/	hypothetical protein
i02_0280	c0286	/	hypothetical protein
i02_0280	c0287	/	hypothetical protein
i02_0281	c0288	/	hypothetical protein
i02_0281	c0289	/	hypothetical protein
i02_0282	c0290	/	hypothetical protein
i02_0283	c0291	/	hypothetical protein
i02_0284		/	conserved hypothetical protein
i02_0285	c0293	/	hypothetical protein
i02_0286	c0294	/	hypothetical protein
i02_0287	c0295	/	hypothetical protein
i02_0288	c0296	/	hypothetical protein
i02_0289	c0297	/	hypothetical protein
i02_0290	c0298	/	hypothetical protein
i02_0291	c0299	/	hypothetical protein
	c0300	/	hypothetical protein
i02_0292	c0301	/	hypothetical protein
i02_0293	c0302	/	hypothetical protein
i02_0294	c0303	/	hypothetical protein
i02_0295	c0304	/	hypothetical protein
i02_0296	c0305	/	hypothetical protein
	c0306	/	hypothetical protein
i02_0297	c0307	/	hypothetical protein
i02_0298	c0308	/	hemolysin expression modulating protein
i02_0299	c0309	/	hypothetical protein
i02_0300	c0310	/	hypothetical protein
i02_0301	c0311	/	hypothetical protein
i02_0302	c0312	/	hypothetical protein
i02_0303	c0313	/	hypothetical protein
i02_0304	c0314	/	hypothetical protein
i02_0305	c0315	/	hypothetical protein
	c0317	/	hypothetical protein
i02_0306	c0316	/	hypothetical protein
i02_0307	c0318	/	hypothetical protein
i02_0308	c0319	/	putative oligogalacturonide lyase
	c0320	/	hypothetical protein
i02_0309	c0321	/	gluconate 5-dehydrogenase
i02_0310	c0322	/	putative oligogalacturonide transporter
i02_0311	c0323	/	putative exopolygalacturonate lyase
	c0324	/	hypothetical protein
i02_0313	c0325	/	hypothetical protein
i02_0314	c0326	/	hypothetical protein
i02_0315	c0327	/	hypothetical protein
i02_0316	c0328	/	hypothetical protein
i02_0317	c0329	/	hypothetical protein
i02_0318	c0330	/	putative deoxyribose operon repressor
i02_0319	c0331	/	putative ribokinase
i02_0320	c0332	/	putative L-fucose permease
i02_0321	c0333	/	putative cytoplasmic protein

Clone D	CFT 073	Gene	Product
i02_0322	c0334	ulaA	ascorbate-specific PTS system enzyme IIC
i02_0323	c0335	/	hypothetical protein
i02_0324	c0336	/	PTS system, mannitol (Cryptic)-specific IIA
i02_0325	c0337	/	hypothetical protein
i02_0326	c0338	/	hypothetical protein
i02_0327	c0339	/	hypothetical protein
i02_0328	c0340	/	hypothetical protein
i02_0329	c0341	/	hypothetical protein
i02_0330		/	hypothetical protein
i02_0331	c0345	/	ShlA/HecA/FhaA exofamily protein
	c0348	/	hypothetical protein
i02_0332	c0349	/	transposase
i02_0333		/	PefB protein
i02_0334	c0350	/	Pic serine protease precursor
i02_0335	c0351	/	hypothetical protein
-	c0352	/	partial transposase
-	c0354	/	hypothetical protein
	c0355	/	hypothetical protein
i02_0336	c0357	/	hypothetical protein
i02_0337	c0358	/	hypothetical protein
	c0359	/	hypothetical protein
i02_0339	c0360	/	hypothetical protein
i02_0340	c0361	/	putative cytoplasmic membrane export protein
i02_0341	c0362	/	putative membrane spanning export protein
i02_0342	c0363	/	RTX family exoprotein A gene
i02_0343	c0364	/	hypothetical protein
i02_0344	c0365	/	hypothetical protein
i02_0345	c0366	/	hypothetical protein
i02_0346	c0367	/	hypothetical protein
i02_0347		/	hypothetical protein
i02_0348	c0368	/	hypothetical protein
i02_0349	c0369	yafV	hypothetical protein
i02_0350	c0370	ykfE	C-lysozyme inhibitor
i02_0351	c0371	fadE	acyl-CoA dehydrogenase
i02_0352	c0372	gmhA	phosphoheptose isomerase
i02_0353	c0373	yafJ	hypothetical protein
i02_0354	c0374	yafK	hypothetical protein
	c0375	/	hypothetical protein
i02_0355	c0376	yafL	lipoprotein yafL precursor
i02_0357	c0378	mbhA	hypothetical protein
i02_0356	c0377	fhiA	FhiA protein
i02_0358	c0379	dinP	DNA polymerase IV
i02_0359	c0380	yafP	hypothetical protein
i02_0360	c0381	/	hypothetical protein
i02_0361	c0382	prfH	peptide chain release factor-like protein
i02_0362	c0383	pepD	aminoacyl-histidine dipeptidase
i02_0363	c0384	gpt	xanthine-guanine phosphoribosyltransferase
i02_0365	c0386	frsA	fermentation/respiration switch protein
i02_0364	c0385	/	hypothetical protein
i02_0366	c0387	crl	DNA-binding transcriptional regulator Crl
i02_0367	c0388	phoE	outer membrane phosphoporin protein
i02_0368	c0389	proB	gamma-glutamyl kinase

Clone D	CFT 073	Gene	Product
i02_0369	c0390	proA	gamma-glutamyl phosphate reductase
i02_0370	c0391	/	CP4-like integrase
i02_0371	c0392	/	hypothetical protein
i02_0372	c0393	/	haemoglobin protease
i02_0373	c0394	/	hypothetical protein
i02_0374	c0395	/	hypothetical protein
i02_0375	c0396	/	insertion element IS1 1/2/3/5/6 protein insA
i02_0377	c0397	/	InsB protein
i02_0376		/	hypothetical protein
i02_0378	c0398	/	hypothetical protein
i02_0379	c0399	yagU	hypothetical protein
i02_0380		ykgJ	putative ferredoxin
i02_0381	c0400	yagV	hypothetical protein
i02_0382	c0401	yagW	hypothetical protein
i02_0383	c0402	yagX	hypothetical protein
i02_0384	c0403	yagY	hypothetical protein
i02_0385	c0404	matB	hypothetical protein
i02_0386	c0405	matA	hypothetical protein
i02_0387		/	hypothetical protein
i02_0388	c0406	rpmJ	50S ribosomal protein L36
i02_0389	c0407	rpmE2	50S ribosomal protein L31 type B
i02_0390		/	hypothetical protein
i02_0391	c0408	/	hypothetical protein
i02_0391	c0409	/	putative oxidoreductase
i02_0392	c0410	/	hypothetical protein
i02_0393	c0411	/	LysR family transcriptional regulator
i02_0394	c0412	/	putative transcriptional regulator YcjZ
i02_0395	c0413	/	putative aldo/keto reductase
i02_0396	c0414	/	2,5-diketo-D-gluconic acid reductase A
i02_0397	c0415	eaeH	putative adhesin
i02_0398	c0416	ykgA	putative transcriptional regulator YkgA
i02_0399	c0417	/	2,5-diketo-D-gluconic acid reductase A
i02_0400	c0418	ykgB	hypothetical protein
i02_0401	c0419	ykgI	hypothetical protein
i02_0402	c0420	ykgC	pyridine nucleotide-disulfide oxidoreductase
i02_0403	c0421	ykgD	putative transcriptional regulator YkgD
i02_0404	c0422	ykgE	hypothetical protein
i02_0405	c0423	ykgF	putative electron transport protein ykgF
i02_0407	c0424	ykgG	hypothetical protein
i02_0408	c0425	ykgH	hypothetical protein
i02_0409	c0426	/	hypothetical protein
i02_0410	c0427	/	hypothetical protein
i02_0411		/	hypothetical protein
i02_0412	c0428	/	hypothetical protein
i02_0413	c0429	/	hypothetical protein
i02_0414	c0430	/	Type 1 fimbriae regulatory protein fimB
i02_0415		/	hypothetical protein
i02_0416	c0431	betA	choline dehydrogenase
i02_0417	c0432	betB	betaine aldehyde dehydrogenase
i02_0418	c0433	betI	transcriptional regulator BetI
i02_0419	c0434	betT	choline transport protein BetT
i02_0420	c0435	/	hypothetical protein

Clone D	CFT 073	Gene	Product
i02_0421	c0436	yahB	putative transcriptional regulator YahB
i02_0422	c0437	yahC	hypothetical protein
i02_0423	c0438	/	hypothetical protein
i02_0424	c0439	yahD	ankyrin repeat-containing protein
i02_0425	c0440	yahE	hypothetical protein
i02_0426	c0441	/	hypothetical protein
i02_0427		/	hypothetical protein
i02_0428	c0444	/	putative carbamate kinase
i02_0429	c0445	yahJ	deaminase
i02_0430	c0446	/	hypothetical protein
i02_0431	c0447	yahK	hypothetical protein
i02_0432	c0448	yahN	hypothetical protein
i02_0433	c0449	yahO	hypothetical protein
i02_0434	c0450	prpR	propionate catabolism operon regulatory protein
i02_0435	c0451	prpB	2-methylisocitrate lyase
i02_0436	c0452	prpC	methylcitrate synthase
i02_0437	c0453	prpD	2-methylcitrate dehydratase
i02_0438	c0454	prpE	propionyl-CoA synthetase
i02_0439	c0455	codB	cytosine permease
i02_0440	c0456	codA	cytosine deaminase
i02_0441	c0457	lacA	galactoside O-acetyltransferase
i02_0442	c0458	lacY	galactoside permease
i02_0443	c0459	lacZ	beta-D-galactosidase
i02_0444	c0460	lacI	LacI protein
i02_0445	c0462	yaiL	hypothetical protein
	c0463	/	hypothetical protein
i02_0446	c0464	yaiM	hypothetical protein
i02_0447	c0465	adhC	alcohol dehydrogenase class III
i02_0448	c0466	yaiN	regulator protein FrmR
i02_0449	c0467	/	hypothetical protein
i02_0450	c0468	/	putative transferase
i02_0451	c0469	/	hypothetical protein
i02_0452	c0470	/	hypothetical protein
i02_0454	c0472	tauA	taurine transporter substrate binding subunit
i02_0453	c0471	/	hypothetical protein
i02_0455	c0473	tauB	taurine transporter ATP-binding subunit
	c0474	/	hypothetical protein
i02_0457	c0475	tauC	taurine transporter subunit
i02_0458	c0476	tauD	taurine dioxygenase
i02_0459	c0477	hemB	delta-aminolevulinic acid dehydratase
i02_0460	c0478	/	putative structural protein
i02_0461	c0479	yaiV	putative DNA-binding transcriptional regulator
i02_0462	c0480	yaiH	beta-lactam binding protein AmpH
i02_0463	c0481	/	hypothetical protein
i02_0464	c0482	sbmA	transport protein
i02_0465	c0483	yaiW	hypothetical protein
i02_0467	c0485	/	hypothetical protein
i02_0466	c0484	yaiY	hypothetical protein
i02_0468	c0486	yaiZ	hypothetical protein
i02_0469	c0487	ddl	D-alanyl-alanine synthetase A
i02_0470	c0488	/	hypothetical protein
i02_0471	c0489	yaiB	hypothetical protein

Clone D	CFT 073	Gene	Product
i02_0472	c0490	phoA	alkaline phosphatase
i02_0473	c0491	psiF	hypothetical protein
i02_0474	c0492	adrA	diguanylate cyclase AdrA
i02_0475	c0493	proC	pyrroline-5-carboxylate reductase
i02_0476	c0494	yaiI	hypothetical protein
i02_0477	c0495	aroL	shikimate kinase II
i02_0479	c0496	yaiA	hypothetical protein
	c0497	/	hypothetical protein
i02_0480	c0498	aroM	hypothetical protein
i02_0481	c0499	yaiE	hypothetical protein
i02_0483	c0501	ykiA	hypothetical protein
i02_0482	c0500	/	hypothetical protein
i02_0485	c0503	/	fructokinase
i02_0484	c0502	rdgC	recombination associated protein
i02_0486	c0504	araJ	MFS transport protein AraJ
i02_0487	c0505	sbcC	exonuclease subunit SbcC
i02_0489	c0507	/	hypothetical protein
i02_0488	c0506	sbcD	exonuclease subunit SbcD
i02_0490	c0508	phoB	transcriptional regulator PhoB
i02_0491	c0509	phoR	phosphate regulon sensor protein
i02_0492	c0510	/	hypothetical protein
i02_0493	c0511	brnQ	branched chain amino acid ABC transporter
i02_0494	c0512	proY	putative proline-specific permease
i02_0495	c0513	malZ	maltodextrin glucosidase
i02_0496	c0514	yajB	acyl carrier protein phosphodiesterase
i02_0497	c0515	queA	S-adenosylmethionine:tRNA
i02_0498	c0516	tgt	queuine tRNA-ribosyltransferase
i02_0499	c0517	yajC	preprotein translocase subunit YajC
i02_0500	c0518	secD	preprotein translocase subunit SecD
i02_0501	c0519	secF	preprotein translocase subunit SecF
i02_0502	c0520	yajD	hypothetical protein
i02_0503	c0521	tsx	nucleoside-specific channel-forming protein tsx
i02_0504	c0522	yajI	hypothetical protein
i02_0505	c0523	nrdR	transcriptional regulator NrdR
i02_0506	c0524	ribD	bifunctional
i02_0507	c0525	ribH	6,7-dimethyl-8-ribityllumazine synthase
	c0526	/	hypothetical protein
i02_0508	c0527	nusB	transcription antitermination protein NusB
i02_0509	c0528	thiL	thiamine monophosphate kinase
i02_0510	c0529	pgpA	phosphatidylglycerophosphatase A
i02_0511	c0530	yajO	oxidoreductase yajO
i02_0512	c0531	dxs	1-deoxy-D-xylulose-5-phosphate synthase
i02_0513	c0532	ispA	geranyltranstransferase
i02_0514	c0533	xseB	exodeoxyribonuclease VII small subunit
i02_0515	c0534	yajK	thiamine biosynthesis protein ThiI
i02_0516	c0535	thiJ	hypothetical protein
i02_0517	c0536	apbA	2-dehydropantoate 2-reductase
i02_0518	c0537	yajQ	putative nucleotide-binding protein
i02_0519	c0538	yajR	putative transport protein YajR
i02_0520	c0539	cyoE	protoheme IX farnesyltransferase
i02_0521	c0540	cyoD	cytochrome o ubiquinol oxidase subunit IV
i02_0522	c0541	cyoC	cytochrome o ubiquinol oxidase subunit III

Clone D	CFT 073	Gene	Product
i02_0523	c0542	cyoB	cytochrome o ubiquinol oxidase subunit I
i02_0524	c0543	cyoA	cytochrome o ubiquinol oxidase subunit II
i02_0525	c0544	ampG	muropeptide transporter
	c0545	/	hypothetical protein
i02_0526	c0546	yajG	hypothetical protein
i02_0527	c0547	/	hypothetical protein
i02_0528	c0548	bolA	transcriptional regulator BolA
	c0549	/	hypothetical protein
i02_0529	c0550	/	hypothetical protein
i02_0530	c0551	tig	trigger factor
	c0552	/	hypothetical protein
i02_0531	c0553	clpP	ATP-dependent Clp protease proteolytic subunit
i02_0532	c0554	clpX	ATP-dependent protease ATP-binding subunit ClpX
i02_0533	c0555	lon	DNA-binding ATP-dependent protease La
i02_0534	c0556	hupB	transcriptional regulator HU subunit beta
i02_0535	c0557	ybaU	peptidyl-prolyl cis-trans isomerase (rotamase
i02_0536	c0558	ybaV	hypothetical protein
i02_0537	c0559	ybaW	hypothetical protein
i02_0538	c0560	ybaX	queuosine biosynthesis protein QueC
i02_0539	c0561	ybaE	hypothetical protein
i02_0540	c0562	cof	Cof protein
i02_0541	c0564	ybaO	putative transcriptional regulator YbaO
-	c0563	/	hypothetical protein
i02_0542	c0565	mdlA	putative multidrug transporter
i02_0543	c0566	mdlB	putative multidrug transporter
	c0567	/	hypothetical protein
i02_0544	c0568	glnK	nitrogen regulatory protein P-II 2
	c0569	/	hypothetical protein
i02_0546	c0570	amtB	ammonium transporter
i02_0548		/	hypothetical protein
i02_0547	c0571	tesB	acyl-CoA thioesterase II
i02_0549	c0572	ybaY	hypothetical protein
i02_0550	c0573	ybaZ	hypothetical protein
i02_0551	c0574	ybaA	hypothetical protein
i02_0552	c0575	ylaB	hypothetical protein
i02_0553	c0576	ylaC	hypothetical protein
i02_0554	c0577	ylaD	maltose O-acetyltransferase
i02_0555	c0578	hha	hemolysin expression-modulating protein
i02_0556	c0579	ybaJ	hypothetical protein
i02_0557	c0580	acrB	acriflavin resistance protein B
i02_0558	c0581	acrA	acriflavin resistance protein A
i02_0559	c0582	acrR	DNA-binding transcriptional repressor AcrR
	c0583	/	hypothetical protein
i02_0560	c0584	aefA	potassium efflux protein KefA
i02_0561	c0585	ybaM	hypothetical protein
i02_0562	c0586	priC	primosomal replication protein N''
i02_0563	c0587	ybaN	hypothetical protein
i02_0564	c0588	apt	adenine phosphoribosyltransferase
i02_0565	c0589	dnaX	DNA polymerase III subunits gamma and tau
	c0590	/	hypothetical protein

Clone D	CFT 073	Gene	Product
i02_0566	c0591	ybaB	hypothetical protein
i02_0567	c0592	recR	recombination protein RecR
i02_0568	c0593	htpG	heat shock protein 90
i02_0569	c0594	adk	adenylate kinase
i02_0570	c0595	hemH	ferrochelatase
i02_0571	c0596	ybaC	acetyl esterase
i02_0572	c0597	gsk	inosine-guanosine kinase
i02_0573	c0598	ybaL	putative cation:proton antiport protein
i02_0574	c0599	fsr	fosmidomycin resistance protein
i02_0575	c0600	ushA	bifunctional UDP-sugar hydrolase/5'-nucleotidase
i02_0576	c0601	ybaK	hypothetical protein
i02_0577	c0602	ybaP	hypothetical protein
i02_0578		/	hypothetical protein
i02_0579	c0603	ybaQ	hypothetical protein
i02_0580	c0604	copA	copper exporting ATPase
i02_0581	c0605	ybaS	glutaminase
i02_0582	c0606	ybaT	putative transport protein YbaT
i02_0583	c0607	ybbI	DNA-binding transcriptional regulator CueR
i02_0584	c0608	/	hypothetical protein
i02_0585	c0609	ybbJ	hypothetical protein
i02_0586	c0610	ybbK	hypothetical protein
i02_0587	c0611	ybbL	putative ABC transporter ATP-binding protein
i02_0588		ybbM	putative metal resistance protein
i02_0589	c0613	ybbN	hypothetical protein
i02_0590	c0614	ybbO	short chain dehydrogenase
i02_0592	c0616	ybbA	putative ABC transporter ATP-binding protein
i02_0591	c0615	tesA	multifunctional acyl-CoA thioesterase
i02_0593	c0617	ybbP	hypothetical protein
i02_0594	c0618	ybbB	tRNA 2-selenouridine synthase
i02_0595	c0619	ybbS	DNA-binding transcriptional activator AllS
i02_0596	c0620	ybbT	ureidoglycolate hydrolase
i02_0597	c0621	ybbU	DNA-binding transcriptional repressor AllR
i02_0598	c0622	gcl	glyoxylate carboligase
i02_0599	c0623	gip	hydroxypyruvate isomerase
i02_0600	c0624	ybbQ	2-hydroxy-3-oxopropionate reductase
i02_0601	c0625	ybbW	allantoin permease
i02_0602	c0626	ybbX	allantoinase
i02_0603	c0627	ybbY	putative purine permease YbbY
i02_0604	c0628	ybbZ	glycerate kinase II
i02_0605	c0629	ylbA	hypothetical protein
i02_0606	c0630	ylbB	allantoate amidohydrolase
i02_0607	c0631	/	ureidoglycolate dehydrogenase
i02_0608	c0632	fdrA	membrane protein FdrA
i02_0609	c0633	ylbE	hypothetical protein
i02_0610	c0634	ylbF	hypothetical protein
i02_0611	c0635	arcC	carbamate kinase
i02_0612	c0636	purK	phosphoribosylaminoimidazole carboxylase ATPase
i02_0613	c0637	purE	phosphoribosylaminoimidazole carboxylase
i02_0614	c0638	/	hypothetical protein
	c0640	/	hypothetical protein
i02_0615	c0639	ybbF	UDP-2,3-diacylglucosamine hydrolase

Clone D	CFT 073	Gene	Product
i02_0616	c0641	ppiB	peptidyl-prolyl cis-trans isomerase B
i02_0617	c0642	cysS	cysteinyl-tRNA synthetase
i02_0618	c0643	ybcI	hypothetical protein
i02_0619	c0644	ybcJ	hypothetical protein
i02_0620	c0645	folD	bifunctional 5,10-methylene-tetrahydrofolate
i02_0621	c0646	/	hypothetical protein
i02_0622		intD	prophage DLP12 integrase
i02_0624	c0649	ydfM	tail fiber assembly protein
i02_0623		intD	prophage DLP12 integrase
	c0650	/	hypothetical protein
i02_0625	c0651	/	hypothetical protein
i02_0626	c0652	ompT	outer membrane protease
i02_0627	c0653	ybcH	hypothetical protein
i02_0628	c0654	nfrA	bacteriophage N4 receptor, outer membrane
i02_0629	c0655	nfrB	bacteriophage N4 adsorption protein B
i02_0630	c0656	cusS	sensor kinase CusS
i02_0631	c0657	cusR	DNA-binding transcriptional activator CusR
i02_0632	c0658	cusC	copper/silver efflux system outer membrane
i02_0633	c0659	cusX	periplasmic copper-binding protein
i02_0634	c0660	cusB	copper/silver efflux system membrane fusion
i02_0635	c0661	cusA	putative cation efflux system protein cusA
i02_0636	c0662	pheP	phenylalanine transporter
i02_0637	c0663	ybdG	hypothetical protein
i02_0638	c0664	nfnB	dihydropteridine reductase
i02_0639	c0665	ybdF	hypothetical protein
i02_0640	c0666	ybdJ	hypothetical protein
i02_0641	c0667	ybdK	carboxylate-amine ligase
i02_0642		/	hypothetical protein
i02_0643	c0668	entD	phosphopantetheinyltransferase
i02_0645	c0670	/	hypothetical protein
i02_0644	c0669	fepA	outer membrane receptor FepA
i02_0646	c0671	fes	enterobactin/ferric enterobactin esterase
	c0672	/	hypothetical protein
i02_0647	c0673	entF	enterobactin synthase subunit F
i02_0648	c0674	fepE	ferric enterobactin transport protein FepE
i02_0649	c0675	fepC	iron-enterobactin transporter ATP-binding component
i02_0650	c0676	fepG	iron-enterobactin transporter permease
i02_0651	c0677	fepD	iron-enterobactin transporter membrane protein
i02_0652	c0678	ybdA	enterobactin exporter EntS
i02_0653	c0679	fepB	iron-enterobactin transporter periplasmic
i02_0654	c0680	entC	isochorismate synthase
i02_0655	c0681	entE	enterobactin synthase subunit E
i02_0656	c0682	entB	isochorismatase
i02_0657	c0683	entA	2,3-dihydroxybenzoate-2,3-dehydrogenase
i02_0658	c0684	ybdB	hypothetical protein
i02_0659	c0685	cstA	carbon starvation protein A
i02_0660	c0686	/	hypothetical protein
i02_0661	c0687	ybdH	hypothetical protein
i02_0662	c0688	ybdL	putative aminotransferase
i02_0663	c0689	/	hypothetical protein
i02_0664	c0690	/	hypothetical protein

Clone D	CFT 073	Gene	Product
i02_0665	c0691	ybdO	putative transcriptional regulator YbdO
i02_0666	c0692	dsbG	disulfide isomerase/thiol-disulfide oxidase
	c0693	/	hypothetical protein
i02_0667	c0694	ahpC	alkyl hydroperoxide reductase subunit C
i02_0668	c0695	ahpF	Alkyl hydroperoxide reductase subunit F
i02_0669	c0696	ybdQ	hypothetical protein
	c0697	/	hypothetical protein
i02_0670	c0698	rnk	nucleoside diphosphate kinase regulator
i02_0671	c0699	rna	ribonuclease I
i02_0672	c0700	ybdS	citrate transporter
i02_0673	c0701	citG	triphosphoribosyl-dephospho-CoA synthase
	c0703	/	hypothetical protein
i02_0674	c0702	citX	apo-citrate lyase phosphoribosyl-dephospho-CoA transferase
	c0705	/	hypothetical protein
i02_0675	c0704	citF	citrate lyase alpha chain
i02_0676	c0706	citE	citrate lyase beta chain
i02_0677	c0709	citC	[citrate [pro-3S]-lyase] ligase
i02_0678	c0710	citA	sensor kinase dpiB
i02_0679	c0711	dpiA	two-component response regulator DpiA
i02_0680	c0712	dcuC	C4-dicarboxylate transporter DcuC
i02_0681	c0713	pagP	palmitoyl transferase
i02_0682	c0714	cspE	cold shock protein CspE
i02_0683	c0715	ccrB	camphor resistance protein CrcB
i02_0684	c0716	ybeM	putative amidase
i02_0685	c0717	tatE	twin arginine translocase protein E
i02_0686	c0718	lipA	lipoyl synthase
i02_0687	c0719	/	hypothetical protein
i02_0688	c0720	lipB	lipoyltransferase
i02_0689	c0721	ybeD	hypothetical protein
i02_0690	c0722	dacA	D-alanyl-D-alanine carboxypeptidase fraction A
i02_0691	c0723	/	hypothetical protein
i02_0692	c0724	rlpA	rare lipoprotein A
i02_0693	c0725	mrdB	cell wall shape-determining protein
i02_0694	c0726	mrdA	penicillin-binding protein 2
i02_0695	c0727	ybeA	rRNA large subunit methyltransferase
i02_0696	c0728	ybeB	hypothetical protein
i02_0697		/	hypothetical protein
i02_0698	c0729	phpB	Alpha-ribazole-5'-phosphate phosphatase
i02_0699	c0730	nadD	nicotinic acid mononucleotide
i02_0700	c0731	holA	DNA polymerase III subunit delta
i02_0701	c0732	rlpB	LPS-assembly lipoprotein RlpB
i02_0702	c0733	leuS	leucyl-tRNA synthetase
i02_0703	c0734	ybeL	hypothetical protein
i02_0704	c0735	rihA	ribonucleoside hydrolase 1
i02_0705	c0736	gltL	glutamate/aspartate transport ATP-binding component
i02_0706	c0737	gltK	glutamate/aspartate transport system permease component
i02_0707	c0738	gltJ	glutamate/aspartate transport system permease component
i02_0708	c0739	ybeJ	glutamate and aspartate transporter subunit

Clone D	CFT 073	Gene	Product
i02_0709	c0740	/	hypothetical protein
i02_0710	c0741	/	hypothetical protein
i02_0711	c0742	lnt	apolipoprotein N-acyltransferase
i02_0712	c0743	ybeX	magnesium and cobalt efflux protein corC
i02_0713	c0744	ybeY	putative metalloprotease
i02_0715	c0746	/	hypothetical protein
i02_0714	c0745	ybeZ	PhoH-like protein
i02_0716	c0747	yleA	hypothetical protein
i02_0717	c0748	ubiF	2-octaprenyl-3-methyl-6-methoxy-1,4-benzoquinone hydroxylase
i02_0718	c0749	asnB	asparagine synthetase B
i02_0719	c0750	nagD	UMP phosphatase
i02_0720	c0751	nagC	N-acetylglucosamine repressor
i02_0721	c0752	nagA	N-acetylglucosamine-6-phosphate deacetylase
i02_0722	c0753	nagB	glucosamine-6-phosphate deaminase
i02_0723	c0754	/	hypothetical protein
i02_0724	c0755	nagE	N-acetyl glucosamine specific PTS system
i02_0725	c0756	/	hypothetical protein
i02_0726	c0757	/	hypothetical protein
i02_0727	c0758	/	hypthetical protein
i02_0728	c0759	/	hypthetical protein
i02_0729	c0760	/	hypothetical protein
i02_0731	c0761	/	putative dihydrodipicolinate synthase
i02_0732	c0762	/	putative alcohol dehydrogenase
i02_0733	c0763	/	putative inner membrane protein
i02_0734	c0764	pdxA	4-hydroxythreonine-4-phosphate dehydrogenase 2
i02_0735	c0765	/	putative transcriptional regulator
i02_0736	c0766	glnS	glutaminyl-tRNA synthetase
-	c0767	/	hypothetical protein
i02_0737	c0768	ybfM	hypothetical protein
i02_0738	c0769	ybfN	lipoprotein ybfN precursor
i02_0739	c0770	fur	ferric uptake regulator
i02_0740	c0771	fldA	flavodoxin FldA
i02_0741	c0772	ybfE	LexA regulated protein
i02_0742	c0773	ybfF	hypothetical protein
i02_0743	c0774	seqA	replication initiation regulator SeqA
i02_0744	c0775	pgm	phosphoglucomutase
i02_0745	c0776	potE	putrescine transporter
i02_0746	c0777	speF	ornithine decarboxylase
i02_0747	c0778	/	hypothetical protein
i02_0748		/	hypothetical protein
i02_0749		/	conserved hypothetical protein
i02_0750	c0779	kdpE	DNA-binding transcriptional activator KdpE
i02_0751	c0780	kdpD	sensor protein KdpD
i02_0752	c0781	kdpC	potassium-transporting ATPase subunit C
i02_0753	c0782	kdpB	potassium-transporting ATPase subunit B
i02_0754	c0783	kdpA	potassium-transporting ATPase subunit A
i02_0755	c0784	/	hypothetical protein
i02_0756	c0785	ybfA	hypothetical protein
i02_0757	c0786	ybgA	hypothetical protein
i02_0758	c0787	phrB	deoxyribodipyrimidine photolyase

Clone D	CFT 073	Gene	Product
i02_0759	c0788	ybgH	putative transporter YbgH
i02_0760	c0789	ybgI	putative hydrolase-oxidase
i02_0761	c0790	ybgJ	hypothetical protein
i02_0762	c0791	ybgK	hypothetical protein
i02_0763	c0792	ybgL	LamB/YcsF family protein
i02_0764	c0793	nei	endonuclease VIII
i02_0765	c0794	abrB	AbrB protein
i02_0766	c0795	/	hypothetical protein
i02_0768	c0797	/	hypothetical protein
i02_0767	c0796	gltA	type II citrate synthase
i02_0769	c0798	sdhC	succinate dehydrogenase cytochrome b556 large subunit
	c0799	/	hypothetical protein
i02_0770	c0800	sdhD	succinate dehydrogenase cytochrome b556 small subunit
i02_0771	c0801	sdhA	succinate dehydrogenase flavoprotein subunit
i02_0772	c0802	sdhB	succinate dehydrogenase iron-sulfur subunit
i02_0773	c0803	sucA	2-oxoglutarate dehydrogenase E1 component
i02_0774	c0804	sucB	dihydrolipoamide succinyltransferase
i02_0775	c0805	sucC	succinyl-CoA synthetase subunit beta
i02_0776	c0806	sucD	succinyl-CoA synthetase subunit alpha
i02_0777	c0807	/	hypothetical protein
i02_0778	c0808	/	hypothetical protein
i02_0779	c0809	/	hypothetical protein
i02_0780	c0810	/	hypothetical protein
i02_0781	c0811	cydA	cytochrome D ubiquinol oxidase subunit I
i02_0782	c0812	cydB	cytochrome D ubiquinol oxidase subunit II
	c0813	/	hypothetical protein
i02_0783		/	hypothetical protein
i02_0784	c0814	ybgE	hypothetical protein
i02_0785	c0815	ybgC	acyl-CoA thioester hydrolase YbgC
i02_0786	c0816	tolQ	colicin uptake protein TolQ
i02_0787	c0817	tolR	colicin uptake protein TolR
i02_0788	c0818	tolA	cell envelope integrity inner membrane protein
i02_0789	c0819	tolB	translocation protein TolB
	c0820	/	hypothetical protein
i02_0790	c0821	pal	peptidoglycan-associated outer membrane
i02_0791	c0822	ybgF	hypothetical protein
i02_0791	c0823	/	hypothetical protein
i02_0792	c0824	/	hypothetical protein
i02_0793	c0825	nadA	quinolinate synthetase
i02_0794	c0826	pnuC	protein pnuC
i02_0795	c0827	/	hypothetical protein
	c0828	/	hypothetical protein
i02_0796	c0829	ybgS	hypothetical protein
i02_0797	c0830	aroG	phospho-2-dehydro-3-deoxyheptonate aldolase
i02_0798	c0831	gpmA	phosphoglyceromutase
i02_0799	c0832	galM	aldose 1-epimerase
i02_0800	c0833	galK	galactokinase
i02_0801	c0834	galT	galactose-1-phosphate uridylyltransferase
i02_0802	c0835	galE	UDP-galactose-4-epimerase

Clone D	CFT 073	Gene	Product
i02_0803	c0836	modF	putative molybdenum transport ATP-binding protein
i02_0804	c0837	modE	DNA-binding transcriptional regulator ModE
i02_0805	c0838	ybhT	hypothetical protein
i02_0806	c0839	/	hypothetical protein
i02_0807	c0840	modA	molybdate transporter periplasmic protein
i02_0808	c0841	modB	molybdate ABC transporter permease protein
i02_0809	c0842	modC	molybdate transporter ATP-binding protein
i02_0810	c0843	ybhA	phosphotransferase
i02_0811	c0844	ybhE	6-phosphogluconolactonase
i02_0812	c0845	ybhD	putative transcriptional regulator YbhD
i02_0813	c0846	ybhH	hypothetical protein
i02_0814	c0847	ybhI	hypothetical protein
i02_0815	c0848	ybhJ	hypothetical protein
i02_0816	c0849	ybhC	putative pectinesterase
i02_0817	c0850	ybhB	putative kinase inhibitor protein
i02_0818	c0851	gipA	Peyer's patch-specific virulence factor GipA
	c0852	/	hypothetical protein
	c0854	/	hypothetical protein
i02_0820	c0853	bioA	adenosylmethionine-8-amino-7-oxononanoate aminotransferase monomer
i02_0821	c0855	bioB	biotin synthase
i02_0822	c0856	bioF	8-amino-7-oxononanoate synthase
i02_0823	c0857	bioC	putative methyltransferase
i02_0824	c0858	bioD	dithiobiotin synthetase
i02_0825	c0859	/	hypothetical protein
i02_0826	c0860	uvrB	excinuclease ABC subunit B
i02_0827	c0861	ybhK	hypothetical protein
i02_0828	c0862	moaA	molybdenum cofactor biosynthesis protein A
i02_0829	c0863	moaB	molybdenum cofactor biosynthesis protein B
i02_0831	c0865	moaC	molybdenum cofactor biosynthesis protein C
i02_0830	c0864	/	hypothetical protein
i02_0832	c0866	moaD	molybdopterin synthase small subunit
i02_0833	c0867	moaE	molybdopterin synthase large subunit
i02_0834	c0868	ybhL	hypothetical protein
i02_0835	c0869	/	hypothetical protein
i02_0836	c0870	ybhM	hypothetical protein
i02_0837	c0871	ybhN	hypothetical protein
i02_0838	c0872	ybhO	cardiolipin synthase 2
i02_0839	c0873	ybhP	hypothetical protein
i02_0840	c0874	ybhQ	hypothetical protein
i02_0841	c0875	ybhR	hypothetical protein
i02_0842	c0876	ybhS	hypothetical protein
i02_0843	c0877	ybhF	ABC transporter ATP-binding protein
i02_0844	c0878	ybhG	hypothetical protein
i02_0845	c0879	ybiH	putative DNA-binding transcriptional regulator
i02_0846	c0880	rhlE	ATP-dependent RNA helicase RhlE
i02_0848	c0883	dinG	ATP-dependent DNA helicase DinG
i02_0847	c0882	/	hypothetical protein
i02_0849	c0884	ybiB	glycosyl transferase family protein
i02_0850	c0885	ybiC	hypothetical protein
i02_0851	c0886	ybiJ	hypothetical protein

Clone D	CFT 073	Gene	Product
i02_0852	c0888	/	hypothetical protein
i02_0853	c0889	ybiX	putative hydroxylase
i02_0854	c0890	ybiL	catecholate siderophore receptor Fiu
i02_0855	c0891	ybiM	hypothetical protein
i02_0856	c0892	ybiN	putative SAM-dependent methyltransferase
i02_0857	c0893	ybiO	hypothetical protein
i02_0858	c0894	glnQ	glutamine ABC transporter ATP-binding protein
i02_0859	c0895	glnP	glutamine ABC transporter permease protein
i02_0860	c0896	glnH	glutamine ABC transporter periplasmic protein
i02_0861	c0897	/	hypothetical protein
i02_0862	c0898	dps	DNA starvation/stationary phase protection
i02_0863	c0899	ybiF	threonine and homoserine efflux system
i02_0864	c0900	ompX	outer membrane protein X
i02_0865	c0901	ybiP	hypothetical protein
i02_0866	c0902	/	hypothetical protein
i02_0867	c0903	/	manganese transport regulator MntR
i02_0868	c0904	ybiR	hypothetical protein
i02_0869	c0905	ybiS	hypothetical protein
i02_0870	c0906	ybiT	ABC transporter ATP-binding protein
i02_0871	c0907	ybiV	hypothetical protein
i02_0872	c0908	ybiW	putative formate acetyltransferase 3
i02_0873	c0909	/	putative pyruvate formate-lyase 3 activating protein
i02_0874	c0910	mipB	fructose-6-phosphate aldolase
i02_0875	c0911	moeB	molybdopterin biosynthesis protein MoeB
i02_0876	c0912	moeA	molybdopterin biosynthesis protein MoeA
i02_0877	c0913	ybiK	L-asparaginase
i02_0878	c0914	yliA	glutathione transporter ATP-binding protein
i02_0879	c0915	/	putative binding protein yliB precursor
i02_0880	c0916	yliC	ABC transporter permease
i02_0881	c0917	yliD	ABC transporter permease
i02_0882	c0918	yliE	hypothetical protein
i02_0883	c0919	yliF	membrane protein yliF
i02_0884	c0920	yliG	hypothetical protein
i02_0885	c0921	bssR	biofilm formation regulatory protein BssR
i02_0886	c0922	yliI	hypothetical protein
i02_0887	c0923	yliJ	GST-like protein yliJ
i02_0888	c0924	dacC	D-alanyl-D-alanine carboxypeptidase fraction C
i02_0889	c0925	deoR	DNA-binding transcriptional repressor DeoR
i02_0890	c0926	ybjG	undecaprenyl pyrophosphate phosphatase
i02_0891	c0927	cmr	multidrug translocase mdfA
i02_0892	c0928	ybjH	hypothetical protein
i02_0893	c0929	/	hypothetical protein
i02_0895	c0931	ybjK	hypothetical protein
i02_0894	c0930	ybjJ	hypothetical protein
-	c0932	intT	integrase for prophage
-	c0933	/	hypothetical protein
-	c0934	/	hypothetical protein
-	c0935	coxT	putative regulator for prophage
-	c0936	/	hypothetical protein
-	c0937	/	hypothetical protein
-	c0938	/	hypothetical protein

Clone D	CFT 073	Gene	Product
-	c0939	/	hypothetical protein
-	c0940	/	hypothetical protein
-	c0941	/	DNA adenine methylase
-	c0942	/	putative replication protein for prophage
-	c0943	/	hypothetical protein
-	c0944	/	hypothetical protein
-	c0945	/	hypothetical protein
-	c0946	/	hypothetical protein
-	c0947	/	capsid portal protein
-	c0948	/	terminase, ATPase subunit
-	c0949	/	hypothetical protein
-	c0950	/	putative capsid scaffolding protein
-	c0951	/	hypothetical protein
-	c0952	/	major capsid protein
-	c0953	/	terminase, endonuclease subunit
-	c0954	/	putative capsid completion protein
-	c0955	/	phage tail protein
-	c0956	/	secretory protein
-	c0957	/	Fels-2 prophage: prophage lysozyme
-	c0958	/	hypothetical protein
-	c0959	/	putative regulatory protein
-	c0960	/	putative phage tail protein
-	c0961	/	hypothetical protein
-	c0962	/	putative phage tail protein
-	c0963	/	putative Phage baseplate assembly protein
-	c0964	/	Phage baseplate assembly protein
-	c0965	/	Phage baseplate assembly protein
-	c0966	/	putative phage tail protein
-	c0967	/	hypothetical protein
-	c0968	/	variable tail fibre protein
-	c0969	yfdK	hypothetical protein
-	c0970	/	hypothetical protein
-	c0971	/	major tail sheath protein
-	c0972	/	putative tail fiber component of prophage
-	c0973	/	hypothetical protein
-	c0974	/	putative phage tail protein
-	c0975	/	hypothetical protein
-	c0976	/	hypothetical protein
-	c0977	/	putative tail fiber protein of prophage
-	c0978	/	putative regulator of late gene expression
-	c0979	ogrK	prophage P2 Ogr protein
i02_0896	c0980	ybjL	hypothetical protein
i02_0897	c0981	ybjM	hypothetical protein
i02_0898	c0982	grxA	glutaredoxin 1
i02_0899	c0983	ybjC	hypothetical protein
i02_0900	c0984	mdaA	nitroreductase A
i02_0901	c0985	rimK	ribosomal protein S6 modification protein
i02_0902	c0986	ybjN	hypothetical protein
i02_0903	c0987	potF	putrescine transporter subunit
i02_0904	c0988	potG	putrescine transporter ATP-binding subunit
i02_0905	c0989	potH	putrescine transporter subunit
i02_0906	c0990	potI	putrescine transporter subunit

Clone D	CFT 073	Gene	Product
i02_0907	c0991	ybjO	hypothetical protein
i02_0908	c0992	rumB	23S rRNA methyluridine methyltransferase
i02_0909	c0993	artJ	arginine-binding periplasmic protein 2
i02_0910	c0994	artM	arginine transporter permease subunit ArtM
i02_0911	c0995	artQ	arginine transporter permease subunit ArtQ
i02_0912	c0996	artI	arginine-binding periplasmic protein 1
i02_0913	c0997	artP	arginine transporter ATP-binding subunit
i02_0914	c0998	ybjP	putative lipoprotein
i02_0915	c0999	ybjQ	hypothetical protein
i02_0916	c1000	ybjR	N-acetylmuramoyl-L-alanine amidase ybjR
i02_0917	c1001	/	hypothetical protein
i02_0918	c1002	ybjT	hypothetical protein
i02_0919	c1003	ybjU	L-threonine aldolase
i02_0920	c1004	poxB	pyruvate dehydrogenase
i02_0921	c1005	/	HCP oxidoreductase, NADH-dependent
i02_0922	c1006	ybjW	hydroxylamine reductase
i02_0924	c1008	/	hypothetical protein
i02_0923	c1007	ybjE	hypothetical protein
i02_0925	c1009	aqpZ	aquaporin Z
i02_0926	c1010	/	hypothetical protein
i02_0927	c1011	ybjD	hypothetical protein
i02_0928	c1012	ybjX	hypothetical protein
i02_0929	c1013	/	hypothetical protein
i02_0930	c1014	macA	macrolide transporter subunit MacA
i02_0931	c1016	/	hypothetical protein
i02_0932	c1017	cspD	stationary phase/starvation inducible regulatory
i02_0933	c1018	clpS	ATP-dependent Clp protease adaptor protein ClpS
i02_0934	c1019	clpA	ATP-dependent Clp protease ATP-binding subunit
i02_0935	c1021	infA	translation initiation factor IF-1
i02_0936	c1022	aat	leucyl/phenylalanyl-tRNA--protein transferase
i02_0937	c1023	cydC	cysteine/glutathione ABC transporter
i02_0938	c1024	cydD	cysteine/glutathione ABC transporter
i02_0939	c1025	trxB	thioredoxin reductase
i02_0940	c1026	lrp	leucine-responsive transcriptional regulator
i02_0941	c1027	ftsK	DNA translocase FtsK
i02_0942	c1028	lolA	outer-membrane lipoprotein carrier protein
i02_0943	c1029	ycaJ	recombination factor protein RarA
i02_0944	c1030	serS	seryl-tRNA synthetase
i02_0945	c1031	dmsA	anaerobic dimethyl sulfoxide reductase chain A
i02_0946	c1032	dmsB	anaerobic dimethyl sulfoxide reductase chain B
i02_0947	c1033	dmsC	anaerobic dimethyl sulfoxide reductase chain C
i02_0948	c1034	ycaC	hypothetical protein
	c1036	/	hypothetical protein
i02_0949	c1035	/	hypothetical protein
i02_0950	c1037	ycaD	putative MFS family transporter protein
i02_0951	c1038	pflA	pyruvate formate lyase-activating enzyme 1
	c1040	/	hypothetical protein
i02_0952		pflB	PflB
i02_0954	c1042	focA_2	formate transporter
i02_0955	c1043	ycaO	hypothetical protein
i02_0956	c1044	ycaP	hypothetical protein

Clone D	CFT 073	Gene	Product
i02_0957	c1045	serC	phosphoserine aminotransferase
i02_0958	c1046	aroA	3-phosphoshikimate 1-carboxyvinyltransferase
i02_0959	c1047	ycaL	putative metalloprotease ycaL
i02_0960	c1048	cmk	cytidylate kinase
i02_0961	c1049	rpsA	30S ribosomal protein S1
i02_0962	c1050	ihfB	integration host factor subunit beta
	c1051	/	hypothetical protein
i02_0963	c1052	ycaI	hypothetical protein
i02_0964	c1054	msbA	lipid transporter ATP-binding/permease protein
i02_0965		lpxK	tetraacyldisaccharide 4'-kinase
i02_0966	c1057	ycaQ	hypothetical protein
i02_0967	c1058	ycaR	hypothetical protein
i02_0968	c1059	kdsB	3-deoxy-manno-octulosonate cytidylyltransferase
i02_0969	c1060	ycbJ	hypothetical protein
i02_0970	c1061	ycbC	hypothetical protein
i02_0971	c1062	/	hypothetical protein
i02_0972	c1063	smtA	putative metallothionein SmtA
i02_0973	c1064	mukF	condesin subunit F
i02_0974	c1065	mukE	condesin subunit E
i02_0975	c1066	mukB	cell division protein MukB
i02_0976	c1067	ycbB	hypothetical protein
i02_0977	c1068	ycbK	hypothetical protein
i02_0978	c1069	ycbL	hypothetical protein
i02_0979	c1070	aspC	aromatic amino acid aminotransferase
i02_0980	c1071	ompF	outer membrane protein F
i02_0981	c1072	asnC	asparaginyl-tRNA synthetase
	c1074	/	hypothetical protein
i02_0982	c1073	pncB	nicotinate phosphoribosyltransferase
i02_0983	c1075	pepN	aminopeptidase N
i02_0984	c1076	ssuB	aliphatic sulfonates transport ATP-binding component
i02_0985	c1077	ssuC	alkanesulfonate transporter permease subunit
i02_0986	c1078	ycbN	alkanesulfonate monooxygenase
i02_0987	c1079	ycbO	alkanesulfonate transporter substrate-binding component
i02_0988	c1080	ycbP	NAD(P)H-dependent FMN reductase
i02_0989	c1081	pyrD	dihydroorotate dehydrogenase 2
i02_0990	c1082	ycbW	hypothetical protein
i02_0991	c1083	ycbX	hypothetical protein
i02_0992	c1084	rlmL	23S rRNA m(2)G2445 methyltransferase
i02_0993	c1085	uup	ABC transporter ATPase component
i02_0994	c1086	pqiA	paraquat-inducible protein A
i02_0995	c1087	pqiB	paraquat-inducible protein B
i02_0996	c1088	ymbA	hypothetical protein
i02_0997	c1089	/	hypothetical protein
i02_0998	c1090	fabA	3-hydroxydecanoyl-(acyl carrier protein)
i02_0999	c1091	/	putative protease La-like protein
i02_1000	c1092	ycbG	hypothetical protein
i02_1001	c1093	ompA	outer membrane protein A
i02_1002	c1094	/	hypothetical protein
i02_1003	c1095	sulA	SOS cell division inhibitor
i02_1004	c1096	yccR	hypothetical protein

Clone D	CFT 073	Gene	Product
i02_1005	c1097	yccS	hypothetical protein
i02_1006	c1098	yccF	hypothetical protein
i02_1007	c1099	helD	DNA helicase IV
i02_1008	c1100	mgsA	methylglyoxal synthase
i02_1009	c1101	yccT	hypothetical protein
i02_1010	c1102	/	hypothetical protein
	c1103	/	hypothetical protein
i02_1011	c1104	yccV	hypothetical protein
i02_1013	c1106	/	putative acylphosphatase
i02_1012	c1105	yccW	hypothetical protein
i02_1014	c1107	yccK	sulfur transfer protein TusE
i02_1015	c1110	yccA	hypothetical protein
i02_1017	c1113	hyaA	hydrogenase-1 small chain precursor
i02_1016	c1112	/	hypothetical protein
i02_1018	c1114	hyaB	hydrogenase 1 large subunit
i02_1019	c1115	hyaC	hydrogenase 1 b-type cytochrome subunit
i02_1020	c1116	hyaD	hydrogenase 1 maturation protease
i02_1021	c1117	hyaE	hydrogenase-1 operon protein HyaE
i02_1022	c1118	hyaF	hydrogenase-1 operon protein HyaF
i02_1023	c1119	appC	cytochrome bd-II oxidase subunit I
i02_1024	c1120	appB	cytochrome bd-II oxidase subunit II
i02_1025	c1121	appA	phosphoanhydride phosphorylase
i02_1026	c1122	cspH	cold shock-like protein cspH
i02_1027	c1123	cspG	cold shock protein CspG
i02_1028	c1124	sfa	cold shock gene
i02_1029	c1125	/	GnsB protein
	c1126	/	hypothetical protein
i02_1030	c1127	yccM	putative electron transport protein yccM
i02_1031	c1128	torS	hybrid sensory histidine kinase TorS
i02_1032	c1130	torT	TMAO reductase system periplasmic protein TorT
i02_1033	c1131	torR	DNA-binding transcriptional regulator TorR
i02_1034	c1132	torC	cytochrome c-type protein torC
i02_1035	c1133	torA	trimethylamine-N-oxide reductase 1 precursor
i02_1036	c1134	torD	chaperone protein TorD
i02_1037	c1135	yccD	chaperone-modulator protein CbpM
i02_1038	c1136	cbpA	curved DNA-binding protein CbpA
i02_1039	c1137	agp	glucose-1-phosphatase/inositol phosphatase
i02_1039	c1138	/	hypothetical protein
i02_1040	c1139	yccJ	hypothetical protein
i02_1041	c1140	wrbA	TrpR binding protein WrbA
i02_1042	c1141	/	hypothetical protein
	c1142	/	hypothetical protein
i02_1043	c1143	ycdG	putative purine permease ycdG
i02_1044	c1144	/	putative flavin:NADH reductase ycdH
i02_1045	c1145	/	hypothetical protein
i02_1046	c1146	ycdJ	hypothetical protein
i02_1047	c1147	ycdK	hypothetical protein
i02_1048	c1148	ycdL	isochorismatase family protein ycdL
i02_1049	c1149	/	putative monooxygenase ycdM
i02_1050	c1150	ycdC	putative transcriptional regulator YcdC
i02_1051	c1151	putA	trifunctional transcriptional regulator/proline
i02_1052	c1152	/	hypothetical protein

Clone D	CFT 073	Gene	Product
i02_1053	c1153	/	hypothetical protein
i02_1054	c1154	putP	Sodium/proline symporter
i02_1055	c1155	/	putative cytochrome
i02_1056	c1156	ycdO	hypothetical protein
i02_1057	c1157	ycdB	hypothetical protein
i02_1058	c1158	/	hypothetical protein
i02_1059	c1159	phoH	hypothetical protein
i02_1060	c1160	ycdP	hypothetical protein
i02_1061	c1161	ycdQ	N-glycosyltransferase PgaC
i02_1062	c1162	ycdR	lipoprotein ycdR precursor
i02_1063	c1163	pgaA	outer membrane protein PgaA
i02_1064	c1164	ycdT	hypothetical protein
i02_1065	c1165	/	P4 family integrase
i02_1066	c1166	/	hypothetical protein
i02_1067	c1167	/	hypothetical protein
i02_1068	c1168	/	hypothetical protein
i02_1069	c1169	alpA	prophage CP4-57 regulatory protein alpA
i02_1070	c1170	/	hypothetical protein
i02_1071	c1171	/	hypothetical protein
	c1172	/	hypothetical protein
i02_1072	c1173	/	hypothetical protein
i02_1073	c1174	/	hypothetical protein
i02_1074	c1175	/	putative aminotransferase
i02_1075	c1176	/	hypothetical protein
i02_1076	c1183	/	hypothetical protein
i02_1077	c1184	/	hypothetical protein
	c1185	/	hypothetical protein
i02_1079	c1186	/	3-oxoacyl-(acyl carrier protein) synthase II
i02_1080	c1187	fabG	3-ketoacyl-(acyl-carrier-protein) reductase
i02_1081	c1188	/	hypothetical protein
i02_1082	c1189	/	3-oxoacyl-(acyl carrier protein) synthase I
i02_1083	c1190	/	hypothetical protein
i02_1084	c1191	/	hypothetical protein
i02_1085	c1192	/	hypothetical protein
i02_1086	c1193	/	hypothetical protein
i02_1087	c1194	/	hypothetical protein
i02_1088	c1195	/	hypothetical protein
i02_1089	c1196	/	hypothetical protein
i02_1090	c1197	/	hypothetical protein
i02_1091	c1198	/	hypothetical protein
i02_1092	c1199	/	acyl carrier protein
i02_1093	c1200	/	putative acyl carrier protein
i02_1094	c1201	/	putative phospholipid biosynthesis
i02_1095	c1202	/	hypothetical protein
i02_1096	c1203	/	putative O-methyltransferase
i02_1097	c1204	/	hypothetical protein
i02_1098	c1205	/	hypothetical protein
	c1206	/	hypothetical protein
i02_1099	c1207	/	hypothetical protein
i02_1100	c1208	/	hypothetical protein
i02_1101	c1209	/	hypothetical protein
	c1210	/	hypothetical protein

Clone D	CFT 073	Gene	Product
i02_1103		/	hypothetical protein
i02_1104		/	conserved hypothetical protein
i02_1106	c1213	/	hypothetical protein
i02_1105		/	hypothetical protein
i02_1107	c1214	/	Cea protein
i02_1108	c1215	/	entry exclusion protein 2
i02_1109	c1216	/	hypothetical protein
i02_1110	c1217	/	hypothetical protein
i02_1111	c1218	/	hypothetical protein
i02_1112	c1219	/	putative transposase
i02_1113		/	conserved hypothetical protein
i02_1114	c1220	/	phospho-2-dehydro-3-deoxyheptonate aldolase
i02_1115	c1221	/	hypothetical protein
i02_1116	c1222	/	hypothetical protein
i02_1117	c1224	/	transposase insF
i02_1118	c1225	/	transposase insE
i02_1119	c1227	mchB	MchB protein
i02_1120	c1229	mchC	MchC protein
i02_1121	c1230	mchD	MchD protein
i02_1122	c1231	mchE	microcin H47 secretion protein
i02_1123	c1232	mchF	microcin H47 secretion ATP-binding protein
i02_1124		/	conserved domain protein
i02_1125	c1233	/	hypothetical protein
i02_1126	c1234	/	hypothetical protein
i02_1127	c1235	/	hypothetical protein
i02_1128	c1236	/	hypothetical protein
i02_1129		/	hypothetical protein
i02_1130	c1237	sfaC	putative F1C and S fimbrial switch regulatory protein
i02_1131	c1238	sfaB	putative F1C and S fimbrial switch regulatory protein
i02_1132	c1239	focA	F1C major fimbrial subunit precursor
i02_1133	c1240	sfaD	putative minor F1C fimbrial subunit precursor
i02_1134	c1241	focC	F1C periplasmic chaperone
i02_1135	c1242	focD	F1C fimbrial usher
i02_1136	c1243	focF	F1C minor fimbrial subunit F precursor
i02_1137	c1244	focG	F1C minor fimbrial subunit protein G presursor
i02_1138	c1245	focH	F1C putative fimbrial adhesin precursor
i02_1139		/	conserved hypothetical protein
i02_1140	c1246	/	hypothetical protein
i02_1141	c1247	focX	putative regulatory protein
i02_1142	c1248	/	hypothetical protein
i02_1143	c1249	/	hypothetical protein
i02_1144	c1250	iroN	outer membrane receptor FepA
i02_1145	c1251	iroE	IroE protein
i02_1146	c1252	iroD	ferric enterochelin esterase
i02_1147	c1253	iroC	ABC transporter ATP-binding protein
i02_1148	c1254	iroB	putative glucosyltransferase
i02_1149	c1255	/	hypothetical protein
i02_1150	c1256	/	hypothetical protein
i02_1151	c1257	/	hypothetical protein
i02_1152		/	conserved hypothetical protein

Clone D	CFT 073	Gene	Product
i02_1153	c1258	/	hypothetical protein
i02_1154	c1259	/	hypothetical protein
i02_1155	c1260	/	putative transposase
-	c1262	/	transposase IS629
-	c1263	/	hypothetical protein
i02_1157	c1261	/	hypothetical protein
i02_1157	c1264	/	hypothetical protein
i02_1158	c1265	/	Outer membrane heme/hemoglobin receptor
i02_1159	c1266	/	hypothetical protein
i02_1160	c1267	/	hypothetical protein
i02_1161	c1268	/	hypothetical protein
i02_1162	c1269	/	hypothetical protein
i02_1163	c1270	/	hypothetical protein
i02_1164	c1271	/	hypothetical protein
i02_1165	c1272	/	hypothetical protein
i02_1166	c1273	/	antigen 43 precursor
i02_1167	c1274	/	hypothetical protein
i02_1168	c1275	/	hypothetical protein
i02_1169	c1276	/	hypothetical protein
i02_1170	c1277	/	hypothetical protein
i02_1171	c1278	/	hypothetical protein
i02_1172	c1279	/	hypothetical protein
i02_1173	c1280	/	hypothetical protein
	c1281	/	hypothetical protein
i02_1174	c1282	/	putative radC-like protein yeeS
	c1284	/	hypothetical protein
i02_1175	c1285	/	hypothetical protein
	c1286	/	hypothetical protein
	c1287	/	hypothetical protein
i02_1176	c1288	/	hypothetical protein
i02_1177	c1289	/	hypothetical protein
i02_1178	c1290	/	hypothetical protein
-	c1291	/	hypothetical protein
i02_1179	c1292	/	hypothetical protein
	c1293	/	hypothetical protein
	c1294	/	hypothetical protein
i02_1181	c1295	ycdW	putative 2-hydroxyacid dehydrogenase ycdW
i02_1182	c1296	ycdX	putative hydrolase
i02_1183	c1297	ycdY	hypothetical protein
i02_1184	c1298	ycdZ	hypothetical protein
i02_1185	c1299	csgG	curli production assembly/transport component
i02_1186	c1300	csgF	curli assembly protein CsgF
i02_1187	c1301	csgE	curli assembly protein CsgE
i02_1189	c1303	/	hypothetical protein
i02_1188	c1302	csgD	DNA-binding transcriptional regulator CsgD
i02_1190	c1304	/	hypothetical protein
i02_1191	c1305	csgB	curlin minor subunit
i02_1192	c1306	csgA	cryptic curlin major subunit
i02_1193	c1307	csgC	putative autoagglutination protein
i02_1194	c1308	ymdA	hypothetical protein
i02_1195	c1309	ymdB	hypothetical protein
i02_1196	c1310	ymdC	hypothetical protein

Clone D	CFT 073	Gene	Product
i02_1197	c1311	mdoC	glucans biosynthesis protein
i02_1198	c1312	/	hypothetical protein
i02_1199	c1313	mdoG	glucan biosynthesis protein G
i02_1200	c1314	mdoH	glucosyltransferase MdoH
i02_1201	c1315	/	hypothetical protein
i02_1202	c1316	yceK	hypothetical protein
	c1317	/	hypothetical protein
i02_1203	c1318	msyB	hypothetical protein
i02_1204	c1319	yceE	drug efflux system protein MdtG
i02_1205	c1320	htrB	lipid A biosynthesis lauroyl acyltransferase
i02_1206	c1321	yceA	hypothetical protein
i02_1207	c1322	yceI	hypothetical protein
i02_1209	c1324	/	hypothetical protein
i02_1208	c1323	/	cytochrome b561 2
i02_1210	c1325	yceO	hypothetical protein
i02_1211	c1326	solA	N-methyltryptophan oxidase
i02_1212	c1327	bssS	biofilm formation regulatory protein BssS
i02_1213	c1328	dinI	DNA damage-inducible protein I
i02_1214	c1329	pyrC	dihydroorotase
i02_1215	c1330	yceB	hypothetical protein
i02_1216	c1331	grxB	glutaredoxin 2
i02_1217	c1332	yceL	multidrug resistance protein MdtH
i02_1218	c1333	rimJ	ribosomal-protein-S5-alanine
i02_1219	c1334	yceH	hypothetical protein
i02_1220		/	hypothetical protein
i02_1221	c1337	/	hypothetical protein
i02_1222	c1338	mviN	virulence factor mviN-like protein
i02_1223	c1339	flgN	flagella synthesis protein FlgN
i02_1224	c1340	flgM	anti-sigma28 factor FlgM
i02_1225	c1341	flgA	flagellar basal body P-ring biosynthesis protein
i02_1226	c1342	flgB	flagellar basal body rod protein FlgB
i02_1227	c1343	flgC	flagellar basal body rod protein FlgC
i02_1228	c1344	flgD	flagellar basal body rod modification protein
i02_1229	c1345	flgE	flagellar hook protein FlgE
i02_1230	c1346	flgF	flagellar basal body rod protein FlgF
i02_1231	c1347	flgG	flagellar basal body rod protein FlgG
i02_1232	c1348	flgH	flagellar basal body L-ring protein
i02_1233	c1349	flgI	flagellar basal body P-ring protein
i02_1234	c1350	flgJ	flagellar rod assembly protein/muramidase FlgJ
i02_1235	c1351	flgK	flagellar hook-associated protein FlgK
i02_1236	c1352	flgL	flagellar hook-associated protein FlgL
i02_1237	c1353	rne	ribonuclease E
i02_1238	c1354	/	hypothetical protein
i02_1239	c1355	rluC	23S rRNA pseudouridylate synthase C
i02_1240	c1356	maf	Maf-like protein
i02_1241	c1357	/	hypothetical protein
i02_1242	c1358	yceD	hypothetical protein
i02_1243		/	50S ribosomal protein L32
i02_1244	c1359	plsX	putative glycerol-3-phosphate acyltransferase
i02_1245	c1360	fabH	3-oxoacyl-(acyl carrier protein) synthase III
i02_1246	c1361	fabD	acyl carrier protein S-malonyltransferase
i02_1247		/	gluconate 5-dehydrogenase

Clone D	CFT 073	Gene	Product
	c1363	/	hypothetical protein
i02_1248	c1364	acpP	acyl carrier protein
i02_1249	c1365	fabF	3-oxoacyl-(acyl carrier protein) synthase II
i02_1250	c1366	pabC	4-amino-4-deoxychorismate lyase
i02_1251	c1369	yceG	hypothetical protein
i02_1252	c1370	tmk	thymidylate kinase
i02_1253	c1371	holB	DNA polymerase III subunit delta'
i02_1254	c1372	ycfH	putative metallodependent hydrolase
i02_1255	c1373	ptsG	glucose-specific PTS system IIBC components
i02_1256	c1374	fhuE	ferric-rhodotorulic acid outer membrane
i02_1257	c1375	/	hypothetical protein
i02_1258	c1376	ycfF	purine nucleoside phosphoramidase
i02_1259	c1377	ycfL	hypothetical protein
i02_1260	c1378	ycfM	hypothetical protein
i02_1261	c1379	thiK	thiamine kinase
i02_1262	c1380	nagZ	beta-hexosaminidase
i02_1263	c1381	ycfP	hypothetical protein
i02_1264	c1382	ndh	NADH dehydrogenase
i02_1265	c1383	ycfJ	hypothetical protein
-	c1384	/	hypothetical protein
i02_1266	c1385	ycfQ	putative transcriptional regulator YcfQ
i02_1267	c1386	ycfR	hypothetical protein
i02_1268	c1387	ycfS	hypothetical protein
i02_1269	c1389	mfd	transcription-repair coupling factor
i02_1270	c1390	ycfT	hypothetical protein
i02_1271		ycfU	outer membrane-specific lipoprotein transporter subunit LolC
i02_1272	c1391	lolC	outer membrane-specific lipoprotein transporter
i02_1273	c1392	lolD	lipoprotein transporter ATP-binding subunit
i02_1274	c1393	lolE	outer membrane-specific lipoprotein transporter
i02_1275	c1394	ycfX	N-acetyl-D-glucosamine kinase
i02_1276	c1395	cobB	NAD-dependent deacetylase
i02_1277	c1396	ycfZ	hypothetical protein
i02_1278	c1397	ymfA	hypothetical protein
i02_1279	c1398	potD	spermidine/putrescine ABC transporter
i02_1280	c1399	potC	spermidine/putrescine ABC transporter membrane
i02_1281	c1400	/	prophage lambda integrase
i02_1282	c1401	xisN	putative excisionase for prophage
i02_1283	c1402	recE	exodeoxyribonuclease VIII
	c1403	/	hypothetical protein
i02_1284	c1404	ydfD	hypothetical protein
i02_1285	c1405	dicB	division inhibition protein dicB
i02_1286	c1406	/	hypothetical protein
i02_1287		/	hypothetical protein
i02_1288	c1407	ydfC	hypothetical protein
i02_1289	c1408	ydfB	hypothetical protein
i02_1290	c1409	ydfA	hypothetical protein
i02_1291	c1410	/	hypothetical protein
i02_1292	c1411	/	hypothetical protein
i02_1293	c1412	/	hypothetical protein
i02_1294	c1413	/	hypothetical protein

Clone D	CFT 073	Gene	Product
i02_1295	c1414	/	hypothetical protein
	c1415	/	hypothetical protein
i02_1296	c1416	/	hypothetical protein
i02_1297	c1417	/	hypothetical protein
i02_1298	c1418	/	hypothetical protein
i02_1299	c1419	/	hypothetical protein
	c1420	/	hypothetical protein
i02_1300	c1421	/	hypothetical protein
	c1422	/	hypothetical protein
i02_1302	c1423	/	hypothetical protein
i02_1303	c1425	/	hypothetical protein
i02_1304	c1426	/	cryptic prophage antitermination protein Q
-	c1427	/	hypothetical protein
i02_1305	c1428	/	hypothetical protein
i02_1306	c1429	ycgW	hypothetical protein
i02_1308	c1431	/	hypothetical protein
i02_1307	c1430	/	hypothetical protein
i02_1309	c1432	/	hypothetical protein
i02_1310	c1433	/	lambdoid prophage DLP12 lysis protein S
i02_1311	c1434	ydfR	hypothetical protein
i02_1312	c1435	/	hypothetical protein
i02_1313	c1436	/	lysozyme from lambdoid prophage Qin
i02_1314	c1437	/	putative Rz endopeptidase from lambdoid prophage
i02_1315	c1438	/	hypothetical protein
i02_1316	c1439	/	hypothetical protein
i02_1317	c1440	ydfO	hypothetical protein
	c1441	/	hypothetical protein
i02_1318	c1442	/	hypothetical protein
i02_1319	c1443	/	hypothetical protein
i02_1320	c1444	/	prophage Qin DNA packaging protein NU1-like
i02_1321	c1445	/	putative DNA packaging protein of prophage
i02_1322	c1446	/	putative DNA packaging protein of prophage
i02_1323	c1447	/	putative capsid protein of prophage
i02_1324	c1448	/	putative capsid assembly protein of prophage
	c1449	/	hypothetical protein
i02_1325	c1450	/	putative capsid protein of prophage
i02_1326	c1451	/	putative capsid protein of prophage
i02_1327	c1452	/	hypothetical protein
i02_1328	c1453	/	putative head-tail joining protein of prophage
i02_1329	c1454	/	putative tail component of prophage
i02_1330	c1455	/	putative tail component of prophage
i02_1331	c1456	/	putative tail fiber component V of prophage
	c1457	/	hypothetical protein
i02_1332	c1458	/	putative tail component of prophage
i02_1333	c1459	/	putative tail component of prophage
i02_1334	c1460	/	putative tail component of prophage
i02_1335	c1461	/	putative tail fiber component M of prophage
i02_1336	c1462	/	putative tail component of prophage
	c1463	/	hypothetical protein
i02_1337	c1464	/	putative tail fiber component K of prophage
i02_1338	c1465	/	putative tail assembly protein

Clone D	CFT 073	Gene	Product
i02_1339	c1466	/	putative tail component of prophage
i02_1340	c1467	lomP	putative Lom-like outer membrane protein
i02_1341	c1468	/	hypothetical protein
	c1469	/	hypothetical protein
i02_1342	c1470	/	hypothetical protein
i02_1343	c1471	/	hypothetical protein
	c1472	/	hypothetical protein
	c1473	/	hypothetical protein
i02_1344	c1474	/	hypothetical protein
i02_1345	c1475	ylcE	hypothetical protein
i02_1346	c1476	potB	spermidine/putrescine ABC transporter membrane
i02_1347	c1477	potA	putrescine/spermidine ABC transporter ATPase
i02_1349	c1479	pepT	peptidase T
i02_1348	c1478	/	hypothetical protein
i02_1350	c1480	ycfD	hypothetical protein
-	c1481	/	hypothetical protein
-	c1483	/	putative integrase of prophage
-	c1485	/	hypothetical protein
-	c1486	/	hypothetical protein
-	c1487	/	hypothetical protein
-	c1488	/	hypothetical protein
-	c1489	/	hypothetical protein
-	c1490	/	hypothetical protein
-	c1491	/	hypothetical protein
-	c1492	/	hypothetical protein
-	c1493	/	hypothetical protein
-	c1494	/	hypothetical protein
-	c1495	/	hypothetical protein
-	c1496	/	hypothetical protein
-	c1497	/	putative single stranded DNA-binding protein
-	c1498	/	hypothetical protein
-	c1499	/	putative capsid protein of prophage
-	c1500	/	hypothetical protein
-	c1501	/	hypothetical protein
-	c1502	/	hypothetical protein
-	c1503	/	hypothetical protein
-	c1504	/	hypothetical protein
-	c1505	/	hypothetical protein
-	c1506	/	hypothetical protein
-	c1507	/	hypothetical protein
i02_1351	c1508	phoQ	sensor protein PhoQ
i02_1352	c1509	phoP	DNA-binding transcriptional regulator PhoP
i02_1353	c1510	purB	adenylosuccinate lyase
i02_1354	c1511	ycfC	hypothetical protein
i02_1355	c1512	mnmA	tRNA-specific 2-thiouridylase MnmA
i02_1356	c1513	/	putative Nudix hydrolase ymfB
i02_1358	c1515	/	hypothetical protein
i02_1357	c1514	/	hypothetical protein
	c1516	/	hypothetical protein
i02_1359	c1517	icdA	isocitrate dehydrogenase
	c1518	/	hypothetical protein

Clone D	CFT 073	Gene	Product
i02_1360	c1519	/	prophage lambda integrase
-	c1520	/	hypothetical protein
-	c1521	/	transposase IS629
i02_1361	c1522	/	hypothetical protein
i02_1362	c1523	/	hypothetical protein
i02_1363	c1524	/	hypothetical protein
i02_1364	c1525	/	hypothetical protein
i02_1365	c1526	/	hypothetical protein
i02_1366	c1527	/	hypothetical protein
-	c1529	/	hypothetical protein
-	c1528	/	hypothetical protein
-	c1530	/	hypothetical protein
-	c1529	/	hypothetical protein
-	c1530	/	hypothetical protein
i02_1367	c1531	/	hypothetical protein
i02_1369	c1532	/	hypothetical protein
i02_1370		/	hypothetical protein
i02_1371	c1533	/	hypothetical protein
	c1535	/	hypothetical protein
i02_1372	c1534	/	putative exonuclease encoded by prophage
i02_1373	c1536	/	putative recombination protein Bet of prophage
i02_1374	c1537	gamW	putative host-nuclease inhibitor protein Gam
	c1539	/	hypothetical protein
i02_1375	c1538	/	hypothetical protein
i02_1376	c1540	/	lambda regulatory protein CIII
i02_1377	c1541	/	putative single-stranded DNA binding protein
i02_1378	c1542	/	lambda ant-restriction protein
i02_1379	c1543	/	putative superinfection exclusion protein B
i02_1380		/	N protein
i02_1381	c1544	/	hypothetical protein
i02_1382	c1545	/	hypothetical protein
i02_1383	c1546	/	repressor protein
i02_1384	c1547	/	hypothetical protein
i02_1385	c1548	/	putative regulatory protein CII
i02_1386	c1549	/	putative replication protein O of bacteriophage
i02_1387	c1550	/	putative replication protein P of bacteriophage
i02_1388	c1551	/	putative exclusion protein ren of prophage
-	c1552	/	hypothetical protein
-	c1553	/	transposase IS629
i02_1389	c1554	/	hypothetical protein
i02_1390	c1555	/	putative DNA N-6-adenine-methyltransferase
i02_1391	c1556	/	hypothetical protein
i02_1392	c1557	rus	endodeoxyribonuclease RUS
i02_1393	c1558	/	hypothetical protein
i02_1394	c1559	ybcQ	lambdoid prophage DLP12 antitermination protein
i02_1395	c1560	nmpC	Outer membrane porin protein nmpC precursor
i02_1396	c1561	/	lambdoid prophage DLP12 lysis protein S
i02_1397	c1562	ybcS	lysozyme from lambdoid prophage DLP12
i02_1398	c1563	/	putative Rz endopeptidase from lambdoid prophage
i02_1399	c1564	ybcU	Bor protein homolog from lambdoid prophage DLP12

Clone D	CFT 073	Gene	Product
i02_1400	c1565	/	partial tonB-like membrane protein encoded
	c1566	/	hypothetical protein
	c1567	/	hypothetical protein
i02_1401		/	hypothetical protein
i02_1403		/	hypothetical protein
i02_1404		nohA2	prophage Qin DNA packaging protein NU1-like protein
i02_1405	c1568	nohA	prophage Qin DNA packaging protein NU1-like protein
i02_1406	c1569	/	putative DNA packaging protein of prophage
i02_1407	c1570	/	putative DNA packaging protein of prophage
i02_1408	c1571	/	putative capsid protein of prophage
	c1572	/	putative capsid assembly protein of prophage
	c1573	/	hypothetical protein
i02_1409	c1574	/	putative capsid protein of prophage
i02_1410	c1575	/	putative capsid protein of prophage
i02_1411	c1576	/	hypothetical protein
i02_1412	c1577	/	putative head-tail joining protein of prophage
i02_1413	c1578	/	putative tail fiber component Z of prophage
i02_1414	c1579	/	putative tail component of prophage
i02_1415	c1580	/	tail protein
	c1581	/	hypothetical protein
i02_1416	c1583	/	putative tail component of prophage
i02_1417	c1584	/	putative tail component of prophage
i02_1418	c1585	/	putative tail component of prophage
i02_1419		/	putative tail component of prophage
	c1586	/	hypothetical protein
i02_1420	c1587	/	putative tail component of prophage
i02_1421	c1588	/	putative tail component of prophage
i02_1422	c1589	/	putative tail component of prophage
i02_1423	c1590	/	putative tail component of prophage
i02_1424	c1591	/	hypothetical protein
	c1592	/	hypothetical protein
i02_1424	c1593	/	hypothetical protein
	c1592	/	hypothetical protein
i02_1425	c1594	/	hypothetical protein
i02_1426	c1595	/	hypothetical protein
	c1596	/	hypothetical protein
i02_1427	c1597	sitD	SitD protein
i02_1428	c1598	sitC	SitC protein
i02_1429	c1599	sitB	SitB protein
i02_1430	c1600	sitA	SitA protein
i02_1431	c1601	/	hypothetical protein
	c1602	/	hypothetical protein
i02_1432	c1603	ycgX	hypothetical protein
i02_1433	c1604	ycgE	putative transcriptional regulator YcgE
i02_1434	c1605	/	hypothetical protein
i02_1435	c1606	ycgF	hypothetical protein
i02_1436	c1607	ycgZ	hypothetical protein
	c1608	/	hypothetical protein
i02_1437		/	hypothetical protein
i02_1438	c1609	ymgB	hypothetical protein

Clone D	CFT 073	Gene	Product
i02_1439		/	hypothetical protein
i02_1440	c1610	/	hypothetical protein
i02_1441	c1611	/	hypothetical protein
i02_1442		/	hypothetical protein
i02_1443		/	conserved hypothetical protein
i02_1444		/	hypothetical protein
i02_1445		/	hypothetical protein
i02_1446	c1615	/	hypothetical protein
i02_1447	c1616	ymgD	hypothetical protein
i02_1448	c1617	/	hypothetical protein
i02_1449	c1618	/	hypothetical protein
i02_1450	c1619	/	hypothetical protein
i02_1451	c1620	/	hypothetical protein
i02_1452	c1621	minE	cell division topological specificity factor
i02_1453	c1622	minD	cell division inhibitor MinD
i02_1454	c1623	minC	septum formation inhibitor
i02_1455	c1624	/	hypothetical protein
i02_1456	c1625	ycgJ	hypothetical protein
i02_1457	c1626	ycgK	hypothetical protein
i02_1458	c1627	ycgL	hypothetical protein
i02_1459	c1628	/	hypothetical protein
i02_1460	c1629	ycgN	hypothetical protein
i02_1461	c1630	/	hypothetical protein
i02_1462	c1631	umuD	DNA polymerase V subunit UmuD
i02_1463	c1632	umuC	DNA polymerase V subunit UmuC
i02_1464	c1633	dsbB	disulfide bond formation protein B
i02_1465	c1634	nhaB	sodium/proton antiporter
i02_1466	c1635	fadR	fatty acid metabolism regulator
i02_1467	c1636	/	hypothetical protein
i02_1468	c1637	ycgB	SpoVR family protein
i02_1469	c1638	dadA	D-amino acid dehydrogenase small subunit
i02_1470	c1639	dadX	alanine racemase
i02_1471	c1640	ycgO	potassium/proton antiporter
i02_1473	c1643	mltE	membrane-bound lytic murein transglycosylase E
i02_1472	c1641	ldcA	L,D-carboxypeptidase A
i02_1474	c1644	ycgR	hypothetical protein
i02_1475	c1645	ymgE	transglycosylase associated protein
i02_1476	c1646	prrA	outer membrane receptor
i02_1477	c1647	modD	molybdenum transport protein ModD
	c1649	/	hypothetical protein
i02_1478	c1648	/	hypothetical protein
i02_1479	c1650	/	putative iron compound ABC transporter
i02_1480	c1651	fecD	Iron(III) dicitrate transport system permease
i02_1481	c1652	/	hypothetical protein
i02_1482	c1653	/	hypothetical protein
i02_1484	c1655	/	hypothetical protein
i02_1483	c1654	treA	trehalase
i02_1485		/	hypothetical protein
i02_1486	c1656	ycgC	dihydroxyacetone kinase subunit M
i02_1487	c1657	/	dihydroxyacetone kinase ADP-binding subunit
i02_1488	c1658	ycgT	dihydroxyacetone kinase subunit DhaK
i02_1489	c1659	/	DNA-binding transcriptional regulator DhaR

Clone D	CFT 073	Gene	Product
i02_1490	c1660	/	hypothetical protein
i02_1491	c1661	ychF	GTP-dependent nucleic acid-binding protein EngD
i02_1492	c1662	pth	peptidyl-tRNA hydrolase
i02_1493	c1663	ychH	hypothetical protein
i02_1494	c1664	ychM	putative sulfate transporter YchM
i02_1495	c1665	prsA	ribose-phosphate pyrophosphokinase
i02_1496	c1666	ipk	4-diphosphocytidyl-2-C-methylerythritol kinase
i02_1497	c1667	lolB	outer membrane lipoprotein LolB
i02_1498	c1668	hemA	glutamyl-tRNA reductase
i02_1499	c1669	prfA	peptide chain release factor 1
i02_1500	c1670	hemK	N5-glutamine S-adenosyl-L-methionine-dependent
i02_1501	c1671	/	putative transcriptional regulator
i02_1502	c1672	ychA	putative transcriptional regulator
	c1673	/	hypothetical protein
i02_1503	c1674	kdsA	2-dehydro-3-deoxyphosphooctonate aldolase
i02_1504	c1675	/	hypothetical protein
i02_1505		/	hypothetical protein
i02_1506	c1676	chaA	calcium/sodium:proton antiporter
i02_1507	c1677	chaB	cation transport regulator
i02_1508	c1678	chaC	cation transport protein chaC
i02_1509	c1679	ychN	hypothetical protein
i02_1510	c1680	ychP	hypothetical protein
i02_1511	c1681	narL	transcriptional regulator NarL
i02_1512	c1682	narX	nitrate/nitrite sensor protein NarX
i02_1513	c1683	/	hypothetical protein
i02_1514	c1684	narK	nitrite extrusion protein 1
i02_1515	c1685	narG	respiratory nitrate reductase 1 alpha chain
i02_1516	c1686	narH	respiratory nitrate reductase 1 beta chain
i02_1517	c1687	narJ	respiratory nitrate reductase 1 delta chain
i02_1518	c1688	narI	respiratory nitrate reductase 1 gamma chain
i02_1519	c1689	/	hypothetical protein
i02_1520	c1690	/	hypothetical protein
i02_1521	c1691	/	putative gumP-like protein
i02_1522	c1692	/	hypothetical protein
i02_1523	c1693	/	hypothetical protein
i02_1524	c1694	/	hypothetical protein
i02_1525	c1695	/	hypothetical protein
i02_1527	c1696	purU	formyltetrahydrofolate deformylase
i02_1528	c1697	ychJ	hypothetical protein
i02_1529	c1698	ychK	hypothetical protein
i02_1530	c1699	hnr	response regulator of RpoS
i02_1531	c1700	galU	UTP--glucose-1-phosphate uridylyltransferase
i02_1532	c1701	hns	global DNA-binding transcriptional factor
i02_1533	c1702	/	hypothetical protein
i02_1534	c1703	tdk	thymidine kinase
i02_1535	c1704	/	transposase insG
i02_1537	c1705	adhE	bifunctional acetaldehyde-CoA/alcohol dehydrogenase
i02_1538	c1706	ychE	hypothetical protein
i02_1539		/	hypothetical protein

Clone D	CFT 073	Gene	Product
i02_1540	c1707	oppA	periplasmic oligopeptide-binding protein
i02_1541	c1708	oppB	oligopeptide transporter permease
i02_1542	c1709	oppC	oligopeptide transport system permease protein
i02_1543	c1710	oppD	oligopeptide transporter ATP-binding component
i02_1544	c1711	oppF	oligopeptide transport ATP-binding protein oppF
i02_1545	c1712	yciU	dsDNA-mimic protein
i02_1546	c1713	cls	cardiolipin synthetase
i02_1547	c1714	/	hypothetical protein
i02_1548	c1715	kch	voltage-gated potassium channel
i02_1549	c1716	yciI	YciI-like protein
i02_1550	c1717	tonB	transport protein TonB
	c1718	/	hypothetical protein
i02_1551	c1719	yciA	acyl-CoA thioester hydrolase
i02_1552	c1720	ispZ	intracellular septation protein A
i02_1553	c1721	yciC	hypothetical protein
i02_1554	c1722	ompW	outer membrane protein W
i02_1555	c1723	yciE	hypothetical protein
i02_1556	c1724	yciF	hypothetical protein
i02_1557	c1725	trpA	tryptophan synthase subunit alpha
i02_1558	c1726	trpB	tryptophan synthase subunit beta
i02_1559		trpC	bifunctional indole-3-glycerol phosphate synthase/phosphoribosylanthranilate isomerase
i02_1560	c1729	trpD	bifunctional glutamine
i02_1561	c1730	trpE	anthranilate synthase component
	c5494	trpL	Trp operon leader peptide
i02_1562	c1731	trpH	protein trpH
i02_1563	c1732	yciO	hypothetical protein
i02_1564	c1733	yciQ	hypothetical protein
i02_1565	c1734	yciL	23S rRNA pseudouridylate synthase B
i02_1566	c1735	btuR	cobinamide adenosyltransferase / cobalamin adenosyltransferase
i02_1567	c1736	yciK	short chain dehydrogenase
i02_1568	c1737	sohB	putative periplasmic protease
-	c1738	/	hypothetical protein
i02_1570	c1740	/	hypothetical protein
i02_1569	c1739	yciN	hypothetical protein
i02_1571	c1741	topA	DNA topoisomerase I
i02_1572	c1742	cysB	transcriptional regulator CysB
i02_1573	c1743	/	hypothetical protein
i02_1574	c1744	/	hypothetical protein
i02_1575	c1745	acnA	aconitate hydratase
i02_1576	c1746	ribA	GTP cyclohydrolase II
i02_1577	c1747	pgpB	phosphatidylglycerophosphatase B
i02_1578	c1748	yciS	hypothetical protein
i02_1579	c1749	yciM	tetratricopeptide repeat protein
i02_1580	c1750	pyrF	orotidine 5'-phosphate decarboxylase
i02_1581	c1751	yciH	translation initiation factor Sui1
i02_1582	c1752	/	hypothetical protein
i02_1583	c1753	osmB	lipoprotein
i02_1584	c1754	yciT	putative transcriptional regulator YciT
i02_1585	c1755	/	hypothetical protein
i02_1586	c1756	yciR	RNase II stability modulator

Clone D	CFT 073	Gene	Product
i02_1587	c1757	rnb	exoribonuclease II
i02_1588	c1758	yciW	hypothetical protein
i02_1589	c1759	fabI	enoyl-(acyl carrier protein) reductase
i02_1590	c1760	/	putative transcriptional repressor
i02_1591	c1761	/	acriflavin resistance protein A
	c1762	/	hypothetical protein
	c1763	/	hypothetical protein
i02_1593	c1764	/	acriflavin resistance protein B
i02_1594	c1765	/	partial putative outer membrane channel protein
i02_1595	c1766	/	membrane transporter
i02_1596	c1767	sapF	peptide transport system ATP-binding protein
i02_1597	c1768	sapD	peptide transport system ATP-binding protein
i02_1598	c1769	sapC	peptide transport system permease protein sapC
i02_1599	c1770	sapB	peptide transport system permease protein sapB
i02_1600	c1771	sapA	peptide transport periplasmic protein sapA
i02_1601	c1772	ymjA	hypothetical protein
i02_1602	c1773	pspF	phage shock protein
i02_1603	c1774	pspA	phage shock protein PspA
i02_1604	c1775	pspB	phage shock protein B
	c1776	/	hypothetical protein
i02_1605	c1777	pspC	DNA-binding transcriptional activator PspC
i02_1606	c1778	pspD	peripheral inner membrane phage-shock protein
i02_1607	c1779	pspE	thiosulfate:cyanide sulfurtransferase
i02_1608	c1780	ycjM	putative sucrose phosphorylase
	c1781	/	hypothetical protein
i02_1609	c1782	ycjN	ABC transporter periplasmic-binding protein
i02_1610	c1783	ycjO	ABC transporter permease
i02_1611	c1784	ycjP	ABC transporter permease
i02_1612	c1785	ycjQ	hypothetical protein
i02_1613	c1786	ycjR	hypothetical protein
i02_1614	c1787	ycjS	oxidoreductase ycjS
i02_1615	c1788	ycjT	putative transport protein YcjT
i02_1616	c1789	ycjU	putative beta-phosphoglucomutase
i02_1617	c1790	ycjV	ABC transporter ATP-binding protein
i02_1618	c1791	ompG	Outer membrane protein G precursor
i02_1619	c1792	ycjW	putative transcriptional regulator YcjW
i02_1620	c1793	ycjX	hypothetical protein
i02_1621	c1794	ycjF	hypothetical protein
i02_1622	c1795	tyrR	DNA-binding transcriptional regulator TyrR
i02_1623	c1796	tpx	thiol peroxidase
i02_1624	c1797	ycjG	hypothetical protein
i02_1625		ycjI	murein peptide amidase A
i02_1626	c1800	/	hypothetical protein
i02_1627	c1801	/	hypothetical protein
i02_1628	c1802	/	putative transcriptional regulator YcjZ
i02_1629	c1803	mppA	periplasmic murein peptide-binding protein
i02_1630	c1804	ynaI	hypothetical protein
i02_1631	c1805	ynaJ	hypothetical protein
i02_1632	c1806	ydaA	universal stress protein UspE
i02_1633	c1807	fnr	fumarate/nitrate reduction transcriptional regulator
i02_1634	c1808	ogt	O-6-alkylguanine-DNA:cysteine-protein
i02_1635	c1809	/	hypothetical protein

Clone D	CFT 073	Gene	Product
i02_1636	c1810	/	hypothetical protein
i02_1637	c1811	/	hypothetical protein
i02_1638	c1812	/	hypothetical protein
	c1813	/	hypothetical protein
i02_1639	c1814	ydaL	hypothetical protein
i02_1640	c1815	ydaM	hypothetical protein
i02_1641	c1816	zntB	zinc transporter
i02_1642	c1817	dbpA	ATP-dependent RNA helicase DbpA
i02_1643	c1818	ydaO	C32 tRNA thiolase
i02_1644	c1819	/	hypothetical protein
i02_1645	c1820	/	hypothetical protein
i02_1646	c1821	ynaF	hypothetical protein
i02_1647	c1822	ompN	Outer membrane protein N precursor
i02_1648	c1823	ydbK	pyruvate-flavodoxin oxidoreductase
i02_1650	c1825	/	hypothetical protein
i02_1649	c1824	/	hypothetical protein
i02_1651	c1826	hslJ	heat-inducible protein
i02_1652	c1827	ldhA	D-lactate dehydrogenase
i02_1653	c1828	ydbH	hypothetical protein
i02_1654	c1829	ynbE	hypothetical protein
i02_1655	c1830	ydbL	hypothetical protein
i02_1656		/	possible autotransporter/adhesin
i02_1657	c1832	ydbC	putative oxidoreductase
i02_1658	c1833	/	hypothetical protein
i02_1659	c1834	ynbA	hypothetical protein
i02_1660	c1835	ynbB	hypothetical protein
i02_1661	c1836	ynbC	hypothetical protein
i02_1662	c1837	ynbD	hypothetical protein
	c1838	/	hypothetical protein
i02_1663	c1839	acpD	azoreductase
i02_1664	c1840	hrpA	ATP-dependent RNA helicase HrpA
i02_1665	c1841	ydcF	hypothetical protein
i02_1666	c1842	aldA	aldehyde dehydrogenase A
i02_1667	c1843	/	glyceraldehyde 3-phosphate dehydrogenase A
i02_1668	c1844	cybB	cytochrome b561
i02_1669	c1845	ydcA	hypothetical protein
i02_1670	c1846	/	hypothetical protein
i02_1671	c1847	ycdI	putative transcriptional regulator YdcI
i02_1672	c1848	ydcJ	hypothetical protein
i02_1673	c1849	mdoD	glucan biosynthesis protein D
i02_1674	c1850	ydcH	hypothetical protein
i02_1675	c1851	rimL	ribosomal-protein-L7/L12-serine
i02_1676	c1852	ydcK	hypothetical protein
i02_1677	c1853	tehA	potassium-tellurite ethidium and proflavin
i02_1678	c1854	tehB	tellurite resistance protein TehB
i02_1679		/	hypothetical protein
	c1855	/	hypothetical protein
i02_1680	c1856	ydcL	lipoprotein ydcL precursor
i02_1682	c1858	ydcN	hypothetical protein
i02_1681	c1857	ydcO	hypothetical protein
i02_1683	c1859	ydcP	putative protease ydcP precursor
i02_1684	c1860	yncJ	hypothetical protein

Clone D	CFT 073	Gene	Product
i02_1685	c1861	/	hypothetical protein
i02_1686	c1862	/	hypothetical protein
i02_1687	c1863	ydcR	hypothetical protein
i02_1688	c1864	ydcS	ABC transporter periplasmic-binding protein
i02_1689	c1865	ydcT	ABC transporter ATP-binding protein
i02_1690	c1866	ydcU	ABC transporter permease
i02_1691	c1867	ydcV	ABC transporter permease
i02_1692	c1869	ydcW	gamma-aminobutyraldehyde dehydrogenase
i02_1693		/	hypothetical protein
i02_1695	c1870	/	hypothetical protein
i02_1694		/	hypothetical protein
i02_1696	c1871	ydc	hypothetical protein
i02_1697	c1872	ydcZ	hypothetical protein
i02_1699		/	hypothetical protein
i02_1698	c1873	yncA	acetyltransferase yncA
i02_1700	c1874	/	hypothetical protein
i02_1701		yncD	iron outer membrane transporter
i02_1702	c1877	yncE	hypothetical protein
i02_1703	c1878	ansP	L-asparagine permease
i02_1704	c1879	/	hypothetical protein
i02_1705	c1880	/	hypothetical protein
i02_1707	c1882	/	hypothetical protein
i02_1706	c1881	/	hypothetical protein
i02_1708	c1883	/	hypothetical protein
i02_1709	c1884	/	hypothetical protein
i02_1710	c1885	/	hypothetical protein
i02_1711	c1886	/	hypothetical protein
i02_1712	c1887	/	hypothetical protein
i02_1713	c1888	/	hypothetical protein
i02_1714	c1889	/	hypothetical protein
i02_1715	c1890	/	hypothetical protein
i02_1717	c1891	/	hypothetical protein
i02_1717	c1892	/	hypothetical protein
i02_1718	c1893	/	hypothetical protein
i02_1719		/	hypothetical protein
i02_1720	c1894	yddH	hypothetical protein
i02_1721	c1895	nhoA	N-hydroxyarylamine O-acetyltransferase
i02_1722	c1896	yddE	hypothetical protein
i02_1723	c1897	narV	respiratory nitrate reductase 2 gamma chain
i02_1724	c1898	narW	respiratory nitrate reductase 2 delta chain
i02_1725	c1899	narY	respiratory nitrate reductase 2 beta chain
i02_1726	c1900	narZ	respiratory nitrate reductase 2 alpha chain
i02_1727	c1901	narU	nitrite extrusion protein 2
i02_1728	c1902	yddG	hypothetical protein
i02_1729	c5623	fdnG	formate dehydrogenase
	c1905	/	hypothetical protein
i02_1730	c1906	fdnH	formate dehydrogenase-N beta subunit
i02_1731	c1907	fdnI	formate dehydrogenase-N subunit gamma
	c1908	/	hypothetical protein
i02_1732	c1909	yddM	hypothetical protein
i02_1733	c1910	/	hypothetical protein
i02_1734	c1911	adhP	alcohol dehydrogenase

Clone D	CFT 073	Gene	Product
i02_1735	c1912	sfcA	malate dehydrogenase
i02_1736	c1913	rpsV	30S ribosomal subunit S22
i02_1737	c1914	/	biofilm-dependent modulation protein
i02_1738	c1915	/	hypothetical protein
i02_1739	c1916	osmC	osmotically inducible protein C
i02_1740	c1918	/	putative sensor kinase
i02_1741	c1919	/	hypothetical protein
i02_1742	c1920	yddW	lipoprotein yddW precursor
i02_1743	c1921	xasA	amino acid antiporter
i02_1744	c1922	gadB	glutamate decarboxylase beta
i02_1745	c1923	pqqL	zinc protease pqqL
i02_1746	c1924	yddB	hypothetical protein
i02_1747	c1925	yddA	ABC transporter ATP-binding protein
i02_1748	c1926	ydeM	hypothetical protein
i02_1749	c1927	ydeN	putative sulfatase ydeN precursor
i02_1750	c1928	ydeO	transcriptional regulator YdeO
i02_1751	c1929	/	hypothetical protein
i02_1752	c1930	ydeP	putative oxidoreductase
i02_1753	c1931	ydeQ	fimbrial-like protein ydeQ precursor
i02_1754	c1932	ydeR	fimbrial-like protein ydeR precursor
i02_1755	c1933	ydeS	fimbrial-like protein ydeS precursor
i02_1756	c1934	/	Outer membrane usher protein fimD precursor
i02_1757	c1935	/	chaperone protein fimC precursor
i02_1758	c1936	/	Type-1 fimbrial protein, A chain precursor
i02_1759	c1937	/	hypothetical protein
-	c1938	oprR	putative transcriptional regulator YcjZ
i02_1760	c1939	/	hypothetical protein
i02_1761	c1940	hipA	protein hipA
i02_1762	c1941	hipB	DNA-binding transcriptional regulator HipB
i02_1763	c1942	tam	trans-aconitate 2-methyltransferase
i02_1764	c1943	yneE	hypothetical protein
i02_1765		uxaB	altronate oxidoreductase
i02_1766	c1945	yneF	hypothetical protein
i02_1767	c1946	yneG	hypothetical protein
i02_1768	c1947	yneH	glutaminase
i02_1769	c1948	yneI	putative succinate semialdehyde dehydrogenase
i02_1770	c1949	yneJ	putative transcriptional regulator YneJ
i02_1771		/	hypothetical protein
i02_1772	c1950	sotB	sugar efflux transporter
i02_1773	c1951	marC	multiple drug resistance protein MarC
i02_1774	c1952	marR	DNA-binding transcriptional repressor MarR
i02_1775	c1953	marA	DNA-binding transcriptional activator MarA
i02_1776	c1954	marB	hypothetical protein
i02_1777	c1955	celA	6-phospho-beta-glucosidase
i02_1778	c1956	/	hypothetical protein
i02_1779	c1957	/	PTS system, cellobiose-specific IIA component
i02_1780	c1958	/	hypothetical protein
i02_1781	c1959	/	PTS system, cellobiose-specific IIB component
i02_1782	c1960	/	hypothetical protein
i02_1783	c1961	ydeD	O-acetylserine/cysteine export protein
i02_1784	c1962	ydeF	putative MFS-type transporter YdeE
i02_1785	c1963	ydeH	hypothetical protein

Clone D	CFT 073	Gene	Product
i02_1786		/	conserved hypothetical protein
i02_1787	c1964	dcp	dipeptidyl carboxypeptidase II
i02_1788	c1965	ydfG	3-hydroxy acid dehydrogenase
i02_1789	c1966	ydfH	putative transcriptional regulator YdfH
i02_1790	c1967	ydfZ	hypothetical protein
i02_1791	c1968	/	oxidoreductase ydfI
i02_1792	c1969	ydfJ	metabolite transport protein
i02_1793	c1970	rspB	putative dehydrogenase
i02_1794	c1971	rspA	starvation sensing protein rspA
i02_1795	c1972	ynfA	hypothetical protein
i02_1796	c1973	ynfB	hypothetical protein
i02_1797	c1974	speG	spermidine N(1)-acetyltransferase
i02_1798	c1975	ynfC	hypothetical protein
i02_1799	c1976	ynfD	hypothetical protein
i02_1800	c1977	ynfE	putative dimethyl sulfoxide reductase chain ynfE
i02_1801	c1978	ynfF	dimethyl sulfoxide reductase chain ynfF
i02_1802	c1979	ynfG	anaerobic dimethyl sulfoxide reductase chain
i02_1803	c1981	ynfH	anaerobic dimethyl sulfoxide reductase chain
	c1980	/	hypothetical protein
i02_1804	c1982	ynfI	twin-argninine leader-binding protein DmsD
i02_1805	c1983	ynfJ	putative voltage-gated ClC-type chloride channel
i02_1806	c1984	bioD	putative dithiobiotin synthetase
i02_1807	c1985	mlc	Mlc protein
i02_1808	c1986	ynfL	putative transcriptional regulator YnfL
i02_1809	c1987	ynfM	putative transport protein YnfM
	c1988	asr	acid shock protein precursor
i02_1810	c1990	/	putative protease ydgD precursor
i02_1811	c1991	ydgE	multidrug efflux system protein MdtI
i02_1812	c1992	ydgF	multidrug efflux system protein MdtJ
i02_1813	c1993	tqsA	putative transport protein
i02_1814	c1994	pntB	pyridine nucleotide transhydrogenase
i02_1815	c1995	pntA	NAD(P) transhydrogenase subunit alpha
i02_1816	c1996	ydgH	hypothetical protein
i02_1817	c1997	ydgI	putative arginine/ornithine antiporter
i02_1818	c1998	ydgB	short chain dehydrogenase
i02_1819	c1999	ydgC	hypothetical protein
i02_1820	c2000	rstA	DNA-binding transcriptional regulator RstA
i02_1821	c2001	rstB	sensor protein RstB
i02_1822	c2002	tus	DNA replication terminus site-binding protein
i02_1823	c2003	fumC	fumarate hydratase
i02_1824	c2004	fumA	fumarate hydratase class I, aerobic
i02_1825	c2005	manA	mannose-6-phosphate isomerase
i02_1826	c2006	ydgA	hypothetical protein
i02_1827	c2007	uidC	putative outer membrane porin protein
i02_1828	c2008	uidB	glucuronide transporter
i02_1829	c2009	uidA	beta-D-glucuronidase
i02_1830	c2010	uidR	uid operon repressor
i02_1831	c2011	hdhA	7-alpha-hydroxysteroid dehydrogenase
i02_1832	c2012	malI	DNA-binding transcriptional repressor MalI
i02_1833	c2013	malX	bifunctional maltose and glucose-specific PTS
i02_1834	c2014	malY	cystathionine beta-lyase
i02_1835	c2015	add	adenosine deaminase

Clone D	CFT 073	Gene	Product
i02_1836	c2016	ydgJ	putative oxidoreductase
i02_1837		/	hypothetical protein
i02_1838	c2017	ydgT	oriC-binding nucleoid-associated protein
i02_1839	c2018	ydgK	hypothetical protein
i02_1840	c2019	/	Na(+)-translocating NADH-quinone reductase
i02_1841	c2020	/	electron transport complex protein RnfB
i02_1842	c2021	/	electron transport complex protein RnfC
i02_1843	c2022	rnfD	electron transport complex protein RnfD
i02_1844	c2023	/	electron transport complex protein RnfG
i02_1845	c2024	ydgQ	electron transport complex RsxE subunit
i02_1846	c2025	nth	endonuclease III
i02_1847		/	hypothetical protein
i02_1848	c2026	tppB	putative tripeptide transporter permease
i02_1849	c2027	gst	glutathionine S-transferase
i02_1850	c2028	pdxY	pyridoxamine kinase
i02_1851	c2029	tyrS	tyrosyl-tRNA synthetase
i02_1852	c2030	pdxH	pyridoxamine 5'-phosphate oxidase
i02_1853	c2031	ydhA	lysozyme inhibitor
i02_1854	c2032	anmK	anhydro-N-acetylmuramic acid kinase
i02_1855	c2033	slyB	Outer membrane lipoprotein slyB precursor
i02_1856	c2034	slyA	transcriptional regulator SlyA
i02_1857		/	hypothetical protein
i02_1858	c2035	ydhI	hypothetical protein
i02_1859	c2036	ydhJ	hypothetical protein
i02_1860	c2037	ydhK	hypothetical protein
i02_1861	c2038	sodC	superoxide dismutase
i02_1862	c2039	ydhF	oxidoreductase ydhF
	c2041	/	hypothetical protein
i02_1864	c2042	ydhM	putative transcriptional regulator YdhM
i02_1863	c2040	ydhL	hypothetical protein
i02_1865	c2043	nemA	N-ethylmaleimide reductase
i02_1866	c2044	gloA	glyoxalase I
i02_1867	c2045	rnt	ribonuclease T
i02_1869	c2047	/	hypothetical protein
i02_1868	c2046	/	hypothetical protein
i02_1870	c2048	ydhD	hypothetical protein
i02_1871	c2049	ydhO	hypothetical protein
i02_1872	c2050	sodB	superoxide dismutase
i02_1873	c2051	ydhP	putative transport protein YdhP
i02_1874		/	hypothetical protein
i02_1875	c2052	purR	DNA-binding transcriptional repressor PurR
i02_1876	c2053	ydhB	putative DNA-binding transcriptional regulator
i02_1877	c2054	ydhC	inner membrane transport protein YdhC
i02_1878	c2055	cfa	cyclopropane fatty acyl phospholipid synthase
i02_1879	c2056	ribE	riboflavin synthase subunit alpha
i02_1880	c2057	ydhE	multidrug efflux protein
i02_1881	c2058	ydhQ	hypothetical protein
i02_1882	c2059	/	hypothetical protein
i02_1883	c2060	ydhS	hypothetical protein
	c2061	/	hypothetical protein
i02_1885	c2063	/	hypothetical protein
i02_1884	c2062	ydhT	hypothetical protein

Clone D	CFT 073	Gene	Product
i02_1886	c2064	ydhU	PhsC protein
i02_1887	c2065	/	putative ferredoxin-like protein ydhX
i02_1888	c2066	ydhW	hypothetical protein
i02_1889	c2067	ydhV	putative oxidoreductase
i02_1890	c2068	/	hypothetical protein
i02_1891	c2069	ydhZ	hypothetical protein
i02_1892	c2070	/	hypothetical protein
i02_1893	c2071	pykF	pyruvate kinase
i02_1894	c2072	lpp	major outer membrane lipoprotein precursor
i02_1895	c2073	ynhG	hypothetical protein
i02_1896	c2074	ynhA	cysteine desufuration protein SufE
i02_1897	c2075	/	bifunctional cysteine desulfurase/selenocysteine
i02_1898	c2076	ynhC	cysteine desulfurase activator complex subunit
i02_1899	c2077	sufC	cysteine desulfurase ATPase component
i02_1900	c2078	ynhE	cysteine desulfurase activator complex subunit
i02_1901	c2079	sufA	iron-sulfur cluster assembly scaffold protein
i02_1902	c2080	ydiH	hypothetical protein
i02_1903	c2081	ydiI	hypothetical protein
i02_1904	c2082	ydiJ	hypothetical protein
i02_1905	c2083	ydiK	putative inner membrane protein
i02_1906	c2084	ydiL	hypothetical protein
i02_1907	c2085	ydiM	putative transport protein YdiM
i02_1908	c2086	ydiN	putative transport protein YdiN
i02_1909	c2087	aroE	quinate/shikimate dehydrogenase
i02_1910	c2088	aroD	3-dehydroquinate dehydratase
i02_1911	c2089	ydiF	hypothetical protein
i02_1912	c2090	ydiO	putative acyl-CoA dehydrogenase
i02_1913	c2091	ydiP	putative transcriptional regulator YdiP
i02_1914	c2092	/	hypothetical protein
i02_1915	c2093	ydiQ	putative electron transfer flavoprotein YdiQ
i02_1916	c2094	ydiR	electron transfer flavoprotein subunit YdiR
i02_1917	c2095	ydiS	hypothetical protein
i02_1918	c2096	ydiT	ferredoxin-like protein ydiT
i02_1919	c2097	ydiD	short chain acyl-CoA synthetase
i02_1920	c2098	ppsA	phosphoenolpyruvate synthase
i02_1921	c2099	ydiA	hypothetical protein
i02_1922	c2100	aroH	phospho-2-dehydro-3-deoxyheptonate aldolase
i02_1923	c2101	ydiE	hypothetical protein
i02_1924	c2102	ydiU	hypothetical protein
i02_1925	c2103	ydiV	hypothetical protein
i02_1926	c2104	nlpC	lipoprotein NlpC
i02_1927	c2105	btuD	vitamin B12-transporter ATPase
i02_1928	c2106	btuE	putative glutathione peroxidase
i02_1929	c2107	btuC	vtamin B12-transporter permease
i02_1930	c2108	/	hypothetical protein
i02_1931	c2109	ihfA	integration host factor subunit alpha
i02_1932		pheT	phenylalanyl-tRNA synthetase subunit beta
i02_1933	c2112	pheS	phenylalanyl-tRNA synthetase subunit alpha
	c5495	pheM	phenylalanyl-tRNA synthetase operon leader
i02_1934	c2113	rplT	50S ribosomal protein L20
i02_1935	c2114	/	hypothetical protein
i02_1936	c2115	infC	translation initiation factor IF-3

Clone D	CFT 073	Gene	Product
i02_1937	c2116	thrS	threonyl-tRNA synthetase
i02_1938	c2117	/	hypothetical protein
i02_1939	c2118	/	hypothetical protein
i02_1940	c2119	/	hypothetical protein
i02_1941		/	hypothetical protein
i02_1942	c2120	ydiY	hypothetical protein
i02_1943	c2121	pfkB	6-phosphofructokinase 2
	c2122	/	hypothetical protein
i02_1944	c2123	ydiZ	hypothetical protein
i02_1945	c2124	yniA	hypothetical protein
i02_1946	c2125	yniB	hypothetical protein
i02_1947	c2126	yniC	2-deoxyglucose-6-phosphatase
i02_1948	c2127	ydjM	hypothetical protein
i02_1949	c2128	ydjN	putative symporter ydjN
i02_1950	c2129	/	cell division modulator
	c2130	/	hypothetical protein
i02_1951	c2131	katE	hydroperoxidase II
i02_1952	c2132	ydjC	hypothetical protein
i02_1953	c2133	celF	6-phospho-beta-glucosidase
i02_1954	c2134	celD	DNA-binding transcriptional regulator ChbR
i02_1955	c2135	celC	N,N'-diacetylchitobiose-specific PTS system
i02_1956	c2136	celB	N,N'-diacetylchitobiose-specific PTS system
i02_1957	c2137	celA	N,N'-diacetylchitobiose-specific PTS system
i02_1958	c2138	osmE	DNA-binding transcriptional activator OsmE
i02_1959	c2139	nadE	NAD synthetase
i02_1960	c2140	ydjQ	nucleotide excision repair endonuclease
i02_1961	c2141	ydjR	hypothetical protein
	c2142	/	hypothetical protein
i02_1962	c2143	spy	periplasmic protein
i02_1963	c2144	ydjS	succinylglutamate desuccinylase
i02_1964	c2145	/	succinylarginine dihydrolase
i02_1965	c2146	astD	succinylglutamic semialdehyde dehydrogenase
i02_1966	c2147	/	arginine succinyltransferase
i02_1967	c2148	argD	bifunctional succinylornithine
i02_1968	c2149	/	hypothetical protein
i02_1969	c2150	xthA	exonuclease III
i02_1970	c2151	ydjX	hypothetical protein
i02_1971	c2152	ydjY	hypothetical protein
i02_1972	c2153	ydjZ	hypothetical protein
i02_1973	c2154	ynjA	hypothetical protein
i02_1974	c2155	/	putative ABC transporter solute-binding protein
i02_1975	c2156	ynjC	ABC transporter permease
i02_1976	c2157	ynjD	ABC transporter ATP-binding protein
i02_1977	c2158	/	putative thiosulfate sulfurtransferase ynjE
i02_1978	c2159	ynjF	hypothetical protein
i02_1979	c2160	/	pyrimidine (deoxy)nucleoside triphosphate
i02_1980	c2161	ynjH	hypothetical protein
i02_1981	c2162	gdhA	glutamate dehydrogenase
i02_1982	c2163	/	hypothetical protein
i02_1983	c2164	ybeW	chaperone protein hscC
i02_1984	c2165	/	hypothetical protein
i02_1985	c2166	topB	DNA topoisomerase III

Clone D	CFT 073	Gene	Product
i02_1986	c2167	selD	selenophosphate synthetase
i02_1987	c2168	ydjA	hypothetical protein
i02_1988	c2169	/	hypothetical protein
i02_1989	c2170	sppA	protease 4
i02_1990	c2171	ansA	cytoplasmic asparaginase I
i02_1991	c2172	ydjB	nicotinamidase/pyrazinamidase
i02_1992	c2173	ydjE	metabolite transporter
i02_1993	c2174	ydjF	putative transcriptional regulator YdjF
i02_1994	c2175	ydjG	oxidoreductase ydjG
i02_1995	c2176	ydjH	putative sugar kinase ydjH
i02_1996	c2177	ydjI	hypothetical protein
i02_1997	c2178	ydjJ	hypothetical protein
i02_1998	c2179	ydjK	metabolite transporter
i02_1999	c2180	/	hypothetical protein
i02_2000	c2181	ydjL	hypothetical protein
i02_2001	c2182	yeaC	hypothetical protein
i02_2002	c2183	yeaA	methionine sulfoxide reductase B
i02_2003	c2184	gapA	glyceraldehyde-3-phosphate dehydrogenase
i02_2004	c2185	yeaD	hypothetical protein
i02_2005	c2186	yeaE	hypothetical protein
i02_2006	c2187	yeaF	MltA-interacting protein precursor
i02_2007	c2188	yeaG	hypothetical protein
i02_2008	c2189	yeaH	hypothetical protein
i02_2009	c2190	yeaI	hypothetical protein
i02_2010	c2191	yeaJ	hypothetical protein
i02_2011	c2192	yeaK	hypothetical protein
i02_2012	c2193	/	hypothetical protein
i02_2013	c2194	yeaL	hypothetical protein
i02_2014	c2195	yeaM	putative transcriptional regulator YeaM
i02_2015	c2196	yeaN	putative transport protein YeaN
i02_2016	c2197	yeaO	hypothetical protein
i02_2017	c2198	yoaF	hypothetical protein
i02_2018	c2199	yeaP	hypothetical protein
i02_2019		/	hypothetical protein
i02_2020	c2200	yeaQ	hypothetical protein
i02_2021	c2201	/	hypothetical protein
	c2203	/	hypothetical protein
i02_2022	c2202	yoaG	hypothetical protein
i02_2024		/	hypothetical protein
i02_2023	c2204	yeaR	hypothetical protein
i02_2025	c2205	yeaS	leucine export protein LeuE
	c2207	/	hypothetical protein
i02_2026	c2206	/	hypothetical protein
i02_2028	c2208	rnd	ribonuclease D
i02_2029	c2209	fadD	long-chain-fatty-acid--CoA ligase
i02_2030	c2210	yeaY	lipoprotein yeaY precursor
i02_2031	c2211	yeaZ	hypothetical protein
i02_2032	c2212	/	ATP-dependent helicase yoaA
i02_2033	c2213	yoaB	hypothetical protein
i02_2034		/	hypothetical protein
i02_2035	c2214	yoaC	hypothetical protein
i02_2036	c2215	/	hypothetical protein

Clone D	CFT 073	Gene	Product
	c2216	yoaH	hypothetical protein
i02_2037	c2217	pabB	para-aminobenzoate synthase component I
i02_2038	c2218	yeaB	hypothetical protein
i02_2039	c2219	sdaA	L-serine dehydratase 1
	c2220	/	hypothetical protein
i02_2040	c2221	yoaD	hypothetical protein
i02_2041	c2222	yoaE	hypothetical protein
i02_2042	c2223	manX	PTS system, mannose-specific IIAB component
i02_2043	c2224	manY	PTS system, mannose-specific IIC component
	c2225	/	hypothetical protein
i02_2044	c2226	manZ	mannose-specific PTS system protein IID
i02_2045	c2227	yobD	hypothetical protein
i02_2046	c2228	yebN	hypothetical protein
i02_2047	c2229	rrmA	23S rRNA methyltransferase A
	c2230	/	hypothetical protein
i02_2048	c2231	cspC	cold shock-like protein CspC
i02_2050		/	hypothetical protein
i02_2049	c2232	yobF	hypothetical protein
i02_2051	c2233	yebO	hypothetical protein
i02_2052	c2234	yobG	hypothetical protein
i02_2053	c2235	/	hypothetical protein
i02_2054	c2236	/	transcriptional regulator kdgR
i02_2055	c2237	yebQ	putative transport protein YebQ
i02_2056	c2238	htpX	heat shock protein HtpX
i02_2057	c2239	prc	carboxy-terminal protease
i02_2058	c2240	proQ	putative solute/DNA competence effector
i02_2059	c2241	/	hypothetical protein
i02_2060	c2242	yebS	hypothetical protein
i02_2061	c2243	yebT	hypothetical protein
i02_2062	c2244	yebU	rRNA (cytosine-C(5)-)-methyltransferase RsmF
i02_2063	c2245	yebV	hypothetical protein
i02_2064	c2246	yebW	hypothetical protein
i02_2065	c2247	pphA	serine/threonine protein phosphatase 1
i02_2066	c2248	/	hypothetical protein
i02_2067	c2249	yebY	hypothetical protein
i02_2068	c2250	yebZ	hypothetical protein
i02_2069	c2251	/	hypothetical protein
i02_2070	c2252	holE	DNA polymerase III subunit theta
i02_2071	c2253	yobB	hypothetical protein
i02_2072	c2254	/	exodeoxyribonuclease X
i02_2073	c2256	ptrB	protease II
i02_2074	c2257	/	hypothetical protein
i02_2075	c2258	yebE	hypothetical protein
i02_2076	c2259	yebF	hypothetical protein
i02_2077	c2260	yebG	DNA damage-inducible protein YebG
i02_2078		purK	phosphoribosylaminoimidazole carboxylase ATPase
i02_2079	c2263	eda	keto-hydroxyglutarate-aldolase/ 2-keto-4-hydroxyglutarate aldolase decarboxylase
i02_2080	c2264	edd	phosphogluconate dehydratase
i02_2081	c2265	zwf	glucose-6-phosphate 1-dehydrogenase
i02_2082	c2266	/	hypothetical protein

Clone D	CFT 073	Gene	Product
i02_2083	c2267	yebK	DNA-binding transcriptional regulator HexR
i02_2084	c2268	pykA	pyruvate kinase
i02_2085	c2269	msbB	lipid A biosynthesis (KDO)2-(lauroyl)-lipid IVA
i02_2086	c2270	yebA	hypothetical protein
i02_2087	c2271	znuA	high-affinity zinc transporter periplasmic
i02_2088	c2272	znuC	high-affinity zinc transporter ATPase
i02_2089	c2273	znuB	high-affinity zinc transporter membrane
i02_2090	c2274	ruvB	Holliday junction DNA helicase RuvB
i02_2091	c2275	ruvA	Holliday junction DNA helicase RuvA
i02_2092	c2276	yebB	hypothetical protein
i02_2093	c2277	ruvC	Holliday junction resolvase
i02_2094	c2278	yebC	hypothetical protein
i02_2095	c2279	ntpA	dATP pyrophosphohydrolase
i02_2096	c2280	aspS	aspartyl-tRNA synthetase
i02_2097	c2281	yecD	hypothetical protein
i02_2098	c2282	yecE	hypothetical protein
i02_2099	c2283	yecN	hypothetical protein
i02_2100	c2284	yecO	hypothetical protein
i02_2101	c2285	yecP	hypothetical protein
i02_2102	c2286	bisZ	trimethylamine-N-oxide reductase 2 precursor
i02_2104	c2287	yecK	cytochrome c-type protein torY
i02_2105	c2288	/	hypothetical protein
i02_2106	c2289	/	copper homeostasis protein CutC
i02_2107	c2290	yecM	hypothetical protein
i02_2108	c2291	argS	arginyl-tRNA synthetase
i02_2109	c2292	flhE	flagellar protein flhE precursor
i02_2110	c2293	flhA	flagellar biosynthesis protein FlhA
i02_2111	c2294	flhB	flagellar biosynthesis protein FlhB
i02_2112	c2295	/	hypothetical protein
i02_2113	c2296	cheZ	chemotaxis regulator CheZ
i02_2114	c2297	cheY	chemotaxis regulatory protein CheY
i02_2115	c2298	cheB	chemotaxis-specific methylesterase
i02_2116	c2299	cheR	chemotaxis methyltransferase CheR
i02_2117		/	methyl-accepting protein IV
i02_2118	c2300	/	hypothetical protein
i02_2119	c2301	tar	methyl-accepting chemotaxis protein II
i02_2120	c2302	cheW	purine-binding chemotaxis protein
i02_2121	c2303	cheA	chemotaxis protein CheA
i02_2122	c2304	motB	flagellar motor protein MotB
i02_2123	c2305	motA	flagellar motor protein MotA
i02_2124	c2306	flhC	transcriptional activator FlhC
i02_2125	c2308	flhD	transcriptional activator FlhD
i02_2126	c2309	yecG	universal stress protein UspC
i02_2127	c2310	otsA	trehalose-6-phosphate synthase
i02_2128	c2311	otsB	trehalose-6-phosphate phosphatase
i02_2129	c2312	araH	L-arabinose transporter permease protein
i02_2130	c2313	araG	L-arabinose transporter ATP-binding protein
i02_2131	c2314	araF	L-arabinose-binding periplasmic protein
i02_2132	c2315	yecI	Ferritin-like protein 2
i02_2133	c2316	/	hypothetical protein
i02_2134	c2317	/	hypothetical protein
	c2318	/	hypothetical protein

Clone D	CFT 073	Gene	Product
i02_2135	c2319	yecR	hypothetical protein
i02_2136	c2320	/	hypothetical protein
i02_2137	c2321	ftn	ferritin
i02_2138	c2322	yecH	hypothetical protein
i02_2139	c2323	tyrP	tyrosine transporter
i02_2140	c2324	yecA	hypothetical protein
i02_2141	c2325	pgsA	phosphatidylglycerophosphate synthetase
i02_2142	c2326	uvrC	excinuclease ABC subunit C
i02_2143	c2327	uvrY	response regulator
i02_2144	c2328	/	hypothetical protein
i02_2145	c2329	yecF	hypothetical protein
i02_2146	c2330	sdiA	DNA-binding transcriptional activator SdiA
i02_2147	c2331	yecC	putative amino-acid ABC transporter ATP-binding component
i02_2148	c2332	yecS	amino-acid ABC transporter permease protein
-	c2334	/	transposase
i02_2149	c2333	yedO	D-cysteine desulfhydrase
i02_2150	c2335	fliY	cystine transporter subunit
i02_2151	c2336	fliZ	flagella biosynthesis protein FliZ
i02_2152	c2337	fliA	flagellar biosynthesis sigma factor
i02_2153		/	hypothetical protein
i02_2154	c2338	fliC	flagellin
i02_2155	c2339	fliD	flagellar capping protein
i02_2156	c2340	fliS	flagellar protein FliS
i02_2157	c2341	fliT	flagellar biosynthesis protein FliT
i02_2158	c2342	amyA	cytoplasmic alpha-amylase
i02_2159	c2343	yedD	hypothetical protein
i02_2160	c2344	yedE	putative inner membrane protein
i02_2161	c2345	yedF	hypothetical protein
i02_2162	c2346	yedK	hypothetical protein
	c2347	/	hypothetical protein
i02_2163	c2348	/	Outer membrane porin protein nmpC precursor
i02_2164	c2349	ybcM	putative transcriptional regulator YbcM
	c2351	/	hypothetical protein
i02_2165	c2350	ybcL	putative kinase inhibitor
i02_2167	c2352	emrE	multidrug efflux protein
i02_2168	c2353	fliE	flagellar hook-basal body protein FliE
i02_2169	c2354	fliF	flagellar MS-ring protein
i02_2170	c2355	fliG	flagellar motor switch protein G
i02_2172	c2357	fliH	flagellar assembly protein H
i02_2171	c2356	/	hypothetical protein
i02_2173	c2358	fliI	flagellum-specific ATP synthase
i02_2174	c2359	fliJ	flagellar biosynthesis chaperone
i02_2175	c2360	fliK	flagellar hook-length control protein
i02_2176	c2361	fliL	flagellar basal body-associated protein FliL
i02_2177	c2362	fliM	flagellar motor switch protein FliM
i02_2178	c2363	fliN	flagellar motor switch protein FliN
i02_2179	c2364	fliO	flagellar biosynthesis protein FliO
i02_2180	c2365	fliP	flagellar biosynthesis protein FliP
i02_2181	c2366	fliQ	flagellar biosynthesis protein FliQ
i02_2182	c2367	fliR	flagellar biosynthesis protein FliR

Clone D	CFT 073	Gene	Product
i02_2183	c2369	rcsA	colanic acid capsular biosynthesis activation protein
	c2370	dsrB	hypothetical protein
i02_2184		/	hypothetical protein
	c2371	/	hypothetical protein
i02_2185	c2372	yodD	hypothetical protein
i02_2186	c2373	yedP	mannosyl-3-phosphoglycerate phosphatase
i02_2187	c2374	yedQ	hypothetical protein
i02_2188	c2375	/	hypothetical protein
i02_2189	c2376	yodC	hypothetical protein
i02_2190	c2377	yedI	hypothetical protein
i02_2191	c2378	yedA	hypothetical protein
i02_2192	c2379	vsr	very short patch repair protein
i02_2193	c2380	dcm	DNA cytosine methylase
i02_2194	c2381	yedJ	hypothetical protein
i02_2195	c2382	yedR	hypothetical protein
i02_2196	c2383	/	Outer membrane protein N precursor
i02_2197		/	conserved hypothetical protein
i02_2198	c2385	yedU	chaperone protein HchA
i02_2199	c2386	yedV	putative sensor-like histidine kinase yedV
i02_2200	c2387	yedW	transcriptional regulatory protein YedW
i02_2201	c2388	/	transthyretin-like protein precursor
i02_2202	c2389	yedY	putative sulfite oxidase subunit YedY
i02_2203	c2390	yedZ	putative sulfite oxidase subunit YedZ
i02_2204	c2391	yodA	hypothetical protein
i02_2205		/	hypothetical protein
i02_2206	c2392	/	P4 family integrase
i02_2207	c2393	/	hypothetical protein
i02_2208	c2394	/	PilV-like protein
i02_2209	c2395	/	putative type IV pilin protein precursor
i02_2210	c2396	/	hypothetical protein
i02_2211	c2397	/	hypothetical protein
i02_2212	c2398	/	hypothetical protein
i02_2213	c2399	/	hypothetical protein
i02_2214	c2400	/	hypothetical protein
i02_2215	c2401	/	hypothetical protein
i02_2216	c2402	/	hypothetical protein
i02_2217	c2403	/	hypothetical protein
i02_2218	c2404	/	hypothetical protein
i02_2219		/	hypothetical protein
i02_2220	c2405	/	hypothetical protein
i02_2221	c2406	/	hypothetical protein
i02_2222	c2407	/	hypothetical protein
i02_2223	c2408	/	hypothetical protein
i02_2225		/	hypothetical protein
i02_2224	c2409	/	hypothetical protein
i02_2226	c2410	/	hypothetical protein
i02_2227	c2411	/	DNA-binding protein H-NS
i02_2228	c2412	/	hypothetical protein
i02_2229	c2413	/	hypothetical protein
i02_2230	c2414	/	hypothetical protein
i02_2231	c2415	/	hypothetical protein

Clone D	CFT 073	Gene	Product
i02_2232		/	putative regulatory protein
i02_2233	c2416	/	hypothetical protein
i02_2234	c2417	yeeI	hypothetical protein
i02_2235	c2418	/	prophage P4 integrase
i02_2236	c2419	/	salicylate synthase Irp9
i02_2237	c2420	/	putative cytoplasmic transmembrane protein
i02_2238	c2421	/	ABC transporter
i02_2239	c2422	/	putative inner membrane ABC-transporter
i02_2240	c2423	/	putative AraC type regulator
i02_2241	c2424	/	putative peptide synthetase
-	c2425	/	similar Transposase for insertion sequence element IS200
i02_2241	c2426	/	putative peptide synthetase
i02_2242	c2427	/	putative peptide/polyketide synthetase protein
i02_2242	c2428	/	hypothetical protein
i02_2242	c2429	/	hypothetical protein
i02_2244	c2430	/	hypothetical protein
	c2431	/	hypothetical protein
i02_2245	c2432	/	putative thioesterase
i02_2246	c2433	/	2,3-dihydroxybenzoate-AMP ligase
i02_2246	c2434	/	putative salicyl-AMP ligase
	c2435	/	hypothetical protein
i02_2247	c2436	fyuA	putative pesticin receptor precursor
i02_2248	c2437	/	hypothetical protein
i02_2249	c2438	/	hypothetical protein
i02_2250	c2439	/	hypothetical protein
i02_2250a	c2440	/	hypothetical protein
i02_2251	c2441	/	hypothetical protein
i02_2251	c2442	/	hypothetical protein
i02_2252	c2443	shiA	shikimate transporter
i02_2253	c2444	amn	AMP nucleosidase
i02_2254	c2445	yeeN	hypothetical protein
i02_2255		/	hypothetical protein
i02_2256	c2446	nac	nitrogen assimilation transcriptional regulator
i02_2257	c2447	cbl	transcriptional regulator Cbl
i02_2258	c2448	yeeO	hypothetical protein
i02_2259	c2449	/	prophage P4 integrase
i02_2260	c2450	/	hypothetical protein
i02_2261	c2451	/	putative thioesterase
i02_2262	c2452	/	hypothetical protein
i02_2263	c2453	/	putative polyketide synthase
i02_2264	c2454	/	hypothetical protein
i02_2264	c2455	/	putative peptide synthetase
i02_2266	c2456	/	hypothetical protein
i02_2267	c2457	/	putative amidase
i02_2268	c2458	/	putative peptide synthetase
i02_2269	c2459	/	putative peptide synthetase
i02_2270	c2460	/	putative polyketide synthase
	c2462	/	hypothetical protein
i02_2271	c2461	/	hypothetical protein
i02_2273	c2463	/	putative transacylase
i02_2274	c2464	/	putative acyl-coa dehydrogenase

Clone D	CFT 073	Gene	Product
	c2465	/	hypothetical protein
i02_2275	c2466	/	hypothetical protein
i02_2276	c2467	/	putative 3-hydroxyacyl-CoA dehydrogenase
i02_2277	c2468	/	putative polyketide synthase
i02_2279	c2469	/	putative polyketide synthase
i02_2279	c2470	/	putative peptide/polyketide synthase
i02_2280	c2471	/	hypothetical protein
i02_2281	c2472	/	transposase
i02_2283	c2473	/	transposase
i02_2282		/	hypothetical protein
i02_2284	c2474	/	transposase
i02_2285	c2475	/	hypothetical protein
i02_2286	c2476	erfK	hypothetical protein
i02_2287	c2477	cobT	nicotinate-nucleotide--dimethylbenzimidazole
i02_2288	c2478	cobS	cobalamin synthase
i02_2289	c2479	cobU	adenosylcobinamide
i02_2290	c2480	/	hypothetical protein
i02_2291	c2481	/	hypothetical protein
i02_2292	c2482	/	putative outer membrane receptor for iron
i02_2293	c2483	/	hypothetical protein
i02_2294	c2484	/	hypothetical protein
i02_2295	c2485	/	hypothetical protein
i02_2296	c2486	/	hypothetical protein
i02_2297	c2489	/	putative transferase
i02_2298	c2490	/	hypothetical protein
i02_2299		/	hypothetical protein
i02_2300	c2492	/	putative carbohydrate kinase
i02_2301	c2493	/	hypothetical protein
i02_2302	c2494	/	hypothetical protein
i02_2303	c2495	/	putative phosphotriesterase-related protein
	c2496	/	hypothetical protein
i02_2304	c2497	/	transposase
i02_2305	c2498	/	hypothetical protein
i02_2306	c2499	/	hypothetical protein
i02_2307	c2500	/	hypothetical protein
i02_2309	c2502	/	hypothetical protein
i02_2308	c2501	/	hypothetical protein
i02_2310	c2503	/	transposase
	c2504	/	hypothetical protein
i02_2312	c2505	/	hypothetical protein
i02_2313	c2506	/	hypothetical protein
-	c2508	/	hypothetical protein
-	c2509	/	insertion sequence ATP-binding protein
i02_2313	c2510	/	hypothetical protein
-	c2508	/	hypothetical protein
-	c2509	/	insertion sequence ATP-binding protein
i02_2314	c2511	/	transposase/IS protein
i02_2315	c2512	/	transposase
i02_2316		/	putative transposase subunit
i02_2317	c2513	/	hypothetical protein
i02_2318	c2514	/	hypothetical protein
i02_2319	c2515	/	putative ABC transporter

Clone D	CFT 073	Gene	Product
i02_2320	c2516	/	ABC transporter
i02_2321	c2517	/	periplasmic binding protein
i02_2322	c2518	/	TonB dependent receptor
i02_2323	c2520	/	hypothetical protein
	c2521	/	hypothetical protein
i02_2325	c2522	/	hypothetical protein
i02_2326	c2523	/	hypothetical protein
i02_2327	c2524	/	hypothetical protein
i02_2328	c2525	/	hypothetical protein
i02_2329	c2526	/	hypothetical protein
	c2527	/	hypothetical protein
	c2529	/	putative radC-like protein yeeS
i02_2330	c2528	/	hypothetical protein
i02_2331	c2530	/	hypothetical protein
i02_2332	c2531	/	hypothetical protein
i02_2333	c2532	/	hypothetical protein
i02_2334	c2533	yeeW	hypothetical protein
i02_2335		yoeF	hypothetical protein
i02_2336	c2534	yeeX	hypothetical protein
	c2535	/	hypothetical protein
i02_2337	c2536	yeeA	hypothetical protein
i02_2338	c2537	sbmC	DNA gyrase inhibitor
i02_2339	c2538	dacD	D-alanyl-D-alanine carboxypeptidase
i02_2340	c2539	sbcB	exonuclease I
i02_2341	c2540	yeeF	putative transport protein YeeF
	c2541	/	hypothetical protein
i02_2342	c2542	yeeY	putative transcriptional regulator YeeY
i02_2343	c2543	/	hypothetical protein
i02_2344	c2544	/	hypothetical protein
i02_2345	c2545	yefM	antitoxin YefM
	c5496	hisL	his operon leader peptide
i02_2346	c2546	hisG	ATP phosphoribosyltransferase
i02_2347	c2547	hisD	histidinol dehydrogenase
i02_2348	c2548	hisC	histidinol-phosphate aminotransferase
i02_2349	c2549	hisB	imidazole glycerol-phosphate
i02_2350	c2550	hisH	imidazole glycerol phosphate synthase subunit
i02_2351	c2551	hisA	N-(5'-phospho-L-ribosyl-formimino)-5-amino-1-(5'-phosphoribosyl)-4-imidazolecarboxamide isomerase
i02_2352	c2552	hisF	imidazole glycerol phosphate synthase subunit
i02_2353	c2553	hisI	phosphoribosyl-AMP cyclohydrolase / phosphoribosyl-ATP pyrophosphatase
i02_2354	c2554	wzzB	chain length determinant protein
i02_2355	c2555	ugd	UDP-glucose 6-dehydrogenase
i02_2356	c2556	gnd	6-phosphogluconate dehydrogenase
i02_2357	c2557	manB	phosphomannomutase
i02_2358	c2558	/	mannose-1-phosphate guanylyltransferase
i02_2359	c2559	/	hypothetical protein
i02_2360	c2560	/	UDP-glucose 4-epimerase
i02_2361	c2561	/	hypothetical protein
i02_2362	c2562	/	glycosyl transferase
i02_2363	c2563	/	glycosyl transferase

Clone D	CFT 073	Gene	Product
i02_2364	c2564	/	hypothetical protein
i02_2365	c2566	/	hypothetical protein
i02_2366	c2567	galF	UTP--glucose-1-phosphate uridylyltransferase
i02_2367	c2568	wcaM	putative colanic acid biosynthesis protein
i02_2368	c2569	wcaL	putative colanic acid biosynthesis glycosyltransferase
i02_2369	c2570	wcaK	putative pyruvyl transferase
i02_2370	c2571	wzxC	colanic acid exporter
i02_2371	c2572	wcaJ	putative UDP-glucose lipid carrier transferase
i02_2373	c2574	/	hypothetical protein
i02_2372	c2573	cpsG	phosphomannomutase
i02_2374	c2575	cpsB	mannose-1-phosphate guanylyltransferase
i02_2375	c2576	wcaI	putative glycosyl transferase
i02_2376	c2577	wcaH	GDP-mannose mannosyl hydrolase
i02_2377	c2578	wcaG	GDP-4-keto-6-L-galactose reductase
i02_2378	c2579	gmd	GDP-mannose 4,6-dehydratase
i02_2379	c2580	wcaF	putative colanic acid biosynthesis
i02_2380	c2581	wcaE	putative glycosyl transferase
i02_2381	c2582	wcaD	putative colanic acid biosynthesis protein
i02_2382	c2583	wcaC	putative glycosyl transferase
i02_2383	c2584	wcaB	putative colanic acid biosynthesis
i02_2384	c2585	wcaA	putative glycosyl transferase
i02_2385	c2586	/	tyrosine kinase
i02_2386	c2587	/	hypothetical protein
i02_2387	c2588	/	hypothetical protein
i02_2387	c2589	/	hypothetical protein
i02_2388	c2590	yegH	hypothetical protein
i02_2389	c2591	asmA	putative assembly protein
i02_2390	c2592	dcd	deoxycytidine triphosphate deaminase
i02_2391	c2593	udk	uridine kinase
i02_2392	c2594	yegE	putative sensor protein
i02_2393	c2595	alkA	3-methyl-adenine DNA glycosylase II
i02_2394	c2596	yegD	putative chaperone
i02_2396	c2597	yegI	hypothetical protein
i02_2397	c2598	yegK	hypothetical protein
i02_2398	c2599	yegL	hypothetical protein
i02_2399	c2600	yegM	multidrug efflux system subunit MdtA
i02_2400	c2601	yegN	multidrug efflux system subunit MdtB
i02_2401	c2602	yegO	multidrug efflux system subunit MdtC
i02_2402	c2603	yegB	multidrug efflux system protein MdtE
i02_2403	c2604	baeS	signal transduction histidine-protein kinase
i02_2404	c2605	baeR	DNA-binding transcriptional regulator BaeR
i02_2405	c2606	yegP	hypothetical protein
i02_2406	c2607	/	hypothetical protein
i02_2407	c2608	/	hypothetical protein
i02_2409	c2609	/	hypothetical protein
i02_2410	c2610	/	hypothetical protein
i02_2411	c2611	yegQ	putative protease yegQ
i02_2412	c2612	/	hypothetical protein
i02_2413		/	hypothetical protein
i02_2414	c2613	yegR	hypothetical protein
i02_2415	c2614	yegS	lipid kinase

Clone D	CFT 073	Gene	Product
i02_2416	c2615	/	hypothetical protein
i02_2417	c2616	gatD	galactitol-1-phosphate dehydrogenase
i02_2418	c2617	gatC	PTS system, galactitol-specific IIC component
i02_2419	c2618	gatB	galactitol-specific PTS system component IIB
i02_2420	c2619	gatA	galactitol-specific PTS system component IIA
i02_2421	c2620	gatZ	putative tagatose 6-phosphate kinase gatZ
i02_2422	c2621	gatY	tagatose-bisphosphate aldolase
-	c2622	/	transposase
i02_2424	c2624	/	hypothetical protein
i02_2423	c2623	/	fructose-bisphosphate aldolase
i02_2425	c2625	yegT	putative nucleoside transporter yegT
i02_2426	c2626	yegU	hypothetical protein
i02_2427	c2627	yegV	putative sugar kinase yegV
i02_2428	c2628	yegW	putative transcriptional regulator YegW
i02_2429	c2629	yegX	hypothetical protein
i02_2430	c2630	thiD	phosphomethylpyrimidine kinase
i02_2431	c2631	thiM	hydroxyethylthiazole kinase
i02_2432	c2632	yohL	hypothetical protein
i02_2433	c2633	yohM	nickel/cobalt efflux protein RcnA
i02_2434	c2634	yohN	hypothetical protein
i02_2435	c2635	yehA	hypothetical protein
i02_2436	c2636	yehB	outer membrane usher protein yehB precursor
i02_2437	c2637	yehC	fimbrial chaperone yehC precursor
i02_2438	c2638	yehD	hypothetical protein
-	c2639	/	transposase
i02_2439	c2640	yehE	hypothetical protein
i02_2440	c2641	mrp	putative ATPase
i02_2441	c2642	metG	methionyl-tRNA synthetase
i02_2442	c2643	/	hypothetical protein
i02_2443	c2644	yehI	hypothetical protein
i02_2444	c2645	/	hypothetical protein
i02_2445	c2646	/	hypothetical protein
i02_2446	c2647	/	yehK protein
	c2648	/	hypothetical protein
i02_2447	c2649	yehL	hypothetical protein
i02_2448	c2650	yehM	hypothetical protein
i02_2449	c2651	yehP	hypothetical protein
i02_2450	c2652	yehQ	hypothetical protein
i02_2451	c2653	yehR	lipoprotein yehR precursor
i02_2452	c2654	yehS	hypothetical protein
i02_2453	c2655	yehT	putative two-component response-regulator
i02_2454	c2656	yehU	hypothetical protein
i02_2455	c2657	yehV	MerR-like regulator A
i02_2456	c2658	yehW	ABC transporter permease
i02_2457	c2659	yehX	ABC transporter ATP-binding protein
i02_2458	c2660	yehY	ABC transporter permease
i02_2459	c2661	yehZ	hypothetical protein
i02_2462	c2662	/	hypothetical protein
i02_2463	c2663	bglX	periplasmic beta-glucosidase precursor
i02_2464	c2664	dld	D-lactate dehydrogenase
i02_2465	c2665	pbpG	D-alanyl-D-alanine endopeptidase
i02_2466	c2666	/	hypothetical protein

Clone D	CFT 073	Gene	Product
i02_2467	c2667	yohC	hypothetical protein
i02_2468	c2668	yohD	hypothetical protein
i02_2469	c2669	/	hypothetical protein
i02_2469a	c2670	/	hypothetical protein
i02_2470	c2671	yohG	multidrug resistance outer membrane protein
i02_2471	c2672	yohI	tRNA-dihydrouridine synthase C
i02_2472	c2673	yohJ	hypothetical protein
i02_2473	c2674	yohK	hypothetical protein
i02_2474	c2675	cdd	cytidine deaminase
	c2676	/	hypothetical protein
i02_2476	c2677	sanA	hypothetical protein
i02_2477	c2678	yeiS	hypothetical protein
i02_2478	c2679	yeiT	putative oxidoreductase
i02_2479	c2680	yeiA	dihydropyrimidine dehydrogenase
i02_2480	c2681	/	hypothetical protein
i02_2481	c2682	mglC	beta-methylgalactoside transporter
i02_2482	c2683	mglA	galactose/methyl galaxtoside transporter
i02_2483	c2684	mglB	D-galactose-binding periplasmic protein
i02_2485	c2686	/	hypothetical protein
i02_2484	c2685	galS	DNA-binding transcriptional regulator GalS
i02_2486	c2687	yeiB	hypothetical protein
i02_2487	c2688	folE	GTP cyclohydrolase I
i02_2488	c2689	yeiG	hypothetical protein
i02_2489	c2690	cirA	colicin I receptor
i02_2490	c2691	lysP	lysine transporter
i02_2491	c2692	yeiE	putative DNA-binding transcriptional regulator
i02_2492	c2693	yeiH	hypothetical protein
i02_2493	c2694	nfo	endonuclease IV
i02_2494	c2695	yeiI	hypothetical protein
i02_2495		/	hypothetical protein
i02_2497	c2696	rihB	ribonucleoside hydrolase 2
i02_2498	c2697	yeiL	DNA-binding transcriptional activator YeiL
i02_2499	c2698	yeiM	putative transport protein YeiM
i02_2500		yeiN	hypothetical protein
i02_2501	c2701	yeiC	hypothetical protein
i02_2502	c2702	fruA	fructose-specific PTS system IIBC component
i02_2503	c2703	fruK	1-phosphofructokinase
i02_2504	c2704	fruB	fructose PTS transporter
i02_2505	c2705	/	hypothetical protein
i02_2506	c2706	yeiO	sugar efflux transporter B
i02_2508	c2708	yeiP	elongation factor P
i02_2507	c2707	/	hypothetical protein
i02_2509	c2709	yeiQ	oxidoreductase yeiQ
i02_2510	c2710	yeiR	hypothetical protein
i02_2511	c2711	yeiU	hypothetical protein
i02_2512	c2712	spr	putative outer membrane lipoprotein
i02_2513	c2713	rtn	hypothetical protein
i02_2514	c2714	yejA	hypothetical protein
i02_2515	c2715	yejB	ABC transporter permease
i02_2516	c2716	yejE	ABC transporter permease
i02_2517	c2717	yejF	ABC transporter ATP-binding protein
i02_2518	c2718	yejG	hypothetical protein

Clone D	CFT 073	Gene	Product
i02_2519	c2719	bcr	bicyclomycin/multidrug efflux system
i02_2520	c2720	rsuA	16S rRNA pseudouridylate synthase A
i02_2521	c2721	yejH	hypothetical protein
	c2723	/	hypothetical protein
i02_2522	c2722	rplY	50S ribosomal protein L25
i02_2523	c2724	yejK	nucleoid-associated protein NdpA
i02_2524		/	hypothetical protein
i02_2526	c2725	yejL	hypothetical protein
i02_2527	c2726	yejM	hypothetical protein
	c2728	/	hypothetical protein
i02_2528	c2727	/	hypothetical protein
i02_2529	c2729	/	hypothetical protein
i02_2530		/	hypothetical protein
i02_2531	c2730	narP	transcriptional regulator NarP
i02_2532	c2731	ccmH	cytochrome c-type biogenesis protein ccmH
i02_2533	c2732	dsbE	Thiol:disulfide interchange protein dsbE
i02_2534	c2733	ccmF	cytochrome c-type biogenesis protein ccmF
i02_2535	c2734	ccmE	cytochrome c-type biogenesis protein CcmE
i02_2536	c2735	ccmD	Heme exporter protein D
i02_2537	c2736	ccmC	Heme exporter protein C
i02_2538	c2737	ccmB	Heme exporter protein B
i02_2539	c2738	ccmA	cytochrome c biogenesis protein CcmA
	c2740	/	hypothetical protein
i02_2540	c2739	napC	cytochrome c-type protein NapC
	c2742	/	hypothetical protein
i02_2541	c2741	napB	citrate reductase cytochrome c-type subunit
i02_2542	c2743	napH	quinol dehydrogenase membrane component
i02_2543	c2744	napG	quinol dehydrogenase periplasmic component
i02_2544	c2745	napA	nitrate reductase catalytic subunit
i02_2545	c2746	napD	assembly protein for periplasmic nitrate
i02_2546	c2747	napF	ferredoxin-type protein
i02_2547	c2748	/	hypothetical protein
i02_2548	c2749	/	hypothetical protein
i02_2549	c2750	eco	ecotin
i02_2550		eco	ecotin precursor
i02_2551	c2751	yojH	malate:quinone oxidoreductase
i02_2552	c2752	yojI	multidrug transporter membrane
i02_2553	c2753	alkB	alkylated DNA repair protein AlkB
	c2755	/	hypothetical protein
i02_2554	c2754	ada	ADA regulatory protein
i02_2555	c2756	yojL	thiamine biosynthesis lipoprotein ApbE
	c2757	/	hypothetical protein
i02_2556	c2758	ompC	outer membrane porin protein C
i02_2557	c2759	yojN	phosphotransfer intermediate protein
i02_2558	c2760	rcsB	transcriptional regulator RcsB
i02_2559	c2761	rcsC	hybrid sensory kinase in two-component
i02_2560	c2762	atoS	sensory histidine kinase AtoS
i02_2561	c2763	atoC	acetoacetate metabolism regulatory protein AtoC
i02_2562	c2764	atoD	acetyl-CoA:acetoacetyl-CoA transferase subunit
i02_2563	c2765	atoA	acetate CoA-transferase beta subunit
i02_2564	c2766	atoE	Short-chain fatty acids transporter
i02_2565	c2767	atoB	Acetyl-CoA acetyltransferase

Clone D	CFT 073	Gene	Product
i02_2566	c2768	yfaP	hypothetical protein
i02_2567	c2769	yfaQ	hypothetical protein
i02_2568	c2770	/	hypothetical protein
i02_2569	c2771	yfaT	hypothetical protein
i02_2570	c2772	yfaA	hypothetical protein
i02_2571	c2773	gyrA	DNA gyrase subunit A
i02_2572	c2774	ubiG	3-demethylubiquinone-9 3-methyltransferase
i02_2573	c2775	yfaL	adhesin
i02_2574		/	conserved domain protein
i02_2575	c2776	nrdA	ribonucleotide-diphosphate reductase subunit
i02_2576	c2777	nrdB	ribonucleotide-diphosphate reductase subunit
i02_2577	c2778	yfaE	2Fe-2S ferredoxin YfaE
i02_2578	c2779	inaA	hypothetical protein
i02_2579	c2780	glpQ	glycerophosphodiester phosphodiesterase
i02_2580	c2781	glpT	sn-glycerol-3-phosphate transporter
i02_2581	c2782	glpA	sn-glycerol-3-phosphate dehydrogenase subunit A
i02_2582	c2783	glpB	anaerobic glycerol-3-phosphate dehydrogenase
i02_2583	c2784	glpC	sn-glycerol-3-phosphate dehydrogenase subunit C
i02_2584	c2785	/	hypothetical protein
i02_2585	c2786	yfaD	hypothetical protein
i02_2586	c2787	yfaU	putative aldolase
i02_2587	c2788	yfaV	putative transport protein YfaV
i02_2588	c2789	yfaW	hypothetical protein
i02_2589	c2790	yfaX	putative transcriptional regulator YfaX
i02_2590	c2791	/	competence damage-inducible protein A
i02_2591	c2792	yfaZ	hypothetical protein
i02_2592	c2793	yfaO	putative Nudix hydrolase yfaO
i02_2593	c2794	ais	Ais protein
i02_2594	c2795	yfbE	UDP-L-Ara4O C-4 transaminase
i02_2595	c2796	/	undecaprenyl phosphate
i02_2596	c2797	yfbG	bifunctional UDP-glucuronic acid
i02_2597	c2798	yfbH	hypothetical protein
i02_2598	c2799	arnT	4-amino-4-deoxy-L-arabinose transferase
i02_2599	c2800	/	hypothetical protein
i02_2600	c2801	yfbJ	hypothetical protein
i02_2601	c2802	pmrD	polymyxin B resistance protein pmrD
i02_2602	c2803	menE	O-succinylbenzoic acid--CoA ligase
i02_2603	c2804	menC	O-succinylbenzoate synthase
i02_2605	c2806	/	hypothetical protein
i02_2604	c2805	menB	naphthoate synthase
i02_2606	c2807	yfbB	acyl-CoA thioester hydrolase YfbB
i02_2607	c2808	menD	2-hydroxyglutarate synthase
i02_2608	c2809	menF	menaquinone-specific isochorismate synthase
i02_2609	c2810	elaB	hypothetical protein
i02_2610	c2811	elaA	hypothetical protein
i02_2611	c2812	elaC	ribonuclease Z
i02_2612	c2813	yfbK	hypothetical protein
	c2814	/	hypothetical protein
i02_2613	c2815	yfbL	hypothetical protein
i02_2614	c2816	yfbM	hypothetical protein

Clone D	CFT 073	Gene	Product
i02_2615	c2817	nuoN	NADH dehydrogenase subunit N
i02_2616	c2818	nuoM	NADH dehydrogenase subunit M
i02_2617	c2819	nuoL	NADH dehydrogenase subunit L
i02_2618	c2820	nuoK	NADH dehydrogenase subunit K
i02_2619	c2821	nuoJ	NADH dehydrogenase subunit J
i02_2620	c2822	nuoI	NADH dehydrogenase subunit I
i02_2621	c2823	nuoH	NADH dehydrogenase subunit H
i02_2622	c2824	nuoG	NADH dehydrogenase subunit G
i02_2623	c2825	nuoF	NADH dehydrogenase I subunit F
i02_2624	c2826	nuoE	NADH dehydrogenase subunit E
i02_2625	c2827	nuoC	bifunctional NADH:ubiquinone oxidoreductase
i02_2626	c2828	nuoB	NADH dehydrogenase subunit B
i02_2627	c2829	nuoA	NADH dehydrogenase subunit A
i02_2628		/	hypothetical protein
i02_2629	c2830	lrhA	transcriptional regulator lrhA
i02_2630	c2831	/	aminotransferase AlaT
i02_2631	c2832	yfbR	hypothetical protein
i02_2632	c2833	yfbS	hypothetical protein
i02_2633	c2834	yfbT	putative phosphatase
i02_2634	c2835	/	hypothetical protein
i02_2635	c2836	yfbV	hypothetical protein
i02_2636	c2837	/	hypothetical protein
i02_2637	c2838	ackA	acetate kinase
	c2839	/	hypothetical protein
i02_2638	c2840	pta	phosphate acetyltransferase
i02_2639	c2841	yfcC	hypothetical protein
i02_2640	c2842	yfcD	hypothetical protein
i02_2641	c2843	yfcE	phosphodiesterase
i02_2642	c2844	yfcF	GST-like protein yfcF
i02_2643	c2845	yfcG	glutathione S-transferase
i02_2644	c2846	folX	D-erythro-7,8-dihydroneopterin triphosphate
i02_2645	c2847	yfcH	hypothetical protein
i02_2646	c2848	hisP	histidine/lysine/arginine/ornithine transporter
i02_2647	c2849	hisM	histidine transport system permease protein
i02_2648	c2850	hisQ	histidine transport system permease protein
i02_2649	c2851	hisJ	histidine-binding periplasmic protein precursor
i02_2650		hisJ	histidine-binding periplasmic protein precursor
i02_2651	c2854	/	hypothetical protein
i02_2652	c2855	ubiX	3-octaprenyl-4-hydroxybenzoate carboxy-lyase
i02_2653	c2856	purF	amidophosphoribosyltransferase
i02_2654	c2857	cvpA	colicin V production protein
i02_2655	c2858	/	hypothetical protein
i02_2656	c2859	dedD	hypothetical protein
i02_2657	c2860	folC	bifunctional folylpolyglutamate synthase
i02_2658	c2861	accD	acetyl-CoA carboxylase subunit beta
i02_2659	c2862	dedA	hypothetical protein
i02_2660	c2863	truA	tRNA pseudouridine synthase A
i02_2662		/	hypothetical protein
i02_2661	c2864	usg	putative semialdehyde dehydrogenase
i02_2663	c2865	pdxB	erythronate-4-phosphate dehydrogenase
i02_2664	c2866	flk	flagella biosynthesis regulator
i02_2665		yfcJ	hypothetical protein

Clone D	CFT 073	Gene	Product
i02_2666	c2869	fabB	3-oxoacyl-(acyl carrier protein) synthase I
i02_2667	c2870	mnmC	5-methylaminomethyl-2-thiouridine
i02_2668	c2871	yfcL	hypothetical protein
i02_2669	c2872	yfcM	hypothetical protein
i02_2670	c2873	yfcA	hypothetical protein
i02_2671	c2874	mepA	penicillin-insensitive murein endopeptidase
i02_2672	c2875	aroC	chorismate synthase
i02_2674	c2877	yfcN	hypothetical protein
i02_2673	c2876	yfcB	N5-glutamine S-adenosyl-L-methionine-dependent
i02_2675	c2878	yfcO	hypothetical protein
i02_2676	c2879	yfcP	fimbrial-like protein yfcP precursor
i02_2677	c2880	yfcQ	fimbrial-like protein yfcQ precursor
i02_2678	c2881	yfcR	hypothetical protein
i02_2679	c2882	yfcS	fimbrial chaperone yfcS precursor
i02_2680	c2883	yfcU	hypothetical protein
i02_2681	c2884	yfcV	fimbrial-like protein yfcV precursor
i02_2682	c2885	/	phosphohistidine phosphatase
i02_2683	c2886	fadJ	multifunctional fatty acid oxidation complex
i02_2684	c2887	fadI	3-ketoacyl-CoA thiolase
i02_2685	c2888	yfcZ	hypothetical protein
i02_2686		/	hypothetical protein
i02_2687	c2889	fadL	long-chain fatty acid outer membrane
i02_2688	c2890	vacJ	VacJ lipoprotein precursor
i02_2689	c2891	/	hypothetical protein
i02_2690	c2892	yfdC	hypothetical protein
i02_2691	c2893	/	hypothetical protein
i02_2693	c2894	ydeU	hypothetical protein
i02_2693	c2895	/	yapH-like protein
i02_2694	c2896	/	hypothetical protein
i02_2695	c2897	/	Type 1 fimbriae regulatory protein fimB
i02_2696	c2898	/	Type 1 fimbriae regulatory protein fimB
i02_2697	c2899	dsdC	DNA-binding transcriptional regulator DsdC
i02_2698	c2900	dsdX	permease DsdX
i02_2699	c2901	dsdA	D-serine dehydratase
i02_2701	c2903	/	hypothetical protein
i02_2700	c2902	emrY	multidrug resistance protein Y
i02_2702	c2904	emrK	multidrug resistance protein K
i02_2703	c2905	evgA	DNA-binding transcriptional activator EvgA
i02_2704	c2906	evgS	hybrid sensory histidine kinase in two-component
i02_2705	c2907	yfdE	hypothetical protein
i02_2706	c2908	yfdV	putative transporter YfdV
i02_2707	c2909	/	putative oxalyl-CoA decarboxylase
i02_2708	c2910	yfdW	formyl-coenzyme A transferase
i02_2709	c2911	/	hypothetical protein
i02_2710	c2912	ypdI	lipoprotein ypdI precursor
	c2913	/	hypothetical protein
i02_2711	c2914	yfdY	hypothetical protein
i02_2712	c2915	ddg	lipid A biosynthesis protein
i02_2713	c2916	yfdZ	aminotransferase
i02_2715	c2918	/	hypothetical protein
i02_2716	c2919	ypdA	hypothetical protein

Clone D	CFT 073	Gene	Product
i02_2717	c2920	ypdB	hypothetical protein
i02_2718	c2921	ypdC	putative transcriptional regulator YpdC
i02_2719	c2922	/	putative phosphoenolpyruvate-protein
i02_2720	c2923	ypdE	exoaminopeptidase
i02_2721	c2924	/	aminopeptidase
i02_2722	c2925	/	putative PTS system IIC component ypdG
i02_2723	c2926	/	putative PTS system IIB component ypdH
i02_2724	c2927	glk	glucokinase
i02_2725	c2928	yfeO	hypothetical protein
i02_2726	c2929	ypeC	hypothetical protein
i02_2727	c2931	/	manganese transport protein MntH
i02_2728	c2932	nupC	nucleoside permease nupC
i02_2729	c2933	yfeA	hypothetical protein
i02_2730	c2934	yfeC	hypothetical protein
i02_2731	c2935	yfeD	hypothetical protein
i02_2732	c2936	gltX	glutamyl-tRNA synthetase
i02_2733		/	hypothetical protein
i02_2734	c2937	xapR	DNA-binding transcriptional activator XapR
i02_2735	c2938	/	hypothetical protein
i02_2736	c2939	xapB	xanthosine permease
i02_2737	c2940	xapA	purine nucleoside phosphorylase
i02_2738		/	hypothetical protein
i02_2739	c2941	yfeN	hypothetical protein
i02_2740	c2942	yfeR	putative transcriptional regulator YfeR
i02_2741	c2943	yfeH	hypothetical protein
i02_2742	c2944	/	hypothetical protein
i02_2743	c2945	ligA	NAD-dependent DNA ligase LigA
i02_2744	c2946	zipA	cell division protein ZipA
i02_2745	c2947	cysZ	putative sulfate transport protein CysZ
i02_2746	c2948	cysK	cysteine synthase A
i02_2747	c2949	/	hypothetical protein
i02_2748	c2950	ptsH	phosphohistidinoprotein-hexose
i02_2749	c2951	ptsI	phosphoenolpyruvate-protein phosphotransferase
i02_2750	c2952	crr	glucose-specific PTS system component
i02_2751	c2953	pdxK	pyridoxal kinase
i02_2752	c2954	yfeK	hypothetical protein
i02_2753	c2955	cysM	cysteine synthase B
i02_2754	c2956	cysA	sulfate/thiosulfate transporter subunit
i02_2755	c2957	cysW	sulfate/thiosulfate transporter permease
i02_2756	c2958	cysU	sulfate/thiosulfate transporter subunit
i02_2757	c2959	cysP	thiosulfate transporter subunit
i02_2758	c2960	ucpA	short chain dehydrogenase
i02_2759	c2961	murQ	N-acetylmuramic acid-6-phosphate etherase
i02_2760	c2962	murP	N-acetylmuramic acid phosphotransfer permease
i02_2761	c2963	/	hypothetical protein
i02_2762	c2964	/	hypothetical protein
	c2965	yfeX	hypothetical protein
i02_2763	c2966	yfeY	hypothetical protein
i02_2764	c2967	yfeZ	hypothetical protein
i02_2765	c2968	ypeA	putative acetyltransferase
i02_2766	c2969	amiA	N-acetylmuramoyl-l-alanine amidase I
i02_2767	c2970	hemF	coproporphyrinogen III oxidase

Clone D	CFT 073	Gene	Product
i02_2768	c2971	yfeG	transcriptional regulator EutR
i02_2769	c2972	/	ethanolamine utilization protein eutK precursor
i02_2770	c2973	/	ethanolamine utilization protein eutL
i02_2771	c2974	eutC	ethanolamine ammonia-lyase small subunit
i02_2772	c2975	eutB	ethanolamine ammonia-lyase heavy chain
i02_2773	c2976	eutA	reactivating factor for ethanolamine ammonia
i02_2774	c2977	eutH	ethanolamine utilization protein eutH
i02_2775	c2978	eutG	ethanolamine utilization protein eutG
i02_2776	c2979	eutJ	ethanolamine utilization protein eutJ
i02_2777	c2980	eutE	ethanolamine utilization protein eutE
i02_2778	c2981	cchB	ethanolamine utilization protein eutN
i02_2779	c2982	cchA	ethanolamine utilization protein eutM precursor
i02_2780	c2983	eutD	phosphotransacetylase
i02_2781	c2984	/	ethanolamine utilization cobalamin
i02_2782	c2985	/	ethanolamine utilization protein EutQ
i02_2783	c2986	/	ethanolamine utilization protein eutP
i02_2784	c2987	/	ethanolamine utilization protein eutS
i02_2785	c2988	/	malic enzyme
i02_2786	c2989	talA	transaldolase A
i02_2787	c2990	tktB	transketolase
	c2992	/	hypothetical protein
	c2993	/	hypothetical protein
i02_2788	c2991	ypfG	hypothetical protein
i02_2789	c2994	yffH	hypothetical protein
i02_2790	c2995	yffG	putative oxidoreductase Fe-S binding subunit
i02_2791	c2996	narQ	nitrate/nitrite sensor protein NarQ
i02_2792	c2997	acrD	aminoglycoside/multidrug efflux system
i02_2793		/	conserved hypothetical protein
i02_2794	c2998	yffB	hypothetical protein
i02_2795	c2999	dapE	succinyl-diaminopimelate desuccinylase
i02_2796	c3000	/	hypothetical protein
i02_2797	c3001	ypfH	esterase YpfH
i02_2798	c3002	ypfI	hypothetical protein
i02_2799	c3003	ypfJ	hypothetical protein
i02_2800	c3004	purC	phosphoribosylaminoimidazole-succinocarboxamide
i02_2801	c3005	nlpB	lipoprotein
i02_2802	c3006	dapA	dihydrodipicolinate synthase
i02_2803	c3007	gcvR	predicted transcriptional regulator
i02_2804	c3008	bcp	thioredoxin-dependent thiol peroxidase
i02_2805	c3009	perM	putative permease PerM
i02_2806	c3010	/	hypothetical protein
i02_2807	c3011	yfgC	hypothetical protein
i02_2808	c3012	/	hypothetical protein
i02_2809	c3013	yfgE	DNA replication initiation factor
i02_2810	c3014	uraA	uracil transporter
i02_2812	c3016	/	hypothetical protein
i02_2811	c3015	upp	uracil phosphoribosyltransferase
i02_2813	c3017	purM	phosphoribosylaminoimidazole synthetase
i02_2814	c3018	purN	phosphoribosylglycinamide formyltransferase
i02_2815	c3019	ppk	polyphosphate kinase
i02_2816	c3020	ppx	exopolyphosphatase

Clone D	CFT 073	Gene	Product
i02_2817	c3021	yfgF	hypothetical protein
	c3022	/	hypothetical protein
i02_2819	c3023	/	hypothetical protein
i02_2820	c3024	yfgH	lipoprotein yfgH precursor
i02_2821	c3025	yfgI	hypothetical protein
i02_2822	c3026	guaA	GMP synthase
i02_2823	c3027	guaB	inosine 5'-monophosphate dehydrogenase
i02_2824	c3028	xseA	exodeoxyribonuclease VII large subunit
i02_2825	c3029	/	RatA-like protein
i02_2826	c3030	/	SinI-like protein
i02_2827	c3031	/	SinH-like protein
i02_2828	c3032	yfgJ	hypothetical protein
i02_2829	c3033	engA	GTP-binding protein EngA
i02_2830	c3034	yfgL	outer membrane protein assembly complex subunit
i02_2831	c3035	yfgM	hypothetical protein
i02_2832	c3036	hisS	histidyl-tRNA synthetase
i02_2833	c3037	ispG	4-hydroxy-3-methylbut-2-en-1-yl diphosphate
i02_2834	c3038	yfgA	hypothetical protein
i02_2835	c3039	yfgB	hypothetical protein
	c3040	/	hypothetical protein
i02_2836	c3041	ndk	nucleoside diphosphate kinase
i02_2837	c3042	pbpC	penicillin-binding protein 1C
i02_2838	c3043	yfhM	lipoprotein yfhM precursor
i02_2839	c3044	sseA	3-mercaptopyruvate sulfurtransferase
i02_2840	c3045	/	hypothetical protein
i02_2841	c3046	/	hypothetical protein
i02_2842	c3047	sseB	enhanced serine sensitivity protein SseB
i02_2843	c3048	pepB	aminopeptidase B
i02_2844	c3049	yfhJ	hypothetical protein
i02_2845	c3050	fdx	ferredoxin, 2Fe-2S
i02_2846	c3051	hscA	chaperone protein HscA
i02_2847	c3052	hscB	co-chaperone HscB
i02_2848		/	hypothetical protein
	c3054	/	hypothetical protein
i02_2849	c3053	iscA	iron-sulfur cluster assembly protein
i02_2850	c3055	/	scaffold protein
i02_2851	c3056	yfhO	cysteine desulfurase
i02_2852	c3057	yfhP	DNA-binding transcriptional regulator IscR
i02_2853	c3058	yfhQ	putative tRNA/rRNA methyltransferase YfhQ
i02_2854	c3059	suhB	inositol monophosphatase
i02_2855	c3060	yfhR	hypothetical protein
i02_2856	c3061	csiE	stationary phase inducible protein CsiE
i02_2857	c3062	hcaT	putative 3-phenylpropionic acid transporter
i02_2858	c3063	/	hypothetical protein
i02_2859	c3064	/	hypothetical protein
i02_2860	c3065	yphA	hypothetical protein
i02_2861	c3066	yphB	hypothetical protein
i02_2862	c3067	yphC	hypothetical protein
i02_2863	c3068	yphD	ABC transporter permease
i02_2864	c3069	yphE	ABC transporter ATP-binding protein
i02_2865	c3070	yphF	ABC transporter periplasmic-binding protein

Clone D	CFT 073	Gene	Product
i02_2866	c3071	yphG	hypothetical protein
i02_2867	c3072	yphH	hypothetical protein
i02_2868	c3073	glyA	serine hydroxymethyltransferase
i02_2869		/	hypothetical protein
i02_2870	c3075	hmpA	nitric oxide dioxygenase
i02_2871	c3076	glnB	nitrogen regulatory protein P-II 1
i02_2872	c3077	yfhA	hypothetical protein
i02_2873	c3078	yfhG	hypothetical protein
i02_2874	c3079	yfhK	putative sensor-like histidine kinase yfhK
i02_2875	c3080	purL	phosphoribosylformylglycinamidine synthase
i02_2876	c3081	yfhD	putative transglycosylase
i02_2877	c3082	yfhC	tRNA-specific adenosine deaminase
i02_2878	c3083	yfhB	hypothetical protein
i02_2879	c3084	/	hypothetical protein
i02_2880	c3085	yfhH	putative DNA-binding transcriptional regulator
i02_2881	c3086	yfhL	putative ferredoxin-like protein yfhL
i02_2882	c3087	acpS	4'-phosphopantetheinyl transferase
i02_2883	c3088	pdxJ	pyridoxine 5'-phosphate synthase
i02_2884	c3089	recO	DNA repair protein RecO
i02_2885	c3090	era	GTP-binding protein Era
i02_2886	c3091	rnc	ribonuclease III
i02_2887	c3092	lepB	signal peptidase I
i02_2888	c3093	lepA	GTP-binding protein LepA
i02_2889	c3094	rseC	SoxR reducing system protein RseC
i02_2890	c3095	rseB	periplasmic negative regulator of sigmaE
i02_2891	c3096	rseA	anti-RNA polymerase sigma factor SigE
i02_2892	c3097	rpoE	RNA polymerase sigma factor RpoE
i02_2893	c3098	nadB	L-aspartate oxidase
i02_2894	c3099	yfiC	hypothetical protein
i02_2895	c3100	srmB	ATP-dependent RNA helicase SrmB
i02_2896	c3101	yfiE	putative transcriptional regulator YfiE
i02_2897	c3102	yfiK	neutral amino-acid efflux protein
i02_2899	c3104	/	hypothetical protein
i02_2898	c3103	yfiD	autonomous glycyl radical cofactor GrcA
i02_2900	c3105	ung	uracil-DNA glycosylase
i02_2902		yfiF	rRNA methyltransferase YfiF
i02_2901	c3106	yfiF	putative methyltransferase
i02_2903	c3107	trxC	thioredoxin 2
i02_2904	c3108	yfiP	hypothetical protein
i02_2905	c3109	yfiQ	hypothetical protein
i02_2906	c3110	pssA	phosphatidylserine synthase
i02_2907	c3111	yfiM	hypothetical protein
i02_2908	c3112	kgtP	alpha-ketoglutarate transporter
i02_2910	c3113	/	hypothetical protein
i02_2911	c3114	clpB	protein disaggregation chaperone
i02_2912	c3115	yfiH	hypothetical protein
i02_2913	c3116	rluD	23S rRNA pseudouridine synthase D
i02_2914	c3117	yfiO	outer membrane protein assembly complex subunit
	c3118	/	hypothetical protein
i02_2915	c3119	yfiA	translation inhibitor protein RaiA
	c5497	pheL	Phe leader peptide

Clone D	CFT 073	Gene	Product
i02_2916	c3120	pheA	bifunctional chorismate mutase/prephenate
i02_2917	c3121	tyrA	bifunctional chorismate mutase/prephenate
i02_2918	c3122	aroF	phospho-2-dehydro-3-deoxyheptonate aldolase
i02_2919	c3123	yfiL	hypothetical protein
i02_2920	c3124	yfiR	hypothetical protein
i02_2921	c3125	yfiN	hypothetical protein
i02_2922	c3126	yfiB	putative outer membrane lipoprotein
i02_2923	c3127	rplS	50S ribosomal protein L19
i02_2924	c3128	trmD	tRNA (guanine-N(1)-)-methyltransferase
i02_2925	c3129	rimM	16S rRNA-processing protein RimM
i02_2926	c3130	rpsP	30S ribosomal protein S16
i02_2927	c3131	ffh	signal recognition particle protein
i02_2928	c3132	ypjD	hypothetical protein
i02_2929	c3133	/	hypothetical protein
	c3134	/	hypothetical protein
i02_2930	c3135	grpE	heat shock protein GrpE
i02_2932	c3137	ppnK	inorganic polyphosphate/ATP-NAD kinase
i02_2931	c3136	/	hypothetical protein
i02_2933	c3138	recN	recombination and repair protein
i02_2934	c3139	/	hypothetical protein
i02_2935	c3140	/	hypothetical protein
i02_2936	c3141	yfjG	hypothetical protein
i02_2937	c3142	smpB	SsrA-binding protein
-	c3143	/	hypothetical protein
-	c3144	/	DNA-damage-inducible protein I
-	c3145	ydfK	hypothetical protein
-	c3146	/	putative DNA-invertase from lambdoid prophage
-	c3147	/	hypothetical protein
-	c3148	/	hypothetical protein
-	c3149	/	hypothetical protein
-	c3150	/	hypothetical protein
-	c3151	/	hypothetical protein
-	c3152	/	hypothetical protein
-	c3153	/	putative outer membrane protein of prophage
-	c3154	/	putative tail component of prophage
-	c3155	/	putative tail component of prophage
-	c3156	/	putative tail fiber component K of prophage
-	c3157	/	hypothetical protein
-	c3158	/	putative tail component of prophage
-	c3159	/	putative tail component of prophage
-	c3160	/	putative tail component of prophage
-	c3161	/	putative tail component of prophage
-	c3162	/	putative tail component of prophage
-	c3163	/	putative tail component of prophage
-	c3164	/	putative tail component of prophage
-	c3165	/	putative tail fiber component Z of prophage
-	c3166	/	putative head-tail joining protein of prophage
-	c3167	/	putative DNA packaging protein of prophage
-	c3168	/	putative capsid protein of prophage
-	c3169	/	putative head-DNA stabilization protein
-	c3170	/	putative capsid protein of prophage
-	c3171	/	putative capsid structural protein of prophage

Clone D	CFT 073	Gene	Product
-	c3172	/	putative head-tail joining protein of prophage
-	c3173	/	putative DNA packaging protein of prophage
-	c3174	nohB	prophage qsr' DNA packaging protein NU1-like
-	c3175	/	hypothetical protein
-	c3176	/	GnsB protein
-	c3177	cspI	cold shock-like protein cspI
-	c3178	/	hypothetical protein
-	c3179	ydfP	hypothetical protein
-	c3180	/	lysozyme from lambdoid prophage Qin
-	c3181	/	hypothetical protein
-	c3182	/	lambdoid prophage Qin lysis protein S
-	c3183	/	hypothetical protein
-	c3184	cspB	cold shock-like protein cspB
-	c3185	cspF	cold shock-like protein cspF
-	c3186	/	lambdoid prophage Qin antitermination protein Q
-	c3187	/	hypothetical protein
-	c3188	/	hypothetical protein
-	c3189	/	hypothetical protein
-	c3190	/	hypothetical protein
-	c3191	yfdN	hypothetical protein
-	c3192	/	hypothetical protein
-	c3193	/	hypothetical protein
-	c3194	/	hypothetical protein
-	c3195	ymfL	hypothetical protein
-	c3196	/	hypothetical protein
-	c3197	/	putative repressor protein of prophage
-	c3198	/	hypothetical protein
-	c3199	/	hypothetical protein
-	c3200	/	hypothetical protein
-	c3201	yfdQ	hypothetical protein
-	c3202	yfdR	hypothetical protein
-	c3203	/	hypothetical protein
-	c3204	/	hypothetical protein
-	c3205	/	hypothetical protein
-	c3206	/	hypothetical protein
i02_2938	c3207	ygaT	hypothetical protein
i02_2939	c3208	ygaF	hydroxyglutarate oxidase
i02_2940	c3209	gabD	succinate-semialdehyde dehydrogenase I
i02_2941	c3210	gabT	4-aminobutyrate aminotransferase
i02_2942	c3211	gabP	gamma-aminobutyrate transporter
i02_2943	c3212	ygaE	DNA-binding transcriptional regulator CsiR
i02_2944	c3213	ygaU	LysM domain/BON superfamily protein
i02_2945	c3214	/	hypothetical protein
	c3215	yqaE	hypothetical protein
i02_2946	c3216	ygaV	putative transcriptional regulator YgaV
i02_2947	c3217	ygaP	hypothetical protein
i02_2948	c3218	stpA	DNA binding protein, nucleoid-associated
i02_2949	c3219	/	hypothetical protein
i02_2950	c3220	/	hypothetical protein
i02_2951	c3221	ygaW	hypothetical protein
i02_2952	c3222	ygaC	hypothetical protein
i02_2953	c3223	ygaM	hypothetical protein

Clone D	CFT 073	Gene	Product
i02_2954	c3224	/	hypothetical protein
i02_2955	c3225	/	hypothetical protein
i02_2956	c3226	nrdH	glutaredoxin-like protein
i02_2957	c3227	nrdI	ribonucleotide reductase stimulatory protein
i02_2958	c3228	nrdE	ribonucleotide-diphosphate reductase subunit
i02_2959	c3229	nrdF	ribonucleotide-diphosphate reductase subunit
i02_2960	c3230	proV	glycine betaine transporter ATP-binding subunit
i02_2961	c3231	proW	glycine betaine transporter membrane protein
i02_2962	c3232	proX	glycine betaine transporter periplasmic subunit
i02_2963	c3233	/	hypothetical protein
i02_2964	c3234	/	putative transport protein
i02_2965	c3235	ygaZ	hypothetical protein
i02_2966	c3236	ygaH	hypothetical protein
i02_2967	c3237	emrR	transcriptional repressor MprA
i02_2968	c3238	emrA	multidrug resistance protein A
i02_2969	c3239	emrB	multidrug resistance protein B
i02_2970	c3240	/	hypothetical protein
	c3241	/	hypothetical protein
i02_2972	c3242	/	hypothetical protein
i02_2973	c3243	/	hypothetical protein
i02_2974	c3244	ygaG	S-ribosylhomocysteinase
i02_2975	c3245	gshA	glutamate--cysteine ligase
i02_2976	c3246	yqaA	hypothetical protein
i02_2977	c3247	yqaB	fructose-1-phosphatase
i02_2978	c3248	/	hypothetical protein
i02_2979	c3249	csrA	carbon storage regulator
i02_2980		/	hypothetical protein
i02_2982	c3251	/	hypothetical protein
i02_2981		alaS	alanyl-tRNA synthetase
i02_2983	c3252	recX	recombination regulator RecX
i02_2984	c3253	recA	recombinase A
i02_2985	c3254	ygaD	competence damage-inducible protein A
i02_2986	c3255	mltB	murein hydrolase B
i02_2987	c3256	srlA	PTS system, glucitol/sorbitol-specific IIC2
i02_2988	c3257	srlE	PTS system, glucitol/sorbitol-specific IIBC
i02_2989	c3258	srlB	glucitol/sorbitol-specific PTS system component
i02_2990	c3259	srlD	sorbitol-6-phosphate dehydrogenase
i02_2991	c3260	gutM	DNA-binding transcriptional activator GutM
i02_2992	c3261	srlR	DNA-bindng transcriptional repressor SrlR
i02_2993	c3262	gutQ	D-arabinose 5-phosphate isomerase
i02_2994	c3263	ygaA	anaerobic nitric oxide reductase transcription
i02_2995		norV	anaerobic nitric oxide reductase flavorubredoxin
	c3264	/	hypothetical protein
i02_2997	c3267	ygbD	nitric oxide reductase
i02_2998	c3268	hypF	hydrogenase maturation protein hypF
i02_2999	c3269	hydN	electron transport protein HydN
i02_3000	c3270	/	hypothetical protein
i02_3001	c3271	/	hypothetical protein
i02_3002	c3272	/	hypothetical protein
i02_3003	c3273	/	hypothetical protein
	c3275	/	hypothetical protein
i02_3004	c3274	/	hypothetical protein

Clone D	CFT 073	Gene	Product
i02_3005	c3276	/	hypothetical protein
i02_3006	c3277	hycI	hydrogenase 3 maturation protease
i02_3007	c3278	hycH	formate hydrogenlyase maturation protein hycH
i02_3008	c3279	hycG	formate hydrogenlyase subunit 7
i02_3009	c3280	hycF	formate hydrogenlyase complex iron-sulfur
i02_3010	c3281	hycE	formate hydrogenlyase subunit 5 precursor
i02_3011	c3282	hycD	formate hydrogenlyase subunit 4
i02_3012	c3283	hycC	formate hydrogenlyase subunit 3
i02_3013	c3284	hycB	formate hydrogenlyase subunit 2
i02_3014	c3285	hycA	formate hydrogenlyase regulatory protein HycA
i02_3015	c3286	hypA	hydrogenase nickel incorporation protein
i02_3016	c3287	hypB	hydrogenase nickel incorporation protein HypB
i02_3017	c3288	hypC	hydrogenase assembly chaperone
i02_3018	c3289	hypD	hydrogenase isoenzyme formation protein hypD
i02_3019	c3290	hypE	hydrogenase isoenzyme formation protein hypE
i02_3020	c3291	fhlA	formate hydrogenlyase transcriptional activator
i02_3021	c3292	/	molybdenum-pterin-binding-protein
i02_3022	c3293	ygbA	hypothetical protein
i02_3023	c3294	mutS	DNA mismatch repair protein MutS
i02_3024	c3295	pphB	serine/threonine-specific protein phosphatase 2
i02_3025	c3296	ygbI	putative transcriptional regulator YgbI
i02_3026	c3297	ygbJ	oxidoreductase ygbJ
i02_3027	c3298	ygbK	hypothetical protein
i02_3028	c3299	ygbL	putative aldolase
i02_3029	c3300	ygbM	hypothetical protein
i02_3030	c3301	ygbN	inner membrane permease YgbN
i02_3031	c3302	/	hypothetical protein
i02_3032	c3303	/	hypothetical protein
i02_3033	c3304	/	hypothetical protein
	c3305		hypothetical protein
	c3305	/	hypothetical protein
i02_3034	c3306	/	hypothetical protein
i02_3034	c3307	/	hypothetical protein
i02_3036	c3309	/	hypothetical protein
i02_3035	c3308	nlpD	lipoprotein NlpD
i02_3037	c3310	pcm	protein-L-isoaspartate O-methyltransferase
i02_3038	c3311	surE	stationary phase survival protein SurE
i02_3039	c3312	truD	tRNA pseudouridine synthase D
i02_3040	c3313	ispF	2-C-methyl-D-erythritol 2,4-cyclodiphosphate synthase monomer
i02_3041	c3314	ispD	4-diphosphocytidyl-2C-methyl-D-erythritol synthetase monomer
i02_3042	c3315	ftsB	cell division protein FtsB
i02_3043	c3316	ygbE	hypothetical protein
i02_3044	c3317	cysC	adenylylsulfate kinase
i02_3045	c3318	cysN	sulfate adenylyltransferase subunit 1
i02_3046	c3319	cysD	sulfate adenylyltransferase subunit 2
i02_3047	c3320	iap	alkaline phosphatase isozyme conversion
i02_3048	c3321	cysH	phosphoadenosine phosphosulfate reductase
i02_3049	c3322	cysI	sulfite reductase subunit beta
i02_3050	c3323	cysJ	sulfite reductase subunit alpha
i02_3051	c3324	ygcM	putative 6-pyruvoyl tetrahydrobiopterin

Clone D	CFT 073	Gene	Product
i02_3052	c3325	ygcN	electron transfer flavoprotein-quinone
i02_3053	c3326	ygcO	ferredoxin-like protein ygcO
i02_3054		/	putative anti-terminator regulatory protein
i02_3055	c3328	ygcQ	putative electron transfer flavoprotein subunit
i02_3056	c3329	ygcR	putative electron transfer flavoprotein subunit
i02_3057	c3330	ygcS	metabolite transport protein
i02_3058	c3331	/	hypothetical protein
i02_3059	c3332	ygcW	oxidoreductase ygcW
i02_3060	c3333	yqcE	hypothetical protein
i02_3061	c3334	ygcE	putative sugar kinase ygcE
i02_3062	c3335	ygcF	hypothetical protein
i02_3063	c3336	/	hypothetical protein
i02_3064	c3337	/	hypothetical protein
i02_3065	c3338	/	hypothetical protein
	c3339	/	hypothetical protein
i02_3067	c3340	/	hypothetical protein
i02_3068	c3342	ygcG	hypothetical protein
	c3343	/	hypothetical protein
i02_3069	c3344	eno	phosphopyruvate hydratase
i02_3070	c3345	pyrG	CTP synthetase
i02_3071	c3346	mazG	nucleoside triphosphate pyrophosphohydrolase
i02_3072	c3347	relA	GDP/GTP pyrophosphokinase
i02_3073	c3348	rumA	23S rRNA 5-methyluridine methyltransferase
i02_3074	c3349	barA	hybrid sensory histidine kinase BarA
i02_3075		/	hypothetical protein
i02_3076	c3350	ygcX	glucarate dehydratase
i02_3077	c3352	ygcY	glucarate dehydratase related protein
i02_3078	c3353	/	glucarate transporter
i02_3080	c3355	/	hypothetical protein
i02_3079	c3354	/	hypothetical protein
i02_3081	c3356	yqcA	flavodoxin
i02_3082	c3357	yqcB	tRNA pseudouridine synthase C
i02_3083	c3358	yqcC	hypothetical protein
i02_3084	c3359	syd	SecY interacting protein Syd
i02_3085	c3360	queF	7-cyano-7-deazaguanine reductase
i02_3086	c3361	ygdH	hypothetical protein
i02_3087	c3362	/	hypothetical protein
	c3363	/	hypothetical protein
i02_3088	c3364	sdaC	Serine transporter
i02_3089	c3365	sdaB	L-serine dehydratase 2
i02_3090	c3366	xni	exonuclease IX
i02_3091	c3367	fucO	L-1,2-propanediol oxidoreductase
i02_3092	c3368	fucA	L-fuculose phosphate aldolase
i02_3093	c3369	/	hypothetical protein
i02_3094	c3370	fucP	L-fucose transporter
i02_3095	c3371	fucI	L-fucose isomerase
i02_3096		fucK	culokinase
i02_3097	c3374	fucU	fucose operon fucU protein
i02_3098	c3375	fucR	DNA-binding transcriptional activator FucR
i02_3099	c3376	ygdE	putative RNA 2'-O-ribose methyltransferase
i02_3100	c3377	ygdD	hypothetical protein
i02_3101	c3378	gcvA	DNA-binding transcriptional activator GcvA

Clone D	CFT 073	Gene	Product
	c3379	/	hypothetical protein
i02_3102	c3380	ygdI	lipoprotein ygdI precursor
i02_3103	c3381	/	cysteine sulfinate desulfinase
i02_3104	c3382	ygdK	hypothetical protein
i02_3105	c3383	ygdL	hypothetical protein
i02_3106	c3384	mltA	murein transglycosylase A
i02_3107	c3385	/	hypothetical protein
i02_3108	c3386	/	hypothetical protein
i02_3109	c3387	/	hypothetical protein
i02_3110	c3388	/	hypothetical protein
i02_3111	c3389	/	hypothetical protein
	c3390	/	hypothetical protein
i02_3112	c3391	/	secreted protein Hcp
i02_3113	c3392	/	ClpB protein
i02_3114	c3393	/	hypothetical protein
i02_3115	c3394	/	hypothetical protein
i02_3116	c3395	/	hypothetical protein
i02_3117	c3396	/	hypothetical protein
i02_3118	c3397	/	hypothetical protein
i02_3119	c3398	/	hypothetical protein
i02_3120	c3399	/	hypothetical protein
i02_3121	c3400	/	hypothetical protein
i02_3122	c3400	/	hypothetical protein
i02_3123	c3401	/	hypothetical protein
i02_3124	c3402	/	hypothetical protein
i02_3125	c3403	/	hypothetical protein
	c3404	/	hypothetical protein
i02_3127	c3405	/	2-hydroxyacid dehydrogenase
i02_3128	c3406	/	phosphosugar isomerase
i02_3129	c3407	/	Beta-cystathionase
i02_3130	c3408	/	PTS system, maltose and glucose-specific IIABC
i02_3131	c3409	/	antiterminator
i02_3132	c3410	/	hypothetical protein
i02_3133	c3411	/	N-acetylmuramoyl-L-alanine amidase amiC
i02_3134	c3412	argA	N-acetylglutamate synthase
i02_3135	c3413	recD	exonuclease V subunit alpha
i02_3136	c3414	recB	exonuclease V subunit beta
i02_3137	c3415	ptr	protease III
i02_3138	c3416	recC	exonuclease V subunit gamma
i02_3139	c3417	ppdC	hypothetical protein
i02_3140	c3418	ygdB	hypothetical protein
i02_3141	c3419	ppdB	hypothetical protein
i02_3142	c3420	ppdA	hypothetical protein
i02_3143	c3422	thyA	thymidylate synthase
i02_3144	c3423	lgt	prolipoprotein diacylglyceryl transferase
i02_3145	c3424	ptsP	fused phosphoenolpyruvate-protein
i02_3146	c3425	ygdP	dinucleoside polyphosphate hydrolase
i02_3147	c3426	mutH	DNA mismatch repair protein
i02_3148	c3427	/	hypothetical protein
i02_3149	c3428	ygdR	lipoprotein ygdR precursor
i02_3150	c3429	tas	putative aldo-keto reductase
i02_3151	c3430	ygeD	lysophospholipid transporter LplT

Clone D	CFT 073	Gene	Product
i02_3152	c3431	aas	bifunctional acyl-[acyl carrier protein]
-	c3432	/	transposase
-	c3433	/	hypothetical protein
i02_3153	c3434	galR	DNA-binding transcriptional regulator GalR
i02_3154	c3435	lysA	diaminopimelate decarboxylase
i02_3155	c3436	lysR	DNA-binding transcriptional regulator LysR
i02_3156	c3437	ygeA	putative racemase
i02_3157	c3438	araE	arabinose-proton symporter
i02_3158	c3439	kduD	2-deoxy-D-gluconate 3-dehydrogenase
i02_3159	c3440	kduI	5-keto-4-deoxyuronate isomerase
i02_3160	c3441	yqeF	acetyl-CoA acetyltransferase
i02_3161	c3442	yqeG	putative transport protein YqeG
i02_3162	c3443	ygeR	lipoprotein ygeR precursor
i02_3163	c3444	/	xanthine dehydrogenase subunit XdhA
i02_3164	c3445	ygeT	xanthine dehydrogenase subunit XdhB
i02_3165	c3446	/	xanthine dehydrogenase subunit XdhC
i02_3166	c3447	ygeV	putative sigma-54-dependent transcriptional
i02_3167	c3448	ygeW	aspartate/ornithine carbamoyltransferase family
i02_3168	c3449	ygeX	diaminopropionate ammonia-lyase
i02_3169	c3450	ygeY	peptidase
i02_3170	c3451	ygeZ	phenylhydantoinase
i02_3171	c3452	yqeA	carbamate kinase
i02_3172	c3453	yqeB	hypothetical protein
i02_3173	c3454	yqeC	hypothetical protein
i02_3174	c3455	ygfJ	hypothetical protein
i02_3175	c3456	ygfK	putative selenate reductase subunit YgfK
i02_3176	c3457	/	putative chlorohydrolase/aminohydrolase
i02_3177	c3458	ygfM	putative selenate reductase subunit YgfM
i02_3178	c3459	/	putative selenate reductase subunit YgfN
i02_3179	c3460	ygfO	putative purine permease ygfO
i02_3180	c3461	ygfP	guanine deaminase
i02_3181	c3462	/	hypothetical protein
i02_3182	c3463	ygfS	putative electron transport protein ygfS
i02_3183	c3464	ygfT	putative oxidoreductase Fe-S binding subunit
i02_3184	c3465	ygfU	putative purine permease ygfU
i02_3185	c3466	/	hypothetical protein
i02_3186	c3467	/	isopentenyl-diphosphate delta-isomerase
-	c3468	/	hypothetical protein
i02_3188	c3470	/	hypothetical protein
i02_3187	c3469	lysS	lysyl-tRNA synthetase
i02_3189		prfB	peptide chain release factor 2
i02_3190		/	conserved hypothetical protein
i02_3191	c5622	prfB	peptide chain release factor 2
i02_3192	c3472	recJ	ssDNA exonuclease RecJ
i02_3193	c3473	dsbC	thiol:disulfide interchange protein DsbC
i02_3194	c3474	xerD	site-specific tyrosine recombinase XerD
i02_3195	c3475	fldB	flavodoxin FldB
i02_3196	c3476	ygfX	hypothetical protein
i02_3198	c3478	/	hypothetical protein
i02_3197	c3477	ygfY	hypothetical protein
i02_3199	c3479	ygfZ	putative global regulator
i02_3200	c3480	yqfA	hypothetical protein

Clone D	CFT 073	Gene	Product
i02_3201	c3481	yqfB	hypothetical protein
i02_3202	c3482	bglA	6-phospho-beta-glucosidase BglA
i02_3203	c3483	gcvP	glycine dehydrogenase
i02_3204	c3484	gcvH	glycine cleavage system protein H
i02_3205	c3485	gcvT	glycine cleavage system aminomethyltransferase
i02_3206	c3486	/	hypothetical protein
i02_3207	c3487	/	hypothetical protein
i02_3208	c3488	visC	hypothetical protein
i02_3209	c3489	ubiH	2-octaprenyl-6-methoxyphenyl hydroxylase
i02_3210	c3490	pepP	proline aminopeptidase P II
i02_3211	c3491	ygfB	hypothetical protein
i02_3212	c3492	ygfE	Z-ring-associated protein
i02_3213	c3493	ygfA	putative ligase
i02_3214	c3494	serA	D-3-phosphoglycerate dehydrogenase
i02_3215	c3495	rpiA	ribose-5-phosphate isomerase A
i02_3216	c3496	yqfE	hypothetical protein
i02_3218	c3497	iciA	chromosome replication initiation inhibitor
i02_3217		/	conserved hypothetical protein
i02_3219	c3498	/	hypothetical protein
i02_3220	c3499	ygfI	putative transcriptional regulator YgfI
i02_3221	c3500	yggE	hypothetical protein
i02_3222	c3501	yggA	arginine exporter protein
i02_3223	c3502	yggB	mechanosensitive channel MscS
i02_3224	c3503	fba	fructose-bisphosphate aldolase
i02_3225	c3504	pgk	phosphoglycerate kinase
i02_3226	c3505	epd	erythrose 4-phosphate dehydrogenase
i02_3227	c3506	/	hypothetical protein
i02_3228	c3507	/	hypothetical protein
i02_3229	c3508	/	hypothetical protein
i02_3230	c3509	/	ABC transporter ATP-binding protein
i02_3231	c3510	/	ABC transporter ATP-binding protein
i02_3232	c3511	frcK	putative fructose transport system kinase
i02_3233	c3512	yggD	putative DNA-binding transcriptional regulator
i02_3234	c3513	/	hypothetical protein
i02_3235	c3514	/	putative oxidoreductase
i02_3236	c3515	cmtA	PTS system, mannitol (Cryptic)-specific IIBC
i02_3237	c3516	cmtB	putative mannitol phosphotransferase subunit
i02_3238	c3517	/	hypothetical protein
i02_3239	c3520	tktA	transketolase
i02_3240	c3521	yggG	putative metalloprotease yggG
i02_3241	c3522	speB	agmatinase
i02_3242	c3523	/	hypothetical protein
i02_3243	c3524	speA	arginine decarboxylase
i02_3244	c3525	yqgB	hypothetical protein
	c3526	/	hypothetical protein
i02_3245	c3527	yqgD	hypothetical protein
i02_3246	c3528	metK	S-adenosylmethionine synthetase
i02_3247	c3529	galP	galactose-proton symporter
i02_3248	c3530	sprT	hypothetical protein
i02_3249	c3531	endA	endonuclease I precursor
i02_3250	c3532	yggJ	16S ribosomal RNA methyltransferase RsmE
i02_3251	c3533	gshB	glutathione synthetase

Clone D	CFT 073	Gene	Product
i02_3252	c3534	yqgE	hypothetical protein
i02_3253	c3535	yqgF	Holliday junction resolvase-like protein
i02_3255	c3537	yggS	hypothetical protein
i02_3254	c3536	yggR	hypothetical protein
i02_3256	c3538	yggT	hypothetical protein
i02_3257	c3539	yggU	hypothetical protein
i02_3258	c3540	yggV	putative deoxyribonucleotide triphosphate
i02_3259	c3541	yggW	coproporphyrinogen III oxidase
i02_3260	c3542	yggM	hypothetical protein
i02_3261	c3543	ansB	L-asparaginase II
i02_3262	c3544	yggN	hypothetical protein
i02_3263	c3545	/	hypothetical protein
i02_3264	c3546	yggL	hypothetical protein
i02_3265	c3547	trmB	tRNA (guanine-N(7)-)-methyltransferase
i02_3266	c3548	mutY	adenine DNA glycosylase
	c3549	/	hypothetical protein
i02_3267	c3550	yggX	hypothetical protein
i02_3268	c3551	mltC	murein transglycosylase C
i02_3269	c3552	nupG	nucleoside permease nupG
i02_3270	c3553	speC	ornithine decarboxylase
i02_3271	c3554	/	hypothetical protein
i02_3272	c3555	yqgA	hypothetical protein
i02_3273	c3556	/	prophage P4 integrase
i02_3274	c3557	/	ShiA-like protein
i02_3275	c3558	/	hypothetical protein
i02_3276	-	insA	InsA protein
i02_3277	-	insb	InsB protein
-	c3559	/	hypothetical protein
-	c3560	/	hypothetical protein
-	c3561	/	hypothetical protein
-	c3562	/	hypothetical protein
-	c3563	/	hypothetical protein
-	c3564	/	hypothetical protein
-	c3565	/	putative response regulator
-	c3566	/	hypothetical protein
-	c3567	/	hypothetical protein
-	c3568	/	hypothetical protein
-	c3569	hlyC	hemolysin C
-	c3571	/	hypothetical protein
	c3570	hlyA	hemolysin A
-	c3572	/	hypothetical protein
-	c3573	hlyB	hemolysin B
-	c3574	hlyD	hemolysin D
-	c3575	/	transposase insF
-	c3576	/	hypothetical protein
-	c3577	/	hypothetical protein
-	c3578	/	hypothetical protein
-	c3579	/	hypothetical protein
-	c3580	/	hypothetical protein
-	c3581	/	hypothetical protein
i02_3278	c3582	papX	PapX protein
i02_3279	-	/	hypothetical protein

Clone D	CFT 073	Gene	Product
i02_3280	-	/	IS1 protein InsB
i02_3281	-	/	insertion element IS1 1/2/3/5/6 protein insA
-	c3583	papG	PapG protein
-	c3584	papF	PapF protein
-	c3585	papE	PapE protein
-	c3586	papK	PapK protein
-	c3587	/	hypothetical protein
-	c3588	papJ	PapJ protein
-	c3589	papD	PapD protein
-	c3590	papC	PapC protein
-	c3591	papH	PapH protein
i02_3282	c3592	papA	PapA protein
i02_3284		/	PapB-like protein
i02_3286	c3593	papI	PapI protein
	c3596	/	hypothetical protein
i02_3287	c3594	/	putative transposase
i02_3287	c3595	/	transposase
	c3596	/	Insertion Sequence Associated
i02_3288		/	hypothetical protein
i02_3289	c3597	/	transposase
i02_3290	c3598	/	hypothetical protein
i02_3291	c3599	/	hypothetical protein
i02_3292	c3600	/	hypothetical protein
i02_3293	c3601	/	hypothetical protein
i02_3294	c3612	/	transposase insC
i02_3294a	c3611	/	insertion element IS2 transposase InsD
i02_3295	c3610	iha	bifunctional enterobactin receptor/adhesin
i02_3296	c3609	/	hypothetical protein
-	c3608	/	hypothetical protein
-	c3607	/	hypothetical protein
i02_3297	c3606	/	hypothetical protein
i02_3298	c3605	/	hypothetical protein
	c3604		
i02_3299	c3603	/	hypothetical protein
i02_3300	c3602	/	hypothetical protein
i02_3301		/	conserved hypothetical protein
i02_3302		/	putative transposase
i02_3303	-	/	conserved hypothetical protein
i02_3304		/	IS1353 transposase family protein
i02_3305	-	/	transposase
i02_3307	-	/	transposase IS3 family
-	c3613	/	hypothetical protein
-	c3614	/	hypothetical protein
-	c3615	/	hypothetical protein
-	c3616	/	hypothetical protein
-	c3617	/	hypothetical protein
i02_3308	c3618	/	hypothetical protein
i02_3310	-	insI	transposase insI
i02_3311	-	/	hypothetical protein
i02_3309	-	/	transposase insF
i02_3312	c3618	/	hypothetical protein
	c3621	/	hypothetical protein

Clone D	CFT 073	Gene	Product
i02_3313	c3619	sat	secreted auto transpoter toxin
i02_3315	c3622	/	hypothetical protein
i02_3316	c3623	iutA	IutA protein
i02_3317	c3624	iucD	IucD protein
i02_3318	c3625	iucC	IucC protein
i02_3319	c3626	iucB	IucB protein
i02_3320	c3627	iucA	IucA protein
i02_3321	c3628	shiF	shiF protein
	c3629	/	hypothetical protein
i02_3323	-	/	insertion element IS2 transposase InsD
i02_3324	-	/	transposase insC
i02_3325	c3630	/	hypothetical protein
i02_3326	c3631	/	hypothetical protein
i02_3327	c3632	/	hypothetical protein
i02_3328	c3633	/	hypothetical protein
i02_3329	c3634	/	hypothetical protein
i02_3330	c3635	/	hypothetical protein
i02_3331	c3636	/	hypothetical protein
i02_3332	c3637	/	putative sialic acid transporter
i02_3333	c3638	/	N-acetylmannosamine kinase
i02_3334	c3639	/	N-acetylneuraminate lyase
i02_3335	c3640	/	hypothetical protein
i02_3336	c3641	/	hypothetical protein
	c3642	/	hypothetical protein
i02_3337	c3643	/	hypothetical protein
	c3644	/	hypothetical protein
i02_3338	c3645	/	hypothetical protein
i02_3339	c3646	/	hypothetical protein
i02_3340	c3647	/	hypothetical protein
i02_3341	c3648	/	hypothetical protein
i02_3342		/	predicted protein
i02_3343	c3649	/	hemolysin expression modulating protein
i02_3344		/	hypothetical protein
i02_3345	c3650	/	hypothetical protein
i02_3346	c3651	/	hypothetical protein
i02_3347	c3652	/	hypothetical protein
i02_3348	c3653	/	hypothetical protein
i02_3349		/	conserved hypothetical protein
i02_3350	c3654	/	hypothetical protein
i02_3351	c3655	/	antigen 43 precursor
-	c3656	/	hypothetical protein
-	c3657	/	hypothetical protein
-	c3658	/	hypothetical protein
-	c3659	/	hypothetical protein
-	c3660	/	hypothetical protein
-	c3661	/	hypothetical protein
-	c3662	/	hypothetical protein
-	c3663	/	hypothetical protein
-	c3664	/	hypothetical protein
i02_3352	c3665	/	hypothetical protein
i02_3353	c3666	/	hypothetical protein
	c3667	/	hypothetical protein

Clone D	CFT 073	Gene	Product
i02_3354	c3668	/	hypothetical protein
	c3669	/	hypothetical protein
i02_3356	c3671	/	putative radC-like protein yeeS
i02_3355	c3670	/	hypothetical protein
	c3673	/	hypothetical protein
i02_3358	c3674	/	hypothetical protein
i02_3357	c3672	/	hypothetical protein
	c3676	/	hypothetical protein
i02_3360	c3677	/	hypothetical protein
i02_3359	c3675	/	hypothetical protein
i02_3361	c3678	/	hypothetical protein
	c3679	/	hypothetical protein
i02_3362	c3680	/	hypothetical protein
i02_3363	c3681	/	hypothetical protein
i02_3364	c3682	/	hypothetical protein
i02_3365	c3683	/	hypothetical protein
i02_3366	c3684	/	hypothetical protein
i02_3367	c3685	/	hypothetical protein
i02_3368	c3686	/	hypothetical protein
i02_3369	c3687	kpsE	KpsE protein
i02_3370	c3688	kpsD	KpsD protein
i02_3371	c3689	/	3-deoxy-manno-octulosonate cytidylyltransferase
i02_3372	c3690	kpsC	KpsC protein
i02_3373	c3691	kpsS	KpsS protein
-	c3692	/	hypothetical protein
-	c3693	/	hypothetical protein
i02_3373a		kfiD	UDP-glucose 6-dehydrogenase
i02_3374	-	/	glycosyltransferase KfiC
i02_3375	-	/	conserved hypothetical protein
i02_3376		kfiB	kfiB protein
	c3694	/	hypothetical protein
-	c3695	/	hypothetical protein
-	c3696	/	Putative glycerol-3-phosphate cytidyltransferase
i02_3377	c3697	kpsT	KpsT protein
i02_3378	c3698	kpsM	KpsM protein
i02_3379	c3699	yghD	putative general secretion pathway protein YghD
i02_3380	pseudogene	gspL	GspL-like protein
i02_3381	pseudogene	gspK	hypothetical protein
	c3703	/	IS, phage, Tn; Transposon-related function
i02_3382	-	gspJ	putative type II secretion protein GspJ
i02_3383	-	gspI	putative type II secretion protein GspI
i02_3384	-	gspH	putative type II secretion protein GspH
i02_3385	-	gspG	putative type II secretion protein GspG
i02_3386	-	gspF	putative type II secretion protein GspF
i02_3387	-	gspE	putative type II secretion protein GspE
i02_3388	-	gspD	putative type II secretion protein GspD
i02_3389	-	gspC	putative type II secretion protein GspC
i02_3390	-	/	hypothetical protein
i02_3391	-	pppA	putative prepilin peptidase A
i02_3392	-	yghJ	putative lipoprotein AcfD-like precursor
i02_3393	-	/	hypothetical protein
i02_3394	-	/	conserved hypothetical protein

Clone D	CFT 073	Gene	Product
i02_3395	c3704	yghK	glycolate transporter
i02_3396	c3705	glcB	malate synthase G
i02_3397	c3706	glcG	hypothetical protein
i02_3398		pir	glycolate oxidase iron-sulfur subunit
i02_3399		glcE	glycolate oxidase FAD binding subunit
i02_3400	c3709	glcD	glycolate oxidase subunit GlcD
i02_3401	c3710	glcC	DNA-binding transcriptional regulator GlcC
i02_3402	c3711	yghO	hypothetical protein
i02_3403	c3712	/	acyl-CoA synthetase
i02_3404	c3713	/	hypothetical protein
i02_3405	c3714	/	hypothetical protein
i02_3406	c3715	/	hypothetical protein
i02_3407	c3716	/	hypothetical protein
i02_3408	c3717	/	hypothetical protein
i02_3409	c3718	/	hypothetical protein
i02_3410	c3719	/	hypothetical protein
i02_3411	c3720	yghQ	hypothetical protein
i02_3412	c3721	yghR	ATP-binding protein yghR
i02_3413	c3722	yghS	ATP-binding protein yghS
i02_3414	c3723	yghT	ATP-binding protein yghT
i02_3415	c3724	pitB	inorganic phosphate transporter
i02_3416	c3725	gsp	bifunctional glutathionylspermidine
i02_3417	c3726	yghU	putative glutathione S-transferase YghU
i02_3418	c3727	hybG	hydrogenase 2 accessory protein HypG
i02_3419	c3728	hypA	hydrogenase nickel incorporation protein HybF
i02_3420	c3729	hybE	hydrogenase 2-specific chaperone
i02_3421	c3730	hybD	hydrogenase 2 maturation endopeptidase
i02_3422	c3731	hybC	hydrogenase 2 large subunit
i02_3423	c3732	hybB	putative hydrogenase 2 b cytochrome subunit
i02_3424	c3733	hybA	hydrogenase 2 protein HybA
i02_3426		/	hypothetical protein
i02_3425	c3734	/	hydrogenase 2 small subunit
i02_3427	c3735	yghW	hypothetical protein
i02_3428	c3736	/	hypothetical protein
i02_3429	c3737	yghZ	aldo-keto reductase
i02_3430	c3738	yqhA	hypothetical protein
i02_3431	c3739	yghA	oxidoreductase
i02_3432	c3740	exbD	biopolymer transport protein ExbD
i02_3433	c3741	exbB	biopolymer transport protein ExbB
i02_3434	c3742	metC	cystathionine beta-lyase
i02_3435	c3743	yghB	hypothetical protein
i02_3437	c3745	yqhD	oxidoreductase yqhD
i02_3436	c3744	yqhC	putative transcriptional regulator YqhC
i02_3438	c3746	dkgA	2,5-diketo-D-gluconate reductase A
i02_3439	c3747	yqhG	hypothetical protein
i02_3440	c3748	yqhH	putative outer membrane lipoprotein
i02_3441	c3749	/	hypothetical protein
i02_3442	c3750	/	putative regulator
i02_3443	c3751	/	oxidoreductase ydfI
i02_3444	c3752	/	hypothetical protein
i02_3445	c3753	/	ureidoglycolate dehydrogenase
i02_3446	c3754	/	putative c4-dicarboxylate transport system

Clone D	CFT 073	Gene	Product
i02_3447	c3755	/	hypothetical protein
i02_3448	c3756	ygiK	c4-dicarboxylate permease
i02_3449	c3757	sufI	repressor protein for FtsI
i02_3450	c3758	plsC	1-acyl-sn-glycerol-3-phosphate acyltransferase
i02_3451	c3759	/	hypothetical protein
i02_3452	c3760	parC	DNA topoisomerase IV subunit A
i02_3453	c3761	/	putative binding protein
i02_3454	c3762	ygiV	hypothetical protein
i02_3455	c3763	ygiW	hypothetical protein
i02_3456	c3764	ygiX	DNA-binding transcriptional regulator QseB
i02_3457	c3765	ygiY	sensor protein QseC
i02_3458		/	hypothetical protein
i02_3459	c3766	/	hypothetical protein
i02_3460	c3767	/	hypothetical protein
i02_3461	c3768	mdaB	modulator of drug activity B
i02_3462	c3769	ygiN	hypothetical protein
i02_3463	c3770	/	hypothetical protein
i02_3464	c3771	/	iron ABC transporter substrate-binding protein
i02_3465	c3772	/	iron ABC transporter permease
i02_3466	c3773	/	iron ABC transporter permease
i02_3467	c3774	/	ferric enterobactin transport ATP-binding
i02_3468		/	hypothetical protein
i02_3469	c3775	/	putative iron compound receptor
i02_3470	c3776	parE	DNA topoisomerase IV subunit B
i02_3471	c3777	yqiA	esterase YqiA
i02_3472	c3778	icc	cyclic 3',5'-adenosine monophosphate
i02_3473	c3779	yqiB	hypothetical protein
i02_3474	c3780	nudF	ADP-ribose pyrophosphatase NudF
i02_3475	c3781	tolC	outer membrane channel protein
i02_3476	c3782	ygiA	hypothetical protein
i02_3477	c3783	ygiB	hypothetical protein
i02_3478	c3784	ygiC	hypothetical protein
i02_3479	c3785	asst	arylsulfate sulfotransferase
i02_3480	c3786	/	putative disulfide isomerase
i02_3481	c3787	/	putative disulfide oxidoreductase
i02_3482	c3788	ygiD	hypothetical protein
i02_3483	c3789	ygiE	zinc transporter ZupT
i02_3484	c3790	/	hypothetical protein
i02_3485	c3791	ygiL	putative fimbrial protein
i02_3486	c3792	yqiG	outer membrane usher protein yqiG precursor
i02_3487	c3793	yqiH	fimbrial chaperone yqiH precursor
i02_3488	c3794	/	hypothetical protein
i02_3489	c3795	ribB	3,4-dihydroxy-2-butanone 4-phosphate synthase
i02_3490	c3796	yqiC	hypothetical protein
i02_3491	c3797	glgS	glycogen synthesis protein GlgS
i02_3492	c3798	yqiJ	hypothetical protein
i02_3493	c3799	yqiK	hypothetical protein
i02_3494	c3800	/	bifunctional heptose 7-phosphate kinase/heptose
i02_3495	c3801	glnE	bifunctional glutamine-synthetase
i02_3496	c3802	ygiF	hypothetical protein
i02_3497	c3803	/	putative transposase
i02_3498	c3804	ydcM	hypothetical protein

Clone D	CFT 073	Gene	Product
i02_3499		/	hypothetical protein
i02_3500	c3805	ygiM	putative signal transduction protein
i02_3501	c3806	cca	multifunctional tRNA nucleotidyl
i02_3502	c3807	uppP	undecaprenyl pyrophosphate phosphatase
i02_3503	c3808	folB	bifunctional dihydroneopterin
i02_3504	c3809	ygiH	putative glycerol-3-phosphate acyltransferase
i02_3506	c3811	/	hypothetical protein
i02_3505	c3810	ygiP	transcriptional activator TtdR
i02_3507	c3812	ttdA	tartrate dehydratase subunit alpha
i02_3508	c3813	ttdB	L(+)-tartrate dehydratase subunit beta
i02_3509	c3814	ygjE	putative tartrate carrier/transporter
i02_3510	c3815	ygjD	putative DNA-binding/iron metalloprotein
i02_3511	c3816	rpsU	30S ribosomal protein S21
i02_3512	c3817	dnaG	DNA primase
i02_3513	c3818	/	hypothetical protein
i02_3514	c3819	/	hypothetical protein
i02_3514	c3821	rpoD	RpoD protein
i02_3515	c3820	/	hypothetical protein
i02_3516	c3822	ygjF	G/U mismatch-specific DNA glycosylase
i02_3517	c3823	yqjH	hypothetical protein
i02_3518	c3824	yqjI	hypothetical protein
i02_3519	c3825	aer	aerotaxis receptor
	c3826	/	hypothetical protein
	c3827	/	hypothetical protein
i02_3520	c3828	ygjG	putrescine--2-oxoglutarate aminotransferase
i02_3521	c3829	/	hypothetical protein
	c3830	ygjH	hypothetical protein
i02_3522	c3831	/	hypothetical protein
i02_3523	c3832	ebgR	DNA-binding transcriptional repressor EbgR
i02_3524	c3833	ebgA	cryptic beta-D-galactosidase subunit alpha
i02_3525	c3834	ebgC	cryptic beta-D-galactosidase subunit beta
	c3835	/	hypothetical protein
i02_3527	c3836	ygjI	putative transporter YgjI
i02_3528	c3837	ygjJ	hypothetical protein
i02_3529	c3838	ygjK	putative glycosyl hydrolase
i02_3530	c3839	ygjL	2,4-dienoyl-CoA reductase
i02_3531	c3840	/	hypothetical protein
i02_3532	c3841	/	hypothetical protein
i02_3533	c3842	ygjO	putative ribosomal RNA small subunit
i02_3534	c3843	ygjP	hypothetical protein
i02_3535	c3844	ygjQ	hypothetical protein
i02_3536	c3845	ygjR	oxidoreductase ygjR
i02_3537	c3846	ygjT	hypothetical protein
i02_3538	c3847	ygjU	serine/threonine transporter SstT
i02_3539	c3848	ygjV	hypothetical protein
i02_3540	c3849	uxaA	Altronate hydrolase
i02_3541	c3850	uxaC	glucuronate isomerase
i02_3542	c3851	exuT	hexuronate transporter
i02_3543	c3852	exuR	DNA-binding transcriptional repressor ExuR
i02_3544	c3853	yqjA	hypothetical protein
i02_3545	c3854	yqjB	hypothetical protein
i02_3546	c3855	yqjC	hypothetical protein

Clone D	CFT 073	Gene	Product
i02_3547	c3856	yqjD	hypothetical protein
i02_3548	c3857	yqjE	hypothetical protein
i02_3549	c3858	yqjK	hypothetical protein
i02_3550	c3859	yqjF	hypothetical protein
i02_3551		yqjG	putative glutathione S-transferase
i02_3552	c3862	yhaH	hypothetical protein
i02_3553	c3863	yhaJ	putative transcriptional regulator YhaJ
i02_3554	c3864	yhaK	hypothetical protein
	c3865	/	hypothetical protein
i02_3555	c3866	yhaL	hypothetical protein
i02_3556	c3867	/	hypothetical protein
i02_3557		/	putative amino acid permease
i02_3558	c3870	/	L-serine dehydratase 1
i02_3559	c3871	yhaR	hypothetical protein
i02_3560	c3872	tdcE	keto-acid formate acetyltransferase
i02_3561	c3873	tdcD	propionate/acetate kinase
i02_3562	c3874	tdcC	threonine/serine transporter TdcC
i02_3563	c3875	tdcB	threonine dehydratase
i02_3564	c3876	tdcA	DNA-binding transcriptional activator TdcA
i02_3565	c3877	tdcR	DNA-binding transcriptional activator TdcR
i02_3566	c3878	/	hypothetical protein
i02_3567		/	hypothetical protein
i02_3568	c3879	yhaD	glycerate kinase I
i02_3569	c3880	garR	tartronate semialdehyde reductase
i02_3570	c3881	yhaF	alpha-dehydro-beta-deoxy-D-glucarate aldolase
i02_3571	c3882	yhaU	galactarate transporter
i02_3572		/	hypothetical protein
i02_3573	c3883	yhaG	D-galactarate dehydratase
i02_3574	c3884	sohA	putative regulator PrlF
i02_3575	c3885	yhaV	hypothetical protein
i02_3576	c3886	agaR	DNA-binding transcriptional regulator AgaR
i02_3577	c3887	agaZ	putative tagatose 6-phosphate kinase agaZ
i02_3578	c3888	agaV	N-acetylgalactosamine-specific PTS system
i02_3579	c3889	/	putative phosphotransferase system enzyme
i02_3580	c3890	/	PTS system, N-acetylgalactosamine-specific IID
i02_3581	c3891	/	putative phosphotransferase system enzyme
i02_3582	c3892	/	putative N-acetylgalctosamine-6-phosphate
i02_3583	c3893	agaS	putative tagatose-6-phosphate aldose/ketose isomerase
i02_3584	c3894	kbaY	tagatose-bisphosphate aldolase
i02_3585	c3895	agaB	N-acetylgalactosamine-specific PTS system
i02_3586	c3896	agaC	N-acetylgalactosamine-specific PTS system
i02_3587	c3897	agaD	N-acetylgalactosamine-specific PTS system
i02_3588	c3898	agaI	galactosamine-6-phosphate isomerase
i02_3590	c3900	yraM	hypothetical protein
i02_3589	c3899	yraL	hypothetical protein
i02_3591	c3901	yraN	hypothetical protein
	c3902	/	hypothetical protein
i02_3593	c3903	yraO	DnaA initiator-associating protein DiaA
i02_3594	c3904	yraP	hypothetical protein
i02_3595	c3905	yraQ	hypothetical protein
i02_3596	c3906	yraR	hypothetical protein

Clone D	CFT 073	Gene	Product
i02_3597	c3907	yhbP	hypothetical protein
i02_3598	c3908	yhbQ	GIY-YIG nuclease superfamily protein
i02_3599	c3909	yhbS	acetyltransferase YhbS
i02_3600	c3910	yhbT	hypothetical protein
i02_3601	c3911	yhbU	putative protease yhbU precursor
i02_3602	c3912	yhbV	hypothetical protein
i02_3603	c3913	yhbW	hypothetical protein
i02_3604	c3914	mtr	tryptophan permease
i02_3605	c3915	/	hypothetical protein
i02_3606	c3916	deaD	ATP-dependent RNA helicase DeaD
	c3917	/	hypothetical protein
i02_3607	c3918	yhbM	lipoprotein NlpI
	c3919	/	hypothetical protein
i02_3608	c3920	pnp	polynucleotide phosphorylase/polyadenylase
i02_3609	c3921	rpsO	30S ribosomal protein S15
i02_3610	c3922	truB	tRNA pseudouridine synthase B
i02_3611	c3923	rbfA	ribosome-binding factor A
i02_3613	c3925	/	hypothetical protein
i02_3612	c3924	infB	translation initiation factor IF-2
i02_3614	c3926	nusA	transcription elongation factor NusA
i02_3615	c3927	yhbC	hypothetical protein
i02_3616	c3929	argG	argininosuccinate synthase
i02_3617	c3930	yhbX	Outer-membrane protein yhbX precursor
i02_3618	c3931	secG	preprotein translocase subunit SecG
i02_3619	c3932	glmM	phosphoglucosamine mutase
i02_3620	c3933	folP	dihydropteroate synthase
i02_3622	c3935	/	hypothetical protein
i02_3621		hflB	ATP-dependent metalloprotease
i02_3623	c3936	rrmJ	23S rRNA methyltransferase J
i02_3624	c3937	yhbY	RNA-binding protein YhbY
i02_3625	c3938	greA	transcription elongation factor GreA
i02_3626	c3939	dacB	D-alanyl-D-alanine
i02_3627	c3940	obgE	GTPase ObgE
i02_3628	c3941	yhbE	putative transport protein YhbE
	c3943	/	hypothetical protein
i02_3630	c3944	/	hypothetical protein
i02_3629	c3942	rpmA	50S ribosomal protein L27
i02_3631	c3945	ispB	octaprenyl diphosphate synthase
i02_3632	c3946	nlp	DNA-binding transcriptional regulator Nlp
i02_3633	c3947	murA	UDP-N-acetylglucosamine
	c3949	/	hypothetical protein
i02_3634	c3948	yrbA	hypothetical protein
	c3951	/	hypothetical protein
i02_3635	c3950	yrbB	hypothetical protein
i02_3636	c3952	yrbC	hypothetical protein
i02_3637	c3953	yrbD	hypothetical protein
i02_3638	c3954	yrbE	hypothetical protein
i02_3639	c3955	yrbF	putative ABC transporter ATP-binding protein
i02_3640	c3956	yrbG	putative calcium/sodium:proton antiporter
i02_3641	c3957	/	D-arabinose 5-phosphate isomerase
i02_3642	c3958	yrbI	3-deoxy-D-manno-octulosonate 8-phosphate phosphatase monomer

Clone D	CFT 073	Gene	Product
i02_3643	c3959	yrbK	hypothetical protein
i02_3644	c3960	yhbN	lipopolysaccharide transport periplasmic protein
i02_3645	c3961	yhbG	putative ABC transporter ATP-binding protein
i02_3646	c3962	rpoN	RNA polymerase factor sigma-54
i02_3647	c3963	yhbH	putative sigma(54) modulation protein
i02_3648	c3964	ptsN	PTS IIA-like nitrogen-regulatory protein PtsN
i02_3649	c3965	yhbJ	hypothetical protein
i02_3650	c3966	ptsO	phosphohistidinoprotein-hexose
i02_3651	c3967	yrbL	hypothetical protein
i02_3652	c3968	mtgA	monofunctional biosynthetic peptidoglycan
i02_3653	c3969	yhbL	isoprenoid biosynthesis protein
i02_3654	c3970	arcB	aerobic respiration control sensor protein ArcB
i02_3655	c3971	yhcC	hypothetical protein
i02_3656	c3972	/	hypothetical protein
i02_3657	c3973	gltB	glutamate synthase subunit alpha
i02_3658	c3974	gltD	glutamate synthase subunit beta
i02_3659	c3975	yhcH	hypothetical protein
i02_3660	c3976	/	N-acetylmannosamine kinase
i02_3661	c3977	yhcJ	N-acetylmannosamine-6-phosphate 2-epimerase
i02_3662	c3978	nanT	putative sialic acid transporter
i02_3663	c3979	nanA	N-acetylneuraminate lyase
i02_3664	c3980	yhcK	transcriptional regulator NanR
i02_3665	c3981	sspB	ClpXP protease specificity-enhancing factor
i02_3667	c3983	/	hypothetical protein
i02_3666	c3982	sspA	stringent starvation protein A
i02_3668	c3984	rpsI	30S ribosomal protein S9
i02_3669	c3985	rplM	50S ribosomal protein L13
i02_3670	c3986	yhcM	hypothetical protein
i02_3671	c3987	yhcB	cytochrome d ubiquinol oxidase subunit III
	c3988	/	hypothetical protein
i02_3672	c3989	degQ	serine endoprotease
i02_3673	c3990	degS	serine endoprotease
i02_3674	c3991	mdh	malate dehydrogenase
i02_3675	c3992	argR	arginine repressor
i02_3676	c3993	yhcN	hypothetical protein
i02_3677	c3994	yhcO	hypothetical protein
i02_3678	c3995	yhcP	p-hydroxybenzoic acid efflux subunit AaeB
i02_3679	c3996	yhcQ	p-hydroxybenzoic acid efflux subunit AaeA
i02_3680	c3997	yhcR	hypothetical protein
i02_3681	c3998	yhcS	putative DNA-binding transcriptional regulator
i02_3682	c3999	tldD	protease TldD
i02_3683	c4000	yhdP	hypothetical protein
i02_3684	c4001	cafA	ribonuclease G
i02_3685	c4002	maf	Maf-like protein
i02_3686	c4003	mreD	rod shape-determining protein MreD
i02_3687	c4005	mreC	rod shape-determining protein MreC
i02_3688	c4006	mreB	rod shape-determining protein MreB
i02_3689	c4007	/	hypothetical protein
i02_3690	c4008	yhdA	regulatory protein CsrD
i02_3691	c4009	yhdH	hypothetical protein
i02_3692	c4010	/	hypothetical protein
i02_3693	c4011	accB	acetyl-CoA carboxylase biotin carboxyl carrier

Clone D	CFT 073	Gene	Product
i02_3694	c4012	accC	acetyl-CoA carboxylase biotin carboxylase
i02_3695	c4013	/	hypothetical protein
i02_3696	c4014	/	hypothetical protein
i02_3697	c4015	/	ribose ABC transporter permease
i02_3698	c4016	/	ribose ABC transporter ATP-binding protein
i02_3699	c4017	/	putative ribose ABC transporter
i02_3701	c4019	/	hypothetical protein
i02_3700	c4018	gatY	tagatose-bisphosphate aldolase
i02_3702	c4020	/	hypothetical protein
i02_3703	c4021	/	hypothetical protein
i02_3704	c4022	yhdT	hypothetical protein
i02_3705	c4023	panF	sodium/panthothenate symporter
i02_3706	c4024	prmA	ribosomal protein L11 methyltransferase
i02_3707	c4025	/	hypothetical protein
i02_3708	c4026	yhdG	tRNA-dihydrouridine synthase B
i02_3709	c4027	fis	DNA-binding protein Fis
i02_3710	c4028	yhdJ	putative methyltransferase
i02_3711	c4029	yhdU	hypothetical protein
i02_3712	c4030	envR	DNA-binding transcriptional regulator EnvR
i02_3713	c4031	acrE	acriflavin resistance protein E
i02_3714	c4032	acrF	acriflavin resistance protein F
i02_3715	c4033	yhdV	hypothetical protein
i02_3716		/	hypothetical protein
i02_3717	c4034	yhdW	putative amino-acid ABC transporter
i02_3718	c4035	yhdX	amino-acid ABC transporter permease protein
i02_3719	c4036	yhdY	amino-acid ABC transporter permease protein
i02_3720	c4037	yhdZ	amino-acid ABC transporter ATP-binding protein
i02_3722	c4040	yrdA	hypothetical protein
i02_3723	c4041	yrdB	hypothetical protein
i02_3724	c4042	aroE	shikimate 5-dehydrogenase
i02_3725	c4043	yrdC	putative ribosome maturation factor
i02_3726	c4044	yrdD	hypothetical protein
i02_3727	c4045	smg	hypothetical protein
i02_3728	c4046	smf	DNA protecting protein DprA
i02_3729	c4047	def	peptide deformylase
i02_3730	c4048	fmt	methionyl-tRNA formyltransferase
i02_3731	c4049	sun	16S rRNA methyltransferase B
i02_3732	c4050	trkA	potassium transporter
i02_3733	c4051	mscL	large-conductance mechanosensitive channel
i02_3734	c4052	yhdL	putative regulator
i02_3735	c4053	zntR	zinc-responsive transcriptional regulator
i02_3736	c4054	yhdN	hypothetical protein
i02_3737	c4055	rplQ	50S ribosomal protein L17
i02_3738	c4056	rpoA	DNA-directed RNA polymerase subunit alpha
i02_3739	c4057	rpsD	30S ribosomal protein S4
	c4059	/	hypothetical protein
i02_3740	c4058	rpsK	30S ribosomal protein S11
i02_3741	c4060	rpsM	30S ribosomal protein S13
i02_3742		/	predicted protein
i02_3743	c4061	secY	preprotein translocase subunit SecY
i02_3744	c4062	rplO	50S ribosomal protein L15
	c4064	/	hypothetical protein

Clone D	CFT 073	Gene	Product
i02_3745	c4063	rpmD	50S ribosomal protein L30
i02_3746	c4065	rpsE	30S ribosomal protein S5
	c4067	/	hypothetical protein
i02_3747	c4066	rplR	50S ribosomal protein L18
	c4069	/	hypothetical protein
i02_3748	c4068	rplF	50S ribosomal protein L6
i02_3750	c4070	rpsH	30S ribosomal protein S8
i02_3751	c4071	rpsN	30S ribosomal protein S14
i02_3752	c4072	rplE	50S ribosomal protein L5
	c4074	/	hypothetical protein
i02_3753	c4073	rplX	50S ribosomal protein L24
i02_3755	c4075	rplN	50S ribosomal protein L14
i02_3756	c4076	rpsQ	30S ribosomal protein S17
	c4078	/	hypothetical protein
i02_3757	c4077	rpmC	50S ribosomal protein L29
i02_3758	c4079	rplP	50S ribosomal protein L16
i02_3759	c4080	rpsC	30S ribosomal protein S3
	c4081	/	hypothetical protein
i02_3760	c4082	rplV	50S ribosomal protein L22
	c4084	/	hypothetical protein
i02_3761	c4083	rpsS	30S ribosomal protein S19
i02_3762	c4085	rplB	50S ribosomal protein L2
	c4086	/	hypothetical protein
	c4088	/	hypothetical protein
i02_3763	c4087	rplW	50S ribosomal protein L23
	c4090	/	hypothetical protein
i02_3764	c4089	rplD	50S ribosomal protein L4
i02_3765	c4091	rplC	50S ribosomal protein L3
i02_3766	c4092	rpsJ	30S ribosomal protein S10
i02_3767	c4093	pinO	chromosome replication protein PioO
i02_3768	c4094	yheD	general secretion pathway protein A
i02_3769	c4095	yheE	general secretion pathway protein C
i02_3770	c4096	yheF	general secretion pathway protein D precursor
i02_3771	c4097	yheG	general secretion pathway protein E
i02_3772	c4098	hofF	putative general secretion pathway protein F
i02_3773	c4099	hofG	putative general secretion pathway protein G
i02_3774	c4100	hofH	putative general secretion pathway protein H
i02_3775	c4101	yheH	general secretion pathway protein I precursor
i02_3776	c4102	yheI	general secretion pathway protein J precursor
i02_3777	c4103	yheJ	general secretion pathway protein K
i02_3778	c4104	yheK	general secretion pathway protein L
i02_3779	c4105	pshM	putative general secretion pathway protein M
i02_3780	c4106	hofD	leader peptidase
i02_3781	c4107	bfr	bacterioferritin
i02_3782	c4108	yheA	bacterioferritin-associated ferredoxin
i02_3783		/	hypothetical protein
i02_3784	c4109	chiA	secreted endochitinase
i02_3785	c4110	/	hypothetical protein
i02_3785a	c4111	tuf	elongation factor Tu
i02_3786	c4112	fusA	elongation factor G
	c4113	/	hypothetical protein
i02_3787	c4114	rpsG	30S ribosomal protein S7

Clone D	CFT 073	Gene	Product
	c4115	/	hypothetical protein
i02_3788	c4116	rpsL	30S ribosomal protein S12
i02_3789	c4117	yheL	sulfur transfer complex subunit TusB
i02_3790	c4118	yheM	sulfur relay protein TusC
i02_3791	c4119	yheN	sulfur transfer complex subunit TusD
i02_3792	c4120	yheO	hypothetical protein
i02_3793	c4121	fkpA	FKBP-type peptidyl-prolyl cis-trans isomerase
i02_3794	c4122	slyX	hypothetical protein
i02_3795	c4123	slyD	FKBP-type peptidyl-prolyl cis-trans isomerase
i02_3796	c4124	/	hypothetical protein
i02_3797	c4125	kefB	glutathione-regulated potassium-efflux system
i02_3798	c4126	yheR	glutathione-regulated potassium-efflux system
i02_3799	c4127	yheS	putative ABC transporter ATP-binding protein
i02_3800	c4128	yheT	putative hydrolase
i02_3801	c4129	yheU	hypothetical protein
i02_3802	c4130	prkB	phosphoribulokinase
i02_3803	c4131	yhfA	hypothetical protein
i02_3804	c4132	crp	cAMP-regulatory protein
i02_3805	c4133	yhfK	hypothetical protein
i02_3806	c4134	argD	bifunctional
i02_3807	c4135	pabA	para-aminobenzoate synthase component II
i02_3808	c4136	fic	cell filamentation protein Fic
i02_3809	c4137	yhfG	hypothetical protein
i02_3810	c4138	ppiA	peptidyl-prolyl cis-trans isomerase A
i02_3811	c4139	yhfC	hypothetical protein
i02_3813	c4141	nirB	nitrite reductase [NAD(P)H] large subunit
i02_3812	c4140	/	hypothetical protein
i02_3814	c4142	nirD	nitrite reductase small subunit
i02_3815	c4143	nirC	nitrite transporter NirC
i02_3816	c4144	cysG	siroheme synthase
i02_3817	c4145	yhfL	hypothetical protein
i02_3818	c4146	yhfS	hypothetical protein
i02_3819	c4147	yhfT	hypothetical protein
i02_3820	c4148	yhfU	hypothetical protein
i02_3821	c4149	yhfV	putative hydrolase
i02_3822	c4150	yhfW	putative mutase
i02_3823	c4151	yhfX	hypothetical protein
i02_3824	c4152	yhfY	hypothetical protein
i02_3825	c4153	yhfZ	hypothetical protein
i02_3826	c4154	trpS	tryptophanyl-tRNA synthetase
i02_3827	c4155	gph	phosphoglycolate phosphatase
i02_3828	c4156	rpe	ribulose-phosphate 3-epimerase
i02_3829	c4157	dam	DNA adenine methylase
i02_3830	c4158	damX	hypothetical protein
i02_3831	c4159	aroB	3-dehydroquinate synthase
i02_3832	c4160	aroK	shikimate kinase I
i02_3833	c4161	hofQ	outer membrane porin HofQ
i02_3834	c4162	yrfA	hypothetical protein
i02_3835	c4163	yrfB	hypothetical protein
i02_3836	c4164	yrfC	hypothetical protein
i02_3837	c4165	yrfD	hypothetical protein
i02_3838	c4166	mrcA	peptidoglycan synthetase

Clone D	CFT 073	Gene	Product
i02_3839	c4167	nudE	ADP-ribose diphosphatase NudE
i02_3840	c4168	/	hypothetical protein
i02_3841	c4169	yrfF	putative membrane protein igaA-like protein
i02_3842	c4170	yrfG	hypothetical protein
i02_3843	c4171	yrfH	ribosome-associated heat shock protein Hsp15
i02_3844	c4172	hslO	Hsp33-like chaperonin
i02_3845	c4173	yhgE	hypothetical protein
	c4174	/	hypothetical protein
i02_3846	c4175	/	hypothetical protein
i02_3847	c4176	pckA	phosphoenolpyruvate carboxykinase
i02_3848	c4177	/	hypothetical protein
i02_3849	c4178	/	hypothetical protein
i02_3850	c4179	/	hypothetical protein
i02_3851	c4180	envZ	osmolarity sensor protein
i02_3852	c4181	ompR	osmolarity response regulator
i02_3854	c4183	greB	transcription elongation factor GreB
i02_3853	c4182	/	hypothetical protein
i02_3855	c4184	yhgF	hypothetical protein
i02_3856	c4185	feoA	ferrous iron transport protein A
i02_3857	c4186	feoB	ferrous iron transport protein B
i02_3858	c4187	yhgG	hypothetical protein
i02_3859	c4188	yhgA	hypothetical protein
i02_3861	c4190	yhgH	gluconate periplasmic binding protein
i02_3860	c4189	bioH	carboxylesterase BioH
i02_3862	c4191	yhgI	putative DNA uptake protein
i02_3863	c4192	gntT	high-affinity gluconate transporter
i02_3864	c4193	malQ	4-alpha-glucanotransferase
i02_3865	c4194	malP	maltodextrin phosphorylase
i02_3866	c4195	/	hypothetical protein
i02_3867	c4196	malT	transcriptional regulator MalT
i02_3868	c4197	/	RNA 3'-terminal-phosphate cyclase
i02_3869	c4198	rtcB	protein rtcB
i02_3870	c4199	rtcR	transcriptional regulatory protein rtcR
i02_3872	c4200	glpR	DNA-binding transcriptional repressor GlpR
i02_3873	c4201	glpG	intramembrane serine protease GlpG
i02_3874	c4202	glpE	thiosulfate sulfurtransferase
i02_3875	c4203	glpD	glycerol-3-phosphate dehydrogenase
i02_3876	c4204	/	hypothetical protein
i02_3877	c4205	/	hypothetical protein
i02_3878	c4206	/	hypothetical protein
i02_3879	c4207	/	putative fimbrial adhesin precursor
i02_3880	c4208	/	putative fimbrial chaperone precursor
i02_3881	c4209	/	putative minor fimbrial subunit precursor
	c4211	/	hypothetical protein
i02_3882	c4210	/	putative minor fimbrial subunit precursor
i02_3883	c4212	ycbS	outer membrane usher protein ycbS precursor
i02_3884	c4213	/	chaperone protein fimC precursor
i02_3885	c4214	/	putative major fimbrial subunit precursor
i02_3886	c4215	glgP	glycogen phosphorylase
i02_3887	c4216	glgA	glycogen synthase
i02_3888	c4217	glgC	glucose-1-phosphate adenylyltransferase
i02_3889	c4218	glgX	glycogen debranching enzyme

Clone D	CFT 073	Gene	Product
i02_3890	c4219	glgB	glycogen branching enzyme
i02_3891	c4220	asd	aspartate-semialdehyde dehydrogenase
i02_3892	c4221	yhgN	putative dITP- and XTP- hydrolase
i02_3893	c4222	/	putative DNA processing protein
i02_3894	c4223	/	hypothetical protein
i02_3895	c4224	gntU	low affinity gluconate transporter
	c4226	/	hypothetical protein
i02_3896	c4225	gntK	gluconate kinase 1
i02_3897	c4227	gntR	gluconate utilization system GNT-I
i02_3898	c4228	yhhW	hypothetical protein
i02_3900	c4230	/	hypothetical protein
i02_3899	c4229	yhhX	putative dehydrogenase
i02_3901		/	hypothetical protein
i02_3902		yhhY	putative acetyltransferase YhhY
i02_3903	c4233	yhhZ	hypothetical protein
i02_3904	c4234	yrhA	hypothetical protein
i02_3905	c4235	yrhB	hypothetical protein
i02_3906	c4236	ggt	gamma-glutamyltranspeptidase
i02_3907	c4237	yhhA	hypothetical protein
i02_3908	c4238	ugpQ	cytoplasmic glycerophosphodiester
i02_3909	c4239	ugpC	glycerol-3-phosphate transporter ATP-binding component
i02_3910	c4240	ugpE	glycerol-3-phosphate transporter membrane component
i02_3911	c4241	ugpA	glycerol-3-phosphate transporter permease component
i02_3912	c4242	ugpB	glycerol-3-phosphate transporter periplasmic component
i02_3913	c4243	/	hypothetical protein
i02_3914	c4244	livF	leucine/isoleucine/valine transporter
i02_3915	c4245	livG	leucine/isoleucine/valine transporter
i02_3916	c4246	livM	leucine/isoleucine/valine transporter permease
i02_3917	c4247	livH	branched-chain amino acid transporter permease
i02_3919	c4249	/	hypothetical protein
i02_3918	c4248	livK	leucine-specific binding protein precursor
i02_3920	c4250	/	hypothetical protein
i02_3921	c4251	yhhK	hypothetical protein
i02_3922	c4252	/	hypothetical protein
i02_3923	c4253	livJ	Leu/Ile/Val-binding protein precursor
i02_3924	c4254	rpoH	RNA polymerase factor sigma-32
i02_3925	c4255	ftsX	cell division protein FtsX
i02_3926	c4256	ftsE	cell division protein FtsE
i02_3927	c4257	ftsY	cell division protein FtsY
i02_3928	c4258	rsmD	16S rRNA m(2)G966-methyltransferase
i02_3929	c4259	yhhL	hypothetical protein
i02_3930	c4260	yhhM	hypothetical protein
i02_3931	c4261	yhhN	hypothetical protein
i02_3932	c4262	zntA	zinc/cadmium/mercury/lead-transporting ATPase
i02_3933	c4263	yhhP	sulfur transfer protein SirA
i02_3934	c4264	yhhQ	hypothetical protein
i02_3935	c4265	/	hypothetical protein
i02_3936	c4266	yhhS	major facilitator superfamily transporter

Clone D	CFT 073	Gene	Product
i02_3937	c4267	yhhT	hypothetical protein
i02_3938	c4268	yhhU	holo-(acyl carrier protein) synthase 2
i02_3939	c4269	nikA	nickel-binding periplasmic protein precursor
i02_3940	c4270	nikB	nickel transporter permease NikB
i02_3941	c4271	nikC	nickel transporter permease NikC
i02_3942	c4272	nikD	nickel transporter ATP-binding protein NikD
i02_3943	c4273	nikE	nickel transporter ATP-binding protein NikE
i02_3944	c4274	yhhG	nickel responsive regulator
	c4275	/	hypothetical protein
i02_3946	c4276	/	putative regulator
i02_3947	c4277	/	putative phosphotransferase system enzyme
i02_3948	c4278	/	putative phosphotransferase system enzyme
i02_3949	c4279	/	PTS system, galactitol-specific IIC component
i02_3950	c4280	/	putative xylulose kinase
i02_3950	c4282	/	hypothetical protein
i02_3952	c4283	/	putative phosphocarrier protein
i02_3951	c4281	/	hypothetical protein
i02_3953	c4284	gatY	putative fructose-1,6-bisphosphate aldolase
i02_3954	c4285	yhhJ	hypothetical protein
i02_3955	c4286	yhiH	ABC transporter ATP-binding protein
i02_3956	c4287	yhiI	hypothetical protein
i02_3957	c4288	/	hypothetical protein
i02_3958	c4289	yhiM	hypothetical protein
i02_3959	c4290	yhiN	hypothetical protein
i02_3960	c4291	pitA	inorganic phosphate transporter
i02_3961	c4292	yhiO	universal stress protein UspB
i02_3962	c4293	uspA	universal stress protein A
i02_3964	c4295	yhiP	inner membrane transporter YhiP
i02_3963	c4294	/	hypothetical protein
i02_3965	c4296	yhiQ	putative methyltransferase
i02_3966	c4297	prlC	oligopeptidase A
i02_3967	c4298	yhiR	hypothetical protein
i02_3968	c4299	gor	glutathione reductase
i02_3969	c4300	/	hypothetical protein
i02_3970	c4301	arsC	arsenate reductase
i02_3971	c4302	/	hypothetical protein
i02_3972	c4303	/	hypothetical protein
i02_3973	c4304	slp	Outer membrane protein Slp precursor
i02_3974	c4305	/	hypothetical protein
i02_3975	c4306	yhiF	putative transcriptional regulator YhiF
i02_3976	c4307	chuS	putative heme/hemoglobin transport protein
i02_3978	c4309	/	hypothetical protein
i02_3977	c4308	chuA	Outer membrane heme/hemoglobin receptor
i02_3979	c4310	/	hypothetical protein
i02_3980	c4311	/	hypothetical protein
	c4312	/	hypothetical protein
i02_3981	c4313	chuT	putative periplasmic binding protein
i02_3982	c4314	chuW	coproporphyrinogen III oxidase
i02_3983	c4315	chuX	hypothetical protein
i02_3984	c4316	chuY	hypothetical protein
i02_3985	c4317	chuU	iron ABC transporter permease
i02_3986	c4318	hmuV	hemin importer ATP-binding subunit

Clone D	CFT 073	Gene	Product
i02_3987	c4319	yhiD	putative Mg(2+) transport ATPase
i02_3988	c4320	hdeB	acid-resistance protein
i02_3989	c4321	hdeA	acid-resistance protein
i02_3990	c4322	hdeD	acid-resistance membrane protein
i02_3991	c4323	yhiE	hypothetical protein
i02_3992		/	hypothetical protein
i02_3993	c4324	yhiU	multidrug efflux system protein MdtE
i02_3994	c4325	yhiV	hypothetical protein
i02_3995	c4326	yhiW	putative transcriptional regulator YhiW
i02_3996	c4327	yhiX	DNA-binding transcriptional regulator GadX
i02_3997	c4328	gadA	glutamate decarboxylase alpha
i02_3998	c4329	yhjA	cytochrome C peroxidase
i02_3999	c4330	treF	trehalase
i02_4000	c4331	yhjB	putative transcriptional regulator YhjB
i02_4001		/	conserved hypothetical protein
i02_4002	c4332	yhjC	putative transcriptional regulator YhjC
i02_4003	c4333	yhjD	hypothetical protein
i02_4004	c4334	yhjE	metabolite transport protein
i02_4005	c4335	yhjG	hypothetical protein
i02_4007	c4337	kdgK	2-dehydro-3-deoxygluconokinase
i02_4006	c4336	yhjH	EAL domain-containing protein
i02_4008	c4339	yhjJ	hypothetical protein
i02_4009	c4340	dctA	C4-dicarboxylate transporter DctA
i02_4010	c4341	yhjK	putative phosphodiesterase
i02_4011	c4342	yhjL	cellulose synthase subunit BcsC
i02_4012	c4343	yhjM	endo-1,4-D-glucanase
i02_4013	c4344	yhjN	cellulose synthase regulator protein
i02_4014	c4345	bcsA	cellulose synthase catalytic subunit
i02_4015	c4346	yhjQ	cell division protein
i02_4016	c4347	yhjR	hypothetical protein
i02_4017	c4348	yhjS	hypothetical protein
i02_4018	c4349	yhjT	hypothetical protein
i02_4019	c4350	yhjU	hypothetical protein
i02_4020	c4351	/	hypothetical protein
i02_4021	c4352	/	hypothetical protein
i02_4023		/	hypothetical protein
i02_4024	c4353	/	hypothetical protein
i02_4025	c4354	yhjV	putative transport protein YhjV
i02_4026	c4355	dppF	dipeptide transporter ATP-binding subunit
i02_4027	c4356	dppD	dipeptide transporter ATP-binding subunit
i02_4028	c4357	dppC	dipeptide transporter
i02_4029	c4358	dppB	dipeptide transporter permease DppB
i02_4030	c4361	dppA	periplasmic dipeptide transport protein
i02_4031		/	Hypothetical protein c4363
i02_4032	c4364	yhjW	phosphoethanolamine transferase
i02_4033	c4365	yhjX	hypothetical protein
i02_4034	c4366	yhjY	hypothetical protein
i02_4035	c4367	tag	3-methyl-adenine DNA glycosylase I
i02_4036	c4368	yiaC	hypothetical protein
i02_4037	c4369	bisC	biotin sulfoxide reductase
i02_4038	c4370	yiaD	putative outer membrane lipoprotein
	c4371	/	hypothetical protein

Clone D	CFT 073	Gene	Product
i02_4039	c4372	yiaE	2-hydroxyacid dehydrogenase
i02_4040	c4373	yiaF	hypothetical protein
i02_4041	c4374	/	hypothetical protein
i02_4042	c4375	/	putative transcriptional regulator
	c4376	/	hypothetical protein
i02_4043	c4377	cspA	major cold shock protein
i02_4044		/	small toxic polypeptide
i02_4045	c4378	glyS	glycyl-tRNA synthetase subunit beta
i02_4046	c4379	glyQ	glycyl-tRNA synthetase subunit alpha
i02_4047	c4380	/	hypothetical protein
i02_4048	c4381	yiaH	hypothetical protein
i02_4049	c4382	yiaA	hypothetical protein
i02_4050	c4383	yiaB	hypothetical protein
i02_4051	c4384	xylB	xylulokinase
i02_4052	c4385	xylA	xylose isomerase
i02_4053	c4386	xylF	D-xylose transporter subunit XylF
i02_4054	c4387	xylG	xylose transporter ATP-binding subunit
i02_4055	c4388	xylH	xylose transport system permease protein xylH
i02_4056	c4389	xylR	xylose operon regulatory protein
i02_4057	c4390	bax	hypothetical protein
i02_4058	c4391	/	hypothetical protein
i02_4059	c4392	malS	periplasmic alpha-amylase precursor
i02_4060	c4393	avtA	valine--pyruvate transaminase
i02_4061	c4394	yiaI	putative electron transport protein ysaA
i02_4062	c4395	yiaJ	putative transcriptional regulator YiaJ
i02_4063	c4396	yiaK	2,3-diketo-L-gulonate reductase
i02_4064	c4397	yiaL	hypothetical protein
i02_4065	c4398	/	hypothetical protein
i02_4066	c4399	yiaM	2,3-diketo-L-gulonate TRAP transporter
i02_4067	c4400	yiaN	hypothetical protein
	c4401	/	hypothetical protein
i02_4068	c4402	yiaO	ABC transporter periplasmic-binding protein
i02_4069	c4403	lyxK	Cryptic L-xylulose kinase
i02_4070	c4404	sgbH	3-keto-L-gulonate-6-phosphate decarboxylase
i02_4071	c4405	sgbU	putative L-xylulose 5-phosphate 3-epimerase
i02_4072	c4406	sgbE	L-ribulose-5-phosphate 4-epimerase
	c4407	/	hypothetical protein
i02_4073	c4408	aldB	aldehyde dehydrogenase B
i02_4074	c4409	/	hypothetical protein
i02_4075	c4410	yiaY	putative alcohol dehydrogenase
i02_4076	c4411	selB	selenocysteinyl-tRNA-specific translation factor
i02_4077	c4412	selA	selenocysteine synthase
i02_4078	c4413	yibF	putative glutathione S-transferase
i02_4079	c4414	yibH	hypothetical protein
i02_4080	c4415	yibI	hypothetical protein
i02_4081	c4416	mtlA	PTS system, mannitol-specific IIABC component
i02_4082	c4417	mtlD	mannitol-1-phosphate 5-dehydrogenase
i02_4083	c4418	mtlR	mannitol repressor protein
i02_4084	c4419	/	hypothetical protein
	c4420	/	hypothetical protein
i02_4085	c4421	yibL	hypothetical protein
i02_4086	c4422	/	hypothetical protein

Clone D	CFT 073	Gene	Product
i02_4087	c4423	/	hypothetical protein
i02_4088	c4424	/	putative adhesin
i02_4089	c4425	lldP	L-lactate permease
i02_4090	c4426	lldR	DNA-binding transcriptional repressor LldR
i02_4091	c4427	lldD	L-lactate dehydrogenase
i02_4092	c4428	yibK	putative tRNA/rRNA methyltransferase YibK
i02_4093	c4429	cysE	serine acetyltransferase
i02_4095	c4431	/	hypothetical protein
i02_4094	c4430	gpsA	NAD(P)H-dependent glycerol-3-phosphate
i02_4096	c4432	secB	preprotein translocase subunit SecB
i02_4098	c4434	/	hypothetical protein
i02_4097	c4433	grxC	glutaredoxin 3
	c4435	/	hypothetical protein
i02_4099	c4436	yibN	hypothetical protein
i02_4100	c4437	/	hypothetical protein
i02_4101	c4438	yibO	phosphoglyceromutase
i02_4102	c4439	yibP	hypothetical protein
i02_4103	c4440	yibQ	hypothetical protein
i02_4104	c4441	yibD	putative glycosyl transferase
	c4442	/	hypothetical protein
i02_4105	c4443	tdh	L-threonine 3-dehydrogenase
i02_4106	c4444	kbl	2-amino-3-ketobutyrate coenzyme A ligase
i02_4107	c4445	rfaD	ADP-L-glycero-D-mannoheptose-6-epimerase
i02_4108	c4446	rfaF	ADP-heptose:LPS heptosyltransferase II
i02_4109	c4447	rfaC	ADP-heptose:LPS heptosyl transferase I
i02_4110	c4448	waaL	Lipid A-core, surface polymer ligase
i02_4111	c4449	waaV	putative beta1,3-glucosyltransferase
i02_4112	c4450	waaW	UDP-galactose:(galactosyl) LPS
i02_4113	c4451	rfaY	lipopolysaccharide core biosynthesis protein
i02_4114	c4452	rfaJ	lipopolysaccharide 1,2-glucosyltransferase
i02_4115	c4453	rfaI	lipopolysaccharide 1,3-galactosyltransferase
i02_4116	c4454	rfaP	lipopolysaccharide core biosynthesis protein
i02_4117	c4455	rfaG	lipopolysaccharide core biosynthesis protein
i02_4118	c4456	rfaQ	lipopolysaccharide core biosynthesis protein
i02_4119	c4457	kdtA	3-deoxy-D-manno-octulosonic-acid transferase
i02_4120	c4458	coaD	phosphopantetheine adenylyltransferase
i02_4121	c4459	mutM	formamidopyrimidine-DNA glycosylase
i02_4122	c4460	rpmG	50S ribosomal protein L33
i02_4123	c4461	rpmB	50S ribosomal protein L28
i02_4124	c4462	radC	DNA repair protein RadC
i02_4125	c4463	dfp	bifunctional phosphopantothenoylcysteine
i02_4126	c4464	dut	deoxyuridine triphosphatase
i02_4127	c4465	slmA	nucleoid occlusion protein
i02_4128	c4466	pyrE	orotate phosphoribosyltransferase
i02_4129	c4467	rph	ribonuclease PH
i02_4130	c4468	yicC	hypothetical protein
i02_4131	c4469	dinD	DNA-damage-inducible protein D
i02_4132	c4470	yicG	hypothetical protein
i02_4133	c4471	ligB	NAD-dependent DNA ligase LigB
i02_4134	c4472	/	hypothetical protein
i02_4135	c4473	gmk	guanylate kinase
i02_4136	c4474	rpoZ	DNA-directed RNA polymerase subunit omega

Clone D	CFT 073	Gene	Product
i02_4137	c4475	spoT	bifunctional (p)ppGpp synthetase II
i02_4138	c4476	spoU	tRNA guanosine-2'-O-methyltransferase
i02_4139	c4477	recG	ATP-dependent DNA helicase RecG
i02_4140	c4478	gltS	Sodium/glutamate symport carrier protein
i02_4141	c4479	yicE	putative purine permease yicE
i02_4142	c4480	yicH	hypothetical protein
i02_4143	c4481	/	hypothetical protein
i02_4144	c4482	/	hypothetical protein
i02_4145	c4483	/	hypothetical protein
i02_4146	c4484	/	putative aldolase
i02_4147	c4485	/	putative PTS enzyme-II fructose
i02_4148	c4486	/	PTS system, fructose-like-2 IIB component 1
i02_4149	c4487	/	putative phosphotransferase system (PTS),
i02_4150	c4488	/	putative transcriptional antiterminator
i02_4151		/	hypothetical protein
i02_4152	c4489	yicI	alpha-xylosidase YicI
i02_4153	c4490	yicJ	putative transporter
i02_4154	c4491	intC	putative prophage integrase
i02_4155	-	/	conserved hypothetical protein
i02_4156	-	/	conserved hypothetical protein
i02_4157	-	/	conserved hypothetical protein
i02_4158	-	/	hexuronate transporter
i02_4159	-	/	small GTP-binding domain protein
i02_4160	-	/	conserved hypothetical protein
i02_4161	-	/	hypothetical protein
i02_4162	-	/	hypothetical protein
i02_4164	-	/	hypothetical protein
i02_4163	-	/	conserved hypothetical protein
i02_4165	-	/	DNA repair protein RadC
i02_4166	-	/	hypothetical protein
i02_4167	-	/	ATP binding protein
i02_4168	-	/	conserved hypothetical protein
i02_4169	-	/	hypothetical protein
i02_4170	-	/	hypothetical protein
i02_4171	-	/	hypothetical protein
-	c4492	/	ShiA-like protein
-	c4493	/	hypothetical protein
-	c4494	/	putative transcriptional regulator
-	c4495		hexuronate transporter
-	c4496	/	putative glucosidase
-	c4497	/	putative glucosidase
-	c4498	/	IS1 protein InsB
-	c4499	/	hypothetical protein
-	c4500	/	putative amino acid antiporter
-	c4501	/	hypothetical protein
-	c4502	/	putative antiporter
-	c4503	/	insertion element IS1 1/2/3/5/6 protein insA
-	c4504	insB	insertion element IS1 1/5/6 protein insB
-	c4505	/	hypothetical protein
-	c4506	insI	transposase insI
	c4507		transposase insF
-	c4508	/	hypothetical protein

Clone D	CFT 073	Gene	Product
-	c4509	/	hypothetical protein
-	c4510	/	hypothetical protein
-	c4511	/	hypothetical protein
-	c4512	/	hypothetical protein
-	c4513	/	hypothetical protein
-	c4514	/	hypothetical protein
-	c4517	/	hypothetical protein
-	c4518	/	hypothetical protein
-	c4519	/	hypothetical protein
-	c4520	/	hypothetical protein
-	c4521	/	hypothetical protein
-	c4522	/	hypothetical protein
-	c4523	/	hypothetical protein
-	c4524	/	putative propanol dehydrogenase
-	c4525	/	putative chormophorylate of CpcA
-	c4526	/	ethanolamine utilization protein eutJ
-	c4527	/	hypothetical protein
-	c4528	/	ethanolamine utilization protein eutN
-	c4529	/	hypothetical protein
-	c4530	tdcD	propionate/acetate kinase
-	c4531	/	hypothetical protein
-	c4532	/	hypothetical protein
-	c4533	/	putative pduB protein
-	c4534	/	ethanolamine utilization protein eutE
-	c4535	/	hypothetical protein
-	c4536	/	hypothetical protein
-	c4537	/	hypothetical protein
-	c4538	/	putative pyruvate formate-lyase
-	c4539	/	hypothetical protein
-	c4540	/	putative maturase-related protein
-	c4541	/	putative maturase-related protein
-	c4542	/	hypothetical protein
-	c4543	/	hypothetical protein
-	c4544	/	hypothetical protein
-	c4545	/	hypothetical protein
-	c4546	/	hypothetical protein
-	c4547	/	S-adenosylmethionine synthetase
-	c4548	/	hypothetical protein
-	c4549	/	hypothetical protein
-	c4550	/	hypothetical protein
-	c4551	/	hypothetical protein
-	c4552	/	transposase insC
-	c4556	/	hypothetical protein
-	c4557	/	hypothetical protein
-	c4558	/	hypothetical protein
-	c4559	/	hypothetical protein
-	c4560	/	hypothetical protein
-	c4562	/	hypothetical protein
-	c4561	/	hypothetical protein
-	c4563	/	hypothetical protein
-	c4564	/	hypothetical protein
-	c4565	/	hypothetical protein

Clone D	CFT 073	Gene	Product
-	c4566	/	putative transcriptional regulator YfjR
-	c4567	/	hypothetical protein
-	c4568	/	hypothetical protein
-	c4569	/	hypothetical protein
-	c4570	/	hypothetical protein
-	c4571	/	hypothetical protein
-	c4572	/	hypothetical protein
-	c4573	/	putative radC-like protein yeeS
-	c4574	/	hypothetical protein
-	c4575	/	hypothetical protein
-	c4576	/	hypothetical protein
-	c4577	/	hypothetical protein
-	c4578	/	hypothetical protein
-	c4579	/	hypothetical protein
i02_4172	c4580	/	hypothetical protein
i02_4173	c4581	/	hypothetical protein
i02_4174	c4582	yicL	putative transport protein YicL
i02_4175	c4583	nlpA	cytoplasmic membrane lipoprotein-28
i02_4176	c4584	/	hypothetical protein
i02_4177	c4585	/	hypothetical protein
i02_4178	c4586	nepI	ribonucleoside transporter
i02_4179	c4587	yicN	hypothetical protein
i02_4180	c4588	yicO	hypothetical protein
i02_4181	c4589	yicP	cryptic adenine deaminase
i02_4182	c4590	uhpT	sugar phosphate antiporter
i02_4183	c4591	uhpC	regulatory protein UhpC
i02_4184	c4592	uhpB	sensory histidine kinase UhpB
i02_4185	c4593	uhpA	DNA-binding transcriptional activator UhpA
i02_4186	c4594	/	hypothetical protein
i02_4187	c4595	ilvN	acetolactate synthase 1 regulatory subunit
i02_4189		/	hypothetical protein
i02_4188	c4596	ilvB	acetolactate synthase catalytic subunit
	c5498	ivbL	ilvB operon leader peptide
i02_4190	c4597	emrD	multidrug resistance protein D
i02_4191	c4598	yidF	hypothetical protein
i02_4192	c4599	yidG	hypothetical protein
i02_4193	c4600	yidH	hypothetical protein
i02_4194	c4601	yidJ	putative sulfatase yidJ
i02_4195	c4602	yidK	putative symporter YidK
i02_4196	c4603	yidL	putative transcriptional regulator YidL
i02_4197	c4604	yidE	hypothetical protein
i02_4198	c4605	/	hypothetical protein
i02_4199	c4606	ibpB	heat shock chaperone IbpB
i02_4200	c4607	ibpA	heat shock protein IbpA
i02_4201	c4608	yidQ	hypothetical protein
i02_4203	c4610	/	hypothetical protein
i02_4202	c4609	yidR	hypothetical protein
i02_4204	c4611	yidS	putative oxidoreductase
i02_4205	c4612	dgoT	D-galactonate transporter
i02_4206		rspA	starvation sensing protein rspA
i02_4207		dgoA	2-dehydro-3-deoxy-6-phosphogalactonate aldolase

Clone D	CFT 073	Gene	Product
i02_4208	c4615	dgoK	2-dehydro-3-deoxygalactonokinase
i02_4209	c4616	/	hypothetical protein
i02_4210	c4617	/	hypothetical protein
i02_4210	c4618	/	hypothetical protein
i02_4212	c4619	yidA	sugar phosphatase
i02_4213	c4620	yidB	hypothetical protein
i02_4214	c4621	gyrB	DNA gyrase subunit B
i02_4215	c4622	recF	recombination protein F
i02_4216	c4623	dnaN	DNA polymerase III subunit beta
i02_4217		yfgE	DNA replication initiation factor
i02_4218	c4626	/	hypothetical protein
i02_4219	c4627	rpmH	50S ribosomal protein L34
i02_4220	c4628	rnpA	ribonuclease P
i02_4221	c4629	yidC	putative inner membrane protein translocase
i02_4222	c4630	trmE	tRNA modification GTPase TrmE
i02_4223	c5499	tnaL	tryptophanase leader peptide
i02_4224	c4631	tnaA	tryptophanase
i02_4225	c4632	tnaB	tryptophan permease TnaB
i02_4226	c4633	yidY	multidrug efflux system protein MdtL
i02_4227	c4634	yidZ	DNA-binding transcriptional regulator YidZ
i02_4228	c4635	yieE	hypothetical protein
i02_4229	c4636	yieF	hypothetical protein
i02_4230	c4637	yieG	hypothetical protein
i02_4231	c4638	yieH	6-phosphogluconate phosphatase
i02_4232	c4639	yieI	putative inner membrane protein
i02_4233	c4640	yieK	putative 6-phosphogluconolactonase
i02_4234	c4641	yieL	hypothetical protein
i02_4235	c4642	/	hypothetical protein
i02_4236	c4643	bglB	6-phospho-beta-glucosidase bglB
	c4645	/	hypothetical protein
i02_4237	c4644	bglF	beta-glucoside-specific PTS system components
i02_4239	c4646	bglG	transcriptional antiterminator BglG
i02_4240	c4647	/	hypothetical protein
i02_4241	c4648	phoU	transcriptional regulator PhoU
	c4650	/	hypothetical protein
i02_4242	c4649	pstB	phosphate transporter subunit
i02_4243	c4651	pstA	phosphate transporter permease subunit PtsA
i02_4244	c4652	pstC	phosphate transporter permease subunit PstC
i02_4245	c4653	pstS	phosphate ABC transporter periplasmic
i02_4246	c4654	glmS	L-glutamine:D-fructose-6-phosphate aminotransferase
i02_4247	c4655	glmU	glucosamine uridyltransferase
	c4656	/	hypothetical protein
i02_4249	c4657	atpC	F0F1 ATP synthase subunit epsilon
i02_4250	c4658	atpD	F0F1 ATP synthase subunit beta
i02_4251	c4659	atpG	F0F1 ATP synthase subunit gamma
	c4661	/	hypothetical protein
i02_4252	c4660	atpA	F0F1 ATP synthase subunit alpha
i02_4254	c4663	/	hypothetical protein
i02_4253	c4662	atpH	F0F1 ATP synthase subunit delta
i02_4255	c4664	atpF	F0F1 ATP synthase subunit B
i02_4256	c4665	atpE	F0F1 ATP synthase subunit C

Clone D	CFT 073	Gene	Product
i02_4257	c4666	atpB	F0F1 ATP synthase subunit A
i02_4258	c4667	atpI	F0F1 ATP synthase subunit I
i02_4259	c4668	gidB	16S rRNA methyltransferase GidB
i02_4260	c4669	gidA	protein involved in a tRNA modification pathway
i02_4261	c4670	mioC	flavodoxin
i02_4262	c4671	asnC	DNA-binding transcriptional regulator AsnC
i02_4263	c4672	asnA	asparagine synthetase AsnA
i02_4264	c4673	yieM	hypothetical protein
i02_4265	c4674	yieN	regulatory ATPase RavA
i02_4266	c4675	trkD	potassium transport protein Kup
i02_4267	c4676	rbsD	D-ribose pyranase
i02_4268	c4677	rbsA	D-ribose transporter ATP binding protein
i02_4269	c4678	rbsC	ribose ABC transporter permease protein
i02_4270	c4679	rbsB	D-ribose transporter subunit RbsB
i02_4271	c4680	rbsK	ribokinase
i02_4272	c4681	rbsR	transcriptional repressor RbsR
i02_4273	c4682	yieO	putative transport protein YieO
i02_4274	c4683	yieP	putative transcriptional regulator YieP
i02_4276	c4685	yifA	transcriptional regulator HdfR
i02_4277	c4686	yifE	hypothetical protein
i02_4279	c4687	yifB	hypothetical protein
i02_4279	c4688	/	hypothetical protein
i02_4280	c4689	/	hypothetical protein
	c5500	ilvL	ilvG operon leader peptide
i02_4281	c4690	ilvG	acetolactate synthase 2 catalytic subunit
i02_4282	c4691	ilvM	acetolactate synthase 2 regulatory subunit
i02_4283	c4692	ilvE	branched-chain amino acid aminotransferase
i02_4284	c4693	ilvD	dihydroxy-acid dehydratase
i02_4285	c4694	ilvA	threonine dehydratase
i02_4286	c4695	ilvY	DNA-binding transcriptional regulator IlvY
i02_4287	c4696	ilvC	ketol-acid reductoisomerase
i02_4288	c4697	ppiC	peptidyl-prolyl cis-trans isomerase C
i02_4289	c4698	rep	ATP-dependent DNA helicase Rep
i02_4290	c4699	gppA	guanosine pentaphosphate phosphohydrolase
i02_4291		/	hypothetical protein
i02_4292	c4700	rhlB	ATP-dependent RNA helicase RhlB
i02_4293	c4701	trxA	thioredoxin
i02_4294		/	hypothetical protein
	c5501	rhoL	putative rho operon leader peptide
i02_4295	c4702	rho	transcription termination factor Rho
i02_4296	c4703	/	hypothetical protein
i02_4297	c4704	rfe	undecaprenyl-phosphate
i02_4298	c4705	wzzE	lipopolysaccharide biosynthesis protein WzzE
i02_4299	c4706	wecB	UDP-N-acetylglucosamine 2-epimerase
i02_4300	c4707	wecC	UDP-N-acetyl-D-mannosamine dehydrogenase
i02_4301	c4708	rffG	dTDP-glucose 4,6-dehydratase
i02_4302	c4709	rffH	glucose-1-phosphate thymidylyltransferase
i02_4303	c4710	wecD	TDP-fucosamine acetyltransferase
i02_4304	c4711	wecE	TDP-4-oxo-6-deoxy-D-glucose transaminase
i02_4306	c4713	wzxE	WzxE protein
i02_4305	c4712	/	hypothetical protein
i02_4307	c4714	/	4-alpha-L-fucosyltransferase

Clone D	CFT 073	Gene	Product
i02_4308	c4715	wecF	putative common antigen polymerase
i02_4309	c4716	wecG	putative UDP-N-acetyl-D-mannosaminuronic acid transferase
i02_4310	c4717	yifK	putative transport protein YifK
i02_4311	c4718	aslB	putative arylsulfatase regulatory protein
i02_4313	c4720	/	hypothetical protein
i02_4312	c4719	aslA	arylsulfatase
i02_4314	c4721	hemY	putative protoheme IX biogenesis protein
i02_4315	c4722	hemX	putative uroporphyrinogen III
i02_4316	c4723	hemD	uroporphyrinogen-III synthase
i02_4317	c4724	hemC	porphobilinogen deaminase
i02_4318	c4725	cyaA	adenylate cyclase
i02_4319	c4726	cyaY	frataxin-like protein
i02_4320	c4727	/	hypothetical protein
i02_4321	c4728	/	hypothetical protein
i02_4322	c4729	/	hypothetical protein
i02_4323	c4730	dapF	diaminopimelate epimerase
i02_4324	c4731	yigA	hypothetical protein
i02_4325	c4732	xerC	site-specific tyrosine recombinase XerC
i02_4326	c4733	yigB	flavin mononucleotide phosphatase
i02_4327	c4734	uvrD	DNA-dependent helicase II
i02_4328	c4735	/	hypothetical protein
i02_4329	c4736	/	hypothetical protein
i02_4330		/	hypothetical protein
i02_4331	c4737	corA	magnesium/nickel/cobalt transporter CorA
i02_4332	c4738	/	hypothetical protein
i02_4333	c4739	/	hypothetical protein
i02_4334	c4740	rarD	protein rarD
i02_4335	c4741	yigI	hypothetical protein
i02_4336	c4742	pldA	phospholipase A
i02_4337	c4743	/	hypothetical protein
i02_4338	c4744	recQ	ATP-dependent DNA helicase RecQ
i02_4339	c4745	/	threonine efflux system
i02_4340	c4746	rhtB	homoserine/homoserine lactone efflux protein
i02_4341	c4747	pldB	lysophospholipase L2
i02_4342	c4748	/	putative sugar phosphatase
i02_4343	c4749	yigM	membrane protein yigM
i02_4344	c4750	metR	transcriptional activator protein metR
i02_4346	c4751	metE	cobalamin-independent homocysteine transmethylase
i02_4345		/	hypothetical protein
i02_4347	c4752	/	hypothetical protein
i02_4348	c4753	/	hypothetical protein
i02_4349	c4754	/	hypothetical protein
i02_4350	c4755	/	hypothetical protein
i02_4351	c4756	/	hypothetical protein
i02_4352	c4757	/	hypothetical protein
i02_4353	c4758	/	PTS system, glucose-specific IIBC component
i02_4354	c4759	/	transketolase
i02_4355	c4760	/	hypothetical protein
i02_4356	c4761	/	hypothetical protein
i02_4357	c4762	/	putative permease

Clone D	CFT 073	Gene	Product
i02_4358	c4763	/	hypothetical protein
i02_4359	c4764	/	carbamate kinase
	c4766	/	hypothetical protein
i02_4360	c4765	/	hypothetical protein
i02_4361	c4767	/	hypothetical protein
i02_4362	c4768	/	hypothetical protein
i02_4363	c4769	/	hypothetical protein
i02_4365		/	hypothetical protein
i02_4364	c4770	/	hypothetical protein
i02_4366	c4771	/	putative carboxymethylenebutenolidase
i02_4367	c4773	udp	uridine phosphorylase
	c4772	/	hypothetical protein
i02_4368	c4774	/	hypothetical protein
i02_4369	c4775	/	hypothetical protein
i02_4370	c4776	/	hypothetical protein
i02_4372		/	putative aldolase
i02_4371	c4777	/	2-dehydro-3-deoxy-6-phosphogalactonate aldolase
i02_4373	c4778	/	hypothetical protein
i02_4374	c4779	/	putative transcriptional regulator
	c4780	/	hypothetical protein
i02_4375	c4781	yigN	DNA recombination protein RmuC
i02_4376	c4782	ubiE	ubiquinone/menaquinone biosynthesis protein
i02_4377	c4783	yigP	hypothetical protein
i02_4378	c4784	ubiB	putative ubiquinone biosynthesis protein UbiB
i02_4379	c4785	tatA	twin arginine translocase protein A
i02_4380	c4786	/	sec-independent translocase
i02_4381	c4787	yigU	twin-arginine protein translocation system
i02_4382	c4788	yigW	DNase TatD
i02_4383	c4789	rfaH	transcriptional activator RfaH
i02_4384	c4790	yigC	3-octaprenyl-4-hydroxybenzoate decarboxylase
i02_4385	c4791	fre	FMN reductase
i02_4386	c4792	fadA	3-ketoacyl-CoA thiolase
i02_4387	c4793	fadB	multifunctional fatty acid oxidation complex
i02_4388	c4794	pepQ	proline dipeptidase
i02_4389	c4795	yigZ	hypothetical protein
i02_4390	c4796	trkH	potassium transporter
i02_4391	c4797	hemG	protoporphyrinogen oxidase
i02_4393	c4800	mobB	molybdopterin-guanine dinucleotide biosynthesis protien
i02_4394	c4801	mobA	molybdopterin-guanine dinucleotide biosynthesis protien
i02_4395	c4802	yihD	hypothetical protein
i02_4396	c4803	yihE	serine/threonine protein kinase
i02_4397	c4804	dsbA	periplasmic protein disulfide isomerase I
i02_4398	c4805	/	hypothetical protein
i02_4399	c4806	yihF	hypothetical protein
i02_4400	c4807	yihG	putative acyltransferase
i02_4401	c4808	/	hypothetical protein
i02_4402		xni	exonuclease IX
i02_4403	c4811	/	hypothetical protein
i02_4404	c4812	engB	ribosome biogenesis GTP-binding protein YsxC

Clone D	CFT 073	Gene	Product
i02_4405	c4813	/	hypothetical protein
i02_4407	c4815	yihI	hypothetical protein
i02_4406	c4814	/	hypothetical protein
i02_4409	c4816	hemN	coproporphyrinogen III oxidase
i02_4408		/	predicted protein
i02_4410	c4817	glnG	nitrogen regulation protein NR(I)
i02_4411	c4818	glnL	nitrogen regulation protein NR(II)
i02_4412	c4819	glnA	glutamine synthetase
i02_4413	c4820	yihK	GTP-binding protein
i02_4414	c4821	yihL	putative transcriptional regulator YihL
i02_4415	c4822	yihM	hypothetical protein
i02_4416	c4823	yihN	hypothetical protein
i02_4417	c4824	yihW	putative transcriptional regulator YihW
i02_4419	c4826	yihU	oxidoreductase yihU
i02_4418	c4825	yihV	putative sugar kinase yihV
i02_4421	c4827	yihT	hypothetical protein
i02_4422	c4828	/	putative dehydrogenase
i02_4423	c4829	/	hypothetical protein
i02_4424	c4830	/	shikimate transporter
i02_4425	c4831	/	hypothetical protein
i02_4426	c4832	yihX	phosphatase
i02_4427	c4833	rbn	ribonuclease BN
i02_4428	c4834	yihZ	D-tyrosyl-tRNA(Tyr) deacylase
i02_4429	c4835	yiiD	hypothetical protein
i02_4430	c4836	/	putative lipase
i02_4431		/	putative lipase
i02_4432	c4837	/	hypothetical protein
i02_4433	c4838	/	hypothetical protein
i02_4434	c4839	/	hypothetical protein
i02_4435	c4840	yiiE	hypothetical protein
i02_4436	c4841	fdhE	formate dehydrogenase accessory protein FdhE
i02_4437	c4842	fdoI	formate dehydrogenase-O subunit gamma
i02_4438	c4843	fdoH	formate dehydrogenase-O beta subunit
i02_4439	c5624	fdoG	formate dehydrogenase-O, major subunit
i02_4440	c4846	/	hypothetical protein
i02_4441	c4847	fdhD	formate dehydrogenase accessory protein
i02_4442	c4848	/	hypothetical protein
i02_4443	c4849	/	putative glycoporin
i02_4444	c4850	yiiL	hypothetical protein
i02_4445	c4851	rhaD	rhamnulose-1-phosphate aldolase
i02_4446	c4852	rhaA	L-rhamnose isomerase
i02_4447	c4853	rhaB	rhamnulokinase
i02_4448	c4854	/	hypothetical protein
i02_4449	c4855	rhaS	transcriptional activator RhaS
i02_4450	c4856	rhaR	transcriptional activator RhaR
i02_4452	c4858	/	hypothetical protein
i02_4451	c4857	rhaT	rhamnose-proton symporter
i02_4453	c4859	sodA	superoxide dismutase
i02_4454	c4860	/	hypothetical protein
i02_4455	c4861	kdgT	2-keto-3-deoxygluconate permease
i02_4456	c4862	yiiM	hypothetical protein
i02_4457	c4863	cpxA	two-component sensor protein

Clone D	CFT 073	Gene	Product
i02_4458	c4864	cpxR	DNA-binding transcriptional regulator CpxR
i02_4459	c4865	cpxP	periplasmic repressor CpxP
i02_4460	c4866	fieF	ferrous iron efflux protein F
i02_4461	c4867	pfkA	6-phosphofructokinase
	c4868	/	hypothetical protein
i02_4462	c4869	sbp	sulfate transporter subunit
i02_4463	c4870	cdh	CDP-diacylglycerol pyrophosphatase
i02_4464	c4871	tpiA	triosephosphate isomerase
i02_4465	c4872	yiiQ	hypothetical protein
i02_4466	c4873	yiiR	hypothetical protein
i02_4467	c4874	yiiS	hypothetical protein
i02_4468	c4875	yiiT	universal stress protein UspD
i02_4469	c4876	fpr	ferredoxin-NADP reductase
i02_4470	c4877	glpX	fructose 1,6-bisphosphatase II
i02_4471	c4878	glpK	glycerol kinase
i02_4472	c4879	glpF	glycerol uptake facilitator protein
i02_4473	c4880	yiiU	hypothetical protein
	c4882	/	hypothetical protein
i02_4474	c4881	menG	ribonuclease activity regulator protein RraA
i02_4475	c4883	menA	1,4-dihydroxy-2-naphthoate
i02_4476	c4884	hslU	ATP-dependent protease ATP-binding subunit HslU
i02_4477	c4885	hslV	ATP-dependent protease peptidase subunit
i02_4478	c4886	ftsN	essential cell division protein FtsN
i02_4479	c4887	cytR	DNA-binding transcriptional regulator CytR
i02_4480	c4888	priA	primosome assembly protein PriA
i02_4481	c4889	/	hypothetical protein
i02_4482	c4890	yiiX	putative peptidoglycan peptidase
i02_4483	c4891	metJ	transcriptional repressor protein MetJ
i02_4484	c4892	metB	cystathionine gamma-synthase
i02_4485	c4893	metL	bifunctional aspartate kinase II/homoserine
i02_4486	c4894	/	nucleoside-specific channel-forming protein tsx
i02_4487	c4895	/	hypothetical protein
i02_4488	c4896	/	hypothetical protein
i02_4489	c4897	/	hypothetical protein
i02_4490	c4898	/	hypothetical protein
i02_4491	c4899	metF	5,10-methylenetetrahydrofolate reductase
i02_4492	c4900	katG	peroxidase/catalase HPI
i02_4493	c4901	yijE	putative transport protein YijE
i02_4494	c4902	yijF	hypothetical protein
i02_4495	c4903	/	hypothetical protein
i02_4496	c4904	gldA	glycerol dehydrogenase
i02_4497	c4905	talC	fructose-6-phosphate aldolase
i02_4498	c4906	ptsA	phosphoenolpyruvate-protein phosphotransferase
i02_4499	c4907	frwC	putative fructose-like permease EIIC subunit 2
i02_4500	c4908	frwB	putative fructose-like phosphotransferase EIIB
	c4909	/	hypothetical protein
i02_4501	c4910	pflD	putative formate acetyltransferase 2
i02_4502	c4911	pflC	pyruvate formate lyase II activase
i02_4503	c4912	frwD	putative fructose-like phosphotransferase EIIB
i02_4504	c4913	yijO	putative transcriptional regulator YijO
i02_4505	c4914	yijP	hypothetical protein

Clone D	CFT 073	Gene	Product
i02_4506	c4915	ppc	phosphoenolpyruvate carboxylase
i02_4507	c4916	argE	acetylornithine deacetylase
i02_4508	c4917	argC	N-acetyl-gamma-glutamyl-phosphate reductase
i02_4509	c4918	argB	acetylglutamate kinase
i02_4510	c4919	argH	argininosuccinate lyase
i02_4511	c4920	/	starvation sensing protein rspA
i02_4512	c4921	yjiZ	putative transport protein YjjL
i02_4513	c4922	oxyR	DNA-binding transcriptional regulator OxyR
i02_4514	c4923	udhA	soluble pyridine nucleotide transhydrogenase
i02_4515	c4924	/	putative hippuricase
i02_4516	c4925	/	putative citrate permease
i02_4517	c4926	yijC	DNA-binding transcriptional repressor FabR
i02_4518	c4927	yijD	hypothetical protein
i02_4519	c4928	trmA	tRNA (uracil-5-)-methyltransferase
i02_4520	c4929	btuB	vitamin B12/cobalamin outer membrane
i02_4521	c4930	murI	glutamate racemase
i02_4523	c4931	murB	UDP-N-acetylenolpyruvoylglucosamine reductase
i02_4524	c4932	birA	biotin--protein ligase
i02_4525	c4933	coaA	pantothenate kinase
i02_4526	c4934	/	hypothetical protein
i02_4527	c4935	tuf	elongation factor Tu
i02_4528	c4936	secE	preprotein translocase subunit SecE
i02_4529	c4937	nusG	transcription antitermination protein NusG
	c4938	/	hypothetical protein
i02_4530	c4939	rplK	50S ribosomal protein L11
i02_4531	c4940	rplA	50S ribosomal protein L1
i02_4532	c4941	rplJ	50S ribosomal protein L10
	c4942	/	hypothetical protein
i02_4533	c4943	rplL	50S ribosomal protein L7/L12
i02_4534	c4944	rpoB	DNA-directed RNA polymerase subunit beta
i02_4535	c4945	rpoC	DNA-directed RNA polymerase subunit beta'
i02_4536	c4946	thiH	thiamine biosynthesis protein ThiH
i02_4537	c4947	thiG	thiazole synthase
i02_4538	c4948	thiS	sulfur carrier protein ThiS
i02_4539	c4949	thiF	thiamine biosynthesis protein ThiF
i02_4540	c4950	thiE	thiamine-phosphate pyrophosphorylase
i02_4541	c4951	thiC	thiamine biosynthesis protein ThiC
i02_4542	c4952	yjaE	anti-RNA polymerase sigma 70 factor
i02_4543	c4953	nudC	NADH pyrophosphatase
i02_4544	c4954	hemE	uroporphyrinogen decarboxylase
i02_4545	c4955	nfi	endonuclease V
i02_4546	c4956	yjaG	hypothetical protein
i02_4547	c4957	hupA	transcriptional regulator HU subunit alpha
i02_4548	c4958	yjaH	hypothetical protein
i02_4549	c4959	zraP	zinc resistance protein
i02_4551	c4961	hydH	sensor protein ZraS
i02_4552	c4962	hydG	transcriptional regulatory protein ZraR
i02_4553	c4963	purD	phosphoribosylamine--glycine ligase
i02_4554	c4964	purH	bifunctional
i02_4555	c4965	/	hypothetical protein
i02_4556		/	hypothetical protein
i02_4558	c4968	yjaA	hypothetical protein

Clone D	CFT 073	Gene	Product
i02_4559	c4969	yjaB	hypothetical protein
i02_4560	c4970	metA	homoserine O-succinyltransferase
i02_4561	c4971	aceB	malate synthase
i02_4562	c4972	aceA	isocitrate lyase
	c4973	/	hypothetical protein
i02_4563	c4974	aceK	bifunctional isocitrate dehydrogenase
i02_4564	c4975	iclR	transcriptional repressor IclR
i02_4565	c4976	metH	B12-dependent methionine synthase
i02_4566	c4977	/	hypothetical protein
i02_4567	c4978	/	hypothetical protein
i02_4568	c4979	yjbB	hypothetical protein
i02_4569	c4980	pepE	peptidase E
i02_4570	c4981	/	putative oxidoreductase
i02_4571	c4982	/	PTS system, mannose-specific IID component
i02_4572	c4983	/	PTS system, mannose-specific IIC component
i02_4573	c4984	/	putative sorbose PTS component
i02_4574	c4985	/	putative sorbose PTS component
i02_4575	c4986	/	sorbitol-6-phosphate 2-dehydrogenase
i02_4576	c4987	/	putative transcriptional regulator of sorbose
i02_4577	c4988	yjbC	23S rRNA pseudouridine synthase F
i02_4578	c4989	yjbD	hypothetical protein
i02_4579	c4990	lysC	aspartate kinase III
i02_4580	c4991	pgi	glucose-6-phosphate isomerase
	c4994	yjbE	hypothetical protein
i02_4581	c4995	yjbF	lipoprotein yjbF precursor
i02_4582	c4996	yjbG	hypothetical protein
i02_4583	c4997	yjbH	lipoprotein yjbH precursor
i02_4584	c4998	/	hypothetical protein
	c4999	/	hypothetical protein
i02_4585	c5000	/	hypothetical protein
i02_4586	c5001	yjbA	phosphate-starvation-inducible protein PsiE
i02_4587	c5002	malG	maltose transporter permease
i02_4588	c5003	malF	maltose transporter membrane protein
i02_4589	c5004	malE	maltose ABC transporter periplasmic protein
i02_4590	c5005	malK	maltose/maltodextrin transporter
i02_4591	c5006	lamB	maltoporin
i02_4592	c5007	malM	maltose regulon periplasmic protein
i02_4593	c5008	/	hypothetical protein
i02_4594	c5009	ubiC	chorismate pyruvate lyase
i02_4595	c5010	ubiA	4-hydroxybenzoate octaprenyltransferase
i02_4596	c5011	plsB	glycerol-3-phosphate acyltransferase
i02_4598	c5013	dgkA	diacylglycerol kinase
i02_4597	c5012	/	hypothetical protein
i02_4599	c5014	lexA	LexA repressor
i02_4600	c5015	dinF	DNA-damage-inducible SOS response protein
i02_4601	c5016	yjbJ	putative stress-response protein
i02_4603	c5018	yjbN	tRNA-dihydrouridine synthase A
i02_4602	c5017	yjbK	zinc uptake transcriptional repressor
i02_4604	c5019	pspG	phage shock protein G
i02_4605	c5020	qor	quinone oxidoreductase, NADPH-dependent
i02_4606	c5021	/	putative oxidoreductase
i02_4607	c5022	/	putative transmembrane transport protein

Clone D	CFT 073	Gene	Product
i02_4608	c5023	/	putative crotonase
i02_4609	c5024	/	putative propionate CoA-transferase
i02_4610	c5025	/	putative regulator
i02_4611	c5026	dnaB	replicative DNA helicase
i02_4613	c5028	alr	alanine racemase
i02_4612	c5027	/	hypothetical protein
i02_4614	c5029	/	putative transporter
i02_4615	c5030	/	NadR-like protein
i02_4616	c5031	tyrB	aromatic amino acid aminotransferase
i02_4617	c5032	sucA	2-oxoglutarate dehydrogenase E1 component
i02_4619	c5034	/	dihydrolipoamide succinyltransferase component
i02_4618	c5033	/	hypothetical protein
i02_4620	c5035	/	putative 2-oxoglutarate dehydrogenase
i02_4621	c5036	/	succinyl-CoA synthetase beta chain
i02_4622	c5037	/	succinyl-CoA synthetase alpha chain
i02_4623	c5038	/	putative membrane-bound protein
i02_4624	c5039	/	putative lactate dehydrogenase
i02_4625	c5040	/	putative c4-dicarboxylate transport
	c5042	/	hypothetical protein
i02_4626	c5041	/	putative transport sensor protein
i02_4628	c5043	/	hypothetical protein
	c5044	/	hypothetical protein
i02_4629	c5045	aphA	acid phosphatase/phosphotransferase
i02_4630	c5046	yjbQ	hypothetical protein
i02_4631	c5047	yjbR	hypothetical protein
i02_4632	c5048	uvrA	excinuclease ABC subunit A
i02_4633	c5049	ssb	single-stranded DNA-binding protein
i02_4635	c5051	/	hypothetical protein
i02_4634	c5050	yjcB	hypothetical protein
i02_4636	c5052	yjcC	hypothetical protein
i02_4637	c5053	soxS	DNA-binding transcriptional regulator SoxS
i02_4638	c5054	soxR	redox-sensitive transcriptional activator SoxR
i02_4639	c5055	/	hypothetical protein
i02_4640	c5056	/	hypothetical protein
i02_4641	c5057	/	putative Na(+)/H(+) exchanger yjcE
i02_4642	c5058	/	putative transcriptional regulator
	c5059	/	hypothetical protein
i02_4643	c5060	/	hypothetical protein
i02_4644	c5061	/	hypothetical protein
i02_4645	c5062	actP	acetate permease
i02_4646	c5063	yjcH	hypothetical protein
i02_4647		/	hypothetical protein
i02_4648	c5064	acs	acetyl-CoA synthetase
i02_4649	c5065	/	hypothetical protein
i02_4650	c5066	nrfA	cytochrome c552
i02_4651	c5067	nrfB	cytochrome c nitrite reductase pentaheme
i02_4652	c5068	nrfC	NrfC protein
i02_4653	c5069	nrfD	NrfD protein
i02_4654	c5070	nrfE	heme lyase subunit NrfE
i02_4655	c5071	nrfF	formate-dependent nitrite reductase complex
i02_4656	c5072	nrfG	formate-dependent nitrite reductase complex
i02_4657	c5073	/	hypothetical protein

Clone D	CFT 073	Gene	Product
i02_4658	c5074	gltP	glutamate/aspartate:proton symporter
i02_4659	c5075	/	hypothetical protein
i02_4660	c5076	yjcO	hypothetical protein
i02_4661	c5077	yddO	ABC transporter ATP-binding protein
i02_4662	c5078	/	putative oligopeptide ABC transporter
i02_4663	c5079	yddQ	ABC transporter permease
i02_4664	c5080	yddR	ABC transporter permease
i02_4665	c5081	/	hypothetical protein
i02_4667	c5083	/	hypothetical protein
i02_4666	c5625	fdhF	formate dehydrogenase H
i02_4668	c5085	yjcP	putative outer membrane efflux protein MdtP
i02_4669	c5086	yjcQ	multidrug efflux system protein MdtO
i02_4670	c5087	yjcR	multidrug resistance protein MdtN
i02_4671	c5088	/	hypothetical protein
i02_4672	c5089	yjcS	hypothetical protein
i02_4673	c5090	yjcT	D-allose kinase
i02_4674	c5091	yjcU	allulose-6-phosphate 3-epimerase
i02_4675	c5092	yjcV	D-allose transporter subunit
i02_4676	c5093	yjcW	D-allose transporter ATP-binding protein
i02_4677	c5094	yjcX	D-allose transporter subunit
i02_4678	c5095	rpiR	DNA-binding transcriptional repressor RpiR
i02_4679	c5096	rpiB	ribose-5-phosphate isomerase B
i02_4680	c5097	/	hypothetical protein
i02_4681	c5098	phnP	carbon-phosphorus lyase complex accessory
i02_4682	c5099	phnO	aminoalkylphosphonic acid N-acetyltransferase
i02_4683		phnN	ribose 1,5-bisphosphokinase
i02_4684	c5101	phnM	PhnM protein
i02_4685	c5102	phnL	phosphonate ABC transporter ATP-binding protein
i02_4686	c5103	phnK	phosphonate C-P lyase system protein PhnK
i02_4687	c5104	phnJ	PhnJ protein
i02_4689	c5105	phnI	PhnI protein
i02_4690	c5106	phnH	carbon-phosphorus lyase complex subunit
i02_4691	c5107	phnG	PhnG protein
i02_4692	c5108	phnF	phosphonate metabolism transcriptional regulator
i02_4693	c5109	phnE	membrane channel protein component of Pn
i02_4694	c5110	phnD	phosphonates-binding periplasmic protein
i02_4695	c5111	phnC	phosphonate/organophosphate ester transporter
i02_4696	c5112	phnB	hypothetical protein
i02_4698	c5113	phnA	PhnA protein
i02_4699	c5114	yjdA	hypothetical protein
i02_4700	c5115	yjcZ	hypothetical protein
i02_4701	c5116	proP	proline/glycine betaine transporter
i02_4702	c5117	basS	sensor protein BasS/PmrB
i02_4703	c5118	basR	DNA-binding transcriptional regulator BasR
i02_4704	c5119	yjdB	putative cell division protein
i02_4705	c5120	yjdE	arginine:agmatin antiporter
i02_4706	c5121	adiY	putative regulatory protein adiY
i02_4707	c5122	adiA	biodegradative arginine decarboxylase
i02_4708	c5123	melR	DNA-binding transcriptional regulator MelR
i02_4709	c5124	melA	alpha-galactosidase
i02_4710		/	membrane protein

Clone D	CFT 073	Gene	Product
i02_4711		yjdF	hypothetical protein
i02_4712	c5127	fumB	fumarate hydratase class I, anaerobic
i02_4713	c5128	dcuB	anaerobic C4-dicarboxylate transporter
i02_4714	c5129	/	hypothetical protein
i02_4715	c5130	yjdG	DNA-binding transcriptional activator DcuR
i02_4717	c5132	/	hypothetical protein
i02_4716	c5131	yjdH	sensory histidine kinase DcuS
i02_4718	c5133	yjdI	hypothetical protein
	c5134	yjdJ	hypothetical protein
i02_4719	c5135	/	hypothetical protein
i02_4720		yjdK	hypothetical protein
i02_4721	c5136	/	hypothetical protein
i02_4722	c5137	/	hypothetical protein
i02_4723	c5138	lysS	lysyl-tRNA synthetase
i02_4724	c5139	yjdL	putative transporter YjdL
i02_4725	c5140	cadA	lysine decarboxylase
i02_4726	c5141	cadB	lysine/cadaverine antiporter
i02_4727	c5142	cadC	DNA-binding transcriptional activator CadC
-	c5143	/	hypothetical protein
-	c5144	/	hypothetical protein
-	c5145	/	hypothetical protein
-	c5146	/	hypothetical protein
-	c5147	/	hypothetical protein
-	c5148	/	hypothetical protein
-	c5149	/	hypothetical protein
-	c5150	/	hypothetical protein
-	c5151	/	hypothetical protein
-	c5152	/	putative radC-like protein yeeS
-	c5153	/	hypothetical protein
-	c5154	/	hypothetical protein
-	c5155	/	hypothetical protein
-	c5156	/	hypothetical protein
-	c5157	/	hypothetical protein
-	c5158	/	hypothetical protein
-	c5159	/	hypothetical protein
-	c5160	/	putative transcriptional regulator YfjR
-	c5161	/	hypothetical protein
-	c5162	/	hypothetical protein
-	c5163	/	hypothetical protein
-	c5164	/	hypothetical protein
-	c5165	/	hypothetical protein
-	c5166	/	partial transposase
-	c5167	/	transposase IS629
-	c5168	/	hypothetical protein
-	c5169	/	hypothetical protein
-	c5170	/	hypothetical protein
-	c5171	/	hypothetical protein
-	c5172	/	hypothetical protein
-	c5173	/	hypothetical protein
-	c5174	ireA	putative iron-regulated outer membrane virulence
-	c5175	/	hypothetical protein
-	c5176	/	transposase

Clone D	CFT 073	Gene	Product
-	c5177	/	hypothetical protein
-	c5178	/	transposase IS629
-	c5179	papG_2	PapG protein
-	c5180	papF_2	PapF protein
-	c5181	papE_2	PapE protein
-	c5182	papK_2	PapK protein
-	c5183	/	hypothetical protein
-	c5184	papJ_2	PapJ protein
-	c5185	papD_2	PapD protein
-	c5186	papC_2	PapC protein
-	c5187	papH_2	PapH protein
-	c5188	papA_2	PapA protein
-	c5189	papI_2	PapI protein
-	c5190	/	hypothetical protein
-	c5191	/	hypothetical protein
-	c5192	/	hypothetical protein
-	c5193	/	hypothetical protein
-	c5194	/	hypothetical protein
-	c5195	/	hypothetical protein
-	c5196	/	transposase insC
-	c5197	/	transposase insD
-	c5198	/	hypothetical protein
-	c5199	/	hypothetical protein
-	c5200	/	hypothetical protein
-	c5201	pgtP	transporter protein
-	c5202	pgtC	regulatory protein
-	c5203	pgtB	regulatory protein
-	c5204	pgtA	transport activator
-	c5205	/	hypothetical protein
-	c5206	/	hypothetical protein
-	c5207	/	hypothetical protein
-	c5208	/	hypothetical protein
-	c5209	/	hypothetical protein
-	c5210	/	hypothetical protein
-	c5211	/	hypothetical protein
-	c5212	/	hypothetical protein
-	c5213	/	transposase IS629
-	c5214	/	hypothetical protein
-	c5215	/	hypothetical protein
-	c5216	/	prophage P4 integrase
i02_4728	c5217	yjdC	putative transcriptional regulator
i02_4729	c5218	dipZ	thiol:disulfide interchange protein precursor
i02_4730	c5219	cutA	divalent-cation tolerance protein CutA
i02_4731	c5220	dcuA	anaerobic C4-dicarboxylate transporter
	c5221	/	hypothetical protein
i02_4732	c5222	aspA	aspartate ammonia-lyase
i02_4733	c5223	fxsA	FxsA
i02_4734	c5224	yjeH	inner membrane protein YjeH
	c5225	/	hypothetical protein
i02_4736	c5226	groES	co-chaperonin GroES
i02_4735		/	hypothetical protein
i02_4737	c5227	groEL	chaperonin GroEL

Clone D	CFT 073	Gene	Product
i02_4738	c5228	yjeI	hypothetical protein
	c5229	/	hypothetical protein
i02_4739	c5230	yjeJ	hypothetical protein
i02_4740	c5231	yjeK	hypothetical protein
i02_4741	c5232	efp	elongation factor P
	c5233	/	hypothetical protein
i02_4742	c5234	ecnA	entericidin A
i02_4743	c5235	ecnB	entericidin B membrane lipoprotein
i02_4744	c5236	sugE	SugE protein
i02_4745	c5237	blc	outer membrane lipoprotein Blc
i02_4746	c5238	ampC	beta-lactamase
i02_4747	c5239	frdD	fumarate reductase subunit D
i02_4748	c5240	frdC	fumarate reductase subunit C
i02_4749	c5241	frdB	fumarate reductase iron-sulfur subunit
i02_4750	c5242	frdA	fumarate reductase flavoprotein subunit
i02_4751	c5243	yjeA	lysyl-tRNA synthetase
i02_4752	c5244	yjeM	putative transporter YjeM
i02_4754	c5246	/	hypothetical protein
i02_4753	c5245	yjeP	hypothetical protein
i02_4755	c5247	psd	phosphatidylserine decarboxylase
i02_4756	c5248	yjeQ	ribosome-associated GTPase
i02_4757	c5249	yjeR	oligoribonuclease
i02_4759	c5251	yjeF	hypothetical protein
i02_4758	c5250	yjeS	putative electron transport protein yjeS
i02_4760	c5252	yjeE	putative ATPase
i02_4761	c5253	amiB	N-acetylmuramoyl-l-alanine amidase II
i02_4762	c5254	mutL	DNA mismatch repair protein
i02_4763	c5255	miaA	tRNA dimethylallyltransferase
i02_4764	c5256	hfq	RNA-binding protein Hfq
i02_4765	c5257	hflX	putative GTPase HflX
i02_4766	c5258	hflK	FtsH protease regulator HflK
i02_4767	c5259	hflC	FtsH protease regulator HflC
i02_4768	c5260	yjeT	hypothetical protein
i02_4769	c5261	purA	adenylosuccinate synthetase
i02_4770	c5262	yjeB	transcriptional repressor NsrR
i02_4771	c5263	vacB	exoribonuclease R
i02_4772	c5264	yjfH	23S rRNA (guanosine-2'-O-)-methyltransferase
i02_4773	c5265	yjfI	hypothetical protein
i02_4774	c5266	yjfJ	hypothetical protein
i02_4775	c5267	yjfK	hypothetical protein
i02_4776	c5268	yjfL	hypothetical protein
i02_4776	c5269	/	hypothetical protein
	c5270	/	hypothetical protein
	c5271	/	hypothetical protein
i02_4777	c5272	yjfM	hypothetical protein
i02_4778	c5274	yjfC	hypothetical protein
i02_4779	c5275	aidB	isovaleryl CoA dehydrogenase
i02_4780	c5276	yjfN	hypothetical protein
i02_4781	c5277	yjfO	hypothetical protein
i02_4782	c5278	yjfP	esterase
i02_4783	c5279	yjfQ	transcriptional repressor UlaR
i02_4784	c5280	yjfR	putative L-ascorbate 6-phosphate lactonase

Clone D	CFT 073	Gene	Product
i02_4785	c5281	ulaA	ascorbate-specific PTS system enzyme IIC
	c5282	/	hypothetical protein
i02_4786	c5283	sgaB	L-ascorbate-specific enzyme IIB component
i02_4787	c5284	ptxA	L-ascorbate-specific enzyme IIA component
i02_4788	c5285	ulaD	3-keto-L-gulonate-6-phosphate decarboxylase
	c5286	/	hypothetical protein
i02_4789	c5287	sgaU	L-xylulose 5-phosphate 3-epimerase
i02_4790	c5288	sgaE	L-ribulose-5-phosphate 4-epimerase
i02_4791	c5289	yjfY	hypothetical protein
	c5290	/	hypothetical protein
i02_4792	c5291	rpsF	30S ribosomal protein S6
i02_4793		priB	primosomal replication protein N
i02_4794	c5292	rpsR	30S ribosomal protein S18
	c5293	/	hypothetical protein
i02_4795	c5294	rplI	50S ribosomal protein L9
i02_4796	c5295	/	hypothetical protein
i02_4797	c5296	/	hypothetical protein
i02_4799	c5297	/	hypothetical protein
i02_4800	c5298	/	hexuronate transporter
i02_4801	c5299	/	putative oxidoreductase
i02_4802	c5300	/	putative acetyl-CoA:acetoacetyl-CoA transferase
i02_4803	c5301	/	hypothetical protein
i02_4805	c5302	/	hypothetical protein
i02_4806	c5303	fabG	3-ketoacyl-(acyl-carrier-protein) reductase
i02_4807	c5304	/	hypothetical protein
i02_4808	c5305	ytfB	hypothetical protein
i02_4809	c5306	fklB	peptidyl-prolyl cis-trans isomerase
i02_4810	c5307	cycA	D-alanine/D-serine/glycine permease
i02_4811	c5308	ytfE	iron-sulfur cluster repair di-iron protein
i02_4812	c5309	ytfF	hypothetical protein
i02_4814	c5311	ytfH	hypothetical protein
i02_4813	c5310	ytfG	hypothetical protein
i02_4815	c5312	/	hypothetical protein
i02_4816	c5313	cysQ	adenosine-3'(2'),5'-bisphosphate nucleotidase
i02_4817	c5314	/	hypothetical protein
i02_4818	c5315	ytfK	hypothetical protein
i02_4819	c5316	ytfL	hypothetical protein
i02_4820	c5317	msrA	methionine sulfoxide reductase A
i02_4821	c5318	ytfM	hypothetical protein
i02_4822	c5319	ytfN	hypothetical protein
i02_4823	c5320	ytfP	hypothetical protein
i02_4824	c5321	/	hypothetical protein
i02_4825	c5322	/	hypothetical protein
i02_4826	c5323	ppa	inorganic pyrophosphatase
i02_4827	c5324	/	hypothetical protein
i02_4828	c5325	ytfQ	ABC transporter periplasmic-binding protein
i02_4829	c5326	ytfR	ABC transporter ATP-binding protein
i02_4830	c5327	ytfT	ABC transporter permease
i02_4831	c5328	yjfF	inner membrane ABC transporter permease protein
i02_4832	c5329	fbp	fructose-1,6-bisphosphatase
i02_4833	c5330	/	hypothetical protein

Clone D	CFT 073	Gene	Product
i02_4834	c5331	yjfG	UDP-N-acetylmuramate:L-alanyl-gamma-D-glutamyl-meso-diaminopimelate ligase
i02_4835	c5332	yjgA	hypothetical protein
i02_4836	c5333	pmbA	peptidase PmbA
i02_4838	c5335	cybC	soluble cytochrome b562 precursor
i02_4837	c5334	/	hypothetical protein
i02_4839	c5336	nrdG	anaerobic ribonucleotide reductase-activating
i02_4840	c5337	nrdD	anaerobic ribonucleoside triphosphate reductase
i02_4841		/	hypothetical protein
i02_4842	c5338	treC	trehalose-6-phosphate hydrolase
i02_4843	c5339	treB	trehalose(maltose)-specific PTS system
i02_4844	c5340	treR	trehalose repressor
i02_4845	c5341	mgtA	magnesium-transporting ATPase MgtA
i02_4846		/	hypothetical protein
	c5343	/	hypothetical protein
i02_4847	c5342	yjgF	hypothetical protein
i02_4848	c5344	pyrI	aspartate carbamoyltransferase regulatory subunit
i02_4849	c5345	pyrB	aspartate carbamoyltransferase catalytic subunit
i02_4850	c5502	pyrL	pyrBI operon leader peptide
i02_4851	c5346	/	hypothetical protein
i02_4852	c5347	/	hypothetical protein
i02_4853	c5348	/	ornithine carbamoyltransferase
i02_4854	c5349	/	carbamate kinase
i02_4855	c5350	/	arginine deiminase
i02_4856	c5351	/	hypothetical protein
i02_4857	c5352	yjgK	hypothetical protein
i02_4859	c5354	yjgD	hypothetical protein
i02_4858	c5353	argI	ornithine carbamoyltransferase subunit F
i02_4860	c5355	/	hypothetical protein
i02_4861	c5356	/	hypothetical protein
i02_4861	c5357	/	hypothetical protein
i02_4862	c5358	valS	valyl-tRNA synthetase
i02_4863	c5359	holC	DNA polymerase III subunit chi
i02_4864	c5360	pepA	leucyl aminopeptidase
	c5361	/	hypothetical protein
i02_4865	c5362	yjgP	hypothetical protein
i02_4866	c5363	yjgQ	hypothetical protein
i02_4867	c5364	yjgR	hypothetical protein
i02_4868	c5365	idnR	L-idonate regulatory protein
i02_4869	c5366	idnT	Gnt-II system L-idonate transporter
i02_4870	c5367	idnO	gluconate 5-dehydrogenase
i02_4871	c5368	idnD	L-idonate 5-dehydrogenase
i02_4872	c5369	idnK	D-gluconate kinase
i02_4873	c5370	yjgB	hypothetical protein
i02_4873a	-	/	prophage P4 integrase
i02_4874	-	/	HNH nuclease
i02_4874a	-	/	conserved hypothetical protein
i02_4874b	-	/	conserved hypothetical protein
i02_4875	-	/	hypothetical protein
i02_4876	-	/	ATPase-like protein
i02_4877	-	/	hypothetical protein

Clone D	CFT 073	Gene	Product
i02_4878	-	/	hypothetical protein
i02_4879	-	/	hypothetical protein
i02_4880	-	/	predicted protein
i02_4881	-	/	hypothetical protein
i02_4882	-	/	transposase IS629
i02_4883	-	/	hypothetical protein
i02_4884	-	/	hypothetical protein
i02_4885	-	/	hypothetical protein
i02_4885a	-	/	antigen 43 precursor (AG43) (Fluffing protein)
i02_4885b	-	/	conserved hypothetical protein
i02_4885c	-	/	conserved hypothetical protein
i02_4885d	-	/	phospholipase, patatin family
i02_4886	-	/	hypothetical protein
i02_4887	-	/	hypothetical protein
i02_4888	-	/	putative radC-like protein yeeS
i02_4889	-	/	hypothetical protein
i02_4890	-	/	hypothetical protein
i02_4891	c5371	/	prophage P4 integrase
i02_4892	c5372	/	hypothetical protein
i02_4893	c5373	/	hypothetical protein
i02_4894	c5374	/	hypothetical protein
	c5375	/	hypothetical protein
i02_4895	c5376	/	hypothetical protein
i02_4896	c5377	/	hypothetical protein
i02_4897	c5378	/	hypothetical protein
	c5380	/	hypothetical protein
i02_4898	c5379	/	hypothetical protein
i02_4900	c5381	/	hypothetical protein
i02_4901	c5382	/	hypothetical protein
	c5383	/	hypothetical protein
i02_4902	c5384	/	hypothetical protein
i02_4903	c5385	/	hypothetical protein
i02_4904	c5386	/	hypothetical protein
i02_4905	c5387	/	hypothetical protein
i02_4906	c5388	/	hypothetical protein
i02_4907	c5389	/	hypothetical protein
i02_4908	c5390	/	hypothetical protein
i02_4909	c5391	fimB	tyrosine recombinase
i02_4910	c5392	fimE	tyrosine recombinase
i02_4911	c5393	fimA	Type-1 fimbrial protein, A chain precursor
i02_4912	c5394	fimI	fimbrin-like protein fimI precursor
i02_4913	c5395	fimC	chaperone protein fimC precursor
i02_4914	c5396	fimD	Outer membrane usher protein fimD precursor
i02_4915	c5397	fimF	FimF protein precursor
	c5398	/	hypothetical protein
i02_4916	c5399	fimG	FimG protein precursor
i02_4917	c5400	fimH	FimH protein precursor
i02_4918	c5401	gntP	fructuronate transporter
i02_4919	c5402	uxuA	mannonate dehydratase
i02_4920	c5403	uxuB	D-mannonate oxidoreductase
i02_4921	c5404	uxuR	DNA-binding transcriptional repressor UxuR
i02_4922	c5405	yjiD	hypothetical protein

Clone D	CFT 073	Gene	Product
i02_4923	c5406	/	hypothetical protein
i02_4924	c5407	yjiE	putative DNA-binding transcriptional regulator
i02_4925	c5408	iadA	isoaspartyl dipeptidase
i02_4926	c5409	yjiG	hypothetical protein
i02_4927	c5410	yjiH	hypothetical protein
i02_4928	c5411	/	hypothetical protein
i02_4929		yjiI	2'-phosphotransferase-like protein
i02_4930		/	hypothetical protein
i02_4931	c5414	yjiJ	hypothetical protein
i02_4932	c5415	yjiK	hypothetical protein
i02_4933	c5416	/	hypothetical protein
i02_4934	c5417	yjiL	hypothetical protein
i02_4935	c5418	yjiM	hypothetical protein
i02_4936	c5419	yjiN	hypothetical protein
i02_4937	c5420	yfcI	hypothetical protein
i02_4938	c5421	/	hypothetical protein
i02_4939	c5422	yjiW	endoribonuclease SymE
i02_4940	c5423	/	putative restriction modification enzyme S
i02_4941	c5424	/	putative restriction modification enzyme M
i02_4942	c5425	/	putative restriction modification enzyme R
i02_4943	c5426	/	hypothetical protein
i02_4944	c5427	yjiA	putative GTP-binding protein YjiA
i02_4945	c5428	/	hypothetical protein
i02_4946	c5429	yjiY	hypothetical protein
i02_4947	c5430	tsr	methyl-accepting chemotaxis protein I
i02_4948	c5431	/	hypothetical protein
i02_4949	c5432	/	hypothetical protein
i02_4950	c5433	/	putative C4-dicarboxylate-binding periplasmic
i02_4950	c5434	/	hypothetical protein
i02_4951	c5435	/	putative Na(+)/H(+) exchanger yjcE
i02_4952	c5436	yjjM	hypothetical protein
i02_4953	c5437	/	hypothetical protein
i02_4954	c5438	mdoB	phosphoglycerol transferase I
i02_4955	c5439	yjjA	hypothetical protein
i02_4956	c5440	dnaC	DNA replication protein DnaC
i02_4957	c5441	dnaT	primosomal protein DnaI
i02_4958	c5442	yjjB	hypothetical protein
i02_4959	c5443	yjjP	hypothetical protein
i02_4960	c5444	yjjQ	hypothetical protein
i02_4961	c5445	bglJ	DNA-binding transcriptional activator BglJ
i02_4962	c5446	fhuF	ferric iron reductase involved in ferric
i02_4963	c5447	/	hypothetical protein
i02_4964	c5448	/	hypothetical protein
i02_4965	c5449	/	hypothetical protein
i02_4966	c5450	rsmC	16S ribosomal RNA m2G1207 methyltransferase
i02_4967	c5452	holD	DNA polymerase III subunit psi
i02_4968	c5453	rimI	ribosomal-protein-alanine N-acetyltransferase
i02_4969	c5454	yjjG	nucleotidase
	c5455	/	hypothetical protein
i02_4970	c5456	prfC	peptide chain release factor 3
i02_4971	c5457	osmY	periplasmic protein
	c5458	/	hypothetical protein

Clone D	CFT 073	Gene	Product
i02_4972	c5459	/	hypothetical protein
i02_4973	c5460	yjjU	hypothetical protein
i02_4974	c5461	yjjV	putative deoxyribonuclease YjjV
i02_4975	c5462	yjjW	hypothetical protein
i02_4977	c5464	/	hypothetical protein
i02_4976	c5463	yjjI	hypothetical protein
i02_4978	c5465	deoC	deoxyribose-phosphate aldolase
i02_4979	c5466	deoA	thymidine phosphorylase
i02_4980	c5467	deoB	phosphopentomutase
i02_4981	c5468	deoD	purine nucleoside phosphorylase
-	c5469	/	transposase
i02_4982	c5470	yjjJ	hypothetical protein
i02_4983	c5471	lplA	lipoate-protein ligase A
i02_4984	c5472	smp	hypothetical protein
i02_4985	c5473	serB	phosphoserine phosphatase
i02_4986	c5474	sms	DNA repair protein RadA
i02_4987	c5475	nadR	nicotinamide-nucleotide adenylyltransferase
i02_4988	c5476	/	hypothetical protein
i02_4989	c5477	/	hypothetical protein
i02_4991		/	hypothetical protein
i02_4990	c5478	yjjK	putative ABC transporter ATP-binding protein
i02_4992	c5479	slt	lytic murein transglycosylase
i02_4993	c5480	trpR	Trp operon repressor
i02_4994	c5481	yjjX	NTPase
i02_4995	c5482	gpmB	phosphoglycerate mutase
i02_4996	c5483	rob	right origin-binding protein
i02_4997	c5484	creA	hypothetical protein
i02_4998	c5485	creB	DNA-binding response regulator CreB
i02_4999	c5486	creC	sensory histidine kinase CreC
i02_5000	c5487	creD	hypothetical protein
i02_5001	c5488	arcA	two-component response regulator
i02_5002	c5489	yjjY	hypothetical protein
i02_5003	c5490	lasT	putative RNA methyltransferase

[a] Some genes were not predicted in one genome and lef as a blank cell. All insertion or deletion of genes are described as "-". In some cases a gene in one genome was annotated as 2 or more genes in the other genome, and the same gene name appears 2 or more times for a genome. This may be due to mutation to stop codon, frameshift mutation or sequencing errors.
