# Supplementary material for: Rates of Mutation and Host Transmission for an Escherichia coli Clone over 3 Years
Source: PLoS One. 2011 Oct 27;6(10):e26907. doi: 10.1371/journal.pone.0026907 (PMC3203180; doi:10.1371/journal.pone.0026907)
Supplement: Table S3 — Large insertions and deletions. Large indels (more than 20 bp) affecting the genomes are shown with the length (bp), if thought to be an insertion or deletion, the strain affected and the gene or genes affected. (PDF) [file pone.0026907.s004.pdf]

Table S3 Large insertions and deletions. Large indels (more than 20 bp) affecting the genomes are shown with the length (bp), if thought to be an insertion or deletion, the strain affected and the gene or genes affected.

Indel No.	Lineage	type[a]	site[b]	size	Name or range
1	CFT073	ins	306	712	IS1541A-like element
2	CFT073	ins	66919	712	transposase
3	CFT073	ins	80507	711	transposase
4	CFT073	ins	128856	1313	hypothetical protein & IS629
5	CFT073	ins	189257	711	transposase
6	clone D	del	232336	113	16S rRNA
7	clone D	del	232516	135	tRNA
8	CFT073	del	232521	149	tRNA
9	clone D	ins	248919	10849	phage-like element (i02_0242-0259)
10	CFT073	ins	261942	2418	ISEc10
11	clone D	ins	294117	27	intergenic
12	CFT073	ins	335584	1313	IS629
13	?	indel	345655	1005	c0363
14	clone D	del	416025	271	intergenic
15	clone D	del	431892	21	intergenic
16	CFT073	ins	543733	711	IS1541A-like element
17	CFT073	ins	750473	712	IS1541A-like element
18	CFT073	del	761604	63	intergenic
19	clone D	del	799684	45	tolA
20	CFT073	ins	908998	33481	prophage(c0932-c0979)
21	CFT073	ins	1179088	1313	IS629 & hypothetical protein
22	CFT073	ins	1199719	2416	ISEc10
23	CFT073	ins	1202231	711	IS1541A-like element
24	CFT073	ins	1273890	711	IS1541A-like element
25	CFT073	ins	1303018	711	IS1541A-like element
26	CFT073	ins	1338505	11452	phage-like element(c1481-c1507)
27	CFT073	ins	1347911	1313	hypothetical protein & IS629
28	CFT073	ins	1350800	2416	hypothetical proteins
29	CFT073	ins	1361387	1313	hypothetical protein & IS629
30	CFT073	ins	1518294	711	IS1541A-like element
31	clone D	del	1608735	589	pseudogene
32	CFT073	del	1608792	441	i02_1656
33	CFT073	ins	1723367	996	oprR
34	CFT073	del	1758310	26	intergenic
35	CFT073	ins	2090661	712	IS1541A-like element
36	CFT073	ins	2173095	711	IS1541A-like element
37	clone D	del	2281154	74	intergenic
38	CFT073	ins	2284297	2415	ISEc10
39	CFT073	del	2344530	193	intergenic

Indel No.	Lineage	type[a]	site[b]	size	Name or range
40	CFT073	ins	2406118	711	IS1541A-like element
41	CFT073	ins	2421164	712	IS1541A-like element
42	clone D	del	2736465	115	tRNA
43	clone D	del	2739276	122	tRNA
44	CFT073	ins	2929246	106	tRNA
45	CFT073	ins	2955545	48433	prophage (c3143-c3206)
46	CFT073	ins	3172163	711	IS1541A-like element
47	CFT073	ins	3217704	711	IS1541A-like element
48	?	indel	3296661	1149	insA & insB
49	CFT073	ins	3297810	19184	part of genomic island containing hlyDBAC (c3559-c3581)
50	clone D	ins	3298577	1234	hypothetical protein & insA &insB
51	clone D	del	3299811	7868	part of genomic island containing papHCDJKEFG (c3583-3591)
52	clone D	ins	3306522	393	intergenic
53	clone D	ins	3313135	2917	transposases & hypothetical proteins
54	CFT073	ins	3316052	2884	hypothetical proteins
55	clone D	ins	3316634	2490	transposases & hypothetical proteins
56	clone D	ins	3326382	23	intergenic
57	clone D	ins	3333568	1334	insC & insD
58	CFT073	ins	3359352	6892	hypothetical proteins
59	clone D	ins	3377402	8335	kfiB-D
60	CFT073	ins	3385737	7970	hypothetical proteins
61	clone D	del	3387380	116	intergenic
62	CFT073	del	3390277	14762	ETEC-like type II secretion gene cluster (gspJIHGFEDC)
63	CFT073	ins	3390277	1618	transposase
64	CFT073	del	3725965	276	tRNA
65	clone D	del	3725965	30	intergenic
66	CFT073	del	3729401	202	tRNA
67	clone D	del	3729603	214	tRNA
68	clone D	ins	4146811	16963	genomic island (i02_4155-4171)
69	CFT073	ins	4163774	66080	genomic island (c4492-c4579)
70	CFT073	del	4384698	290	tRNA
71	clone D	del	4384698	373	tRNA
72	CFT073	del	4522479	280	tRNA
73	clone D	del	4522759	274	tRNA
74	clone D	del	4652694	43	intergenic
75	CFT073	ins	4741230	52136	genomic island (c5143-c5216)
76	clone D	ins	4885076	38017	genomic island (i02_4873a-4890)
77	CFT073	ins	5018007	712	IS1541A-like element

[a], ins, insertion; del, deletion; indel: deletion or insertion

[b], Using the clone D_i2 genome as reference. For indels the base indicated is the base before the insertion or deletion.
