# Supplementary material for: Rates of Mutation and Host Transmission for an Escherichia coli Clone over 3 Years
Source: PLoS One. 2011 Oct 27;6(10):e26907. doi: 10.1371/journal.pone.0026907 (PMC3203180; doi:10.1371/journal.pone.0026907)
Supplement: Table S4 — Large insertions and deletions in clone D relative to CFT073: allocation to lineage by virtual outgroup analysis. The E. coli/Shigella genomes used for the analysis are shown, with details of the blocks present in outgroup genomes and genomes under analysis, and also the final allocation and a measure of support level for that allocation. (PDF) [file pone.0026907.s005.pdf]

Table S4 Large insertions and deletions in clone D relative to CFT073: allocation to lineage by virtual outgroup analysis. The *E. coli* /*Shigella* genomes used for the analysis are shown, with details of the blocks present in outgroup genomes and genomes under analysis, and also the final allocation and a measure of support level for that allocation.

Indel No.	Clone D[a]	CFT073[a]	Lineage	type	site[b]	size	Inferred ancestral[c]	Support level[d]	ED1a	536	S88	APEC O1	UTI89	E2348/69	ATCC8739	K-12	HS	SMS3-5	IAI39	E24377A	SE11	55989	IAI1	Sakai	UMN026	B4 Sb227	F5 8401	SS Ss046	D1 Sd197	B18 BS512	F2a 2457T	F2a 301
1	-	+	CFT073	ins	306	712	-	++++	-	-	-	-	-	-	-	-	-	-	-	-	-	-	-	-	-	-	-	-	-	-	-	-
2	-	+	CFT073	ins	66919	712	-	++++	-	-	-	-	-	-	-	-	-	-	-	-	-	-	-	-	-	-	-	-	-	-	-	-
3	-	+	CFT073	ins	80507	711	-	++++	-	-	-	-	-	-	-	-	-	-	-	-	-	-	-	-	-	-	-	-	-	-	-	-
4	-	+	CFT073	ins	128856	1313	-	++++	-	-	-	-	-	-	-	-	-	-	/	+	-	-	-	-	-	-	-	+	-	-	-	-
5	-	+	CFT073	ins	189257	711	-	++++	-	-	-	-	-	-	-	-	-	-	-	-	-	-	-	-	-	-	-	-	-	-	-	-
6	-	+	clone D	del	232336	113	+	++++	+	+	+	+	+	+	-	+	+	+	+	+	+	+	+	+	+	+	+	+	+	+	+	+
7	-	+	clone D	del	232516	135	+	++++	+	+	+	+	+	+	-	+	+	+	+	+	+	+	+	+	+	+	+	+	+	+	+	+
8	+	-	CFT073	del	232521	149	+	++++	+	+	+	+	+	-	+	+	+	+	+	+	+	+	+	+	+	+	-	+	+	+	+	+
9	+	-	clone D	ins	248919	10849	-	++++	-	/	/	/	-	/	/	/	/	/	/	-	/	-	/	/	-	/	/	-	-	/	/	-
10	-	+	CFT073	ins	261942	2418	-	++++	-	-	-	-	-	-	/	/	/	-	-	-	/	-	-	-	-	-	/	/	-	-	-	-
11	+	-	clone D	ins	294117	27	-	++	-	/	/	/	/	/	/	/	/	/	/	/	/	/	/	/	/	/	/	/	/	/	/	/
12	-	+	CFT073	ins	335584	1313	-	+++	-	-	/	-	-	/	/	/	/	-	/	/	/	-	/	/	/	/	/	/	/	/	/	/
13	-	+	?	indel	345655	1005	?	+/-	/	/	/	/	/	/	/	/	/	/	/	/	/	/	/	/	/	/	/	/	/	/	/	/
14	-	+	clone D	del	416025	271	+	++++	/	+	+	+	+	/	/	/	+	/	/	/	+	/	/	+	/	/	/	/	/	/	/	/
15	-	+	clone D	del	431892	21	+	++	+	-	-	-	-	+	+	+	+	-	-	+	-	-	-	+	-	/	/	-	/	/	/	/
16	-	+	CFT073	ins	543733	711	-	++++	-	-	-	-	-	-	-	-	-	-	-	-	-	-	-	-	-	-	-	-	-	-	-	-
17	-	+	CFT073	ins	750473	712	-	++++	-	-	-	-	-	-	-	-	-	-	-	-	-	-	-	-	-	-	-	-	-	-	-	-
18	+	-	CFT073	del	761604	63	+	++	+	+	+	+	+	-	-	-	/	-	-	+	+	+	+	+	-	-	+	+	-	-	+	+
19	-	+	clone D	del	799684	45	+	++++	+	+	+	+	+	+	+	+	+	+	+	+	+	+	+	+	+	+	+	+	+	-	+	+
20	-	+	CFT073	ins	908998	33481	-	++++	-	-	-	-	-	+	-	-	+	-	-	-	-	-	-	-	-	-	-	-	-	-	-	-

Indel No.	Clone D[a]	CFT073[a]	Lineage	type	site[b]	size	Inferred ancestral[c]	Support level[d]	ED1a	536	S88	APEC O1	UTI89	E2348/69	ATCC8739	K-12	HS	SMS3-5	IAI39	E24377A	SE11	55989	IAI1	Sakai	UMN026	B4 Sb227	F5 8401	SS Ss046	D1 Sd197	B18 BS512	F2a 2457T	F2a 301
21	-	+	CFT073	ins	1179088	1313	-	++++	/	-	/	-	-	-	/	-	/	-	/	/	/	-	/	/	/	/	/	/	-	/	/	-
22	-	+	CFT073	ins	1199719	2416	-	++++	-	-	-	-	-	-	/	-	/	-	-	-	/	-	/	-	-	-	/	/	-	-	-	-
23	-	+	CFT073	ins	1202231	711	-	++++	-	-	-	-	-	-	/	-	/	-	-	-	/	-	/	-	-	-	/	/	-	/	-	-
24	-	+	CFT073	ins	1273890	711	-	++++	-	-	-	-	-	-	-	-	-	-	-	-	-	-	-	-	-	-	-	-	-	-	-	-
25	-	+	CFT073	ins	1303018	711	-	++++	-	/	-	-	-	-	/	/	/	/	/	/	/	-	/	-	/	/	-	-	-	-	-	-
26	-	+	CFT073	ins	1338505	11452	-	++++	-	-	-	-	-	-	-	-	-	-	-	-	-	-	-	-	-	-	-	-	-	-	-	-
27	-	+	CFT073	ins	1347911	1313	-	++++	/	-	-	-	-	-	/	/	/	/	/	/	-	/	/	-	-	-	/	/	/	/	/	-
28	-	+	CFT073	ins	1350800	2416	-	++++	-	-	-	-	-	/	/	/	/	/	-	/	/	-	-	-	-	/	/	/	/	/	/	/
29	-	+	CFT073	ins	1361387	1313	-	++++	-	/	-	-	-	-	/	-	/	/	-	+	-	-	-	-	-	-	/	/	/	/	/	/
30	-	+	CFT073	ins	1518294	711	-	++++	-	-	-	-	-	-	-	-	-	-	-	-	-	-	-	-	-	-	-	-	-	-	-	-
31	-	+	clone D	del	1608735	589	+	++++	+	+	+	+	+	/	+	+	+	/	/	/	/	/	/	/	/	-	/	/	/	/	/	/
32	+	-	CFT073	del	1608792	441	+	++++	+	+	+	+	+	/	+	+	+	/	/	/	/	/	/	/	/	/	/	/	-	/	/	/
33	-	+	CFT073	ins	1723367	996	-	++++	/	-	-	-	-	-	/	-	/	/	/	/	/	/	-	/	/	-	/	/	/	/	/	-
34	+	-	CFT073	del	1758310	26	+	++++	+	+	+	+	+	+	+	+	+	+	+	+	+	+	+	+	+	/	+	/	+	/	+	+
35	-	+	CFT073	ins	2090661	712	-	++++	-	/	-	-	-	-	-	-	-	-	-	-	-	-	-	-	-	-	-	-	-	-	-	-
36	-	+	CFT073	ins	2173095	711	-	++++	-	-	-	-	-	/	/	/	/	/	-	/	/	-	/	/	-	/	/	/	/	/	/	/
37	-	+	clone D	del	2281154	74	+	++++	+	+	+	+	+	/	/	/	-	+	/	+	/	+	/	/	+	/	/	/	/	/	/	/
38	-	+	CFT073	ins	2284297	2415	-	++++	/	-	-	-	-	/	/	/	/	-	/	-	-	-	/	/	-	-	/	/	/	/	-	/
39	+	-	CFT073	del	2344530	193	+	++	+	+	-	-	+	-	-	+	/	-	/	-	-	-	+	+	-	+	-	/	/	-	-	+
40	-	+	CFT073	ins	2406118	711	-	++++	/	-	-	-	-	-	-	-	-	-	-	-	-	-	-	-	-	-	-	-	-	-	-	-
41	-	+	CFT073	ins	2421164	712	-	++++	-	-	-	-	-	-	-	-	-	-	-	-	-	-	-	-	-	-	-	-	-	-	-	-
42	-	+	clone D	del	2736465	115	+	++++	+	+	+	+	+	+	+	+	+	+	+	+	+	+	+	+	+	+	+	+	+	+	+	+
43	-	+	clone D	del	2739276	122	+	++	+	+	+	+	+	+	+	+	+	+	+	+	+	+	+	+	+	+	-	+	-	+	-	-
44	-	+	CFT073	ins	2929246	106	-	++	-	+	-	-	-	-	+	+	+	-	-	+	+	+	+	-	-	-	-	-	-	+	-	-
45	-	+	CFT073	ins	2955545	48433	-	++	-	-	-	-	-	/	+	-	-	-	-	-	-	-	-	-	-	-	/	/	-	-	-	-

Indel No.	Clone D[a]	CFT073[a]	Lineage	type	site[b]	size	Inferred ancestral[c]	Support level[d]	ED1a	536	S88	APEC O1	UTI89	E2348/69	ATCC8739	K-12	HS	SMS3-5	IAI39	E24377A	SE11	55989	IAI1	Sakai	UMN026	B4 Sb227	F5 8401	SS Ss046	D1 Sd197	B18 BS512	F2a 2457T	F2a 301
46	-	+	CFT073	ins	3172163	711	-	++++	-	-	-	-	-	-	-	-	-	-	-	-	-	-	-	-	-	-	-	-	-	-	-	-
47	-	+	CFT073	ins	3217704	711	-	++++	-	-	-	-	-	-	-	-	-	-	-	-	-	-	-	-	-	-	-	-	-	-	-	-
48	+	-	?	indel	3296661	1149	?	+/-	/	-	+	+	/	/	/	/	/	/	+	/	/	/	/	/	-	/	/	/	/	-	/	/
49	-	+	CFT073	ins	3297810	19184	-	+++	-	-	-	-	-	/	/	/	/	/	-	/	/	/	/	/	-	/	/	/	/	/	/	/
50	+	-	clone D	ins	3298577	1234	-	+++	/	-	-	-	-	/	/	/	/	/	+	/	/	/	/	/	-	/	/	/	/	-	/	/
51	-	+	ClonD	del	3299811	7868	+	+++	/	+	+	+	+	/	/	/	/	/	+	/	/	/	/	/	+	/	/	/	/	/	/	/
52	+	-	clone D	ins	3306522	393	-	++++	/	/	/	-	/	/	/	-	/	-	+	-	-	/	/	/	-	-	-	-	-	-	-	-
53	+	-	clone D	ins	3313135	2917	-	++++	-	/	/	/	/	/	-	/	/	/	/	-	/	-	/	/	-	-	-	-	-	/	-	-
54	-	+	CFT073	ins	3316052	2884	-	++++	/	/	/	-	-	/	/	-	/	-	-	+	-	-	/	-	+	-	-	-	-	-	/	-
55	+	-	clone D	ins	3316634	2490	-	+++	-	/	/	/	/	/	/	/	/	/	/	/	/	-	/	/	-	-	/	/	/	-	/	/
56	+	-	clone D	ins	3326382	23	-	++	-	/	/	+	/	/	/	/	/	+	+	/	/	-	/	/	-	-	-	-	/	-	-	-
57	+	-	clone D	ins	3333568	1334	-	++++	-	/	/	-	/	/	/	/	/	-	+	/	/	-	/	/	-	+	-	-	/	/	-	-
58	-	+	CFT073	ins	3359352	6892	-	++++	-	-	-	/	-	/	/	-	/	-	/	-	/	-	/	-	-	-	/	/	-	/	-	-
59	+	-	clone D	ins	3377402	8335	-	+++	-	/	-	-	-	/	/	/	/	-	-	/	/	/	/	/	-	/	/	/	/	/	/	/
60	-	+	CFT073	ins	3385737	7970	-	++++	-	/	-	-	-	/	/	/	/	-	-	/	/	-	/	/	-	/	/	/	/	/	/	/
61	-	+	clone D	del	3387380	116	+	++	+	-	-	-	-	/	/	/	/	+	-	/	/	/	/	/	-	/	/	/	/	/	/	/
62	+	-	CFT073	del	3390277	14762	+	++	+	+	+	+	+	+	-	-	+	+	+	+	+	+	+	/	+	-	-	/	-	-	-	-
63	-	+	CFT073	ins	3390277	1618	-	++++	-	-	-	-	-	-	/	-	-	-	-	-	-	-	-	/	-	/	-	/	-	/	/	-
64	+	-	CFT073	del	3725965	276	+	++++	+	-	+	+	+	+	+	+	+	+	+	+	+	-	+	+	+	+	+	+	+	+	+	+
65	-	+	clone D	del	3725965	30	+	++	+	+	+	+	+	+	+	-	+	-	+	+	+	+	+	+	+	+	+	+	+	-	+	+
66	+	-	CFT073	del	3729401	202	+	++++	+	+	+	+	+	-	+	+	+	+	+	+	+	+	+	+	+	+	-	+	+	+	+	+
67	-	+	clone D	del	3729603	214	+	++	+	+	+	+	+	+	+	-	+	+	+	+	-	-	-	+	+	+	+	+	+	+	+	+
68	+	-	clone D	ins	4146811	16963	-	++++	-	-	-	/	-	/	/	/	/	/	-	/	/	/	/	/	-	/	-	-	/	/	-	/
69	-	+	CFT073	ins	4163774	66080	-	++++	-	-	/	/	/	-	/	/	/	/	-	/	/	/	/	-	-	-	-	/	/	/	/	/
70	+	-	CFT073	del	4384698	290	+	++++	+	+	+	+	+	-	+	+	+	+	+	+	+	+	+	+	+	+	+	+	+	+	+	+

Indel No.	Clone D[a]	CFT073[a]	Lineage	type	site[b]	size	Inferred ancestral[c]	Support level[d]	ED1a	536	S88	APEC O1	UTI89	E2348/69	ATCC8739	K-12	HS	SMS3-5	IAI39	E24377A	SE11	55989	IAI1	Sakai	UMN026	B4 Sb227	F5 8401	SS Ss046	D1 Sd197	B18 BS512	F2a 2457T	F2a 301
71	-	+	clone D	del	4384698	373	+	++++	+	+	+	+	+	+	+	+	+	+	+	+	+	+	+	+	+	+	-	+	+	+	+	+
72	+	-	CFT073	del	4522479	280	+	++++	+	+	+	+	+	+	+	+	+	+	+	+	+	+	+	+	+	+	-	+	+	+	+	+
73	-	+	clone D	del	4522759	274	+	++++	+	+	+	+	+	+	+	+	+	+	+	+	+	+	+	+	+	+	+	+	+	+	+	+
74	-	+	clone D	del	4652694	43	+	++++	+	+	+	+	+	+	+	+	+	+	+	+	+	+	+	+	+	-	+	+	+	+	+	+
75	-	+	CFT073	ins	4741230	52136	-	++++	-	/	/	-	/	-	-	-	-	-	-	-	-	-	-	-	-	-	-	-	-	-	-	-
76	+	-	clone D	ins	4885076	38017	-	++++	-	-	-	-	-	-	/	-	-	-	-	-	/	-	-	-	-	-	/	-	/	-	/	-
77	-	+	CFT073	ins	5018007	712	-	++++	-	-	-	-	-	-	/	-	-	-	-	-	-	-	-	-	-	-	-	-	-	-	-	-

[a] "+" indicates the block present, "-" indicates the block absent, "/" indicates the block and nearby sequence absent.

[b] Using the clone D_i2 genome as reference.For indels the base indicated is the base before the insertion or deletion.

[c] Presence or absence of indel block in clone D/CFT073 ancestor as inferred from outgroup analysis.

[d] Level of support for allocation of indel blocks as given in previous column

"++++" agreement is high-8 or more outgroup strains with expected type and at most 2 with the opposite, and ED1a agrees or site not present

"+++" agreement good-4 or more outgroup strains with expected type and at most 2 with the opposite, and ED1a agrees or site not present

"++" agreement in ED1a regardless of situation with other outgroup strains

"+" no conflict but very limited support as either the block and nearby sequence absent in ED1a, and/or outgroup support less than given above

"+/-" no conflict but no support (the block and its nearby sequence not present in any outgroup OR both alternative lineages supported equally).
