# Supplementary material for: Rates of Mutation and Host Transmission for an Escherichia coli Clone over 3 Years
Source: PLoS One. 2011 Oct 27;6(10):e26907. doi: 10.1371/journal.pone.0026907 (PMC3203180; doi:10.1371/journal.pone.0026907)
Supplement: Table S5 — Small insertions and deletions. Small indels (not more than 20 bp) affecting the genomes are shown with the length (bp), if thought to be an insertion or deletion, the strain affected and the genes affected. (PDF) [file pone.0026907.s006.pdf]

Table S5 Small insertions and deletions. Small indels (not more than 20 bp) affecting the genomes are shown with the length (bp), if thought to be an insertion or deletion, the strain affected and the genes affected.

Indel No.	Lineage	type[a]	site[b]	size	Locus_tag	Gene
78	CFT073	del	45543	1	i02_0044	fixB
79	clone D	del	72763	1	intergenic	
80	CFT073	ins	133186	1	i02_0131	aceF
81	clone D	ins	144870	6	i02_0139	yacK
82	clone D	ins	154523	12	i02_0149	yadD
83	CFT073	ins	155811	1	i02_0151	panC
84	CFT073	ins	199713	1	i02_0193	dxr
85	CFT073	del	227763	3	i02_0220	metQ
86	CFT073	del	232061	1	rRNA_001	rrs
87	CFT073	ins	232066	1	rRNA_001	rrs
88	CFT073	ins	233460	1	rRNA_002	rrl
89	CFT073	del	233464	3	rRNA_002	rrl
90	CFT073	ins	233469	3	rRNA_002	rrl
91	CFT073	ins	234089	1	rRNA_002	rrl
92	CFT073	del	234785	1	rRNA_002	rrl
93	CFT073	del	234789	1	rRNA_002	rrl
94	CFT073	ins	272526	1	i02_0280	
95	CFT073	ins	273259	1	i02_0281	
96	?	indel	327228	1	intergenic	
97	?	indel	327823	1	intergenic	
98	?	indel	347908	1	i02_0343	
99	clone D	ins	376418	2	i02_0372	
100	CFT073	ins	390718	1	intergenic	
101	CFT073	ins	390903	1	i02_0391	
102	CFT073	del	390967	1	i02_0391	
103	CFT073	ins	390984	1	i02_0391	
104	CFT073	del	391060	1	i02_0391	
105	CFT073	ins	416956	1	intergenic	
106	CFT073	ins	431814	1	i02_0427	
107	CFT073	ins	431828	1	i02_0427	
108	CFT073	ins	453588	1	intergenic	
109	CFT073	del	453852	1	i02_0444	lacI
110	CFT073	ins	453931	1	i02_0444	lacI
111	CFT073	ins	593588	1	i02_0588	ybbM
112	CFT073	ins	599774	1	i02_0594	ybbB
113	CFT073	ins	626234	1	i02_0618	ybcI
114	CFT073	del	793305	1	i02_0779	
115	CFT073	ins	793475	1	i02_0780	

Indel No.	Lineage	type[a]	site[b]	size	Locus_tag	Gene
116	CFT073	ins	802982	1	i02_0791	ybgF
117	CFT073	ins	942703	1	i02_0931	
118	CFT073	ins	970076	1	i02_0953	
119	CFT073	ins	986199	1	i02_0965	lpxK
120	CFT073	del	1066831	1	i02_1039	agp
121	CFT073	ins	1066931	1	i02_1039	agp
122	CFT073	ins	1081238	1	i02_1055	
123	CFT073	ins	1129853	1	intergenic	
124	?	indel	1132746	1	intergenic	
125	CFT073	ins	1141301	1	i02_1117	
126	CFT073	ins	1203554	1	i02_1181	ycdW
127	CFT073	ins	1232838	1	i02_1220	
128	CFT073	ins	1232865	1	i02_1220	
129	CFT073	ins	1232932	1	i02_1220	
130	CFT073	ins	1256746	1	i02_1247	
131	CFT073	ins	1256789	1	i02_1247	
132	CFT073	ins	1256798	1	i02_1247	
133	CFT073	del	1259736	1	i02_1250	pabC
134	CFT073	ins	1259830	1	i02_1251	yceG
135	CFT073	ins	1344697	1	i02_1357	
136	CFT073	del	1346650	12	intergenic	
137	CFT073	ins	1358441	1	i02_1383	
138	CFT073	ins	1358461	1	i02_1383	
139	CFT073	ins	1358472	1	i02_1383	
140	CFT073	ins	1358524	1	i02_1383	
141	CFT073	ins	1358753	1	i02_1384	
142	CFT073	del	1378275	1	i02_1415	
143	CFT073	del	1378427	1	i02_1415	
144	CFT073	del	1378444	1	i02_1415	
145	CFT073	del	1378515	1	i02_1415	
146	CFT073	del	1393522	1	i02_1430	sitA
147	CFT073	del	1393546	1	i02_1430	sitA
148	CFT073	del	1393554	1	i02_1430	sitA
149	CFT073	del	1393563	1	i02_1430	sitA
150	CFT073	del	1393615	1	i02_1430	sitA
151	CFT073	del	1393636	1	i02_1430	sitA
152	CFT073	del	1529699	1	i02_1580	pyrF
153	clone D	del	1541419	1	i02_1593	
154	CFT073	ins	1649103	1	i02_1691	ydcV
155	CFT073	ins	1654282	1	i02_1701	yncD
156	?	indel	1661875	1	i02_1709	
157	?	indel	1662780	1	intergenic	
158	CFT073	del	1668910	1	intergenic	
159	CFT073	del	1670104	1	i02_1717	

Indel No.	Lineage	type[a]	site[b]	size	Locus_tag	Gene
160	CFT073	del	1688286	3	intergenic	
161	CFT073	ins	1693716	1	i02_1740	
162	CFT073	ins	1694715	1	i02_1740	
163	CFT073	del	1701417	1	intergenic	
164	CFT073	ins	1724309	1	i02_1761	hipA
165	CFT073	ins	1895916	1	i02_1932	pheT
166	clone D	ins	1961664	1	intergenic	
167	clone D	ins	1961686	1	intergenic	
168	CFT073	ins	2010211	1	intergenic	
169	CFT073	ins	2010225	3	intergenic	
170	CFT073	ins	2010257	1	intergenic	
171	CFT073	ins	2025584	1	i02_2078	purK
172	CFT073	del	2025609	1	i02_2078	purK
173	clone D	ins	2071197	6	i02_2126	yecG
174	clone D	del	2148157	1	intergenic	
175	CFT073	del	2158529	1	intergenic	
176	clone D	ins	2173925	9	i02_2241	
177	CFT073	ins	2177218	1	i02_2242	
178	CFT073	del	2180042	1	i02_2242	
179	CFT073	del	2180052	2	i02_2242	
180	CFT073	del	2180060	2	i02_2242	
181	CFT073	del	2187699	1	i02_2246	
182	clone D	del	2190262	1	intergenic	
183	CFT073	del	2194430	1	i02_2251	
184	CFT073	del	2194446	1	i02_2251	
185	CFT073	ins	2212027	1	i02_2264	
186	CFT073	ins	2249329	1	i02_2279	
187	CFT073	del	2255272	16	intergenic	
188	CFT073	ins	2272088	1	intergenic	
189	CFT073	ins	2272119	1	intergenic	
190	CFT073	ins	2272141	1	intergenic	
191	CFT073	ins	2281124	1	intergenic	
192	clone D	del	2289140	1	i02_2318	
193	clone D	del	2297026	1	intergenic	
194	CFT073	del	2362248	1	i02_2386	
195	CFT073	del	2362290	1	i02_2386	
196	CFT073	del	2362303	1	i02_2386	
197	CFT073	del	2362896	1	i02_2387	
198	CFT073	ins	2485913	2	i02_2500	yeiN
199	CFT073	del	2658778	1	i02_2665	yfcJ
200	CFT073	del	2658814	1	i02_2665	yfcJ
201	?	indel	2686052	1	i02_2693	
202	?	indel	2686086	1	i02_2693	
203	?	indel	2686164	18	i02_2693	

Indel No.	Lineage	type[a]	site[b]	size	Locus_tag	Gene
204	CFT073	del	2718016	1	i02_2714	
205	CFT073	del	2751344	1	i02_2746	cysK
206	CFT073	ins	2751413	1	intergenic	
207	CFT073	ins	2751487	1	intergenic	
208	CFT073	ins	2752054	1	i02_2749	ptsI
209	clone D	ins	2852951	9	i02_2837	pbpC
210	CFT073	ins	2885705	1	i02_2869	
211	CFT073	ins	2917217	1	intergenic	
212	CFT073	ins	2926828	1	rRNA_005	rrl
213	CFT073	del	2927085	1	rRNA_005	rrl
214	CFT073	del	2927088	1	rRNA_005	rrl
215	CFT073	del	2927224	13	rRNA_005	rrl
216	clone D	del	2927237	13	rRNA_005	rrl
217	CFT073	ins	2927786	1	rRNA_005	rrl
218	CFT073	ins	2928406	3	rRNA_005	rrl
219	CFT073	del	2928408	3	rRNA_005	rrl
220	CFT073	ins	2928417	1	rRNA_005	rrl
221	clone D	del	2928977	2	intergenic	
222	CFT073	del	2928978	16	intergenic	
223	clone D	del	2928978	5	intergenic	
224	CFT073	del	2929090	13	intergenic	
225	?	indel	2929104	13	intergenic	
226	CFT073	del	2929226	20	intergenic	
227	CFT073	del	2929450	1	rRNA_006	rrs
228	CFT073	del	2929550	1	rRNA_006	rrs
229	CFT073	ins	2930036	1	rRNA_006	rrs
230	CFT073	ins	2930063	1	rRNA_006	rrs
231	CFT073	ins	2930079	1	rRNA_006	rrs
232	CFT073	ins	2930102	1	rRNA_006	rrs
233	CFT073	ins	2930210	1	rRNA_006	rrs
234	CFT073	ins	2930267	1	rRNA_006	rrs
235	CFT073	ins	2930289	1	rRNA_006	rrs
236	CFT073	ins	2930305	1	rRNA_006	rrs
237	CFT073	ins	2930427	1	rRNA_006	rrs
238	CFT073	ins	2930474	1	rRNA_006	rrs
239	CFT073	ins	2930497	1	rRNA_006	rrs
240	CFT073	del	2969602	1	i02_2955	
241	CFT073	del	2969636	1	intergenic	
242	CFT073	ins	2978621	1	intergenic	
243	CFT073	ins	2992303	1	i02_2981	alaS
244	CFT073	ins	3004393	1	i02_2995	norV
245	CFT073	ins	3038925	5	i02_3034	
246	CFT073	ins	3045109	2	intergenic	
247	clone D	del	3068496	15	i02_3065	

Indel No.	Lineage	type[a]	site[b]	size	Locus_tag	Gene
248	CFT073	ins	3082607	1	i02_3076	ygcX
249	CFT073	ins	3082619	1	i02_3076	ygcX
250	CFT073	del	3100943	1	i02_3096	fucK
251	CFT073	del	3134313	1	i02_3121	
252	CFT073	del	3134331	1	i02_3121	
253	CFT073	del	3261304	1	i02_3238	
254	CFT073	del	3261317	1	i02_3238	
255	CFT073	del	3261350	2	i02_3238	
256	CFT073	del	3261357	1	i02_3238	
257	CFT073	del	3296532	12	i02_3275	
258	?	indel	3298504	1	intergenic	
259	CFT073	del	3300102	1	intergenic	
260	clone D	del	3300420	1	i02_3284	
261	CFT073	del	3302284	1	i02_3287	
262	?	indel	3303805	8	i02_3289	
263	CFT073	del	3307276	1	i02_3294	
264	clone D	del	3311423	5	intergenic	
265	clone D	del	3326445	1	intergenic	
266	CFT073	del	3336055	1	i02_3326	
267	CFT073	del	3336093	1	i02_3326	
268	CFT073	del	3336107	1	i02_3326	
269	CFT073	del	3336128	1	i02_3326	
270	CFT073	del	3336135	1	i02_3326	
271	CFT073	del	3336142	1	i02_3326	
272	CFT073	del	3336159	1	i02_3326	
273	CFT073	del	3336176	1	i02_3326	
274	CFT073	del	3336181	1	i02_3326	
275	CFT073	del	3336202	1	i02_3326	
276	CFT073	del	3336311	1	i02_3326	
277	CFT073	ins	3337181	1	i02_3328	
278	CFT073	ins	3337246	1	i02_3328	
279	CFT073	ins	3337294	1	i02_3328	
280	clone D	del	3344259	1	intergenic	
281	CFT073	ins	3344306	2	intergenic	
282	CFT073	ins	3344350	2	intergenic	
283	CFT073	ins	3344364	1	intergenic	
284	CFT073	ins	3344381	1	intergenic	
285	CFT073	del	3348189	1	i02_3340	
286	CFT073	del	3348569	1	intergenic	
287	CFT073	del	3349813	1	intergenic	
288	?	indel	3350480	5	intergenic	
289	?	indel	3350750	14	intergenic	
290	CFT073	ins	3350827	7	i02_3344	
291	?	indel	3350918	6	intergenic	

Indel No.	Lineage	type[a]	site[b]	size	Locus_tag	Gene
292	?	indel	3359351	1	intergenic	
293	CFT073	del	3364637	1	i02_3359	
294	clone D	del	3364699	1	i02_3359	
295	CFT073	ins	3367923	1	i02_3364	
296	clone D	ins	3387223	1	intergenic	
297	CFT073	ins	3387673	1	intergenic	
298	CFT073	del	3389261	1	i02_3380	gspL
299	CFT073	ins	3415674	5	i02_3402	yghO
300	clone D	del	3415911	1	intergenic	
301	CFT073	ins	3450140	1	i02_3439	yqhG
302	CFT073	ins	3525549	1	i02_3514	
303	CFT073	ins	3525566	1	i02_3514	
304	CFT073	del	3561339	1	i02_3550	yqjF
305	CFT073	ins	3562225	2	i02_3551	yqjG
306	CFT073	ins	3567159	1	i02_3557	
307	CFT073	ins	3629787	1	i02_3621	hflB
308	CFT073	del	3704908	3	i02_3698	
309	CFT073	del	3726492	1	rRNA_008	rrl
310	CFT073	ins	3728030	1	rRNA_008	rrl
311	CFT073	ins	3728661	1	rRNA_008	rrl
312	CFT073	ins	3729221	2	intergenic	
313	CFT073	del	3729227	11	intergenic	
314	CFT073	ins	3809580	1	i02_3825	yhfZ
315	clone D	del	3913324	6	i02_3927	ftsY
316	CFT073	del	3933212	1	i02_3950	
317	CFT073	ins	4141078	2	intergenic	
318	clone D	ins	4145486	1	intergenic	
319	clone D	ins	4145592	1	intergenic	
320	?	indel	4145717	1	intergenic	
321	CFT073	ins	4196764	1	i02_4207	dgoA
322	CFT073	ins	4199528	1	i02_4210	
323	CFT073	del	4199642	1	i02_4210	
324	CFT073	ins	4206704	1	i02_4217	yfgE
325	CFT073	ins	4266091	1	rRNA_010	rrs
326	CFT073	ins	4267075	1	rRNA_011	rrl
327	CFT073	ins	4267207	1	rRNA_011	rrl
328	CFT073	del	4267213	5	rRNA_011	rrl
329	CFT073	ins	4267220	5	rRNA_011	rrl
330	CFT073	ins	4267838	1	rRNA_011	rrl
331	CFT073	ins	4268433	1	rRNA_011	rrl
332	CFT073	ins	4272677	1	i02_4279	yifB
333	CFT073	ins	4308484	1	intergenic	
334	clone D	del	4310013	6	i02_4315	hemX
335	CFT073	ins	4383360	1	rRNA_013	rrs

Indel No.	Lineage	type[a]	site[b]	size	Locus_tag	Gene
336	CFT073	ins	4385386	1	rRNA_014	rrl
337	CFT073	ins	4385393	1	rRNA_014	rrl
338	CFT073	ins	4386142	1	rRNA_014	rrl
339	CFT073	ins	4386250	1	rRNA_014	rrl
340	CFT073	del	4394962	1	i02_4402	xni
341	clone D	ins	4520822	1	intergenic	
342	clone D	del	4520842	1	intergenic	
343	clone D	del	4520877	1	intergenic	
344	CFT073	ins	4522159	1	rRNA_016	rrs
345	CFT073	ins	4522197	1	rRNA_016	rrs
346	CFT073	ins	4522200	1	rRNA_016	rrs
347	CFT073	ins	4522223	1	rRNA_016	rrs
348	CFT073	ins	4522239	1	rRNA_016	rrs
349	CFT073	ins	4522262	2	rRNA_016	rrs
350	CFT073	ins	4522267	1	rRNA_016	rrs
351	CFT073	ins	4523421	1	rRNA_017	rrl
352	CFT073	del	4523427	2	rRNA_017	rrl
353	CFT073	ins	4523431	1	rRNA_017	rrl
354	CFT073	ins	4523432	1	rRNA_017	rrl
355	CFT073	ins	4524052	1	rRNA_017	rrl
356	CFT073	del	4524601	13	rRNA_017	rrl
357	CFT073	ins	4524614	13	rRNA_017	rrl
358	CFT073	del	4524748	1	rRNA_017	rrl
359	CFT073	del	4524752	1	rRNA_017	rrl
360	CFT073	ins	4562895	1	intergenic	
361	CFT073	ins	4564223	1	rRNA_020	rrl
362	CFT073	ins	4564736	1	rRNA_020	rrl
363	CFT073	ins	4564756	1	rRNA_020	rrl
364	CFT073	ins	4634107	1	intergenic	
365	clone D	ins	4671388	1	intergenic	
366	clone D	ins	4680413	10	intergenic	
367	CFT073	del	4790012	1	i02_4776	yjfL
368	CFT073	del	4790954	1	i02_4778	yjfC
369	CFT073	ins	4868672	1	i02_4861	
370	CFT073	ins	4988920	1	i02_4950	
371	CFT073	ins	4988958	1	i02_4950	
372	clone D	del	5038168	1	i02_5003	lasT

[a], ins, insertion; del, deletion; indel: deletion or insertion.

[b], Using the clone D_i2 genome as reference.For indels the base indicated is the base before the insertion or deletion.
