# Supplementary material for: Rates of Mutation and Host Transmission for an Escherichia coli Clone over 3 Years
Source: PLoS One. 2011 Oct 27;6(10):e26907. doi: 10.1371/journal.pone.0026907 (PMC3203180; doi:10.1371/journal.pone.0026907)
Supplement: Table S6 — Small insertions and deletions in clone D relative to CFT073: allocation to lineage by virtual outgroup analysis. The E. coli/Shigella genomes used for the analysis are shown, with details of the blocks present in outgroup genomes and genomes under analysis, and also the final allocation and a measure of support level for that allocation. (PDF) [file pone.0026907.s007.pdf]

Table S6 Small insertions and deletions in clone D relative to CFT073: allocation to lineage by virtual outgroup analysis. The *E. coli* /*Shigella* genomes used for the analysis are shown, with details of the blocks present in outgroup genomes and genomes under analysis, and also the final allocation and a measure of support level for that allocation.

Indel No.	Clone D[a]	CFT073 [a]	Lineage	type	site[b]	size	Locus_tag	Gene	Inferred ancestral[c]	Support level[d]	ED1a	536	S88	APEC O1	UTI89	E2348/69	ATCC8739	K-12	HS	SMS3-5	IAI39	E24377A	SE11	55989	IAI1	Sakai	UMN026	B4 Sb227	F5 8401	SS Ss046	D1 Sd197	B18 BS512	F2a 2457T	F2a 301
78	+	-	CFT073	del	45543	1	i02_0044	fixB	+	++++	+	+	+	+	+	+	+	+	+	+	+	+	+	+	+	+	+	/	+	+	+	/	+	+
79	-	+	clone D	del	72763	1	intergenic		+	+++	+	+	+	+	+	/	/	/	/	+	+	/	/	/	/	/	/	/	/	/	/	/	/	/
80	-	+	CFT073	ins	133186	1	i02_0131	aceF	-	++++	-	-	-	-	-	-	-	-	-	-	-	-	-	-	-	-	-	/	-	-	-	-	-	-
81	+	-	clone D	ins	144870	6	i02_0139	yacK	-	++++	-	-	-	-	+	-	-	-	-	-	-	-	-	-	-	-	-	-	-	-	-	-	-	-
82	+	-	clone D	ins	154523	12	i02_0149	yadD	-	+++	-	/	/	/	-	-	/	/	/	/	/	/	/	/	/	/	/	/	/	/	/	/	/	/
83	-	+	CFT073	ins	155811	1	i02_0151	panC	-	++++	-	-	-	-	-	-	-	-	-	-	-	-	-	-	-	-	-	-	-	-	-	-	-	-
84	-	+	CFT073	ins	199713	1	i02_0193	dxr	-	++++	-	-	-	-	-	-	-	-	-	-	-	-	-	-	-	-	-	-	-	-	-	-	-	-
85	+	-	CFT073	del	227763	3	i02_0220	metQ	+	++++	+	+	+	+	+	+	+	+	+	+	+	+	+	+	+	+	+	+	+	+	+	+	+	+
86	+	-	CFT073	del	232061	1	rRNA_001	rrs	+	++++	+	+	+	-	+	+	+	-	+	+	-	+	+	+	+	-	+	+	+	-	+	+	+	+
87	-	+	CFT073	ins	232066	1	rRNA_001	rrs	-	++	-	-	-	+	-	-	-	+	-	-	+	-	+	-	/	+	-	-	-	+	+	-	-	-
88	-	+	CFT073	ins	233460	1	rRNA_002	rrl	-	++	-	-	-	+	-	-	/	+	+	-	/	+	+	+	+	-	-	+	+	+	-	+	+	+
89	+	-	CFT073	del	233464	3	rRNA_002	rrl	+	++	+	+	+	+	+	+	+	-	-	+	+	-	-	-	-	+	+	-	-	-	+	-	-	-
90	-	+	CFT073	ins	233469	3	rRNA_002	rrl	-	++	-	-	-	+	-	-	/	+	+	-	/	+	+	+	+	-	-	+	+	+	-	+	+	+
91	-	+	CFT073	ins	234089	1	rRNA_002	rrl	-	++	-	-	-	+	-	-	+	+	-	-	-	+	-	+	+	-	+	-	+	+	-	-	+	+
92	+	-	CFT073	del	234785	1	rRNA_002	rrl	+	++	+	+	+	+	+	+	+	-	-	+	+	-	-	-	-	-	+	-	-	-	-	-	-	-
93	+	-	CFT073	del	234789	1	rRNA_002	rrl	+	++	+	+	+	+	+	+	+	-	-	+	+	-	-	-	-	-	+	-	-	-	-	-	-	-
94	-	+	CFT073	ins	272526	1	i02_0280		-	++++	/	-	-	-	-	-	/	/	/	-	-	/	/	-	/	-	/	/	/	/	/	/	/	/
95	-	+	CFT073	ins	273259	1	i02_0281		-	+++	/	-	-	-	-	/	/	/	/	-	-	/	/	-	/	/	/	/	/	/	/	/	/	/
96	-	+	?	indel	327228	1	intergenic		?	+/-	/	/	/	/	/	/	/	/	/	/	/	/	/	/	/	/	/	/	/	/	/	/	/	/
97	-	+	?	indel	327823	1	intergenic		?	+/-	/	/	/	/	/	/	/	/	/	/	/	/	/	/	/	/	/	/	/	/	/	/	/	/

Indel No.	Clone D[a]	CFT073 [a]	Lineage	type	site[b]	size	Locus_tag	Gene	Inferred ancestral[c]	Support level[d]	ED1a	536	S88	APEC O1	UTI89	E2348/69	ATCC8739	K-12	HS	SMS3-5	IAI39	E24377A	SE11	55989	IAI1	Sakai	UMN026	B4 Sb227	F5 8401	SS Ss046	D1 Sd197	B18 BS512	F2a 2457T	F2a 301
98	-	+	?	indel	347908	1	i02_0343		?	+/-	/	/	/	/	/	/	/	/	/	/	/	/	/	/	/	/	/	/	/	/	/	/	/	/
99	+	-	clone D	ins	376418	2	i02_0372		-	+++	/	-	-	-	-	/	/	/	/	/	/	/	/	/	/	/	/	/	/	/	/	/	/	/
100	-	+	CFT073	ins	390718	1	intergenic		-	++++	-	-	-	-	-	-	-	-	-	-	-	-	-	-	-	-	-	-	/	/	/	/	/	/
101	-	+	CFT073	ins	390903	1	i02_0391		-	++++	-	-	-	-	-	-	/	/	/	-	-	/	/	/	/	-	-	/	/	/	/	/	/	/
102	+	-	CFT073	del	390967	1	i02_0391		+	++++	/	+	+	+	+	+	/	/	/	+	+	/	/	/	/	+	+	/	/	/	/	/	/	/
103	-	+	CFT073	ins	390984	1	i02_0391		-	++++	/	-	-	-	-	-	/	/	/	-	-	/	/	/	/	-	-	/	/	/	/	/	/	/
104	+	-	CFT073	del	391060	1	i02_0391		+	++++	/	+	+	+	+	+	/	/	/	+	+	/	/	/	/	+	+	/	/	/	/	/	/	/
105	-	+	CFT073	ins	416956	1	intergenic		-	++	/	-	+	+	+	/	/	/	/	/	/	/	/	/	/	/	/	/	/	/	/	/	/	/
106	-	+	CFT073	ins	431814	1	i02_0427		-	++++	-	-	-	-	-	-	-	-	-	-	-	-	-	-	-	-	-	/	/	-	/	/	/	/
107	-	+	CFT073	ins	431828	1	i02_0427		-	++++	-	-	-	-	-	-	-	-	-	-	-	-	-	-	-	-	-	/	/	-	/	/	/	/
108	-	+	CFT073	ins	453588	1	intergenic		-	++++	-	-	-	+	-	-	-	-	-	-	-	-	-	-	-	-	-	/	/	-	-	/	/	/
109	+	-	CFT073	del	453852	1	i02_0444	lacI	+	++++	+	+	+	+	+	+	+	+	+	+	+	+	+	+	+	+	+	/	/	+	+	/	/	/
110	-	+	CFT073	ins	453931	1	i02_0444	lacI	-	++++	-	-	-	-	-	-	-	-	-	-	-	-	-	-	-	-	-	/	/	-	-	/	/	/
111	-	+	CFT073	ins	593588	1	i02_0588	ybbM	-	++++	-	-	-	-	-	-	-	-	-	-	-	-	-	-	-	-	-	-	-	-	-	-	-	-
112	-	+	CFT073	ins	599774	1	i02_0594	ybbB	-	++++	-	-	-	-	-	-	-	-	-	-	/	-	-	-	-	-	-	-	-	-	-	-	-	-
113	-	+	CFT073	ins	626234	1	i02_0618	ybcI	-	++++	-	-	-	-	-	-	-	-	-	-	-	-	-	-	-	-	-	-	-	-	-	-	-	-
114	+	-	CFT073	del	793305	1	i02_0779		+	+++	+	+	+	+	+	/	/	/	/	/	/	/	/	/	/	/	/	/	/	/	/	/	/	/
115	-	+	CFT073	ins	793475	1	i02_0780		-	+++	-	-	-	-	-	/	/	/	/	/	/	/	/	/	/	/	/	/	/	/	/	/	/	/
116	-	+	CFT073	ins	802982	1	i02_0791	ybgF	-	++++	-	-	-	-	-	-	-	-	-	-	-	-	-	-	-	-	-	-	-	-	-	-	-	-
117	-	+	CFT073	ins	942703	1	i02_0931		-	++++	-	-	-	-	-	-	-	-	-	-	-	-	-	-	-	-	-	-	-	-	-	-	-	-
118	-	+	CFT073	ins	970076	1	i02_0953		-	++++	-	-	-	-	-	-	-	-	-	-	-	-	-	-	-	-	-	-	-	-	-	-	-	-
119	-	+	CFT073	ins	986199	1	i02_0965	lpxK	-	++++	-	-	-	-	-	-	-	-	-	-	-	-	-	-	-	-	-	-	-	-	-	-	-	-
120	+	-	CFT073	del	1066831	1	i02_1039	agp	+	++++	+	+	+	+	+	+	+	+	+	+	+	+	+	+	+	+	+	/	+	+	+	/	+	+
121	-	+	CFT073	ins	1066931	1	i02_1039	agp	-	++++	-	-	-	-	-	-	-	-	-	-	-	-	-	-	-	-	-	/	-	-	-	/	-	-
122	-	+	CFT073	ins	1081238	1	i02_1055		-	++++	-	-	-	-	-	-	-	-	-	-	-	-	-	-	-	-	-	-	/	-	/	/	/	/

Indel No.	Clone D[a]	CFT073 [a]	Lineage	type	site[b]	size	Locus_tag	Gene	Inferred ancestral[c]	Support level[d]	ED1a	536	S88	APEC O1	UTI89	E2348/69	ATCC8739	K-12	HS	SMS3-5	IAI39	E24377A	SE11	55989	IAI1	Sakai	UMN026	B4 Sb227	F5 8401	SS Ss046	D1 Sd197	B18 BS512	F2a 2457T	F2a 301
123	-	+	CFT073	ins	1129853	1	intergenic		-	++	/	-	/	/	-	/	/	/	/	/	/	/	/	/	/	/	/	/	/	/	/	/	/	/
124	-	+	?	indel	1132746	1	intergenic		?	+/-	/	/	/	/	/	/	/	/	/	/	/	/	/	/	/	/	/	/	/	/	/	/	/	/
125	-	+	CFT073	ins	1141301	1	i02_1117		-	++++	/	-	/	/	/	/	-	-	-	-	/	/	/	/	/	/	/	/	-	-	-	/	-	-
126	-	+	CFT073	ins	1203554	1	i02_1181	ycdW	-	++++	-	-	-	-	-	-	-	-	-	-	-	-	-	-	-	-	-	-	-	-	-	-	-	-
127	-	+	CFT073	ins	1232838	1	i02_1220		-	++++	-	-	-	-	-	-	-	-	-	-	-	-	-	-	-	-	-	-	-	-	-	-	-	-
128	-	+	CFT073	ins	1232865	1	i02_1220		-	++++	-	-	-	-	-	-	-	-	-	-	-	-	-	-	-	-	-	-	-	-	-	-	-	-
129	-	+	CFT073	ins	1232932	1	i02_1220		-	++++	-	-	-	-	-	-	-	-	-	-	-	-	-	-	-	-	-	-	-	-	-	-	-	-
130	-	+	CFT073	ins	1256746	1	i02_1247		-	++++	-	-	-	-	-	-	-	-	-	-	-	-	-	-	-	-	-	-	-	-	-	-	-	-
131	-	+	CFT073	ins	1256789	1	i02_1247		-	++++	-	-	-	-	-	-	-	-	-	-	-	-	-	-	-	-	-	-	-	-	-	-	-	-
132	-	+	CFT073	ins	1256798	1	i02_1247		-	++++	-	-	-	-	-	-	-	-	-	-	-	-	-	-	-	-	-	-	-	-	-	-	-	-
133	+	-	CFT073	del	1259736	1	i02_1250	pabC	+	++++	+	+	+	+	+	+	+	+	+	+	+	+	+	+	+	+	+	+	+	+	+	+	+	+
134	-	+	CFT073	ins	1259830	1	i02_1251	yceG	-	++++	-	-	-	-	-	-	-	-	-	-	-	-	-	-	-	-	-	-	-	-	-	-	-	-
135	-	+	CFT073	ins	1344697	1	i02_1357		-	++++	-	-	-	-	-	-	-	-	-	-	-	-	-	-	-	-	-	-	-	-	-	-	-	-
136	+	-	CFT073	del	1346650	12	intergenic		+	++++	+	+	+	+	+	/	/	/	/	/	+	/	/	/	/	+	+	/	/	/	/	/	/	/
137	-	+	CFT073	ins	1358441	1	i02_1383		-	+++	/	/	-	-	-	-	/	/	/	/	/	/	/	/	/	-	/	/	/	/	/	/	/	/
138	-	+	CFT073	ins	1358461	1	i02_1383		-	+++	/	/	-	-	-	-	/	/	/	/	/	/	/	/	/	-	/	/	/	/	/	/	/	/
139	-	+	CFT073	ins	1358472	1	i02_1383		-	+++	/	/	-	-	-	-	/	/	/	/	/	/	/	/	/	-	/	/	/	/	/	/	/	/
140	-	+	CFT073	ins	1358524	1	i02_1383		-	+++	/	/	-	-	-	-	/	/	/	/	/	/	/	/	/	-	/	/	/	/	/	/	/	/
141	-	+	CFT073	ins	1358753	1	i02_1384		-	+++	/	/	-	-	-	-	/	/	/	/	/	/	/	/	/	-	/	/	/	/	/	/	/	/
142	+	-	CFT073	del	1378275	1	i02_1415		+	++++	/	+	+	+	+	/	+	/	/	/	+	/	+	+	+	+	+	/	/	+	/	/	/	/
143	+	-	CFT073	del	1378427	1	i02_1415		+	++++	/	+	+	+	+	/	+	/	/	/	+	/	+	+	+	+	+	/	/	+	/	/	/	/
144	+	-	CFT073	del	1378444	1	i02_1415		+	++++	/	+	+	+	+	/	+	/	/	/	+	/	+	+	+	+	+	/	/	+	/	/	/	/
145	+	-	CFT073	del	1378515	1	i02_1415		+	++++	/	+	+	+	+	/	+	/	/	/	+	/	+	+	+	+	+	/	/	+	/	/	/	/
146	+	-	CFT073	del	1393522	1	i02_1430	sitA	+	++++	+	+	+	+	+	/	/	/	/	+	+	/	/	/	/	/	+	+	+	+	+	+	+	+
147	+	-	CFT073	del	1393546	1	i02_1430	sitA	+	++++	+	+	+	+	+	/	/	/	/	+	+	/	/	/	/	/	+	+	+	+	+	+	+	+

Indel No.	Clone D[a]	CFT073 [a]	Lineage	type	site[b]	size	Locus_tag	Gene	Inferred ancestral[c]	Support level[d]	ED1a	536	S88	APEC O1	UTI89	E2348/69	ATCC8739	K-12	HS	SMS3-5	IAI39	E24377A	SE11	55989	IAI1	Sakai	UMN026	B4 Sb227	F5 8401	SS Ss046	D1 Sd197	B18 BS512	F2a 2457T	F2a 301
148	+	-	CFT073	del	1393554	1	i02_1430	sitA	+	++++	+	+	+	+	+	/	/	/	/	+	+	/	/	/	/	/	+	+	+	+	+	+	+	+
149	+	-	CFT073	del	1393563	1	i02_1430	sitA	+	++++	+	+	+	+	+	/	/	/	/	+	+	/	/	/	/	/	+	+	+	+	+	+	+	+
150	+	-	CFT073	del	1393615	1	i02_1430	sitA	+	++++	+	+	+	+	+	/	/	/	/	+	+	/	/	/	/	/	+	+	+	+	+	+	+	+
151	+	-	CFT073	del	1393636	1	i02_1430	sitA	+	++++	+	+	+	+	+	/	/	/	/	+	+	/	/	/	/	/	+	+	+	+	+	+	+	+
152	+	-	CFT073	del	1529699	1	i02_1580	pyrF	+	++++	+	+	+	+	+	+	+	+	+	+	+	+	+	+	+	+	+	+	+	+	+	+	+	+
153	-	+	clone D	del	1541419	1	i02_1593		+	++++	+	+	+	+	+	+	/	/	/	+	+	/	/	/	/	+	/	/	/	/	+	/	/	/
154	-	+	CFT073	ins	1649103	1	i02_1691	ydcV	-	++++	-	-	-	-	-	-	-	-	-	-	/	-	-	-	-	-	-	/	-	-	-	/	-	-
155	-	+	CFT073	ins	1654282	1	i02_1701	yncD	-	++++	-	-	-	-	-	-	-	-	-	-	/	-	-	-	-	-	-	-	/	-	-	-	/	/
156	-	+	?	indel	1661875	1	i02_1709		?	+/-	/	/	/	/	/	/	/	/	/	/	/	/	/	/	/	/	-	/	/	/	/	/	/	/
157	-	+	?	indel	1662780	1	intergenic		?	+/-	/	/	/	/	/	/	/	/	/	/	/	/	/	/	/	/	-	/	/	/	/	/	/	/
158	+	-	CFT073	del	1668910	1	intergenic		+	+	/	/	/	/	+	/	/	/	/	/	/	/	/	/	/	/	/	/	/	/	/	/	/	/
159	+	-	CFT073	del	1670104	1	i02_1717		+	+++	+	+	+	+	+	/	/	/	/	/	/	/	/	/	/	/	+	/	/	/	/	/	/	/
160	+	-	CFT073	del	1688286	3	intergenic		+	++++	+	+	+	+	+	/	+	+	+	+	-	+	+	+	+	+	+	+	+	+	+	+	+	+
161	-	+	CFT073	ins	1693716	1	i02_1740		-	++++	-	-	-	-	-	-	-	-	-	-	-	-	-	-	-	-	-	-	-	-	/	-	-	-
162	-	+	CFT073	ins	1694715	1	i02_1740		-	++++	-	-	-	-	-	-	-	-	-	-	-	-	-	-	-	-	-	-	/	-	/	-	/	/
163	+	-	CFT073	del	1701417	1	intergenic		+	++	+	+	+	+	+	+	/	/	/	-	+	-	-	-	-	-	-	/	-	-	/	/	/	-
164	-	+	CFT073	ins	1724309	1	i02_1761	hipA	-	++++	-	-	-	-	-	-	-	-	-	/	/	/	-	-	-	-	-	/	/	-	-	-	/	/
165	-	+	CFT073	ins	1895916	1	i02_1932	pheT	-	++++	-	-	-	-	-	-	-	-	-	-	-	-	-	-	-	-	-	-	-	-	-	-	-	-
166	+	-	clone D	ins	1961664	1	intergenic		-	++++	-	-	-	-	+	-	-	-	-	-	-	-	-	-	-	-	-	-	-	-	-	-	-	-
167	+	-	clone D	ins	1961686	1	intergenic		-	++++	-	-	-	-	+	-	-	-	-	-	-	-	-	-	-	-	-	-	-	-	-	-	-	-
168	-	+	CFT073	ins	2010211	1	intergenic		-	++++	-	-	-	-	-	-	-	-	-	-	-	-	-	-	-	-	-	-	-	-	-	-	-	-
169	-	+	CFT073	ins	2010225	3	intergenic		-	++++	-	-	-	-	-	-	-	-	-	-	-	-	-	-	-	-	-	-	-	-	-	-	-	-
170	-	+	CFT073	ins	2010257	1	intergenic		-	++++	-	-	-	-	-	-	-	-	-	-	-	-	-	-	-	-	-	-	-	-	-	-	-	-
171	-	+	CFT073	ins	2025584	1	i02_2078	purK	-	++++	-	-	-	-	-	-	-	-	-	-	-	-	-	-	-	-	-	-	-	-	-	-	-	-
172	+	-	CFT073	del	2025609	1	i02_2078	purK	+	++++	+	+	+	+	+	+	+	+	+	+	+	+	+	+	+	+	+	+	+	+	+	+	+	+

Indel No.	Clone D[a]	CFT073 [a]	Lineage	type	site[b]	size	Locus_tag	Gene	Inferred ancestral[c]	Support level[d]	ED1a	536	S88	APEC O1	UTI89	E2348/69	ATCC8739	K-12	HS	SMS3-5	IAI39	E24377A	SE11	55989	IAI1	Sakai	UMN026	B4 Sb227	F5 8401	SS Ss046	D1 Sd197	B18 BS512	F2a 2457T	F2a 301
173	+	-	clone D	ins	2071197	6	i02_2126	yecG	-	++++	-	-	-	-	-	-	-	/	-	-	-	-	-	-	-	-	-	-	-	-	-	/	/	/
174	-	+	clone D	del	2148157	1	intergenic		+	+	/	+	/	/	/	+	/	/	/	/	/	/	/	/	/	/	/	/	/	/	/	/	/	/
175	+	-	CFT073	del	2158529	1	intergenic		+	+	/	+	/	/	+	/	/	/	/	/	/	/	/	/	/	/	/	/	/	/	/	/	/	/
176	+	-	clone D	ins	2173925	9	i02_2241		-	+++	-	-	-	-	+	/	/	/	/	/	-	/	/	-	/	/	-	/	/	/	/	/	/	/
177	-	+	CFT073	ins	2177218	1	i02_2242		-	++++	-	-	-	-	-	/	/	/	/	/	-	/	/	-	/	/	-	/	/	/	/	/	/	/
178	+	-	CFT073	del	2180042	1	i02_2242		+	++++	+	+	+	+	+	/	/	/	/	/	+	/	/	+	/	/	+	/	/	/	/	/	/	/
179	+	-	CFT073	del	2180052	2	i02_2242		+	++++	+	+	+	+	+	/	/	/	/	/	+	/	/	+	/	/	+	/	/	/	/	/	/	/
180	+	-	CFT073	del	2180060	2	i02_2242		+	++++	+	+	+	+	+	/	/	/	/	/	+	/	/	+	/	/	+	/	/	/	/	/	/	/
181	+	-	CFT073	del	2187699	1	i02_2246		+	++++	+	+	+	+	+	/	/	/	/	/	+	/	/	+	/	/	+	/	/	/	/	/	/	/
182	-	+	clone D	del	2190262	1	intergenic		+	++++	+	+	+	+	+	/	/	/	/	/	+	/	/	+	/	/	+	/	/	/	/	/	/	/
183	+	-	CFT073	del	2194430	1	i02_2251		+	++++	+	+	+	+	+	+	+	+	+	+	+	+	+	+	+	+	+	/	/	+	/	/	/	/
184	+	-	CFT073	del	2194446	1	i02_2251		+	++++	+	+	+	+	+	+	+	+	+	+	+	+	+	+	+	+	+	/	/	+	/	/	/	/
185	-	+	CFT073	ins	2212027	1	i02_2264		-	+	/	-	/	/	-	/	/	/	/	/	/	/	/	/	/	/	/	/	/	/	/	/	/	/
186	-	+	CFT073	ins	2249329	1	i02_2279		-	+	/	-	/	/	-	/	/	/	/	/	/	/	/	/	/	/	/	/	/	/	/	/	/	/
187	+	-	CFT073	del	2255272	16	intergenic		+	+	/	+	/	/	+	/	/	/	/	/	/	/	/	/	/	/	/	/	/	/	/	/	/	/
188	-	+	CFT073	ins	2272088	1	intergenic		-	+	/	-	/	/	-	/	/	/	/	/	/	/	/	/	/	/	/	/	/	/	/	/	/	/
189	-	+	CFT073	ins	2272119	1	intergenic		-	+	/	-	/	/	-	/	/	/	/	/	/	/	/	/	/	/	/	/	/	/	/	/	/	/
190	-	+	CFT073	ins	2272141	1	intergenic		-	+	/	-	/	/	-	/	/	/	/	/	/	/	/	/	/	/	/	/	/	/	/	/	/	/
191	-	+	CFT073	ins	2281124	1	intergenic		-	++++	-	-	-	-	-	/	/	/	/	-	/	-	/	-	/	/	-	/	/	/	/	/	/	/
192	-	+	clone D	del	2289140	1	i02_2318		+	++++	/	+	+	+	+	/	/	/	/	+	+	+	/	+	/	/	/	/	/	/	/	/	/	/
193	-	+	clone D	del	2297026	1	intergenic		+	+++	/	+	+	+	+	/	/	/	/	+	+	/	/	+	/	/	/	/	/	/	/	/	/	/
194	+	-	CFT073	del	2362248	1	i02_2386		+	++++	+	+	+	+	+	+	+	+	/	+	+	+	+	+	+	+	+	+	+	+	/	+	+	+
195	+	-	CFT073	del	2362290	1	i02_2386		+	++++	+	+	+	+	+	+	+	+	/	+	+	+	+	+	+	+	+	+	+	+	/	+	+	+
196	+	-	CFT073	del	2362303	1	i02_2386		+	++++	+	+	+	+	+	+	+	+	/	+	+	+	+	+	+	+	+	+	+	+	/	+	+	+
197	+	-	CFT073	del	2362896	1	i02_2387		+	++++	+	+	+	+	+	+	+	+	/	+	+	+	+	+	+	+	+	+	+	+	/	+	+	+

Indel No.	Clone D[a]	CFT073[a]	Lineage	type	site[b]	size	Locus_tag	Gene	Inferred ancestral[c]	Support level[d]	ED1a	536	S88	APEC O1	UTI89	E2348/69	ATCC8739	K-12	HS	SMS3-5	IAI39	E24377A	SE11	55989	IAI1	Sakai	UMN026	B4 Sb227	F5 8401	SS Ss046	D1 Sd197	B18 BS512	F2a 2457T	F2a 301
198	-	+	CFT073	ins	2485913	2	i02_2500	yeiN	-	++++	-	-	-	-	-	-	-	-	-	-	-	-	-	/	-	-	-	-	-	-	/	-	-	-
199	+	-	CFT073	del	2658778	1	i02_2665	yfcJ	+	++++	+	+	+	+	+	+	+	+	+	+	+	/	+	+	+	+	+	+	+	+	/	+	+	+
200	+	-	CFT073	del	2658814	1	i02_2665	yfcJ	+	++++	+	+	+	+	+	+	+	+	+	+	+	/	+	+	+	+	+	+	+	+	/	+	+	+
201	-	+	?	indel	2686052	1	i02_2693		?	+/-	/	/	/	/	/	/	/	/	/	-	/	/	/	/	/	/	/	/	/	/	/	/	/	/
202	-	+	?	indel	2686086	1	i02_2693		?	+/-	/	/	/	/	/	/	/	/	/	-	/	/	/	/	/	/	/	/	/	/	/	/	/	/
203	-	+	?	indel	2686164	18	i02_2693		?	+/-	/	/	/	/	/	/	/	/	/	+	/	/	/	/	/	/	/	/	/	/	/	/	/	/
204	+	-	CFT073	del	2718016	1	i02_2714		+	++++	+	+	+	+	+	+	+	+	+	+	+	+	+	+	+	+	+	+	+	+	+	+	+	+
205	+	-	CFT073	del	2751344	1	i02_2746	cysK	+	++++	+	+	+	+	+	+	+	+	+	+	+	+	+	+	+	+	+	+	+	+	+	+	+	+
206	-	+	CFT073	ins	2751413	1	intergenic		-	++++	-	-	-	-	-	-	-	-	-	-	-	-	-	-	-	-	-	-	-	-	-	-	-	-
207	-	+	CFT073	ins	2751487	1	intergenic		-	++++	-	-	-	-	-	-	-	-	-	-	-	-	-	-	-	-	-	-	-	-	-	-	-	-
208	-	+	CFT073	ins	2752054	1	i02_2749	ptsI	-	++++	-	-	-	-	-	-	-	-	-	-	-	-	-	-	-	-	-	-	-	-	-	-	-	-
209	+	-	clone D	ins	2852951	9	i02_2837	pbpC	-	++++	-	-	-	-	-	-	-	-	-	-	-	-	-	-	-	-	-	/	/	-	/	/	/	/
210	-	+	CFT073	ins	2885705	1	i02_2869		-	++++	-	-	-	-	-	-	-	-	-	-	-	-	-	-	-	-	-	-	-	-	-	-	-	-
211	-	+	CFT073	ins	2917217	1	intergenic		-	++++	-	-	-	-	-	-	-	-	-	-	-	-	-	-	-	-	-	-	-	-	-	-	-	-
212	-	+	CFT073	ins	2926828	1	rRNA_005	rrl	-	++++	-	-	-	-	-	-	-	-	-	-	-	-	-	-	-	-	-	-	-	-	-	-	-	-
213	+	-	CFT073	del	2927085	1	rRNA_005	rrl	+	++	+	+	+	+	+	+	+	-	-	+	+	-	-	-	-	-	+	-	-	-	-	-	-	-
214	+	-	CFT073	del	2927088	1	rRNA_005	rrl	+	++	+	+	+	+	+	+	+	-	-	+	+	-	-	-	-	-	+	-	-	-	-	-	-	-
215	+	-	CFT073	del	2927224	13	rRNA_005	rrl	+	++	+	+	+	+	+	+	-	-	-	+	-	-	-	-	-	-	+	-	+	-	-	-	+	+
216	-	+	clone D	del	2927237	13	rRNA_005	rrl	+	++++	+	+	+	+	+	+	+	+	+	+	+	+	+	+	+	+	+	+	+	+	+	+	+	+
217	-	+	CFT073	ins	2927786	1	rRNA_005	rrl	-	++	-	+	-	-	-	-	+	+	+	-	-	+	-	+	+	-	+	+	+	+	-	-	+	+
218	-	+	CFT073	ins	2928406	3	rRNA_005	rrl	-	++	-	+	-	+	-	-	+	+	+	+	-	+	+	+	+	-	-	+	+	+	-	+	+	+
219	+	-	CFT073	del	2928408	3	rRNA_005	rrl	+	++	+	+	+	+	+	+	/	-	-	+	+	-	-	-	-	-	+	-	-	-	+	-	-	-
220	-	+	CFT073	ins	2928417	1	rRNA_005	rrl	-	++	-	+	-	+	-	-	+	+	+	+	-	+	+	+	+	-	-	+	+	+	-	+	+	+
221	-	+	clone D	del	2928977	2	intergenic		+	++++	/	/	+	+	+	/	/	+	+	+	+	+	+	/	/	/	+	/	/	/	+	/	/	/
222	+	-	CFT073	del	2928978	16	intergenic		+	++	+	+	+	+	+	+	+	-	-	+	-	-	-	+	+	-	-	+	+	+	-	+	+	+

Indel No.	Clone D[a]	CFT073 [a]	Lineage	type	site[b]	size	Locus_tag	Gene	Inferred ancestral[c]	Support level[d]	ED1a	536	S88	APEC O1	UTI89	E2348/69	ATCC8739	K-12	HS	SMS3-5	IAI39	E24377A	SE11	55989	IAI1	Sakai	UMN026	B4 Sb227	F5 8401	SS Ss046	D1 Sd197	B18 BS512	F2a 2457T	F2a 301
223	-	+	clone D	del	2928978	5	intergenic		+	++++	/	/	+	+	+	/	/	+	+	+	+	+	+	/	/	/	+	/	/	/	+	/	/	/
224	+	-	CFT073	del	2929090	13	intergenic		+	++	+	+	+	-	+	+	-	-	+	+	+	-	-	+	+	-	+	+	+	+	+	+	+	+
225	-	+	?	indel	2929104	13	intergenic		?	+/-	/	/	/	/	/	/	/	+	/	/	-	+	+	/	/	/	/	/	/	/	/	/	/	/
226	+	-	CFT073	del	2929226	20	intergenic		+	++	+	+	+	-	+	+	-	-	-	+	+	-	-	-	-	+	+	+	+	+	+	-	+	+
227	+	-	CFT073	del	2929450	1	rRNA_006	rrs	+	++++	+	+	+	+	+	+	+	+	+	+	+	+	+	+	+	+	+	+	+	+	+	+	+	+
228	+	-	CFT073	del	2929550	1	rRNA_006	rrs	+	++++	+	+	+	+	+	+	+	+	+	+	+	+	+	+	+	+	+	+	+	+	+	+	+	+
229	-	+	CFT073	ins	2930036	1	rRNA_006	rrs	-	++++	-	-	-	+	-	-	-	-	-	-	-	-	-	-	-	-	-	-	-	-	-	-	-	-
230	-	+	CFT073	ins	2930063	1	rRNA_006	rrs	-	++++	-	-	-	+	-	-	-	-	-	-	-	-	-	-	-	-	-	-	-	-	-	-	-	-
231	-	+	CFT073	ins	2930079	1	rRNA_006	rrs	-	++++	-	-	-	+	-	-	-	-	-	-	-	-	-	-	-	-	-	-	-	-	-	-	-	-
232	-	+	CFT073	ins	2930102	1	rRNA_006	rrs	-	++++	-	-	-	+	-	-	-	-	-	-	-	-	-	-	-	-	-	-	-	-	-	-	-	-
233	-	+	CFT073	ins	2930210	1	rRNA_006	rrs	-	++++	-	-	-	+	-	-	-	-	-	-	-	-	-	-	-	-	-	-	-	-	-	-	-	-
234	-	+	CFT073	ins	2930267	1	rRNA_006	rrs	-	++++	-	-	-	+	-	-	-	-	-	-	-	-	-	-	-	-	-	-	-	-	-	-	-	-
235	-	+	CFT073	ins	2930289	1	rRNA_006	rrs	-	++++	-	-	-	+	-	-	-	-	-	-	-	-	-	-	-	-	-	-	-	-	-	-	-	-
236	-	+	CFT073	ins	2930305	1	rRNA_006	rrs	-	++++	-	-	-	+	-	-	-	-	-	-	-	-	-	-	-	-	-	-	-	-	-	-	-	-
237	-	+	CFT073	ins	2930427	1	rRNA_006	rrs	-	++++	-	-	-	+	-	-	-	-	-	-	-	-	-	-	-	-	-	-	-	-	-	-	-	-
238	-	+	CFT073	ins	2930474	1	rRNA_006	rrs	-	++++	-	-	-	+	-	-	-	-	-	-	-	-	-	-	-	-	-	-	-	-	-	-	-	-
239	-	+	CFT073	ins	2930497	1	rRNA_006	rrs	-	++++	-	-	-	+	-	-	-	-	-	-	-	-	-	-	-	-	-	-	-	-	-	-	-	-
240	+	-	CFT073	del	2969602	1	i02_2955		+	++++	+	+	+	+	+	+	/	/	/	+	+	/	/	/	/	+	+	/	/	/	+	/	/	/
241	+	-	CFT073	del	2969636	1	intergenic		+	++++	+	+	+	+	+	/	/	/	/	+	+	/	/	/	/	+	-	/	/	/	+	/	/	/
242	-	+	CFT073	ins	2978621	1	intergenic		-	++++	-	-	-	-	-	-	-	-	-	-	-	-	-	-	-	-	-	-	-	-	-	-	-	-
243	-	+	CFT073	ins	2992303	1	i02_2981	alaS	-	++++	-	-	-	-	-	-	-	-	-	-	-	-	-	-	-	-	-	-	-	-	-	-	-	-
244	-	+	CFT073	ins	3004393	1	i02_2995	norV	-	++++	-	-	-	-	-	-	-	-	-	-	-	-	-	-	-	/	-	-	-	-	-	-	-	-
245	-	+	CFT073	ins	3038925	5	i02_3034		-	++++	-	-	-	-	-	-	-	-	-	-	-	-	-	-	-	-	-	-	-	-	-	-	-	-
246	-	+	CFT073	ins	3045109	2	intergenic		-	++++	-	-	-	-	-	-	-	-	-	-	-	-	-	-	-	-	-	-	-	-	-	-	-	-
247	-	+	clone D	del	3068496	15	i02_3065		+	++++	+	+	+	+	+	/	/	/	/	+	/	/	/	/	/	+	+	/	/	/	+	/	/	/

Indel No.	Clone D[a]	CFT073 [a]	Lineage	type	site[b]	size	Locus_tag	Gene	Inferred ancestral[c]	Support level[d]	ED1a	536	S88	APEC O1	UTI89	E2348/69	ATCC8739	K-12	HS	SMS3-5	IAI39	E24377A	SE11	55989	IAI1	Sakai	UMN026	B4 Sb227	F5 8401	SS Ss046	D1 Sd197	B18 BS512	F2a 2457T	F2a 301
248	-	+	CFT073	ins	3082607	1	i02_3076	ygcX	-	++++	-	-	-	-	-	-	-	-	-	-	-	-	-	-	-	-	-	-	-	-	-	-	-	-
249	-	+	CFT073	ins	3082619	1	i02_3076	ygcX	-	++++	-	-	-	-	-	-	-	-	-	-	-	-	-	-	-	-	-	-	-	-	-	-	-	-
250	+	-	CFT073	del	3100943	1	i02_3096	fucK	+	++++	+	+	+	+	+	+	+	+	+	+	+	+	+	+	+	+	+	+	+	+	+	+	+	+
251	+	-	CFT073	del	3134313	1	i02_3121		+	+++	/	+	+	+	+	/	/	/	/	/	/	/	/	/	/	/	/	/	/	/	/	/	/	/
252	+	-	CFT073	del	3134331	1	i02_3121		+	+++	/	+	+	+	+	/	/	/	/	/	/	/	/	/	/	/	/	/	/	/	/	/	/	/
253	+	-	CFT073	del	3261304	1	i02_3238		+	++++	+	+	+	+	+	+	/	/	/	+	+	/	/	/	/	/	+	/	+	/	/	/	+	+
254	+	-	CFT073	del	3261317	1	i02_3238		+	++++	+	+	+	+	+	+	/	/	/	+	+	/	/	/	/	/	+	/	+	/	/	/	+	+
255	+	-	CFT073	del	3261350	2	i02_3238		+	++++	+	+	+	+	+	+	/	/	/	+	+	/	/	/	/	/	+	/	+	/	/	/	+	+
256	+	-	CFT073	del	3261357	1	i02_3238		+	++++	+	+	+	+	+	+	/	/	/	+	+	/	/	/	/	/	+	/	+	/	/	/	+	+
257	+	-	CFT073	del	3296532	12	i02_3275		+	+	/	/	+	+	/	/	/	/	/	/	+	/	/	/	/	/	/	/	/	/	/	/	/	/
258	-	+	?	indel	3298504	1	intergenic		?	+/-	/	+	-	-	+	/	/	/	/	/	-	/	/	/	/	/	+	/	/	/	/	/	/	/
259	+	-	CFT073	del	3300102	1	intergenic		+	+++	/	+	+	+	+	/	/	/	/	/	+	/	/	/	/	/	+	/	/	/	/	/	/	/
260	-	+	clone D	del	3300420	1	i02_3284		+	+++	/	+	+	+	+	/	/	/	/	/	-	/	/	/	/	/	+	/	/	/	/	/	/	/
261	+	-	CFT073	del	3302284	1	i02_3287		+	++++	+	+	+	+	+	+	/	/	/	+	+	+	/	+	/	+	-	+	+	+	+	+	+	+
262	+	-	?	indel	3303805	8	i02_3289		?	+/-	/	/	-	/	+	/	/	/	/	+	+	/	/	/	/	/	-	+	/	+	+	+	/	+
263	+	-	CFT073	del	3307276	1	i02_3294		+	++++	/	/	/	/	+	/	/	+	/	+	-	+	/	/	/	+	-	+	+	+	+	+	+	+
264	-	+	clone D	del	3311423	5	intergenic		+	+++	+	/	/	/	/	/	/	/	/	/	/	/	/	+	/	+	+	+	/	/	/	+	/	/
265	-	+	clone D	del	3326445	1	intergenic		+	++++	+	/	/	/	/	/	/	/	/	-	-	/	/	+	/	/	+	+	+	+	/	+	+	+
266	+	-	CFT073	del	3336055	1	i02_3326		+	++	+	/	/	/	+	/	/	/	/	/	/	/	/	/	/	/	+	/	/	/	/	/	/	/
267	+	-	CFT073	del	3336093	1	i02_3326		+	++	+	/	/	/	+	/	/	/	/	/	/	/	/	/	/	/	+	/	/	/	/	/	/	/
268	+	-	CFT073	del	3336107	1	i02_3326		+	++	+	/	/	/	+	/	/	/	/	/	/	/	/	/	/	/	+	/	/	/	/	/	/	/
269	+	-	CFT073	del	3336128	1	i02_3326		+	++	+	/	/	/	+	/	/	/	/	/	/	/	/	/	/	/	+	/	/	/	/	/	/	/
270	+	-	CFT073	del	3336135	1	i02_3326		+	++	+	/	/	/	+	/	/	/	/	/	/	/	/	/	/	/	+	/	/	/	/	/	/	/
271	+	-	CFT073	del	3336142	1	i02_3326		+	++	+	/	/	/	+	/	/	/	/	/	/	/	/	/	/	/	+	/	/	/	/	/	/	/
272	+	-	CFT073	del	3336159	1	i02_3326		+	++	+	/	/	/	+	/	/	/	/	/	/	/	/	/	/	/	+	/	/	/	/	/	/	/

Indel No.	Clone D[a]	CFT073 [a]	Lineage	type	site[b]	size	Locus_tag	Gene	Inferred ancestral[c]	Support level[d]	ED1a	536	S88	APEC O1	UTI89	E2348/69	ATCC8739	K-12	HS	SMS3-5	IAI39	E24377A	SE11	55989	IAI1	Sakai	UMN026	B4 Sb227	F5 8401	SS Ss046	D1 Sd197	B18 BS512	F2a 2457T	F2a 301
273	+	-	CFT073	del	3336176	1	i02_3326		+	++	+	/	/	/	+	/	/	/	/	/	/	/	/	/	/	/	+	/	/	/	/	/	/	/
274	+	-	CFT073	del	3336181	1	i02_3326		+	++	+	/	/	/	+	/	/	/	/	/	/	/	/	/	/	/	+	/	/	/	/	/	/	/
275	+	-	CFT073	del	3336202	1	i02_3326		+	++	+	/	/	/	+	/	/	/	/	/	/	/	/	/	/	/	+	/	/	/	/	/	/	/
276	+	-	CFT073	del	3336311	1	i02_3326		+	++	+	/	/	/	+	/	/	/	/	/	/	/	/	/	/	/	+	/	/	/	/	/	/	/
277	-	+	CFT073	ins	3337181	1	i02_3328		-	++	-	/	/	/	/	/	/	/	/	/	/	/	/	/	/	/	-	/	/	/	/	/	/	/
278	-	+	CFT073	ins	3337246	1	i02_3328		-	++	-	/	/	/	/	/	/	/	/	/	/	/	/	/	/	/	-	/	/	/	/	/	/	/
279	-	+	CFT073	ins	3337294	1	i02_3328		-	++	-	/	/	/	/	/	/	/	/	/	/	/	/	/	/	/	-	/	/	/	/	/	/	/
280	-	+	clone D	del	3344259	1	intergenic		+	++	+	/	/	/	/	/	/	/	/	/	/	/	/	/	/	/	+	/	/	/	/	/	/	/
281	-	+	CFT073	ins	3344306	2	intergenic		-	++	-	/	/	/	/	/	/	/	/	/	/	/	/	/	/	/	-	/	/	/	/	/	/	/
282	-	+	CFT073	ins	3344350	2	intergenic		-	++	-	/	/	/	/	/	/	/	/	/	/	/	/	/	/	/	-	/	/	/	/	/	/	/
283	-	+	CFT073	ins	3344364	1	intergenic		-	++	-	/	/	/	/	/	/	/	/	/	/	/	/	/	/	/	-	/	/	/	/	/	/	/
284	-	+	CFT073	ins	3344381	1	intergenic		-	++	-	/	/	/	/	/	/	/	/	/	/	/	/	/	/	/	-	/	/	/	/	/	/	/
285	+	-	CFT073	del	3348189	1	i02_3340		+	+++	/	+	/	/	+	/	/	/	+	/	/	+	/	+	/	/	+	/	/	/	/	/	+	+
286	+	-	CFT073	del	3348569	1	intergenic		+	+++	/	/	/	/	+	/	/	/	+	+	/	+	/	-	/	/	+	/	/	/	/	/	/	/
287	+	-	CFT073	del	3349813	1	intergenic		+	+++	/	/	/	/	+	/	/	/	+	/	/	/	/	+	/	/	+	/	/	/	/	/	/	/
288	+	-	?	indel	3350480	5	intergenic		?	+/-	/	/	-	/	+	/	/	/	/	/	/	/	/	/	/	/	-	/	/	/	/	/	/	/
289	-	+	?	indel	3350750	14	intergenic		?	+/-	/	/	/	/	/	/	/	/	/	/	/	/	/	/	/	/	-	/	/	/	/	/	/	/
290	-	+	CFT073	ins	3350827	7	i02_3344		-	+	/	/	/	/	/	/	/	/	/	/	-	/	/	/	/	/	-	/	/	/	/	/	/	/
291	+	-	?	indel	3350918	6	intergenic		?	+/-	/	/	/	/	/	/	/	/	/	/	/	/	/	/	/	/	+	/	/	/	/	/	/	/
292	+	-	?	indel	3359351	1	intergenic		?	+/-	/	/	/	/	/	/	/	/	/	/	/	/	/	/	/	/	/	/	/	/	/	/	/	/
293	+	-	CFT073	del	3364637	1	i02_3359		+	++++	+	+	+	+	+	/	/	+	+	-	+	+	/	+	/	+	-	/	/	/	+	+	/	/
294	-	+	clone D	del	3364699	1	i02_3359		+	++	+	-	+	-	+	/	/	-	+	+	+	+	/	+	/	+	+	/	/	/	+	-	/	/
295	-	+	CFT073	indel	3367923	1	i02_3364		?	+/-	/	/	/	/	/	/	/	/	/	/	/	+	/	/	/	/	-	/	/	/	/	/	/	/
296	+	-	clone D	ins	3387223	1	intergenic		-	++	-	-	+	+	+	/	/	/	/	-	-	/	/	/	/	/	-	/	/	/	/	/	/	/
297	-	+	CFT073	ins	3387673	1	intergenic		-	++++	-	-	-	-	-	/	/	/	/	-	-	/	/	/	/	/	-	/	/	/	/	/	/	/

Indel No.	Clone D[a]	CFT073[a]	Lineage	type	site[b]	size	Locus_tag	Gene	Inferred ancestral[c]	Support level[d]	ED1a	536	S88	APEC O1	UTI89	E2348/69	ATCC8739	K-12	HS	SMS3-5	IAI39	E24377A	SE11	55989	IAI1	Sakai	UMN026	B4 Sb227	F5 8401	SS Ss046	D1 Sd197	B18 BS512	F2a 2457T	F2a 301
298	+	-	CFT073	del	3389261	1	i02_3380	gspL	+	++++	+	+	+	+	+	+	/	+	+	+	+	+	+	+	+	/	+	+	/	/	+	+	/	/
299	-	+	CFT073	ins	3415674	5	i02_3402	yghO	-	++++	-	-	-	-	-	-	+	/	+	-	-	-	-	/	-	/	-	/	-	/	/	/	-	-
300	-	+	clone D	del	3415911	1	intergenic		+	++	+	+	+	+	+	+	+	/	+	-	-	-	-	/	-	/	+	/	-	/	/	/	-	-
301	-	+	CFT073	ins	3450140	1	i02_3439	yqhG	-	++++	-	-	-	-	-	-	-	-	-	-	-	-	-	-	-	-	-	-	-	-	/	-	-	-
302	-	+	CFT073	ins	3525549	1	i02_3514		-	++++	-	-	-	-	-	-	-	-	-	-	-	-	-	-	-	-	-	-	-	-	-	-	-	-
303	-	+	CFT073	ins	3525566	1	i02_3514		-	++++	-	-	-	-	-	-	-	-	-	-	-	-	-	-	-	-	-	-	-	-	-	-	-	-
304	+	-	CFT073	del	3561339	1	i02_3550	yqjF	+	++++	+	+	+	+	+	+	+	+	+	+	+	+	+	+	+	+	+	+	+	+	+	+	+	+
305	-	+	CFT073	ins	3562225	2	i02_3551	yqjG	-	++++	-	-	-	-	-	-	-	-	-	-	-	-	-	-	-	-	-	-	-	-	-	-	-	-
306	-	+	CFT073	ins	3567159	1	i02_3557		-	++++	-	-	-	-	-	-	-	-	-	-	-	-	-	-	-	-	-	-	-	-	-	-	-	-
307	-	+	CFT073	ins	3629787	1	i02_3621	hflB	-	++++	-	-	-	-	-	-	-	-	-	-	-	-	-	-	-	-	-	-	-	-	-	-	-	-
308	+	-	CFT073	del	3704908	3	i02_3698		+	++++	+	+	+	+	+	/	/	/	/	/	+	/	/	/	/	/	/	+	/	/	/	+	/	/
309	+	-	CFT073	del	3726492	1	rRNA_008	rrl	+	++++	+	+	+	+	+	+	+	+	+	+	+	+	+	+	+	+	+	+	+	+	+	+	+	+
310	-	+	CFT073	ins	3728030	1	rRNA_008	rrl	-	++	-	+	-	-	-	-	+	+	+	-	-	+	-	+	+	-	+	+	+	+	-	-	+	+
311	-	+	CFT073	ins	3728661	1	rRNA_008	rrl	-	++	-	+	+	-	+	-	+	+	+	-	-	+	+	+	+	+	+	+	+	+	+	+	+	+
312	-	+	CFT073	ins	3729221	2	intergenic		-	++	-	-	+	-	+	-	-	+	+	+	+	+	+	-	-	-	+	-	-	-	+	-	-	-
313	+	-	CFT073	del	3729227	11	intergenic		+	++++	+	+	+	+	+	+	+	/	/	+	/	/	/	+	+	+	+	+	/	/	/	/	/	/
314	-	+	CFT073	ins	3809580	1	i02_3825	yhfZ	-	++++	-	-	-	-	-	-	-	-	-	-	-	-	-	-	-	/	-	-	-	-	-	-	-	-
315	-	+	clone D	del	3913324	6	i02_3927	ftsY	+	++	+	+	+	+	+	+	+	-	+	+	+	+	+	+	+	+	+	-	/	+	+	+	+	+
316	+	-	CFT073	del	3933212	1	i02_3950		+	++++	/	/	+	+	+	/	/	/	/	+	+	/	/	/	/	+	+	/	/	/	/	/	/	/
317	-	+	CFT073	ins	4141078	2	intergenic		-	++++	-	-	-	-	-	-	/	/	/	-	-	/	/	/	/	/	/	/	/	/	/	/	/	/
318	+	-	clone D	ins	4145486	1	intergenic		-	++	-	/	-	-	-	+	/	/	/	/	-	/	/	/	/	+	/	/	-	-	-	/	-	-
319	+	-	clone D	ins	4145592	1	intergenic		-	+++	/	-	/	/	+	/	/	/	/	/	-	/	/	/	/	/	/	/	-	-	-	/	-	-
320	-	+	?	indel	4145717	1	intergenic		?	+/-	/	/	/	/	/	/	/	/	/	/	+	/	/	/	/	-	/	/	+	+	+	/	+	+
321	-	+	CFT073	ins	4196764	1	i02_4207	dgoA	-	++++	-	-	-	-	-	-	-	-	-	-	-	-	-	-	-	/	-	/	-	-	/	/	-	-
322	-	+	CFT073	ins	4199528	1	i02_4210		-	++++	-	-	-	-	-	-	/	/	/	-	-	/	/	/	/	/	-	/	/	/	/	/	/	/

Indel No.	Clone D[a]	CFT073[a]	Lineage	type	site[b]	size	Locus_tag	Gene	Inferred ancestral[c]	Support level[d]	ED1a	536	S88	APEC O1	UTI89	E2348/69	ATCC8739	K-12	HS	SMS3-5	IAI39	E24377A	SE11	55989	IAI1	Sakai	UMN026	B4 Sb227	F5 8401	SS Ss046	D1 Sd197	B18 BS512	F2a 2457T	F2a 301
323	+	-	CFT073	del	4199642	1	i02_4210		+	++++	+	+	+	+	+	+	/	/	/	+	+	/	/	/	/	/	+	/	/	/	/	/	/	/
324	-	+	CFT073	ins	4206704	1	i02_4217	yfgE	-	++++	-	-	-	-	-	-	-	-	-	-	-	-	-	-	-	-	-	-	-	-	-	-	-	-
325	-	+	CFT073	ins	4266091	1	rRNA_010	rrs	-	++++	-	+	-	-	-	-	-	-	-	-	-	-	-	-	-	-	-	-	-	-	-	-	-	-
326	-	+	CFT073	ins	4267075	1	rRNA_011	rrl	-	++++	-	-	-	-	-	-	-	-	-	-	-	-	-	-	-	-	-	-	-	-	-	-	-	-
327	-	+	CFT073	ins	4267207	1	rRNA_011	rrl	-	++	-	+	-	+	-	-	+	+	+	-	-	+	+	+	+	-	-	+	+	+	-	+	+	+
328	+	-	CFT073	del	4267213	5	rRNA_011	rrl	+	++	+	+	+	+	+	+	+	-	-	-	+	-	-	-	-	+	+	-	-	-	/	-	/	-
329	-	+	CFT073	ins	4267220	5	rRNA_011	rrl	-	++	-	+	-	+	-	-	+	+	+	-	-	+	+	+	+	-	-	+	+	+	-	+	+	+
330	-	+	CFT073	ins	4267838	1	rRNA_011	rrl	-	++	-	-	-	-	-	-	+	+	+	-	-	+	-	+	+	-	-	+	+	+	-	-	+	+
331	-	+	CFT073	ins	4268433	1	rRNA_011	rrl	-	++++	-	-	-	-	-	-	-	-	-	-	-	-	-	-	-	-	-	-	-	-	-	-	-	-
332	-	+	CFT073	ins	4272677	1	i02_4279	yifB	-	++++	-	-	-	-	-	-	-	-	-	-	-	-	-	-	-	-	-	-	-	-	-	-	-	-
333	-	+	CFT073	ins	4308484	1	intergenic		-	++++	-	-	-	-	-	-	-	-	-	-	-	-	-	-	-	-	-	-	-	-	-	-	-	-
334	-	+	clone D	del	4310013	6	i02_4315	hemX	+	++++	+	+	+	+	+	+	+	+	+	+	+	+	+	-	+	+	+	+	+	+	+	+	+	+
335	-	+	CFT073	ins	4383360	1	rRNA_013	rrs	-	++++	-	-	-	+	-	-	-	-	-	-	-	-	-	-	-	-	-	-	-	-	-	-	-	-
336	-	+	CFT073	ins	4385386	1	rRNA_014	rrl	-	++++	-	-	-	+	-	-	-	-	-	-	-	-	-	-	-	-	-	-	-	-	-	-	-	-
337	-	+	CFT073	ins	4385393	1	rRNA_014	rrl	-	++++	-	-	-	+	-	-	-	-	-	-	-	-	-	-	-	-	-	-	-	-	-	-	-	-
338	-	+	CFT073	ins	4386142	1	rRNA_014	rrl	-	++++	-	-	-	+	-	-	-	-	-	-	-	-	-	-	-	-	-	-	-	-	-	-	-	-
339	-	+	CFT073	ins	4386250	1	rRNA_014	rrl	-	++++	-	-	-	+	-	-	-	-	-	-	-	-	-	-	-	-	-	-	-	-	-	-	-	-
340	+	-	CFT073	del	4394962	1	i02_4402	xni	+	++++	+	+	+	+	+	+	+	+	+	+	+	+	+	+	+	+	+	+	+	+	+	+	+	+
341	+	-	clone D	ins	4520822	1	intergenic		-	++++	-	-	-	-	+	-	-	-	-	-	-	-	-	-	-	-	-	-	-	-	-	-	-	-
342	-	+	clone D	del	4520842	1	intergenic		+	++++	+	+	+	+	+	+	+	+	+	+	+	+	+	+	+	+	+	+	+	+	+	+	+	+
343	-	+	clone D	del	4520877	1	intergenic		+	++++	+	+	+	+	+	+	+	+	+	+	+	+	+	+	+	+	+	+	+	+	+	+	+	+
344	-	+	CFT073	ins	4522159	1	rRNA_016	rrs	-	++++	-	+	-	+	-	-	-	-	-	-	-	-	-	-	-	-	-	-	-	-	-	-	-	-
345	-	+	CFT073	ins	4522197	1	rRNA_016	rrs	-	++++	-	+	-	+	-	-	-	-	-	-	-	-	-	-	-	-	-	-	-	-	-	-	-	-
346	-	+	CFT073	ins	4522200	1	rRNA_016	rrs	-	++++	-	+	-	+	-	-	-	-	-	-	-	-	-	-	-	-	-	-	-	-	-	-	-	-
347	-	+	CFT073	ins	4522223	1	rRNA_016	rrs	-	++++	-	+	-	+	-	-	-	-	-	-	-	-	-	-	-	-	-	-	-	-	-	-	-	-

Indel No.	Clone D[a]	CFT073 [a]	Lineage	type	site[b]	size	Locus_tag	Gene	Inferred ancestral[c]	Support level[d]	ED1a	536	S88	APEC O1	UTI89	E2348/69	ATCC8739	K-12	HS	SMS3-5	IAI39	E24377A	SE11	55989	IAI1	Sakai	UMN026	B4 Sb227	F5 8401	SS Ss046	D1 Sd197	B18 BS512	F2a 2457T	F2a 301
348	-	+	CFT073	ins	4522239	1	rRNA_016	rrs	-	++++	-	+	-	+	-	-	-	-	-	-	-	-	-	-	-	-	-	-	-	-	-	-	-	-
349	-	+	CFT073	ins	4522262	2	rRNA_016	rrs	-	++++	-	+	-	+	-	-	-	-	-	-	-	-	-	-	-	-	-	-	-	-	-	-	-	-
350	-	+	CFT073	ins	4522267	1	rRNA_016	rrs	-	++++	-	+	-	+	-	-	-	-	-	-	-	-	-	-	-	-	-	-	-	-	-	-	-	-
351	-	+	CFT073	ins	4523421	1	rRNA_017	rrl	-	++	-	+	+	+	+	-	+	+	+	-	-	+	+	+	+	-	-	+	+	+	+	+	+	+
352	+	-	CFT073	del	4523427	2	rRNA_017	rrl	+	++	+	+	+	+	+	+	+	-	-	+	+	-	-	-	-	-	+	-	-	-	+	-	-	-
353	-	+	CFT073	ins	4523431	1	rRNA_017	rrl	-	++	-	+	-	+	-	-	+	+	+	-	-	+	+	+	+	-	-	+	+	+	-	+	+	+
354	-	+	CFT073	ins	4523432	1	rRNA_017	rrl	-	++	-	+	-	+	-	-	+	+	+	-	-	+	+	+	+	-	-	+	+	+	-	+	+	+
355	-	+	CFT073	ins	4524052	1	rRNA_017	rrl	-	++	-	+	-	+	-	-	+	+	+	-	-	+	-	+	+	-	+	+	+	+	-	-	+	+
356	+	-	CFT073	del	4524601	13	rRNA_017	rrl	+	++	+	+	+	+	+	+	-	-	-	+	-	+	-	+	-	-	-	-	+	-	-	-	-	+
357	-	+	CFT073	ins	4524614	13	rRNA_017	rrl	-	++	-	+	-	-	-	-	+	+	+	+	+	+	+	+	+	+	+	+	-	+	+	+	-	-
358	+	-	CFT073	del	4524748	1	rRNA_017	rrl	+	++	+	-	+	-	+	+	+	+	-	+	+	-	-	-	-	-	+	-	-	-	-	-	-	-
359	+	-	CFT073	del	4524752	1	rRNA_017	rrl	+	++	+	-	+	-	+	+	+	+	-	+	+	-	-	-	-	-	+	-	-	-	-	-	-	-
360	-	+	CFT073	ins	4562895	1	intergenic		-	++	-	-	-	+	-	-	-	-	-	-	+	-	-	-	-	-	-	-	-	-	-	-	-	-
361	-	+	CFT073	ins	4564223	1	rRNA_020	rrl	-	++	-	-	-	+	-	-	+	+	+	-	-	+	-	+	+	-	+	+	+	+	-	-	+	+
362	-	+	CFT073	ins	4564736	1	rRNA_020	rrl	-	++++	-	-	-	+	-	-	-	-	-	-	-	-	-	-	-	-	-	-	-	-	-	-	-	-
363	-	+	CFT073	ins	4564756	1	rRNA_020	rrl	-	++++	-	-	-	+	-	-	-	-	-	-	-	-	-	-	-	-	-	-	-	-	-	-	-	-
364	-	+	CFT073	ins	4634107	1	intergenic		-	++	-	+	+	+	+	+	/	/	/	/	/	/	/	/	/	/	/	/	/	/	/	/	/	/
365	+	-	clone D	ins	4671388	1	intergenic		-	+++	-	-	/	/	/	/	-	-	/	/	/	/	/	/	/	/	/	/	/	/	/	/	/	/
366	+	-	clone D	ins	4680413	10	intergenic		-	++++	-	-	-	-	+	-	-	-	-	-	-	-	-	-	-	-	-	-	-	-	/	/	-	-
367	+	-	CFT073	del	4790012	1	i02_4776	yjfL	+	++++	+	+	+	+	+	+	+	+	+	+	+	+	+	+	+	+	+	+	+	/	/	+	+	+
368	+	-	CFT073	del	4790954	1	i02_4778	yjfC	+	++++	+	+	+	+	+	+	+	+	+	+	+	+	+	+	+	+	+	+	+	+	+	+	+	+
369	-	+	CFT073	ins	4868672	1	i02_4861		-	+++	-	-	/	-	-	-	/	/	/	/	-	/	/	/	/	/	/	/	/	/	/	/	/	/
370	-	+	CFT073	ins	4988920	1	i02_4950		-	+++	-	-	/	-	-	-	/	/	/	/	-	/	/	/	/	/	/	/	/	/	/	/	/	/
371	-	+	CFT073	ins	4988958	1	i02_4950		-	+++	-	-	/	-	-	-	/	/	/	/	-	/	/	/	/	/	/	/	/	/	/	/	/	/
372	-	+	clone D	del	5038168	1	i02_5003	lasT	+	++++	+	+	+	+	+	+	+	+	+	+	+	+	+	+	+	+	+	+	+	+	+	+	+	+

Indel No.	Clone D[a]	CFT073 [a]	Lineage	type	site[b]	size	Locus_tag	Gene	Inferred ancestral[c]	Support level[d]	ED1a	536	S88	APEC O1	UTI89	E2348/69	ATCC8739	K-12	HS	SMS3-5	IAI39	E24377A	SE11	55989	IAI1	Sakai	UMN026	B4 Sb227	F5 8401	SS Ss046	D1 Sd197	B18 BS512	F2a 2457T	F2a 301

[a] "+" indicates the block present, "-" indicates the block absent, "/" indicates the block and nearby sequence absent.

[b] Using the clone D_i2 genome as reference.For indels the base indicated is the base before the insertion or deletion.

[c] Presence or absence of indel block in clone D/CFT073 ancestor as inferred from outgroup analysis.

[d] Level of support for allocation of indel blocks as given in previous column.
"++++" agreement is high-8 or more outgroup strains with expected type and at most 2 with the opposite, and ED1a agrees or site not present
"+++" agreement good-4 or more outgroup strains with expected type and at most 2 with the opposite, and ED1a agrees or site not present
"++" agreement in ED1a regardless of situation with other outgroup strains
"+" no conflict but very limited support as either the block and nearby sequence absent in ED1a, and/or outgroup support less than given above
"+/-" no conflict but no support (the block and its nearby sequence not present in any outgroup OR both alternative lineages supported equally).
