# Supplementary material for: Rates of Mutation and Host Transmission for an Escherichia coli Clone over 3 Years
Source: PLoS One. 2011 Oct 27;6(10):e26907. doi: 10.1371/journal.pone.0026907 (PMC3203180; doi:10.1371/journal.pone.0026907)
Supplement: Table S7 — SNPs between clone D and CFT073 genomes. A full list of single base differences between clone D and CFT073 genomes, including location, nature of difference, and name of gene affected. (PDF) [file pone.0026907.s008.pdf]

Table S7 SNPs between clone D and CFT073 genomes. A full list of single base differences between clone D and CFT073 genomes, including location, nature of difference, and name of gene affected.

Lineage	Clone D site	CFT 073 site	mutation type[a]	recomb	Gene	Gene name	Type
clone D	7482	8194	s	out	i02_0007	yaaJ	CDS
CFT073	37232	37944	s	out	i02_0037	caiE	CDS
clone D	68282	69705	ns	out	i02_0064	araA	CDS
clone D	141616	145065	ns	out	i02_0135	yacL	CDS
CFT073	142637	146086	s	out	i02_0137	speE	CDS
CFT073	148054	151497	nc	out			
clone D	165170	168602	s	out	i02_0161	pcnB	CDS
clone D	166117	169549	ns	out	i02_0161	pcnB	CDS
clone D	175531	178963	ns	out	i02_0170	fhuA	CDS
CFT073	220289	224434	ns	out	i02_0211	yaeQ	CDS
CFT073	224221	228366	ns	out	i02_0216	yaeF	CDS
clone D	229285	233427	ns	rec	i02_0222	metN	CDS
clone D	230078	234220	nc	rec	intergenic		
CFT073	230842	234984	ns	rec	i02_0224	/	CDS
clone D	230892	235034	s	rec	i02_0224	/	CDS
clone D	231008	235150	nc	rec	intergenic		
CFT073	231133	235275	nc	rec	16s rRNA	rrs	rRNA
clone D	231226	235368	nc	rec	16s rRNA	rrs	rRNA
CFT073	232045	236187	nc	rec	16s rRNA	rrs	rRNA
CFT073	232049	236191	nc	rec	16s rRNA	rrs	rRNA
CFT073	232053	236195	nc	rec	16s rRNA	rrs	rRNA
CFT073	232065	236206	nc	rec	16s rRNA	rrs	rRNA
CFT073	232081	236223	nc	rec	16s rRNA	rrs	rRNA
clone D	232521	236911	nc	rec	intergenic		
CFT073	232747	236988	nc	rec	intergenic		
clone D	232846	237087	nc	rec	intergenic		
clone D	232849	237090	nc	rec	intergenic		
CFT073	232891	237132	nc	rec	intergenic		
CFT073	233001	237242	nc	rec	23s rRNA	rrl	rRNA
clone D	233234	237475	nc	rec	23s rRNA	rrl	rRNA
CFT073	233274	237515	nc	rec	23s rRNA	rrl	rRNA
clone D	233275	237516	nc	rec	23s rRNA	rrl	rRNA
CFT073	233459	237700	nc	rec	23s rRNA	rrl	rRNA
CFT073	233763	238005	nc	rec	23s rRNA	rrl	rRNA
CFT073	234088	238330	nc	rec	23s rRNA	rrl	rRNA
CFT073	234094	238337	nc	rec	23s rRNA	rrl	rRNA
CFT073	234135	238378	nc	rec	23s rRNA	rrl	rRNA
CFT073	234136	238379	nc	rec	23s rRNA	rrl	rRNA
CFT073	234145	238388	nc	rec	23s rRNA	rrl	rRNA
CFT073	234146	238389	nc	rec	23s rRNA	rrl	rRNA
CFT073	234422	238665	nc	rec	23s rRNA	rrl	rRNA
CFT073	234464	238707	nc	rec	23s rRNA	rrl	rRNA
clone D	234523	238766	nc	rec	23s rRNA	rrl	rRNA
clone D	234787	239029	nc	rec	23s rRNA	rrl	rRNA
CFT073	235123	239364	nc	rec	23s rRNA	rrl	rRNA

Lineage	Clone D site	CFT 073 site	mutation type[a]	recomb	Gene	Gene name	Type
CFT073	235720	239961	nc	rec	23s rRNA	rrl	rRNA
clone D	235919	240160	nc	rec	intergenic		
CFT073	241720	245961	ns	out	i02_0231	gloB	CDS
CFT073	262792	258602	ns	rec	i02_0266	/	CDS
clone D	262803	258613	ns	rec	i02_0267	/	CDS
CFT073	262805	258615	ns	rec	i02_0267	/	CDS
clone D	262806	258616	ns	rec	i02_0267	/	CDS
CFT073	262807	258617	ns	rec	i02_0267	/	CDS
CFT073	262828	258638	s	rec	i02_0267	/	CDS
CFT073	262832	258642	ns	rec	i02_0267	/	CDS
CFT073	262834	258644	s	rec	i02_0267	/	CDS
CFT073	262841	258651	ns	rec	i02_0267	/	CDS
CFT073	262843	258653	ns	rec	i02_0267	/	CDS
CFT073	262845	258655	ns	rec	i02_0267	/	CDS
CFT073	262855	258665	s	rec	i02_0267	/	CDS
?	262897	258707	s	rec	i02_0267	/	CDS
CFT073	262900	258710	s	rec	i02_0267	/	CDS
clone D	262908	258718	s	rec	i02_0267	/	CDS
CFT073	264168	259978	s	rec	i02_0268	yeeS	CDS
clone D	264177	259987	ns	rec	i02_0268	yeeS	CDS
clone D	264179	259989	ns	rec	i02_0268	yeeS	CDS
clone D	264182	259992	ns	rec	i02_0268	yeeS	CDS
clone D	264183	259993	s	rec	i02_0268	yeeS	CDS
clone D	264209	260019	ns	rec	i02_0268	yeeS	CDS
clone D	264213	260023	s	rec	i02_0268	yeeS	CDS
clone D	264222	260032	nc	rec	intergenic		
clone D	264229	260039	nc	rec	intergenic		
clone D	264232	260042	nc	rec	intergenic		
clone D	264240	260050	s	rec	i02_0269	/	CDS
clone D	264243	260053	s	rec	i02_0269	/	CDS
CFT073	264415	260225	ns	rec	i02_0269	/	CDS
CFT073	264417	260227	s	rec	i02_0269	/	CDS
CFT073	264420	260230	s	rec	i02_0269	/	CDS
CFT073	264426	260236	s	rec	i02_0269	/	CDS
CFT073	264459	260269	s	rec	i02_0269	/	CDS
CFT073	264462	260272	s	rec	i02_0269	/	CDS
CFT073	264474	260284	s	rec	i02_0269	/	CDS
CFT073	264477	260287	s	rec	i02_0269	/	CDS
CFT073	264521	260331	ns	rec	i02_0269	/	CDS
CFT073	264525	260335	ns	rec	i02_0269	/	CDS
clone D	264526	260336	ns	rec	i02_0269	/	CDS
clone D	264552	260362	s	rec	i02_0269	/	CDS
CFT073	278428	274240	s	out	i02_0286	/	CDS
CFT073	281024	276836	s	out	i02_0287	/	CDS
CFT073	292352	288164	ns	out	i02_0303	/	CDS
clone D	310680	306465	s	out	i02_0322	ulaA	CDS
?	326514	322299	nc	rec	intergenic		
?	326758	322543	nc	rec	intergenic		
?	327001	322786	nc	rec	intergenic		
?	327109	322894	nc	rec	intergenic		
?	327152	322937	nc	rec	intergenic		

Lineage	Clone D site	CFT 073 site	mutation type[a]	recomb	Gene	Gene name	Type
?	327153	322938	nc	rec	intergenic		
?	327245	323031	nc	rec	intergenic		
?	327378	323164	nc	rec	intergenic		
CFT073	364934	363040	ns	out	i02_0362	pepD	CDS
CFT073	364961	363067	ns	out	i02_0362	pepD	CDS
clone D	370492	368598	s	out	i02_0368	proB	CDS
clone D	371371	369477	s	out	i02_0369	proA	CDS
clone D	385561	383665	ns	out	i02_0383	yagX	CDS
CFT073	387348	385452	ns	out	i02_0385	matB	CDS
clone D	388324	386428	ns	out	i02_0386	matA	CDS
CFT073	389023	387127	ns	out	i02_0387	/	CDS
CFT073	390877	388982	ns	rec	i02_0391	/	CDS
CFT073	390890	388995	ns	rec	i02_0391	/	CDS
CFT073	390901	389006	s	rec	i02_0391	/	CDS
clone D	390930	389036	s	rec	i02_0391	/	CDS
CFT073	390959	389066	s	rec	i02_0391	/	CDS
CFT073	390983	389089	ns	rec	i02_0391	/	CDS
?	391040	389147	s	rec	i02_0391	/	CDS
clone D	391041	389148	s	rec	i02_0391	/	CDS
clone D	391059	389167	ns	rec	i02_0391	/	CDS
CFT073	403397	401504	ns	out	i02_0399	/	CDS
CFT073	418710	417089	s	out	i02_0416	betA	CDS
CFT073	423896	422275	nc	out	intergenic		
CFT073	437807	436282	s	out	i02_0433	yahO	CDS
CFT073	450348	448823	ns	out	i02_0442	lacY	CDS
CFT073	457462	455938	s	out	i02_0447	adhC	CDS
CFT073	457464	455940	ns	out	i02_0447	adhC	CDS
clone D	462126	460602	ns	out	i02_0454	tauA	CDS
CFT073	464761	463237	ns	out	i02_0458	tauD	CDS
CFT073	469174	467650	ns	out	i02_0460	/	CDS
clone D	483921	482397	ns	out	i02_0481	yaiE	CDS
clone D	488150	486626	s	out	i02_0486	araJ	CDS
CFT073	492766	491242	ns	out	i02_0490	phoB	CDS
CFT073	494798	493274	nc	out	intergenic		
clone D	526368	524844	ns	out	i02_0524	cyoA	CDS
clone D	530872	529348	ns	out	i02_0530	tig	CDS
clone D	532814	531290	nc	out	intergenic		
clone D	535100	533576	s	out	i02_0533	lon	CDS
clone D	535138	533614	ns	out	i02_0533	lon	CDS
clone D	552216	551403	ns	out	i02_0551	ybaA	CDS
CFT073	558643	557830	s	out	i02_0557	acrB	CDS
CFT073	562872	562059	ns	out	i02_0560	aefA	CDS
clone D	571721	570908	ns	out	i02_0568	htpG	CDS
clone D	580712	579899	ns	out	i02_0575	ushA	CDS
clone D	586799	585986	ns	out	i02_0580	copA	CDS
clone D	614703	613892	ns	out	i02_0606	ylbB	CDS
CFT073	629849	629039	nc	out	intergenic		
CFT073	629856	629046	nc	out	intergenic		
clone D	662386	661576	ns	out	i02_0648	fepE	CDS
CFT073	664386	663576	s	out	i02_0651	fepD	CDS
CFT073	680371	679561	ns	out	i02_0665	ybdO	CDS

Lineage	Clone D site	CFT 073 site	mutation type[a]	recomb	Gene	Gene name	Type
CFT073	683562	682752	ns	out	i02_0668	ahpF	CDS
CFT073	693054	692244	ns	rec	i02_0677	citC	CDS
CFT073	693092	692282	ns	rec	i02_0677	citC	CDS
CFT073	693107	692297	ns	rec	i02_0677	citC	CDS
CFT073	693108	692298	ns	rec	i02_0677	citC	CDS
CFT073	693114	692304	s	rec	i02_0677	citC	CDS
clone D	695486	694676	s	out	i02_0679	dpiA	CDS
clone D	697657	696847	nc	out	intergenic		
CFT073	701919	701109	ns	out	i02_0687	/	CDS
CFT073	722795	721985	ns	rec	i02_0710	/	CDS
CFT073	722796	721986	ns	rec	i02_0710	/	CDS
CFT073	722797	721987	ns	rec	i02_0710	/	CDS
CFT073	722804	721994	ns	rec	i02_0710	/	CDS
clone D	732505	731695	ns	out	i02_0718	asnB	CDS
CFT073	733624	732814	ns	out	i02_0719	nagD	CDS
CFT073	754691	754593	s	out	i02_0742	ybfF	CDS
CFT073	778958	778797	ns	out	i02_0765	abrB	CDS
?	792960	792799	s	out	i02_0778	/	CDS
CFT073	806217	806102	ns	out	i02_0795	/	CDS
CFT073	824956	824841	s	out	i02_0814	ybhI	CDS
CFT073	835133	835018	ns	out	i02_0823	bioC	CDS
clone D	837075	836960	s	out	i02_0826	uvrB	CDS
clone D	848444	848329	s	out	i02_0841	ybhR	CDS
CFT073	863423	863308	ns	out	i02_0854	ybiL	CDS
CFT073	864421	864306	s	out	i02_0855	ybiM	CDS
clone D	864770	864655	ns	out	i02_0855	ybiM	CDS
clone D	866117	866002	ns	out	i02_0857	ybiO	CDS
CFT073	883494	883379	ns	out	i02_0872	ybiW	CDS
CFT073	892207	892092	ns	out	i02_0880	yliC	CDS
CFT073	909245	942611	s	out	i02_0896	ybjL	CDS
clone D	918512	951878	ns	out	i02_0906	potI	pseudogene
clone D	982886	1016254	ns	out	i02_0963	ycaI	CDS
CFT073	987068	1020437	ns	out	i02_0966	ycaQ	CDS
CFT073	987082	1020451	ns	out	i02_0966	ycaQ	CDS
clone D	988324	1021693	ns	out	i02_0967	ycaR	CDS
CFT073	1002042	1035411	ns	out	i02_0979	aspC	CDS
clone D	1011558	1044927	ns	out	i02_0984	ssuB	CDS
CFT073	1013482	1046851	ns	out	i02_0986	ycbN	CDS
CFT073	1017277	1050646	ns	out	i02_0991	ycbX	CDS
CFT073	1017536	1050905	s	out	i02_0991	ycbX	CDS
clone D	1017888	1051257	ns	out	i02_0991	ycbX	CDS
CFT073	1025532	1058901	ns	out	i02_0996	ymbA	CDS
clone D	1026896	1060265	ns	out	i02_0998	fabA	CDS
CFT073	1043140	1076509	s	out	i02_1017	hyaA	CDS
CFT073	1048389	1081758	ns	out	i02_1023	appC	CDS
CFT073	1051361	1084730	ns	out	i02_1025	appA	CDS
CFT073	1063558	1096927	ns	out	i02_1036	torD	CDS
clone D	1065603	1098972	nc	out	intergenic		
clone D	1071559	1104928	s	out	i02_1046	ycdJ	CDS
CFT073	1073352	1106721	s	out	i02_1049	/	CDS
clone D	1074028	1107397	ns	out	i02_1049	/	CDS

Lineage	Clone D site	CFT 073 site	mutation type[a]	recomb	Gene	Gene name	Type
?	1125611	1158981	nc	out	intergenic		
CFT073	1129793	1163163	nc	out	intergenic		
?	1131681	1165052	nc	out	intergenic		
?	1132196	1165567	s	out	i02_1100	/	CDS
?	1145111	1178484	s	out	i02_1120	mchC	CDS
clone D	1153965	1187338	nc	out	intergenic		
CFT073	1172295	1205668	s	out	i02_1147	iroC	CDS
CFT073	1194598	1229284	s	out	i02_1167	/	CDS
CFT073	1195856	1230542	ns	out	i02_1167	/	CDS
CFT073	1200857	1237959	nc	out	intergenic		
CFT073	1203224	1241037	nc	out	intergenic		
CFT073	1210308	1248122	ns	out	i02_1192	csgA	CDS
clone D	1212641	1250455	ns	out	i02_1196	ymdC	CDS
clone D	1235940	1273757	ns	out	i02_1225	flgA	CDS
CFT073	1236058	1273875	s	out	i02_1225	flgA	CDS
clone D	1249884	1287701	ns	out	i02_1237	rne	CDS
CFT073	1294835	1333367	ns	out	i02_1290	ydfA	CDS
clone D	1308118	1347361	nc	out	intergenic		
CFT073	1311991	1351234	s	out	i02_1322	/	CDS
clone D	1320170	1359413	s	out	i02_1334	/	CDS
clone D	1322018	1361261	s	rec	i02_1334	/	CDS
CFT073	1322019	1361262	ns	rec	i02_1334	/	CDS
clone D	1322021	1361264	ns	rec	i02_1334	/	CDS
clone D	1322033	1361276	s	rec	i02_1334	/	CDS
clone D	1322046	1361289	ns	rec	i02_1334	/	CDS
clone D	1322054	1361297	s	rec	i02_1334	/	CDS
clone D	1322058	1361301	ns	rec	i02_1334	/	CDS
clone D	1322060	1361303	ns	rec	i02_1334	/	CDS
clone D	1322063	1361306	s	rec	i02_1334	/	CDS
clone D	1322072	1361315	s	rec	i02_1334	/	CDS
clone D	1322084	1361327	s	rec	i02_1334	/	CDS
CFT073	1323330	1362573	s	out	i02_1336	/	CDS
clone D	1332492	1371735	nc	rec	intergenic		
clone D	1332511	1371754	nc	rec	intergenic		
clone D	1332512	1371755	nc	rec	intergenic		
clone D	1332525	1371768	nc	rec	intergenic		
clone D	1332558	1371801	nc	rec	intergenic		
clone D	1332560	1371803	nc	rec	intergenic		
clone D	1332561	1371804	nc	rec	intergenic		
clone D	1332579	1371822	nc	rec	intergenic		
clone D	1332627	1371870	ns	rec	i02_1344	/	CDS
CFT073	1343579	1394274	s	out	i02_1355	mnmA	CDS
CFT073	1343615	1394310	ns	out	i02_1355	mnmA	CDS
CFT073	1358433	1412847	s	out	i02_1383	/	CDS
CFT073	1358517	1412935	ns	out	i02_1383	/	CDS
clone D	1363135	1418868	ns	out	i02_1392	rus	CDS
clone D	1364569	1420302	ns	out	i02_1395	nmpC	CDS
clone D	1365402	1421135	ns	rec	i02_1395	nmpC	CDS
clone D	1365404	1421137	ns	rec	i02_1395	nmpC	CDS
clone D	1365406	1421139	ns	rec	i02_1395	nmpC	CDS
clone D	1365427	1421160	nc	rec	intergenic		

Lineage	Clone D site	CFT 073 site	mutation type[a]	recomb	Gene	Gene name	Type
clone D	1371117	1426850	ns	out	i02_1406	/	CDS
CFT073	1372049	1427782	s	out	i02_1408	/	CDS
clone D	1373356	1429089	ns	out	i02_1408	/	CDS
CFT073	1374811	1430544	nc	out	intergenic		
CFT073	1374984	1430717	ns	out	i02_1409	/	CDS
CFT073	1374985	1430718	ns	out	i02_1409	/	CDS
CFT073	1378342	1434074	ns	out	i02_1415	/	CDS
clone D	1378488	1434218	s	rec	i02_1415	/	CDS
CFT073	1378491	1434221	s	rec	i02_1415	/	CDS
CFT073	1378492	1434222	ns	rec	i02_1415	/	CDS
clone D	1382651	1438380	s	out	i02_1420	/	CDS
CFT073	1389904	1445633	ns	out	i02_1424	/	CDS
clone D	1408061	1463784	s	out	i02_1454	minC	CDS
CFT073	1413461	1469184	ns	out	i02_1463	umuC	CDS
CFT073	1415941	1471664	s	out	i02_1465	nhaB	CDS
CFT073	1422229	1477952	s	out	i02_1471	ycgO	CDS
CFT073	1427882	1483605	ns	out	i02_1476	prrA	CDS
CFT073	1439811	1495534	ns	out	i02_1489	/	CDS
CFT073	1452981	1508704	ns	out	i02_1503	kdsA	CDS
CFT073	1480109	1535832	ns	out	i02_1530	hnr	CDS
CFT073	1486554	1542277	ns	out	i02_1537	adhE	CDS
CFT073	1506794	1562517	s	out	i02_1559	trpC	CDS
CFT073	1529648	1586082	nc	out	i02_1580	pyrF	pseudogene
clone D	1531673	1588106	s	out	i02_1584	yciT	CDS
CFT073	1586860	1643294	ns	out	i02_1636	/	CDS
CFT073	1593037	1649471	ns	out	i02_1642	dbpA	CDS
CFT073	1596208	1652642	ns	out	i02_1645	/	CDS
clone D	1618956	1675538	s	out	i02_1662	ynbD	CDS
CFT073	1622436	1679018	ns	out	i02_1664	hrpA	CDS
CFT073	1631370	1687952	ns	out	i02_1672	ydcJ	CDS
CFT073	1643881	1700463	ns	out	i02_1687	ydcR	CDS
clone D	1663549	1720135	ns	out	i02_1710	/	CDS
clone D	1673112	1729696	nc	out	intergenic		
CFT073	1701270	1757853	nc	out	intergenic		
CFT073	1701329	1757912	nc	out	intergenic		
CFT073	1715067	1771649	ns	out	i02_1752	ydeP	CDS
CFT073	1724309	1781887	ns	out	i02_1761	hipA	CDS
clone D	1733001	1790581	ns	out	i02_1769	yneI	CDS
CFT073	1741553	1799133	s	out	i02_1778	/	CDS
clone D	1742588	1800168	s	out	i02_1780	/	CDS
CFT073	1760775	1818329	ns	out	i02_1799	ynfD	CDS
clone D	1781828	1839382	s	out	i02_1816	ydgH	CDS
clone D	1786712	1844266	s	out	i02_1821	rstB	CDS
CFT073	1793213	1850767	ns	out	i02_1826	ydgA	CDS
CFT073	1796475	1854029	ns	out	i02_1828	uidB	CDS
clone D	1802483	1860037	s	out	i02_1833	malX	CDS
CFT073	1830257	1887811	s	out	i02_1865	nemA	CDS
clone D	1840635	1898189	s	out	i02_1878	cfa	CDS
clone D	1853236	1910790	nc	out	intergenic		
CFT073	1855927	1913481	nc	rec	intergenic		
CFT073	1855928	1913482	nc	rec	intergenic		

Lineage	Clone D site	CFT 073 site	mutation type[a]	recomb	Gene	Gene name	Type
CFT073	1855940	1913494	nc	rec	intergenic		
CFT073	1855949	1913503	nc	rec	intergenic		
CFT073	1883806	1941360	ns	out	i02_1920	ppsA	CDS
CFT073	1895916	1953470	ns	out	i02_1932	pheT	CDS
CFT073	1908823	1966378	s	out	i02_1949	ydjN	CDS
CFT073	1926109	1983664	ns	out	i02_1966	/	CDS
CFT073	1929047	1986602	s	out	i02_1969	xthA	CDS
clone D	1946402	2003957	ns	out	i02_1985	topB	CDS
CFT073	1961111	2018666	nc	out	intergenic		
clone D	1977237	2034790	nc	out	intergenic		
CFT073	1986053	2043606	s	out	i02_2032	/	CDS
CFT073	1987686	2045239	s	out	i02_2033	yoaB	CDS
CFT073	1988983	2046536	ns	out	i02_2037	pabB	CDS
clone D	1998937	2056490	s	out	i02_2045	yobD	CDS
clone D	2002055	2059608	nc	out	intergenic		
?	2010225	2067779	nc	out	intergenic		
CFT073	2017482	2075040	nc	out	intergenic		
CFT073	2044891	2102449	ns	out	i02_2097	yecD	CDS
CFT073	2044949	2102507	s	out	i02_2097	yecD	CDS
clone D	2054332	2111890	s	out	i02_2108	argS	CDS
CFT073	2070718	2128276	nc	out	intergenic		
CFT073	2070754	2128312	nc	out	intergenic		
CFT073	2077269	2134821	ns	out	i02_2131	araF	CDS
CFT073	2078871	2136423	s	out	i02_2133	/	CDS
CFT073	2107023	2165287	s	out	i02_2169	fliF	CDS
CFT073	2109941	2168205	s	out	i02_2172	fliH	CDS
clone D	2112295	2170559	ns	out	i02_2175	fliK	CDS
CFT073	2135380	2193644	ns	out	i02_2204	yodA	CDS
CFT073	2143727	2201991	ns	out	i02_2211	/	CDS
CFT073	2159887	2218151	nc	out	intergenic		
clone D	2165206	2223470	ns	out	i02_2238	/	CDS
CFT073	2170495	2228759	ns	out	i02_2241	/	CDS
clone D	2180055	2239019	s	rec	i02_2242	/	CDS
clone D	2180056	2239020	s	rec	i02_2242	/	CDS
clone D	2180064	2239026	ns	rec	i02_2242	/	CDS
clone D	2185505	2244467	ns	out	i02_2244	/	CDS
clone D	2190481	2249443	nc	out	intergenic		
CFT073	2199173	2258133	s	out	i02_2255	/	CDS
CFT073	2202789	2261749	ns	out	i02_2258	yeeO	CDS
clone D	2203718	2262678	nc	out	intergenic		
CFT073	2237404	2296365	s	out	i02_2271	/	CDS
CFT073	2240758	2299719	s	out	i02_2274	/	CDS
CFT073	2240778	2299739	ns	out	i02_2274	/	CDS
CFT073	2246105	2305066	s	out	i02_2279	/	CDS
CFT073	2249273	2308234	s	out	i02_2279	/	CDS
clone D	2258968	2317914	ns	out	i02_2286	erfK	CDS
clone D	2275876	2334825	ns	out	i02_2302	/	CDS
clone D	2282740	2341764	ns	out	i02_2310	/	CDS
CFT073	2290069	2351509	nc	out	intergenic		
clone D	2290636	2352076	nc	out	intergenic		
CFT073	2293206	2354646	s	out	i02_2321	/	CDS

Lineage	Clone D site	CFT 073 site	mutation type[a]	recomb	Gene	Gene name	Type
CFT073	2296423	2357863	nc	rec	intergenic		
CFT073	2296445	2357885	nc	rec	intergenic		
CFT073	2296515	2357955	nc	rec	intergenic		
clone D	2296517	2357957	nc	rec	intergenic		
CFT073	2296518	2357958	nc	rec	intergenic		
clone D	2296557	2357997	nc	rec	intergenic		
clone D	2296559	2357999	nc	rec	intergenic		
CFT073	2296666	2358106	nc	rec	intergenic		
CFT073	2296690	2358130	nc	rec	intergenic		
clone D	2296802	2358242	nc	rec	intergenic		
clone D	2296803	2358243	nc	rec	intergenic		
clone D	2296816	2358256	nc	rec	intergenic		
clone D	2296817	2358257	nc	rec	intergenic		
clone D	2296820	2358260	nc	rec	intergenic		
clone D	2296825	2358265	nc	rec	intergenic		
clone D	2296826	2358266	nc	rec	intergenic		
clone D	2296835	2358275	nc	rec	intergenic		
clone D	2296838	2358278	nc	rec	intergenic		
clone D	2296845	2358285	nc	rec	intergenic		
clone D	2296990	2358430	nc	rec	intergenic		
CFT073	2296992	2358432	nc	rec	intergenic		
clone D	2296994	2358434	nc	rec	intergenic		
CFT073	2298518	2359959	s	rec	i02_2323	/	CDS
CFT073	2299407	2360848	ns	rec	i02_2323	/	CDS
clone D	2299410	2360851	s	rec	i02_2323	/	CDS
CFT073	2299700	2361141	ns	rec	i02_2325	/	CDS
CFT073	2299712	2361153	ns	rec	i02_2325	/	CDS
clone D	2299990	2361431	s	rec	i02_2325	/	CDS
clone D	2300703	2362144	s	rec	i02_2326	/	CDS
clone D	2300793	2362234	s	rec	i02_2326	/	CDS
CFT073	2309336	2370777	nc	out	intergenic		
CFT073	2315229	2376670	s	out	i02_2343	/	CDS
CFT073	2320221	2381662	s	out	i02_2349	hisB	CDS
CFT073	2336808	2398250	ns	out	i02_2364	/	CDS
CFT073	2339703	2401145	ns	out	i02_2366	galF	CDS
clone D	2345037	2406286	s	out	i02_2370	wzxC	CDS
CFT073	2356361	2417610	ns	out	i02_2381	wcaD	CDS
CFT073	2361324	2422573	ns	out	i02_2385	/	CDS
CFT073	2362173	2423422	s	out	i02_2386	/	CDS
CFT073	2362253	2423501	ns	out	i02_2386	/	CDS
CFT073	2362254	2423502	ns	out	i02_2386	/	CDS
CFT073	2365761	2427006	s	out	i02_2388	yegH	CDS
clone D	2376994	2438239	s	out	i02_2396	yegI	CDS
clone D	2394630	2455875	s	out	i02_2410	/	CDS
clone D	2405478	2466723	ns	out	i02_2422	gatY	CDS
clone D	2406271	2468227	ns	out	i02_2423	/	CDS
CFT073	2409395	2471351	s	rec	i02_2426	yegU	CDS
CFT073	2409418	2471374	ns	rec	i02_2426	yegU	CDS
CFT073	2409443	2471399	ns	rec	i02_2426	yegU	CDS
CFT073	2409465	2471421	s	rec	i02_2426	yegU	CDS
clone D	2414055	2476011	nc	out	intergenic		

Lineage	Clone D site	CFT 073 site	mutation type[a]	recomb	Gene	Gene name	Type
CFT073	2428483	2491151	ns	out	i02_2442	/	CDS
CFT073	2433356	2496024	nc	out	intergenic		
CFT073	2440988	2503656	ns	out	i02_2452	yehS	CDS
clone D	2443259	2505927	ns	out	i02_2454	yehU	CDS
clone D	2443878	2506546	nc	out	intergenic		
clone D	2444543	2507211	s	out	i02_2455	yehV	CDS
CFT073	2485863	2548531	ns	rec	i02_2500	yeiN	CDS
CFT073	2485896	2548564	ns	rec	i02_2500	yeiN	CDS
CFT073	2485899	2548567	s	rec	i02_2500	yeiN	CDS
clone D	2485913	2548581	ns	rec	i02_2500	yeiN	CDS
?	2485914	2548584	ns	rec	i02_2500	yeiN	CDS
CFT073	2485917	2548587	ns	rec	i02_2500	yeiN	CDS
CFT073	2485922	2548592	ns	rec	i02_2500	yeiN	CDS
CFT073	2497637	2560307	nc	out	intergenic		
CFT073	2506161	2568831	nc	out	intergenic		
CFT073	2517522	2580192	ns	out	i02_2533	dsbE	CDS
CFT073	2525558	2588228	s	out	i02_2544	napA	CDS
CFT073	2542586	2605256	s	out	i02_2559	rcsC	CDS
CFT073	2618491	2681161	ns	out	i02_2622	nuoG	CDS
CFT073	2625164	2687834	ns	out	i02_2628	/	CDS
CFT073	2652749	2715419	ns	out	i02_2658	accD	CDS
CFT073	2658773	2721443	ns	out	i02_2665	yfcJ	CDS
clone D	2665170	2727838	s	out	i02_2671	mepA	CDS
clone D	2685309	2747977	s	out	i02_2693	/	CDS
CFT073	2695383	2758071	nc	out	intergenic		
CFT073	2704159	2766847	ns	out	i02_2704	evgS	CDS
clone D	2715730	2778418	ns	out	i02_2712	ddg	CDS
CFT073	2717882	2780570	ns	out	i02_2713	yfdZ	CDS
CFT073	2718009	2780697	ns	out	i02_2714	/	CDS
CFT073	2718020	2780707	nc	out	intergenic		
CFT073	2724895	2787582	ns	out	i02_2720	ypdE	CDS
CFT073	2739621	2802545	ns	out	i02_2734	xapR	CDS
clone D	2757922	2820848	ns	out	i02_2755	cysW	CDS
CFT073	2783069	2845995	ns	out	i02_2783	/	CDS
clone D	2792952	2855878	s	out	i02_2790	yffG	CDS
CFT073	2811130	2874056	s	out	i02_2807	yfgC	CDS
CFT073	2838616	2901542	ns	out	i02_2826	/	CDS
CFT073	2848250	2911176	ns	out	i02_2834	yfgA	CDS
CFT073	2863200	2926117	ns	out	i02_2846	hscA	CDS
CFT073	2871211	2934128	ns	out	i02_2856	csiE	CDS
CFT073	2871849	2934766	ns	out	i02_2857	hcaT	CDS
CFT073	2874724	2937641	ns	out	i02_2861	yphB	CDS
clone D	2885814	2948732	s	out	i02_2870	hmpA	CDS
CFT073	2890031	2952949	s	out	i02_2874	yfhK	CDS
clone D	2896986	2959904	ns	out	i02_2876	yfhD	pseudogene
clone D	2927090	2990008	nc	rec	23s rRNA	rrl	rRNA
clone D	2927353	2990271	nc	rec	23s rRNA	rrl	rRNA
CFT073	2927412	2990330	nc	rec	23s rRNA	rrl	rRNA
CFT073	2927454	2990372	nc	rec	23s rRNA	rrl	rRNA
CFT073	2927730	2990648	nc	rec	23s rRNA	rrl	rRNA
CFT073	2927731	2990649	nc	rec	23s rRNA	rrl	rRNA

Lineage	Clone D site	CFT 073 site	mutation type[a]	recomb	Gene	Gene name	Type
CFT073	2927740	2990658	nc	rec	23s rRNA	rrl	rRNA
CFT073	2927741	2990659	nc	rec	23s rRNA	rrl	rRNA
CFT073	2927749	2990667	nc	rec	23s rRNA	rrl	rRNA
CFT073	2927782	2990700	nc	rec	23s rRNA	rrl	rRNA
CFT073	2927788	2990707	nc	rec	23s rRNA	rrl	rRNA
CFT073	2928416	2991335	nc	rec	23s rRNA	rrl	rRNA
clone D	2928601	2991521	nc	rec	23s rRNA	rrl	rRNA
CFT073	2928602	2991522	nc	rec	23s rRNA	rrl	rRNA
clone D	2928642	2991562	nc	rec	23s rRNA	rrl	rRNA
CFT073	2928694	2991614	nc	rec	23s rRNA	rrl	rRNA
CFT073	2928875	2991795	nc	rec	23s rRNA	rrl	rRNA
CFT073	2929027	2991938	nc	rec	intergenic		
CFT073	2929030	2991941	nc	rec	intergenic		
CFT073	2929083	2991994	nc	rec	intergenic		
CFT073	2929118	2992029	nc	rec	intergenic		
CFT073	2929122	2992033	nc	rec	intergenic		
CFT073	2929123	2992034	nc	rec	intergenic		
CFT073	2929126	2992037	nc	rec	intergenic		
CFT073	2929257	2992254	nc	rec	intergenic		
CFT073	2929266	2992263	nc	rec	intergenic		
CFT073	2929267	2992264	nc	rec	intergenic		
CFT073	2929475	2992471	nc	out	16s rRNA	rrs	rRNA
CFT073	2930220	2993220	nc	out	16s rRNA	rrs	rRNA
CFT073	2930451	2993455	nc	out	16s rRNA	rrs	rRNA
CFT073	2955443	3018449	nc	out	intergenic		
CFT073	2955497	3018503	nc	out	intergenic		
CFT073	2962639	3074078	ns	out	i02_2942	gabP	CDS
clone D	2966998	3078437	ns	out	i02_2952	ygaC	CDS
clone D	2968995	3080434	s	out	i02_2954	/	CDS
clone D	2995994	3107433	s	out	i02_2986	mltB	CDS
clone D	3029683	3141123	s	out	i02_3024	pphB	CDS
CFT073	3061684	3173132	ns	out	i02_3058	/	CDS
CFT073	3065845	3177293	nc	out	intergenic		
CFT073	3067813	3179261	ns	out	i02_3065	/	CDS
CFT073	3082607	3194070	ns	out	i02_3076	ygcX	CDS
CFT073	3086823	3198288	ns	out	i02_3082	yqcB	CDS
CFT073	3098292	3209757	ns	out	i02_3094	fucP	CDS
CFT073	3115270	3226734	s	out	i02_3110	/	CDS
CFT073	3116851	3228315	s	out	i02_3111	/	CDS
clone D	3128970	3240434	ns	out	i02_3119	/	CDS
clone D	3132900	3244364	s	out	i02_3121	/	CDS
clone D	3155664	3267126	ns	out	i02_3138	recC	CDS
CFT073	3168315	3279777	ns	out	i02_3150	tas	CDS
CFT073	3180307	3292480	s	out	i02_3160	yqeF	CDS
CFT073	3185198	3297371	ns	out	i02_3163	/	CDS
CFT073	3187493	3299666	s	out	i02_3164	ygeT	CDS
clone D	3196692	3308865	nc	out	intergenic		
clone D	3216776	3328949	s	out	i02_3184	ygfU	CDS
clone D	3237513	3350397	s	out	i02_3210	pepP	CDS
CFT073	3255888	3368772	s	out	i02_3232	frcK	CDS
CFT073	3256744	3369628	ns	out	i02_3233	yggD	CDS

Lineage	Clone D site	CFT 073 site	mutation type[a]	recomb	Gene	Gene name	Type
clone D	3261350	3374232	s	out	i02_3238	/	CDS
clone D	3261353	3374233	ns	out	i02_3238	/	CDS
CFT073	3266500	3379379	s	out	i02_3242	/	CDS
CFT073	3269374	3382253	nc	out	intergenic		
CFT073	3269377	3382256	nc	out	intergenic		
CFT073	3275727	3388606	s	out	i02_3251	gshB	CDS
clone D	3293438	3406317	nc	out	intergenic		
CFT073	3294224	3407103	s	rec	i02_3273	/	CDS
CFT073	3294230	3407109	s	rec	i02_3273	/	CDS
CFT073	3294242	3407121	s	rec	i02_3273	/	CDS
CFT073	3294267	3407146	ns	rec	i02_3273	/	CDS
CFT073	3294270	3407149	ns	rec	i02_3273	/	CDS
CFT073	3294281	3407160	s	rec	i02_3273	/	CDS
CFT073	3294296	3407175	s	rec	i02_3273	/	CDS
CFT073	3294299	3407178	s	rec	i02_3273	/	CDS
CFT073	3294302	3407181	s	rec	i02_3273	/	CDS
CFT073	3294332	3407211	s	rec	i02_3273	/	CDS
CFT073	3294408	3407287	ns	rec	i02_3273	/	CDS
CFT073	3294431	3407310	s	rec	i02_3273	/	CDS
CFT073	3294435	3407314	ns	rec	i02_3273	/	CDS
clone D	3295195	3408074	ns	rec	i02_3274	/	CDS
CFT073	3295338	3408217	ns	rec	i02_3274	/	CDS
clone D	3295341	3408220	s	rec	i02_3274	/	CDS
CFT073	3295391	3408270	s	rec	i02_3274	/	CDS
clone D	3295410	3408289	ns	rec	i02_3274	/	CDS
CFT073	3296118	3408997	ns	rec	i02_3274	/	CDS
CFT073	3296224	3409103	nc	rec	intergenic		
CFT073	3296276	3409155	nc	rec	intergenic		
CFT073	3296420	3409299	ns	rec	i02_3275	/	CDS
CFT073	3296553	3409420	s	rec	i02_3275	/	CDS
clone D	3296658	3409525	nc	rec	intergenic		
clone D	3296659	3409526	nc	rec	intergenic		
CFT073	3297837	3428739	nc	rec	intergenic		
CFT073	3297854	3428756	nc	rec	intergenic		
CFT073	3297859	3428761	nc	rec	intergenic		
CFT073	3297865	3428767	nc	rec	intergenic		
CFT073	3297866	3428768	nc	rec	intergenic		
CFT073	3297872	3428774	nc	rec	intergenic		
CFT073	3297874	3428776	nc	rec	intergenic		
CFT073	3297887	3428789	ns	rec	i02_3278	papX	CDS
CFT073	3297893	3428795	ns	rec	i02_3278	papX	CDS
CFT073	3297894	3428796	ns	rec	i02_3278	papX	CDS
CFT073	3297897	3428799	ns	rec	i02_3278	papX	CDS
CFT073	3297901	3428803	s	rec	i02_3278	papX	CDS
clone D	3297910	3428812	s	rec	i02_3278	papX	CDS
CFT073	3297959	3428861	ns	rec	i02_3278	papX	CDS
CFT073	3298070	3428972	ns	rec	i02_3278	papX	CDS
CFT073	3298174	3429076	ns	rec	i02_3278	papX	CDS
CFT073	3298175	3429077	ns	rec	i02_3278	papX	CDS
CFT073	3298211	3429113	ns	rec	i02_3278	papX	CDS
CFT073	3298288	3429190	s	rec	i02_3278	papX	CDS

Lineage	Clone D site	CFT 073 site	mutation type[a]	recomb	Gene	Gene name	Type
CFT073	3298303	3429205	s	rec	i02_3278	papX	CDS
CFT073	3298472	3429374	nc	rec	intergenic		
CFT073	3298487	3429389	nc	rec	intergenic		
CFT073	3298490	3429392	nc	rec	intergenic		
CFT073	3298494	3429396	nc	rec	intergenic		
CFT073	3298495	3429397	nc	rec	intergenic		
CFT073	3298496	3429398	nc	rec	intergenic		
CFT073	3298498	3429400	nc	rec	intergenic		
CFT073	3298501	3429403	nc	rec	intergenic		
CFT073	3298505	3429408	nc	rec	intergenic		
CFT073	3298512	3429415	nc	rec	intergenic		
CFT073	3298524	3429427	nc	rec	intergenic		
CFT073	3298528	3429431	nc	rec	intergenic		
CFT073	3299818	3437355	nc	rec	i02_3282	/	pseudogene
CFT073	3299820	3437357	nc	rec	i02_3282	/	pseudogene
CFT073	3299821	3437358	nc	rec	i02_3282	/	pseudogene
CFT073	3299839	3437376	nc	rec	i02_3282	/	pseudogene
CFT073	3299848	3437385	nc	rec	i02_3282	/	pseudogene
clone D	3299851	3437388	nc	rec	i02_3282	/	pseudogene
clone D	3299853	3437390	nc	rec	i02_3282	/	pseudogene
clone D	3299854	3437391	nc	rec	i02_3282	/	pseudogene
clone D	3299855	3437392	nc	rec	i02_3282	/	pseudogene
clone D	3299856	3437393	nc	rec	i02_3282	/	pseudogene
clone D	3299858	3437395	nc	rec	i02_3282	/	pseudogene
clone D	3299859	3437396	nc	rec	i02_3282	/	pseudogene
CFT073	3299863	3437400	nc	rec	i02_3282	/	pseudogene
CFT073	3299865	3437402	nc	rec	i02_3282	/	pseudogene
CFT073	3299866	3437403	nc	rec	i02_3282	/	pseudogene
CFT073	3299872	3437409	nc	rec	i02_3282	/	pseudogene
CFT073	3299878	3437415	nc	rec	i02_3282	/	pseudogene
CFT073	3299881	3437418	nc	rec	i02_3282	/	pseudogene
clone D	3299896	3437433	nc	rec	i02_3282	/	pseudogene
clone D	3299903	3437440	nc	rec	i02_3282	/	pseudogene
CFT073	3299917	3437454	nc	rec	i02_3282	/	pseudogene
clone D	3299921	3437458	nc	rec	i02_3282	/	pseudogene
clone D	3299927	3437464	nc	rec	i02_3282	/	pseudogene
CFT073	3299937	3437474	nc	rec	i02_3282	/	pseudogene
CFT073	3299939	3437476	nc	rec	i02_3282	/	pseudogene
clone D	3300156	3437692	nc	rec	intergenic		
CFT073	3300217	3437753	nc	rec	intergenic		
CFT073	3300438	3437975	s	rec	i02_3284	/	CDS
clone D	3300442	3437979	ns	rec	i02_3284	/	CDS
clone D	3300450	3437987	s	rec	i02_3284	/	CDS
clone D	3300651	3438188	nc	rec	intergenic		
CFT073	3300656	3438193	nc	rec	intergenic		
clone D	3300661	3438198	nc	rec	intergenic		
clone D	3300662	3438199	nc	rec	intergenic		
CFT073	3300667	3438204	nc	rec	intergenic		
CFT073	3300669	3438206	nc	rec	intergenic		
CFT073	3300675	3438212	nc	rec	intergenic		
CFT073	3300676	3438213	nc	rec	intergenic		

Lineage	Clone D site	CFT 073 site	mutation type[a]	recomb	Gene	Gene name	Type
CFT073	3300677	3438214	nc	rec	intergenic		
CFT073	3300678	3438215	nc	rec	intergenic		
CFT073	3300700	3438237	nc	rec	intergenic		
CFT073	3300736	3438273	nc	rec	intergenic		
CFT073	3300758	3438295	nc	rec	intergenic		
CFT073	3300770	3438307	nc	rec	intergenic		
CFT073	3300773	3438310	nc	rec	intergenic		
CFT073	3300799	3438336	nc	rec	intergenic		
CFT073	3300804	3438341	nc	rec	intergenic		
CFT073	3300827	3438364	nc	rec	intergenic		
CFT073	3300844	3438381	nc	rec	intergenic		
CFT073	3300968	3438505	ns	rec	i02_3286	papI	CDS
CFT073	3300982	3438519	s	rec	i02_3286	papI	CDS
CFT073	3300994	3438531	s	rec	i02_3286	papI	CDS
clone D	3301018	3438555	s	rec	i02_3286	papI	CDS
CFT073	3301024	3438561	s	rec	i02_3286	papI	CDS
CFT073	3301053	3438590	ns	rec	i02_3286	papI	CDS
clone D	3301058	3438595	ns	rec	i02_3286	papI	CDS
CFT073	3301072	3438609	s	rec	i02_3286	papI	CDS
CFT073	3301081	3438618	s	rec	i02_3286	papI	CDS
CFT073	3301084	3438621	s	rec	i02_3286	papI	CDS
CFT073	3301103	3438640	ns	rec	i02_3286	papI	CDS
clone D	3301134	3438671	ns	rec	i02_3286	papI	CDS
CFT073	3301207	3438744	nc	rec	intergenic		
CFT073	3301259	3438796	nc	rec	intergenic		
clone D	3301601	3439138	nc	rec	intergenic		
clone D	3301681	3439218	nc	rec	intergenic		
clone D	3301806	3439343	nc	rec	i02_3287	/	pseudogene
CFT073	3301871	3439408	nc	rec	i02_3287	/	pseudogene
CFT073	3302088	3439625	nc	rec	i02_3287	/	pseudogene
CFT073	3302195	3439732	nc	rec	i02_3287	/	pseudogene
clone D	3302450	3439986	nc	rec	i02_3287	/	pseudogene
clone D	3302473	3440009	nc	rec	i02_3287	/	pseudogene
clone D	3302474	3440010	nc	rec	i02_3287	/	pseudogene
clone D	3302475	3440011	nc	rec	i02_3287	/	pseudogene
clone D	3302503	3440039	nc	rec	i02_3287	/	pseudogene
CFT073	3302505	3440041	nc	rec	i02_3287	/	pseudogene
clone D	3302546	3440082	nc	rec	i02_3287	/	pseudogene
CFT073	3302780	3440316	nc	rec	intergenic		
CFT073	3302812	3440348	nc	rec	intergenic		
clone D	3302823	3440359	nc	rec	intergenic		
CFT073	3302843	3440379	nc	rec	intergenic		
clone D	3302954	3440490	nc	rec	intergenic		
CFT073	3303385	3440921	nc	rec	intergenic		
CFT073	3303388	3440924	nc	rec	intergenic		
CFT073	3303391	3440927	nc	rec	intergenic		
clone D	3303526	3441062	nc	rec	intergenic		
CFT073	3303660	3441196	nc	rec	intergenic		
CFT073	3304080	3441608	ns	rec	i02_3289	/	CDS
CFT073	3304283	3441811	ns	rec	i02_3289	/	CDS
CFT073	3304329	3441857	ns	rec	i02_3289	/	CDS

Lineage	Clone D site	CFT 073 site	mutation type[a]	recomb	Gene	Gene name	Type
CFT073	3304390	3441918	ns	rec	i02_3290	/	CDS
CFT073	3304962	3442490	nc	rec	intergenic		
CFT073	3305225	3442753	s	rec	i02_3291	/	CDS
CFT073	3305488	3443016	s	rec	i02_3292	/	CDS
clone D	3305778	3443306	ns	rec	i02_3292	/	CDS
CFT073	3306079	3443607	s	rec	i02_3293	/	CDS
CFT073	3306278	3443806	ns	rec	i02_3293	/	CDS
?	3306947	3444082	nc	rec	intergenic		
?	3307024	3444159	s	rec	i02_3294	/	CDS
CFT073	3307069	3444204	s	rec	i02_3294	/	CDS
CFT073	3307156	3444291	s	rec	i02_3294	/	CDS
?	3307182	3444317	ns	rec	i02_3294	/	CDS
?	3307192	3444327	s	rec	i02_3294	/	CDS
clone D	3307195	3444330	s	rec	i02_3294	/	CDS
clone D	3307231	3444366	s	rec	i02_3294	/	CDS
clone D	3307261	3444396	s	rec	i02_3294	/	CDS
clone D	3307270	3444405	s	rec	i02_3294	/	CDS
clone D	3307321	3444455	s	rec	i02_3294	/	CDS
?	3307344	3444478	ns	rec	i02_3294.2	/	CDS
?	3307377	3444511	ns	rec	i02_3294.2	/	CDS
CFT073	3307432	3444566	ns	rec	i02_3294.2	/	CDS
CFT073	3307445	3444579	ns	rec	i02_3294.2	/	CDS
CFT073	3307449	3444583	s	rec	i02_3294.2	/	CDS
?	3307461	3444595	s	rec	i02_3294.2	/	CDS
?	3307470	3444604	s	rec	i02_3294.2	/	CDS
?	3307490	3444624	ns	rec	i02_3294.2	/	CDS
?	3307498	3444632	ns	rec	i02_3294.2	/	CDS
?	3307518	3444652	s	rec	i02_3294.2	/	CDS
?	3307524	3444658	s	rec	i02_3294.2	/	CDS
?	3307563	3444697	s	rec	i02_3294.2	/	CDS
?	3307564	3444698	ns	rec	i02_3294.2	/	CDS
clone D	3307566	3444700	ns	rec	i02_3294.2	/	CDS
?	3307613	3444747	ns	rec	i02_3294.2	/	CDS
?	3307648	3444782	ns	rec	i02_3294.2	/	CDS
?	3307680	3444814	s	rec	i02_3294.2	/	CDS
CFT073	3307685	3444819	ns	rec	i02_3294.2	/	CDS
clone D	3307692	3444826	s	rec	i02_3294.2	/	CDS
clone D	3307704	3444838	s	rec	i02_3294.2	/	CDS
?	3307731	3444865	s	rec	i02_3294.2	/	CDS
CFT073	3307764	3444898	s	rec	i02_3294.2	/	CDS
?	3307818	3444952	s	rec	i02_3294.2	/	CDS
?	3307824	3444958	s	rec	i02_3294.2	/	CDS
?	3307866	3445000	s	rec	i02_3294.2	/	CDS
clone D	3307881	3445015	s	rec	i02_3294.2	/	CDS
clone D	3307886	3445020	ns	rec	i02_3294.2	/	CDS
?	3307908	3445042	s	rec	i02_3294.2	/	CDS
?	3307944	3445078	s	rec	i02_3294.2	/	CDS
?	3307956	3445090	s	rec	i02_3294.2	/	CDS
clone D	3307966	3445100	s	rec	i02_3294.2	/	CDS
?	3307998	3445132	s	rec	i02_3294.2	/	CDS
?	3308022	3445156	s	rec	i02_3294.2	/	CDS

Lineage	Clone D site	CFT 073 site	mutation type[a]	recomb	Gene	Gene name	Type
?	3308073	3445207	ns	rec	i02_3294.2	/	CDS
clone D	3308155	3445289	s	rec	i02_3294.2	/	CDS
clone D	3308191	3445325	ns	rec	i02_3294.2	/	CDS
?	3308197	3445331	ns	rec	i02_3294.2	/	CDS
?	3308200	3445334	ns	rec	i02_3294.2	/	CDS
clone D	3308220	3445354	s	rec	i02_3294.2	/	CDS
CFT073	3308660	3445794	nc	rec	intergenic		
CFT073	3308694	3445828	nc	rec	intergenic		
clone D	3308827	3445961	s	rec	i02_3295	/	CDS
CFT073	3309474	3446608	ns	rec	i02_3295	/	CDS
CFT073	3309942	3447076	ns	rec	i02_3295	/	CDS
CFT073	3310139	3447273	ns	rec	i02_3295	/	CDS
CFT073	3310171	3447305	s	rec	i02_3295	/	CDS
CFT073	3310870	3448004	nc	rec	intergenic		
clone D	3311048	3448182	nc	rec	intergenic		
clone D	3311422	3450966	nc	rec	intergenic		
?	3311423	3450967	nc	rec	intergenic		
?	3311686	3451235	s	rec	i02_3297	/	CDS
?	3311716	3451265	s	rec	i02_3297	/	CDS
clone D	3311782	3451331	s	rec	i02_3297	/	CDS
clone D	3311819	3451368	ns	rec	i02_3297	/	CDS
clone D	3311914	3451463	s	rec	i02_3297	/	CDS
CFT073	3311941	3451490	nc	rec	intergenic		
CFT073	3312233	3451782	ns	rec	i02_3299	/	CDS
clone D	3312309	3451858	ns	rec	i02_3299	/	CDS
CFT073	3312316	3451865	ns	rec	i02_3299	/	CDS
CFT073	3312383	3451932	ns	rec	i02_3299	/	CDS
CFT073	3312384	3451933	ns	rec	i02_3299	/	CDS
CFT073	3312390	3451939	s	rec	i02_3299	/	CDS
CFT073	3312394	3451943	ns	rec	i02_3299	/	CDS
CFT073	3312426	3451975	s	rec	i02_3299	/	CDS
CFT073	3312716	3452265	ns	rec	i02_3300	/	CDS
clone D	3312792	3452341	s	rec	i02_3300	/	CDS
?	3312987	3452536	ns	rec	i02_3301	/	CDS
clone D	3313102	3452651	s	rec	i02_3301	/	CDS
clone D	3316143	3455659	nc	rec	intergenic		
clone D	3316224	3455740	s	rec	i02_3308	/	CDS
CFT073	3316401	3455917	s	rec	i02_3308	/	CDS
CFT073	3316496	3456012	ns	rec	i02_3308	/	CDS
clone D	3319441	3456467	s	rec	i02_3313	sat	CDS
clone D	3319594	3456620	s	rec	i02_3313	sat	CDS
CFT073	3320101	3457127	ns	rec	i02_3313	sat	CDS
CFT073	3320544	3457570	ns	rec	i02_3313	sat	CDS
CFT073	3320698	3457724	s	rec	i02_3313	sat	CDS
clone D	3320950	3457976	s	rec	i02_3313	sat	CDS
CFT073	3321039	3458065	ns	rec	i02_3313	sat	CDS
CFT073	3321218	3458244	ns	rec	i02_3313	sat	CDS
clone D	3321352	3458378	s	rec	i02_3313	sat	CDS
clone D	3321390	3458416	ns	rec	i02_3313	sat	CDS
CFT073	3321403	3458429	s	rec	i02_3313	sat	CDS
CFT073	3321489	3458515	ns	rec	i02_3313	sat	CDS

Lineage	Clone D site	CFT 073 site	mutation type[a]	recomb	Gene	Gene name	Type
CFT073	3321796	3458822	s	rec	i02_3313	sat	CDS
CFT073	3322170	3459196	ns	rec	i02_3314	/	CDS
clone D	3322270	3459296	s	rec	i02_3313	sat	CDS
CFT073	3322805	3459831	ns	rec	i02_3313	sat	CDS
CFT073	3322831	3459857	s	rec	i02_3313	sat	CDS
CFT073	3323432	3460458	nc	rec	intergenic		
clone D	3323620	3460646	nc	rec	intergenic		
CFT073	3323879	3460905	nc	rec	intergenic		
CFT073	3324164	3461190	nc	rec	intergenic		
clone D	3324179	3461205	s	rec	i02_3316	iutA	CDS
clone D	3324208	3461234	s	rec	i02_3316	iutA	CDS
clone D	3324230	3461256	s	rec	i02_3316	iutA	CDS
clone D	3324233	3461259	s	rec	i02_3316	iutA	CDS
clone D	3324260	3461286	s	rec	i02_3316	iutA	CDS
clone D	3324272	3461298	s	rec	i02_3316	iutA	CDS
clone D	3324287	3461313	s	rec	i02_3316	iutA	CDS
clone D	3324308	3461334	s	rec	i02_3316	iutA	CDS
clone D	3324313	3461339	ns	rec	i02_3316	iutA	CDS
clone D	3324329	3461355	s	rec	i02_3316	iutA	CDS
clone D	3324344	3461370	ns	rec	i02_3316	iutA	CDS
clone D	3324345	3461371	ns	rec	i02_3316	iutA	CDS
clone D	3324350	3461376	ns	rec	i02_3316	iutA	CDS
clone D	3324352	3461378	ns	rec	i02_3316	iutA	CDS
clone D	3324355	3461381	ns	rec	i02_3316	iutA	CDS
clone D	3324356	3461382	ns	rec	i02_3316	iutA	CDS
clone D	3324358	3461384	ns	rec	i02_3316	iutA	CDS
clone D	3324362	3461388	s	rec	i02_3316	iutA	CDS
clone D	3324368	3461394	s	rec	i02_3316	iutA	CDS
clone D	3324371	3461397	s	rec	i02_3316	iutA	CDS
clone D	3324374	3461400	s	rec	i02_3316	iutA	CDS
clone D	3324379	3461405	ns	rec	i02_3316	iutA	CDS
clone D	3324380	3461406	ns	rec	i02_3316	iutA	CDS
clone D	3324382	3461408	ns	rec	i02_3316	iutA	CDS
clone D	3324383	3461409	ns	rec	i02_3316	iutA	CDS
clone D	3324385	3461411	ns	rec	i02_3316	iutA	CDS
clone D	3324388	3461414	ns	rec	i02_3316	iutA	CDS
clone D	3324389	3461415	s	rec	i02_3316	iutA	CDS
clone D	3324394	3461420	ns	rec	i02_3316	iutA	CDS
clone D	3324395	3461421	s	rec	i02_3316	iutA	CDS
clone D	3324398	3461424	s	rec	i02_3316	iutA	CDS
clone D	3324404	3461430	s	rec	i02_3316	iutA	CDS
clone D	3324410	3461436	s	rec	i02_3316	iutA	CDS
clone D	3324413	3461439	s	rec	i02_3316	iutA	CDS
clone D	3324416	3461442	s	rec	i02_3316	iutA	CDS
clone D	3324428	3461454	s	rec	i02_3316	iutA	CDS
clone D	3324431	3461457	s	rec	i02_3316	iutA	CDS
clone D	3324446	3461472	ns	rec	i02_3316	iutA	CDS
clone D	3324452	3461478	s	rec	i02_3316	iutA	CDS
clone D	3324459	3461485	ns	rec	i02_3316	iutA	CDS
clone D	3324460	3461486	ns	rec	i02_3316	iutA	CDS
clone D	3324467	3461493	s	rec	i02_3316	iutA	CDS

Lineage	Clone D site	CFT 073 site	mutation type[a]	recomb	Gene	Gene name	Type
clone D	3324479	3461505	s	rec	i02_3316	iutA	CDS
clone D	3324485	3461511	s	rec	i02_3316	iutA	CDS
clone D	3324490	3461516	ns	rec	i02_3316	iutA	CDS
clone D	3324492	3461518	ns	rec	i02_3316	iutA	CDS
clone D	3324493	3461519	ns	rec	i02_3316	iutA	CDS
clone D	3324500	3461526	s	rec	i02_3316	iutA	CDS
clone D	3324503	3461529	s	rec	i02_3316	iutA	CDS
clone D	3324509	3461535	s	rec	i02_3316	iutA	CDS
clone D	3324515	3461541	ns	rec	i02_3316	iutA	CDS
clone D	3324516	3461542	ns	rec	i02_3316	iutA	CDS
clone D	3324517	3461543	ns	rec	i02_3316	iutA	CDS
clone D	3324536	3461562	s	rec	i02_3316	iutA	CDS
clone D	3324548	3461574	s	rec	i02_3316	iutA	CDS
clone D	3324560	3461586	s	rec	i02_3316	iutA	CDS
clone D	3324581	3461607	s	rec	i02_3316	iutA	CDS
clone D	3324584	3461610	s	rec	i02_3316	iutA	CDS
clone D	3324626	3461652	ns	rec	i02_3316	iutA	CDS
clone D	3324627	3461653	ns	rec	i02_3316	iutA	CDS
clone D	3324636	3461662	ns	rec	i02_3316	iutA	CDS
clone D	3324637	3461663	ns	rec	i02_3316	iutA	CDS
clone D	3324641	3461667	s	rec	i02_3316	iutA	CDS
clone D	3324663	3461689	ns	rec	i02_3316	iutA	CDS
clone D	3324664	3461690	ns	rec	i02_3316	iutA	CDS
clone D	3324665	3461691	ns	rec	i02_3316	iutA	CDS
clone D	3324666	3461692	ns	rec	i02_3316	iutA	CDS
clone D	3324679	3461705	ns	rec	i02_3316	iutA	CDS
clone D	3324680	3461706	s	rec	i02_3316	iutA	CDS
clone D	3324685	3461711	ns	rec	i02_3316	iutA	CDS
clone D	3324689	3461715	s	rec	i02_3316	iutA	CDS
clone D	3324692	3461718	s	rec	i02_3316	iutA	CDS
clone D	3324695	3461721	s	rec	i02_3316	iutA	CDS
clone D	3324707	3461733	s	rec	i02_3316	iutA	CDS
clone D	3324710	3461736	s	rec	i02_3316	iutA	CDS
clone D	3324716	3461742	s	rec	i02_3316	iutA	CDS
clone D	3324719	3461745	ns	rec	i02_3316	iutA	CDS
clone D	3324721	3461747	ns	rec	i02_3316	iutA	CDS
clone D	3324722	3461748	s	rec	i02_3316	iutA	CDS
clone D	3324725	3461751	s	rec	i02_3316	iutA	CDS
clone D	3324743	3461769	s	rec	i02_3316	iutA	CDS
clone D	3324776	3461802	s	rec	i02_3316	iutA	CDS
clone D	3324794	3461820	s	rec	i02_3316	iutA	CDS
clone D	3324803	3461829	s	rec	i02_3316	iutA	CDS
clone D	3324806	3461832	s	rec	i02_3316	iutA	CDS
clone D	3324827	3461853	s	rec	i02_3316	iutA	CDS
clone D	3324851	3461877	s	rec	i02_3316	iutA	CDS
clone D	3324857	3461883	s	rec	i02_3316	iutA	CDS
clone D	3324863	3461889	s	rec	i02_3316	iutA	CDS
clone D	3324871	3461897	s	rec	i02_3316	iutA	CDS
clone D	3324872	3461898	ns	rec	i02_3316	iutA	CDS
clone D	3324873	3461899	ns	rec	i02_3316	iutA	CDS
clone D	3324878	3461904	s	rec	i02_3316	iutA	CDS

Lineage	Clone D site	CFT 073 site	mutation type[a]	recomb	Gene	Gene name	Type
clone D	3324887	3461913	s	rec	i02_3316	iutA	CDS
clone D	3324890	3461916	s	rec	i02_3316	iutA	CDS
clone D	3324895	3461921	ns	rec	i02_3316	iutA	CDS
clone D	3324908	3461934	s	rec	i02_3316	iutA	CDS
clone D	3324935	3461961	s	rec	i02_3316	iutA	CDS
clone D	3324938	3461964	s	rec	i02_3316	iutA	CDS
clone D	3324947	3461973	s	rec	i02_3316	iutA	CDS
clone D	3324953	3461979	s	rec	i02_3316	iutA	CDS
clone D	3324962	3461988	s	rec	i02_3316	iutA	CDS
clone D	3324965	3461991	s	rec	i02_3316	iutA	CDS
clone D	3324968	3461994	s	rec	i02_3316	iutA	CDS
clone D	3324971	3461997	s	rec	i02_3316	iutA	CDS
clone D	3324989	3462015	s	rec	i02_3316	iutA	CDS
clone D	3324993	3462019	ns	rec	i02_3316	iutA	CDS
clone D	3324995	3462021	s	rec	i02_3316	iutA	CDS
clone D	3325007	3462033	s	rec	i02_3316	iutA	CDS
clone D	3325015	3462041	ns	rec	i02_3316	iutA	CDS
clone D	3325017	3462043	ns	rec	i02_3316	iutA	CDS
clone D	3325027	3462053	ns	rec	i02_3316	iutA	CDS
clone D	3325032	3462058	ns	rec	i02_3316	iutA	CDS
clone D	3325046	3462072	ns	rec	i02_3316	iutA	CDS
clone D	3325048	3462074	ns	rec	i02_3316	iutA	CDS
clone D	3325050	3462076	ns	rec	i02_3316	iutA	CDS
clone D	3325055	3462081	s	rec	i02_3316	iutA	CDS
clone D	3325061	3462087	s	rec	i02_3316	iutA	CDS
clone D	3325083	3462109	ns	rec	i02_3316	iutA	CDS
clone D	3325085	3462111	ns	rec	i02_3316	iutA	CDS
clone D	3325087	3462113	ns	rec	i02_3316	iutA	CDS
clone D	3325094	3462120	ns	rec	i02_3316	iutA	CDS
clone D	3325096	3462122	ns	rec	i02_3316	iutA	CDS
clone D	3325099	3462125	ns	rec	i02_3316	iutA	CDS
clone D	3325103	3462129	s	rec	i02_3316	iutA	CDS
clone D	3325113	3462139	ns	rec	i02_3316	iutA	CDS
clone D	3325114	3462140	ns	rec	i02_3316	iutA	CDS
clone D	3325115	3462141	s	rec	i02_3316	iutA	CDS
clone D	3325136	3462162	s	rec	i02_3316	iutA	CDS
clone D	3325151	3462177	s	rec	i02_3316	iutA	CDS
clone D	3325181	3462207	ns	rec	i02_3316	iutA	CDS
clone D	3325183	3462209	ns	rec	i02_3316	iutA	CDS
clone D	3325205	3462231	s	rec	i02_3316	iutA	CDS
clone D	3325220	3462246	s	rec	i02_3316	iutA	CDS
clone D	3325256	3462282	ns	rec	i02_3316	iutA	CDS
CFT073	3325295	3462321	ns	rec	i02_3316	iutA	CDS
clone D	3325296	3462322	ns	rec	i02_3316	iutA	CDS
clone D	3325300	3462326	ns	rec	i02_3316	iutA	CDS
clone D	3325337	3462363	s	rec	i02_3316	iutA	CDS
clone D	3325352	3462378	s	rec	i02_3316	iutA	CDS
clone D	3325365	3462391	ns	rec	i02_3316	iutA	CDS
clone D	3325373	3462399	s	rec	i02_3316	iutA	CDS
clone D	3325403	3462429	s	rec	i02_3316	iutA	CDS
clone D	3325442	3462468	s	rec	i02_3316	iutA	CDS

Lineage	Clone D site	CFT 073 site	mutation type[a]	recomb	Gene	Gene name	Type
CFT073	3325450	3462476	ns	rec	i02_3316	iutA	CDS
clone D	3325454	3462480	s	rec	i02_3316	iutA	CDS
clone D	3325458	3462484	ns	rec	i02_3316	iutA	CDS
clone D	3325460	3462486	s	rec	i02_3316	iutA	CDS
clone D	3325508	3462534	s	rec	i02_3316	iutA	CDS
clone D	3325514	3462540	s	rec	i02_3316	iutA	CDS
clone D	3325526	3462552	ns	rec	i02_3316	iutA	CDS
clone D	3325536	3462562	ns	rec	i02_3316	iutA	CDS
clone D	3325541	3462567	s	rec	i02_3316	iutA	CDS
clone D	3325548	3462574	ns	rec	i02_3316	iutA	CDS
clone D	3325553	3462579	ns	rec	i02_3316	iutA	CDS
clone D	3325562	3462588	s	rec	i02_3316	iutA	CDS
clone D	3325565	3462591	s	rec	i02_3316	iutA	CDS
clone D	3325594	3462620	ns	rec	i02_3316	iutA	CDS
?	3325601	3462627	s	rec	i02_3316	iutA	CDS
clone D	3325607	3462633	s	rec	i02_3316	iutA	CDS
clone D	3325622	3462648	s	rec	i02_3316	iutA	CDS
clone D	3325625	3462651	s	rec	i02_3316	iutA	CDS
clone D	3325630	3462656	s	rec	i02_3316	iutA	CDS
clone D	3325631	3462657	s	rec	i02_3316	iutA	CDS
clone D	3325667	3462693	s	rec	i02_3316	iutA	CDS
clone D	3325690	3462716	ns	rec	i02_3316	iutA	CDS
clone D	3325696	3462722	ns	rec	i02_3316	iutA	CDS
clone D	3325726	3462752	s	rec	i02_3316	iutA	CDS
clone D	3325799	3462825	ns	rec	i02_3316	iutA	CDS
clone D	3325820	3462846	s	rec	i02_3316	iutA	CDS
clone D	3325832	3462858	s	rec	i02_3316	iutA	CDS
clone D	3325854	3462880	ns	rec	i02_3316	iutA	CDS
clone D	3325868	3462894	s	rec	i02_3316	iutA	CDS
clone D	3325930	3462956	s	rec	i02_3316	iutA	CDS
clone D	3325931	3462957	s	rec	i02_3316	iutA	CDS
clone D	3325934	3462960	s	rec	i02_3316	iutA	CDS
clone D	3325943	3462969	s	rec	i02_3316	iutA	CDS
clone D	3325949	3462975	s	rec	i02_3316	iutA	CDS
clone D	3325960	3462986	ns	rec	i02_3316	iutA	CDS
clone D	3325967	3462993	s	rec	i02_3316	iutA	CDS
clone D	3326006	3463032	ns	rec	i02_3316	iutA	CDS
clone D	3326008	3463034	ns	rec	i02_3316	iutA	CDS
clone D	3326032	3463058	ns	rec	i02_3316	iutA	CDS
clone D	3326048	3463074	s	rec	i02_3316	iutA	CDS
clone D	3326054	3463080	s	rec	i02_3316	iutA	CDS
clone D	3326062	3463088	ns	rec	i02_3316	iutA	CDS
clone D	3326066	3463092	s	rec	i02_3316	iutA	CDS
clone D	3326069	3463095	s	rec	i02_3316	iutA	CDS
clone D	3326089	3463115	ns	rec	i02_3316	iutA	CDS
clone D	3326090	3463116	s	rec	i02_3316	iutA	CDS
clone D	3326108	3463134	s	rec	i02_3316	iutA	CDS
clone D	3326115	3463141	ns	rec	i02_3316	iutA	CDS
clone D	3326126	3463152	s	rec	i02_3316	iutA	CDS
clone D	3326135	3463161	s	rec	i02_3316	iutA	CDS
clone D	3326141	3463167	s	rec	i02_3316	iutA	CDS

Lineage	Clone D site	CFT 073 site	mutation type[a]	recomb	Gene	Gene name	Type
clone D	3326143	3463169	s	rec	i02_3316	iutA	CDS
clone D	3326162	3463188	s	rec	i02_3316	iutA	CDS
clone D	3326171	3463197	s	rec	i02_3316	iutA	CDS
clone D	3326189	3463215	s	rec	i02_3316	iutA	CDS
clone D	3326201	3463227	s	rec	i02_3316	iutA	CDS
clone D	3326240	3463266	s	rec	i02_3316	iutA	CDS
clone D	3326243	3463269	s	rec	i02_3316	iutA	CDS
clone D	3326256	3463282	ns	rec	i02_3316	iutA	CDS
clone D	3326267	3463293	ns	rec	i02_3316	iutA	CDS
clone D	3326269	3463295	ns	rec	i02_3316	iutA	CDS
clone D	3326272	3463298	ns	rec	i02_3316	iutA	CDS
clone D	3326274	3463300	ns	rec	i02_3316	iutA	CDS
clone D	3326275	3463301	ns	rec	i02_3316	iutA	CDS
clone D	3326276	3463302	ns	rec	i02_3316	iutA	CDS
clone D	3326278	3463304	ns	rec	i02_3316	iutA	CDS
clone D	3326286	3463312	ns	rec	i02_3316	iutA	CDS
clone D	3326291	3463317	s	rec	i02_3316	iutA	CDS
clone D	3326294	3463320	s	rec	i02_3316	iutA	CDS
clone D	3326300	3463326	s	rec	i02_3316	iutA	CDS
clone D	3326306	3463332	ns	rec	i02_3316	iutA	CDS
clone D	3326308	3463334	ns	rec	i02_3316	iutA	CDS
clone D	3326311	3463337	ns	rec	i02_3316	iutA	CDS
clone D	3326314	3463340	ns	rec	i02_3316	iutA	CDS
clone D	3326316	3463342	ns	rec	i02_3316	iutA	CDS
clone D	3326317	3463343	ns	rec	i02_3316	iutA	CDS
clone D	3326327	3463353	s	rec	i02_3316	iutA	CDS
clone D	3326330	3463356	ns	rec	i02_3316	iutA	CDS
clone D	3326331	3463357	ns	rec	i02_3316	iutA	CDS
clone D	3326332	3463358	ns	rec	i02_3316	iutA	CDS
clone D	3326333	3463359	s	rec	i02_3316	iutA	CDS
clone D	3326341	3463367	ns	rec	i02_3316	iutA	CDS
clone D	3326342	3463368	ns	rec	i02_3316	iutA	CDS
clone D	3326343	3463369	ns	rec	i02_3316	iutA	CDS
clone D	3326346	3463372	ns	rec	i02_3316	iutA	CDS
clone D	3326359	3463385	ns	rec	i02_3316	iutA	CDS
clone D	3326360	3463386	ns	rec	i02_3316	iutA	CDS
clone D	3326367	3463393	ns	rec	i02_3316	iutA	CDS
clone D	3326376	3463402	ns	rec	i02_3316	iutA	CDS
clone D	3326377	3463403	ns	rec	i02_3316	iutA	CDS
clone D	3326381	3463407	s	rec	i02_3316	iutA	CDS
clone D	3326382	3463408	s	rec	i02_3316	iutA	CDS
CFT073	3326408	3463411	nc	rec	intergenic		
clone D	3326412	3463415	nc	rec	intergenic		
clone D	3326416	3463419	nc	rec	intergenic		
CFT073	3326426	3463429	nc	rec	intergenic		
clone D	3326444	3463447	nc	rec	intergenic		
clone D	3326448	3463452	nc	rec	intergenic		
clone D	3326449	3463453	nc	rec	intergenic		
clone D	3326451	3463455	ns	rec	i02_3317	iucD	CDS
clone D	3326452	3463456	ns	rec	i02_3317	iucD	CDS
clone D	3326454	3463458	ns	rec	i02_3317	iucD	CDS

Lineage	Clone D site	CFT 073 site	mutation type[a]	recomb	Gene	Gene name	Type
clone D	3326477	3463481	s	rec	i02_3317	iucD	CDS
clone D	3326510	3463514	s	rec	i02_3317	iucD	CDS
clone D	3326515	3463519	ns	rec	i02_3317	iucD	CDS
clone D	3326546	3463550	s	rec	i02_3317	iucD	CDS
clone D	3326582	3463586	s	rec	i02_3317	iucD	CDS
clone D	3326591	3463595	s	rec	i02_3317	iucD	CDS
clone D	3326604	3463608	ns	rec	i02_3317	iucD	CDS
clone D	3326606	3463610	s	rec	i02_3317	iucD	CDS
clone D	3326615	3463619	s	rec	i02_3317	iucD	CDS
clone D	3326618	3463622	s	rec	i02_3317	iucD	CDS
clone D	3326692	3463696	s	rec	i02_3317	iucD	CDS
clone D	3326723	3463727	s	rec	i02_3317	iucD	CDS
clone D	3326774	3463778	s	rec	i02_3317	iucD	CDS
CFT073	3326781	3463785	ns	rec	i02_3317	iucD	CDS
clone D	3326795	3463799	s	rec	i02_3317	iucD	CDS
clone D	3326803	3463807	s	rec	i02_3317	iucD	CDS
clone D	3326804	3463808	s	rec	i02_3317	iucD	CDS
clone D	3326816	3463820	s	rec	i02_3317	iucD	CDS
clone D	3326995	3463999	ns	rec	i02_3317	iucD	CDS
clone D	3327083	3464087	s	rec	i02_3317	iucD	CDS
CFT073	3327173	3464177	s	rec	i02_3317	iucD	CDS
clone D	3327191	3464195	s	rec	i02_3317	iucD	CDS
clone D	3327244	3464248	ns	rec	i02_3317	iucD	CDS
clone D	3327318	3464322	ns	rec	i02_3317	iucD	CDS
clone D	3327320	3464324	s	rec	i02_3317	iucD	CDS
clone D	3327326	3464330	s	rec	i02_3317	iucD	CDS
clone D	3327329	3464333	s	rec	i02_3317	iucD	CDS
clone D	3327346	3464350	ns	rec	i02_3317	iucD	CDS
clone D	3327353	3464357	s	rec	i02_3317	iucD	CDS
clone D	3327365	3464369	s	rec	i02_3317	iucD	CDS
clone D	3327422	3464426	s	rec	i02_3317	iucD	CDS
clone D	3327512	3464516	s	rec	i02_3317	iucD	CDS
clone D	3327536	3464540	s	rec	i02_3317	iucD	CDS
CFT073	3327563	3464567	s	rec	i02_3317	iucD	CDS
clone D	3327623	3464627	s	rec	i02_3317	iucD	CDS
clone D	3327640	3464644	ns	rec	i02_3317	iucD	CDS
clone D	3327665	3464669	s	rec	i02_3317	iucD	CDS
CFT073	3327674	3464678	s	rec	i02_3317	iucD	CDS
clone D	3327698	3464702	s	rec	i02_3317	iucD	CDS
clone D	3327771	3464775	ns	rec	i02_3318	iucC	CDS
clone D	3327796	3464800	s	rec	i02_3318	iucC	CDS
CFT073	3327809	3464813	ns	rec	i02_3318	iucC	CDS
clone D	3327811	3464815	s	rec	i02_3318	iucC	CDS
clone D	3327814	3464818	s	rec	i02_3318	iucC	CDS
clone D	3327868	3464872	s	rec	i02_3318	iucC	CDS
clone D	3327910	3464914	ns	rec	i02_3318	iucC	CDS
clone D	3328057	3465061	s	rec	i02_3318	iucC	CDS
clone D	3328066	3465070	s	rec	i02_3318	iucC	CDS
clone D	3328075	3465079	s	rec	i02_3318	iucC	CDS
clone D	3328105	3465109	s	rec	i02_3318	iucC	CDS
clone D	3328109	3465113	ns	rec	i02_3318	iucC	CDS

Lineage	Clone D site	CFT 073 site	mutation type[a]	recomb	Gene	Gene name	Type
clone D	3328123	3465127	s	rec	i02_3318	iucC	CDS
clone D	3328165	3465169	s	rec	i02_3318	iucC	CDS
clone D	3328186	3465190	s	rec	i02_3318	iucC	CDS
clone D	3328201	3465205	s	rec	i02_3318	iucC	CDS
clone D	3328246	3465250	s	rec	i02_3318	iucC	CDS
clone D	3328255	3465259	s	rec	i02_3318	iucC	CDS
clone D	3328269	3465273	s	rec	i02_3318	iucC	CDS
clone D	3328297	3465301	s	rec	i02_3318	iucC	CDS
clone D	3328309	3465313	s	rec	i02_3318	iucC	CDS
clone D	3328483	3465487	s	rec	i02_3318	iucC	CDS
clone D	3328510	3465514	s	rec	i02_3318	iucC	CDS
clone D	3328531	3465535	s	rec	i02_3318	iucC	CDS
clone D	3328561	3465565	s	rec	i02_3318	iucC	CDS
clone D	3328594	3465598	s	rec	i02_3318	iucC	CDS
clone D	3328663	3465667	s	rec	i02_3318	iucC	CDS
clone D	3328672	3465676	s	rec	i02_3318	iucC	CDS
clone D	3328777	3465781	s	rec	i02_3318	iucC	CDS
clone D	3328837	3465841	s	rec	i02_3318	iucC	CDS
CFT073	3328924	3465928	s	rec	i02_3318	iucC	CDS
CFT073	3328986	3465990	ns	rec	i02_3318	iucC	CDS
CFT073	3329323	3466327	s	rec	i02_3318	iucC	CDS
clone D	3329590	3466594	ns	rec	i02_3319	iucB	CDS
CFT073	3329793	3466797	s	rec	i02_3319	iucB	CDS
CFT073	3330006	3467010	s	rec	i02_3319	iucB	CDS
CFT073	3330033	3467037	s	rec	i02_3319	iucB	CDS
CFT073	3330144	3467148	s	rec	i02_3319	iucB	CDS
CFT073	3330206	3467210	ns	rec	i02_3319	iucB	CDS
clone D	3330246	3467250	s	rec	i02_3319	iucB	CDS
CFT073	3331062	3468066	s	rec	i02_3320	iucA	CDS
clone D	3331144	3468148	ns	rec	i02_3320	iucA	CDS
CFT073	3331725	3468729	s	rec	i02_3320	iucA	CDS
CFT073	3331755	3468759	s	rec	i02_3320	iucA	CDS
CFT073	3331760	3468764	ns	rec	i02_3320	iucA	CDS
CFT073	3331798	3468802	ns	rec	i02_3320	iucA	CDS
CFT073	3331800	3468804	s	rec	i02_3320	iucA	CDS
CFT073	3331863	3468867	s	rec	i02_3320	iucA	CDS
CFT073	3332024	3469028	ns	rec	i02_3320	iucA	CDS
clone D	3332160	3469164	nc	rec	intergenic		
CFT073	3332205	3469209	nc	rec	intergenic		
CFT073	3332272	3469276	nc	rec	intergenic		
CFT073	3332282	3469286	s	rec	i02_3321	shiF	CDS
CFT073	3332357	3469361	s	rec	i02_3321	shiF	CDS
clone D	3332474	3469478	ns	rec	i02_3321	shiF	CDS
CFT073	3332699	3469703	s	rec	i02_3322	/	CDS
CFT073	3332899	3469903	ns	rec	i02_3321	shiF	CDS
clone D	3333533	3470537	nc	rec	intergenic		
CFT073	3334960	3470630	s	rec	i02_3325	/	CDS
CFT073	3336081	3471750	s	rec	i02_3326	/	CDS
CFT073	3336087	3471756	ns	rec	i02_3326	/	CDS
CFT073	3336088	3471757	ns	rec	i02_3326	/	CDS
CFT073	3336104	3471772	ns	rec	i02_3326	/	CDS

Lineage	Clone D site	CFT 073 site	mutation type[a]	recomb	Gene	Gene name	Type
CFT073	3336174	3471837	s	rec	i02_3326	/	CDS
CFT073	3336256	3471916	ns	rec	i02_3326	/	CDS
CFT073	3336258	3471918	s	rec	i02_3326	/	CDS
CFT073	3336287	3471947	s	rec	i02_3326	/	CDS
clone D	3336875	3472534	ns	rec	i02_3328	/	CDS
CFT073	3336945	3472604	s	rec	i02_3328	/	CDS
CFT073	3337166	3472825	s	rec	i02_3328	/	CDS
CFT073	3337243	3472903	ns	rec	i02_3328	/	CDS
CFT073	3337245	3472905	ns	rec	i02_3328	/	CDS
clone D	3337247	3472908	ns	rec	i02_3328	/	CDS
CFT073	3338056	3473718	s	rec	i02_3329	/	CDS
CFT073	3338618	3474280	ns	rec	i02_3329	/	CDS
clone D	3338773	3474435	s	rec	i02_3329	/	CDS
CFT073	3339684	3475346	ns	rec	i02_3331	/	CDS
CFT073	3340438	3476100	s	rec	i02_3331	/	CDS
CFT073	3343615	3479277	s	rec	i02_3334	/	CDS
clone D	3344306	3479969	nc	rec	intergenic		
?	3344309	3479974	nc	rec	intergenic		
CFT073	3344315	3479980	nc	rec	intergenic		
CFT073	3344334	3479999	nc	rec	intergenic		
CFT073	3344336	3480001	nc	rec	intergenic		
clone D	3344352	3480019	nc	rec	intergenic		
CFT073	3344358	3480025	nc	rec	intergenic		
CFT073	3344369	3480037	nc	rec	intergenic		
CFT073	3344375	3480043	nc	rec	intergenic		
CFT073	3344384	3480053	nc	rec	intergenic		
CFT073	3344389	3480058	nc	rec	intergenic		
CFT073	3344401	3480070	nc	rec	intergenic		
clone D	3346076	3481745	s	rec	i02_3337	/	CDS
CFT073	3346180	3481849	ns	rec	i02_3337	/	CDS
clone D	3347981	3483650	ns	rec	i02_3339	/	CDS
CFT073	3348195	3483863	ns	rec	i02_3340	/	CDS
?	3348207	3483875	s	rec	i02_3340	/	CDS
clone D	3348420	3484088	s	rec	i02_3340	/	CDS
?	3348426	3484094	s	rec	i02_3340	/	CDS
CFT073	3348434	3484102	ns	rec	i02_3340	/	CDS
CFT073	3348435	3484103	s	rec	i02_3340	/	CDS
?	3348482	3484150	ns	rec	i02_3340	/	CDS
clone D	3348483	3484151	s	rec	i02_3340	/	CDS
clone D	3348492	3484160	s	rec	i02_3340	/	CDS
clone D	3348495	3484163	s	rec	i02_3340	/	CDS
clone D	3348498	3484166	s	rec	i02_3340	/	CDS
?	3348567	3484235	ns	rec	i02_3340	/	CDS
?	3348569	3484237	ns	rec	i02_3340	/	CDS
CFT073	3348577	3484244	nc	rec	intergenic		
clone D	3348578	3484245	nc	rec	intergenic		
clone D	3348579	3484246	nc	rec	intergenic		
clone D	3348593	3484260	nc	rec	intergenic		
clone D	3348704	3484371	nc	rec	intergenic		
clone D	3348706	3484373	nc	rec	intergenic		
CFT073	3349168	3484835	ns	rec	i02_3341	/	CDS

Lineage	Clone D site	CFT 073 site	mutation type[a]	recomb	Gene	Gene name	Type
clone D	3349227	3484894	ns	rec	i02_3341	/	CDS
clone D	3349507	3485174	ns	rec	i02_3342	/	CDS
clone D	3349575	3485242	s	rec	i02_3342	/	CDS
clone D	3349703	3485370	nc	rec	intergenic		
CFT073	3349720	3485387	nc	rec	intergenic		
clone D	3349775	3485442	nc	rec	intergenic		
CFT073	3349818	3485484	nc	rec	intergenic		
CFT073	3350001	3485667	nc	rec	intergenic		
CFT073	3350158	3485824	ns	rec	i02_3343	/	CDS
CFT073	3350162	3485828	ns	rec	i02_3343	/	CDS
clone D	3350250	3485916	s	rec	i02_3343	/	CDS
clone D	3350282	3485948	nc	rec	intergenic		
clone D	3350288	3485954	nc	rec	intergenic		
clone D	3350326	3485992	nc	rec	intergenic		
clone D	3350438	3486104	nc	rec	intergenic		
clone D	3350453	3486119	nc	rec	intergenic		
?	3350750	3486411	nc	rec	intergenic		
CFT073	3350803	3486478	ns	rec	i02_3344	/	CDS
clone D	3350826	3486501	ns	rec	i02_3344	/	CDS
?	3350834	3486516	ns	rec	i02_3344	/	CDS
CFT073	3350835	3486517	ns	rec	i02_3344	/	CDS
CFT073	3350879	3486561	ns	rec	i02_3344	/	CDS
clone D	3350953	3486629	nc	rec	intergenic		
clone D	3351038	3486714	nc	rec	intergenic		
clone D	3351058	3486734	nc	rec	intergenic		
CFT073	3351122	3486798	ns	rec	i02_3345	/	CDS
CFT073	3351129	3486805	ns	rec	i02_3345	/	CDS
CFT073	3351130	3486806	ns	rec	i02_3345	/	CDS
clone D	3351170	3486846	ns	rec	i02_3345	/	CDS
clone D	3351184	3486860	s	rec	i02_3345	/	CDS
CFT073	3351190	3486866	s	rec	i02_3345	/	CDS
clone D	3351205	3486881	s	rec	i02_3345	/	CDS
CFT073	3351217	3486893	s	rec	i02_3345	/	CDS
CFT073	3351225	3486901	ns	rec	i02_3345	/	CDS
CFT073	3351232	3486908	s	rec	i02_3345	/	CDS
clone D	3351241	3486917	s	rec	i02_3345	/	CDS
CFT073	3351253	3486929	s	rec	i02_3345	/	CDS
clone D	3351262	3486938	s	rec	i02_3345	/	CDS
CFT073	3351265	3486941	s	rec	i02_3345	/	CDS
CFT073	3351299	3486975	s	rec	i02_3345	/	CDS
clone D	3351307	3486983	s	rec	i02_3345	/	CDS
clone D	3351314	3486990	ns	rec	i02_3345	/	CDS
CFT073	3352578	3488254	s	rec	i02_3347	/	CDS
clone D	3352725	3488401	s	rec	i02_3347	/	CDS
clone D	3353049	3488725	s	rec	i02_3347	/	CDS
clone D	3353055	3488731	s	rec	i02_3347	/	CDS
clone D	3353073	3488749	ns	rec	i02_3347	/	CDS
clone D	3353075	3488751	ns	rec	i02_3347	/	CDS
clone D	3353090	3488766	ns	rec	i02_3347	/	CDS
clone D	3353091	3488767	s	rec	i02_3347	/	CDS
clone D	3353100	3488776	s	rec	i02_3347	/	CDS

Lineage	Clone D site	CFT 073 site	mutation type[a]	recomb	Gene	Gene name	Type
clone D	3353103	3488779	s	rec	i02_3347	/	CDS
clone D	3353418	3489094	s	rec	i02_3347	/	CDS
clone D	3353746	3489422	nc	rec	intergenic		
CFT073	3353788	3489464	nc	rec	intergenic		
clone D	3353789	3489465	nc	rec	intergenic		
clone D	3353799	3489475	ns	rec	i02_3348	/	CDS
clone D	3353940	3489616	ns	rec	i02_3348	/	CDS
clone D	3354454	3490130	nc	rec	intergenic		
CFT073	3354823	3490499	nc	rec	intergenic		
clone D	3355836	3491512	nc	rec	intergenic		
clone D	3355840	3491516	nc	rec	intergenic		
clone D	3355899	3491575	nc	rec	intergenic		
clone D	3355920	3491596	nc	rec	intergenic		
clone D	3356251	3491927	s	rec	i02_3351	/	CDS
CFT073	3356276	3491952	ns	rec	i02_3351	/	CDS
clone D	3356291	3491967	ns	rec	i02_3351	/	CDS
clone D	3356315	3491991	ns	rec	i02_3351	/	CDS
clone D	3356378	3492054	ns	rec	i02_3351	/	CDS
CFT073	3357516	3493192	ns	rec	i02_3351	/	CDS
?	3357593	3493269	ns	rec	i02_3351	/	CDS
CFT073	3357598	3493274	s	rec	i02_3351	/	CDS
CFT073	3357772	3493448	s	rec	i02_3351	/	CDS
CFT073	3357808	3493484	s	rec	i02_3351	/	CDS
CFT073	3357811	3493487	s	rec	i02_3351	/	CDS
CFT073	3357812	3493488	ns	rec	i02_3351	/	CDS
CFT073	3357814	3493490	ns	rec	i02_3351	/	CDS
CFT073	3357819	3493495	ns	rec	i02_3351	/	CDS
CFT073	3357853	3493529	s	rec	i02_3351	/	CDS
CFT073	3357905	3493581	ns	rec	i02_3351	/	CDS
CFT073	3357907	3493583	ns	rec	i02_3351	/	CDS
CFT073	3357918	3493594	ns	rec	i02_3351	/	CDS
CFT073	3357919	3493595	ns	rec	i02_3351	/	CDS
CFT073	3357922	3493598	s	rec	i02_3351	/	CDS
CFT073	3357925	3493601	s	rec	i02_3351	/	CDS
CFT073	3357929	3493605	ns	rec	i02_3351	/	CDS
CFT073	3357931	3493607	ns	rec	i02_3351	/	CDS
CFT073	3357938	3493614	ns	rec	i02_3351	/	CDS
CFT073	3357940	3493616	ns	rec	i02_3351	/	CDS
CFT073	3358000	3493676	s	rec	i02_3351	/	CDS
CFT073	3358033	3493709	s	rec	i02_3351	/	CDS
CFT073	3358063	3493739	s	rec	i02_3351	/	CDS
CFT073	3358086	3493762	ns	rec	i02_3351	/	CDS
CFT073	3358087	3493763	ns	rec	i02_3351	/	CDS
CFT073	3358088	3493764	ns	rec	i02_3351	/	CDS
CFT073	3358104	3493780	ns	rec	i02_3351	/	CDS
CFT073	3358126	3493802	s	rec	i02_3351	/	CDS
CFT073	3358129	3493805	s	rec	i02_3351	/	CDS
CFT073	3358162	3493838	s	rec	i02_3351	/	CDS
CFT073	3358177	3493853	s	rec	i02_3351	/	CDS
CFT073	3358179	3493855	ns	rec	i02_3351	/	CDS
clone D	3358181	3493857	s	rec	i02_3351	/	CDS

Lineage	Clone D site	CFT 073 site	mutation type[a]	recomb	Gene	Gene name	Type
CFT073	3358205	3493881	ns	rec	i02_3351	/	CDS
CFT073	3358264	3493940	s	rec	i02_3351	/	CDS
clone D	3358330	3494006	s	rec	i02_3351	/	CDS
clone D	3358425	3494101	ns	rec	i02_3351	/	CDS
clone D	3358432	3494108	s	rec	i02_3351	/	CDS
CFT073	3358471	3494147	s	rec	i02_3351	/	CDS
CFT073	3358489	3494165	s	rec	i02_3351	/	CDS
CFT073	3358498	3494174	ns	rec	i02_3351	/	CDS
CFT073	3358513	3494189	s	rec	i02_3351	/	CDS
CFT073	3358514	3494190	ns	rec	i02_3351	/	CDS
CFT073	3358519	3494195	s	rec	i02_3351	/	CDS
CFT073	3358520	3494196	ns	rec	i02_3351	/	CDS
CFT073	3358525	3494201	s	rec	i02_3351	/	CDS
CFT073	3358526	3494202	ns	rec	i02_3351	/	CDS
CFT073	3358528	3494204	ns	rec	i02_3351	/	CDS
CFT073	3358624	3494300	s	rec	i02_3351	/	CDS
CFT073	3358625	3494301	ns	rec	i02_3351	/	CDS
CFT073	3358630	3494306	s	rec	i02_3351	/	CDS
clone D	3358634	3494310	ns	rec	i02_3351	/	CDS
clone D	3358635	3494311	ns	rec	i02_3351	/	CDS
CFT073	3358642	3494318	s	rec	i02_3351	/	CDS
CFT073	3358666	3494342	s	rec	i02_3351	/	CDS
CFT073	3358690	3494366	s	rec	i02_3351	/	CDS
CFT073	3358707	3494383	ns	rec	i02_3351	/	CDS
CFT073	3358726	3494402	s	rec	i02_3351	/	CDS
clone D	3358743	3494419	ns	rec	i02_3351	/	CDS
clone D	3358768	3494444	s	rec	i02_3351	/	CDS
clone D	3358803	3494479	ns	rec	i02_3351	/	CDS
CFT073	3358804	3494480	ns	rec	i02_3351	/	CDS
clone D	3358813	3494489	s	rec	i02_3351	/	CDS
clone D	3358825	3494501	s	rec	i02_3351	/	CDS
CFT073	3358831	3494507	s	rec	i02_3351	/	CDS
clone D	3358843	3494519	s	rec	i02_3351	/	CDS
clone D	3358844	3494520	ns	rec	i02_3351	/	CDS
clone D	3358849	3494525	s	rec	i02_3351	/	CDS
CFT073	3358959	3494635	ns	rec	i02_3351	/	CDS
CFT073	3358961	3494637	ns	rec	i02_3351	/	CDS
CFT073	3358969	3494645	s	rec	i02_3351	/	CDS
clone D	3358976	3494652	ns	rec	i02_3351	/	CDS
clone D	3358985	3494661	ns	rec	i02_3351	/	CDS
clone D	3358991	3494667	ns	rec	i02_3351	/	CDS
clone D	3359002	3494678	s	rec	i02_3351	/	CDS
CFT073	3359026	3494702	s	rec	i02_3351	/	CDS
clone D	3359053	3494729	s	rec	i02_3351	/	CDS
CFT073	3359056	3494732	s	rec	i02_3351	/	CDS
CFT073	3359060	3494736	ns	rec	i02_3351	/	CDS
CFT073	3359062	3494738	ns	rec	i02_3351	/	CDS
CFT073	3359063	3494739	ns	rec	i02_3351	/	CDS
CFT073	3359068	3494744	s	rec	i02_3351	/	CDS
CFT073	3359071	3494747	s	rec	i02_3351	/	CDS
CFT073	3359072	3494748	ns	rec	i02_3351	/	CDS

Lineage	Clone D site	CFT 073 site	mutation type[a]	recomb	Gene	Gene name	Type
CFT073	3359075	3494751	ns	rec	i02_3351	/	CDS
CFT073	3359077	3494753	ns	rec	i02_3351	/	CDS
CFT073	3359080	3494756	s	rec	i02_3351	/	CDS
CFT073	3359086	3494762	s	rec	i02_3351	/	CDS
CFT073	3359087	3494763	ns	rec	i02_3351	/	CDS
CFT073	3359088	3494764	ns	rec	i02_3351	/	CDS
CFT073	3359089	3494765	ns	rec	i02_3351	/	CDS
CFT073	3359101	3494777	s	rec	i02_3351	/	CDS
CFT073	3359104	3494780	s	rec	i02_3351	/	CDS
clone D	3359107	3494783	s	rec	i02_3351	/	CDS
clone D	3359109	3494785	ns	rec	i02_3351	/	CDS
CFT073	3359119	3494795	s	rec	i02_3351	/	CDS
clone D	3359200	3494876	s	rec	i02_3351	/	CDS
clone D	3359211	3494887	ns	rec	i02_3351	/	CDS
?	3359224	3494900	s	rec	i02_3351	/	CDS
CFT073	3359242	3494918	s	rec	i02_3351	/	CDS
clone D	3359277	3494953	nc	rec	intergenic		
clone D	3359288	3494964	nc	rec	intergenic		
clone D	3359298	3494974	nc	rec	intergenic		
?	3359324	3495000	nc	rec	intergenic		
CFT073	3359506	3502073	nc	rec	intergenic		
clone D	3359631	3502198	nc	rec	intergenic		
CFT073	3360045	3502612	nc	rec	intergenic		
CFT073	3360247	3502814	nc	rec	intergenic		
clone D	3361096	3503663	ns	rec	i02_3353	/	CDS
CFT073	3361932	3504499	ns	rec	i02_3354	/	CDS
CFT073	3362618	3505185	ns	rec	i02_3355	/	CDS
CFT073	3362901	3505468	ns	rec	i02_3356	/	CDS
CFT073	3363111	3505678	ns	rec	i02_3356	/	CDS
clone D	3363486	3506053	s	rec	i02_3357	/	CDS
CFT073	3363551	3506118	ns	rec	i02_3357	/	CDS
clone D	3363561	3506128	s	rec	i02_3357	/	CDS
CFT073	3363566	3506133	ns	rec	i02_3357	/	CDS
clone D	3363568	3506135	ns	rec	i02_3357	/	CDS
CFT073	3363569	3506136	ns	rec	i02_3357	/	CDS
clone D	3363577	3506144	ns	rec	i02_3357	/	CDS
CFT073	3363845	3506412	ns	rec	i02_3358	/	CDS
CFT073	3363861	3506428	ns	rec	i02_3358	/	CDS
CFT073	3363863	3506430	ns	rec	i02_3358	/	CDS
CFT073	3363866	3506433	ns	rec	i02_3358	/	CDS
CFT073	3363870	3506437	ns	rec	i02_3358	/	CDS
CFT073	3363877	3506444	ns	rec	i02_3358	/	CDS
CFT073	3363887	3506454	ns	rec	i02_3358	/	CDS
CFT073	3363888	3506455	ns	rec	i02_3358	/	CDS
CFT073	3363893	3506460	ns	rec	i02_3358	/	CDS
CFT073	3363896	3506463	s	rec	i02_3358	/	CDS
CFT073	3363902	3506469	ns	rec	i02_3358	/	CDS
CFT073	3363920	3506487	ns	rec	i02_3358	/	CDS
CFT073	3363923	3506490	ns	rec	i02_3358	/	CDS
CFT073	3364022	3506589	s	rec	i02_3358	/	CDS
CFT073	3364025	3506592	s	rec	i02_3358	/	CDS

Lineage	Clone D site	CFT 073 site	mutation type[a]	recomb	Gene	Gene name	Type
CFT073	3364345	3506912	nc	rec	i02_3359	/	pseudogene
CFT073	3364353	3506920	nc	rec	i02_3359	/	pseudogene
CFT073	3364356	3506923	nc	rec	i02_3359	/	pseudogene
CFT073	3364362	3506929	nc	rec	i02_3359	/	pseudogene
CFT073	3364365	3506932	nc	rec	i02_3359	/	pseudogene
clone D	3364369	3506936	nc	rec	i02_3359	/	pseudogene
CFT073	3364425	3506992	nc	rec	i02_3359	/	pseudogene
CFT073	3364438	3507005	nc	rec	i02_3359	/	pseudogene
CFT073	3364449	3507016	nc	rec	i02_3359	/	pseudogene
clone D	3364457	3507024	nc	rec	i02_3359	/	pseudogene
CFT073	3364467	3507034	nc	rec	i02_3359	/	pseudogene
CFT073	3364473	3507040	nc	rec	i02_3359	/	pseudogene
clone D	3364490	3507057	nc	rec	i02_3359	/	pseudogene
clone D	3364545	3507112	nc	rec	i02_3359	/	pseudogene
clone D	3364560	3507127	nc	rec	i02_3359	/	pseudogene
CFT073	3364572	3507139	nc	rec	i02_3359	/	pseudogene
clone D	3364578	3507145	nc	rec	i02_3359	/	pseudogene
clone D	3364584	3507151	nc	rec	i02_3359	/	pseudogene
CFT073	3364587	3507154	nc	rec	i02_3359	/	pseudogene
clone D	3364595	3507162	nc	rec	i02_3359	/	pseudogene
CFT073	3364620	3507187	nc	rec	i02_3359	/	pseudogene
CFT073	3364621	3507188	nc	rec	i02_3359	/	pseudogene
CFT073	3364624	3507191	nc	rec	i02_3359	/	pseudogene
clone D	3364631	3507198	nc	rec	i02_3359	/	pseudogene
CFT073	3364633	3507200	nc	rec	i02_3359	/	pseudogene
clone D	3364637	3507204	nc	rec	i02_3359	/	pseudogene
clone D	3364643	3507209	nc	rec	i02_3359	/	pseudogene
clone D	3364648	3507214	nc	rec	i02_3359	/	pseudogene
CFT073	3364655	3507221	nc	rec	i02_3359	/	pseudogene
clone D	3364664	3507230	nc	rec	i02_3359	/	pseudogene
clone D	3364675	3507241	nc	rec	i02_3359	/	pseudogene
CFT073	3364680	3507246	nc	rec	i02_3359	/	pseudogene
CFT073	3364812	3507379	ns	rec	i02_3360	/	CDS
clone D	3364944	3507511	ns	rec	i02_3360	/	CDS
clone D	3365279	3507846	ns	rec	i02_3361	/	CDS
?	3365280	3507847	ns	rec	i02_3361	/	CDS
clone D	3365554	3508121	ns	rec	i02_3361	/	CDS
clone D	3365639	3508206	nc	rec	intergenic		
CFT073	3365781	3508348	nc	rec	intergenic		
CFT073	3365801	3508368	nc	rec	intergenic		
CFT073	3365825	3508392	nc	rec	intergenic		
clone D	3365848	3508415	nc	rec	intergenic		
clone D	3365850	3508417	nc	rec	intergenic		
clone D	3365864	3508431	nc	rec	intergenic		
clone D	3365870	3508437	nc	rec	intergenic		
clone D	3365871	3508438	nc	rec	intergenic		
clone D	3365872	3508439	nc	rec	intergenic		
CFT073	3365874	3508441	nc	rec	intergenic		
clone D	3365883	3508450	nc	rec	intergenic		
clone D	3365885	3508452	nc	rec	intergenic		
clone D	3365890	3508457	nc	rec	intergenic		

Lineage	Clone D site	CFT 073 site	mutation type[a]	recomb	Gene	Gene name	Type
clone D	3365896	3508463	s	rec	i02_3362	/	CDS
clone D	3365906	3508473	ns	rec	i02_3362	/	CDS
CFT073	3365907	3508474	ns	rec	i02_3362	/	CDS
clone D	3365914	3508481	s	rec	i02_3362	/	CDS
clone D	3365921	3508488	ns	rec	i02_3362	/	CDS
clone D	3365932	3508499	s	rec	i02_3362	/	CDS
CFT073	3365941	3508508	ns	rec	i02_3362	/	CDS
CFT073	3365964	3508531	ns	rec	i02_3362	/	CDS
CFT073	3365986	3508553	s	rec	i02_3362	/	CDS
clone D	3365990	3508557	ns	rec	i02_3362	/	CDS
clone D	3365992	3508559	ns	rec	i02_3362	/	CDS
clone D	3366028	3508595	s	rec	i02_3362	/	CDS
CFT073	3366040	3508607	s	rec	i02_3362	/	CDS
CFT073	3366046	3508613	s	rec	i02_3362	/	CDS
CFT073	3366052	3508619	s	rec	i02_3362	/	CDS
CFT073	3366068	3508635	ns	rec	i02_3362	/	CDS
CFT073	3366072	3508639	ns	rec	i02_3362	/	CDS
clone D	3366082	3508649	s	rec	i02_3362	/	CDS
CFT073	3366127	3508694	s	rec	i02_3362	/	CDS
CFT073	3366133	3508700	s	rec	i02_3362	/	CDS
clone D	3366157	3508724	s	rec	i02_3362	/	CDS
clone D	3366160	3508727	s	rec	i02_3362	/	CDS
clone D	3366163	3508730	s	rec	i02_3362	/	CDS
CFT073	3366166	3508733	s	rec	i02_3362	/	CDS
clone D	3366192	3508759	ns	rec	i02_3362	/	CDS
CFT073	3366197	3508764	ns	rec	i02_3362	/	CDS
CFT073	3366205	3508772	s	rec	i02_3362	/	CDS
CFT073	3366208	3508775	s	rec	i02_3362	/	CDS
clone D	3366228	3508795	ns	rec	i02_3362	/	CDS
CFT073	3366244	3508811	s	rec	i02_3362	/	CDS
CFT073	3366262	3508829	s	rec	i02_3362	/	CDS
clone D	3366280	3508847	s	rec	i02_3362	/	CDS
clone D	3366286	3508853	s	rec	i02_3362	/	CDS
clone D	3366289	3508856	s	rec	i02_3362	/	CDS
clone D	3366295	3508862	s	rec	i02_3362	/	CDS
clone D	3366298	3508865	s	rec	i02_3362	/	CDS
CFT073	3366302	3508869	ns	rec	i02_3362	/	CDS
CFT073	3366319	3508886	s	rec	i02_3362	/	CDS
?	3366328	3508895	ns	rec	i02_3362	/	CDS
CFT073	3366340	3508907	s	rec	i02_3362	/	CDS
clone D	3366343	3508910	s	rec	i02_3362	/	CDS
clone D	3366353	3508920	ns	rec	i02_3362	/	CDS
CFT073	3366361	3508928	s	rec	i02_3362	/	CDS
CFT073	3366364	3508931	s	rec	i02_3362	/	CDS
CFT073	3366379	3508946	s	rec	i02_3362	/	CDS
CFT073	3366388	3508955	s	rec	i02_3362	/	CDS
clone D	3366390	3508957	ns	rec	i02_3362	/	CDS
CFT073	3366391	3508958	ns	rec	i02_3362	/	CDS
CFT073	3366406	3508973	s	rec	i02_3362	/	CDS
clone D	3366408	3508975	ns	rec	i02_3362	/	CDS
CFT073	3366415	3508982	s	rec	i02_3362	/	CDS

Lineage	Clone D site	CFT 073 site	mutation type[a]	recomb	Gene	Gene name	Type
CFT073	3366418	3508985	s	rec	i02_3362	/	CDS
CFT073	3366419	3508986	ns	rec	i02_3362	/	CDS
CFT073	3366445	3509012	s	rec	i02_3362	/	CDS
CFT073	3366449	3509016	ns	rec	i02_3362	/	CDS
CFT073	3366454	3509021	ns	rec	i02_3362	/	CDS
CFT073	3366469	3509036	s	rec	i02_3362	/	CDS
CFT073	3366476	3509043	ns	rec	i02_3362	/	CDS
CFT073	3366478	3509045	ns	rec	i02_3362	/	CDS
CFT073	3366481	3509048	s	rec	i02_3362	/	CDS
clone D	3366490	3509057	s	rec	i02_3362	/	CDS
CFT073	3366493	3509060	s	rec	i02_3362	/	CDS
clone D	3366508	3509075	s	rec	i02_3362	/	CDS
CFT073	3366522	3509089	ns	rec	i02_3362	/	CDS
CFT073	3366532	3509099	s	rec	i02_3362	/	CDS
CFT073	3366540	3509107	ns	rec	i02_3362	/	CDS
CFT073	3366550	3509117	s	rec	i02_3362	/	CDS
CFT073	3366555	3509122	ns	rec	i02_3362	/	CDS
CFT073	3366556	3509123	ns	rec	i02_3362	/	CDS
CFT073	3366562	3509129	s	rec	i02_3362	/	CDS
CFT073	3366570	3509137	ns	rec	i02_3362	/	CDS
CFT073	3366579	3509146	ns	rec	i02_3362	/	CDS
CFT073	3366580	3509147	ns	rec	i02_3362	/	CDS
CFT073	3366592	3509159	s	rec	i02_3362	/	CDS
clone D	3366597	3509164	ns	rec	i02_3362	/	CDS
CFT073	3366599	3509166	ns	rec	i02_3362	/	CDS
CFT073	3366610	3509177	s	rec	i02_3362	/	CDS
CFT073	3366613	3509180	s	rec	i02_3362	/	CDS
CFT073	3367165	3509732	ns	rec	i02_3363	/	CDS
CFT073	3368458	3511026	nc	rec	intergenic		
CFT073	3368624	3511192	ns	rec	i02_3366	/	CDS
clone D	3369004	3511572	s	rec	i02_3367	/	CDS
clone D	3369013	3511581	ns	rec	i02_3367	/	CDS
CFT073	3369020	3511588	ns	rec	i02_3367	/	CDS
clone D	3369073	3511641	nc	rec	intergenic		
CFT073	3369091	3511659	nc	rec	intergenic		
CFT073	3369092	3511660	nc	rec	intergenic		
clone D	3369093	3511661	nc	rec	intergenic		
clone D	3369094	3511662	nc	rec	intergenic		
clone D	3369096	3511664	nc	rec	intergenic		
CFT073	3369211	3511779	nc	rec	intergenic		
clone D	3369240	3511808	nc	rec	intergenic		
CFT073	3369479	3512047	nc	rec	intergenic		
CFT073	3369745	3512313	ns	rec	i02_3368	/	CDS
CFT073	3369916	3512484	s	rec	i02_3368	/	CDS
CFT073	3369977	3512545	s	rec	i02_3368	/	CDS
CFT073	3370318	3512886	s	rec	i02_3368	/	CDS
CFT073	3370335	3512903	ns	rec	i02_3368	/	CDS
CFT073	3370351	3512919	s	rec	i02_3368	/	CDS
CFT073	3370522	3513090	nc	rec	intergenic		
CFT073	3370656	3513224	s	rec	i02_3369	kpsE	CDS
CFT073	3370665	3513233	s	rec	i02_3369	kpsE	CDS

Lineage	Clone D site	CFT 073 site	mutation type[a]	recomb	Gene	Gene name	Type
CFT073	3371250	3513818	s	rec	i02_3369	kpsE	CDS
CFT073	3371671	3514239	s	rec	i02_3369	kpsE	CDS
CFT073	3371673	3514241	s	rec	i02_3369	kpsE	CDS
clone D	3371811	3514379	ns	rec	i02_3370	kpsD	CDS
CFT073	3371812	3514380	ns	rec	i02_3370	kpsD	CDS
clone D	3372174	3514742	s	rec	i02_3370	kpsD	CDS
CFT073	3372413	3514981	s	rec	i02_3370	kpsD	CDS
clone D	3372449	3515017	s	rec	i02_3370	kpsD	CDS
clone D	3372491	3515059	s	rec	i02_3370	kpsD	CDS
CFT073	3372695	3515263	s	rec	i02_3370	kpsD	CDS
CFT073	3372698	3515266	s	rec	i02_3370	kpsD	CDS
CFT073	3372734	3515302	s	rec	i02_3370	kpsD	CDS
clone D	3372789	3515357	s	rec	i02_3370	kpsD	CDS
CFT073	3372815	3515383	s	rec	i02_3370	kpsD	CDS
clone D	3372938	3515506	s	rec	i02_3370	kpsD	CDS
clone D	3373148	3515716	s	rec	i02_3370	kpsD	CDS
clone D	3373181	3515749	s	rec	i02_3370	kpsD	CDS
clone D	3373184	3515752	s	rec	i02_3370	kpsD	CDS
clone D	3373214	3515782	s	rec	i02_3370	kpsD	CDS
clone D	3373274	3515842	s	rec	i02_3370	kpsD	CDS
clone D	3373277	3515845	s	rec	i02_3370	kpsD	CDS
clone D	3373286	3515854	s	rec	i02_3370	kpsD	CDS
clone D	3373295	3515863	s	rec	i02_3370	kpsD	CDS
clone D	3373307	3515875	s	rec	i02_3370	kpsD	CDS
clone D	3373310	3515878	s	rec	i02_3370	kpsD	CDS
CFT073	3373352	3515920	s	rec	i02_3370	kpsD	CDS
clone D	3373430	3515998	nc	rec	intergenic		
CFT073	3373448	3516016	s	rec	i02_3371	/	CDS
CFT073	3373460	3516028	s	rec	i02_3371	/	CDS
clone D	3373469	3516037	s	rec	i02_3371	/	CDS
clone D	3373478	3516046	s	rec	i02_3371	/	CDS
CFT073	3373561	3516129	ns	rec	i02_3371	/	CDS
clone D	3373597	3516165	ns	rec	i02_3371	/	CDS
clone D	3373608	3516176	ns	rec	i02_3371	/	CDS
clone D	3373616	3516184	s	rec	i02_3371	/	CDS
clone D	3373622	3516190	s	rec	i02_3371	/	CDS
CFT073	3373635	3516203	ns	rec	i02_3371	/	CDS
CFT073	3373713	3516281	ns	rec	i02_3371	/	CDS
CFT073	3373751	3516319	s	rec	i02_3371	/	CDS
CFT073	3373762	3516330	ns	rec	i02_3371	/	CDS
clone D	3373770	3516338	s	rec	i02_3371	/	CDS
clone D	3373787	3516355	s	rec	i02_3371	/	CDS
clone D	3373790	3516358	s	rec	i02_3371	/	CDS
clone D	3373802	3516370	s	rec	i02_3371	/	CDS
clone D	3373834	3516402	ns	rec	i02_3371	/	CDS
clone D	3373842	3516410	ns	rec	i02_3371	/	CDS
clone D	3373844	3516412	ns	rec	i02_3371	/	CDS
clone D	3373868	3516436	s	rec	i02_3371	/	CDS
clone D	3373871	3516439	s	rec	i02_3371	/	CDS
CFT073	3373898	3516466	s	rec	i02_3371	/	CDS
clone D	3373904	3516472	s	rec	i02_3371	/	CDS

Lineage	Clone D site	CFT 073 site	mutation type[a]	recomb	Gene	Gene name	Type
clone D	3373907	3516475	s	rec	i02_3371	/	CDS
CFT073	3373916	3516484	s	rec	i02_3371	/	CDS
clone D	3373958	3516526	s	rec	i02_3371	/	CDS
CFT073	3374021	3516589	s	rec	i02_3371	/	CDS
clone D	3374030	3516598	s	rec	i02_3371	/	CDS
clone D	3374049	3516617	s	rec	i02_3371	/	CDS
CFT073	3374054	3516622	s	rec	i02_3371	/	CDS
CFT073	3374075	3516643	s	rec	i02_3371	/	CDS
CFT073	3374081	3516649	s	rec	i02_3371	/	CDS
CFT073	3374088	3516656	ns	rec	i02_3371	/	CDS
CFT073	3374149	3516717	ns	rec	i02_3371	/	CDS
CFT073	3374239	3516807	s	rec	i02_3372	kpsC	CDS
clone D	3374272	3516840	s	rec	i02_3372	kpsC	CDS
CFT073	3374276	3516844	ns	rec	i02_3372	kpsC	CDS
CFT073	3374278	3516846	ns	rec	i02_3372	kpsC	CDS
CFT073	3374281	3516849	s	rec	i02_3372	kpsC	CDS
clone D	3374444	3517012	ns	rec	i02_3372	kpsC	CDS
CFT073	3374473	3517041	s	rec	i02_3372	kpsC	CDS
CFT073	3374476	3517044	s	rec	i02_3372	kpsC	CDS
clone D	3374494	3517062	s	rec	i02_3372	kpsC	CDS
CFT073	3374510	3517078	ns	rec	i02_3372	kpsC	CDS
CFT073	3374512	3517080	ns	rec	i02_3372	kpsC	CDS
?	3374515	3517083	s	rec	i02_3372	kpsC	CDS
?	3374516	3517084	s	rec	i02_3372	kpsC	CDS
?	3374519	3517087	ns	rec	i02_3372	kpsC	CDS
clone D	3374520	3517088	ns	rec	i02_3372	kpsC	CDS
clone D	3374521	3517089	ns	rec	i02_3372	kpsC	CDS
clone D	3374522	3517090	ns	rec	i02_3372	kpsC	CDS
clone D	3374523	3517091	ns	rec	i02_3372	kpsC	CDS
clone D	3374524	3517092	ns	rec	i02_3372	kpsC	CDS
CFT073	3374584	3517152	s	rec	i02_3372	kpsC	CDS
CFT073	3374590	3517158	s	rec	i02_3372	kpsC	CDS
clone D	3374615	3517183	ns	rec	i02_3372	kpsC	CDS
CFT073	3374653	3517221	s	rec	i02_3372	kpsC	CDS
CFT073	3374659	3517227	s	rec	i02_3372	kpsC	CDS
CFT073	3374710	3517278	s	rec	i02_3372	kpsC	CDS
CFT073	3374718	3517286	ns	rec	i02_3372	kpsC	CDS
clone D	3374794	3517362	s	rec	i02_3372	kpsC	CDS
clone D	3374796	3517364	ns	rec	i02_3372	kpsC	CDS
clone D	3374803	3517371	s	rec	i02_3372	kpsC	CDS
clone D	3374824	3517392	s	rec	i02_3372	kpsC	CDS
clone D	3374854	3517422	s	rec	i02_3372	kpsC	CDS
CFT073	3374887	3517455	s	rec	i02_3372	kpsC	CDS
clone D	3374893	3517461	s	rec	i02_3372	kpsC	CDS
clone D	3374926	3517494	s	rec	i02_3372	kpsC	CDS
clone D	3374929	3517497	s	rec	i02_3372	kpsC	CDS
clone D	3374935	3517503	s	rec	i02_3372	kpsC	CDS
clone D	3375031	3517599	s	rec	i02_3372	kpsC	CDS
CFT073	3375076	3517644	s	rec	i02_3372	kpsC	CDS
CFT073	3375081	3517649	ns	rec	i02_3372	kpsC	CDS
CFT073	3375082	3517650	ns	rec	i02_3372	kpsC	CDS

Lineage	Clone D site	CFT 073 site	mutation type[a]	recomb	Gene	Gene name	Type
CFT073	3375088	3517656	s	rec	i02_3372	kpsC	CDS
CFT073	3375090	3517658	ns	rec	i02_3372	kpsC	CDS
CFT073	3375091	3517659	ns	rec	i02_3372	kpsC	CDS
CFT073	3375093	3517661	ns	rec	i02_3372	kpsC	CDS
clone D	3375098	3517666	ns	rec	i02_3372	kpsC	CDS
CFT073	3375103	3517671	s	rec	i02_3372	kpsC	CDS
CFT073	3375112	3517680	s	rec	i02_3372	kpsC	CDS
CFT073	3375133	3517701	s	rec	i02_3372	kpsC	CDS
CFT073	3375136	3517704	s	rec	i02_3372	kpsC	CDS
CFT073	3375137	3517705	ns	rec	i02_3372	kpsC	CDS
CFT073	3375138	3517706	ns	rec	i02_3372	kpsC	CDS
CFT073	3375139	3517707	ns	rec	i02_3372	kpsC	CDS
CFT073	3375144	3517712	ns	rec	i02_3372	kpsC	CDS
CFT073	3375160	3517728	s	rec	i02_3372	kpsC	CDS
CFT073	3375196	3517764	s	rec	i02_3372	kpsC	CDS
CFT073	3375199	3517767	s	rec	i02_3372	kpsC	CDS
CFT073	3375218	3517786	ns	rec	i02_3372	kpsC	CDS
CFT073	3375219	3517787	ns	rec	i02_3372	kpsC	CDS
CFT073	3375220	3517788	ns	rec	i02_3372	kpsC	CDS
CFT073	3375223	3517791	s	rec	i02_3372	kpsC	CDS
CFT073	3375233	3517801	s	rec	i02_3372	kpsC	CDS
CFT073	3375235	3517803	s	rec	i02_3372	kpsC	CDS
CFT073	3375243	3517811	ns	rec	i02_3372	kpsC	CDS
CFT073	3375250	3517818	s	rec	i02_3372	kpsC	CDS
CFT073	3375253	3517821	s	rec	i02_3372	kpsC	CDS
CFT073	3375267	3517835	ns	rec	i02_3372	kpsC	CDS
CFT073	3375268	3517836	ns	rec	i02_3372	kpsC	CDS
CFT073	3375271	3517839	s	rec	i02_3372	kpsC	CDS
CFT073	3375309	3517877	ns	rec	i02_3372	kpsC	CDS
CFT073	3375322	3517890	s	rec	i02_3372	kpsC	CDS
CFT073	3375324	3517892	ns	rec	i02_3372	kpsC	CDS
clone D	3375364	3517932	s	rec	i02_3372	kpsC	CDS
?	3375412	3517980	s	rec	i02_3372	kpsC	CDS
CFT073	3375466	3518034	s	rec	i02_3372	kpsC	CDS
clone D	3375490	3518058	s	rec	i02_3372	kpsC	CDS
clone D	3375708	3518276	ns	rec	i02_3372	kpsC	CDS
clone D	3375841	3518409	s	rec	i02_3372	kpsC	CDS
clone D	3375970	3518538	s	rec	i02_3372	kpsC	CDS
clone D	3375979	3518547	s	rec	i02_3372	kpsC	CDS
clone D	3375987	3518555	ns	rec	i02_3372	kpsC	CDS
clone D	3375988	3518556	ns	rec	i02_3372	kpsC	CDS
clone D	3375990	3518558	ns	rec	i02_3372	kpsC	CDS
clone D	3376006	3518574	s	rec	i02_3372	kpsC	CDS
clone D	3376033	3518601	s	rec	i02_3372	kpsC	CDS
clone D	3376039	3518607	s	rec	i02_3372	kpsC	CDS
clone D	3376045	3518613	s	rec	i02_3372	kpsC	CDS
clone D	3376056	3518624	ns	rec	i02_3372	kpsC	CDS
clone D	3376063	3518631	s	rec	i02_3372	kpsC	CDS
clone D	3376078	3518646	s	rec	i02_3372	kpsC	CDS
clone D	3376081	3518649	s	rec	i02_3372	kpsC	CDS
clone D	3376084	3518652	s	rec	i02_3372	kpsC	CDS

Lineage	Clone D site	CFT 073 site	mutation type[a]	recomb	Gene	Gene name	Type
clone D	3376096	3518664	s	rec	i02_3372	kpsC	CDS
clone D	3376111	3518679	s	rec	i02_3372	kpsC	CDS
clone D	3376144	3518712	s	rec	i02_3372	kpsC	CDS
clone D	3376147	3518715	s	rec	i02_3372	kpsC	CDS
clone D	3376211	3518779	nc	rec	intergenic		
clone D	3376228	3518796	nc	rec	intergenic		
clone D	3376244	3518812	s	rec	i02_3373	kpsS	CDS
clone D	3376247	3518815	s	rec	i02_3373	kpsS	CDS
clone D	3376257	3518825	ns	rec	i02_3373	kpsS	CDS
clone D	3376268	3518836	s	rec	i02_3373	kpsS	CDS
clone D	3376295	3518863	s	rec	i02_3373	kpsS	CDS
clone D	3376298	3518866	s	rec	i02_3373	kpsS	CDS
clone D	3376307	3518875	s	rec	i02_3373	kpsS	CDS
clone D	3376315	3518883	ns	rec	i02_3373	kpsS	CDS
clone D	3376319	3518887	s	rec	i02_3373	kpsS	CDS
CFT073	3376322	3518890	s	rec	i02_3373	kpsS	CDS
clone D	3376328	3518896	s	rec	i02_3373	kpsS	CDS
clone D	3376332	3518900	s	rec	i02_3373	kpsS	CDS
clone D	3376346	3518914	s	rec	i02_3373	kpsS	CDS
CFT073	3376367	3518935	s	rec	i02_3373	kpsS	CDS
CFT073	3376376	3518944	s	rec	i02_3373	kpsS	CDS
clone D	3376412	3518980	s	rec	i02_3373	kpsS	CDS
clone D	3376424	3518992	s	rec	i02_3373	kpsS	CDS
clone D	3376427	3518995	s	rec	i02_3373	kpsS	CDS
CFT073	3376456	3519024	ns	rec	i02_3373	kpsS	CDS
clone D	3376481	3519049	s	rec	i02_3373	kpsS	CDS
clone D	3376484	3519052	s	rec	i02_3373	kpsS	CDS
clone D	3376499	3519067	s	rec	i02_3373	kpsS	CDS
CFT073	3376517	3519085	s	rec	i02_3373	kpsS	CDS
clone D	3376559	3519127	s	rec	i02_3373	kpsS	CDS
clone D	3376571	3519139	s	rec	i02_3373	kpsS	CDS
CFT073	3376598	3519166	s	rec	i02_3373	kpsS	CDS
CFT073	3376631	3519199	s	rec	i02_3373	kpsS	CDS
CFT073	3376667	3519235	s	rec	i02_3373	kpsS	CDS
clone D	3376680	3519248	ns	rec	i02_3373	kpsS	CDS
clone D	3376721	3519289	s	rec	i02_3373	kpsS	CDS
clone D	3376724	3519292	s	rec	i02_3373	kpsS	CDS
clone D	3376733	3519301	s	rec	i02_3373	kpsS	CDS
clone D	3376775	3519343	s	rec	i02_3373	kpsS	CDS
clone D	3376838	3519406	s	rec	i02_3373	kpsS	CDS
CFT073	3376874	3519442	s	rec	i02_3373	kpsS	CDS
clone D	3376883	3519451	s	rec	i02_3373	kpsS	CDS
clone D	3376898	3519466	s	rec	i02_3373	kpsS	CDS
clone D	3376901	3519469	s	rec	i02_3373	kpsS	CDS
clone D	3376907	3519475	s	rec	i02_3373	kpsS	CDS
clone D	3376925	3519493	s	rec	i02_3373	kpsS	CDS
CFT073	3376967	3519535	s	rec	i02_3373	kpsS	CDS
clone D	3376969	3519537	ns	rec	i02_3373	kpsS	CDS
CFT073	3377018	3519586	s	rec	i02_3373	kpsS	CDS
clone D	3377102	3519670	s	rec	i02_3373	kpsS	CDS
clone D	3377105	3519673	s	rec	i02_3373	kpsS	CDS

Lineage	Clone D site	CFT 073 site	mutation type[a]	recomb	Gene	Gene name	Type
clone D	3377128	3519696	ns	rec	i02_3373	kpsS	CDS
clone D	3377136	3519704	ns	rec	i02_3373	kpsS	CDS
clone D	3377139	3519707	ns	rec	i02_3373	kpsS	CDS
clone D	3377192	3519760	s	rec	i02_3373	kpsS	CDS
clone D	3377279	3519847	s	rec	i02_3373	kpsS	CDS
clone D	3377321	3519889	s	rec	i02_3373	kpsS	CDS
clone D	3377333	3519901	s	rec	i02_3373	kpsS	CDS
CFT073	3377336	3519904	s	rec	i02_3373	kpsS	CDS
clone D	3377360	3519928	s	rec	i02_3373	kpsS	CDS
CFT073	3377392	3519960	ns	rec	i02_3373	kpsS	CDS
CFT073	3377395	3519963	ns	rec	i02_3373	kpsS	CDS
clone D	3385786	3527989	s	rec	i02_3377	kpsT	CDS
clone D	3385798	3528001	s	rec	i02_3377	kpsT	CDS
clone D	3385831	3528034	s	rec	i02_3377	kpsT	CDS
clone D	3385834	3528037	s	rec	i02_3377	kpsT	CDS
CFT073	3385858	3528061	s	rec	i02_3377	kpsT	CDS
?	3385861	3528064	s	rec	i02_3377	kpsT	CDS
clone D	3385939	3528142	s	rec	i02_3377	kpsT	CDS
clone D	3385951	3528154	s	rec	i02_3377	kpsT	CDS
clone D	3385963	3528166	s	rec	i02_3377	kpsT	CDS
CFT073	3385972	3528175	s	rec	i02_3377	kpsT	CDS
CFT073	3385975	3528178	s	rec	i02_3377	kpsT	CDS
CFT073	3385993	3528196	s	rec	i02_3377	kpsT	CDS
CFT073	3386017	3528220	s	rec	i02_3377	kpsT	CDS
clone D	3386083	3528286	s	rec	i02_3377	kpsT	CDS
clone D	3386092	3528295	s	rec	i02_3377	kpsT	CDS
CFT073	3386095	3528298	s	rec	i02_3377	kpsT	CDS
clone D	3386116	3528319	s	rec	i02_3377	kpsT	CDS
clone D	3386125	3528328	s	rec	i02_3377	kpsT	CDS
clone D	3386140	3528343	s	rec	i02_3377	kpsT	CDS
clone D	3386143	3528346	s	rec	i02_3377	kpsT	CDS
clone D	3386218	3528421	s	rec	i02_3377	kpsT	CDS
CFT073	3386241	3528444	ns	rec	i02_3377	kpsT	CDS
clone D	3386263	3528466	s	rec	i02_3377	kpsT	CDS
clone D	3386269	3528472	s	rec	i02_3377	kpsT	CDS
clone D	3386281	3528484	s	rec	i02_3377	kpsT	CDS
CFT073	3386346	3528549	s	rec	i02_3378	kpsM	CDS
CFT073	3386361	3528564	s	rec	i02_3378	kpsM	CDS
CFT073	3386370	3528573	s	rec	i02_3378	kpsM	CDS
CFT073	3386373	3528576	ns	rec	i02_3378	kpsM	CDS
CFT073	3386375	3528578	ns	rec	i02_3378	kpsM	CDS
CFT073	3386378	3528581	s	rec	i02_3378	kpsM	CDS
CFT073	3386379	3528582	s	rec	i02_3378	kpsM	CDS
CFT073	3386391	3528594	s	rec	i02_3378	kpsM	CDS
CFT073	3386409	3528612	s	rec	i02_3378	kpsM	CDS
CFT073	3386418	3528621	s	rec	i02_3378	kpsM	CDS
CFT073	3386436	3528639	s	rec	i02_3378	kpsM	CDS
CFT073	3386442	3528645	s	rec	i02_3378	kpsM	CDS
CFT073	3386454	3528657	s	rec	i02_3378	kpsM	CDS
CFT073	3386468	3528671	s	rec	i02_3378	kpsM	CDS
clone D	3386502	3528705	s	rec	i02_3378	kpsM	CDS

Lineage	Clone D site	CFT 073 site	mutation type[a]	recomb	Gene	Gene name	Type
clone D	3386508	3528711	s	rec	i02_3378	kpsM	CDS
clone D	3386511	3528714	s	rec	i02_3378	kpsM	CDS
clone D	3386514	3528717	s	rec	i02_3378	kpsM	CDS
CFT073	3386544	3528747	s	rec	i02_3378	kpsM	CDS
CFT073	3386547	3528750	s	rec	i02_3378	kpsM	CDS
CFT073	3386556	3528759	s	rec	i02_3378	kpsM	CDS
CFT073	3386568	3528771	s	rec	i02_3378	kpsM	CDS
CFT073	3386583	3528786	s	rec	i02_3378	kpsM	CDS
clone D	3386621	3528824	ns	rec	i02_3378	kpsM	CDS
CFT073	3386624	3528827	ns	rec	i02_3378	kpsM	CDS
CFT073	3386625	3528828	s	rec	i02_3378	kpsM	CDS
CFT073	3386640	3528843	s	rec	i02_3378	kpsM	CDS
CFT073	3386642	3528845	s	rec	i02_3378	kpsM	CDS
CFT073	3386645	3528848	s	rec	i02_3378	kpsM	CDS
CFT073	3386655	3528858	ns	rec	i02_3378	kpsM	CDS
CFT073	3386656	3528859	ns	rec	i02_3378	kpsM	CDS
CFT073	3386657	3528860	ns	rec	i02_3378	kpsM	CDS
clone D	3386661	3528864	s	rec	i02_3378	kpsM	CDS
clone D	3386673	3528876	s	rec	i02_3378	kpsM	CDS
clone D	3386694	3528897	s	rec	i02_3378	kpsM	CDS
CFT073	3386705	3528908	ns	rec	i02_3378	kpsM	CDS
CFT073	3386709	3528912	s	rec	i02_3378	kpsM	CDS
CFT073	3386712	3528915	s	rec	i02_3378	kpsM	CDS
clone D	3386718	3528921	s	rec	i02_3378	kpsM	CDS
clone D	3386723	3528926	s	rec	i02_3378	kpsM	CDS
clone D	3386724	3528927	s	rec	i02_3378	kpsM	CDS
CFT073	3386735	3528938	ns	rec	i02_3378	kpsM	CDS
clone D	3386760	3528963	s	rec	i02_3378	kpsM	CDS
clone D	3386766	3528969	s	rec	i02_3378	kpsM	CDS
CFT073	3386796	3528999	s	rec	i02_3378	kpsM	CDS
clone D	3386810	3529013	s	rec	i02_3378	kpsM	CDS
clone D	3386832	3529035	ns	rec	i02_3378	kpsM	CDS
CFT073	3386834	3529037	ns	rec	i02_3378	kpsM	CDS
CFT073	3386844	3529047	ns	rec	i02_3378	kpsM	CDS
CFT073	3386904	3529107	s	rec	i02_3378	kpsM	CDS
clone D	3386993	3529196	s	rec	i02_3378	kpsM	CDS
CFT073	3387093	3529296	nc	rec	intergenic		
CFT073	3387107	3529310	nc	rec	intergenic		
CFT073	3387120	3529323	nc	rec	intergenic		
CFT073	3387131	3529334	nc	rec	intergenic		
CFT073	3387165	3529368	nc	rec	intergenic		
CFT073	3387169	3529372	nc	rec	intergenic		
clone D	3387189	3529392	nc	rec	intergenic		
CFT073	3387191	3529394	nc	rec	intergenic		
clone D	3387217	3529420	nc	rec	intergenic		
CFT073	3387225	3529427	nc	rec	intergenic		
clone D	3387309	3529511	nc	rec	intergenic		
CFT073	3387347	3529549	nc	rec	intergenic		
clone D	3387354	3529556	nc	rec	intergenic		
CFT073	3387355	3529557	nc	rec	intergenic		
clone D	3387358	3529560	nc	rec	intergenic		

Lineage	Clone D site	CFT 073 site	mutation type[a]	recomb	Gene	Gene name	Type
CFT073	3387383	3529701	nc	rec	intergenic		
clone D	3387385	3529703	nc	rec	intergenic		
clone D	3387416	3529734	nc	rec	intergenic		
CFT073	3387419	3529737	nc	rec	intergenic		
clone D	3387423	3529741	nc	rec	intergenic		
CFT073	3387436	3529754	nc	rec	intergenic		
CFT073	3387437	3529755	nc	rec	intergenic		
clone D	3387440	3529758	nc	rec	intergenic		
CFT073	3387446	3529764	nc	rec	intergenic		
clone D	3387451	3529769	nc	rec	intergenic		
CFT073	3387452	3529770	nc	rec	intergenic		
clone D	3387454	3529772	nc	rec	intergenic		
CFT073	3387470	3529788	nc	rec	intergenic		
clone D	3387495	3529813	nc	rec	intergenic		
clone D	3387497	3529815	nc	rec	intergenic		
clone D	3387498	3529816	nc	rec	intergenic		
clone D	3387530	3529848	nc	rec	intergenic		
CFT073	3387556	3529874	nc	rec	intergenic		
clone D	3387569	3529887	nc	rec	intergenic		
clone D	3387572	3529890	nc	rec	intergenic		
clone D	3387585	3529903	nc	rec	intergenic		
clone D	3387598	3529916	nc	rec	intergenic		
CFT073	3387611	3529929	nc	rec	intergenic		
clone D	3387612	3529930	nc	rec	intergenic		
clone D	3387614	3529932	nc	rec	intergenic		
clone D	3387617	3529935	nc	rec	intergenic		
clone D	3387625	3529943	nc	rec	intergenic		
clone D	3387629	3529947	nc	rec	intergenic		
clone D	3387630	3529948	nc	rec	intergenic		
CFT073	3387631	3529949	nc	rec	intergenic		
CFT073	3387643	3529961	nc	rec	intergenic		
CFT073	3387678	3529997	nc	rec	intergenic		
CFT073	3387824	3530143	nc	rec	intergenic		
clone D	3387830	3530149	nc	rec	intergenic		
CFT073	3387832	3530151	nc	rec	intergenic		
clone D	3387833	3530152	nc	rec	intergenic		
CFT073	3387854	3530173	nc	rec	intergenic		
CFT073	3387876	3530195	nc	rec	intergenic		
CFT073	3387945	3530264	nc	rec	intergenic		
CFT073	3387957	3530276	nc	rec	intergenic		
clone D	3388019	3530338	nc	rec	intergenic		
clone D	3388047	3530366	nc	rec	intergenic		
clone D	3388061	3530380	nc	rec	intergenic		
clone D	3388079	3530398	nc	rec	intergenic		
clone D	3388156	3530475	s	rec	i02_3379	yghD	CDS
clone D	3388172	3530491	ns	rec	i02_3379	yghD	CDS
clone D	3388180	3530499	s	rec	i02_3379	yghD	CDS
clone D	3388189	3530508	s	rec	i02_3379	yghD	CDS
CFT073	3388199	3530518	ns	rec	i02_3379	yghD	CDS
CFT073	3388201	3530520	s	rec	i02_3379	yghD	CDS
CFT073	3388221	3530540	ns	rec	i02_3379	yghD	CDS

Lineage	Clone D site	CFT 073 site	mutation type[a]	recomb	Gene	Gene name	Type
CFT073	3388234	3530553	s	rec	i02_3379	yghD	CDS
CFT073	3388237	3530556	s	rec	i02_3379	yghD	CDS
CFT073	3388249	3530568	s	rec	i02_3379	yghD	CDS
CFT073	3388252	3530571	ns	rec	i02_3379	yghD	CDS
clone D	3388261	3530580	ns	rec	i02_3379	yghD	CDS
clone D	3388267	3530586	s	rec	i02_3379	yghD	CDS
CFT073	3388324	3530643	s	rec	i02_3379	yghD	CDS
CFT073	3388330	3530649	s	rec	i02_3379	yghD	CDS
CFT073	3388342	3530661	s	rec	i02_3379	yghD	CDS
clone D	3388363	3530682	s	rec	i02_3379	yghD	CDS
clone D	3388388	3530707	ns	rec	i02_3379	yghD	CDS
CFT073	3388425	3530744	ns	rec	i02_3379	yghD	CDS
clone D	3388432	3530751	ns	rec	i02_3379	yghD	CDS
clone D	3388434	3530753	ns	rec	i02_3379	yghD	CDS
clone D	3388498	3530817	s	rec	i02_3379	yghD	CDS
clone D	3388532	3530851	ns	rec	i02_3379	yghD	CDS
clone D	3388539	3530858	ns	rec	i02_3379	yghD	CDS
clone D	3388552	3530871	ns	rec	i02_3379	yghD	CDS
clone D	3388553	3530872	ns	rec	i02_3379	yghD	CDS
clone D	3388558	3530877	s	rec	i02_3379	yghD	CDS
clone D	3388585	3530904	s	rec	i02_3379	yghD	CDS
clone D	3388603	3530922	s	rec	i02_3379	yghD	CDS
clone D	3388608	3530927	ns	rec	i02_3379	yghD	CDS
clone D	3388787	3531106	s	rec	i02_3380	gspL	CDS
CFT073	3388820	3531139	s	rec	i02_3380	gspL	CDS
clone D	3388876	3531195	ns	rec	i02_3380	gspL	CDS
CFT073	3388908	3531227	ns	rec	i02_3380	gspL	CDS
clone D	3388930	3531249	ns	rec	i02_3380	gspL	CDS
CFT073	3388975	3531294	ns	rec	i02_3380	gspL	CDS
CFT073	3389010	3531329	ns	rec	i02_3380	gspL	CDS
clone D	3389100	3531419	ns	rec	i02_3380	gspL	CDS
clone D	3389174	3531493	s	rec	i02_3380	gspL	CDS
clone D	3389189	3531508	s	rec	i02_3380	gspL	CDS
clone D	3389192	3531511	s	rec	i02_3380	gspL	CDS
clone D	3389195	3531514	s	rec	i02_3380	gspL	CDS
clone D	3389246	3531565	s	rec	i02_3380	gspL	CDS
clone D	3389249	3531568	s	rec	i02_3380	gspL	CDS
clone D	3389267	3531585	s	rec	i02_3380	gspL	CDS
clone D	3389323	3531641	ns	rec	i02_3380	gspL	CDS
clone D	3389332	3531650	ns	rec	i02_3380	gspL	CDS
clone D	3389442	3531760	ns	rec	i02_3380	gspL	CDS
clone D	3389444	3531762	s	rec	i02_3380	gspL	CDS
clone D	3389468	3531786	s	rec	i02_3380	gspL	CDS
clone D	3389510	3531828	s	rec	i02_3380	gspL	CDS
clone D	3389522	3531840	s	rec	i02_3380	gspL	CDS
clone D	3389534	3531852	s	rec	i02_3380	gspL	CDS
clone D	3389546	3531864	nc	rec	intergenic		
CFT073	3389585	3531903	nc	rec	intergenic		
clone D	3389609	3531927	nc	rec	intergenic		
clone D	3389642	3531960	nc	rec	intergenic		
clone D	3389662	3531980	nc	rec	intergenic		

Lineage	Clone D site	CFT 073 site	mutation type[a]	recomb	Gene	Gene name	Type
clone D	3389663	3531981	nc	rec	intergenic		
clone D	3389729	3532047	nc	rec	intergenic		
CFT073	3389752	3532070	nc	rec	intergenic		
CFT073	3389756	3532074	nc	rec	intergenic		
clone D	3389783	3532101	nc	rec	intergenic		
clone D	3389789	3532107	nc	rec	intergenic		
clone D	3389799	3532117	nc	rec	intergenic		
clone D	3389810	3532128	nc	rec	intergenic		
clone D	3389815	3532133	nc	rec	intergenic		
clone D	3389818	3532136	nc	rec	intergenic		
clone D	3389819	3532137	nc	rec	intergenic		
clone D	3389822	3532140	nc	rec	intergenic		
clone D	3389824	3532142	nc	rec	intergenic		
clone D	3389826	3532144	nc	rec	intergenic		
clone D	3389852	3532170	nc	rec	intergenic		
CFT073	3389856	3532174	nc	rec	intergenic		
clone D	3389929	3532247	ns	rec	i02_3381	/	CDS
clone D	3389986	3532304	ns	rec	i02_3381	/	CDS
clone D	3390043	3532361	ns	rec	i02_3381	/	CDS
clone D	3390121	3532439	ns	rec	i02_3381	/	CDS
clone D	3390178	3532496	ns	rec	i02_3381	/	CDS
clone D	3390180	3532498	ns	rec	i02_3381	/	CDS
clone D	3390181	3532499	ns	rec	i02_3381	/	CDS
clone D	3390184	3532502	ns	rec	i02_3381	/	CDS
clone D	3390207	3532525	ns	rec	i02_3381	/	CDS
clone D	3390228	3532546	ns	rec	i02_3381	/	CDS
clone D	3390250	3532568	ns	rec	i02_3381	/	CDS
CFT073	3390268	3532586	ns	rec	i02_3381	/	CDS
clone D	3405079	3534253	nc	rec	intergenic		
clone D	3405139	3534313	s	rec	i02_3395	yghK	CDS
clone D	3405142	3534316	s	rec	i02_3395	yghK	CDS
clone D	3405151	3534325	s	rec	i02_3395	yghK	CDS
CFT073	3405196	3534370	s	rec	i02_3395	yghK	CDS
clone D	3405241	3534415	s	rec	i02_3395	yghK	CDS
clone D	3405244	3534418	s	rec	i02_3395	yghK	CDS
clone D	3405274	3534448	s	rec	i02_3395	yghK	CDS
clone D	3405292	3534466	s	rec	i02_3395	yghK	CDS
clone D	3405346	3534520	s	rec	i02_3395	yghK	CDS
clone D	3405352	3534526	s	rec	i02_3395	yghK	CDS
clone D	3405355	3534529	s	rec	i02_3395	yghK	CDS
clone D	3405358	3534532	s	rec	i02_3395	yghK	CDS
clone D	3405409	3534583	s	rec	i02_3395	yghK	CDS
clone D	3405412	3534586	s	rec	i02_3395	yghK	CDS
clone D	3405433	3534607	s	rec	i02_3395	yghK	CDS
clone D	3405463	3534637	s	rec	i02_3395	yghK	CDS
CFT073	3405487	3534661	s	rec	i02_3395	yghK	CDS
CFT073	3405490	3534664	s	rec	i02_3395	yghK	CDS
CFT073	3405513	3534687	s	rec	i02_3395	yghK	CDS
clone D	3405559	3534733	s	rec	i02_3395	yghK	CDS
clone D	3405571	3534745	s	rec	i02_3395	yghK	CDS
clone D	3405576	3534750	ns	rec	i02_3395	yghK	CDS

Lineage	Clone D site	CFT 073 site	mutation type[a]	recomb	Gene	Gene name	Type
CFT073	3405625	3534799	s	rec	i02_3395	yghK	CDS
clone D	3405631	3534805	s	rec	i02_3395	yghK	CDS
clone D	3405637	3534811	s	rec	i02_3395	yghK	CDS
clone D	3405658	3534832	s	rec	i02_3395	yghK	CDS
clone D	3405670	3534844	s	rec	i02_3395	yghK	CDS
clone D	3405673	3534847	s	rec	i02_3395	yghK	CDS
clone D	3405688	3534862	s	rec	i02_3395	yghK	CDS
clone D	3405700	3534874	s	rec	i02_3395	yghK	CDS
clone D	3405807	3534981	s	rec	i02_3395	yghK	CDS
CFT073	3405912	3535086	ns	rec	i02_3395	yghK	CDS
CFT073	3406465	3535639	s	rec	i02_3395	yghK	CDS
clone D	3406495	3535669	s	rec	i02_3395	yghK	CDS
CFT073	3406631	3535805	ns	rec	i02_3395	yghK	CDS
clone D	3406768	3535942	nc	rec	intergenic		
clone D	3406950	3536124	nc	rec	intergenic		
clone D	3407250	3536424	ns	rec	i02_3396	glcB	CDS
clone D	3407346	3536520	ns	rec	i02_3396	glcB	CDS
CFT073	3407419	3536593	s	rec	i02_3396	glcB	CDS
clone D	3407524	3536698	s	rec	i02_3396	glcB	CDS
clone D	3407548	3536722	s	rec	i02_3396	glcB	CDS
clone D	3407603	3536777	ns	rec	i02_3396	glcB	CDS
clone D	3407620	3536794	s	rec	i02_3396	glcB	CDS
clone D	3407671	3536845	s	rec	i02_3396	glcB	CDS
clone D	3407674	3536848	s	rec	i02_3396	glcB	CDS
clone D	3407680	3536854	s	rec	i02_3396	glcB	CDS
clone D	3407695	3536869	s	rec	i02_3396	glcB	CDS
clone D	3407715	3536889	s	rec	i02_3396	glcB	CDS
clone D	3407770	3536944	s	rec	i02_3396	glcB	CDS
clone D	3407791	3536965	s	rec	i02_3396	glcB	CDS
clone D	3407794	3536968	s	rec	i02_3396	glcB	CDS
clone D	3407797	3536971	s	rec	i02_3396	glcB	CDS
clone D	3407890	3537064	s	rec	i02_3396	glcB	CDS
clone D	3407962	3537136	s	rec	i02_3396	glcB	CDS
clone D	3408004	3537178	s	rec	i02_3396	glcB	CDS
clone D	3408066	3537240	ns	rec	i02_3396	glcB	CDS
clone D	3408072	3537246	ns	rec	i02_3396	glcB	CDS
clone D	3408291	3537465	s	rec	i02_3396	glcB	CDS
clone D	3408319	3537493	s	rec	i02_3396	glcB	CDS
clone D	3408331	3537505	s	rec	i02_3396	glcB	CDS
clone D	3408337	3537511	ns	rec	i02_3396	glcB	CDS
clone D	3408379	3537553	s	rec	i02_3396	glcB	CDS
clone D	3408382	3537556	s	rec	i02_3396	glcB	CDS
clone D	3408466	3537640	s	rec	i02_3396	glcB	CDS
clone D	3408526	3537700	s	rec	i02_3396	glcB	CDS
CFT073	3408534	3537708	ns	rec	i02_3396	glcB	CDS
clone D	3408565	3537739	s	rec	i02_3396	glcB	CDS
clone D	3408571	3537745	s	rec	i02_3396	glcB	CDS
clone D	3408574	3537748	s	rec	i02_3396	glcB	CDS
clone D	3408586	3537760	s	rec	i02_3396	glcB	CDS
clone D	3408622	3537796	ns	rec	i02_3396	glcB	CDS
clone D	3408624	3537798	ns	rec	i02_3396	glcB	CDS

Lineage	Clone D site	CFT 073 site	mutation type[a]	recomb	Gene	Gene name	Type
clone D	3408649	3537823	s	rec	i02_3396	glcB	CDS
clone D	3408652	3537826	s	rec	i02_3396	glcB	CDS
clone D	3408655	3537829	s	rec	i02_3396	glcB	CDS
CFT073	3408745	3537919	s	rec	i02_3396	glcB	CDS
CFT073	3408909	3538083	s	rec	i02_3396	glcB	CDS
clone D	3408928	3538102	s	rec	i02_3396	glcB	CDS
clone D	3408976	3538150	s	rec	i02_3396	glcB	CDS
?	3408997	3538171	s	rec	i02_3396	glcB	CDS
clone D	3409001	3538175	ns	rec	i02_3396	glcB	CDS
clone D	3409003	3538177	ns	rec	i02_3396	glcB	CDS
clone D	3409006	3538180	s	rec	i02_3396	glcB	CDS
clone D	3409009	3538183	s	rec	i02_3396	glcB	CDS
clone D	3409012	3538186	s	rec	i02_3396	glcB	CDS
clone D	3409123	3538297	s	rec	i02_3396	glcB	CDS
clone D	3409144	3538318	s	rec	i02_3396	glcB	CDS
clone D	3409150	3538324	s	rec	i02_3396	glcB	CDS
clone D	3409176	3538350	ns	rec	i02_3396	glcB	CDS
clone D	3409181	3538355	ns	rec	i02_3396	glcB	CDS
CFT073	3409186	3538360	s	rec	i02_3396	glcB	CDS
clone D	3409192	3538366	s	rec	i02_3396	glcB	CDS
clone D	3409198	3538372	s	rec	i02_3396	glcB	CDS
clone D	3409203	3538377	s	rec	i02_3396	glcB	CDS
clone D	3409204	3538378	ns	rec	i02_3396	glcB	CDS
clone D	3409205	3538379	ns	rec	i02_3396	glcB	CDS
clone D	3409207	3538381	ns	rec	i02_3396	glcB	CDS
clone D	3409208	3538382	ns	rec	i02_3396	glcB	CDS
clone D	3409209	3538383	ns	rec	i02_3396	glcB	CDS
clone D	3409210	3538384	s	rec	i02_3396	glcB	CDS
clone D	3409213	3538387	s	rec	i02_3396	glcB	CDS
clone D	3409254	3538428	ns	rec	i02_3396	glcB	CDS
clone D	3409296	3538470	nc	rec	intergenic		
clone D	3409432	3538606	s	rec	i02_3397	glcG	CDS
clone D	3409441	3538615	s	rec	i02_3397	glcG	CDS
clone D	3409443	3538617	s	rec	i02_3397	glcG	CDS
clone D	3409495	3538669	s	rec	i02_3397	glcG	CDS
clone D	3409639	3538813	s	rec	i02_3397	glcG	CDS
clone D	3409880	3539054	s	rec	i02_3398	pir	CDS
CFT073	3410114	3539288	s	rec	i02_3398	pir	CDS
CFT073	3410124	3539298	ns	rec	i02_3398	pir	CDS
CFT073	3410192	3539366	s	rec	i02_3398	pir	CDS
clone D	3410195	3539369	s	rec	i02_3398	pir	CDS
clone D	3410204	3539378	s	rec	i02_3398	pir	CDS
clone D	3410207	3539381	s	rec	i02_3398	pir	CDS
clone D	3410216	3539390	s	rec	i02_3398	pir	CDS
CFT073	3410456	3539630	s	rec	i02_3398	pir	CDS
CFT073	3410480	3539654	s	rec	i02_3398	pir	CDS
clone D	3410675	3539849	s	rec	i02_3398	pir	CDS
clone D	3410756	3539930	ns	rec	i02_3398	pir	CDS
CFT073	3410762	3539936	s	rec	i02_3398	pir	CDS
clone D	3410825	3539999	s	rec	i02_3398	pir	CDS
clone D	3410903	3540077	s	rec	i02_3398	pir	CDS

Lineage	Clone D site	CFT 073 site	mutation type[a]	recomb	Gene	Gene name	Type
clone D	3410927	3540101	s	rec	i02_3398	pir	CDS
clone D	3410934	3540108	ns	rec	i02_3398	pir	CDS
clone D	3410956	3540130	s	rec	i02_3399	glcE	CDS
clone D	3410961	3540135	s	rec	i02_3399	glcE	CDS
clone D	3410982	3540156	s	rec	i02_3399	glcE	CDS
clone D	3411000	3540174	s	rec	i02_3399	glcE	CDS
clone D	3411042	3540216	s	rec	i02_3399	glcE	CDS
clone D	3411060	3540234	s	rec	i02_3399	glcE	CDS
clone D	3411066	3540240	s	rec	i02_3399	glcE	CDS
clone D	3411099	3540273	s	rec	i02_3399	glcE	CDS
clone D	3411111	3540285	s	rec	i02_3399	glcE	CDS
clone D	3411129	3540303	s	rec	i02_3399	glcE	CDS
clone D	3411213	3540387	s	rec	i02_3399	glcE	CDS
clone D	3411222	3540396	s	rec	i02_3399	glcE	CDS
CFT073	3411235	3540409	ns	rec	i02_3399	glcE	CDS
clone D	3411297	3540471	s	rec	i02_3399	glcE	CDS
clone D	3411312	3540486	s	rec	i02_3399	glcE	CDS
clone D	3411407	3540581	s	rec	i02_3399	glcE	CDS
clone D	3411408	3540582	s	rec	i02_3399	glcE	CDS
clone D	3411471	3540645	s	rec	i02_3399	glcE	CDS
clone D	3411479	3540653	ns	rec	i02_3399	glcE	CDS
clone D	3411486	3540660	s	rec	i02_3399	glcE	CDS
clone D	3411525	3540699	s	rec	i02_3399	glcE	CDS
clone D	3411528	3540702	s	rec	i02_3399	glcE	CDS
clone D	3411594	3540768	s	rec	i02_3399	glcE	CDS
clone D	3411699	3540873	s	rec	i02_3399	glcE	CDS
clone D	3411704	3540878	ns	rec	i02_3399	glcE	CDS
clone D	3411720	3540894	s	rec	i02_3399	glcE	CDS
CFT073	3411738	3540912	s	rec	i02_3399	glcE	CDS
CFT073	3411780	3540954	s	rec	i02_3399	glcE	CDS
clone D	3411813	3540987	s	rec	i02_3399	glcE	CDS
CFT073	3411894	3541068	s	rec	i02_3399	glcE	CDS
CFT073	3412028	3541202	s	rec	i02_3400	glcD	CDS
CFT073	3412100	3541274	s	rec	i02_3400	glcD	CDS
CFT073	3412103	3541277	s	rec	i02_3400	glcD	CDS
clone D	3412208	3541382	s	rec	i02_3400	glcD	CDS
clone D	3412274	3541448	s	rec	i02_3400	glcD	CDS
CFT073	3412367	3541541	s	rec	i02_3400	glcD	CDS
clone D	3412421	3541595	s	rec	i02_3400	glcD	CDS
CFT073	3412427	3541601	s	rec	i02_3400	glcD	CDS
clone D	3412430	3541604	s	rec	i02_3400	glcD	CDS
clone D	3412448	3541622	s	rec	i02_3400	glcD	CDS
clone D	3412466	3541640	s	rec	i02_3400	glcD	CDS
clone D	3412469	3541643	s	rec	i02_3400	glcD	CDS
clone D	3412472	3541646	s	rec	i02_3400	glcD	CDS
clone D	3412475	3541649	s	rec	i02_3400	glcD	CDS
clone D	3412478	3541652	s	rec	i02_3400	glcD	CDS
clone D	3412487	3541661	s	rec	i02_3400	glcD	CDS
clone D	3412490	3541664	s	rec	i02_3400	glcD	CDS
clone D	3412493	3541667	s	rec	i02_3400	glcD	CDS
clone D	3412499	3541673	s	rec	i02_3400	glcD	CDS

Lineage	Clone D site	CFT 073 site	mutation type[a]	recomb	Gene	Gene name	Type
clone D	3412631	3541805	s	rec	i02_3400	glcD	CDS
clone D	3412634	3541808	s	rec	i02_3400	glcD	CDS
CFT073	3412649	3541823	s	rec	i02_3400	glcD	CDS
clone D	3412670	3541844	s	rec	i02_3400	glcD	CDS
clone D	3412685	3541859	s	rec	i02_3400	glcD	CDS
clone D	3412799	3541973	s	rec	i02_3400	glcD	CDS
clone D	3412834	3542008	ns	rec	i02_3400	glcD	CDS
CFT073	3412877	3542051	s	rec	i02_3400	glcD	CDS
CFT073	3412904	3542078	s	rec	i02_3400	glcD	CDS
CFT073	3412925	3542099	s	rec	i02_3400	glcD	CDS
CFT073	3412934	3542108	s	rec	i02_3400	glcD	CDS
CFT073	3412943	3542117	s	rec	i02_3400	glcD	CDS
CFT073	3412985	3542159	s	rec	i02_3400	glcD	CDS
CFT073	3413006	3542180	s	rec	i02_3400	glcD	CDS
CFT073	3413012	3542186	s	rec	i02_3400	glcD	CDS
CFT073	3413024	3542198	s	rec	i02_3400	glcD	CDS
CFT073	3413042	3542216	s	rec	i02_3400	glcD	CDS
CFT073	3413063	3542237	s	rec	i02_3400	glcD	CDS
CFT073	3413066	3542240	s	rec	i02_3400	glcD	CDS
CFT073	3413072	3542246	s	rec	i02_3400	glcD	CDS
CFT073	3413078	3542252	s	rec	i02_3400	glcD	CDS
CFT073	3413093	3542267	s	rec	i02_3400	glcD	CDS
clone D	3413115	3542289	ns	rec	i02_3400	glcD	CDS
CFT073	3413129	3542303	s	rec	i02_3400	glcD	CDS
clone D	3413144	3542318	s	rec	i02_3400	glcD	CDS
CFT073	3413279	3542453	s	rec	i02_3400	glcD	CDS
CFT073	3413300	3542474	s	rec	i02_3400	glcD	CDS
CFT073	3413315	3542489	s	rec	i02_3400	glcD	CDS
CFT073	3413549	3542723	nc	rec	intergenic		
CFT073	3413660	3542834	nc	rec	intergenic		
CFT073	3414107	3543281	s	rec	i02_3401	glcC	CDS
clone D	3414256	3543430	ns	rec	i02_3401	glcC	CDS
CFT073	3414281	3543455	s	rec	i02_3401	glcC	CDS
CFT073	3414296	3543470	s	rec	i02_3401	glcC	CDS
CFT073	3414512	3543686	ns	rec	i02_3401	glcC	CDS
CFT073	3414514	3543688	ns	rec	i02_3401	glcC	CDS
CFT073	3414516	3543690	ns	rec	i02_3401	glcC	CDS
CFT073	3414518	3543692	ns	rec	i02_3401	glcC	CDS
CFT073	3414520	3543694	s	rec	i02_3401	glcC	CDS
CFT073	3414521	3543695	s	rec	i02_3401	glcC	CDS
CFT073	3414525	3543699	nc	rec	intergenic		
CFT073	3414526	3543700	nc	rec	intergenic		
CFT073	3414529	3543703	ns	rec	i02_3402	yghO	CDS
clone D	3414689	3543863	s	rec	i02_3402	yghO	CDS
CFT073	3414729	3543903	ns	rec	i02_3402	yghO	CDS
clone D	3414777	3543951	s	rec	i02_3402	yghO	CDS
clone D	3414852	3544026	ns	rec	i02_3402	yghO	CDS
CFT073	3414900	3544074	ns	rec	i02_3402	yghO	CDS
clone D	3414936	3544110	ns	rec	i02_3402	yghO	CDS
CFT073	3415002	3544176	ns	rec	i02_3402	yghO	CDS
CFT073	3415047	3544221	ns	rec	i02_3402	yghO	CDS

Lineage	Clone D site	CFT 073 site	mutation type[a]	recomb	Gene	Gene name	Type
clone D	3415074	3544248	ns	rec	i02_3402	yghO	CDS
clone D	3415077	3544251	ns	rec	i02_3402	yghO	CDS
clone D	3415175	3544349	s	rec	i02_3402	yghO	CDS
clone D	3415206	3544380	ns	rec	i02_3402	yghO	CDS
clone D	3415212	3544386	ns	rec	i02_3402	yghO	CDS
clone D	3415236	3544410	ns	rec	i02_3402	yghO	CDS
clone D	3415239	3544413	ns	rec	i02_3402	yghO	CDS
?	3415672	3544846	ns	rec	i02_3402	yghO	CDS
?	3415673	3544847	ns	rec	i02_3402	yghO	CDS
CFT073	3415681	3544860	nc	rec	intergenic		
clone D	3415698	3544877	nc	rec	intergenic		
CFT073	3415702	3544881	nc	rec	intergenic		
CFT073	3415731	3544910	nc	rec	intergenic		
clone D	3415745	3544924	nc	rec	intergenic		
clone D	3415747	3544926	nc	rec	intergenic		
clone D	3415759	3544938	nc	rec	intergenic		
?	3415760	3544939	nc	rec	intergenic		
CFT073	3415762	3544941	nc	rec	intergenic		
clone D	3415772	3544951	nc	rec	intergenic		
clone D	3415796	3544975	nc	rec	intergenic		
clone D	3415800	3544979	nc	rec	intergenic		
clone D	3415803	3544982	nc	rec	intergenic		
clone D	3415808	3544987	nc	rec	intergenic		
clone D	3415809	3544988	nc	rec	intergenic		
clone D	3415831	3545010	nc	rec	intergenic		
clone D	3415832	3545011	nc	rec	intergenic		
clone D	3415841	3545020	nc	rec	intergenic		
clone D	3415845	3545024	nc	rec	intergenic		
clone D	3415846	3545025	nc	rec	intergenic		
clone D	3415848	3545027	nc	rec	intergenic		
clone D	3415856	3545035	nc	rec	intergenic		
CFT073	3415858	3545037	nc	rec	intergenic		
clone D	3415859	3545038	nc	rec	intergenic		
clone D	3415869	3545048	nc	rec	intergenic		
clone D	3415880	3545059	nc	rec	intergenic		
clone D	3415905	3545084	nc	rec	intergenic		
clone D	3415911	3545090	nc	rec	intergenic		
CFT073	3415916	3545096	nc	rec	intergenic		
CFT073	3415917	3545097	nc	rec	intergenic		
clone D	3415938	3545118	nc	rec	intergenic		
clone D	3415940	3545120	nc	rec	intergenic		
clone D	3415943	3545123	nc	rec	intergenic		
CFT073	3415947	3545127	nc	rec	intergenic		
CFT073	3416015	3545195	nc	rec	intergenic		
CFT073	3416100	3545280	s	rec	i02_3403	/	CDS
clone D	3416118	3545298	s	rec	i02_3403	/	CDS
clone D	3416136	3545316	s	rec	i02_3403	/	CDS
CFT073	3416202	3545382	s	rec	i02_3403	/	CDS
CFT073	3416280	3545460	s	rec	i02_3403	/	CDS
CFT073	3416288	3545468	ns	rec	i02_3403	/	CDS
CFT073	3416292	3545472	s	rec	i02_3403	/	CDS

Lineage	Clone D site	CFT 073 site	mutation type[a]	recomb	Gene	Gene name	Type
CFT073	3416361	3545541	s	rec	i02_3403	/	CDS
CFT073	3416389	3545569	s	rec	i02_3403	/	CDS
clone D	3416397	3545577	s	rec	i02_3403	/	CDS
CFT073	3416473	3545653	ns	rec	i02_3403	/	CDS
clone D	3416478	3545658	s	rec	i02_3403	/	CDS
clone D	3416541	3545721	s	rec	i02_3403	/	CDS
clone D	3416544	3545724	s	rec	i02_3403	/	CDS
clone D	3416556	3545736	s	rec	i02_3403	/	CDS
clone D	3416613	3545793	s	rec	i02_3403	/	CDS
clone D	3416616	3545796	s	rec	i02_3403	/	CDS
clone D	3416619	3545799	s	rec	i02_3403	/	CDS
clone D	3416622	3545802	s	rec	i02_3403	/	CDS
clone D	3416625	3545805	s	rec	i02_3403	/	CDS
CFT073	3416664	3545844	s	rec	i02_3403	/	CDS
clone D	3416667	3545847	s	rec	i02_3403	/	CDS
CFT073	3416841	3546021	s	rec	i02_3403	/	CDS
CFT073	3416859	3546039	s	rec	i02_3403	/	CDS
CFT073	3416952	3546132	s	rec	i02_3403	/	CDS
CFT073	3416964	3546144	s	rec	i02_3403	/	CDS
CFT073	3417019	3546199	ns	rec	i02_3403	/	CDS
CFT073	3417089	3546269	ns	rec	i02_3403	/	CDS
CFT073	3417132	3546312	s	rec	i02_3403	/	CDS
CFT073	3417159	3546339	s	rec	i02_3403	/	CDS
CFT073	3417162	3546342	s	rec	i02_3403	/	CDS
CFT073	3417195	3546375	s	rec	i02_3403	/	CDS
CFT073	3417207	3546387	s	rec	i02_3403	/	CDS
CFT073	3417216	3546396	s	rec	i02_3403	/	CDS
CFT073	3417239	3546419	ns	rec	i02_3403	/	CDS
CFT073	3417243	3546423	ns	rec	i02_3403	/	CDS
CFT073	3417246	3546426	s	rec	i02_3403	/	CDS
CFT073	3417285	3546465	s	rec	i02_3403	/	CDS
CFT073	3417291	3546471	s	rec	i02_3403	/	CDS
CFT073	3417303	3546483	s	rec	i02_3403	/	CDS
CFT073	3417369	3546549	s	rec	i02_3403	/	CDS
CFT073	3417465	3546645	s	rec	i02_3403	/	CDS
CFT073	3417468	3546648	s	rec	i02_3403	/	CDS
CFT073	3417480	3546660	s	rec	i02_3403	/	CDS
CFT073	3417569	3546749	ns	rec	i02_3404	/	CDS
CFT073	3417600	3546780	ns	rec	i02_3404	/	CDS
CFT073	3417603	3546783	ns	rec	i02_3404	/	CDS
CFT073	3417627	3546807	ns	rec	i02_3404	/	CDS
CFT073	3417635	3546815	ns	rec	i02_3404	/	CDS
CFT073	3417672	3546852	ns	rec	i02_3404	/	CDS
CFT073	3417739	3546919	ns	rec	i02_3404	/	CDS
CFT073	3417815	3546995	s	rec	i02_3404	/	CDS
CFT073	3417893	3547073	s	rec	i02_3404	/	CDS
CFT073	3417920	3547100	s	rec	i02_3404	/	CDS
CFT073	3417926	3547106	s	rec	i02_3404	/	CDS
CFT073	3417935	3547115	s	rec	i02_3404	/	CDS
CFT073	3417955	3547135	ns	rec	i02_3404	/	CDS
CFT073	3417956	3547136	ns	rec	i02_3404	/	CDS

Lineage	Clone D site	CFT 073 site	mutation type[a]	recomb	Gene	Gene name	Type
CFT073	3418004	3547184	s	rec	i02_3404	/	CDS
CFT073	3418013	3547193	s	rec	i02_3404	/	CDS
CFT073	3418046	3547226	s	rec	i02_3404	/	CDS
CFT073	3418112	3547292	s	rec	i02_3404	/	CDS
CFT073	3418124	3547304	s	rec	i02_3404	/	CDS
CFT073	3418130	3547310	ns	rec	i02_3404	/	CDS
CFT073	3418137	3547317	ns	rec	i02_3404	/	CDS
CFT073	3418148	3547328	s	rec	i02_3404	/	CDS
CFT073	3418154	3547334	s	rec	i02_3404	/	CDS
CFT073	3418158	3547338	ns	rec	i02_3404	/	CDS
CFT073	3418160	3547340	ns	rec	i02_3404	/	CDS
CFT073	3418169	3547349	s	rec	i02_3404	/	CDS
CFT073	3418182	3547362	s	rec	i02_3404	/	CDS
CFT073	3418262	3547442	s	rec	i02_3404	/	CDS
CFT073	3418280	3547460	s	rec	i02_3404	/	CDS
CFT073	3418281	3547461	ns	rec	i02_3404	/	CDS
CFT073	3418289	3547469	ns	rec	i02_3404	/	CDS
clone D	3418294	3547474	ns	rec	i02_3404	/	CDS
CFT073	3418307	3547487	s	rec	i02_3404	/	CDS
CFT073	3418322	3547502	s	rec	i02_3404	/	CDS
CFT073	3418400	3547580	s	rec	i02_3404	/	CDS
CFT073	3418460	3547640	s	rec	i02_3404	/	CDS
CFT073	3418466	3547646	s	rec	i02_3404	/	CDS
CFT073	3418469	3547649	s	rec	i02_3404	/	CDS
CFT073	3418482	3547662	ns	rec	i02_3404	/	CDS
CFT073	3418491	3547671	ns	rec	i02_3404	/	CDS
clone D	3418514	3547694	s	rec	i02_3404	/	CDS
CFT073	3418520	3547700	s	rec	i02_3404	/	CDS
CFT073	3418523	3547703	s	rec	i02_3404	/	CDS
CFT073	3418529	3547709	s	rec	i02_3404	/	CDS
CFT073	3418541	3547721	s	rec	i02_3404	/	CDS
CFT073	3418550	3547730	s	rec	i02_3404	/	CDS
CFT073	3418553	3547733	s	rec	i02_3404	/	CDS
CFT073	3418562	3547742	s	rec	i02_3404	/	CDS
clone D	3418574	3547754	s	rec	i02_3404	/	CDS
CFT073	3418577	3547757	s	rec	i02_3404	/	CDS
clone D	3418946	3548126	ns	rec	i02_3405	/	CDS
CFT073	3419024	3548204	s	rec	i02_3406	/	CDS
clone D	3419075	3548255	s	rec	i02_3406	/	CDS
CFT073	3419138	3548318	s	rec	i02_3406	/	CDS
CFT073	3419142	3548322	ns	rec	i02_3406	/	CDS
CFT073	3419156	3548336	s	rec	i02_3406	/	CDS
CFT073	3419174	3548354	s	rec	i02_3406	/	CDS
clone D	3419177	3548357	s	rec	i02_3406	/	CDS
clone D	3419216	3548396	s	rec	i02_3406	/	CDS
CFT073	3419222	3548402	s	rec	i02_3406	/	CDS
clone D	3419226	3548406	ns	rec	i02_3406	/	CDS
clone D	3419306	3548486	s	rec	i02_3406	/	CDS
CFT073	3419393	3548573	s	rec	i02_3406	/	CDS
CFT073	3419402	3548582	s	rec	i02_3406	/	CDS
CFT073	3419423	3548603	s	rec	i02_3406	/	CDS

Lineage	Clone D site	CFT 073 site	mutation type[a]	recomb	Gene	Gene name	Type
CFT073	3419426	3548606	s	rec	i02_3406	/	CDS
CFT073	3419428	3548608	ns	rec	i02_3406	/	CDS
CFT073	3419429	3548609	ns	rec	i02_3406	/	CDS
CFT073	3419432	3548612	ns	rec	i02_3406	/	CDS
CFT073	3419453	3548633	s	rec	i02_3406	/	CDS
CFT073	3419486	3548666	s	rec	i02_3406	/	CDS
CFT073	3419495	3548675	s	rec	i02_3406	/	CDS
CFT073	3419498	3548678	s	rec	i02_3406	/	CDS
CFT073	3419507	3548687	s	rec	i02_3406	/	CDS
CFT073	3419529	3548709	s	rec	i02_3406	/	CDS
CFT073	3419531	3548711	s	rec	i02_3406	/	CDS
CFT073	3419534	3548714	s	rec	i02_3406	/	CDS
clone D	3419564	3548744	s	rec	i02_3406	/	CDS
clone D	3419567	3548747	s	rec	i02_3406	/	CDS
clone D	3419573	3548753	s	rec	i02_3406	/	CDS
CFT073	3419588	3548768	s	rec	i02_3406	/	CDS
clone D	3419629	3548809	ns	rec	i02_3406	/	CDS
clone D	3419636	3548816	s	rec	i02_3406	/	CDS
clone D	3419639	3548819	s	rec	i02_3406	/	CDS
clone D	3419651	3548831	s	rec	i02_3406	/	CDS
clone D	3419654	3548834	s	rec	i02_3406	/	CDS
clone D	3419669	3548849	s	rec	i02_3406	/	CDS
CFT073	3419819	3548999	s	rec	i02_3406	/	CDS
CFT073	3419826	3549006	ns	rec	i02_3406	/	CDS
CFT073	3419861	3549041	s	rec	i02_3406	/	CDS
clone D	3419879	3549059	s	rec	i02_3406	/	CDS
CFT073	3419900	3549080	s	rec	i02_3406	/	CDS
clone D	3419901	3549081	ns	rec	i02_3406	/	CDS
CFT073	3419933	3549113	s	rec	i02_3406	/	CDS
CFT073	3419957	3549137	ns	rec	i02_3406	/	CDS
CFT073	3420032	3549212	s	rec	i02_3406	/	CDS
CFT073	3420068	3549248	s	rec	i02_3406	/	CDS
clone D	3420115	3549295	ns	rec	i02_3406	/	CDS
CFT073	3420131	3549311	nc	rec	intergenic		
CFT073	3420177	3549357	ns	rec	i02_3407	/	CDS
CFT073	3420216	3549396	ns	rec	i02_3407	/	CDS
CFT073	3420304	3549484	s	rec	i02_3407	/	CDS
CFT073	3420316	3549496	s	rec	i02_3407	/	CDS
CFT073	3420358	3549538	s	rec	i02_3407	/	CDS
CFT073	3420397	3549577	s	rec	i02_3407	/	CDS
CFT073	3420402	3549582	s	rec	i02_3407	/	CDS
CFT073	3420514	3549694	s	rec	i02_3407	/	CDS
CFT073	3420523	3549703	s	rec	i02_3407	/	CDS
CFT073	3420540	3549720	ns	rec	i02_3407	/	CDS
CFT073	3420555	3549735	ns	rec	i02_3407	/	CDS
clone D	3420573	3549753	ns	rec	i02_3407	/	CDS
CFT073	3420577	3549757	ns	rec	i02_3407	/	CDS
CFT073	3420578	3549758	ns	rec	i02_3407	/	CDS
clone D	3420587	3549767	ns	rec	i02_3407	/	CDS
CFT073	3420649	3549829	s	rec	i02_3407	/	CDS
CFT073	3420670	3549850	s	rec	i02_3407	/	CDS

Lineage	Clone D site	CFT 073 site	mutation type[a]	recomb	Gene	Gene name	Type
clone D	3420700	3549880	s	rec	i02_3407	/	CDS
CFT073	3420706	3549886	s	rec	i02_3407	/	CDS
CFT073	3420778	3549958	s	rec	i02_3407	/	CDS
CFT073	3420790	3549970	s	rec	i02_3407	/	CDS
CFT073	3420852	3550032	s	rec	i02_3407	/	CDS
CFT073	3420865	3550045	s	rec	i02_3407	/	CDS
CFT073	3420877	3550057	ns	rec	i02_3407	/	CDS
clone D	3420878	3550058	ns	rec	i02_3407	/	CDS
CFT073	3420915	3550095	s	rec	i02_3407	/	CDS
clone D	3420922	3550102	s	rec	i02_3407	/	CDS
CFT073	3420965	3550145	ns	rec	i02_3407	/	CDS
clone D	3421002	3550182	s	rec	i02_3407	/	CDS
CFT073	3421009	3550189	s	rec	i02_3407	/	CDS
CFT073	3421012	3550192	s	rec	i02_3407	/	CDS
clone D	3421021	3550201	s	rec	i02_3407	/	CDS
CFT073	3421042	3550222	s	rec	i02_3407	/	CDS
CFT073	3421078	3550258	s	rec	i02_3407	/	CDS
CFT073	3421093	3550273	s	rec	i02_3407	/	CDS
CFT073	3421120	3550300	s	rec	i02_3407	/	CDS
CFT073	3421207	3550387	s	rec	i02_3407	/	CDS
CFT073	3421225	3550405	s	rec	i02_3407	/	CDS
CFT073	3421244	3550424	ns	rec	i02_3408	/	CDS
CFT073	3421260	3550440	ns	rec	i02_3408	/	CDS
clone D	3421271	3550451	ns	rec	i02_3408	/	CDS
CFT073	3421286	3550466	ns	rec	i02_3408	/	CDS
CFT073	3421311	3550491	ns	rec	i02_3408	/	CDS
CFT073	3421374	3550554	s	rec	i02_3408	/	CDS
CFT073	3421394	3550574	ns	rec	i02_3408	/	CDS
CFT073	3421415	3550595	s	rec	i02_3408	/	CDS
CFT073	3421418	3550598	s	rec	i02_3408	/	CDS
CFT073	3421434	3550614	s	rec	i02_3408	/	CDS
CFT073	3421488	3550668	s	rec	i02_3408	/	CDS
CFT073	3421506	3550686	s	rec	i02_3408	/	CDS
CFT073	3421548	3550728	s	rec	i02_3408	/	CDS
CFT073	3421569	3550749	s	rec	i02_3408	/	CDS
CFT073	3421608	3550788	s	rec	i02_3408	/	CDS
CFT073	3421614	3550794	s	rec	i02_3408	/	CDS
CFT073	3421647	3550827	s	rec	i02_3408	/	CDS
CFT073	3421667	3550847	ns	rec	i02_3408	/	CDS
CFT073	3421677	3550857	s	rec	i02_3408	/	CDS
CFT073	3421680	3550860	s	rec	i02_3408	/	CDS
CFT073	3421686	3550866	s	rec	i02_3408	/	CDS
CFT073	3421878	3551058	s	rec	i02_3408	/	CDS
CFT073	3421902	3551082	s	rec	i02_3408	/	CDS
CFT073	3421905	3551085	s	rec	i02_3408	/	CDS
CFT073	3421938	3551118	s	rec	i02_3408	/	CDS
clone D	3421979	3551159	s	rec	i02_3408	/	CDS
CFT073	3422013	3551193	s	rec	i02_3408	/	CDS
CFT073	3422037	3551217	s	rec	i02_3408	/	CDS
CFT073	3422070	3551250	s	rec	i02_3408	/	CDS
clone D	3422094	3551274	ns	rec	i02_3408	/	CDS

Lineage	Clone D site	CFT 073 site	mutation type[a]	recomb	Gene	Gene name	Type
CFT073	3422095	3551275	ns	rec	i02_3408	/	CDS
clone D	3422100	3551280	s	rec	i02_3408	/	CDS
CFT073	3422103	3551283	s	rec	i02_3408	/	CDS
CFT073	3422117	3551297	s	rec	i02_3408	/	CDS
CFT073	3422136	3551316	s	rec	i02_3408	/	CDS
CFT073	3422310	3551490	nc	rec	intergenic		
CFT073	3422332	3551512	nc	rec	intergenic		
CFT073	3422363	3551543	s	rec	i02_3409	/	CDS
CFT073	3422391	3551571	s	rec	i02_3409	/	CDS
CFT073	3422422	3551602	ns	rec	i02_3409	/	CDS
CFT073	3422438	3551618	s	rec	i02_3409	/	CDS
CFT073	3422439	3551619	s	rec	i02_3409	/	CDS
CFT073	3422469	3551649	ns	rec	i02_3409	/	CDS
CFT073	3422470	3551650	ns	rec	i02_3409	/	CDS
CFT073	3422488	3551668	ns	rec	i02_3409	/	CDS
CFT073	3422490	3551670	s	rec	i02_3409	/	CDS
CFT073	3422556	3551736	s	rec	i02_3409	/	CDS
CFT073	3422601	3551781	s	rec	i02_3409	/	CDS
CFT073	3422627	3551807	ns	rec	i02_3409	/	CDS
CFT073	3422679	3551859	s	rec	i02_3409	/	CDS
CFT073	3422688	3551868	s	rec	i02_3409	/	CDS
CFT073	3422723	3551903	ns	rec	i02_3409	/	CDS
CFT073	3422724	3551904	s	rec	i02_3409	/	CDS
CFT073	3422808	3551988	s	rec	i02_3409	/	CDS
CFT073	3422842	3552022	ns	rec	i02_3409	/	CDS
CFT073	3422843	3552023	ns	rec	i02_3409	/	CDS
CFT073	3422845	3552025	ns	rec	i02_3409	/	CDS
CFT073	3422847	3552027	s	rec	i02_3409	/	CDS
CFT073	3422850	3552030	s	rec	i02_3409	/	CDS
CFT073	3422853	3552033	s	rec	i02_3409	/	CDS
CFT073	3422862	3552042	ns	rec	i02_3409	/	CDS
CFT073	3422863	3552043	ns	rec	i02_3409	/	CDS
CFT073	3422864	3552044	ns	rec	i02_3409	/	CDS
CFT073	3422868	3552048	s	rec	i02_3409	/	CDS
CFT073	3422873	3552053	s	rec	i02_3409	/	CDS
CFT073	3422874	3552054	s	rec	i02_3409	/	CDS
CFT073	3422877	3552057	ns	rec	i02_3409	/	CDS
CFT073	3422879	3552059	ns	rec	i02_3409	/	CDS
CFT073	3422884	3552064	ns	rec	i02_3409	/	CDS
CFT073	3422894	3552074	ns	rec	i02_3409	/	CDS
CFT073	3422898	3552078	s	rec	i02_3410	/	CDS
CFT073	3422909	3552089	ns	rec	i02_3410	/	CDS
CFT073	3422915	3552095	s	rec	i02_3410	/	CDS
CFT073	3422918	3552098	s	rec	i02_3410	/	CDS
CFT073	3422930	3552110	s	rec	i02_3410	/	CDS
CFT073	3422936	3552116	s	rec	i02_3410	/	CDS
CFT073	3422942	3552122	s	rec	i02_3410	/	CDS
CFT073	3422948	3552128	s	rec	i02_3410	/	CDS
CFT073	3422984	3552164	s	rec	i02_3410	/	CDS
CFT073	3422988	3552168	ns	rec	i02_3410	/	CDS
CFT073	3422990	3552170	s	rec	i02_3410	/	CDS

Lineage	Clone D site	CFT 073 site	mutation type[a]	recomb	Gene	Gene name	Type
CFT073	3422996	3552176	ns	rec	i02_3410	/	CDS
CFT073	3423026	3552206	s	rec	i02_3410	/	CDS
CFT073	3423032	3552212	s	rec	i02_3410	/	CDS
CFT073	3423041	3552221	s	rec	i02_3410	/	CDS
CFT073	3423059	3552239	s	rec	i02_3410	/	CDS
CFT073	3423062	3552242	s	rec	i02_3410	/	CDS
CFT073	3423068	3552248	s	rec	i02_3410	/	CDS
CFT073	3423072	3552252	ns	rec	i02_3410	/	CDS
CFT073	3423167	3552347	s	rec	i02_3410	/	CDS
CFT073	3423170	3552350	s	rec	i02_3410	/	CDS
CFT073	3423177	3552357	ns	rec	i02_3410	/	CDS
CFT073	3423185	3552365	s	rec	i02_3410	/	CDS
CFT073	3423204	3552384	ns	rec	i02_3410	/	CDS
CFT073	3423206	3552386	ns	rec	i02_3410	/	CDS
CFT073	3423208	3552388	ns	rec	i02_3410	/	CDS
CFT073	3423218	3552398	s	rec	i02_3410	/	CDS
CFT073	3423221	3552401	s	rec	i02_3410	/	CDS
CFT073	3423224	3552404	s	rec	i02_3410	/	CDS
CFT073	3423230	3552410	s	rec	i02_3410	/	CDS
CFT073	3423238	3552418	ns	rec	i02_3410	/	CDS
CFT073	3423240	3552420	ns	rec	i02_3410	/	CDS
CFT073	3423242	3552422	s	rec	i02_3410	/	CDS
CFT073	3423246	3552426	ns	rec	i02_3410	/	CDS
CFT073	3423266	3552446	s	rec	i02_3410	/	CDS
CFT073	3423283	3552463	s	rec	i02_3410	/	CDS
CFT073	3423285	3552465	ns	rec	i02_3410	/	CDS
CFT073	3423293	3552473	s	rec	i02_3410	/	CDS
CFT073	3423296	3552476	s	rec	i02_3410	/	CDS
CFT073	3423302	3552482	s	rec	i02_3410	/	CDS
CFT073	3423310	3552490	ns	rec	i02_3410	/	CDS
CFT073	3423311	3552491	s	rec	i02_3410	/	CDS
CFT073	3423323	3552503	s	rec	i02_3410	/	CDS
CFT073	3423330	3552510	ns	rec	i02_3410	/	CDS
CFT073	3423365	3552545	s	rec	i02_3410	/	CDS
CFT073	3423375	3552555	ns	rec	i02_3410	/	CDS
CFT073	3423380	3552560	s	rec	i02_3410	/	CDS
CFT073	3423455	3552635	s	rec	i02_3410	/	CDS
CFT073	3423485	3552665	s	rec	i02_3410	/	CDS
CFT073	3423497	3552677	s	rec	i02_3410	/	CDS
CFT073	3423500	3552680	ns	rec	i02_3410	/	CDS
CFT073	3423502	3552682	ns	rec	i02_3410	/	CDS
CFT073	3423503	3552683	s	rec	i02_3410	/	CDS
CFT073	3423521	3552701	s	rec	i02_3410	/	CDS
CFT073	3423524	3552704	s	rec	i02_3410	/	CDS
CFT073	3423533	3552713	ns	rec	i02_3410	/	CDS
CFT073	3423535	3552715	ns	rec	i02_3410	/	CDS
CFT073	3423559	3552739	ns	rec	i02_3410	/	CDS
CFT073	3423563	3552743	s	rec	i02_3410	/	CDS
CFT073	3423568	3552748	ns	rec	i02_3410	/	CDS
CFT073	3423569	3552749	s	rec	i02_3410	/	CDS
CFT073	3423580	3552760	ns	rec	i02_3410	/	CDS

Lineage	Clone D site	CFT 073 site	mutation type[a]	recomb	Gene	Gene name	Type
CFT073	3423605	3552785	s	rec	i02_3410	/	CDS
CFT073	3423632	3552812	s	rec	i02_3410	/	CDS
CFT073	3423650	3552830	s	rec	i02_3410	/	CDS
CFT073	3423665	3552845	s	rec	i02_3410	/	CDS
CFT073	3423750	3552930	ns	rec	i02_3411	yghQ	CDS
CFT073	3423753	3552933	ns	rec	i02_3411	yghQ	CDS
CFT073	3423796	3552976	s	rec	i02_3411	yghQ	CDS
CFT073	3423804	3552984	s	rec	i02_3411	yghQ	CDS
CFT073	3423812	3552992	ns	rec	i02_3411	yghQ	CDS
CFT073	3423817	3552997	s	rec	i02_3411	yghQ	CDS
CFT073	3423847	3553027	s	rec	i02_3411	yghQ	CDS
CFT073	3423850	3553030	s	rec	i02_3411	yghQ	CDS
CFT073	3423853	3553033	s	rec	i02_3411	yghQ	CDS
CFT073	3423862	3553042	s	rec	i02_3411	yghQ	CDS
CFT073	3423973	3553153	s	rec	i02_3411	yghQ	CDS
CFT073	3424141	3553321	s	rec	i02_3411	yghQ	CDS
clone D	3424160	3553340	ns	rec	i02_3411	yghQ	CDS
CFT073	3424168	3553348	s	rec	i02_3411	yghQ	CDS
CFT073	3424192	3553372	s	rec	i02_3411	yghQ	CDS
CFT073	3424204	3553384	s	rec	i02_3411	yghQ	CDS
CFT073	3424210	3553390	s	rec	i02_3411	yghQ	CDS
CFT073	3424234	3553414	s	rec	i02_3411	yghQ	CDS
CFT073	3424270	3553450	s	rec	i02_3411	yghQ	CDS
CFT073	3424383	3553563	ns	rec	i02_3411	yghQ	CDS
CFT073	3424431	3553611	s	rec	i02_3411	yghQ	CDS
clone D	3424539	3553719	ns	rec	i02_3411	yghQ	CDS
clone D	3424579	3553759	s	rec	i02_3411	yghQ	CDS
clone D	3424596	3553776	s	rec	i02_3411	yghQ	CDS
clone D	3424669	3553849	s	rec	i02_3411	yghQ	CDS
CFT073	3424687	3553867	s	rec	i02_3411	yghQ	CDS
clone D	3424720	3553900	s	rec	i02_3411	yghQ	CDS
clone D	3424723	3553903	s	rec	i02_3411	yghQ	CDS
clone D	3424726	3553906	s	rec	i02_3411	yghQ	CDS
CFT073	3424741	3553921	s	rec	i02_3411	yghQ	CDS
CFT073	3424858	3554038	s	rec	i02_3411	yghQ	CDS
clone D	3424894	3554074	s	rec	i02_3411	yghQ	CDS
CFT073	3424915	3554095	s	rec	i02_3411	yghQ	CDS
CFT073	3424975	3554155	s	rec	i02_3411	yghQ	CDS
CFT073	3424984	3554164	s	rec	i02_3411	yghQ	CDS
CFT073	3424993	3554173	s	rec	i02_3411	yghQ	CDS
CFT073	3425197	3554377	s	rec	i02_3412	yghR	CDS
CFT073	3425206	3554386	s	rec	i02_3412	yghR	CDS
CFT073	3425255	3554435	ns	rec	i02_3412	yghR	CDS
clone D	3425263	3554443	s	rec	i02_3412	yghR	CDS
CFT073	3425323	3554503	ns	rec	i02_3412	yghR	CDS
CFT073	3425335	3554515	s	rec	i02_3412	yghR	CDS
CFT073	3425340	3554520	s	rec	i02_3412	yghR	CDS
CFT073	3425347	3554527	s	rec	i02_3412	yghR	CDS
CFT073	3425356	3554536	s	rec	i02_3412	yghR	CDS
CFT073	3425359	3554539	s	rec	i02_3412	yghR	CDS
CFT073	3425362	3554542	s	rec	i02_3412	yghR	CDS

Lineage	Clone D site	CFT 073 site	mutation type[a]	recomb	Gene	Gene name	Type
CFT073	3425376	3554556	ns	rec	i02_3412	yghR	CDS
CFT073	3425395	3554575	s	rec	i02_3412	yghR	CDS
clone D	3425411	3554591	ns	rec	i02_3412	yghR	CDS
CFT073	3425455	3554635	s	rec	i02_3412	yghR	CDS
CFT073	3425542	3554722	s	rec	i02_3412	yghR	CDS
CFT073	3425656	3554836	s	rec	i02_3412	yghR	CDS
CFT073	3425665	3554845	s	rec	i02_3412	yghR	CDS
CFT073	3425770	3554950	ns	rec	i02_3412	yghR	CDS
CFT073	3425771	3554951	ns	rec	i02_3412	yghR	CDS
CFT073	3425773	3554953	s	rec	i02_3412	yghR	CDS
CFT073	3425857	3555037	ns	rec	i02_3412	yghR	CDS
CFT073	3425869	3555049	s	rec	i02_3412	yghR	CDS
CFT073	3425892	3555072	ns	rec	i02_3412	yghR	CDS
CFT073	3425972	3555152	s	rec	i02_3413	yghS	CDS
CFT073	3425975	3555155	ns	rec	i02_3413	yghS	CDS
CFT073	3425978	3555158	s	rec	i02_3413	yghS	CDS
CFT073	3425981	3555161	s	rec	i02_3413	yghS	CDS
CFT073	3426122	3555302	s	rec	i02_3413	yghS	CDS
?	3426134	3555314	s	rec	i02_3413	yghS	CDS
CFT073	3426161	3555341	s	rec	i02_3413	yghS	CDS
CFT073	3426230	3555410	s	rec	i02_3413	yghS	CDS
CFT073	3426233	3555413	s	rec	i02_3413	yghS	CDS
CFT073	3426276	3555456	ns	rec	i02_3413	yghS	CDS
CFT073	3426288	3555468	ns	rec	i02_3413	yghS	CDS
CFT073	3426350	3555530	s	rec	i02_3413	yghS	CDS
CFT073	3426412	3555592	s	rec	i02_3413	yghS	CDS
CFT073	3426443	3555623	s	rec	i02_3413	yghS	CDS
CFT073	3426514	3555694	ns	rec	i02_3413	yghS	CDS
CFT073	3426646	3555826	nc	rec	intergenic		
clone D	3426949	3556129	s	rec	i02_3414	yghT	CDS
CFT073	3427051	3556231	s	rec	i02_3414	yghT	CDS
clone D	3427081	3556261	s	rec	i02_3414	yghT	CDS
CFT073	3427613	3556793	s	rec	i02_3415	pitB	CDS
CFT073	3427688	3556868	s	rec	i02_3415	pitB	CDS
CFT073	3427694	3556874	s	rec	i02_3415	pitB	CDS
CFT073	3428123	3557303	s	rec	i02_3415	pitB	CDS
CFT073	3428285	3557465	s	rec	i02_3415	pitB	CDS
CFT073	3428363	3557543	s	rec	i02_3415	pitB	CDS
CFT073	3428372	3557552	s	rec	i02_3415	pitB	CDS
CFT073	3428375	3557555	s	rec	i02_3415	pitB	CDS
clone D	3428465	3557645	s	rec	i02_3415	pitB	CDS
clone D	3428471	3557651	s	rec	i02_3415	pitB	CDS
clone D	3428474	3557654	s	rec	i02_3415	pitB	CDS
clone D	3428627	3557807	s	rec	i02_3415	pitB	CDS
clone D	3428630	3557810	ns	rec	i02_3415	pitB	CDS
clone D	3428631	3557811	ns	rec	i02_3415	pitB	CDS
clone D	3429193	3558373	nc	rec	intergenic		
clone D	3429216	3558396	nc	rec	intergenic		
CFT073	3429305	3558485	nc	rec	intergenic		
CFT073	3429321	3558501	nc	rec	intergenic		
CFT073	3429409	3558589	s	rec	i02_3416	gsp	CDS

Lineage	Clone D site	CFT 073 site	mutation type[a]	recomb	Gene	Gene name	Type
CFT073	3429410	3558590	s	rec	i02_3416	gsp	CDS
CFT073	3429413	3558593	s	rec	i02_3416	gsp	CDS
CFT073	3429416	3558596	s	rec	i02_3416	gsp	CDS
CFT073	3429422	3558602	s	rec	i02_3416	gsp	CDS
CFT073	3429428	3558608	s	rec	i02_3416	gsp	CDS
CFT073	3429431	3558611	s	rec	i02_3416	gsp	CDS
CFT073	3429437	3558617	s	rec	i02_3416	gsp	CDS
CFT073	3429443	3558623	s	rec	i02_3416	gsp	CDS
CFT073	3429449	3558629	s	rec	i02_3416	gsp	CDS
CFT073	3429455	3558635	s	rec	i02_3416	gsp	CDS
CFT073	3429458	3558638	s	rec	i02_3416	gsp	CDS
CFT073	3429461	3558641	s	rec	i02_3416	gsp	CDS
CFT073	3429476	3558656	s	rec	i02_3416	gsp	CDS
CFT073	3429481	3558661	s	rec	i02_3416	gsp	CDS
CFT073	3429482	3558662	s	rec	i02_3416	gsp	CDS
clone D	3429497	3558677	s	rec	i02_3416	gsp	CDS
clone D	3429500	3558680	s	rec	i02_3416	gsp	CDS
clone D	3429503	3558683	s	rec	i02_3416	gsp	CDS
clone D	3429512	3558692	s	rec	i02_3416	gsp	CDS
clone D	3429527	3558707	s	rec	i02_3416	gsp	CDS
clone D	3429533	3558713	ns	rec	i02_3416	gsp	CDS
clone D	3429535	3558715	ns	rec	i02_3416	gsp	CDS
clone D	3429536	3558716	s	rec	i02_3416	gsp	CDS
CloneD	3429539	3558719	s	rec	i02_3416	gsp	CDS
clone D	3429563	3558743	s	rec	i02_3416	gsp	CDS
clone D	3429569	3558749	s	rec	i02_3416	gsp	CDS
clone D	3429575	3558755	s	rec	i02_3416	gsp	CDS
clone D	3429578	3558758	s	rec	i02_3416	gsp	CDS
clone D	3429587	3558767	s	rec	i02_3416	gsp	CDS
clone D	3429593	3558773	s	rec	i02_3416	gsp	CDS
clone D	3429758	3558938	s	rec	i02_3416	gsp	CDS
clone D	3429760	3558940	s	rec	i02_3416	gsp	CDS
clone D	3429763	3558943	s	rec	i02_3416	gsp	CDS
clone D	3429764	3558944	s	rec	i02_3416	gsp	CDS
clone D	3429770	3558950	s	rec	i02_3416	gsp	CDS
clone D	3429773	3558953	s	rec	i02_3416	gsp	CDS
clone D	3429779	3558959	s	rec	i02_3416	gsp	CDS
clone D	3429791	3558971	s	rec	i02_3416	gsp	CDS
clone D	3429797	3558977	s	rec	i02_3416	gsp	CDS
CFT073	3429815	3558995	s	rec	i02_3416	gsp	CDS
CFT073	3429818	3558998	s	rec	i02_3416	gsp	CDS
CFT073	3429824	3559004	s	rec	i02_3416	gsp	CDS
CFT073	3429836	3559016	s	rec	i02_3416	gsp	CDS
CFT073	3429842	3559022	s	rec	i02_3416	gsp	CDS
CFT073	3429845	3559025	s	rec	i02_3416	gsp	CDS
CFT073	3429848	3559028	s	rec	i02_3416	gsp	CDS
CFT073	3429857	3559037	s	rec	i02_3416	gsp	CDS
CFT073	3429860	3559040	s	rec	i02_3416	gsp	CDS
CFT073	3429884	3559064	s	rec	i02_3416	gsp	CDS
CFT073	3429887	3559067	s	rec	i02_3416	gsp	CDS
clone D	3429950	3559130	s	rec	i02_3416	gsp	CDS

Lineage	Clone D site	CFT 073 site	mutation type[a]	recomb	Gene	Gene name	Type
CFT073	3429952	3559132	s	rec	i02_3416	gsp	CDS
clone D	3429956	3559136	s	rec	i02_3416	gsp	CDS
clone D	3429986	3559166	ns	rec	i02_3416	gsp	CDS
CFT073	3430010	3559190	s	rec	i02_3416	gsp	CDS
CFT073	3430022	3559202	s	rec	i02_3416	gsp	CDS
CFT073	3430025	3559205	s	rec	i02_3416	gsp	CDS
CFT073	3430028	3559208	s	rec	i02_3416	gsp	CDS
CFT073	3430064	3559244	s	rec	i02_3416	gsp	CDS
CFT073	3430067	3559247	s	rec	i02_3416	gsp	CDS
CFT073	3430082	3559262	s	rec	i02_3416	gsp	CDS
CFT073	3430085	3559265	s	rec	i02_3416	gsp	CDS
CFT073	3430106	3559286	s	rec	i02_3416	gsp	CDS
CFT073	3430256	3559436	s	rec	i02_3416	gsp	CDS
CFT073	3430268	3559448	s	rec	i02_3416	gsp	CDS
CFT073	3430325	3559505	s	rec	i02_3416	gsp	CDS
CFT073	3430330	3559510	s	rec	i02_3416	gsp	CDS
CFT073	3430424	3559604	s	rec	i02_3416	gsp	CDS
CFT073	3430472	3559652	s	rec	i02_3416	gsp	CDS
CFT073	3430598	3559778	s	rec	i02_3416	gsp	CDS
CFT073	3430601	3559781	s	rec	i02_3416	gsp	CDS
CFT073	3430613	3559793	s	rec	i02_3416	gsp	CDS
CFT073	3430853	3560033	s	rec	i02_3416	gsp	CDS
CFT073	3430859	3560039	s	rec	i02_3416	gsp	CDS
CFT073	3431413	3560593	s	rec	i02_3417	yghU	CDS
CFT073	3431472	3560652	ns	rec	i02_3417	yghU	CDS
CFT073	3431905	3561085	s	rec	i02_3417	yghU	CDS
CFT073	3432043	3561223	s	rec	i02_3417	yghU	CDS
CFT073	3432073	3561253	s	rec	i02_3417	yghU	CDS
CFT073	3432079	3561259	s	rec	i02_3417	yghU	CDS
CFT073	3432115	3561295	s	rec	i02_3417	yghU	CDS
CFT073	3432118	3561298	s	rec	i02_3417	yghU	CDS
CFT073	3432346	3561526	nc	rec	intergenic		
CFT073	3432911	3562091	s	rec	i02_3419	hypA	CDS
clone D	3433060	3562240	ns	rec	i02_3420	hybE	CDS
CFT073	3433194	3562374	s	rec	i02_3420	hybE	CDS
CFT073	3433441	3562621	ns	rec	i02_3420	hybE	CDS
clone D	3433575	3562755	ns	rec	i02_3421	hybD	CDS
clone D	3433660	3562840	s	rec	i02_3421	hybD	CDS
CFT073	3433831	3563011	s	rec	i02_3421	hybD	CDS
CFT073	3434375	3563555	ns	rec	i02_3422	hybC	CDS
CFT073	3434622	3563802	s	rec	i02_3422	hybC	CDS
CFT073	3434625	3563805	s	rec	i02_3422	hybC	CDS
clone D	3434706	3563886	s	rec	i02_3422	hybC	CDS
CFT073	3434863	3564043	ns	rec	i02_3422	hybC	CDS
CFT073	3436040	3565220	s	rec	i02_3423	hybB	CDS
clone D	3436103	3565283	s	rec	i02_3423	hybB	CDS
clone D	3436292	3565472	s	rec	i02_3423	hybB	CDS
clone D	3436343	3565523	s	rec	i02_3423	hybB	CDS
clone D	3436378	3565558	ns	rec	i02_3423	hybB	CDS
CFT073	3437256	3566436	s	rec	i02_3424	hybA	CDS
CFT073	3437451	3566631	s	rec	i02_3424	hybA	CDS

Lineage	Clone D site	CFT 073 site	mutation type[a]	recomb	Gene	Gene name	Type
CFT073	3438041	3567221	s	rec	i02_3425	/	CDS
clone D	3439896	3569076	ns	rec	i02_3428	/	CDS
clone D	3439897	3569077	ns	rec	i02_3428	/	CDS
CFT073	3440016	3569196	ns	rec	i02_3428	/	CDS
clone D	3440066	3569246	ns	rec	i02_3428	/	CDS
clone D	3440067	3569247	ns	rec	i02_3428	/	CDS
clone D	3440150	3569330	s	rec	i02_3428	/	CDS
clone D	3440324	3569504	s	rec	i02_3428	/	CDS
clone D	3440341	3569521	ns	rec	i02_3428	/	CDS
clone D	3440415	3569595	ns	rec	i02_3428	/	CDS
clone D	3440423	3569603	s	rec	i02_3428	/	CDS
clone D	3440536	3569716	nc	rec	intergenic		
clone D	3440578	3569758	nc	rec	intergenic		
clone D	3440668	3569848	s	rec	i02_3429	yghZ	CDS
clone D	3441061	3570241	s	rec	i02_3429	yghZ	CDS
CFT073	3441694	3570874	s	rec	i02_3430	yqhA	CDS
CFT073	3442087	3571267	ns	rec	i02_3430	yqhA	CDS
clone D	3442357	3571537	nc	rec	intergenic		
CFT073	3442992	3572172	ns	rec	i02_3431	yghA	CDS
CFT073	3443723	3572903	s	rec	i02_3433	exbB	CDS
CFT073	3443753	3572933	s	rec	i02_3433	exbB	CDS
CFT073	3443794	3572974	s	rec	i02_3433	exbB	CDS
CFT073	3443933	3573113	s	rec	i02_3433	exbB	CDS
CFT073	3444193	3573373	ns	rec	i02_3433	exbB	CDS
CFT073	3444268	3573448	s	rec	i02_3433	exbB	CDS
CFT073	3444588	3573768	s	rec	i02_3434	metC	CDS
CFT073	3444704	3573884	ns	rec	i02_3434	metC	CDS
CFT073	3444727	3573907	s	rec	i02_3434	metC	CDS
CFT073	3444759	3573939	s	rec	i02_3434	metC	CDS
CFT073	3444816	3573996	s	rec	i02_3434	metC	CDS
CFT073	3445368	3574548	s	rec	i02_3434	metC	CDS
CFT073	3445473	3574653	s	rec	i02_3434	metC	CDS
CFT073	3445476	3574656	s	rec	i02_3434	metC	CDS
CFT073	3445479	3574659	s	rec	i02_3434	metC	CDS
CFT073	3445482	3574662	s	rec	i02_3434	metC	CDS
CFT073	3445485	3574665	s	rec	i02_3434	metC	CDS
CFT073	3445486	3574666	s	rec	i02_3434	metC	CDS
CFT073	3445491	3574671	s	rec	i02_3434	metC	CDS
CFT073	3446192	3575372	s	rec	i02_3435	yghB	CDS
CFT073	3446197	3575377	s	rec	i02_3435	yghB	CDS
CFT073	3446575	3575755	s	rec	i02_3435	yghB	CDS
CFT073	3447044	3576224	s	rec	i02_3436	yqhC	CDS
CFT073	3447071	3576251	s	rec	i02_3436	yqhC	CDS
clone D	3447137	3576317	s	rec	i02_3436	yqhC	CDS
clone D	3447155	3576335	s	rec	i02_3436	yqhC	CDS
clone D	3447182	3576362	s	rec	i02_3436	yqhC	CDS
clone D	3447185	3576365	s	rec	i02_3436	yqhC	CDS
clone D	3447197	3576377	s	rec	i02_3436	yqhC	CDS
clone D	3447201	3576381	ns	rec	i02_3436	yqhC	CDS
clone D	3447230	3576410	s	rec	i02_3436	yqhC	CDS
clone D	3447239	3576419	s	rec	i02_3436	yqhC	CDS

Lineage	Clone D site	CFT 073 site	mutation type[a]	recomb	Gene	Gene name	Type
clone D	3447254	3576434	s	rec	i02_3436	yqhC	CDS
clone D	3447263	3576443	s	rec	i02_3436	yqhC	CDS
clone D	3447269	3576449	s	rec	i02_3436	yqhC	CDS
clone D	3447284	3576464	ns	rec	i02_3436	yqhC	CDS
clone D	3447398	3576578	s	rec	i02_3436	yqhC	CDS
clone D	3447401	3576581	s	rec	i02_3436	yqhC	CDS
clone D	3447410	3576590	s	rec	i02_3436	yqhC	CDS
clone D	3447413	3576593	s	rec	i02_3436	yqhC	CDS
clone D	3447455	3576635	s	rec	i02_3436	yqhC	CDS
clone D	3447995	3577175	s	rec	i02_3437	yqhD	CDS
clone D	3448070	3577250	s	rec	i02_3437	yqhD	CDS
clone D	3448079	3577259	s	rec	i02_3437	yqhD	CDS
clone D	3448082	3577262	s	rec	i02_3437	yqhD	CDS
CFT073	3448133	3577313	s	rec	i02_3437	yqhD	CDS
clone D	3448190	3577370	s	rec	i02_3437	yqhD	CDS
clone D	3448371	3577551	ns	rec	i02_3437	yqhD	CDS
clone D	3448837	3578017	ns	rec	i02_3437	yqhD	CDS
CFT073	3449337	3578517	ns	rec	i02_3438	dkgA	CDS
clone D	3449438	3578618	ns	rec	i02_3438	dkgA	CDS
CFT073	3450138	3579318	ns	rec	i02_3439	yqhG	CDS
clone D	3450266	3579447	s	rec	i02_3439	yqhG	CDS
clone D	3450275	3579456	s	rec	i02_3439	yqhG	CDS
CFT073	3450449	3579630	s	rec	i02_3439	yqhG	CDS
CFT073	3452185	3581366	s	rec	i02_3441	/	CDS
clone D	3452197	3581378	s	rec	i02_3441	/	CDS
clone D	3452212	3581393	s	rec	i02_3441	/	CDS
clone D	3452281	3581462	s	rec	i02_3441	/	CDS
CFT073	3453043	3582224	s	rec	i02_3441	/	CDS
CFT073	3453049	3582230	s	rec	i02_3441	/	CDS
CFT073	3453060	3582241	s	rec	i02_3441	/	CDS
CFT073	3453064	3582245	s	rec	i02_3441	/	CDS
CFT073	3453082	3582263	s	rec	i02_3441	/	CDS
CFT073	3453172	3582353	s	rec	i02_3441	/	CDS
CFT073	3453222	3582403	ns	rec	i02_3441	/	CDS
CFT073	3453226	3582407	s	rec	i02_3441	/	CDS
CFT073	3453399	3582580	ns	rec	i02_3441	/	CDS
clone D	3453490	3582671	s	rec	i02_3441	/	CDS
clone D	3453794	3582975	nc	rec	intergenic		
clone D	3453822	3583003	nc	rec	intergenic		
CFT073	3455079	3584260	s	rec	i02_3443	/	CDS
clone D	3455085	3584266	s	rec	i02_3443	/	CDS
CFT073	3455093	3584274	ns	rec	i02_3443	/	CDS
CFT073	3455112	3584293	s	rec	i02_3443	/	CDS
CFT073	3455223	3584404	s	rec	i02_3443	/	CDS
CFT073	3455224	3584405	ns	rec	i02_3443	/	CDS
CFT073	3455245	3584426	ns	rec	i02_3443	/	CDS
CFT073	3455246	3584427	ns	rec	i02_3443	/	CDS
CFT073	3455247	3584428	ns	rec	i02_3443	/	CDS
CFT073	3455271	3584452	ns	rec	i02_3443	/	CDS
CFT073	3455275	3584456	s	rec	i02_3443	/	CDS
CFT073	3455278	3584459	ns	rec	i02_3443	/	CDS

Lineage	Clone D site	CFT 073 site	mutation type[a]	recomb	Gene	Gene name	Type
CFT073	3455325	3584506	s	rec	i02_3443	/	CDS
CFT073	3455331	3584512	s	rec	i02_3443	/	CDS
CFT073	3455338	3584519	ns	rec	i02_3443	/	CDS
CFT073	3455346	3584527	s	rec	i02_3443	/	CDS
CFT073	3455349	3584530	s	rec	i02_3443	/	CDS
CFT073	3455352	3584533	s	rec	i02_3443	/	CDS
CFT073	3455377	3584558	ns	rec	i02_3443	/	CDS
CFT073	3455408	3584589	ns	rec	i02_3443	/	CDS
CFT073	3455412	3584593	s	rec	i02_3443	/	CDS
CFT073	3455422	3584603	ns	rec	i02_3443	/	CDS
CFT073	3455455	3584636	ns	rec	i02_3443	/	CDS
CFT073	3455460	3584641	s	rec	i02_3443	/	CDS
CFT073	3455475	3584656	s	rec	i02_3443	/	CDS
CFT073	3455487	3584668	s	rec	i02_3443	/	CDS
CFT073	3455520	3584701	s	rec	i02_3443	/	CDS
clone D	3455631	3584812	s	rec	i02_3443	/	CDS
clone D	3455718	3584899	s	rec	i02_3443	/	CDS
clone D	3455735	3584916	ns	rec	i02_3443	/	CDS
clone D	3455736	3584917	ns	rec	i02_3443	/	CDS
clone D	3455739	3584920	s	rec	i02_3443	/	CDS
clone D	3455745	3584926	s	rec	i02_3443	/	CDS
clone D	3455746	3584927	ns	rec	i02_3443	/	CDS
clone D	3455760	3584941	s	rec	i02_3443	/	CDS
CFT073	3455761	3584942	ns	rec	i02_3443	/	CDS
clone D	3455820	3585001	s	rec	i02_3443	/	CDS
clone D	3455824	3585005	ns	rec	i02_3443	/	CDS
clone D	3455841	3585022	s	rec	i02_3443	/	CDS
CFT073	3455868	3585049	s	rec	i02_3443	/	CDS
CFT073	3455896	3585077	ns	rec	i02_3443	/	CDS
CFT073	3455968	3585149	ns	rec	i02_3443	/	CDS
CFT073	3455971	3585152	ns	rec	i02_3443	/	CDS
CFT073	3455972	3585153	ns	rec	i02_3443	/	CDS
clone D	3456057	3585238	s	rec	i02_3443	/	CDS
clone D	3456075	3585256	s	rec	i02_3443	/	CDS
clone D	3456168	3585349	ns	rec	i02_3443	/	CDS
clone D	3456195	3585376	s	rec	i02_3443	/	CDS
clone D	3456210	3585391	ns	rec	i02_3443	/	CDS
clone D	3456214	3585395	ns	rec	i02_3443	/	CDS
clone D	3456494	3585675	s	rec	i02_3444	/	CDS
CFT073	3456629	3585810	s	rec	i02_3444	/	CDS
CFT073	3456630	3585811	ns	rec	i02_3444	/	CDS
CFT073	3456631	3585812	ns	rec	i02_3444	/	CDS
clone D	3456942	3586123	s	rec	i02_3444	/	CDS
clone D	3457139	3586320	s	rec	i02_3444	/	CDS
CFT073	3457284	3586465	ns	rec	i02_3444	/	CDS
clone D	3457338	3586519	ns	rec	i02_3444	/	CDS
CFT073	3457400	3586581	s	rec	i02_3444	/	CDS
clone D	3457866	3587047	s	rec	i02_3445	/	CDS
clone D	3458790	3587971	s	rec	i02_3446	/	CDS
clone D	3458952	3588133	s	rec	i02_3446	/	CDS
CFT073	3459219	3588400	s	rec	i02_3446	/	CDS

Lineage	Clone D site	CFT 073 site	mutation type[a]	recomb	Gene	Gene name	Type
clone D	3459249	3588430	s	rec	i02_3446	/	CDS
clone D	3459327	3588508	s	rec	i02_3446	/	CDS
clone D	3459707	3588888	s	rec	i02_3447	/	CDS
clone D	3459731	3588912	s	rec	i02_3447	/	CDS
clone D	3460485	3589666	s	rec	i02_3448	ygiK	CDS
clone D	3460642	3589823	ns	rec	i02_3448	ygiK	CDS
CFT073	3460662	3589843	s	rec	i02_3448	ygiK	CDS
CFT073	3460668	3589849	s	rec	i02_3448	ygiK	CDS
clone D	3460673	3589854	ns	rec	i02_3448	ygiK	CDS
clone D	3460674	3589855	ns	rec	i02_3448	ygiK	CDS
clone D	3460689	3589870	s	rec	i02_3448	ygiK	CDS
?	3460752	3589933	s	rec	i02_3448	ygiK	CDS
CFT073	3460863	3590044	s	rec	i02_3448	ygiK	CDS
CFT073	3460866	3590047	s	rec	i02_3448	ygiK	CDS
CFT073	3461001	3590182	s	rec	i02_3448	ygiK	CDS
CFT073	3461130	3590311	s	rec	i02_3448	ygiK	CDS
CFT073	3461588	3590769	s	rec	i02_3449	sufI	CDS
clone D	3462221	3591402	s	rec	i02_3449	sufI	CDS
CFT073	3462269	3591450	s	rec	i02_3449	sufI	CDS
CFT073	3462314	3591495	s	rec	i02_3449	sufI	CDS
CFT073	3462488	3591669	s	rec	i02_3449	sufI	CDS
CFT073	3462539	3591720	s	rec	i02_3449	sufI	CDS
CFT073	3462636	3591817	ns	rec	i02_3449	sufI	CDS
CFT073	3463054	3592235	s	rec	i02_3450	plsC	CDS
CFT073	3486515	3615696	ns	out	i02_3475	tolC	CDS
clone D	3508124	3637305	ns	out	i02_3495	glnE	CDS
CFT073	3509103	3638284	ns	out	i02_3495	glnE	CDS
CFT073	3529000	3658183	ns	out	i02_3519	aer	CDS
clone D	3536702	3665885	s	out	i02_3524	ebgA	CDS
CFT073	3538980	3668163	s	out	i02_3527	ygjI	CDS
CFT073	3546455	3675638	s	out	i02_3533	ygjO	CDS
clone D	3555708	3684891	nc	out	intergenic		
CFT073	3555892	3685075	nc	out	intergenic		
?	3562222	3691404	ns	out	i02_3551	yqjG	CDS
clone D	3562224	3691406	ns	out	i02_3551	yqjG	CDS
clone D	3562225	3691407	ns	out	i02_3551	yqjG	CDS
CFT073	3567137	3696321	ns	rec	i02_3557	/	CDS
?	3567155	3696339	ns	rec	i02_3557	/	CDS
clone D	3567156	3696340	ns	rec	i02_3557	/	CDS
clone D	3567159	3696343	s	rec	i02_3557	/	CDS
clone D	3577387	3706573	nc	out	intergenic		
CFT073	3577761	3706947	ns	out	i02_3565	tdcR	CDS
CFT073	3584797	3713983	s	out	i02_3573	yhaG	CDS
CFT073	3588877	3718063	ns	out	i02_3579	/	CDS
CFT073	3590882	3720068	s	out	i02_3581	/	CDS
clone D	3601227	3730413	ns	out	i02_3593	yraO	CDS
CFT073	3603303	3732489	ns	out	i02_3596	yraR	CDS
CFT073	3638598	3767785	s	out	i02_3631	ispB	CDS
clone D	3638601	3767788	s	out	i02_3631	ispB	CDS
CFT073	3638947	3768134	ns	out	i02_3631	ispB	CDS
CFT073	3652545	3781732	nc	out	intergenic		

Lineage	Clone D site	CFT 073 site	mutation type[a]	recomb	Gene	Gene name	Type
CFT073	3658455	3787642	nc	out	intergenic		
CFT073	3667542	3796729	ns	out	i02_3662	nanT	CDS
CFT073	3669095	3798282	ns	out	i02_3663	nanA	CDS
clone D	3671503	3800690	ns	out	i02_3666	sspA	CDS
CFT073	3696116	3825303	ns	out	i02_3690	yhdA	CDS
clone D	3696462	3825649	ns	out	i02_3690	yhdA	CDS
CFT073	3699268	3828455	nc	out	intergenic		
CFT073	3700920	3830107	ns	out	i02_3694	accC	CDS
clone D	3704606	3833793	s	out	i02_3698	/	CDS
CFT073	3710481	3839665	ns	out	i02_3705	panF	CDS
CFT073	3716028	3845212	ns	out	i02_3712	envR	CDS
CFT073	3717679	3846863	ns	out	i02_3714	acrF	CDS
CFT073	3726285	3855223	nc	out	intergenic		
CFT073	3726400	3855338	nc	rec	23s rRNA	rrl	rRNA
CFT073	3726403	3855341	nc	rec	23s rRNA	rrl	rRNA
clone D	3726406	3855344	nc	rec	23s rRNA	rrl	rRNA
CFT073	3726408	3855346	nc	rec	23s rRNA	rrl	rRNA
CFT073	3727656	3856593	nc	rec	23s rRNA	rrl	rRNA
clone D	3727657	3856594	nc	rec	23s rRNA	rrl	rRNA
CFT073	3727698	3856635	nc	rec	23s rRNA	rrl	rRNA
CFT073	3727974	3856911	nc	rec	23s rRNA	rrl	rRNA
CFT073	3727975	3856912	nc	rec	23s rRNA	rrl	rRNA
CFT073	3727984	3856921	nc	rec	23s rRNA	rrl	rRNA
CFT073	3727985	3856922	nc	rec	23s rRNA	rrl	rRNA
CFT073	3727993	3856930	nc	rec	23s rRNA	rrl	rRNA
CFT073	3728026	3856963	nc	rec	23s rRNA	rrl	rRNA
CFT073	3728032	3856970	nc	rec	23s rRNA	rrl	rRNA
CFT073	3728651	3857589	nc	rec	23s rRNA	rrl	rRNA
CFT073	3728653	3857591	nc	rec	23s rRNA	rrl	rRNA
CFT073	3728654	3857592	nc	rec	23s rRNA	rrl	rRNA
CFT073	3728655	3857593	nc	rec	23s rRNA	rrl	rRNA
CFT073	3728660	3857598	nc	rec	23s rRNA	rrl	rRNA
clone D	3728845	3857784	nc	rec	23s rRNA	rrl	rRNA
CFT073	3728846	3857785	nc	rec	23s rRNA	rrl	rRNA
clone D	3728886	3857825	nc	rec	23s rRNA	rrl	rRNA
CFT073	3728938	3857877	nc	rec	23s rRNA	rrl	rRNA
CFT073	3729119	3858058	nc	rec	23s rRNA	rrl	rRNA
clone D	3729224	3858165	nc	rec	intergenic		
clone D	3729225	3858166	nc	rec	intergenic		
clone D	3729271	3858201	nc	rec	intergenic		
clone D	3731286	3860228	nc	out	intergenic		
CFT073	3734598	3863540	ns	out	i02_3727	smg	CDS
clone D	3744679	3873621	ns	out	i02_3741	rpsM	CDS
clone D	3784618	3913560	ns	out	i02_3799	yheS	CDS
CFT073	3854455	3983398	ns	out	i02_3868	/	CDS
CFT073	3863638	3992581	ns	out	i02_3879	/	CDS
CFT073	3892161	4021104	nc	out	intergenic		
CFT073	3922606	4051555	ns	out	i02_3937	yhhT	CDS
clone D	3928972	4057921	ns	out	i02_3946	/	CDS
CFT073	3930175	4059124	ns	out	i02_3948	/	CDS
clone D	3935166	4064114	ns	out	i02_3954	yhhJ	CDS

Lineage	Clone D site	CFT 073 site	mutation type[a]	recomb	Gene	Gene name	Type
CFT073	3957995	4086943	s	out	i02_3977	chuA	CDS
clone D	3961763	4090711	ns	out	i02_3982	chuW	CDS
CFT073	3961856	4090804	ns	out	i02_3982	chuW	CDS
clone D	3975179	4104127	ns	out	i02_3996	yhiX	pseudogene
clone D	3985397	4114345	ns	out	i02_4004	yhjE	CDS
CFT073	4018418	4147366	s	rec	i02_4031	/	CDS
CFT073	4018423	4147371	ns	rec	i02_4031	/	CDS
CFT073	4018508	4147456	nc	rec	intergenic		
clone D	4018509	4147457	nc	rec	intergenic		
CFT073	4018510	4147458	nc	rec	intergenic		
CFT073	4018511	4147459	nc	rec	intergenic		
CFT073	4018512	4147460	nc	rec	intergenic		
clone D	4018536	4147484	nc	rec	intergenic		
CFT073	4034206	4163154	s	rec	i02_4047	/	CDS
CFT073	4034217	4163165	s	rec	i02_4047	/	CDS
CFT073	4034221	4163169	s	rec	i02_4047	/	CDS
CFT073	4034252	4163200	ns	rec	i02_4047	/	CDS
CFT073	4034303	4163251	ns	rec	i02_4047	/	CDS
clone D	4044235	4173183	ns	out	i02_4056	xylR	CDS
clone D	4055331	4184279	ns	out	i02_4067	yiaN	CDS
CFT073	4059433	4188381	ns	out	i02_4071	sgbU	CDS
clone D	4061881	4190829	ns	out	i02_4073	aldB	CDS
clone D	4068252	4197200	s	out	i02_4078	yibF	CDS
clone D	4071641	4200589	ns	out	i02_4081	mtlA	CDS
clone D	4072205	4201153	ns	out	i02_4081	mtlA	CDS
CFT073	4091151	4220099	ns	out	i02_4101	yibO	CDS
clone D	4094205	4223153	ns	out	i02_4103	yibQ	CDS
CFT073	4102115	4231063	s	out	i02_4110	waaL	CDS
CFT073	4102133	4231081	ns	out	i02_4110	waaL	CDS
clone D	4118049	4246997	ns	out	i02_4129	rph	CDS
CFT073	4119400	4248348	ns	out	i02_4130	yicC	CDS
?	4141078	4270026	nc	out	intergenic		CDS
CFT073	4145453	4274403	nc	rec	intergenic		
CFT073	4145454	4274404	nc	rec	intergenic		
CFT073	4145455	4274405	nc	rec	intergenic		
CFT073	4145457	4274407	nc	rec	intergenic		
CFT073	4145476	4274426	nc	rec	intergenic		
CFT073	4145484	4274434	nc	rec	intergenic		
clone D	4145489	4274438	nc	rec	intergenic		
clone D	4145491	4274440	nc	rec	intergenic		
clone D	4145505	4274454	nc	rec	intergenic		
clone D	4145514	4274463	nc	rec	intergenic		
clone D	4145515	4274464	nc	rec	intergenic		
clone D	4145519	4274468	nc	rec	intergenic		
clone D	4145523	4274472	nc	rec	intergenic		
CFT073	4145529	4274478	nc	rec	intergenic		
clone D	4145540	4274489	nc	rec	intergenic		
clone D	4145547	4274496	nc	rec	intergenic		
clone D	4145551	4274500	nc	rec	intergenic		
clone D	4145552	4274501	nc	rec	intergenic		
clone D	4145561	4274510	nc	rec	intergenic		

Lineage	Clone D site	CFT 073 site	mutation type[a]	recomb	Gene	Gene name	Type
clone D	4145562	4274511	nc	rec	intergenic		
CFT073	4145568	4274517	nc	rec	intergenic		
clone D	4145573	4274522	nc	rec	intergenic		
clone D	4145575	4274524	nc	rec	intergenic		
clone D	4145578	4274527	nc	rec	intergenic		
clone D	4145586	4274535	nc	rec	intergenic		
clone D	4145587	4274536	nc	rec	intergenic		
clone D	4145596	4274544	nc	rec	intergenic		
clone D	4145599	4274547	nc	rec	intergenic		
clone D	4145605	4274553	nc	rec	intergenic		
clone D	4145621	4274569	nc	rec	intergenic		
clone D	4145623	4274571	nc	rec	intergenic		
clone D	4145634	4274582	nc	rec	intergenic		
clone D	4145644	4274592	nc	rec	intergenic		
clone D	4145647	4274595	nc	rec	intergenic		
clone D	4145654	4274602	nc	rec	intergenic		
clone D	4145655	4274603	nc	rec	intergenic		
clone D	4145665	4274613	nc	rec	intergenic		
clone D	4145670	4274618	nc	rec	intergenic		
clone D	4145679	4274627	nc	rec	intergenic		
clone D	4145697	4274645	nc	rec	intergenic		
clone D	4145723	4274672	nc	rec	intergenic		
clone D	4145724	4274673	nc	rec	intergenic		
clone D	4145728	4274677	nc	rec	intergenic		
clone D	4145758	4274707	s	rec	i02_4154	intC	CDS
clone D	4145759	4274708	s	rec	i02_4154	intC	CDS
clone D	4145768	4274717	ns	rec	i02_4154	intC	CDS
clone D	4145770	4274719	ns	rec	i02_4154	intC	CDS
clone D	4145785	4274734	s	rec	i02_4154	intC	CDS
clone D	4145788	4274737	s	rec	i02_4154	intC	CDS
clone D	4145791	4274740	s	rec	i02_4154	intC	CDS
clone D	4145797	4274746	ns	rec	i02_4154	intC	CDS
clone D	4145800	4274749	s	rec	i02_4154	intC	CDS
clone D	4145812	4274761	s	rec	i02_4154	intC	CDS
clone D	4145819	4274768	ns	rec	i02_4154	intC	CDS
clone D	4145836	4274785	s	rec	i02_4154	intC	CDS
clone D	4145846	4274795	ns	rec	i02_4154	intC	CDS
clone D	4145857	4274806	s	rec	i02_4154	intC	CDS
clone D	4145869	4274818	s	rec	i02_4154	intC	CDS
clone D	4145884	4274833	s	rec	i02_4154	intC	CDS
clone D	4145893	4274842	s	rec	i02_4154	intC	CDS
clone D	4145905	4274854	s	rec	i02_4154	intC	CDS
clone D	4145917	4274866	s	rec	i02_4154	intC	CDS
clone D	4145935	4274884	s	rec	i02_4154	intC	CDS
clone D	4145941	4274890	s	rec	i02_4154	intC	CDS
clone D	4145947	4274896	s	rec	i02_4154	intC	CDS
clone D	4145972	4274921	ns	rec	i02_4154	intC	CDS
clone D	4145974	4274923	ns	rec	i02_4154	intC	CDS
clone D	4145977	4274926	s	rec	i02_4154	intC	CDS
clone D	4145980	4274929	s	rec	i02_4154	intC	CDS
clone D	4145986	4274935	s	rec	i02_4154	intC	CDS

Lineage	Clone D site	CFT 073 site	mutation type[a]	recomb	Gene	Gene name	Type
clone D	4145995	4274944	s	rec	i02_4154	intC	CDS
clone D	4145998	4274947	s	rec	i02_4154	intC	CDS
clone D	4146010	4274959	s	rec	i02_4154	intC	CDS
clone D	4146027	4274976	ns	rec	i02_4154	intC	CDS
clone D	4146038	4274987	s	rec	i02_4154	intC	CDS
CFT073	4146041	4274990	ns	rec	i02_4154	intC	CDS
clone D	4146046	4274995	s	rec	i02_4154	intC	CDS
clone D	4146049	4274998	s	rec	i02_4154	intC	CDS
clone D	4146058	4275007	s	rec	i02_4154	intC	CDS
clone D	4146059	4275008	ns	rec	i02_4154	intC	CDS
clone D	4146070	4275019	s	rec	i02_4154	intC	CDS
clone D	4146076	4275025	s	rec	i02_4154	intC	CDS
clone D	4146082	4275031	s	rec	i02_4154	intC	CDS
clone D	4146094	4275043	s	rec	i02_4154	intC	CDS
clone D	4146124	4275073	s	rec	i02_4154	intC	CDS
clone D	4146127	4275076	s	rec	i02_4154	intC	CDS
clone D	4146142	4275091	s	rec	i02_4154	intC	CDS
clone D	4146145	4275094	s	rec	i02_4154	intC	CDS
clone D	4146157	4275106	s	rec	i02_4154	intC	CDS
clone D	4146160	4275109	s	rec	i02_4154	intC	CDS
clone D	4146175	4275124	s	rec	i02_4154	intC	CDS
clone D	4146181	4275130	s	rec	i02_4154	intC	CDS
clone D	4146193	4275142	s	rec	i02_4154	intC	CDS
clone D	4146194	4275143	s	rec	i02_4154	intC	CDS
clone D	4146211	4275160	s	rec	i02_4154	intC	CDS
clone D	4146214	4275163	s	rec	i02_4154	intC	CDS
clone D	4146220	4275169	s	rec	i02_4154	intC	CDS
clone D	4146226	4275175	s	rec	i02_4154	intC	CDS
clone D	4146232	4275181	s	rec	i02_4154	intC	CDS
clone D	4146256	4275205	s	rec	i02_4154	intC	CDS
clone D	4146271	4275220	s	rec	i02_4154	intC	CDS
clone D	4146292	4275241	s	rec	i02_4154	intC	CDS
clone D	4146299	4275248	s	rec	i02_4154	intC	CDS
clone D	4146301	4275250	s	rec	i02_4154	intC	CDS
clone D	4146304	4275253	s	rec	i02_4154	intC	CDS
clone D	4146316	4275265	s	rec	i02_4154	intC	CDS
clone D	4146319	4275268	s	rec	i02_4154	intC	CDS
clone D	4146334	4275283	s	rec	i02_4154	intC	CDS
clone D	4146337	4275286	s	rec	i02_4154	intC	CDS
clone D	4146364	4275313	s	rec	i02_4154	intC	CDS
clone D	4146370	4275319	s	rec	i02_4154	intC	CDS
clone D	4146379	4275328	s	rec	i02_4154	intC	CDS
clone D	4146391	4275340	s	rec	i02_4154	intC	CDS
clone D	4146400	4275349	s	rec	i02_4154	intC	CDS
clone D	4146424	4275373	s	rec	i02_4154	intC	CDS
clone D	4146430	4275379	s	rec	i02_4154	intC	CDS
clone D	4146436	4275385	s	rec	i02_4154	intC	CDS
clone D	4146445	4275394	s	rec	i02_4154	intC	CDS
clone D	4146469	4275418	s	rec	i02_4154	intC	CDS
clone D	4146475	4275424	s	rec	i02_4154	intC	CDS
clone D	4146478	4275427	s	rec	i02_4154	intC	CDS

Lineage	Clone D site	CFT 073 site	mutation type[a]	recomb	Gene	Gene name	Type
clone D	4146487	4275436	s	rec	i02_4154	intC	CDS
clone D	4146493	4275442	s	rec	i02_4154	intC	CDS
clone D	4146505	4275454	s	rec	i02_4154	intC	CDS
clone D	4146511	4275460	s	rec	i02_4154	intC	CDS
clone D	4146527	4275476	s	rec	i02_4154	intC	CDS
clone D	4146529	4275478	s	rec	i02_4154	intC	CDS
clone D	4146550	4275499	s	rec	i02_4154	intC	CDS
clone D	4146562	4275511	s	rec	i02_4154	intC	CDS
clone D	4146565	4275514	s	rec	i02_4154	intC	CDS
clone D	4146568	4275517	s	rec	i02_4154	intC	CDS
clone D	4146586	4275535	s	rec	i02_4154	intC	CDS
clone D	4146589	4275538	s	rec	i02_4154	intC	CDS
clone D	4146595	4275544	s	rec	i02_4154	intC	CDS
clone D	4146600	4275549	ns	rec	i02_4154	intC	CDS
clone D	4146604	4275553	s	rec	i02_4154	intC	CDS
clone D	4146610	4275559	s	rec	i02_4154	intC	CDS
clone D	4146622	4275571	s	rec	i02_4154	intC	CDS
clone D	4146625	4275574	s	rec	i02_4154	intC	CDS
clone D	4146640	4275589	s	rec	i02_4154	intC	CDS
clone D	4146643	4275592	s	rec	i02_4154	intC	CDS
clone D	4146655	4275604	s	rec	i02_4154	intC	CDS
clone D	4146661	4275610	s	rec	i02_4154	intC	CDS
clone D	4146673	4275622	s	rec	i02_4154	intC	CDS
clone D	4146676	4275625	s	rec	i02_4154	intC	CDS
clone D	4146679	4275628	s	rec	i02_4154	intC	CDS
clone D	4146691	4275640	s	rec	i02_4154	intC	CDS
clone D	4146692	4275641	ns	rec	i02_4154	intC	CDS
clone D	4146697	4275646	s	rec	i02_4154	intC	CDS
clone D	4146700	4275649	s	rec	i02_4154	intC	CDS
clone D	4146703	4275652	s	rec	i02_4154	intC	CDS
clone D	4146706	4275655	s	rec	i02_4154	intC	CDS
clone D	4146715	4275664	s	rec	i02_4154	intC	CDS
clone D	4146724	4275673	s	rec	i02_4154	intC	CDS
clone D	4146727	4275676	s	rec	i02_4154	intC	CDS
clone D	4146730	4275679	s	rec	i02_4154	intC	CDS
clone D	4146733	4275682	s	rec	i02_4154	intC	CDS
clone D	4146736	4275685	s	rec	i02_4154	intC	CDS
clone D	4146749	4275698	s	rec	i02_4154	intC	CDS
clone D	4146751	4275700	s	rec	i02_4154	intC	CDS
clone D	4146757	4275706	s	rec	i02_4154	intC	CDS
clone D	4146766	4275715	s	rec	i02_4154	intC	CDS
clone D	4146779	4275728	ns	rec	i02_4154	intC	CDS
clone D	4146790	4275739	s	rec	i02_4154	intC	CDS
clone D	4146793	4275742	s	rec	i02_4154	intC	CDS
clone D	4146796	4275745	s	rec	i02_4154	intC	CDS
clone D	4146807	4275756	ns	rec	i02_4154	intC	CDS
?	4163785	4341851	nc	rec	intergenic		
clone D	4163807	4341873	nc	rec	intergenic		
clone D	4163808	4341874	nc	rec	intergenic		
?	4163809	4341875	nc	rec	intergenic		
CFT073	4163816	4341882	nc	rec	intergenic		

Lineage	Clone D site	CFT 073 site	mutation type[a]	recomb	Gene	Gene name	Type
clone D	4163819	4341885	nc	rec	intergenic		
clone D	4163826	4341892	nc	rec	intergenic		
?	4163828	4341894	nc	rec	intergenic		
CFT073	4163836	4341902	nc	rec	intergenic		
CFT073	4163838	4341904	nc	rec	intergenic		
CFT073	4163845	4341911	nc	rec	intergenic		
CFT073	4163846	4341912	nc	rec	intergenic		
CFT073	4163847	4341913	nc	rec	intergenic		
CFT073	4163850	4341916	nc	rec	intergenic		
CFT073	4163853	4341919	nc	rec	intergenic		
CFT073	4164317	4342383	s	out	i02_4173	/	CDS
CFT073	4173011	4351077	ns	out	i02_4182	uhpT	CDS
CFT073	4186694	4364760	s	out	i02_4195	yidK	CDS
clone D	4193039	4371105	ns	out	i02_4204	yidS	CDS
CFT073	4199513	4377580	ns	out	i02_4210	/	CDS
CFT073	4208516	4386584	ns	out	i02_4220	rnpA	CDS
CFT073	4232942	4411010	ns	rec	i02_4244	pstC	CDS
CFT073	4233021	4411089	ns	rec	i02_4244	pstC	CDS
CFT073	4233031	4411099	s	rec	i02_4244	pstC	CDS
CFT073	4233035	4411103	ns	rec	i02_4244	pstC	CDS
CFT073	4247246	4425314	s	out	i02_4260	gidA	CDS
clone D	4265908	4443976	nc	rec	16s rRNA	rrs	rRNA
CFT073	4266132	4444201	nc	rec	16s rRNA	rrs	rRNA
CFT073	4266214	4444283	nc	rec	16s rRNA	rrs	rRNA
CFT073	4266223	4444292	nc	rec	16s rRNA	rrs	rRNA
CFT073	4266231	4444300	nc	rec	16s rRNA	rrs	rRNA
CFT073	4266250	4444319	nc	rec	16s rRNA	rrs	rRNA
CFT073	4266255	4444324	nc	rec	16s rRNA	rrs	rRNA
CFT073	4266257	4444326	nc	rec	16s rRNA	rrs	rRNA
CFT073	4266261	4444330	nc	rec	16s rRNA	rrs	rRNA
CFT073	4266262	4444331	nc	rec	16s rRNA	rrs	rRNA
CFT073	4266328	4444397	nc	rec	intergenic		
CFT073	4266360	4444429	nc	rec	intergenic		
CFT073	4266598	4444667	nc	rec	intergenic		
CFT073	4266645	4444714	nc	rec	intergenic		
CFT073	4266750	4444819	nc	rec	23s rRNA	rrl	rRNA
CFT073	4266931	4445000	nc	rec	23s rRNA	rrl	rRNA
CFT073	4266952	4445021	nc	rec	23s rRNA	rrl	rRNA
clone D	4266983	4445052	nc	rec	23s rRNA	rrl	rRNA
CFT073	4267209	4445280	nc	rec	23s rRNA	rrl	rRNA
CFT073	4267837	4445908	nc	rec	23s rRNA	rrl	rRNA
CFT073	4267843	4445915	nc	rec	23s rRNA	rrl	rRNA
CFT073	4267876	4445948	nc	rec	23s rRNA	rrl	rRNA
CFT073	4267884	4445956	nc	rec	23s rRNA	rrl	rRNA
CFT073	4267885	4445957	nc	rec	23s rRNA	rrl	rRNA
CFT073	4267894	4445966	nc	rec	23s rRNA	rrl	rRNA
CFT073	4267895	4445967	nc	rec	23s rRNA	rrl	rRNA
CFT073	4268171	4446243	nc	rec	23s rRNA	rrl	rRNA
CFT073	4268213	4446285	nc	rec	23s rRNA	rrl	rRNA
CFT073	4269751	4447824	nc	out	intergenic		
CFT073	4270509	4448582	s	out	i02_4276	yifA	CDS

Lineage	Clone D site	CFT 073 site	mutation type[a]	recomb	Gene	Gene name	Type
clone D	4289827	4467901	ns	out	i02_4296	/	CDS
CFT073	4323599	4501680	s	out	i02_4327	uvrD	CDS
CFT073	4383374	4561456	nc	rec	16s rRNA	rrs	rRNA
clone D	4383463	4561545	nc	rec	16s rRNA	rrs	rRNA
CFT073	4384669	4562751	nc	rec	intergenic		
clone D	4384675	4562757	nc	rec	intergenic		
CFT073	4385091	4563256	nc	rec	23s rRNA	rrl	rRNA
CFT073	4385272	4563437	nc	rec	23s rRNA	rrl	rRNA
CFT073	4386383	4564552	nc	rec	23s rRNA	rrl	rRNA
CFT073	4386427	4564596	nc	rec	23s rRNA	rrl	rRNA
CFT073	4386512	4564681	nc	rec	23s rRNA	rrl	rRNA
CFT073	4386554	4564723	nc	rec	23s rRNA	rrl	rRNA
CFT073	4386875	4565044	nc	rec	23s rRNA	rrl	rRNA
clone D	4386877	4565046	nc	rec	23s rRNA	rrl	rRNA
CFT073	4386880	4565049	nc	rec	23s rRNA	rrl	rRNA
clone D	4412393	4590561	s	out	i02_4418	yihV	CDS
CFT073	4414684	4592852	s	out	i02_4422	/	CDS
CFT073	4436307	4614475	ns	out	i02_4446	rhaA	CDS
clone D	4436680	4614848	s	out	i02_4446	rhaA	CDS
CFT073	4450344	4628512	s	out	i02_4462	sbp	CDS
clone D	4456745	4634913	ns	out	i02_4470	glpX	CDS
clone D	4466828	4644996	s	out	i02_4480	priA	CDS
clone D	4484996	4663164	s	out	i02_4493	yijE	CDS
CFT073	4516087	4694255	s	out	i02_4518	yijD	CDS
CFT073	4517164	4695332	ns	out	i02_4519	trmA	CDS
CFT073	4519756	4697924	s	out	i02_4521	murI	CDS
CFT073	4520807	4698975	nc	rec	intergenic		
clone D	4520842	4699009	nc	rec	intergenic		
clone D	4520877	4699045	nc	rec	intergenic		
clone D	4520878	4699047	nc	rec	intergenic		
CFT073	4520883	4699052	nc	rec	intergenic		
CFT073	4520973	4699142	nc	rec	16s rRNA	rrs	rRNA
CFT073	4520982	4699151	nc	rec	16s rRNA	rrs	rRNA
CFT073	4521157	4699326	nc	rec	16s rRNA	rrs	rRNA
clone D	4522043	4700212	nc	rec	16s rRNA	rrs	rRNA
CFT073	4522236	4700409	nc	rec	16s rRNA	rrs	rRNA
CFT073	4522289	4700467	nc	rec	16s rRNA	rrs	rRNA
CFT073	4522456	4700634	nc	rec	intergenic		
CFT073	4522859	4701031	nc	rec	intergenic		
CFT073	4522964	4701136	nc	rec	23s rRNA	rrl	rRNA
CFT073	4523145	4701317	nc	rec	23s rRNA	rrl	rRNA
clone D	4523197	4701369	nc	rec	23s rRNA	rrl	rRNA
CFT073	4523237	4701409	nc	rec	23s rRNA	rrl	rRNA
clone D	4523238	4701410	nc	rec	23s rRNA	rrl	rRNA
CFT073	4523423	4701596	nc	rec	23s rRNA	rrl	rRNA
CFT073	4524051	4702224	nc	rec	23s rRNA	rrl	rRNA
CFT073	4524057	4702231	nc	rec	23s rRNA	rrl	rRNA
CFT073	4524090	4702264	nc	rec	23s rRNA	rrl	rRNA
CFT073	4524098	4702272	nc	rec	23s rRNA	rrl	rRNA
CFT073	4524099	4702273	nc	rec	23s rRNA	rrl	rRNA
CFT073	4524108	4702282	nc	rec	23s rRNA	rrl	rRNA

Lineage	Clone D site	CFT 073 site	mutation type[a]	recomb	Gene	Gene name	Type
CFT073	4524109	4702283	nc	rec	23s rRNA	rrl	rRNA
CFT073	4524385	4702559	nc	rec	23s rRNA	rrl	rRNA
CFT073	4524427	4702601	nc	rec	23s rRNA	rrl	rRNA
clone D	4524486	4702660	nc	rec	23s rRNA	rrl	rRNA
clone D	4524750	4702923	nc	rec	23s rRNA	rrl	rRNA
clone D	4525066	4703238	nc	rec	23s rRNA	rrl	rRNA
clone D	4526087	4704259	nc	out	intergenic		
clone D	4561232	4739404	nc	rec	16s rRNA	rrs	rRNA
clone D	4561235	4739407	nc	rec	16s rRNA	rrs	rRNA
clone D	4561246	4739418	nc	rec	16s rRNA	rrs	rRNA
CFT073	4561786	4739958	nc	rec	16s rRNA	rrs	rRNA
clone D	4562418	4740590	nc	rec	16s rRNA	rrs	rRNA
clone D	4562781	4740953	nc	rec	intergenic		
CFT073	4562983	4741156	nc	rec	intergenic		
CFT073	4563030	4741203	nc	rec	intergenic		
CFT073	4563135	4741308	nc	rec	23s rRNA	rrl	rRNA
CFT073	4563392	4741565	nc	rec	23s rRNA	rrl	rRNA
CFT073	4564222	4742395	nc	rec	23s rRNA	rrl	rRNA
CFT073	4564228	4742402	nc	rec	23s rRNA	rrl	rRNA
CFT073	4564261	4742435	nc	rec	23s rRNA	rrl	rRNA
CFT073	4564269	4742443	nc	rec	23s rRNA	rrl	rRNA
CFT073	4564270	4742444	nc	rec	23s rRNA	rrl	rRNA
CFT073	4564279	4742453	nc	rec	23s rRNA	rrl	rRNA
CFT073	4564280	4742454	nc	rec	23s rRNA	rrl	rRNA
clone D	4564657	4742831	nc	rec	23s rRNA	rrl	rRNA
CFT073	4569851	4748027	ns	out	i02_4561	aceB	CDS
CFT073	4585244	4763420	s	out	i02_4573	/	CDS
clone D	4590689	4768865	ns	out	i02_4579	lysC	CDS
CFT073	4602361	4780537	nc	out	intergenic		
CFT073	4622406	4800582	s	out	i02_4609	/	CDS
CFT073	4626099	4804275	s	out	i02_4613	alr	CDS
clone D	4627342	4805518	s	out	i02_4614	/	CDS
clone D	4627905	4806081	s	out	i02_4615	/	CDS
CFT073	4631749	4809925	s	out	i02_4617	sucA	CDS
CFT073	4633577	4811753	ns	out	i02_4619	/	CDS
clone D	4655252	4833472	s	out	i02_4641	/	CDS
CFT073	4658923	4837143	ns	out	i02_4645	actP	CDS
clone D	4667340	4845560	s	out	i02_4654	nrfE	CDS
clone D	4668303	4846523	ns	out	i02_4655	nrfF	CDS
clone D	4671386	4849606	nc	out	intergenic		
clone D	4681579	4859788	s	out	i02_4668	yjcP	CDS
CFT073	4685538	4863747	nc	out	intergenic		
CFT073	4685730	4863939	s	out	i02_4672	yjcS	CDS
clone D	4686261	4864470	ns	out	i02_4672	yjcS	CDS
CFT073	4695657	4873866	ns	rec	i02_4681	phnP	CDS
CFT073	4695924	4874133	nc	rec	intergenic		
CFT073	4696327	4874536	ns	rec	i02_4682	phnO	CDS
CFT073	4696345	4874554	ns	rec	i02_4682	phnO	CDS
CFT073	4696372	4874581	ns	rec	i02_4683	phnN	CDS
CFT073	4696409	4874618	ns	rec	i02_4683	phnN	CDS
clone D	4706808	4885017	ns	out	i02_4698	phnA	CDS

Lineage	Clone D site	CFT 073 site	mutation type[a]	recomb	Gene	Gene name	Type
clone D	4727547	4905756	ns	out	i02_4714	/	CDS
CFT073	4739802	4918011	ns	out	i02_4727	cadC	CDS
clone D	4753727	4984072	ns	out	i02_4740	yjeK	CDS
CFT073	4757619	4987964	ns	out	i02_4747	frdD	CDS
CFT073	4758195	4988540	s	out	i02_4749	frdB	CDS
CFT073	4759117	4989462	s	out	i02_4750	frdA	CDS
CFT073	4764748	4995093	ns	out	i02_4753	yjeP	CDS
CFT073	4767795	4998140	ns	out	i02_4755	psd	CDS
clone D	4790240	5020584	s	out	i02_4777	yjfM	CDS
CFT073	4790974	5021317	s	out	i02_4778	yjfC	CDS
CFT073	4823568	5053911	s	out	i02_4816	cysQ	CDS
CFT073	4825471	5055814	s	out	i02_4819	ytfL	CDS
CFT073	4831334	5061677	ns	out	i02_4822	ytfN	CDS
CFT073	4864851	5095194	nc	out	intergenic		
clone D	4868624	5098967	ns	out	i02_4861	/	CDS
CFT073	4883853	5114197	ns	out	i02_4873	yjgB	CDS
CFT073	4939176	5131503	ns	out	i02_4905	/	CDS
CFT073	4953849	5146176	nc	out	intergenic		
CFT073	4963811	5156138	ns	out	i02_4931	yjiJ	CDS
clone D	4964151	5156478	s	out	i02_4931	yjiJ	CDS
CFT073	4967637	5159964	ns	out	i02_4936	yjiN	CDS
CFT073	5003843	5196172	s	out	i02_4966	rsmC	CDS
CFT073	5013999	5206328	s	out	i02_4978	deoC	CDS
CFT073	5015018	5207347	s	out	i02_4979	deoA	CDS
CFT073	5019370	5212411	ns	out	i02_4982	yjjJ	CDS

[a]nc: in non-coding region; ns, non-synonymous; s, synonymous
