# Supplementary material for: Rates of Mutation and Host Transmission for an Escherichia coli Clone over 3 Years
Source: PLoS One. 2011 Oct 27;6(10):e26907. doi: 10.1371/journal.pone.0026907 (PMC3203180; doi:10.1371/journal.pone.0026907)
Supplement: Table S8 — Allocation of SNPs to lineages by virtual outgroup analysis. The E. coli/Shigella genomes used for the analysis are shown, with details of the base in outgroup genomes and genomes under analysis, and also the final allocation and a measure of support level for that allocation. (PDF) [file pone.0026907.s009.pdf]

Table S8 Allocation of SNPs to lineages by virtual outgroup analysis. The *E. coli* /*Shigella* genomes used for the analysis are shown, with details of the base in outgroup genomes and genomes under analysis, and also the final allocation and a measure of support level for that allocation.

Clone D base	Clone D site	CFT 073 base	CFT 073 site	mutation type[a]	Inferred ancestral base[b]	lineage inferred to mutate	Support level[c]	ED1a[d]	536	S88	APEC O1	UTI89	E2348/69	ATCC8739	K-12	HS	SMS3-5	IAI39	E24377A	SE11	55989	IAI1	Sakai	UMN026	B4 Sb227	F5 8401	SS Ss046	D1 Sd197	B18 BS512	F2a 2457T	F2a 301
c	7482	t	8194	s	t	clone D	++++	t	t	t	t	t	t	t	t	t	t	t	t	t	t	t	t	t	t	t	t	t	t	t	t
c	37232	t	37944	s	c	CFT073	++	c	c	c	c	c	c	c	c	c	c	c	c	c	c	c	c	c		c	c	c		c	c
g	68282	t	69705	ns	t	clone D	++++	t	t	t	t	t	t	t	t	t	t	t	t	t	t	t	t	t	t	t	t	t	t	t	t
a	141616	g	145065	ns	g	clone D	++++	g	g	g	g	g	g	g	g	g	g	g	g	g	g	g	g	g	g	g	g	g	g	g	g
c	142637	t	146086	s	c	CFT073	++++	c	c	c	c	c	c	c	c	c	c	c	c	c	c	c	c	c	c	c	c	c	c	c	c
t	148054	a	151497	nc	t	CFT073	++	t	t	t	t	t	t	t	t	t	t	t	t	t	t	t	t	t	t	c	c	t	t	c	c
g	165170	a	168602	s	a	clone D	++++	a	a	a	a	a	a	a	a	a	a	a	a	a	a	a	a	a	a	a	a	a	a	a	a
t	166117	c	169549	ns	c	clone D	++++	c	c	c	c	c	c	c	c	c	c	c	c	c	c	c	c	c	c	c	c	c	c	c	c
a	175531	g	178963	ns	g	clone D	++++	g	g	g	g	g		g	g	g	g	g	g	g	g	g	g	g	g	g	g	g	g	g	g
a	220289	t	224434	ns	a	CFT073	++++	a	a	a	a	a	a	a	a	a	a	a	a	a	a	a	a	a	a	a	a	a	a	a	a
c	224221	a	228366	ns	c	CFT073	++++	c	c	c	c	c	c	c	c	c	c	c	c	c	c	c	c	c	c	c	c	c	c	c	c
t	229285	c	233427	ns	c	clone D	++++	c	c	c	c	c	c	c	c	c	c	c	c	c	c	c	c	c	c	c	c	c	c	c	c
t	230078	c	234220	nc	c	clone D	++++	c	c	c	c	c	c	c	c	c	c	c	c	c	c	c	c	c	c	c	c	c	c	c	c
a	230842	g	234984	ns	a	CFT073	++++	a	a	a	a	a	a	a	a	a	a	a	a	a	a	a	a	a	a	a	a	a	a	a	a
a	230892	g	235034	s	g	clone D	++++	g	g	g	g	g	g	g	g	g	g	g	g	g	g	g	g	g	g	g	g	g	g	g	g
t	231008	c	235150	nc	c	clone D	++++	c	c	c	c	c	c	c	c	c	c	c	c	c	c	c	c	c	c	c	c	c	c	c	c
c	231133	a	235275	nc	c	CFT073	++	c	a	c	a	c	c	c	c	c	c	t	c	c	c	c	c	c	a	t	t	c	t	t	c
t	231226	c	235368	nc	c	clone D	++++	c	c	c	c	c	c	c	c	c	t	c	c	c	c	c	c	c	t	c	c	c	t	c	c
g	232045	a	236187	nc	g	CFT073	++++	g	g	g	a	g	g	g	a	g	g	a	g	g	g	g	a	g	g	g	a	a	g	g	g
g	232049	c	236191	nc	g	CFT073	++++	g	g	g	c	g	g	g	c	g	g	c	g	g	g	g	c	g	g	g	c	c	g	g	g
t	232053	c	236195	nc	t	CFT073	++++	t	t	t	c	t	t	t	c	t	t	c	t	t	t	t	c	t	t	t	c	g	t	t	t
a	232065	t	236206	nc	a	CFT073	++++	a	a	a	t	a	a	a	t	a	a	t	a	a	a	a	t	a	a	a	t	a	a	a	a
c	232081	t	236223	nc	c	CFT073	++++	c	c	c	t	c	c	c	t	c	c	t	c	c	c	c	t	c	c	c	t	t	c	c	c

Clone D base	Clone D site	CFT 073 base	CFT 073 site	mutation type[a]	Inferred ancestral base[b]	lineage inferred to mutate	Support level[c]	ED1a[d]	536	S88	APEC O1	UTI89	E2348/69	ATCC8739	K-12	HS	SMS3-5	IAI39	E24377A	SE11	55989	IAI1	Sakai	UMN026	B4 Sb227	F5 8401	SS Ss046	D1 Sd197	B18 BS512	F2a 2457T	F2a 301
a	232521	g	236911	nc	g	clone D	++++	g	g	g	g	g	g	g	g	g	g	g	g	g	g	g	g	g	g	g	g	g	g	g	g
a	232747	g	236988	nc	a	CFT073	++++	a	a	a	g	a	a	a	g	a	a	a	a	a	a	a	a	a	a		a	a	a	a	a
c	232846	t	237087	nc	t	clone D	++++	t	c	t	t	t	c	c	t	c	t	t	c	c	c	c	c	c	c	c	t	t	c	c	c
g	232849	a	237090	nc	a	clone D	++++	a	a	a	a	a	g	a	a	a	a	a	a	g	a	a	a	a	a	a	a	a	a	a	a
a	232891	g	237132	nc	a	CFT073	++	a	a	t	g	t	g	a	g	t	t	t	a	a	a	a	a	a	a	a	a	t	a	a	a
g	233001	a	237242	nc	g	CFT073	++++	g	a	g	a	g	g	a	a	a	a	a	a	a	a	a	a	a	a	a	a	a	a	a	a
t	233234	c	237475	nc	c	clone D	++++	c	c	c	c	c	c	c	c	c	c	t	c	t	c	t	c	c	t	t	c	c	t	t	t
a	233274	g	237515	nc	a	CFT073	++++	a	g	a	g	a	a	g	g	g	a	a	g	g	g	a	a	g	a	a	a	a	a	a	a
t	233275	c	237516	nc	c	clone D	++	c	c	t	c	t	t	c	c	c	c	c	c	c	c	t	c	c	t	c	t	t	t	c	c
c	233459	a	237700	nc	c	CFT073	++++	c	c	c	a	c	c	c	a	a	c	c	a	a	a	a	c	c	a	a	a	c	a	a	a
t	233763	c	238005	nc	t	CFT073	++++	t	t	t	c	t	t	t	c	t	t	t	t	t	t	t	t	c	t	t	t	c	t	t	t
a	234088	g	238330	nc	a	CFT073	++++	a	a	a	g	a	a	g	g	a	a	a	g	a	a	g	a	g	g	g	g	a	a	g	g
t	234094	c	238337	nc	t	CFT073	++++	t	t	t	c	t	t	c	c	t	t	t	c	t	t	c	t	c	c	c	c	t	t	c	c
g	234135	t	238378	nc	g	CFT073	++++	g	g	g	t	g	g	t	t	t	t	t	t	t	t	t	t	t	t	t	t	t	t	t	t
c	234136	a	238379	nc	c	CFT073	++	c	c	c	a	c	c	g	a	g	g	a	g	a	a	g	a	a	g	g	g	a	a	g	g
g	234145	t	238388	nc	g	CFT073	++	g	g	g	t	g	g	c	t	c	c	t	c	t	t	c	t	t	t	t	c	t	t	t	t
t	234146	a	238389	nc	t	CFT073	++++	t	t	t	a	t	t	a	a	a	a	a	a	a	a	a	a	a	a	a	a	a	a	a	a
c	234422	t	238665	nc	c	CFT073	++++	c	c	c	t	c	c	t	t	t	t	t	t	t	t	t	t	t	c	t	t	t	c	t	t
g	234464	a	238707	nc	g	CFT073	++++	g	g	g	a	g	g	a	a	a	a	a	a	a	a	a	a	a	a	a	a	a	a	a	a
a	234523	c	238766	nc	c	clone D	++++	c	c	c	c	c	c	c	c	c	c	c	c	c	c	c	c	c	c	c	c	c	c	c	c
t	234787	c	239029	nc	c	clone D	++	c	g	g	c	g	g	g	c	c	g	g	c	c	c	c	c	g	c	c	c	c	c	c	c
g	235123	a	239364	nc	g	CFT073	++	g	g	g	a	g	g	a	a	a	g	g	a	a	a	a	a	g	a	a	a	a	a	a	a
a	235720	g	239961	nc	a	CFT073	+	t	t	t	t	t	g	a	a	a	a	a	a	a	a	a	g	a	a	g	g	a	a	g	g
t	235919	c	240160	nc	c	clone D	++++	c	c	c	c	c	c	c	c	c	c	c	c	c	c	c	c	c	c	c	c	c	c	c	c
g	241720	t	245961	ns	g	CFT073	++++	g	g	t	t	t	g	g	g	g	g	g	g	t	g	g	g	g	g	g	g	g	g	g	g
g	262792	a	258602	ns	g	CFT073	++	g	a	g		a	a				g		g				a	g				g			
t	262803	c	258613	ns	c	clone D	++	c	c			c	c				c		t				c	c				c			
c	262805	t	258615	ns	c	CFT073	++	c	c			t	c				c		c				c	c				c			

Clone D base	Clone D site	CFT 073 base	CFT 073 site	mutation type[a]	Inferred ancestral base[b]	lineage inferred to mutate	Support level[c]	ED1a[d]	536	S88	APEC O1	UTI89	E2348/69	ATCC8739	K-12	HS	SMS3-5	IAI39	E24377A	SE11	55989	IAI1	Sakai	UMN026	B4 Sb227	F5 8401	SS Ss046	D1 Sd197	B18 BS512	F2a 2457T	F2a 301
a	262806	g	258616	ns	g	clone D	++	g	g			g	g				g		a				g	g				g			
c	262807	g	258617	ns	c	CFT073	++	c	c			g	c				c		c				c	c				c			
g	262828	a	258638	s	g	CFT073	++	g	g			a	g				a		g				g	a				a			
t	262832	g	258642	ns	t	CFT073	++	t	t			g	g				t		t				g	t				t			
g	262834	a	258644	s	g	CFT073	++	g	g			a	a				g		g				a	g				g			
a	262841	t	258651	ns	a	CFT073	++	a	a			t	a				a		a				a	a				a			
c	262843	a	258653	ns	c	CFT073	++	c	c			a	a				a		c				a	a				c			
g	262845	t	258655	ns	g	CFT073	++	g	g			t	t				g		g				t	g				g			
c	262855	t	258665	s	c	CFT073	++	c	c			t	c				c		c				c	c				c			
t	262897	c	258707	s	?	?	+/-	g	g			c	g				t		g				g	c				t			
a	262900	g	258710	s	a	CFT073	++	a	a			g	a				a		a				a	g				a			
a	262908	g	258718	s	g	clone D	+					g					a							g				g			
a	264168	g	259978	s	a	CFT073	++	a	a	g	g	a	a		g		a	g	g		g		g	g				-	g	g	g
g	264177	t	259987	ns	t	clone D	++	t	g	g	a	g	g		g		g	a	g		t		g	g				-	g	t	t
c	264179	t	259989	ns	t	clone D	++	t	c	c	c	c	c		c		c	c	c		t		c	c				-	c	t	t
g	264182	t	259992	ns	t	clone D	++	t	g	g	g	g	g		g		g	g	g		t		g	g				t	g	t	t
c	264183	t	259993	s	t	clone D	++	t	c	c	c	c	c		c		c	c	c		t		c	c				t	a	t	t
t	264209	g	260019	ns	g	clone D	++	g	t	g	t	g	g		g		g	g	g		g		t	g				t	g	g	g
t	264213	c	260023	s	c	clone D	++	c	t	t	t	t	c		t		c	t	t		c		t	t				t	t	c	c
t	264222	c	260032	nc	c	clone D	++	c	t	a	t	t	c		t		c	g	a		c		t	a				t	g	c	c
c	264229	t	260039	nc	t	clone D	++	t	c	t	-	c	t		t		t	t	t		a		-	t				c	t	a	a
t	264232	a	260042	nc	a	clone D	++	a	t	a	t	t	a		g		a	a	a		a		t	a				t	a	a	a
a	264240	g	260050	s	g	clone D	++	g	a	g	a	a	a		g		g	g	g		g		a	g				a	g	g	g
a	264243	g	260053	s	g	clone D	++	g	g	g	g	a	g		a		g	g	g		g		g	g				g	g	g	g
a	264415	g	260225	ns	a	CFT073	++	a	g	g	g	g	g		g		g	g	g		g		g	g				g	g	g	g
g	264417	a	260227	s	g	CFT073	++	g	a	a	a	a	a		a		a	a	a		a		a	a				a	a	a	a
a	264420	g	260230	s	a	CFT073	++	a	g	g	g	g	g		a		g	g	g		g		g	g				g	g	g	g
c	264426	t	260236	s	c	CFT073	++	c	t	t	t	t	t		a		t	t	t		t		t	t				t	t	t	t

Clone D base	Clone D site	CFT 073 base	CFT 073 site	mutation type[a]	Inferred ancestral base[b]	lineage inferred to mutate	Support level[c]	ED1a[d]	536	S88	APEC O1	UTI89	E2348/69	ATCC8739	K-12	HS	SMS3-5	IAI39	E24377A	SE11	55989	IAI1	Sakai	UMN026	B4 Sb227	F5 8401	SS Ss046	D1 Sd197	B18 BS512	F2a 2457T	F2a 301
g	264459	a	260269	s	g	CFT073	++	g	g	g	a	a	g		g		g	g	g		g		g	g				a	g	g	g
t	264462	g	260272	s	t	CFT073	++	t	t	t	t	g	t		g		t	t	t		t		g	t				g	t	t	t
g	264474	c	260284	s	g	CFT073	++	g	g	c	g	c	c		g		c	c	c		c		c	c				c	c	c	c
a	264477	g	260287	s	a	CFT073	++	a	a	g	a	g	g		g		g	g	g		g		g	g				g	g	g	g
c	264521	t	260331	ns	c	CFT073	++	c	c	c	c	t	c		c		c	c	c		c		c	c				t	c	c	c
a	264525	g	260335	ns	a	CFT073	++	a	a	g	g	g	g		a		a	g	g		g		g	g				g	g	g	g
t	264526	c	260336	ns	c	clone D	++	c	t	c	c	c	c		c		c	c	c		c		c	c				c	c	c	c
c	264552	a	260362	s	a	clone D	++	a	c	a	a	a	a		a		a	a	a		a		a	a				a	a	a	a
t	278428	a	274240	s	t	CFT073	+		t			t																			
c	281024	t	276836	s	c	CFT073	+		c			c																			
g	292352	c	288164	ns	g	CFT073	+		g										g		g										
t	310680	c	306465	s	c	clone D	+														c										
t	326514	c	322299	nc	?	?	+/-																								
g	326758	a	322543	nc	?	?	+/-																								
g	327001	t	322786	nc	?	?	+/-																								
t	327109	c	322894	nc	?	?	+/-																								
t	327152	c	322937	nc	?	?	+/-																								
a	327153	t	322938	nc	?	?	+/-																								
g	327245	t	323031	nc	?	?	+/-																								
t	327378	c	323164	nc	?	?	+/-																								
c	364934	a	363040	ns	c	CFT073	++++	c	c	c	c	c	c	c	c	c	c	c	c	c	c	c	c	c	c	c	c	c	c	c	c
g	364961	c	363067	ns	g	CFT073	++++	g	g	g	g	g	g	g	g	g	g	g	g	g	g	g	g	g	g	g	g	g	g	g	g
t	370492	c	368598	s	c	clone D	++++	c	c	c	c	c	c	c	c	c	c	c	c	c	c	c	c	c	c	c	c	c	c	c	c
t	371371	c	369477	s	c	clone D	++++	c	c	c	c	c	c	c	c	c	c	c	c	c	c	c	c	c	c	c	c	c	c	c	c
g	385561	c	383665	ns	c	clone D	++++	c	c	c	c	c	c	c	c		c	c	c	c	c	c	c	c	c						
c	387348	t	385452	ns	c	CFT073	++++	c	c	c	c	c	c	c	c		c	c	c	c	c	c	c	c	c						
c	388324	t	386428	ns	t	clone D	++++	t	t	t	t	t	t	t	t	t	t	t	t	t	t	t	t	t	t						
c	389023	t	387127	ns	c	CFT073	++++	c	c	c	c	c	c	c	c	c	c	c	c	c	c	c	c	c	c						

Clone D base	Clone D site	CFT 073 base	CFT 073 site	mutation type[a]	Inferred ancestral base[b]	lineage inferred to mutate	Support level[c]	ED1a[d]	536	S88	APEC O1	UTI89	E2348/69	ATCC8739	K-12	HS	SMS3-5	IAI39	E24377A	SE11	55989	IAI1	Sakai	UMN026	B4 Sb227	F5 8401	SS Ss046	D1 Sd197	B18 BS512	F2a 2457T	F2a 301
t	390877	c	388982	ns	t	CFT073	++	t	t	t	t	t	t				t	t					t	t							
a	390890	g	388995	ns	a	CFT073	++	a	a	a	a	a	a				a	a					a	a							
a	390901	g	389006	s	a	CFT073	++	a	a	a	a	a	a				a	a					a	a							
a	390930	g	389036	s	g	clone D	+		g	g	-	-	-				g	g					-	g							
c	390959	t	389066	s	c	CFT073	++		c	c	c	c	c				c	c					c	c							
g	390983	c	389089	ns	g	CFT073	+		c	c	g	g	g				c	c					g	c							
g	391040	a	389147	s	?	?	+/-		-	-	-	-	-				-	-					-	-							
a	391041	g	389148	s	g	clone D	++		g	g	g	g	g				g	g					g	g							
c	391059	g	389167	ns	g	clone D	++		g	g	g	g	g				g	g					g	g							
a	403397	g	401504	ns	a	CFT073	++	a	a	a	a	a	a			a	a	a	a	a			a	a			a		a		
a	418710	g	417089	s	a	CFT073	++		a	a	a	a	a	a	a	a	a	a	a	a	a	a	a	a		a					
g	423896	t	422275	nc	g	CFT073	++		g	g	g	g	g	a	a		g	g		g				g							
g	437807	t	436282	s	g	CFT073	++++	g	g	g	g	g	g	g	g	g	g	g	g	g	g	g	g	g							
a	450348	t	448823	ns	a	CFT073	++	a	a	a	a	a	a	a	a	a	a	a	a	a	a	a	a	a			a	t			
c	457462	t	455938	s	c	CFT073	++	c	c	c	c	c	c	c	c	c	c	c	c	c	c	c	c	c			c		c		
g	457464	t	455940	ns	g	CFT073	++	g	g	g	g	g	g	g	g	g	g	g	g	g	g	g	g	g			g		g		
a	462126	g	460602	ns	g	clone D	++++	g	g	g	g	g	g	g	g	g	g	g	g	g	g	g	g	g	g	g	g	g	g	g	g
g	464761	a	463237	ns	g	CFT073	++++	g	g	g	g	g	g	g	g	a	g	g	g	a	a	a	g	g	g	g	g	g	g	g	g
a	469174	g	467650	ns	a	CFT073	++		a	a	a	a	a	a	a	a	a	a	a	a	a	a	a	a	a		a	a	a		
t	483921	c	482397	ns	c	clone D	++++	c	c	c	c	c	c	c	c	c	c	c	c	c	c	c	c	c	c	c	c	c	c	c	c
t	488150	c	486626	s	c	clone D	++++	c	c	c	c	c	c	c	c	c	c	c	c	c	c	c	c	c	c	c	c	c	c	c	c
c	492766	t	491242	ns	c	CFT073	++++	c	c	c	c	c	c	c	c	c	c	c	c	c	c	c	c	c	c	c	c	c	c	c	c
c	494798	a	493274	nc	c	CFT073	++++	c	c	c	c	c	c	c	c	c	c	c	c	c	c	c	c	c	c	c	c	c	c	c	c
c	526368	t	524844	ns	t	clone D	++++	t	t	t	t	t	t	t	t	t	t	t	t	t	t	t	t	t	t	t	t		t	t	t
c	530872	t	529348	ns	t	clone D	++++	t	t	t	t	t	t	t	t	t	t	t	t	t	t	t	t	t	t	t	t	t	t	t	t
t	532814	c	531290	nc	c	clone D	++++	c	c	c	c	c	c	c	c	c	c	c	c	c	c	c	c	c	c	c	c	c	c	c	c
t	535100	g	533576	s	g	clone D	++++	g	g	g	g	g	g	g	g	g	g	g	g	g	g	g	g	g	g	g	g	g	g	g	g
t	535138	c	533614	ns	c	clone D	++++	c	c	c	c	c	c	c	c	c	c	c	c	c	c	c	c	c	c	c	c	c	c	c	c

Clone D base	Clone D site	CFT 073 base	CFT 073 site	mutation type[a]	Inferred ancestral base[b]	lineage inferred to mutate	Support level[c]	ED1a[d]	536	S88	APEC O1	UTI89	E2348/69	ATCC8739	K-12	HS	SMS3-5	IAI39	E24377A	SE11	55989	IAI1	Sakai	UMN026	B4 Sb227	F5 8401	SS Ss046	D1 Sd197	B18 BS512	F2a 2457T	F2a 301
t	552216	c	551403	ns	c	clone D	++++	c	c	c	c	c	c	c	c	c	c	c	c	c	c	c	c	c	c	c	c	c	c	c	c
g	558643	a	557830	s	g	CFT073	++++	g	g	g	g	g	g	g	g	g	g	g	g	g	g	g	g	g	g	g	g	g	g	g	g
g	562872	a	562059	ns	g	CFT073	++++	g	g	g	g	g	g	g	g	g	g	g	g	g	g	g	g	g	g	g	g	g	g	g	g
a	571721	t	570908	ns	t	clone D	++++	t	t	t	t	t	t	t	t	t	t	t	t	t	t	t	t	t	t	t	t	t	t	t	t
a	580712	g	579899	ns	g	clone D	++++	g	g	g	g	g	g	g	g	g	g		g	g	g	g	g	g	g	g	g	g	g	g	g
a	586799	g	585986	ns	g	clone D	++++	g	g	g	g	g	g	g	g	g	g		a	a	a	a	g	g	g	g	g	g	g	g	g
t	614703	c	613892	ns	c	clone D	++	c	c	c	c	c	c	c	c	c	c	c	c	c	c	c	c	c		c				c	c
a	629849	c	629039	nc	a	CFT073	++	a	a	a	a	a	a					a		a			a								
a	629856	c	629046	nc	a	CFT073	++	a	a	a	a	a	a					a		a			a								
a	662386	g	661576	ns	g	clone D	++++	g	g	g	g	g	g	g	g	g	g	g	g	g	g	g	g	g	g	g	g	g	g	g	g
g	664386	a	663576	s	g	CFT073	++++	g	g	g	g	g	g	g	g	g	g	g	g	g	g	g	g	g	g	g	g	g	g	g	g
t	680371	c	679561	ns	t	CFT073	++	t	t	t	t	t	t	t	t	t	t	t	t	t	t	t	t	t		t				t	t
g	683562	a	682752	ns	g	CFT073	++++	g	g	g	g	g	g	g	g	g	g	g	g	g	g	g	g	g	g	g	g	g	g	g	g
g	693054	c	692244	ns	g	CFT073	++++	g	g	g	g	g	g	g	g	g	g	g	g	g	g	g	g	g	g	g	g		g	g	g
c	693092	t	692282	ns	c	CFT073	++++	c	c	c	c	c	c	c	c	c	c	c	c	c	c	c	c	c	c	c	c		c	c	c
c	693107	a	692297	ns	c	CFT073	++++	c	c	c	c	c	c	c	c	c	c	c	c	c	c	c	c	c	c	c	c		c	c	c
a	693108	c	692298	ns	a	CFT073	++++	a	a	a	a	a	a	a	a	a	a	a	a	a	a	a	a	a	a	a	a		a	a	a
t	693114	a	692304	s	t	CFT073	++++	t	t	t	t	t	t	t	t	t	t	t	t	t	t	t	t	t	t	t	t		t	t	t
a	695486	g	694676	s	g	clone D	++	g	g	g	g	g	g	g	g	g	g	g	g	g	g	g	g	g	g		g		g		
a	697657	g	696847	nc	g	clone D	++	g	g	a	a	a		g	g	g	g	g	g	g	g	g	g	g	g	g			g	g	g
c	701919	t	701109	ns	c	CFT073	+++	c	c	c	c	c	c																		
g	722795	c	721985	ns	g	CFT073	++	g	g	g	g	g	g	g		g			g	g	g	g			g		g		g		
t	722796	c	721986	ns	t	CFT073	++	t	t	t	t	t	t	t		t			t	t	t	t			t		t		t		
t	722797	c	721987	ns	t	CFT073	+	g	t	g	g	g	t	t		t			t	t	t	t			t		t		t		
c	722804	g	721994	ns	c	CFT073	++	c	c	c	c	c	c	c		c			c	c	c	c			c		c		c		
t	732505	c	731695	ns	c	clone D	++++	c	c	c	c	c	c	c	c	c	c	c	c	c	c	c	c	c	c	c	c	c	c	c	c
c	733624	t	732814	ns	c	CFT073	++++	c	c	c	c	c	c	c	c	c	c	c	c	c	c	c	c	c	c	c	c	c	c	c	c
a	754691	g	754593	s	a	CFT073	++	a	a	a	a	a	t	a	a	a	a	t	a	a	a	a	a	a	a	a	a	a	a	a	a

Clone D base	Clone D site	CFT 073 base	CFT 073 site	mutation type[a]	Inferred ancestral base[b]	lineage inferred to mutate	Support level[c]	ED1a[d]	536	S88	APEC O1	UTI89	E2348/69	ATCC8739	K-12	HS	SMS3-5	IAI39	E24377A	SE11	55989	IAI1	Sakai	UMN026	B4 Sb227	F5 8401	SS Ss046	D1 Sd197	B18 BS512	F2a 2457T	F2a 301
g	778958	t	778797	ns	g	CFT073	++++	g	g	g	g	g	g	g	g	g	g	g	g	g	g	g	g	g	g	g	g	g	g	g	g
c	792960	t	792799	s	?	?	+/-	a	a	a	a	a																			
c	806217	g	806102	ns	c	CFT073	++++	c	c	c	c	c	c	c	c	c	c	c	c	c	c	c	c	c	c	c	c	c	c	c	c
g	824956	a	824841	s	g	CFT073	++	g	g	g	g	g	g	g	g	g	g		g	g	g	g	g	g		g			g	g	g
a	835133	c	835018	ns	a	CFT073	++++	a	a	a	a	a	a	a	a	a	a	a	a	a	a	a	a	a	a	a	a	a	a	a	a
g	837075	a	836960	s	a	clone D	++++	a	g	a	a	a	a	a	a	a	a	a	a	a	a	a	a	a	a	a	a	a	a	a	a
a	848444	g	848329	s	g	clone D	++++	g	g	g	g	g	g	g	g	g	g	g	g	g	g	g	g	g	g	g	g	g	g	g	g
g	863423	a	863308	ns	g	CFT073	++++	g	g	g	g	g	g	g	g	g	g	g	g	g	g	g	g	g	g	g	g	g	g	g	g
t	864421	c	864306	s	t	CFT073	++++	t	t	t	t	t	t	t	t	t	t	t	t	t	t	t	t	t	t	t	t	t	t	t	t
t	864770	c	864655	ns	c	clone D	++++	c	c	c	c	c	c	c	c	c	c	c	c	c	c	c	c	c	c	c	c	c	c	c	c
g	866117	t	866002	ns	t	clone D	++++	t	t	t	t	t	t	t	t	t	t	t	t	t	t	t	t	t	t	t	t	t	t	t	t
t	883494	g	883379	ns	t	CFT073	++++	t	t	t	t	t	t	t	t	t	t	t	t	t	t	t	t	t	t	t	t	t	t	t	t
t	892207	g	892092	ns	t	CFT073	++++	t	t	t	t	t	t	t	t	t	t	t	t	t	t	t	t	t	t	t	t	t	t	t	t
a	909245	g	942611	s	a	CFT073	++++	a	a	a	a	a	a	a	a	a	a	a	a	a	a	a	a	a	a	a	a	a	a	a	a
a	918512	g	951878	ns	g	clone D	++++	g	g	g	g	g	g	g	g	g	g	g	g	g	g	g	g	g	g	g	g	g	g	g	g
t	982886	a	1016254	ns	a	clone D	++++	a	a	a	a	a	a	a	a	a	a	a	a	a	a	a	a	a	a	a	a	a	a	a	a
c	987068	t	1020437	ns	c	CFT073	++++	c	c	c	c	c	c	c	c	c	c	c	c	c	c	c	c	c	c	c	c	c	c	c	c
c	987082	t	1020451	ns	c	CFT073	++++	c	c	c	c	c	c	c	c	c	c	c	c	c	c	c	c	c	c	c	c	c	c	c	c
g	988324	a	1021693	ns	a	clone D	++++	a	a	a	a	a	a	a	a	a	a	a	a	a	a	a	a	a	a	a	a	a	a	a	a
c	1002042	g	1035411	ns	c	CFT073	++++	c	c	c	c	c	c	c	c	c	c	c	c	c	c	c	c	c	c	c	c	c	c	c	c
a	1011558	g	1044927	ns	g	clone D	++++	g	g	g	g	g	g	g	g	g	g	g	g	g	g	g	g	g	g	g	g	g	g	g	g
c	1013482	t	1046851	ns	c	CFT073	++++	c	c	c	c	c	c	c	c	c	c	c	c	c	c	c	c	c	c	c	c	c	c	c	c
t	1017277	a	1050646	ns	t	CFT073	++++	t	t	t	t	t	t	t	t	t	t	t	t	t	t	t	t	t	t	t	t	t	t	t	t
g	1017536	t	1050905	s	g	CFT073	++++	g	g	g	g	g	g	g	g	g	g	g	g	g	g	g	g	g	g	g	g	g	g	g	g
t	1017888	g	1051257	ns	g	clone D	++++	g	g	g	g	g	g	g	g	g	g	g	g	g	g	g	g	g	g	g	g	g	g	g	g
a	1025532	c	1058901	ns	a	CFT073	++++	a	a	a	a	a	a	a	a	a	a	a	a	a	a	a	a	a	a	a	a	a	a	a	a
c	1026896	t	1060265	ns	t	clone D	++++	t	t	t	t	t	t	t	t	t	t	t	t	t	t	t	t	t	t	t	t	t	t	t	t
c	1043140	t	1076509	s	c	CFT073	++++	c	c	c	c	c	c	c	c	c	c	c	c	c	c	c	c	c	c	c	c	c	c	c	c

Clone D base	Clone D site	CFT 073 base	CFT 073 site	mutation type[a]	Inferred ancestral base[b]	lineage inferred to mutate	Support level[c]	ED1a[d]	536	S88	APEC O1	UTI89	E2348/69	ATCC8739	K-12	HS	SMS3-5	IAI39	E24377A	SE11	55989	IAI1	Sakai	UMN026	B4 Sb227	F5 8401	SS Ss046	D1 Sd197	B18 BS512	F2a 2457T	F2a 301
t	1048389	g	1081758	ns	t	CFT073	++++	t	t	t	t	t	t	t	t	t	t	t	t	t	t	t	t	t	t	t	t	t	t	t	t
g	1051361	t	1084730	ns	g	CFT073	++++	g	g	g	g	g	g	g	g	g	g	t	g	g	g	g	g	g	g	g	g	g	g	g	g
t	1063558	c	1096927	ns	t	CFT073	++++	t	t	t	t	t	t	t	t	t	t	t	t	t	t	t	t	t	t	t	t	t	t	t	t
t	1065603	a	1098972	nc	a	clone D	++	a	a	a	a	a	a	a	a	a	a	a	a	a	a	a	a	a		a	a	a		a	a
g	1071559	a	1104928	s	a	clone D	++	a	a	a	a	a	a	a	a	a	a	a	a	a	a	a	a	a		a	a	a		a	a
c	1073352	a	1106721	s	c	CFT073	++	c	c	c	c	c	c	c	c	c	c	c	c	c	c	c	c	c		c	c	c		c	c
a	1074028	g	1107397	ns	g	clone D	++++	g	g	g	g	g	g	g	g	g	g	g	g	g	g	g	g	g	g	g	g	g	g	g	g
g	1125611	c	1158981	nc	?	?	+/-																								
g	1129793	t	1163163	nc	g	CFT073	+		g			g																			
a	1131681	g	1165052	nc	?	?	+/-																								
a	1132196	g	1165567	s	?	?	+/-																								
g	1145111	a	1178484	s	?	?	+/-																								
g	1153965	a	1187338	nc	a	clone D	+		a			a																			
a	1172295	t	1205668	s	a	CFT073	+		a			a																a			
t	1194598	g	1229284	s	t	CFT073	++	t							t						t			t						t	t
g	1195856	a	1230542	ns	g	CFT073	++	g													g			g						g	g
c	1200857	t	1237959	nc	c	CFT073	++	c	c	c	c	c	c		c		c	c	c		c		c	c				c	c	c	c
t	1203224	c	1241037	nc	t	CFT073	+					t	t				t						t					t			
t	1210308	a	1248122	ns	t	CFT073	++	t	t	t	t	t	t	t	t	t	t	t	t	t	t	t	t	t	t	t	t			t	t
a	1212641	g	1250455	ns	g	clone D	++++	g	g	g	g	g	g	g	g	g	g	g	g	g	g	g	g	g	g	g	g	g	g	g	g
g	1235940	c	1273757	ns	c	clone D	++++	c	c	c	c	c	c	c	c	c	c	c	c	c	c	c	c	c	c	c	c	c	c	c	c
c	1236058	a	1273875	s	c	CFT073	++++	c	c	c	c	c	c	c	c	c	c	c	c	c	c	c	c	c	c	c	c	c	c	c	c
a	1249884	g	1287701	ns	g	clone D	++++	g	g	g	g	g	g	g	g	g	g	g	g	g	g	g	g	g	g	g	g	g	g	g	g
t	1294835	a	1333367	ns	t	CFT073	++	t		t	t	t	t	t	t		t		t	t	t	t	t						t		
a	1308118	c	1347361	nc	c	clone D	+						c						c		c			c							
c	1311991	t	1351234	s	c	CFT073	++	c		t	t	t	t					t		t	t		t	c		g			t	g	g
t	1320170	c	1359413	s	c	clone D	++	c	c	c	c	c	c	c				c		c	c	c	c	c			c		c		
t	1322018	c	1361261	s	g	clone D	++	g	c	c	c	c	c	c				c		c	t	c	c	c			c		t		

Clone D base	Clone D site	CFT 073 base	CFT 073 site	mutation type[a]	Inferred ancestral base[b]	lineage inferred to mutate	Support level[c]	ED1a[d]	536	S88	APEC O1	UTI89	E2348/69	ATCC8739	K-12	HS	SMS3-5	IAI39	E24377A	SE11	55989	IAI1	Sakai	UMN026	B4 Sb227	F5 8401	SS Ss046	D1 Sd197	B18 BS512	F2a 2457T	F2a 301
g	1322019	a	1361262	ns	g	CFT073	++	g	a	a	a	a	a	a				a		a	g	a	a	a			a		g		
t	1322021	c	1361264	ns	a	clone D	++	a	c	c	c	c	c	c				c		c	t	c	c	c			c		t		
t	1322033	c	1361276	s	g	clone D	++	g	c	c	c	c	c	c				c		c	t	c	c	c			c		c		
c	1322046	a	1361289	ns	a	clone D	++	a	a	a	a	a	a	a				a		a	c	a	a	a			a		c		
g	1322054	a	1361297	s	a	clone D	++	a	a	a	a	a	a	a				a		a	g	a	a	a			a		g		
t	1322058	c	1361301	ns	c	clone D	++	c	c	c	c	c	c	c				c		c	t	c	c	c			c		t		
t	1322060	g	1361303	ns	g	clone D	++	g	g	g	g	g	g	g				g		g	t	g	g	g			g		t		
g	1322063	t	1361306	s	t	clone D	++	t	t	t	t	t	t	t				t		t	g	t	t	g			t		g		
g	1322072	a	1361315	s	c	clone D	++	c	a	a	a	a	a	a				a		a	g	a	a	a			a		g		
a	1322084	t	1361327	s	t	clone D	++	t	t	t	t	t	t	t				t		t	a	t	t	t			t		t		
c	1323330	t	1362573	s	c	CFT073	+	g	c	c	c	c	c	c			c	t		c	t	c	a	c	t	c	c		t	c	c
a	1332492	c	1371735	nc	c	clone D	++	c	c	a	a	a	c		a			a	a		a			a			a		a		
a	1332511	g	1371754	nc	g	clone D	++	g	g	a	a	a	g		a			a	a		a			a			a		a		
c	1332512	t	1371755	nc	t	clone D	++	t	t	c	c	c	t		c			c	c		c			c			c		c		
g	1332525	a	1371768	nc	a	clone D	++	a	a	g	g	g	a		g			g	g		g			g			g		g		
t	1332558	g	1371801	nc	g	clone D	++	g	g	t	t	t	g		t			t	t	t	t		g	t	t	t	t	t	t	t	t
a	1332560	g	1371803	nc	g	clone D	++	g	g	a	a	a	g		a			a	a	a	a		a	a	a	a	a	a	a	a	a
a	1332561	c	1371804	nc	c	clone D	++	c	c	a	a	a	c		a			a	a	t	a		t	a	a	a	a	a	a	a	a
t	1332579	c	1371822	nc	c	clone D	++	c	c	t	t	t	c		t			t	t	t	t		t	t	t	t	t	t	t	t	t
g	1332627	t	1371870	ns	t	clone D	++	t	t	g	g	g	t		g			g	g	g	g		g	g	g	g	g	g	g	g	g
g	1343579	t	1394274	s	g	CFT073	++++	g	g	g	g	g	g	g	g	g	g	g	g	g	g	g	g	g	g	g	g	g	g	g	g
c	1343615	a	1394310	ns	c	CFT073	++++	c	c	c	c	c	c	c	c	c	c	c	c	c	c	c	c	c	c	c	c	c	c	c	c
t	1358433	c	1412847	s	t	CFT073	+			t	t	t	t										t								
a	1358517	t	1412935	ns	a	CFT073	+			a	a	a	a										a								
g	1363135	a	1418868	ns	a	clone D	++		a	a	a	a			a				a	a	a	a	a	a			a				
t	1364569	c	1420302	ns	c	clone D	++		c	c	c	c			g				c	g	c	g		c		c	c			c	c
a	1365402	g	1421135	ns	g	clone D	++		g	g	g	g			g				g	g	g	g		g		a	g			a	a
t	1365404	g	1421137	ns	g	clone D	++		g	g	g	g			g				g	g	g	g		g		t	g			t	t

Clone D base	Clone D site	CFT 073 base	CFT 073 site	mutation type[a]	Inferred ancestral base[b]	lineage inferred to mutate	Support level[c]	ED1a[d]	536	S88	APEC O1	UTI89	E2348/69	ATCC8739	K-12	HS	SMS3-5	IAI39	E24377A	SE11	55989	IAI1	Sakai	UMN026	B4 Sb227	F5 8401	SS Ss046	D1 Sd197	B18 BS512	F2a 2457T	F2a 301
c	1365406	t	1421139	ns	t	clone D	++		t	t	t	t			t				t	t	t	t		t		c	t			c	c
a	1365427	t	1421160	nc	t	clone D	+		t	t	t	t			t				t	t	t	t		t		a	t			a	a
a	1371117	g	1426850	ns	g	clone D	++	g	g	g	g	g	g	g				g	g	g	g	g	g	g		g	g		g	g	g
c	1372049	t	1427782	s	c	CFT073	++	c		t	t	t	c					c		a	t		a	c		c			c	c	c
a	1373356	c	1429089	ns	c	clone D	++	c		a	a	a	c					c		c	c		c	c		c			c	c	c
c	1374811	a	1430544	nc	c	CFT073	++	c		a	a	a	c					c		-	c		c	c		a			c	a	a
g	1374984	c	1430717	ns	g	CFT073	+	a		g	g	g	g					g		g	g		g	g		g			g	g	g
c	1374985	g	1430718	ns	c	CFT073	++	c		c	c	c	c					c		c	c		c	c		c			c	c	c
g	1378342	a	1434074	ns	g	CFT073	++	g	g	g	g	g	g	g				g		g	g	g	g	g			g		g		
c	1378488	t	1434218	s	t	clone D	++	t	c	c	c	c	t	c				t		c	c	c	c	c			c		t		
g	1378491	c	1434221	s	g	CFT073	++	g	g	g	g	g	c	g				g		g	g	g	g	g			g		g		
a	1378492	g	1434222	ns	a	CFT073	++	a	a	a	a	a	g	a				a		a	a	a	a	a			a		a		
t	1382651	c	1438380	s	c	clone D	++	c	c	t	t	t	c	c			c	c		c	c	c	c	c	c	c	c		c	c	c
a	1389904	t	1445633	ns	a	CFT073	++	a	a	a	a	a						a													
t	1408061	c	1463784	s	c	clone D	++++	c	c	c	c	c	c	c	c	c	c	c	c	c	c	c	c	c	c	c	c	c	c	c	c
a	1413461	g	1469184	ns	a	CFT073	++++	a	a	a	a	a	a	a	a	a	a	a	a	a	a	a	a	a	a	a	a	a	a	a	a
a	1415941	g	1471664	s	a	CFT073	++++	a	a	a	a	a	g	g	g	g	g	g	g	g	g	g	g	g	g	g	g	g	g	g	g
c	1422229	t	1477952	s	c	CFT073	++++	c	c	c	c	c	c	c	c	c	c	c	c	c	c	c	c	c	c	c	c	c	c	c	c
c	1427882	t	1483605	ns	c	CFT073	++	c	c	c	c	c	c				c	c					c	c		c		c		c	c
t	1439811	c	1495534	ns	t	CFT073	++	t	t	t	t	t	t	t	t	t	t	t	t	t	t	t		t	t	t			t	t	t
g	1452981	a	1508704	ns	g	CFT073	++++	g	g	g	g	g	g	g	g	g	g	g	g	g	g	g	g	g	g	g	g	g	g	g	g
g	1480109	a	1535832	ns	g	CFT073	++++	g	g	g	g	g	g	g	g	g	g	g	g	g	g	g	g	g	g	g	g	g	g	g	g
g	1486554	a	1542277	ns	g	CFT073	++++	g	g	g	g	g	g	g	g	g	g	g	g	g	g	g	g	g	g	g	g	g	g	g	g
g	1506794	t	1562517	s	g	CFT073	++++	g	g	g	g	g	g	g	g	g	g	g	g	g	g	g	g	g	g	g	g	g	g	g	g
c	1529648	t	1586082	nc	c	CFT073	++++	c	c	c	c	c	c	c	c	a	c	c	c	c	c	c	c	c	c	c	c	c	c	c	c
g	1531673	a	1588106	s	a	clone D	++++	a	a	a	a	a	a	a	a	a	a	a	a	a	a	a	a	a	a	a	a	a	a	a	a
a	1586860	g	1643294	ns	a	CFT073	++	a	a	a	a	a	a													a				a	a
g	1593037	c	1649471	ns	g	CFT073	++++	g	g	g	g	g	g	g	g	g	g	g	g	g	g	g	g	g	g	g	g	g	g	g	g

Clone D base	Clone D site	CFT 073 base	CFT 073 site	mutation type[a]	Inferred ancestral base[b]	lineage inferred to mutate	Support level[c]	ED1a[d]	536	S88	APEC O1	UTI89	E2348/69	ATCC8739	K-12	HS	SMS3-5	IAI39	E24377A	SE11	55989	IAI1	Sakai	UMN026	B4 Sb227	F5 8401	SS Ss046	D1 Sd197	B18 BS512	F2a 2457T	F2a 301
c	1596208	t	1652642	ns	c	CFT073	+++	c	c	c	c	c	c																		
t	1618956	c	1675538	s	c	clone D	++		c	c	c	c		c	c	c	c	c	c	c	c	c	c	c	c	a	a	c	c	a	a
g	1622436	t	1679018	ns	g	CFT073	++++	g	g	g	g	g	g	g	g	g	g	g	g	g	g	g	g	g	g	g	g	g	g	g	g
t	1631370	g	1687952	ns	t	CFT073	++	t	t	t	t	t	t	t	t	t	t		t	t	t	t	t	t	t	t	t	t	t	t	t
t	1643881	g	1700463	ns	t	CFT073	++	t	t	t	t	t	t	t	t	t	t		t	t	t	t	t	t			t	t			
c	1663549	t	1720135	ns	t	clone D	+																	t							
a	1673112	g	1729696	nc	g	clone D	++	g	g	g	g	g	g	g	g	g	g	g	g	g	g	g	g	-			g	g			
t	1701270	c	1757853	nc	t	CFT073	++	t	t	t	t	t	t	t	t	t	t	t	t	t	t	t	-	t	t	t	t	t	t	t	t
t	1701329	g	1757912	nc	t	CFT073	++++	t	t	t	t	t	t	t	t	t	t	t	t	t	t	t	t	t	t	t	t	t	t	t	t
c	1715067	g	1771649	ns	c	CFT073	++++	c	c	c	c	c	c	c	c	c	c	c	c	c	c	c	c	c	c	c	c	c	c	c	c
c	1724309	g	1781887	ns	c	CFT073	++	c	c	c	c	c	c	c	c	c				c	c	c	c	c			c	c	c		
g	1733001	c	1790581	ns	c	clone D	++++	c	c	c	c	c	c	c	c	c	c	c	c	c	c	c	c	c	c	c	c	c	c	c	c
t	1741553	c	1799133	s	t	CFT073	++			t	t	t				t	t	t	t	t	t	t									
a	1742588	g	1800168	s	g	clone D	+			g	g	g				g	g	g	g	g	g	g									
c	1760775	t	1818329	ns	c	CFT073	++++	c	c	c	c	c	c	c	c	c	c	c	c	c	c	c	c	c	c	c		c	c	c	c
t	1781828	c	1839382	s	c	clone D	++++	c	c	c	c	c	c	c	c	c	c	c	c	c	c	c	c	c	c	c	c	c	c	c	c
a	1786712	g	1844266	s	g	clone D	++++	g	t	g	g	g	g	g	g	g	g	g	g	g	g	g	g	g	g	g	g	g	g	g	g
a	1793213	g	1850767	ns	a	CFT073	++++	a	a	a	a	a	a	a	a	a	a	a	a	a	a	a	a	a	a	a	a	a	a	a	a
g	1796475	a	1854029	ns	g	CFT073	++++	g	g	g	g	g	g	g	g	g	g	g	g	g	g	g	g	g	g	g	g	g	g	g	g
a	1802483	g	1860037	s	g	clone D	++++	g	g	g	g	g	g	g	g	g	g	g	g	g	g	g	g	g	g	g	g	g	g	g	g
g	1830257	t	1887811	s	g	CFT073	++++	g	g	g	g	g	g	g	g	g	g	g	g	g	g	g	g	g	g	g	g	g	g	g	g
a	1840635	g	1898189	s	g	clone D	++++	g	g	g	g	g	g	g	g	g	g	g	g	g	g	g	g	g	g	g	g	g	g	g	g
g	1853236	a	1910790	nc	a	clone D	++	a	a	a	a	a	a	a	a	a	a	a	a	a	a	a	a	a	a	a	a			a	a
a	1855927	t	1913481	nc	a	CFT073	++++	a	a	a	a	a	a	a	a	a	a	a	a	a	a	a	a	a	a	a	a	a	a	a	a
a	1855928	g	1913482	nc	a	CFT073	++++	a	a	a	a	a	a	a	a	a	a	a	a	a	a	a	a	a	a	a	a	a	a	a	a
a	1855940	g	1913494	nc	a	CFT073	++++	a	a	a	a	a	a	a	a	a	a	a	a	a	a	a	a	a	a	a	a	a	a	a	a
a	1855949	g	1913503	nc	a	CFT073	++++	a	a	a	a	a	a	a	a	a	a	a	a	a	a	a	a	a	a	a	a	a	a	a	a
c	1883806	t	1941360	ns	c	CFT073	++++	c	c	c	c	c	c	c	c	c	c	c	c	c	c	c	c	c	c	c	c	c	c	c	c

Clone D base	Clone D site	CFT 073 base	CFT 073 site	mutation type[a]	Inferred ancestral base[b]	lineage inferred to mutate	Support level[c]	ED1a[d]	536	S88	APEC O1	UTI89	E2348/69	ATCC8739	K-12	HS	SMS3-5	IAI39	E24377A	SE11	55989	IAI1	Sakai	UMN026	B4 Sb227	F5 8401	SS Ss046	D1 Sd197	B18 BS512	F2a 2457T	F2a 301
g	1895916	c	1953470	ns	g	CFT073	++	g	c	c	c	c	g	c	c	c	c	c	c	g	c	c	c	c	g	g	g	c	g	c	c
g	1908823	a	1966378	s	g	CFT073	++++	g	g	g	g	g	g	g	g	g	g	g	a	g	a	a	g	g	g	g	g	g	g	g	g
a	1926109	t	1983664	ns	a	CFT073	++++	a	a	a	a	a	a	a	a	a	a	a	a	a	a	a	a	a	a	a	a	a	a	a	a
c	1929047	t	1986602	s	c	CFT073	++++	c	c	c	c	c	c	c	c	c	c	c	c	c	c	c	c	c	c	c	c	c	c	c	c
t	1946402	c	2003957	ns	c	clone D	++++	c	c	c	c	c	c	c	c	c	c	c	c	c	c	c	c	c	c	c	c	c	c	c	c
t	1961111	c	2018666	nc	t	CFT073	++	t	t	t	t	t	t	t	t	t	t	t	t	t	t	t	t	t		t		t		t	t
g	1977237	a	2034790	nc	a	clone D	++	a	g	a	a	a	g	c	a	a	g	g	c	g	c	g	a	g	g		c	a	g	a	a
g	1986053	c	2043606	s	g	CFT073	++++	g	g	g	g	g	g	g	g	g	g	g	g	g	g	g	g	g	g	g	g	g	g	g	g
g	1987686	a	2045239	s	g	CFT073	++++	g	g	g	g	g	g	g	g	g	g	g	g	g	g	g	g	g	g	g	g	g	g	g	g
t	1988983	g	2046536	ns	t	CFT073	++++	t	t	t	t	t	t	t	t	t	t	t	t	t	t	t	t	t	t	t	t	t	t	t	t
a	1998937	g	2056490	s	g	clone D	++++	g	g	g	g	g	g	g	g	g	g	g	g	g	g	g	g	g	g	g	g	g	g	g	g
c	2002055	t	2059608	nc	t	clone D	++++	t	t	t	t	t	c	t	t	t	c	c	t	t	t	t	t	t	t	t	t	t		t	t
g	2010225	a	2067779	nc	?	?	+/-	-	-	-	-	-	g	g	-	-	-	-	-	-	g	g	-	-	g	-	-	-	-	-	-
a	2017482	c	2075040	nc	a	CFT073	++++	a	a	a	a	a	a	a	a	a	a	a	a	a	a	a	a	a	a	a	a	a	a	a	a
c	2044891	t	2102449	ns	c	CFT073	++++	c	c	c	c	c	c	c	c	c	c	c	c	c	c	c	c	c	c	c	c	c	c	c	c
g	2044949	a	2102507	s	g	CFT073	++++	g	g	g	g	g	g	g	g	g	g	g	g	g	g	g	g	g	g	g	g	g	g	g	g
a	2054332	g	2111890	s	g	clone D	++++	g	g	g	g	g	g	g	g	g	g	g	g	g	g	g	g	g	g	g	g	g	g	g	g
t	2070718	a	2128276	nc	t	CFT073	++++	t	t	t	t	t	t	t	t	t	t	t	t	t	t	t	t	t	t	t	t	t			
g	2070754	a	2128312	nc	g	CFT073	++++	g	g	g	g	g	g	g	a	g	g	g	g	g	g	g	g	g	g	g	g	g			
a	2077269	g	2134821	ns	a	CFT073	++++	a	a	a	a	a	a	a	a	a	a	a	a	a	a	a	a	a	a	a	a	a	a	a	a
c	2078871	t	2136423	s	c	CFT073	++++	c	c	c	c	c	c	c	c	c	c	c	c	c	c	c	c	c	c	c	c	c	c	c	c
g	2107023	a	2165287	s	g	CFT073	++	g	g	g	g	g	g	g	g	g	g	g	g	g	g	g	g	g	g	g	g			-	-
c	2109941	t	2168205	s	c	CFT073	++	c	c	c	c	c	c	c	c	c	c	c	c	c	c	c	c	c	c	c	c			c	c
a	2112295	g	2170559	ns	g	clone D	++	g	g	g	g	g	g	g	g	g	g	g	g	g	g	g	g	g	g	g	g			g	g
g	2135380	t	2193644	ns	g	CFT073	++++	g	g	g	g	g	g	g	g	g	g	g	g	g	g	g	g	g	g	g	g	g	g	g	g
c	2143727	t	2201991	ns	c	CFT073	++	c	c				c																		
a	2159887	c	2218151	nc	a	CFT073	++	a	a	a	a	a		a				a			c			a	a	c			a	a	a
g	2165206	a	2223470	ns	a	clone D	++	a	a	a	a	a						a			a			a							

Clone D base	Clone D site	CFT 073 base	CFT 073 site	mutation type[a]	Inferred ancestral base[b]	lineage inferred to mutate	Support level[c]	ED1a[d]	536	S88	APEC O1	UTI89	E2348/69	ATCC8739	K-12	HS	SMS3-5	IAI39	E24377A	SE11	55989	IAI1	Sakai	UMN026	B4 Sb227	F5 8401	SS Ss046	D1 Sd197	B18 BS512	F2a 2457T	F2a 301
g	2170495	t	2228759	ns	g	CFT073	++	g	g	g	g	g						g			g			g							
a	2180055	c	2239019	s	c	clone D	++	c	c	c	c	c						c			c			c							
g	2180056	a	2239020	s	a	clone D	++	a	a	a	a	a						a			a			a							
g	2180064	a	2239026	ns	a	clone D	++	a	a	a	a	a						a			a			a							
a	2185505	c	2244467	ns	c	clone D	++	c	c	c	c	c						c			c			c							
a	2190481	c	2249443	nc	c	clone D	++	c	a	c	c	c						c			c			c							
a	2199173	g	2258133	s	a	CFT073	++	a	g	g	g	g	a	g	a	g	g	g	a	a	g	g	g	a	g	a	a		g	a	a
a	2202789	g	2261749	ns	a	CFT073	++++	a	a	a	a	a	a	a	a	a	a	a	a	a	a	a	a	a	a	a	a	a	a	a	a
a	2203718	g	2262678	nc	g	clone D	++++	g	g	g	g	g	g	g	g	g	g	g	g	g	g	g	g	g	g	g	g	g	g	g	g
g	2237404	a	2296365	s	g	CFT073	+		g			g																			
c	2240758	t	2299719	s	c	CFT073	+		c			c																			
c	2240778	t	2299739	ns	c	CFT073	+		c			c																			
c	2246105	t	2305066	s	c	CFT073	+		c			c																			
c	2249273	g	2308234	s	c	CFT073	+		c			c																			
t	2258968	c	2317914	ns	c	clone D	++++	c	c	c	c	c	c	c	c	c	c	c	c	c	c	c	c	c	c	c	c	c	c	c	c
t	2275876	c	2334825	ns	c	clone D	++	c	c	c	c	c					c	c			c										
a	2282740	g	2341764	ns	g	clone D	+		g			g													g	g	g	g	g	g	g
t	2290069	c	2351509	nc	t	CFT073	+		t	t	t	t					t	t			t										
a	2290636	g	2352076	nc	g	clone D	+		g			g																			
g	2293206	a	2354646	s	g	CFT073	+		g			g																			
g	2296423	a	2357863	nc	g	CFT073	++	g	a			a	g						g												
c	2296445	t	2357885	nc	c	CFT073	++	c	t			t	c						c												
t	2296515	c	2357955	nc	t	CFT073	++	t	c			c	t						t												
a	2296517	g	2357957	nc	g	clone D	++	g	g			g	a						a												
c	2296518	a	2357958	nc	c	CFT073	++	c	a			a	c						c												
a	2296557	c	2357997	nc	c	clone D	++	c	c			c	a						a												
c	2296559	t	2357999	nc	t	clone D	++	t	t			t	c						t												
c	2296666	t	2358106	nc	c	CFT073	+		t	c	c	t					c	c			c										

Clone D base	Clone D site	CFT 073 base	CFT 073 site	mutation type[a]	Inferred ancestral base[b]	lineage inferred to mutate	Support level[c]	ED1a[d]	536	S88	APEC O1	UTI89	E2348/69	ATCC8739	K-12	HS	SMS3-5	IAI39	E24377A	SE11	55989	IAI1	Sakai	UMN026	B4 Sb227	F5 8401	SS Ss046	D1 Sd197	B18 BS512	F2a 2457T	F2a 301
c	2296690	a	2358130	nc	c	CFT073	+		a	c	c	a					c	c			c										
t	2296802	c	2358242	nc	c	clone D	+		c	c	c	c					t	t			c										
g	2296803	a	2358243	nc	a	clone D	+		a	a	a	a					a	g			a										
t	2296816	c	2358256	nc	c	clone D	+		c	c	c	c					c	t			c										
c	2296817	a	2358257	nc	a	clone D	+		a	a	a	a					a	c			a										
t	2296820	c	2358260	nc	c	clone D	+		c	c	c	c					c	t			c										
c	2296825	t	2358265	nc	t	clone D	+		t	t	t	t					t	c			t										
c	2296826	t	2358266	nc	t	clone D	+		t	t	t	t					t	c			t										
a	2296835	c	2358275	nc	c	clone D	+		c	c	c	c					c	a			c										
t	2296838	c	2358278	nc	c	clone D	+		c	c	c	c					c	t			c										
a	2296845	t	2358285	nc	t	clone D	+		t	t	t	t					t	a			t										
c	2296990	t	2358430	nc	t	clone D	+		t	t	t	c					t	c			t										
t	2296992	c	2358432	nc	t	CFT073	+		c	t	t	t					t	t			t										
c	2296994	t	2358434	nc	t	clone D	+		t	t	t	c					t	c			t										
g	2298518	a	2359959	s	g	CFT073	+		g	g	g	g	g				g	g			g		g								
g	2299407	a	2360848	ns	g	CFT073	+		a	g	g	a	g				g	g			g		g								
a	2299410	c	2360851	s	c	clone D	+		c	c	c	c	c				c	c			c		c								
c	2299700	g	2361141	ns	c	CFT073	+		g	c	c	g	c				c	c			c		c								
g	2299712	c	2361153	ns	g	CFT073	+		c	g	g	c	g				g	g			g		g								
c	2299990	t	2361431	s	t	clone D	+		t	t	t	t	t				t	c			t		t								
g	2300703	a	2362144	s	a	clone D	+		a	a	a	a	a				a	a			a		a								
t	2300793	c	2362234	s	c	clone D	+		c	c	c	c	c				c	t			c		c								
t	2309336	c	2370777	nc	t	CFT073	++	t	t	t	c	t	c	t	t	t	t	c	t	t	t	t	t	t			t	t	t		
g	2315229	a	2376670	s	g	CFT073	++++	g	g	g	g	g	g	g	g	g	g	g	g	g	g	g	g	g	g	g	g	g	g	g	g
g	2320221	a	2381662	s	g	CFT073	++++	g	g	g	g	g	g	g	g	g	g	g	g	g	g	g	g	g	g	g	g	g	g	g	g
t	2336808	c	2398250	ns	t	CFT073	+		t																						
t	2339703	g	2401145	ns	t	CFT073	++++	t	t	t	t	t	t	t	t	t	t	t	t	t	t	t	t	t	t	t		t	t	t	t
c	2345037	t	2406286	s	t	clone D	++	t	t	t	t	t	t	t	t		t	t	t	t	t	t	t	t	t	t			t	t	t

Clone D base	Clone D site	CFT 073 base	CFT 073 site	mutation type[a]	Inferred ancestral base[b]	lineage inferred to mutate	Support level[c]	ED1a[d]	536	S88	APEC O1	UTI89	E2348/69	ATCC8739	K-12	HS	SMS3-5	IAI39	E24377A	SE11	55989	IAI1	Sakai	UMN026	B4 Sb227	F5 8401	SS Ss046	D1 Sd197	B18 BS512	F2a 2457T	F2a 301
c	2356361	t	2417610	ns	c	CFT073	++	c	c	c	c	c	c	c	c		c	c	c	c	c	c	c	c	c	c	c		c	c	c
g	2361324	a	2422573	ns	g	CFT073	++	g	g	g	g	g	g	g	g		g	g	g	g	g	g	g	g	g	g	g		g	g	g
g	2362173	a	2423422	s	g	CFT073	++	g	g	g	g	g	g	g	g		g	g	g	g	g	g	g	g	g	g	g		g	g	g
g	2362253	a	2423501	ns	g	CFT073	++	g	g	g	g	g	g	g	g		g	g	g	g	g	g	g	g	g	g	g		g	g	g
c	2362254	g	2423502	ns	c	CFT073	++	c	c	c	c	c	c	c	c		c	c	c	c	c	c	c	c	c	c	c		c	c	c
g	2365761	a	2427006	s	g	CFT073	++++	g	g	g	g	g	g	g	g	g	g	g	g	g	g	g	g	g	g	g	g	g	g	g	g
g	2376994	a	2438239	s	a	clone D	++	a	g	g	g	g	g	a	a	a	g	g	a	a	a	a	g	g	a	a	a	g	g	a	a
a	2394630	g	2455875	s	g	clone D	++	g	g	g	g	g																			
t	2405478	g	2466723	ns	g	clone D	++		g	g	g	g	g	g	g	g	g	g	g	g	g	g	g	g	g	g	g	g	g	g	g
a	2406271	g	2468227	ns	g	clone D	++		g	g	g	g	g	g	g	g	g	g	g	g	g	g	g	g	g	g	g	g	g	g	g
a	2409395	c	2471351	s	a	CFT073	++++	a	a	a	a	a	a	c	c	a	a	a	a	a	a	a	a	a	a	a	c	a	a	a	a
c	2409418	t	2471374	ns	c	CFT073	++++	c	c	c	c	c	c	c	c	c	c	c	c	c	c	c	c	c	c	c	c	c	c	c	c
c	2409443	a	2471399	ns	c	CFT073	++++	c	c	c	c	c	c	c	c	c	c	c	c	c	c	c	c	c	c	c	c	c	c	c	c
c	2409465	t	2471421	s	c	CFT073	++++	c	c	c	c	c	c	c	c	c	c	c	c	c	c	c	c	c	c	c	c	c	c	c	c
a	2414055	g	2476011	nc	g	clone D	++++	g	g	g	g	g	g	g	g	g	g	g	g	g	g	g	g	g	g	g	g	g	g	g	g
g	2428483	a	2491151	ns	g	CFT073	++	g	g	g	g	g	g	g	g	g	g	g	g	g		g	g	g	g	g		g		g	g
c	2433356	a	2496024	nc	c	CFT073	++	c	c	c	c	c	c	c	c	c	c	c	c	c		c	c	c	c					c	c
c	2440988	a	2503656	ns	c	CFT073	++++	c	c	c	c	c	c	c	c	c	c	c	c	c	c	c	c	c	c	c	c	c	c	c	c
a	2443259	g	2505927	ns	g	clone D	++++	g	g	g	g	g	g	g	g	g	g	g	g	g	g	g	g	g	g	g	g	g	g	g	g
t	2443878	c	2506546	nc	c	clone D	++	c	c	c	c	c	c	c	c	c	c	c	c	c	c	c	c	c	c		c	c	c		
a	2444543	g	2507211	s	g	clone D	++++	g	g	g	g	g	g	g	g	g	g	g	g	g	g	g	g	g	g	g	g	g		g	g
a	2485863	t	2548531	ns	a	CFT073	++++	a	a	a	a	a	a	a	a	a	a	a	a	a	a	a	a	a	a	a	a		a	a	a
a	2485896	g	2548564	ns	a	CFT073	++++	a	a	a	a	a	a	a	a	a	a	a	a	a	a	a	a	a	a	a	a		a	a	a
a	2485899	t	2548567	s	a	CFT073	++++	a	a	a	a	a	a	a	a	a	a	a	a	a	a	a	a	a	a	a	a		a	a	a
a	2485913	g	2548581	ns	g	clone D	++	g	-	-	-	-	-	g	g	g	g	g	g	g	g	g	g	g	g	g	-		g	g	g
c	2485914	g	2548584	ns	?	?	+/-	-	g	g	g	g	g	-	-	-	-	-	-	-	-	-	-	-	-	-	g		-	-	-
c	2485917	a	2548587	ns	c	CFT073	++++	c	c	c	c	c	c	c	c	c	c	c	c	c	c	c	c	c	c	c	c		c	c	c
t	2485922	a	2548592	ns	t	CFT073	++++	t	t	t	t	t	t	t	t	t	t	t	t	t	t	t	t	t	t	t	t		t	t	t

Clone D base	Clone D site	CFT 073 base	CFT 073 site	mutation type[a]	Inferred ancestral base[b]	lineage inferred to mutate	Support level[c]	ED1a[d]	536	S88	APEC O1	UTI89	E2348/69	ATCC8739	K-12	HS	SMS3-5	IAI39	E24377A	SE11	55989	IAI1	Sakai	UMN026	B4 Sb227	F5 8401	SS Ss046	D1 Sd197	B18 BS512	F2a 2457T	F2a 301
c	2497637	a	2560307	nc	c	CFT073	++++	c	c	c	c	c	c	c	c	c	c	c	c	c	c	c	c	c	c	c	c	c	c	c	c
c	2506161	a	2568831	nc	c	CFT073	++++	c	c	c	c	c	c	c	c	c	c	c	c	c	c	c	c	c	c	c	c	c	c	c	c
g	2517522	a	2580192	ns	g	CFT073	++++	g	g	g	g	g	g	g	g	g	g	g	g	g	g	g	g	g	g	g	g	g	g	g	g
c	2525558	a	2588228	s	c	CFT073	++++	c	c	c	c	c	c	c	c	c	c	c	c	c	c	c	c	c	c	c	c	c	c	c	c
g	2542586	a	2605256	s	g	CFT073	++++	g	g	g	g	g	g	g	g	g	g	g	g	g	g	g	g	g	g	g	g	g	g	g	g
c	2618491	t	2681161	ns	c	CFT073	++++	c	c	c	c	c	c	c	c	c	c	c	c	c	c	c	c	c	c	c	c	c	c	c	c
a	2625164	c	2687834	ns	a	CFT073	++++	a	a	a	a	a	a	a	a	a	a	a	a	a	a	a	a	a	a	a	a	a	a	a	a
c	2652749	t	2715419	ns	c	CFT073	++++	c	c	c	c	c	c	c	c	c	c	c	c	c	c	c	c	c	c	c	c	c	c	c	c
c	2658773	g	2721443	ns	c	CFT073	++++	c	c	c	c	c	c	c	c	c	c	c	c	c	c	c	c	c	c	c	c		c	c	c
a	2665170	g	2727838	s	g	clone D	++++	g	g	g	g	g	g	g	g	g	g	g	g	g	g	g	g	g	g	g	g	g	g	g	g
t	2685309	c	2747977	s	c	clone D	+										c														
t	2695383	c	2758071	nc	t	CFT073	+										t	t													
g	2704159	a	2766847	ns	g	CFT073	++++	g	g	g	g	g	g	g	g	g	g	g	g	g	g	g	g	g	g	g	g	g	g	g	g
a	2715730	g	2778418	ns	g	clone D	++++	g	g	g	g	g	g	g	g	g	g	g	g	g	g	g	g	g	g	g	g	g	g	g	g
c	2717882	t	2780570	ns	c	CFT073	++++	c	c	c	c	c	c	c	c	c	c	c	c	c	c	c	c	c	c	c	c	c	c	c	c
t	2718009	g	2780697	ns	t	CFT073	++++	t	t	t	t	t	t	t	t	t	t	t	t	t	t	t	t	t	t	t	t	t	t	t	t
a	2718020	g	2780707	nc	a	CFT073	++	a	g	g	g	g	a	a	a	a	a	a	a	a	a	g	a	a	a	a	a	a	a	a	a
t	2724895	c	2787582	ns	t	CFT073	++++	t	t	t	t	t	t	t	t	t	t	t	t	t	t	t	t	t	t	t	t	t	t	t	t
t	2739621	c	2802545	ns	t	CFT073	++	t	t	t	t	t	t		t		t			t	t										
a	2757922	g	2820848	ns	g	clone D	++++	g	g	g	g	g	g	g	g	g	g	g	g	g	g	g	g	g	g	g	g	g	g	g	g
g	2783069	t	2845995	ns	g	CFT073	++++	g	g	g	g	g	g	g	g	g	g	g	g	g	g	g	g	g	g	g	g		g	g	g
t	2792952	c	2855878	s	c	clone D	++++	c	c	c	c	c	c	c	c	c	c	c	c	c	c	c	c	c	c	c	c	c	c	c	c
a	2811130	g	2874056	s	a	CFT073	++++	a	a	a	a	a	a	a	a	a	a	a	a	a	a	a	a	a	a	a	a	a	a	a	a
g	2838616	a	2901542	ns	g	CFT073	++	g	g	g	g	g					g	g						g							
t	2848250	c	2911176	ns	t	CFT073	++++	t	t	t	t	t	t	t	t	t	t	t	t	t	t	t	t	t	t	t	t	t	t	t	t
t	2863200	c	2926117	ns	t	CFT073	++++	t	t	t	t	t	t	t	t	t	t	t	t	t	t	t	t	t	t	t	t	t	t	t	t
a	2871211	g	2934128	ns	a	CFT073	++++	a	a	a	a	a	a	a	a	a	a	a	a	a	a	a	a	a	a	a	a	a	a	a	a
t	2871849	c	2934766	ns	t	CFT073	++++	t	t	t	t	t	t	t	t	t	t	t	t	t	t	t	t	t	t	t	t	t	t	t	t

Clone D base	Clone D site	CFT 073 base	CFT 073 site	mutation type[a]	Inferred ancestral base[b]	lineage inferred to mutate	Support level[c]	ED1a[d]	536	S88	APEC O1	UTI89	E2348/69	ATCC8739	K-12	HS	SMS3-5	IAI39	E24377A	SE11	55989	IAI1	Sakai	UMN026	B4 Sb227	F5 8401	SS Ss046	D1 Sd197	B18 BS512	F2a 2457T	F2a 301
g	2874724	a	2937641	ns	g	CFT073	++	g	g	g	g	g	g	g	g	g	g	g	g	g	g	g	g	g	g	g	g			g	g
t	2885814	c	2948732	s	c	clone D	++++	c	c	c	c	c	c	c	c	c	c	c	c	c	c	c	c	c	c	c	c	c	c	c	c
t	2890031	a	2952949	s	t	CFT073	++++	t	t	t	t	t	t	t	t	t	t	t	t	t	t	t	t	t	t	t	t	t	t	t	t
t	2896986	c	2959904	ns	c	clone D	++++	c	c	c	c	c	c	c	c	c	c	c	c	c	c	c	c	c	c	c	c	c	c	c	c
c	2927090	g	2990008	nc	g	clone D	++	g	g	c	g	c	c	t	g	g	c	c	g	g	g	g	g	t	g	g	g	g	g	g	g
t	2927353	g	2990271	nc	g	clone D	++++	g	g	g	g	g	g	g	g	g	g	g	g	g	g	g	g	g	g	g	g	g	g	g	g
c	2927412	t	2990330	nc	c	CFT073	++++	c	t	c	t	c	c	t	t	t	t	t	t	t	t	t	t	t	t	t	t	t	t	t	t
g	2927454	a	2990372	nc	g	CFT073	++++	g	a	g	a	g	g	a	a	g	a	a	a	a	a	a	a	a	g	a	a	a	g	a	a
a	2927730	t	2990648	nc	a	CFT073	++++	a	t	a	t	a	a	t	t	t	t	t	t	t	t	t	t	t	t	t	t	t	t	t	t
c	2927731	g	2990649	nc	c	CFT073	++	c	g	c	g	c	c	g	g	g	a	a	a	a	g	g	a	g	a	a	g	a	a	a	a
g	2927740	c	2990658	nc	g	CFT073	++	g	c	g	c	g	g	c	c	c	t	t	t	t	c	c	t	c	c	c	c	t	t	c	c
c	2927741	a	2990659	nc	c	CFT073	++++	c	a	c	a	c	c	a	a	a	a	a	a	a	a	a	a	a	a	a	a	a	a	a	a
a	2927749	g	2990667	nc	a	CFT073	++++	a	g	a	g	a	a	a	g	g	a	a	a	a	g	g	a	g	a	a	g	a	a	a	a
a	2927782	g	2990700	nc	a	CFT073	++++	a	g	a	g	a	a	g	g	g	a	a	a	a	g	g	a	g	g	g	g	a	a	g	g
t	2927788	c	2990707	nc	t	CFT073	++++	t	c	t	c	t	t	c	c	c	t	t	t	t	c	c	t	c	c	c	c	t	t	c	c
a	2928416	c	2991335	nc	a	CFT073	++	a	c	g	c	g	a	c	c	c	a	a	c	c	c	c	a	g	c	c	c	g	c	c	c
a	2928601	g	2991521	nc	g	clone D	++	g	g	a	g	a	a	a	g	g	g	g	g	g	a	g	g	g	a	g	a	a	a	g	g
t	2928602	c	2991522	nc	t	CFT073	++++	t	c	t	c	t	t	t	c	c	t	t	c	c	t	c	t	c	t	t	t	t	t	t	t
a	2928642	g	2991562	nc	g	clone D	++++	g	g	g	g	g	g	g	g	g	g	a	g	a	g	g	g	g	a	a	a	g	a	a	a
c	2928694	g	2991614	nc	c	CFT073	++++	c	c	c	g	c	c	c	g	g	c	c	g	g	g	g	g	g	g	g	g	g	g	g	g
c	2928875	t	2991795	nc	c	CFT073	++++	c	t	c	t	c	c	t	t	t	t	c	t	t	t	t	t	t	t	t	t	t	t	t	t
c	2929027	t	2991938	nc	c	CFT073	++++	c	t	c	c	c	c	t	t	t	t	t	t	t	c	c	t	t	t	t	t	t	t	t	t
g	2929030	a	2991941	nc	g	CFT073	++++	g	g	g	g	g	g	g	a	g	g	a	g	g	g	g	g	a	g	g	g	a	g	g	g
c	2929083	a	2991994	nc	c	CFT073	++++	c	c	c	c	c	c	c	a	c	c	c	c	c	c	c	c	c	c	c	c	c	c	c	c
a	2929118	g	2992029	nc	a	CFT073	++++	a	a	a	a	a	a	a	g	a	a	a	g	g	a	a	a	a	a	a	a	a	a	a	a
c	2929122	t	2992033	nc	c	CFT073	++++	c	t	c	t	c	c	c	t	c	c	t	t	t	c	c	t	c	c	c	c	c	c	c	c
a	2929123	c	2992034	nc	a	CFT073	++++	a	c	a	c	a	a	a	c	a	a	c	c	c	a	a	c	a	a	a	a	a	a	a	a
t	2929126	c	2992037	nc	t	CFT073	++++	t	c	t	c	t	t	t	c	t	t	c	c	c	t	t	c	t	t	t	t	t	t	t	t

Clone D base	Clone D site	CFT 073 base	CFT 073 site	mutation type[a]	Inferred ancestral base[b]	lineage inferred to mutate	Support level[c]	ED1a[d]	536	S88	APEC O1	UTI89	E2348/69	ATCC8739	K-12	HS	SMS3-5	IAI39	E24377A	SE11	55989	IAI1	Sakai	UMN026	B4 Sb227	F5 8401	SS Ss046	D1 Sd197	B18 BS512	F2a 2457T	F2a 301
t	2929257	a	2992254	nc	t	CFT073	++++	t	t	t	t	t	t	a	a	a	t	t	a	a	a	a	t	t	t	t	t	t	a	t	t
c	2929266	t	2992263	nc	c	CFT073	++++	c	t	c	t	c	c	t	t	t	c	c	t	t	t	t	c	c	t	c	c	c	t	c	c
t	2929267	c	2992264	nc	t	CFT073	++++	t	t	t	c	t	t	c	c	c	t	t	c	c	c	c	t	t	c	t	t	t	c	t	t
c	2929475	a	2992471	nc	c	CFT073	++++	c	c	c	c	c	c	c	c	c	c	c	c	c	c	c	c	c	c	c	c	c	c	c	c
g	2930220	c	2993220	nc	g	CFT073	++++	g	g	g	c	g	g	g	g	g	g	g	g	g	g	g	g	g	g	g	g	g	g	g	g
g	2930451	c	2993455	nc	g	CFT073	++++	g	g	g	c	g	g	g	g	g	g	g	g	g	g	g	g	g	g	g	g	g	g	g	g
g	2955443	a	3018449	nc	g	CFT073	++++	g	g	g	g	g	g	g	g	g	g	g	g	g	g	g	g	g	g	g	g	g	g	g	g
g	2955497	a	3018503	nc	g	CFT073	++++	g	g	g	g	g	g	g	g	g	g	g	g	g	g	g	g	g	g	g	g	g	g	g	g
g	2962639	a	3074078	ns	g	CFT073	++++	g	g	g	g	g	g	g	g	g	g	g	g	g	g	g	g	g	g	g	g	g	g	g	g
a	2966998	t	3078437	ns	t	clone D	++++	t	t	t	t	t	t	t	t	t	t	t	t	t	t	t	t	t	t	t	t	t	t	t	t
t	2968995	c	3080434	s	c	clone D	++	c	c	c	c	c	c				c	c					c	c				c			
a	2995994	g	3107433	s	g	clone D	++++	g	g	g	g	g	g	g	g	g	g	g	g	g	g	g	g	g	g	g	g	g	g	g	g
t	3029683	c	3141123	s	c	clone D	++	c	c	c	c		c		c	c	c	c	c	c	c	c	c	c	c	c	c		c	c	c
t	3061684	g	3173132	ns	t	CFT073	++	t	t	t	t	t	t	t	t	t	t	t	t	t	t	t	t	t			t	t			
g	3065845	a	3177293	nc	g	CFT073	++	g		g	g	g		g	g	g			g	g	g	g	g	g	g		g		g		
c	3067813	a	3179261	ns	c	CFT073	++	c	c	c	c	c					c					c	c	c				c			
c	3082607	t	3194070	ns	c	CFT073	++++	c	c	c	c	c	c	c	c	c	c	c	c	c	c	c	c	t	c	t	c	c	c	t	t
a	3086823	g	3198288	ns	a	CFT073	++++	a	a	a	a	a	a	a	a	a	a	a	a	a	a	a	a	a	a	a	a	a	a	a	a
t	3098292	g	3209757	ns	t	CFT073	++++	t	t	t	t	t	t	t	t	t	t	t	t	t	t	t	t	t	t	t	t	t		t	t
c	3115270	t	3226734	s	c	CFT073	++		c	c	c	c							c												
a	3116851	c	3228315	s	a	CFT073	++		a	a	a	a							a												
a	3128970	g	3240434	ns	g	clone D	++		g	g	g	g																			
c	3132900	t	3244364	s	t	clone D	++		c	t	t	t																			
t	3155664	g	3267126	ns	g	clone D	++++	g	g	g	g	g	g	g	g	g	g	g	g	g	g	g	g	g	g	g	g	g	g	g	g
a	3168315	c	3279777	ns	a	CFT073	++++	a	a	a	a	a	a	a	a	a	a	a	a	a	a	a	c	a	a	a	a	a	a	a	a
g	3180307	a	3292480	s	g	CFT073	++++	g	g	g	g	g	g	g	g	g	g	g	g	g	g	g	g	g	g	g	g		g	g	g
g	3185198	a	3297371	ns	g	CFT073	++	g	g	g	g	g	g	g	g	g	g	g	g	g	g	g	g	g	g	g	g			g	g
a	3187493	g	3299666	s	a	CFT073	++	a	a	a	a	a	a	a	a	a	a	a	a	a	a	a	a	a	a	a	a			a	a

Clone D base	Clone D site	CFT 073 base	CFT 073 site	mutation type[a]	Inferred ancestral base[b]	lineage inferred to mutate	Support level[c]	ED1a[d]	536	S88	APEC O1	UTI89	E2348/69	ATCC8739	K-12	HS	SMS3-5	IAI39	E24377A	SE11	55989	IAI1	Sakai	UMN026	B4 Sb227	F5 8401	SS Ss046	D1 Sd197	B18 BS512	F2a 2457T	F2a 301
a	3196692	g	3308865	nc	g	clone D	++++	g	g	g	g	g	g	g	g	g	g	g	g	g	g	g	g	g	g		g				
t	3216776	c	3328949	s	c	clone D	++	c	t	c	c	c	c	c	c	c	c	c	c	c	c	c	c	c	c	c	c			c	c
t	3237513	c	3350397	s	c	clone D	++++	c	c	c	c	c	c	c	c	c	c	c	c	c	c	c	c	c	c	c	c	c	c	c	c
t	3255888	a	3368772	s	t	CFT073	++++	t	t	t	t	t	t	t	t	t	t	t	t	t	t	t	t	t	t	t	t	t	t	t	t
g	3256744	a	3369628	ns	g	CFT073	++++	g	g	g	g	g	g	g	g	g	g	g	g	g	g	g	g	g	g	g		g	g	g	g
a	3261350	t	3374232	s	t	clone D	++	t	t	t	t	t	t				t	t						t		t				t	t
g	3261353	a	3374233	ns	a	clone D	++	a	a	a	a	a	a				a	a						a		a				a	a
g	3266500	a	3379379	s	g	CFT073	++	g	g	g	g	g	g				t	g	g	g	g	g	g	g		g		g		g	g
c	3269374	a	3382253	nc	c	CFT073	++++	c	c	c	c	c	c	c	c	c	c	c	c	c	c	c	c	c	c	c	c	c	c	c	c
c	3269377	g	3382256	nc	c	CFT073	++++	c	c	c	c	c	c	c	c	c	c	c	c	c	c	c	c	c	c	c	c	c	c	c	c
t	3275727	c	3388606	s	t	CFT073	++++	t	t	t	t	t	c	c	c	c	c	c	c	c	c	c	c	c	c	c	c	c	c	c	c
t	3293438	c	3406317	nc	c	clone D	++	c	c	c	c		c	c				c	c		c		c	c	c		c	c	c	c	c
t	3294224	c	3407103	s	t	CFT073	++	t	c	c	c		c	t				c	c		c		c	t	c		c		c	t	t
g	3294230	t	3407109	s	g	CFT073	++	g	g	t	t		t	g				t	g		g		t	g	t		t		t	g	g
c	3294242	t	3407121	s	c	CFT073	++	c	c	c	c		c	c				c	c		c		c	c	c		c		c	c	c
g	3294267	a	3407146	ns	g	CFT073	++	g	g	a	a		g	g				a	g		g		g	g	g		g		g	g	g
g	3294270	a	3407149	ns	g	CFT073	++	g	g	a	a		a	g				a	g		g		a	g	a		a		a	g	g
c	3294281	t	3407160	s	c	CFT073	++	c	c	g	g		g	c				g	c		c		g	c	g		g		g	c	c
a	3294296	g	3407175	s	a	CFT073	++	a	g	g	g		g	a				g	g		g		g	a	g		g		g	a	a
g	3294299	t	3407178	s	g	CFT073	++	g	t	t	t		t	g				t	t		t		t	g	t		t		t	g	g
a	3294302	t	3407181	s	a	CFT073	++	a	c	c	c		c	a				c	c		c		c	a	c		c		c	a	a
t	3294332	c	3407211	s	t	CFT073	++	t	t	t	t		t	t				t	t		t		t	t	t		t		t	t	t
g	3294408	a	3407287	ns	g	CFT073	++	g	g	g	g		g	g				g	g		g		g	g	g		g		g	g	g
t	3294431	c	3407310	s	t	CFT073	++	t	c	c	c		c	t				c	c		c		c	t	c		c		c	c	c
t	3294435	c	3407314	ns	t	CFT073	++	t	c	c	c		c	t				c	c		c		c	t	c		c		c	c	c
t	3295195	c	3408074	ns	c	clone D	+			c	c							c						c		c	c	c		c	c
c	3295338	t	3408217	ns	c	CFT073	+			c	c							c						c		c	c	c		c	c
t	3295341	c	3408220	s	c	clone D	+			t	t							t						t		c	c	c		c	c

Clone D base	Clone D site	CFT 073 base	CFT 073 site	mutation type[a]	Inferred ancestral base[b]	lineage inferred to mutate	Support level[c]	ED1a[d]	536	S88	APEC O1	UTI89	E2348/69	ATCC8739	K-12	HS	SMS3-5	IAI39	E24377A	SE11	55989	IAI1	Sakai	UMN026	B4 Sb227	F5 8401	SS Ss046	D1 Sd197	B18 BS512	F2a 2457T	F2a 301
t	3295391	a	3408270	s	t	CFT073	+			t	t							t						t		c	c	c		c	c
a	3295410	g	3408289	ns	g	clone D	+			g	g							a						a		g	g	g		g	g
a	3296118	g	3408997	ns	a	CFT073	+			g	g							g						g		a	a	a		a	a
t	3296224	c	3409103	nc	t	CFT073	+			t	t							t						c		t	t	t		t	t
t	3296276	g	3409155	nc	t	CFT073	+			t	t							t						g		t	t	t		t	t
c	3296420	t	3409299	ns	c	CFT073	+			c	c							c						t		c	c			c	c
t	3296553	c	3409420	s	t	CFT073	+		c	t	t	c						t						t							
g	3296658	a	3409525	nc	a	clone D	+		a	a	a	a		a	a		a	a			a			a							
g	3296659	a	3409526	nc	a	clone D	+		a	a	a	a		a	a		a	a			a			a							
c	3297837	t	3428739	nc	c	CFT073	+		t	c	c	t						c						t							
a	3297854	g	3428756	nc	a	CFT073	+		g	a	a	g						a						g							
c	3297859	a	3428761	nc	c	CFT073	+		a	c	c	c						c						a							
a	3297865	g	3428767	nc	a	CFT073	+		g	a	a	a						a						g							
t	3297866	c	3428768	nc	t	CFT073	+		c	t	t	t						t						c							
g	3297872	a	3428774	nc	g	CFT073	+		a	g	g	g						g						a							
a	3297874	g	3428776	nc	a	CFT073	+		g	a	a	a						a						g							
g	3297887	a	3428789	ns	g	CFT073	+		a	g	g	g						g						a							
g	3297893	t	3428795	ns	g	CFT073	+		t	g	g	g						g						t							
a	3297894	c	3428796	ns	a	CFT073	+		c	a	a	a						a						c							
a	3297897	g	3428799	ns	a	CFT073	+		g	a	a	a						a						g							
t	3297901	c	3428803	s	t	CFT073	+		c	t	t	t						t						c							
c	3297910	t	3428812	s	t	clone D	+		t	t	t	t						t						t							
t	3297959	c	3428861	ns	t	CFT073	+		c	t	t	t						t						c							
g	3298070	a	3428972	ns	g	CFT073	+		a	g	g	g						g						a							
g	3298174	a	3429076	ns	g	CFT073	+		a	g	g	g						g						a							
t	3298175	c	3429077	ns	t	CFT073	+		c	t	t	c						t						c							
g	3298211	a	3429113	ns	g	CFT073	+		a	g	g	g						g						a							
c	3298288	t	3429190	s	c	CFT073	+		t	c	c	c						c						t							

Clone D base	Clone D site	CFT 073 base	CFT 073 site	mutation type[a]	Inferred ancestral base[b]	lineage inferred to mutate	Support level[c]	ED1a[d]	536	S88	APEC O1	UTI89	E2348/69	ATCC8739	K-12	HS	SMS3-5	IAI39	E24377A	SE11	55989	IAI1	Sakai	UMN026	B4 Sb227	F5 8401	SS Ss046	D1 Sd197	B18 BS512	F2a 2457T	F2a 301
t	3298303	c	3429205	s	t	CFT073	+		c	t	t	t						t						c							
a	3298472	g	3429374	nc	a	CFT073	+		g	a	a	g						a						g							
g	3298487	a	3429389	nc	g	CFT073	+		a	g	g	a						g						a							
a	3298490	t	3429392	nc	a	CFT073	+		t	a	a	t						a						t							
t	3298494	a	3429396	nc	t	CFT073	+		a	t	t	a						t						a							
a	3298495	t	3429397	nc	a	CFT073	+		t	a	a	t						a						t							
g	3298496	a	3429398	nc	g	CFT073	+		a	g	g	a						g						a							
c	3298498	t	3429400	nc	c	CFT073	+		c	c	c	c						c						t							
g	3298501	t	3429403	nc	g	CFT073	+		g	g	g	g						g						t							
g	3298505	a	3429408	nc	g	CFT073	+		a	g	g	a						g						a							
g	3298512	a	3429415	nc	g	CFT073	+		a	g	g	a						g						a							
g	3298524	t	3429427	nc	g	CFT073	+		t	g	g	t						g						t							
g	3298528	a	3429431	nc	g	CFT073	+		a	g	g	a						g						a							
a	3299818	t	3437355	nc	a	CFT073	+		t	a	a	a						a						t							
g	3299820	c	3437357	nc	g	CFT073	+		t	g	g	g						g						c							
c	3299821	t	3437358	nc	c	CFT073	+		c	c	c	c						c						t							
c	3299839	t	3437376	nc	c	CFT073	+		c	c	c	c						c						t							
t	3299848	a	3437385	nc	t	CFT073	+		t	t	t	t						t						a							
t	3299851	c	3437388	nc	c	clone D	+		c	c	c	c						c						c							
t	3299853	g	3437390	nc	g	clone D	+		g	g	g	g						g						g							
c	3299854	a	3437391	nc	a	clone D	+		c	a	a	a						a						a							
t	3299855	g	3437392	nc	g	clone D	+		g	g	g	g						g						g							
g	3299856	c	3437393	nc	c	clone D	+		c	a	a	a						a						c							
g	3299858	t	3437395	nc	t	clone D	+		t	-	-	-						-						t							
a	3299859	c	3437396	nc	c	clone D	+		c	-	-	-						-						c							
g	3299863	a	3437400	nc	g	CFT073	+		a	g	g	g						g						a							
t	3299865	c	3437402	nc	t	CFT073	+		c	t	t	t						t						c							
g	3299866	a	3437403	nc	g	CFT073	+		a	g	g	g						g						a							

Clone D base	Clone D site	CFT 073 base	CFT 073 site	mutation type[a]	Inferred ancestral base[b]	lineage inferred to mutate	Support level[c]	ED1a[d]	536	S88	APEC O1	UTI89	E2348/69	ATCC8739	K-12	HS	SMS3-5	IAI39	E24377A	SE11	55989	IAI1	Sakai	UMN026	B4 Sb227	F5 8401	SS Ss046	D1 Sd197	B18 BS512	F2a 2457T	F2a 301
a	3299872	t	3437409	nc	a	CFT073	+		t	a	a	a						a						t							
a	3299878	t	3437415	nc	a	CFT073	+		a	a	a	a						a						t							
a	3299881	t	3437418	nc	a	CFT073	+		c	a	a	a						a						t							
g	3299896	a	3437433	nc	a	clone D	+		a	a	a	g						g						a							
c	3299903	t	3437440	nc	t	clone D	+		t	t	t	c						c						t							
t	3299917	a	3437454	nc	t	CFT073	+		a	t	t	t						t						-							
g	3299921	a	3437458	nc	a	clone D	+		a	a	a	g						g						-							
a	3299927	g	3437464	nc	g	clone D	+		g	g	g	g						a						g							
t	3299937	a	3437474	nc	t	CFT073	+		t	t	t	t						t						t							
a	3299939	g	3437476	nc	a	CFT073	+		a	a	a	a						a						g							
c	3300156	t	3437692	nc	t	clone D	+		t	t	t	t						c						t							
t	3300217	g	3437753	nc	t	CFT073	+		c	c	c	c						t						c							
g	3300438	c	3437975	s	g	CFT073	+		g	g	g	g						g						c							
a	3300442	g	3437979	ns	g	clone D	+		g	g	g	g						a						g							
c	3300450	t	3437987	s	t	clone D	+		t	t	t	t						c						t							
c	3300651	t	3438188	nc	t	clone D	+		c	t	t	c						t						t							
t	3300656	c	3438193	nc	t	CFT073	+		t	t	t	t						t						c							
t	3300661	c	3438198	nc	c	clone D	+		c	c	c	c						t						c							
t	3300662	a	3438199	nc	a	clone D	+		a	a	a	a						a						a							
c	3300667	t	3438204	nc	c	CFT073	+		c	t	t	c						c						t							
a	3300669	g	3438206	nc	a	CFT073	+		a	a	a	a						a						g							
a	3300675	g	3438212	nc	a	CFT073	+		a	a	a	a						a						g							
t	3300676	g	3438213	nc	t	CFT073	+		t	t	t	t						t						g							
a	3300677	g	3438214	nc	a	CFT073	+		a	g	g	a						a						g							
c	3300678	t	3438215	nc	c	CFT073	+		c	c	c	c						c						t							
t	3300700	c	3438237	nc	t	CFT073	+		t	t	t	t						t						c							
c	3300736	a	3438273	nc	c	CFT073	+		c	c	c	c						c						a							
a	3300758	g	3438295	nc	a	CFT073	+		a	a	a	a						a						g							

Clone D base	Clone D site	CFT 073 base	CFT 073 site	mutation type[a]	Inferred ancestral base[b]	lineage inferred to mutate	Support level[c]	ED1a[d]	536	S88	APEC O1	UTI89	E2348/69	ATCC8739	K-12	HS	SMS3-5	IAI39	E24377A	SE11	55989	IAI1	Sakai	UMN026	B4 Sb227	F5 8401	SS Ss046	D1 Sd197	B18 BS512	F2a 2457T	F2a 301
c	3300770	a	3438307	nc	c	CFT073	+		c	a	a	c						c						a							
a	3300773	g	3438310	nc	a	CFT073	+		a	g	g	a						a						g							
g	3300799	t	3438336	nc	g	CFT073	+		t	g	g	g						g						t							
c	3300804	t	3438341	nc	c	CFT073	+		c	t	t	c						c						t							
c	3300827	a	3438364	nc	c	CFT073	+		c	a	a	c						c						a							
c	3300844	t	3438381	nc	c	CFT073	+		t	c	c	c						c						t							
a	3300968	g	3438505	ns	a	CFT073	+		a	g	g	a						a						g							
c	3300982	g	3438519	s	c	CFT073	+		c	g	g	c						c						g							
g	3300994	a	3438531	s	g	CFT073	+		g	g	g	g						g						a							
t	3301018	c	3438555	s	c	clone D	+		t	c	c	c						t						c							
a	3301024	c	3438561	s	a	CFT073	+		a	c	c	a						a						c							
a	3301053	c	3438590	ns	a	CFT073	+		a	c	c	a						a						c							
g	3301058	a	3438595	ns	a	clone D	+		g	a	a	a						g						a							
t	3301072	a	3438609	s	t	CFT073	+		t	a	a	t						t						a							
g	3301081	a	3438618	s	g	CFT073	+		g	a	a	g						g						a							
a	3301084	g	3438621	s	a	CFT073	+		a	g	g	a						a						g							
c	3301103	t	3438640	ns	c	CFT073	+		t	c	c	t						c						t							
a	3301134	g	3438671	ns	g	clone D	+		g	g	g	a						g						g							
g	3301207	a	3438744	nc	g	CFT073	+		g			g						g						a							
t	3301259	g	3438796	nc	t	CFT073	+		t			t												g							
c	3301601	t	3439138	nc	t	clone D	+																	t							
t	3301681	g	3439218	nc	g	clone D	++	g	g	g	g	g	g				g	g	g		g		g	g	g	g	g		g	g	g
a	3301806	g	3439343	nc	g	clone D	++	g	g	g	g	g	g				g	g	g		g		g	g	g	g	g		g	g	g
c	3301871	a	3439408	nc	c	CFT073	++	c	c	c	c	c	c				c	c	c		c		c	a	c	c	c		c	c	c
c	3302088	t	3439625	nc	c	CFT073	++	c	c	c	c	c	c				c	c	c		c		c	t	c	c	c		c	c	c
g	3302195	t	3439732	nc	g	CFT073	++	g	g	g	g	g	g				g	g	g		g		g	t	g	g	g		g	g	g
c	3302450	t	3439986	nc	t	clone D	++	t	c	c	c	c	t				c	c	t		t		c	t	t	t	t		t	t	t
a	3302473	c	3440009	nc	c	clone D	++	c	t	c	c	t	c				c	c	c		c		c	c	c	c	c		c	c	c

Clone D base	Clone D site	CFT 073 base	CFT 073 site	mutation type[a]	Inferred ancestral base[b]	lineage inferred to mutate	Support level[c]	ED1a[d]	536	S88	APEC O1	UTI89	E2348/69	ATCC8739	K-12	HS	SMS3-5	IAI39	E24377A	SE11	55989	IAI1	Sakai	UMN026	B4 Sb227	F5 8401	SS Ss046	D1 Sd197	B18 BS512	F2a 2457T	F2a 301
c	3302474	g	3440010	nc	g	clone D	++	g	g	g	g	g	g				g	g	g		g		g	g	g	g	g		g	g	g
a	3302475	c	3440011	nc	c	clone D	++	c	c	c	c	c	c				c	c	c		c		c	c	c	c	c		c	c	c
c	3302503	t	3440039	nc	t	clone D	++	t	t	c	c	t	t				t	c	t		t		t	c	t	t	t		t	t	t
g	3302505	t	3440041	nc	g	CFT073	++	g	g	g	g	g	g				g	g	g		g		g	g	g	g	g		g	g	g
t	3302546	a	3440082	nc	a	clone D	++	a	a	a	a	a	a				a	t	a		a		a	a	a	a	a		a	a	a
c	3302780	t	3440316	nc	c	CFT073	++	c	c	c	c	c	c				c	c	c		c		c	t	c	c	c		c	c	c
a	3302812	g	3440348	nc	a	CFT073	++	a	g	g	g	g	a				a	a	a		a		a	g	a	a	a		a	a	a
t	3302823	a	3440359	nc	a	clone D	++	a	a	a	a	a	a				a	a	a		a		a	a	a	a	a		a	a	a
a	3302843	t	3440379	nc	a	CFT073	++	a	a	a	a	a	a				a	a	a		a		a	t	a	a	a		a	a	a
a	3302954	g	3440490	nc	g	clone D	++	g	g	a	a	g	g				g	a	g		g		g	g	g	g	g		g	g	g
g	3303385	a	3440921	nc	g	CFT073	+											g						a							
t	3303388	c	3440924	nc	t	CFT073	+											t						c							
t	3303391	c	3440927	nc	t	CFT073	+											t						c							
t	3303526	c	3441062	nc	c	clone D	+											c						c							
g	3303660	a	3441196	nc	g	CFT073	+										g	g						a							
t	3304080	c	3441608	ns	t	CFT073	+										t	t						c							
c	3304283	t	3441811	ns	c	CFT073	+										c	c						t							
g	3304329	a	3441857	ns	g	CFT073	+										g	g						a							
t	3304390	g	3441918	ns	t	CFT073	+										t	t						g							
t	3304962	c	3442490	nc	t	CFT073	+											t						c							
c	3305225	t	3442753	s	c	CFT073	+											c						t							
a	3305488	t	3443016	s	a	CFT073	+											a						t							
t	3305778	c	3443306	ns	c	clone D	+											c						c							
a	3306079	g	3443607	s	a	CFT073	+											a						g							
t	3306278	c	3443806	ns	t	CFT073	+											t						c							
a	3306947	g	3444082	nc	?	?	+/-	c																c							
g	3307024	a	3444159	s	?	?	+/-	t																t							
c	3307069	t	3444204	s	c	CFT073	++	c																c							

Clone D base	Clone D site	CFT 073 base	CFT 073 site	mutation type[a]	Inferred ancestral base[b]	lineage inferred to mutate	Support level[c]	ED1a[d]	536	S88	APEC O1	UTI89	E2348/69	ATCC8739	K-12	HS	SMS3-5	IAI39	E24377A	SE11	55989	IAI1	Sakai	UMN026	B4 Sb227	F5 8401	SS Ss046	D1 Sd197	B18 BS512	F2a 2457T	F2a 301
t	3307156	c	3444291	s	t	CFT073	++	t																t							
c	3307182	g	3444317	ns	?	?	++	t																t							
t	3307192	c	3444327	s	?	?	+/-	a																a							
c	3307195	a	3444330	s	a	clone D	++	a																a							
c	3307231	t	3444366	s	t	clone D	++	t																t							
t	3307261	c	3444396	s	c	clone D	++	c																c							
c	3307270	t	3444405	s	t	clone D	++	t																t							
a	3307321	t	3444455	s	t	clone D	++	t																t							
c	3307344	t	3444478	ns	?	?	+/-	a																a							
c	3307377	a	3444511	ns	?	?	+/-	g																g							
g	3307432	a	3444566	ns	g	CFT073	++	g																g							
a	3307445	t	3444579	ns	a	CFT073	++	a																a							
c	3307449	t	3444583	s	c	CFT073	++	c																c							
c	3307461	t	3444595	s	?	?	+/-	g																g							
g	3307470	t	3444604	s	?	?	+/-	c																c							
g	3307490	t	3444624	ns	?	?	+/-	a																a							
c	3307498	t	3444632	ns	?	?	+/-	a																a							
a	3307518	g	3444652	s	?	?	+/-	t																t							
a	3307524	g	3444658	s	?	?	++	t																t							
a	3307563	g	3444697	s	?	?	+/-	t																t							
a	3307564	g	3444698	ns	?	?	++	t																t							
a	3307566	c	3444700	ns	c	clone D	++	c																c							
c	3307613	t	3444747	ns	?	?	+/-	g																g							
c	3307648	a	3444782	ns	?	?	+/-	t																t							
c	3307680	t	3444814	s	?	?	+/-	g																g							
g	3307685	a	3444819	ns	g	CFT073	++	g																g							
t	3307692	c	3444826	s	c	clone D	++	c																c							
t	3307704	c	3444838	s	c	clone D	++	c																c							

Clone D base	Clone D site	CFT 073 base	CFT 073 site	mutation type[a]	Inferred ancestral base[b]	lineage inferred to mutate	Support level[c]	ED1a[d]	536	S88	APEC O1	UTI89	E2348/69	ATCC8739	K-12	HS	SMS3-5	IAI39	E24377A	SE11	55989	IAI1	Sakai	UMN026	B4 Sb227	F5 8401	SS Ss046	D1 Sd197	B18 BS512	F2a 2457T	F2a 301
t	3307731	c	3444865	s	?	?	+/-	a																g							
g	3307764	a	3444898	s	g	CFT073	++	g																g							
a	3307818	t	3444952	s	?	?	+/-	g																g							
t	3307824	c	3444958	s	?	?	+/-	a																a							
a	3307866	g	3445000	s	?	?	++	t																t							
t	3307881	c	3445015	s	c	clone D	++	c																c							
g	3307886	a	3445020	ns	a	clone D	++	a																a							
g	3307908	a	3445042	s	?	?	+/-	c																c							
g	3307944	t	3445078	s	?	?	+/-	a																a							
t	3307956	c	3445090	s	?	?	+/-	g																g							
a	3307966	c	3445100	s	c	clone D	++	c																c							
a	3307998	g	3445132	s	?	?	+/-	c																c							
a	3308022	c	3445156	s	?	?	+/-	t																t							
g	3308073	a	3445207	ns	?	?	++	t																t							
c	3308155	t	3445289	s	t	clone D	++	t																t							
t	3308191	c	3445325	ns	c	clone D	++	c																c							
a	3308197	t	3445331	ns	?	?	+/-	g																g							
g	3308200	a	3445334	ns	?	?	+/-	t																t							
g	3308220	a	3445354	s	a	clone D	++	a																a							
c	3308660	t	3445794	nc	c	CFT073	+		g																						
a	3308694	g	3445828	nc	a	CFT073	+		t																						
t	3308827	g	3445961	s	g	clone D	+		g																						
c	3309474	a	3446608	ns	c	CFT073	+		c																						
t	3309942	c	3447076	ns	t	CFT073	+																								
g	3310139	a	3447273	ns	g	CFT073	+																								
a	3310171	g	3447305	s	a	CFT073	+																								
t	3310870	c	3448004	nc	t	CFT073	+																								
g	3311048	t	3448182	nc	t	clone D	++	t																t	t				t		

Clone D base	Clone D site	CFT 073 base	CFT 073 site	mutation type[a]	Inferred ancestral base[b]	lineage inferred to mutate	Support level[c]	ED1a[d]	536	S88	APEC O1	UTI89	E2348/69	ATCC8739	K-12	HS	SMS3-5	IAI39	E24377A	SE11	55989	IAI1	Sakai	UMN026	B4 Sb227	F5 8401	SS Ss046	D1 Sd197	B18 BS512	F2a 2457T	F2a 301
a	3311422	t	3450966	nc	t	clone D	++	t													t		t	t	t				t		
t	3311423	c	3450967	nc	?	?	+/-	g													g		g	g	a				a		
a	3311686	c	3451235	s	?	?	++	t													t		t	t							
c	3311716	t	3451265	s	?	?	+/-	g													g		g	g							
g	3311782	a	3451331	s	a	clone D	++	a													a		a	a							
g	3311819	a	3451368	ns	a	clone D	+						a		a		a	a	a				a	a	a	a	a	a	a	a	a
t	3311914	c	3451463	s	c	clone D	+						c		c		c	c	c				c	c	c	c	c	c	c	c	c
t	3311941	c	3451490	nc	t	CFT073	+						g		g		g	g	g				g	g	g	g	g	g	g	g	g
g	3312233	a	3451782	ns	g	CFT073	+								t		t	t	t				t	t	t	t	t	t	t	t	t
c	3312309	a	3451858	ns	a	clone D	+								a		a	a	a				a	a	a	a	a	a	a	a	a
a	3312316	g	3451865	ns	a	CFT073	+								a		a	a	a				a	a	a	a	a	a	a	a	a
c	3312383	a	3451932	ns	c	CFT073	+								g		g	g	g				g	g	g	g	g	g	g	g	g
a	3312384	g	3451933	ns	a	CFT073	+								t		t	t	t				t	t	t	t	t	t	t	t	t
t	3312390	c	3451939	s	t	CFT073	+								t		t	t	t				t	t	t	t	t	t	t	t	t
c	3312394	t	3451943	ns	c	CFT073	+								g		g	g	g				g	g	g	g	g	g	g	g	g
t	3312426	c	3451975	s	t	CFT073	+								a		a	a	a				a	a	a	a	a	a	a	a	a
a	3312716	g	3452265	ns	a	CFT073	+		t			t			t		t	t	t				t	t	t	t	t	t	t	t	t
a	3312792	g	3452341	s	g	clone D	++	g	g	g	g	g			g		g	g	g				g	g	g	g	g	g	g	g	g
a	3312987	g	3452536	ns	?	?	+/-	c	c	c	c	c	c		c		c	c	c				c	c	c	c	c	c	c	c	c
c	3313102	t	3452651	s	t	clone D	++	t	t	t	t	t	t		t		t	t	t				t	t	t	t	t	t	t	t	t
t	3316143	c	3455659	nc	c	clone D	+														c			c	c		c			c	c
a	3316224	g	3455740	s	g	clone D	+														g			g	g		g			g	g
t	3316401	a	3455917	s	t	CFT073	+														t			a	t		t		t	t	t
c	3316496	t	3456012	ns	c	CFT073	+														c			c	c		c		c	c	c
a	3319441	g	3456467	s	g	clone D	+	t					c								g			g	g		g		g	g	g
c	3319594	g	3456620	s	g	clone D	++	g					g								g			g	g		g		g	g	g
c	3320101	a	3457127	ns	c	CFT073	++	c					c								c			a	c		c		c	c	c
c	3320544	t	3457570	ns	c	CFT073	+																	t						c	c

Clone D base	Clone D site	CFT 073 base	CFT 073 site	mutation type[a]	Inferred ancestral base[b]	lineage inferred to mutate	Support level[c]	ED1a[d]	536	S88	APEC O1	UTI89	E2348/69	ATCC8739	K-12	HS	SMS3-5	IAI39	E24377A	SE11	55989	IAI1	Sakai	UMN026	B4 Sb227	F5 8401	SS Ss046	D1 Sd197	B18 BS512	F2a 2457T	F2a 301
g	3320698	a	3457724	s	g	CFT073	+																	a						g	g
c	3320950	t	3457976	s	t	clone D	+											c						t						t	t
t	3321039	c	3458065	ns	t	CFT073	+											t						c						t	t
g	3321218	a	3458244	ns	g	CFT073	+											g						a						g	g
t	3321352	a	3458378	s	a	clone D	+											t						a						a	a
g	3321390	a	3458416	ns	a	clone D	+											g						a						a	a
c	3321403	t	3458429	s	c	CFT073	+											c						c						c	c
t	3321489	a	3458515	ns	t	CFT073	+											t						a						t	t
g	3321796	a	3458822	s	g	CFT073	+											g						a						g	g
t	3322170	c	3459196	ns	t	CFT073	+											t						c						t	t
t	3322270	a	3459296	s	a	clone D	+											t						a						a	a
t	3322805	c	3459831	ns	t	CFT073	+											t						c						t	t
a	3322831	g	3459857	s	a	CFT073	+											a						g						a	a
g	3323432	a	3460458	nc	g	CFT073	+											g						g			g			g	g
a	3323620	t	3460646	nc	t	clone D	++	t										t			t			t			t			t	t
g	3323879	a	3460905	nc	g	CFT073	++	g										g			g			a			g			g	g
c	3324164	a	3461190	nc	c	CFT073	++	c									c	c			c			a	c	c	c		c	c	c
a	3324179	t	3461205	s	t	clone D	++	t									t	a			t			t	t	t	t		t	t	t
t	3324208	g	3461234	s	g	clone D	++	g									t	t			g			g	g	g	g		g	g	g
c	3324230	a	3461256	s	a	clone D	++	a									a	c			a			a	a	a	a		a	a	a
t	3324233	a	3461259	s	a	clone D	++	a									a	t			a			a	a	a	a		a	a	a
t	3324260	a	3461286	s	a	clone D	++	a									t	t			a			a	a	a	a		a	a	a
c	3324272	t	3461298	s	t	clone D	++	t									c	c			t			t	t	t	t		t	t	t
g	3324287	a	3461313	s	a	clone D	++	a									g	g			a			a	a	a	a		a	a	a
t	3324308	c	3461334	s	c	clone D	++	c									t	t			c			c	c	c	c		c	c	c
t	3324313	c	3461339	ns	c	clone D	++	c									t	t			c			c	c	c	c		c	c	c
a	3324329	t	3461355	s	t	clone D	++	t									a	a			t			t	t	t	t		t	t	t
a	3324344	c	3461370	ns	c	clone D	++	c									a	a			c			c	c	c	c		c	c	c

Clone D base	Clone D site	CFT 073 base	CFT 073 site	mutation type[a]	Inferred ancestral base[b]	lineage inferred to mutate	Support level[c]	ED1a[d]	536	S88	APEC O1	UTI89	E2348/69	ATCC8739	K-12	HS	SMS3-5	IAI39	E24377A	SE11	55989	IAI1	Sakai	UMN026	B4 Sb227	F5 8401	SS Ss046	D1 Sd197	B18 BS512	F2a 2457T	F2a 301
t	3324345	c	3461371	ns	c	clone D	++	c									t	t			c			c	c	c	c		c	c	c
g	3324350	a	3461376	ns	a	clone D	++	a									g	g			a			a	a	a	a		a	a	a
c	3324352	t	3461378	ns	t	clone D	++	t									c	c			t			t	t	t	t		t	t	t
g	3324355	t	3461381	ns	t	clone D	++	t									g	g			t			t	t	t	t		t	t	t
c	3324356	a	3461382	ns	a	clone D	++	a									c	c			a			a	a	a	a		a	a	a
g	3324358	a	3461384	ns	a	clone D	++	a									g	g			a			a	a	a	a		a	a	a
c	3324362	g	3461388	s	g	clone D	++	g									c	c			g			g	g	g	g		g	g	g
g	3324368	a	3461394	s	a	clone D	++	a									g	g			a			a	a	a	a		a	a	a
a	3324371	g	3461397	s	g	clone D	++	g									a	a			g			g	g	g	g		g	g	g
g	3324374	a	3461400	s	a	clone D	++	a									g	g			a			a	a	a	a		a	a	a
c	3324379	t	3461405	ns	t	clone D	++	t									c	c			t			t	t	t	t		t	t	t
g	3324380	a	3461406	ns	a	clone D	++	a									g	g			a			a	a	a	a		a	a	a
c	3324382	t	3461408	ns	t	clone D	++	t									c	c			t			t	t	t	t		t	t	t
c	3324383	a	3461409	ns	a	clone D	++	a									c	c			a			a	a	a	a		a	a	a
t	3324385	c	3461411	ns	c	clone D	++	c									t	t			c			c	c	c	c		c	c	c
a	3324388	t	3461414	ns	t	clone D	++	t									a	a			t			t	t	t	t		t	t	t
g	3324389	a	3461415	s	a	clone D	++	a									g	g			a			a	a	a	a		a	a	a
g	3324394	c	3461420	ns	c	clone D	++	c									g	g			c			c	c	c	c		c	c	c
c	3324395	t	3461421	s	t	clone D	++	t									c	c			t			t	t	t	t		t	t	t
g	3324398	a	3461424	s	a	clone D	++	a									g	g			a			a	a	a	a		a	a	a
c	3324404	t	3461430	s	t	clone D	++	t									c	c			t			t	t	t	t		t	t	t
a	3324410	g	3461436	s	g	clone D	++	g									a	a			g			g	g	g	g		g	g	g
g	3324413	a	3461439	s	a	clone D	++	a									g	g			a			a	a	a	a		a	a	a
g	3324416	a	3461442	s	a	clone D	++	a									g	g			a			a	a	a	a		a	a	a
c	3324428	t	3461454	s	t	clone D	++	t									c	c			t			t	t	t	t		t	t	t
g	3324431	a	3461457	s	a	clone D	++	a									g	g			a			a	a	a	a		a	a	a
g	3324446	t	3461472	ns	t	clone D	++	t									g	g			t			t	t	t	t		t	t	t
t	3324452	c	3461478	s	c	clone D	++	c									t	t			c			c	c	c	c		c	c	c

Clone D base	Clone D site	CFT 073 base	CFT 073 site	mutation type[a]	Inferred ancestral base[b]	lineage inferred to mutate	Support level[c]	ED1a[d]	536	S88	APEC O1	UTI89	E2348/69	ATCC8739	K-12	HS	SMS3-5	IAI39	E24377A	SE11	55989	IAI1	Sakai	UMN026	B4 Sb227	F5 8401	SS Ss046	D1 Sd197	B18 BS512	F2a 2457T	F2a 301
c	3324459	t	3461485	ns	t	clone D	++	t									c	c			t			t	t	t	t		t	t	t
c	3324460	t	3461486	ns	t	clone D	++	t									c	c			t			t	t	t	t		t	t	t
g	3324467	a	3461493	s	a	clone D	++	a									g	g			a			a	a	a	a		a	a	a
t	3324479	c	3461505	s	c	clone D	++	c									t	t			c			c	c	c	c		c	c	c
g	3324485	a	3461511	s	a	clone D	++	a									g	g			a			a	a	a	a		a	a	a
c	3324490	a	3461516	ns	a	clone D	++	a									c	c			a			a	a	a	a		a	a	a
g	3324492	t	3461518	ns	t	clone D	++	t									g	g			t			t	t	t	t		t	t	t
t	3324493	c	3461519	ns	c	clone D	++	c									t	t			c			c	c	c	c		c	c	c
a	3324500	g	3461526	s	g	clone D	++	g									a	a			g			g	g	g	g		g	g	g
g	3324503	a	3461529	s	a	clone D	++	a									g	g			a			a	a	a	a		a	a	a
c	3324509	t	3461535	s	t	clone D	++	t									c	c			t			t	t	t	t		t	t	t
g	3324515	t	3461541	ns	t	clone D	++	t									g	g			t			t	t	t	t		t	t	t
g	3324516	t	3461542	ns	t	clone D	++	t									g	g			t			t	t	t	t		t	t	t
t	3324517	g	3461543	ns	g	clone D	++	g									t	t			g			g	g	g	g		g	g	g
a	3324536	g	3461562	s	g	clone D	++	g									a	a			g			g	g	g	g		g	g	g
g	3324548	a	3461574	s	a	clone D	++	a									g	g			a			a	a	a	a		a	a	a
t	3324560	a	3461586	s	a	clone D	++	a									t	t			a			a	a	a	a		a	a	a
a	3324581	c	3461607	s	c	clone D	++	c									a	a			c			c	c	c	c		c	c	c
a	3324584	g	3461610	s	g	clone D	++	g									a	a			g			g	g	g	g		g	g	g
t	3324626	c	3461652	ns	c	clone D	++	c									t	t			c			c	c	c	c		c	c	c
t	3324627	c	3461653	ns	c	clone D	++	c									t	t			c			c	c	c	c		c	c	c
a	3324636	t	3461662	ns	t	clone D	++	t									a	a			t			t	t	t	t		t	t	t
c	3324637	t	3461663	ns	t	clone D	++	t									c	c			t			t	t	t	t		t	t	t
g	3324641	a	3461667	s	a	clone D	++	a									g	g			a			a	a	a	a		a	a	a
g	3324663	c	3461689	ns	c	clone D	++	c									g	g			c			c	c	c	c		c	c	c
c	3324664	t	3461690	ns	t	clone D	++	t									c	c			t			t	t	t	t		t	t	t
c	3324665	t	3461691	ns	t	clone D	++	t									c	c			t			t	t	t	t		t	t	t
a	3324666	t	3461692	ns	t	clone D	++	t									a	a			t			t	t	t	t		t	t	t

Clone D base	Clone D site	CFT 073 base	CFT 073 site	mutation type[a]	Inferred ancestral base[b]	lineage inferred to mutate	Support level[c]	ED1a[d]	536	S88	APEC O1	UTI89	E2348/69	ATCC8739	K-12	HS	SMS3-5	IAI39	E24377A	SE11	55989	IAI1	Sakai	UMN026	B4 Sb227	F5 8401	SS Ss046	D1 Sd197	B18 BS512	F2a 2457T	F2a 301
c	3324679	t	3461705	ns	t	clone D	++	t									c	c			t			t	t	t	t		t	t	t
a	3324680	g	3461706	s	g	clone D	++	g									a	a			g			g	g	g	g		g	g	g
t	3324685	g	3461711	ns	g	clone D	++	g									t	t			g			g	g	g	g		g	g	g
a	3324689	g	3461715	s	g	clone D	++	g									a	a			g			g	g	g	g		g	g	g
g	3324692	a	3461718	s	a	clone D	++	a									g	g			a			a	a	a	a		a	a	a
g	3324695	t	3461721	s	t	clone D	++	t									g	g			t			t	t	t	t		t	t	t
g	3324707	a	3461733	s	a	clone D	++	a									g	g			a			a	a	a	a		a	a	a
g	3324710	c	3461736	s	c	clone D	++	c									a	g			c			c	c	c	c		c	c	c
a	3324716	g	3461742	s	g	clone D	++	g									a	a			g			g	g	g	g		g	g	g
a	3324719	g	3461745	ns	g	clone D	++	g									a	a			g			g	g	g	g		g	g	g
t	3324721	c	3461747	ns	c	clone D	++	c									t	t			c			c	c	c	c		c	c	c
g	3324722	a	3461748	s	a	clone D	++	a									g	g			a			a	a	a	a		a	a	a
a	3324725	g	3461751	s	g	clone D	++	g									a	a			g			g	g	g	g		g	g	g
c	3324743	t	3461769	s	t	clone D	++	t									c	c			t			t	t	t	t		t	t	t
g	3324776	a	3461802	s	a	clone D	++	a									g	g			a			a	a	a	a		a	a	a
a	3324794	g	3461820	s	g	clone D	++	g									a	a			g			g	g	g	g		g	g	g
t	3324803	c	3461829	s	c	clone D	++	c									t	t			c			c	c	c	c		c	c	c
a	3324806	g	3461832	s	g	clone D	++	g									a	a			g			g	g	g	g		g	g	g
a	3324827	t	3461853	s	t	clone D	++	t									a	a			t			t	t	t	t		t	t	t
g	3324851	a	3461877	s	a	clone D	++	a									g	g			a			a	a	a	a		a	a	a
a	3324857	c	3461883	s	c	clone D	++	c									a	a			c			c	c	c	c		c	c	c
g	3324863	a	3461889	s	a	clone D	++	a									g	g			a			a	a	a	a		a	a	a
g	3324871	a	3461897	s	a	clone D	++	a									g	g			a			a	a	a	a		a	a	a
c	3324872	t	3461898	ns	t	clone D	++	t									c	c			t			t	t	t	t		t	t	t
t	3324873	g	3461899	ns	g	clone D	++	g									t	t			g			g	g	g	g		g	g	g
g	3324878	c	3461904	s	c	clone D	++	c									g	g			c			c	c	c	c		c	c	c
g	3324887	a	3461913	s	a	clone D	++	a									g	g			a			a	a	a	a		a	a	a
g	3324890	a	3461916	s	a	clone D	++	a									g	g			a			a	a	a	a		a	a	a

Clone D base	Clone D site	CFT 073 base	CFT 073 site	mutation type[a]	Inferred ancestral base[b]	lineage inferred to mutate	Support level[c]	ED1a[d]	536	S88	APEC O1	UTI89	E2348/69	ATCC8739	K-12	HS	SMS3-5	IAI39	E24377A	SE11	55989	IAI1	Sakai	UMN026	B4 Sb227	F5 8401	SS Ss046	D1 Sd197	B18 BS512	F2a 2457T	F2a 301
g	3324895	a	3461921	ns	a	clone D	++	a									g	g			a			a	a	a	a		a	a	a
g	3324908	a	3461934	s	a	clone D	++	a									g	g			a			a	a	a	a		a	a	a
g	3324935	a	3461961	s	a	clone D	++	a									g	g			a			a	a	a	a		a	a	a
g	3324938	a	3461964	s	a	clone D	++	a									g	g			a			a	a	a	a		a	a	a
g	3324947	a	3461973	s	a	clone D	++	a									g	g			a			a	a	a	a		a	a	a
g	3324953	a	3461979	s	a	clone D	++	a									g	g			a			a	a	a	a		a	a	a
g	3324962	t	3461988	s	t	clone D	++	t									g	g			t			t	t	t	t		t	t	t
t	3324965	c	3461991	s	c	clone D	++	c									t	t			c			c	c	c	c		c	c	c
g	3324968	a	3461994	s	a	clone D	++	a									g	g			a			a	a	a	a		a	a	a
g	3324971	a	3461997	s	a	clone D	++	a									g	g			a			a	a	a	a		a	a	a
g	3324989	a	3462015	s	a	clone D	++	a									g	g			a			a	a	a	a		a	a	a
g	3324993	a	3462019	ns	a	clone D	++	a									g	g			a			a	a	a	a		a	a	a
a	3324995	c	3462021	s	c	clone D	++	c									a	a			c			c	c	c	c		c	c	c
g	3325007	a	3462033	s	a	clone D	++	a									g	g			a			a	a	a	a		a	a	a
g	3325015	t	3462041	ns	t	clone D	++	t									g	g			t			t	t	t	t		t	t	t
c	3325017	t	3462043	ns	t	clone D	++	t									c	c			t			t	t	t	t		t	t	t
c	3325027	t	3462053	ns	t	clone D	++	t									c	c			t			t	t	t	t		t	t	t
c	3325032	t	3462058	ns	t	clone D	++	t									c	c			t			t	t	t	t		t	t	t
a	3325046	t	3462072	ns	t	clone D	++	t									a	a			t			t	t	t	t		t	t	t
t	3325048	c	3462074	ns	c	clone D	++	c									t	t			c			c	c	c	c		c	c	c
t	3325050	c	3462076	ns	c	clone D	++	c									t	t			c			c	c	c	c		c	c	c
t	3325055	c	3462081	s	c	clone D	++	c									t	t			c			c	c	c	c		c	c	c
g	3325061	a	3462087	s	a	clone D	++	a									g	g			a			a	a	a	a		a	a	a
t	3325083	c	3462109	ns	c	clone D	++	c									t	t			c			c	c	c	c		c	c	c
g	3325085	a	3462111	ns	a	clone D	++	a									g	g			a			a	a	a	a		a	a	a
g	3325087	c	3462113	ns	c	clone D	++	c									g	g			c			c	c	c	c		c	c	c
g	3325094	a	3462120	ns	a	clone D	++	a									g	g			a			a	a	a	a		a	a	a
g	3325096	t	3462122	ns	t	clone D	++	t									g	g			t			t	t	t	t		t	t	t

Clone D base	Clone D site	CFT 073 base	CFT 073 site	mutation type[a]	Inferred ancestral base[b]	lineage inferred to mutate	Support level[c]	ED1a[d]	536	S88	APEC O1	UTI89	E2348/69	ATCC8739	K-12	HS	SMS3-5	IAI39	E24377A	SE11	55989	IAI1	Sakai	UMN026	B4 Sb227	F5 8401	SS Ss046	D1 Sd197	B18 BS512	F2a 2457T	F2a 301
t	3325099	c	3462125	ns	c	clone D	++	c									t	t			c			c	c	c	c		c	c	c
g	3325103	a	3462129	s	a	clone D	++	a									g	g			a			a	a	a	a		a	a	a
t	3325113	c	3462139	ns	c	clone D	++	c									c	c			c			c	c	c	c		c	c	c
c	3325114	t	3462140	ns	t	clone D	++	t									c	c			t			t	t	t	t		t	t	t
t	3325115	g	3462141	s	g	clone D	++	g									t	t			g			g	g	g	g		g	g	g
g	3325136	a	3462162	s	a	clone D	++	a									g	g			a			a	a	a	a		a	a	a
c	3325151	t	3462177	s	t	clone D	++	t									c	c			t			t	t	t	t		t	t	t
c	3325181	a	3462207	ns	a	clone D	++	a									c	c			a			a	a	a	a		a	a	a
t	3325183	g	3462209	ns	g	clone D	++	g									t	t			g			g	g	g	g		g	g	g
g	3325205	a	3462231	s	a	clone D	++	a									g	g			a			a	a	a	a		a	a	a
g	3325220	a	3462246	s	a	clone D	++	a									g	g			a			a	a	a	a		a	a	a
a	3325256	c	3462282	ns	c	clone D	++	c									a	a			c			c	c	c	c		c	c	c
c	3325295	a	3462321	ns	c	CFT073	++	c									c	c			c			a	c	c	c		c	c	c
g	3325296	a	3462322	ns	a	clone D	++	a									g	g			a			a	a	a	a		a	a	a
t	3325300	g	3462326	ns	g	clone D	++	g									t	t			g			g	g	g	g		g	g	g
g	3325337	a	3462363	s	a	clone D	++	a									g	g			a			a	a	a	a		a	a	a
a	3325352	g	3462378	s	g	clone D	++	g									a	a			g			g	g	g	g		g	g	g
a	3325365	g	3462391	ns	g	clone D	++	g									a	a			g			g	g	g	g		g	g	g
g	3325373	a	3462399	s	a	clone D	++	a									g	g			a			a	a	a	a		a	a	a
t	3325403	c	3462429	s	c	clone D	++	c									t	t			c			c	c	c	c		c	c	c
c	3325442	t	3462468	s	t	clone D	++	t									c	c			t			t	t	t	t		t	t	t
c	3325450	t	3462476	ns	c	CFT073	++	c									c	c			c			t	c	c	c		c	c	c
a	3325454	g	3462480	s	g	clone D	++	g									a	a			g			g	g	g	g		g	g	g
g	3325458	t	3462484	ns	t	clone D	++	t									g	g			t			t	t	t	t		t	t	t
g	3325460	a	3462486	s	a	clone D	++	a									g	g			a			a	a	a	a		a	a	a
a	3325508	g	3462534	s	g	clone D	++	g									a	a			g			g	g	g	g		g	g	g
g	3325514	a	3462540	s	a	clone D	++	a									g	g			a			a	a	a	a		a	a	a
g	3325526	c	3462552	ns	c	clone D	++	c									g	g			c			c	c	c	c		c	c	c

Clone D base	Clone D site	CFT 073 base	CFT 073 site	mutation type[a]	Inferred ancestral base[b]	lineage inferred to mutate	Support level[c]	ED1a[d]	536	S88	APEC O1	UTI89	E2348/69	ATCC8739	K-12	HS	SMS3-5	IAI39	E24377A	SE11	55989	IAI1	Sakai	UMN026	B4 Sb227	F5 8401	SS Ss046	D1 Sd197	B18 BS512	F2a 2457T	F2a 301
a	3325536	t	3462562	ns	t	clone D	++	t									a	a			t			t	t	t	t		t	t	t
c	3325541	t	3462567	s	t	clone D	++	t									c	c			t			t	t	t	t		t	t	t
g	3325548	c	3462574	ns	c	clone D	++	c									g	g			c			c	c	c	c		c	c	c
t	3325553	g	3462579	ns	g	clone D	++	g									t	t			g			g	g	g	g		g	g	g
a	3325562	g	3462588	s	g	clone D	++	g									a	a			g			g	g	g	g		g	g	g
g	3325565	a	3462591	s	a	clone D	++	a									g	g			a			a	a	a	a		a	a	a
c	3325594	t	3462620	ns	t	clone D	++	t									c	c			t			t	t	t	t		t	t	t
g	3325601	c	3462627	s	?	?	+/-	t									g	g			t			c	t	t	t		t	t	t
g	3325607	a	3462633	s	a	clone D	++	a									g	g			a			a	a	a	a		a	a	a
t	3325622	c	3462648	s	c	clone D	++	c									t	t			c			c	c	c	c		c	c	c
g	3325625	t	3462651	s	t	clone D	++	t									g	g			t			t	t	t	t		t	t	t
a	3325630	g	3462656	s	g	clone D	++	g									a	a			g			g	g	g	g		g	g	g
c	3325631	t	3462657	s	t	clone D	++	t									c	c			t			t	t	t	t		t	t	t
a	3325667	g	3462693	s	g	clone D	++	g									a	a			g			g	g	g	g		g	g	g
c	3325690	t	3462716	ns	t	clone D	++	t									c	c			t			t	t	t	t		t	t	t
a	3325696	g	3462722	ns	g	clone D	++	g									a	a			g			g	g	g	g		g	g	g
a	3325726	g	3462752	s	g	clone D	++	g									a	a			g			g	g	g	g		g	g	g
c	3325799	g	3462825	ns	g	clone D	++	g									c	c			g			g	g	g	g		g	g	g
t	3325820	a	3462846	s	a	clone D	++	a									t	t			a			a	a	a	a		a	a	a
t	3325832	c	3462858	s	c	clone D	++	c									t	t			c			c	c	c	c		c	c	c
c	3325854	t	3462880	ns	t	clone D	++	t									c	c			t			t	t	t	t		t	t	t
g	3325868	t	3462894	s	t	clone D	++	t									g	g			t			t	t	t	t		t	t	t
g	3325930	a	3462956	s	a	clone D	++	a									g	g			a			a	a	a	a		a	a	a
g	3325931	c	3462957	s	c	clone D	++	c									g	g			c			c	c	c	c		c	c	c
a	3325934	t	3462960	s	t	clone D	++	t									a	a			t			t	t	t	t		t	t	t
g	3325943	c	3462969	s	c	clone D	++	c									g	g			c			c	c	c	c		c	c	c
g	3325949	a	3462975	s	a	clone D	++	a									g	g			a			a	a	a	a		a	a	a
a	3325960	c	3462986	ns	c	clone D	++	c									a	a			c			c	c	c	c		c	c	c

Clone D base	Clone D site	CFT 073 base	CFT 073 site	mutation type[a]	Inferred ancestral base[b]	lineage inferred to mutate	Support level[c]	ED1a[d]	536	S88	APEC O1	UTI89	E2348/69	ATCC8739	K-12	HS	SMS3-5	IAI39	E24377A	SE11	55989	IAI1	Sakai	UMN026	B4 Sb227	F5 8401	SS Ss046	D1 Sd197	B18 BS512	F2a 2457T	F2a 301
a	3325967	g	3462993	s	g	clone D	++	g									a	a			g			g	g	g	g		g	g	g
t	3326006	g	3463032	ns	g	clone D	++	g									t	t			g			g	g	g	g		g	g	g
t	3326008	c	3463034	ns	c	clone D	++	c									t	t			c			c	c	c	c		c	c	c
t	3326032	a	3463058	ns	a	clone D	++	a									t	t			a			a	a	a	a		a	a	a
a	3326048	g	3463074	s	g	clone D	++	g									a	a			g			g	g	g	g		g	g	g
g	3326054	a	3463080	s	a	clone D	++	a									g	g			a			a	a	a	a		a	a	a
c	3326062	t	3463088	ns	t	clone D	++	t									c	c			t			t	t	t	t		t	t	t
c	3326066	g	3463092	s	g	clone D	++	g									c	c			g			g	g	g	g		g	g	g
g	3326069	a	3463095	s	a	clone D	++	a									g	g			a			a	a	a	a		a	a	a
c	3326089	t	3463115	ns	t	clone D	++	t									c	c			t			t	t	t	t		t	t	t
a	3326090	g	3463116	s	g	clone D	++	g									a	a			g			g	g	g	g		g	g	g
g	3326108	t	3463134	s	t	clone D	++	t									g	g			t			t	t	t	t		t	t	t
c	3326115	t	3463141	ns	t	clone D	++	t									c	c			t			t	t	t	t		t	t	t
g	3326126	a	3463152	s	a	clone D	++	a									g	g			a			a	a	a	a		a	a	a
a	3326135	g	3463161	s	g	clone D	++	g									a	a			g			g	g	g	g		g	g	g
c	3326141	t	3463167	s	t	clone D	++	t									c	c			t			t	t	t	t		t	t	t
g	3326143	a	3463169	s	a	clone D	++	a									g	g			a			a	a	a	a		a	a	a
a	3326162	c	3463188	s	c	clone D	++	c									a	a			c			c	c	c	c		c	c	c
g	3326171	a	3463197	s	a	clone D	++	a									g	g			a			a	a	a	a		a	a	a
t	3326189	c	3463215	s	c	clone D	++	c									t	t			c			c	c	c	c		c	c	c
g	3326201	a	3463227	s	a	clone D	++	a									g	g			a			a	a	a	a		a	a	a
g	3326240	a	3463266	s	a	clone D	++	a									g	g			a			a	a	a	a		a	a	a
g	3326243	t	3463269	s	t	clone D	++	t									g	g			t			t	t	t	t		t	t	t
t	3326256	c	3463282	ns	c	clone D	++	c									t	t			c			c	c	c	c		c	c	c
c	3326267	t	3463293	ns	t	clone D	++	t									c	c			t			t	t	t	t		t	t	t
c	3326269	t	3463295	ns	t	clone D	++	t									c	c			t			t	t	t	t		t	t	t
a	3326272	t	3463298	ns	t	clone D	++	t									a	a			t			t	t	t	t		t	t	t
g	3326274	t	3463300	ns	t	clone D	++	t									g	g			t			t	t	t	t		t	t	t

Clone D base	Clone D site	CFT 073 base	CFT 073 site	mutation type[a]	Inferred ancestral base[b]	lineage inferred to mutate	Support level[c]	ED1a[d]	536	S88	APEC O1	UTI89	E2348/69	ATCC8739	K-12	HS	SMS3-5	IAI39	E24377A	SE11	55989	IAI1	Sakai	UMN026	B4 Sb227	F5 8401	SS Ss046	D1 Sd197	B18 BS512	F2a 2457T	F2a 301
t	3326275	c	3463301	ns	c	clone D	++	c									t	t			c			c	c	c	c		c	c	c
t	3326276	a	3463302	ns	a	clone D	++	a									t	t			a			a	a	a	a		a	a	a
c	3326278	t	3463304	ns	t	clone D	++	t									c	c			t			t	t	t	t		t	t	t
g	3326286	t	3463312	ns	t	clone D	++	t									g	g			t			t	t	t	t		t	t	t
t	3326291	c	3463317	s	c	clone D	++	c									t	t			c			c	c	c	c		c	c	c
a	3326294	g	3463320	s	g	clone D	++	g									a	a			g			g	g	g	g		g	g	g
t	3326300	a	3463326	s	a	clone D	++	a									t	t			a			a	a	a	a		a	a	a
c	3326306	t	3463332	ns	t	clone D	++	t									c	c			t			t	t	t	t		t	t	t
c	3326308	a	3463334	ns	a	clone D	++	a									c	c			a			a	a	a	a		a	a	a
t	3326311	g	3463337	ns	g	clone D	++	g									t	t			g			g	g	g	g		g	g	g
t	3326314	c	3463340	ns	c	clone D	++	c									t	t			c			c	c	c	c		c	c	c
g	3326316	a	3463342	ns	a	clone D	++	a									g	g			a			a	a	a	a		a	a	a
t	3326317	c	3463343	ns	c	clone D	++	c									t	t			c			c	c	c	c		c	c	c
t	3326327	c	3463353	s	c	clone D	++	c									t	t			c			c	c	c	c		c	c	c
g	3326330	a	3463356	ns	a	clone D	++	a									g	g			a			a	a	a	a		a	a	a
t	3326331	c	3463357	ns	c	clone D	++	c									t	t			c			c	c	c	c		c	c	c
t	3326332	c	3463358	ns	c	clone D	++	c									t	t			c			c	c	c	c		c	c	c
g	3326333	a	3463359	s	a	clone D	++	a									g	g			a			a	a	a	a		a	a	a
a	3326341	g	3463367	ns	g	clone D	++	g									a	a			g			g	g	g	g		g	g	g
a	3326342	g	3463368	ns	g	clone D	++	g									a	a			g			g	g	g	g		g	g	g
a	3326343	g	3463369	ns	g	clone D	++	g									a	a			g			g	g	g	g		g	g	g
g	3326346	a	3463372	ns	a	clone D	++	a									g	g			a			a	a	a	a		a	a	a
t	3326359	g	3463385	ns	g	clone D	++	g									t	t			g			g	g	g	g		g	g	g
t	3326360	c	3463386	ns	c	clone D	++	c									t	t			c			c	c	c	c		c	c	c
a	3326367	g	3463393	ns	g	clone D	++	g									a	a			g			g	g	g	g		g	g	g
c	3326376	g	3463402	ns	g	clone D	++	g													g			g	g	g	g		g	g	g
c	3326377	a	3463403	ns	a	clone D	++	a													a			a	a	a	a		a	a	a
c	3326381	t	3463407	s	t	clone D	++	t													t			t	t	t	t		t	t	t

Clone D base	Clone D site	CFT 073 base	CFT 073 site	mutation type[a]	Inferred ancestral base[b]	lineage inferred to mutate	Support level[c]	ED1a[d]	536	S88	APEC O1	UTI89	E2348/69	ATCC8739	K-12	HS	SMS3-5	IAI39	E24377A	SE11	55989	IAI1	Sakai	UMN026	B4 Sb227	F5 8401	SS Ss046	D1 Sd197	B18 BS512	F2a 2457T	F2a 301
g	3326382	t	3463408	s	t	clone D	++	t													t			t	t	t	t		t	t	t
c	3326408	t	3463411	nc	c	CFT073	++	c									c	c			c			t	c	c	c		c	c	c
g	3326412	a	3463415	nc	a	clone D	++	a									g	g			a			a	a	a	a		a	a	a
c	3326416	t	3463419	nc	t	clone D	++	t									c	c			t			t	t	t	t		t	t	t
a	3326426	c	3463429	nc	a	CFT073	++	a									a	a			a			c	a	a	a		a	a	a
t	3326444	g	3463447	nc	g	clone D	++	g									-	-			g			g	g	g	g		g	g	g
c	3326448	t	3463452	nc	t	clone D	++	t									c	c			t			t	t	t	t		t	t	t
c	3326449	t	3463453	nc	t	clone D	++	t									c	c			t			t	t	t	t		t	t	t
t	3326451	c	3463455	ns	c	clone D	++	c									t	t			c			c	c	c	c		c	c	c
a	3326452	g	3463456	ns	g	clone D	++	g									a	a			g			g	g	g	g		g	g	g
g	3326454	c	3463458	ns	c	clone D	++	c									g	g			c			c	c	c	c		c	c	c
g	3326477	t	3463481	s	t	clone D	++	t									g	g			t			t	t	t	t		t	t	t
t	3326510	c	3463514	s	c	clone D	++	c									t	t			c			c	c	c	c		c	c	c
t	3326515	g	3463519	ns	g	clone D	++	g									t	t			g			g	g	g	g		g	g	g
t	3326546	g	3463550	s	g	clone D	++	g									t	t			g			g	g	g	g		g	g	g
g	3326582	a	3463586	s	a	clone D	++	a									g	g			a			a	a	a	a		a	a	a
a	3326591	g	3463595	s	g	clone D	++	g									a	a			g			g	g	g	g		g	g	g
a	3326604	g	3463608	ns	g	clone D	++	g									a	a			g			g	g	g	g		g	g	g
g	3326606	a	3463610	s	a	clone D	++	a									g	g			a			a	a	a	a		a	a	a
g	3326615	a	3463619	s	a	clone D	++	a									g	g			a			a	a	a	a		a	a	a
c	3326618	t	3463622	s	t	clone D	++	t									c	c			t			t	t	t	t		t	t	t
g	3326692	a	3463696	s	a	clone D	++	a									g	g			a			a	a	a	a		a	a	a
c	3326723	t	3463727	s	t	clone D	++	t									c	c			t			t	t	t	t		t	t	t
c	3326774	t	3463778	s	t	clone D	++	t									c	c			t			t	t	t	t		t	t	t
t	3326781	c	3463785	ns	t	CFT073	++	t									t	t			t			c	t	t	t		t	t	t
c	3326795	t	3463799	s	t	clone D	++	t									c	c			t			t	t	t	t		t	t	t
g	3326803	a	3463807	s	a	clone D	++	a									g	g			a			a	a	a	a		a	a	a
c	3326804	t	3463808	s	t	clone D	++	t									c	c			t			t	t	t	t		t	t	t

Clone D base	Clone D site	CFT 073 base	CFT 073 site	mutation type[a]	Inferred ancestral base[b]	lineage inferred to mutate	Support level[c]	ED1a[d]	536	S88	APEC O1	UTI89	E2348/69	ATCC8739	K-12	HS	SMS3-5	IAI39	E24377A	SE11	55989	IAI1	Sakai	UMN026	B4 Sb227	F5 8401	SS Ss046	D1 Sd197	B18 BS512	F2a 2457T	F2a 301
a	3326816	c	3463820	s	c	clone D	++	c									a	a			c			c	c	c	c		c	c	c
c	3326995	t	3463999	ns	t	clone D	++	t									c	c			t			t	t	t	t		t	t	t
g	3327083	t	3464087	s	t	clone D	++	t									g	g			t			t	t	t	t		t	t	t
g	3327173	a	3464177	s	g	CFT073	++	g									g	g			g			a	g	g	g		g	g	g
a	3327191	c	3464195	s	c	clone D	++	c									a	a			c			c	c	c	c		c	c	c
t	3327244	c	3464248	ns	c	clone D	++	c									t	t			c			c	c	c	c		c	c	c
t	3327318	c	3464322	ns	c	clone D	++	c									t	t			c			c	c	c	c		c	c	c
g	3327320	a	3464324	s	a	clone D	++	a									g	g			a			a	a	a	a		a	a	a
t	3327326	c	3464330	s	c	clone D	++	c									t	t			c			c	c	c	c		c	c	c
c	3327329	a	3464333	s	a	clone D	++	a									c	c			a			a	a	a	a		a	a	a
g	3327346	t	3464350	ns	t	clone D	++	t									g	g			t			t	t	t	t		t	t	t
a	3327353	g	3464357	s	g	clone D	++	g									a	a			g			g	g	g	g		g	g	g
g	3327365	a	3464369	s	a	clone D	++	a									g	g			a			a	a	a	a		a	a	a
g	3327422	a	3464426	s	a	clone D	++	a									g	g			a			a	a	a	a		a	a	a
a	3327512	g	3464516	s	g	clone D	++	g									a	a			g			g	g	g	g		g	g	g
a	3327536	t	3464540	s	t	clone D	++	t									a	a			t			t	t	t	t		t	t	t
g	3327563	a	3464567	s	g	CFT073	++	g									g	g			g			a	g	g	g		g	g	g
a	3327623	g	3464627	s	g	clone D	++	g									a	a			g			g	g	g	g		g	g	g
c	3327640	t	3464644	ns	t	clone D	++	t									c	c			t			t	t	t	t		t	t	t
c	3327665	t	3464669	s	t	clone D	++	t									c	c			t			t	t	t	t		t	t	t
g	3327674	a	3464678	s	g	CFT073	++	g									g	g			g			a	g	g	g		g	g	g
t	3327698	g	3464702	s	g	clone D	++	g									t	t			g			g	g	g	g		g	g	g
c	3327771	t	3464775	ns	t	clone D	++	t									c	c			t			t	t	t	t		t	t	t
g	3327796	a	3464800	s	a	clone D	++	a									g	g			a			a	a	a	a		a	a	a
a	3327809	t	3464813	ns	a	CFT073	++	a									a	a			a			t	a	a	a		a	a	a
a	3327811	g	3464815	s	g	clone D	++	g									a	a			a			g	g	g	g		g	g	g
a	3327814	c	3464818	s	c	clone D	++	c									a	a			c			c	c	c	c		c	c	c
a	3327868	g	3464872	s	g	clone D	++	g									a	a			g			g	g	g	g		g	g	g

Clone D base	Clone D site	CFT 073 base	CFT 073 site	mutation type[a]	Inferred ancestral base[b]	lineage inferred to mutate	Support level[c]	ED1a[d]	536	S88	APEC O1	UTI89	E2348/69	ATCC8739	K-12	HS	SMS3-5	IAI39	E24377A	SE11	55989	IAI1	Sakai	UMN026	B4 Sb227	F5 8401	SS Ss046	D1 Sd197	B18 BS512	F2a 2457T	F2a 301
t	3327910	g	3464914	ns	g	clone D	++	g									t	t			g			g	g	g	g		g	g	g
g	3328057	a	3465061	s	a	clone D	++	a									g	g			a			a	a	a	a		a	a	a
a	3328066	g	3465070	s	g	clone D	++	g									a	a			g			g	g	g	g		g	g	g
a	3328075	c	3465079	s	c	clone D	++	c									a	a			c			c	c	c	c		c	c	c
g	3328105	a	3465109	s	a	clone D	++	a									g	g			a			a	a	a	a		a	a	a
t	3328109	g	3465113	ns	g	clone D	++	g									t	t			g			g	g	g	g		g	g	g
t	3328123	c	3465127	s	c	clone D	++	c									c	t			c			c	c	c	c		c	c	c
a	3328165	t	3465169	s	t	clone D	++	t									a	a			t			t	t	t	t		t	t	t
a	3328186	c	3465190	s	c	clone D	++	c									c	a			c			c	c	c	c		c	c	c
a	3328201	g	3465205	s	g	clone D	++	g									a	a			g			g	g	g	g		g	g	g
a	3328246	c	3465250	s	c	clone D	++	c									a	a			c			c	c	c	c		c	c	c
g	3328255	a	3465259	s	a	clone D	++	a									g	g			a			a	a	a	a		a	a	a
g	3328269	a	3465273	s	a	clone D	++	a									g	g			a			a	a	a	a		a	a	a
c	3328297	t	3465301	s	t	clone D	++	t									c	c			t			t	t	t	t		t	t	t
t	3328309	g	3465313	s	g	clone D	++	g									t	t			g			g	g	g	g		g	g	g
g	3328483	c	3465487	s	c	clone D	++	c									g	g			c			c	c	c	c		c	c	c
t	3328510	g	3465514	s	g	clone D	++	g									g	t			g			g	g	g	g		g	g	g
t	3328531	c	3465535	s	c	clone D	++	c									c	t			c			c	c	c	c		c	c	c
t	3328561	c	3465565	s	c	clone D	++	c									t	t			c			c	c	c	c		c	c	c
g	3328594	a	3465598	s	a	clone D	++	a									g	g			a			a	a	a	a		a	a	a
g	3328663	t	3465667	s	t	clone D	++	t									a	g			t			t	t	t	t		t	t	t
a	3328672	g	3465676	s	g	clone D	++	g									a	a			g			g	g	g	g		g	g	g
a	3328777	g	3465781	s	g	clone D	++	g									g	a			g			g	g	g	g		g	g	g
t	3328837	c	3465841	s	c	clone D	++	c									t	c			c			c	c	c	c		c	c	c
a	3328924	g	3465928	s	a	CFT073	++	a									a	a			a			g	a	a	a		a	a	a
t	3328986	c	3465990	ns	t	CFT073	++	t									c	t			t			c	t	t	t		t	t	t
g	3329323	a	3466327	s	g	CFT073	++	g									g	g			g			a	g	g	g		g	g	g
a	3329590	t	3466594	ns	t	clone D	++	t									t	t			t			t	t	t	t		t	t	t

Clone D base	Clone D site	CFT 073 base	CFT 073 site	mutation type[a]	Inferred ancestral base[b]	lineage inferred to mutate	Support level[c]	ED1a[d]	536	S88	APEC O1	UTI89	E2348/69	ATCC8739	K-12	HS	SMS3-5	IAI39	E24377A	SE11	55989	IAI1	Sakai	UMN026	B4 Sb227	F5 8401	SS Ss046	D1 Sd197	B18 BS512	F2a 2457T	F2a 301
g	3329793	a	3466797	s	g	CFT073	++	g									g	g			a			a	g	g	g		g	g	g
a	3330006	g	3467010	s	a	CFT073	++	a									a	a			a			a	a	a	a		a	a	a
t	3330033	c	3467037	s	t	CFT073	++	t									c	t			c			c	t	t	t		t	t	t
a	3330144	g	3467148	s	a	CFT073	++	a									g	a			g			g	a	a	a		a	a	a
g	3330206	a	3467210	ns	g	CFT073	++	g									a	g			a			a	g	g	g		g	g	g
a	3330246	t	3467250	s	t	clone D	++	t									t	t			t			t	t	t	t		t	t	t
a	3331062	g	3468066	s	a	CFT073	++	a									g	a			g			g	a	a	a		a	a	a
c	3331144	a	3468148	ns	a	clone D	++	a									c	c			a			a	a	a	a		a	a	a
c	3331725	t	3468729	s	c	CFT073	++	c									c	c			t			t	c	c	c		c	c	c
a	3331755	g	3468759	s	a	CFT073	++	a									g	a			g			g	a	a	a		a	a	a
c	3331760	t	3468764	ns	c	CFT073	++	c									c	c			t			t	c	c	c		c	c	c
a	3331798	g	3468802	ns	a	CFT073	++	a									g	a			g			g	a	a	a		a	a	a
a	3331800	g	3468804	s	a	CFT073	++	a									g	a			g			g	a	a	a		a	a	a
g	3331863	c	3468867	s	g	CFT073	++	g									c	g			c			c	g	g	g		g	g	g
g	3332024	t	3469028	ns	g	CFT073	++	g									t	g			t			t	g	g	g		g	g	g
c	3332160	t	3469164	nc	t	clone D	++	t									t	c			t			t	t	t	t		t	t	t
t	3332205	g	3469209	nc	t	CFT073	++	t									g	t			g			g	t	t	t		t	t	t
t	3332272	a	3469276	nc	t	CFT073	++	t									c	t			a			a	t	t	t		t	t	t
a	3332282	t	3469286	s	a	CFT073	++	a									t	a			t			t	a	a	a		a	a	a
a	3332357	g	3469361	s	a	CFT073	++	a									a	a			g			g	a	a	a		a	a	a
c	3332474	g	3469478	ns	g	clone D	++	g									g	g			g			g	g	g	g		g	g	g
t	3332699	a	3469703	s	t	CFT073	++	t									g	t			t			t	t	t	t		t	t	t
g	3332899	a	3469903	ns	g	CFT073	++	g									g	g			g			g	g	g	g		g	g	g
a	3333533	g	3470537	nc	g	clone D	++	g										g			g			g	g	g	g		g	g	g
c	3334960	t	3470630	s	c	CFT073	++	c																t							
a	3336081	t	3471750	s	a	CFT073	++	a																a							
g	3336087	c	3471756	ns	g	CFT073	++	g																g							
a	3336088	g	3471757	ns	a	CFT073	++	a																a							

Clone D base	Clone D site	CFT 073 base	CFT 073 site	mutation type[a]	Inferred ancestral base[b]	lineage inferred to mutate	Support level[c]	ED1a[d]	536	S88	APEC O1	UTI89	E2348/69	ATCC8739	K-12	HS	SMS3-5	IAI39	E24377A	SE11	55989	IAI1	Sakai	UMN026	B4 Sb227	F5 8401	SS Ss046	D1 Sd197	B18 BS512	F2a 2457T	F2a 301
t	3336104	g	3471772	ns	t	CFT073	++	t																t							
a	3336174	g	3471837	s	a	CFT073	++	a																g							
c	3336256	t	3471916	ns	c	CFT073	++	c																c							
c	3336258	t	3471918	s	c	CFT073	++	c																c							
a	3336287	g	3471947	s	a	CFT073	++	a																a							
a	3336875	g	3472534	ns	g	clone D	++	g																g							
g	3336945	a	3472604	s	g	CFT073	++	g																g							
a	3337166	g	3472825	s	a	CFT073	++	a																a							
a	3337243	c	3472903	ns	a	CFT073	++	a																c							
a	3337245	c	3472905	ns	a	CFT073	++	a																c							
a	3337247	c	3472908	ns	c	clone D	++	c																a							
c	3338056	t	3473718	s	c	CFT073	++	c																c							
t	3338618	g	3474280	ns	t	CFT073	++	t																t							
t	3338773	c	3474435	s	c	clone D	++	c																t							
g	3339684	a	3475346	ns	g	CFT073	++	g																g							
g	3340438	a	3476100	s	g	CFT073	++	g																g							
t	3343615	g	3479277	s	t	CFT073	++	t																t							
g	3344306	a	3479969	nc	a	clone D	++	a																a							
c	3344309	t	3479974	nc	?	?	+/-	-																-							
t	3344315	a	3479980	nc	t	CFT073	++	t																t							
c	3344334	a	3479999	nc	c	CFT073	++	c																c							
c	3344336	a	3480001	nc	c	CFT073	++	c																c							
c	3344352	t	3480019	nc	t	clone D	++	t																t							
a	3344358	t	3480025	nc	a	CFT073	++	a																a							
c	3344369	g	3480037	nc	c	CFT073	++	c																c							
c	3344375	t	3480043	nc	c	CFT073	++	c																c							
a	3344384	c	3480053	nc	a	CFT073	++	a																a							
a	3344389	c	3480058	nc	a	CFT073	++	a																a							

Clone D base	Clone D site	CFT 073 base	CFT 073 site	mutation type[a]	Inferred ancestral base[b]	lineage inferred to mutate	Support level[c]	ED1a[d]	536	S88	APEC O1	UTI89	E2348/69	ATCC8739	K-12	HS	SMS3-5	IAI39	E24377A	SE11	55989	IAI1	Sakai	UMN026	B4 Sb227	F5 8401	SS Ss046	D1 Sd197	B18 BS512	F2a 2457T	F2a 301
a	3344401	c	3480070	nc	a	CFT073	++	a																a							
a	3346076	g	3481745	s	g	clone D	++	g		g		g	g			g	g				g		g	g	g				g		
a	3346180	g	3481849	ns	a	CFT073	++	a		a		g	g			g	g				g		g	g	a				a		
t	3347981	c	3483650	ns	c	clone D	++	c	c	c			c						c		c		c	c	c		c	c	c	c	c
c	3348195	g	3483863	ns	c	CFT073	+		c							c			c		c			c						c	c
a	3348207	g	3483875	s	?	?	+/-	t	t	t	t	t				a	t		g		g			a						g	g
c	3348420	a	3484088	s	a	clone D	++	a	a	a	a	a				a	a		a		a			c						c	c
g	3348426	c	3484094	s	?	?	+/-	a	a	a	a	a				a	a		a		a			g						a	a
a	3348434	g	3484102	ns	a	CFT073	++	a	a	a	a	a				a	a		a		a			a						a	a
t	3348435	c	3484103	s	t	CFT073	++	t	t	t	t	t				t	t		c		t			t						t	t
g	3348482	a	3484150	ns	?	?	+/-	t	g	t	t	g				g	t		g		g			g						t	t
g	3348483	a	3484151	s	a	clone D	++	a	a	a	a	a				a	a		a		a			g						a	a
a	3348492	t	3484160	s	t	clone D	++	t	t	t	t	t				t	t		t		c			a						t	t
a	3348495	g	3484163	s	g	clone D	++	g	g	g	g	g				g	g		g		t			a						a	a
t	3348498	c	3484166	s	c	clone D	++	c	c	c	c	c				c	c		c		c			c						c	c
a	3348567	g	3484235	ns	?	?	+/-	c	a	c	c	a				a	c		c		c			a						a	a
a	3348569	g	3484237	ns	?	?	+/-	t	a	t	t	a				a	t		t		t			t						t	t
c	3348577	t	3484244	nc	c	CFT073	++	c	t	c	c	t				t	c		c		c			c						c	c
t	3348578	a	3484245	nc	a	clone D	++	a	a	a	a	a				a	a		a		a			t						a	a
g	3348579	c	3484246	nc	c	clone D	++	c	c	c	c	c				c	c		c		c			g						c	c
a	3348593	g	3484260	nc	g	clone D	++	g	g	g	g	g				g	g		g		g			a						g	g
a	3348704	g	3484371	nc	g	clone D	+		g			g				g					a			a							
g	3348706	a	3484373	nc	a	clone D	+		a			a				a					g			g							
g	3349168	c	3484835	ns	g	CFT073	++	g	g	g	g	g				g	g		g		g			g							
a	3349227	g	3484894	ns	g	clone D	++	g	g	g	g	g				g	g		g		g			g							
t	3349507	c	3485174	ns	c	clone D	++	c	c	c	c	c				c	c		c		c			c							
c	3349575	t	3485242	s	t	clone D	++	t	t	t	t	t				c	t		t		c			c							
a	3349703	g	3485370	nc	g	clone D	+									g					g			g							

Clone D base	Clone D site	CFT 073 base	CFT 073 site	mutation type[a]	Inferred ancestral base[b]	lineage inferred to mutate	Support level[c]	ED1a[d]	536	S88	APEC O1	UTI89	E2348/69	ATCC8739	K-12	HS	SMS3-5	IAI39	E24377A	SE11	55989	IAI1	Sakai	UMN026	B4 Sb227	F5 8401	SS Ss046	D1 Sd197	B18 BS512	F2a 2457T	F2a 301
a	3349720	g	3485387	nc	a	CFT073	+									a					a			a							
c	3349775	a	3485442	nc	a	clone D	+									a					a			a							
c	3349818	t	3485484	nc	c	CFT073	+									c					c			c							
g	3350001	a	3485667	nc	g	CFT073	++	g								g					g			g							
t	3350158	c	3485824	ns	t	CFT073	++	t								t					t			t							
a	3350162	t	3485828	ns	a	CFT073	++	a								a					a			a							
t	3350250	c	3485916	s	c	clone D	++	c																c							
a	3350282	t	3485948	nc	t	clone D	++	t																a							
a	3350288	g	3485954	nc	g	clone D	++	g																g							
a	3350326	c	3485992	nc	c	clone D	++	c																c							
a	3350438	g	3486104	nc	g	clone D	+																	g							
c	3350453	a	3486119	nc	a	clone D	+																	a							
g	3350750	t	3486411	nc	?	?	+/-	-													-			-							
c	3350803	a	3486478	ns	c	CFT073	++	c		t	t					t	t		t		c			c							
g	3350826	a	3486501	ns	a	clone D	++	a		a	a					a	a		a		a			a							
g	3350834	a	3486516	ns	?	?	+/-	c		t	t					t	t		t		c			g							
c	3350835	t	3486517	ns	c	CFT073	++	c		c	c					c	c		c		c			c							
c	3350879	t	3486561	ns	c	CFT073	++	c		c	c					c	c		c		c			c							
t	3350953	c	3486629	nc	c	clone D	++	c	c	c	c	c				c	c		c		c			t							
t	3351038	c	3486714	nc	c	clone D	++	c	c	c	c	c				c	c		c		c			t						c	c
g	3351058	t	3486734	nc	t	clone D	++	t	t	t	t	t				t	t		t		t			g						t	t
t	3351122	c	3486798	ns	t	CFT073	++	t	c	c	c	c				c	c		c		t			t						c	c
a	3351129	c	3486805	ns	a	CFT073	++	a	c	c	c	c				c	c		c		a			a						a	a
t	3351130	c	3486806	ns	t	CFT073	++	t	t	t	t	t				t	t		t		t			t						t	t
t	3351170	g	3486846	ns	g	clone D	++	g	g	g	g	g				g	g		g		g			t						t	t
g	3351184	a	3486860	s	a	clone D	++	a	a	a	a	a				a	a		a		a			g						g	g
c	3351190	t	3486866	s	c	CFT073	++	c	c	c	c	c				c	c		c		c			c						c	c
a	3351205	g	3486881	s	g	clone D	++	g	g	g	g	g				g	g		g		g			a						g	g

Clone D base	Clone D site	CFT 073 base	CFT 073 site	mutation type[a]	Inferred ancestral base[b]	lineage inferred to mutate	Support level[c]	ED1a[d]	536	S88	APEC O1	UTI89	E2348/69	ATCC8739	K-12	HS	SMS3-5	IAI39	E24377A	SE11	55989	IAI1	Sakai	UMN026	B4 Sb227	F5 8401	SS Ss046	D1 Sd197	B18 BS512	F2a 2457T	F2a 301
g	3351217	a	3486893	s	g	CFT073	++	g	a	g	a	a				g	a		g		g			g						a	a
a	3351225	g	3486901	ns	a	CFT073	++	a	a	g	a	a				g	a		g		a			a						a	a
t	3351232	g	3486908	s	t	CFT073	++	t	g	g	g	g				g	g		g		t			t						a	a
t	3351241	g	3486917	s	g	clone D	++	g	t	g	t	t				g	t		g		g			t						g	g
a	3351253	g	3486929	s	a	CFT073	++	a	a	g	a	a				g	a		g		a			a						a	a
t	3351262	c	3486938	s	c	clone D	++	c	c	c	c	c				c	c		c		c			t						t	t
c	3351265	t	3486941	s	c	CFT073	++	c	c	c	c	c				c	c		c		c			c						c	c
t	3351299	c	3486975	s	t	CFT073	++	t	c	c	c	c				c	c		c		t			t						c	c
a	3351307	g	3486983	s	g	clone D	++	g	g	g	g	g				g	g		g		g			a						g	g
a	3351314	g	3486990	ns	g	clone D	++	g	g	g	g	g				g	g		g		g			a						g	g
t	3352578	c	3488254	s	t	CFT073	+																	t							
c	3352725	t	3488401	s	t	clone D	+																	t							
t	3353049	a	3488725	s	a	clone D	+																	a							
a	3353055	g	3488731	s	g	clone D	+																	g							
t	3353073	c	3488749	ns	c	clone D	+																	c							
t	3353075	g	3488751	ns	g	clone D	+																	g							
t	3353090	c	3488766	ns	c	clone D	+																	c							
c	3353091	t	3488767	s	t	clone D	+																	t							
c	3353100	t	3488776	s	t	clone D	+																	t							
a	3353103	t	3488779	s	t	clone D	+																	t							
t	3353418	c	3489094	s	c	clone D	+																	c							
c	3353746	t	3489422	nc	t	clone D	+																	t							
a	3353788	g	3489464	nc	a	CFT073	+																	a							
c	3353789	t	3489465	nc	t	clone D	+																	t							
a	3353799	t	3489475	ns	t	clone D	+																	t							
a	3353940	g	3489616	ns	g	clone D	+																	g							
a	3354454	c	3490130	nc	c	clone D	+																	c							
g	3354823	t	3490499	nc	g	CFT073	+																	g							

Clone D base	Clone D site	CFT 073 base	CFT 073 site	mutation type[a]	Inferred ancestral base[b]	lineage inferred to mutate	Support level[c]	ED1a[d]	536	S88	APEC O1	UTI89	E2348/69	ATCC8739	K-12	HS	SMS3-5	IAI39	E24377A	SE11	55989	IAI1	Sakai	UMN026	B4 Sb227	F5 8401	SS Ss046	D1 Sd197	B18 BS512	F2a 2457T	F2a 301
a	3355836	g	3491512	nc	g	clone D	++	g	g						g						g			g						g	g
a	3355840	c	3491516	nc	c	clone D	++	c	c						c						c			c						c	c
a	3355899	t	3491575	nc	t	clone D	++	t	t						t						t			t						t	t
c	3355920	t	3491596	nc	t	clone D	++	t	t						t						t			t						t	t
g	3356251	t	3491927	s	t	clone D	++	t	t	t		t			t	t	t		t		t		t	t						t	t
t	3356276	c	3491952	ns	t	CFT073	++	t	t	t		t			t	t	c		t		t		t	t						t	t
g	3356291	t	3491967	ns	t	clone D	++	t	g	g		g			g	g	g		g		g		g	g						g	g
t	3356315	g	3491991	ns	g	clone D	++	g	g	g		g			g	g	g		g		g		g	g						g	g
g	3356378	a	3492054	ns	a	clone D	++	a	g	a		a			a	a	a		a		g		a	g						g	g
c	3357516	t	3493192	ns	c	CFT073	++	c	t						t						t			t						t	t
g	3357593	t	3493269	ns	?	?	+/-	a	t						t						g			t						g	g
t	3357598	c	3493274	s	t	CFT073	++	t	c						c						t			c						c	c
a	3357772	t	3493448	s	a	CFT073	++	a	t						t						t			t						t	t
c	3357808	t	3493484	s	c	CFT073	++	c	t						t						t			t						t	t
g	3357811	c	3493487	s	g	CFT073	++	g	c						c						c			c						c	c
a	3357812	g	3493488	ns	a	CFT073	++	a	g						g						g			g						g	g
a	3357814	c	3493490	ns	a	CFT073	++	a	c						c						c			c						c	c
g	3357819	a	3493495	ns	g	CFT073	++	g	g						g						g			g						g	g
t	3357853	g	3493529	s	t	CFT073	++	t	g						g						g			g						g	g
g	3357905	t	3493581	ns	g	CFT073	++	g	a						a						a			a						a	a
c	3357907	a	3493583	ns	c	CFT073	++	c	c						c						c			c						c	c
a	3357918	c	3493594	ns	a	CFT073	++	a	a						a						a			a						a	a
g	3357919	a	3493595	ns	g	CFT073	++	g	g						g						g			g						g	g
c	3357922	t	3493598	s	c	CFT073	++	c	c						c						c			c						c	c
t	3357925	g	3493601	s	t	CFT073	++	t	a						a						a			a						a	a
a	3357929	g	3493605	ns	a	CFT073	++	a	g						g						g			g						g	g
a	3357931	g	3493607	ns	a	CFT073	++	a	a						a						a			a						a	a
c	3357938	a	3493614	ns	c	CFT073	++	c	a						a						a			a						a	a

Clone D base	Clone D site	CFT 073 base	CFT 073 site	mutation type[a]	Inferred ancestral base[b]	lineage inferred to mutate	Support level[c]	ED1a[d]	536	S88	APEC O1	UTI89	E2348/69	ATCC8739	K-12	HS	SMS3-5	IAI39	E24377A	SE11	55989	IAI1	Sakai	UMN026	B4 Sb227	F5 8401	SS Ss046	D1 Sd197	B18 BS512	F2a 2457T	F2a 301
g	3357940	a	3493616	ns	g	CFT073	++	g	a						a						a			a						a	a
g	3358000	a	3493676	s	g	CFT073	++	g	a	a		a			a		a		a		a		a	a				a		a	a
t	3358033	c	3493709	s	t	CFT073	++	t	c	t		t			c		t		t		t		t	t				t		t	t
t	3358063	c	3493739	s	t	CFT073	++	t	c	g		g			c		g		g		c		g	c				g		c	c
g	3358086	c	3493762	ns	g	CFT073	++	g	c	c		c			c		c		c		c		c	c				c		c	c
t	3358087	g	3493763	ns	t	CFT073	++	t	g	c		c			g		c		c		g		c	g				c		g	g
a	3358088	t	3493764	ns	a	CFT073	++	a	t	t		t			t		t		t		t		t	t				t		t	t
c	3358104	g	3493780	ns	c	CFT073	++	c	g	c		c			g		c		c		g		c	g				c		g	g
t	3358126	g	3493802	s	t	CFT073	++	t	t	g		g			g		g		g		g		g	g				g		g	g
g	3358129	a	3493805	s	g	CFT073	++	g	a	t		t			a		t		t		a		t	a				t		a	a
t	3358162	c	3493838	s	t	CFT073	++	t	c	g		g			c		g		g		t		g	c				a		c	c
t	3358177	c	3493853	s	t	CFT073	++	t	c	c		c			c		c		c		t		c	t				c		t	t
t	3358179	g	3493855	ns	t	CFT073	++	t	g	c		c			g		c		c		t		c	a				c		a	a
t	3358181	c	3493857	s	c	clone D	++	c	c	c		c			c		c		c		c		c	c				c		c	c
a	3358205	t	3493881	ns	a	CFT073	++	a	t	a		a			t		a		a		a		a	t				a		t	t
t	3358264	a	3493940	s	t	CFT073	++	t	a	t		t			a		t		t		t		t	a				t		a	a
c	3358330	a	3494006	s	a	clone D	++	a	c	c		c			c		c		c		a		c	c				c		c	c
t	3358425	c	3494101	ns	c	clone D	++	c	c	c		c			c		c		c		c		c	c				c		c	c
t	3358432	g	3494108	s	g	clone D	++	g	g	a		g			g		g		a		g		a	g				g		a	a
t	3358471	c	3494147	s	t	CFT073	++	t	t	t		t			t		t		t		t		t	t				t		t	t
c	3358489	g	3494165	s	c	CFT073	++	c	g	c		c			g		g		c		c		c	g				g		c	c
g	3358498	t	3494174	ns	g	CFT073	++	g	g	g		g			g		g		g		g		g	g				g		g	g
a	3358513	t	3494189	s	a	CFT073	++	a	t	t		a			t		t		t		a		t	a				a		t	t
c	3358514	g	3494190	ns	c	CFT073	++	c	g	g		c			g		g		c		c		c	c				c		g	g
g	3358519	c	3494195	s	g	CFT073	++	g	c	c		g			c		c		g		g		g	g				g		c	c
a	3358520	g	3494196	ns	a	CFT073	++	a	g	g		a			g		g		a		a		a	a				a		g	g
a	3358525	g	3494201	s	a	CFT073	++	a	g	g		a			g		g		g		a		g	a				a		g	g
g	3358526	a	3494202	ns	g	CFT073	++	g	g	g		g			g		g		g		g		g	g				g		g	g

Clone D base	Clone D site	CFT 073 base	CFT 073 site	mutation type[a]	Inferred ancestral base[b]	lineage inferred to mutate	Support level[c]	ED1a[d]	536	S88	APEC O1	UTI89	E2348/69	ATCC8739	K-12	HS	SMS3-5	IAI39	E24377A	SE11	55989	IAI1	Sakai	UMN026	B4 Sb227	F5 8401	SS Ss046	D1 Sd197	B18 BS512	F2a 2457T	F2a 301
g	3358528	a	3494204	ns	g	CFT073	++	g	a	a		g			a		a		g		g		g	g				g		a	a
c	3358624	t	3494300	s	c	CFT073	++	c	c	c		c			c		c		c		t		c	t				c		c	c
c	3358625	a	3494301	ns	c	CFT073	++	c	c	c		c			c		c		c		a		c	a				c		c	c
t	3358630	c	3494306	s	t	CFT073	++	t	t	t		t			t		t		t		c		t	c				t		t	t
g	3358634	a	3494310	ns	a	clone D	++	a	a	a		g			g		g		a		a		g	a				g		a	a
t	3358635	c	3494311	ns	c	clone D	++	c	c	c		t			t		t		c		c		t	c				t		t	t
g	3358642	c	3494318	s	g	CFT073	+	a	g	g		g			g		g		g		c		a	c				g		c	c
t	3358666	a	3494342	s	t	CFT073	++	t	t	t		t			t		t		t		t		t	a				t		t	t
c	3358690	t	3494366	s	c	CFT073	++	c	c	c		c			c		c		c		t		c	t				c		c	c
a	3358707	g	3494383	ns	a	CFT073	++	a	a	a		a			a		a		a		g		a	g				a		a	a
c	3358726	a	3494402	s	c	CFT073	++	c	c	c		c			c		c		c		a		c	a				c		c	c
a	3358743	t	3494419	ns	t	clone D	++	t	t	t		t			t		t		t		t		t	t				t		t	t
a	3358768	c	3494444	s	c	clone D	++	c	c	c		c			c		c		c		c		c	c				c		c	c
a	3358803	g	3494479	ns	g	clone D	++	g	a	a		a			a		a		a		g		a	g				a		a	a
a	3358804	g	3494480	ns	a	CFT073	++	a	a	a		a			a		a		a		g		a	g				a		a	a
t	3358813	c	3494489	s	c	clone D	++	c	c	t		c			t		c		t		c		c	c				t		c	c
a	3358825	g	3494501	s	g	clone D	++	g	a	a		a			a		a		a		g		a	g				a		a	a
c	3358831	g	3494507	s	c	CFT073	++	c	c	c		c			c		c		c		g		c	g				c		c	c
c	3358843	t	3494519	s	t	clone D	++	t	c	c		c			t		c		c		t		c	t				c		c	c
c	3358844	a	3494520	ns	a	clone D	++	a	c	c		c			a		c		c		a		c	a				c		c	c
t	3358849	c	3494525	s	c	clone D	++	c	t	t		t			c		t		t		c		t	c				t		t	t
a	3358959	c	3494635	ns	a	CFT073	++	a	a	a		a			c		a		a		c		a	c				a		a	a
a	3358961	c	3494637	ns	a	CFT073	++	a	a	a		a			c		a		a		c		a	c				a		a	a
c	3358969	t	3494645	s	c	CFT073	++	c	t	c		c			t		c		c		t		c	t				c		c	c
a	3358976	g	3494652	ns	g	clone D	++	g	g	a		g			g		g		g		g		g	g				a		g	g
g	3358985	a	3494661	ns	a	clone D	++	a	a	c		a			a		c		c		a		a	a				g		a	a
a	3358991	c	3494667	ns	c	clone D	++	c	c	a		c			a		c		c		c		a	c				a		c	c
t	3359002	g	3494678	s	g	clone D	++	g	g	g		g			g		g		g		g		g	g				t		g	g

Clone D base	Clone D site	CFT 073 base	CFT 073 site	mutation type[a]	Inferred ancestral base[b]	lineage inferred to mutate	Support level[c]	ED1a[d]	536	S88	APEC O1	UTI89	E2348/69	ATCC8739	K-12	HS	SMS3-5	IAI39	E24377A	SE11	55989	IAI1	Sakai	UMN026	B4 Sb227	F5 8401	SS Ss046	D1 Sd197	B18 BS512	F2a 2457T	F2a 301
t	3359026	c	3494702	s	t	CFT073	++	t	c	t		t			t		t		t		c		t	c				t		t	t
a	3359053	t	3494729	s	t	clone D	+	g	t	t		a			a		t		t		t		t	t				a		g	g
c	3359056	t	3494732	s	c	CFT073	++	c	c	c		c			t		c		c		t		c	t				c		c	c
a	3359060	c	3494736	ns	a	CFT073	++	a	a	a		a			c		a		a		c		a	c				a		a	a
c	3359062	t	3494738	ns	c	CFT073	++	c	c	c		c			t		c		c		t		c	t				c		c	c
a	3359063	g	3494739	ns	a	CFT073	++	a	a	a		a			g		a		a		g		a	g				a		a	a
t	3359068	g	3494744	s	t	CFT073	++	t	g	t		t			g		g		g		g		g	g				t		t	t
a	3359071	c	3494747	s	a	CFT073	++	a	a	a		a			t		a		a		c		a	c				a		a	a
g	3359072	t	3494748	ns	g	CFT073	++	g	g	g		g			t		g		g		t		g	t				g		g	g
g	3359075	a	3494751	ns	g	CFT073	++	g	g	g		g			a		g		g		a		g	a				g		g	g
a	3359077	t	3494753	ns	a	CFT073	++	a	a	c		a			t		c		c		c		a	c				a		a	a
c	3359080	a	3494756	s	c	CFT073	++	c	c	c		c			a		c		c		a		c	a				c		c	c
t	3359086	c	3494762	s	t	CFT073	++	t	t	t		t			c		t		t		c		t	c				t		t	t
a	3359087	g	3494763	ns	a	CFT073	++	a	a	a		a			g		a		a		g		a	g				a		a	a
a	3359088	g	3494764	ns	a	CFT073	++	a	a	a		a			g		a		a		g		a	g				a		a	a
c	3359089	g	3494765	ns	c	CFT073	++	c	c	c		c			g		c		c		g		c	g				c		c	c
a	3359101	t	3494777	s	a	CFT073	++	a	a	a		a			t		a		a		t		a	a				a		a	a
g	3359104	c	3494780	s	g	CFT073	++	g	g	g		g			c		g		g		c		g	c				g		g	g
c	3359107	a	3494783	s	a	clone D	++	a	a	c		c			a		c		c		a		c	a				c		a	a
g	3359109	a	3494785	ns	a	clone D	++	a	a	g		g			a		a		g		a		g	a				g		a	a
g	3359119	a	3494795	s	g	CFT073	++	g	g	g		g			a		g		g		t		g	t				g		g	g
t	3359200	c	3494876	s	c	clone D	++	c	c	c		t			c		c		t		c		c	c				t		c	c
a	3359211	g	3494887	ns	g	clone D	++	g	g	g		g			g		g		g		g		g	g				g		g	g
c	3359224	g	3494900	s	?	?	+/-	t	c	t		t			g		c		t		t		c	t				t		t	t
g	3359242	a	3494918	s	g	CFT073	++	g	g	g		a			a		g		g		g		g	g				g		a	a
t	3359277	c	3494953	nc	c	clone D	++	c	-	t		-			c		-		t		c			c				t		c	c
a	3359288	g	3494964	nc	g	clone D	++	g	g	g		g			g		g		g		g			g				g		g	g
t	3359298	c	3494974	nc	c	clone D	++	c	c	c		c			c		c		c		c			c				c		c	c

Clone D base	Clone D site	CFT 073 base	CFT 073 site	mutation type[a]	Inferred ancestral base[b]	lineage inferred to mutate	Support level[c]	ED1a[d]	536	S88	APEC O1	UTI89	E2348/69	ATCC8739	K-12	HS	SMS3-5	IAI39	E24377A	SE11	55989	IAI1	Sakai	UMN026	B4 Sb227	F5 8401	SS Ss046	D1 Sd197	B18 BS512	F2a 2457T	F2a 301
a	3359324	c	3495000	nc	?	?	+/-	t							c						-			t						t	t
c	3359506	a	3502073	nc	c	CFT073	++	c													c			c						c	c
a	3359631	g	3502198	nc	g	clone D	++	g													g			g						g	g
g	3360045	a	3502612	nc	g	CFT073	++	g													g			g						g	g
t	3360247	a	3502814	nc	t	CFT073	++	t													t			t						t	t
t	3361096	c	3503663	ns	c	clone D	++	c	c	c									c		c			c				c		c	c
g	3361932	a	3504499	ns	g	CFT073	++	g	g	g	g	g	g	g			g	g	g		g		g	g				g	g	g	g
g	3362618	a	3505185	ns	g	CFT073	++	g	g	g	g	g	g		a		g	g	g		g		g	g				g	g	g	g
t	3362901	c	3505468	ns	t	CFT073	++	t	c	c	c	c	c		c		c	c	c		c		c	c				c	c	c	c
a	3363111	g	3505678	ns	a	CFT073	++	a	g	a	a	g	g		a		g	g	g		a		g	g				g	a	g	g
t	3363486	c	3506053	s	c	clone D	++	c	c	c	c	c	c		c		c	c	c		c		c	c				c	c	c	c
c	3363551	t	3506118	ns	c	CFT073	++	c	c	c	c	t	c		c		t	c	c		c		c	t				t	c	c	c
a	3363561	g	3506128	s	g	clone D	++	g	g	g	g	g			g		g	g	g		g		g	g				g	g	g	g
g	3363566	t	3506133	ns	g	CFT073	++	g	-	-	-	t			-		t	-	g		-		-	t				t	-	g	g
c	3363568	a	3506135	ns	a	clone D	++	a	-	-	-	a			-		a	-	a		-		-	a				a	-	a	a
g	3363569	a	3506136	ns	g	CFT073	++	g	g	g	g	g			g		g	-	g		g		g	g				g	g	g	g
a	3363577	t	3506144	ns	t	clone D	++	t	t	t	t	t			t		t	t	a		t		t	t				t	t	t	t
g	3363845	a	3506412	ns	g	CFT073	+					g					a							g				a			
c	3363861	t	3506428	ns	c	CFT073	+					c					t							c				t			
a	3363863	t	3506430	ns	a	CFT073	+					a					a							a				t			
g	3363866	a	3506433	ns	g	CFT073	+					g					a							g				a			
c	3363870	g	3506437	ns	c	CFT073	+					c					g							c				g			
a	3363877	g	3506444	ns	a	CFT073	+					a					g							a				g			
a	3363887	g	3506454	ns	a	CFT073	+					a					g							a				g			
t	3363888	c	3506455	ns	t	CFT073	+					t					t							t				c			
c	3363893	a	3506460	ns	c	CFT073	+					c					c							c				a			
t	3363896	g	3506463	s	t	CFT073	+					t					g							t				g			
c	3363902	g	3506469	ns	c	CFT073	+					c					c							c				g			

Clone D base	Clone D site	CFT 073 base	CFT 073 site	mutation type[a]	Inferred ancestral base[b]	lineage inferred to mutate	Support level[c]	ED1a[d]	536	S88	APEC O1	UTI89	E2348/69	ATCC8739	K-12	HS	SMS3-5	IAI39	E24377A	SE11	55989	IAI1	Sakai	UMN026	B4 Sb227	F5 8401	SS Ss046	D1 Sd197	B18 BS512	F2a 2457T	F2a 301
c	3363920	t	3506487	ns	c	CFT073	+					c					c							c				t			
g	3363923	t	3506490	ns	g	CFT073	+					g					g							g				t			
c	3364022	t	3506589	s	c	CFT073	+					c					c							c				c			
c	3364025	t	3506592	s	c	CFT073	+					c					c							c				c			
a	3364345	g	3506912	nc	a	CFT073	++	a	a	a	a	a	a		a	a	a	a	a		a		a	a				a	a	a	a
g	3364353	a	3506920	nc	g	CFT073	++	g	g	g	g	g	g		g	g	g	g	a		g		g	g				g	g	g	g
c	3364356	t	3506923	nc	c	CFT073	++	c	c	c	c	c	c		c	c	c	c	t		c		c	c				c	c	c	c
c	3364362	t	3506929	nc	c	CFT073	++	c	c	t	t	c	t		c	c	c	c	t		t		c	c				c	c	c	c
g	3364365	c	3506932	nc	g	CFT073	++	g	c	t	t	g	c		g	c	g	c	c		g		g	c				g	g	g	g
t	3364369	c	3506936	nc	c	clone D	++	c	c	c	c	c	c		c	c	c	c	c		c		c	c				c	c	c	c
a	3364425	g	3506992	nc	a	CFT073	++	a	a	a	a	a	a		a	a	a	a	a		a		a	g				a	a	a	a
g	3364438	a	3507005	nc	g	CFT073	++	g	g	g	g	a	g		g	g	a	g	g		g		g	a				a	g	g	g
g	3364449	a	3507016	nc	g	CFT073	++	g	g	g	g	a	a		g	g	a	a	a		g		g	a				a	g	g	g
c	3364457	t	3507024	nc	t	clone D	++	t	t	t	t	t	t		t	t	t	t	t		t		c	t				t	t	t	t
c	3364467	t	3507034	nc	c	CFT073	++	c	c	c	c	t	c		c	c	t	c	c		c		c	t				t	c	c	c
g	3364473	a	3507040	nc	g	CFT073	++	g	g	g	g	a	g		g	g	a	a	g		g		g	a				a	g	g	g
c	3364490	t	3507057	nc	t	clone D	++	t	t	t	t	t	t		t	t	t	t	t		t		t	t				t	t	t	t
a	3364545	g	3507112	nc	g	clone D	++	g	g	g	g	g	g		g	a	g	g	g		g		g	g				a	g	a	a
a	3364560	g	3507127	nc	g	clone D	+	t	a	g	g	g	g		g	g	g	g	g		g		g	g				g	g	g	g
a	3364572	t	3507139	nc	a	CFT073	++	a	a	a	a	a	a		a	a	t	a	t		a		a	t				a	a	a	a
t	3364578	c	3507145	nc	c	clone D	++	c	c	c	c	t	c		t	c	c	t	c		c		t	c				t	t	c	c
g	3364584	c	3507151	nc	c	clone D	++	c	c	c	c	t	c		c	c	c	t	c		c		c	c				t	c	c	c
c	3364587	g	3507154	nc	c	CFT073	+	t	c	c	c	c	c		c	c	c	c	c		c		g	g				c	c	c	c
a	3364595	g	3507162	nc	g	clone D	++	g	g	g	g	g	g		a	g	g	g	g		g		g	g				g	g	g	g
g	3364620	t	3507187	nc	g	CFT073	++	g	a	g	g	g			g	g	t	g	g		g		g	t				g	g		
a	3364621	g	3507188	nc	a	CFT073	++	a	a	a	a	a			a	a	g	a	a		a		a	g				a	a		
c	3364624	a	3507191	nc	c	CFT073	++	c	t	c	c	c			c	c	a	c	c		c		c	a				c	c		
t	3364631	c	3507198	nc	c	clone D	++	c	c	c	c	c			t	c	c	c	c		c		c	c				c	t		

Clone D base	Clone D site	CFT 073 base	CFT 073 site	mutation type[a]	Inferred ancestral base[b]	lineage inferred to mutate	Support level[c]	ED1a[d]	536	S88	APEC O1	UTI89	E2348/69	ATCC8739	K-12	HS	SMS3-5	IAI39	E24377A	SE11	55989	IAI1	Sakai	UMN026	B4 Sb227	F5 8401	SS Ss046	D1 Sd197	B18 BS512	F2a 2457T	F2a 301
a	3364633	g	3507200	nc	a	CFT073	++	a	a	a	a	a			a	a	g	a	a		a		a	g				a	a		
a	3364637	g	3507204	nc	g	clone D	++	g	g	g	g	g			t	g	g	g	g		g		g	g				g	t		
t	3364643	c	3507209	nc	c	clone D	++	c	c	t	t	t			t	c	c	c	c		t		t	c				c	t		
a	3364648	g	3507214	nc	g	clone D	++	g	g	a	a	a			a	a	g	g	g		a		a	g				a	a		
c	3364655	t	3507221	nc	c	CFT073	++	c	c	c	c	c			c	c	c	t	t		c		c	t				c	c		
a	3364664	c	3507230	nc	c	clone D	++	c	c	c	c	c			a	c	c	c	c		c		c	c				c	a		
t	3364675	c	3507241	nc	c	clone D	++	c	t	c	c	c			t	t	c	c	c		c		t	c				t	t		
a	3364680	t	3507246	nc	a	CFT073	++	a	a	a	a	a			a	a	a	a	a		a		a	t				a	a		
g	3364812	a	3507379	ns	g	CFT073	++	g	a	g	g	g	g	a	g	g	g	g	g		g		g	g				g	g	g	g
a	3364944	t	3507511	ns	t	clone D	++	t	t	t	t	t	t	t	t	t	t	t	t		t		t	t				t	t	t	t
t	3365279	c	3507846	ns	c	clone D	++	c	c	c	c	c	c	t			-	c	c		c		c	t				c		c	c
a	3365280	c	3507847	ns	?	?	+/-	g	g	c	c	g	g	c			-	g	c		c		g	a				g		c	c
a	3365554	g	3508121	ns	g	clone D	++	g	g	g	g	g	g	g			g	g	g		g		g	g				g		g	g
t	3365639	g	3508206	nc	g	clone D	++	g	g	g	g	g	g	a			g	g	g		g		g	g				g		g	g
t	3365781	a	3508348	nc	t	CFT073	++	t	t	t	t	t	t	t			a	t	t		t		t	t				t		t	t
c	3365801	a	3508368	nc	c	CFT073	++	c	c	c	c	c	c	a			a	c	c		c		c	a				c		c	c
c	3365825	t	3508392	nc	c	CFT073	++	c	c	c	c	c	c	t			c	c	c		c		c	t				c		c	c
t	3365848	c	3508415	nc	c	clone D	++	c	t	t	t	c	c	c			t	t	t		t		c	t				c		t	t
c	3365850	g	3508417	nc	g	clone D	++	g	c	c	c	g	g	g			c	c	c		c		g	c				g		c	c
g	3365864	a	3508431	nc	a	clone D	++	a	a	g	g	g	a	c			g	g	g		g		a	g				g		g	g
a	3365870	c	3508437	nc	c	clone D	++	c	c	a	a	a	c	a			a	a	a		c		c	a				a		c	c
a	3365871	t	3508438	nc	t	clone D	++	t	a	a	a	a	t	t			a	a	a		t		t	a				a		t	t
a	3365872	g	3508439	nc	g	clone D	++	g	g	a	a	a	g	-			a	a	a		g		g	a				a		g	g
c	3365874	a	3508441	nc	c	CFT073	+	g	a	c	c	c	a	-			c	c	c		a		a	c				c		a	a
a	3365883	t	3508450	nc	t	clone D	++	t	a	a	a	a	t	-			t	a	a		t		t	a				a		t	t
a	3365885	t	3508452	nc	t	clone D	++	t	c	c	c	a	t	-			t	c	c		t		t	a				a		t	t
g	3365890	t	3508457	nc	t	clone D	++	t	t	a	a	g	t	a			t	a	a		t		t	g				g		t	t
a	3365896	t	3508463	s	t	clone D	++	t	a	a	a	a	a	a			t	a	a		t		a	a				a		t	t

Clone D base	Clone D site	CFT 073 base	CFT 073 site	mutation type[a]	Inferred ancestral base[b]	lineage inferred to mutate	Support level[c]	ED1a[d]	536	S88	APEC O1	UTI89	E2348/69	ATCC8739	K-12	HS	SMS3-5	IAI39	E24377A	SE11	55989	IAI1	Sakai	UMN026	B4 Sb227	F5 8401	SS Ss046	D1 Sd197	B18 BS512	F2a 2457T	F2a 301
a	3365906	g	3508473	ns	g	clone D	++	g	a	a	a	a	a	c			c	a	a		g		a	a				a		g	g
t	3365907	c	3508474	ns	t	CFT073	++	t	t	t	t	t	t	t			t	t	t		c		t	t				t		c	c
c	3365914	t	3508481	s	t	clone D	++	t	t	t	t	c	t	a			t	t	t		t		t	c				c		t	t
c	3365921	g	3508488	ns	g	clone D	++	g	g	g	g	g	g	g			g	g	g		g		g	c				g		g	g
a	3365932	g	3508499	s	g	clone D	++	g	a	g	g	a	g	c			g	g	g		g		g	a				a		g	g
c	3365941	a	3508508	ns	c	CFT073	++	c	c	c	c	c	c	c			c	c	c		c		c	c				c		c	c
g	3365964	a	3508531	ns	g	CFT073	++	g	g	g	g	g	g	a			g	g	g		g		g	g				g		g	g
g	3365986	a	3508553	s	g	CFT073	++	g	g	t	t	g	g	t			g	t	g		g		g	g				g		g	g
g	3365990	a	3508557	ns	a	clone D	++	a	g	g	g	a	a	g			a	g	a		a		a	g				a		a	a
g	3365992	a	3508559	ns	a	clone D	++	a	g	a	a	a	a	g			a	a	a		a		a	g				a		a	a
a	3366028	t	3508595	s	t	clone D	++	t	a	a	a	t	t	c			t	a	t		t		t	t				t		t	t
a	3366040	t	3508607	s	a	CFT073	++	a	a	g	g	a	g	g			a	g	a		a		g	a				a		a	a
a	3366046	g	3508613	s	a	CFT073	++	a	a	a	a	a	g	c			a	a	a		a		g	g				a		a	a
t	3366052	c	3508619	s	t	CFT073	++	t	t	c	c	t	c	t			t	c	t		t		c	c				t		t	t
g	3366068	a	3508635	ns	g	CFT073	++	g	g	g	g	g	g	g			g	g	g		g		g	g				g		g	g
t	3366072	g	3508639	ns	t	CFT073	++	t	t	t	t	t	c	t			c	t	c		t		c	g				t		t	t
g	3366082	c	3508649	s	c	clone D	++	c	g	g	g	c	c	t			c	g	c		c		c	c				c		c	c
g	3366127	t	3508694	s	g	CFT073	++	g	t	g	g	t	c	a			c	g	t		g		c	g				t		g	g
c	3366133	g	3508700	s	c	CFT073	++	c	c	a	a	a	a	g			a	a	a		c		a	c				a		c	c
t	3366157	c	3508724	s	c	clone D	++	c	t	t	t	t	t	t			t	t	c		t		t	t				t		t	t
c	3366160	t	3508727	s	t	clone D	++	t	c	c	c	c	t	c			t	c	t		t		t	c				c		t	t
t	3366163	c	3508730	s	c	clone D	++	c	t	t	t	t	t	t			c	t	c		t		t	t				t		t	t
c	3366166	t	3508733	s	c	CFT073	++	c	t	a	a	c	c	c			a	a	c		c		c	c				c		c	c
a	3366192	g	3508759	ns	g	clone D	++	g	g	g	g	g	g	a			g	g	g		g		g	g				g		g	g
g	3366197	a	3508764	ns	g	CFT073	++	g	g	g	g	g	g	g			g	g	g		g		g	g				g		g	g
g	3366205	t	3508772	s	g	CFT073	++	g	t	t	t	t	g	t			t	t	g		g		g	g				t		g	g
a	3366208	c	3508775	s	a	CFT073	++	a	c	c	c	c	a	g			c	c	a		t		a	a				c		t	t
a	3366228	g	3508795	ns	g	clone D	++	g	g	g	g	g	g	g			g	g	g		g		g	a				g		g	g

Clone D base	Clone D site	CFT 073 base	CFT 073 site	mutation type[a]	Inferred ancestral base[b]	lineage inferred to mutate	Support level[c]	ED1a[d]	536	S88	APEC O1	UTI89	E2348/69	ATCC8739	K-12	HS	SMS3-5	IAI39	E24377A	SE11	55989	IAI1	Sakai	UMN026	B4 Sb227	F5 8401	SS Ss046	D1 Sd197	B18 BS512	F2a 2457T	F2a 301
c	3366244	g	3508811	s	c	CFT073	++	c	g	a	a	a	g	g			g	a	g		g		g	c				a		g	g
c	3366262	t	3508829	s	c	CFT073	++	c	t	t	t	t	t	c			t	t	t		t		t	c				t		t	t
g	3366280	a	3508847	s	a	clone D	++	a	g	a	a	a	a	a			a	a	a		a		a	g				a		a	a
t	3366286	c	3508853	s	c	clone D	++	c	t	c	c	t	c	t			c	c	c		c		c	t				t		c	c
c	3366289	t	3508856	s	t	clone D	++	t	c	t	t	t	t	t			t	t	t		t		t	c				t		t	t
t	3366295	c	3508862	s	c	clone D	++	c	c	c	c	t	c	c			c	c	c		c		c	t				t		c	c
g	3366298	a	3508865	s	a	clone D	++	a	a	a	a	g	a	g			a	a	a		a		a	g				g		a	a
g	3366302	a	3508869	ns	g	CFT073	++	g	g	g	g	g	g	g			g	g	g		g		g	g				g		g	g
t	3366319	c	3508886	s	t	CFT073	++	t	t	c	c	t	t	c			c	c	t		c		t	t				t		c	c
c	3366328	g	3508895	ns	?	?	+/-	a	g	a	a	a	g	g			a	a	g		a		g	c				a		a	a
a	3366340	g	3508907	s	a	CFT073	++	a	a	a	a	a	a	c			a	a	g		g		a	a				a		g	g
t	3366343	a	3508910	s	a	clone D	++	a	g	g	g	a	g	a			g	g	g		a		g	t				a		a	a
c	3366353	g	3508920	ns	g	clone D	++	g	g	g	g	c	g	g			g	g	g		g		g	c				c		g	g
a	3366361	g	3508928	s	a	CFT073	++	a	g	g	g	a	a	a			g	g	g		a		a	a				a		a	a
g	3366364	a	3508931	s	g	CFT073	++	g	a	a	a	g	g	t			a	a	a		g		g	t				g		g	g
c	3366379	t	3508946	s	c	CFT073	++	c	c	c	c	c	c	t			c	c	c		c		c	c				c		c	c
t	3366388	c	3508955	s	t	CFT073	++	t	t	t	t	t	t	t			t	t	t		c		t	t				t		c	c
g	3366390	c	3508957	ns	c	clone D	++	c	g	g	g	g	g	c			g	g	c		c		g	g				g		c	c
c	3366391	t	3508958	ns	c	CFT073	++	c	c	c	c	c	c	-			c	c	a		a		c	c				c		a	a
c	3366406	a	3508973	s	c	CFT073	++	c	c	c	c	c	c	g			c	c	c		c		c	c				c		c	c
a	3366408	g	3508975	ns	g	clone D	++	g	g	g	g	a	g	c			g	g	g		g		g	a				a		g	g
t	3366415	c	3508982	s	t	CFT073	++	t	t	t	t	t	t	t			t	t	t		t		t	t				t		t	t
c	3366418	t	3508985	s	c	CFT073	++	c	c	c	c	c	c	c			c	c	c		c		c	c				c		c	c
g	3366419	a	3508986	ns	g	CFT073	++	g	g	g	g	g	g	g			g	g	g		g		g	g				g		g	g
a	3366445	g	3509012	s	a	CFT073	++	a	g	g	g	g	g	g			g	g	g		g		g	a				g		g	g
g	3366449	a	3509016	ns	g	CFT073	++	g	g	g	g	g	g	g			g	g	g		g		g	g				g		g	g
g	3366454	t	3509021	ns	g	CFT073	++	g	t	t	t	g	t	t			t	t	t		t		t	g				g		t	t
g	3366469	a	3509036	s	g	CFT073	++	g	g	g	g	g	a	c			g	g	g		g		a	g				g		g	g

Clone D base	Clone D site	CFT 073 base	CFT 073 site	mutation type[a]	Inferred ancestral base[b]	lineage inferred to mutate	Support level[c]	ED1a[d]	536	S88	APEC O1	UTI89	E2348/69	ATCC8739	K-12	HS	SMS3-5	IAI39	E24377A	SE11	55989	IAI1	Sakai	UMN026	B4 Sb227	F5 8401	SS Ss046	D1 Sd197	B18 BS512	F2a 2457T	F2a 301
g	3366476	a	3509043	ns	g	CFT073	++	g	g	g	g	g	g	c			g	g	g		a		g	g				g		a	a
a	3366478	t	3509045	ns	a	CFT073	++	a	a	a	a	a	t	t			a	a	t		t		t	a				a		t	t
t	3366481	c	3509048	s	t	CFT073	++	t	t	t	t	t	c	c			t	t	t		c		c	t				t		c	c
t	3366490	c	3509057	s	c	clone D	++	c	c	c	c	c	c	t			c	c	c		c		c	t				c		c	c
c	3366493	t	3509060	s	c	CFT073	++	c	t	c	c	c	t	c			c	c	t		c		t	c				c		c	c
g	3366508	a	3509075	s	a	clone D	++	a	a	g	g	g	g	g			g	g	a		a		g	g				g		a	a
g	3366522	a	3509089	ns	g	CFT073	++	g	g	g	g	g	g	g			g	g	g		g		g	g				g		g	g
c	3366532	t	3509099	s	c	CFT073	++	c	c	t	t	c	c	t			c	t	c		c		c	c				c		c	c
g	3366540	a	3509107	ns	g	CFT073	++	g	a	a	a	a	a	a			a	a	a		a		a	g				a		a	a
t	3366550	c	3509117	s	t	CFT073	++	t	c	c	c	c	c	c			c	c	c		c		c	t				c		c	c
t	3366555	c	3509122	ns	t	CFT073	++	t	c	c	c	c	c	c			c	c	c		c		c	t				c		c	c
c	3366556	t	3509123	ns	c	CFT073	++	c	c	c	c	t	c	c			c	c	t		t		c	c				t		t	t
g	3366562	t	3509129	s	g	CFT073	++	g	t	t	t	t	t	t			t	t	t		t		t	g				t		t	t
a	3366570	g	3509137	ns	a	CFT073	++	a	a	a	a	a	a	a			a	a	a		g		a	a				a		g	g
g	3366579	a	3509146	ns	g	CFT073	++	g	a	a	a	g	a	a			a	a	a		a		a	g				g		a	a
c	3366580	g	3509147	ns	c	CFT073	++	c	g	c	c	g	g	-			g	c	g		g		g	c				g		g	g
c	3366592	t	3509159	s	c	CFT073	++	c	t	t	t	a	t	c			t	t	t		t		t	c				a		t	t
c	3366597	a	3509164	ns	a	clone D	+	t	a	c	c	a	a	a			a	c	a		a		a	c				a		a	a
t	3366599	c	3509166	ns	t	CFT073	++	t	t	t	t	t	a	c			g	t	g		c		a	t				t		c	c
t	3366610	c	3509177	s	t	CFT073	++	t	t	t	t	t	t	t			t	t	t		c		t	t				t		c	c
g	3366613	a	3509180	s	g	CFT073	++	g	g	g	g	g	g	g			g	g	g		g		g	g				g		g	g
a	3367165	g	3509732	ns	a	CFT073	+												g					a							
a	3368458	g	3511026	nc	a	CFT073	++	a	a	a	a	a					a							a							
g	3368624	c	3511192	ns	g	CFT073	++	g	t	g	g	g					g							g							
t	3369004	c	3511572	s	c	clone D	++	c	c	c	c	c					c							t							
t	3369013	c	3511581	ns	c	clone D	++	c	c	c	c	c					c							t							
t	3369020	c	3511588	ns	t	CFT073	++	t	t	t	t	t					t							t							
a	3369073	g	3511641	nc	g	clone D	++	g	g	g	g	g					g							a							

Clone D base	Clone D site	CFT 073 base	CFT 073 site	mutation type[a]	Inferred ancestral base[b]	lineage inferred to mutate	Support level[c]	ED1a[d]	536	S88	APEC O1	UTI89	E2348/69	ATCC8739	K-12	HS	SMS3-5	IAI39	E24377A	SE11	55989	IAI1	Sakai	UMN026	B4 Sb227	F5 8401	SS Ss046	D1 Sd197	B18 BS512	F2a 2457T	F2a 301
c	3369091	a	3511659	nc	c	CFT073	++	c	c	c	c	c					c							c							
c	3369092	a	3511660	nc	c	CFT073	++	c	c	c	c	c					c							c							
g	3369093	t	3511661	nc	a	clone D	++	a	g	g	t	g					g							g							
a	3369094	c	3511662	nc	c	clone D	++	c	c	c	c	c					c							a							
t	3369096	c	3511664	nc	c	clone D	++	c	c	-	-	-					c							t							
t	3369211	c	3511779	nc	t	CFT073	+	g	t	t	t	t					g							t							
c	3369240	t	3511808	nc	t	clone D	++	t	t	t	t	t					t							c							
g	3369479	a	3512047	nc	g	CFT073	++	g	g	g	g	g					g							g							
g	3369745	a	3512313	ns	g	CFT073	++	g	g	g	g	g					g							g							
c	3369916	t	3512484	s	c	CFT073	++	c	c	c	c	c					c							c							
c	3369977	t	3512545	s	c	CFT073	++	c	c	c	c	c					c							c							
c	3370318	t	3512886	s	c	CFT073	++	c	c	c	c	c					c	c						c							
a	3370335	g	3512903	ns	a	CFT073	++	a	a	a	a	a					a	a						a							
c	3370351	t	3512919	s	c	CFT073	++	c	c	c	c	c					t	c						c							
g	3370522	t	3513090	nc	g	CFT073	++	g	g	t	t	t					t	g						g							
a	3370656	g	3513224	s	a	CFT073	++	a	a	a	a	a					a	a						a							
g	3370665	a	3513233	s	g	CFT073	++	g	g	g	g	g					g	g						g							
t	3371250	c	3513818	s	t	CFT073	++	t	t	t	t	t					t	t						t							
c	3371671	t	3514239	s	c	CFT073	++	c	c	c	c	c					c	c						c							
g	3371673	a	3514241	s	g	CFT073	++	g	g	g	g	g					g	g						g							
a	3371811	g	3514379	ns	g	clone D	++	g	g	g	g	g					g	g						a							
c	3371812	t	3514380	ns	c	CFT073	++	c	c	c	c	c					c	c						c							
t	3372174	c	3514742	s	c	clone D	++	c	c	c	c	c					c	c						t							
c	3372413	t	3514981	s	c	CFT073	++	c	c	c	c	c					c	c						c							
a	3372449	g	3515017	s	g	clone D	++	g	g	g	g	g					g	g						a							
a	3372491	g	3515059	s	g	clone D	++	g	g	g	g	g					g	g						a							
a	3372695	g	3515263	s	a	CFT073	++	a	a	a	a	a					a	a						a							
t	3372698	c	3515266	s	t	CFT073	++	t	t	t	t	t					t	t						t							

Clone D base	Clone D site	CFT 073 base	CFT 073 site	mutation type[a]	Inferred ancestral base[b]	lineage inferred to mutate	Support level[c]	ED1a[d]	536	S88	APEC O1	UTI89	E2348/69	ATCC8739	K-12	HS	SMS3-5	IAI39	E24377A	SE11	55989	IAI1	Sakai	UMN026	B4 Sb227	F5 8401	SS Ss046	D1 Sd197	B18 BS512	F2a 2457T	F2a 301
c	3372734	t	3515302	s	c	CFT073	++	c	c	c	c	c					c	c						c							
t	3372789	c	3515357	s	c	clone D	++	c	c	c	c	c					c	c						t							
g	3372815	a	3515383	s	g	CFT073	++	g	g	g	g	g					g	g						g							
c	3372938	a	3515506	s	a	clone D	++	a	a	a	a	a					a	a						c							
g	3373148	a	3515716	s	a	clone D	++	a	a	a	a	a					a	a						g							
a	3373181	g	3515749	s	g	clone D	++	g	g	g	g	g					g	g						a							
g	3373184	t	3515752	s	t	clone D	++	t	t	t	t	t					t	t						g							
c	3373214	t	3515782	s	t	clone D	++	t	t	t	t	t					t	t						c							
g	3373274	a	3515842	s	a	clone D	++	a	a	t	t	t					g	a						g							
a	3373277	c	3515845	s	c	clone D	++	c	c	c	c	c					a	c						a							
t	3373286	a	3515854	s	a	clone D	++	a	a	a	a	a					t	a						t							
a	3373295	g	3515863	s	g	clone D	++	g	g	g	g	g					g	g						a							
g	3373307	a	3515875	s	a	clone D	++	a	a	a	a	a					a	a						g							
t	3373310	g	3515878	s	g	clone D	++	g	g	g	g	g					g	g						t							
c	3373352	t	3515920	s	c	CFT073	++	c	c	c	c	c					c	c						c							
g	3373430	a	3515998	nc	a	clone D	++	a	a	a	a	a					g	a						g							
a	3373448	t	3516016	s	a	CFT073	++	a	t	a	a	a					a	a						a							
t	3373460	c	3516028	s	t	CFT073	++	t	c	t	t	t					t	t						t							
c	3373469	t	3516037	s	t	clone D	++	t	t	t	t	t					c	c						c							
c	3373478	a	3516046	s	a	clone D	++	a	a	a	a	a					c	c						c							
c	3373561	t	3516129	ns	c	CFT073	++	c	c	c	c	c					c	c						c							
c	3373597	a	3516165	ns	a	clone D	++	a	c	a	a	a					c	c						c							
c	3373608	a	3516176	ns	a	clone D	++	a	c	a	a	a					a	a						c							
a	3373616	g	3516184	s	g	clone D	++	g	g	g	g	g					g	g						a							
g	3373622	a	3516190	s	a	clone D	++	a	a	a	a	a					a	g						g							
g	3373635	t	3516203	ns	g	CFT073	++	g	g	g	g	g					g	g						g							
t	3373713	a	3516281	ns	t	CFT073	++	t	t	t	t	t					t	t						t							
g	3373751	a	3516319	s	g	CFT073	++	g	g	g	g	g					g	g						g							

Clone D base	Clone D site	CFT 073 base	CFT 073 site	mutation type[a]	Inferred ancestral base[b]	lineage inferred to mutate	Support level[c]	ED1a[d]	536	S88	APEC O1	UTI89	E2348/69	ATCC8739	K-12	HS	SMS3-5	IAI39	E24377A	SE11	55989	IAI1	Sakai	UMN026	B4 Sb227	F5 8401	SS Ss046	D1 Sd197	B18 BS512	F2a 2457T	F2a 301
a	3373762	g	3516330	ns	a	CFT073	++	a	a	a	a	a					a	a						a							
c	3373770	t	3516338	s	t	clone D	++	t	t	t	t	t					t	t						c							
c	3373787	t	3516355	s	t	clone D	++	t	t	t	t	t					t	t						c							
t	3373790	c	3516358	s	c	clone D	++	c	c	c	c	c					c	t						t							
g	3373802	a	3516370	s	a	clone D	++	a	a	a	a	a					a	g						g							
c	3373834	a	3516402	ns	a	clone D	++	a	a	a	a	a					a	a						c							
g	3373842	a	3516410	ns	a	clone D	++	a	a	a	a	a					a	a						g							
t	3373844	c	3516412	ns	c	clone D	++	c	c	c	c	c					c	c						t							
t	3373868	g	3516436	s	g	clone D	++	g	g	g	g	g					t	g						t							
g	3373871	a	3516439	s	a	clone D	++	a	a	a	a	a					g	a						g							
c	3373898	t	3516466	s	c	CFT073	++	c	c	c	c	c					c	c						c							
c	3373904	t	3516472	s	t	clone D	++	t	t	t	t	t					t	t						c							
a	3373907	g	3516475	s	g	clone D	++	g	g	g	g	g					g	g						a							
g	3373916	c	3516484	s	g	CFT073	++	g	g	g	g	g					g	c						g							
c	3373958	t	3516526	s	t	clone D	++	t	t	c	c	c					t	t						c							
g	3374021	a	3516589	s	g	CFT073	++	g	g	a	a	a					a	a						g							
g	3374030	a	3516598	s	a	clone D	++	a	a	a	a	a					-	a						g							
a	3374049	c	3516617	s	c	clone D	++	c	c	c	c	c					c	c						a							
g	3374054	a	3516622	s	g	CFT073	++	g	g	g	g	g					a	g						g							
c	3374075	t	3516643	s	c	CFT073	++	c	c	c	c	c					t	c						c							
a	3374081	g	3516649	s	a	CFT073	++	a	a	a	a	a					a	a						a							
g	3374088	a	3516656	ns	g	CFT073	++	g	g	g	g	g					g	g						g							
t	3374149	g	3516717	ns	t	CFT073	++	t	t	t	t	t					t	t						t							
g	3374239	a	3516807	s	g	CFT073	++	g	g	g	g	g					g	g						g							
a	3374272	g	3516840	s	g	clone D	++	g	g	g	g	g					g	g						a							
g	3374276	a	3516844	ns	g	CFT073	++	g	g	g	g	g					a	g						g							
a	3374278	c	3516846	ns	a	CFT073	++	a	a	a	a	a					c	a						a							
t	3374281	c	3516849	s	t	CFT073	++	t	t	t	t	t					c	t						t							

Clone D base	Clone D site	CFT 073 base	CFT 073 site	mutation type[a]	Inferred ancestral base[b]	lineage inferred to mutate	Support level[c]	ED1a[d]	536	S88	APEC O1	UTI89	E2348/69	ATCC8739	K-12	HS	SMS3-5	IAI39	E24377A	SE11	55989	IAI1	Sakai	UMN026	B4 Sb227	F5 8401	SS Ss046	D1 Sd197	B18 BS512	F2a 2457T	F2a 301
t	3374444	g	3517012	ns	g	clone D	++	g	g	g	g	g					g	g						t							
a	3374473	g	3517041	s	a	CFT073	++	a	a	g	g	g					a	a						a							
a	3374476	g	3517044	s	a	CFT073	++	a	a	g	g	g					a	a						a							
a	3374494	g	3517062	s	g	clone D	++	g	g	g	g	g					g	g						g							
a	3374510	g	3517078	ns	a	CFT073	++	a	a	a	a	a					a	a						a							
a	3374512	g	3517080	ns	a	CFT073	++	a	a	a	a	a					a	a						a							
t	3374515	a	3517083	s	?	?	+/-	-	-	-	-	-					-	-						-							
c	3374516	t	3517084	s	?	?	+/-	-	-	-	-	-					-	-						-							
a	3374519	g	3517087	ns	?	?	+/-	t	t	t	t	t					t	t						t							
t	3374520	c	3517088	ns	c	clone D	++	c	c	c	c	c					c	c						c							
a	3374521	t	3517089	ns	t	clone D	++	t	t	t	t	t					t	t						t							
a	3374522	g	3517090	ns	g	clone D	++	g	g	g	g	g					g	g						g							
g	3374523	a	3517091	ns	a	clone D	++	a	a	a	a	a					a	a						a							
c	3374524	t	3517092	ns	t	clone D	++	t	t	t	t	t					t	t						t							
a	3374584	g	3517152	s	a	CFT073	++	a	a	g	g	g					a	a						a							
g	3374590	a	3517158	s	g	CFT073	++	g	g	g	g	g					g	g						g							
t	3374615	g	3517183	ns	g	clone D	++	g	t	a	a	a					t	g						t							
t	3374653	c	3517221	s	t	CFT073	++	t	t	t	t	t					t	t						t							
a	3374659	c	3517227	s	a	CFT073	++	a	a	a	a	a					a	a						a							
c	3374710	t	3517278	s	c	CFT073	++	c	t	c	c	c					c	c						c							
c	3374718	a	3517286	ns	c	CFT073	++	c	c	c	c	c					c	c						c							
c	3374794	t	3517362	s	t	clone D	++	t	t	c	c	c					c	t						c							
c	3374796	a	3517364	ns	a	clone D	++	a	a	a	a	a					a	a						c							
c	3374803	t	3517371	s	t	clone D	++	t	c	c	c	c					c	t						c							
a	3374824	g	3517392	s	g	clone D	++	g	g	a	a	a					a	g						a							
g	3374854	a	3517422	s	a	clone D	++	a	g	g	g	g					g	a						g							
c	3374887	a	3517455	s	c	CFT073	++	c	a	c	c	c					c	c						c							
g	3374893	a	3517461	s	a	clone D	++	a	a	a	a	a					g	g						g							

Clone D base	Clone D site	CFT 073 base	CFT 073 site	mutation type[a]	Inferred ancestral base[b]	lineage inferred to mutate	Support level[c]	ED1a[d]	536	S88	APEC O1	UTI89	E2348/69	ATCC8739	K-12	HS	SMS3-5	IAI39	E24377A	SE11	55989	IAI1	Sakai	UMN026	B4 Sb227	F5 8401	SS Ss046	D1 Sd197	B18 BS512	F2a 2457T	F2a 301
g	3374926	a	3517494	s	a	clone D	++	a	a	a	a	a					a	a						g							
g	3374929	a	3517497	s	a	clone D	++	a	a	a	a	a					a	a						g							
c	3374935	t	3517503	s	t	clone D	++	t	t	t	t	t					t	t						c							
g	3375031	a	3517599	s	a	clone D	++	a	a	g	g	g					g	g						g							
t	3375076	c	3517644	s	t	CFT073	++	t	c	t	t	t					c	t						t							
a	3375081	t	3517649	ns	a	CFT073	++	a	a	a	a	a					a	a						a							
a	3375082	c	3517650	ns	a	CFT073	++	a	a	a	a	a					a	a						a							
a	3375088	g	3517656	s	a	CFT073	++	a	a	a	a	a					a	a						a							
a	3375090	c	3517658	ns	a	CFT073	++	a	c	a	a	a					a	a						a							
a	3375091	c	3517659	ns	a	CFT073	++	a	a	a	a	a					a	a						a							
t	3375093	c	3517661	ns	t	CFT073	++	t	t	t	t	t					t	t						t							
a	3375098	g	3517666	ns	g	clone D	++	g	g	g	g	g					a	g						a							
a	3375103	g	3517671	s	a	CFT073	++	a	g	a	a	a					a	a						a							
g	3375112	a	3517680	s	g	CFT073	++	g	a	g	g	g					g	g						g							
a	3375133	g	3517701	s	a	CFT073	++	a	g	a	a	a					a	a						a							
t	3375136	c	3517704	s	t	CFT073	++	t	c	t	t	t					t	t						t							
c	3375137	a	3517705	ns	c	CFT073	++	c	a	c	c	c					c	c						c							
g	3375138	a	3517706	ns	g	CFT073	++	g	a	g	g	g					g	g						g							
a	3375139	c	3517707	ns	a	CFT073	++	a	c	a	a	a					a	a						a							
t	3375144	a	3517712	ns	t	CFT073	++	t	a	t	t	t					t	t						t							
t	3375160	c	3517728	s	t	CFT073	++	t	c	t	t	t					t	t						t							
a	3375196	g	3517764	s	a	CFT073	++	a	a	g	g	g					a	a						a							
g	3375199	a	3517767	s	g	CFT073	++	g	g	g	g	g					g	g						g							
c	3375218	t	3517786	ns	c	CFT073	++	c	c	c	c	c					c	c						c							
a	3375219	g	3517787	ns	a	CFT073	++	a	a	g	g	g					a	a						a							
a	3375220	t	3517788	ns	a	CFT073	++	a	a	a	a	a					a	a						a							
g	3375223	c	3517791	s	g	CFT073	++	g	g	g	g	g					g	g						g							
c	3375233	a	3517801	s	c	CFT073	++	c	c	c	c	c					c	c						c							

Clone D base	Clone D site	CFT 073 base	CFT 073 site	mutation type[a]	Inferred ancestral base[b]	lineage inferred to mutate	Support level[c]	ED1a[d]	536	S88	APEC O1	UTI89	E2348/69	ATCC8739	K-12	HS	SMS3-5	IAI39	E24377A	SE11	55989	IAI1	Sakai	UMN026	B4 Sb227	F5 8401	SS Ss046	D1 Sd197	B18 BS512	F2a 2457T	F2a 301
g	3375235	a	3517803	s	g	CFT073	++	g	g	g	g	g					g	g						g							
t	3375243	a	3517811	ns	t	CFT073	++	t	t	t	t	t					t	t						t							
t	3375250	c	3517818	s	t	CFT073	++	t	t	t	t	t					t	t						t							
c	3375253	t	3517821	s	c	CFT073	++	c	c	c	c	c					c	c						c							
g	3375267	c	3517835	ns	g	CFT073	++	g	g	g	g	g					g	g						g							
c	3375268	g	3517836	ns	c	CFT073	++	c	c	c	c	c					c	c						c							
c	3375271	t	3517839	s	c	CFT073	++	c	c	c	c	c					c	c						c							
g	3375309	t	3517877	ns	g	CFT073	++	g	g	g	g	g					g	g						g							
g	3375322	a	3517890	s	g	CFT073	++	g	g	g	g	g					g	g						g							
g	3375324	a	3517892	ns	g	CFT073	++	g	g	g	g	g					g	g						g							
a	3375364	g	3517932	s	g	clone D	++	g	g	g	g	g					g	g						a							
c	3375412	a	3517980	s	?	?	+/-	c	c	a	a	a					g	g						c							
g	3375466	c	3518034	s	g	CFT073	++	g	g	g	g	g					g	g						g							
a	3375490	g	3518058	s	g	clone D	++	g	a	g	g	g					g	g						a							
c	3375708	a	3518276	ns	a	clone D	++	a		a	a	a					a	a						a							
t	3375841	c	3518409	s	c	clone D	++	c		t	t	t					t	t						t							
c	3375970	t	3518538	s	t	clone D	++	t		t	t	t					c	c						t							
t	3375979	c	3518547	s	c	clone D	++	c		t	t	t					t	t						c							
a	3375987	g	3518555	ns	g	clone D	++	g		g	g	g					g	g						g							
a	3375988	c	3518556	ns	c	clone D	++	c		a	a	a					a	a						c							
a	3375990	g	3518558	ns	g	clone D	++	g		a	a	a					a	a						g							
t	3376006	c	3518574	s	c	clone D	++	c		t	t	t					t	t						c							
t	3376033	c	3518601	s	c	clone D	++	c		t	t	t					t	t						c							
c	3376039	a	3518607	s	a	clone D	++	a		c	c	c					c	c						a							
c	3376045	t	3518613	s	t	clone D	++	t		c	c	c					c	c						t							
t	3376056	g	3518624	ns	g	clone D	++	g		g	g	g					g	g						g							
a	3376063	g	3518631	s	g	clone D	++	g		a	a	a					a	a						g							
a	3376078	g	3518646	s	g	clone D	++	g		a	a	a					a	a						g							

Clone D base	Clone D site	CFT 073 base	CFT 073 site	mutation type[a]	Inferred ancestral base[b]	lineage inferred to mutate	Support level[c]	ED1a[d]	536	S88	APEC O1	UTI89	E2348/69	ATCC8739	K-12	HS	SMS3-5	IAI39	E24377A	SE11	55989	IAI1	Sakai	UMN026	B4 Sb227	F5 8401	SS Ss046	D1 Sd197	B18 BS512	F2a 2457T	F2a 301
g	3376081	a	3518649	s	a	clone D	++	a		g	g	g					a	g						a							
g	3376084	t	3518652	s	t	clone D	++	t		g	g	g					t	g						t							
g	3376096	a	3518664	s	a	clone D	++	a		g	g	g					a	g						a							
c	3376111	t	3518679	s	t	clone D	++	t		c	c	c					c	c						t							
a	3376144	g	3518712	s	g	clone D	++	g		g	g	g					-	g						g							
a	3376147	g	3518715	s	g	clone D	++	g		a	a	a					g	a						g							
c	3376211	t	3518779	nc	t	clone D	++	t		t	t	t					t	t						t							
a	3376228	g	3518796	nc	g	clone D	++	g		a	a	a					a	a						a							
t	3376244	c	3518812	s	c	clone D	++	c		t	t	t					t	t						t							
t	3376247	c	3518815	s	c	clone D	++	c		t	t	t					t	t						t							
g	3376257	a	3518825	ns	a	clone D	++	a		g	g	g					g	g						g							
c	3376268	g	3518836	s	g	clone D	++	g		c	c	c					c	c						c							
g	3376295	a	3518863	s	a	clone D	++	a		g	g	g					g	g						g							
a	3376298	g	3518866	s	g	clone D	++	g		g	g	g					a	g						g							
c	3376307	t	3518875	s	t	clone D	++	t		t	t	t					t	t						t							
g	3376315	a	3518883	ns	a	clone D	++	a		a	a	a					a	a						a							
t	3376319	c	3518887	s	c	clone D	++	c		c	c	c					c	c						t							
t	3376322	c	3518890	s	t	CFT073	++	t		c	c	c					c	c						c							
g	3376328	a	3518896	s	a	clone D	++	a		a	a	a					a	a						a							
c	3376332	t	3518900	s	t	clone D	++	t		t	t	t					t	t						t							
t	3376346	c	3518914	s	c	clone D	++	c		c	c	c					c	a						a							
a	3376367	c	3518935	s	a	CFT073	++	a		a	a	a					c	a						a							
t	3376376	c	3518944	s	t	CFT073	++	t		t	t	t					t	t						t							
a	3376412	g	3518980	s	g	clone D	++	g		g	g	g					g	g						g							
g	3376424	a	3518992	s	a	clone D	++	a		a	a	a					a	a						a							
a	3376427	g	3518995	s	g	clone D	++	g		g	g	g					g	g						g							
a	3376456	c	3519024	ns	a	CFT073	++	a		a	a	a					a	a						a							
c	3376481	t	3519049	s	t	clone D	++	t		t	t	t					t	t						t							

Clone D base	Clone D site	CFT 073 base	CFT 073 site	mutation type[a]	Inferred ancestral base[b]	lineage inferred to mutate	Support level[c]	ED1a[d]	536	S88	APEC O1	UTI89	E2348/69	ATCC8739	K-12	HS	SMS3-5	IAI39	E24377A	SE11	55989	IAI1	Sakai	UMN026	B4 Sb227	F5 8401	SS Ss046	D1 Sd197	B18 BS512	F2a 2457T	F2a 301
a	3376484	c	3519052	s	c	clone D	++	c		c	c	c					c	c						c							
c	3376499	t	3519067	s	t	clone D	++	t		t	t	t					t	t						t							
t	3376517	c	3519085	s	t	CFT073	++	t		c	c	c					c	c						c							
c	3376559	t	3519127	s	t	clone D	++	t		t	t	t					t	t						t							
a	3376571	g	3519139	s	g	clone D	++	g		g	g	g					g	a						g							
t	3376598	a	3519166	s	t	CFT073	++	t		t	t	t					t	t						t							
a	3376631	c	3519199	s	a	CFT073	++	a		a	a	a					a	a						a							
a	3376667	g	3519235	s	a	CFT073	++	a		a	a	a					a	a						a							
a	3376680	g	3519248	ns	g	clone D	++	g		a	a	a					a	g						g							
t	3376721	c	3519289	s	c	clone D	++	c		t	t	t					t	t						t							
a	3376724	t	3519292	s	t	clone D	++	t		a	a	a					a	a						a							
t	3376733	g	3519301	s	g	clone D	++	g		g	g	g					g	g						g							
t	3376775	c	3519343	s	c	clone D	++	c		c	c	c					c	c						c							
a	3376838	g	3519406	s	g	clone D	++	g		a	a	a					g	a						a							
t	3376874	c	3519442	s	t	CFT073	++	t		t	t	t					t	t						t							
a	3376883	g	3519451	s	g	clone D	++	g		a	a	a					g	g						a							
c	3376898	t	3519466	s	t	clone D	++	t		c	c	c					t	c						t							
a	3376901	g	3519469	s	g	clone D	++	g		g	g	g					g	g						g							
c	3376907	t	3519475	s	t	clone D	++	t		c	c	c					t	c						c							
t	3376925	c	3519493	s	c	clone D	++	c		c	c	c					c	c						c							
c	3376967	t	3519535	s	c	CFT073	++	c		c	c	c					c	c						c							
g	3376969	a	3519537	ns	a	clone D	++	a		a	a	a					a	a						a							
g	3377018	a	3519586	s	g	CFT073	++	g		a	a	a					g	a						a							
a	3377102	g	3519670	s	g	clone D	++	g		g	g	g					g	g						g							
g	3377105	a	3519673	s	a	clone D	++	a		g	g	g					g	g						g							
g	3377128	a	3519696	ns	a	clone D	++	a		g	g	g					g	g						g							
a	3377136	g	3519704	ns	g	clone D	++	g		g	g	g					g	g						g							
c	3377139	a	3519707	ns	a	clone D	++	a		a	a	a					a	a						a							

Clone D base	Clone D site	CFT 073 base	CFT 073 site	mutation type[a]	Inferred ancestral base[b]	lineage inferred to mutate	Support level[c]	ED1a[d]	536	S88	APEC O1	UTI89	E2348/69	ATCC8739	K-12	HS	SMS3-5	IAI39	E24377A	SE11	55989	IAI1	Sakai	UMN026	B4 Sb227	F5 8401	SS Ss046	D1 Sd197	B18 BS512	F2a 2457T	F2a 301
g	3377192	a	3519760	s	a	clone D	++	a		g	g	g					g	g						g							
g	3377279	a	3519847	s	a	clone D	++	a		g	g	g					g	g						a							
t	3377321	c	3519889	s	c	clone D	++	c		c	c	c					c	c						c							
a	3377333	g	3519901	s	g	clone D	++	g		g	g	g					g	g						a							
t	3377336	c	3519904	s	t	CFT073	++	t		t	t	t					t	t						t							
t	3377360	g	3519928	s	g	clone D	++	g		g	g	g					g	g						c							
c	3377392	g	3519960	ns	c	CFT073	++	c		g	g	g					g	g						g							
t	3377395	g	3519963	ns	t	CFT073	++	t		t	t	t					t	t						t							
c	3385786	t	3527989	s	t	clone D	++	t		t	t	t					t	t													
c	3385798	t	3528001	s	t	clone D	++	t		t	t	t					t	t													
c	3385831	t	3528034	s	t	clone D	++	t		t	t	t					t	t													
c	3385834	t	3528037	s	t	clone D	++	t		c	c	c					c	c													
a	3385858	c	3528061	s	a	CFT073	+	t		a	a	a					a	a													
t	3385861	c	3528064	s	?	?	+/-	a		a	a	a					a	a													
g	3385939	t	3528142	s	t	clone D	++	t		a	a	a					a	a													
g	3385951	a	3528154	s	a	clone D	++	a		a	a	a					a	a													
a	3385963	g	3528166	s	g	clone D	++	g		g	g	g					g	g													
a	3385972	g	3528175	s	a	CFT073	++	a		t	t	t					t	t													
g	3385975	a	3528178	s	g	CFT073	++	g		g	g	g					g	g													
g	3385993	a	3528196	s	g	CFT073	++	g		a	a	a					a	a													
c	3386017	t	3528220	s	c	CFT073	++	c		c	c	c					c	c													
g	3386083	a	3528286	s	a	clone D	++	a		a	a	a					a	a													
g	3386092	t	3528295	s	t	clone D	++	t		a	a	a					a	a													
g	3386095	a	3528298	s	g	CFT073	++	g		c	c	c					c	c													
c	3386116	t	3528319	s	t	clone D	++	t		t	t	t					t	t													
g	3386125	a	3528328	s	a	clone D	++	a		t	t	t					t	t													
g	3386140	a	3528343	s	a	clone D	++	a		a	a	a					a	a													
a	3386143	g	3528346	s	g	clone D	++	g		a	a	a					a	a													

Clone D base	Clone D site	CFT 073 base	CFT 073 site	mutation type[a]	Inferred ancestral base[b]	lineage inferred to mutate	Support level[c]	ED1a[d]	536	S88	APEC O1	UTI89	E2348/69	ATCC8739	K-12	HS	SMS3-5	IAI39	E24377A	SE11	55989	IAI1	Sakai	UMN026	B4 Sb227	F5 8401	SS Ss046	D1 Sd197	B18 BS512	F2a 2457T	F2a 301
g	3386218	a	3528421	s	a	clone D	++	a		g	g	g					g	g													
t	3386241	g	3528444	ns	t	CFT073	++	t		t	t	t					t	t													
g	3386263	a	3528466	s	a	clone D	++	a		a	a	a					a	a													
c	3386269	a	3528472	s	a	clone D	++	a		c	c	c					c	c													
a	3386281	g	3528484	s	g	clone D	++	g		g	g	g					g	g													
g	3386346	a	3528549	s	g	CFT073	++	g		g	g	g					g	g						g							
g	3386361	a	3528564	s	g	CFT073	++	g		g	g	g					g	g						a							
g	3386370	a	3528573	s	g	CFT073	++	g		g	g	g					g	g						a							
g	3386373	t	3528576	ns	g	CFT073	++	g		g	g	g					g	g						t							
c	3386375	t	3528578	ns	c	CFT073	++	c		c	c	c					c	c						t							
g	3386378	a	3528581	s	g	CFT073	++	g		g	g	g					g	g						a							
a	3386379	t	3528582	s	a	CFT073	++	a		a	a	a					a	a						t							
a	3386391	c	3528594	s	a	CFT073	++	a		a	a	a					a	a						c							
g	3386409	a	3528612	s	g	CFT073	++	g		g	g	g					g	g						g							
g	3386418	a	3528621	s	g	CFT073	++	g		g	g	g					g	g						g							
t	3386436	g	3528639	s	t	CFT073	++	t		t	t	t					t	t						t							
g	3386442	a	3528645	s	g	CFT073	++	g		g	g	g					g	g						g							
t	3386454	c	3528657	s	t	CFT073	++	t		c	c	c					c	c						t							
a	3386468	g	3528671	s	a	CFT073	++	a		a	a	a					a	a						a							
c	3386502	t	3528705	s	t	clone D	++	t		t	t	t					t	t						c							
c	3386508	t	3528711	s	t	clone D	++	t		c	c	c					c	c						c							
a	3386511	g	3528714	s	g	clone D	++	g		g	g	g					g	g						g							
t	3386514	c	3528717	s	c	clone D	++	c		t	t	t					t	t						t							
c	3386544	a	3528747	s	c	CFT073	++	c		c	c	c					c	c						c							
c	3386547	g	3528750	s	c	CFT073	++	c		c	c	c					c	c						c							
c	3386556	a	3528759	s	c	CFT073	++	c		c	c	c					c	c						c							
g	3386568	a	3528771	s	g	CFT073	++	g		g	g	g					g	g						g							
a	3386583	g	3528786	s	a	CFT073	++	a		a	a	a					a	a						a							

Clone D base	Clone D site	CFT 073 base	CFT 073 site	mutation type[a]	Inferred ancestral base[b]	lineage inferred to mutate	Support level[c]	ED1a[d]	536	S88	APEC O1	UTI89	E2348/69	ATCC8739	K-12	HS	SMS3-5	IAI39	E24377A	SE11	55989	IAI1	Sakai	UMN026	B4 Sb227	F5 8401	SS Ss046	D1 Sd197	B18 BS512	F2a 2457T	F2a 301
t	3386621	c	3528824	ns	c	clone D	++	c		c	c	c					c	c						c							
c	3386624	t	3528827	ns	c	CFT073	++	c		c	c	c					c	c						c							
a	3386625	g	3528828	s	a	CFT073	++	a		a	a	a					a	a						a							
t	3386640	c	3528843	s	t	CFT073	++	t		t	t	t					t	t						t							
a	3386642	g	3528845	s	a	CFT073	++	a		a	a	a					a	a						a							
a	3386645	g	3528848	s	a	CFT073	++	a		a	a	a					a	a						a							
g	3386655	a	3528858	ns	g	CFT073	++	g		g	g	g					g	g						a							
a	3386656	g	3528859	ns	a	CFT073	++	a		a	a	a					a	a						a							
g	3386657	c	3528860	ns	g	CFT073	++	g		g	g	g					g	g						g							
t	3386661	a	3528864	s	a	clone D	++	a		a	a	a					a	a						a							
g	3386673	a	3528876	s	a	clone D	++	a		g	g	g					g	g						g							
a	3386694	g	3528897	s	g	clone D	++	g		g	g	g					g	g						g							
a	3386705	t	3528908	ns	a	CFT073	++	a		a	a	a					a	a						a							
g	3386709	a	3528912	s	g	CFT073	++	g		g	g	g					g	g						g							
a	3386712	g	3528915	s	a	CFT073	++	a		a	a	a					a	a						a							
g	3386718	a	3528921	s	a	clone D	++	a		g	g	g					g	g						a							
a	3386723	g	3528926	s	g	clone D	++	g		a	a	a					a	a						a							
t	3386724	a	3528927	s	a	clone D	++	a		a	a	a					a	a						t							
c	3386735	a	3528938	ns	c	CFT073	++	c		c	c	c					c	c						t							
t	3386760	g	3528963	s	g	clone D	++	g		t	t	t					t	t						t							
t	3386766	c	3528969	s	c	clone D	++	c		t	t	t					t	t						c							
t	3386796	g	3528999	s	t	CFT073	++	t	c	t	t	t					t	t						t							
a	3386810	g	3529013	s	g	clone D	++	g	a	g	g	g					g	g						g							
g	3386832	a	3529035	ns	a	clone D	++	a	c	a	a	a					a	a						a							
c	3386834	t	3529037	ns	c	CFT073	++	c	c	c	c	c					c	c						c							
a	3386844	t	3529047	ns	a	CFT073	++	a	c	t	t	t					t	t						a							
t	3386904	c	3529107	s	t	CFT073	++	t	g	c	c	c					c	c						t							
a	3386993	g	3529196	s	g	clone D	++	g	a	a	a	a					a	a						a							

Clone D base	Clone D site	CFT 073 base	CFT 073 site	mutation type[a]	Inferred ancestral base[b]	lineage inferred to mutate	Support level[c]	ED1a[d]	536	S88	APEC O1	UTI89	E2348/69	ATCC8739	K-12	HS	SMS3-5	IAI39	E24377A	SE11	55989	IAI1	Sakai	UMN026	B4 Sb227	F5 8401	SS Ss046	D1 Sd197	B18 BS512	F2a 2457T	F2a 301
a	3387093	g	3529296	nc	a	CFT073	++	a	a	a	a	a					a	a						a							
a	3387107	t	3529310	nc	a	CFT073	++	a	a	a	a	a					a	a						a							
c	3387120	t	3529323	nc	c	CFT073	++	c	c	c	c	c					c	c						c							
t	3387131	c	3529334	nc	t	CFT073	++	t	t	t	t	t					t	t						t							
g	3387165	a	3529368	nc	g	CFT073	++	g	g	a	a	a					g	a						g							
a	3387169	g	3529372	nc	a	CFT073	++	a	a	g	g	g					a	g						a							
c	3387189	t	3529392	nc	t	clone D	++	t	t	t	t	t					t	t						t							
c	3387191	g	3529394	nc	c	CFT073	++	c	c	c	c	c					c	c						c							
t	3387217	c	3529420	nc	c	clone D	++	c	c	c	c	c					c	c						c							
a	3387225	c	3529427	nc	a	CFT073	++	a	a	a	a	a					a	a						a							
g	3387309	t	3529511	nc	t	clone D	++	t	t	t	t	t					t	t						t							
t	3387347	c	3529549	nc	t	CFT073	++	t	t	t	t	t					t	t						t							
c	3387354	t	3529556	nc	t	clone D	++	t	t	c	c	c					t	c						c							
a	3387355	t	3529557	nc	a	CFT073	++	a	a	a	a	a					a	a						a							
t	3387358	g	3529560	nc	g	clone D	++	g	g	g	g	g					g	g						g							
t	3387383	c	3529701	nc	t	CFT073	++	t	t	t	t	t					g	t						c							
t	3387385	c	3529703	nc	c	clone D	++	c	t	t	t	t					-	t						c							
c	3387416	a	3529734	nc	a	clone D	++	a	a	c	c	c					a	c						c							
t	3387419	c	3529737	nc	t	CFT073	++	t	t	t	t	t					c	t						t							
g	3387423	a	3529741	nc	a	clone D	++	a	t	g	g	g					a	g						g							
a	3387436	g	3529754	nc	a	CFT073	++	a	a	a	a	a					g	a						a							
c	3387437	t	3529755	nc	c	CFT073	++	c	c	c	c	c					t	c						c							
t	3387440	c	3529758	nc	c	clone D	++	c	t	c	c	c					c	c						t							
c	3387446	t	3529764	nc	c	CFT073	++	c	c	c	c	c					c	c						c							
c	3387451	t	3529769	nc	t	clone D	++	t	t	t	t	t					t	t						t							
g	3387452	a	3529770	nc	g	CFT073	++	g	g	g	g	g					a	g						g							
t	3387454	c	3529772	nc	c	clone D	++	c	t	t	t	t					c	t						t							
c	3387470	t	3529788	nc	c	CFT073	++	c	c	c	c	c					t	c						t							

Clone D base	Clone D site	CFT 073 base	CFT 073 site	mutation type[a]	Inferred ancestral base[b]	lineage inferred to mutate	Support level[c]	ED1a[d]	536	S88	APEC O1	UTI89	E2348/69	ATCC8739	K-12	HS	SMS3-5	IAI39	E24377A	SE11	55989	IAI1	Sakai	UMN026	B4 Sb227	F5 8401	SS Ss046	D1 Sd197	B18 BS512	F2a 2457T	F2a 301
c	3387495	t	3529813	nc	t	clone D	++	t	c	c	c	c					t	c						t							
c	3387497	g	3529815	nc	g	clone D	++	g	c	c	c	c					g	c						g							
a	3387498	c	3529816	nc	c	clone D	+	t	t	c	c	c					c	c						c							
g	3387530	a	3529848	nc	a	clone D	++	a	a	g	g	g					a	g						a							
c	3387556	a	3529874	nc	c	CFT073	++	c	c	c	c	c					c	c						c							
c	3387569	t	3529887	nc	t	clone D	++	t	t	c	c	c					t	c						t							
t	3387572	c	3529890	nc	c	clone D	++	c	c	t	t	t					c	t						c							
t	3387585	c	3529903	nc	c	clone D	++	c	c	t	t	t					c	c						c							
t	3387598	c	3529916	nc	c	clone D	++	c	c	t	t	t					c	t						c							
g	3387611	c	3529929	nc	g	CFT073	++	g	g	g	g	g					g	g						c							
t	3387612	a	3529930	nc	c	clone D	++	c	c	t	t	t					c	t						a							
g	3387614	c	3529932	nc	c	clone D	++	c	c	g	g	g					c	g						c							
c	3387617	t	3529935	nc	t	clone D	++	t	t	t	t	t					t	t						t							
c	3387625	t	3529943	nc	t	clone D	++	t	t	c	c	c					t	c						t							
a	3387629	c	3529947	nc	t	clone D	++	t	t	a	a	a					t	a						t							
a	3387630	g	3529948	nc	g	clone D	++	g	g	a	a	a					a	a						g							
g	3387631	a	3529949	nc	g	CFT073	++	g	a	g	g	g					g	g						a							
c	3387643	t	3529961	nc	c	CFT073	++	c	c	c	c	c					c	c						c							
a	3387678	g	3529997	nc	a	CFT073	++	a	a	a	a	a					g	a						a							
t	3387824	c	3530143	nc	t	CFT073	++	t	t	t	t	t					t	t						t							
a	3387830	g	3530149	nc	g	clone D	++	g	g	g	g	g					g	g						g							
c	3387832	t	3530151	nc	c	CFT073	++	c	c	c	c	c					c	c						c							
a	3387833	t	3530152	nc	t	clone D	++	t	t	t	t	t					t	t						t							
a	3387854	t	3530173	nc	a	CFT073	++	a	a	a	a	a					a	a						a							
a	3387876	g	3530195	nc	a	CFT073	++	a	a	a	a	a					a	a						a							
t	3387945	a	3530264	nc	t	CFT073	++	t	c	t	t	t					t	t						t							
t	3387957	g	3530276	nc	t	CFT073	++	t	g	t	t	t					t	t						t							
c	3388019	t	3530338	nc	t	clone D	++	t	t	t	t	t					t	t						t							

Clone D base	Clone D site	CFT 073 base	CFT 073 site	mutation type[a]	Inferred ancestral base[b]	lineage inferred to mutate	Support level[c]	ED1a[d]	536	S88	APEC O1	UTI89	E2348/69	ATCC8739	K-12	HS	SMS3-5	IAI39	E24377A	SE11	55989	IAI1	Sakai	UMN026	B4 Sb227	F5 8401	SS Ss046	D1 Sd197	B18 BS512	F2a 2457T	F2a 301
a	3388047	t	3530366	nc	t	clone D	++	t	t	t	t	t					t	t						t							
g	3388061	a	3530380	nc	a	clone D	++	a	g	a	a	a					a	a						a							
t	3388079	g	3530398	nc	g	clone D	++	g	g	g	g	g					g	g						g							
t	3388156	c	3530475	s	c	clone D	++	c	t	t	t	t	c		t	t	t	t	t	t	t	t		t	t			t	t		
a	3388172	t	3530491	ns	t	clone D	++	t	t	t	t	t	t		t	t	t	t	t	t	t	t		t	t			t	t		
t	3388180	c	3530499	s	c	clone D	++	c	c	c	c	c	c		c	c	c	c	c	c	c	c		c	c			c	c		
t	3388189	a	3530508	s	a	clone D	++	a	a	a	a	a	a		a	a	a	a	a	a	a	a		a	a			a	a		
g	3388199	c	3530518	ns	g	CFT073	++	g	g	g	g	g	c		g	g	c	g	g	g	g	g		c	g			g	g		
a	3388201	c	3530520	s	a	CFT073	++	a	a	a	a	a	t		a	a	t	a	a	a	a	a		t	a			a	a		
t	3388221	c	3530540	ns	t	CFT073	++	t	t	t	t	t	t		t	t	t	t	t	t	t	t		t	t			t	t		
a	3388234	g	3530553	s	a	CFT073	++	a	a	a	a	a	a		a	a	a	a	a	a	a	a		a	a			a	a		
t	3388237	c	3530556	s	t	CFT073	++	t	t	t	t	t	t		t	c	t	t	c	c	c	c		t	c			t	c		
t	3388249	c	3530568	s	t	CFT073	++	t	t	t	t	t	c		t	c	t	c	c	c	c	c		c	c			t	c		
g	3388252	c	3530571	ns	g	CFT073	++	g	g	g	g	g	g		g	g	c	g	g	g	g	g		g	g			g	g		
t	3388261	g	3530580	ns	g	clone D	++	g	g	t	t	t	g		t	g	g	g	g	g	g	g		g	g			t	g		
a	3388267	c	3530586	s	c	clone D	++	c	c	a	a	a	c		c	c	c	c	c	c	c	c		c	c			c	c		
t	3388324	a	3530643	s	t	CFT073	++	t	t	t	t	t	a		t	t	a	t	t	t	t	t		t	t			t	t		
c	3388330	t	3530649	s	c	CFT073	++	c	c	c	c	c	t		c	c	t	c	c	c	c	c		c	c			c	c		
c	3388342	a	3530661	s	c	CFT073	+	t	a	c	c	c	c		c	c	c	t	c	c	c	c		a	c			c	c		
a	3388363	g	3530682	s	g	clone D	++	g	g	a	a	a	a		a	a	a	g	a	a	a	a		a	a			a	a		
t	3388388	g	3530707	ns	g	clone D	++	g	g	g	g	g	g		g	g	g	g	g	g	g	g		g	g			g	g		
c	3388425	t	3530744	ns	c	CFT073	++	c	c	c	c	c	c		c	c	c	c	c	c	c	c		t	c			c	c		
a	3388432	g	3530751	ns	g	clone D	++	g	g	g	g	g	g		g	g	g	g	g	g	g	g		a	g			g	g		
t	3388434	c	3530753	ns	c	clone D	++	c	c	c	c	c	c		c	c	c	c	c	c	c	c		t	c			c	c		
c	3388498	t	3530817	s	t	clone D	++	t	t	t	t	t	t		t	t	t	t	t	t	t	t		t	t			t	t		
a	3388532	c	3530851	ns	c	clone D	++	c	c	c	c	c	c		c	c	c	c	c	c	c	c		c	c			c	c		
a	3388539	t	3530858	ns	t	clone D	++	t	t	t	t	t	t		t	t	t	t	t	t	t	t		t	t			t	t		
g	3388552	t	3530871	ns	t	clone D	++	t	t	c	c	c	t		c	c	c	t	c	c	c	c		c	c			c	c		

Clone D base	Clone D site	CFT 073 base	CFT 073 site	mutation type[a]	Inferred ancestral base[b]	lineage inferred to mutate	Support level[c]	ED1a[d]	536	S88	APEC O1	UTI89	E2348/69	ATCC8739	K-12	HS	SMS3-5	IAI39	E24377A	SE11	55989	IAI1	Sakai	UMN026	B4 Sb227	F5 8401	SS Ss046	D1 Sd197	B18 BS512	F2a 2457T	F2a 301
g	3388553	a	3530872	ns	a	clone D	++	a	a	a	a	a	a		a	a	g	a	a	a	a	a		a	a			a	a		
g	3388558	t	3530877	s	t	clone D	++	t	t	t	t	t	t		t	t	t	t	t	t	t	t		t	t			t	t		
c	3388585	a	3530904	s	a	clone D	++	a	a	a	a	a	a		a	a	a	a	a	a	a	a		c	a			c	a		
c	3388603	t	3530922	s	t	clone D	++	t	t	t	t	t	t		t	t	t	t	t	t	t	t		c	t			t	t		
c	3388608	t	3530927	ns	t	clone D	++	t	t	t	t	t	t		c	c	t	t	c	c	c	c		c	c			c	c		
a	3388787	g	3531106	s	g	clone D	++	g	g	g	g	g	g		g	g	g	g	g	g	g	g		g	g			g	g		
g	3388820	a	3531139	s	g	CFT073	++	g	g	a	a	a	g		g	g	g	g	g	g	g	g		g	g			g	g		
t	3388876	a	3531195	ns	a	clone D	++	a	a	a	a	a	t		a	a	t	t	a	a	a	a		t	a			a	a		
g	3388908	a	3531227	ns	g	CFT073	++	g	g	g	g	g	g		g	g	g	g	g	g	g	g		g	g			g	g		
g	3388930	a	3531249	ns	c	clone D	++	c	g	g	g	g	g		g	g	g	g	g	g	g	g		g	g			g	g		
c	3388975	t	3531294	ns	c	CFT073	++	c	c	c	c	c	c		c	c	c	c	c	c	c	c		c	c			c	c		
g	3389010	a	3531329	ns	g	CFT073	++	g	g	g	g	g	g		g	g	g	g	g	g	g	g		g	g			g	g		
g	3389100	a	3531419	ns	a	clone D	++	a	a	a	a	a	a		g	g	a	a	g	g	g	g		a	g			g	g		
g	3389174	a	3531493	s	a	clone D	++	a	a	a	a	a	a		g	g	a	a	g	g	g	g		a	g			g	g		
t	3389189	c	3531508	s	c	clone D	++	c	c	c	c	c	c		c	c	c	c	c	c	c	c		c	c			c	c		
a	3389192	t	3531511	s	t	clone D	++	t	t	t	t	t	t		t	t	t	t	t	t	t	t		t	t			t	t		
c	3389195	t	3531514	s	t	clone D	++	t	t	t	t	t	t		c	c	t	t	c	c	c	c		t	c			t	c		
c	3389246	a	3531565	s	a	clone D	++	a	a	a	a	a	a		a	a	a	a	a	a	a	a		a	a			a	a		
t	3389249	g	3531568	s	g	clone D	++	g	g	g	g	g	g		g	g	g	g	g	g	g	g		t	g			g	g		
t	3389267	c	3531585	s	c	clone D	++	c	c	c	c	c	c		t	t	c	c	t	t	t	t		t	t			t	t		
a	3389323	t	3531641	ns	t	clone D	++	t	g	t	t	t	g		g	g	g	t	g	g	g	g		g	g			g	g		
c	3389332	t	3531650	ns	t	clone D	++	t	c	t	t	t	c		c	t	c	t	t	c	c	c		t	c			c	c		
t	3389442	a	3531760	ns	a	clone D	++	a	a	a	a	a	a		a	a	t	a	a	a	a	a		t	a			a	a		
g	3389444	c	3531762	s	c	clone D	++	c	c	c	c	c	c		c	c	g	c	c	c	c	c		g	c			t	c		
t	3389468	c	3531786	s	c	clone D	++	c	c	c	c	c	c		c	c	t	t	c	c	c	c		t	t			t	t		
t	3389510	c	3531828	s	c	clone D	++	c	c	c	c	c	c		c	c	c	c	c	c	c	c		c	c			c	c		
a	3389522	g	3531840	s	g	clone D	++	g	g	g	g	g	g		g	g	g	g	g	g	g	g		g	g			g	g		
t	3389534	c	3531852	s	c	clone D	++	c	c	c	c	c	c		t	t	c	c	t	t	t	t		c	t			t	t		

Clone D base	Clone D site	CFT 073 base	CFT 073 site	mutation type[a]	Inferred ancestral base[b]	lineage inferred to mutate	Support level[c]	ED1a[d]	536	S88	APEC O1	UTI89	E2348/69	ATCC8739	K-12	HS	SMS3-5	IAI39	E24377A	SE11	55989	IAI1	Sakai	UMN026	B4 Sb227	F5 8401	SS Ss046	D1 Sd197	B18 BS512	F2a 2457T	F2a 301
t	3389546	a	3531864	nc	a	clone D	++	a	a	a	a	a	a		t	g	a	a	g	t	t	t		a	t			t	t		
g	3389585	a	3531903	nc	g	CFT073	++	g	g	g	g	g	g		g	g	g	g	g	g	g	g		g	g			g	g		
a	3389609	g	3531927	nc	g	clone D	++	g	g	g	g	g	g		g	g	g	g	g	g	g	g		g	g			g	g		
a	3389642	c	3531960	nc	c	clone D	++	c	c	c	c	c	c		a	a	c	c	a	a	g	a		c	g			a	g		
c	3389662	g	3531980	nc	g	clone D	++	g	g	g	g	g	g			c	g	g	c	c	c	c		g	c			g	c		
g	3389663	c	3531981	nc	c	clone D	++	c	c	c	c	c	c			g	c	c	g	g	g	g		c	g			g	g		
g	3389729	a	3532047	nc	a	clone D	++	a	a	a	a	a	a			a	t	t	a	a	a	a		a	g			a	g		
g	3389752	a	3532070	nc	g	CFT073	++	g	a	g	g	g	g			g	a	a	g	g	g	g		g	g			g	g		
g	3389756	a	3532074	nc	g	CFT073	++	g	a	g	g	g	g			g	g	g	g	g	g	g		g	g			g	g		
t	3389783	c	3532101	nc	c	clone D	++	c	c	c	c	c	c			c	c	c	c	c	c	c		c	c			c	c		
a	3389789	g	3532107	nc	g	clone D	++	g	g	g	g	g	g			a	g	g	a	a	a	a		g	a			g	a		
c	3389799	t	3532117	nc	t	clone D	++	t	c	t	t	t	t			c	c	c	c	c	c	c		c	c			c	c		
g	3389810	a	3532128	nc	a	clone D	++	a	a	a	a	a	a			a	a	a	a	a	a	a		a	a			g	a		
c	3389815	t	3532133	nc	t	clone D	++	t	t	t	t	t	t			t	t	t	t	t	t	t		t	t			t	t		
t	3389818	g	3532136	nc	g	clone D	++	g	g	g	g	g	g			g	g	g	g	g	g	g		g	g			g	g		
g	3389819	a	3532137	nc	a	clone D	++	a	a	a	a	a	a			c	a	a	a	a	a	a		a	a			a	a		
g	3389822	a	3532140	nc	a	clone D	++	a	g	a	a	a	a			a	a	a	a	a	a	a		a	a			a	a		
a	3389824	c	3532142	nc	c	clone D	++	c	a	c	c	c	c			c	c	c	c	c	c	c		t	c			t	c		
t	3389826	g	3532144	nc	g	clone D	++	g	g	g	g	g	g			g	g	g	g	g	g	g		g	g			g	g		
g	3389852	c	3532170	nc	c	clone D	++	c	c	c	c	c	c			g	c	c	g	g	g	g		g	g			c	g		
g	3389856	a	3532174	nc	g	CFT073	++	g	g	g	g	g	g			g	g	g	g	g	g	g		g	g			g	g		
g	3389929	a	3532247	ns	a	clone D	++	a	a	a	a	a	a			g	a	a	g	g	g	g		a	g			g	g		
c	3389986	g	3532304	ns	g	clone D	++	g	g	g	g	g	g			g	g	g	g	g	g	g		g	g			g	g		
g	3390043	a	3532361	ns	a	clone D	++	a	a	a	a	a	a			a	a	a	a	g	a	a		a	a			a	a		
t	3390121	a	3532439	ns	a	clone D	++	a	a	a	a	a	a			a	a	a	a	a	a	a		a	a			a	a		
c	3390178	t	3532496	ns	t	clone D	++	t	t	t	t	t	t			t	t	t	t	t	t	t		c	t			t	t		
g	3390180	a	3532498	ns	a	clone D	++	a	a	a	a	a	a			a	a	a	a	a	a	a		g	a			a	a		
g	3390181	t	3532499	ns	t	clone D	++	t	t	t	t	t	t			a	t	t	a	a	a	a		g	a			a	a		

Clone D base	Clone D site	CFT 073 base	CFT 073 site	mutation type[a]	Inferred ancestral base[b]	lineage inferred to mutate	Support level[c]	ED1a[d]	536	S88	APEC O1	UTI89	E2348/69	ATCC8739	K-12	HS	SMS3-5	IAI39	E24377A	SE11	55989	IAI1	Sakai	UMN026	B4 Sb227	F5 8401	SS Ss046	D1 Sd197	B18 BS512	F2a 2457T	F2a 301
g	3390184	a	3532502	ns	a	clone D	++	a	a	a	a	a	a			a	a	a	a	a	a	a		a	a			a	a		
t	3390207	c	3532525	ns	c	clone D	++	c	c	c	c	c	c			t	c	c	t	t	t	t		t	t			t	t		
g	3390228	a	3532546	ns	a	clone D	++	a	g	a	a	a	a			g	g	g	g	g	g	g		g	g			g	g		
t	3390250	c	3532568	ns	c	clone D	++	c	c	c	c	c	c			c	c	t	c	c	c	c		c	c			c	c		
c	3390268	t	3532586	ns	c	CFT073	++	c	t	c	c	c	c			c	c	c	c	c	c	c		t	t			c	t		
a	3405079	g	3534253	nc	g	clone D	++	g	g	g	g	g	g	g	g	g	g	g	g	g	g	g		g		g				g	g
c	3405139	a	3534313	s	a	clone D	++	a	c	a	a	a	a	a	a	a	a	c	a	a	a	a		a		a				-	-
a	3405142	g	3534316	s	g	clone D	++	g	a	g	g	g	g	g	g	g	g	a	g	g	g	g		g		g				g	g
g	3405151	a	3534325	s	a	clone D	++	a	g	a	a	a	a	a	a	a	a	g	a	a	a	a		a		a				a	a
g	3405196	a	3534370	s	g	CFT073	+	c	g	c	c	c	g	g	g	g	g	g	g	g	g	g		g		g				g	g
a	3405241	g	3534415	s	g	clone D	++	g	g	g	g	g	g	g	g	g	g	g	g	g	g	g		g		g				g	g
a	3405244	g	3534418	s	g	clone D	++	g	a	g	g	g	g	g	g	g	g	a	g	g	g	g		g		g				g	g
a	3405274	g	3534448	s	g	clone D	++	g	a	g	g	g	a	g	g	g	g	a	a	a	a	a		a		a				a	a
a	3405292	g	3534466	s	g	clone D	++	g	a	g	g	g	g	g	g	g	g	a	a	a	a	a		g		a				a	a
a	3405346	g	3534520	s	g	clone D	++	g	g	g	g	g	g	g	g	g	g	g	g	g	g	g		g		g				g	g
a	3405352	t	3534526	s	t	clone D	++	t	a	a	a	a	a	a	a	a	a	a	t	t	t	a		a		t				t	t
g	3405355	a	3534529	s	a	clone D	++	a	g	g	g	g	g	g	g	g	g	g	a	a	a	g		g		a				a	a
g	3405358	a	3534532	s	a	clone D	++	a	g	g	g	g	g	g	g	g	g	g	a	a	a	g		a		a				a	a
a	3405409	g	3534583	s	g	clone D	++	g	a	a	a	a	g	g	g	g	g	a	a	a	a	g		g		a				a	a
c	3405412	t	3534586	s	t	clone D	++	t	c	c	c	c	c	c	c	c	c	c	c	c	c	c		c		c				c	c
t	3405433	a	3534607	s	a	clone D	++	a	t	t	t	t	t	t	t	t	t	t	t	t	t	t		t		t				t	t
g	3405463	a	3534637	s	a	clone D	++	a	g	a	a	a	a	g	g	g	g	g	g	g	g	g		a		g				g	g
g	3405487	a	3534661	s	g	CFT073	++	g	g	g	g	g	g	g	g	g	g	g	g	g	g	g		g		g				g	g
c	3405490	t	3534664	s	c	CFT073	++	c	c	c	c	c	c	c	c	c	c	c	c	c	c	c		c		t				t	t
a	3405513	g	3534687	s	a	CFT073	++	a	a	a	a	a	g	g	g	g	g	a	a	a	a	a		g		a				a	a
c	3405559	a	3534733	s	a	clone D	++	a	c	c	c	c	c	a	a	a	a	c	c	c	c	a		a		c				c	c
g	3405571	c	3534745	s	c	clone D	++	c	g	g	g	g	c	c	c	c	c	g	g	g	g	g		c		g				g	g
t	3405576	c	3534750	ns	c	clone D	++	c	c	c	c	c	c	c	c	c	c	c	c	c	c	c		c		c				c	c

Clone D base	Clone D site	CFT 073 base	CFT 073 site	mutation type[a]	Inferred ancestral base[b]	lineage inferred to mutate	Support level[c]	ED1a[d]	536	S88	APEC O1	UTI89	E2348/69	ATCC8739	K-12	HS	SMS3-5	IAI39	E24377A	SE11	55989	IAI1	Sakai	UMN026	B4 Sb227	F5 8401	SS Ss046	D1 Sd197	B18 BS512	F2a 2457T	F2a 301
g	3405625	a	3534799	s	g	CFT073	++	g	g	g	g	g	g	g	g	g	g	g	g	g	g	g		g		g				g	g
c	3405631	a	3534805	s	g	clone D	++	g	c	c	c	c	c	c	c	c	c	c	c	c	c	c		a		c				c	c
a	3405637	g	3534811	s	g	clone D	++	g	a	a	a	a	a	g	g	g	g	a	a	a	a	a		a		a				a	a
a	3405658	g	3534832	s	g	clone D	++	g	a	g	g	g	a	a	a	a	a	a	a	a	a	a		a		a				a	a
t	3405670	c	3534844	s	c	clone D	++	c	t	t	t	t	t	t	t	t	t	t	t	t	t	t		t		t				t	t
t	3405673	c	3534847	s	c	clone D	++	c	t	t	t	t	t	t	t	t	t	t	t	t	t	t		c		t				t	t
t	3405688	g	3534862	s	g	clone D	++	g	t	t	t	t	t	t	t	t	t	t	t	t	t	t		t		t				t	t
c	3405700	g	3534874	s	g	clone D	++	g	c	c	c	c	c	c	c	c	c	c	c	c	c	c		c		c				c	c
g	3405807	a	3534981	s	a	clone D	++	a	g	g	g	g	g	g	g	g	g	g	g	g	g	g		g		g				g	g
c	3405912	a	3535086	ns	c	CFT073	++	c	c	c	c	c	c	c	c	c	c	c	c	c	c	c		c		c				c	c
g	3406465	a	3535639	s	g	CFT073	++	g	g	g	g	g	g	g	g	g	g	g	g	g	g	g		g		g				g	g
g	3406495	c	3535669	s	c	clone D	++	c	g	c	c	c	c	c	c	c	c	g	g	g	g	g		g		c				c	c
a	3406631	g	3535805	ns	a	CFT073	++	a	a	a	a	a	a	a	a	a	a	a	a	a	a	a		a		a				a	a
t	3406768	g	3535942	nc	g	clone D	++	g	g	g	g	g	g	g	g	g	g	t	g	g	g	g		g		g				g	g
a	3406950	g	3536124	nc	g	clone D	++	g	a	g	g	g	g	g	g	g	g	a	g	g	g	g		a		g				g	g
a	3407250	c	3536424	ns	c	clone D	++	c	c	c	c	c	c	c	c	c	c	a	c	c	c	c		c		c				c	c
t	3407346	c	3536520	ns	c	clone D	++	c	c	c	c	c	c	c	c	c	c	t	c	c	c	t		c		c				c	c
g	3407419	a	3536593	s	g	CFT073	++	g	g	g	g	g	g	g	g	g	g	g	g	g	g	g		g		g				g	g
a	3407524	c	3536698	s	c	clone D	++	c	t	c	c	c	c	c	c	c	t	a	c	c	c	c		c		c				c	c
c	3407548	a	3536722	s	a	clone D	++	a	c	a	a	a	a	a	a	a	a	c	c	c	a	c		c		c				c	c
c	3407603	g	3536777	ns	g	clone D	++	g	c	g	g	g	c	g	g	g	g	c	g	g	g	g		c		g				g	g
c	3407620	t	3536794	s	t	clone D	++	t	t	t	t	t	c	t	t	t	t	c	t	t	t	t		c		t				t	t
a	3407671	g	3536845	s	g	clone D	++	g	a	g	g	g	a	a	a	a	a	a	a	a	a	a		a		a				a	a
g	3407674	a	3536848	s	a	clone D	++	a	g	a	a	a	g	g	g	g	g	g	g	g	g	g		g		g				g	g
t	3407680	c	3536854	s	c	clone D	++	c	t	c	c	c	t	t	t	t	t	t	t	t	t	t		t		t				t	t
t	3407695	c	3536869	s	c	clone D	++	c	c	c	c	c	t	t	t	t	t	t	t	t	t	t		t		t				t	t
g	3407715	a	3536889	s	a	clone D	++	a	g	a	a	a	g	g	g	g	g	g	g	g	g	g		g		g				g	g
c	3407770	g	3536944	s	g	clone D	++	g	g	g	g	g	g	g	g	g	g	c	g	g	g	g		g		g				g	g

Clone D base	Clone D site	CFT 073 base	CFT 073 site	mutation type[a]	Inferred ancestral base[b]	lineage inferred to mutate	Support level[c]	ED1a[d]	536	S88	APEC O1	UTI89	E2348/69	ATCC8739	K-12	HS	SMS3-5	IAI39	E24377A	SE11	55989	IAI1	Sakai	UMN026	B4 Sb227	F5 8401	SS Ss046	D1 Sd197	B18 BS512	F2a 2457T	F2a 301
a	3407791	t	3536965	s	t	clone D	++	t	a	t	t	t	a	a	a	a	a	a	a	a	a	a		a		a				a	a
g	3407794	a	3536968	s	a	clone D	++	a	g	a	a	a	g	g	g	g	g	g	g	g	g	g		g		g				g	g
c	3407797	t	3536971	s	t	clone D	++	t	c	t	t	t	c	c	c	c	c	c	c	c	c	c		c		c				c	c
a	3407890	c	3537064	s	c	clone D	++	c	a	c	c	c	a	a	a	a	a	a	a	a	a	a		a		a				a	a
a	3407962	g	3537136	s	g	clone D	++	g	g	g	g	g	g	g	g	g	g	a	g	g	g	g		g		g				g	g
a	3408004	g	3537178	s	g	clone D	++	g	a	g	g	g	g	a	a	a	a	a	a	a	a	a		a		g				g	g
t	3408066	c	3537240	ns	c	clone D	++	c	t	c	c	c	c	t	t	t	t	t	t	t	t	t		t		t				t	t
t	3408072	a	3537246	ns	a	clone D	++	a	t	a	a	a	t	t	t	t	t	t	t	t	t	t		t		t				t	t
g	3408291	a	3537465	s	a	clone D	++	a	g	a	a	a	g	g	g	g	g	g	g	g	g	g		a		a				a	a
g	3408319	t	3537493	s	t	clone D	++	t	g	t	t	t	g	g	g	g	g	g	g	g	g	g		g		g				g	g
g	3408331	a	3537505	s	a	clone D	++	a	g	a	a	a	g	g	g	g	g	g	g	g	g	g		g		g				g	g
a	3408337	c	3537511	ns	c	clone D	++	c	a	c	c	c	c	a	a	a	a	a	a	a	a	a		c		c				c	c
t	3408379	c	3537553	s	c	clone D	++	c	t	c	c	c	t	t	t	t	t	t	t	t	t	t		t		t				t	t
t	3408382	c	3537556	s	c	clone D	++	c	t	c	c	c	c	c	c	c	c	t	c	c	c	c		c		c				c	c
g	3408466	a	3537640	s	a	clone D	++	a	g	a	a	a	g	g	g	g	g	g	g	g	g	g		g		g				g	g
a	3408526	g	3537700	s	g	clone D	++	g	a	g	g	g	a	g	g	g	g	a	g	g	g	g		a		g				g	g
g	3408534	a	3537708	ns	g	CFT073	++	g	g	g	g	g	g	g	g	g	g	g	g	g	g	g		g		g				g	g
a	3408565	g	3537739	s	g	clone D	++	g	a	g	g	g	g	g	g	g	a	a	a	a	a	a		g		a				a	a
c	3408571	t	3537745	s	t	clone D	++	t	c	t	t	t	c	c	c	c	c	c	c	c	c	c		c		c				c	c
t	3408574	c	3537748	s	c	clone D	++	c	t	c	c	c	c	c	c	c	t	t	t	t	t	t		c		t				t	t
a	3408586	g	3537760	s	g	clone D	++	g	a	g	g	g	g	g	g	g	g	a	g	g	g	g		g		g				g	g
g	3408622	a	3537796	ns	a	clone D	++	a	g	a	a	a	g	g	g	g	g	g	g	g	g	g		g		g				g	g
c	3408624	t	3537798	ns	t	clone D	++	t	c	t	t	t	c	c	c	c	c	c	c	c	c	c		c		c				c	c
c	3408649	t	3537823	s	t	clone D	++	t	c	t	t	t	c	t	t	t	c	c	c	c	c	c		c		t				t	t
c	3408652	t	3537826	s	t	clone D	++	t	c	t	t	t	c	t	t	t	c	c	c	c	c	c		c		t				t	t
g	3408655	a	3537829	s	a	clone D	++	a	g	a	a	a	g	a	a	a	g	g	g	g	g	g		g		a				a	a
g	3408745	a	3537919	s	g	CFT073	++	g	g	a	a	a	g	g	g	g	g	g	g	g	g	g		g		g				g	g
g	3408909	a	3538083	s	g	CFT073	++	g	g	g	g	g	g	g	g	g	g	g	g	g	g	g		g		g				g	g

Clone D base	Clone D site	CFT 073 base	CFT 073 site	mutation type[a]	Inferred ancestral base[b]	lineage inferred to mutate	Support level[c]	ED1a[d]	536	S88	APEC O1	UTI89	E2348/69	ATCC8739	K-12	HS	SMS3-5	IAI39	E24377A	SE11	55989	IAI1	Sakai	UMN026	B4 Sb227	F5 8401	SS Ss046	D1 Sd197	B18 BS512	F2a 2457T	F2a 301
t	3408928	c	3538102	s	c	clone D	++	c	t	c	c	c	t	t	t	t	t	t	t	t	t	t		t		t				t	t
a	3408976	g	3538150	s	g	clone D	++	g	a	g	g	g	a	a	a	a	a	a	a	a	a	a		a		a				a	a
t	3408997	a	3538171	s	?	?	+/-	c	c	a	a	a	c	c	c	c	c	t	c	c	c	c		c		c				c	c
c	3409001	t	3538175	ns	t	clone D	++	t	c	t	t	t	c	c	c	c	c	c	c	c	c	c		c		c				c	c
c	3409003	g	3538177	ns	g	clone D	++	g	c	g	g	g	a	c	c	c	c	c	c	c	c	c		c		c				c	c
c	3409006	a	3538180	s	a	clone D	++	a	c	a	a	a	c	c	c	c	c	c	c	c	c	c		c		c				c	c
t	3409009	c	3538183	s	c	clone D	++	c	t	c	c	c	t	t	t	t	t	t	t	t	t	t		t		t				t	t
c	3409012	t	3538186	s	t	clone D	++	t	t	t	t	t	t	c	c	c	c	c	c	c	c	c		c		c				c	c
t	3409123	c	3538297	s	c	clone D	++	c	t	c	c	c	t	t	t	t	c	t	t	t	t	t		t		t				t	t
t	3409144	c	3538318	s	c	clone D	++	c	t	c	c	c	t	t	t	t	c	t	t	t	t	t		t		t				t	t
t	3409150	c	3538324	s	c	clone D	++	c	t	c	c	c	t	t	t	t	c	t	t	t	t	t		t		t				t	t
a	3409176	c	3538350	ns	c	clone D	++	c	a	c	c	c	c	a	a	a	c	a	a	a	a	a		c		c				c	c
c	3409181	t	3538355	ns	t	clone D	++	t	c	t	t	t	c	c	c	c	t	c	c	c	c	c		c		t				t	t
g	3409186	a	3538360	s	g	CFT073	++	g	g	g	g	g	g	g	g	g	g	g	g	g	g	g		g		g				g	g
c	3409192	g	3538366	s	g	clone D	++	g	c	g	g	g	g	c	c	c	g	c	c	c	c	c		g		a				a	a
g	3409198	a	3538372	s	a	clone D	++	a	g	a	a	a	g	g	g	g	a	g	g	g	g	g		a		g				g	g
a	3409203	g	3538377	s	g	clone D	++	g	a	g	g	g	g	g	g	g	g	a	g	g	g	g		g		g				g	g
c	3409204	t	3538378	ns	t	clone D	++	t	c	t	t	t	t	c	c	c	t	c	c	c	c	c		t		t				t	t
c	3409205	t	3538379	ns	t	clone D	++	t	c	t	t	t	t	c	c	c	t	c	c	c	c	c		t		t				t	t
t	3409207	c	3538381	ns	c	clone D	++	c	t	c	c	c	c	t	t	t	c	t	t	t	t	t		t		c				c	c
g	3409208	a	3538382	ns	a	clone D	++	a	g	a	a	a	a	g	g	g	a	g	g	g	g	g		a		a				a	a
t	3409209	c	3538383	ns	c	clone D	++	c	t	c	c	c	c	t	t	t	c	t	t	t	t	t		c		c				c	c
t	3409210	g	3538384	s	g	clone D	++	g	t	g	g	g	g	t	t	t	g	t	t	t	t	t		g		g				g	g
c	3409213	a	3538387	s	a	clone D	++	a	c	a	a	a	c	c	c	c	a	c	c	c	c	c		c		c				c	c
t	3409254	c	3538428	ns	c	clone D	++	c	t	c	c	c	c	c	c	c	c	t	c	c	c	c		c		c				c	c
c	3409296	a	3538470	nc	a	clone D	++	a	a	a	a	a	c	a	a	a	c	c	a	a	a	a		a		c				c	c
t	3409432	c	3538606	s	c	clone D	++	c	c	c	c	c	c	c	c	c	c	t	c	c	c	c		c		c				c	c
t	3409441	c	3538615	s	c	clone D	++	c	c	c	c	c	c	t	t	t	c	t	c	t	t	t		c		c				c	c

Clone D base	Clone D site	CFT 073 base	CFT 073 site	mutation type[a]	Inferred ancestral base[b]	lineage inferred to mutate	Support level[c]	ED1a[d]	536	S88	APEC O1	UTI89	E2348/69	ATCC8739	K-12	HS	SMS3-5	IAI39	E24377A	SE11	55989	IAI1	Sakai	UMN026	B4 Sb227	F5 8401	SS Ss046	D1 Sd197	B18 BS512	F2a 2457T	F2a 301
a	3409443	g	3538617	s	g	clone D	++	g	g	g	g	g	g	a	a	a	g	a	g	a	a	a		g		g				g	g
c	3409495	g	3538669	s	g	clone D	++	g	g	g	g	g	g	g	g	g	g	c	g	g	g	g		g		g				g	g
g	3409639	a	3538813	s	a	clone D	++	a	a	a	a	a	a	a	a	a	a	g	a	a	a	a		a		a				a	a
c	3409880	t	3539054	s	t	clone D	++	t	c	t	t	t	t	t	t	t	t	c	t	t	t	t		t		t				t	t
c	3410114	t	3539288	s	c	CFT073	++	c	c	c	c	c	t	t	t	t	t	c	t	t	t	t		t		t				t	t
t	3410124	a	3539298	ns	t	CFT073	++	t	t	t	t	t	c	t	t	t	t	t	t	t	t	t		t		t				t	t
g	3410192	a	3539366	s	g	CFT073	++	g	g	a	a	a	g	g	g	g	g	g	g	g	g	g		g		g				g	g
g	3410195	a	3539369	s	a	clone D	++	a	g	a	a	a	a	g	g	g	a	g	a	a	a	a		a		a				a	a
a	3410204	g	3539378	s	g	clone D	++	g	a	g	g	g	g	g	g	g	g	a	g	g	g	g		g		g				g	g
c	3410207	t	3539381	s	t	clone D	++	t	c	t	t	t	t	t	t	t	t	c	t	t	t	t		t		t				t	t
a	3410216	t	3539390	s	t	clone D	++	t	a	t	t	t	t	t	t	t	t	a	t	t	t	t		t		t				t	t
c	3410456	t	3539630	s	c	CFT073	++	c	c	t	t	t	c	c	c	c	c	c	c	c	c	c		c		c				c	c
c	3410480	g	3539654	s	c	CFT073	++	c	g	g	g	g	c	c	c	c	c	c	c	c	c	c		g		c				c	c
g	3410675	a	3539849	s	a	clone D	++	a	g	a	a	a	g	g	g	g	g	g	g	g	g	g		g		g				g	g
g	3410756	t	3539930	ns	t	clone D	++	t	g	t	t	t	t	t	t	t	t	g	t	t	t	t		t		t				t	t
g	3410762	a	3539936	s	g	CFT073	++	g	g	g	g	g	a	g	g	g	g	g	g	g	g	g		a		g				g	g
g	3410825	a	3539999	s	a	clone D	++	a	g	a	a	a	g	a	a	a	g	g	a	a	a	a		g		a				a	a
c	3410903	g	3540077	s	g	clone D	++	g	c	g	g	g	c	g	g	g	c	c	c	g	g	g		c		g				g	g
g	3410927	t	3540101	s	t	clone D	++	t	g	t	t	t	g	a	a	a	g	g	a	a	a	a		g		g				g	g
t	3410934	c	3540108	ns	c	clone D	++	c	t	c	c	c	c	t	t	t	t	t	t	t	t	t		c		c				c	c
t	3410956	c	3540130	s	c	clone D	++	c	t	c	c	c	c	c	c	c	t	t	c	c	c	c		c		c				c	c
c	3410961	t	3540135	s	t	clone D	++	t	c	t	t	t	t	t	t	t	c	c	t	t	t	t		t		t				t	t
a	3410982	g	3540156	s	g	clone D	++	g	a	g	g	g	g	g	g	g	a	a	g	g	g	g		g		g				g	g
g	3411000	a	3540174	s	a	clone D	++	a	g	a	a	a	g	g	g	g	g	g	g	g	g	g		g		g				g	g
a	3411042	t	3540216	s	t	clone D	++	t	a	t	t	t	a	a	a	a	a	a	a	a	a	a		a		a				a	a
a	3411060	g	3540234	s	g	clone D	++	g	a	g	g	g	a	g	g	g	a	a	g	g	g	g		a		g				g	g
a	3411066	g	3540240	s	g	clone D	++	g	a	g	g	g	a	a	a	a	a	a	c	a	a	c		a		a				a	a
a	3411099	c	3540273	s	c	clone D	++	c	a	a	a	a	a	a	a	a	a	a	a	a	a	a		a		a				a	a

Clone D base	Clone D site	CFT 073 base	CFT 073 site	mutation type[a]	Inferred ancestral base[b]	lineage inferred to mutate	Support level[c]	ED1a[d]	536	S88	APEC O1	UTI89	E2348/69	ATCC8739	K-12	HS	SMS3-5	IAI39	E24377A	SE11	55989	IAI1	Sakai	UMN026	B4 Sb227	F5 8401	SS Ss046	D1 Sd197	B18 BS512	F2a 2457T	F2a 301
a	3411111	g	3540285	s	g	clone D	++	g	g	g	g	g	g	g	g	g	a	a	g	g	g	g		g		g				g	g
g	3411129	a	3540303	s	a	clone D	++	a	g	a	a	a	g	g	g	g	g	g	g	g	g	g		g		g				g	g
g	3411213	a	3540387	s	a	clone D	++	a	g	a	a	a	g	g	a	a	g	g	a	g	g	g		g		g				g	g
g	3411222	t	3540396	s	t	clone D	++	t	g	t	t	t	g	t	t	t	t	g	t	t	t	t		g		t				t	t
a	3411235	g	3540409	ns	a	CFT073	++	a	a	a	a	a	a	a	a	a	a	a	a	a	a	a		a		a				a	a
c	3411297	t	3540471	s	t	clone D	++	t	c	t	t	t	c	c	g	g	c	c	c	c	c	c		c		c				c	c
c	3411312	a	3540486	s	a	clone D	++	a	c	a	a	a	c	c	a	a	a	c	c	c	c	c		a		a				a	a
a	3411407	g	3540581	s	g	clone D	++	g	a	g	g	g	a	g	a	a	a	a	g	g	g	g		a		a				a	a
t	3411408	c	3540582	s	c	clone D	++	c	t	c	c	c	t	c	t	t	t	t	c	c	c	c		t		t				t	t
a	3411471	g	3540645	s	g	clone D	++	g	a	g	g	g	a	g	g	g	a	a	g	g	g	g		a		g				g	g
t	3411479	a	3540653	ns	a	clone D	++	a	t	a	a	a	t	a	a	a	t	t	a	a	a	a		t		a				a	a
t	3411486	c	3540660	s	c	clone D	++	c	t	c	c	c	t	c	c	c	t	t	c	c	c	c		t		c				c	c
a	3411525	g	3540699	s	g	clone D	++	g	a	g	g	g	c	g	g	g	a	a	g	g	g	g		a		g				g	g
c	3411528	a	3540702	s	a	clone D	++	a	c	a	a	a	c	a	a	a	c	c	a	a	a	a		c		a				a	a
a	3411594	g	3540768	s	g	clone D	++	g	g	g	g	g	g	g	g	g	g	a	g	g	g	g		g		g				g	g
g	3411699	a	3540873	s	a	clone D	++	a	g	a	a	a	g	g	g	g	g	g	g	g	g	g		g		g				g	g
t	3411704	c	3540878	ns	c	clone D	++	c	c	c	c	c	c	c	c	c	t	t	c	c	c	c		t		c				c	c
a	3411720	g	3540894	s	g	clone D	++	g	g	g	g	g	g	g	g	g	g	a	g	g	g	g		a		g				g	g
t	3411738	c	3540912	s	t	CFT073	++	t	t	c	c	c	t	c	c	c	c	t	c	c	c	c		t		c				c	c
c	3411780	t	3540954	s	c	CFT073	++	c	c	t	t	t	c	c	c	c	c	c	c	c	c	c		c		c				c	c
a	3411813	c	3540987	s	c	clone D	++	c	a	c	c	c	c	c	c	c	c	a	c	c	c	c		a		a				a	a
a	3411894	g	3541068	s	a	CFT073	++	a	a	g	g	g	g	g	g	g	a	a	g	g	g	g		a		g				g	g
g	3412028	a	3541202	s	g	CFT073	++	g	g	a	a	a	g	g	g	g	a	g	g	g	g	g		g		a				a	a
c	3412100	t	3541274	s	c	CFT073	++	c	c	t	t	t	t	c	c	c	t	c	c	c	c	c		c		c				c	c
a	3412103	t	3541277	s	a	CFT073	++	a	a	t	t	t	t	a	a	a	t	a	a	a	a	a		a		a				a	a
a	3412208	g	3541382	s	g	clone D	++	g	g	g	g	g	g	g	g	g	a	a	g	g	g	g		g		g				g	g
c	3412274	t	3541448	s	t	clone D	++	t	c	t	t	t	t	c	c	c	c	c	c	c	c	c		c		c				c	c
g	3412367	a	3541541	s	g	CFT073	++	g	g	g	g	g	g	a	a	a	g	g	a	a	a	a		g		a				a	a

Clone D base	Clone D site	CFT 073 base	CFT 073 site	mutation type[a]	Inferred ancestral base[b]	lineage inferred to mutate	Support level[c]	ED1a[d]	536	S88	APEC O1	UTI89	E2348/69	ATCC8739	K-12	HS	SMS3-5	IAI39	E24377A	SE11	55989	IAI1	Sakai	UMN026	B4 Sb227	F5 8401	SS Ss046	D1 Sd197	B18 BS512	F2a 2457T	F2a 301
c	3412421	g	3541595	s	g	clone D	++	g	c	g	g	g	g	g	g	g	g	c	g	g	g	g		g		g				g	g
g	3412427	a	3541601	s	g	CFT073	++	g	g	g	g	g	g	a	a	a	g	g	a	a	a	a		g		a				a	a
t	3412430	c	3541604	s	c	clone D	++	c	t	c	c	c	c	c	c	c	c	t	c	c	c	c		c		c				c	c
a	3412448	g	3541622	s	g	clone D	++	g	a	g	g	g	g	g	g	g	g	a	g	g	g	g		g		g				g	g
a	3412466	g	3541640	s	g	clone D	++	g	a	g	g	g	g	g	g	g	g	a	g	g	g	g		g		g				g	g
g	3412469	a	3541643	s	a	clone D	++	a	g	a	a	a	a	a	a	a	a	g	a	a	a	a		a		a				a	a
c	3412472	t	3541646	s	t	clone D	++	t	c	t	t	t	t	t	t	t	t	c	t	t	t	t		t		t				t	t
c	3412475	t	3541649	s	t	clone D	++	t	c	t	t	t	t	t	t	t	t	c	t	t	t	t		t		t				t	t
t	3412478	c	3541652	s	c	clone D	++	c	t	c	c	c	c	c	c	c	c	t	c	c	c	c		c		c				c	c
t	3412487	c	3541661	s	c	clone D	++	c	g	c	c	c	c	c	c	c	c	t	c	c	c	c		c		c				c	c
g	3412490	a	3541664	s	a	clone D	++	a	g	a	a	a	a	a	a	a	a	g	a	a	a	a		a		a				a	a
c	3412493	t	3541667	s	t	clone D	++	t	c	t	t	t	t	t	t	t	t	c	t	t	t	t		t		t				t	t
g	3412499	a	3541673	s	a	clone D	++	a	g	a	a	a	a	a	a	a	a	g	a	a	a	a		a		a				a	a
c	3412631	t	3541805	s	t	clone D	++	t	c	t	t	t	t	t	t	t	t	c	t	t	t	t		t		t				t	t
t	3412634	c	3541808	s	c	clone D	++	c	t	c	c	c	c	c	c	c	c	t	c	c	c	c		c		c				c	c
a	3412649	g	3541823	s	a	CFT073	++	a	a	a	a	a	a	g	g	g	a	a	g	g	g	g		g		g				g	g
t	3412670	a	3541844	s	a	clone D	++	a	t	a	a	a	a	a	a	a	a	t	a	a	a	a		a		a				a	a
a	3412685	c	3541859	s	c	clone D	++	c	c	c	c	c	c	c	c	c	c	a	c	c	c	c		t		c				c	c
c	3412799	a	3541973	s	a	clone D	++	a	a	a	a	a	a	a	a	a	c	c	a	a	a	a		a		a				a	a
a	3412834	c	3542008	ns	c	clone D	++	c	c	c	c	c	c	c	c	c	c	a	c	c	c	c		c		c				c	c
a	3412877	g	3542051	s	a	CFT073	++	a	g	a	a	a	a	g	g	g	a	a	g	g	g	g		g		g				g	g
c	3412904	t	3542078	s	c	CFT073	++	c	c	c	c	c	c	t	t	t	c	c	t	t	t	t		c		t				t	t
c	3412925	a	3542099	s	c	CFT073	++	c	c	c	c	c	c	c	a	c	c	c	c	c	c	c		c		c				c	c
c	3412934	t	3542108	s	c	CFT073	++	c	c	c	c	c	c	t	t	t	c	c	c	c	c	c		c		c				c	c
a	3412943	g	3542117	s	a	CFT073	++	a	g	a	a	a	a	g	g	g	g	a	g	g	g	g		g		g				g	g
t	3412985	c	3542159	s	t	CFT073	++	t	c	t	t	t	t	c	c	c	t	t	c	c	c	c		c		c				c	c
a	3413006	g	3542180	s	a	CFT073	++	a	g	a	a	a	a	g	g	g	a	a	g	g	g	g		g		g				g	g
a	3413012	g	3542186	s	a	CFT073	++	a	a	a	a	a	a	g	g	g	a	a	a	g	a	g		a		a				a	a

Clone D base	Clone D site	CFT 073 base	CFT 073 site	mutation type[a]	Inferred ancestral base[b]	lineage inferred to mutate	Support level[c]	ED1a[d]	536	S88	APEC O1	UTI89	E2348/69	ATCC8739	K-12	HS	SMS3-5	IAI39	E24377A	SE11	55989	IAI1	Sakai	UMN026	B4 Sb227	F5 8401	SS Ss046	D1 Sd197	B18 BS512	F2a 2457T	F2a 301
c	3413024	a	3542198	s	c	CFT073	++	c	c	c	c	c	c	a	a	a	c	c	c	t	c	t		c		c				c	c
c	3413042	g	3542216	s	c	CFT073	++	c	t	c	c	c	c	g	g	g	c	c	t	g	t	g		t		t				t	t
c	3413063	a	3542237	s	c	CFT073	++	c	c	c	c	c	c	a	a	a	c	c	a	a	a	a		a		a				a	a
a	3413066	g	3542240	s	a	CFT073	++	a	a	a	a	a	a	g	g	g	a	a	g	g	g	g		a		a				a	a
c	3413072	t	3542246	s	c	CFT073	++	c	c	c	c	c	c	t	t	t	c	c	t	t	t	t		c		c				c	c
a	3413078	g	3542252	s	a	CFT073	++	a	a	a	a	a	a	g	g	g	a	a	g	g	g	g		a		a				a	a
c	3413093	t	3542267	s	c	CFT073	++	c	c	c	c	c	c	t	t	t	c	c	c	t	c	t		c		c				c	c
t	3413115	a	3542289	ns	a	clone D	++	a	a	a	a	a	a	a	a	a	a	a	a	a	a	a		a		a				a	a
c	3413129	t	3542303	s	c	CFT073	++	c	c	c	c	c	c	t	t	t	c	c	a	t	a	t		c		c				c	c
a	3413144	g	3542318	s	g	clone D	++	g	a	g	g	g	g	g	g	g	a	a	g	g	g	g		g		g				g	g
g	3413279	a	3542453	s	g	CFT073	++	g	g	g	g	g	g	a	a	a	g	g	a	a	a	a		g		a				a	a
c	3413300	t	3542474	s	c	CFT073	++	c	t	c	c	c	c	t	t	t	c	c	t	t	t	t		t		t				t	t
c	3413315	t	3542489	s	c	CFT073	++	c	t	c	c	c	c	t	t	t	c	c	t	t	t	t		t		t				t	t
t	3413549	g	3542723	nc	t	CFT073	++	t	t	t	t	t	t	g	g	g	t	t	g	g	g	g		g		g				g	g
t	3413660	c	3542834	nc	t	CFT073	++	t	t	t	t	t	t	c	c	a	t	t	c	c	c	c		t		t				t	t
c	3414107	g	3543281	s	c	CFT073	++	c	c	c	c	c	g	g	g	g	c	c	g	g	g	g		g		g				g	g
a	3414256	t	3543430	ns	t	clone D	++	t	t	t	t	t	t	t	t	t	t	a	t	t	t	t		t		t				t	t
c	3414281	t	3543455	s	c	CFT073	++	c	t	c	c	c	c	t	t	t	t	c	t	t	t	t		c		t				t	t
g	3414296	a	3543470	s	g	CFT073	++	g	a	g	g	g	a	a	a	a	a	g	a	a	a	a		a		a				a	a
g	3414512	c	3543686	ns	g	CFT073	++	g	g	g	g	g	g	c	c	c	c	g	c	c		c		c		c				c	c
a	3414514	t	3543688	ns	a	CFT073	++	a	a	a	a	a	a	t	t	t		a	t	t		t		a							
g	3414516	a	3543690	ns	g	CFT073	++	g	g	g	g	g	g	a	a	a		g	a	a		a		a							
g	3414518	t	3543692	ns	g	CFT073	++	g	g	g	g	g	g	t	t	t		g	t	t		t		t							
g	3414520	a	3543694	s	g	CFT073	++	g	g	g	g	g	g	a	a	a		g	a	a		a		a							
a	3414521	g	3543695	s	a	CFT073	++	a	a	a	a	a	a	g	g	g		a	g	g		g		g							
c	3414525	g	3543699	nc	c	CFT073	++	c	c	c	c	c	t	g	g	g	g	c	g	g		g		g		g				g	g
c	3414526	t	3543700	nc	c	CFT073	++	c	c	c	c	c	c	t	t	t	t	c	t	t		t		t		t				t	t
c	3414529	t	3543703	ns	c	CFT073	++	c	c	c	c	c	c	t	t	t	t	c	t	t		t		t		t				t	t

Clone D base	Clone D site	CFT 073 base	CFT 073 site	mutation type[a]	Inferred ancestral base[b]	lineage inferred to mutate	Support level[c]	ED1a[d]	536	S88	APEC O1	UTI89	E2348/69	ATCC8739	K-12	HS	SMS3-5	IAI39	E24377A	SE11	55989	IAI1	Sakai	UMN026	B4 Sb227	F5 8401	SS Ss046	D1 Sd197	B18 BS512	F2a 2457T	F2a 301
a	3414689	g	3543863	s	g	clone D	++	g	a	g	g	g	g	g	g	g	g	a	g	g		g		g		g				g	g
c	3414729	g	3543903	ns	c	CFT073	++	c	c	c	c	c	c	g	g	g	c	c	g	g		g		c		g				g	g
t	3414777	c	3543951	s	c	clone D	++	c	t	c	c	c	c	c	c	c	t	t	c	c		c		t		c				c	c
t	3414852	c	3544026	ns	c	clone D	++	c	t	c	c	c	c	c	c	c	t	t	c	c		c		t		a				a	a
g	3414900	a	3544074	ns	g	CFT073	++	g	g	g	g	g	g	a	a	a	g	g	g	a		a		g		g				g	g
g	3414936	a	3544110	ns	a	clone D	++	a	a	a	a	a	a	a	a	a	g	g	a	a		a		a		g				g	g
a	3415002	g	3544176	ns	a	CFT073	++	a	g	a	a	a	a	g	g	g	g	a	g	g		g		g		g				g	g
g	3415047	a	3544221	ns	g	CFT073	++	g	g	g	g	g	g	a	a	a	a	g	a	a		a		g		a				a	a
c	3415074	t	3544248	ns	t	clone D	++	t	c	t	t	t	t	t	t	t	t	c	t	t		t		c		t				t	t
a	3415077	g	3544251	ns	g	clone D	++	g	a	g	g	g	g	g	g	g	g	a	g	g		g		a		g				g	g
a	3415175	g	3544349	s	g	clone D	++	g	a	g	g	g	g	g	g	g	a	a	g	g		g		a		g				g	g
a	3415206	g	3544380	ns	g	clone D	++	g	a	g	g	g	g	g	g	g	g	a	g	g		g		a		g				g	g
c	3415212	t	3544386	ns	t	clone D	++	t	c	t	t	t	t	t	t	t	t	c	t	t		t		c		t				t	t
a	3415236	g	3544410	ns	g	clone D	++	g	a	g	g	g	g	g	g	g	a	a	g	g		g		a		g				g	g
g	3415239	a	3544413	ns	a	clone D	++	a	g	a	a	a	a	a	a	a	g	g	a	a		a		g		a				a	a
t	3415672	g	3544846	ns	?	?	+/-	-	g	-	-	-	g	g		g	g	-	-	-		-		g		-				-	-
c	3415673	t	3544847	ns	?	?	+/-	-	t	-	-	-	t	t		t	t	-	-	-		-		t		-				-	-
a	3415681	g	3544860	nc	a	CFT073	++	a	g	a	a	a	g	g		g	g	a	a	a		a		g		a				a	a
a	3415698	g	3544877	nc	g	clone D	++	g	g	g	g	g	g	g		g	g	a	g	g		g		g		g				g	g
a	3415702	c	3544881	nc	a	CFT073	++	a	c	a	a	a	c	c		c	c	a	a	a		a		c		a				a	a
t	3415731	c	3544910	nc	t	CFT073	++	t	c	t	t	t	c	c		c	c	t	c	c		c		c		t				t	t
a	3415745	c	3544924	nc	c	clone D	++	c	c	c	c	c	c	c		c	c	a	c	c		c		c		t				t	t
c	3415747	a	3544926	nc	a	clone D	++	a	a	a	a	a	a	a		a	a	c	a	a		a		a		a				a	a
a	3415759	c	3544938	nc	c	clone D	++	c	c	c	c	c	c	c		c	c	a	c	c		c		c		c				c	c
t	3415760	g	3544939	nc	?	?	+/-	a	g	a	a	a	g	g		g	a	t	a	a		a		a		g				g	g
a	3415762	c	3544941	nc	a	CFT073	++	a	c	a	a	a	c	c		c	c	a	a	a		a		a		c				c	c
t	3415772	g	3544951	nc	g	clone D	++	g	g	g	g	g	g	g		g	g	t	g	g		g		g		g				g	g
a	3415796	g	3544975	nc	g	clone D	++	g	g	g	g	g	g	g		g	g	a	g	g		g		a		g				g	g

Clone D base	Clone D site	CFT 073 base	CFT 073 site	mutation type[a]	Inferred ancestral base[b]	lineage inferred to mutate	Support level[c]	ED1a[d]	536	S88	APEC O1	UTI89	E2348/69	ATCC8739	K-12	HS	SMS3-5	IAI39	E24377A	SE11	55989	IAI1	Sakai	UMN026	B4 Sb227	F5 8401	SS Ss046	D1 Sd197	B18 BS512	F2a 2457T	F2a 301
c	3415800	g	3544979	nc	g	clone D	++	g	g	a	a	a	g	g		g	g	c	g	g		g		g		g				g	g
c	3415803	a	3544982	nc	a	clone D	++	a	a	a	a	a	a	a		a	a	c	a	a		a		a		g				g	g
g	3415808	a	3544987	nc	a	clone D	++	a	a	a	a	a	a	a		a	a	g	a	a		a		a		a				a	a
a	3415809	c	3544988	nc	c	clone D	++	c	c	c	c	c	c	c		c	c	a	c	c		c		c		c				c	c
a	3415831	g	3545010	nc	g	clone D	++	g	g	g	g	g	g	g		g	g	a	g	g		g		g		g				g	g
g	3415832	t	3545011	nc	t	clone D	++	t	t	t	t	t	t	t		t	t	g	t	t		t		t		t				t	t
t	3415841	g	3545020	nc	g	clone D	++	g	g	g	g	g	g	g		g	g	t	g	g		g		g		g				g	g
g	3415845	a	3545024	nc	a	clone D	++	a	a	a	a	a	a	a		a	a	g	a	a		a		a		a				a	a
t	3415846	c	3545025	nc	c	clone D	++	c	c	c	c	c	c	c		c	c	t	c	c		c		c		c				c	c
a	3415848	g	3545027	nc	g	clone D	++	g	g	g	g	g	g	g		g	g	a	g	g		g		g		g				g	g
t	3415856	c	3545035	nc	c	clone D	++	c	c	c	c	c	c	c		c	c	t	c	c		c		c		c				c	c
c	3415858	t	3545037	nc	c	CFT073	++	c	t	c	c	c	t	t		t	c	c	t	t		t		t		t				t	t
a	3415859	c	3545038	nc	c	clone D	++	c	c	c	c	c	c	c		c	c	a	c	c		c		c		c				c	c
c	3415869	t	3545048	nc	t	clone D	++	t	t	t	t	t	t	t		t	t	c	t	t		t		t		t				t	t
c	3415880	t	3545059	nc	t	clone D	++	t	t	t	t	t	t	t		t	t	c	t	t		t		t		t				t	t
c	3415905	a	3545084	nc	a	clone D	++	a	a	a	a	a	a	a		a	a	c	a	a		a		a		a				a	a
a	3415911	t	3545090	nc	t	clone D	+	-	t	-	-	-	t	t		t	t	a	t	t		t		t		t				t	t
c	3415916	t	3545096	nc	c	CFT073	++	c	t	t	t	t	t	t		t	t	t	t	t		t		t		t				t	t
t	3415917	c	3545097	nc	t	CFT073	++	t	c	t	t	t	c	c		c	c	-	c	c		c		c		c				c	c
a	3415938	t	3545118	nc	t	clone D	++	t	a	t	t	t	t	t		t	t	a	t	t		t		t		t				t	t
a	3415940	t	3545120	nc	t	clone D	++	t	a	t	t	t	t	t		t	t	a	t	t		t		t		t				t	t
g	3415943	a	3545123	nc	a	clone D	++	a	a	a	a	a	a	a		a	a	g	a	a		a		a		a				a	a
c	3415947	t	3545127	nc	c	CFT073	++	c	c	c	c	c	t	t		t	t	c	c	c		c		t		c				c	c
t	3416015	g	3545195	nc	t	CFT073	++	t	t	t	t	t	g	g		g	t	t	t	t		t		t						t	t
g	3416100	t	3545280	s	g	CFT073	++	g	g	g	g	g	g	g		g	g	g	g	g		g		g				g		g	g
t	3416118	c	3545298	s	c	clone D	++	c	c	c	c	c	c	c		c	t	t	c	c		c		c				c		c	c
t	3416136	c	3545316	s	c	clone D	++	c	t	c	c	c	c	c		c	t	t	c	c		c		c				c		c	c
g	3416202	a	3545382	s	g	CFT073	++	g	g	g	g	g	g	a		a	g	g	g	g		g		g				a		g	g

Clone D base	Clone D site	CFT 073 base	CFT 073 site	mutation type[a]	Inferred ancestral base[b]	lineage inferred to mutate	Support level[c]	ED1a[d]	536	S88	APEC O1	UTI89	E2348/69	ATCC8739	K-12	HS	SMS3-5	IAI39	E24377A	SE11	55989	IAI1	Sakai	UMN026	B4 Sb227	F5 8401	SS Ss046	D1 Sd197	B18 BS512	F2a 2457T	F2a 301
g	3416280	a	3545460	s	g	CFT073	++	g	c	g	g	g	c	a		a	c	g	g	g		g		g				c		g	g
g	3416288	a	3545468	ns	g	CFT073	++	g	g	g	g	g	g	g		g	g	g	g	g		g		g				g		g	g
t	3416292	c	3545472	s	t	CFT073	++	t	t	t	t	t	c	c		c	c	t	t	t		t		t				c		t	t
c	3416361	t	3545541	s	c	CFT073	+	a	c	a	a	a	c	t		t	c	c	c	c		c		c				t		c	c
c	3416389	t	3545569	s	c	CFT073	++	c	c	c	c	c	c	t		t	c	c	c	c		c		c				t		c	c
g	3416397	t	3545577	s	t	clone D	++	t	t	t	t	t	t	t		t	t	g	t	t		t		t				t		t	t
a	3416473	g	3545653	ns	a	CFT073	++	a	a	a	a	a	a	g		g	a	a	a	a		a		a				g		a	a
t	3416478	c	3545658	s	c	clone D	++	c	t	c	c	c	c	c		c	c	t	c	c		c		c				c		c	c
t	3416541	c	3545721	s	c	clone D	++	c	t	c	c	c	c	c		c	c	t	c	c		c		c				c		c	c
a	3416544	g	3545724	s	g	clone D	++	g	a	g	g	g	g	g		g	g	a	g	g		g		g				g		g	g
a	3416556	g	3545736	s	g	clone D	++	g	a	a	a	a	a	g		g	g	a	g	g		g		g				g		g	g
t	3416613	c	3545793	s	c	clone D	++	c	c	c	c	c	c	c		c	c	t	c	c		c		c				c		c	c
c	3416616	t	3545796	s	t	clone D	++	t	t	t	t	t	t	t		t	t	c	t	t		t		t				t		t	t
t	3416619	c	3545799	s	c	clone D	++	c	c	c	c	c	c	c		c	c	t	c	c		c		c				c		c	c
t	3416622	c	3545802	s	c	clone D	++	c	c	c	c	c	c	c		c	c	t	c	c		c		c				c		c	c
c	3416625	t	3545805	s	t	clone D	++	t	t	t	t	t	t	t		t	t	c	t	t		t		t				t		t	t
t	3416664	c	3545844	s	t	CFT073	++	t	t	t	t	t	t	c		c	t	t	t	t		t		t				t		t	t
g	3416667	c	3545847	s	c	clone D	++	c	c	g	g	g	c	c		c	c	g	c	c		c		c				c		c	c
t	3416841	c	3546021	s	t	CFT073	++	t	c	t	t	t	c	c		c	c	c	c	c		c		c				c		c	c
a	3416859	g	3546039	s	a	CFT073	++	a	g	a	a	a	a	g		g	g	g	g	g		g		g				g		g	g
c	3416952	t	3546132	s	c	CFT073	++	c	t	c	c	c	a	t		t	t	t	t	t		t		c				t		t	t
a	3416964	c	3546144	s	a	CFT073	++	a	c	a	a	a	c	c		c	c	c	c	c		c		c				c		c	c
g	3417019	a	3546199	ns	g	CFT073	++	g	g	g	g	g	g	a		g	g	g	g	g		g		g				a		a	a
g	3417089	c	3546269	ns	g	CFT073	++	g	c	g	g	g	g	c		c	c	c	c	c		c		c				c		c	c
c	3417132	t	3546312	s	c	CFT073	++	c	t	c	c	c	c	t		t	t	c	t	t		t		t				t		t	t
t	3417159	a	3546339	s	t	CFT073	++	t	t	t	t	t	t	a		a	t	t	t	t		t		t				a		a	a
a	3417162	g	3546342	s	a	CFT073	++	a	a	a	a	a	a	g		g	a	a	a	a		a		a				g		g	g
t	3417195	c	3546375	s	t	CFT073	++	t	t	t	t	t	t	c		c	t	t	c	c		c		t				c		c	c

Clone D base	Clone D site	CFT 073 base	CFT 073 site	mutation type[a]	Inferred ancestral base[b]	lineage inferred to mutate	Support level[c]	ED1a[d]	536	S88	APEC O1	UTI89	E2348/69	ATCC8739	K-12	HS	SMS3-5	IAI39	E24377A	SE11	55989	IAI1	Sakai	UMN026	B4 Sb227	F5 8401	SS Ss046	D1 Sd197	B18 BS512	F2a 2457T	F2a 301
t	3417207	g	3546387	s	t	CFT073	++	t	g	g	g	g	g	g		g	g	g	g	g		g		g				g		g	g
c	3417216	t	3546396	s	c	CFT073	++	c	c	c	c	c	c	t		t	c	c	c	c		c		c				t		t	t
a	3417239	g	3546419	ns	a	CFT073	++	a	g	a	a	a	a	g		g	g	g	g	g		g		g				g		g	g
a	3417243	g	3546423	ns	a	CFT073	++	a	a	a	a	a	a	g		g	a	a	g	g		g		a				g		g	g
t	3417246	g	3546426	s	t	CFT073	++	t	t	t	t	t	t	g		g	t	t	g	g		g		t				g		g	g
c	3417285	t	3546465	s	c	CFT073	++	c	c	c	c	c	c	t		t	c	c	c	c		c		c				t		t	t
c	3417291	t	3546471	s	c	CFT073	++	c	c	c	c	c	c	t		t	c	c	c	c		c		c				t		t	t
g	3417303	t	3546483	s	g	CFT073	++	g	t	g	g	g	g	t		t	t	t	t	t		t		t				t		t	t
t	3417369	c	3546549	s	t	CFT073	++	t	c	t	t	t	t	c		c	c	c	c	c		c		c				c		c	c
c	3417465	t	3546645	s	c	CFT073	++	c	t	c	c	c	c	t		t	t	t	t	t		t		c				t		t	t
t	3417468	a	3546648	s	t	CFT073	++	t	t	t	t	t	t	a		t	t	t	t	t		t		t				a		a	a
g	3417480	a	3546660	s	g	CFT073	++	g	a	g	g	g	a	a		a	a	a	a	a		a		a				a		a	a
c	3417569	a	3546749	ns	c	CFT073	++	c	a	a	a	a	a	a		a	a	a	a	a		a		a				a		a	a
a	3417600	g	3546780	ns	a	CFT073	++	a	a	a	a	a	a	g		g	a	a	a	g		g		a				a		g	g
t	3417603	a	3546783	ns	t	CFT073	++	t	a	t	t	t	t	a		a	a	a	a	a		a		a				a		a	a
t	3417627	g	3546807	ns	t	CFT073	++	t	t	t	t	t	t	g		g	t	t	a	a		a		g				g		g	g
a	3417635	c	3546815	ns	a	CFT073	++	a	a	a	a	a	a	c		c	a	a	c	c		c		c				c		c	c
a	3417672	g	3546852	ns	a	CFT073	++	a	g	a	a	a	a	g		g	a	a	g	a		a		g				g		g	g
c	3417739	a	3546919	ns	c	CFT073	++	c	c	c	c	c	c	a		a	c	c	c	c		c		c				c		a	a
t	3417815	c	3546995	s	t	CFT073	++	t	c	t	t	t	t	c		c	t	t	c	c		c		c				c		c	c
t	3417893	c	3547073	s	t	CFT073	++	t	c	t	t	t	c	c		c	t	t	t	c		c		c				t		c	c
a	3417920	t	3547100	s	a	CFT073	++	a	t	a	a	a	a	t		t	a	a	a	t		t		t				a		t	t
g	3417926	a	3547106	s	g	CFT073	++	g	a	g	g	g	g	a		a	g	g	g	a		a		a				g		a	a
a	3417935	g	3547115	s	a	CFT073	++	a	a	a	a	a	a	g		g	a	a	a	g		g		a				a		g	g
c	3417955	t	3547135	ns	c	CFT073	++	c	c	c	c	c	c	t		c	c	c	c	c		c		c				c		t	t
g	3417956	a	3547136	ns	g	CFT073	++	g	g	g	g	g	g	a		a	g	g	g	a		a		g				g		a	a
t	3418004	c	3547184	s	t	CFT073	++	t	t	t	t	t	t	c		c	t	t	t	c		c		t				t		c	c
c	3418013	t	3547193	s	c	CFT073	++	c	t	c	c	c	t	t		t	c	c	t	t		t		t				t		t	t

Clone D base	Clone D site	CFT 073 base	CFT 073 site	mutation type[a]	Inferred ancestral base[b]	lineage inferred to mutate	Support level[c]	ED1a[d]	536	S88	APEC O1	UTI89	E2348/69	ATCC8739	K-12	HS	SMS3-5	IAI39	E24377A	SE11	55989	IAI1	Sakai	UMN026	B4 Sb227	F5 8401	SS Ss046	D1 Sd197	B18 BS512	F2a 2457T	F2a 301
g	3418046	a	3547226	s	g	CFT073	++	g	g	g	g	g	g	a		a	g	g	a	a		a		g				a		a	a
a	3418112	g	3547292	s	a	CFT073	++	a	a	a	a	a	a	g		g	a	a	g	g		g		a				g		g	g
c	3418124	t	3547304	s	c	CFT073	++	c	c	c	c	c	c	t		c	c	c	c	c		c		c				c		t	t
t	3418130	a	3547310	ns	t	CFT073	++	t	t	t	t	t	t	a		t	t	t	t	t		t		t				t		a	a
c	3418137	t	3547317	ns	c	CFT073	++	c	c	c	c	c	c	t		c	c	c	c	c		c		c				c		t	t
a	3418148	g	3547328	s	a	CFT073	++	a	a	a	a	a	a	g		a	a	a	a	a		a		a				a		g	g
g	3418154	a	3547334	s	g	CFT073	++	g	g	g	g	g	a	a		g	g	g	t	g		g		g				t		a	a
a	3418158	g	3547338	ns	a	CFT073	++	a	a	a	a	a	a	g		a	a	a	a	a		a		a				a		g	g
t	3418160	a	3547340	ns	t	CFT073	++	t	t	t	t	t	c	a		t	t	t	t	t		t		t				t		a	a
t	3418169	g	3547349	s	t	CFT073	++	t	t	t	t	t	t	g		t	t	t	t	t		t		t				t		g	g
t	3418182	c	3547362	s	t	CFT073	++	t	c	t	t	t	c	c		c	t	t	c	c		c		c				c		c	c
t	3418262	c	3547442	s	t	CFT073	++	t	c	t	t	t	c	c		c	t	t	c	c		c		c				c		c	c
c	3418280	t	3547460	s	c	CFT073	++	c	t	c	c	c	t	t		t	c	c	t	t		t		c				t		t	t
a	3418281	g	3547461	ns	a	CFT073	++	a	g	a	a	a	g	g		g	a	a	g	g		g		a				g		g	g
g	3418289	t	3547469	ns	g	CFT073	++	g	t	g	g	g	t	t		t	g	g	t	t		t		g				t		t	t
t	3418294	a	3547474	ns	a	clone D	++	a	a	a	a	a	a	a		a	a	a	a	a		a		a				a		a	a
t	3418307	g	3547487	s	t	CFT073	++	t	g	t	t	t	g	g		g	g	g	g	g		g		t				g		t	t
t	3418322	c	3547502	s	t	CFT073	++	t	t	t	t	t	t	c		t	t	t	c	t		t		c				c		t	t
a	3418400	c	3547580	s	a	CFT073	++	a	c	a	a	a	c	c		c	a	a	c	c		c		c				c		t	t
g	3418460	a	3547640	s	g	CFT073	++	g	g	g	g	g	g	a		a	g	g	a	a		a		a				a		a	a
c	3418466	t	3547646	s	c	CFT073	++	c	t	c	c	c	t	t		t	c	c	t	t		t		t				t		t	t
t	3418469	c	3547649	s	t	CFT073	++	t	c	t	t	t	c	c		t	t	t	t	t		t		t				c		c	c
c	3418482	g	3547662	ns	c	CFT073	++	c	g	c	c	c	c	g		g	c	c	g	g		g		g				g		g	g
t	3418491	g	3547671	ns	t	CFT073	++	t	t	t	t	t	t	g		t	t	t	t	t		t		t				t		g	g
a	3418514	g	3547694	s	g	clone D	++	g	g	g	g	g	g	g		g	g	g	g	g		g		g				g		g	g
c	3418520	t	3547700	s	c	CFT073	++	c	t	c	c	c	t	t		t	c	c	t	t		t		t				t		t	t
t	3418523	c	3547703	s	t	CFT073	++	t	c	t	t	t	c	c		c	t	t	c	c		c		c				c		c	c
g	3418529	a	3547709	s	g	CFT073	++	g	a	g	g	g	a	a		g	g	g	a	a		a		a				a		a	a

Clone D base	Clone D site	CFT 073 base	CFT 073 site	mutation type[a]	Inferred ancestral base[b]	lineage inferred to mutate	Support level[c]	ED1a[d]	536	S88	APEC O1	UTI89	E2348/69	ATCC8739	K-12	HS	SMS3-5	IAI39	E24377A	SE11	55989	IAI1	Sakai	UMN026	B4 Sb227	F5 8401	SS Ss046	D1 Sd197	B18 BS512	F2a 2457T	F2a 301
g	3418541	t	3547721	s	g	CFT073	++	g	t	g	g	g	t	t		t	g	g	t	t		t		t				t		t	t
t	3418550	c	3547730	s	t	CFT073	++	t	c	t	t	t	c	c		c	t	t	c	c		c		c				c		c	c
t	3418553	c	3547733	s	t	CFT073	++	t	c	t	t	t	c	c		c	t	t	c	c		c		c				c		c	c
c	3418562	t	3547742	s	c	CFT073	++	c	t	c	c	c	t	t		t	c	c	t	t		t		c				t		t	t
t	3418574	c	3547754	s	c	clone D	++	c	c	c	c	c	c	c		c	c	c	c	c		c		c				c		c	c
t	3418577	c	3547757	s	t	CFT073	++	t	c	t	t	t	c	c		c	t	t	c	c		c		c				c		c	c
t	3418946	a	3548126	ns	a	clone D	++	a	a	a	a	a	a	a		a	a	a	a	a	a	a		a						a	a
a	3419024	g	3548204	s	a	CFT073	++	a	g	a	a	a	a	g		g	g	g	g	g	g	g		g						g	g
a	3419075	c	3548255	s	c	clone D	++	c	c	c	c	c	c	c		c	c	c	c	c	c	c		c						c	c
t	3419138	c	3548318	s	t	CFT073	++	t	c	t	t	t	t	c		c	c	c	c	c	c	c		c						c	c
c	3419142	a	3548322	ns	c	CFT073	++	c	g	c	c	c	c	a		g	g	g	a	a	a	a		g						a	a
a	3419156	c	3548336	s	a	CFT073	++	a	a	a	a	a	a	c		a	a	a	a	c	c	c		a						c	c
c	3419174	t	3548354	s	c	CFT073	++	c	c	c	c	c	c	t		c	c	c	c	t	t	t		c						c	c
t	3419177	c	3548357	s	c	clone D	++	c	c	c	c	c	t	c		c	c	c	c	c	c	c		c						c	c
a	3419216	g	3548396	s	g	clone D	++	g	g	g	g	g	g	g		g	g	g	g	g	g	g		g						g	g
t	3419222	c	3548402	s	t	CFT073	++	t	c	t	t	t	t	c		c	t	c	c	c	c	c		t						c	c
a	3419226	g	3548406	ns	g	clone D	++	g	g	g	g	g	g	g		g	g	g	g	g	g	g		g						g	g
t	3419306	c	3548486	s	c	clone D	++	c	c	c	c	c	c	c		c	c	c	c	c	c	c		c						c	c
c	3419393	t	3548573	s	c	CFT073	++	c	t	c	c	c	c	t		t	t	t	t	t	t	t		t						t	t
g	3419402	a	3548582	s	g	CFT073	++	g	a	g	g	g	g	a		a	a	a	a	a	a	a		a						a	a
t	3419423	c	3548603	s	t	CFT073	++	t	c	t	t	t	t	c		c	c	c	c	c	c	c		c						c	c
c	3419426	t	3548606	s	c	CFT073	++	c	t	c	c	c	c	t		t	t	t	t	t	t	t		t						t	t
t	3419428	c	3548608	ns	t	CFT073	++	t	c	t	t	t	t	c		c	c	c	c	c	c	c		c						c	c
c	3419429	t	3548609	ns	c	CFT073	++	c	t	c	c	c	c	t		t	t	t	t	t	t	t		t						t	t
c	3419432	a	3548612	ns	c	CFT073	++	c	a	c	c	c	c	a		a	a	a	a	a	a	a		a						a	a
c	3419453	t	3548633	s	c	CFT073	++	c	t	c	c	c	c	t		t	t	t	t	t	t	t		t						t	t
t	3419486	c	3548666	s	t	CFT073	++	t	c	t	t	t	t	c		c	c	c	c	c	c	c		c						c	c
a	3419495	g	3548675	s	a	CFT073	++	a	g	a	a	a	a	g		g	g	g	g	g	g	g		g						g	g

Clone D base	Clone D site	CFT 073 base	CFT 073 site	mutation type[a]	Inferred ancestral base[b]	lineage inferred to mutate	Support level[c]	ED1a[d]	536	S88	APEC O1	UTI89	E2348/69	ATCC8739	K-12	HS	SMS3-5	IAI39	E24377A	SE11	55989	IAI1	Sakai	UMN026	B4 Sb227	F5 8401	SS Ss046	D1 Sd197	B18 BS512	F2a 2457T	F2a 301
c	3419498	t	3548678	s	c	CFT073	++	c	c	c	c	c	c	t		c	t	c	c	c	c	c		c						c	c
t	3419507	c	3548687	s	t	CFT073	++	t	c	t	t	t	t	c		t	c	c	c	c	c	c		c						c	c
c	3419529	t	3548709	s	c	CFT073	++	c	t	c	c	c	c	t		c	t	t	c	c	c	c		c						c	c
g	3419531	a	3548711	s	g	CFT073	++	g	a	g	g	g	g	a		g	a	a	g	g	g	g		g						g	g
a	3419534	g	3548714	s	a	CFT073	++	a	g	a	a	a	a	g		g	g	g	g	g	g	g		g						g	g
c	3419564	t	3548744	s	t	clone D	++	t	t	c	c	c	t	t		t	t	t	t	t	t	t		t						t	t
a	3419567	t	3548747	s	t	clone D	++	t	t	a	a	a	t	t		t	t	t	t	t	t	t		t						t	t
a	3419573	g	3548753	s	g	clone D	++	g	g	a	a	a	g	g		g	g	g	g	g	g	g		g						g	g
a	3419588	g	3548768	s	a	CFT073	++	a	g	a	a	a	a	g		g	g	g	g	g	g	g		g						g	g
t	3419629	c	3548809	ns	c	clone D	++	c	c	c	c	c	t	c		c	c	c	c	c	c	c		c						c	c
c	3419636	t	3548816	s	t	clone D	++	t	c	t	t	t	c	t		t	c	c	t	t	t	t		t						t	t
t	3419639	c	3548819	s	c	clone D	++	c	t	c	c	c	t	c		c	t	t	c	c	c	c		c						c	c
c	3419651	t	3548831	s	t	clone D	++	t	t	c	c	c	c	t		t	t	t	t	t	t	t		t						t	t
c	3419654	t	3548834	s	t	clone D	++	t	t	c	c	c	c	t		t	t	t	t	t	t	t		t						t	t
c	3419669	t	3548849	s	t	clone D	++	t	t	t	t	t	c	t		t	t	t	t	t	t	t		t						t	t
c	3419819	t	3548999	s	c	CFT073	++	c	c	c	c	c	c	t		c	c	c	t	c	t	c		c						t	t
t	3419826	a	3549006	ns	t	CFT073	++	t	t	t	t	t	a	a		a	a	a	a	a	a	a		t						a	a
c	3419861	t	3549041	s	c	CFT073	++	c	c	c	c	c	c	t		c	c	c	c	c	c	c		c						c	c
t	3419879	c	3549059	s	c	clone D	++	c	c	t	t	t	c	c		c	t	c	c	c	c	c		c						c	c
c	3419900	t	3549080	s	c	CFT073	++	c	t	c	c	c	c	t		t	t	t	t	t	t	t		t						t	t
a	3419901	g	3549081	ns	g	clone D	++	g	g	g	g	g	g	g		g	g	g	g	g	g	g		g						g	g
a	3419933	g	3549113	s	a	CFT073	++	a	g	a	a	a	a	g		a	a	a	a	a	a	a		a						a	a
a	3419957	c	3549137	ns	a	CFT073	++	a	c	a	a	a	a	c		a	a	a	a	a	a	a		a						a	a
t	3420032	a	3549212	s	t	CFT073	++	t	a	t	t	t	a	a		a	a	a	a	a	a	a		a						a	a
c	3420068	t	3549248	s	c	CFT073	++	c	c	c	c	c	c	t		c	c	c	t	c	c	c		c						c	c
a	3420115	t	3549295	ns	t	clone D	++	t	t	a	a	a	t	t		t	t	t	t	t	t	t		t						t	t
a	3420131	g	3549311	nc	a	CFT073	++	a	g	a	a	a	a	g		g	g	g	g	g	g	g		g						g	g
g	3420177	a	3549357	ns	g	CFT073	++	g	a	a	a	a	g	a		a	a	a	a	a	a	a		a						a	a

Clone D base	Clone D site	CFT 073 base	CFT 073 site	mutation type[a]	Inferred ancestral base[b]	lineage inferred to mutate	Support level[c]	ED1a[d]	536	S88	APEC O1	UTI89	E2348/69	ATCC8739	K-12	HS	SMS3-5	IAI39	E24377A	SE11	55989	IAI1	Sakai	UMN026	B4 Sb227	F5 8401	SS Ss046	D1 Sd197	B18 BS512	F2a 2457T	F2a 301
c	3420216	t	3549396	ns	c	CFT073	++	c	c	c	c	c	c	t		c	c	c	c	c	t	c		t						c	c
a	3420304	g	3549484	s	a	CFT073	++	a	g	a	a	a	a	g		g	g	g	g	g	g	g		g						g	g
g	3420316	a	3549496	s	g	CFT073	++	g	g	g	g	g	c	a		g	g	g	g	g	g	g		g						g	g
a	3420358	g	3549538	s	a	CFT073	++	a	g	a	a	a	a	g		g	g	g	a	g	g	g		a						a	a
a	3420397	g	3549577	s	a	CFT073	++	a	g	a	a	a	a	g		g	g	g	g	g	g	g		g						g	g
a	3420402	g	3549582	s	a	CFT073	++	a	g	a	a	a	a	g		g	g	g	g	g	a	g		g						g	g
c	3420514	g	3549694	s	c	CFT073	++	c	c	c	c	c	t	g		c	c	c	c	c	g	c		g						c	c
c	3420523	t	3549703	s	c	CFT073	++	c	c	c	c	c	c	t		c	c	c	t	c	c	c		t						t	t
t	3420540	c	3549720	ns	t	CFT073	++	t	c	t	t	t	t	c		c	c	c	t	c	t	c		t						t	t
t	3420555	c	3549735	ns	t	CFT073	++	t	c	c	c	c	t	c		c	c	c	c	c	t	c		c						c	c
a	3420573	t	3549753	ns	t	clone D	++	t	t	t	t	t	a	t		t	t	t	t	t	t	t		t						t	t
a	3420577	t	3549757	ns	a	CFT073	++	a	t	t	t	t	a	t		t	t	t	t	t	t	t		t						t	t
g	3420578	a	3549758	ns	g	CFT073	++	g	g	a	a	a	g	a		g	g	g	a	g	a	g		a						a	a
t	3420587	a	3549767	ns	a	clone D	++	a	a	a	a	a	t	a		a	a	a	a	a	a	a		a						a	a
a	3420649	g	3549829	s	a	CFT073	++	a	g	g	g	g	a	g		g	g	g	g	g	g	g		g						g	g
c	3420670	t	3549850	s	c	CFT073	++	c	t	c	c	c	c	t		t	t	t	t	t	t	t		t						t	t
a	3420700	g	3549880	s	g	clone D	++	g	g	g	g	g	a	g		g	g	g	g	g	g	g		g						g	g
g	3420706	a	3549886	s	g	CFT073	++	g	a	g	g	g	g	a		a	a	a	a	a	a	a		g						a	a
g	3420778	a	3549958	s	g	CFT073	++	g	g	g	g	g	a	a		g	g	g	a	g	g	g		a						a	a
a	3420790	c	3549970	s	a	CFT073	++	a	a	a	a	a	c	c		c	a	a	c	c	c	c		c						c	c
g	3420852	a	3550032	s	g	CFT073	++	g	g	g	g	g	g	a		g	g	g	a	g	a	g		g						a	a
c	3420865	t	3550045	s	c	CFT073	++	c	c	c	c	c	c	t		c	c	c	c	c	c	c		c						c	c
c	3420877	t	3550057	ns	c	CFT073	++	c	c	c	c	c	c	t		c	c	c	c	c	c	c		c						c	c
c	3420878	a	3550058	ns	a	clone D	++	a	a	a	a	a	a	a		a	a	a	a	a	a	a		a						a	a
g	3420915	a	3550095	s	g	CFT073	++	g	a	g	g	g	a	a		a	a	a	a	a	a	a		a						a	a
g	3420922	t	3550102	s	t	clone D	++	t	g	t	t	t	g	t		g	g	g	g	g	g	g		g						g	g
g	3420965	a	3550145	ns	g	CFT073	++	g	g	g	g	g	g	a		g	g	g	g	g	g	g		g						g	g
g	3421002	a	3550182	s	a	clone D	++	a	a	a	a	a	a	a		a	a	a	a	a	a	a		g						a	a

Clone D base	Clone D site	CFT 073 base	CFT 073 site	mutation type[a]	Inferred ancestral base[b]	lineage inferred to mutate	Support level[c]	ED1a[d]	536	S88	APEC O1	UTI89	E2348/69	ATCC8739	K-12	HS	SMS3-5	IAI39	E24377A	SE11	55989	IAI1	Sakai	UMN026	B4 Sb227	F5 8401	SS Ss046	D1 Sd197	B18 BS512	F2a 2457T	F2a 301
g	3421009	c	3550189	s	g	CFT073	++	g	c	g	g	g	c	c		c	c	c	c	c	c	c		c						c	c
a	3421012	g	3550192	s	a	CFT073	++	a	g	a	a	a	g	g		g	g	g	g	g	t	g		g						t	t
a	3421021	c	3550201	s	c	clone D	++	c	c	c	c	c	c	c		c	c	c	c	c	a	c		c						a	a
c	3421042	t	3550222	s	c	CFT073	++	c	c	c	c	c	c	t		c	t	c	c	c	c	c		c						c	c
c	3421078	t	3550258	s	c	CFT073	++	c	c	c	c	c	c	t		t	c	c	c	t	t	t		c						t	t
t	3421093	c	3550273	s	t	CFT073	++	t	c	t	t	t	c	c		c	c	c	c	c	c	c		c						c	c
a	3421120	g	3550300	s	a	CFT073	++	a	g	a	a	a	g	g		g	g	g	g	g	g	g		g						g	g
g	3421207	a	3550387	s	g	CFT073	++	g	a	g	g	g	a	a		a	a	a	a	a	a	a		a						a	a
a	3421225	g	3550405	s	a	CFT073	++	a	a	a	a	a	a	g		a	a	a	a	a	a	a		g						a	a
g	3421244	a	3550424	ns	g	CFT073	++	g	a	g	g	g	a	a		a	a	a	a	a	a	a		a						a	a
g	3421260	a	3550440	ns	g	CFT073	++	g	a	g	g	g	a	a		a	a	a	a	a	a	a		a						a	a
t	3421271	g	3550451	ns	g	clone D	++	g	g	g	g	g	g	g		g	g	g	g	g	g	g		g						g	g
a	3421286	g	3550466	ns	a	CFT073	++	a	g	a	a	a	g	g		g	g	g	g	g	g	g		g						g	g
c	3421311	t	3550491	ns	c	CFT073	++	c	t	c	c	c	t	c		c	t	t	t	c	t	c		t						c	c
g	3421374	a	3550554	s	g	CFT073	++	g	g	g	g	g	g	a		g	g	g	g	g	g	g		g						g	g
c	3421394	t	3550574	ns	c	CFT073	++	c	c	c	c	c	c	t		c	c	c	t	t	t	t		c						t	t
g	3421415	a	3550595	s	g	CFT073	++	g	a	g	g	g	a	a		a	a	a	a	a	a	a		a						a	a
g	3421418	a	3550598	s	g	CFT073	++	g	a	g	g	g	a	a		a	a	a	a	a	a	a		a						a	a
c	3421434	t	3550614	s	c	CFT073	++	c	t	c	c	c	t	t		t	t	t	t	t	t	t		t						t	t
t	3421488	g	3550668	s	t	CFT073	++	t	g	t	t	t	t	g		g	t	t	t	g	t	g		t						g	g
t	3421506	c	3550686	s	t	CFT073	++	t	t	t	t	t	t	c		t	t	t	t	t	t	t		t						t	t
t	3421548	c	3550728	s	t	CFT073	++	t	c	t	t	t	c	c		c	c	c	c	c	c	c		c						c	c
t	3421569	c	3550749	s	t	CFT073	++	t	t	t	t	t	t	c		t	t	t	c	t	t	t		t						c	c
g	3421608	c	3550788	s	g	CFT073	++	g	c	g	g	g	c	c		c	c	c	c	c	c	c		c						c	c
c	3421614	t	3550794	s	c	CFT073	++	c	c	c	c	c	t	t		t	t	t	t	t	t	t		t						t	t
g	3421647	a	3550827	s	g	CFT073	++	g	g	g	g	g	g	a		g	g	g	g	g	g	g		g						g	g
g	3421667	t	3550847	ns	g	CFT073	++	g	g	g	g	g	g	t		g	g	g	g	g	g	g		g						g	g
g	3421677	a	3550857	s	g	CFT073	++	g	g	g	g	g	g	a		g	g	g	a	a	a	a		g						a	a

Clone D base	Clone D site	CFT 073 base	CFT 073 site	mutation type[a]	Inferred ancestral base[b]	lineage inferred to mutate	Support level[c]	ED1a[d]	536	S88	APEC O1	UTI89	E2348/69	ATCC8739	K-12	HS	SMS3-5	IAI39	E24377A	SE11	55989	IAI1	Sakai	UMN026	B4 Sb227	F5 8401	SS Ss046	D1 Sd197	B18 BS512	F2a 2457T	F2a 301
a	3421680	g	3550860	s	a	CFT073	++	a	c	a	a	a	c	g		g	c	c	g	g	g	g		c						g	g
a	3421686	g	3550866	s	a	CFT073	++	a	g	a	a	a	a	g		g	a	a	g	g	g	g		a						g	g
a	3421878	g	3551058	s	a	CFT073	++	a	g	a	a	a	g	g		g	g	g	g	g	g	g		g						g	g
t	3421902	c	3551082	s	t	CFT073	++	t	c	t	t	t	c	c		c	c	c	c	c	c	c		c						c	c
t	3421905	c	3551085	s	t	CFT073	++	t	t	t	t	t	t	c		c	t	t	c	c	c	c		t						c	c
g	3421938	c	3551118	s	g	CFT073	++	g	c	g	g	g	c	c		c	c	c	c	c	c	c		c						c	c
a	3421979	g	3551159	s	g	clone D	++	g	g	g	g	g	g	g		g	g	g	g	g	g	g		g						g	g
a	3422013	g	3551193	s	a	CFT073	++	a	g	a	a	a	g	g		g	g	g	g	g	g	g		g						g	g
g	3422037	a	3551217	s	g	CFT073	++	g	a	g	g	g	a	a		a	a	a	a	a	a	a		a						a	a
t	3422070	c	3551250	s	t	CFT073	++	t	c	t	t	t	c	c		c	c	c	c	c	c	c		c						c	c
a	3422094	c	3551274	ns	c	clone D	++	c	c	c	c	c	c	c		c	c	c	c	c	c	c		c						c	c
g	3422095	a	3551275	ns	g	CFT073	++	g	a	g	g	g	a	a		a	a	a	a	a	a	a		a						a	a
t	3422100	c	3551280	s	c	clone D	++	c	c	c	c	c	c	c		c	c	c	c	c	c	c		c						c	c
t	3422103	a	3551283	s	t	CFT073	++	t	a	t	t	t	t	a		a	a	a	a	a	a	a		t						a	a
g	3422117	a	3551297	s	g	CFT073	++	g	a	g	g	g	a	a		a	a	a	a	a	a	a		a						a	a
c	3422136	t	3551316	s	c	CFT073	++	c	c	c	c	c	c	t		c	c	c	c	c	c	c		c						t	t
g	3422310	a	3551490	nc	g	CFT073	++	g	a	g	g	g	a	a		a	a	a	a	a	a	a		g						a	a
a	3422332	g	3551512	nc	a	CFT073	++	a	a	a	a	a	a	g		g	a	a	a	a	a	a		a						g	g
g	3422363	a	3551543	s	g	CFT073	++	g	g	g	g	g	g	a		a	g	g	g	g	g	g		g						a	a
t	3422391	c	3551571	s	t	CFT073	++	t	c	t	t	t	c	c		c	c	c	c	c	c	c		c						c	c
c	3422422	t	3551602	ns	c	CFT073	++	c	t	c	c	c	t	t		t	t	t	t	t	t	t		t						t	t
a	3422438	g	3551618	s	a	CFT073	++	a	g	a	a	a	g	g		g	g	g	g	g	g	g		g						g	g
t	3422439	c	3551619	s	t	CFT073	++	t	c	t	t	t	c	c		c	c	c	c	c	c	c		c						c	c
t	3422469	g	3551649	ns	t	CFT073	++	t	c	t	t	t	g	g		g	c	c	g	g	g	g		g						g	g
t	3422470	c	3551650	ns	t	CFT073	++	t	c	t	t	t	t	c		c	c	c	c	c	c	c		t						c	c
a	3422488	t	3551668	ns	a	CFT073	++	a	a	a	a	a	t	t		t	a	a	t	t	t	t		t						t	t
a	3422490	t	3551670	s	a	CFT073	++	a	t	a	a	a	t	t		t	t	t	t	t	t	t		t						t	t
g	3422556	a	3551736	s	g	CFT073	++	g	g	g	g	g	g	a		a	g	g	a	a	a	a		g						a	a

Clone D base	Clone D site	CFT 073 base	CFT 073 site	mutation type[a]	Inferred ancestral base[b]	lineage inferred to mutate	Support level[c]	ED1a[d]	536	S88	APEC O1	UTI89	E2348/69	ATCC8739	K-12	HS	SMS3-5	IAI39	E24377A	SE11	55989	IAI1	Sakai	UMN026	B4 Sb227	F5 8401	SS Ss046	D1 Sd197	B18 BS512	F2a 2457T	F2a 301
a	3422601	g	3551781	s	a	CFT073	++	a	g	a	a	a	g	g		g	g	g	g	g	g	g		g						g	g
a	3422627	g	3551807	ns	a	CFT073	++	a	g	a	a	a	g	g		g	g	g	g	g	g	g		g						g	g
a	3422679	c	3551859	s	a	CFT073	++	a	c	a	a	a	c	c		c	c	c	c	c	c	c		c						c	c
t	3422688	c	3551868	s	t	CFT073	++	t	c	t	t	t	c	c		c	c	c	c	c	c	c		c						c	c
t	3422723	c	3551903	ns	t	CFT073	++	t	c	t	t	t	c	c		c	c	c	c	c	c	c		c						c	c
c	3422724	t	3551904	s	c	CFT073	++	c	c	c	c	c	c	t		c	c	c	t	t	t	t		c						t	t
a	3422808	g	3551988	s	a	CFT073	++	a	g	a	a	a	g	g		g	g	g	g	g	g	g		g						g	g
g	3422842	t	3552022	ns	g	CFT073	++	g	t	g	g	g	t	t		t	t	t	t	t	t	t		t						t	t
c	3422843	g	3552023	ns	c	CFT073	++	c	g	c	c	c	g	g		g	g	g	g	g	g	g		g						g	g
g	3422845	a	3552025	ns	g	CFT073	++	g	a	g	g	g	a	a		a	a	a	a	a	a	a		a						a	a
g	3422847	a	3552027	s	g	CFT073	++	g	a	g	g	g	a	a		a	a	a	a	a	a	a		a						a	a
t	3422850	c	3552030	s	t	CFT073	++	t	c	t	t	t	c	c		c	c	c	c	c	c	c		c						c	c
c	3422853	g	3552033	s	c	CFT073	++	c	g	c	c	c	g	g		g	g	g	g	g	g	g		g						g	g
t	3422862	c	3552042	ns	t	CFT073	++	t	c	t	t	t	c	c		c	c	c	c	c	c	c		c						c	c
g	3422863	a	3552043	ns	g	CFT073	++	g	a	g	g	g	a	a		a	a	a	a	a	a	a		a						a	a
c	3422864	t	3552044	ns	c	CFT073	++	c	t	c	c	c	t	t		t	t	t	t	t	t	t		t						t	t
g	3422868	a	3552048	s	g	CFT073	++	g	a	g	g	g	a	a		a	a	a	a	a	a	a		a						a	a
g	3422873	a	3552053	s	g	CFT073	++	g	g	g	g	g	g	a		g	g	g	g	a	g	a		g						a	a
a	3422874	c	3552054	s	a	CFT073	++	a	c	a	a	a	c	c		c	c	c	c	c	c	c		c						c	c
g	3422877	a	3552057	ns	g	CFT073	++	g	a	g	g	g	a	a		a	a	a	a	a	a	a		a						a	a
c	3422879	t	3552059	ns	c	CFT073	++	c	t	c	c	c	t	t		t	t	t	t	t	t	t		t						t	t
c	3422884	a	3552064	ns	c	CFT073	++	c	a	c	c	c	a	a		a	a	a	a	a	a	a		a						a	a
g	3422894	a	3552074	ns	g	CFT073	++	g	a	g	g	g	a	a		a	a	a	a	a	a	a		a						a	a
t	3422898	c	3552078	s	t	CFT073	++	t	c	t	t	t	c	c		c	c	c	c	c	c	c		c						c	c
a	3422909	c	3552089	ns	a	CFT073	++	a	t	a	a	a	t	c		t	t	t	t	c	t	c		t						c	c
t	3422915	c	3552095	s	t	CFT073	++	t	c	t	t	t	c	c		c	a	c	c	c	c	c		c						c	c
g	3422918	a	3552098	s	g	CFT073	++	g	a	g	g	g	a	a		a	a	a	a	a	a	a		a						a	a
a	3422930	c	3552110	s	a	CFT073	++	a	c	a	a	a	c	c		c	c	c	c	c	c	c		c						c	c

Clone D base	Clone D site	CFT 073 base	CFT 073 site	mutation type[a]	Inferred ancestral base[b]	lineage inferred to mutate	Support level[c]	ED1a[d]	536	S88	APEC O1	UTI89	E2348/69	ATCC8739	K-12	HS	SMS3-5	IAI39	E24377A	SE11	55989	IAI1	Sakai	UMN026	B4 Sb227	F5 8401	SS Ss046	D1 Sd197	B18 BS512	F2a 2457T	F2a 301
t	3422936	c	3552116	s	t	CFT073	++	t	c	t	t	t	t	c		c	t	c	c	c	c	c		t						c	c
a	3422942	g	3552122	s	a	CFT073	++	a	g	a	a	a	g	g		g	g	g	g	g	g	g		g						g	g
g	3422948	a	3552128	s	g	CFT073	++	g	g	g	g	g	g	a		g	a	g	g	a	a	a		a						a	a
t	3422984	c	3552164	s	t	CFT073	++	t	c	t	t	t	c	c		c	c	c	c	c	c	c		c						c	c
c	3422988	t	3552168	ns	c	CFT073	++	c	t	c	c	c	t	t		t	t	t	t	t	t	t		t						t	t
a	3422990	g	3552170	s	a	CFT073	++	a	g	a	a	a	g	g		g	g	g	g	g	g	g		g						g	g
a	3422996	t	3552176	ns	a	CFT073	++	a	t	a	a	a	t	t		t	t	t	t	t	t	t		t						t	t
c	3423026	t	3552206	s	c	CFT073	++	c	t	c	c	c	t	t		t	t	t	t	t	t	t		t						t	t
c	3423032	t	3552212	s	c	CFT073	++	c	c	c	c	c	c	t		c	c	c	t	t	t	t		c						t	t
a	3423041	g	3552221	s	a	CFT073	++	a	g	a	a	a	g	g		g	g	g	g	g	g	g		g						g	g
a	3423059	g	3552239	s	a	CFT073	++	a	g	a	a	a	g	g		g	g	g	g	g	g	g		g						g	g
a	3423062	g	3552242	s	a	CFT073	++	a	g	a	a	a	g	g		a	g	g	a	a	a	a		g						a	a
a	3423068	t	3552248	s	a	CFT073	++	a	a	a	a	a	a	t		a	a	a	a	a	a	a		a						a	a
a	3423072	t	3552252	ns	a	CFT073	++	a	t	a	a	a	t	t		t	t	t	t	t	t	t		t						t	t
g	3423167	a	3552347	s	g	CFT073	++	g	g	g	g	g	g	a		g	g	g	g	g	g	g		g						g	g
a	3423170	g	3552350	s	a	CFT073	++	a	g	a	a	a	a	g		g	g	g	g	g	g	g		a						g	g
t	3423177	c	3552357	ns	t	CFT073	++	t	c	t	t	t	t	c		c	c	c	c	c	c	c		c						c	c
a	3423185	g	3552365	s	a	CFT073	++	a	g	a	a	a	a	g		g	g	g	g	g	g	g		g						g	g
a	3423204	g	3552384	ns	a	CFT073	++	a	g	a	a	a	a	g		g	g	g	g	g	g	g		g						g	g
g	3423206	a	3552386	ns	g	CFT073	++	g	a	g	g	g	g	a		a	a	a	a	a	a	a		a						a	a
a	3423208	g	3552388	ns	a	CFT073	++	a	g	a	a	a	a	g		g	g	g	g	g	g	g		g						g	g
c	3423218	t	3552398	s	c	CFT073	++	c	t	c	c	c	c	t		t	t	t	t	t	t	t		t						t	t
g	3423221	c	3552401	s	g	CFT073	++	g	c	g	g	g	g	c		c	c	c	c	c	c	c		c						c	c
g	3423224	a	3552404	s	g	CFT073	++	g	a	g	g	g	g	a		a	a	a	a	a	a	a		a						a	a
g	3423230	a	3552410	s	g	CFT073	++	g	a	g	g	g	g	a		a	a	a	a	a	a	a		a						a	a
c	3423238	t	3552418	ns	c	CFT073	++	c	c	c	c	c	c	t		c	c	c	t	t	t	t		c						t	t
t	3423240	c	3552420	ns	t	CFT073	++	t	c	t	t	t	t	c		c	c	c	c	c	c	c		c						c	c
c	3423242	t	3552422	s	c	CFT073	++	c	t	c	c	c	c	t		t	t	t	t	t	t	t		t						t	t

Clone D base	Clone D site	CFT 073 base	CFT 073 site	mutation type[a]	Inferred ancestral base[b]	lineage inferred to mutate	Support level[c]	ED1a[d]	536	S88	APEC O1	UTI89	E2348/69	ATCC8739	K-12	HS	SMS3-5	IAI39	E24377A	SE11	55989	IAI1	Sakai	UMN026	B4 Sb227	F5 8401	SS Ss046	D1 Sd197	B18 BS512	F2a 2457T	F2a 301
g	3423246	c	3552426	ns	g	CFT073	++	g	a	g	g	g	g	c		c	c	c	c	c	c	c		c						c	c
c	3423266	t	3552446	s	c	CFT073	++	c	t	c	c	c	c	t		t	t	t	t	t	t	t		t						t	t
g	3423283	a	3552463	s	g	CFT073	++	g	a	g	g	g	g	a		a	a	a	a	a	a	a		a						a	a
c	3423285	g	3552465	ns	c	CFT073	++	c	g	c	c	c	c	g		g	g	g	g	g	g	g		g						g	g
a	3423293	c	3552473	s	a	CFT073	++	a	c	a	a	a	a	c		c	c	c	c	c	c	c		c						c	c
g	3423296	a	3552476	s	g	CFT073	++	g	a	g	g	g	g	a		a	a	a	a	a	a	a		a						a	a
c	3423302	t	3552482	s	c	CFT073	++	c	t	c	c	c	c	t		t	t	t	t	t	t	t		t						t	t
c	3423310	t	3552490	ns	c	CFT073	++	c	c	c	c	c	c	t		t	c	c	t	t	t	t		c						t	t
a	3423311	c	3552491	s	a	CFT073	++	a	a	a	a	a	a	c		c	c	c	c	c	c	c		c						c	c
a	3423323	g	3552503	s	a	CFT073	++	a	g	a	a	a	a	g		g	g	g	g	g	g	g		g						g	g
a	3423330	t	3552510	ns	a	CFT073	++	a	t	a	a	a	a	t		t	t	t	t	t	t	t		t						t	t
a	3423365	g	3552545	s	a	CFT073	++	a	g	a	a	a	a	g		g	g	g	g	g	g	g		g						g	g
t	3423375	c	3552555	ns	t	CFT073	++	t	c	t	t	t	t	c		c	c	c	c	c	c	c		c						c	c
a	3423380	c	3552560	s	a	CFT073	++	a	c	a	a	a	a	c		c	c	c	c	c	c	c		c						c	c
a	3423455	g	3552635	s	a	CFT073	++	a	g	a	a	a	a	g		g	g	g	g	g	g	g		a						g	g
c	3423485	t	3552665	s	c	CFT073	++	c	t	c	c	c	c	t		t	t	t	t	t	t	t		c						t	t
g	3423497	a	3552677	s	g	CFT073	++	g	a	g	g	g	g	a		a	a	a	a	a	a	a		g						a	a
t	3423500	a	3552680	ns	t	CFT073	++	t	a	t	t	t	t	a		a	a	a	a	a	a	a		t						a	a
t	3423502	c	3552682	ns	t	CFT073	++	t	c	t	t	t	t	c		c	c	c	c	c	c	c		t						c	c
c	3423503	a	3552683	s	c	CFT073	++	c	a	c	c	c	c	a		a	a	a	a	a	a	a		c						a	a
c	3423521	g	3552701	s	c	CFT073	++	c	g	c	c	c	c	g		g	g	g	g	g	g	g		c						g	g
c	3423524	t	3552704	s	c	CFT073	++	c	c	c	c	c	c	t		t	c	c	t	t	t	t		c						t	t
g	3423533	a	3552713	ns	g	CFT073	++	g	t	g	g	g	g	a		a	t	t	a	a	a	a		g						a	a
t	3423535	c	3552715	ns	t	CFT073	++	t	t	t	t	t	t	c		c	t	t	c	c	c	c		t						c	c
t	3423559	c	3552739	ns	t	CFT073	++	t	t	t	t	t	t	c		c	t	t	c	t	c	t		t						c	c
a	3423563	g	3552743	s	a	CFT073	++	a	g	a	a	a	a	g		g	g	g	g	g	g	g		a						g	g
c	3423568	t	3552748	ns	c	CFT073	++	c	t	c	c	c	c	t		t	t	t	t	t	t	t		c						t	t
c	3423569	g	3552749	s	c	CFT073	++	c	g	c	c	c	c	g		g	g	g	g	g	g	g		c						g	g

Clone D base	Clone D site	CFT 073 base	CFT 073 site	mutation type[a]	Inferred ancestral base[b]	lineage inferred to mutate	Support level[c]	ED1a[d]	536	S88	APEC O1	UTI89	E2348/69	ATCC8739	K-12	HS	SMS3-5	IAI39	E24377A	SE11	55989	IAI1	Sakai	UMN026	B4 Sb227	F5 8401	SS Ss046	D1 Sd197	B18 BS512	F2a 2457T	F2a 301
t	3423580	g	3552760	ns	t	CFT073	++	t	g	t	t	t	t	g		g	g	g	g	g	g	g		t						g	g
g	3423605	a	3552785	s	g	CFT073	++	g	g	g	g	g	g	a		a	g	g	a	a	a	a		g						a	a
g	3423632	a	3552812	s	g	CFT073	++	g	g	g	g	g	g	a		a	g	g	a	a	a	a		g						a	a
g	3423650	a	3552830	s	g	CFT073	++	g	g	g	g	g	g	a		g	g	g	a	a	a	a		g						a	a
a	3423665	g	3552845	s	a	CFT073	++	a	g	a	a	a	a	g		g	g	g	g	g	g	g		g						g	g
t	3423750	c	3552930	ns	t	CFT073	++	t	c	t	t	t	t	c		c	c	c	c	c	c	c		c			c			c	c
a	3423753	c	3552933	ns	a	CFT073	++	a	c	a	a	a	a	c		c	c	c	c	c	c	c		c			c			c	c
c	3423796	t	3552976	s	c	CFT073	++	c	t	c	c	c	c	t		t	t	c	t	t	t	t		c			t			t	t
g	3423804	a	3552984	s	g	CFT073	++	g	g	g	g	g	g	a		a	g	a	a	a	a	a		g			a			a	a
a	3423812	g	3552992	ns	a	CFT073	++	a	a	a	a	a	a	g		a	a	a	g	g	g	g		a			a			g	g
a	3423817	g	3552997	s	a	CFT073	++	a	a	a	a	a	a	g		a	a	a	g	g	g	g		a			a			g	g
t	3423847	c	3553027	s	t	CFT073	++	t	c	t	t	t	t	c		t	c	c	c	c	c	c		t			c			c	c
a	3423850	t	3553030	s	a	CFT073	++	a	t	a	a	a	a	t		a	t	t	t	t	t	t		a			t			t	t
c	3423853	t	3553033	s	c	CFT073	++	c	t	c	c	c	c	t		c	t	t	t	t	t	t		c			t			t	t
g	3423862	a	3553042	s	g	CFT073	++	g	g	g	g	g	g	a		g	g	g	a	a	a	a		g			a			a	a
t	3423973	a	3553153	s	t	CFT073	++	t	t	t	t	t	t	a		c	a	t	a	a	a	a		t			c			c	c
g	3424141	a	3553321	s	g	CFT073	++	g	g	g	g	g	g	a	a	g	g	g	a	a	a	a		g			a			a	a
a	3424160	g	3553340	ns	g	clone D	++	g	g	g	g	g	g	g	g	g	g	g	g	g	g	g		g			g			g	g
t	3424168	c	3553348	s	t	CFT073	++	t	c	t	t	t	t	c	c	c	c	c	c	c	c	c		c			c			c	c
a	3424192	g	3553372	s	a	CFT073	++	a	g	a	a	a	a	g	g	g	g	g	g	g	g	g		g			g			g	g
g	3424204	a	3553384	s	g	CFT073	++	g	g	g	g	g	g	a	a	a	g	g	a	a	a	a		a			a			a	a
t	3424210	c	3553390	s	t	CFT073	++	t	t	t	t	t	t	c	c	t	t	t	c	c	c	c		t			c			c	c
a	3424234	g	3553414	s	a	CFT073	++	a	g	a	a	a	a	g	g	g	g	g	g	g	g	g		g			g			g	g
a	3424270	g	3553450	s	a	CFT073	++	a	g	g	g	g	a	g	g	g	g	g	g	g	g	g		g			g			g	g
g	3424383	a	3553563	ns	g	CFT073	++	g	g	g	g	g	g	g	a	g	g	g	g	g	g	g		g			g			g	g
t	3424431	g	3553611	s	t	CFT073	++	t	t	t	t	t	t	g	g	t	t	t	t	t	t	t		t			t			t	t
t	3424539	c	3553719	ns	c	clone D	++	c	c	c	c	c	c	c	c	c	c	c	c	c	c	c		c			c			c	c
t	3424579	c	3553759	s	c	clone D	++	c	t	c	c	c	c	c	c	c	t	t	c	c	c	c		c			c			c	c

Clone D base	Clone D site	CFT 073 base	CFT 073 site	mutation type[a]	Inferred ancestral base[b]	lineage inferred to mutate	Support level[c]	ED1a[d]	536	S88	APEC O1	UTI89	E2348/69	ATCC8739	K-12	HS	SMS3-5	IAI39	E24377A	SE11	55989	IAI1	Sakai	UMN026	B4 Sb227	F5 8401	SS Ss046	D1 Sd197	B18 BS512	F2a 2457T	F2a 301
g	3424596	a	3553776	s	a	clone D	++	a	g	a	a	a	a	a	a	a	g	g	a	a	a	a		a			a			a	a
c	3424669	g	3553849	s	g	clone D	++	g	c	g	g	g	g	g	g	c	c	c	c	c	c	c		g			c			c	c
g	3424687	a	3553867	s	g	CFT073	++	g	g	g	g	g	g	a	a	g	g	g	g	g	g	g		g			g			g	g
a	3424720	g	3553900	s	g	clone D	++	g	a	g	g	g	g	g	g	g	a	a	g	g	g	g		g			g			g	g
a	3424723	t	3553903	s	t	clone D	++	t	a	t	t	t	t	t	t	t	a	a	t	t	t	t		t			t			t	t
g	3424726	c	3553906	s	c	clone D	++	c	g	c	c	c	c	c	c	c	g	g	c	c	c	c		c			c			c	c
g	3424741	a	3553921	s	g	CFT073	++	g	g	g	g	g	g	a	a	g	g	g	g	g	g	g		g			g			g	g
c	3424858	t	3554038	s	c	CFT073	++	c	c	c	c	c	t	t	t	c	c	c	t	c	t	c		c			c			c	c
t	3424894	c	3554074	s	c	clone D	++	c	c	c	c	c	t	c	c	c	c	c	c	c	c	c		c			c			c	c
t	3424915	c	3554095	s	t	CFT073	++	t	c	t	t	t	t	c	c	c	c	c	c	c	c	c		c			c			c	c
c	3424975	a	3554155	s	c	CFT073	++	c	a	c	c	c	c	a	a	c	a	a	a	c	a	c		a			c			c	c
a	3424984	g	3554164	s	a	CFT073	++	a	a	a	a	a	a	g	g	g	a	a	g	g	g	g		a			g			g	g
a	3424993	g	3554173	s	a	CFT073	++	a	g	a	a	a	a	g	g	g	g	g	g	g	g	g		g			g			g	g
c	3425197	t	3554377	s	c	CFT073	++	c	t	c	c	c	c	t	t	t	t	t	t	t	t	t		t			t			t	t
a	3425206	g	3554386	s	a	CFT073	++	a	a	a	a	a	a	g	g	g	a	g	g	g	g	g		g			g			g	g
g	3425255	t	3554435	ns	g	CFT073	++	g	g	g	g	g	g	t	t	g	g	g	g	g	g	g		g			t			g	g
a	3425263	g	3554443	s	g	clone D	++	g	g	a	a	a	g	g	g	a	g	g	g	g	g	g		g			g			g	g
a	3425323	c	3554503	ns	a	CFT073	++	a	a	a	a	a	a	c	c	c	a	a	c	c	c	c		a			c			a	a
c	3425335	g	3554515	s	c	CFT073	++	c	c	c	c	c	c	g	g	c	c	c	g	g	g	g		c			g			c	c
a	3425340	g	3554520	s	a	CFT073	++	a	a	a	a	a	a	g	g	a	a	a	g	g	g	g		a			g			a	a
g	3425347	a	3554527	s	g	CFT073	++	g	g	g	g	g	g	a	a	g	g	g	a	a	a	a		g			a			g	g
a	3425356	c	3554536	s	a	CFT073	++	a	a	a	a	a	a	c	c	a	a	a	c	c	c	c		a			c			a	a
a	3425359	g	3554539	s	a	CFT073	++	a	a	a	a	a	a	g	g	a	a	a	g	g	g	g		a			g			a	a
g	3425362	a	3554542	s	g	CFT073	++	g	a	g	g	g	g	a	a	g	g	g	a	a	a	a		g			a			g	g
t	3425376	g	3554556	ns	t	CFT073	++	t	g	t	t	t	g	g	g	g	g	t	g	g	g	g		g			g			t	t
a	3425395	g	3554575	s	a	CFT073	++	a	g	a	a	a	g	g	g	g	g	g	g	g	g	g		g			g			g	g
c	3425411	a	3554591	ns	a	clone D	++	a	a	c	c	c	a	a	a	a	a	a	a	a	a	a		a			a			a	a
g	3425455	a	3554635	s	g	CFT073	++	g	g	g	g	g	g	a	a	g	g	g	a	g	a	g		g			g			g	g

Clone D base	Clone D site	CFT 073 base	CFT 073 site	mutation type[a]	Inferred ancestral base[b]	lineage inferred to mutate	Support level[c]	ED1a[d]	536	S88	APEC O1	UTI89	E2348/69	ATCC8739	K-12	HS	SMS3-5	IAI39	E24377A	SE11	55989	IAI1	Sakai	UMN026	B4 Sb227	F5 8401	SS Ss046	D1 Sd197	B18 BS512	F2a 2457T	F2a 301
c	3425542	t	3554722	s	c	CFT073	++	c	t	c	c	c	c	t	t	t	t	t	t	c	t	c		t			t			t	t
g	3425656	c	3554836	s	g	CFT073	++	g	c	g	g	g	c	c	c	c	g	g	c	c	c	c		g			c			c	c
a	3425665	g	3554845	s	a	CFT073	++	a	g	a	a	a	g	g	g	g	g	g	g	g	g	g		g			g			g	g
a	3425770	g	3554950	ns	a	CFT073	++	a	g	a	a	a	a	g	g	g	g	g	g	g	g	g		g			g			g	g
t	3425771	g	3554951	ns	t	CFT073	++	t	g	t	t	t	g	g	g	g	g	g	g	g	g	g		g			g			g	g
t	3425773	g	3554953	s	t	CFT073	++	t	g	t	t	t	g	g	g	g	g	g	g	g	g	g		g			g			g	g
c	3425857	a	3555037	ns	c	CFT073	++	c	a	c	c	c	c	a	a	a	a	a	a	a	a	a		a			a			a	a
t	3425869	a	3555049	s	t	CFT073	++	t	t	t	t	t	t	a	a	t	t	t	a	t	a	t		t			t			t	t
t	3425892	a	3555072	ns	t	CFT073	++	t	a	t	t	t	a	a	a	a	a	a	a	a	a	a		a			a			a	a
t	3425972	g	3555152	s	t	CFT073	++	t	t	t	t	t	t	g	g	t	t	t	g	t	g	t		t			t			t	t
t	3425975	g	3555155	ns	t	CFT073	++	t	t	t	t	t	t	g	g	t	t	t	g	t	g	t		t			t			t	t
c	3425978	t	3555158	s	c	CFT073	++	c	c	c	c	c	c	t	t	c	c	c	t	c	t	c		c			c			c	c
a	3425981	c	3555161	s	a	CFT073	++	a	a	a	a	a	a	c	c	a	a	a	c	a	c	a		a			a			a	a
a	3426122	g	3555302	s	a	CFT073	++	a	g	a	a	a	g	g	g	g	g	g	g	g	g	g		g			g			g	g
a	3426134	t	3555314	s	?	?	+/-	c	c	c	c	c	c	t	t	c	c	t	t	c	t	c		c			t			c	c
a	3426161	g	3555341	s	a	CFT073	++	a	g	a	a	a	g	g	g	g	g	g	g	g	g	g		g			g			g	g
g	3426230	a	3555410	s	g	CFT073	++	g	a	g	g	g	a	a	a	a	a	a	a	a	a	a		a			a			a	a
a	3426233	c	3555413	s	a	CFT073	++	a	a	a	a	a	a	c	c	c	a	a	a	c	a	c		a			a			a	a
t	3426276	a	3555456	ns	t	CFT073	++	t	t	t	t	t	t	a	a	a	t	t	a	a	a	a		t			a			t	t
t	3426288	c	3555468	ns	t	CFT073	++	t	t	t	t	t	c	c	c	c	c	c	c	c	c	c		c			c			c	c
c	3426350	t	3555530	s	c	CFT073	++	c	c	c	c	c	c	t	t	t	c	c	a	c	a	c		c			a			c	c
a	3426412	g	3555592	s	a	CFT073	++	a	g	a	a	a	a	g	g	g	g	g	a	g	a	g		g			g			g	g
t	3426443	g	3555623	s	t	CFT073	++	t	g	t	t	t	g	g	g	g	g	g	g	g	g	g		g			g			g	g
a	3426514	c	3555694	ns	a	CFT073	++	a	c	a	a	a	a	c	c	c	c	c	c	c	c	c		c			c			c	c
c	3426646	t	3555826	nc	c	CFT073	++	c	c	c	c	c	c	t	t	t	c	c	t	t	t	t		c			t			t	t
t	3426949	c	3556129	s	c	clone D	++	c	c	c	c	c	c	c	c	c	c	c	c	c	c	c		c			c	c		c	c
g	3427051	t	3556231	s	g	CFT073	++	g	g	g	g	g	g	g	g	g	g	g	g	g	g	g		g			g	g		g	g
c	3427081	t	3556261	s	t	clone D	++	t	t	t	t	t	c	t	t	t	t	t	t	t	t	t		t			t	t		t	t

Clone D base	Clone D site	CFT 073 base	CFT 073 site	mutation type[a]	Inferred ancestral base[b]	lineage inferred to mutate	Support level[c]	ED1a[d]	536	S88	APEC O1	UTI89	E2348/69	ATCC8739	K-12	HS	SMS3-5	IAI39	E24377A	SE11	55989	IAI1	Sakai	UMN026	B4 Sb227	F5 8401	SS Ss046	D1 Sd197	B18 BS512	F2a 2457T	F2a 301
c	3427613	t	3556793	s	c	CFT073	++	c	c	t	t	t	t	t	t	c	c	c	t	c	t	c		c	c	c	c	t	c	c	c
t	3427688	g	3556868	s	t	CFT073	++	t	t	g	g	g	t	t	t	c	g	g	c	c	t	c		c	a	a	c	t	a	c	c
c	3427694	t	3556874	s	c	CFT073	++	c	c	t	t	t	c	c	c	c	t	t	c	c	c	c		c	g	g	c	c	g	c	c
c	3428123	t	3557303	s	c	CFT073	++	c	c	c	c	c	c	c	c	c	c	c	c	c	c	c	c	c	c	c	c	c	c	c	c
a	3428285	g	3557465	s	a	CFT073	++	a	a	a	a	a	a	a	a	a	g	g	a	a	g	a	g	g	a	a	g	g	a	a	a
g	3428363	a	3557543	s	g	CFT073	++	g	g	g	g	g	g	g	g	g	g	g	g	g	g	g	g	g	g	g	g	g	g	g	g
g	3428372	a	3557552	s	g	CFT073	++	g	g	g	g	g	g	g	g	g	g	g	g	g	g	g	g	g	g	g	g	g	g	g	g
c	3428375	t	3557555	s	c	CFT073	++	c	c	c	c	c	c	c	c	c	c	c	c	c	c	c	c	c	c	c	c	c	c	c	c
c	3428465	a	3557645	s	a	clone D	++	a	c	a	a	a	c	c	c	c	c	c	c	c	c	c	c	c	t	t	c	c	t	c	c
g	3428471	a	3557651	s	a	clone D	++	a	g	a	a	a	g	g	g	g	g	g	g	g	g	g	g	g	c	c	g	g	c	g	g
g	3428474	a	3557654	s	a	clone D	++	a	g	a	a	a	g	g	g	g	g	g	g	g	g	g	g	g	g	g	g	g	g	g	g
a	3428627	g	3557807	s	g	clone D	++	g	g	g	g	g	a	g	g	g	g	g	g	g	g	g	g	g	c	c	g	g	c	g	g
a	3428630	g	3557810	ns	g	clone D	++++	g	g	g	g	g	a	g	g	g	g	g	g	g	g	g	g	g	g	g	g	g	g	g	g
t	3428631	g	3557811	ns	g	clone D	++++	g	g	g	g	g	t	g	g	g	g	g	g	g	g	g	g	g	g	g	g	g	g	g	g
a	3429193	g	3558373	nc	g	clone D	++++	g	g	g	g	g	g	g	g	g	g	g	g	g	g	g	g	g	g	g	g	g	g	g	g
c	3429216	t	3558396	nc	t	clone D	++++	t	c	t	t	t	t	c	c	c	c	c	c	c	c	c	c	c	c	c	c	c	c	c	c
t	3429305	a	3558485	nc	t	CFT073	++++	t	t	t	t	t	a	t	t	t	t	t	t	t	t	t	t	t	t	t	t	t	t	t	t
g	3429321	a	3558501	nc	g	CFT073	++++	g	g	g	g	g	a	g	g	g	g	g	g	g	g	g	g	g	g	g	g	g	g	g	g
a	3429409	g	3558589	s	a	CFT073	++++	a	a	a	a	a	a	a	a	a	a	a	a	a	a	a	a	a	a	a	a	a	a	a	a
a	3429410	g	3558590	s	a	CFT073	++++	a	a	a	a	a	g	a	a	a	a	a	a	a	a	a	a	a	a	a	a	a	a	a	a
c	3429413	a	3558593	s	c	CFT073	++++	c	c	c	c	c	a	c	c	c	c	c	c	c	c	c	c	c	c	c	c	c	c	c	c
c	3429416	t	3558596	s	c	CFT073	++++	c	c	c	c	c	c	c	c	c	c	c	c	c	c	c	c	c	c	c	c	c	c	c	c
g	3429422	a	3558602	s	g	CFT073	++++	g	g	g	g	g	g	g	g	g	g	g	g	g	g	g	g	g	g	g	g	g	g	g	g
g	3429428	a	3558608	s	g	CFT073	++++	g	g	g	g	g	g	g	g	g	g	g	g	g	g	g	g	g	g	g	g	g	g	g	g
g	3429431	a	3558611	s	g	CFT073	++++	g	g	g	g	g	g	g	g	g	g	g	g	g	g	g	g	g	g	g	g	g	g	g	g
g	3429437	a	3558617	s	g	CFT073	++++	g	g	g	g	g	g	g	g	g	g	g	g	g	g	g	g	g	g	g	g	g	g	g	g
a	3429443	g	3558623	s	a	CFT073	++++	a	g	a	a	a	g	g	g	g	g	g	g	g	g	g	g	g	g	g	g	g	g	g	g
t	3429449	c	3558629	s	t	CFT073	++++	t	t	t	t	t	t	t	t	t	t	t	t	t	t	t	t	t	t	t	t	t	t	t	t

Clone D base	Clone D site	CFT 073 base	CFT 073 site	mutation type[a]	Inferred ancestral base[b]	lineage inferred to mutate	Support level[c]	ED1a[d]	536	S88	APEC O1	UTI89	E2348/69	ATCC8739	K-12	HS	SMS3-5	IAI39	E24377A	SE11	55989	IAI1	Sakai	UMN026	B4 Sb227	F5 8401	SS Ss046	D1 Sd197	B18 BS512	F2a 2457T	F2a 301
a	3429455	g	3558635	s	a	CFT073	++++	a	a	a	a	a	a	a	a	a	a	a	a	a	a	a	a	a	a	a	a	a	a	a	a
g	3429458	a	3558638	s	g	CFT073	++++	g	g	g	g	g	g	g	g	g	g	g	g	g	g	g	g	g	g	g	g	g	g	g	g
t	3429461	c	3558641	s	t	CFT073	++++	t	t	t	t	t	t	t	t	t	t	t	t	t	t	t	t	t	t	t	t	t	t	t	t
c	3429476	t	3558656	s	c	CFT073	++++	c	c	c	c	c	c	c	c	c	c	c	c	c	c	c	c	c	c	c	c	c	c	c	c
a	3429481	g	3558661	s	a	CFT073	++++	a	a	a	a	a	a	a	a	a	a	a	a	a	a	a	a	a	a	a	a	a	a	a	a
a	3429482	g	3558662	s	a	CFT073	++++	a	a	a	a	a	a	a	a	a	a	a	a	a	a	a	a	a	a	a	a	a	a	a	a
a	3429497	g	3558677	s	g	clone D	++	g	a	g	g	g	a	a	a	a	a	a	a	a	a	a	a	a	a	a	a	a	a	a	a
g	3429500	a	3558680	s	a	clone D	++	a	g	a	a	a	g	g	g	g	g	g	g	g	g	g	g	g	g	g	g	g	g	g	g
g	3429503	a	3558683	s	a	clone D	++	a	g	a	a	a	g	g	g	g	g	g	g	g	g	g	g	g	g	g	g	g	g	g	g
c	3429512	t	3558692	s	t	clone D	++	t	c	t	t	t	c	c	c	c	c	c	c	c	c	c	c	c	c	c	c	c	c	c	c
g	3429527	a	3558707	s	a	clone D	++	a	g	a	a	a	g	g	g	g	g	g	g	g	g	g	a	g	g	g	g	g	g	g	g
t	3429533	c	3558713	ns	c	clone D	++++	c	t	t	t	t	t	t	t	t	t	t	t	t	t	t	t	t	t	t	t	t	t	t	t
t	3429535	g	3558715	ns	g	clone D	++++	g	t	t	t	t	t	t	t	t	t	t	t	t	t	t	t	t	t	t	t	t	t	t	t
g	3429536	a	3558716	s	a	clone D	++++	a	g	g	g	g	g	g	g	g	g	g	g	g	g	g	g	g	g	g	g	g	g	g	g
c	3429539	a	3558719	s	a	CloneD	++	a	c	c	c	c	c	c	c	c	c	c	c	c	c	c	c	c	c	c	c	c	c	c	c
g	3429563	c	3558743	s	c	clone D	++	c	c	c	c	c	g	g	g	c	c	g	g	c	g	c	g	c	c	c	c	c	c	c	c
g	3429569	a	3558749	s	a	clone D	++++	a	g	a	a	a	g	g	g	g	g	g	g	g	g	g	g	g	g	g	g	g	g	g	g
a	3429575	g	3558755	s	g	clone D	++++	g	g	g	g	g	a	g	g	g	g	a	g	g	g	g	g	g	g	g	g	g	g	g	g
g	3429578	a	3558758	s	a	clone D	++++	a	a	a	a	a	g	g	g	a	a	g	g	a	g	a	g	a	a	a	a	a	a	a	a
a	3429587	c	3558767	s	c	clone D	++++	c	a	c	c	c	a	a	a	a	a	a	a	a	a	a	a	a	a	a	a	a	a	a	a
g	3429593	a	3558773	s	a	clone D	++++	a	g	a	a	a	g	g	g	g	g	g	g	a	g	a	g	g	a	g	a	a	a	g	g
t	3429758	c	3558938	s	c	clone D	++++	c	c	c	c	c	c	c	c	c	c	c	c	c	c	c	c	c	c	c	c	c	c	c	c
a	3429760	g	3558940	s	g	clone D	++++	g	g	g	g	g	g	g	g	g	g	g	g	g	g	g	g	g	g	g	g	g	g	g	g
g	3429763	a	3558943	s	a	clone D	++++	a	a	a	a	a	a	a	a	a	a	a	a	a	a	a	a	a	a	a	a	a	a	a	a
a	3429764	t	3558944	s	t	clone D	++++	t	t	t	t	t	t	t	t	t	t	t	t	t	t	t	t	t	t	t	t	t	t	t	t
t	3429770	g	3558950	s	g	clone D	++++	g	g	g	g	g	g	g	g	g	g	g	g	g	g	g	g	g	g	g	g	g	g	g	g
c	3429773	a	3558953	s	a	clone D	++++	a	a	a	a	a	a	a	a	a	a	a	a	a	a	a	a	a	a	a	a	a	a	a	a
a	3429779	c	3558959	s	c	clone D	++++	c	c	c	c	c	c	c	c	c	c	c	c	c	c	c	c	c	c	c	c	c	c	c	c

Clone D base	Clone D site	CFT 073 base	CFT 073 site	mutation type[a]	Inferred ancestral base[b]	lineage inferred to mutate	Support level[c]	ED1a[d]	536	S88	APEC O1	UTI89	E2348/69	ATCC8739	K-12	HS	SMS3-5	IAI39	E24377A	SE11	55989	IAI1	Sakai	UMN026	B4 Sb227	F5 8401	SS Ss046	D1 Sd197	B18 BS512	F2a 2457T	F2a 301
t	3429791	c	3558971	s	c	clone D	++	c	c	c	c	c	c	c	c	c	c	c	c	c	c	c	c	c	c	c	c	c	c	c	c
c	3429797	a	3558977	s	a	clone D	++	a	a	a	a	a	a	a	a	a	a	a	a	a	a	a	a	a	a	a	a	a	a	a	a
t	3429815	c	3558995	s	t	CFT073	++++	t	c	t	t	t	c	c	c	c	c	c	c	c	c	c	c	c	c	c	c	c	c	c	c
g	3429818	a	3558998	s	g	CFT073	++++	g	a	g	g	g	a	a	a	a	a	a	a	a	a	a	a	a	a	a	a	a	a	a	a
t	3429824	c	3559004	s	t	CFT073	++++	t	c	t	t	t	c	c	c	c	c	c	c	c	c	c	c	c	c	c	c	c	c	c	c
c	3429836	a	3559016	s	c	CFT073	++++	c	a	c	c	c	a	a	a	a	g	a	a	a	a	a	a	a	a	a	a	a	a	a	a
g	3429842	a	3559022	s	g	CFT073	++++	g	a	g	g	g	a	a	a	a	a	a	a	a	a	a	a	a	a	a	a	a	a	a	a
g	3429845	a	3559025	s	g	CFT073	++++	g	a	g	g	g	a	a	a	a	a	a	a	a	a	a	a	a	a	a	a	a	a	a	a
t	3429848	c	3559028	s	t	CFT073	++++	t	c	t	t	t	c	c	c	c	c	c	c	c	c	c	c	c	c	c	c	c	c	c	c
t	3429857	c	3559037	s	t	CFT073	++++	t	c	t	t	t	c	c	c	c	c	c	c	c	c	c	c	c	c	c	c	c	c	c	c
c	3429860	g	3559040	s	c	CFT073	++++	c	g	c	c	c	g	g	g	g	g	g	g	g	g	g	g	g	g	g	g	g	g	g	g
t	3429884	c	3559064	s	t	CFT073	++++	t	c	t	t	t	c	c	c	c	c	c	c	c	c	c	c	c	c	c	c	c	c	c	c
g	3429887	a	3559067	s	g	CFT073	++++	g	g	g	g	g	g	g	g	g	g	g	g	g	g	g	g	g	g	g	g	g	g	g	g
c	3429950	t	3559130	s	t	clone D	++	t	c	t	t	t	c	c	c	c	c	c	c	c	c	c	c	c	c	c	c	c	c	c	c
a	3429952	g	3559132	s	a	CFT073	++++	a	g	a	a	a	g	a	a	a	a	a	g	g	g	a	g	g	a	g	a	g	a	g	g
a	3429956	g	3559136	s	g	clone D	++	g	g	g	g	g	g	a	a	a	a	a	g	g	g	a	g	g	a	g	a	g	a	g	g
g	3429986	t	3559166	ns	t	clone D	++	t	g	t	t	t	g	g	g	g	g	g	g	g	g	g	g	g	g	g	g	g	g	g	g
c	3430010	a	3559190	s	c	CFT073	++++	c	c	c	c	c	c	c	c	c	c	c	c	c	c	c	c	c	c	c	c	c	c	c	c
t	3430022	c	3559202	s	t	CFT073	++++	t	t	t	t	t	t	t	t	t	t	t	t	t	t	t	t	t	t	t	t	t	t	t	t
c	3430025	t	3559205	s	c	CFT073	++++	c	c	c	c	c	c	c	c	c	c	c	c	c	c	c	c	c	c	c	c	c	c	c	c
g	3430028	a	3559208	s	g	CFT073	++++	g	g	g	g	g	g	g	g	g	g	g	g	g	g	g	g	g	g	g	g	g	g	g	g
t	3430064	c	3559244	s	t	CFT073	++++	t	t	t	t	t	t	t	t	t	t	t	t	t	t	t	t	t	t	t	t	t	t	t	t
a	3430067	g	3559247	s	a	CFT073	++++	a	a	a	a	a	a	a	a	a	a	a	a	a	a	a	a	a	a	a	a	a	a	a	a
c	3430082	g	3559262	s	c	CFT073	++++	c	g	g	g	g	g	g	g	g	g	g	g	g	g	g	g	g	g	g	g	g	g	g	g
g	3430085	a	3559265	s	g	CFT073	++++	g	a	a	a	a	a	a	a	a	a	a	a	a	a	a	a	a	a	a	a	a	a	a	a
t	3430106	c	3559286	s	t	CFT073	++++	t	c	t	t	t	c	c	c	c	c	c	c	c	c	c	c	c	c	c	c	c	c	c	c
a	3430256	g	3559436	s	a	CFT073	++++	a	g	a	a	a	g	a	a	a	g	g	g	a	g	a	g	g	a	a	a	g	a	a	a
a	3430268	g	3559448	s	a	CFT073	++++	a	g	a	a	a	g	g	g	g	g	g	g	g	g	g	g	g	g	g	g	g	g	g	g

Clone D base	Clone D site	CFT 073 base	CFT 073 site	mutation type[a]	Inferred ancestral base[b]	lineage inferred to mutate	Support level[c]	ED1a[d]	536	S88	APEC O1	UTI89	E2348/69	ATCC8739	K-12	HS	SMS3-5	IAI39	E24377A	SE11	55989	IAI1	Sakai	UMN026	B4 Sb227	F5 8401	SS Ss046	D1 Sd197	B18 BS512	F2a 2457T	F2a 301
a	3430325	g	3559505	s	a	CFT073	++	a	g	g	g	g	g	g	g	g	g	g	g	g	g	g	g	g	g	g	g	g	g	g	g
g	3430330	a	3559510	s	g	CFT073	++	g	g	a	a	a	g	g	g	g	g	g	g	g	g	g	g	g	g	g	g	g	g	g	g
a	3430424	g	3559604	s	a	CFT073	++	a	g	g	g	g	g	a	a	a	a	g	a	a	a	a	g	g	a	a	a	g	a	a	a
g	3430472	a	3559652	s	g	CFT073	++	g	a	a	a	a	a	a	a	a	g	a	a	a	a	a	a	a	a	a	a	a	a	a	a
t	3430598	c	3559778	s	t	CFT073	++++	t	t	t	t	t	c	c	c	c	c	t	c	c	c	c	c	c	c	c	c	c	c	c	c
c	3430601	t	3559781	s	c	CFT073	++++	c	c	c	c	c	t	t	t	t	t	c	t	t	t	t	t	t	t	t	t	t	t	t	t
g	3430613	a	3559793	s	g	CFT073	++++	g	g	g	g	g	a	a	a	a	g	g	a	a	a	a	a	g	a	a	a	a	a	a	a
c	3430853	a	3560033	s	c	CFT073	++	c	a	c	c	c	c	a	a	a	g	c	a	a	a	a	a	g	a	a	a	a	a	a	a
t	3430859	g	3560039	s	t	CFT073	++++	t	g	t	t	t	t	g	g	g	g	t	g	g	g	g	g	g	g	g	g	g	g	g	g
t	3431413	c	3560593	s	t	CFT073	++	t	t	t	t	t	t	t	t	t	t	t	t	t	t	t	t	t	t	t	t	t	t	t	t
g	3431472	a	3560652	ns	g	CFT073	++++	g	a	a	a	a	g	a	a	a	a	a	a	a	a	a	a	a	a	a	a	a	a	a	a
g	3431905	a	3561085	s	g	CFT073	++	g	g	g	g	g	a	a	a	a	g	g	g	g	g	g	g	g	a	g	g	a	a	g	g
a	3432043	g	3561223	s	a	CFT073	++++	a	g	a	a	a	g	g	g	g	g	g	g	g	g	g	g	g	g	g	g	g	g	g	g
a	3432073	g	3561253	s	a	CFT073	++++	a	g	a	a	a	a	g	g	g	g	g	g	g	g	g	g	g	g	g	g	g	g	g	g
c	3432079	t	3561259	s	c	CFT073	++	c	t	t	t	t	t	t	t	t	t	t	t	t	t	t	t	t	t	t	t	t	t	t	t
t	3432115	c	3561295	s	t	CFT073	++++	t	c	t	t	t	t	t	t	t	c	c	t	t	t	t	t	t	t	t	t	t	t	t	t
a	3432118	g	3561298	s	a	CFT073	++++	a	a	a	a	a	a	a	a	a	a	g	a	a	a	a	g	g	a	g	a	g	a	g	g
g	3432346	a	3561526	nc	g	CFT073	++	g		g	g	g	g	g	g	g			g	g	g	g	g	g	g	g	g	g	g	g	g
a	3432911	g	3562091	s	a	CFT073	++++	a	g	a	a	a	g	g	g	g	a	g	g	g	g	g	g	g	g	g	g	g	g	g	g
a	3433060	g	3562240	ns	g	clone D	++++	g	g	g	g	g	g	g	g	g	g	g	g	g	g	g	g	g	g	g	g	g	g	g	g
g	3433194	a	3562374	s	g	CFT073	++	g	g	a	a	a	g	g	g	g	g	g	g	g	g	g	g	g	g	g	g	g	g	g	g
a	3433441	g	3562621	ns	a	CFT073	++	a	g	g	g	g	a	g	g	g	g	g	g	g	g	g	g	g	g	g	g	g	g	g	g
a	3433575	c	3562755	ns	c	clone D	++++	c	c	c	c	c	c	c	c	c	c	c	c	c	c	c	c	c	c	c	c	c	c	c	c
a	3433660	g	3562840	s	g	clone D	++++	g	g	g	g	g	g	g	g	g	g	g	g	g	g	g	g	g	g	g	g	g	g	g	g
a	3433831	g	3563011	s	a	CFT073	++	a	g	g	g	g	a	g	g	g	g	g	g	g	g	g	g	g	g	g	g	g	g	g	g
c	3434375	t	3563555	ns	c	CFT073	++++	c	c	c	c	c	c	c	c	c	c	c	c	c	c	c	c	c	c	c	c	c	c	c	c
a	3434622	g	3563802	s	a	CFT073	++++	a	a	a	a	a	a	a	a	a	a	a	a	a	a	a	a	a	a	a	a	a	a	a	a
a	3434625	g	3563805	s	a	CFT073	++++	a	a	a	a	a	a	a	a	a	a	a	a	a	a	a	a	a	a	a	a	a	a	a	a

Clone D base	Clone D site	CFT 073 base	CFT 073 site	mutation type[a]	Inferred ancestral base[b]	lineage inferred to mutate	Support level[c]	ED1a[d]	536	S88	APEC O1	UTI89	E2348/69	ATCC8739	K-12	HS	SMS3-5	IAI39	E24377A	SE11	55989	IAI1	Sakai	UMN026	B4 Sb227	F5 8401	SS Ss046	D1 Sd197	B18 BS512	F2a 2457T	F2a 301
t	3434706	c	3563886	s	c	clone D	++	c	c	c	c	c	t	g	g	g	g	g	g	g	g	g	g	g	g	g	g	g	g	g	g
g	3434863	a	3564043	ns	g	CFT073	++++	g	g	g	g	g	g	t	g	g	g	g	g	g	g	g	g	g	g	g	g	g	g	g	g
g	3436040	a	3565220	s	g	CFT073	++++	g	g	g	g	g	g	g	g	g	g	g	g	g	g	g	g	g	g	g	g	g	g	g	g
a	3436103	g	3565283	s	g	clone D	++++	g	g	a	a	a	g	g	g	g	g	g	g	g	g	g	g	g	g	g	g	g	g	g	g
t	3436292	c	3565472	s	c	clone D	++++	c	c	t	t	t	t	c	c	c	c	c	c	c	c	c	c	c	c	c	c	c	c	c	c
a	3436343	g	3565523	s	g	clone D	++++	g	g	a	a	a	a	g	g	g	g	g	g	g	g	g	g	g	g	g	g	g	g	g	g
g	3436378	c	3565558	ns	c	clone D	++++	c	c	g	g	g	g	c	c	c	c	c	c	c	c	c	c	c	c	c	c	c	c	c	c
a	3437256	g	3566436	s	a	CFT073	++++	a	a	g	g	g	g	g	g	g	g	g	g	g	g	g	g	g	g	g	g	g	g	g	g
t	3437451	c	3566631	s	t	CFT073	++++	t	t	c	c	c	c	t	t	t	t	t	c	c	c	c	t	c	t	t	t	t	t	t	t
g	3438041	a	3567221	s	g	CFT073	++++	g	g	g	g	g	g	g	g	g	g	g	g	g	g	g	g	g	g	g	g	g	g	g	g
t	3439896	c	3569076	ns	c	clone D	++++	c	c	c	c	c	c	c	c	c	c	c	c	c	c	c	c	c	c	c	c	c	c	c	c
a	3439897	c	3569077	ns	c	clone D	++++	c	c	c	c	c	c	c	c	c	c	c	c	c	c	c	c	c	c	c	c	c	c	c	c
g	3440016	a	3569196	ns	g	CFT073	++++	g	g	g	g	g	g	g	g	g	g	g	g	g	g	g	g	g	g	g	g	g	g	g	g
t	3440066	c	3569246	ns	c	clone D	++++	c	c	c	c	c	c	c	c	c	c	c	c	c	c	c	c	c	c	c	c	c	c	c	c
t	3440067	g	3569247	ns	g	clone D	++++	g	g	g	g	g	g	g	g	g	g	g	g	g	g	g	g	g	g	g	g	g	g	g	g
a	3440150	g	3569330	s	g	clone D	++++	g	g	g	g	g	g	g	g	g	g	g	g	g	g	g	g	g	g	g	g	g	g	g	g
c	3440324	t	3569504	s	t	clone D	++++	t	c	t	t	t	c	c	c	c	c	c	c	c	c	c	c	c	c	c	c	c	c	c	c
g	3440341	a	3569521	ns	a	clone D	++++	a	g	g	g	g	g	g	g	g	g	g	g	g	g	g	g	g	g	g	g	g	g	g	g
g	3440415	t	3569595	ns	t	clone D	++++	t	t	t	t	t	t	t	t	t	t	t	t	t	t	t	t	t	t	t	t	t	t	t	t
a	3440423	g	3569603	s	g	clone D	++++	g	g	g	g	g	g	g	g	g	g	g	g	g	g	g	g	g	g	g	g	t	g	g	g
a	3440536	t	3569716	nc	t	clone D	++++	t	t	t	t	t	t	t	t	t	t	t	t	t	t	t	t	t	t	t	t	t	t	t	t
g	3440578	a	3569758	nc	a	clone D	++++	a	g	g	g	g	a	g	g	g	g	g	g	g	g	g	g	g	g	g	g	g	g	g	g
a	3440668	g	3569848	s	g	clone D	++++	g	g	g	g	g	g	g	g	g	g	g	g	g	g	g	g	g	g	g	g	g	g	g	g
g	3441061	t	3570241	s	t	clone D	++++	t	g	g	g	g	g	g	g	g	g	g	g	g	g	g	g	g	g	g	g	g	g	g	g
g	3441694	a	3570874	s	g	CFT073	++++	g	g	g	g	g	g	g	g	g	g	g	g	g	g	g	g	g	g	g	g	g	g	g	g
c	3442087	t	3571267	ns	c	CFT073	++++	c	c	c	c	c	c	c	c	c	c	c	c	c	c	c	c	c	c	c	c	c	c	c	c
c	3442357	t	3571537	nc	t	clone D	++++	t	c	t	t	t	c	c	c	c	c	c	c	c	c	c	c	c	c	c	c	c	c	c	c
g	3442992	a	3572172	ns	g	CFT073	++++	g	g	g	g	g	g	g	g	g	g	g	g	g	g	g	g	g	g	g	g	g	g	g	g

Clone D base	Clone D site	CFT 073 base	CFT 073 site	mutation type[a]	Inferred ancestral base[b]	lineage inferred to mutate	Support level[c]	ED1a[d]	536	S88	APEC O1	UTI89	E2348/69	ATCC8739	K-12	HS	SMS3-5	IAI39	E24377A	SE11	55989	IAI1	Sakai	UMN026	B4 Sb227	F5 8401	SS Ss046	D1 Sd197	B18 BS512	F2a 2457T	F2a 301
a	3443723	g	3572903	s	a	CFT073	++++	a	g	a	a	a	g	g	g	g	g	g	g	g	g	g	g	g	g	g	g	g	g	g	g
c	3443753	t	3572933	s	c	CFT073	++++	c	c	c	c	c	t	c	c	c	c	c	c	c	c	c	c	c	c	c	c	c	c	c	c
g	3443794	a	3572974	s	g	CFT073	++++	g	g	g	g	g	a	g	g	g	g	g	g	g	g	g	g	g	g	g	g	g	g	g	g
c	3443933	t	3573113	s	c	CFT073	++++	c	c	t	t	t	c	c	c	c	c	c	c	c	c	c	c	c	c	c	c	c	c	c	c
g	3444193	a	3573373	ns	g	CFT073	++++	g	g	g	g	g	g	g	g	g	g	g	g	g	g	g	g	g	g	g	g	g	g	g	g
g	3444268	a	3573448	s	g	CFT073	++++	g	g	g	g	g	a	g	g	g	g	g	g	g	g	g	g	g	g	g	g	g	g	g	g
a	3444588	c	3573768	s	a	CFT073	++++	a	c	a	a	a	c	c	c	c	c	c	c	c	c	c	c	c	c	c	c	c	c	c	c
c	3444704	t	3573884	ns	c	CFT073	++++	c	c	c	c	c	c	c	c	c	c	c	c	c	c	c	c	c	c	c	c	c	c	c	c
c	3444727	t	3573907	s	c	CFT073	++++	c	c	c	c	c	c	c	c	c	c	c	c	c	c	c	c	c	c	c	c	c	c	c	c
g	3444759	t	3573939	s	g	CFT073	++++	g	t	g	g	g	g	t	t	t	t	t	t	t	t	t	t	t	t	t	t	t	t	t	t
a	3444816	g	3573996	s	a	CFT073	++++	a	g	g	g	g	a	g	a	g	g	g	g	g	g	g	g	a	g	g	g	g	g	g	g
g	3445368	c	3574548	s	g	CFT073	++++	g	c	c	c	c	g	c	c	c	g	c	c	c	c	c	c	c	c	c	c	c	c	c	c
a	3445473	t	3574653	s	a	CFT073	++++	a	t	t	t	t	t	a	a	a	t	a	a	a	a	a	a	a	a	a	a	a	a	a	a
a	3445476	g	3574656	s	a	CFT073	++++	a	g	g	g	g	g	a	a	a	g	a	a	a	a	a	a	a	a	a	a	a	a	a	a
t	3445479	g	3574659	s	t	CFT073	++++	t	t	g	g	g	g	t	t	t	t	t	t	t	t	t	t	t	t	t	t	t	t	t	t
g	3445482	t	3574662	s	g	CFT073	++++	g	t	t	t	t	t	g	g	g	t	g	g	g	g	g	g	g	g	g	g	g	g	g	g
t	3445485	c	3574665	s	t	CFT073	++++	t	c	c	c	c	c	t	t	t	c	t	t	t	t	t	t	t	t	t	t	t	t	t	t
t	3445486	c	3574666	s	t	CFT073	++++	t	c	c	c	c	c	t	t	t	c	t	t	t	t	t	t	t	t	t	t	t	t	t	t
c	3445491	t	3574671	s	c	CFT073	++++	c	t	t	t	t	t	t	t	t	t	t	t	t	t	t	t	t	t	t	t	t	t	t	t
c	3446192	t	3575372	s	c	CFT073	++++	c	t	c	c	c	t	c	c	c	t	c	c	c	c	c	t	c	c	c	c	t	c	c	c
a	3446197	t	3575377	s	a	CFT073	++++	a	t	a	a	a	a	a	a	a	t	a	a	a	a	a	t	a	a	a	a	t	a	a	a
a	3446575	c	3575755	s	a	CFT073	++	a	c	a	a	a	c	c	c	c	c	c	c	c	c	c	c	c	c	c	c	c	c	c	c
a	3447044	g	3576224	s	a	CFT073	++++	a	g	a	a	a	g	g	g	g	g	g	g	g	g	g	g	g	g	g	g	g	g	g	g
g	3447071	a	3576251	s	g	CFT073	++++	g	a	g	g	g	g	g	a	a	a	a	a	a	a	a	a	a	a	a	a	a	a	a	a
g	3447137	a	3576317	s	a	clone D	++++	a	g	a	a	a	a	g	g	g	g	g	g	g	g	g	g	g	g	g	g	g	g	g	g
a	3447155	g	3576335	s	g	clone D	++++	g	a	g	g	g	g	a	a	a	a	a	a	a	a	a	a	a	a	a	a	a	a	a	a
g	3447182	a	3576362	s	a	clone D	++++	a	g	a	a	a	a	g	g	g	g	g	g	g	g	g	g	g	g	g	g	g	g	g	g
g	3447185	c	3576365	s	c	clone D	++++	c	g	c	c	c	c	g	g	g	g	g	g	g	g	g	g	g	g	g	g	g	g	g	g

Clone D base	Clone D site	CFT 073 base	CFT 073 site	mutation type[a]	Inferred ancestral base[b]	lineage inferred to mutate	Support level[c]	ED1a[d]	536	S88	APEC O1	UTI89	E2348/69	ATCC8739	K-12	HS	SMS3-5	IAI39	E24377A	SE11	55989	IAI1	Sakai	UMN026	B4 Sb227	F5 8401	SS Ss046	D1 Sd197	B18 BS512	F2a 2457T	F2a 301
c	3447197	t	3576377	s	t	clone D	++	t	c	t	t	t	t	c	c	c	c	c	c	c	c	c	c	c	c	c	c	c	c	c	c
a	3447201	g	3576381	ns	g	clone D	++++	g	g	g	g	g	g	g	g	g	g	g	g	g	g	g	g	g	g	g	g	g	g	g	g
a	3447230	g	3576410	s	g	clone D	++++	g	g	g	g	g	g	g	g	g	g	a	g	g	g	g	g	g	g	g	g	g	g	g	g
c	3447239	a	3576419	s	a	clone D	++++	a	c	a	a	a	a	c	c	c	c	c	c	c	c	c	c	c	c	c	c	c	c	c	c
c	3447254	t	3576434	s	t	clone D	++++	t	c	t	t	t	t	c	c	c	c	c	c	c	c	c	c	c	c	c	c	c	c	c	c
g	3447263	a	3576443	s	a	clone D	++++	a	g	a	a	a	a	g	g	g	g	g	g	g	g	g	g	g	g	g	g	g	g	g	g
c	3447269	t	3576449	s	t	clone D	++++	t	c	t	t	t	t	c	c	c	c	c	c	c	c	c	c	c	c	c	c	c	c	c	c
t	3447284	g	3576464	ns	g	clone D	++	g	g	g	g	g	g	g	g	g	g	g	g	g	g	g	a	g	g	g	g	g	g	g	g
c	3447398	g	3576578	s	g	clone D	++++	g	c	g	g	g	c	c	c	c	c	c	c	c	c	c	c	c	c	c	c	c	c	c	c
g	3447401	a	3576581	s	a	clone D	++++	a	g	a	a	a	g	g	g	g	g	g	g	g	g	g	g	g	g	g	g	g	g	g	g
g	3447410	a	3576590	s	a	clone D	++++	a	g	a	a	a	g	g	g	g	g	g	g	g	g	g	g	g	g	g	g	g	g	g	g
a	3447413	g	3576593	s	g	clone D	++++	g	a	g	g	g	a	a	a	a	a	a	a	a	a	a	a	a	a	a	a	a	a	a	a
c	3447455	t	3576635	s	t	clone D	++++	t	t	t	t	t	c	c	c	c	t	c	t	c	c	c	t	c	c	c	c	t	c	c	c
a	3447995	g	3577175	s	g	clone D	++++	g	g	g	g	g	g	g	g	g	g	g	g	g	g	g	g	g	g	g	g	g	g	g	g
g	3448070	a	3577250	s	a	clone D	++++	a	g	a	a	a	g	g	g	g	a	g	g	g	g	g	g	g	g	g	g	g	g	g	g
g	3448079	t	3577259	s	t	clone D	++++	t	c	t	t	t	t	t	t	t	t	t	t	t	t	t	t	t	t	t	t	t	t	t	t
c	3448082	t	3577262	s	t	clone D	++++	t	c	t	t	t	c	c	c	c	c	c	c	c	c	c	c	c	c	c	c	c	c	c	c
c	3448133	t	3577313	s	c	CFT073	++++	c	c	c	c	c	t	c	c	c	c	c	c	c	c	c	c	c	c	c	c	c	c	c	c
t	3448190	c	3577370	s	c	clone D	++++	c	t	c	c	c	c	t	t	t	c	t	t	t	t	t	t	t	t	t	t	t	t	t	t
c	3448371	t	3577551	ns	t	clone D	++++	t	c	t	t	t	c	c	c	c	c	c	c	c	c	c	c	c	c	c	c	c	c	c	c
t	3448837	c	3578017	ns	c	clone D	++++	c	c	c	c	c	c	c	c	c	c	c	c	c	c	c	c	c	c	c	c	c	c	c	c
g	3449337	a	3578517	ns	g	CFT073	++++	g	g	g	g	g	g	g	g	g	g	g	g	g	g	g	g	g	g	g	g	g	g	g	g
a	3449438	g	3578618	ns	g	clone D	++++	g	g	g	g	g	a	g	g	g	g	g	g	g	g	g	g	g	g	g	g	g	g	g	g
g	3450138	t	3579318	ns	g	CFT073	++++	g	g	g	g	g	g	g	g	g	g	g	g	g	g	g	g	g	g	g	g		g	g	g
a	3450266	g	3579447	s	g	clone D	++	g	a	a	a	a	a	g	g	g	a	g	g	g	g	g	t	g	g	g	g		g	g	g
t	3450275	a	3579456	s	a	clone D	++++	a	t	t	t	t	t	t	t	t	t	t	t	t	t	t	t	t	t	t	t		t	t	t
t	3450449	c	3579630	s	t	CFT073	++++	t	t	t	t	t	t	t	t	t	t	t	t	t	t	t	t	t	t	t	t	t	t	t	t
g	3452185	a	3581366	s	g	CFT073	++	g	g	a	a	a	g	g	g	g	g	g	g	g	g	g	g	g	g	g	g	g	g	g	g

Clone D base	Clone D site	CFT 073 base	CFT 073 site	mutation type[a]	Inferred ancestral base[b]	lineage inferred to mutate	Support level[c]	ED1a[d]	536	S88	APEC O1	UTI89	E2348/69	ATCC8739	K-12	HS	SMS3-5	IAI39	E24377A	SE11	55989	IAI1	Sakai	UMN026	B4 Sb227	F5 8401	SS Ss046	D1 Sd197	B18 BS512	F2a 2457T	F2a 301
a	3452197	g	3581378	s	g	clone D	++++	g	g	g	g	g	g	g	g	g	a	g	g	g	g	g	g	g	g	g	g	g	g	g	g
g	3452212	a	3581393	s	a	clone D	++++	a	a	a	a	a	a	g	g	g	g	a	g	g	g	g	a	g	g	g	g	a	g	g	g
g	3452281	a	3581462	s	a	clone D	++++	a	g	a	a	a	a	g	g	g	g	g	g	g	g	g	g	g	g	g	g	g	g	g	g
a	3453043	g	3582224	s	a	CFT073	++++	a	g	g	g	g	g	g	g	g	g	g	g	g	g	g	g	g	g	g	g	g	g	g	g
g	3453049	c	3582230	s	g	CFT073	++++	g	g	c	c	c	a	c	c	c	c	c	c	c	c	c	c	c	c	c	c	c	c	c	c
a	3453060	g	3582241	s	a	CFT073	++++	a	g	g	g	g	g	g	g	g	a	g	g	g	g	g	g	g	g	g	g	g	g	g	g
c	3453064	a	3582245	s	c	CFT073	++	c	g	g	g	g	g	g	g	g	g	g	g	g	g	g	g	g	g	g	g	g	g	g	g
c	3453082	g	3582263	s	c	CFT073	++++	c	g	c	c	c	g	g	g	g	g	g	g	g	g	g	g	g	g	g	g	g	g	g	g
c	3453172	t	3582353	s	c	CFT073	++	c	c	t	t	t	c	c	c	c	t	c	c	c	c	c	c	c	c	c	c	c	c	c	c
c	3453222	t	3582403	ns	c	CFT073	++++	c	c	c	c	c	t	c	c	c	c	c	c	c	c	c	c	c	c	c	c	c	c	c	c
t	3453226	c	3582407	s	t	CFT073	++++	t	c	c	c	c	c	c	c	c	c	c	c	c	c	c	c	c	c	c	c	c	c	c	c
c	3453399	t	3582580	ns	c	CFT073	++++	c	c	c	c	c	c	c	c	c	c	c	c	c	c	c	c	c	c	c	c	c	c	c	c
t	3453490	a	3582671	s	a	clone D	++	a	a	a	a	a	a	a	a	a	a	a	a	a	a	a	a	a	a	a	a	a	a	a	a
c	3453794	a	3582975	nc	a	clone D	++	a	a	a	a	a	a				a														
t	3453822	a	3583003	nc	a	clone D	++	a	a	a	a	a	a				a														
g	3455079	a	3584260	s	g	CFT073	++	g	a	a	a	a	a				a														
t	3455085	g	3584266	s	g	clone D	++	g	g	g	g	g	g				g														
a	3455093	g	3584274	ns	a	CFT073	++	a	g	g	g	g	g				g														
c	3455112	t	3584293	s	c	CFT073	++	c	c	c	c	c	t				c														
a	3455223	g	3584404	s	a	CFT073	++	a	g	g	g	g	g				g														
a	3455224	g	3584405	ns	a	CFT073	++	a	g	g	g	g	g				g														
g	3455245	a	3584426	ns	g	CFT073	++	g	a	a	a	a	a				a														
t	3455246	g	3584427	ns	t	CFT073	++	t	g	g	g	g	g				g														
g	3455247	a	3584428	ns	g	CFT073	++	g	a	a	a	a	a				a														
g	3455271	t	3584452	ns	g	CFT073	++	g	t	t	t	t	t				t														
t	3455275	c	3584456	s	t	CFT073	++	t	c	c	c	c	c				c														
a	3455278	g	3584459	ns	a	CFT073	++	a	g	g	g	g	g				g														
c	3455325	t	3584506	s	c	CFT073	++	c	t	c	c	c	c				t														

Clone D base	Clone D site	CFT 073 base	CFT 073 site	mutation type[a]	Inferred ancestral base[b]	lineage inferred to mutate	Support level[c]	ED1a[d]	536	S88	APEC O1	UTI89	E2348/69	ATCC8739	K-12	HS	SMS3-5	IAI39	E24377A	SE11	55989	IAI1	Sakai	UMN026	B4 Sb227	F5 8401	SS Ss046	D1 Sd197	B18 BS512	F2a 2457T	F2a 301
t	3455331	c	3584512	s	t	CFT073	++	t	c	t	t	t	t				c														
t	3455338	c	3584519	ns	t	CFT073	++	t	c	c	c	c	c				c														
g	3455346	a	3584527	s	g	CFT073	++	g	a	g	g	g	g				a														
a	3455349	t	3584530	s	a	CFT073	++	a	t	a	a	a	a				t														
g	3455352	a	3584533	s	g	CFT073	++	g	a	a	a	a	g				a														
a	3455377	c	3584558	ns	a	CFT073	++	a	c	a	a	a	a				c														
a	3455408	t	3584589	ns	a	CFT073	++	a	a	a	a	a	a				t														
g	3455412	a	3584593	s	g	CFT073	++	g	g	g	g	g	g				a														
t	3455422	g	3584603	ns	t	CFT073	++	t	t	g	g	g	g				g														
g	3455455	a	3584636	ns	g	CFT073	++	g	g	g	g	g	g				a														
g	3455460	a	3584641	s	g	CFT073	++	g	g	a	a	a	a				a														
c	3455475	t	3584656	s	c	CFT073	++	c	c	t	t	t	t				t														
c	3455487	t	3584668	s	c	CFT073	++	c	c	t	t	t	c				t														
a	3455520	c	3584701	s	a	CFT073	++	a	a	c	c	c	a				c														
c	3455631	t	3584812	s	t	clone D	++	t	c	t	t	t	t				t														
t	3455718	c	3584899	s	c	clone D	++	c	c	c	c	c	c				c														
a	3455735	g	3584916	ns	g	clone D	++	g	a	g	g	g	g				g														
t	3455736	c	3584917	ns	c	clone D	++	c	t	c	c	c	c				c														
g	3455739	a	3584920	s	a	clone D	++	a	g	a	a	a	a				a														
a	3455745	g	3584926	s	g	clone D	++	g	a	g	g	g	g				a														
c	3455746	g	3584927	ns	g	clone D	++	g	c	g	g	g	g				c														
a	3455760	c	3584941	s	c	clone D	++	c	a	c	c	c	c				c														
g	3455761	a	3584942	ns	g	CFT073	++	g	g	g	g	g	g				g														
c	3455820	t	3585001	s	t	clone D	++	t	c	t	t	t	t				t														
g	3455824	a	3585005	ns	a	clone D	++	a	a	a	a	a	a				a														
c	3455841	t	3585022	s	t	clone D	++	t	c	t	t	t	t				t														
a	3455868	g	3585049	s	a	CFT073	++	a	a	g	g	g	a				g														
g	3455896	a	3585077	ns	g	CFT073	++	g	g	a	a	a	g				a														

Clone D base	Clone D site	CFT 073 base	CFT 073 site	mutation type[a]	Inferred ancestral base[b]	lineage inferred to mutate	Support level[c]	ED1a[d]	536	S88	APEC O1	UTI89	E2348/69	ATCC8739	K-12	HS	SMS3-5	IAI39	E24377A	SE11	55989	IAI1	Sakai	UMN026	B4 Sb227	F5 8401	SS Ss046	D1 Sd197	B18 BS512	F2a 2457T	F2a 301
g	3455968	a	3585149	ns	g	CFT073	++	g	g	a	a	a	g				a														
g	3455971	a	3585152	ns	g	CFT073	++	g	g	a	a	a	g				a														
c	3455972	t	3585153	ns	c	CFT073	++	c	c	t	t	t	c				t														
a	3456057	g	3585238	s	g	clone D	++	g	g	a	a	a	g				a														
t	3456075	a	3585256	s	a	clone D	++	a	a	t	t	t	a				a														
a	3456168	t	3585349	ns	t	clone D	++	t	a	t	t	t	t				t														
g	3456195	t	3585376	s	t	clone D	++	t	t	t	t	t	t				t														
a	3456210	c	3585391	ns	c	clone D	++	c	a	c	c	c	c				c														
t	3456214	c	3585395	ns	c	clone D	++	c	t	t	t	t	c				t														
a	3456494	c	3585675	s	c	clone D	++	c	c	c	c	c	c				c														
a	3456629	g	3585810	s	a	CFT073	++	a	a	a	a	a	g				a														
t	3456630	c	3585811	ns	t	CFT073	++	t	t	t	t	t	c				t														
c	3456631	t	3585812	ns	c	CFT073	++	c	c	c	c	c	c				c														
c	3456942	a	3586123	s	a	clone D	++	a	a	a	a	a	a				a														
c	3457139	a	3586320	s	a	clone D	++	a	a	c	c	c	a				a														
g	3457284	a	3586465	ns	g	CFT073	++	g	g	g	g	g	g				g														
a	3457338	g	3586519	ns	g	clone D	++	g	a	a	a	a	g				a														
g	3457400	a	3586581	s	g	CFT073	++	g	g	g	g	g	g				g														
t	3457866	c	3587047	s	c	clone D	++	c	t	t	t	t	t				t														
t	3458790	c	3587971	s	c	clone D	++	c	t	c	c	c	c				c														
c	3458952	t	3588133	s	t	clone D	++	t	c	c	c	c	c				c														
t	3459219	c	3588400	s	t	CFT073	++	t	t	t	t	t	t				t														
a	3459249	g	3588430	s	g	clone D	++	g	g	g	g	g	g				g														
c	3459327	t	3588508	s	t	clone D	++	t	c	t	t	t	t				c														
a	3459707	g	3588888	s	g	clone D	++	g	a	g	g	g	g				a														
g	3459731	t	3588912	s	t	clone D	++	t	g	t	t	t	t				g														
a	3460485	c	3589666	s	c	clone D	++	c	a	c	c	c	c				c														
a	3460642	c	3589823	ns	c	clone D	++	c	a	c	c	c	c				a														

Clone D base	Clone D site	CFT 073 base	CFT 073 site	mutation type[a]	Inferred ancestral base[b]	lineage inferred to mutate	Support level[c]	ED1a[d]	536	S88	APEC O1	UTI89	E2348/69	ATCC8739	K-12	HS	SMS3-5	IAI39	E24377A	SE11	55989	IAI1	Sakai	UMN026	B4 Sb227	F5 8401	SS Ss046	D1 Sd197	B18 BS512	F2a 2457T	F2a 301
a	3460662	g	3589843	s	a	CFT073	++	a	a	a	a	a	a				a														
c	3460668	t	3589849	s	c	CFT073	++	c	c	c	c	c	c				c														
t	3460673	a	3589854	ns	a	clone D	++	a	t	a	a	a	a				t														
t	3460674	c	3589855	ns	c	clone D	++	c	t	c	c	c	c				t														
a	3460689	g	3589870	s	g	clone D	++	g	g	g	g	g	g				g														
c	3460752	g	3589933	s	?	?	+/-	a	a	a	a	a	a				a														
t	3460863	c	3590044	s	t	CFT073	++	t	t	t	t	t	t				t														
g	3460866	c	3590047	s	g	CFT073	++	g	g	g	g	g	g				g														
g	3461001	t	3590182	s	g	CFT073	++	g	a	g	g	g	g				g														
a	3461130	c	3590311	s	a	CFT073	++	a	c	a	a	a	c				t														
g	3461588	a	3590769	s	g	CFT073	++++	g	g	g	g	g	g	g	g	g	a	g	g	g	g	g	a	g	a	g	a	a	a	g	g
g	3462221	a	3591402	s	a	clone D	++++	a	g	a	a	a	g	g	g	g	g	g	g	g	g	g	g	g	g	g	g	g	g	g	g
a	3462269	c	3591450	s	a	CFT073	++++	a	c	a	a	a	c	c	c	c	c	c	c	c	c	c	c	c	c	c	c	c	c	c	c
a	3462314	g	3591495	s	a	CFT073	++++	a	g	a	a	a	a	g	g	g	g	g	g	g	g	g	g	g	g	g	g	g	g	g	g
g	3462488	a	3591669	s	g	CFT073	++++	g	g	g	g	g	g	g	g	g	g	g	g	g	g	g	g	g	g	g	g	g	g	g	g
g	3462539	a	3591720	s	g	CFT073	++++	g	g	g	g	g	g	g	g	g	g	g	g	g	g	g	g	g	g	g	g	g	g	g	g
g	3462636	a	3591817	ns	g	CFT073	++++	g	g	g	g	g	g	g	g	g	g	g	g	g	g	g	g	g	g	g	g	a	g	g	g
g	3463054	a	3592235	s	g	CFT073	++++	g	g	g	g	g	g	g	g	g	g	g	g	g	g	g	g	g	g	g	g	g	g	g	g
a	3486515	g	3615696	ns	a	CFT073	++++	a	a	a	a	a	a	a	a	a	a	a	a	a	a	a	a	a	a	a	a	a	a	a	a
g	3508124	a	3637305	ns	a	clone D	++	a	g	g	g	g	a	g	g	g	g	g	g	g	g	g	g	g	g	g	g	g	g	g	g
c	3509103	a	3638284	ns	c	CFT073	++++	c	c	c	c	c	c	c	c	c	c	c	c	c	c	c	c	c	c	a	c	c	c	a	a
g	3529000	c	3658183	ns	g	CFT073	++++	g	g	g	g	g	g	g	g	g	g	g	g	g	g	g	g	g	g	g	g	g	g	g	g
t	3536702	c	3665885	s	c	clone D	++	c	t	t	t	t	c	c	c	c	c	c	c	c	c	c	c	c	c	c	c			c	c
c	3538980	t	3668163	s	c	CFT073	++	c	c	c	c	c	c	c	c	c	c	c	c	c	c	c	a	c	c	c	c		c	c	c
g	3546455	a	3675638	s	g	CFT073	++++	g	g	g	g	g	g	g	g	g	g	g	g	g	g	g	g	g	g	g	g	g	g	g	g
t	3555708	g	3684891	nc	g	clone D	++	g	g	g	g	g	a	g	g	g	g	g	g	g	g	g	g	g	g	g	g	-	g	g	g
g	3555892	c	3685075	nc	g	CFT073	++++	g	g	g	g	g	g	g	g	g	g	g	g	g	g	g	g	g	g	g	g	g	g	g	g
g	3562222	t	3691404	ns	?	?	+/-	-	-	-	-	-	-	-	-	-	-	-	-	-	-	-	-	-	-	-	-	-	-	-	-

Clone D base	Clone D site	CFT 073 base	CFT 073 site	mutation type[a]	Inferred ancestral base[b]	lineage inferred to mutate	Support level[c]	ED1a[d]	536	S88	APEC O1	UTI89	E2348/69	ATCC8739	K-12	HS	SMS3-5	IAI39	E24377A	SE11	55989	IAI1	Sakai	UMN026	B4 Sb227	F5 8401	SS Ss046	D1 Sd197	B18 BS512	F2a 2457T	F2a 301
a	3562224	g	3691406	ns	g	clone D	++++	g	g	g	g	g	g	g	g	g	g	g	g	g	g	g	g	g	g	g	g	g	g	g	g
c	3562225	a	3691407	ns	a	clone D	++++	a	a	a	a	a	a	a	a	a	a	a	a	a	a	a	a	a	a	a	a	a	a	a	a
t	3567137	c	3696321	ns	t	CFT073	++++	t	t	t	t	t	t	c	c	c	c	c	c	c	c	c	c	t	c	c	c	c	c	c	c
g	3567155	a	3696339	ns	?	?	+/-	-	-	-	-	-	-	-	-	-	-	-	-	-	-	-	-	-	-	-	-	-	-	-	-
t	3567156	g	3696340	ns	g	clone D	++++	g	g	g	g	g	g	g	g	g	g	g	g	g	g	g	g	g	g	g	g	g	g	g	g
a	3567159	c	3696343	s	c	clone D	++++	c	c	c	c	c	c	c	c	c	c	c	c	c	c	c	c	c	c	c	c	c	c	c	c
c	3577387	t	3706573	nc	a	clone D	++	a	t	c	c	c	t	t	t	t	t	t	t	t	t	t	t	t	t	t	t	t	t	t	t
c	3577761	g	3706947	ns	c	CFT073	++++	c	c	c	c	c	t	c	c	c	c	c	c	c	c	c	c	c	c	c	c	c	c	c	c
g	3584797	a	3713983	s	g	CFT073	++	g	g	g	g	g	g	g	g	g	g	g	g	g	g	g	g	g			g			g	g
c	3588877	g	3718063	ns	c	CFT073	++	c	c	c	c	c	c	c	c	c	c	c	c	c	c	c	c	c						c	c
g	3590882	a	3720068	s	g	CFT073	++	g	g	g	g	g	g	g		g	g	g	g	g	g	g	g	g						g	g
t	3601227	c	3730413	ns	c	clone D	++++	c	c	c	c	c	c	c	c	c	c	c	c	c	c	c	c	c	c	c	c	c	c	c	c
t	3603303	c	3732489	ns	t	CFT073	++++	t	t	t	t	t	t	t	t	t	t	t	t	t	t	t	t	t	t	t	t	t	t	t	t
a	3638598	g	3767785	s	a	CFT073	++++	a	a	a	a	a	g	g	g	g	g	g	g	g	g	g	g	g	g	g	g	g	g	g	g
a	3638601	g	3767788	s	g	clone D	++	g	a	a	a	a	g	g	g	g	g	g	g	g	g	g	g	g	g	g	g	g	g	g	g
g	3638947	a	3768134	ns	g	CFT073	++++	g	g	g	g	g	g	g	g	g	g	g	g	g	g	g	g	g	g	g	g	g	g	g	g
t	3652545	g	3781732	nc	t	CFT073	++++	t	t	t	t	t	t	t	t	t	t	t	t	t	t	t	t	t	t	t	t	t	t	t	t
t	3658455	c	3787642	nc	t	CFT073	++++	t	t	t	t	t	t	t	t	t	t	c	t	t	t	t	t	t	t	t	t	t	t	t	t
g	3667542	t	3796729	ns	g	CFT073	++++	g	g	g	g	g	g	g	g	g	g	g	g	g	g	g	g	g	g	g	g	g	g	g	g
t	3669095	c	3798282	ns	t	CFT073	++++	t	t	t	t	t	t	t	t	t	t	t	t	t	t	t	t	t	t	t	t	t	t	t	t
t	3671503	c	3800690	ns	c	clone D	++++	c	c	c	c	c	c	c	c	c	c	c	c	c	c	c	c	c	c	c	c	c	c	c	c
c	3696116	g	3825303	ns	c	CFT073	++++	c	c	c	c	c	c	c	c	c	c	c	c	c	c	c	c	c	c	c	c	c	c	c	c
t	3696462	g	3825649	ns	g	clone D	++++	g	g	g	g	g	g	g	g	g	g	g	g	g	g	g	g	g	g	g	g	g	g	g	g
a	3699268	c	3828455	nc	a	CFT073	++++	a	a	a	a	a	a	a	a	a	a	a	a	a	a	a	a	a	a	a	a	a	a	a	a
g	3700920	c	3830107	ns	g	CFT073	++++	g	g	g	g	g	g	g	g	g	g	g	g	g	g	g	g	g	g	g	g	g	g	g	g
t	3704606	c	3833793	s	c	clone D	++	c	c	c	c	c						c							c				c		
g	3710481	t	3839665	ns	g	CFT073	++++	g	g	g	g	g	g	g	g	g	g	g	g	g	g	g	g	g	g	g	g	g	g	g	g
c	3716028	t	3845212	ns	c	CFT073	++	c	c	c	c	c	c	c	c	c	c	c	c	c	c	c	c	c	c		c		c		

Clone D base	Clone D site	CFT 073 base	CFT 073 site	mutation type[a]	Inferred ancestral base[b]	lineage inferred to mutate	Support level[c]	ED1a[d]	536	S88	APEC O1	UTI89	E2348/69	ATCC8739	K-12	HS	SMS3-5	IAI39	E24377A	SE11	55989	IAI1	Sakai	UMN026	B4 Sb227	F5 8401	SS Ss046	D1 Sd197	B18 BS512	F2a 2457T	F2a 301
g	3717679	a	3846863	ns	g	CFT073	++++	g	g	g	g	g	g	g	g	g	g	g	g	g	g	g	g	g	g	g	g	g	g	g	g
t	3726285	g	3855223	nc	t	CFT073	++	t	t	t	t	t	t	t	g	g	t	c	t	c	g	g	c	t	c	t	t	t	c	t	t
t	3726400	c	3855338	nc	t	CFT073	+	a	a	a	t	t	c	t	c	t	t	t	t	t	c	t	c	t	t	c	c	t	t	c	c
c	3726403	t	3855341	nc	c	CFT073	++++	c	c	c	c	c	t	c	t	c	c	c	c	c	t	c	t	c	c	t	t	c	c	t	t
g	3726406	a	3855344	nc	a	clone D	++++	a	a	a	g	g	a	g	a	g	g	g	g	g	a	g	a	g	g	a	a	g	g	a	a
a	3726408	g	3855346	nc	a	CFT073	+	t	t	t	a	a	g	a	g	a	a	a	a	a	g	a	g	a	a	g	g	a	a	g	g
c	3727656	t	3856593	nc	c	CFT073	++++	c	c	c	t	c	c	t	t	t	t	t	t	t	t	t	t	t	t	t	t	t	t	t	t
a	3727657	g	3856594	nc	g	clone D	++++	g	a	g	g	g	g	g	g	g	g	g	g	g	g	g	g	g	g	g	g	g	g	g	g
g	3727698	a	3856635	nc	g	CFT073	++++	g	g	g	a	g	g	a	a	g	a	a	a	a	a	g	a	a	g	a	a	a	g	a	a
a	3727974	t	3856911	nc	a	CFT073	++++	a	a	a	t	a	a	t	t	t	t	t	t	t	t	t	t	t	t	t	t	t	t	t	t
c	3727975	g	3856912	nc	c	CFT073	++	c	c	c	g	c	c	g	g	g	g	a	g	a	g	g	g	a	a	a	g	a	a	a	a
g	3727984	c	3856921	nc	g	CFT073	++	g	g	g	c	g	g	c	c	c	c	t	c	t	c	c	c	t	c	c	c	t	t	c	c
c	3727985	a	3856922	nc	c	CFT073	++++	c	c	c	a	c	c	a	a	a	a	a	a	a	a	a	a	a	a	a	a	a	a	a	a
a	3727993	g	3856930	nc	a	CFT073	++++	a	a	a	g	a	a	a	g	g	a	a	a	a	g	g	a	g	a	a	g	a	a	a	a
a	3728026	g	3856963	nc	a	CFT073	++++	a	a	a	g	a	a	g	g	g	a	a	g	g	g	g	a	g	g	g	g	a	a	g	g
t	3728032	c	3856970	nc	t	CFT073	++++	t	t	t	c	t	t	c	c	c	t	t	c	c	c	c	t	c	c	c	c	t	t	c	c
c	3728651	a	3857589	nc	c	CFT073	++	c	c	-	-	-	c	-	a	a	c	c	a	a	a	a	a	-	a	a	a	-	a	a	a
c	3728653	g	3857591	nc	c	CFT073	++++	c	c	c	c	c	c	c	g	g	c	c	g	g	g	g	g	c	g	g	g	c	g	g	g
a	3728654	c	3857592	nc	a	CFT073	++++	a	a	c	c	c	a	c	c	c	a	a	c	c	c	c	a	c	c	c	c	c	c	c	c
t	3728655	c	3857593	nc	t	CFT073	++	t	t	a	a	a	t	a	c	c	t	t	c	c	c	c	t	a	c	c	c	a	c	c	c
a	3728660	c	3857598	nc	a	CFT073	++	a	a	g	g	g	a	g	c	c	a	a	c	c	c	c	c	g	c	c	c	g	c	c	c
a	3728845	g	3857784	nc	g	clone D	++	g	g	a	a	a	a	g	g	g	g	g	g	g	g	a	g	g	a	g	a	a	a	g	g
t	3728846	c	3857785	nc	t	CFT073	++++	t	c	t	t	t	t	c	c	c	t	t	c	c	c	t	c	c	t	t	t	t	t	t	t
a	3728886	g	3857825	nc	g	clone D	++++	g	g	g	a	g	g	g	g	g	g	a	g	g	g	a	g	g	a	a	g	g	a	a	a
c	3728938	g	3857877	nc	c	CFT073	++++	c	c	c	c	c	c	g	g	g	g	g	c	g	g	g	c	c	g	g	g	g	g	g	g
c	3729119	t	3858058	nc	c	CFT073	++++	c	t	c	t	c	c	t	t	t	t	t	t	t	t	t	t	t	t	t	t	t	t	t	t
g	3729224	c	3858165	nc	c	clone D	++++	c	c	c	c	c	c	c	c	c	c	c	c	c	c	c	c	c	c	c	c	c	c	c	c
c	3729225	a	3858166	nc	a	clone D	++++	a	a	a	a	a	a	a	a	a	a	a	a	a	a	a	a	a	a	a	a	a	a	a	a

Clone D base	Clone D site	CFT 073 base	CFT 073 site	mutation type[a]	Inferred ancestral base[b]	lineage inferred to mutate	Support level[c]	ED1a[d]	536	S88	APEC O1	UTI89	E2348/69	ATCC8739	K-12	HS	SMS3-5	IAI39	E24377A	SE11	55989	IAI1	Sakai	UMN026	B4 Sb227	F5 8401	SS Ss046	D1 Sd197	B18 BS512	F2a 2457T	F2a 301
c	3729271	t	3858201	nc	t	clone D	++++	t	t	t	t	t	c	t	t	t	t	t	t	t	c	c	c	t	t	t	t	t	t	t	t
a	3731286	g	3860228	nc	g	clone D	++++	g	g	g	g	g	g	g	g	g	g	g	g	g	g	g	g	g	g	g	g	g	g	g	g
g	3734598	t	3863540	ns	g	CFT073	++++	g	g	g	g	g	g	g	g	g	g	g	g	g	g	g	g	g	g	g	g	g	g	g	g
t	3744679	c	3873621	ns	c	clone D	++++	c	c	c	c	c	c	c	c	c	c	c	c	c	c	c	c	c	c	c	c	c	c	c	c
g	3784618	a	3913560	ns	a	clone D	++++	a	a	a	a	a	a	a	a	a	a	a	a	a	a	a	a	a	a	a	a	a	a	a	a
a	3854455	g	3983398	ns	a	CFT073	++++	a	a	a	a	a	a	a	a	a	a	a	a	a	a	a	a	a	a	a	a	a	a	a	a
g	3863638	a	3992581	ns	g	CFT073	++	g	g	g	g	g												g							
c	3892161	t	4021104	nc	c	CFT073	++	c	c	c	c	c		c	c	c		c	c	c	c	c		c	c				c		
c	3922606	t	4051555	ns	c	CFT073	++++	c	c	c	c	c	c	c	c	c	c	c	c	c	c	c	c	c	c	c	c	c	c	c	c
a	3928972	g	4057921	ns	g	clone D	++			g	g	g					g	g					g	g				g			
c	3930175	a	4059124	ns	c	CFT073	++			c	c	c					c	c					c	c				c			
g	3935166	a	4064114	ns	a	clone D	++++	a	a	a	a	a	a	a	a	a	a	a	a	a	a	a	a	a	a	a	a		a	a	a
g	3957995	a	4086943	s	g	CFT073	++	g	g	g	g	g	g				g	g					g	g				g			
a	3961763	g	4090711	ns	g	clone D	++	g	g	g	g	g	g				g	g					g	g				a			
c	3961856	t	4090804	ns	c	CFT073	++	c	c	c	c	c	c				c	c					c	c				c			
a	3975179	g	4104127	ns	g	clone D	++++	g	g	g	g	g	g	g	g	g	g	g	g	g	g	g	g	g	g	g	g	g	g	g	g
a	3985397	c	4114345	ns	c	clone D	++++	c	c	c	c	c	c	c	c	c	c	c	c	c	c	c	c	c	c	c	c	c	c	c	c
a	4018418	t	4147366	s	a	CFT073	++++	a	a	a	a	a	a	a	a	a	a	a	a	a	a	a	a	a	a	a	a	a	a	a	a
a	4018423	c	4147371	ns	a	CFT073	++	a	a	a	a	a	a	a	a	a	a	a	a	a	a	a	a	a	a	a	a	a	a	-	-
g	4018508	t	4147456	nc	g	CFT073	++++	g	g	g	g	g	g	g	g	g	g	g	g	g	t	g	g	t	t	g	t	g	t	g	t
a	4018509	g	4147457	nc	g	clone D	++++	g	g	g	g	g	g	g	g	g	g	g	g	g	g	g	g	g	g	g	g	g	g	g	g
c	4018510	a	4147458	nc	c	CFT073	++	c	c	c	c	c	c	c	c	c	c	c	c	c	g	c	c	g	g	-	g	c	g	-	g
a	4018511	c	4147459	nc	a	CFT073	++++	a	a	a	a	a	a	a	a	a	a	a	a	a	c	a	a	c	c	c	c	a	c	c	c
g	4018512	a	4147460	nc	g	CFT073	++++	g	g	g	g	g	g	g	g	g	g	g	g	g	a	g	g	a	a	a	a	g	a	a	a
g	4018536	a	4147484	nc	a	clone D	++	a	a	g	g	g	a	a	a	a	g	g	a	a	a	a	a	a	a	a	a	a	a	a	a
g	4034206	a	4163154	s	g	CFT073	++	g	g	g	g	g	g	g	g	g	a	a	g	g	g	g	g	a	g	g	g	g	g	g	g
c	4034217	a	4163165	s	c	CFT073	++++	c	c	c	c	c	c	c	c	c	c	c	c	c	c	c	c	c	c	c	c	c	c	c	c
g	4034221	a	4163169	s	g	CFT073	++++	g	g	g	g	g	g	g	g	g	g	g	g	g	g	g	g	g	g	g	g	g	g	g	g

Clone D base	Clone D site	CFT 073 base	CFT 073 site	mutation type[a]	Inferred ancestral base[b]	lineage inferred to mutate	Support level[c]	ED1a[d]	536	S88	APEC O1	UTI89	E2348/69	ATCC8739	K-12	HS	SMS3-5	IAI39	E24377A	SE11	55989	IAI1	Sakai	UMN026	B4 Sb227	F5 8401	SS Ss046	D1 Sd197	B18 BS512	F2a 2457T	F2a 301
g	4034252	c	4163200	ns	g	CFT073	++++	g	g	g	g	g	g	g	g	g	g	g	g	g	g	g	g	g	g	g	g	g	g	g	g
a	4034303	c	4163251	ns	a	CFT073	++++	a	a	a	a	a	a	a	a	a	a	a	a	a	a	a	a	a	a	a	a	a	a	a	a
t	4044235	c	4173183	ns	c	clone D	++++	c	c	c	c	c	c	c	c	c	c	c	c	c	c	c	c	c	c	c		c	c	c	c
t	4055331	c	4184279	ns	c	clone D	++	c	c	c	c	c	c	c	c	c	c	c	c	c	c	c		c		c				c	c
g	4059433	t	4188381	ns	g	CFT073	++	g	g	g	g	g	g	g	g	g	g	g	g	g	g	g		g		g					
a	4061881	g	4190829	ns	g	clone D	++	g	g	g	g	g	g	g	g	g	g	g	g	g	g	g	g	g	g	g			g	g	g
g	4068252	a	4197200	s	a	clone D	++++	a	a	a	a	a	a	a	a	a	a	a	a	a	a	a	a	a	a	a	a	a	a	a	a
a	4071641	g	4200589	ns	g	clone D	++++	g	g	g	g	g	g	g	g	g	g	g	g	g	g	g	g	g	g	g	g		g	g	g
t	4072205	c	4201153	ns	c	clone D	++++	c	c	c	c	c	c	c	c	c	c	c	c	c	c	c	c	c	c	c	c		c	c	c
g	4091151	a	4220099	ns	g	CFT073	++++	g	g	g	g	g	g	g	g	g	g	g	g	g	g	g	g	g	g	g	g	g	g	g	g
g	4094205	a	4223153	ns	a	clone D	++++	a	a	a	a	a	a	a	a	a	a	a	a	a	a	a	a	a	a	a	a	a	a	a	a
a	4102115	c	4231063	s	a	CFT073	++		a	a	a	a		a		a	a	a	a			a			a		a		a		
t	4102133	g	4231081	ns	t	CFT073	++		t	t	t	t		t		t	t	t	t			t			t		t		t		
a	4118049	c	4246997	ns	c	clone D	++++	c	c	c	c	c	t	c	c	c	c	c	c	c	c	c	c	c	c	c	c	c	c	c	c
t	4119400	c	4248348	ns	t	CFT073	++++	t	t	t	t	t	t	t	t	t	t	t	t	t	t	t	t	t	t	t	t	t	t	t	t
a	4141078	t	4270026	nc	?	?	+/-	-	-	-	-	-	-				-	-													
a	4145453	g	4274403	nc	a	CFT073	++	a	a	a	a	a	a		a	a		a	a	a	a	a	g	a	a	g	g	g	a	g	g
a	4145454	t	4274404	nc	a	CFT073	++	a	c	a	a	a	a		a	a		a	a	a	a	a	t	t	a	t	t	t	a	t	t
a	4145455	t	4274405	nc	a	CFT073	++	a	t	a	a	a	a		a	a		a	a	a	a	a	t	t	a	t	t	t	a	t	t
g	4145457	a	4274407	nc	g	CFT073	++	g	a	g	g	g	g		g	g		g	g	g	g	g	g	g	g	a	a	a	g	a	a
g	4145476	a	4274426	nc	g	CFT073	++	g	a	g	g	g	g					a					g			a	a	a		a	a
c	4145484	t	4274434	nc	c	CFT073	++	c	t	c	c	c	a					t					c			t	t	t		t	t
c	4145489	t	4274438	nc	t	clone D	+		c				c					t					c			t	t	t		t	t
c	4145491	t	4274440	nc	t	clone D	+		t				c					t					c			t	t	t		t	t
a	4145505	g	4274454	nc	g	clone D	+		g				a					g					a			g	g	g		g	g
g	4145514	t	4274463	nc	t	clone D	+		c				g					t					g			t	t	t		t	t
t	4145515	a	4274464	nc	a	clone D	+		g				c					a					t			a	a	a		a	a
c	4145519	a	4274468	nc	a	clone D	+		c				c					a					c			a	a	a		a	a

Clone D base	Clone D site	CFT 073 base	CFT 073 site	mutation type[a]	Inferred ancestral base[b]	lineage inferred to mutate	Support level[c]	ED1a[d]	536	S88	APEC O1	UTI89	E2348/69	ATCC8739	K-12	HS	SMS3-5	IAI39	E24377A	SE11	55989	IAI1	Sakai	UMN026	B4 Sb227	F5 8401	SS Ss046	D1 Sd197	B18 BS512	F2a 2457T	F2a 301
a	4145523	g	4274472	nc	g	clone D	+		g				g					g					g			g	g	g		g	g
t	4145529	a	4274478	nc	t	CFT073	+		a				a					t					t			t	t	a		t	t
g	4145540	t	4274489	nc	t	clone D	+		t				g					t					g			t	t	t		t	t
a	4145547	c	4274496	nc	c	clone D	+		c				a					c					a			c	c	c		c	c
a	4145551	g	4274500	nc	g	clone D	+		g				g					g					a			g	a	g		g	g
c	4145552	t	4274501	nc	t	clone D	+		t				t					t					t			t	t	t		t	t
g	4145561	t	4274510	nc	t	clone D	+		t				g					t					g			t	t	t		t	t
t	4145562	c	4274511	nc	c	clone D	+		c				c					c					t			c	c	c		c	c
g	4145568	t	4274517	nc	g	CFT073	+		g				g					g					g			g	g	g		g	g
g	4145573	a	4274522	nc	a	clone D	+		a				a					a					g			a	a	a		a	a
a	4145575	t	4274524	nc	t	clone D	+		g				t					t					a			t	t	t		t	t
c	4145578	a	4274527	nc	a	clone D	+		a				a					a					g			a	a	a		a	a
c	4145586	t	4274535	nc	t	clone D	+		t				t					t								t	t	t		t	t
c	4145587	t	4274536	nc	t	clone D	+		t				t					t								t	t	t		t	t
c	4145596	a	4274544	nc	a	clone D	+		g				c					a								a	a	a		a	a
a	4145599	g	4274547	nc	g	clone D	+		g				g					g								g	g	g		g	g
t	4145605	c	4274553	nc	c	clone D	+		a				t					c								c	c	c		c	c
g	4145621	a	4274569	nc	a	clone D	+		a				a					a								a	a	a		a	a
t	4145623	g	4274571	nc	g	clone D	+		g				g					g								g	g	g		g	g
g	4145634	t	4274582	nc	t	clone D	+		g				t					t								t	t	t		t	t
g	4145644	c	4274592	nc	c	clone D	+		c				t					c								c	c	c		c	c
g	4145647	c	4274595	nc	c	clone D	+		t				t					c								c	c	c		c	c
a	4145654	c	4274602	nc	c	clone D	+		c				c					c								c	c	c		c	c
g	4145655	a	4274603	nc	a	clone D	+		a				a					a								a	a	a		a	a
c	4145665	t	4274613	nc	t	clone D	+		t				t					t								t	t	t		t	t
t	4145670	c	4274618	nc	c	clone D	+		c				c					c								c	c	c		c	c
c	4145679	t	4274627	nc	t	clone D	+		t				c					t					c			t	t	t		t	t
g	4145697	a	4274645	nc	a	clone D	+		a									a					g			a	a	a		a	a

Clone D base	Clone D site	CFT 073 base	CFT 073 site	mutation type[a]	Inferred ancestral base[b]	lineage inferred to mutate	Support level[c]	ED1a[d]	536	S88	APEC O1	UTI89	E2348/69	ATCC8739	K-12	HS	SMS3-5	IAI39	E24377A	SE11	55989	IAI1	Sakai	UMN026	B4 Sb227	F5 8401	SS Ss046	D1 Sd197	B18 BS512	F2a 2457T	F2a 301
g	4145723	a	4274672	nc	a	clone D	+		a									a					g			a	a	a		a	a
g	4145724	c	4274673	nc	c	clone D	+		c									c					g			c	c	c		c	c
g	4145728	a	4274677	nc	a	clone D	+		a									a					g			a	a	a		a	a
g	4145758	a	4274707	s	a	clone D	+		a									a					g			a	a	a		a	a
c	4145759	t	4274708	s	t	clone D	+		t									t					c			t	t	t		t	t
g	4145768	a	4274717	ns	a	clone D	+		a									a					g			a	a	a		a	a
t	4145770	c	4274719	ns	c	clone D	+		c									c					t			c	c	c		c	c
g	4145785	a	4274734	s	a	clone D	+		a									a					g			a	a	a		a	a
g	4145788	a	4274737	s	a	clone D	+		a									a					g			a	a	a		a	a
a	4145791	c	4274740	s	c	clone D	+		a									c					a			c	c	c		c	c
a	4145797	c	4274746	ns	c	clone D	+		c									c					a			c	c	c		c	c
g	4145800	a	4274749	s	a	clone D	+		g									a					g			a	a	a		a	a
g	4145812	t	4274761	s	t	clone D	+		t									t					g			t	t	t		t	t
a	4145819	g	4274768	ns	g	clone D	+		g									g					g			g	g	g		g	g
g	4145836	a	4274785	s	a	clone D	+		a									a					g			a	a	a		a	a
g	4145846	a	4274795	ns	a	clone D	+		a									a					g			a	a	a		a	a
c	4145857	t	4274806	s	t	clone D	+		t									t					c			t	t	t		t	t
t	4145869	c	4274818	s	c	clone D	+		c									c					t			c	c	c		c	c
c	4145884	t	4274833	s	t	clone D	+		t									t					c			t	t	t		t	t
g	4145893	a	4274842	s	a	clone D	+		a									a					a			a	a	a		a	a
g	4145905	t	4274854	s	t	clone D	+		t									t					g			t	t	t		t	t
t	4145917	a	4274866	s	a	clone D	+		a									a					a			a	a	a		a	a
t	4145935	a	4274884	s	a	clone D	+		a									a					t			a	a	a		a	a
c	4145941	t	4274890	s	t	clone D	+		c									t					c			t	t	t		t	t
t	4145947	c	4274896	s	c	clone D	+		c									c					t			c	c	c		c	c
a	4145972	c	4274921	ns	c	clone D	+		a									c					a			c	c	c		c	c
a	4145974	c	4274923	ns	c	clone D	+		c									c					a			c	c	c		c	c
t	4145977	c	4274926	s	c	clone D	+		c									c					t			c	c	c		c	c

Clone D base	Clone D site	CFT 073 base	CFT 073 site	mutation type[a]	Inferred ancestral base[b]	lineage inferred to mutate	Support level[c]	ED1a[d]	536	S88	APEC O1	UTI89	E2348/69	ATCC8739	K-12	HS	SMS3-5	IAI39	E24377A	SE11	55989	IAI1	Sakai	UMN026	B4 Sb227	F5 8401	SS Ss046	D1 Sd197	B18 BS512	F2a 2457T	F2a 301
g	4145980	t	4274929	s	t	clone D	+		t									t					g			t	t	t		t	t
t	4145986	a	4274935	s	a	clone D	+		a									a					t			a	a	a		a	a
g	4145995	a	4274944	s	a	clone D	+		t									a					g			a	a	a		a	a
t	4145998	g	4274947	s	g	clone D	+		g									g					t			g	g	g		g	g
a	4146010	g	4274959	s	g	clone D	+		a									g					a			g	g	g		g	g
t	4146027	a	4274976	ns	a	clone D	+		t									a					t			a	a	a		a	a
t	4146038	c	4274987	s	c	clone D	+		t									c					t			c	c	c		c	c
a	4146041	c	4274990	ns	a	CFT073	+		a									a					a			a	a	a		a	a
c	4146046	t	4274995	s	t	clone D	+		c									t					c			t	t	t		t	t
t	4146049	g	4274998	s	g	clone D	+		g									g					t			g	g	g		g	g
c	4146058	t	4275007	s	t	clone D	+		t									t					c			t	t	t		t	t
g	4146059	a	4275008	ns	a	clone D	+		a									a					g			a	a	a		a	a
g	4146070	a	4275019	s	a	clone D	+		a									a					g			a	a	a		a	a
t	4146076	c	4275025	s	c	clone D	+		c									c					t			c	c	c		c	c
g	4146082	a	4275031	s	a	clone D	+		a									a					g			a	a	a		a	a
c	4146094	g	4275043	s	g	clone D	+		g									g					c			g	g	g		g	g
c	4146124	t	4275073	s	t	clone D	+		t									t					c			t	t			t	t
c	4146127	t	4275076	s	t	clone D	+		t									t					c			t	t			t	t
t	4146142	c	4275091	s	c	clone D	+		c									c					t			c	c			c	c
c	4146145	t	4275094	s	t	clone D	+		t									t					c			t	t			t	t
c	4146157	t	4275106	s	t	clone D	+		t									t					c			t	t			t	t
t	4146160	c	4275109	s	c	clone D	+		t									c					c			c	c			c	c
t	4146175	c	4275124	s	c	clone D	+		c									c					c			c	c			c	c
t	4146181	c	4275130	s	c	clone D	+		a									c					c			c	c			c	c
a	4146193	g	4275142	s	g	clone D	+		g									g					g			g	g			g	g
c	4146194	t	4275143	s	t	clone D	+		t									t					c			t	t			t	t
c	4146211	t	4275160	s	t	clone D	+		t									t					t			t	t			t	t
g	4146214	a	4275163	s	a	clone D	+		a									a					g			a	a			a	a

Clone D base	Clone D site	CFT 073 base	CFT 073 site	mutation type[a]	Inferred ancestral base[b]	lineage inferred to mutate	Support level[c]	ED1a[d]	536	S88	APEC O1	UTI89	E2348/69	ATCC8739	K-12	HS	SMS3-5	IAI39	E24377A	SE11	55989	IAI1	Sakai	UMN026	B4 Sb227	F5 8401	SS Ss046	D1 Sd197	B18 BS512	F2a 2457T	F2a 301
g	4146220	a	4275169	s	a	clone D	+		a									a					g			a	a			a	a
c	4146226	t	4275175	s	t	clone D	+		t									t					t			t	t			t	t
g	4146232	t	4275181	s	t	clone D	+		t									t					a			t	t			t	t
t	4146256	c	4275205	s	c	clone D	+		c									c					c			c	c			c	c
a	4146271	g	4275220	s	g	clone D	+		g									g					g			g	g			g	g
c	4146292	t	4275241	s	t	clone D	+		t									t					t			t	t			t	t
a	4146299	c	4275248	s	c	clone D	+		c									c					c			c	c			c	c
a	4146301	g	4275250	s	g	clone D	+		g									g					a			g	g			g	g
t	4146304	a	4275253	s	a	clone D	+		a									a					c			a	a			a	a
c	4146316	a	4275265	s	a	clone D	+		t									a					t			a	a			a	a
t	4146319	c	4275268	s	c	clone D	+		c									c					t			c	c			c	c
c	4146334	t	4275283	s	t	clone D	+		t									t					t			t	t			t	t
g	4146337	t	4275286	s	t	clone D	+		t									t					t			t	t			t	t
c	4146364	t	4275313	s	t	clone D	+		t									t					t			t	t			t	t
c	4146370	t	4275319	s	t	clone D	+		t									t					t			t	t			t	t
c	4146379	t	4275328	s	t	clone D	+		t									t					c			t	t			t	t
t	4146391	c	4275340	s	c	clone D	+		c									c					c			c	c			c	c
t	4146400	c	4275349	s	c	clone D	+		c									c					t			c	c			c	c
c	4146424	a	4275373	s	a	clone D	+		a									a					a			a	a			a	a
c	4146430	a	4275379	s	a	clone D	+		c									a					c			a	a			a	a
g	4146436	t	4275385	s	t	clone D	+		t									t					t			t	t			t	t
c	4146445	t	4275394	s	t	clone D	+		t									t					t			t	t			t	t
t	4146469	c	4275418	s	c	clone D	+		t									c					t			c	c			c	c
c	4146475	a	4275424	s	a	clone D	+		c									a					a			a	a			a	a
g	4146478	a	4275427	s	a	clone D	+		a									a					g			a	a			a	a
a	4146487	g	4275436	s	g	clone D	+		g									g					g			g	g			g	g
c	4146493	t	4275442	s	t	clone D	+		c									t					c			t	t			t	t
g	4146505	a	4275454	s	a	clone D	+		g									a					g			a	a			a	a

Clone D base	Clone D site	CFT 073 base	CFT 073 site	mutation type[a]	Inferred ancestral base[b]	lineage inferred to mutate	Support level[c]	ED1a[d]	536	S88	APEC O1	UTI89	E2348/69	ATCC8739	K-12	HS	SMS3-5	IAI39	E24377A	SE11	55989	IAI1	Sakai	UMN026	B4 Sb227	F5 8401	SS Ss046	D1 Sd197	B18 BS512	F2a 2457T	F2a 301
a	4146511	c	4275460	s	c	clone D	+		c									c					t			c	c			c	c
c	4146527	t	4275476	s	t	clone D	+		t									t					t			t	t			t	t
a	4146529	g	4275478	s	g	clone D	+		g									g					a			g	g			g	g
g	4146550	a	4275499	s	a	clone D	+		a									a					a			a	a			a	a
a	4146562	g	4275511	s	g	clone D	+		g									g					a			g	g			g	g
g	4146565	a	4275514	s	a	clone D	+		a									a					g			a	a			a	a
c	4146568	t	4275517	s	t	clone D	+		t									t					t			t	t			t	t
a	4146586	t	4275535	s	t	clone D	+		t									t					t			t	t			t	t
a	4146589	g	4275538	s	g	clone D	+		g									g					g			g	g			g	g
t	4146595	c	4275544	s	c	clone D	+		c									t					t			c	c			c	c
c	4146600	t	4275549	ns	t	clone D	+		c									t					c			t	t			t	t
g	4146604	a	4275553	s	a	clone D	+		a									a					a			a	a			a	a
a	4146610	g	4275559	s	g	clone D	+		g									g					g			g	g			g	g
g	4146622	a	4275571	s	a	clone D	+		a									a					a			a	a			a	a
a	4146625	c	4275574	s	c	clone D	+		c									c					c			c	c			c	c
g	4146640	c	4275589	s	c	clone D	+		t									c					t			c	c			c	c
g	4146643	a	4275592	s	a	clone D	+		g									a					g			a	a			a	a
c	4146655	t	4275604	s	t	clone D	+		t									t					t			t	t			t	t
c	4146661	t	4275610	s	t	clone D	+		t									t					c			t	t			t	t
g	4146673	a	4275622	s	a	clone D	+		g									a					g			a	a			a	a
t	4146676	a	4275625	s	a	clone D	+		a									a					a			a	a			a	a
c	4146679	t	4275628	s	t	clone D	+		t									t					t			t	t			t	t
t	4146691	c	4275640	s	c	clone D	+		c									c					c			c	c			c	c
g	4146692	a	4275641	ns	a	clone D	+		a									a					a			a	a			a	a
t	4146697	c	4275646	s	c	clone D	+		c									t					c			c	c			c	c
g	4146700	a	4275649	s	a	clone D	+		a									a					g			a	a			a	a
t	4146703	a	4275652	s	a	clone D	+		t									a					c			a	a			a	a
c	4146706	t	4275655	s	t	clone D	+		t									t					t			t	t			t	t

Clone D base	Clone D site	CFT 073 base	CFT 073 site	mutation type[a]	Inferred ancestral base[b]	lineage inferred to mutate	Support level[c]	ED1a[d]	536	S88	APEC O1	UTI89	E2348/69	ATCC8739	K-12	HS	SMS3-5	IAI39	E24377A	SE11	55989	IAI1	Sakai	UMN026	B4 Sb227	F5 8401	SS Ss046	D1 Sd197	B18 BS512	F2a 2457T	F2a 301
g	4146715	t	4275664	s	t	clone D	+		a									t					g			t	t			t	t
a	4146724	g	4275673	s	g	clone D	+		a									g					g			g	g			g	g
c	4146727	a	4275676	s	a	clone D	+		a									a					a			a	a			a	a
g	4146730	a	4275679	s	a	clone D	+		a									a					a			a	a			a	a
g	4146733	c	4275682	s	c	clone D	+		c									c					c			c	c			c	c
g	4146736	a	4275685	s	a	clone D	+		a									a					a			a	a			a	a
c	4146749	a	4275698	s	a	clone D	+		a									a					a			a	a			a	a
c	4146751	a	4275700	s	a	clone D	+		a									a					g			a	a			a	a
g	4146757	c	4275706	s	c	clone D	+		t									c					c			c	c			c	c
t	4146766	a	4275715	s	a	clone D	+		a									a					a			a	a			a	a
a	4146779	c	4275728	ns	c	clone D	+		c									c					c			c	c			c	c
c	4146790	t	4275739	s	t	clone D	+		t									c					t			t	t			t	t
a	4146793	c	4275742	s	c	clone D	+		c									c					t			c	c			c	c
g	4146796	c	4275745	s	c	clone D	+		c									c					c			c	c			c	c
c	4146807	t	4275756	ns	t	clone D	+		c									t					t			t	t			t	t
g	4163785	t	4341851	nc	?	?	+/-	a	t	a	a	a						t					t								
c	4163807	t	4341873	nc	t	clone D	++	t	t	t	t	t						t					t								
a	4163808	c	4341874	nc	c	clone D	++	c	c	c	c	c						c					c								
t	4163809	c	4341875	nc	?	?	+/-	a	c	a	a	a						c					c								
a	4163816	g	4341882	nc	a	CFT073	++	a	a	a	a	a						g					c								
a	4163819	t	4341885	nc	t	clone D	++	t	t	t	t	t						t					t								
a	4163826	t	4341892	nc	t	clone D	++	t	t	t	t	t						t					t								
t	4163828	a	4341894	nc	?	?	+/-	-	a	-	-	-						a					a								
t	4163836	a	4341902	nc	t	CFT073	++	t	t	t	t	t						a					t								
a	4163838	c	4341904	nc	a	CFT073	++	a	t	a	a	a						c					c								
c	4163845	a	4341911	nc	c	CFT073	++	c	a	c	c	c						a					a								
a	4163846	c	4341912	nc	a	CFT073	++	a	c	a	a	a						c					a								
t	4163847	c	4341913	nc	t	CFT073	++	t	t	t	t	t						t					t								

Clone D base	Clone D site	CFT 073 base	CFT 073 site	mutation type[a]	Inferred ancestral base[b]	lineage inferred to mutate	Support level[c]	ED1a[d]	536	S88	APEC O1	UTI89	E2348/69	ATCC8739	K-12	HS	SMS3-5	IAI39	E24377A	SE11	55989	IAI1	Sakai	UMN026	B4 Sb227	F5 8401	SS Ss046	D1 Sd197	B18 BS512	F2a 2457T	F2a 301
a	4163850	t	4341916	nc	a	CFT073	++	a	t	a	a	a						t					t								
g	4163853	t	4341919	nc	g	CFT073	++	g	t	g	g	g						t					t								
g	4164317	a	4342383	s	g	CFT073	+++	g	g	g	g	g																			
c	4173011	a	4351077	ns	c	CFT073	++++	c	c	c	c	c	c	c	c	c	c	c	c	c	c	c	c	c	c	c	c	c	c	c	c
t	4186694	c	4364760	s	t	CFT073	++	t	t	t	t	t	t		t		t	t	t	t	t	t	t	t		t		t		t	t
t	4193039	g	4371105	ns	g	clone D	++	g	g	g	g	g	g	g	g	g	g	g	g	g	g	g	g	g		g	g	g		g	g
t	4199513	a	4377580	ns	t	CFT073	++	t	t	t	t	t	t	t	t	t	t	t	t	t	t	t	t	t		t	t			t	t
t	4208516	c	4386584	ns	t	CFT073	++++	t	t	t	t	t	t	t	t	t	t	t	t	t	t	t	t	t	t	t	t	t	t	t	t
a	4232942	c	4411010	ns	a	CFT073	++++	a	a	a	a	a	a	a	a	a	a	a	a	a	a	a	a	a	a	a	a	a	a	a	a
c	4233021	a	4411089	ns	c	CFT073	++++	c	c	c	c	c	c	c	c	c	c	c	c	c	c	c	c	c	c	c	c	c	c	c	c
c	4233031	g	4411099	s	c	CFT073	++++	c	c	c	c	c	c	c	c	c	c	t	c	c	c	c	c	c	c	c	c	c	c	c	c
c	4233035	g	4411103	ns	c	CFT073	++++	c	c	c	c	c	c	c	c	c	c	c	c	c	c	c	c	c	c	c	c	c	c	c	c
g	4247246	t	4425314	s	g	CFT073	++++	g	g	g	g	g	g	g	g	g	g	g	g	g	g	g	g	g	g	g	g	g	g	g	g
t	4265908	c	4443976	nc	c	clone D	++++	c	c	c	c	c	c	c	c	c	c	c	c	c	c	c	c	c	c	c	c	c	c	c	c
g	4266132	a	4444201	nc	g	CFT073	++++	g	a	g	g	g	g	g	g	g	g	g	g	g	g	g	g	g	g	g	g	g	g	g	g
c	4266214	a	4444283	nc	c	CFT073	++++	c	a	c	c	c	c	c	c	c	c	c	c	c	c	c	c	c	c	c	c	c	c	c	c
c	4266223	a	4444292	nc	c	CFT073	++++	c	a	c	c	c	c	c	c	c	c	c	c	c	c	c	c	c	c	c	c	c	c	c	c
c	4266231	t	4444300	nc	c	CFT073	++++	c	t	c	c	c	c	c	c	c	c	c	c	c	c	c	c	c	c	c	c	c	c	c	c
c	4266250	t	4444319	nc	c	CFT073	++++	c	t	c	c	c	c	c	c	c	c	c	c	c	c	c	c	c	c	c	c	c	c	c	c
c	4266255	g	4444324	nc	c	CFT073	++++	c	g	c	c	c	c	c	c	c	c	c	c	c	c	c	c	c	c	c	c	c	c	c	c
g	4266257	t	4444326	nc	g	CFT073	++++	g	t	g	g	g	g	g	g	g	g	g	g	g	g	g	g	g	g	g	g	g	g	g	g
t	4266261	g	4444330	nc	t	CFT073	++++	t	g	t	t	t	t	t	t	t	t	t	t	t	t	t	t	t	t	t	t	t	t	t	t
g	4266262	t	4444331	nc	g	CFT073	++++	g	t	g	g	g	g	g	g	g	g	g	g	g	g	g	g	g	g	g	g	g	g	g	g
a	4266328	t	4444397	nc	a	CFT073	++++	a	t	a	a	a	a	t	t	t	a	a	t	t	t	t	a	t	t	t	a	t	a	t	t
a	4266360	g	4444429	nc	a	CFT073	++++	a	a	a	a	a	a	a	g	a	a	a	a	a	a	a	a	a	a	a	a	a	a	a	a
g	4266598	a	4444667	nc	g	CFT073	++++	g	a	a	a	g	g	a	a	g	a	a	a	g	g	a	a	a	a	a	a	a	a	a	a
c	4266645	a	4444714	nc	c	CFT073	++++	c	a	c	c	c	c	a	a	a	a	a	c	c	a	c	a	a	a	a	a	a	t	a	a
g	4266750	a	4444819	nc	g	CFT073	++++	g	a	g	g	g	g	a	a	a	a	g	a	a	a	a	a	a	a	a	a	a	a	a	a

Clone D base	Clone D site	CFT 073 base	CFT 073 site	mutation type[a]	Inferred ancestral base[b]	lineage inferred to mutate	Support level[c]	ED1a[d]	536	S88	APEC O1	UTI89	E2348/69	ATCC8739	K-12	HS	SMS3-5	IAI39	E24377A	SE11	55989	IAI1	Sakai	UMN026	B4 Sb227	F5 8401	SS Ss046	D1 Sd197	B18 BS512	F2a 2457T	F2a 301
g	4266931	c	4445000	nc	g	CFT073	++++	g	g	g	g	g	g	g	c	g	g	g	g	c	c	c	g	c	c	c	g	c	c	c	c
g	4266952	a	4445021	nc	g	CFT073	++++	g	g	g	g	g	g	g	a	g	g	g	g	g	g	g	g	g	g	g	g	g	g	g	g
t	4266983	c	4445052	nc	c	clone D	++++	c	c	t	c	c	c	c	c	c	c	t	t	t	t	t	c	c	t	t	c	c	t	t	t
t	4267209	g	4445280	nc	t	CFT073	++	t	t	t	c	c	t	g	g	g	t	t	g	g	g	g	g	c	g	g	g	t	g	g	g
a	4267837	g	4445908	nc	a	CFT073	++++	a	a	a	a	a	a	g	g	g	g	a	g	a	g	g	a	a	g	g	g	a	a	g	g
t	4267843	c	4445915	nc	t	CFT073	++++	t	t	t	t	t	t	c	c	c	c	t	c	t	c	c	t	t	c	c	c	t	t	c	c
t	4267876	c	4445948	nc	t	CFT073	++++	t	t	t	t	t	t	t	c	t	t	t	t	t	t	c	t	t	t	t	c	t	t	t	t
g	4267884	t	4445956	nc	g	CFT073	++++	g	t	g	g	g	g	t	t	t	t	t	t	t	t	t	t	t	t	t	t	t	t	t	t
c	4267885	g	4445957	nc	c	CFT073	++	c	g	c	c	c	c	g	g	g	a	a	g	a	a	g	g	a	g	g	g	a	a	g	g
g	4267894	c	4445966	nc	g	CFT073	++	g	c	g	g	g	g	c	c	c	t	t	c	t	t	c	c	t	t	t	c	t	t	t	t
t	4267895	a	4445967	nc	t	CFT073	++++	t	a	t	t	t	t	a	a	a	a	a	a	a	a	a	a	a	a	a	a	a	a	a	a
c	4268171	t	4446243	nc	c	CFT073	++++	c	t	c	c	c	c	t	t	t	t	t	t	t	t	t	t	t	t	t	t	t	c	t	t
g	4268213	a	4446285	nc	g	CFT073	++++	g	a	g	g	g	g	a	a	a	a	a	a	a	a	a	a	a	a	a	a	a	a	a	a
t	4269751	g	4447824	nc	t	CFT073	++++	t	t	t	t	t	t	t	t	t	t	t	t	t	t	t	t	t	t	t	t	t	t	t	t
c	4270509	t	4448582	s	c	CFT073	++++	c	c	c	c	c	c	c	c	c	c	c	c	c	c	c	c	c	c	c	c	c	c	c	c
a	4289827	c	4467901	ns	c	clone D	++++	c	c	c	c	c	c	c	c	c	c	c	c	c	c	c	c	c	c	c	c	c	c	c	c
g	4323599	a	4501680	s	g	CFT073	++++	g	g	g	g	g	g	g	g	g	g	g	g	g	g	g	g	g	g	g	g	g	g	g	g
a	4383374	g	4561456	nc	a	CFT073	++++	a	a	a	g	a	a	a	a	a	a	a	a	a	a	a	a	a	a	a	a	a	a	a	a
a	4383463	g	4561545	nc	g	clone D	++++	g	g	g	g	g	g	g	g	g	g	g	g	g	g	g	g	g	g	g	g	g	g	g	g
a	4384669	t	4562751	nc	a	CFT073	++++	a	a	t	t	t	a	a	t	t	g	a	a	a	t	t	a	t	a	t	a	a	a	t	t
c	4384675	a	4562757	nc	a	clone D	++	a	a	t	a	t	a	c	a	c	c	c	c	c	a	a	c	a	c	t	c	t	c	t	t
g	4385091	a	4563256	nc	g	CFT073	++++	g	a	g	a	g	g	a	a	a	a	a	a	a	a	a	a	a	a	a	a	a	a	a	a
g	4385272	c	4563437	nc	g	CFT073	++++	g	g	g	c	g	g	c	c	g	g	c	g	c	c	c	g	g	c	c	c	c	c	c	c
g	4386383	t	4564552	nc	g	CFT073	++++	g	g	g	t	g	g	g	g	g	g	g	g	g	g	g	g	g	g	g	g	g	g	g	g
g	4386427	a	4564596	nc	g	CFT073	++++	g	g	g	a	g	g	g	g	g	g	g	g	g	g	g	g	g	g	g	g	g	g	g	g
c	4386512	t	4564681	nc	c	CFT073	++++	c	c	c	t	c	c	t	t	t	t	t	t	t	t	c	t	t	c	t	t	t	c	t	t
g	4386554	a	4564723	nc	g	CFT073	++++	g	g	g	a	g	g	a	a	a	a	a	a	a	a	a	a	a	a	a	a	a	a	a	a
g	4386875	c	4565044	nc	g	CFT073	++	g	g	g	c	g	g	g	c	c	g	g	c	c	c	c	c	c	c	-	-	c	c	c	-

Clone D base	Clone D site	CFT 073 base	CFT 073 site	mutation type[a]	Inferred ancestral base[b]	lineage inferred to mutate	Support level[c]	ED1a[d]	536	S88	APEC O1	UTI89	E2348/69	ATCC8739	K-12	HS	SMS3-5	IAI39	E24377A	SE11	55989	IAI1	Sakai	UMN026	B4 Sb227	F5 8401	SS Ss046	D1 Sd197	B18 BS512	F2a 2457T	F2a 301
t	4386877	c	4565046	nc	c	clone D	++++	c	t	t	c	t	t	t	c	c	t	t	c	c	c	c	c	c	c	c	c	c	c	c	c
c	4386880	g	4565049	nc	c	CFT073	++	c	c	c	g	c	c	c	g	-	c	c	-	-	-	-	-	-	-	g	g	-	-	-	g
g	4412393	c	4590561	s	c	clone D	++		c	c	c	c	c	c		c					c				c		c		c		
t	4414684	a	4592852	s	t	CFT073	++		t	t	t	t	t	t		t					t				t		t		t		
c	4436307	t	4614475	ns	c	CFT073	++++	c	c	c	c	c	c	c	c	c	c	c	c	c	c	c	c	c	c	c	c	c		c	c
a	4436680	g	4614848	s	g	clone D	++	g	g	g	g	g	g	g	g	g	g	g	g	g	g	g	g	g	g	g	g			g	g
g	4450344	t	4628512	s	g	CFT073	++	g	g	g	g	g	g	g	g	g	g	g	g	g	g	g	g	g	g	g	g	g	g	g	g
c	4456745	t	4634913	ns	t	clone D	++++	t	t	t	t	t	t	t	t	t	t	t	t	t	t	t	t	t	t	t	t	t	t	t	t
t	4466828	a	4644996	s	a	clone D	++	a	a	a	a	a	a	g	g	g	a	a	a	g	a	a	a	a	g	a	a	a	g	a	a
a	4484996	g	4663164	s	g	clone D	++++	g	g	g	g	g	g	g	g	g	g	g	g	g	g	g	g	g	g	g	g	g	g	g	g
g	4516087	t	4694255	s	g	CFT073	++++	g	g	g	g	g	g	g	g	g	g	g	g	g	g	g	g	g	g	g	g	g	g	g	g
t	4517164	g	4695332	ns	t	CFT073	++++	t	t	t	t	t	t	t	t	t	t	t	t	t	t	t	t	t	t	t	t	t	t	t	t
c	4519756	a	4697924	s	c	CFT073	++++	c	c	c	c	c	c	c	c	c	c	c	c	c	c	c	c	c	c	c	c	c	c	c	c
g	4520807	a	4698975	nc	g	CFT073	++++	g	a	a	a	a	a	a	a	a	a	a	a	a	a	a	a	a	a	a	a	a	a	a	a
t	4520842	a	4699009	nc	a	clone D	++++	a	a	a	a	a	a	a	a	a	a	a	a	a	a	a	a	a	a	a	a	a	a	a	a
a	4520877	t	4699045	nc	t	clone D	++	t	t	t	t	t	t	t	t	t	t	t	t	t	t	t	t	t	t	t	t	t	t	t	t
c	4520878	t	4699047	nc	t	clone D	++	t	t	t	t	t	t	t	t	t	t	t	t	t	t	t	t	t	t	t	t	t	t	t	t
a	4520883	g	4699052	nc	a	CFT073	++++	a	g	a	a	a	a	a	g	a	a	a	a	a	a	a	a	a	a	a	a	a	a	a	a
c	4520973	a	4699142	nc	c	CFT073	++++	c	a	c	c	c	c	c	a	a	a	c	c	a	c	c	a	a	c	c	c	c	c	c	c
g	4520982	t	4699151	nc	g	CFT073	++++	g	t	g	g	g	g	g	t	t	t	g	g	t	g	g	t	t	g	g	g	g	g	g	g
a	4521157	c	4699326	nc	a	CFT073	++	a	c	c	c	c	c	c	c	c	c	c	c	c	c	c	c	c	c	c	c	c	c	c	c
g	4522043	a	4700212	nc	a	clone D	++++	a	a	a	a	a	a	a	a	a	a	a	a	a	a	a	a	a	a	a	a	a	a	a	a
g	4522236	c	4700409	nc	g	CFT073	++++	g	c	g	c	g	g	g	g	g	g	g	g	g	g	g	g	g	g	g	g	g	g	g	g
a	4522289	c	4700467	nc	a	CFT073	++++	a	c	a	c	a	a	a	a	a	a	a	a	a	a	a	a	a	a	a	a	a	a	a	a
c	4522456	t	4700634	nc	c	CFT073	+	a	t	c	t	c	c	t	t	a	a	c	a	c	a	a	c	c	c	t	c	a	c	t	t
c	4522859	a	4701031	nc	c	CFT073	++	c	a	c	a	c	c	a	a	a	c	a	a	a	a	a	a	a	t	a	a	a	t	a	a
g	4522964	a	4701136	nc	g	CFT073	++++	g	a	g	a	g	g	a	a	a	a	g	a	a	a	a	a	a	a	a	a	a	a	a	a
g	4523145	c	4701317	nc	g	CFT073	++++	g	g	g	c	g	g	c	c	c	g	g	c	c	c	c	g	c	c	c	c	c	c	c	c

Clone D base	Clone D site	CFT 073 base	CFT 073 site	mutation type[a]	Inferred ancestral base[b]	lineage inferred to mutate	Support level[c]	ED1a[d]	536	S88	APEC O1	UTI89	E2348/69	ATCC8739	K-12	HS	SMS3-5	IAI39	E24377A	SE11	55989	IAI1	Sakai	UMN026	B4 Sb227	F5 8401	SS Ss046	D1 Sd197	B18 BS512	F2a 2457T	F2a 301
t	4523197	c	4701369	nc	c	clone D	++++	c	c	c	c	c	c	c	c	c	c	t	c	t	c	c	c	c	t	t	t	c	t	t	t
a	4523237	g	4701409	nc	a	CFT073	++++	a	g	a	g	a	a	g	g	g	a	a	g	g	a	g	g	a	a	a	a	a	a	a	a
t	4523238	c	4701410	nc	c	clone D	++	c	c	t	c	t	t	c	c	c	c	c	c	c	t	c	c	c	t	c	t	t	t	c	c
t	4523423	g	4701596	nc	t	CFT073	++	t	g	c	g	c	t	c	g	g	t	t	g	g	g	g	g	t	g	g	g	c	g	g	g
a	4524051	g	4702224	nc	a	CFT073	++++	a	g	a	g	a	a	g	g	g	a	a	a	a	g	g	a	g	g	g	g	a	a	g	g
t	4524057	c	4702231	nc	t	CFT073	++++	t	c	t	c	t	t	c	c	c	t	t	t	t	c	c	t	c	c	c	c	t	t	c	c
t	4524090	c	4702264	nc	t	CFT073	++++	t	c	t	c	t	t	t	c	c	t	t	t	t	c	c	t	c	t	t	c	t	t	t	t
g	4524098	t	4702272	nc	g	CFT073	++++	g	t	g	t	g	g	t	t	t	t	t	t	t	t	t	t	t	t	t	t	t	t	t	t
c	4524099	g	4702273	nc	c	CFT073	++	c	g	c	g	c	c	g	g	g	a	a	a	a	g	g	g	g	g	g	g	a	a	g	g
g	4524108	c	4702282	nc	g	CFT073	++	g	c	g	c	g	g	c	c	c	t	t	t	t	c	c	c	c	t	t	c	t	t	t	t
t	4524109	a	4702283	nc	t	CFT073	++++	t	a	t	a	t	t	a	a	a	a	a	a	a	a	a	a	a	a	a	a	a	a	a	a
c	4524385	t	4702559	nc	c	CFT073	++++	c	t	c	t	c	c	t	t	c	t	t	t	t	t	t	t	t	c	t	t	t	c	t	t
g	4524427	a	4702601	nc	g	CFT073	++++	g	a	g	a	g	g	a	a	a	a	a	a	a	a	a	a	a	a	a	a	a	a	a	a
a	4524486	c	4702660	nc	c	clone D	++++	c	c	c	c	c	c	c	c	c	c	c	c	c	c	c	c	c	c	c	c	c	c	c	c
t	4524750	c	4702923	nc	c	clone D	++	c	c	g	c	g	g	c	c	c	g	g	c	c	c	c	c	g	c	c	c	c	c	c	c
c	4525066	t	4703238	nc	t	clone D	++++	t	t	t	t	t	t	t	t	t	t	t	t	t	t	t	t	t	t	t	t	t	t	t	t
t	4526087	c	4704259	nc	c	clone D	++++	c	c	c	c	c	c	c	c	c	c	c	c	c	c	c	c	c	c	c	c	c	c	c	c
g	4561232	a	4739404	nc	a	clone D	++	a	a	g	a	g	g	g	g	a	g	g	g	g	g	a	g	g	g	g	g	a	g	g	g
t	4561235	g	4739407	nc	g	clone D	++	g	g	g	g	g	a	g	g	g	g	a	g	g	a	g	g	g	t	a	a	g	a	g	g
a	4561246	c	4739418	nc	c	clone D	++	c	c	t	c	c	t	c	c	c	c	t	t	c	t	c	c	c	a	t	t	c	t	t	t
a	4561786	g	4739958	nc	a	CFT073	++++	a	a	a	g	a	a	a	a	a	a	a	a	a	a	a	a	a	a	a	a	a	a	a	a
t	4562418	c	4740590	nc	c	clone D	++++	c	c	c	c	c	c	c	c	c	c	c	c	c	c	c	c	a	c	c	c	c	c	c	c
c	4562781	t	4740953	nc	t	clone D	++++	t	t	t	t	t	t	t	t	t	t	t	t	t	t	t	t	t	t	t	t	t	t	t	t
g	4562983	a	4741156	nc	g	CFT073	++++	g	a	g	a	g	g	g	a	g	a	a	a	g	g	g	g	a	a	a	a	a	a	a	a
c	4563030	t	4741203	nc	c	CFT073	++	c	a	c	t	c	c	a	t	a	a	t	c	c	a	a	a	a	t	a	a	t	t	a	a
g	4563135	a	4741308	nc	g	CFT073	++++	g	a	g	a	g	g	a	a	a	a	g	a	a	a	a	a	a	a	a	a	a	a	a	a
a	4563392	c	4741565	nc	a	CFT073	++++	a	a	a	c	a	a	a	a	a	a	a	a	a	a	a	a	a	a	a	a	a	a	a	a
a	4564222	g	4742395	nc	a	CFT073	++++	a	a	a	g	a	a	g	g	g	a	a	g	a	g	g	a	g	g	g	g	a	a	g	g

Clone D base	Clone D site	CFT 073 base	CFT 073 site	mutation type[a]	Inferred ancestral base[b]	lineage inferred to mutate	Support level[c]	ED1a[d]	536	S88	APEC O1	UTI89	E2348/69	ATCC8739	K-12	HS	SMS3-5	IAI39	E24377A	SE11	55989	IAI1	Sakai	UMN026	B4 Sb227	F5 8401	SS Ss046	D1 Sd197	B18 BS512	F2a 2457T	F2a 301
t	4564228	c	4742402	nc	t	CFT073	++++	t	t	t	c	t	t	c	c	c	t	t	c	t	c	c	t	c	c	c	c	t	t	c	c
t	4564261	c	4742435	nc	t	CFT073	++++	t	t	t	c	t	t	t	c	c	c	t	t	t	t	c	t	c	t	t	c	t	t	t	t
g	4564269	t	4742443	nc	g	CFT073	++++	g	g	g	t	g	g	t	t	t	t	t	t	t	t	t	t	t	t	t	t	t	t	t	t
c	4564270	g	4742444	nc	c	CFT073	++	c	c	c	g	c	c	g	g	g	a	a	g	a	a	g	g	g	g	g	g	a	a	g	g
g	4564279	c	4742453	nc	g	CFT073	++	g	g	g	c	g	g	c	c	c	t	t	c	t	t	c	c	c	t	t	c	t	t	t	t
t	4564280	a	4742454	nc	t	CFT073	++++	t	t	t	a	t	t	a	a	a	a	a	a	a	a	a	a	a	a	a	a	a	a	a	a
a	4564657	c	4742831	nc	c	clone D	++++	c	c	c	c	c	c	c	c	c	c	c	c	c	c	c	c	c	c	c	c	c	c	c	c
c	4569851	a	4748027	ns	c	CFT073	++++	c	c	c	c	c	c	c	c	c	c	c	c	c	c	c	c	c	c	c	c	c	c	c	c
c	4585244	t	4763420	s	c	CFT073	++	c	c	c	c	c	c				c	c					c	c	c	c	c		c	c	c
t	4590689	c	4768865	ns	c	clone D	++++	c	c	c	c	c	c	c	c	c	c	c	c	c	c	c	c	c	c	c	c	c	c	c	c
a	4602361	t	4780537	nc	a	CFT073	++	a	a	a	a	a	a	a	a	a	a	a	a	a	a	a	a	a		a	a			a	a
c	4622406	a	4800582	s	c	CFT073	+			c	c	c																			
t	4626099	g	4804275	s	t	CFT073	++++	t	t	t	t	t	t	t	t	t	t	t	t	t	t	t	t	t	t	t	t	t	t	t	t
t	4627342	g	4805518	s	g	clone D	+		g	g	g	g	g																		
t	4627905	c	4806081	s	c	clone D	+		c	c	c	c	c																		
a	4631749	g	4809925	s	a	CFT073	+++	a	a	a	a	a	a																		
t	4633577	a	4811753	ns	t	CFT073	+++	t	t	t	t	t	t																		
t	4655252	c	4833472	s	c	clone D	++++	c	c	c	c	c	c	c	c	c	c	c	c	c	c	c	c	c	c	c	c	c	c	c	c
c	4658923	t	4837143	ns	c	CFT073	++++	c	c	c	c	c	c	c	c	c	c	c	c	c	c	c	c	c	c	c	c		c	c	c
t	4667340	c	4845560	s	c	clone D	++++	c	c	c	c	c	c	c	c	c	c	c	c	c	c	c	c	c	c	c	c		c	c	c
c	4668303	t	4846523	ns	t	clone D	++++	t	t	t	t	t	t	t	t	t	t	t	t	t	t	t	t	t	t	t	t		t	t	t
g	4671386	a	4849606	nc	a	clone D	++	a	a					a	a																
a	4681579	t	4859788	s	t	clone D	++	t	t	t	t	t	t	t	t	t	t	t	t	t	t	t	t	t	t	t	t			t	t
c	4685538	a	4863747	nc	c	CFT073	++++	c	a	c	c	c	c	c	c	c	c	c	c	c	c	c	c	a	a		c				
g	4685730	a	4863939	s	g	CFT073	++++	g	g	g	g	g	g	g	g	g	a	g	g	g	g	g	g	g	g		g				
t	4686261	g	4864470	ns	g	clone D	++	g	g	g	g	g	a	g	g	g	a	g	g	g	g	g	g	g	g		g				
c	4695657	a	4873866	ns	c	CFT073	++	c	c	c	c	c	c	c	c	c	c	c	c	c	c	c	c	c	c		c	c			
t	4695924	g	4874133	nc	t	CFT073	++	t	t	t	t	t	t	t	t	t	t	t	t	t	t	t	t	t	t		t	t			

Clone D base	Clone D site	CFT 073 base	CFT 073 site	mutation type[a]	Inferred ancestral base[b]	lineage inferred to mutate	Support level[c]	ED1a[d]	536	S88	APEC O1	UTI89	E2348/69	ATCC8739	K-12	HS	SMS3-5	IAI39	E24377A	SE11	55989	IAI1	Sakai	UMN026	B4 Sb227	F5 8401	SS Ss046	D1 Sd197	B18 BS512	F2a 2457T	F2a 301
c	4696327	t	4874536	ns	c	CFT073	++	c	c	c	c	c	c	c	c	c	c	c	c	c	c	c	c	c	c		c	c			
c	4696345	t	4874554	ns	c	CFT073	++	c	c	c	c	c	c	c	c	c	c	c	c	c	c	c	c	c	c		c	c			
t	4696372	g	4874581	ns	t	CFT073	++	t	t	t	t	t	t	t	t	t	t	t	t	t	t	t	t	t	t		t	t			
c	4696409	t	4874618	ns	c	CFT073	++	c	c	c	c	c	c	c	c	c	c	c	c	c	c	c	c	c	c		c	c			
t	4706808	c	4885017	ns	c	clone D	++++	c	c	c	c	c	c	c	c	c	c	c	c	c	c	c	c	c	c	c	c	c	c	c	c
a	4727547	t	4905756	ns	t	clone D	++++	t	t	t	t	t	t	t	t	t	t	t	t	t	t	t	t	t	t	t	t	t		t	t
c	4739802	a	4918011	ns	c	CFT073	++++	c	c	c	c	c	c	c	c	c	c	c	c	c	c	c	c	c			c				
t	4753727	c	4984072	ns	c	clone D	++++	c	c	c	c	c	c	c	c	c	c	c	c	c	c	c	c	c	c	c	c	c	c	c	c
g	4757619	t	4987964	ns	g	CFT073	++++	g	g	g	g	g	g	g	g	g	g	g	g	g	g	g	g	g	g	g	g	g	g	g	g
c	4758195	t	4988540	s	c	CFT073	++++	c	c	c	c	c	c	c	c	c	c	c	c	c	c	c	c	c	c	c	c	c	c	c	c
a	4759117	g	4989462	s	a	CFT073	++++	a	a	a	a	a	a	a	a	a	a	a	a	a	a	a	a	a	a	a	a	a	a	a	a
t	4764748	c	4995093	ns	t	CFT073	++++	t	t	t	t	t	t	t	t	t	t	t	t	t	t	t	t	t	t	t	t	t	t	t	t
a	4767795	g	4998140	ns	a	CFT073	++++	a	a	a	a	a	a	g	g	g	g	g	g	g	g	g	g	a	g	g	g	g	g	g	g
a	4790240	g	5020584	s	g	clone D	++	g	a	a	a	a	a	g	g	g	g	g	g	g	g	g	g	g	g	g	g	g	g	g	g
g	4790974	t	5021317	s	g	CFT073	++++	g	g	g	g	g	g	g	g	g	g	g	g	g	g	g	g	g	g	g	g	g	g	g	g
c	4823568	t	5053911	s	c	CFT073	++++	c	c	c	c	c	c	c	c	c	c	c	c	c	c	c	c	c	c	c	c	c	c	c	c
c	4825471	t	5055814	s	c	CFT073	++++	c	c	c	c	c	c	c	c	c	c	c	c	c	c	c	c	c	c	c	c	c	c	c	c
g	4831334	t	5061677	ns	g	CFT073	++++	g	g	g	g	g	g	g	g	g	g	g	g	g	g	g	g	g	g	g	g	g	g	g	g
g	4864851	t	5095194	nc	g	CFT073	++	g	g		g	g						g													
g	4868624	a	5098967	ns	a	clone D	++	a	a		a	a	a					a													
a	4883853	g	5114197	ns	a	CFT073	++++	a	a	a	a	a	a	a	a	a	a	a	a		a	a	a	a	a	a	a	a	a	a	a
t	4939176	c	5131503	ns	t	CFT073	++	t	t	t	t	t	t		t	t	a	t	t			t	t	t	t	t	t			t	t
a	4953849	g	5146176	nc	a	CFT073	++	a	a	a	a	a	a	a	a	a	a	a	a	a	a	a	a	a	a	a	a		a	a	a
c	4963811	t	5156138	ns	c	CFT073	++		c	c	c	c	c	c	c	c	c	c	c	c	c	c	c	c	c				c		
a	4964151	g	5156478	s	g	clone D	+		g	g	g	g	g	g	g	g	g	g	g	g	g	g	g	g	g				g		
a	4967637	g	5159964	ns	a	CFT073	++	a	a	a	a	a	a	a	a	a	a	a	a	a	a	a	a	a	a			a	a		
c	5003843	t	5196172	s	c	CFT073	++++	c	c	c	c	c	c	c	c	c	c	c	c	c	c	c	c	c	c	c	c	c	c	c	c
c	5013999	t	5206328	s	c	CFT073	++++	c	c	c	c	c	c	c	c	c	c	c	c	c	c	c	c	c	c	c	c	c	c	c	c

Clone D base	Clone D site	CFT 073 base	CFT 073 site	mutation type[a]	Inferred ancestral base[b]	lineage inferred to mutate	Support level[c]	ED1a[d]	536	S88	APEC O1	UTI89	E2348/69	ATCC8739	K-12	HS	SMS3-5	IAI39	E24377A	SE11	55989	IAI1	Sakai	UMN026	B4 Sb227	F5 8401	SS Ss046	D1 Sd197	B18 BS512	F2a 2457T	F2a 301
c	5015018	t	5207347	s	c	CFT073	++++	c	c	c	c	c	c	c	c	c	c	c	c	c	c	c	c	c	c	c	c	c	c	c	c
g	5019370	a	5212411	ns	g	CFT073	++	g		g	g	g			g																

[a]s: synonymous; ns: non-synonymous; nc: in non-coding gene.

[b]The base present in ED1a OR if site absent in ED1a, the base in majority outgroup strains OR if base present in ED1a is not that in any of the clone D and CFT073 strains, the base in majority outgroup strains.

[c]Level of support for allocation of mutation as given in previous column.

"++++" agreement is high - 8 or more outgroup strains with expected base and at most 1 with an alternative base, and do not present alternative base in ED1a

"+++" agreement good - 4 or more outgroup strains with expected base and at most 1 with an alternative base, and do not present alternative base in ED1a

"++" agreement in ED1a regardless of situation with other outgroup strains

"+" no conflict but very limited support as either site absent in ED1a, and/or support is less than required for any of the higher levels of support

"+/-" no no conflict but no support (base not present in any outgroup OR base when present is not that in any of clone D and CFT073 strains OR both alternative lineages supported equally)

[d] "-" indicates absence of the base. Blank means the site not present.
