# Supplementary material for: Rates of Mutation and Host Transmission for an Escherichia coli Clone over 3 Years
Source: PLoS One. 2011 Oct 27;6(10):e26907. doi: 10.1371/journal.pone.0026907 (PMC3203180; doi:10.1371/journal.pone.0026907)
Supplement: Table S9 — Genbank accession numbers for genome sequences included in Figure 1 . (PDF) [file pone.0026907.s010.pdf]

Table S9 Genbank accession numbers for genome sequences included in Figure 1.

**Strain**	**Accession number**
12009	AP010958.1
55989	CU928145.2
W	CP002185.1
KO11	CP002516.1
E24377A	CP000800.1
SE11	AP009240.1
IAI1	CU928160.2
11368	AP010953.1
11128	AP010960.1
B(REL606)	CP000819.1
B (BL21(DE3))	CP001509.3
B(BL21(DE3))	AM946981.2
B(BL21(DE3))	CP001665.1
HS	CP000802.1
ATCC 8739	CP000946.1
UMNK88	CP002729
ECTC H10407	FN649414.1
UMNF18	CP002890
K12 (BW2952)	CP001396.1
K12 (DH1)	CP001637.1
K12 (DH1)	AP012030.1
K12 (DH10B)	CP000948.1
K12 (MG1655)	U00096.2
K12 (W3110)	AP009048
CB9615(O55)	CP001846.1
O157(EC4115)	CP001164.1
O157(EDL933)	AE005174.2
O157(Sakai)	BA000007.2
O157(TW14349)	CP001368.1
UMN026	CU928163
042	FN554766.1
SMS-3-5	CP000970.1
IAI39	CU928164.2
E2348/69	FM180568.1
SE15	AP009378.1
NA114	CP002797.1
clone D(i2)	CP002211
CFT073	AE014075.1
ABU 83972	CP001671.1
NRG 857C	CP001855.1

LF82	CU651637.1
536	CP000247.1
ED1a	CU928162.2
clone A(i1)	AEYT00000000
S88	CU928161.2
APECO1	CP000468.1
IHE3034	CP001969.1
UTI89	CP000243.1
UM146	CP002167.1
SS Ss046(*Shigella*)	CP000038.1
B4 Sb227(*Shigella*)	CP000036.1
B18 BS512(*Shigella*)	CP001063.1
F5b Sf8401(*Shigella*)	CP000266.1
F2a 2457T(*Shigella*)	AE014073.1
Fxv 2002017(*Shigella*)	CP001383.1
D1 Sd197(*Shigella*)	CP000034.1
E. fergusonii ATCC 35469	CU928158.2
